# miR-29a contributes to breast cancer cells epithelial–mesenchymal transition, migration, and invasion via down-regulating histone H4K20 trimethylation through directly targeting SUV420H2

**DOI:** 10.1038/s41419-019-1437-0

**Published:** 2019-02-21

**Authors:** You Wu, Wanyue Shi, Tingting Tang, Yidong Wang, Xin Yin, Yanlin Chen, Yanfeng Zhang, Yun Xing, Yumeng Shen, Tiansong Xia, Changying Guo, Yi Pan, Liang Jin

**Affiliations:** 1 State Key Laboratory of Natural Medicines, Jiangsu Key Laboratory of Druggability of Biopharmaceuticals, School of life Science and Technology, China Pharmaceutical University, 24 Tongjiaxiang, Nanjing, Jiangsu province China; 2 Department of Breast Surgery, Breast Disease Center of Jiangsu Province, First Affiliated Hospital of Nanjing Medical University, 300 Guangzhou Road, Nanjing, Jiangsu province China

**Keywords:** Cancer stem cells, Cell invasion

## Abstract

Breast cancer is the most prevalent cancer in women worldwide, which remains incurable once metastatic. Breast cancer stem cells (BCSCs) are a small subset of breast cancer cells which are essential in tumor formation, metastasis, and drug resistance. microRNAs (miRNAs) play important roles in the breast cancer cells and BCSCs by regulating specific genes. In this study, we found that miR-29a was up-regulated in BCSCs, in aggressive breast cancer cell line and in breast cancer tissues. We also confirmed suppressor of variegation 4–20 homolog 2 (SUV420H2), which is a histone methyltransferase that specifically trimethylates Lys-20 of histone H4 (H4K20), as the target of miR-29a. Both miR-29a overexpression and SUV420H2 knockdown in breast cancer cells promoted their migration and invasion in vitro and in vivo. Furthermore, we discovered that SUV420H2-targeting miR-29a attenuated the repression of connective tissue growth factor (CTGF) and growth response protein-1 (EGR1) by H4K20 trimethylation and promoted the EMT progress of breast cancer cells. Taken together, our findings reveal that miR-29a plays critical roles in the EMT and metastasis of breast cancer cells through targeting SUV420H2. These findings may provide new insights into novel molecular therapeutic targets for breast cancer.

## Introduction

Breast cancer is the most frequently diagnosed cancer and the leading cause of cancer death among females worldwide. The decrease in breast cancer-related deaths has been observed since the early 1990s due to improved strategies to diagnose and treat breast cancer. However, metastatic disease remains the underlying cause of death in the majority of breast cancer patients who succumb to their disease^1^. Breast cancer stem cells (BCSCs) were a tumorigenic subset of breast cancer cells first isolated from human breast tumors with the expression of the surface markers CD44+/CD24−, which are the radical cause of drug resistance, tumor relapse, and metastasis in breast cancer. Thus, to achieve a breakthrough in the treatment of breast cancers may require the successful targeting of BCSCs.

Recent studies showed that putative BCSCs exhibit a distinct miRNA expression profile compared to the other breast cancer cells^2^. The deregulated miRNAs may contribute to carcinogenesis and self-renewal of BCSCs via multiple pathways^3–5^. For example, miR-210 was reported by our lab to be up-regulated in BCSCs and promoted BCSCs invasion by decreasing the expression of E-cadherin^6^. However, the importance of many other differentially expressed miRNAs and their roles in regulating breast cancer cells or BCSCs properties remains to be determined.

Epigenetic alterations such as DNA methylation and histone modifications occur in many cancers^7–9^. Aberrant histone modifications are associated with carcinogenesis and cancer progression by affecting genomic integrity and by altering the expressions of related genes. Global histone modification patterns can predict clinical outcome, as recently shown for many types of cancer^10,11^. Loss of histone H4 lysine 20 trimethylation (H4K20me3) is considered to be a hallmark of human cancer and a potential prognostic marker in many types of cancer including breast cancer^12–14^. The decrease in H4K20me3 in cancer cells is found associated with diminished expression of SUV420H2, which is a histone lysine methyltransferase that specifically trimethylates histone H4K20. It has been shown that ectopic expression of SUV420H2, which caused the increase of H4K20me3, suppressed MDA-MB-231 cells invasion by targeting tensin-3^15^.

Our laboratory previously found miR-29a was both up-regulated in the MCF-7 spheroid cells and BCSCs MCF-7 cells compared to MCF-7 cells by performing miRNAs expression profiling. In this study, we first demonstrated that miR-29a was significantly up-regulated in BCSCs and the aggressive breast cancer cell line, MDA-MB-231 cells, as well as in human breast cancer tissues. Subsequently, we found miR-29a could be induced by basic fibroblast growth factor (bFGF) and significantly promoted breast cancer cells migration and invasion. We then identified SUV420H2 as a direct target gene of miR-29a, SUV420H2 overexpression compromised the migration and invasion abilities of miR-29a-overexpressing breast cancer cells both in vitro and in vivo. Our further study discovered that SUV420H2-targeting miR-29a could promote EMT of breast cancer cells via down-regulating H4K20me3, which attenuated the repression of EGR1 and CTGF. Taken together, our findings indicate that bFGF-induced miR-29a might play a critical role in the EMT and metastasis of breast cancer cells through down-regulating H4K20me3 via directly targeting SUV420H2. Therefore, miR-29a and SUV420H2 might represent the potential targets of breast cancer therapy.

## Materials and methods

### Cell line and monolayer culture

Two human breast cancer cell lines, MCF-7 and MDA-MB-231, and an embryonic kidney cell line, HEK-293T, were purchased from the Institute of Biochemistry and Cell Biology of the Chinese Academy of Sciences (Shanghai, China). MCF-7 and HEK-293T cells were maintained in DMEM medium (Gibco). MDA-MB-231 cells were cultured in L-15 medium (Gibco). The medium was supplemented with 10% fetal bovine serum (FBS, Gibco) and 1% penicillin/streptomycin (Gibco). All cells were cultured in humidified incubators at 37 °C with 5% CO_2_.

### 3D semi-solid spheres culture

Three thousand single cells were seeded into 24-well Ultra-Low Attachment Microplates (Corning) in serum-free DMEM/F12 (Invitrogen), supplemented with B27 (1:50, Invitrogen), 20 ng/ml EGF (Peprotech), 10 ng/ml bFGF (Invitrogen), 4 μg/ml insulin (Sigma), and 20% methylcellulose (Sigma). Spheres were collected 7 days after seeding.

### RNA extraction and quantitative RT-PCR

Total RNA was extracted from the cell lines using TRIzol Reagent (Invitrogen) according to the manufacturer’s instructions. Assays to quantify miRNAs were performed using TaqMan miRNA probes (ABI) according to the manufacturer’s instructions. The expression levels of mRNAs were determined using the SYBR Green (Vazyme) method according to the manufacturer’s protocol. The sequences of the primers were listed in Supplementary Tab. S1. Results were normalized to the threshold cycle (Ct) of GAPDH or U6, referred to as ∆Ct. The relative expression level was determined using the 2^−ΔΔCt^ analysis method. The experiments were performed in triplicate.

### Flow cytometry

To detect the BCSCs subpopulations, the following antibodies were applied: anti-CD44-APC, anti-CD24-PE, IgG1-PE, and IgG1-APC (BD). Human breast specimens were mechanically dissociated and incubated with 200 U/ml Liberase Blendzyme 4 (Roche) for 2 h to obtain single cell suspensions. Then cell staining and flow cytometry were performed as described previously^16^. Cells were sorted on a flow cytometer (FACSAriaII) and analyzed on another flow cytometer (BD C6) with BD FACS Diva software.

### Cell transfection and virus Infection

For transient transfection, miRNA mimics, inhibitors, siRNAs (Genepharma), and plasmids were transfected using Lipofectamine 2000 (Invitrogen) according to the manufacturer’s protocol. Lentivirus encoding miR-29a (Genepharma) or SUV420H2 were imported into MCF-7 cells as previously described^17^. The clones with the stable miR-29a or SUV420H2 expression were selected by green fluorescence protein (GFP) expression.

### Migration and invasion assays

To analyze the migration and invasion ability of cells, wound healing assay, transwell migration assay, and transwell invasion assay were performed according to the published methods^17^. For the cell transmembrane migration assay, all the steps were carried out similarly to those in the invasion assay except for the Matrigel coating. After incubation at 37 °C for 24 h, the filters were removed. The cells adhering to the lower surface were fixed and stained with 0.1% Crystal Violet. To image the cells, 10 randomly selected fields in each well were photographed at the magnification of 200 and counted. *n* = 3 independent experiments.

### Western blotting and immunofluorescence

Antibodies against SUV420H2 (Abcam 1:1000), H4K20me3 (Abcam 1:1000), CTGF(Abcam 1:1000), EGR1(Abcam 1:1000), E-cadherin (Abcam 1:1000), Vimentin (Abcam 1:2000), and Snail (CST 1:1000) were used in the western blot analysis to study the protein levels of whole-cell extracts, which were then detected with horseradish peroxidase-conjugated anti-IgG (Santa 1:2000) and visualized with an ECL kit (Tanon). Results were normalized with GAPDH (Santa 1:1000). Immunofluorescence assay was performed 48 h after transfection as described elsewhere^16^, in which the monoclonal antibodies, including anti-E-cadherin (CST 1:100) and anti-Vimentin (Abcam 1:200) antibodies were used. Cell images were captured using a laser scanning confocal microscopy (Olympus).

### Luciferase assay

The putative miR-29a MREs of the human SUV420H2 3′UTR were synthesized and inserted between the SpeI and HindIII sites of the pMIR-Report plasmid. We also constructed a pMIR-Report plasmid that carried the mutant SUV420H2 3′UTR region. For the luciferase reporter assays, HEK-293T cells were cultured in 24-well plates, and each well was transfected with 0.4 μg firefly luciferase reporter plasmid, 0.15 μg phRL-TK vector (renilla luciferase) expression vector, and equal amounts (20pmol) of control mimic, miR-29a, control inhibitor, or miR-29a inhibitor using Lipofectamine2000 (Invitrogen). The phRL-TK vector was used as control. The luciferase assay was performed 24 h after transfection using double-luciferase assay system (Beyotime, Shanghai, China).

### High throughput sequencing

Three batches of control MCF-7 cells and SUV420H2-overexpressing MCF-7 cells samples were harvested in parallel after being transfected with control or SUV40H2 vector for 24 h. Before the high-throughput sequencing, three batches of each group of cells were pooled together and mixed, then the total RNAs were extracted with Trizol. The quality control of the samples, the experiments, and the data analysis were completely finished by Genergy Inc (Shanghai, China). The alignment, gene expression, and assembly analysis were performed by HISAT2 (V2.0.1), HTSEQ (V0.6.1), and Stringtie (V1.0.4), respectively. EdgeR (V3.4.6, |FC| ≥ 1, FDR ≤ 0.05) was used to analyze the differentially expressed genes. The threshold used to screen up-regulated or down-regulated RNAs was fold change >2.0 with FDR (adjusted *P*-value) ≤0.05.

### Chromatin immunoprecipitation

Chromatin immunoprecipitation (ChIP) assays were performed using EZ-CHIP KIT (Millipore), according to the manufacturer’s instructions. In brief, the cultured MCF-7 cells were treated with 1% formaldehyde and incubated for 10 min to generate cross-links of protein-DNA. Cell lysates were then sonicated to generate chromatin fragments of 200–300 bp and immunoprecipitated with anti- SUV420H2 pAb, anti-H4K20me3 pAb (Abcam), or normal rabbit IgG as control. The primer sequences used for ChIP were listed in Supplementary Tab. S5. DNAs eluted from the ChIP assay were amplified by qPCR. Normal rabbit IgG was used as negative control. ChIP data were analyzed and shown by the percentage relative to the input DNA amount by the equation 2^[Input Ct- Target Ct]^ × 0.1 × 100.

### Human tissue

Twelve breast tumor tissues were collected from patients during surgical procedures at the First Affiliated Hospital of Nanjing Medical University (Nanjing, China). All the tumor tissues were confirmed histologically. All the patients provided written consent, and the experiments were approved by the Ethics Committee of China Pharmaceutical University, (Nanjing, China). The clinical features of the patients are listed in Supplementary Tab. S2.

### Xenograft assays in nude mice

Six-week-old female BALB/c nude mice were purchased from the Model Animal Research Center at Nanjing University (Nanjing, China) and maintained under specific pathogen-free conditions at Nanjing University. The mice were randomly divided into 4 groups and were injected intravenously through the tail vein with MCF-7 cells (2 × 10^6^ cells per mouse, 5 mice per group) that were infected with control lentivirus, miR-29a lentivirus, SUV420H2 overexpression lentivirus, or with both miR-29a lentivirus and SUV420H2 overexpression lentivirus. The number of tumor nodules in lung was counted after 8 weeks. Each tissue was excised and embedded in paraffin for histopathological examination. All animal experiments were approved by the Ethics Committee of China Pharmaceutical University. Permit Number: 2162326.

### Statistical analysis

All experimental data were expressed as the mean and standard deviation (mean ± SD). Statistical analysis was performed using Student’s independent *t*-test via GraphPad Prism 5 software between experimental groups. The level of significance was set at *P < 0.05.

## Results

### miR-29a is up-regulated in breast cancer due to bFGF

**Fig. 1:**
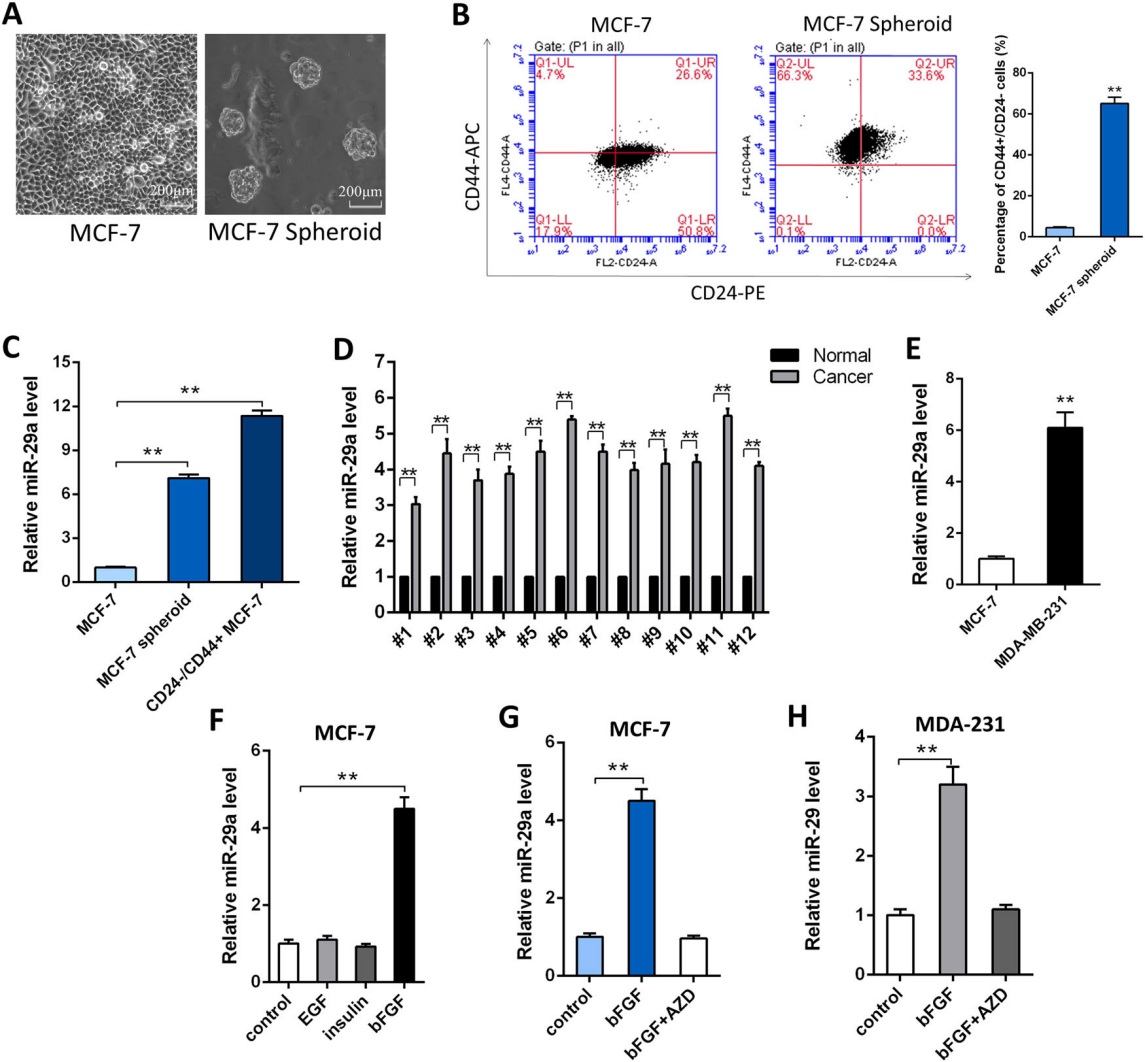
miR-29a is up-regulated in breast cancer cells. **a** Bright field images of MCF-7 cells formation under monolayer culture condition and 3D culture condition (Magnification: ×20, scale bar: 200 μm), respectively. **b** The percentage of CD44+/CD24− population in MCF-7 cells and MCF-7 spheroid cells respectively. **c** miR-29a levels in MCF-7 cells, MCF-7 spheroid cells and CD44+/CD24− MCF-7 cells. **d** miR-29a levels in 12 pairs of human breast cancer tissues (Cancer) and corresponding distal non-cancerous tissues (Normal). **e** miR-29a levels in MCF-7 cells and MDA-MB-231 cells. **f** miR-29a levels in MCF-7 cells under stimulation of EGF, bFGF, and insulin. **g**, **h** miR-29a levels in MCF-7 cells (**g**) and MDA-MB-231 cells (**h**) under stimulation of bFGF, and bFGF plus AZD. **P < 0.01

miR-29a was reported up-regulated in MCF-7 spheroid cells, and CD44+/CD24− MCF-7 cell compared with MCF-7 cells by our laboratory previously^6^. Accordingly, we used the 3D semi-solid culture system to culture MCF-7 spheroid cell as shown in Fig. 1a, b. Then we verified that miR-29a was up-regulated in both MCF-7 spheroid cells and CD44+/CD24− MCF-7 cells compared to MCF-7 cells (Fig. 1c). We also compared the expression levels of miR-29a in 12 pairs of human breast cancer tissues and their corresponding distal non-cancerous tissues, and in a more aggressive breast cancer cell line, MDA-MB-231 cells, which contains high ratio of CD44+/CD24− cells (~80%). miR-29a levels were remarkably higher in breast cancer tissues compared with those noncancerous counterparts (Fig. 1d), as well as in MDA-MB-231 cells compared to MCF-7 cells (Fig. 1e). Our results suggest that miR-29a may contribute to the tumorigenicity of breast cancer.

Next, we explored the mechanism by which miR-29a expression is up-regulated in BCSCs and breast cancer tissues. Since EGF, bFGF, and insulin are the three main components in our sphere-formation pelletizing system, which also exist in the tumor microenvironment^18,19^, we exposed MCF-7 cells to EGF, bFGF, or insulin separately. As shown in Fig. 1f, miR-29a was only up-regulated by bFGF. Furthermore, this induction by bFGF could be blocked by a potent inhibitor of the FGFR family (Fig. 1g, h). These results demonstrate that miR-29a expression was specifically up-regulated by bFGF signaling in breast cancer cells.

### miR-29a promotes breast cancer cells migration and invasion in vitro

**Fig. 2:**
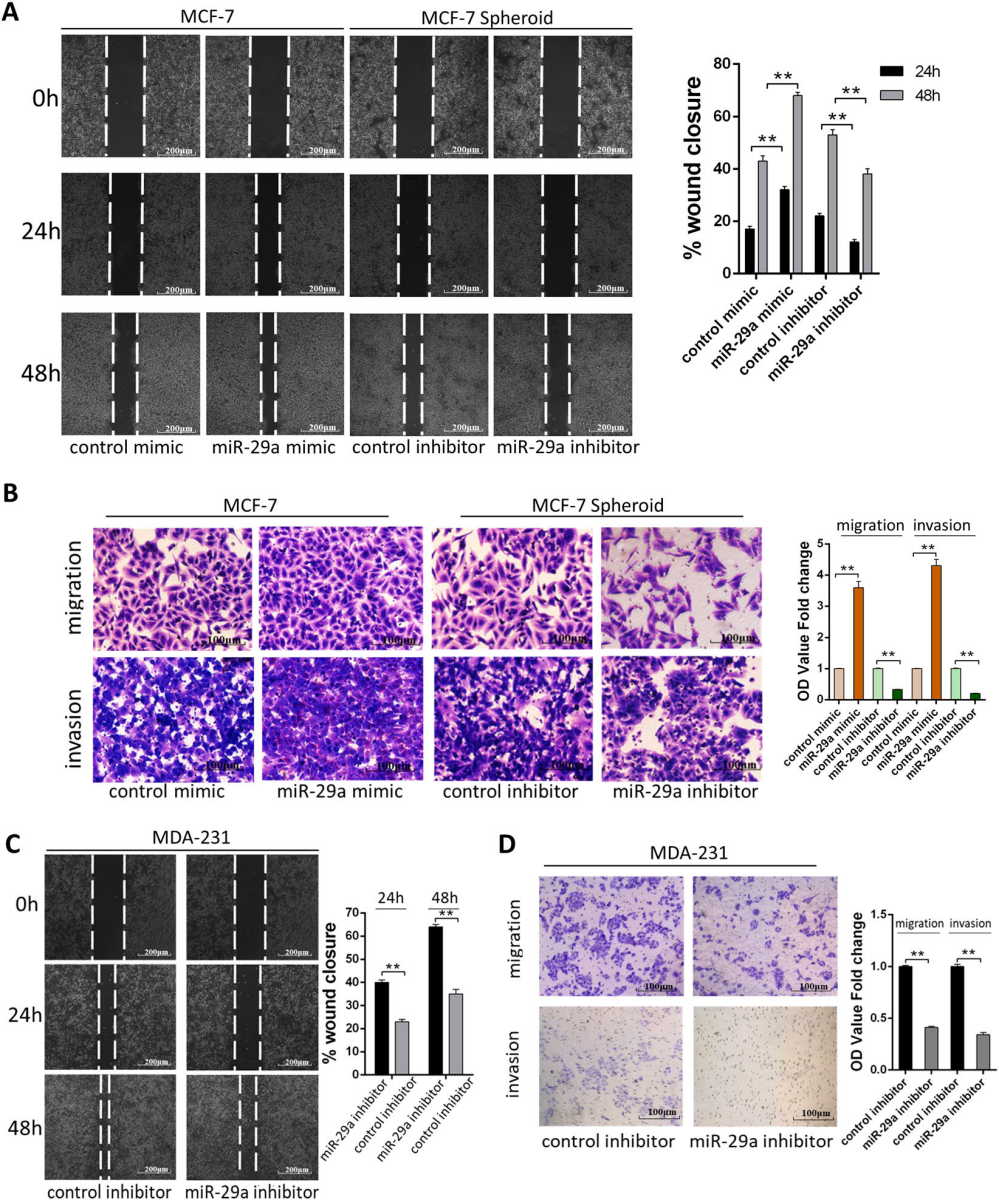
miR-29a promotes breast cancer cells migration and invasion in vitro. **a**, **b** Migration and invasion of MCF-7 cells transfected with control mimic or miR-29a mimic and MCF-7 spheroid cells transfected with control inhibitor or miR-29a inhibitor detected by wound healing assay (**a**), transwell migration assay and transwell invasion assay (**b**). **c**, **d** Migration and invasion of MDA-MB-231 cells transfected with control inhibitor or miR-29a inhibitor detected by wound healing assay (**c**), transwell migration assay and transwell invasion assay (**d**). **P < 0.01

To explore the role of miR-29a in breast cancer progress, MCF-7 cells were transfected with miR-29a mimic and MCF-7 spheroid cells were transfected with miR-29a inhibitor. The ability of cell migration, proliferation, self-renewal, anti-apoptosis, and drug resistance were evaluated. The wound healing assays and transwell assays showed that the migration and invasion capacities of MCF-7 cells was significantly enhanced by overexpression of miR-29a. In contrast, the migration and invasion capacities of MCF-7 spheroid cells was strongly reduced by depleting miR-29a (Fig. 2a, b). Overexpression or knockdown of miR-29a in MCF-7 cells or MCF-7 spheroid cells did not have significant effect on their proliferation, self-renewal, anti-apoptosis, or drug resistance ability (data not shown). We also knocked down miR-29a in the miR-29a highly expressed MDA-MB-231 cells, which similarly suppressed the migration and invasion abilities of MDA-MB-231 cells (Fig. 2c, d). Our findings suggest that miR-29a is required for the high migration and invasion ability of BSCSs and aggressive breast cancer cells.

### Identification of SUV420H2 as a direct target gene of miR-29a in breast cancer cells

**Fig. 3:**
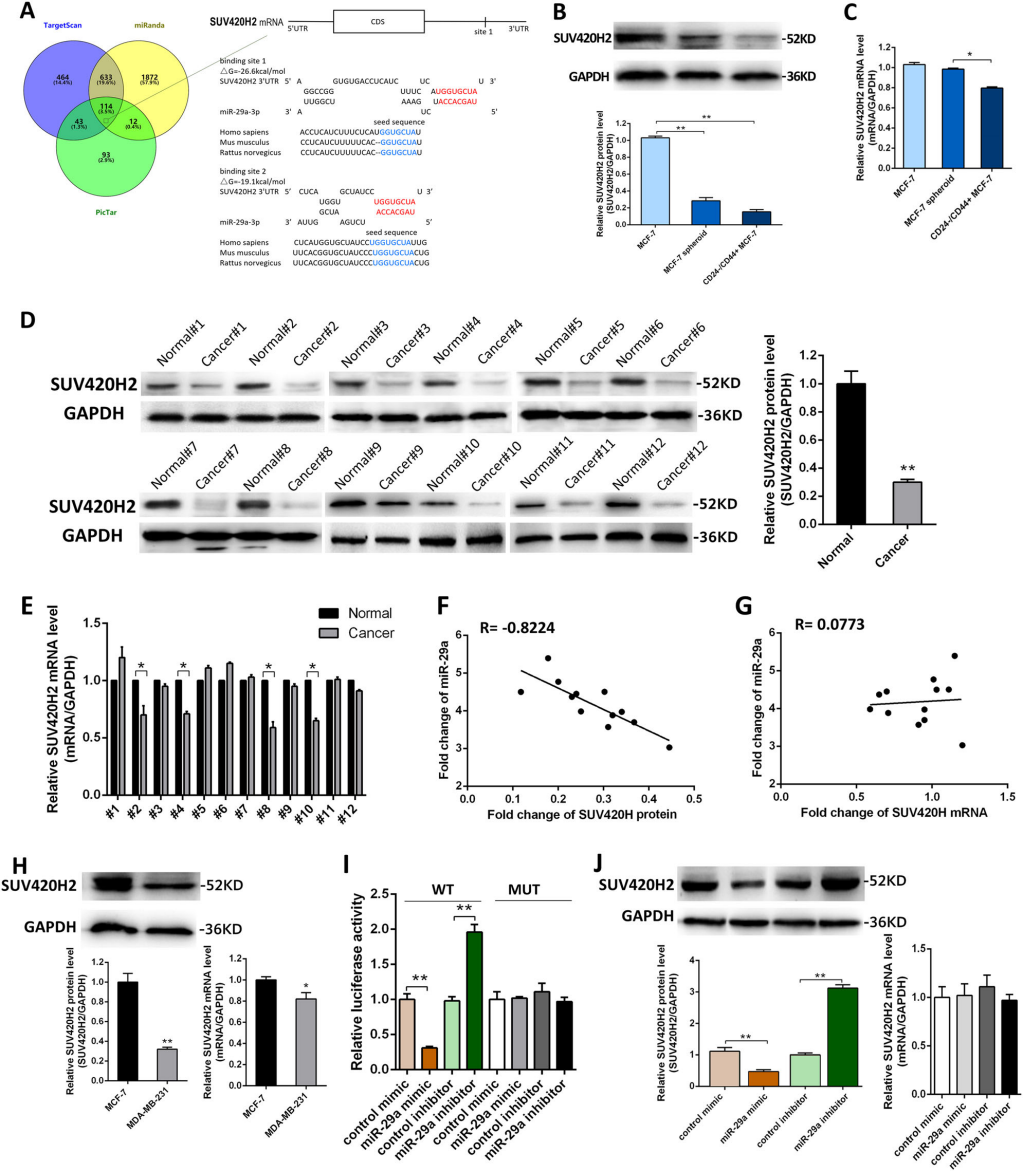
Identification of SUV420H2 as a direct target gene of miR-29a in breast cancer cells. **a** Left: Venn diagram analysis of three independent databases reveals four possible targets of miR-29a. Right: schematic description of the hypothetical duplexes formed by the interactions between the binding sites in the SUV420H2 3′-UTR and miR-29a. The predicted free energy value of each hybrid is indicated. The seed recognition sites are denoted, and all nucleotides in these regions are highly conserved across species, including human, mouse, and rat. **b**, **c** SUV420H2 protein (**b**) and mRNA (**c**) levels in MCF-7 cells, MCF-7 spheroid cells and CD44+/CD24− MCF-7 cells. **d**, **e** SUV420H2 protein (**d**) and mRNA (**e**) levels in 12 pairs of human breast cancer tissues (Cancer) and corresponding distal non-cancerous tissues (Normal). **f** Pearson’s correlation scatter plot of the fold change of miR-29a and SUV420H2 protein in human breast cancer tissues. **g** Pearson’s correlation scatter plot of the fold change of miR-29a and SUV420H2 mRNA in human breast cancer tissues. **h**, **i** SUV420H2 protein (**h**) and mRNA (**i**) levels in MCF-7 cells and MDA-MB-231 cells. **j** Direct recognition of the SUV420H2 3′-UTR by miR-29a. Firefly luciferase reporters containing either wild-type (WT) or mutant (MUT) miR-29a-binding sites in the SUV420H2 3′-UTR were co-transfected into MCF-7 cells with either the control mimic, control inhibitor, miR-29a mimic or miR-29a inhibitor. Twenty-four hours post-transfection, the cells were assayed using a luciferase assay kit. The results were calculated as the ratio of firefly luciferase activity normalized to the control cells. **k**, **l** SUV420H2 protein (**i**) and mRNA (**j**) levels in MCF-7 cells transfected with scrambled negative control RNA, miR-29a mimic, or miR-29a inhibitor. *P < 0.05; **P < 0.01

To investigate the underlying molecular mechanism by which miR-29a promotes breast cancer cells migration and invasion, we performed the Venn diagram analysis of predicted miR-29a targets from three independent databases: TargetScan, miRanda, and PicTar^20^. miRNAs (114) were found in the intersection part (Fig. 3a and Supplementary Tab. S3). Remarkably, SUV420H2 was the top candidate among the 11 tumor suppressors, which was also related to breast cancer metastasis and has not been reported as a miR-29a target in cancer, and was thus selected for further experimental verification. The predicted interaction between miR-29a and the target sites in the SUV420H2 3′-UTR was illustrated in the right panel of Fig. 3a. There was perfect base-pairing between the seed region and the cognate target. The free energy value of the hybrid was well within the range of genuine miRNA-target pairs (−26.6 kcal/mol).

In most cases, miRNAs generally have the expression patterns that are opposite to that of their targets. Therefore, we investigated whether miR-29a expression is inversely correlated with SUV420H2 expression in breast cancer cells and clinical specimens. We measured the SUV420H2 mRNA and protein levels in MCF-7 cells, MCF-7 spheroid cells, CD44+/CD24− MCF-7 cells, MDA-MB-231 cells and in breast cancer tissues compared to their corresponding distal non-cancerous tissues. Notably, we found that SUV420H2 protein levels of MCF-7 spheroid cells and MDA-MB-231 cells were lower than MCF-7 cells, and the trend was much more obvious in CD44+/CD24− MCF-7 cells (Fig. 3b–h). Consistently, SUV420H2 protein levels were lower in the cancer tissues than noncancerous tissues (Fig. 3d). In contrast, SUV420H2 mRNA levels did not differ significantly in either the breast cancer cells or clinical specimens (Fig. 3c–i), which is in accordance with a post-transcriptional mechanism that is involved in the regulation of SUV420H2. The inverse correlation between miR-29a and SUV420H2 protein levels (Fig. 3f) and the disparity between the miR-29a and SUV420H2 mRNA levels (Fig. 3g) were further illustrated using Pearson’s correlation scatter plots, which suggested that miR-29a regulated SUV420H2 protein expression at the posttranslational level.

Subsequently, we confirmed that miR-29a directly targeted the predicted binding sites in the SUV420H2 3′-UTR by a luciferase reporter assay (Fig. 3j). We next detected that SUV420H2 protein level was significantly decreased in the cells transfected with miR-29a mimic, while remarkably increased by suppression of the endogenous miR-29a (Fig. 3k). SUV420H2 mRNA levels had no alteration after either overexpression or knockdown of miR-29a (Fig. 3l). To verify these findings, we repeated the above experiments in MDA-MB-231 cells and observed similar results (Supplementary Fig. S1A and B). Taken together, these results strongly suggested that miR-29a down-regulated SUV420H2 protein level through directly binding to its 3′-UTR.

### miR-29a promotes breast cancer cells migration and invasion via targeting SUV420H2

**Fig. 4:**
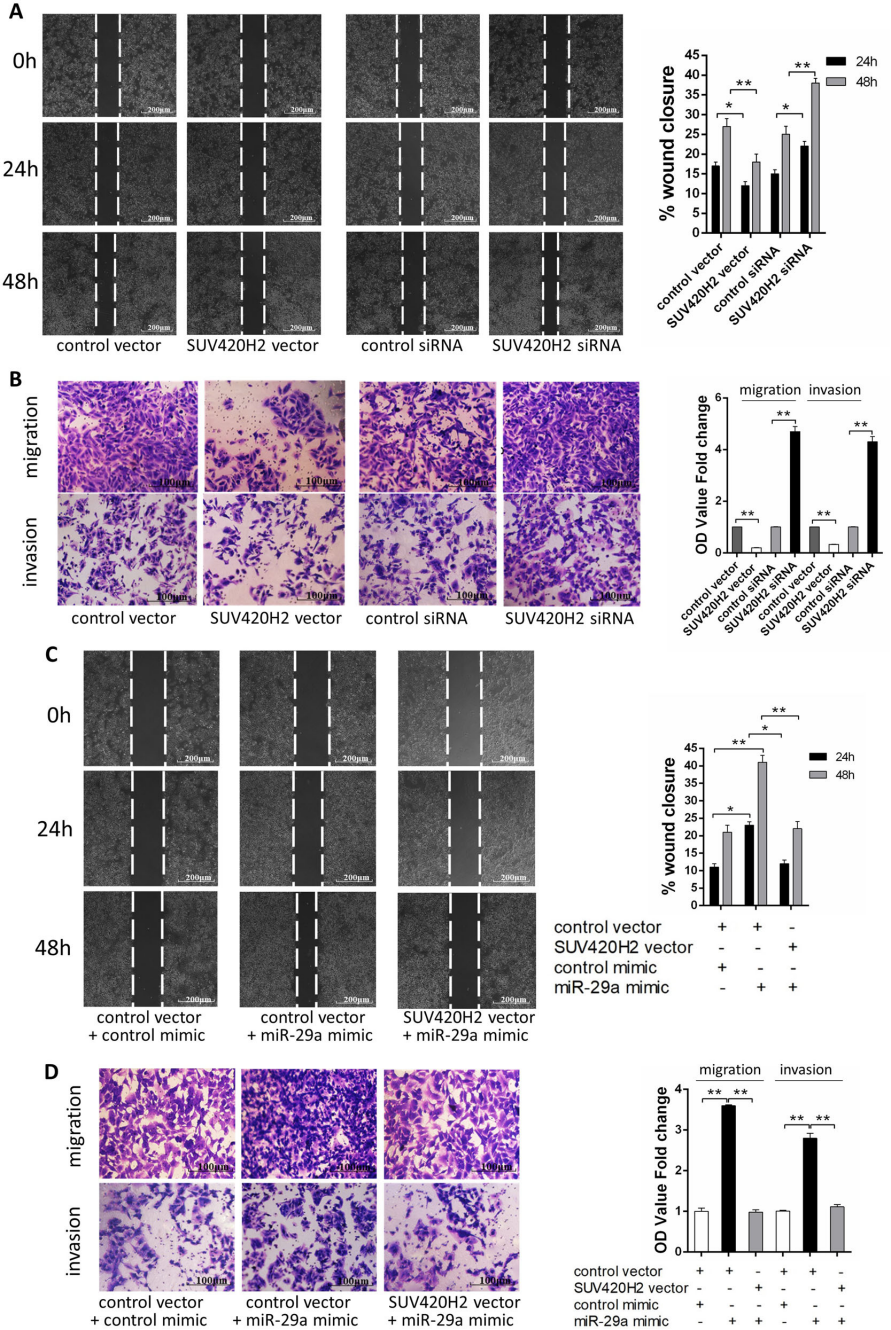
miR-29a promotes breast cancer cells migration and invasion via targeting SUV420H2. **a**, **b** Migration and invasion of MCF-7 cells transfected with either the control vector, SUV420H2 vector, control siRNA, or SUV420H2 siRNA as indicated, detected by wound healing assay (**a**), transwell migration assay, and transwell invasion assay (**b**). **c**, **d** Migration and invasion of MCF-7 cells transfected with either the control mimic plus control vector, miR-29a mimic plus control vector or miR-29a mimic plus SUV420H2 vector detected by wound healing assay (**c**), transwell migration assay and transwell invasion assay (**d**). *P < 0.05; **P < 0.01

We next focused on studying whether miR-29a facilitates breast cancer cells migration and invasion by repressing SUV420H2 expression. We observed that transfecting SUV420H2 siRNAs markedly promoted cell migration and invasion, while transfection of SUV40H2-overexpression plasmid repressed MCF-7 cells migration and invasion (Fig. 4a, b). Moreover, compared to cells transfected with miR-29a mimic, the cells transfected with miR-29a mimic and the SUV420H2- overexpression plasmid exhibited significantly lower migration and invasion abilities (Fig. 4c, d). The similar result was also observed in MDA-MB-231 cells (Supplementary Fig. S1D and E), suggesting that SUV420H2 can attenuate the pro-migration and pro-invasion effect of miR-29a. Taken together, these data suggested that miR-29a promoted the migration and invasion of breast cancer cells in a SUV420H2-dependent manner.

### miR-29a-mediated SUV420H2 decreases trimethylation of H4K20 and promotes EMT progress in MCF-7 cells

**Fig. 5:**
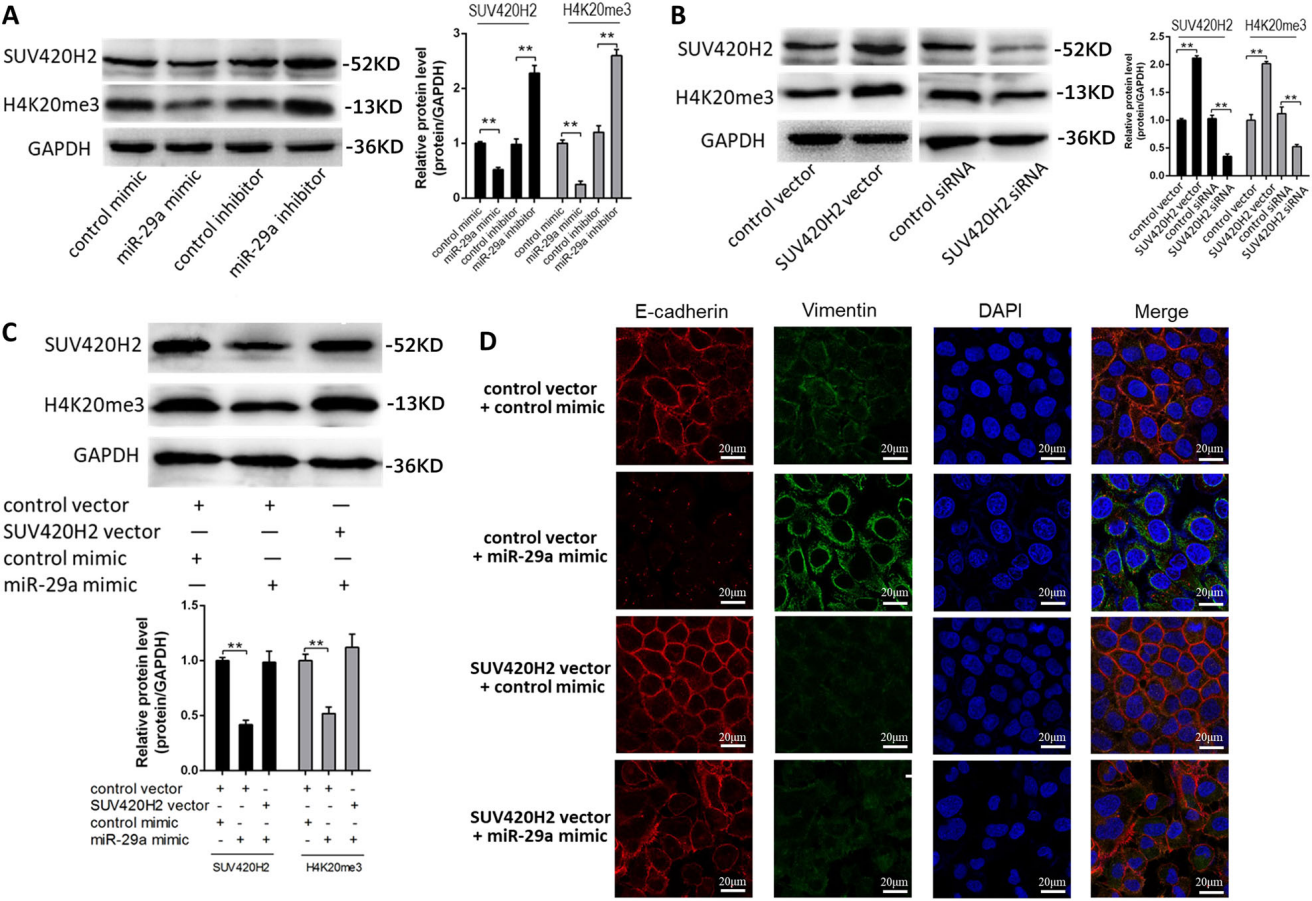
miR-29a-mediated SUV420H2 decrease trimethylation of H4K20 and promotes EMT progress in MCF-7 cells. **a** SUV420H2 and H4K20me3 protein levels in MCF-7 cells transfected with either the scrambled negative control RNA, miR-29a mimic, or miR-29a inhibitor. **b** SUV420H2 and H4K20me3 protein levels in MCF-7 cells transfected with either the control vector, SUV420H2 vector, control siRNA, or SUV420H2 siRNA. **c** SUV420H2 and H4K20me3 protein levels in MCF-7 cells transfected with either the control mimic plus control vector, miR-29a mimic plus control vector, or miR-29a mimic plus SUV420H2 vector. **d** Immunofluorescent staining of E-cadherin and Vimentin in MCF-7 cells transfected with either the control mimic plus control vector, miR-29a mimic plus control vector, control mimic plus SUV420H2 vector, or miR-29a mimic plus SUV420H2 vector. **P < 0.01

SUV420H2 has been known as a histone lysine methyltransferase that specifically trimethylated Lys-20 of histone H4. The loss of H4K20me3 has been reported in multiple types of human tumors including breast cancer. Thus, we repeated the miR-29a mimic and inhibitor transfection experiment in MCF-7 cells and measured both the SUV420H2 protein level and H4K20me3 level. As shown in Fig. 5a, H4K20me3 displayed the same trend in the alteration as the SUV420H2 protein after treatment with the miR-29a mimic or inhibitor in MCF-7 cells. The similar result was also observed in MDA-MB-231 cells (Supplementary Fig. S1A). When the SUV420H2-overexpression plasmid or siRNA was used to study the regulations of H4K20me3, the changes in H4K20me3 level were consistent with the changes of SUV420H2 protein level (Fig. 5b). Then we co-transfected MCF-7 cells with the SUV420H2-overexpression plasmid and miR-29a mimic to perform the rescue experiment and found that ectopic expression of SUV420H2 attenuated miR-29a-mediated H4K20me3 down-regulation in MCF-7 cells (Fig. 5c). The similar result was also observed in MDA-MB-231 cells (Supplementary Fig. S1C). In addition, we found that H4K20me3 levels in MCF-7 spheroid cells, CD44+/CD24− MCF-7 cells and MDA-MB-231 cells were lower than in MCF-7 cells (Supplementary Fig. S2A and B). Consistently, H4K20me3 levels were lower in the cancer tissues than noncancerous tissues (Supplementary Fig. S2C), the trends of which were in line with SUV420H2.

Since SUV420H2 or H4K20me3 could not directly participate in the process of cell migration and invasion but could regulate related genes expression, and miR-29a was reported to promote EMT in breast cancer^21,22^, which contributed to the migration and invasion of cells, we next test whether miR-29a could promote EMT of MCF-7 cells via targeting SUV420H2 by immunofluorescence. Figure 5d showed clearly that E-cadherin protein level was decreased in miR-29a-overexpressing MCF-7 cells, which is a typical protein expressed by epithelial cells, while Vimentin protein level was increased, which is a typical protein expressed in mesenchymal cells. The reverse trend was found in the SUV420H2-overexpressing MCF-7 cells. Then the rescue experiment showed that the suppression of E-cadherin protein and up-regulation of Vimentin protein by miR-29a was reversed when co-transfected with SUV420H2 vector (Fig. 5d). Taken together, the results suggest that miR-29a can affect H4K20me3 level by regulating SUV420H2 protein level, which promote the EMT progress in breast cancer cells.

### miR-29a promotes breast cancer cells EMT, migration and invasion by overexpressing EGR1 and CTGF genes through down-regulation of H4K20me3

**Fig. 6:**
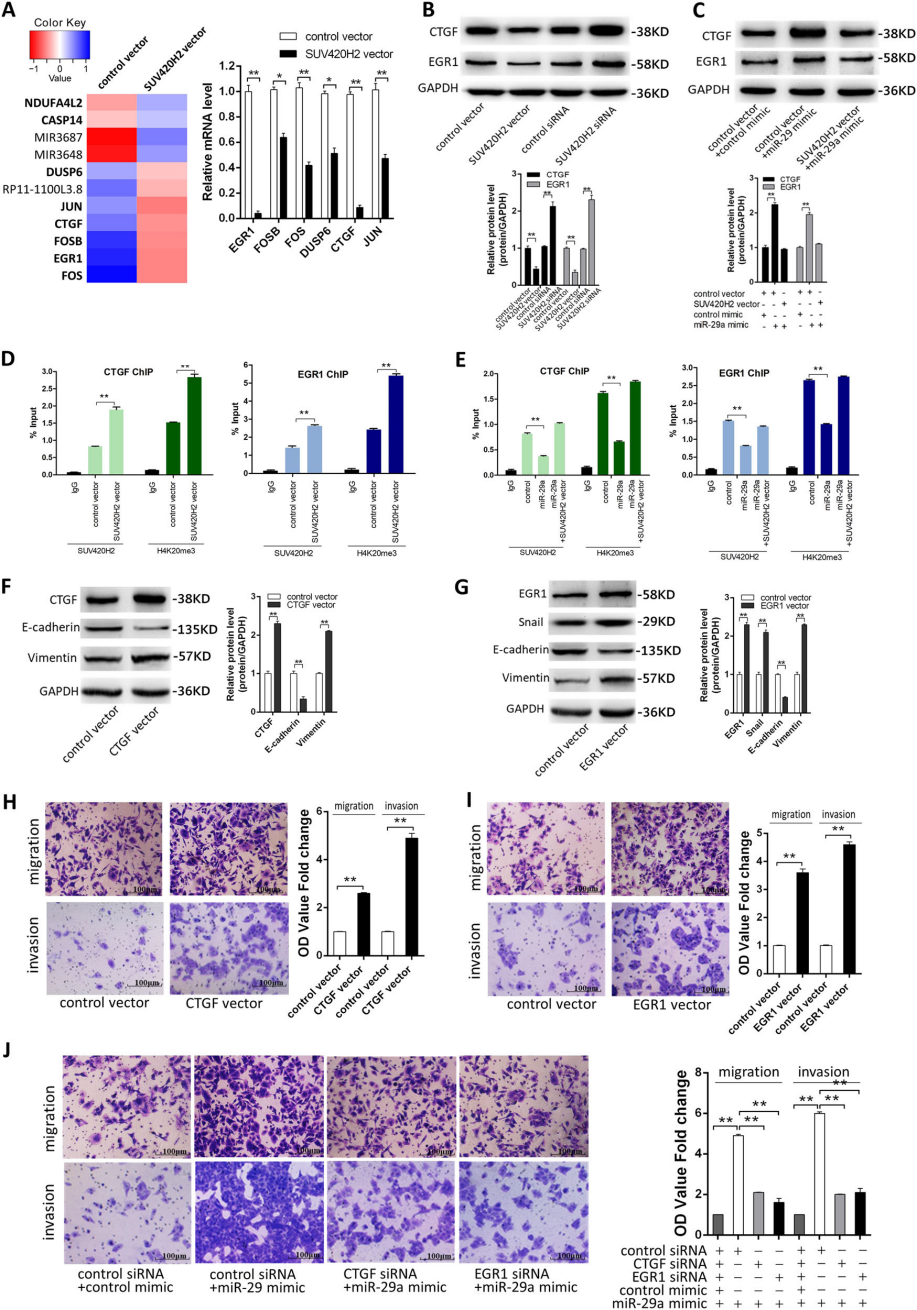
miR-29a promotes breast cancer cells EMT, migration and invasion by overexpressing EGR1 and CTGF genes through down-regulation of H4K20me3. **a** The heat map of significantly up- or down-regulated miRNAs between MCF-7 transfected with control vector and MCF-7 transfected with SUV420H2 vector. Each group contained three batches of individual samples, which were pooled and mixed. **b** Verification of EGR1, FOS, FOSB, JUN, DUSP6, and CTGF mRNA levels by qRT-PCR in the two groups. **c** CTGF and EGR1 protein levels in MCF-7 cells transfected with either the control vector, SUV420H2 vector, control siRNA, or SUV420H2 siRNA. **d** CTGF and EGR1 protein levels in MCF-7 cells transfected with either the control mimic plus control vector, miR-29a mimic plus control vector, or miR-29a mimic plus SUV420H2 vector. **e**, **f** ChIP-qPCR of SUV420H2/H4K20me3 of the promoter region of CTGF or EGR1 locus in MCF-7 cells transfected with either the control vector or SUV420H2 vector (**e**), and in MCF-7 cells transfected with either the control mimic plus control vector, miR-29a mimic plus control vector or miR-29a mimic plus SUV420H2 vector (**f**). Antibody enrichment was quantified relative to the amount of input DNA. Antibody directed against IgG was used as a negative control. **g** CTGF, E-cadherin, and Vimentin protein levels in MCF-7 cells transfected with control vector or CTGF vector. **h** EGR1, Snail, E-cadherin, and Vimentin protein levels in MCF-7 cells transfected with control vector or EGR1 vector. **i–k** Migration and invasion of MCF-7 cells transfected with control vector or CTGF vector (**i**); control vector or EGR1 vector (**j**); with either the control mimic plus control vector, miR-29a mimic plus control vector, miR-29a mimic plus CTGF vector, or miR-29a mimic plus EGR1 vector (**k**). *P < 0.05; **P < 0.01

SUV420H2 is involved in cancer progression by specifically regulating H4K20me3, which affects and alters the expression of specific cancer-related genes^23,24^, so we up-regulated H4K20me3 level by transfecting SUV420H2 vector and compared genome-wide gene expression profiles between control MCF-7 cells and SUV420H2-overexpressing MCF-7 cells. We found 12 genes were significantly regulated in SUV420H2-overexpressing MCF-7 cells (Fig. 6a, supplementary Tab. S4). Among these genes, EGR1, Fos Proto-Oncogene (FOS), FosB Proto-Oncogene (FOSB), Jun Proto-Oncogene (JUN), Dual Specificity Phosphatase 6 (DUSP6) and CTGF have been previously implicated in cancer metastasis^25–29^. Then we detected them in SUV420H2-overexpressing MCF-7 cells by qRT-PCR. As shown in Fig. 6b, all of the 6 genes were down-regulated in SUV420H2-overexpressing MCF-7 cells than control, and we noticed that EGR1 and CTGF mRNA levels were reduced the most. These regulations were also observed at the protein levels (Fig. 6c).

To investigate whether the suppression of SUV420H2 expression by miR-29a could affect EGR1 and CTGF expression in the MCF-7 cells, we compared the EGR1 and CTGF protein levels between the miR-29a-overexpressing MCF-7 cells and control MCF-7 cells. As anticipated, miR-29a overexpression led to the up-regulation of both EGR1 and CTGF protein levels, while ectopic expression of SUV420H2 attenuated miR-29a-mediated EGR1 and CTGF up-regulation in MCF-7 cells (Fig. 6d). The above results suggest that miR-29a can affect EGR1 and CTGF expression by regulating SUV420H2 protein levels.

It is known that H4K20me3 in cancer cells is responsible for suppressed transcription via enriching in the upstream of transcription start site^30–32^. To validate SUV420H2-targeting miR-29a regulates CTGF and EGR1 expression through decreasing H4K20me3 levels and its binding levels across the promoters of CTGF and EGR1, SUV420H2 and H4K20me3 modifications at the CTGF and EGR1 gene locus were examined using MCF-7 cells by ChIP. The ChIP assays demonstrated that overexpression of SUV420H2 increased the binding of SUV420H2 and H4K20me3 across the promoters of the CTGF and EGR1 (Fig. 6e), while overexpression of miR-29a decreased the binding levels. Furthermore, SUV420H2 vector and miR-29a mimic co-transfected into MCF-7 cells attenuated miR-29a-induced reduction of binding levels of SUV420H2/H4K20me3 across the promoters of the CTGF and EGR1 in Fig. 6f.

CTGF has been reported to promote EMT process in colorectal cancer^33^, and EGR1 was also involved in EMT of non-small-cell lung cancer cells and renal epithelial cells by modulating Snail^34,35^. To determine whether miR-29a-SUV420H2-H4K20me3 axis can promote EMT of MCF-7 cells by affecting CTGF and EGR1 expression, we first examined the E-cadherin and Vimentin levels in the CTGF-overexpressing MCF-7 cells, as well as the Snail, E-cadherin and Vimentin levels in the EGR1- overexpressing MCF-7 cells. As shown in Fig. 6h, the Snail protein level was increased in EGR1-overexpressing MCF-7 cells. For both CTGF-overexpressing MCF-7 cells and EGR1- overexpressing MCF-7 cells, we observed the down-regulation of E-cadherin and the up-regulation of Vimentin (Fig. 6g, h). In addition, both the CTGF-overexpressing MCF-7 cells and EGR1- overexpressing MCF-7 cells showed higher migration and invasion abilities than control MCF-7 cells (Fig. 6i, j). Moreover, the rescue experiment showed that compared to cells transfected with miR-29a mimic, the cells transfected with miR-29a mimic and the CTGF siRNA or EGR1 siRNA exhibited significantly lower migration and invasion abilities (Fig. 6k). Taken together, the results suggest that miR-29a can restore the expression of CTGF and EGR1 by down-regulating H4K20me3 and its binding levels across the promoters of the CTGF and EGR1, which promote EMT progress, migration, and invasion in MCF-7 cells.

### Targeting SUV420H2 by miR-29a promotes breast tumor cell metastasis in mouse model

**Fig. 7:**
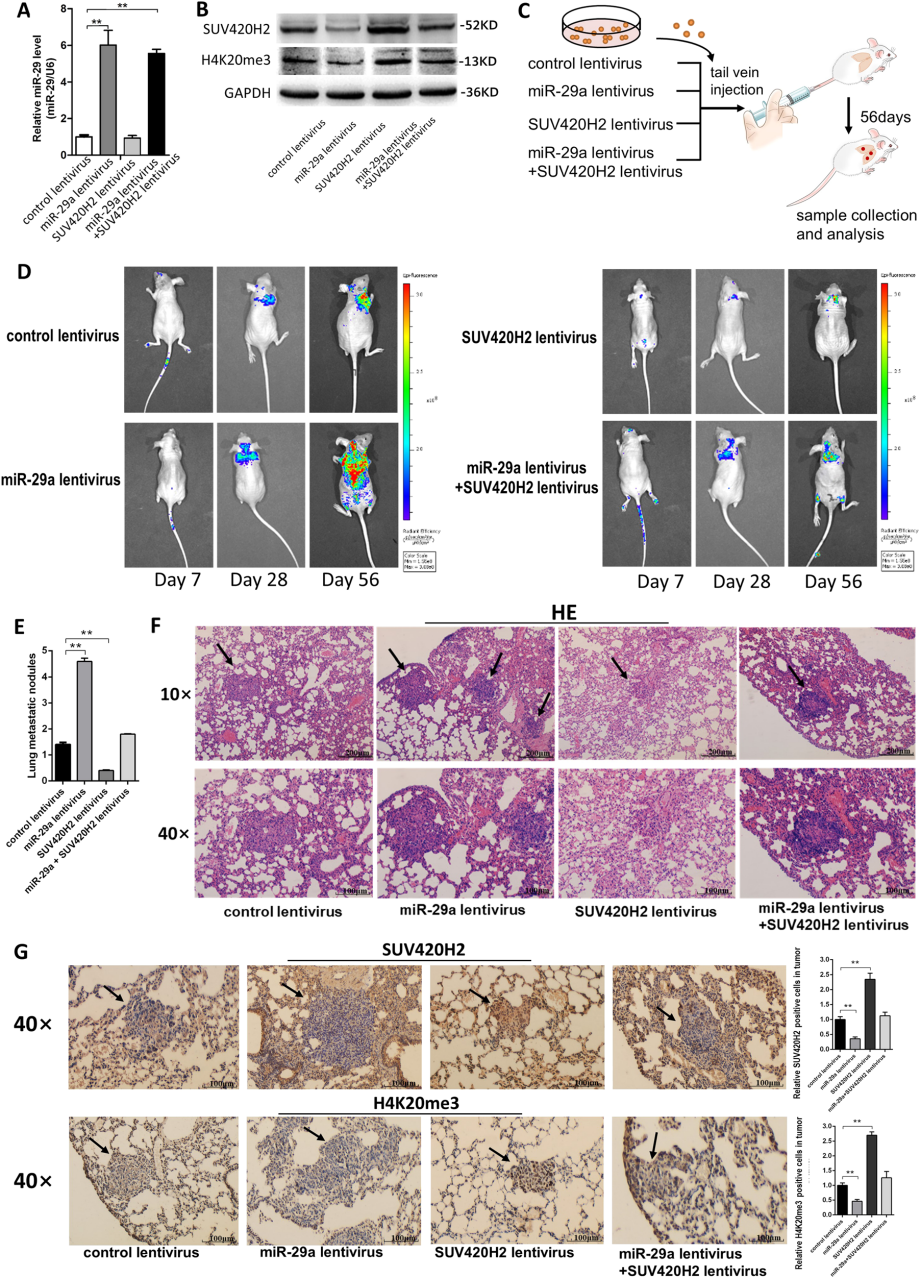
Effects of SUV420H2-targeted miR-29a on the lung colonization of MCF-7 cells xenografts in mice. **a**, **b** miR-29a levels (**a**), SUV420H2 protein and H4K20me3 levels (**b**) in MCF-7 cells transfected with either the control lentivirus, miR-29a lentivirus, SUV420H2 lentivirus or miR-29a lentivirus plus SUV420H2 lentivirus. **c** Experimental design. Immunocompromised mice were injected through tail vein with MCF-7 cells transfected with either the control lentivirus, miR-29a lentivirus, SUV420H2 lentivirus or miR-29a lentivirus plus SUV420H2 lentivirus. **d** Representative BLI images of four groups. The BLI was performed on days 7, 28, and 56 after injection. The intensity of BLI is represented by the color. **e** The numbers of tumor nodules in the lungs. Results were derived from five mice in each group. **f**, **g** Mouse lungs were subjected to H&E staining (**f**) and immunohistochemical staining for SUV420H2 and H4K20me3 (**g**), respectively. **P < 0.01

Finally, we investigated the role of miR-29a in mediating breast cancer cells metastasis in the mouse model. We first successfully achieved four types of modified MCF-7 cell lines: cells infected with control lentivirus, cells stably transfected with miR-29a lentivirus, cells stably transfected with SUV420H2 lentivirus, cells stably co-transfected with miR-29a, and SUV420H2 lentiviruses (Fig. 7a, b). Subsequently, we injected these four modified MCF-7 cell lines into female nude mice (6 weeks, 22–24 g) through the tail vein (Fig. 7c). The metastasis was assessed by bioluminescent imaging (BLI) on days 7, 28, and 56 after implantation. Figure 7d showed that the GFP-labeled migrating cells were mainly distributed in the lungs of mice. The fluorescent intensities of lung were significantly stronger in miR-29a-overexpressing MCF-7 cells group and weaker in SUV420H2-overexpressing MCF-7 cells group compared to the control groups. Likewise, SUV420H2 overexpression can attenuate the metastasis of MCF-7 cells caused by miR-29a-overexpression. After 8 weeks, whole lung tissues were harvested and the numbers of macroscopically visible tumor nodules on the lung surface were counted (Fig. 7e). Mouse lung tissues were also sectioned for H&E staining to evaluate tumor metastasis or to immunohistochemical staining for detecting SUV420H2 and H4K20me3 expression. In mice injected with miR-29a-overexpressing MCF-7 cells, 3–5 different sized tumors with clear boundaries (arrows) were found in the lungs, while only 1–2 smaller tumor masses were scattered in the lungs of control group, and less cell mitosis was showed in control group than 29a-overexpressing group. In mice injected with SUV420H2-overexpressing MCF-7 cells, few tumor masses could be seen in the lungs, while the sizes or number of tumors in the SUV420H2 and miR-29a both overexpressed group were much smaller than the miR-29a-overexpressing group (Fig. 7f). Moreover, SUV420H2 and H4K20me3 labeling revealed that tumors with miR-29a overexpression had lower levels of SUV420H2 and H4K20me3 than the control group, and tumors with both miR-29a and SUV420H2 overexpression exhibited higher levels of SUV420H2 and H4K20me3 than tumors with miR-29a overexpression (Fig. 7g). These results supported the role of miR-29a in promoting breast cancer cells metastasis in mice through suppressing SUV420H2 expression.

## Discussion

**Fig. 8:**
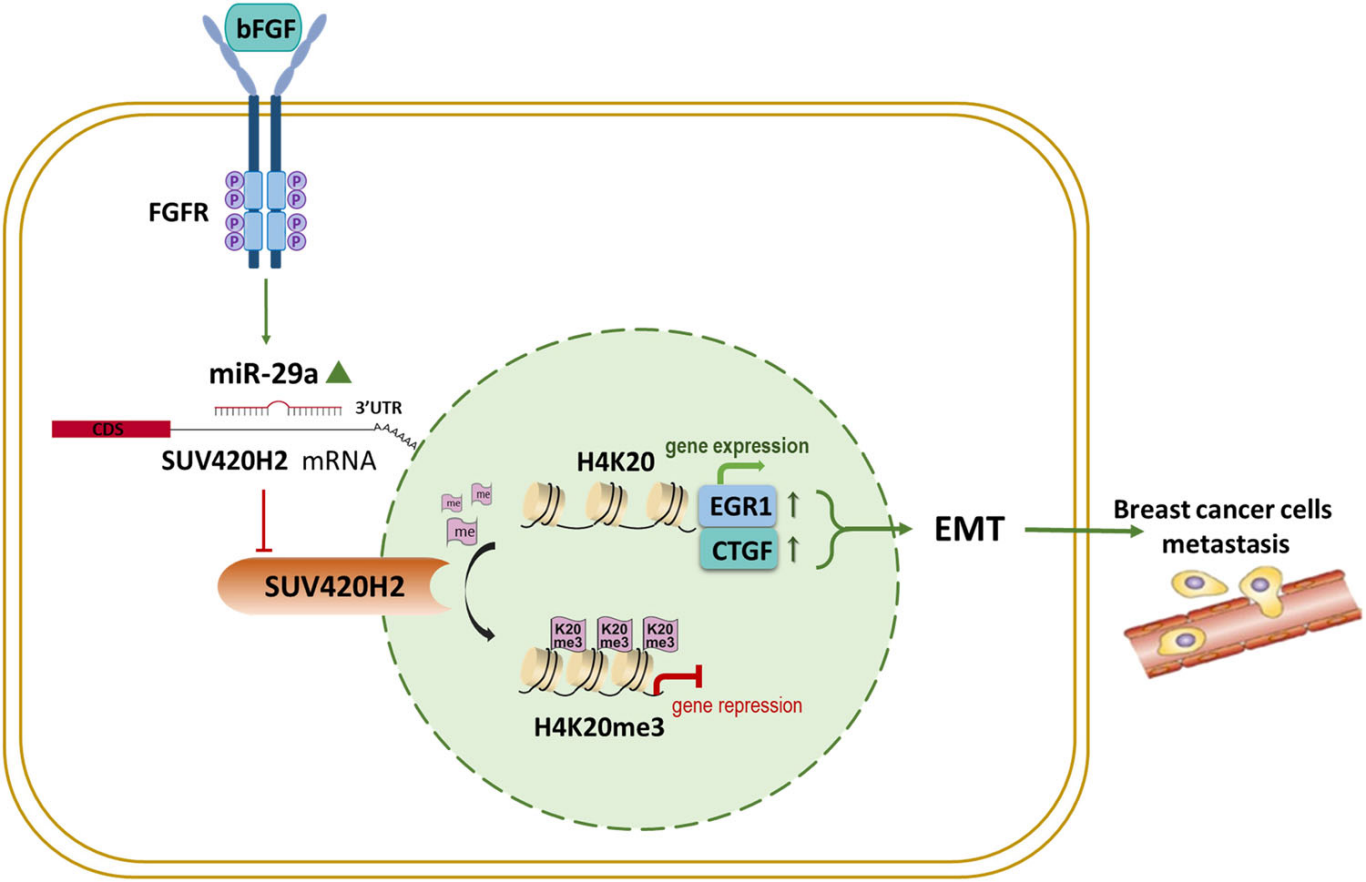
A working model for the role of SUV420H2-targeted miR-29a in breast cancer. During breast tumorigenesis, miR-29a activated by bFGF will directly inhibit SUV420H2 expression, consequently triggering loss of H4K20me3 and EGR1 and CTGF genes expression, which eventually promotes EMT and results in the distal metastasis of human breast cancer

In this study, we report the finding of up-regulation of miR-29a in BCSCs, aggressive breast cancer cell line and breast cancer tissues, which can be induced by bFGF and contributes to the migration and invasion of BCSCs and other breast cancer cells. By suppressing the expression of SUV420H2, which leads to the loss of H4K20me3, miR-29a attenuates the repression of CTGF and EGR1 by H4K20 trimethylation and promotes the EMT progress and metastasis of breast cancer cells. Our study has been summarized in a working model in Fig. 8.

Previously, our lab identified miR-29a among the top 10 up-regulated miRNAs in BCSCs by microRNA microarray^6,36^. Herein, we confirmed that miR-29a levels were not only increased in MCF-7 spheroid cells, CD44+/CD24− MCF-7 cells (BCSCs) and MDA-MB-231 cells compared with MCF-7 cells, but also in breast cancer tissues compared with corresponding distal non-cancerous tissues. bFGF is a growth factor secreted by tumor microvascular endothelial cells (tMVECs) and exists in tumor microenvironment, which has been reported to enhance the invasive potential of cancer cells^19^. It has also been used to culture cancer stem cells (CSCs) due to its participation in stemness maintenance^37^. Here we reported that bFGF signaling promoted the expression of miR-29a, which might partially explain the pro-invasive effect of bFGF. miR-29a has been identified as a tumor suppressor that down-regulated in some cancers such as gastric cancer^38^, pancreatic cancer^39^ and prostate cancer^40^. However, up-regulation miR-29a was detected in some other cancers^21,41^, including breast cancer. These conflicting reports regarding the roles of miR-29a in different cancer types have left poorly understood, especially in BCSCs and breast cancer cells.

SUV420H2 specifically trimethylates Lys-20 of histone H4 causing transcriptional repression of associated genes^10,12,42^, and loss of histone H4K20 trimethylation predicted poor prognosis in breast cancer and was associated with invasive activity^14,15^, suggesting that SUV420H2 and H4K20 trimethylation were involved in the progression of metastatic breast cancer. However, few studies provide the underlying mechanism for the phenomenon or the regulation of SUV420H2 in breast cancer. In this study, we first demonstrated that miR-29a targeted SUV420H2 and caused the loss of H4K20me3 by directly binding the 3′-UTR of the SUV420H2 mRNA, which was required for breast cancer cells migration and invasion both in vitro and in vivo. Our results demonstrated that miR-29a functioned as an oncogene in breast cancer by targeting SUV420H2 for the first time. Aberrant epigenetic alterations in the genome including DNA methylation, histone modification, non-coding RNA expression, and chromatin remodeling, play significant roles in breast cancer development^43^ and BCSC genes turn on^44^. Methyltransferase such as DNA methyltransferases (DNMTs), demethylase such as Ten eleven translocation (TET) enzymes, histone-modifying enzymes such as histone lysine-specific demethylase 1 (LSD1)^45^, and zeste homolog 2(EZH2)^46^ are important regulators associated with epigenetic silencing of tumor suppressor genes. Interestingly, miR-29a has been shown to target various types of epigenetics regulators in cancer cells. miR-29a down-regulates DNMT3A and −3B in lung cancer cells^47^ and hepatocytes^48^ while targets TET in malignant hematopoietic cells^49^, hepatocellular carcinoma cells^50^, breast cancer cells and embryonic stem cells^51,52^. These findings together with our data indicate that miR-29a-targeting epigenetics regulators was a critical approach to facilitate tumorigenesis.

EMT is often thought to accompany the progression of early cancer lesions to invasive malignancies and eventually metastasis^53,54^. We revealed that miR-29a contributed to the migration and invasion of breast cancer cells via triggering the EMT-promoting genes expressions downstream of SUV420H2 and H4K20me3. miR-29a was shown to play a role in extracellular matrix (ECM) production and fibrosis by directly regulating extracellular matrix proteins including collagens, integrins, and metallopeptidases (MMPs) in hepatic stellate cells^55^, lung fibroblast cells^56^, and retinal epithelial cells^57^, separately, which might also contribute to the miR-29a-promoted EMT process in breast cancer.

In conclusion, the bFGF-induced miR-29a-SUV420H2 axis generated a significant effect on breast cancer cells EMT, migration and invasion in vitro and tumor dissemination in vivo, which provided a potential target for breast cancer therapy in the future.

### Data availability

All data generated or analyzed during this study have been included in this published article and its supplementary information files.

## Supplementary information

Supplementary Figure S1

Supplementary Figure S2

Supplementary Table S1

Supplementary Table S2

Supplementary Table S3

Supplementary Table S4

Supplementary Table S5

supplemental figure legends
