# Correction: miR-29a contributes to breast cancer cells epithelial–mesenchymal transition, migration, and invasion via downregulating histone H4K20 trimethylation through directly targeting SUV420H2

**DOI:** 10.1038/s41419-019-2096-x

**Published:** 2019-11-12

**Authors:** You Wu, Wanyue Shi, Tingting Tang, Yidong Wang, Xin Yin, Yanlin Chen, Yanfeng Zhang, Yun Xing, Yumeng Shen, Tiansong Xia, Changying Guo, Yi Pan, Liang Jin

**Affiliations:** 1 State Key Laboratory of Natural Medicines, Jiangsu Key Laboratory of Druggability of Biopharmaceuticals, School of life Science and Technology, China Pharmaceutical University, 24 Tongjiaxiang, Nanjing, Jiangsu province China; 2 Department of Breast Surgery, Breast Disease Center of Jiangsu Province, First Affiliated Hospital of Nanjing Medical University, 300 Guangzhou Road, Nanjing, Jiangsu province China

**Keywords:** Cancer stem cells, Cell invasion

**Correction to: Cell Death and Disease**

10.1038/s41419-019-1437-0, published online 21 February 2019.

Since online publication of this article, the authors noticed that there was an error in the images used to compile Fig. 4d. The corrected image is provided below. The authors apologise for any inconvenience caused.

**Fig. 4:**
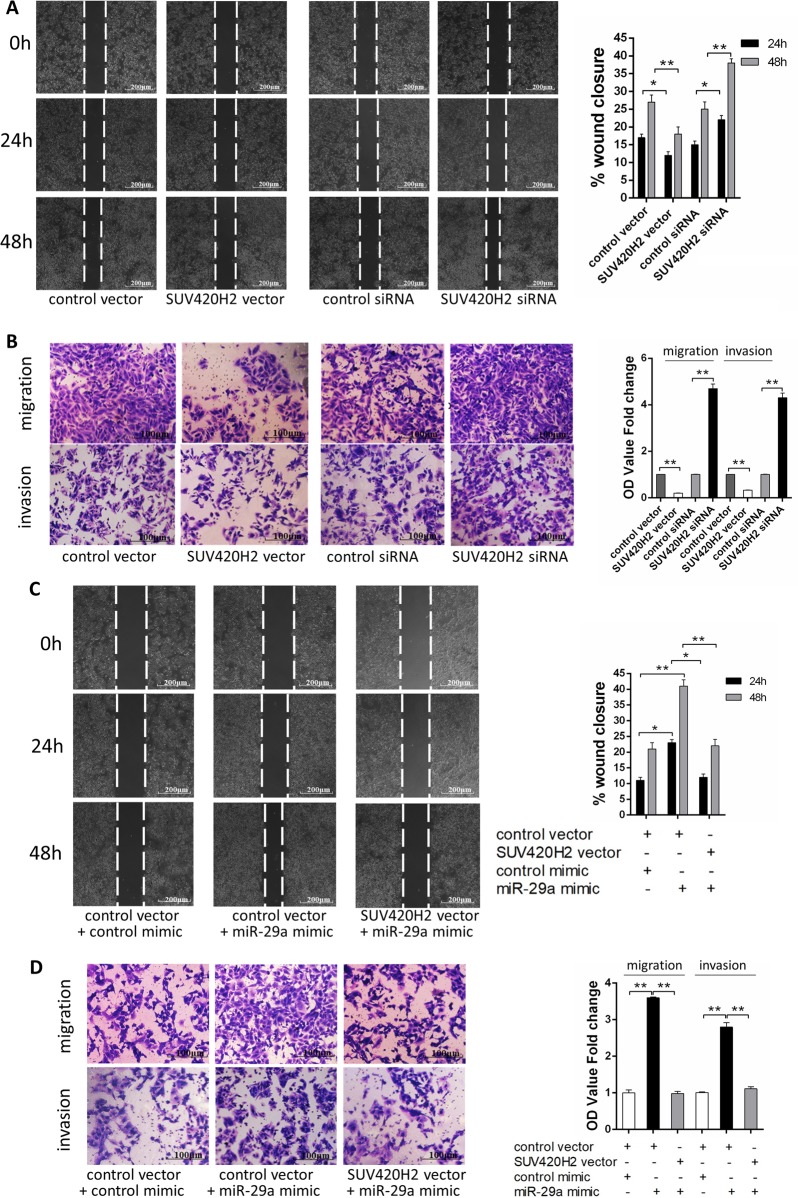
miR-29a promotes breast cancer cells migration and invasion via targeting SUV420H2. **a**, **b**. Migration and invasion of MCF-7 cells transfected with either the control vector, SUV420H2 vector, control siRNA, or SUV420H2 siRNA as indicated, detected by wound healing assay (**a**), transwell migration assay, and transwell invasion assay (**b**). **c**, **d** Migration and invasion of MCF-7 cells transfected with either the control mimic plus control vector, miR-29a mimic plus control vector or miR-29a mimic plus SUV420H2 vector detected by wound healing assay (**c**), transwell migration assay and transwell invasion assay (**d**). *P < 0.05; **P < 0.01

