# Supplementary material for: miR-29a contributes to breast cancer cells epithelial–mesenchymal transition, migration, and invasion via down-regulating histone H4K20 trimethylation through directly targeting SUV420H2
Source: Cell Death Dis. 2019 Feb 21;10(3):176. doi: 10.1038/s41419-019-1437-0 (PMC6385178; doi:10.1038/s41419-019-1437-0)
Supplement: Supplementary file 3 — Supplementary Table S1 [file 41419_2019_1437_MOESM3_ESM.docx]

**Table S1.** The primers used in Real-time PCR.

| **Gene Name** | **Forward Primer** | **Reverse Primer** |
| --- | --- | --- |
| ***SUV420H2*** | CGCCTTCATCAACCATGACTG | GCCGTAGAAGCATGTCACC |
| ***EGR1*** | GGTCAGTGGCCTAGTGAGC | GTGCCGCTGAGTAAATGGGA |
| ***FOS*** | GGGGCAAGGTGGAACAGTTAT | CCGCTTGGAGTGTATCAGTCA |
| ***FOSB*** | GCTGCAAGATCCCCTACGAAG | ACGAAGAAGTGTACGAAGGGTT |
| ***JUN*** | TCCAAGTGCCGAAAAAGGAAG | CGAGTTCTGAGCTTTCAAGGT |
| ***DUSP6*** | GAAATGGCGATCAGCAAGACG | CGACGACTCGTATAGCTCCTG |
| ***CTGF*** | ACCGACTGGAAGACACGTTTG | CCAGGTCAGCTTCGCAAGG |
| ***GAPDH*** | GAGTCAACGGATTTGGTCGT | TTGATTTTGGAGGGATCTCG |
