# Supplementary material for: miR-29a contributes to breast cancer cells epithelial–mesenchymal transition, migration, and invasion via down-regulating histone H4K20 trimethylation through directly targeting SUV420H2
Source: Cell Death Dis. 2019 Feb 21;10(3):176. doi: 10.1038/s41419-019-1437-0 (PMC6385178; doi:10.1038/s41419-019-1437-0)
Supplement: Supplementary file 4 — Supplementary Table S2 [file 41419_2019_1437_MOESM4_ESM.docx]

**Supplementary Table S2.** Patients’ Characteristics

| Patients’ characteristics | | | | |
| --- | --- | --- | --- | --- |
| Case No. | Clinical History | Gender | Age (years) | TNM Stage |
| BC #1 | IDC | Female | 54 | II-III |
| BC #2 | IDC | Female | 49 | III |
| BC #3 | IDC | Female | 61 | III |
| BC #4 | IDC | Female | 45 | II-III |
| BC #5 | IDC | Female | 53 | II-III |
| BC #6 | IDC | Female | 52 | II-III |
| BC #7 | IDC | Female | 65 | II-III |
| BC #8 | IDC | Female | 47 | III |
| BC #9 | IDC | Female | 58 | III |
| BC #10 | IDC | Female | 54 | II-III |
| BC #11 | IDC | Female | 47 | II |
| BC #12 | IDC | Female | 45 | II-III |

IDC：invasive ductal carcinoma
