# Supplementary material for: miR-29a contributes to breast cancer cells epithelial–mesenchymal transition, migration, and invasion via down-regulating histone H4K20 trimethylation through directly targeting SUV420H2
Source: Cell Death Dis. 2019 Feb 21;10(3):176. doi: 10.1038/s41419-019-1437-0 (PMC6385178; doi:10.1038/s41419-019-1437-0)
Supplement: Supplementary file 5 — Supplementary Table S3 [file 41419_2019_1437_MOESM5_ESM.docx]

**Table S3.** The functions of 114 genes predicted to be miR-29a targets by all the three algorithms (TargetScan, miRanda and PicTar).

| **Gene Name** | **Tumor activator** | **Tumor suppressor** | **Tumor activator or suppressor** | **miR-29a target** | **Related to tumor metastasis** | **Function unknown** |
| --- | --- | --- | --- | --- | --- | --- |
| COL1A1 | √ |  |  | √ | √ |  |
| COL3A1 | √ |  |  | √ | √ |  |
| TRIB2 | √ |  |  |  | √ |  |
| SUV420H2[^1^](#_ENREF_1)^,^ [^2^](#_ENREF_2) |  | √ |  |  | √ |  |
| COL1A2 |  |  | √ |  |  |  |
| EIF4E2 | √ |  |  |  | √ |  |
| HBP1 |  | √ |  | √ | √ |  |
| IFI30 |  |  |  |  |  | √ |
| ADAMTS9 |  | √ |  |  | √ |  |
| COL2A1 |  |  |  |  |  | √ |
| COL5A3 |  |  |  |  |  | √ |
| IREB2 | √ |  |  |  |  |  |
| NASP | √ |  |  |  | √ |  |
| COL4A5 | √ |  |  |  |  |  |
| COL4A1 |  |  |  | √ |  | √ |
| SPARC |  |  | √ | √ | √ |  |
| PMP22 |  |  | √ | √ | √ |  |
| PPIC | √ |  |  |  |  |  |
| GPR37 | √ |  |  |  | √ |  |
| HIF3A |  |  | √ |  | √ |  |
| COL5A2 |  |  |  |  |  | √ |
| COL7A1 |  |  |  |  |  | √ |
| COL9A1 |  |  |  |  |  | √ |
| SESTD1 |  |  |  |  |  | √ |
| ZBTB5 |  |  |  |  |  | √ |
| ZNF282 | √ |  |  |  |  |  |
| COL19A1 |  |  |  |  |  | √ |
| HAS3 |  |  | √ | √ | √ |  |
| NAV1 |  |  |  |  |  | √ |
| TRAF4 | √ |  |  | √ | √ |  |
| ZFP36L1 |  | √ |  |  |  |  |
| PTEN |  | √ |  | √ | √ |  |
| RNF39 |  |  |  |  |  | √ |
| ELOVL4 |  |  |  |  |  | √ |
| FEM1B |  | √ |  |  |  |  |
| KCTD5 |  |  |  |  |  | √ |
| NAV3 |  | √ |  |  | √ |  |
| TFEB |  |  |  |  |  | √ |
| LSM11 |  |  |  |  |  | √ |
| FOXJ2 |  |  | √ |  | √ |  |
| MYBL2 | √ |  |  | √ | √ |  |
| ENTPD7 |  |  |  |  |  | √ |
| ABCB6 |  |  |  |  |  | √ |
| BMF |  | √ |  | √ | √ |  |
| TNFRSF1A | √ |  |  | √ | √ |  |
| TFAP2C |  |  | √ |  | √ |  |
| COL15A1 |  |  |  |  |  | √ |
| DNMT3B |  |  | √ | √ | √ |  |
| PDGFC |  |  | √ | √ | √ |  |
| MFAP3 |  |  |  |  |  | √ |
| CAV2 | √ |  |  | √ | √ |  |
| FSTL1 |  |  | √ | √ | √ |  |
| ADAMTS7 |  |  |  | √ |  | √ |
| GNG12 |  |  |  |  |  | √ |
| KCTD3 |  |  |  |  |  | √ |
| COL6A3 |  | √ |  |  | √ |  |
| TLL1 |  |  |  |  |  | √ |
| RAB30 |  |  |  |  |  | √ |
| MCL1 | √ |  |  | √ | √ |  |
| PPP1R3D |  |  |  |  |  | √ |
| COL22A1 |  |  |  |  |  | √ |
| DGKD |  |  |  |  |  | √ |
| ELF2 |  |  | √ |  |  |  |
| SCAMP5 |  |  |  |  |  | √ |
| DGKH |  |  |  |  |  | √ |
| RAPGEFL1 |  |  |  |  |  | √ |
| LPL |  |  |  |  |  | √ |
| GLIS2 |  |  |  |  |  | √ |
| PALM |  |  |  |  |  | √ |
| SETDB1 | √ |  |  | √ | √ |  |
| TP53INP2 |  |  |  |  |  | √ |
| CALM3 |  |  |  |  |  | √ |
| AKT3 |  |  | √ | √ | √ |  |
| EHD2 |  | √ |  |  | √ |  |
| CCNT2 | √ |  |  |  | √ |  |
| SCHIP1 |  |  |  |  |  | √ |
| EML5 |  |  |  |  |  | √ |
| BACE1 |  |  |  | √ |  | √ |
| E2F7 | √ |  |  |  | √ |  |
| PLAG1 | √ |  |  |  | √ |  |
| PRKG1 |  |  |  |  |  | √ |
| CX3CL1 | √ |  |  |  | √ |  |
| FKBP4 |  |  |  |  |  | √ |
| MTMR4 |  |  |  |  |  | √ |
| LAMC1 | √ |  |  | √ | √ |  |
| NAV2 |  |  |  |  |  | √ |
| SVIL | √ |  |  |  |  |  |
| PHC1 |  |  |  |  |  | √ |
| SLK |  |  |  |  |  | √ |
| SLC16A7 | √ |  |  |  |  |  |
| OXTR |  |  |  |  |  | √ |
| TP53INP1 |  | √ |  | √ | √ |  |
| PLXNA1 |  |  |  |  |  | √ |
| PCDHA1 |  |  |  |  |  | √ |
| RLF |  |  |  |  |  | √ |
| GOLGA7 |  |  |  |  |  | √ |
| SLC31A1 |  |  |  |  |  | √ |
| PCDHA7 |  |  |  |  |  | √ |
| PCDHA5 |  |  |  |  |  | √ |
| PCDHA12 |  |  |  |  |  | √ |
| PCDHA6 |  |  |  |  |  | √ |
| PCDHA8 |  |  |  |  |  | √ |
| PCDHA10 |  |  |  |  |  | √ |
| PCDHA11 |  |  |  |  |  | √ |
| PCDHA3 |  |  |  |  |  | √ |
| PCDHA2 |  |  |  |  |  | √ |
| PCDHA4 |  |  |  |  |  | √ |
| PCDHA9 |  |  |  |  |  | √ |
| PCDHA13 |  |  |  |  |  | √ |
| PCDHAC1 |  |  |  |  |  | √ |
| PCDHAC2 |  |  |  |  |  | √ |
| DICER1 |  | √ |  |  | √ |  |
| ETV6 |  |  |  |  |  | √ |
| CFL2 | √ |  |  |  |  |  |

**Reference**

1. Shinchi, Y. et al. SUV420H2 suppresses breast cancer cell invasion through down regulation of the SH2 domain-containing focal adhesion protein tensin-3. *Exp. Cell Res.* **334,** 90-99 (2015).

2. Yokoyama, Y. et al. Loss of histone H4K20 trimethylation predicts poor prognosis in breast cancer and is associated with invasive activity. *Breast Cancer Res* **16,** (2014).
