# Supplementary material for: miR-29a contributes to breast cancer cells epithelial–mesenchymal transition, migration, and invasion via down-regulating histone H4K20 trimethylation through directly targeting SUV420H2
Source: Cell Death Dis. 2019 Feb 21;10(3):176. doi: 10.1038/s41419-019-1437-0 (PMC6385178; doi:10.1038/s41419-019-1437-0)
Supplement: Supplementary file 7 — Supplementary Table S5 [file 41419_2019_1437_MOESM7_ESM.docx]

**Table S5.** The primers used in ChIP-qPCR.

| **Gene Name** | **Forward Primer** | **Reverse Primer** | **Amplicon size (bases)** |
| --- | --- | --- | --- |
| ***EGR1*** | ACAGCACCTCCTCTGGATTCAGAGC | ACACTCCTCACCCCGAGCCTG | 222 |
| ***CTGF*** | GAGAGTTTCAAGAGCCTATAGCCTC | ACCACTCCTGATTCATATCATTTAT | 221 |
