# Supplementary material for: miR-29a contributes to breast cancer cells epithelial–mesenchymal transition, migration, and invasion via down-regulating histone H4K20 trimethylation through directly targeting SUV420H2
Source: Cell Death Dis. 2019 Feb 21;10(3):176. doi: 10.1038/s41419-019-1437-0 (PMC6385178; doi:10.1038/s41419-019-1437-0)
Supplement: Supplementary file 8 — supplemental figure legends [file 41419_2019_1437_MOESM8_ESM.docx]

**Figure S1. miR-29a promotes MDA-MB-231 cells migration and invasion through targeting SUV420H2 and down-regulation of H4K20me3. (A, B)** SUV420H2 and H4K20me3 protein levels (A) and SUV420H2 mRNA levels (B) in MDA-MB-231 cells transfected with control inhibitor or miR-29a inhibitor. **(C)** SUV420H2 and H4K20me3 protein levels in MDA-MB-231 cells transfected with either the control inhibitor plus control siRNA, miR-29a inhibitor plus control siRNA or miR-29a inhibitor plus SUV420H2 siRNA. **(D)** Migration and invasion of MDA-MB-231 cells transfected with control vector or SUV420H2 vector. **(E)** Migration and invasion of MDA-MB-231 cells transfected with either the control inhibitor plus control siRNA, miR-29a inhibitor plus control siRNA or miR-29a inhibitor plus SUV420H2 siRNA. **P<0.01.

**Figure S2. H4K20me3 levels in breast cancer cells and breast cancer tissues. (A)** H4K20me3 levels in MCF-7 cells, MCF-7 spheroid cells and CD44+/CD24- MCF-7 cells. (B) H4K20me3 levels in MCF-7 cells and MDA-MB-231 cells. **(C)** **H4K20me3** levels in 12 pairs of human breast cancer tissues (Cancer) and corresponding distal non-cancerous tissues (Normal). **P<0.01.
