# Supplementary material for: miR-29a contributes to breast cancer cells epithelial–mesenchymal transition, migration, and invasion via down-regulating histone H4K20 trimethylation through directly targeting SUV420H2
Source: Cell Death Dis. 2019 Feb 21;10(3):176. doi: 10.1038/s41419-019-1437-0 (PMC6385178; doi:10.1038/s41419-019-1437-0)
Supplement: Supplementary file 6 — Supplementary Table S4 [file 41419_2019_1437_MOESM6_ESM.pdf]

Table.S4 Original HTS data sets for mRNAs, lncRNAs and miRNAs

id	logFC	logCPM	PValue	FDR	GeneSymbol	KO
ENSG00000120738	-8.697006	5.758244	6.56E-17	3.79E-12	EGR1	K09203
ENSG00000125740	-6.777072	6.596969	8.26E-15	2.39E-10	FOSB	K09029
ENSG00000170345	-5.936364	8.032833	3.55E-13	6.84E-09	FOS	K04379
ENSG00000133247	5.807581	9.677545	4.97E-13	7.18E-09	SUV420H2	K11429
ENSG00000264063	5.841717	3.602726	9.35E-11	1.08E-06	MIR3687	_
ENSG00000264462	5.326716	3.450118	7.36E-10	7.09E-06	MIR3648	_
ENSG00000139318	-5.600219	3.327065	3.20E-09	2.64E-05	DUSP6	K04459
ENSG00000118523	-4.244039	5.235228	3.92E-08	0.000283	CTGF	K06827
ENSG00000259884	-6.068432	2.34902	5.46E-08	0.000351	RP11-1100L3.8	_
ENSG00000177606	-3.700662	7.245693	4.30E-07	0.002484	JUN	K04448
ENSG00000185633	3.267867	4.582454	8.15E-06	0.040627	NDUFA4L2	K03948
ENSG00000105141	3.656326	2.563451	8.44E-06	0.040627	CASP14	K04401
ENSG00000172927	-3.855786	2.424798	1.36E-05	0.060534	MYEOV	_
ENSG00000107159	3.169387	4.123207	1.63E-05	0.06721	CA9	K01672
ENSG00000103888	3.792367	1.770133	1.79E-05	0.068799	KIAA1199	K19031
ENSG00000169247	-3.58845	1.538828	0.000228	0.76169	SH3TC2	_
ENSG00000143847	2.876429	2.273319	0.000229	0.76169	PPFIA4	_
ENSG00000136244	-2.594806	5.386732	0.000237	0.76169	IL6	K05405
ENSG00000271334	-4.437644	0.923403	0.000278	0.763163	CTD-2078B5.2	_
ENSG00000111684	2.638303	3.466878	0.000281	0.763163	LPCAT3	K13515
ENSG00000090539	2.628442	3.667742	0.00029	0.763163	CHRD	K04657
ENSG00000137411	7.525734	-0.201984	0.000291	0.763163	VARS2	K01873
ENSG00000168398	4.176532	0.210671	0.000385	0.967705	BDKRB2	K03916
ENSG00000212296	-7.623848	0.430946	0.000622	1	SNORD72	_
ENSG00000183668	2.782004	1.563753	0.00064	1	PSG9	K06500
ENSG00000135919	-2.349955	5.336969	0.000782	1	SERPINE2	K16643
ENSG00000259001	-2.39362	3.729735	0.000983	1	RPPH1	_
ENSG00000171617	-3.635757	1.077053	0.001	1	ENC1	K10462
ENSG00000104419	2.238523	9.15477	0.00106	1	NDRG1	K18266
ENSG00000162006	2.350925	3.135771	0.001205	1	MSLNL	_
ENSG00000147400	2.356563	3.048054	0.00124	1	CETN2	K10840
ENSG00000130558	2.583425	1.78425	0.00125	1	OLFM1	_
ENSG00000099998	-3.174474	1.195342	0.001348	1	GGT5	K18592
ENSG00000102221	-6.975945	-0.09654	0.001409	1	JADE3	_
ENSG00000185955	4.284764	-0.498289	0.001409	1	C7orf61	_
ENSG00000229869	4.284764	-0.498289	0.001409	1	RP11-363N22.2	_
ENSG00000270141	4.284764	-0.498289	0.001409	1	TERC	_
ENSG00000271855	4.284764	-0.498289	0.001409	1	RP11-214N9.1	_
ENSG00000124102	-2.866892	1.588263	0.001545	1	PI3	_
ENSG00000127528	-2.395977	3.041663	0.001624	1	KLF2	K17845
ENSG00000258486	2.191572	4.555525	0.001657	1	RN7SL1	_
ENSG00000127129	2.346137	2.553597	0.001661	1	EDN2	K16367
ENSG00000118160	3.837144	-0.075214	0.001744	1	SLC8A2	K05849
ENSG00000267909	3.837144	-0.075214	0.001744	1	CCDC177	_
ENSG00000165449	-3.983834	0.548515	0.001774	1	SLC16A9	K08186
ENSG00000142871	-2.12509	9.574213	0.001806	1	CYR61	K06829
ENSG00000214049	2.574721	1.496551	0.001831	1	UCA1	_
ENSG00000111348	-2.512138	2.203	0.001909	1	ARHGDIB	K12462
ENSG00000166866	-6.906128	-0.151709	0.001996	1	MYO1A	K10356
ENSG00000267102	-6.906128	-0.151709	0.001996	1	RP11-686D22.7	_
ENSG00000148677	-2.443119	2.496856	0.002132	1	ANKRD1	_
ENSG00000248323	2.32121	2.240121	0.002347	1	LUCAT1	_
ENSG00000119922	-2.07312	7.342721	0.002393	1	IFIT2	_
ENSG00000183604	-2.360795	2.622775	0.002637	1	RP11-347C12.2	K08873
ENSG00000197561	3.073855	0.1776	0.002812	1	ELANE	K01327
ENSG00000101230	-6.755461	-0.26948	0.002864	1	ISM1	_

ENSG00000102271	-6.755461	-0.26948	0.002864	1	KLHL4	K10442
ENSG00000122877	-6.755461	-0.26948	0.002864	1	EGR2	K12496
ENSG00000181773	-2.681576	1.67202	0.002897	1	GPR3	K04311
ENSG00000168497	-2.956987	1.019106	0.00342	1	SDPR	K19387
ENSG00000132938	2.524765	1.235391	0.003561	1	MTUS2	_
ENSG00000272240	2.986824	0.11002	0.003568	1	RP5-855D21.1	_
ENSG00000244405	-1.999542	5.36455	0.003709	1	ETV5	K15593
ENSG00000078401	-2.097854	3.4481	0.003938	1	EDN1	K16366
ENSG00000105929	-2.375526	1.800058	0.004333	1	ATP6V0A4	K02154
ENSG00000137801	-1.960391	5.185914	0.004581	1	THBS1	K16857
ENSG00000265749	-2.897075	0.971111	0.00475	1	RP11-849F2.5	_
ENSG00000139289	-1.946287	5.278541	0.00483	1	PHLDA1	K15696
ENSG00000057149	2.014816	3.340603	0.004912	1	SERPINB3	K13963
ENSG00000081041	-2.081853	3.095187	0.005032	1	CXCL2	K05505
ENSG00000200534	-2.447645	1.484869	0.005074	1	SNORA33	_
ENSG00000172379	1.902223	5.761522	0.005234	1	ARNT2	K15589
ENSG00000228799	3.517172	-0.326535	0.005479	1	AC113607.2	_
ENSG00000165887	-2.036709	3.263776	0.005654	1	ANKRD2	_
ENSG00000177685	2.52509	0.489568	0.00576	1	EFCAB4A	K17199
ENSG00000101276	2.894204	0.039653	0.005837	1	SLC52A3	K17985
ENSG00000088827	6.625678	-0.938041	0.006149	1	SIGLEC1	K06548
ENSG00000262652	6.625678	-0.938041	0.006149	1	RP13-638C3.2	_
ENSG00000099251	-6.495095	-0.468486	0.006149	1	HSD17B7P2	K13373
ENSG00000198237	-6.495095	-0.468486	0.006149	1	RP11-98J23.2	K01195
ENSG00000206885	-6.495095	-0.468486	0.006149	1	SNORA75	_
ENSG00000228323	-6.495095	-0.468486	0.006149	1	AC008753.6	_
ENSG00000253130	-6.495095	-0.468486	0.006149	1	CTD-3023L14.2	_
ENSG00000188517	3.829323	-0.831149	0.006149	1	COL25A1	K16617
ENSG00000196337	3.829323	-0.831149	0.006149	1	CGB7	_
ENSG00000198074	3.829323	-0.831149	0.006149	1	AKR1B10	K00011
ENSG00000260139	3.829323	-0.831149	0.006149	1	CSPG4P13	K08115
ENSG00000223401	2.341458	0.835963	0.006382	1	RP11-211G3.2	_
ENSG00000216775	2.26226	1.246239	0.006447	1	RP1-152L7.5	_
ENSG00000104043	1.907971	3.529101	0.006583	1	ATP8B4	K01530
ENSG00000170848	2.344655	1.092615	0.006733	1	PSG6	K06500
ENSG00000266651	-2.162959	2.166562	0.00675	1	RP11-138I1.3	_
ENSG00000225431	1.925387	3.111864	0.00737	1	AP001626.1	_
ENSG00000259621	3.392529	-0.419302	0.007447	1	RP11-654A16.1	_
ENSG00000131018	-3.611481	0.250392	0.007531	1	SYNE1	K19326
ENSG00000207445	-3.611481	0.250392	0.007531	1	SNORD15B	_
ENSG00000234614	3.190993	-0.092839	0.007531	1	AL450992.2	_
ENSG00000187678	-2.735416	0.842862	0.007925	1	SPRY4	K17385
ENSG00000107731	1.886617	3.196417	0.008033	1	UNC5B	K07521
ENSG00000231924	1.880489	3.434867	0.00828	1	PSG1	K06500
ENSG00000269399	2.28276	0.791669	0.008737	1	CTD-3222D19.7	_
ENSG00000202314	-2.427456	1.236582	0.008776	1	SNORD6	_
ENSG00000143512	1.856236	3.541112	0.008778	1	HHIPL2	_
ENSG00000175832	-1.790744	5.222372	0.008987	1	ETV4	K15592
ENSG00000268927	-6.396701	-0.542071	0.009224	1	FLJ00418	_
ENSG00000272512	2.064057	1.672039	0.00941	1	RP11-54O7.17	_
ENSG00000171522	-1.845386	3.488854	0.009698	1	PTGER4	K04261
ENSG00000119917	-1.745832	6.931421	0.009767	1	IFIT3	K17985
ENSG00000196409	-3.501164	0.163959	0.009775	1	ZNF658	K09228
ENSG00000102575	2.688957	-0.110405	0.009775	1	ACP5	K14379
ENSG00000204052	2.245399	1.016359	0.010042	1	LRRC73	_
ENSG00000185745	-1.735871	7.157495	0.010135	1	IFIT1	K14217
ENSG00000254772	1.803498	3.692464	0.010201	1	EEF1G	K03233
ENSG00000112511	2.590503	0.334087	0.010331	1	PHF1	K11467

ENSG00000240583	2.37374	0.377362	0.010331	1	AQP1	K09864
ENSG00000173702	1.94625	1.889519	0.010333	1	MUC13	K17298
ENSG00000104081	1.84681	2.846423	0.010345	1	BMF	K17460
ENSG00000204949	2.030568	1.713235	0.010635	1	FAM83A-AS1	_
ENSG00000181143	1.748698	4.130725	0.011175	1	MUC16	K16145
ENSG00000196549	-2.628943	0.75944	0.011294	1	MME	K01389
ENSG00000215021	-2.628943	0.75944	0.011294	1	PHB2	K17081
ENSG00000175592	-1.755012	4.515068	0.011325	1	FOSL1	K04502
ENSG00000214274	1.815473	2.822484	0.011778	1	ANG	_
ENSG00000265150	1.75963	3.703117	0.01196	1	RN7SL2	_
ENSG00000079337	-1.876778	2.82133	0.012065	1	RAPGEF3	K08014
ENSG00000178033	-3.381708	0.071438	0.012767	1	FAM26E	_
ENSG00000221491	-3.381708	0.071438	0.012767	1	SNORA34	_
ENSG00000181634	-2.005399	1.803191	0.013893	1	TNFSF15	K05478
ENSG00000110436	6.435118	-1.075553	0.014079	1	SLC1A2	K05613
ENSG00000184825	6.435118	-1.075553	0.014079	1	HIST1H2AH	K11251
ENSG00000221826	6.435118	-1.075553	0.014079	1	PSG3	K06500
ENSG00000248576	6.435118	-1.075553	0.014079	1	RP11-834C11.8	_
ENSG00000181333	-6.291103	-0.619978	0.014079	1	HEPHL1	_
ENSG00000205861	-6.17716	-0.702741	0.014079	1	C1QTNF9B-AS1	_
ENSG00000228360	-6.17716	-0.702741	0.014079	1	RP11-365F18.1	K02921
ENSG00000243777	-6.17716	-0.702741	0.014079	1	RP11-864G5.1	K02991
ENSG00000258424	-6.17716	-0.702741	0.014079	1	RP11-471B22.2	_
ENSG00000265590	-6.17716	-0.702741	0.014079	1	AP000275.65	_
ENSG00000112137	3.638763	-0.958503	0.014079	1	PHACTR1	_
ENSG00000132357	3.638763	-0.958503	0.014079	1	CARD6	K12797
ENSG00000172482	3.638763	-0.958503	0.014079	1	AGXT	K00830
ENSG00000199094	3.638763	-0.958503	0.014079	1	MIR30C2	_
ENSG00000271267	3.638763	-0.958503	0.014079	1	RP11-29H23.7	K02130
ENSG00000135744	3.256091	-0.51742	0.014146	1	AGT	K09821
ENSG00000196415	2.147427	0.82254	0.014761	1	PRTN3	K01350
ENSG00000186832	1.650935	5.489847	0.015104	1	KRT16	K07604
ENSG00000166863	-2.29295	1.131813	0.015186	1	TAC3	K05240
ENSG00000196407	2.138808	0.936477	0.015186	1	THEM5	_
ENSG00000198794	2.138808	0.936477	0.015186	1	SCAMP5	_
ENSG00000120129	-1.626859	8.517028	0.015303	1	DUSP1	K04459
ENSG00000099834	2.508425	0.27324	0.015514	1	CDHR5	K16505
ENSG00000135519	2.508425	0.27324	0.015514	1	KCNH3	K04906
ENSG00000186019	2.291662	0.318378	0.015514	1	AC084219.4	_
ENSG00000105341	1.833944	2.058738	0.015955	1	ATP5SL	_
ENSG00000200087	-2.037469	1.536445	0.016233	1	SNORA73B	_
ENSG00000167701	1.793445	2.360514	0.016382	1	GPT	K00814
ENSG00000108018	1.951434	1.44404	0.016397	1	SORCS1	_
ENSG00000260822	1.77203	2.551949	0.016576	1	GS1-358P8.4	_
ENSG00000272256	-3.318053	0.022615	0.016783	1	RP11-489E7.4	_
ENSG00000206176	2.969997	-0.256746	0.016783	1	AC023490.1	_
ENSG00000143546	2.574232	-0.190662	0.016783	1	S100A8	_
ENSG00000269959	2.574232	-0.190662	0.016783	1	SPACA6P-AS	_
ENSG00000173237	1.628509	5.060519	0.01681	1	C11orf86	_
ENSG00000039068	2.24017	0.616352	0.01681	1	CDH1	K05689
ENSG00000174332	2.24017	0.616352	0.01681	1	GLIS1	K09232
ENSG00000163293	-1.811221	2.361257	0.017284	1	NIPAL1	_
ENSG00000179988	-2.235428	1.08745	0.017485	1	PSTK	K10837
ENSG00000142910	-1.614795	5.305006	0.017941	1	TINAGL1	_
ENSG00000174374	-1.994508	1.503632	0.018081	1	WBSCR16	K16535
ENSG00000185686	1.640957	3.642271	0.018163	1	PRAME	_
ENSG00000128242	2.484987	0.022663	0.018258	1	GAL3ST1	K01019
ENSG00000264577	-1.707655	3.010315	0.018294	1	AC010761.8	_

ENSG00000206073	1.810299	1.8945	0.01835	1	SERPINB4	K13963
ENSG00000136738	1.703005	2.682006	0.019202	1	STAM	K04705
ENSG00000150995	1.567784	6.989814	0.019566	1	ITPR1	K04958
ENSG00000187608	-1.561295	9.550716	0.019606	1	ISG15	K12159
ENSG00000117525	-1.589812	5.121864	0.019663	1	F3	K03901
ENSG00000102158	1.642802	3.493818	0.019713	1	MAGT1	K19478
ENSG00000115828	3.105388	-0.621547	0.019787	1	QPCT	K00683
ENSG00000172602	3.105388	-0.621547	0.019787	1	RND1	K07531
ENSG00000196604	3.105388	-0.621547	0.019787	1	POTEF	K17299
ENSG00000227471	2.022342	0.732391	0.020078	1	AKR1B15	K00011
ENSG00000215190	-2.205784	1.064693	0.020166	1	LINC00680	_
ENSG00000130477	-1.691448	3.217489	0.020193	1	UNC13A	K15293
ENSG00000107554	1.563037	5.403846	0.02092	1	DNMBP	_
ENSG00000187720	-1.587463	4.405372	0.021227	1	THSD4	_
ENSG00000117289	1.540569	7.07241	0.021637	1	TXNIP	_
ENSG00000156097	6.215502	-1.225459	0.0219	1	GPR61	K08414
ENSG00000226958	6.215502	-1.225459	0.0219	1	CTD-2328D6.1	K01979
ENSG00000241781	6.215502	-1.225459	0.0219	1	AL161626.1	_
ENSG00000259524	6.215502	-1.225459	0.0219	1	RP11-461F11.1	_
ENSG00000271948	6.215502	-1.225459	0.0219	1	RP11-242F4.2	_
ENSG00000100298	-6.05344	-0.790999	0.0219	1	APOBEC3H	K18750
ENSG00000109452	-6.05344	-0.790999	0.0219	1	INPP4B	K01109
ENSG00000118194	-6.05344	-0.790999	0.0219	1	TNNT2	K12045
ENSG00000233538	-6.05344	-0.790999	0.0219	1	AC017104.2	K02927
ENSG00000270332	-6.05344	-0.790999	0.0219	1	RP11-82L2.1	_
ENSG00000118946	3.419147	-1.096319	0.0219	1	PCDH17	K16499
ENSG00000026025	-1.419147	-1.096319	0.0219	1	VIM	K07606
ENSG00000159307	3.419147	-1.096319	0.0219	1	SCUBE1	_
ENSG00000176771	3.419147	-1.096319	0.0219	1	NCKAP5	_
ENSG00000234630	3.419147	-1.096319	0.0219	1	LL22NC03-2H8.4	_
ENSG00000235523	3.419147	-1.096319	0.0219	1	RP11-63P12.7	_
ENSG00000236090	3.419147	-1.096319	0.0219	1	LDHAP3	K00016
ENSG00000244480	3.419147	-1.096319	0.0219	1	AC005154.7	K17985
ENSG00000260100	3.419147	-1.096319	0.0219	1	RP11-220I1.5	_
ENSG00000261227	3.419147	-1.096319	0.0219	1	AC140912.1	_
ENSG00000163131	-1.878556	1.706537	0.021972	1	CTSS	K01368
ENSG00000172123	-1.71013	2.694692	0.022191	1	SLFN12	_
ENSG00000204311	-3.25146	-0.028093	0.022208	1	DFNB59	_
ENSG00000257303	-3.25146	-0.028093	0.022208	1	RP11-977G19.11	_
ENSG00000270066	2.845355	-0.344996	0.022208	1	SCARNA2	_
ENSG00000172061	2.449589	-0.27485	0.022208	1	LRRC15	_
ENSG00000272455	1.653159	2.398137	0.022854	1	RP4-758J18.13	_
ENSG00000004799	1.542432	5.270717	0.022964	1	PDK4	K00898
ENSG00000160471	1.919266	0.991199	0.023091	1	COX6B2	_
ENSG00000135114	-1.519845	8.264306	0.023107	1	OASL	K14608
ENSG00000226738	2.378716	-0.05098	0.023239	1	CTA-384D8.31	_
ENSG00000169047	-1.608133	3.634296	0.023298	1	IRS1	K16172
ENSG00000058091	-2.769589	0.384104	0.02363	1	CDK14	K08821
ENSG00000207280	-2.769589	0.384104	0.02363	1	SNORD20	_
ENSG00000261220	2.328774	0.144665	0.02363	1	RP11-629O1.2	_
ENSG00000143365	1.543529	4.241139	0.024082	1	RORC	K08534
ENSG00000013441	1.511169	5.804496	0.025001	1	CLK1	K08823
ENSG00000204060	1.589255	3.157631	0.025326	1	FOXO6	K17847
ENSG00000273301	-1.657906	2.595057	0.026135	1	RP11-314B1.2	_
ENSG00000124602	1.862864	0.951401	0.026429	1	UNC5CL	_
ENSG00000237649	1.752833	1.372889	0.027504	1	KIFC1	K10405
ENSG00000149609	2.937088	-0.732473	0.027969	1	C20orf144	K04613
ENSG00000235774	2.937088	-0.732473	0.027969	1	AC023347.1	_

ENSG00000259925	2.937088	-0.732473	0.027969	1	CTA-363E6.2	_
ENSG00000169248	-2.108555	0.713311	0.028123	1	CXCL11	K12672
ENSG00000138100	2.096369	0.513414	0.028123	1	TRIM54	K10653
ENSG00000170482	2.096369	0.513414	0.028123	1	SLC23A1	K14611
ENSG00000141750	1.947015	0.551073	0.028123	1	STAC2	K17985
ENSG00000107201	-1.473025	6.907786	0.028134	1	DDX58	K12646
ENSG00000111859	-1.483575	5.80497	0.028147	1	NEDD9	K16832
ENSG00000228950	2.103254	0.361901	0.028744	1	AC023137.2	_
ENSG00000235568	2.103254	0.361901	0.028744	1	NFAM1	_
ENSG00000144802	-1.506387	4.504417	0.028773	1	NFKBIZ	K14242
ENSG00000264772	-1.576177	2.912215	0.029562	1	SNORA67	_
ENSG00000160182	-3.181644	-0.080836	0.029588	1	TFF1	_
ENSG00000200913	-3.108276	-0.135784	0.029588	1	SNORD46	_
ENSG00000070808	2.31315	-0.363371	0.029588	1	CAMK2A	K04515
ENSG00000205002	2.31315	-0.363371	0.029588	1	AARD	K17985
ENSG00000213222	2.31315	-0.363371	0.029588	1	AC093724.2	K11518
ENSG00000000971	-2.498302	0.1787	0.029722	1	CFH	K04004
ENSG00000105991	-2.498302	0.1787	0.029722	1	HOXA1	K09301
ENSG00000113916	1.447216	6.442326	0.030956	1	BCL6	K15618
ENSG00000224897	-2.08071	0.969491	0.031233	1	POT1-AS1	_
ENSG00000165795	1.766353	1.23404	0.031424	1	NDRG2	_
ENSG00000198223	-1.680717	1.904531	0.031743	1	CSF2RA	K05066
ENSG00000168333	1.532466	2.693006	0.032386	1	C8orf22	_
ENSG00000066468	1.835355	1.11769	0.032387	1	FGFR2	K05093
ENSG00000273284	1.869356	0.498627	0.03354	1	RP11-888D10.4	_
ENSG00000168685	-1.431992	5.673678	0.033926	1	IL7R	K05072
ENSG00000115155	1.662865	1.310028	0.03402	1	OTOF	_
ENSG00000197142	-1.61591	2.365041	0.034452	1	ACSL5	K01897
ENSG00000137868	-1.91636	1.075805	0.034765	1	STRA6	_
ENSG00000105668	5.956345	-1.390299	0.034783	1	UPK1A	K17347
ENSG00000124134	5.956345	-1.390299	0.034783	1	KCNS1	K04931
ENSG00000135409	5.956345	-1.390299	0.034783	1	AMHR2	K04672
ENSG00000196358	5.956345	-1.390299	0.034783	1	NTNG2	K16359
ENSG00000221643	5.956345	-1.390299	0.034783	1	SNORA77	_
ENSG00000223692	5.956345	-1.390299	0.034783	1	DIP2A-IT1	_
ENSG00000224614	5.956345	-1.390299	0.034783	1	TNK2-AS1	_
ENSG00000225556	5.956345	-1.390299	0.034783	1	C2CD4D	_
ENSG00000234880	5.956345	-1.390299	0.034783	1	LINC00163	_
ENSG00000256282	5.956345	-1.390299	0.034783	1	RP11-504G3.4	K17885
ENSG00000259116	5.956345	-1.390299	0.034783	1	RP11-973N13.4	_
ENSG00000259635	5.956345	-1.390299	0.034783	1	AC100830.3	_
ENSG00000263681	5.956345	-1.390299	0.034783	1	MIR3197	_
ENSG00000269937	5.956345	-1.390299	0.034783	1	RP11-20I23.8	_
ENSG00000031544	-5.918107	-0.885521	0.034783	1	NR2E3	_
ENSG00000137434	-5.918107	-0.885521	0.034783	1	C6orf52	_
ENSG00000137561	-5.918107	-0.885521	0.034783	1	TTPA	K12756
ENSG00000163082	-5.918107	-0.885521	0.034783	1	SGPP2	K04717
ENSG00000163347	-5.918107	-0.885521	0.034783	1	CLDN1	K06087
ENSG00000164199	-5.918107	-0.885521	0.034783	1	GPR98	K18263
ENSG00000214249	-5.918107	-0.885521	0.034783	1	CTAGE11P	_
ENSG00000218052	-5.918107	-0.885521	0.034783	1	ADAMTS7P4	_
ENSG00000234961	-5.918107	-0.885521	0.034783	1	RP11-124N14.3	_
ENSG00000252061	-5.918107	-0.885521	0.034783	1	RNU6-415P	_
ENSG00000263011	-5.918107	-0.885521	0.034783	1	RP11-473M20.11	_
ENSG00000273391	-5.918107	-0.885521	0.034783	1	RP11-634H22.1	_
ENSG00000076258	-5.768753	-0.987252	0.034783	1	FMO4	K00485
ENSG00000215349	-5.768753	-0.987252	0.034783	1	MRPL3P1	K02906
ENSG00000224086	-5.768753	-0.987252	0.034783	1	LL22NC03-86G7.1	_

ENSG00000225213	-5.768753	-0.987252	0.034783	1	RP11-197M22.2	_
ENSG00000225766	-5.768753	-0.987252	0.034783	1	RP11-468E2.9	K11147
ENSG00000226645	-5.768753	-0.987252	0.034783	1	AP006216.10	_
ENSG00000227253	-5.768753	-0.987252	0.034783	1	RP11-166N17.1	_
ENSG00000230650	-5.768753	-0.987252	0.034783	1	AC112229.1	_
ENSG00000234076	-5.768753	-0.987252	0.034783	1	TPRG1-AS1	_
ENSG00000238005	-5.768753	-0.987252	0.034783	1	RP11-443B7.1	_
ENSG00000248498	-5.768753	-0.987252	0.034783	1	ASNSP1	K01953
ENSG00000254872	-5.768753	-0.987252	0.034783	1	RP13-870H17.3	_
ENSG00000255566	-5.768753	-0.987252	0.034783	1	RP11-114F3.5	K17404
ENSG00000258539	-5.768753	-0.987252	0.034783	1	RP11-12J10.3	_
ENSG00000260238	-5.768753	-0.987252	0.034783	1	PMF1-BGLAP	K11546
ENSG00000267174	-5.768753	-0.987252	0.034783	1	CTC-510F12.4	_
ENSG00000272088	-5.768753	-0.987252	0.034783	1	RP11-168F9.2	_
ENSG00000086159	3.15999	-1.246527	0.034783	1	AQP6	K09868
ENSG00000145861	3.15999	-1.246527	0.034783	1	C1QTNF2	K09228
ENSG00000170579	3.15999	-1.246527	0.034783	1	DLGAP1	K15008
ENSG00000181322	3.15999	-1.246527	0.034783	1	NME9	_
ENSG00000184719	3.15999	-1.246527	0.034783	1	RNLS	K18208
ENSG00000228686	3.15999	-1.246527	0.034783	1	RP11-492I21.1	_
ENSG00000229178	3.15999	-1.246527	0.034783	1	AC069513.4	_
ENSG00000235499	3.15999	-1.246527	0.034783	1	AC073046.25	_
ENSG00000239559	3.15999	-1.246527	0.034783	1	RPL37P2	K02922
ENSG00000240616	3.15999	-1.246527	0.034783	1	AD000092.3	K02991
ENSG00000241563	3.15999	-1.246527	0.034783	1	CORT	K05238
ENSG00000243094	3.15999	-1.246527	0.034783	1	RP11-1084A12.1	K02912
ENSG00000252874	3.15999	-1.246527	0.034783	1	Y_RNA	_
ENSG00000257913	3.15999	-1.246527	0.034783	1	RP11-386G11.5	_
ENSG00000270599	3.15999	-1.246527	0.034783	1	RP11-324O2.6	_
ENSG00000272343	3.15999	-1.246527	0.034783	1	RP11-140I16.3	_
ENSG00000272650	3.15999	-1.246527	0.034783	1	RP11-571I18.4	_
ENSG00000131737	-2.299335	0.50689	0.034945	1	KRT34	K07604
ENSG00000133321	-1.495657	3.60817	0.034984	1	RARRES3	K16817
ENSG00000166046	1.602998	1.890875	0.035292	1	TCP11L2	_
ENSG00000035664	1.458472	3.791632	0.035294	1	DAPK2	K08803
ENSG00000138772	-1.41337	6.415189	0.035302	1	ANXA3	K17089
ENSG00000087074	-1.409769	6.199033	0.035736	1	PPP1R15A	K14019
ENSG00000265752	1.634663	1.817782	0.036232	1	RP11-403A21.1	_
ENSG00000273445	1.683016	1.392752	0.036519	1	RP11-1399P15.1	_
ENSG00000010219	2.229797	0.07659	0.036521	1	DYRK4	K18669
ENSG00000272849	2.013034	0.128255	0.036521	1	RP11-347I19.8	_
ENSG00000102452	-1.553444	2.517756	0.037568	1	NALCN	_
ENSG00000136158	-1.4144	5.002863	0.037852	1	SPRY2	K17383
ENSG00000088726	1.76447	0.978603	0.038112	1	TMEM40	_
ENSG00000170298	-2.437338	0.133445	0.038199	1	LGALS9B	K10093
ENSG00000184995	-2.437338	0.133445	0.038199	1	IFNE	K05442
ENSG00000225177	-2.437338	0.133445	0.038199	1	RP11-390P2.4	_
ENSG00000263235	-2.437338	0.133445	0.038199	1	RP11-461A8.4	_
ENSG00000272760	-2.437338	0.133445	0.038199	1	RP11-5C23.1	_
ENSG00000269165	2.263991	-0.127909	0.038199	1	AC069547.1	_
ENSG00000004846	-3.030976	-0.193128	0.039696	1	ABCB5	K05660
ENSG00000088002	2.708916	-0.438045	0.039696	1	SULT2B1	K01015
ENSG00000138606	2.708916	-0.438045	0.039696	1	SHF	_
ENSG00000187134	2.708916	-0.438045	0.039696	1	AKR1C1	K00089
ENSG00000259881	2.708916	-0.438045	0.039696	1	RP11-830F9.5	_
ENSG00000253500	-1.723051	1.715137	0.039747	1	AF121898.3	_
ENSG00000223975	1.742979	0.868905	0.039955	1	AP001048.4	_
ENSG00000175899	1.936632	0.403921	0.040094	1	A2M	K03910

ENSG00000109738	-1.759268	1.479487	0.040452	1	GLRB	K05196
ENSG00000185499	1.372421	5.617529	0.041018	1	MUC1	K06568
ENSG00000137393	-1.979233	0.893245	0.042147	1	RNF144B	K11975
ENSG00000170873	-1.979233	0.893245	0.042147	1	MTSS1	_
ENSG00000173432	-1.979233	0.893245	0.042147	1	SAA1	K17310
ENSG00000134061	1.530768	2.169576	0.042185	1	CD180	K06555
ENSG00000224321	-2.203656	0.435314	0.042606	1	RP11-169K16.6	K02870
ENSG00000095539	1.923603	0.241389	0.042606	1	SEMA4G	K06521
ENSG00000124243	1.923603	0.241389	0.042606	1	BCAS4	K03685
ENSG00000239653	1.923603	0.241389	0.042606	1	PSMD6-AS2	_
ENSG00000115194	1.705772	0.938641	0.043422	1	SLC30A3	K14690
ENSG00000137752	-1.397771	3.94732	0.044496	1	CASP1	K01370
ENSG00000104894	1.811682	0.587597	0.044792	1	CD37	K06475
ENSG00000169994	1.811682	0.587597	0.044792	1	MYO7B	K10359
ENSG00000119900	-1.351139	5.535768	0.045165	1	OGFRL1	K17985
ENSG00000005102	1.356884	4.785271	0.045201	1	MEOX1	K09322
ENSG00000185133	1.38801	3.820727	0.045285	1	INPP5J	K01106
ENSG00000130821	2.123527	0.005722	0.045652	1	SLC6A8	K05039
ENSG00000272983	2.123527	0.005722	0.045652	1	RP11-508N22.12	_
ENSG00000137101	1.906764	0.05994	0.045652	1	CD72	K06504
ENSG00000231412	1.48162	1.895812	0.04596	1	CTC-490G23.2	_
ENSG00000273151	1.679081	0.826119	0.045981	1	RP11-449P15.2	_
ENSG00000134013	1.325682	7.48902	0.046595	1	LOXL2	K00280
ENSG00000114268	1.359875	4.09032	0.047102	1	PFKFB4	K19030
ENSG00000202198	1.34918	4.651359	0.047506	1	RN7SK	_
ENSG00000106025	-1.374024	4.107733	0.047514	1	TSPAN12	K17355
ENSG00000237330	1.50588	1.823183	0.047648	1	RNF223	K08857
ENSG00000011347	1.350298	4.456313	0.04795	1	SYT7	_
ENSG00000188211	-1.939468	0.587885	0.048037	1	NCR3LG1	K16349
ENSG00000251169	-1.939468	0.587885	0.048037	1	AC005355.2	_
ENSG00000186862	1.399292	2.921386	0.048403	1	PDZD7	_
ENSG00000157368	-2.30709	0.037949	0.049336	1	IL34	_
ENSG00000159167	2.139349	-0.208426	0.049336	1	STC1	_
ENSG00000238198	2.139349	-0.208426	0.049336	1	RP11-31F15.2	_
ENSG00000246089	-1.556178	2.08976	0.049338	1	RP11-115C21.2	_
ENSG00000140795	1.441039	2.143492	0.050732	1	MYLK3	K00907
ENSG00000115594	1.35131	3.501766	0.051637	1	IL1R1	K04386
ENSG00000115267	-1.315725	5.076924	0.052047	1	IFIH1	K12647
ENSG00000085741	1.585781	1.031228	0.052818	1	WNT11	_
ENSG00000066923	1.734023	0.5365	0.052888	1	STAG3	K13055
ENSG00000144230	1.734023	0.5365	0.052888	1	GPR17	K08404
ENSG00000187550	1.734023	0.5365	0.052888	1	SBK2	K08858
ENSG00000230154	-2.949299	-0.253087	0.053633	1	AC018463.4	_
ENSG00000240038	2.558214	-0.536447	0.053633	1	AMY2B	K01176
ENSG00000270705	2.558214	-0.536447	0.053633	1	RP11-736N17.9	_
ENSG00000017373	2.162448	-0.456693	0.053633	1	SRCIN1	_
ENSG00000136457	2.162448	-0.456693	0.053633	1	CHAD	K06248
ENSG00000147127	2.162448	-0.456693	0.053633	1	RAB41	K07896
ENSG00000250548	2.162448	-0.456693	0.053633	1	RP11-47I22.2	_
ENSG00000257279	2.162448	-0.456693	0.053633	1	RP11-231I16.1	_
ENSG00000168079	1.336011	3.827507	0.053756	1	SCARA5	_
ENSG00000171368	1.325377	3.830721	0.053756	1	TPPP	K18932
ENSG00000174343	1.35482	2.951035	0.05388	1	CHRNA9	K04810
ENSG00000251495	1.54587	1.298941	0.055216	1	PPIAP11	K03767
ENSG00000084636	-1.33845	3.749719	0.056136	1	COL16A1	_
ENSG00000159399	1.269774	7.460273	0.056338	1	HK2	K00844
ENSG00000123342	-5.602132	-1.097364	0.056522	1	MMP19	K07998
ENSG00000128872	-5.602132	-1.097364	0.056522	1	TMOD2	K10370

ENSG00000162461	-5.602132	-1.097364	0.056522	1	SLC25A34	K15117
ENSG00000171560	-5.602132	-1.097364	0.056522	1	FGA	K03903
ENSG00000189143	-5.602132	-1.097364	0.056522	1	CLDN4	K11835
ENSG00000225871	-5.602132	-1.097364	0.056522	1	RP11-495P10.10	_
ENSG00000227220	-5.602132	-1.097364	0.056522	1	RP11-69I8.3	_
ENSG00000228021	-5.602132	-1.097364	0.056522	1	RP11-383C5.3	_
ENSG00000232486	-5.602132	-1.097364	0.056522	1	RP11-18B3.2	_
ENSG00000232615	-5.602132	-1.097364	0.056522	1	CTD-2012J19.1	K15425
ENSG00000242147	-5.602132	-1.097364	0.056522	1	RP13-463N16.6	_
ENSG00000259212	-5.602132	-1.097364	0.056522	1	CTD-3065B20.2	_
ENSG00000270084	-5.602132	-1.097364	0.056522	1	GAS5-AS1	_
ENSG00000272379	-5.602132	-1.097364	0.056522	1	RP1-257A7.5	_
ENSG00000272576	-5.602132	-1.097364	0.056522	1	RP11-365H22.2	_
ENSG00000272841	-5.602132	-1.097364	0.056522	1	RP3-428L16.2	_
ENSG00000272216	1.644585	0.897725	0.056561	1	LL22NC03-2H8.5	_
ENSG00000249087	1.517997	1.52572	0.056867	1	C1orf213	_
ENSG00000261087	1.460319	1.837306	0.057268	1	KB-1460A1.5	_
ENSG00000125266	1.792039	-0.011168	0.057273	1	EFNB2	K05463
ENSG00000127084	1.792039	-0.011168	0.057273	1	FGD3	K05722
ENSG00000164061	1.792039	-0.011168	0.057273	1	BSN	_
ENSG00000167772	1.283187	4.906567	0.057368	1	ANGPTL4	K08767
ENSG00000116117	-1.893908	0.554546	0.057683	1	PARD3B	_
ENSG00000141540	1.526456	1.35121	0.057716	1	TTYH2	_
ENSG00000136425	2.746528	-0.851163	0.057771	1	CIB2	_
ENSG00000205710	2.746528	-0.851163	0.057771	1	C17orf107	K04817
ENSG00000260923	2.746528	-0.851163	0.057771	1	AC137934.1	_
ENSG00000265996	2.746528	-0.851163	0.057771	1	MIR3671	_
ENSG00000260563	1.358746	2.933825	0.057938	1	RP13-516M14.1	_
ENSG00000009950	1.589798	1.18843	0.058699	1	MLXIPL	K09113
ENSG00000260317	1.516431	1.211115	0.058699	1	RP11-48B3.4	_
ENSG00000260942	1.448229	1.587672	0.058988	1	CAPN10-AS1	_
ENSG00000164638	1.346485	2.905113	0.059252	1	SLC29A4	K03323
ENSG00000182319	-1.356709	3.231093	0.059644	1	SGK223	K17537
ENSG00000215196	1.290254	4.195585	0.05982	1	AC091878.1	_
ENSG00000136653	1.527083	0.992746	0.059836	1	RASSF5	K08015
ENSG00000174473	1.71782	0.750838	0.061138	1	GALNTL6	K00710
ENSG00000258279	1.612221	0.782244	0.061138	1	LINC00592	_
ENSG00000164056	-1.353328	2.652331	0.062452	1	SPRY1	K04704
ENSG00000186897	1.378124	2.135929	0.062548	1	C1QL4	_
ENSG00000101198	1.787278	0.444541	0.062562	1	NKAIN4	_
ENSG00000116690	1.787278	0.444541	0.062562	1	PRG4	K09291
ENSG00000166589	1.787278	0.444541	0.062562	1	CDH16	K06810
ENSG00000234678	1.787278	0.444541	0.062562	1	RP11-465N4.4	_
ENSG00000185669	1.651945	0.483852	0.062562	1	SNAI3	K09217
ENSG00000104899	1.263274	4.590839	0.063027	1	AMH	K04665
ENSG00000163485	-2.101179	0.359582	0.063872	1	ADORA1	K04265
ENSG00000144583	-2.047075	0.319999	0.063872	1	4-Mar	K10659
ENSG00000197415	-2.047075	0.319999	0.063872	1	VEPH1	_
ENSG00000104760	1.824626	0.17784	0.063872	1	FGL1	_
ENSG00000132130	1.824626	0.17784	0.063872	1	LHX1	_
ENSG00000233974	1.824626	0.17784	0.063872	1	RP11-823P9.3	_
ENSG00000234353	1.824626	0.17784	0.063872	1	AP000347.4	K06554
ENSG00000116032	-2.237274	-0.012548	0.06403	1	GRIN3B	K05214
ENSG00000255423	-2.237274	-0.012548	0.06403	1	EBLN2	_
ENSG00000263551	-2.163906	-0.065072	0.06403	1	RP11-78F17.1	_
ENSG00000161958	2.00291	-0.292876	0.06403	1	FGF11	K04358
ENSG00000146674	1.230584	7.035789	0.064413	1	IGFBP3	K10138
ENSG00000113083	1.226197	7.343302	0.065143	1	LOX	K00277

ENSG00000159314	1.464119	1.176821	0.065616	1	ARHGAP27	_
ENSG00000247903	-1.305439	3.414012	0.066029	1	RP11-421F16.3	_
ENSG00000168298	1.508486	1.40167	0.066082	1	HIST1H1E	K11275
ENSG00000022267	1.447522	1.420998	0.066082	1	FHL1	K14365
ENSG00000267480	1.447522	1.420998	0.066082	1	RP11-703I16.1	_
ENSG00000167992	1.43552	1.630686	0.066313	1	VWCE	_
ENSG00000264991	-1.831771	0.784112	0.066846	1	AP001205.1	_
ENSG00000248015	1.687962	0.623053	0.066846	1	AC005329.7	_
ENSG00000153140	-1.250927	4.436207	0.067098	1	CETN3	K16466
ENSG00000140854	-1.342599	2.878099	0.067138	1	KATNB1	K18643
ENSG00000163993	1.210131	7.908954	0.068361	1	S100P	_
ENSG00000119938	1.214901	6.34361	0.068649	1	PPP1R3C	K07189
ENSG00000006704	1.333508	2.331947	0.068833	1	GTF2IRD1	K03121
ENSG00000124216	-0.972539	3.957933	0.069137	1	SNAI1	K05707
ENSG00000232759	-1.846861	0.520329	0.069419	1	AC002480.3	_
ENSG00000146376	-1.322793	2.947629	0.069562	1	ARHGAP18	_
ENSG00000130234	-1.452212	1.836012	0.069913	1	ACE2	K09708
ENSG00000128482	-1.684599	0.905107	0.070633	1	RNF112	_
ENSG00000246898	-1.684599	0.905107	0.070633	1	LINC00920	_
ENSG00000267745	-1.684599	0.905107	0.070633	1	RP11-686D22.8	_
ENSG00000214856	1.647711	0.7048	0.070633	1	KRT16P1	K07604
ENSG00000173531	1.542112	0.737225	0.070633	1	MST1	_
ENSG00000228873	1.542112	0.737225	0.070633	1	AC012307.2	_
ENSG00000271918	1.542112	0.737225	0.070633	1	CTD-2287O16.5	_
ENSG00000272145	1.504104	1.056704	0.070754	1	NFYC-AS1	_
ENSG00000272994	-1.408501	2.064127	0.071084	1	RP11-332H14.2	_
ENSG00000129521	1.229951	4.35086	0.071304	1	EGLN3	K09592
ENSG00000138646	-1.200224	6.540482	0.071709	1	HERC5	_
ENSG00000232070	2.008802	-0.06817	0.072109	1	TMEM253	_
ENSG00000233122	2.008802	-0.06817	0.072109	1	CTAGE7P	_
ENSG00000270109	2.008802	-0.06817	0.072109	1	RP11-393M11.2	_
ENSG00000175155	1.39608	1.604479	0.072121	1	YPEL2	_
ENSG00000120885	1.19037	9.453988	0.072468	1	CLU	K17252
ENSG00000005486	1.341674	2.002663	0.072494	1	RHBDD2	K09652
ENSG00000120875	-1.199938	5.855855	0.07282	1	DUSP4	K04459
ENSG00000152292	1.239341	3.589968	0.07284	1	SH2D6	_
ENSG00000227292	-2.77061	-0.381879	0.072973	1	AC009229.5	_
ENSG00000259332	-2.77061	-0.381879	0.072973	1	ST20-MTHFS	K01934
ENSG00000101188	2.389913	-0.640856	0.072973	1	NTSR1	K04211
ENSG00000101210	2.389913	-0.640856	0.072973	1	EEF1A2	K03231
ENSG00000129910	2.389913	-0.640856	0.072973	1	CDH15	K06809
ENSG00000178404	2.389913	-0.640856	0.072973	1	DDC8	_
ENSG00000184471	2.389913	-0.640856	0.072973	1	C1QTNF8	K19470
ENSG00000224388	2.389913	-0.640856	0.072973	1	BACE2-IT1	_
ENSG00000226318	2.389913	-0.640856	0.072973	1	RP11-474D14.2	K02984
ENSG00000230189	2.389913	-0.640856	0.072973	1	GS1-124K5.2	K03121
ENSG00000232712	2.389913	-0.640856	0.072973	1	RP4-777D9.2	_
ENSG00000234636	2.389913	-0.640856	0.072973	1	MED14-AS1	_
ENSG00000260710	2.389913	-0.640856	0.072973	1	RP11-616M22.7	_
ENSG00000260936	2.389913	-0.640856	0.072973	1	FTO-IT1	_
ENSG00000273402	2.389913	-0.640856	0.072973	1	RP5-855D21.2	_
ENSG00000112559	1.994147	-0.555366	0.072973	1	MDFI	_
ENSG00000161939	1.994147	-0.555366	0.072973	1	RNASEK-C17orf49	K19770
ENSG00000176723	1.994147	-0.555366	0.072973	1	ZNF843	K17985
ENSG00000227006	1.994147	-0.555366	0.072973	1	RP5-956O18.2	_
ENSG00000236194	1.994147	-0.555366	0.072973	1	AC003104.1	_
ENSG00000040731	1.188254	7.077362	0.073852	1	CDH10	K06802
ENSG00000105939	-1.179432	7.859229	0.07565	1	ZC3HAV1	K15259

ENSG00000254780	-1.563906	1.336146	0.075828	1	RP11-793I11.1	K09481
ENSG00000259863	1.446614	1.233381	0.075828	1	SH3RF3-AS1	_
ENSG00000232320	1.356699	1.935492	0.076042	1	AC009299.5	_
ENSG00000178107	1.365959	1.53334	0.076689	1	RP11-998D10.4	_
ENSG00000113790	-1.634237	1.063957	0.077014	1	EHHADH	K07514
ENSG00000164342	-1.634237	1.063957	0.077014	1	TLR3	K05401
ENSG00000167676	1.552476	0.925874	0.077014	1	PLIN4	_
ENSG00000174482	1.465896	0.953382	0.077014	1	LINGO2	_
ENSG00000175886	1.465896	0.953382	0.077014	1	RPL7AP66	K02936
ENSG00000176383	1.465896	0.953382	0.077014	1	B3GNT4	K07971
ENSG00000171984	-1.751974	0.72591	0.078188	1	C20orf196	K11847
ENSG00000108823	1.718356	0.111884	0.078524	1	SGCA	K12565
ENSG00000112139	1.718356	0.111884	0.078524	1	MDGA1	_
ENSG00000164684	-1.216141	3.995834	0.078939	1	ZNF704	K12795
ENSG00000182463	1.207786	3.749344	0.079206	1	TSHZ2	K09236
ENSG00000138356	-1.242547	3.279694	0.079387	1	AOX1	K00157
ENSG00000101977	-1.38285	1.964498	0.080032	1	MCF2	_
ENSG00000143320	1.336559	1.663016	0.080134	1	CRABP2	K17289
ENSG00000135678	1.16156	6.997744	0.080469	1	CPM	K01296
ENSG00000165272	1.156182	8.523291	0.081037	1	AQP3	K09876
ENSG00000136826	-1.161256	6.651521	0.081153	1	KLF4	K17846
ENSG00000143641	1.154718	8.753367	0.08135	1	GALNT2	K00710
ENSG00000128262	-1.610917	0.85192	0.081703	1	POM121L9P	K08878
ENSG00000134569	1.468421	0.691007	0.081703	1	LRP4	K03068
ENSG00000138685	-1.157584	7.059718	0.081709	1	FGF2	K18497
ENSG00000162366	1.273374	2.513037	0.082288	1	PDZK1IP1	_
ENSG00000137699	1.148708	7.616227	0.083354	1	TRIM29	K12010
ENSG00000125122	-2.086606	-0.119794	0.083497	1	LRRC29	K10275
ENSG00000178919	-2.086606	-0.119794	0.083497	1	FOXE1	K09398
ENSG00000237836	-2.086606	-0.119794	0.083497	1	PHKA2-AS1	_
ENSG00000240204	-2.086606	-0.119794	0.083497	1	SMKR1	_
ENSG00000256690	-2.086606	-0.119794	0.083497	1	RP11-727F15.9	_
ENSG00000106477	-1.7479	0.449069	0.08372	1	CEP41	K16455
ENSG00000174721	-1.414424	1.604809	0.083771	1	FGFBP3	_
ENSG00000269772	1.322995	1.505501	0.083771	1	RP11-178G16.5	_
ENSG00000093100	2.526912	-0.978809	0.084472	1	XXbac-B461K10.4	_
ENSG00000105383	2.526912	-0.978809	0.084472	1	CD33	K06473
ENSG00000110169	2.526912	-0.978809	0.084472	1	HPX	K18977
ENSG00000136206	2.526912	-0.978809	0.084472	1	SPDYE1	K08694
ENSG00000144130	2.526912	-0.978809	0.084472	1	NT5DC4	K01081
ENSG00000158815	2.526912	-0.978809	0.084472	1	FGF17	K04358
ENSG00000183562	2.526912	-0.978809	0.084472	1	AC131971.1	_
ENSG00000219607	2.526912	-0.978809	0.084472	1	PPP1R3G	_
ENSG00000225125	2.526912	-0.978809	0.084472	1	RANP4	K07936
ENSG00000225975	2.526912	-0.978809	0.084472	1	AC074138.3	_
ENSG00000231010	2.526912	-0.978809	0.084472	1	RP6-109B7.2	_
ENSG00000235237	2.526912	-0.978809	0.084472	1	RP1-151B14.6	_
ENSG00000238184	2.526912	-0.978809	0.084472	1	CD81-AS1	_
ENSG00000248968	2.526912	-0.978809	0.084472	1	CTD-2256P15.1	_
ENSG00000258927	2.526912	-0.978809	0.084472	1	RP11-1070N10.5	_
ENSG00000263624	2.526912	-0.978809	0.084472	1	RP11-45M22.3	_
ENSG00000267277	2.526912	-0.978809	0.084472	1	CTD-2342J14.6	_
ENSG00000269242	2.526912	-0.978809	0.084472	1	CTD-2192J16.22	K12311
ENSG00000272574	2.526912	-0.978809	0.084472	1	RP11-359K18.4	_
ENSG00000106258	1.513828	0.812856	0.084778	1	CYP3A5	K17690
ENSG00000213937	1.513828	0.812856	0.084778	1	CLDN9	K06087
ENSG00000110092	-1.144352	7.124054	0.085106	1	CCND1	K04503
ENSG00000188505	1.219796	3.144811	0.085112	1	NCCRP1	K12606

ENSG00000132623	-1.229964	3.107141	0.085616	1	ANKEF1	_
ENSG00000182379	1.163401	4.622046	0.086415	1	NXPH4	K16656
ENSG00000167656	1.297119	1.63741	0.086932	1	LY6D	K06846
ENSG00000142156	1.207235	3.120025	0.087237	1	COL6A1	K06238
ENSG00000269929	1.700247	0.388717	0.088001	1	RP11-2B6.2	_
ENSG00000161249	1.564914	0.429563	0.088001	1	DMKN	_
ENSG00000183657	1.564914	0.429563	0.088001	1	PP13439	_
ENSG00000184828	1.564914	0.429563	0.088001	1	ZBTB7C	K10494
ENSG00000228393	1.354658	1.360525	0.088271	1	LINC01004	_
ENSG00000156515	1.156621	4.498895	0.088846	1	HK1	K00844
ENSG00000150456	-1.266747	2.31172	0.089023	1	N6AMT2	_
ENSG00000232442	1.179031	3.241625	0.090151	1	CTD-3184A7.4	_
ENSG00000186073	-1.237147	2.570237	0.090293	1	C15orf41	K09513
ENSG00000121900	1.884159	-0.14535	0.091098	1	TMEM54	_
ENSG00000197459	1.884159	-0.14535	0.091098	1	HIST1H2BH	K11252
ENSG00000215458	1.884159	-0.14535	0.091098	1	AP001053.11	_
ENSG00000259856	1.528225	0.521938	0.091583	1	RAB43P1	K07930
ENSG00000268785	1.528225	0.521938	0.091583	1	RPL7P50	K02937
ENSG00000064195	1.133998	4.803491	0.092678	1	DLX3	K09315
ENSG00000223960	-1.279753	2.257755	0.093229	1	AC009948.5	_
ENSG00000005243	5.640214	-1.573505	0.094203	1	COPZ2	_
ENSG00000068831	5.640214	-1.573505	0.094203	1	RASGRP2	K12361
ENSG00000110799	5.640214	-1.573505	0.094203	1	VWF	K03900
ENSG00000129988	5.640214	-1.573505	0.094203	1	LBP	K05399
ENSG00000159337	5.640214	-1.573505	0.094203	1	PLA2G4D	K16342
ENSG00000161267	5.640214	-1.573505	0.094203	1	BDH1	K00019
ENSG00000177791	5.640214	-1.573505	0.094203	1	MYOZ1	_
ENSG00000180155	5.640214	-1.573505	0.094203	1	LYNX1	_
ENSG00000185792	5.640214	-1.573505	0.094203	1	NLRP9	_
ENSG00000189409	5.640214	-1.573505	0.094203	1	MMP23B	K08001
ENSG00000196811	5.640214	-1.573505	0.094203	1	CHRNG	K04818
ENSG00000201820	5.640214	-1.573505	0.094203	1	Y_RNA	_
ENSG00000203258	5.640214	-1.573505	0.094203	1	RP11-47J17.2	_
ENSG00000204894	5.640214	-1.573505	0.094203	1	RP11-208G20.2	K01079
ENSG00000205106	5.640214	-1.573505	0.094203	1	CTD-2210P24.4	_
ENSG00000217624	5.640214	-1.573505	0.094203	1	RP11-204C16.4	K16197
ENSG00000223356	5.640214	-1.573505	0.094203	1	RP11-66D17.5	_
ENSG00000223416	5.640214	-1.573505	0.094203	1	RPS26P15	K02976
ENSG00000223466	5.640214	-1.573505	0.094203	1	AC064834.2	_
ENSG00000224005	5.640214	-1.573505	0.094203	1	AC022400.1	_
ENSG00000234750	5.640214	-1.573505	0.094203	1	CTD-2666L21.2	K02981
ENSG00000244159	5.640214	-1.573505	0.094203	1	RP11-1070A24.1	K02977
ENSG00000247473	5.640214	-1.573505	0.094203	1	CARS-AS1	_
ENSG00000253307	5.640214	-1.573505	0.094203	1	RP11-10J21.4	_
ENSG00000253730	5.640214	-1.573505	0.094203	1	RP11-893F2.13	_
ENSG00000256443	5.640214	-1.573505	0.094203	1	RP11-794G24.1	_
ENSG00000259165	5.640214	-1.573505	0.094203	1	DDX18P1	K13179
ENSG00000260367	5.640214	-1.573505	0.094203	1	RP11-264B17.4	_
ENSG00000261457	5.640214	-1.573505	0.094203	1	AC002519.6	K04382
ENSG00000267560	5.640214	-1.573505	0.094203	1	RP11-173A16.1	_
ENSG00000267645	5.640214	-1.573505	0.094203	1	POLR2J2	K03008
ENSG00000267676	5.640214	-1.573505	0.094203	1	THA1P	K01620
ENSG00000268204	5.640214	-1.573505	0.094203	1	CTD-3214H19.6	_
ENSG00000271921	5.640214	-1.573505	0.094203	1	RP4-740C4.9	_
ENSG00000100253	-5.413723	-1.217337	0.094203	1	MIOX	K00469
ENSG00000101076	-5.413723	-1.217337	0.094203	1	HNF4A	K07292
ENSG00000115009	-5.413723	-1.217337	0.094203	1	CCL20	K14625
ENSG00000148143	-5.413723	-1.217337	0.094203	1	ZNF462	_

ENSG00000157653	-5.413723	-1.217337	0.094203	1	C9orf43	K07524
ENSG00000179921	-5.413723	-1.217337	0.094203	1	GPBAR1	K08400
ENSG00000180209	-5.413723	-1.217337	0.094203	1	MYLPF	K12758
ENSG00000182261	-5.413723	-1.217337	0.094203	1	NLRP10	_
ENSG00000184502	-5.413723	-1.217337	0.094203	1	GAST	K13768
ENSG00000184564	-5.413723	-1.217337	0.094203	1	SLITRK6	_
ENSG00000184787	-5.413723	-1.217337	0.094203	1	UBE2G2	K04555
ENSG00000197576	-5.413723	-1.217337	0.094203	1	HOXA4	K09304
ENSG00000203401	-5.413723	-1.217337	0.094203	1	AC009061.1	K04613
ENSG00000225506	-5.413723	-1.217337	0.094203	1	CYP4A22-AS1	_
ENSG00000229167	-5.413723	-1.217337	0.094203	1	RP11-73M7.1	_
ENSG00000235078	-5.413723	-1.217337	0.094203	1	AC142528.1	_
ENSG00000238832	-5.413723	-1.217337	0.094203	1	snoU109	_
ENSG00000240211	-5.413723	-1.217337	0.094203	1	RP11-758P17.3	_
ENSG00000242569	-5.413723	-1.217337	0.094203	1	RP11-435B5.3	_
ENSG00000243819	-5.413723	-1.217337	0.094203	1	RN7SL832P	_
ENSG00000254619	-5.413723	-1.217337	0.094203	1	RP11-646J21.4	_
ENSG00000259316	-5.413723	-1.217337	0.094203	1	CTD-2116N17.1	_
ENSG00000259523	-5.413723	-1.217337	0.094203	1	RP11-680F8.3	_
ENSG00000260394	-5.413723	-1.217337	0.094203	1	LA16c-313D11.9	_
ENSG00000264573	-5.413723	-1.217337	0.094203	1	RN7SL15P	_
ENSG00000266411	-5.413723	-1.217337	0.094203	1	RP11-180P8.3	_
ENSG00000267093	-5.413723	-1.217337	0.094203	1	CTD-2105E13.13	_
ENSG00000267199	-5.413723	-1.217337	0.094203	1	RP11-861E21.2	_
ENSG00000267340	-5.413723	-1.217337	0.094203	1	RP11-242D8.3	_
ENSG00000269999	-5.413723	-1.217337	0.094203	1	CTD-3185P2.2	_
ENSG00000270757	-5.413723	-1.217337	0.094203	1	HSPE1-MOB4	K04078
ENSG00000270761	-5.413723	-1.217337	0.094203	1	RP11-385F7.1	_
ENSG00000270923	-5.413723	-1.217337	0.094203	1	TAS2R6	K08474
ENSG00000272524	-5.413723	-1.217337	0.094203	1	RP11-254F7.4	_
ENSG00000273489	-5.413723	-1.217337	0.094203	1	RP11-180C16.1	_
ENSG00000107593	2.843859	-1.411664	0.094203	1	PKD2L1	K04990
ENSG00000135439	2.843859	-1.411664	0.094203	1	AGAP2	K17848
ENSG00000137878	2.843859	-1.411664	0.094203	1	GCOM1	_
ENSG00000149926	2.843859	-1.411664	0.094203	1	FAM57B	_
ENSG00000166313	2.843859	-1.411664	0.094203	1	APBB1	K04529
ENSG00000172818	2.843859	-1.411664	0.094203	1	OVOL1	K09216
ENSG00000174038	2.843859	-1.411664	0.094203	1	C9orf131	_
ENSG00000179059	2.843859	-1.411664	0.094203	1	ZFP42	K11516
ENSG00000180316	2.843859	-1.411664	0.094203	1	PNPLA1	K16813
ENSG00000201390	2.843859	-1.411664	0.094203	1	RNU6-1141P	_
ENSG00000213453	2.843859	-1.411664	0.094203	1	FTH1P3	K00522
ENSG00000214278	2.843859	-1.411664	0.094203	1	CTD-2228K2.2	K02520
ENSG00000215481	2.843859	-1.411664	0.094203	1	BCRP3	K08878
ENSG00000215704	2.843859	-1.411664	0.094203	1	CELA2B	K01346
ENSG00000225037	2.843859	-1.411664	0.094203	1	EIF1AX-AS1	_
ENSG00000225071	2.843859	-1.411664	0.094203	1	GS1-184P14.2	K02976
ENSG00000227218	2.843859	-1.411664	0.094203	1	RP11-203J24.8	_
ENSG00000227698	2.843859	-1.411664	0.094203	1	AP001619.2	_
ENSG00000230666	2.843859	-1.411664	0.094203	1	CEACAM22P	K06499
ENSG00000231703	2.843859	-1.411664	0.094203	1	RP4-669H2.1	_
ENSG00000232386	2.843859	-1.411664	0.094203	1	RP11-66B24.2	_
ENSG00000232490	2.843859	-1.411664	0.094203	1	OSBPL10-AS1	_
ENSG00000234354	2.843859	-1.411664	0.094203	1	RPS26P47	K02976
ENSG00000250292	2.843859	-1.411664	0.094203	1	RP11-451F20.1	_
ENSG00000250645	2.843859	-1.411664	0.094203	1	CTD-2228K2.1	_
ENSG00000250742	2.843859	-1.411664	0.094203	1	RP11-834C11.4	_
ENSG00000255342	2.843859	-1.411664	0.094203	1	RP11-762B21.5	K17986

ENSG00000258521	2.843859	-1.411664	0.094203	1	RP11-638I2.9	_
ENSG00000259287	2.843859	-1.411664	0.094203	1	RP11-3D4.2	_
ENSG00000262585	2.843859	-1.411664	0.094203	1	RP11-353N14.5	_
ENSG00000262686	2.843859	-1.411664	0.094203	1	AC005356.1	_
ENSG00000266261	2.843859	-1.411664	0.094203	1	RP11-640I15.1	_
ENSG00000267632	2.843859	-1.411664	0.094203	1	RP11-400F19.18	_
ENSG00000269481	2.843859	-1.411664	0.094203	1	CTD-2521M24.6	_
ENSG00000272024	2.843859	-1.411664	0.094203	1	RP11-546K22.3	_
ENSG00000273289	2.843859	-1.411664	0.094203	1	RP6-109B7.5	_
ENSG00000043591	-1.533267	0.796459	0.094618	1	ADRB1	K04141
ENSG00000242759	-1.533267	0.796459	0.094618	1	LINC00882	_
ENSG00000135709	1.159004	3.461358	0.094929	1	KIAA0513	_
ENSG00000259820	1.289513	1.678863	0.095587	1	AC083843.1	_
ENSG00000253217	1.228682	2.291599	0.095685	1	KB-1991G8.1	_
ENSG00000178222	1.380021	1.255244	0.095725	1	RNF212	K04354
ENSG00000256087	1.138191	3.998924	0.095837	1	ZNF432	K09228
ENSG00000137825	1.195795	2.560603	0.095921	1	ITPKA	K00911
ENSG00000171219	1.232514	2.039225	0.096179	1	CDC42BPG	K16307
ENSG00000175984	1.232514	2.039225	0.096179	1	DENND2C	K11447
ENSG00000079335	-1.219766	2.656063	0.096547	1	CDC14A	K06639
ENSG00000117228	-1.93237	0.237034	0.096786	1	GBP1	_
ENSG00000196335	-1.93237	0.237034	0.096786	1	STK31	K16316
ENSG00000208317	-1.93237	0.237034	0.096786	1	SNORD78	_
ENSG00000225377	-1.93237	0.237034	0.096786	1	RP5-1103G7.4	_
ENSG00000111181	1.60363	0.043337	0.096786	1	SLC6A12	K05039
ENSG00000180720	1.60363	0.043337	0.096786	1	CHRM4	K04132
ENSG00000197653	1.60363	0.043337	0.096786	1	DNAH10	K10408
ENSG00000213626	1.60363	0.043337	0.096786	1	LBH	K09228
ENSG00000231274	1.60363	0.043337	0.096786	1	SBK3	K08858
ENSG00000244176	1.60363	0.043337	0.096786	1	RP11-810P12.1	K02981
ENSG00000110881	1.257908	2.090216	0.097062	1	ASIC1	K04829
ENSG00000134531	-1.111643	5.678995	0.097164	1	EMP1	_
ENSG00000163577	-1.257042	2.47896	0.097831	1	EIF5A2	K03263
ENSG00000229891	1.153114	3.379342	0.098405	1	Z83851.1	_
ENSG00000185650	-1.101937	6.273747	0.098547	1	ZFP36L1	K18753
ENSG00000261326	1.263104	1.833833	0.098858	1	RP5-1057J7.6	_
ENSG00000166068	-1.118768	4.451535	0.099672	1	SPRED1	K04703
ENSG00000258168	-1.278978	1.972212	0.099813	1	RP11-588H23.3	_
ENSG00000104951	-2.672216	-0.451326	0.099976	1	IL4I1	K03334
ENSG00000118997	-2.672216	-0.451326	0.099976	1	DNAH7	K10408
ENSG00000171943	-2.672216	-0.451326	0.099976	1	SRGAP2C	K07526
ENSG00000197980	-2.672216	-0.451326	0.099976	1	LEKR1	_
ENSG00000225968	-2.672216	-0.451326	0.099976	1	ELFN1	K17567
ENSG00000241476	-2.672216	-0.451326	0.099976	1	SSX2	K15625
ENSG00000250629	-2.672216	-0.451326	0.099976	1	RP11-122C5.3	_
ENSG00000007312	1.803588	-0.660047	0.099976	1	CD79B	K06507
ENSG00000009724	1.803588	-0.660047	0.099976	1	MASP2	K03993
ENSG00000125841	1.803588	-0.660047	0.099976	1	NRSN2	_
ENSG00000133020	1.803588	-0.660047	0.099976	1	MYH8	K10352
ENSG00000135346	1.803588	-0.660047	0.099976	1	CGA	K08522
ENSG00000135436	1.803588	-0.660047	0.099976	1	FAM186B	_
ENSG00000159761	1.803588	-0.660047	0.099976	1	C16orf86	_
ENSG00000177854	1.803588	-0.660047	0.099976	1	TMEM187	_
ENSG00000182472	1.803588	-0.660047	0.099976	1	CAPN12	K04740
ENSG00000184786	1.803588	-0.660047	0.099976	1	TCTE3	_
ENSG00000255994	1.803588	-0.660047	0.099976	1	CCDC177	_
ENSG00000268265	1.803588	-0.660047	0.099976	1	AP000304.1	_
ENSG00000272502	1.803588	-0.660047	0.099976	1	RP11-713M15.2	_

ENSG00000273355	1.803588	-0.660047	0.099976	1	RP11-672L10.6	_
ENSG00000086289	-1.189726	3.043969	0.100357	1	EPDR1	_
ENSG00000198959	-1.246844	2.026495	0.100711	1	TGM2	K05625
ENSG00000145198	1.658005	0.225508	0.101169	1	VWA5B2	_
ENSG00000165995	1.658005	0.225508	0.101169	1	CACNB2	K04863
ENSG00000271851	1.658005	0.225508	0.101169	1	RP11-565F19.2	_
ENSG00000183018	-1.641647	0.373671	0.101169	1	SPNS2	_
ENSG00000233247	-1.641647	0.373671	0.101169	1	GS1-257G1.1	K06689
ENSG00000248126	-1.443677	1.098022	0.101443	1	CTD-2012J19.2	_
ENSG00000205336	1.09197	5.638787	0.102288	1	GPR56	K08450
ENSG00000105289	1.161545	3.105736	0.102301	1	TJP3	K06097
ENSG00000123405	1.107606	4.2604	0.102326	1	NFE2	K09039
ENSG00000257596	1.305203	1.594748	0.102406	1	RP11-968A15.2	_
ENSG00000173295	-1.176161	2.961622	0.102572	1	FAM86B3P	_
ENSG00000123609	-1.109213	4.746511	0.102576	1	NMI	_
ENSG00000188660	1.098535	4.729168	0.102576	1	LINC00319	_
ENSG00000147689	1.078486	7.93307	0.10334	1	FAM83A	_
ENSG00000269729	-1.338507	1.754887	0.10344	1	AC006262.4	_
ENSG00000103056	1.129424	3.539505	0.103813	1	SMPD3	K12352
ENSG00000176697	-1.115324	4.100602	0.104096	1	BDNF	K04355
ENSG00000126218	1.607627	0.331044	0.104627	1	F10	K01314
ENSG00000235052	1.607627	0.331044	0.104627	1	RP1-150O5.3	_
ENSG00000261360	1.607627	0.331044	0.104627	1	CTD-2165H16.4	_
ENSG00000220848	1.472294	0.373533	0.104627	1	RPS18P9	K02964
ENSG00000166407	1.134241	3.27005	0.104927	1	LMO1	_
ENSG00000227896	1.275572	1.758402	0.10529	1	RP11-77P6.2	_
ENSG00000196911	-1.164427	2.915588	0.105472	1	KPNA5	K15042
ENSG00000153391	-1.127115	3.529792	0.105499	1	INO80C	K11667
ENSG00000178922	-1.106055	4.390289	0.105543	1	HYI	K01816
ENSG00000165300	-1.112833	4.194265	0.105587	1	SLITRK5	_
ENSG00000123358	-1.079061	6.199129	0.105828	1	NR4A1	K04465
ENSG00000197582	-1.296609	1.903377	0.10612	1	GPX1P1	K00432
ENSG00000266036	1.227552	1.810929	0.10612	1	MIR3615	_
ENSG00000109062	1.070751	7.262845	0.106233	1	SLC9A3R1	K13365
ENSG00000168874	1.084553	4.847161	0.106551	1	ATOH8	K09084
ENSG00000185168	1.25735	1.870136	0.106896	1	LINC00482	_
ENSG00000173281	1.09984	3.911356	0.107168	1	PPP1R3B	K07189
ENSG00000136869	-1.667503	0.664977	0.107412	1	TLR4	K10160
ENSG00000196511	-1.230387	2.015003	0.107623	1	TPK1	K00949
ENSG00000168016	-1.221467	2.454065	0.107908	1	TRANK1	_
ENSG00000197608	1.102161	3.857702	0.108432	1	ZNF841	K09228
ENSG00000174483	1.296166	1.380375	0.108433	1	BBS1	K16746
ENSG00000205221	1.09947	3.920108	0.10926	1	VIT	_
ENSG00000216657	-2.004929	-0.176905	0.109379	1	RP3-406P24.1	_
ENSG00000233589	-2.004929	-0.176905	0.109379	1	RP4-694A7.2	_
ENSG00000259712	-2.004929	-0.176905	0.109379	1	CTD-2184D3.5	_
ENSG00000126759	1.852208	-0.381659	0.109379	1	CFP	K15412
ENSG00000182782	1.852208	-0.381659	0.109379	1	HCAR2	K08402
ENSG00000185507	1.852208	-0.381659	0.109379	1	IRF7	K09447
ENSG00000186376	1.852208	-0.381659	0.109379	1	ZNF75D	K09229
ENSG00000228204	1.852208	-0.381659	0.109379	1	RP4-724E13.2	_
ENSG00000255503	1.852208	-0.381659	0.109379	1	RP11-113K21.4	_
ENSG00000269927	1.852208	-0.381659	0.109379	1	RP6-91H8.3	_
ENSG00000168772	-1.347485	1.557274	0.109441	1	CXXC4	K03344
ENSG00000220378	1.287132	1.429583	0.109441	1	KRT8P42	K07605
ENSG00000151229	-1.107066	3.804591	0.109463	1	SLC2A13	K08150
ENSG00000183186	1.496361	0.608886	0.109695	1	C2CD4C	_
ENSG00000256061	-1.492817	0.767812	0.109695	1	DYX1C1	K19758

ENSG00000227963	1.390762	0.643529	0.109695	1	RP5-1074L1.1	_
ENSG00000234373	1.370027	0.723613	0.111592	1	SNX18P7	K17299
ENSG00000137965	-1.05073	7.17996	0.113145	1	IFI44	_
ENSG00000260000	-1.50046	0.96784	0.113191	1	RP3-467N11.1	_
ENSG00000169271	1.421718	0.842714	0.113191	1	HSPB3	K09544
ENSG00000228451	1.421718	0.842714	0.113191	1	SDAD1P1	K14856
ENSG00000142279	1.335138	0.871857	0.113191	1	WTIP	K16682
ENSG00000144045	1.335138	0.871857	0.113191	1	DQX1	K14433
ENSG00000136999	-1.059817	5.541446	0.113428	1	NOV	_
ENSG00000184481	1.108746	3.489808	0.113482	1	FOXO4	K12358
ENSG00000058668	1.047695	7.062058	0.114003	1	ATP2B4	K05850
ENSG00000257315	1.222653	1.847765	0.114505	1	ZBED6	_
ENSG00000260604	1.183306	1.861539	0.114505	1	RP1-140K8.5	_
ENSG00000175221	1.061738	5.09675	0.114518	1	MED16	K15159
ENSG00000166073	-1.145312	2.604438	0.115066	1	GPR176	K08442
ENSG00000091262	1.74772	-0.226119	0.11546	1	ABCC6	K05669
ENSG00000105707	1.74772	-0.226119	0.11546	1	HPN	K08665
ENSG00000157999	1.74772	-0.226119	0.11546	1	ANKRD61	_
ENSG00000171450	1.74772	-0.226119	0.11546	1	CDK5R2	K11716
ENSG00000179083	1.74772	-0.226119	0.11546	1	FAM133A	_
ENSG00000203288	1.74772	-0.226119	0.11546	1	RP11-98D18.9	_
ENSG00000135698	-1.078551	4.008228	0.115894	1	MPHOSPH6	K12593
ENSG00000147872	1.046801	6.02781	0.115953	1	PLIN2	K17284
ENSG00000163395	1.353437	1.1061	0.11674	1	IGFN1	_
ENSG00000261553	1.353437	1.1061	0.11674	1	RP11-29G8.3	_
ENSG00000145632	-1.038055	8.005947	0.116757	1	PLK2	K08861
ENSG00000131459	1.043011	5.932914	0.117173	1	GFPT2	K00820
ENSG00000131370	1.072063	3.963349	0.117778	1	SH3BP5	K04613
ENSG00000197614	1.035576	6.949947	0.118284	1	MFAP5	K12581
ENSG00000260285	1.255403	1.29782	0.118412	1	RP11-600F24.7	_
ENSG00000103089	1.049247	4.606391	0.11918	1	FA2H	K19703
ENSG00000163660	-1.032392	7.223037	0.119395	1	CCNL1	_
ENSG00000183172	-1.871406	0.193481	0.119582	1	SMDT1	_
ENSG00000261114	-1.871406	0.193481	0.119582	1	RP11-325K4.2	_
ENSG00000271614	-1.871406	0.193481	0.119582	1	LINC00936	_
ENSG00000010319	-1.807751	0.148421	0.119582	1	SEMA3G	K06840
ENSG00000058085	-1.807751	0.148421	0.119582	1	LAMC2	K06246
ENSG00000167995	-1.807751	0.148421	0.119582	1	BEST1	K13878
ENSG00000199477	-1.807751	0.148421	0.119582	1	SNORA31	_
ENSG00000172728	-1.097234	3.179115	0.119821	1	FUT10	K09669
ENSG00000139163	-1.040286	5.528265	0.120305	1	ETNK1	K00894
ENSG00000127952	1.126951	2.562683	0.120456	1	STYXL1	K18047
ENSG00000205426	-1.278108	1.712361	0.121234	1	KRT81	K07605
ENSG00000155792	-1.219677	1.763381	0.121645	1	DEPTOR	_
ENSG00000204790	-1.219677	1.763381	0.121645	1	CBWD6	_
ENSG00000260630	-1.219677	1.763381	0.121645	1	SNAI3-AS1	_
ENSG00000232533	-1.11241	2.954638	0.122361	1	AC093673.5	_
ENSG00000127578	1.070569	3.60324	0.122453	1	WFIKKN1	_
ENSG00000261770	-1.585435	0.334263	0.122481	1	CTC-459F4.1	_
ENSG00000269175	-1.585435	0.334263	0.122481	1	AC093157.1	K00586
ENSG00000272782	-1.585435	0.334263	0.122481	1	RP4-607J23.2	_
ENSG00000166126	1.551735	0.161736	0.122481	1	AMN	K18259
ENSG00000250318	1.551735	0.161736	0.122481	1	CTA-963H5.5	K02872
ENSG00000259891	1.147754	1.839084	0.122678	1	CTA-204B4.2	_
ENSG00000115738	1.044196	4.501456	0.122768	1	ID2	K17693
ENSG00000213047	-1.075706	3.762962	0.123061	1	DENND1B	K02896
ENSG00000115271	-1.101532	3.212665	0.123607	1	GCA	_
ENSG00000023171	1.021534	6.742947	0.123608	1	GRAMD1B	_

ENSG00000113356	-1.038776	5.025047	0.123702	1	POLR3G	K03024
ENSG00000237187	-1.180382	2.188385	0.124131	1	NR2F1-AS1	_
ENSG00000237857	-1.180382	2.188385	0.124131	1	RP11-435O5.2	_
ENSG00000006283	1.120319	2.2503	0.124658	1	CACNA1G	K04854
ENSG00000029153	-1.073622	3.517626	0.124779	1	ARNTL2	K09099
ENSG00000067141	2.267755	-1.116915	0.12496	1	NEO1	K06766
ENSG00000105523	2.267755	-1.116915	0.12496	1	FAM83E	K00272
ENSG00000106809	2.267755	-1.116915	0.12496	1	OGN	K08126
ENSG00000131634	2.267755	-1.116915	0.12496	1	TMEM204	_
ENSG00000183044	2.267755	-1.116915	0.12496	1	ABAT	K13524
ENSG00000184678	2.267755	-1.116915	0.12496	1	HIST2H2BE	K11252
ENSG00000186891	2.267755	-1.116915	0.12496	1	TNFRSF18	K05154
ENSG00000188322	2.267755	-1.116915	0.12496	1	SBK1	K08858
ENSG00000197149	2.267755	-1.116915	0.12496	1	RP11-452G18.2	K02981
ENSG00000225808	2.267755	-1.116915	0.12496	1	DNAJC19P5	K09539
ENSG00000226386	2.267755	-1.116915	0.12496	1	PARD3-AS1	_
ENSG00000230929	2.267755	-1.116915	0.12496	1	RP11-395C3.1	K02942
ENSG00000231154	2.267755	-1.116915	0.12496	1	MORF4L2-AS1	_
ENSG00000233613	2.267755	-1.116915	0.12496	1	DCUN1D2-AS	_
ENSG00000234230	2.267755	-1.116915	0.12496	1	ZFX-AS1	_
ENSG00000235651	2.267755	-1.116915	0.12496	1	AC064850.4	K17265
ENSG00000243056	2.267755	-1.116915	0.12496	1	EIF4EBP3	K18645
ENSG00000253174	2.267755	-1.116915	0.12496	1	RP11-360L9.7	_
ENSG00000260296	2.267755	-1.116915	0.12496	1	RP11-395I6.3	_
ENSG00000261372	2.267755	-1.116915	0.12496	1	RP11-529H20.6	_
ENSG00000264269	2.267755	-1.116915	0.12496	1	RP11-15F12.1	_
ENSG00000267058	2.267755	-1.116915	0.12496	1	RP11-15A1.3	_
ENSG00000267834	2.267755	-1.116915	0.12496	1	RP11-167N5.5	_
ENSG00000268869	2.267755	-1.116915	0.12496	1	ESPNP	_
ENSG00000269834	2.267755	-1.116915	0.12496	1	CTD-3018O17.3	_
ENSG00000271858	2.267755	-1.116915	0.12496	1	XXcos-LUCA11.4	_
ENSG00000272666	2.267755	-1.116915	0.12496	1	CTA-384D8.35	_
ENSG00000264868	1.114459	2.356614	0.125095	1	CTB-167B5.2	_
ENSG00000100867	1.441195	0.469092	0.12613	1	DHRS2	K11164
ENSG00000137501	1.441195	0.469092	0.12613	1	SYTL2	K17598
ENSG00000268575	1.441195	0.469092	0.12613	1	RP1-283E3.8	_
ENSG00000273230	1.077692	2.705135	0.126209	1	RP11-1246C19.1	_
ENSG00000207523	-1.4512	0.738518	0.127301	1	SNORA66	_
ENSG00000214708	1.414283	0.558872	0.127301	1	AC090616.2	K07870
ENSG00000160888	-1.008098	7.64828	0.127745	1	IER2	_
ENSG00000141639	1.025446	4.920432	0.127789	1	MAPK4	K06855
ENSG00000172296	1.059612	3.607009	0.127903	1	SPTLC3	K00654
ENSG00000008517	-1.048926	3.785034	0.128074	1	IL32	_
ENSG00000165775	1.351609	0.799589	0.128892	1	FUNDC2	_
ENSG00000107317	-1.275377	1.131486	0.129867	1	PTGDS	K01830
ENSG00000259673	-1.36815	1.196341	0.130209	1	IQCH-AS1	_
ENSG00000266088	-1.36815	1.196341	0.130209	1	RP5-1028K7.2	_
ENSG00000244558	1.294739	1.069618	0.130209	1	RP11-445H22.4	_
ENSG00000185483	-1.042875	4.03482	0.130449	1	ROR1	K05122
ENSG00000160352	-1.042328	3.634579	0.130682	1	ZNF714	K09228
ENSG00000249803	-1.250375	1.374979	0.130701	1	RP11-114H21.2	_
ENSG00000243335	1.052375	3.158069	0.130964	1	KCTD7	K01623
ENSG00000233280	1.180879	1.466948	0.131133	1	CRYBG3	_
ENSG00000197279	-1.096836	2.830198	0.131208	1	ZNF165	K09230
ENSG00000139537	1.075625	2.783646	0.131208	1	CCDC65	_
ENSG00000155542	-1.104645	2.754309	0.131259	1	SETD9	_
ENSG00000163701	1.199787	1.709952	0.131399	1	IL17RE	K05168
ENSG00000175873	1.199787	1.709952	0.131399	1	AC004840.9	_

ENSG00000163286	1.156933	1.725204	0.131399	1	ALPPL2	K01077
ENSG00000142182	1.137528	1.978344	0.131498	1	DNMT3L	K17400
ENSG00000143797	-1.143766	2.097089	0.131501	1	MBOAT2	K13517
ENSG00000233621	1.021522	4.549375	0.131676	1	LINC01137	_
ENSG00000130720	1.005551	4.995572	0.133327	1	FIBCD1	_
ENSG00000117226	-1.043878	3.70002	0.133337	1	GBP3	_
ENSG00000128016	-1.010042	4.828396	0.133825	1	ZFP36	K15308
ENSG00000166510	-1.03416	3.960862	0.135032	1	CCDC68	_
ENSG00000131389	0.995431	5.924888	0.135037	1	SLC6A6	K05039
ENSG00000204397	-1.056999	3.088478	0.135175	1	CARD16	K01370
ENSG00000173641	1.019469	3.821778	0.135464	1	HSPB7	K09546
ENSG00000183762	1.008376	4.45424	0.136647	1	KREMEN1	K08857
ENSG00000122641	-2.566618	-0.524634	0.137874	1	INHBA	K04667
ENSG00000178146	-2.566618	-0.524634	0.137874	1	RP1-232L22__B.1	K00864
ENSG00000213676	-2.566618	-0.524634	0.137874	1	ATF6B	K09049
ENSG00000223459	-2.566618	-0.524634	0.137874	1	FAM115B	_
ENSG00000237399	-2.566618	-0.524634	0.137874	1	PITRM1-AS1	_
ENSG00000268995	-2.566618	-0.524634	0.137874	1	VN1R82P	_
ENSG00000270574	-2.566618	-0.524634	0.137874	1	RP11-171I2.2	_
ENSG00000271380	-2.566618	-0.524634	0.137874	1	RP11-307C12.12	_
ENSG00000109321	-2.452675	-0.602253	0.137874	1	AREG	K09782
ENSG00000157227	-2.452675	-0.602253	0.137874	1	MMP14	K07763
ENSG00000168539	-2.452675	-0.602253	0.137874	1	CHRM1	K04129
ENSG00000186648	-2.452675	-0.602253	0.137874	1	LRRC16B	_
ENSG00000203711	-2.452675	-0.602253	0.137874	1	C6orf99	_
ENSG00000218537	-2.452675	-0.602253	0.137874	1	AP000350.4	K07253
ENSG00000230555	-2.452675	-0.602253	0.137874	1	RP11-517P14.2	_
ENSG00000233825	-2.452675	-0.602253	0.137874	1	RP11-135A24.4	_
ENSG00000234292	-2.452675	-0.602253	0.137874	1	RP11-213H15.1	_
ENSG00000244733	-2.452675	-0.602253	0.137874	1	RP11-506M13.3	_
ENSG00000135094	2.199354	-0.752064	0.137874	1	SDS	K17989
ENSG00000149781	2.199354	-0.752064	0.137874	1	FERMT3	K17084
ENSG00000202337	2.199354	-0.752064	0.137874	1	RNU6-8	_
ENSG00000214279	2.199354	-0.752064	0.137874	1	RP11-108K14.4	_
ENSG00000218890	2.199354	-0.752064	0.137874	1	NUFIP1P	_
ENSG00000225578	2.199354	-0.752064	0.137874	1	NCBP2-AS1	_
ENSG00000230756	2.199354	-0.752064	0.137874	1	RHOQP3	K07194
ENSG00000236008	2.199354	-0.752064	0.137874	1	AC011747.4	_
ENSG00000236882	2.199354	-0.752064	0.137874	1	C5orf27	_
ENSG00000237940	2.199354	-0.752064	0.137874	1	AC093642.3	_
ENSG00000244399	2.199354	-0.752064	0.137874	1	RN7SL251P	_
ENSG00000259933	2.199354	-0.752064	0.137874	1	RP11-304L19.1	_
ENSG00000261226	2.199354	-0.752064	0.137874	1	RP11-830F9.7	_
ENSG00000132196	-1.018412	3.803602	0.138225	1	HSD17B7	K13373
ENSG00000036672	1.03759	3.148502	0.138775	1	USP2	K11833
ENSG00000132832	1.037549	3.103241	0.139034	1	RP11-445H22.3	_
ENSG00000196284	-1.059202	3.057258	0.139597	1	SUPT3H	K11313
ENSG00000146278	0.977753	6.658973	0.139991	1	PNRC1	K18774
ENSG00000161277	1.104424	1.957542	0.140049	1	THAP8	_
ENSG00000228649	-1.204883	1.839271	0.141201	1	AC005682.5	_
ENSG00000079308	0.979771	5.678829	0.141759	1	TNS1	K18080
ENSG00000174842	-0.987893	4.878193	0.142357	1	GLMN	_
ENSG00000157657	-1.025965	3.220736	0.142496	1	ZNF618	_
ENSG00000102178	1.176235	1.513264	0.142605	1	UBL4A	_
ENSG00000185220	-1.03461	3.1972	0.142675	1	PGBD2	_
ENSG00000188191	-0.999189	4.494161	0.143062	1	PRKAR1B	K04739
ENSG00000261094	1.191179	1.369701	0.143419	1	RP11-355O1.11	_
ENSG00000131778	1.137074	1.389333	0.143419	1	CHD1L	_

ENSG00000261113	1.137074	1.389333	0.143419	1	RP11-141O15.1	_
ENSG00000083750	-1.019985	3.505341	0.143467	1	RRAGB	K16185
ENSG00000167281	-1.91835	-0.236621	0.143889	1	RBFOX3	K14946
ENSG00000179428	-1.91835	-0.236621	0.143889	1	AC073072.5	_
ENSG00000261534	-1.91835	-0.236621	0.143889	1	RP11-244O19.1	_
ENSG00000273387	-1.91835	-0.236621	0.143889	1	RP3-412A9.16	_
ENSG00000232324	-1.82624	-0.29919	0.143889	1	AC008440.10	_
ENSG00000236498	-1.82624	-0.29919	0.143889	1	AC107081.5	_
ENSG00000257242	-1.82624	-0.29919	0.143889	1	C12orf79	_
ENSG00000260060	-1.82624	-0.29919	0.143889	1	RP11-388M20.1	_
ENSG00000260841	-1.82624	-0.29919	0.143889	1	CTC-205M6.5	_
ENSG00000100314	1.683907	-0.475243	0.143889	1	CABP7	K13152
ENSG00000117477	1.683907	-0.475243	0.143889	1	CCDC181	_
ENSG00000143217	1.683907	-0.475243	0.143889	1	PVRL4	K06593
ENSG00000162482	1.683907	-0.475243	0.143889	1	AKR7A3	K15303
ENSG00000179477	1.683907	-0.475243	0.143889	1	ALOX12B	K08021
ENSG00000185527	1.683907	-0.475243	0.143889	1	PDE6G	K13759
ENSG00000196782	1.683907	-0.475243	0.143889	1	MAML3	K06061
ENSG00000249345	1.683907	-0.475243	0.143889	1	RP11-575F12.1	_
ENSG00000251411	1.683907	-0.475243	0.143889	1	RP11-397E7.4	K05757
ENSG00000256849	1.683907	-0.475243	0.143889	1	RP11-405A12.1	K09493
ENSG00000260403	1.683907	-0.475243	0.143889	1	RP11-616M22.1	_
ENSG00000103319	0.976158	5.240112	0.144258	1	EEF2K	K08292
ENSG00000164683	0.976653	5.329348	0.144283	1	HEY1	K09091
ENSG00000146267	-1.09117	2.417737	0.144787	1	FAXC	K12581
ENSG00000235194	1.06257	2.349877	0.144787	1	PPP1R3E	K07189
ENSG00000146233	-1.27614	1.268545	0.144797	1	CYP39A1	K07439
ENSG00000224934	1.217027	1.153562	0.144797	1	RP11-441O15.3	_
ENSG00000236540	1.217027	1.153562	0.144797	1	AC006547.13	_
ENSG00000169288	-1.000405	3.920665	0.144881	1	MRPL1	K02863
ENSG00000198860	-0.976155	5.283386	0.14489	1	TSEN15	K15324
ENSG00000161570	-1.008591	3.655129	0.145044	1	CCL5	K12499
ENSG00000215417	-1.042519	2.907083	0.145556	1	MIR17HG	_
ENSG00000111341	0.979981	4.864223	0.146195	1	MGP	K19481
ENSG00000173991	1.253461	0.900318	0.146339	1	TCAP	_
ENSG00000149289	-1.003228	3.974535	0.146679	1	ZC3H12C	K18668
ENSG00000145819	-0.990602	4.325642	0.146679	1	ARHGAP26	K04613
ENSG00000182873	1.277918	0.755369	0.146868	1	RP11-181G12.2	_
ENSG00000237380	1.277918	0.755369	0.146868	1	HOXD-AS2	_
ENSG00000232274	1.191338	0.786321	0.146868	1	RP11-782C8.2	_
ENSG00000261015	-1.041805	2.752771	0.147358	1	RP1-90J20.11	_
ENSG00000264745	1.292368	0.677217	0.147385	1	RP11-403A21.2	_
ENSG00000272034	-1.408346	0.708544	0.147866	1	SNORD14A	_
ENSG00000203943	-1.36418	0.67786	0.147866	1	SAMD13	_
ENSG00000133818	-1.067724	2.686731	0.148053	1	RRAS2	K07830
ENSG00000233264	-1.741158	0.101745	0.148066	1	AC006042.8	K12735
ENSG00000107551	1.667396	-0.085303	0.148066	1	RASSF4	K09851
ENSG00000133985	1.667396	-0.085303	0.148066	1	TTC9	_
ENSG00000161328	1.478988	-0.028006	0.148066	1	LRRC56	K09228
ENSG00000265692	1.478988	-0.028006	0.148066	1	RP13-516M14.4	_
ENSG00000213366	-1.482098	0.533788	0.14827	1	GSTM2	K00799
ENSG00000228889	-1.482098	0.533788	0.14827	1	UBAC2-AS1	_
ENSG00000253686	1.348574	0.414603	0.14827	1	CTB-43E15.3	_
ENSG00000267424	1.348574	0.414603	0.14827	1	CTD-2265O21.3	_
ENSG00000164167	-1.012493	3.376251	0.14837	1	LSM6	K12625
ENSG00000273373	0.998981	3.343535	0.14837	1	RP5-1074L1.4	_
ENSG00000249328	-1.526942	0.293621	0.14853	1	RP11-26J3.1	_
ENSG00000261136	1.437009	0.095555	0.14853	1	RP11-37C7.3	_

ENSG00000106665	1.50865	0.271403	0.14853	1	CLIP2	K10422
ENSG00000272264	1.373317	0.315652	0.14853	1	RP11-92K15.3	_
ENSG00000141682	-0.958482	6.295375	0.148949	1	PMAIP1	K10131
ENSG00000256683	0.999601	3.771373	0.148969	1	ZNF350	K02927
ENSG00000151773	-1.119241	2.011008	0.149176	1	CCDC122	K17985
ENSG00000150782	-0.956066	6.538382	0.149523	1	IL18	K05482
ENSG00000227184	1.009965	3.244831	0.149638	1	EPPK1	K10388
ENSG00000163435	0.962747	5.310616	0.150216	1	ELF3	K09429
ENSG00000082146	-1.002295	3.49347	0.150298	1	STRADB	K17532
ENSG00000269900	1.036801	2.458085	0.150503	1	RMRP	_
ENSG00000196116	-1.004289	3.206169	0.150781	1	TDRD7	K18405
ENSG00000125148	-0.952783	6.580525	0.150946	1	MT2A	K14739
ENSG00000206077	1.073909	1.793248	0.151025	1	ZDHHC11B	K18932
ENSG00000173614	-1.003717	3.446077	0.151057	1	NMNAT1	K06210
ENSG00000132031	-1.166495	1.812724	0.15172	1	MATN3	K19467
ENSG00000245970	-1.027937	2.822392	0.151877	1	KB-1208A12.3	_
ENSG00000115539	-0.973657	4.353591	0.151992	1	PDCL3	_
ENSG00000119508	0.965288	4.54024	0.152167	1	NR4A3	K08559
ENSG00000197562	0.952287	5.919739	0.152993	1	RAB40C	K07928
ENSG00000110195	0.942312	9.098781	0.153125	1	FOLR1	K13649
ENSG00000141401	0.94316	7.679004	0.153453	1	IMPA2	K01092
ENSG00000162694	-0.951727	5.433422	0.154012	1	EXTL2	_
ENSG00000136631	-1.193429	1.653416	0.154099	1	VPS45	K12479
ENSG00000164308	-1.093053	2.251851	0.154327	1	ERAP2	K13723
ENSG00000157514	0.944093	5.823467	0.1554	1	TSC22D3	_
ENSG00000260006	1.067145	2.097843	0.155689	1	RP11-469M7.1	_
ENSG00000103269	-1.063413	2.594998	0.155702	1	RHBDL1	K02857
ENSG00000268621	0.95059	5.377829	0.155786	1	AC006262.5	_
ENSG00000105499	1.1337	1.438049	0.155983	1	PLA2G4C	K16342
ENSG00000237491	1.1337	1.438049	0.155983	1	RP11-206L10.9	_
ENSG00000258441	-1.089395	2.125879	0.156428	1	LINC00641	_
ENSG00000154359	-0.995771	3.512678	0.156804	1	LONRF1	_
ENSG00000127995	-1.01604	3.092815	0.157104	1	CASD1	_
ENSG00000152518	-0.940914	6.352118	0.15729	1	ZFP36L2	K18753
ENSG00000102393	1.036766	2.434015	0.157497	1	GLA	K01189
ENSG00000100003	0.965878	4.025149	0.157543	1	SEC14L2	_
ENSG00000107020	-0.987708	3.619694	0.158036	1	PLGRKT	_
ENSG00000184788	-1.03689	2.432515	0.158837	1	SATL1	_
ENSG00000203706	-0.969002	3.968627	0.159256	1	SERTAD4-AS1	_
ENSG00000111335	-1.168176	1.318236	0.159401	1	OAS2	K14216
ENSG00000162086	-1.168176	1.318236	0.159401	1	ZNF75A	K09229
ENSG00000155130	-0.931426	7.050736	0.159486	1	MARCKS	K12561
ENSG00000262333	1.036613	2.333562	0.159556	1	HNRNPA1P16	K12741
ENSG00000226608	1.07214	1.866607	0.159851	1	FTLP3	K13625
ENSG00000198774	-0.984002	3.504815	0.16042	1	RASSF9	K09856
ENSG00000259959	-1.217448	1.227876	0.160738	1	RP11-121C2.2	_
ENSG00000149131	0.949137	4.4566	0.160774	1	SERPING1	K04001
ENSG00000230551	0.953602	4.086116	0.161017	1	CTB-89H12.4	_
ENSG00000151491	-0.960586	4.292363	0.161102	1	EPS8	K17277
ENSG00000006047	5.234787	-1.779887	0.161491	1	YBX2	K09277
ENSG00000086506	5.234787	-1.779887	0.161491	1	HBQ1	K13827
ENSG00000107736	5.234787	-1.779887	0.161491	1	CDH23	K06813
ENSG00000109472	5.234787	-1.779887	0.161491	1	CPE	K01294
ENSG00000110375	5.234787	-1.779887	0.161491	1	UPK2	_
ENSG00000111679	5.234787	-1.779887	0.161491	1	PTPN6	K05697
ENSG00000129167	5.234787	-1.779887	0.161491	1	TPH1	K00502
ENSG00000130812	5.234787	-1.779887	0.161491	1	ANGPTL6	_
ENSG00000137745	5.234787	-1.779887	0.161491	1	MMP13	K07994

ENSG00000138769	5.234787	-1.779887	0.161491	1	CDKL2	K08824
ENSG00000145491	5.234787	-1.779887	0.161491	1	ROPN1L	_
ENSG00000165695	5.234787	-1.779887	0.161491	1	AK8	K00939
ENSG00000167641	5.234787	-1.779887	0.161491	1	PPP1R14A	K12328
ENSG00000168528	5.234787	-1.779887	0.161491	1	SERINC2	_
ENSG00000177688	5.234787	-1.779887	0.161491	1	SUMO4	K12160
ENSG00000182742	5.234787	-1.779887	0.161491	1	HOXB4	K09304
ENSG00000183346	5.234787	-1.779887	0.161491	1	C10orf107	_
ENSG00000183570	5.234787	-1.779887	0.161491	1	PCBP3	K04006
ENSG00000184414	5.234787	-1.779887	0.161491	1	RP11-44M6.3	K17985
ENSG00000184497	5.234787	-1.779887	0.161491	1	TMEM255B	_
ENSG00000187686	5.234787	-1.779887	0.161491	1	KRT18P59	K07604
ENSG00000199130	5.234787	-1.779887	0.161491	1	MIR365A	_
ENSG00000199851	5.234787	-1.779887	0.161491	1	U3	_
ENSG00000200120	5.234787	-1.779887	0.161491	1	Y_RNA	_
ENSG00000201003	5.234787	-1.779887	0.161491	1	SNORA58	_
ENSG00000201749	5.234787	-1.779887	0.161491	1	Y_RNA	_
ENSG00000201793	5.234787	-1.779887	0.161491	1	RN7SKP9	_
ENSG00000202415	5.234787	-1.779887	0.161491	1	RN7SKP269	_
ENSG00000203364	5.234787	-1.779887	0.161491	1	RP11-370F5.4	_
ENSG00000203523	5.234787	-1.779887	0.161491	1	TAS2R2P	K08474
ENSG00000204220	5.234787	-1.779887	0.161491	1	PFDN6	K04798
ENSG00000206674	5.234787	-1.779887	0.161491	1	RNU6-945P	_
ENSG00000206881	5.234787	-1.779887	0.161491	1	RNU6-190P	_
ENSG00000213761	5.234787	-1.779887	0.161491	1	MT1P1	K14739
ENSG00000214659	5.234787	-1.779887	0.161491	1	KRT8P26	K07605
ENSG00000216031	5.234787	-1.779887	0.161491	1	MIR298	_
ENSG00000223522	5.234787	-1.779887	0.161491	1	AC093690.1	_
ENSG00000224097	5.234787	-1.779887	0.161491	1	RP11-472B18.1	K14963
ENSG00000225203	5.234787	-1.779887	0.161491	1	TBC1D4-AS1	_
ENSG00000226917	5.234787	-1.779887	0.161491	1	RP11-328M4.3	_
ENSG00000228057	5.234787	-1.779887	0.161491	1	SEC63P1	K09540
ENSG00000228115	5.234787	-1.779887	0.161491	1	RP11-165F24.5	_
ENSG00000228709	5.234787	-1.779887	0.161491	1	AP001065.15	_
ENSG00000230628	5.234787	-1.779887	0.161491	1	RP4-781K5.6	_
ENSG00000230679	5.234787	-1.779887	0.161491	1	ENO1-AS1	_
ENSG00000232692	5.234787	-1.779887	0.161491	1	AP001596.6	_
ENSG00000233266	5.234787	-1.779887	0.161491	1	HMGB1P31	K10802
ENSG00000233799	5.234787	-1.779887	0.161491	1	AC139887.4	_
ENSG00000234796	5.234787	-1.779887	0.161491	1	AC113607.3	_
ENSG00000236426	5.234787	-1.779887	0.161491	1	RP11-79M19.2	_
ENSG00000236658	5.234787	-1.779887	0.161491	1	RP11-83J21.3	_
ENSG00000237452	5.234787	-1.779887	0.161491	1	AC074212.3	_
ENSG00000238651	5.234787	-1.779887	0.161491	1	snoU13	_
ENSG00000239040	5.234787	-1.779887	0.161491	1	Y_RNA	_
ENSG00000239581	5.234787	-1.779887	0.161491	1	RP11-19G24.1	K02981
ENSG00000240634	5.234787	-1.779887	0.161491	1	RP11-1348G14.1	K02957
ENSG00000241120	5.234787	-1.779887	0.161491	1	HMGN1P8	_
ENSG00000242553	5.234787	-1.779887	0.161491	1	AP001432.14	_
ENSG00000244021	5.234787	-1.779887	0.161491	1	RP11-50D9.1	K02889
ENSG00000244556	5.234787	-1.779887	0.161491	1	ODCP	K01581
ENSG00000249664	5.234787	-1.779887	0.161491	1	CTD-2227C6.2	_
ENSG00000250027	5.234787	-1.779887	0.161491	1	RP11-563E2.2	_
ENSG00000250644	5.234787	-1.779887	0.161491	1	RP11-295K3.1	K01379
ENSG00000250938	5.234787	-1.779887	0.161491	1	RP11-679C8.2	_
ENSG00000251056	5.234787	-1.779887	0.161491	1	ANKRD20A17P	K17299
ENSG00000251294	5.234787	-1.779887	0.161491	1	RP11-116O11.1	_
ENSG00000252192	5.234787	-1.779887	0.161491	1	SNORA9	_

ENSG00000252465	5.234787	-1.779887	0.161491	1	RMRPP3	_
ENSG00000252473	5.234787	-1.779887	0.161491	1	SNORA67	_
ENSG00000253305	5.234787	-1.779887	0.161491	1	PCDHGB6	K16496
ENSG00000253559	5.234787	-1.779887	0.161491	1	OSGEPL1-AS1	_
ENSG00000253833	5.234787	-1.779887	0.161491	1	RP11-367E12.4	K10363
ENSG00000254119	5.234787	-1.779887	0.161491	1	RP11-705O24.1	_
ENSG00000255389	5.234787	-1.779887	0.161491	1	C6orf3	_
ENSG00000255398	5.234787	-1.779887	0.161491	1	HCAR3	K08402
ENSG00000256824	5.234787	-1.779887	0.161491	1	RP11-21A7A.2	_
ENSG00000256955	5.234787	-1.779887	0.161491	1	RP11-417L19.2	_
ENSG00000257802	5.234787	-1.779887	0.161491	1	MRS2P2	K16075
ENSG00000257953	5.234787	-1.779887	0.161491	1	RP11-620J15.1	_
ENSG00000258443	5.234787	-1.779887	0.161491	1	RP3-414A15.11	_
ENSG00000259242	5.234787	-1.779887	0.161491	1	AC002306.1	_
ENSG00000259709	5.234787	-1.779887	0.161491	1	CTD-2184D3.7	_
ENSG00000259767	5.234787	-1.779887	0.161491	1	RP11-90B9.2	_
ENSG00000260536	5.234787	-1.779887	0.161491	1	RP5-1085F17.4	_
ENSG00000261442	5.234787	-1.779887	0.161491	1	RP11-709D24.6	_
ENSG00000262519	5.234787	-1.779887	0.161491	1	TXNP4	K03671
ENSG00000262528	5.234787	-1.779887	0.161491	1	LA16c-349E10.1	_
ENSG00000263839	5.234787	-1.779887	0.161491	1	CR381653.1	_
ENSG00000264290	5.234787	-1.779887	0.161491	1	RP11-68I3.4	_
ENSG00000265280	5.234787	-1.779887	0.161491	1	AC018804.1	_
ENSG00000265912	5.234787	-1.779887	0.161491	1	RP11-583F2.2	_
ENSG00000266445	5.234787	-1.779887	0.161491	1	RP13-991F5.2	_
ENSG00000266955	5.234787	-1.779887	0.161491	1	RP11-820I16.3	_
ENSG00000266998	5.234787	-1.779887	0.161491	1	RP11-936I5.1	_
ENSG00000267191	5.234787	-1.779887	0.161491	1	RP11-15A1.2	_
ENSG00000267272	5.234787	-1.779887	0.161491	1	LINC01140	_
ENSG00000267387	5.234787	-1.779887	0.161491	1	CTD-2240E14.4	_
ENSG00000267412	5.234787	-1.779887	0.161491	1	CTC-265F19.2	_
ENSG00000267457	5.234787	-1.779887	0.161491	1	RP5-837J1.4	_
ENSG00000267938	5.234787	-1.779887	0.161491	1	AC007292.1	K03113
ENSG00000268941	5.234787	-1.779887	0.161491	1	MGC4294	_
ENSG00000270482	5.234787	-1.779887	0.161491	1	RP11-131L12.2	_
ENSG00000271715	5.234787	-1.779887	0.161491	1	CTD-2256P15.5	_
ENSG00000271755	5.234787	-1.779887	0.161491	1	RP1-153G14.4	_
ENSG00000272254	5.234787	-1.779887	0.161491	1	RP11-531A24.7	_
ENSG00000272955	5.234787	-1.779887	0.161491	1	KB-226F1.1	_
ENSG00000273272	5.234787	-1.779887	0.161491	1	CTA-384D8.34	_
ENSG00000101096	-5.196961	-1.349079	0.161491	1	NFATC2	K17332
ENSG00000101333	-5.196961	-1.349079	0.161491	1	PLCB4	K05858
ENSG00000105996	-5.196961	-1.349079	0.161491	1	HOXA2	K09302
ENSG00000154646	-5.196961	-1.349079	0.161491	1	TMPRSS15	K01316
ENSG00000160396	-5.196961	-1.349079	0.161491	1	HIPK4	K08826
ENSG00000163530	-5.196961	-1.349079	0.161491	1	DPPA2	_
ENSG00000164746	-5.196961	-1.349079	0.161491	1	C7orf57	_
ENSG00000165168	-5.196961	-1.349079	0.161491	1	CYBB	K08008
ENSG00000167861	-5.196961	-1.349079	0.161491	1	HID1	_
ENSG00000170681	-5.196961	-1.349079	0.161491	1	MURC	K12022
ENSG00000173137	-5.196961	-1.349079	0.161491	1	ADCK5	K08869
ENSG00000175697	-5.196961	-1.349079	0.161491	1	GPR156	K04617
ENSG00000175820	-5.196961	-1.349079	0.161491	1	CCDC168	_
ENSG00000181418	-5.196961	-1.349079	0.161491	1	DDN	_
ENSG00000182168	-5.196961	-1.349079	0.161491	1	UNC5C	K07521
ENSG00000182901	-5.196961	-1.349079	0.161491	1	RGS7	K16449
ENSG00000183281	-5.196961	-1.349079	0.161491	1	PLGLB1	K01315
ENSG00000188536	-5.196961	-1.349079	0.161491	1	HBA2	K13822

ENSG00000214185	-5.196961	-1.349079	0.161491	1	XPOTP1	K14288
ENSG00000215023	-5.196961	-1.349079	0.161491	1	AC114730.5	_
ENSG00000215244	-5.196961	-1.349079	0.161491	1	RP11-563J2.2	_
ENSG00000224647	-5.196961	-1.349079	0.161491	1	AC026954.6	K16833
ENSG00000224777	-5.196961	-1.349079	0.161491	1	OR4F2P	K04257
ENSG00000226193	-5.196961	-1.349079	0.161491	1	RP3-398G3.5	_
ENSG00000229214	-5.196961	-1.349079	0.161491	1	LINC00242	_
ENSG00000232010	-5.196961	-1.349079	0.161491	1	AP001059.5	_
ENSG00000234378	-5.196961	-1.349079	0.161491	1	AC098828.2	_
ENSG00000234406	-5.196961	-1.349079	0.161491	1	AC004129.7	K11366
ENSG00000234427	-5.196961	-1.349079	0.161491	1	RP3-413H6.2	_
ENSG00000235989	-5.196961	-1.349079	0.161491	1	MORC2-AS1	_
ENSG00000236558	-5.196961	-1.349079	0.161491	1	AC138655.6	_
ENSG00000237198	-5.196961	-1.349079	0.161491	1	FAM27E1	_
ENSG00000237356	-5.196961	-1.349079	0.161491	1	AL163953.3	_
ENSG00000238245	-5.196961	-1.349079	0.161491	1	MYO5BP2	K10357
ENSG00000242798	-5.196961	-1.349079	0.161491	1	RP11-506M12.1	_
ENSG00000246477	-5.196961	-1.349079	0.161491	1	AF131216.6	_
ENSG00000246877	-5.196961	-1.349079	0.161491	1	DNM1P35	_
ENSG00000248479	-5.196961	-1.349079	0.161491	1	RP11-807H7.2	_
ENSG00000251348	-5.196961	-1.349079	0.161491	1	HSPD1P11	K04077
ENSG00000251442	-5.196961	-1.349079	0.161491	1	LINC01094	_
ENSG00000254003	-5.196961	-1.349079	0.161491	1	CTB-167B5.1	_
ENSG00000259687	-5.196961	-1.349079	0.161491	1	RP11-293M10.5	_
ENSG00000259940	-5.196961	-1.349079	0.161491	1	CTD-3203P2.1	_
ENSG00000266750	-5.196961	-1.349079	0.161491	1	MIR4645	_
ENSG00000267398	-5.196961	-1.349079	0.161491	1	AC006130.4	K02917
ENSG00000089558	-4.941771	-1.495101	0.161491	1	KCNH4	K04907
ENSG00000102879	-4.941771	-1.495101	0.161491	1	CORO1A	K13882
ENSG00000103942	-4.941771	-1.495101	0.161491	1	HOMER2	_
ENSG00000104237	-4.941771	-1.495101	0.161491	1	RP1	K19538
ENSG00000105792	-4.941771	-1.495101	0.161491	1	C7orf63	_
ENSG00000112041	-4.941771	-1.495101	0.161491	1	TULP1	K19600
ENSG00000119865	-4.941771	-1.495101	0.161491	1	CNRIP1	_
ENSG00000120160	-4.941771	-1.495101	0.161491	1	EQTN	_
ENSG00000128394	-4.941771	-1.495101	0.161491	1	APOBEC3F	K18750
ENSG00000130427	-4.941771	-1.495101	0.161491	1	EPO	K05437
ENSG00000130985	-4.941771	-1.495101	0.161491	1	UBA1	K03178
ENSG00000137968	-4.941771	-1.495101	0.161491	1	SLC44A5	K15377
ENSG00000142765	-4.941771	-1.495101	0.161491	1	SYTL1	K17598
ENSG00000149646	-4.941771	-1.495101	0.161491	1	CNBD2	_
ENSG00000155026	-4.941771	-1.495101	0.161491	1	RSPH10B	_
ENSG00000156966	-4.941771	-1.495101	0.161491	1	B3GNT7	K09664
ENSG00000164099	-4.941771	-1.495101	0.161491	1	PRSS12	K09624
ENSG00000167751	-4.941771	-1.495101	0.161491	1	KLK2	K01325
ENSG00000171509	-4.941771	-1.495101	0.161491	1	RXFP1	K04306
ENSG00000172159	-4.941771	-1.495101	0.161491	1	FRMD3	_
ENSG00000173705	-4.941771	-1.495101	0.161491	1	SUSD5	K17985
ENSG00000174353	-4.941771	-1.495101	0.161491	1	STAG3L3	K13055
ENSG00000178445	-4.941771	-1.495101	0.161491	1	GLDC	K00281
ENSG00000181123	-4.941771	-1.495101	0.161491	1	RP4-539M6.14	_
ENSG00000186281	-4.941771	-1.495101	0.161491	1	GPAT2	K00629
ENSG00000188778	-4.941771	-1.495101	0.161491	1	ADRB3	K04143
ENSG00000188987	-4.941771	-1.495101	0.161491	1	HIST1H4D	K11254
ENSG00000196862	-4.941771	-1.495101	0.161491	1	RGPD4	K12172
ENSG00000198515	-4.941771	-1.495101	0.161491	1	CNGA1	K04948
ENSG00000204929	-4.941771	-1.495101	0.161491	1	AC074391.1	_
ENSG00000205293	-4.941771	-1.495101	0.161491	1	RP11-1112C15.1	_

ENSG00000207704	-4.941771	-1.495101	0.161491	1	MIR548D1	_
ENSG00000208005	-4.941771	-1.495101	0.161491	1	MIR503	_
ENSG00000208028	-4.941771	-1.495101	0.161491	1	MIR616	_
ENSG00000223656	-4.941771	-1.495101	0.161491	1	HMGB3P10	K11296
ENSG00000224152	-4.941771	-1.495101	0.161491	1	AC009506.1	_
ENSG00000227603	-4.941771	-1.495101	0.161491	1	RP11-165J3.6	_
ENSG00000228126	-4.941771	-1.495101	0.161491	1	LINC00568	_
ENSG00000228606	-4.941771	-1.495101	0.161491	1	RP11-574F21.2	_
ENSG00000229766	-4.941771	-1.495101	0.161491	1	RP5-971N18.3	_
ENSG00000229870	-4.941771	-1.495101	0.161491	1	RP11-507K13.6	K02889
ENSG00000230212	-4.941771	-1.495101	0.161491	1	AP000688.14	_
ENSG00000230732	-4.941771	-1.495101	0.161491	1	AC127904.2	_
ENSG00000231811	-4.941771	-1.495101	0.161491	1	RP3-527G5.1	_
ENSG00000233672	-4.941771	-1.495101	0.161491	1	RNASEH2B-AS1	_
ENSG00000233847	-4.941771	-1.495101	0.161491	1	AC104113.3	_
ENSG00000234535	-4.941771	-1.495101	0.161491	1	RP11-37L2.1	_
ENSG00000235670	-4.941771	-1.495101	0.161491	1	RPL21P40	K02889
ENSG00000235721	-4.941771	-1.495101	0.161491	1	AC013268.3	_
ENSG00000235978	-4.941771	-1.495101	0.161491	1	AC018816.3	_
ENSG00000237061	-4.941771	-1.495101	0.161491	1	RP11-956J14.1	_
ENSG00000237361	-4.941771	-1.495101	0.161491	1	LINC01071	_
ENSG00000237689	-4.941771	-1.495101	0.161491	1	AC007064.24	_
ENSG00000239353	-4.941771	-1.495101	0.161491	1	RP11-492E3.51	_
ENSG00000239731	-4.941771	-1.495101	0.161491	1	RN7SL825P	_
ENSG00000239815	-4.941771	-1.495101	0.161491	1	RP11-309L24.4	_
ENSG00000240057	-4.941771	-1.495101	0.161491	1	RP11-572M11.4	_
ENSG00000242575	-4.941771	-1.495101	0.161491	1	AC012501.3	K07374
ENSG00000243024	-4.941771	-1.495101	0.161491	1	RPS11P6	_
ENSG00000244198	-4.941771	-1.495101	0.161491	1	RP4-545C24.1	_
ENSG00000244393	-4.941771	-1.495101	0.161491	1	RP11-592B15.3	_
ENSG00000245281	-4.941771	-1.495101	0.161491	1	CTD-2547L16.1	_
ENSG00000248280	-4.941771	-1.495101	0.161491	1	RP11-33B1.2	K13051
ENSG00000248949	-4.941771	-1.495101	0.161491	1	RP11-661C8.2	_
ENSG00000250654	-4.941771	-1.495101	0.161491	1	RP11-834C11.7	_
ENSG00000250948	-4.941771	-1.495101	0.161491	1	RP11-1079K10.2	_
ENSG00000251400	-4.941771	-1.495101	0.161491	1	ALDH7A1P1	K14085
ENSG00000251402	-4.941771	-1.495101	0.161491	1	FAM90A25P	_
ENSG00000253213	-4.941771	-1.495101	0.161491	1	RP11-546B8.3	K11842
ENSG00000253398	-4.941771	-1.495101	0.161491	1	RP11-775B15.2	_
ENSG00000255650	-4.941771	-1.495101	0.161491	1	FAM222A-AS1	_
ENSG00000256377	-4.941771	-1.495101	0.161491	1	RP11-1060J15.4	_
ENSG00000257464	-4.941771	-1.495101	0.161491	1	RP11-161H23.8	K11299
ENSG00000259022	-4.941771	-1.495101	0.161491	1	DNAJC8P1	K09528
ENSG00000259211	-4.941771	-1.495101	0.161491	1	RP11-64K12.8	_
ENSG00000259216	-4.941771	-1.495101	0.161491	1	RP11-227D13.2	_
ENSG00000260095	-4.941771	-1.495101	0.161491	1	RP11-715J22.3	_
ENSG00000260877	-4.941771	-1.495101	0.161491	1	RP11-211G23.2	_
ENSG00000261122	-4.941771	-1.495101	0.161491	1	5S_rRNA	_
ENSG00000261513	-4.941771	-1.495101	0.161491	1	RP11-432I5.8	_
ENSG00000262728	-4.941771	-1.495101	0.161491	1	AC123768.4	_
ENSG00000263842	-4.941771	-1.495101	0.161491	1	AC104986.1	_
ENSG00000266495	-4.941771	-1.495101	0.161491	1	RP11-17J14.2	_
ENSG00000266896	-4.941771	-1.495101	0.161491	1	RP1-266L20.9	_
ENSG00000267607	-4.941771	-1.495101	0.161491	1	CTD-2369P2.8	_
ENSG00000269949	-4.941771	-1.495101	0.161491	1	RP11-738E22.3	_
ENSG00000270168	-4.941771	-1.495101	0.161491	1	LA16c-380H5.4	_
ENSG00000270177	-4.941771	-1.495101	0.161491	1	CTD-2410N18.3	_
ENSG00000271500	-4.941771	-1.495101	0.161491	1	RP11-288K12.1	K14856

ENSG00000272223	-4.941771	-1.495101	0.161491	1	RP1-20C7.6	_
ENSG00000272544	-4.941771	-1.495101	0.161491	1	CTA-357J21.1	_
ENSG00000272844	-4.941771	-1.495101	0.161491	1	RP11-484K9.4	_
ENSG00000272858	-4.941771	-1.495101	0.161491	1	CTA-292E10.8	_
ENSG00000273116	-4.941771	-1.495101	0.161491	1	LLfos-48D6.1	_
ENSG00000273485	-4.941771	-1.495101	0.161491	1	RP11-225H22.7	_
ENSG00000112186	-0.940976	4.908921	0.161523	1	CAP2	K17261
ENSG00000170271	1.036437	2.225693	0.161941	1	FAXDC2	K07750
ENSG00000254667	0.945277	4.395197	0.162191	1	AP000783.1	_
ENSG00000129317	-0.956724	4.134537	0.162671	1	PUS7L	K06176
ENSG00000235162	-0.965161	3.806127	0.163061	1	C12orf75	_
ENSG00000125848	-1.210038	1.08637	0.163786	1	FLRT3	K16362
ENSG00000249459	-1.210038	1.08637	0.163786	1	ZNF286B	K09228
ENSG00000261762	-1.210038	1.08637	0.163786	1	RP11-650L12.2	_
ENSG00000263826	-1.210038	1.08637	0.163786	1	RP11-573D15.9	_
ENSG00000144724	-1.176225	1.063208	0.163786	1	PTPRG	K16667
ENSG00000272338	-1.176225	1.063208	0.163786	1	RP11-722E23.2	_
ENSG00000187091	1.176162	0.928129	0.163786	1	PLCD1	K05857
ENSG00000243156	0.933565	4.89872	0.164195	1	MICAL3	_
ENSG00000173546	0.9188	6.989561	0.16488	1	CSPG4	K08115
ENSG00000114315	-0.940412	4.704589	0.165189	1	HES1	K06054
ENSG00000095370	1.183352	0.858916	0.165529	1	SH2D3C	_
ENSG00000166592	1.183352	0.858916	0.165529	1	RRAD	K07845
ENSG00000147383	0.983639	2.872452	0.165999	1	NSDHL	K07748
ENSG00000138166	-0.921949	5.996899	0.16637	1	DUSP5	K04459
ENSG00000178172	0.926515	4.919256	0.166378	1	SPINK6	_
ENSG00000233077	1.127603	1.530906	0.166992	1	RP11-290F20.2	_
ENSG00000253785	-1.352998	0.864023	0.167446	1	CTC-308K20.3	K01507
ENSG00000134376	-0.966166	3.492934	0.16791	1	CRB1	K16681
ENSG00000234287	0.924603	4.718198	0.169136	1	RP11-761N21.2	K02978
ENSG00000099958	1.21029	0.629559	0.169557	1	DERL3	K13989
ENSG00000244586	1.21029	0.629559	0.169557	1	WNT5A-AS1	_
ENSG00000157216	0.916199	5.703499	0.16956	1	SSBP3	_
ENSG00000114480	0.914569	5.824209	0.169764	1	GBE1	K00700
ENSG00000139194	1.084926	1.408653	0.170178	1	RBP5	_
ENSG00000127920	-0.907772	7.007695	0.170246	1	GNG11	K04546
ENSG00000253981	-0.966525	3.39645	0.17095	1	ALG1L13P	K03842
ENSG00000142207	0.906533	6.403372	0.171686	1	URB1	K14861
ENSG00000146197	1.327252	0.507388	0.171886	1	SCUBE3	_
ENSG00000199133	1.327252	0.507388	0.171886	1	MIRLET7D	_
ENSG00000225880	1.221653	0.544525	0.171886	1	LINC00115	_
ENSG00000226332	-1.17804	1.439111	0.171981	1	RP11-157P1.4	_
ENSG00000116761	0.903943	6.676488	0.172055	1	CTH	K01758
ENSG00000085063	0.899938	9.042192	0.172165	1	CD59	K04008
ENSG00000123384	0.901808	7.456672	0.172195	1	LRP1	K04550
ENSG00000165389	0.900794	7.565512	0.172573	1	SPTSSA	K01353
ENSG00000137502	0.905244	6.152897	0.172685	1	RAB30	K07917
ENSG00000128422	0.89807	10.31497	0.172777	1	KRT17	K07604
ENSG00000070182	0.956051	3.004671	0.17281	1	SPTB	K06115
ENSG00000227036	0.956051	3.004671	0.17281	1	LINC00511	_
ENSG00000160766	-0.991771	2.677916	0.172973	1	GBAP1	K01201
ENSG00000120211	0.933475	3.640892	0.17318	1	INSL4	K05255
ENSG00000138061	-0.899357	7.68574	0.173356	1	CYP1B1	K07410
ENSG00000230177	1.074865	1.755149	0.1736	1	RP5-1112D6.4	_
ENSG00000168702	1.035518	1.769852	0.1736	1	LRP1B	_
ENSG00000255414	1.090319	1.254434	0.173951	1	LINC01059	_
ENSG00000272341	1.090319	1.254434	0.173951	1	RP1-151F17.2	_
ENSG00000196437	-1.431769	0.498804	0.174456	1	ZNF569	K09228

ENSG00000229666	-1.431769	0.498804	0.174456	1	MAST4-AS1	_
ENSG00000220749	1.249598	0.358371	0.174456	1	RPL21P28	K02889
ENSG00000259328	1.249598	0.358371	0.174456	1	RP11-152F13.7	K08622
ENSG00000064205	0.895785	8.193043	0.174489	1	WISP2	K06827
ENSG00000230715	0.985237	2.352613	0.174694	1	RP11-274B21.4	_
ENSG00000215039	1.035327	1.691453	0.175242	1	CD27-AS1	_
ENSG00000251192	-1.057745	2.104366	0.175703	1	ZNF674	K09228
ENSG00000100342	-0.923043	4.280899	0.176497	1	APOL1	K14480
ENSG00000213672	0.904731	5.088006	0.176803	1	NCKIPSD	_
ENSG00000236901	0.957113	2.708546	0.176873	1	MIR600HG	_
ENSG00000261780	0.967758	2.507298	0.177002	1	CTD-2354A18.1	_
ENSG00000243137	0.918362	4.365793	0.177018	1	PSG4	K06500
ENSG00000141429	-0.895703	6.267455	0.177049	1	GALNT1	K00710
ENSG00000107859	1.035985	1.982731	0.17717	1	PITX3	K09357
ENSG00000097096	-0.938924	3.650743	0.177248	1	SYDE2	K17985
ENSG00000204957	1.40238	0.20966	0.177284	1	AC006486.1	K17985
ENSG00000272525	1.40238	0.20966	0.177284	1	RP11-79P5.9	_
ENSG00000110492	1.267047	0.255801	0.177284	1	MDK	K06828
ENSG00000146090	1.267047	0.255801	0.177284	1	RASGEF1C	_
ENSG00000259146	1.267047	0.255801	0.177284	1	RP1-261D10.2	_
ENSG00000262903	1.267047	0.255801	0.177284	1	RP11-235E17.6	_
ENSG00000270195	1.267047	0.255801	0.177284	1	RP11-572O17.1	_
ENSG00000271119	1.267047	0.255801	0.177284	1	CTD-2012J19.3	_
ENSG00000273449	1.267047	0.255801	0.177284	1	RP11-218F10.3	_
ENSG00000172469	-0.934605	3.400088	0.178845	1	MANEA	K15538
ENSG00000204941	0.897299	5.164889	0.178876	1	PSG5	K06500
ENSG00000127415	-0.969998	2.704559	0.178885	1	IDUA	K14700
ENSG00000143850	-0.983778	2.494774	0.179309	1	PLEKHA6	_
ENSG00000156313	-0.967693	3.122374	0.18032	1	RPGR	K19607
ENSG00000206754	-1.465978	0.251663	0.180372	1	SNORD101	_
ENSG00000151164	-1.402324	0.208302	0.180372	1	RAD9B	K10995
ENSG00000180479	-1.402324	0.208302	0.180372	1	ZNF571	K09228
ENSG00000186377	-1.402324	0.208302	0.180372	1	CYP4X1	K07428
ENSG00000198040	-1.402324	0.208302	0.180372	1	ZNF84	K09228
ENSG00000131037	-1.049841	1.963766	0.1804	1	EPS8L1	K17277
ENSG00000260001	-1.049841	1.963766	0.1804	1	TGFBR3L	K15209
ENSG00000155729	-1.009633	2.46397	0.180548	1	KCTD18	_
ENSG00000125246	-0.99716	2.455596	0.180548	1	CLYBL	K11390
ENSG00000268032	1.081918	1.503199	0.180992	1	CTB-52I2.4	K01647
ENSG00000119227	1.034872	1.520929	0.180992	1	PIGZ	K08098
ENSG00000100968	1.169654	0.994261	0.181118	1	NFATC4	K17334
ENSG00000128965	-0.909143	4.271776	0.181237	1	CHAC1	K07232
ENSG00000084764	0.940503	2.755181	0.181468	1	MAPRE3	K10436
ENSG00000027869	-0.970549	2.621316	0.182243	1	SH2D2A	K08273
ENSG00000174799	-0.959824	3.055231	0.182902	1	CEP135	K16461
ENSG00000168274	1.083371	1.456682	0.183242	1	HIST1H2AE	K11251
ENSG00000073464	-1.671341	0.053334	0.183672	1	CLCN4	K05012
ENSG00000139344	-1.671341	0.053334	0.183672	1	AMDHD1	K01468
ENSG00000258791	-1.671341	0.053334	0.183672	1	LINC00520	_
ENSG00000266278	-1.671341	0.053334	0.183672	1	RP11-41O4.2	_
ENSG00000240975	-1.597974	0.003052	0.183672	1	RP11-313M3.1	K02883
ENSG00000254184	-1.597974	0.003052	0.183672	1	TYW1B	K15449
ENSG00000128918	1.530958	-0.162726	0.183672	1	ALDH1A2	K09877
ENSG00000174871	1.530958	-0.162726	0.183672	1	CNIH2	_
ENSG00000225032	1.530958	-0.162726	0.183672	1	RP11-228B15.4	_
ENSG00000239617	1.530958	-0.162726	0.183672	1	RP11-302B13.1	K02912
ENSG00000226268	1.342549	-0.102376	0.183672	1	RP11-61N20.3	K15116
ENSG00000261798	1.342549	-0.102376	0.183672	1	RP1-118J21.25	_

ENSG00000104883	0.946684	2.849261	0.183742	1	PEX11G	K13353
ENSG00000014123	-0.89874	4.415883	0.184063	1	UFL1	_
ENSG00000251003	-1.016707	1.868626	0.184097	1	RP11-152P17.2	_
ENSG00000080031	0.907013	3.987413	0.184538	1	PTPRH	K18034
ENSG00000119699	0.983255	2.300432	0.184662	1	TGFB3	K13377
ENSG00000124813	-0.983031	2.540841	0.184745	1	RUNX2	K09278
ENSG00000028116	-0.892213	5.02473	0.185022	1	VRK2	K08816
ENSG00000128710	0.927371	3.40827	0.185413	1	HOXD10	K09295
ENSG00000182985	-0.895732	4.276198	0.185685	1	CADM1	K06781
ENSG00000261324	-1.088774	1.483589	0.185698	1	RP11-174G6.5	_
ENSG00000143486	0.996078	1.746125	0.186153	1	EIF2D	K15027
ENSG00000101986	0.954238	2.256512	0.186184	1	ABCD1	K05675
ENSG00000260328	-1.041655	2.093476	0.186235	1	RP11-416I2.1	_
ENSG00000146733	-0.884244	5.499601	0.18631	1	PSPH	K01079
ENSG00000196557	0.927238	3.048411	0.186596	1	CACNA1H	K04855
ENSG00000188707	0.951096	2.920385	0.18678	1	ZBED6CL	_
ENSG00000006611	1.597018	-0.310821	0.187026	1	USH1C	_
ENSG00000172748	1.597018	-0.310821	0.187026	1	ZNF596	K09228
ENSG00000215712	1.597018	-0.310821	0.187026	1	TMEM242	_
ENSG00000222493	1.597018	-0.310821	0.187026	1	Y_RNA	_
ENSG00000224882	1.597018	-0.310821	0.187026	1	IKBKGP1	K07210
ENSG00000237976	1.597018	-0.310821	0.187026	1	RP11-126K1.6	_
ENSG00000253330	1.597018	-0.310821	0.187026	1	RP11-697N18.3	K14012
ENSG00000255142	1.597018	-0.310821	0.187026	1	AP006621.6	_
ENSG00000255559	1.597018	-0.310821	0.187026	1	ZNF252P-AS1	_
ENSG00000150051	-0.923257	3.660952	0.187118	1	MKX	_
ENSG00000115525	-0.970265	2.663544	0.187227	1	ST3GAL5	K03370
ENSG00000078596	-1.200741	0.928838	0.187314	1	ITM2A	K18241
ENSG00000128512	-0.971518	2.486583	0.187495	1	DOCK4	K17697
ENSG00000108821	0.873247	6.107572	0.188267	1	COL1A1	K06236
ENSG00000063127	1.034282	1.328144	0.188387	1	SLC6A16	K05048
ENSG00000182263	-0.924949	3.265193	0.188824	1	FIGN	_
ENSG00000105997	-1.727846	-0.364898	0.189984	1	HOXA3	K09303
ENSG00000233483	-1.727846	-0.364898	0.189984	1	CTD-2020K17.4	_
ENSG00000258682	-1.727846	-0.364898	0.189984	1	CTD-2002H8.2	_
ENSG00000261712	-1.727846	-0.364898	0.189984	1	RP11-358L4.1	_
ENSG00000264885	-1.727846	-0.364898	0.189984	1	RP11-815I9.4	_
ENSG00000015520	1.493347	-0.574178	0.189984	1	NPC1L1	K14461
ENSG00000120693	1.493347	-0.574178	0.189984	1	SMAD9	K16791
ENSG00000149634	1.493347	-0.574178	0.189984	1	SPATA25	_
ENSG00000165716	1.493347	-0.574178	0.189984	1	FAM69B	_
ENSG00000197681	1.493347	-0.574178	0.189984	1	TBC1D3	K11837
ENSG00000214439	1.493347	-0.574178	0.189984	1	FAM185BP	_
ENSG00000223486	1.493347	-0.574178	0.189984	1	AC092198.1	_
ENSG00000225950	1.493347	-0.574178	0.189984	1	NTF4	K12457
ENSG00000227344	1.493347	-0.574178	0.189984	1	HAUS6P1	K16589
ENSG00000228852	1.493347	-0.574178	0.189984	1	RP11-57H12.5	_
ENSG00000235865	1.493347	-0.574178	0.189984	1	GSN-AS1	_
ENSG00000239002	1.493347	-0.574178	0.189984	1	SCARNA10	_
ENSG00000242078	1.493347	-0.574178	0.189984	1	RP11-738B7.1	_
ENSG00000243988	1.493347	-0.574178	0.189984	1	RPS24P17	K02974
ENSG00000244617	1.493347	-0.574178	0.189984	1	ASPRV1	_
ENSG00000261008	1.493347	-0.574178	0.189984	1	AC004158.2	_
ENSG00000261560	1.493347	-0.574178	0.189984	1	RP11-166B2.3	_
ENSG00000271659	1.493347	-0.574178	0.189984	1	RP11-435O5.4	_
ENSG00000198517	0.872238	5.447113	0.190392	1	MAFK	K09037
ENSG00000167106	0.86926	6.088927	0.190438	1	FAM102A	_
ENSG00000188739	-1.023472	1.715308	0.190766	1	RBM34	K14837

ENSG00000163220	0.99226	1.625026	0.190766	1	S100A9	_
ENSG00000110446	-1.313651	0.836713	0.190995	1	SLC15A3	K14638
ENSG00000128298	-1.273201	0.808813	0.190995	1	BAIAP2L2	_
ENSG00000161921	1.200259	0.710002	0.190995	1	CXCL16	K10035
ENSG00000230673	1.200259	0.710002	0.190995	1	PABPC1P3	K13126
ENSG00000205413	-0.877217	5.231902	0.191049	1	SAMD9	_
ENSG00000185973	-2.328955	-0.684716	0.191276	1	TMLHE	K00474
ENSG00000048052	-2.328955	-0.684716	0.191276	1	HDAC9	K11409
ENSG00000197599	-2.328955	-0.684716	0.191276	1	CCDC154	_
ENSG00000200127	-2.328955	-0.684716	0.191276	1	Y_RNA	_
ENSG00000212304	-2.328955	-0.684716	0.191276	1	SNORD12	_
ENSG00000213204	-2.328955	-0.684716	0.191276	1	C6orf165	K15272
ENSG00000225051	-2.328955	-0.684716	0.191276	1	HMGB3P22	K11296
ENSG00000227375	-2.328955	-0.684716	0.191276	1	DLG1-AS1	_
ENSG00000235601	-2.328955	-0.684716	0.191276	1	RP11-231K24.2	_
ENSG00000236773	-2.328955	-0.684716	0.191276	1	RP11-365O16.1	_
ENSG00000237276	-2.328955	-0.684716	0.191276	1	ANO7P1	_
ENSG00000268964	-2.328955	-0.684716	0.191276	1	ERVV-2	_
ENSG00000272523	-2.328955	-0.684716	0.191276	1	LINC01023	_
ENSG00000100079	1.979737	-0.871035	0.191276	1	LGALS2	K10090
ENSG00000108309	1.979737	-0.871035	0.191276	1	RUNDC3A	K11447
ENSG00000128656	1.979737	-0.871035	0.191276	1	CHN1	_
ENSG00000132334	1.979737	-0.871035	0.191276	1	PTPRE	K18033
ENSG00000137843	1.979737	-0.871035	0.191276	1	PAK6	K05735
ENSG00000183055	1.979737	-0.871035	0.191276	1	FAM133CP	_
ENSG00000189129	1.979737	-0.871035	0.191276	1	PLAC9	_
ENSG00000197768	1.979737	-0.871035	0.191276	1	C9orf173	_
ENSG00000203392	1.979737	-0.871035	0.191276	1	AC105020.1	K04613
ENSG00000205696	1.979737	-0.871035	0.191276	1	ADARB2-AS1	_
ENSG00000225234	1.979737	-0.871035	0.191276	1	TRAPPC12-AS1	_
ENSG00000226088	1.979737	-0.871035	0.191276	1	RP11-180O5.2	_
ENSG00000228205	1.979737	-0.871035	0.191276	1	RP11-778D9.4	K02985
ENSG00000231908	1.979737	-0.871035	0.191276	1	IDH1-AS1	_
ENSG00000235888	1.979737	-0.871035	0.191276	1	AF064858.8	_
ENSG00000261819	1.979737	-0.871035	0.191276	1	RP11-680G24.4	_
ENSG00000264278	1.979737	-0.871035	0.191276	1	RP11-162A12.2	K12756
ENSG00000265791	1.979737	-0.871035	0.191276	1	CTD-2349P21.10	_
ENSG00000268470	1.979737	-0.871035	0.191276	1	DNAH17-AS1	K10408
ENSG00000271997	1.979737	-0.871035	0.191276	1	RP11-97O12.6	_
ENSG00000272279	1.979737	-0.871035	0.191276	1	RP11-157J24.2	_
ENSG00000023445	-0.877433	4.702655	0.192019	1	BIRC3	K16060
ENSG00000176171	0.861019	7.127989	0.192505	1	BNIP3	K15464
ENSG00000180801	-0.89753	3.887451	0.193197	1	ARSJ	K12375
ENSG00000136161	-1.081009	1.25889	0.194613	1	RCBTB2	K11494
ENSG00000273002	1.033711	1.165088	0.194613	1	RP11-336K24.12	_
ENSG00000137959	-0.959391	2.656299	0.194796	1	IFI44L	_
ENSG00000153048	0.856104	7.175341	0.194965	1	CARHSP1	_
ENSG00000152348	-0.899871	3.327182	0.19502	1	ATG10	K17888
ENSG00000261211	0.941244	2.051365	0.195242	1	RP1-80N2.3	_
ENSG00000111358	-0.864698	5.441466	0.195319	1	GTF2H3	K03143
ENSG00000232956	-0.861758	5.717035	0.195495	1	SNHG15	_
ENSG00000116661	0.856016	6.47971	0.195616	1	FBXO2	K10099
ENSG00000143819	0.859285	5.595609	0.195952	1	EPHX1	K01253
ENSG00000070669	-0.853084	7.409462	0.196517	1	ASNS	K01953
ENSG00000105856	0.856813	6.048234	0.196647	1	HBP1	K09228
ENSG00000065717	0.8575	5.957636	0.197079	1	TLE2	_
ENSG00000154734	-0.888393	3.792668	0.197905	1	ADAMTS1	K08617
ENSG00000168234	0.853697	5.991928	0.198186	1	TTC39C	_

ENSG00000172260	-1.124674	1.164368	0.198245	1	NEGR1	K06775
ENSG00000244968	-1.124674	1.164368	0.198245	1	LIFR-AS1	_
ENSG00000158555	0.857157	5.443074	0.198995	1	GDPD5	K01124
ENSG00000272667	1.034597	1.427672	0.199277	1	RP11-395A13.2	_
ENSG00000134215	0.853995	5.826465	0.199517	1	VAV3	K05730
ENSG00000250461	-1.019896	1.794038	0.199632	1	RP11-631M6.2	_
ENSG00000182621	-1.271574	0.614213	0.199929	1	PLCB1	K05858
ENSG00000205559	-1.271574	0.614213	0.199929	1	CHKB-AS1	_
ENSG00000265678	-1.271574	0.614213	0.199929	1	RP11-1376P16.2	_
ENSG00000164674	1.234632	0.454348	0.199929	1	SYTL3	K17598
ENSG00000196338	1.234632	0.454348	0.199929	1	NLGN3	K07378
ENSG00000228544	1.234632	0.454348	0.199929	1	CCDC183-AS1	_
ENSG00000270673	1.234632	0.454348	0.199929	1	YTHDF3-AS1	_
ENSG00000138161	-1.222941	0.581173	0.199929	1	CUZD1	_
ENSG00000179698	-1.222941	0.581173	0.199929	1	KIAA1875	_
ENSG00000180089	1.129033	0.492852	0.199929	1	TMEM86B	K18575
ENSG00000236199	1.129033	0.492852	0.199929	1	RP11-264I13.2	_
ENSG00000173391	-0.84568	7.785584	0.199974	1	OLR1	K08763
ENSG00000142102	-0.862037	4.899941	0.200059	1	ATHL1	K06566
ENSG00000070759	0.913545	2.880214	0.200597	1	TESK2	K08842
ENSG00000188206	0.869247	4.3162	0.201287	1	HNRNPU-AS1	_
ENSG00000090238	0.877577	3.501256	0.201419	1	YPEL3	_
ENSG00000178700	-0.945606	2.371371	0.201563	1	DHFRL1	K00287
ENSG00000156017	-0.857628	4.837098	0.201945	1	C9orf41	K19787
ENSG00000260948	1.102794	0.955321	0.202302	1	RP11-552M11.8	_
ENSG00000140961	1.032978	0.981921	0.202302	1	OSGIN1	_
ENSG00000159184	1.032978	0.981921	0.202302	1	HOXB13	_
ENSG00000183128	1.032978	0.981921	0.202302	1	CALHM3	K19740
ENSG00000257122	-1.037398	1.448868	0.202662	1	RRN3P3	K15216
ENSG00000072571	-0.849745	5.569357	0.202717	1	HMMR	K06267
ENSG00000169071	0.847814	5.608293	0.202966	1	ROR2	K05123
ENSG00000164414	-0.90862	2.989371	0.20302	1	SLC35A1	K15272
ENSG00000090674	0.855203	4.989022	0.203371	1	MCOLN1	K04992
ENSG00000165533	-0.891344	3.419989	0.203604	1	TTC8	K16781
ENSG00000212907	0.837746	8.298457	0.203626	1	MT-ND4L	K03882
ENSG00000133641	-0.849508	5.207241	0.203687	1	C12orf29	K16533
ENSG00000112972	-0.838713	6.910369	0.204794	1	HMGCS1	K01641
ENSG00000161960	-0.845801	5.446481	0.204841	1	EIF4A1	K03257
ENSG00000160172	-1.013847	1.939489	0.204851	1	FAM86C2P	K03842
ENSG00000177822	0.999319	1.858034	0.204851	1	AC108142.1	_
ENSG00000049130	-0.840699	6.440541	0.205014	1	KITLG	K05461
ENSG00000162511	-1.379621	0.462847	0.205414	1	LAPTM5	K12387
ENSG00000238058	-1.379621	0.462847	0.205414	1	RP11-432J22.2	_
ENSG00000256546	-1.379621	0.462847	0.205414	1	AC156455.1	_
ENSG00000235109	-1.001832	1.700777	0.205609	1	ZSCAN31	K09230
ENSG00000088367	0.844596	5.221509	0.20604	1	EPB41L1	K06107
ENSG00000135002	-0.837816	6.275516	0.20635	1	RFK	K00861
ENSG00000188542	-1.098849	1.38498	0.206388	1	DUSP28	K14165
ENSG00000125510	1.034106	1.275757	0.206388	1	OPRL1	K04216
ENSG00000236810	-1.106123	1.015596	0.206864	1	RP5-886K2.3	_
ENSG00000257950	-1.106123	1.015596	0.206864	1	P2RX5-TAX1BP3	K05219
ENSG00000165905	1.106052	0.887532	0.206864	1	GYLTL1B	K09668
ENSG00000115758	-0.832449	7.118706	0.207815	1	ODC1	K01581
ENSG00000272870	0.989311	1.538405	0.208992	1	RP11-798M19.6	_
ENSG00000183963	0.834691	5.732701	0.209463	1	SMTN	_
ENSG00000082497	-0.832482	6.091709	0.21003	1	SERTAD4	_
ENSG00000065534	0.957776	1.760896	0.210778	1	MYLK	K00907
ENSG00000237424	0.879763	3.176291	0.211063	1	FOXD2-AS1	_

ENSG00000114019	-0.825047	7.458848	0.211375	1	AMOTL2	K16819
ENSG00000174206	-0.877669	3.286959	0.211481	1	C12orf66	_
ENSG00000167094	1.287655	0.14567	0.21179	1	TTC16	_
ENSG00000182253	1.287655	0.14567	0.21179	1	SYNM	K10376
ENSG00000198513	1.287655	0.14567	0.21179	1	ATL1	K17339
ENSG00000205583	1.287655	0.14567	0.21179	1	STAG3L1	K13055
ENSG00000224397	1.287655	0.14567	0.21179	1	RP11-290F20.3	_
ENSG00000249348	1.287655	0.14567	0.21179	1	UGDH-AS1	_
ENSG00000080947	1.152322	0.193847	0.21179	1	CROCCP3	K16469
ENSG00000100060	1.152322	0.193847	0.21179	1	MFNG	K05948
ENSG00000176222	1.152322	0.193847	0.21179	1	ZNF404	K09228
ENSG00000184551	1.152322	0.193847	0.21179	1	AC132872.1	K08959
ENSG00000257108	1.152322	0.193847	0.21179	1	NHLRC4	K03860
ENSG00000258620	1.152322	0.193847	0.21179	1	RP11-362L22.1	_
ENSG00000107443	-1.162438	0.902753	0.21203	1	CCNJ	_
ENSG00000234840	-1.162438	0.902753	0.21203	1	RP11-399D6.2	_
ENSG00000173894	0.934367	1.953858	0.212069	1	CBX2	K11451
ENSG00000182759	0.858842	3.700606	0.212593	1	MAFA	K07595
ENSG00000184574	-1.056292	1.559671	0.212687	1	LPAR5	K08390
ENSG00000123838	0.987693	1.493116	0.212687	1	C4BPA	K04002
ENSG00000117395	-0.8211	8.021554	0.21298	1	EBNA1BP2	K14823
ENSG00000146707	-0.974206	2.171626	0.213042	1	POMZP3	K14316
ENSG00000128284	-0.99948	1.78033	0.2141	1	APOL3	K14480
ENSG00000137440	-0.871519	3.585673	0.21411	1	FGFBP1	_
ENSG00000033170	-0.853585	3.892877	0.215161	1	FUT8	K00717
ENSG00000161091	0.815561	9.266771	0.215292	1	MFSD12	_
ENSG00000204291	0.84571	4.125425	0.215867	1	COL15A1	K08135
ENSG00000244486	0.896617	2.221735	0.216315	1	SCARF2	_
ENSG00000182578	0.909196	2.355335	0.216725	1	CSF1R	K05090
ENSG00000179240	0.984116	1.398162	0.216737	1	RP11-111M22.2	_
ENSG00000261373	-0.853528	3.769976	0.21674	1	VPS9D1-AS1	_
ENSG00000188641	-0.838511	4.3527	0.217017	1	DPYD	K00207
ENSG00000148180	0.815323	6.791141	0.217375	1	GSN	K05768
ENSG00000129474	-0.818994	6.260253	0.217574	1	AJUBA	K16682
ENSG00000039139	-1.231583	0.780296	0.21793	1	DNAH5	K10408
ENSG00000226688	-1.231583	0.780296	0.21793	1	ENTPD1-AS1	_
ENSG00000080854	1.118181	0.663432	0.21793	1	IGSF9B	_
ENSG00000166736	1.118181	0.663432	0.21793	1	HTR3A	K04819
ENSG00000234028	1.118181	0.663432	0.21793	1	AC062029.1	_
ENSG00000261207	1.118181	0.663432	0.21793	1	LA16c-361A3.3	_
ENSG00000102934	-0.829069	4.870262	0.218237	1	PLLP	_
ENSG00000140022	0.866448	3.297614	0.218356	1	STON2	_
ENSG00000153363	-0.942068	2.089839	0.218709	1	LINC00467	_
ENSG00000107099	-1.335731	0.163439	0.219277	1	DOCK8	K13194
ENSG00000196872	-1.335731	0.163439	0.219277	1	KIAA1211L	_
ENSG00000224113	-1.335731	0.163439	0.219277	1	RP5-837J1.2	_
ENSG00000253347	1.312367	0.026781	0.219277	1	CTD-2026D20.2	_
ENSG00000253846	1.312367	0.026781	0.219277	1	PCDHGA10	K16495
ENSG00000254530	1.312367	0.026781	0.219277	1	RP11-460B17.3	_
ENSG00000272822	1.312367	0.026781	0.219277	1	RP11-302B13.5	K07938
ENSG00000106692	-0.831788	4.348368	0.219683	1	FKTN	_
ENSG00000154035	0.856439	3.215104	0.219685	1	C17orf103	K06975
ENSG00000106829	-0.836365	4.170421	0.219698	1	TLE4	_
ENSG00000131981	0.808269	8.10257	0.219906	1	LGALS3	K06831
ENSG00000111731	-0.822298	5.120666	0.220404	1	C2CD5	_
ENSG00000101811	-0.82623	4.790369	0.220634	1	CSTF2	K14407
ENSG00000270696	-0.840532	3.606952	0.220834	1	RP11-342K6.1	_
ENSG00000234665	-1.011007	1.431151	0.221198	1	RP11-262H14.3	_

ENSG00000139974	-0.928421	2.199113	0.221818	1	SLC38A6	K14993
ENSG00000151413	-0.854272	3.047723	0.222922	1	NUBPL	K03593
ENSG00000099194	0.801858	9.433463	0.222957	1	SCD	K00507
ENSG00000179094	0.814252	5.308517	0.223051	1	PER1	K02633
ENSG00000168259	0.821835	4.609303	0.223562	1	DNAJC7	K09527
ENSG00000162729	0.802545	7.529043	0.223624	1	IGSF8	K06730
ENSG00000169136	-0.834932	3.776152	0.223716	1	ATF5	K09044
ENSG00000079805	0.801554	7.38453	0.224342	1	DNM2	K01528
ENSG00000185864	1.123259	0.580574	0.224728	1	NPIPB4	K01896
ENSG00000101236	0.808985	5.688982	0.224901	1	RNF24	_
ENSG00000054938	0.898815	1.932831	0.225187	1	CHRDL2	K17280
ENSG00000171471	0.898815	1.932831	0.225187	1	MAP1LC3B2	K10435
ENSG00000131094	0.817665	4.715297	0.225372	1	C1QL1	_
ENSG00000137767	-0.806967	5.930592	0.225452	1	SQRDL	K17218
ENSG00000054392	-0.883303	2.647238	0.225946	1	HHAT	_
ENSG00000132376	0.807447	5.0869	0.226481	1	INPP5K	K01106
ENSG00000161513	-0.816783	4.477538	0.226494	1	FDXR	K18914
ENSG00000165996	0.843183	3.11254	0.227412	1	PTPLA	K10703
ENSG00000091622	0.802319	5.930376	0.227483	1	PITPNM3	_
ENSG00000154188	-0.8222	4.28017	0.227513	1	ANGPT1	K05465
ENSG00000177138	-1.520674	-0.04925	0.228158	1	FAM9B	K19528
ENSG00000204011	-1.520674	-0.04925	0.228158	1	COL5A1-AS1	_
ENSG00000261270	-1.520674	-0.04925	0.228158	1	RP11-325K4.3	_
ENSG00000271303	0.862227	2.570918	0.228303	1	SRXN1	K12260
ENSG00000132603	-0.804305	5.561063	0.228398	1	NIP7	K07565
ENSG00000143816	0.831777	3.783099	0.228586	1	WNT9A	K01064
ENSG00000163874	0.802329	5.677843	0.228753	1	ZC3H12A	K18668
ENSG00000168505	-0.921817	1.947348	0.228785	1	GBX2	K09321
ENSG00000178685	-0.957762	1.752474	0.229628	1	PARP10	K15261
ENSG00000105696	0.913809	1.697648	0.229628	1	TMEM59L	_
ENSG00000052344	0.847163	3.142587	0.23031	1	PRSS8	K08664
ENSG00000169604	-0.790042	8.2054	0.230438	1	ANTXR1	_
ENSG00000175787	0.985965	1.446399	0.230584	1	ZNF169	K09228
ENSG00000068308	0.938918	1.464845	0.230584	1	OTUD5	K12655
ENSG00000123444	0.827652	3.754122	0.230794	1	KBTBD4	K10472
ENSG00000079215	-0.793203	6.091789	0.231412	1	SLC1A3	K05614
ENSG00000158079	-0.82754	3.941821	0.231434	1	PTPDC1	K18078
ENSG00000163491	-0.925422	2.078806	0.23149	1	NEK10	K08857
ENSG00000131196	0.975219	1.187832	0.23162	1	NFATC1	K04446
ENSG00000003137	-0.816145	4.350408	0.231947	1	CYP26B1	K12664
ENSG00000174606	-0.812616	4.407131	0.232132	1	ANGEL2	K18729
ENSG00000005801	-0.801679	5.100387	0.232207	1	ZNF195	K09228
ENSG00000260498	0.867426	2.614305	0.232421	1	RP4-536B24.4	_
ENSG00000244567	-1.03244	0.966156	0.232558	1	AC096772.6	_
ENSG00000100647	0.821659	3.776827	0.232642	1	KIAA0247	_
ENSG00000201302	-1.172612	0.547265	0.232646	1	SNORA65	_
ENSG00000143387	1.030056	0.439621	0.232646	1	CTSK	K01371
ENSG00000165269	1.030056	0.439621	0.232646	1	AQP7	K08771
ENSG00000100078	-0.958115	1.902207	0.232659	1	PLA2G3	K01047
ENSG00000174628	-0.958115	1.902207	0.232659	1	IQCK	_
ENSG00000136149	0.825291	3.368113	0.23281	1	RPL13AP25	K02872
ENSG00000102743	-0.822119	3.838507	0.233317	1	SLC25A15	K15101
ENSG00000100445	-0.866353	2.552517	0.233372	1	SDR39U1	K07071
ENSG00000259527	0.827078	3.102712	0.233806	1	LINC00052	_
ENSG00000233822	0.861236	2.374032	0.234472	1	HIST1H2BN	K11252
ENSG00000169519	-0.796089	5.22598	0.234563	1	METTL15	_
ENSG00000205560	-0.923706	2.0146	0.23517	1	CPT1B	K19523
ENSG00000213889	-0.923706	2.0146	0.23517	1	PPM1N	K04461

ENSG00000223784	-0.923706	2.0146	0.23517	1	RP11-554I8.2	_
ENSG00000167103	-0.830443	3.427167	0.235176	1	PIP5KL1	K13712
ENSG00000187049	-0.816287	3.63155	0.235417	1	TMEM216	K19385
ENSG00000120162	-0.796032	4.907051	0.235961	1	MOB3B	K12581
ENSG00000170145	0.786584	5.809216	0.236022	1	SIK2	K16311
ENSG00000073921	0.779981	8.376918	0.236112	1	PICALM	_
ENSG00000126368	0.815829	3.737895	0.236157	1	NR1D1	K03728
ENSG00000185585	0.793175	4.875354	0.236229	1	OLFML2A	K12581
ENSG00000171462	0.847331	2.90552	0.237566	1	DLK2	_
ENSG00000111700	-1.019828	1.217755	0.237758	1	SLCO1B3	K05043
ENSG00000204685	-1.019828	1.217755	0.237758	1	STARD7-AS1	_
ENSG00000137261	1.428717	-0.399857	0.23879	1	KIAA0319	_
ENSG00000178773	1.428717	-0.399857	0.23879	1	CPNE7	_
ENSG00000182851	1.428717	-0.399857	0.23879	1	GPIHBP1	_
ENSG00000197701	1.428717	-0.399857	0.23879	1	ZNF595	K09228
ENSG00000224805	1.428717	-0.399857	0.23879	1	LINC00853	_
ENSG00000242396	1.428717	-0.399857	0.23879	1	RP11-67L3.5	_
ENSG00000254064	1.428717	-0.399857	0.23879	1	CTD-2530N21.4	_
ENSG00000257954	1.428717	-0.399857	0.23879	1	RP11-161H23.10	_
ENSG00000264943	1.428717	-0.399857	0.23879	1	SH3GL1P2	K11247
ENSG00000267348	1.428717	-0.399857	0.23879	1	CTB-179K24.3	_
ENSG00000267546	1.428717	-0.399857	0.23879	1	RP11-666A8.8	_
ENSG00000267940	1.428717	-0.399857	0.23879	1	RP11-290F24.6	_
ENSG00000066583	-0.793499	5.044516	0.238811	1	ISOC1	_
ENSG00000253930	-0.911871	1.640967	0.238889	1	RP11-1149O23.2	_
ENSG00000136828	0.893802	2.193493	0.239089	1	RALGPS1	_
ENSG00000165983	-0.800782	4.266443	0.239395	1	PTER	K07048
ENSG00000129354	-0.789361	4.886487	0.239614	1	AP1M2	K12393
ENSG00000088035	-0.793281	4.864159	0.239948	1	ALG6	K03848
ENSG00000232531	1.113679	0.741933	0.240055	1	AC027612.1	K11428
ENSG00000233230	1.113679	0.741933	0.240055	1	AC079807.2	_
ENSG00000065413	1.032002	0.773053	0.240055	1	ANKRD44	K15503
ENSG00000179583	1.032002	0.773053	0.240055	1	CIITA	K08060
ENSG00000184254	-0.796787	4.453674	0.240228	1	ALDH1A3	K00129
ENSG00000198910	0.780186	5.545576	0.24034	1	L1CAM	K06550
ENSG00000197746	0.771888	10.2595	0.240352	1	PSAP	K12382
ENSG00000136842	0.824843	3.087353	0.24038	1	TMOD1	K10370
ENSG00000181019	-0.776159	6.79651	0.240415	1	NQO1	K00355
ENSG00000180066	0.851453	2.296451	0.240647	1	C10orf91	_
ENSG00000021645	-0.917402	1.802436	0.241368	1	NRXN3	K07377
ENSG00000162078	-0.917402	1.802436	0.241368	1	ZG16B	_
ENSG00000237310	-0.956731	1.394972	0.241443	1	GS1-124K5.4	_
ENSG00000158457	0.927853	1.317319	0.241443	1	TSPAN33	K17346
ENSG00000246334	0.927853	1.317319	0.241443	1	PRR7-AS1	_
ENSG00000198435	-0.787026	4.885002	0.241609	1	NRARP	_
ENSG00000189007	-0.802241	3.941557	0.241667	1	ADAT2	K15441
ENSG00000267939	-1.325516	0.425862	0.241983	1	CTD-2325M2.1	_
ENSG00000272054	-1.325516	0.425862	0.241983	1	RP11-423P10.2	_
ENSG00000103723	-1.269303	0.387787	0.241983	1	AP3B2	K12397
ENSG00000126246	1.143328	0.300286	0.241983	1	IGFLR1	_
ENSG00000254338	1.143328	0.300286	0.241983	1	RP11-909N17.3	_
ENSG00000257497	1.143328	0.300286	0.241983	1	RP11-585P4.5	_
ENSG00000158528	-0.811684	3.686918	0.242563	1	PPP1R9A	K17551
ENSG00000174804	-0.811684	3.686918	0.242563	1	FZD4	K02354
ENSG00000110042	-0.786501	4.624651	0.242574	1	DTX4	K06058
ENSG00000207652	-0.862485	2.316608	0.244008	1	MIR621	_
ENSG00000231721	0.847948	2.269654	0.244008	1	LINC-PINT	_
ENSG00000234978	-0.957315	1.585004	0.244179	1	RP11-423O2.5	_

ENSG00000187607	-0.83792	2.617622	0.24426	1	ZNF286A	K09228
ENSG00000163898	-0.831134	2.872559	0.244366	1	LIPH	K19404
ENSG00000126778	-0.818044	3.083613	0.244408	1	SIX1	K15614
ENSG00000106733	-0.806067	3.58474	0.244409	1	NMRK1	K10524
ENSG00000141564	0.770668	6.21317	0.244571	1	RPTOR	K07204
ENSG00000152582	-1.025522	1.097572	0.244669	1	SPEF2	_
ENSG00000171428	-1.025522	1.097572	0.244669	1	NAT1	K00622
ENSG00000198754	0.966385	1.007955	0.244669	1	OXCT2	K01027
ENSG00000207425	0.966385	1.007955	0.244669	1	Y_RNA	_
ENSG00000256018	0.966385	1.007955	0.244669	1	HIST1H3G	K11253
ENSG00000272767	0.966385	1.007955	0.244669	1	JMJD1C-AS1	_
ENSG00000117174	-0.769854	6.11913	0.245131	1	ZNHIT6	_
ENSG00000133328	-0.869489	2.419164	0.245203	1	HRASLS2	K16817
ENSG00000169908	0.763073	10.44003	0.245677	1	TM4SF1	_
ENSG00000132541	-0.778134	5.271935	0.245729	1	HRSP12	_
ENSG00000083454	-0.803661	3.562644	0.245917	1	P2RX5	K05219
ENSG00000132906	0.772199	5.355524	0.246206	1	CASP9	K04399
ENSG00000178695	-0.785132	4.737114	0.246268	1	KCTD12	_
ENSG00000185842	-0.936441	1.738318	0.246285	1	DNAH14	K10408
ENSG00000074590	-0.792307	4.33516	0.246382	1	NUAK1	K08800
ENSG00000258498	-0.881712	2.168325	0.2464	1	DIO3OS	_
ENSG00000249306	0.856173	2.116649	0.2464	1	RP11-267A15.1	_
ENSG00000116791	-0.770635	5.915522	0.246699	1	CRYZ	K00344
ENSG00000088808	0.815234	2.803328	0.246822	1	PPP1R13B	K17554
ENSG00000198168	-0.808346	3.502174	0.247032	1	SVIP	K14014
ENSG00000115875	-0.76279	7.691686	0.247196	1	SRSF7	K12896
ENSG00000119950	0.764942	6.475483	0.247376	1	MXI1	K09114
ENSG00000138771	-0.774502	5.282078	0.247416	1	SHROOM3	_
ENSG00000174705	0.765947	6.120688	0.24754	1	SH3PXD2B	_
ENSG00000162757	-0.873337	2.272075	0.247574	1	C1orf74	_
ENSG00000184441	0.923599	1.264704	0.247778	1	AP001062.7	_
ENSG00000196391	-0.919727	1.876745	0.247954	1	ZNF774	_
ENSG00000173548	0.76902	5.722676	0.2481	1	SNX33	K17923
ENSG00000102897	-0.786597	4.082351	0.248239	1	LYRM1	_
ENSG00000135069	-0.760227	7.921062	0.248544	1	PSAT1	K00831
ENSG00000120913	0.767856	5.397889	0.248564	1	PDLIM2	_
ENSG00000158483	-0.866116	2.368811	0.248583	1	FAM86C1	_
ENSG00000158246	0.77976	4.449598	0.248628	1	FAM46B	_
ENSG00000183401	-1.144564	0.721298	0.248713	1	CCDC159	_
ENSG00000221500	-1.144564	0.721298	0.248713	1	SNORD100	_
ENSG00000250303	-1.144564	0.721298	0.248713	1	RP11-356J5.12	_
ENSG00000196405	0.762641	6.233178	0.249122	1	EVL	_
ENSG00000155008	-0.872631	2.467796	0.249459	1	APOOL	_
ENSG00000112096	0.75756	8.515058	0.249627	1	SOD2	K04564
ENSG00000229419	0.935483	1.417278	0.249994	1	RALGAPA1P	_
ENSG00000221867	0.790176	3.447361	0.250333	1	MAGEA3	_
ENSG00000139324	-0.770211	5.143018	0.251188	1	TMTC3	_
ENSG00000132000	-1.622248	-0.434073	0.251568	1	PODNL1	_
ENSG00000151623	-1.622248	-0.434073	0.251568	1	NR3C2	K08555
ENSG00000214198	-1.622248	-0.434073	0.251568	1	RP11-642P15.1	_
ENSG00000230042	-1.622248	-0.434073	0.251568	1	AK3P3	K00944
ENSG00000237651	-1.622248	-0.434073	0.251568	1	C2orf74	_
ENSG00000248698	-1.622248	-0.434073	0.251568	1	LINC01085	_
ENSG00000257433	-1.622248	-0.434073	0.251568	1	RP1-197B17.3	_
ENSG00000272825	-1.622248	-0.434073	0.251568	1	LL21NC02-1C16.2	_
ENSG00000108602	-1.508305	-0.507097	0.251568	1	ALDH3A1	K00129
ENSG00000153902	-1.508305	-0.507097	0.251568	1	LGI4	_
ENSG00000196427	-1.508305	-0.507097	0.251568	1	NBPF4	_

ENSG00000197061	-1.508305	-0.507097	0.251568	1	HIST1H4C	K11253
ENSG00000214184	-1.508305	-0.507097	0.251568	1	AC012487.2	_
ENSG00000235475	-1.508305	-0.507097	0.251568	1	RP11-166O4.5	_
ENSG00000238363	-1.508305	-0.507097	0.251568	1	SNORA13	_
ENSG00000250731	-1.508305	-0.507097	0.251568	1	TPM3P6	K09290
ENSG00000259343	-1.508305	-0.507097	0.251568	1	RP11-761I4.3	_
ENSG00000270019	-1.508305	-0.507097	0.251568	1	RP11-141B14.1	_
ENSG00000270587	-1.508305	-0.507097	0.251568	1	RP11-51F16.9	_
ENSG00000179364	0.90636	1.615483	0.251644	1	PACS2	_
ENSG00000143498	-0.803227	3.454955	0.252051	1	TAF1A	K15212
ENSG00000063169	0.775418	4.287905	0.25224	1	GLTSCR1	K12581
ENSG00000186994	1.032685	0.915494	0.252505	1	KANK3	K09654
ENSG00000232833	1.032685	0.915494	0.252505	1	FAM27E3	_
ENSG00000156804	0.800451	3.206764	0.252625	1	FBXO32	K10305
ENSG00000167074	0.800451	3.206764	0.252625	1	TEF	K09058
ENSG00000100319	0.77392	4.045813	0.252979	1	ZMAT5	K13152
ENSG00000181894	0.820511	2.952266	0.253383	1	ZNF329	_
ENSG00000264112	-0.784011	3.706807	0.253613	1	RP11-159D12.2	_
ENSG00000151208	0.762983	4.983701	0.253907	1	DLG5	_
ENSG00000203705	-0.774621	4.417161	0.253915	1	TATDN3	K03424
ENSG00000148842	0.76316	4.625722	0.254213	1	CNNM2	K16302
ENSG00000170522	-0.774256	4.504518	0.254392	1	ELOVL6	K10203
ENSG00000198130	-0.774256	4.504518	0.254392	1	HIBCH	K05605
ENSG00000198830	-0.75347	6.726919	0.254439	1	HMGN2	K11300
ENSG00000103381	-0.782624	3.569729	0.254528	1	CPPED1	_
ENSG00000121671	0.767394	4.535635	0.25461	1	CRY2	K02295
ENSG00000245958	-0.817832	2.829657	0.25521	1	RP11-33B1.1	_
ENSG00000168118	0.770514	4.260669	0.255242	1	RAB4A	K07879
ENSG00000130270	0.775344	4.053764	0.255474	1	ATP8B3	K01530
ENSG00000138459	-0.768134	4.563002	0.255552	1	SLC35A5	K15274
ENSG00000146072	-0.761331	5.130578	0.255806	1	TNFRSF21	K05157
ENSG00000244627	0.81885	2.252547	0.255952	1	RP3-449O17.1	K18079
ENSG00000132109	-0.777455	4.213022	0.256067	1	TRIM21	K10651
ENSG00000119986	0.746688	7.746257	0.256954	1	AVPI1	_
ENSG00000185100	0.773506	3.764564	0.257066	1	ADSSL1	K01939
ENSG00000175279	-0.834027	2.573888	0.25718	1	APITD1	K11511
ENSG00000126838	0.761176	4.79375	0.257269	1	PZP	K03910
ENSG00000142188	0.855113	1.545813	0.257504	1	TMEM50B	_
ENSG00000171282	0.855113	1.545813	0.257504	1	RP11-1055B8.7	_
ENSG00000186847	0.855113	1.545813	0.257504	1	KRT14	K07604
ENSG00000239779	-0.827098	2.727544	0.257756	1	WBP1	_
ENSG00000183722	-0.875684	1.774987	0.258293	1	LHFP	_
ENSG00000167615	0.875506	1.712928	0.258293	1	LENG8	_
ENSG00000196177	-0.777678	3.844102	0.258701	1	ACADSB	K09478
ENSG00000232336	-1.051957	0.659466	0.25882	1	RP11-782C8.3	_
ENSG00000258325	-1.051957	0.659466	0.25882	1	RP4-816N1.6	_
ENSG00000169282	1.030639	0.53019	0.25882	1	KCNAB1	K04882
ENSG00000255320	1.030639	0.53019	0.25882	1	RP11-755F10.1	_
ENSG00000272356	1.030639	0.53019	0.25882	1	RP5-1112D6.8	_
ENSG00000136371	-0.792008	3.12387	0.258872	1	MTHFS	K01934
ENSG00000006638	0.767567	3.923722	0.258921	1	TBXA2R	K04264
ENSG00000165072	0.773799	3.958006	0.258988	1	MAMDC2	_
ENSG00000155893	-0.796734	3.285498	0.259232	1	ACPL2	_
ENSG00000169155	0.753585	4.679973	0.25932	1	ZBTB43	K10514
ENSG00000106991	0.83956	1.991401	0.259338	1	ENG	K06526
ENSG00000185112	-0.776048	3.683043	0.259931	1	FAM43A	_
ENSG00000151025	-0.852332	2.35981	0.26021	1	GPR158	K08469
ENSG00000165475	-0.846906	2.451001	0.2604	1	CRYL1	K13247

ENSG00000116701	-0.76287	4.32844	0.260667	1	NCF2	K08010
ENSG00000120708	0.739994	8.170395	0.260867	1	TGFBI	K19519
ENSG00000205885	0.793807	2.889238	0.261059	1	C1RL-AS1	_
ENSG00000137460	-0.799076	2.981391	0.261123	1	FHDC1	K10408
ENSG00000185261	-0.994138	0.940705	0.261464	1	KIAA0825	_
ENSG00000105793	-0.758099	4.782199	0.261764	1	GTPBP10	_
ENSG00000238266	-0.749022	5.48406	0.261801	1	LINC00707	_
ENSG00000106443	-0.750983	5.171166	0.262375	1	PHF14	_
ENSG00000197555	0.757783	4.65048	0.262585	1	SIPA1L1	K17701
ENSG00000242687	0.908626	1.094705	0.262816	1	AC004893.11	_
ENSG00000180592	0.766856	3.50692	0.263301	1	SKIDA1	_
ENSG00000139915	-0.928808	1.376495	0.263542	1	MDGA2	_
ENSG00000106868	-0.908544	1.552735	0.263939	1	SUSD1	_
ENSG00000139187	-0.908544	1.552735	0.263939	1	KLRG1	K10076
ENSG00000093217	0.893358	1.483016	0.263939	1	XYLB	K00854
ENSG00000226396	0.893358	1.483016	0.263939	1	RPS14P3	K02955
ENSG00000149474	-0.809898	2.753374	0.26394	1	CSRP2BP	K18188
ENSG00000240356	-0.817513	2.520755	0.264114	1	RPL23AP7	K02893
ENSG00000166801	-0.900143	1.863826	0.264252	1	FAM111A	_
ENSG00000078098	-0.880289	1.850776	0.264252	1	FAP	K08674
ENSG00000103226	0.848228	2.055353	0.264288	1	NOMO3	_
ENSG00000248367	0.848228	2.055353	0.264288	1	CTB-129O4.1	_
ENSG00000106078	0.738285	6.08995	0.264608	1	COBL	K18623
ENSG00000168610	0.734721	7.650889	0.264672	1	STAT3	K04692
ENSG00000139428	-0.745893	4.954213	0.26519	1	MMAB	K00798
ENSG00000100226	0.738578	6.153144	0.265384	1	GTPBP1	_
ENSG00000163995	0.763096	3.758218	0.266189	1	ABLIM2	K07520
ENSG00000170955	-0.74293	4.975207	0.266499	1	PRKCDBP	_
ENSG00000183615	0.760126	3.639854	0.266521	1	FAM167B	_
ENSG00000147166	-2.193622	-0.77266	0.266667	1	ITGB1BP2	K16735
ENSG00000148346	-2.193622	-0.77266	0.266667	1	LCN2	_
ENSG00000169429	-2.193622	-0.77266	0.266667	1	IL8	K10030
ENSG00000183826	-2.193622	-0.77266	0.266667	1	BTBD9	K10481
ENSG00000196684	-2.193622	-0.77266	0.266667	1	HSH2D	_
ENSG00000204778	-2.193622	-0.77266	0.266667	1	RP11-15J10.1	_
ENSG00000225721	-2.193622	-0.77266	0.266667	1	RP11-269F19.2	_
ENSG00000234779	-2.193622	-0.77266	0.266667	1	RP11-62F24.2	_
ENSG00000240288	-2.193622	-0.77266	0.266667	1	GHRLOS	_
ENSG00000248783	-2.193622	-0.77266	0.266667	1	RP11-308B16.2	_
ENSG00000253893	-2.193622	-0.77266	0.266667	1	FAM85B	_
ENSG00000266777	-2.193622	-0.77266	0.266667	1	SH3GL1P1	K11247
ENSG00000271933	-2.193622	-0.77266	0.266667	1	RP11-338I21.1	_
ENSG00000102195	1.720581	-0.998959	0.266667	1	GPR50	K04287
ENSG00000120156	1.720581	-0.998959	0.266667	1	TEK	K05121
ENSG00000131730	1.720581	-0.998959	0.266667	1	CKMT2	K00933
ENSG00000132837	1.720581	-0.998959	0.266667	1	DMGDH	K00315
ENSG00000157703	1.720581	-0.998959	0.266667	1	SVOPL	_
ENSG00000160282	1.720581	-0.998959	0.266667	1	FTCD	K13990
ENSG00000173175	1.720581	-0.998959	0.266667	1	ADCY5	K08045
ENSG00000173868	1.720581	-0.998959	0.266667	1	PHOSPHO1	K06124
ENSG00000179774	1.720581	-0.998959	0.266667	1	ATOH7	K09083
ENSG00000188886	1.720581	-0.998959	0.266667	1	ASTL	K08778
ENSG00000206561	1.720581	-0.998959	0.266667	1	COLQ	_
ENSG00000213413	1.720581	-0.998959	0.266667	1	PVRIG	_
ENSG00000226413	1.720581	-0.998959	0.266667	1	OR8T1P	K04257
ENSG00000226864	1.720581	-0.998959	0.266667	1	ATE1-AS1	_
ENSG00000228288	1.720581	-0.998959	0.266667	1	PCAT6	_
ENSG00000230424	1.720581	-0.998959	0.266667	1	RP1-43E13.2	_

ENSG00000233903	1.720581	-0.998959	0.266667	1	Z83851.4	_
ENSG00000235036	1.720581	-0.998959	0.266667	1	RP5-1099D15.1	K09529
ENSG00000236739	1.720581	-0.998959	0.266667	1	RP11-370B11.1	K05024
ENSG00000236986	1.720581	-0.998959	0.266667	1	RP11-544A12.4	_
ENSG00000237984	1.720581	-0.998959	0.266667	1	PTENP1	K01110
ENSG00000243176	1.720581	-0.998959	0.266667	1	RP11-550I24.2	_
ENSG00000251070	1.720581	-0.998959	0.266667	1	BMS1P6	K14569
ENSG00000256655	1.720581	-0.998959	0.266667	1	TMEM75	_
ENSG00000257573	1.720581	-0.998959	0.266667	1	RP11-496I2.5	K11518
ENSG00000260646	1.720581	-0.998959	0.266667	1	LA16c-385E7.1	_
ENSG00000261098	1.720581	-0.998959	0.266667	1	RP11-819C21.1	_
ENSG00000263884	1.720581	-0.998959	0.266667	1	RP11-705O1.8	_
ENSG00000264769	1.720581	-0.998959	0.266667	1	RP11-498C9.12	_
ENSG00000267264	1.720581	-0.998959	0.266667	1	CTC-459F4.6	K04614
ENSG00000267355	1.720581	-0.998959	0.266667	1	RPL9P29	K02940
ENSG00000160584	0.747032	4.671397	0.266697	1	SIK3	K19009
ENSG00000167535	0.747032	4.671397	0.266697	1	CACNB3	K04864
ENSG00000163637	-1.265914	0.116968	0.26675	1	PRICKLE2	K04511
ENSG00000186056	-1.265914	0.116968	0.26675	1	MATN1-AS1	_
ENSG00000218358	-1.265914	0.116968	0.26675	1	RAET1K	K07986
ENSG00000260023	-1.265914	0.116968	0.26675	1	RP11-49C24.1	_
ENSG00000101605	1.175928	-0.044789	0.26675	1	MYOM1	_
ENSG00000116983	1.175928	-0.044789	0.26675	1	HPCAL4	_
ENSG00000175946	1.175928	-0.044789	0.26675	1	KLHL38	K13959
ENSG00000227827	1.175928	-0.044789	0.26675	1	RP11-958N24.2	K04985
ENSG00000246451	1.175928	-0.044789	0.26675	1	RP11-894P9.1	_
ENSG00000254682	1.175928	-0.044789	0.26675	1	RP11-660L16.2	_
ENSG00000172081	0.750623	4.613257	0.266898	1	MOB3A	K17985
ENSG00000110080	0.733292	6.415819	0.267291	1	ST3GAL4	K03494
ENSG00000103534	0.738615	5.196667	0.267548	1	TMC5	K12581
ENSG00000248098	0.777466	3.230811	0.267908	1	BCKDHA	K00166
ENSG00000169019	-0.757739	4.337086	0.268	1	COMMD8	_
ENSG00000152484	0.730921	6.382973	0.268441	1	USP12	K11842
ENSG00000065833	-0.742002	5.097726	0.269244	1	ME1	K00029
ENSG00000080845	0.73499	5.35232	0.269578	1	DLGAP4	_
ENSG00000104320	-0.731084	6.435315	0.26996	1	NBN	K10867
ENSG00000072682	0.728016	6.569642	0.270349	1	P4HA2	K00472
ENSG00000203883	0.744188	4.580572	0.270546	1	SOX18	K09270
ENSG00000102362	-1.120464	0.51244	0.270771	1	SYTL4	K17598
ENSG00000186710	-1.120464	0.51244	0.270771	1	CCDC42B	K14808
ENSG00000222365	-1.120464	0.51244	0.270771	1	SNORD12B	_
ENSG00000228265	-1.120464	0.51244	0.270771	1	RP5-1125A11.1	_
ENSG00000249572	-1.120464	0.51244	0.270771	1	CTD-2203K17.1	_
ENSG00000259456	-1.120464	0.51244	0.270771	1	RP5-914P20.5	_
ENSG00000261556	-0.931127	1.476015	0.271034	1	RP11-296I10.6	K08873
ENSG00000170776	0.728725	6.313853	0.271201	1	AKAP13	K16529
ENSG00000119514	0.919006	1.210162	0.271493	1	GALNT12	K00710
ENSG00000121895	-0.758535	3.89772	0.271569	1	TMEM156	K09228
ENSG00000205084	-0.781335	3.372405	0.271592	1	TMEM231	K19362
ENSG00000165390	-1.041024	0.821171	0.2718	1	ANXA8	K17096
ENSG00000209482	-1.041024	0.821171	0.2718	1	SNORD83A	_
ENSG00000222009	-1.041024	0.821171	0.2718	1	BTBD19	K17985
ENSG00000260196	-1.041024	0.821171	0.2718	1	RP1-239B22.5	_
ENSG00000231890	1.031601	0.696395	0.2718	1	AC093391.2	_
ENSG00000166793	0.949924	0.728498	0.2718	1	YPEL4	_
ENSG00000260774	0.949924	0.728498	0.2718	1	CTD-2083E4.4	_
ENSG00000135596	-0.990897	1.074514	0.271828	1	MICAL1	_
ENSG00000260177	0.780995	2.913049	0.271945	1	RP4-536B24.3	_

ENSG00000133313	-0.731905	5.620047	0.272238	1	CNDP2	K08660
ENSG00000047346	0.737512	4.258633	0.272703	1	FAM214A	_
ENSG00000260260	0.754884	3.900504	0.272853	1	RP11-304L19.5	_
ENSG00000257704	0.751034	3.423677	0.273073	1	PRR24	_
ENSG00000117472	0.756303	3.326232	0.273801	1	TSPAN1	K17348
ENSG00000264281	0.785976	2.623951	0.273923	1	CTD-2031P19.4	K02928
ENSG00000067992	0.77174	2.953475	0.274374	1	PDK3	K00898
ENSG00000173166	0.834923	1.958357	0.274487	1	RAPH1	_
ENSG00000166822	-0.754413	3.70642	0.274563	1	TMEM170A	K04613
ENSG00000178202	-0.737388	4.812005	0.274717	1	KDELC2	K12581
ENSG00000196937	-0.728142	5.394398	0.275054	1	FAM3C	_
ENSG00000173588	-0.748069	4.127762	0.275079	1	CCDC41	K16754
ENSG00000145287	-0.72027	6.99342	0.275248	1	PLAC8	_
ENSG00000117394	0.720797	6.445368	0.275597	1	SLC2A1	K07299
ENSG00000120616	-0.740254	4.406671	0.275813	1	EPC1	K11322
ENSG00000242265	-0.723554	6.124251	0.275994	1	PEG10	_
ENSG00000256667	-0.854363	1.761041	0.276398	1	KLRAP1	_
ENSG00000137628	-0.72054	6.596252	0.276504	1	DDX60	_
ENSG00000149243	-0.780529	3.030118	0.27669	1	KLHL35	K10461
ENSG00000197119	0.717864	6.411645	0.277211	1	SLC25A29	K15109
ENSG00000178904	-0.736555	4.459289	0.277297	1	DPY19L3	K12581
ENSG00000205220	-0.755861	3.182268	0.277647	1	PSMB10	K02733
ENSG00000196914	0.716599	6.708605	0.277839	1	ARHGEF12	K07532
ENSG00000067082	-0.717878	6.560119	0.27804	1	KLF6	K09207
ENSG00000132570	-0.810015	2.558338	0.278422	1	PCBD2	K01724
ENSG00000130382	0.719513	5.379004	0.278782	1	MLLT1	K15187
ENSG00000102738	-0.728403	4.797894	0.278785	1	MRPS31	K17410
ENSG00000196668	0.877524	1.337583	0.278896	1	LINC00173	_
ENSG00000111145	-0.728707	4.568337	0.278962	1	ELK3	K09430
ENSG00000255112	0.714705	6.721581	0.279076	1	CHMP1B	K08834
ENSG00000144749	-0.812647	2.473877	0.279364	1	LRIG1	_
ENSG00000169193	-0.799727	2.465534	0.279364	1	CCDC126	_
ENSG00000145348	-0.772503	2.765361	0.279536	1	TBCK	K17544
ENSG00000186687	-0.727615	4.420954	0.279778	1	LYRM7	K18170
ENSG00000146263	-0.731251	4.308486	0.280123	1	MMS22L	_
ENSG00000164695	-0.729095	4.683435	0.280162	1	CHMP4C	K12194
ENSG00000185186	0.830065	1.924558	0.280167	1	LINC00313	_
ENSG00000139438	0.914031	1.153549	0.280417	1	FAM222A	_
ENSG00000104549	-0.715202	6.400598	0.280496	1	SQLE	K00511
ENSG00000152137	0.733082	3.955617	0.280714	1	HSPB8	K08879
ENSG00000164463	0.745876	3.272922	0.280845	1	CREBRF	_
ENSG00000151012	-0.728946	4.410717	0.280919	1	SLC7A11	K13869
ENSG00000035681	-0.720597	5.338224	0.281021	1	NSMAF	K18953
ENSG00000077044	0.710173	7.372743	0.281122	1	DGKD	K00901
ENSG00000107957	0.709809	7.533837	0.281184	1	SH3PXD2A	_
ENSG00000242221	0.746169	3.398993	0.281301	1	PSG2	K06500
ENSG00000127081	0.814612	2.224949	0.281607	1	ZNF484	K09228
ENSG00000105663	0.958994	0.874741	0.282111	1	KMT2B	_
ENSG00000053918	0.889177	0.902853	0.282111	1	KCNQ1	K04926
ENSG00000175879	0.889177	0.902853	0.282111	1	HOXD8	K09308
ENSG00000220804	0.889177	0.902853	0.282111	1	AC093642.5	K16941
ENSG00000255455	0.889177	0.902853	0.282111	1	RP11-890B15.3	_
ENSG00000008226	1.951624	-1.267408	0.282609	1	DLEC1	K15696
ENSG00000057593	1.951624	-1.267408	0.282609	1	F7	K01320
ENSG00000075886	1.951624	-1.267408	0.282609	1	TUBA3D	K07374
ENSG00000091844	1.951624	-1.267408	0.282609	1	RGS17	K16449
ENSG00000104888	1.951624	-1.267408	0.282609	1	SLC17A7	K12302
ENSG00000109061	1.951624	-1.267408	0.282609	1	MYH1	K10352

ENSG00000115718	1.951624	-1.267408	0.282609	1	PROC	K01344
ENSG00000127588	1.951624	-1.267408	0.282609	1	GNG13	K04547
ENSG00000143199	1.951624	-1.267408	0.282609	1	ADCY10	K11265
ENSG00000151892	1.951624	-1.267408	0.282609	1	GFRA1	_
ENSG00000167748	1.951624	-1.267408	0.282609	1	KLK1	K01325
ENSG00000171747	1.951624	-1.267408	0.282609	1	LGALS4	K10091
ENSG00000178342	1.951624	-1.267408	0.282609	1	KCNG2	K04901
ENSG00000187554	1.951624	-1.267408	0.282609	1	TLR5	K10168
ENSG00000196644	1.951624	-1.267408	0.282609	1	GPR89C	_
ENSG00000197181	1.951624	-1.267408	0.282609	1	PIWIL2	K02156
ENSG00000207146	1.951624	-1.267408	0.282609	1	Y_RNA	_
ENSG00000213609	1.951624	-1.267408	0.282609	1	RP11-170M17.2	K02936
ENSG00000214188	1.951624	-1.267408	0.282609	1	ST7-OT4	_
ENSG00000215105	1.951624	-1.267408	0.282609	1	TTC3P1	K15712
ENSG00000222898	1.951624	-1.267408	0.282609	1	RN7SKP97	_
ENSG00000224688	1.951624	-1.267408	0.282609	1	KB-1592A4.13	K09390
ENSG00000228251	1.951624	-1.267408	0.282609	1	AC012442.6	_
ENSG00000230076	1.951624	-1.267408	0.282609	1	AC016708.2	K02866
ENSG00000232004	1.951624	-1.267408	0.282609	1	CAP1P2	K17261
ENSG00000232355	1.951624	-1.267408	0.282609	1	RP11-263F14.3	K10104
ENSG00000232987	1.951624	-1.267408	0.282609	1	AC051649.6	_
ENSG00000233087	1.951624	-1.267408	0.282609	1	AC073869.1	K07895
ENSG00000237082	1.951624	-1.267408	0.282609	1	COX5BP6	K02265
ENSG00000237505	1.951624	-1.267408	0.282609	1	RP11-76N22.2	_
ENSG00000238133	1.951624	-1.267408	0.282609	1	MLK7-AS1	_
ENSG00000241782	1.951624	-1.267408	0.282609	1	RP11-91P24.1	K02889
ENSG00000248240	1.951624	-1.267408	0.282609	1	RP11-159F24.5	_
ENSG00000255007	1.951624	-1.267408	0.282609	1	CTD-2589M5.4	_
ENSG00000259243	1.951624	-1.267408	0.282609	1	GOLGA6L19	_
ENSG00000260025	1.951624	-1.267408	0.282609	1	RP11-490M8.1	_
ENSG00000265185	1.951624	-1.267408	0.282609	1	SNORD3B-1	_
ENSG00000269028	1.951624	-1.267408	0.282609	1	MTRNR2L12	K01982
ENSG00000269235	1.951624	-1.267408	0.282609	1	HCCAT3	_
ENSG00000269619	1.951624	-1.267408	0.282609	1	CTD-3023J11.2	_
ENSG00000269915	1.951624	-1.267408	0.282609	1	AP006621.9	_
ENSG00000269956	1.951624	-1.267408	0.282609	1	MKNK1-AS1	_
ENSG00000269997	1.951624	-1.267408	0.282609	1	RP11-214K3.21	_
ENSG00000270419	1.951624	-1.267408	0.282609	1	CAHM	_
ENSG00000272072	1.951624	-1.267408	0.282609	1	CTA-363E19.2	_
ENSG00000272953	1.951624	-1.267408	0.282609	1	RP11-1275H24.2	_
ENSG00000273394	1.951624	-1.267408	0.282609	1	RP11-255E6.6	_
ENSG00000118960	0.726484	4.106227	0.282672	1	HS1BP3	K04678
ENSG00000230061	0.795887	2.109087	0.282957	1	AP001065.2	_
ENSG00000099875	0.706982	7.634239	0.282983	1	MKNK2	K04372
ENSG00000182325	-0.74945	3.280128	0.283342	1	FBXL6	K10272
ENSG00000211448	0.708129	6.795507	0.283352	1	DIO2	K17904
ENSG00000151967	-1.438997	-0.103742	0.283653	1	SCHIP1	_
ENSG00000224950	-1.438997	-0.103742	0.283653	1	RP5-1086K13.1	_
ENSG00000227992	-1.438997	-0.103742	0.283653	1	AC108463.2	_
ENSG00000237862	-1.438997	-0.103742	0.283653	1	RP1-63G5.7	_
ENSG00000244274	-1.438997	-0.103742	0.283653	1	DBNDD2	_
ENSG00000260643	-1.438997	-0.103742	0.283653	1	RP11-303E16.8	_
ENSG00000261052	-1.438997	-0.103742	0.283653	1	SULT1A3	K01014
ENSG00000272091	-1.438997	-0.103742	0.283653	1	RP4-758J24.5	_
ENSG00000273271	-1.438997	-0.103742	0.283653	1	AP000254.8	_
ENSG00000080573	1.380255	-0.243739	0.283653	1	COL5A3	K19721
ENSG00000143226	1.380255	-0.243739	0.283653	1	FCGR2A	K06472
ENSG00000154025	1.380255	-0.243739	0.283653	1	SLC5A10	K14390

ENSG00000160460	1.380255	-0.243739	0.283653	1	SPTBN4	K06115
ENSG00000169246	1.380255	-0.243739	0.283653	1	NPIPB3	_
ENSG00000181790	1.380255	-0.243739	0.283653	1	BAI1	K04596
ENSG00000226200	1.380255	-0.243739	0.283653	1	RP11-50E11.3	_
ENSG00000228120	1.380255	-0.243739	0.283653	1	AP001631.10	_
ENSG00000233101	1.380255	-0.243739	0.283653	1	HOXB-AS3	_
ENSG00000254632	1.380255	-0.243739	0.283653	1	RP11-21L23.4	_
ENSG00000259349	1.380255	-0.243739	0.283653	1	RP11-15E18.1	_
ENSG00000261118	1.380255	-0.243739	0.283653	1	RP11-104N10.1	_
ENSG00000269125	1.380255	-0.243739	0.283653	1	RP11-98F14.11	_
ENSG00000164776	1.191847	-0.180035	0.283653	1	PHKG1	K00871
ENSG00000215790	1.191847	-0.180035	0.283653	1	SLC35E2	K15284
ENSG00000231203	1.191847	-0.180035	0.283653	1	KRT8P10	K07605
ENSG00000238268	1.191847	-0.180035	0.283653	1	RP11-229P13.19	_
ENSG00000240758	1.191847	-0.180035	0.283653	1	RP11-155G14.6	_
ENSG00000259448	1.191847	-0.180035	0.283653	1	RP11-16E12.1	_
ENSG00000260160	1.191847	-0.180035	0.283653	1	CTC-471J1.2	_
ENSG00000266983	1.191847	-0.180035	0.283653	1	CTC-232P5.1	_
ENSG00000180914	-1.099004	0.690753	0.283853	1	OXTR	K04229
ENSG00000054965	0.714551	5.2851	0.28388	1	FAM168A	_
ENSG00000134243	0.713132	5.767737	0.284008	1	SORT1	K12388
ENSG00000214013	-0.748755	3.229622	0.284121	1	GANC	_
ENSG00000157557	0.703683	8.552279	0.284531	1	ETS2	K02678
ENSG00000175931	0.713325	5.37795	0.284618	1	UBE2O	K10581
ENSG00000185024	0.720093	4.636582	0.285047	1	BRF1	K15196
ENSG00000197467	-0.824364	2.341621	0.285106	1	COL13A1	K16617
ENSG00000117360	-1.210811	0.348557	0.285115	1	PRPF3	K12843
ENSG00000214050	-1.210811	0.348557	0.285115	1	FBXO16	K10299
ENSG00000236778	-1.210811	0.348557	0.285115	1	INTS6-AS1	_
ENSG00000272752	-1.210811	0.348557	0.285115	1	STAG3L5P-PVRIG2	_
ENSG00000183971	1.028602	0.24023	0.285115	1	NPW	_
ENSG00000235720	1.028602	0.24023	0.285115	1	RP11-340I6.6	K09441
ENSG00000236782	1.028602	0.24023	0.285115	1	RP11-96L14.7	_
ENSG00000244306	-0.883527	1.536294	0.285285	1	CTD-2314B22.3	_
ENSG00000203709	0.842877	1.454637	0.285285	1	C1orf132	_
ENSG00000229097	0.842877	1.454637	0.285285	1	CALM2P2	K02183
ENSG00000116016	0.702855	7.868571	0.285559	1	EPAS1	K09095
ENSG00000005187	4.668855	-2.01648	0.285714	1	ACSM3	K01896
ENSG00000031081	4.668855	-2.01648	0.285714	1	ARHGAP31	_
ENSG00000070031	4.668855	-2.01648	0.285714	1	SCT	K05263
ENSG00000071242	4.668855	-2.01648	0.285714	1	RPS6KA2	K04373
ENSG00000091879	4.668855	-2.01648	0.285714	1	ANGPT2	K05466
ENSG00000099985	4.668855	-2.01648	0.285714	1	OSM	K05418
ENSG00000100146	4.668855	-2.01648	0.285714	1	SOX10	K09270
ENSG00000104413	4.668855	-2.01648	0.285714	1	ESRP1	K14947
ENSG00000104432	4.668855	-2.01648	0.285714	1	IL7	K05431
ENSG00000104808	4.668855	-2.01648	0.285714	1	DHDH	K00078
ENSG00000108947	4.668855	-2.01648	0.285714	1	EFNB3	K05463
ENSG00000111186	4.668855	-2.01648	0.285714	1	WNT5B	K00444
ENSG00000115590	4.668855	-2.01648	0.285714	1	IL1R2	K04387
ENSG00000115956	4.668855	-2.01648	0.285714	1	PLEK	_
ENSG00000116783	4.668855	-2.01648	0.285714	1	TNNI3K	K17535
ENSG00000120088	4.668855	-2.01648	0.285714	1	CRHR1	K04578
ENSG00000121594	4.668855	-2.01648	0.285714	1	CD80	K05412
ENSG00000122420	4.668855	-2.01648	0.285714	1	PTGFR	K04262
ENSG00000124103	4.668855	-2.01648	0.285714	1	FAM209A	_
ENSG00000124212	4.668855	-2.01648	0.285714	1	PTGIS	K01831
ENSG00000124749	4.668855	-2.01648	0.285714	1	COL21A1	K16629

ENSG00000125631	4.668855	-2.01648	0.285714	1	HTR5BP	K04161
ENSG00000125872	4.668855	-2.01648	0.285714	1	LRRN4	_
ENSG00000125965	4.668855	-2.01648	0.285714	1	GDF5	K04664
ENSG00000128606	4.668855	-2.01648	0.285714	1	LRRC17	K10279
ENSG00000129455	4.668855	-2.01648	0.285714	1	KLK8	K08650
ENSG00000129596	4.668855	-2.01648	0.285714	1	CDO1	K00456
ENSG00000131203	4.668855	-2.01648	0.285714	1	IDO1	K00463
ENSG00000132693	4.668855	-2.01648	0.285714	1	CRP	K16143
ENSG00000133665	4.668855	-2.01648	0.285714	1	DYDC2	_
ENSG00000134007	4.668855	-2.01648	0.285714	1	ADAM20	K08609
ENSG00000135960	4.668855	-2.01648	0.285714	1	EDAR	K05162
ENSG00000137078	4.668855	-2.01648	0.285714	1	SIT1	_
ENSG00000137225	4.668855	-2.01648	0.285714	1	CAPN11	K08580
ENSG00000137642	4.668855	-2.01648	0.285714	1	SORL1	_
ENSG00000138079	4.668855	-2.01648	0.285714	1	SLC3A1	K14210
ENSG00000139193	4.668855	-2.01648	0.285714	1	CD27	K05144
ENSG00000140931	4.668855	-2.01648	0.285714	1	CMTM3	_
ENSG00000143119	4.668855	-2.01648	0.285714	1	CD53	K06489
ENSG00000143556	4.668855	-2.01648	0.285714	1	S100A7	_
ENSG00000144369	4.668855	-2.01648	0.285714	1	FAM171B	_
ENSG00000147123	4.668855	-2.01648	0.285714	1	NDUFB11	K11351
ENSG00000152076	4.668855	-2.01648	0.285714	1	CCDC74B	_
ENSG00000153802	4.668855	-2.01648	0.285714	1	TMPRSS11D	K09641
ENSG00000154678	4.668855	-2.01648	0.285714	1	PDE1C	K13755
ENSG00000155530	4.668855	-2.01648	0.285714	1	LRGUK	_
ENSG00000155754	4.668855	-2.01648	0.285714	1	ALS2CR11	_
ENSG00000157927	4.668855	-2.01648	0.285714	1	RADIL	_
ENSG00000158428	4.668855	-2.01648	0.285714	1	C2orf62	_
ENSG00000158486	4.668855	-2.01648	0.285714	1	DNAH3	K10408
ENSG00000159871	4.668855	-2.01648	0.285714	1	LYPD5	_
ENSG00000162592	4.668855	-2.01648	0.285714	1	CCDC27	_
ENSG00000162728	4.668855	-2.01648	0.285714	1	KCNJ9	K05002
ENSG00000163431	4.668855	-2.01648	0.285714	1	LMOD1	_
ENSG00000163531	4.668855	-2.01648	0.285714	1	NFASC	K06757
ENSG00000164122	4.668855	-2.01648	0.285714	1	ASB5	K10327
ENSG00000165240	4.668855	-2.01648	0.285714	1	ATP7A	K17686
ENSG00000165409	4.668855	-2.01648	0.285714	1	TSHR	K04249
ENSG00000165617	4.668855	-2.01648	0.285714	1	DACT1	_
ENSG00000167371	4.668855	-2.01648	0.285714	1	PRRT2	_
ENSG00000168135	4.668855	-2.01648	0.285714	1	KCNJ4	K04998
ENSG00000168824	4.668855	-2.01648	0.285714	1	NSG1	_
ENSG00000170374	4.668855	-2.01648	0.285714	1	SP7	K09197
ENSG00000170615	4.668855	-2.01648	0.285714	1	SLC26A5	K14703
ENSG00000170703	4.668855	-2.01648	0.285714	1	TTLL6	K16582
ENSG00000171217	4.668855	-2.01648	0.285714	1	CLDN20	K06087
ENSG00000171860	4.668855	-2.01648	0.285714	1	C3AR1	K04009
ENSG00000172005	4.668855	-2.01648	0.285714	1	MAL	_
ENSG00000172350	4.668855	-2.01648	0.285714	1	ABCG4	K05680
ENSG00000172967	4.668855	-2.01648	0.285714	1	XKR3	_
ENSG00000173809	4.668855	-2.01648	0.285714	1	TDRD12	K18409
ENSG00000173826	4.668855	-2.01648	0.285714	1	KCNH6	K04909
ENSG00000173917	4.668855	-2.01648	0.285714	1	HOXB2	K09302
ENSG00000176358	4.668855	-2.01648	0.285714	1	TAC4	K05241
ENSG00000176761	4.668855	-2.01648	0.285714	1	ZNF285B	K09228
ENSG00000177098	4.668855	-2.01648	0.285714	1	SCN4B	K04848
ENSG00000178734	4.668855	-2.01648	0.285714	1	C13orf45	_
ENSG00000178809	4.668855	-2.01648	0.285714	1	TRIM73	K12024
ENSG00000179902	4.668855	-2.01648	0.285714	1	C1orf194	_

ENSG00000180019	4.668855	-2.01648	0.285714	1	AC079741.2	K02977
ENSG00000180043	4.668855	-2.01648	0.285714	1	FAM71E2	_
ENSG00000180539	4.668855	-2.01648	0.285714	1	C9orf139	K17985
ENSG00000182376	4.668855	-2.01648	0.285714	1	RP5-1142A6.8	_
ENSG00000182404	4.668855	-2.01648	0.285714	1	RP1-86D1.3	_
ENSG00000182450	4.668855	-2.01648	0.285714	1	KCNK4	K04915
ENSG00000183798	4.668855	-2.01648	0.285714	1	EMILIN3	_
ENSG00000186868	4.668855	-2.01648	0.285714	1	MAPT	K04380
ENSG00000187808	4.668855	-2.01648	0.285714	1	SOWAHD	_
ENSG00000188001	4.668855	-2.01648	0.285714	1	TPRG1	_
ENSG00000188573	4.668855	-2.01648	0.285714	1	FBLL1	K14563
ENSG00000188662	4.668855	-2.01648	0.285714	1	HILS1	_
ENSG00000188771	4.668855	-2.01648	0.285714	1	PLET1	_
ENSG00000188850	4.668855	-2.01648	0.285714	1	RP11-159F24.2	K02942
ENSG00000197054	4.668855	-2.01648	0.285714	1	ZNF763	K09228
ENSG00000197249	4.668855	-2.01648	0.285714	1	SERPINA1	K03984
ENSG00000197604	4.668855	-2.01648	0.285714	1	AC022532.1	K04666
ENSG00000197880	4.668855	-2.01648	0.285714	1	MDS2	K09228
ENSG00000198711	4.668855	-2.01648	0.285714	1	SSBP3-AS1	_
ENSG00000198939	4.668855	-2.01648	0.285714	1	ZFP2	K09228
ENSG00000199177	4.668855	-2.01648	0.285714	1	MIR31	_
ENSG00000199179	4.668855	-2.01648	0.285714	1	MIRLET7I	_
ENSG00000199719	4.668855	-2.01648	0.285714	1	RN7SKP74	_
ENSG00000199815	4.668855	-2.01648	0.285714	1	SNORA43	_
ENSG00000200959	4.668855	-2.01648	0.285714	1	SNORA74A	_
ENSG00000201544	4.668855	-2.01648	0.285714	1	SNORA16B	_
ENSG00000201581	4.668855	-2.01648	0.285714	1	RN7SKP78	_
ENSG00000201684	4.668855	-2.01648	0.285714	1	RN7SKP239	_
ENSG00000202417	4.668855	-2.01648	0.285714	1	Y_RNA	_
ENSG00000203433	4.668855	-2.01648	0.285714	1	AC008147.2	K09228
ENSG00000203520	4.668855	-2.01648	0.285714	1	RP11-697H9.3	_
ENSG00000204380	4.668855	-2.01648	0.285714	1	AC005042.4	_
ENSG00000205177	4.668855	-2.01648	0.285714	1	C11orf91	_
ENSG00000205274	4.668855	-2.01648	0.285714	1	TRBV20OR9-2	_
ENSG00000205863	4.668855	-2.01648	0.285714	1	C1QTNF9B	_
ENSG00000206181	4.668855	-2.01648	0.285714	1	TCEB3B	K15076
ENSG00000206532	4.668855	-2.01648	0.285714	1	RP11-553A10.1	_
ENSG00000207380	4.668855	-2.01648	0.285714	1	Y_RNA	_
ENSG00000207839	4.668855	-2.01648	0.285714	1	MIR33B	_
ENSG00000211683	4.668855	-2.01648	0.285714	1	KB-1572G7.3	_
ENSG00000213253	4.668855	-2.01648	0.285714	1	CTC-250I14.1	K02870
ENSG00000213277	4.668855	-2.01648	0.285714	1	MARCKSL1P1	K13536
ENSG00000213376	4.668855	-2.01648	0.285714	1	GAPDHP71	K00134
ENSG00000213409	4.668855	-2.01648	0.285714	1	RP11-658F2.3	K15414
ENSG00000213519	4.668855	-2.01648	0.285714	1	AC132008.1	K02920
ENSG00000214128	4.668855	-2.01648	0.285714	1	TMEM213	K12581
ENSG00000214772	4.668855	-2.01648	0.285714	1	RP11-174G6.1	_
ENSG00000215014	4.668855	-2.01648	0.285714	1	AL645728.1	K17985
ENSG00000216613	4.668855	-2.01648	0.285714	1	RP3-406A7.5	K01922
ENSG00000216636	4.668855	-2.01648	0.285714	1	RPL7P25	K02937
ENSG00000217527	4.668855	-2.01648	0.285714	1	RPS16P5	K02960
ENSG00000219642	4.668855	-2.01648	0.285714	1	BMPR1APS1	K04673
ENSG00000221387	4.668855	-2.01648	0.285714	1	RNU6ATAC8P	_
ENSG00000221971	4.668855	-2.01648	0.285714	1	TTC4P1	_
ENSG00000222177	4.668855	-2.01648	0.285714	1	RNU4-30P	_
ENSG00000222503	4.668855	-2.01648	0.285714	1	Y_RNA	_
ENSG00000223474	4.668855	-2.01648	0.285714	1	AL020996.1	_
ENSG00000223779	4.668855	-2.01648	0.285714	1	RP11-403I13.4	_

ENSG00000224072	4.668855	-2.01648	0.285714	1	RP11-75A9.3	_
ENSG00000224676	4.668855	-2.01648	0.285714	1	AP000351.8	K00799
ENSG00000224721	4.668855	-2.01648	0.285714	1	AC007182.6	_
ENSG00000225043	4.668855	-2.01648	0.285714	1	RPL18AP2	_
ENSG00000225193	4.668855	-2.01648	0.285714	1	RPS12P26	K02951
ENSG00000225505	4.668855	-2.01648	0.285714	1	RP11-330C7.3	K07059
ENSG00000225637	4.668855	-2.01648	0.285714	1	AP001046.6	_
ENSG00000225674	4.668855	-2.01648	0.285714	1	IPO7P2	K18755
ENSG00000225711	4.668855	-2.01648	0.285714	1	RP11-345I18.4	K00719
ENSG00000226145	4.668855	-2.01648	0.285714	1	AC022596.6	K07604
ENSG00000226148	4.668855	-2.01648	0.285714	1	SLC25A39P1	K15119
ENSG00000227191	4.668855	-2.01648	0.285714	1	TRGC2	_
ENSG00000227200	4.668855	-2.01648	0.285714	1	RP11-121A14.3	_
ENSG00000227307	4.668855	-2.01648	0.285714	1	RP11-95I16.2	_
ENSG00000227388	4.668855	-2.01648	0.285714	1	RP11-112J3.16	_
ENSG00000227528	4.668855	-2.01648	0.285714	1	DIAPH3-AS1	_
ENSG00000227844	4.668855	-2.01648	0.285714	1	RP13-143G15.3	_
ENSG00000228146	4.668855	-2.01648	0.285714	1	CASP16	_
ENSG00000228172	4.668855	-2.01648	0.285714	1	RP1-317E23.3	_
ENSG00000228237	4.668855	-2.01648	0.285714	1	EFCAB14-AS1	_
ENSG00000228742	4.668855	-2.01648	0.285714	1	RP5-884M6.1	_
ENSG00000228828	4.668855	-2.01648	0.285714	1	RP11-162G10.1	K08864
ENSG00000228940	4.668855	-2.01648	0.285714	1	RP11-348A7.1	K04382
ENSG00000229052	4.668855	-2.01648	0.285714	1	RP11-386I23.1	_
ENSG00000229235	4.668855	-2.01648	0.285714	1	RP11-8L18.3	K02922
ENSG00000229591	4.668855	-2.01648	0.285714	1	RP5-981O7.2	_
ENSG00000229694	4.668855	-2.01648	0.285714	1	RP11-305L7.6	_
ENSG00000230287	4.668855	-2.01648	0.285714	1	RP11-305E17.4	_
ENSG00000230295	4.668855	-2.01648	0.285714	1	RP11-458F8.2	K03121
ENSG00000230345	4.668855	-2.01648	0.285714	1	RP13-455A7.1	_
ENSG00000230534	4.668855	-2.01648	0.285714	1	RP11-297A16.2	_
ENSG00000230696	4.668855	-2.01648	0.285714	1	AC011753.5	_
ENSG00000230964	4.668855	-2.01648	0.285714	1	AC018696.4	_
ENSG00000230978	4.668855	-2.01648	0.285714	1	LINC00160	_
ENSG00000231053	4.668855	-2.01648	0.285714	1	RP13-26D14.2	_
ENSG00000231188	4.668855	-2.01648	0.285714	1	RP11-34D15.2	_
ENSG00000231324	4.668855	-2.01648	0.285714	1	AP000696.2	_
ENSG00000231414	4.668855	-2.01648	0.285714	1	AC016700.6	K02924
ENSG00000231697	4.668855	-2.01648	0.285714	1	NANOGP5	K10164
ENSG00000231704	4.668855	-2.01648	0.285714	1	AC004895.4	_
ENSG00000231865	4.668855	-2.01648	0.285714	1	SIK3-IT1	_
ENSG00000232031	4.668855	-2.01648	0.285714	1	RP5-991C6.4	_
ENSG00000232237	4.668855	-2.01648	0.285714	1	ASCL5	K09067
ENSG00000232406	4.668855	-2.01648	0.285714	1	RP11-234K24.3	_
ENSG00000232578	4.668855	-2.01648	0.285714	1	CTC-303L1.1	_
ENSG00000232762	4.668855	-2.01648	0.285714	1	RP4-784A16.4	_
ENSG00000232850	4.668855	-2.01648	0.285714	1	AL590708.2	_
ENSG00000233018	4.668855	-2.01648	0.285714	1	RP4-597N16.1	_
ENSG00000233242	4.668855	-2.01648	0.285714	1	RP11-220I1.2	_
ENSG00000233382	4.668855	-2.01648	0.285714	1	NKAPP1	_
ENSG00000233623	4.668855	-2.01648	0.285714	1	PGAM1P11	K01834
ENSG00000233668	4.668855	-2.01648	0.285714	1	RP11-571F15.3	K02995
ENSG00000233797	4.668855	-2.01648	0.285714	1	UFL1-AS1	_
ENSG00000233922	4.668855	-2.01648	0.285714	1	AL133493.2	_
ENSG00000234134	4.668855	-2.01648	0.285714	1	RP11-383C5.5	_
ENSG00000234144	4.668855	-2.01648	0.285714	1	COX6CP13	K02268
ENSG00000234473	4.668855	-2.01648	0.285714	1	ZNF101P2	K09228
ENSG00000234553	4.668855	-2.01648	0.285714	1	AC022431.3	_

ENSG00000234773	4.668855	-2.01648	0.285714	1	CTD-2666L21.1	_
ENSG00000234996	4.668855	-2.01648	0.285714	1	RP11-480I12.7	_
ENSG00000235005	4.668855	-2.01648	0.285714	1	RP11-195M16.1	_
ENSG00000235254	4.668855	-2.01648	0.285714	1	TMEM185AP1	_
ENSG00000235806	4.668855	-2.01648	0.285714	1	RP4-646N3.1	_
ENSG00000235940	4.668855	-2.01648	0.285714	1	MTND1P21	_
ENSG00000235947	4.668855	-2.01648	0.285714	1	EGOT	_
ENSG00000236022	4.668855	-2.01648	0.285714	1	RP11-160E2.16	_
ENSG00000236065	4.668855	-2.01648	0.285714	1	RP1-117O3.2	_
ENSG00000236091	4.668855	-2.01648	0.285714	1	RP3-473B4.3	_
ENSG00000236258	4.668855	-2.01648	0.285714	1	TIMM8BP1	K17780
ENSG00000236559	4.668855	-2.01648	0.285714	1	RP11-243J16.7	_
ENSG00000236760	4.668855	-2.01648	0.285714	1	AC019118.3	_
ENSG00000236935	4.668855	-2.01648	0.285714	1	AP003774.1	_
ENSG00000237243	4.668855	-2.01648	0.285714	1	AC022173.2	_
ENSG00000237815	4.668855	-2.01648	0.285714	1	AC007000.11	K08694
ENSG00000237928	4.668855	-2.01648	0.285714	1	RP4-668G5.1	_
ENSG00000238305	4.668855	-2.01648	0.285714	1	snoU13	_
ENSG00000238343	4.668855	-2.01648	0.285714	1	snoU13	_
ENSG00000238793	4.668855	-2.01648	0.285714	1	SNORD124	_
ENSG00000238987	4.668855	-2.01648	0.285714	1	RNU7-133P	_
ENSG00000239091	4.668855	-2.01648	0.285714	1	snoU13	_
ENSG00000239219	4.668855	-2.01648	0.285714	1	RP11-379K17.4	_
ENSG00000239272	4.668855	-2.01648	0.285714	1	RPL21P10	K02889
ENSG00000239553	4.668855	-2.01648	0.285714	1	RN7SL12P	_
ENSG00000239608	4.668855	-2.01648	0.285714	1	RUVBL1-AS1	_
ENSG00000240160	4.668855	-2.01648	0.285714	1	RN7SL263P	_
ENSG00000240553	4.668855	-2.01648	0.285714	1	RP1-184J9.2	_
ENSG00000241030	4.668855	-2.01648	0.285714	1	RPL29P24	K02905
ENSG00000241157	4.668855	-2.01648	0.285714	1	RP11-3K24.1	K02912
ENSG00000241890	4.668855	-2.01648	0.285714	1	RPL13P4	_
ENSG00000241954	4.668855	-2.01648	0.285714	1	RP1-149A16.17	_
ENSG00000242017	4.668855	-2.01648	0.285714	1	ALG1L15P	K03842
ENSG00000242267	4.668855	-2.01648	0.285714	1	SKINTL	_
ENSG00000243000	4.668855	-2.01648	0.285714	1	RP11-504P24.3	K16944
ENSG00000243870	4.668855	-2.01648	0.285714	1	RN7SL236P	_
ENSG00000244018	4.668855	-2.01648	0.285714	1	RPL35P6	K02918
ENSG00000244097	4.668855	-2.01648	0.285714	1	RPS4XP17	K02987
ENSG00000244268	4.668855	-2.01648	0.285714	1	RP11-529G21.2	_
ENSG00000244610	4.668855	-2.01648	0.285714	1	RN7SL756P	_
ENSG00000244675	4.668855	-2.01648	0.285714	1	AC108676.1	_
ENSG00000245059	4.668855	-2.01648	0.285714	1	RP11-303E16.7	_
ENSG00000245729	4.668855	-2.01648	0.285714	1	RP11-480D4.1	_
ENSG00000246250	4.668855	-2.01648	0.285714	1	RP11-613D13.5	_
ENSG00000247624	4.668855	-2.01648	0.285714	1	CPEB2-AS1	_
ENSG00000248223	4.668855	-2.01648	0.285714	1	CTD-2139B15.2	_
ENSG00000249241	4.668855	-2.01648	0.285714	1	AC195454.1	_
ENSG00000249840	4.668855	-2.01648	0.285714	1	AC020947.2	K00134
ENSG00000249899	4.668855	-2.01648	0.285714	1	CTD-2175A23.1	_
ENSG00000249908	4.668855	-2.01648	0.285714	1	CTD-2589H19.4	K11723
ENSG00000250091	4.668855	-2.01648	0.285714	1	DNAH10OS	K10408
ENSG00000250379	4.668855	-2.01648	0.285714	1	RP11-23P13.4	_
ENSG00000250536	4.668855	-2.01648	0.285714	1	ABHD17AP3	_
ENSG00000250635	4.668855	-2.01648	0.285714	1	CTD-3224K15.2	_
ENSG00000251093	4.668855	-2.01648	0.285714	1	RP11-414H23.3	_
ENSG00000251281	4.668855	-2.01648	0.285714	1	CTD-2066L21.2	_
ENSG00000251552	4.668855	-2.01648	0.285714	1	COQ10BP2	K18588
ENSG00000251577	4.668855	-2.01648	0.285714	1	RP11-297P16.3	_

ENSG00000251602	4.668855	-2.01648	0.285714	1	RP11-521B24.3	_
ENSG00000251682	4.668855	-2.01648	0.285714	1	RP11-631M6.3	K18656
ENSG00000251884	4.668855	-2.01648	0.285714	1	RNA5SP288	_
ENSG00000251988	4.668855	-2.01648	0.285714	1	RNU4ATAC18P	_
ENSG00000252893	4.668855	-2.01648	0.285714	1	RNU7-58P	_
ENSG00000252943	4.668855	-2.01648	0.285714	1	RNU6-264P	_
ENSG00000253256	4.668855	-2.01648	0.285714	1	CTB-11I22.1	_
ENSG00000253615	4.668855	-2.01648	0.285714	1	RP11-597M17.2	_
ENSG00000253676	4.668855	-2.01648	0.285714	1	TAGLN2P1	_
ENSG00000254120	4.668855	-2.01648	0.285714	1	RP11-705O24.2	_
ENSG00000254267	4.668855	-2.01648	0.285714	1	KB-1458E12.1	_
ENSG00000254274	4.668855	-2.01648	0.285714	1	TDGF1P5	_
ENSG00000254458	4.668855	-2.01648	0.285714	1	RP11-867G23.13	_
ENSG00000254736	4.668855	-2.01648	0.285714	1	RP11-867G23.9	_
ENSG00000254762	4.668855	-2.01648	0.285714	1	RP11-867G23.2	_
ENSG00000255092	4.668855	-2.01648	0.285714	1	RP11-58K22.4	_
ENSG00000255311	4.668855	-2.01648	0.285714	1	RP11-160H12.2	_
ENSG00000256030	4.668855	-2.01648	0.285714	1	CBX3P4	K11586
ENSG00000256176	4.668855	-2.01648	0.285714	1	RP11-627K11.3	K15105
ENSG00000256243	4.668855	-2.01648	0.285714	1	RPL7AP3	K02936
ENSG00000256250	4.668855	-2.01648	0.285714	1	RP11-989F5.1	_
ENSG00000256287	4.668855	-2.01648	0.285714	1	RP11-664H17.1	_
ENSG00000257219	4.668855	-2.01648	0.285714	1	RP11-54A9.1	_
ENSG00000257354	4.668855	-2.01648	0.285714	1	RP11-631N16.2	_
ENSG00000257526	4.668855	-2.01648	0.285714	1	RP11-20E24.1	_
ENSG00000257588	4.668855	-2.01648	0.285714	1	RP11-469H8.6	_
ENSG00000257851	4.668855	-2.01648	0.285714	1	HNRNPA3P10	K12741
ENSG00000258611	4.668855	-2.01648	0.285714	1	RP11-368J22.2	K09277
ENSG00000258689	4.668855	-2.01648	0.285714	1	RP6-65G23.1	_
ENSG00000258821	4.668855	-2.01648	0.285714	1	AC005041.17	_
ENSG00000258858	4.668855	-2.01648	0.285714	1	RP11-982M15.5	_
ENSG00000258904	4.668855	-2.01648	0.285714	1	RP11-638I2.2	_
ENSG00000259155	4.668855	-2.01648	0.285714	1	RP11-831F12.3	K08856
ENSG00000259201	4.668855	-2.01648	0.285714	1	RP11-56B16.5	_
ENSG00000259438	4.668855	-2.01648	0.285714	1	CTD-2650P22.1	_
ENSG00000259531	4.668855	-2.01648	0.285714	1	RP11-295H24.3	K09596
ENSG00000259571	4.668855	-2.01648	0.285714	1	BLID	_
ENSG00000259573	4.668855	-2.01648	0.285714	1	NMNAT1P5	K06210
ENSG00000259584	4.668855	-2.01648	0.285714	1	RP11-521C20.2	_
ENSG00000259632	4.668855	-2.01648	0.285714	1	RP11-56B16.1	K14557
ENSG00000259656	4.668855	-2.01648	0.285714	1	RP11-325E5.1	_
ENSG00000260008	4.668855	-2.01648	0.285714	1	RP11-732A21.2	_
ENSG00000260500	4.668855	-2.01648	0.285714	1	CTD-3193O13.1	_
ENSG00000260589	4.668855	-2.01648	0.285714	1	RP11-390B4.5	_
ENSG00000260635	4.668855	-2.01648	0.285714	1	RP11-21M24.3	_
ENSG00000260908	4.668855	-2.01648	0.285714	1	CTB-134H23.3	K17985
ENSG00000261251	4.668855	-2.01648	0.285714	1	RP3-388M5.9	_
ENSG00000261252	4.668855	-2.01648	0.285714	1	RP11-318L16.6	_
ENSG00000261334	4.668855	-2.01648	0.285714	1	RP11-65J3.14	_
ENSG00000261528	4.668855	-2.01648	0.285714	1	AC002400.1	K18468
ENSG00000261582	4.668855	-2.01648	0.285714	1	RP4-614O4.11	_
ENSG00000261794	4.668855	-2.01648	0.285714	1	GOLGA8H	K10595
ENSG00000261868	4.668855	-2.01648	0.285714	1	RP11-104O19.2	_
ENSG00000262332	4.668855	-2.01648	0.285714	1	RP11-517A5.7	_
ENSG00000262497	4.668855	-2.01648	0.285714	1	FAM187B2P	_
ENSG00000262768	4.668855	-2.01648	0.285714	1	RP11-353N14.1	_
ENSG00000263098	4.668855	-2.01648	0.285714	1	RP11-567O16.1	_
ENSG00000263219	4.668855	-2.01648	0.285714	1	RYKP1	K05128

ENSG00000263370	4.668855	-2.01648	0.285714	1	RP11-68I3.5	_
ENSG00000263675	4.668855	-2.01648	0.285714	1	MIR5581	_
ENSG00000264483	4.668855	-2.01648	0.285714	1	MIR5008	_
ENSG00000265395	4.668855	-2.01648	0.285714	1	MIR3944	_
ENSG00000266306	4.668855	-2.01648	0.285714	1	CTD-2008P7.6	_
ENSG00000266498	4.668855	-2.01648	0.285714	1	RP11-45M22.5	_
ENSG00000266533	4.668855	-2.01648	0.285714	1	MIR3619	_
ENSG00000266642	4.668855	-2.01648	0.285714	1	RP11-20B24.4	_
ENSG00000266826	4.668855	-2.01648	0.285714	1	CTD-2200P10.1	K17606
ENSG00000266946	4.668855	-2.01648	0.285714	1	MRPL37P1	K17418
ENSG00000267203	4.668855	-2.01648	0.285714	1	SNRPGP4	K11099
ENSG00000267332	4.668855	-2.01648	0.285714	1	CTD-2596O15.1	_
ENSG00000267370	4.668855	-2.01648	0.285714	1	CTD-2623N2.3	_
ENSG00000267582	4.668855	-2.01648	0.285714	1	CTD-3252C9.2	_
ENSG00000267659	4.668855	-2.01648	0.285714	1	RP11-118B18.1	_
ENSG00000267724	4.668855	-2.01648	0.285714	1	RP11-49K24.8	_
ENSG00000268742	4.668855	-2.01648	0.285714	1	CTD-2396E7.7	K04674
ENSG00000268866	4.668855	-2.01648	0.285714	1	AC020915.2	_
ENSG00000269058	4.668855	-2.01648	0.285714	1	CALR3	K10098
ENSG00000269246	4.668855	-2.01648	0.285714	1	CTC-246B18.10	_
ENSG00000269476	4.668855	-2.01648	0.285714	1	CTD-2583A14.9	K09228
ENSG00000269516	4.668855	-2.01648	0.285714	1	CYP4F23P	K17726
ENSG00000269578	4.668855	-2.01648	0.285714	1	CTD-3222D19.5	_
ENSG00000269806	4.668855	-2.01648	0.285714	1	CTD-3001H11.1	_
ENSG00000269901	4.668855	-2.01648	0.285714	1	RP11-178L8.9	_
ENSG00000269961	4.668855	-2.01648	0.285714	1	CTD-2033C11.1	_
ENSG00000270001	4.668855	-2.01648	0.285714	1	RP11-218C14.8	_
ENSG00000270157	4.668855	-2.01648	0.285714	1	RP5-894A10.6	_
ENSG00000270390	4.668855	-2.01648	0.285714	1	RP11-470B22.1	_
ENSG00000270510	4.668855	-2.01648	0.285714	1	RP11-777F6.3	_
ENSG00000270839	4.668855	-2.01648	0.285714	1	CTC-571O20.1	_
ENSG00000270995	4.668855	-2.01648	0.285714	1	RP11-29B11.5	_
ENSG00000271075	4.668855	-2.01648	0.285714	1	RP11-589M4.4	_
ENSG00000271181	4.668855	-2.01648	0.285714	1	CTA-221G9.10	_
ENSG00000271185	4.668855	-2.01648	0.285714	1	RP5-855F16.1	_
ENSG00000271443	4.668855	-2.01648	0.285714	1	RP11-541E12.1	_
ENSG00000271892	4.668855	-2.01648	0.285714	1	CTD-2228A4.1	_
ENSG00000271993	4.668855	-2.01648	0.285714	1	RP11-285J16.1	_
ENSG00000272057	4.668855	-2.01648	0.285714	1	CTB-40H15.4	_
ENSG00000272102	4.668855	-2.01648	0.285714	1	RP3-355L5.5	_
ENSG00000272202	4.668855	-2.01648	0.285714	1	RP11-157F20.3	_
ENSG00000272226	4.668855	-2.01648	0.285714	1	RP11-63G10.3	_
ENSG00000272573	4.668855	-2.01648	0.285714	1	MUSTN1	_
ENSG00000272610	4.668855	-2.01648	0.285714	1	MAGI1-IT1	_
ENSG00000272656	4.668855	-2.01648	0.285714	1	RP11-219D15.3	_
ENSG00000272784	4.668855	-2.01648	0.285714	1	RP11-335L23.5	_
ENSG00000272940	4.668855	-2.01648	0.285714	1	CTA-384D8.33	_
ENSG00000272986	4.668855	-2.01648	0.285714	1	RP11-46J23.1	_
ENSG00000273046	4.668855	-2.01648	0.285714	1	RP11-834C11.14	_
ENSG00000273139	4.668855	-2.01648	0.285714	1	XXbac-B444P24.14	_
ENSG00000273257	4.668855	-2.01648	0.285714	1	RP11-177J6.1	_
ENSG00000005421	-4.631531	-1.658813	0.285714	1	PON1	K01045
ENSG00000019186	-4.631531	-1.658813	0.285714	1	CYP24A1	K07436
ENSG00000047644	-4.631531	-1.658813	0.285714	1	WWC3	_
ENSG00000047936	-4.631531	-1.658813	0.285714	1	ROS1	K05088
ENSG00000049323	-4.631531	-1.658813	0.285714	1	LTBP1	K19559
ENSG00000077935	-4.631531	-1.658813	0.285714	1	SMC1B	K06636
ENSG00000078795	-4.631531	-1.658813	0.285714	1	PKD2L2	K04991

ENSG00000096996	-4.631531	-1.658813	0.285714	1	IL12RB1	K05063
ENSG00000102057	-4.631531	-1.658813	0.285714	1	KCND1	K04891
ENSG00000105261	-4.631531	-1.658813	0.285714	1	OVOL3	K09216
ENSG00000107611	-4.631531	-1.658813	0.285714	1	CUBN	K14616
ENSG00000114786	-4.631531	-1.658813	0.285714	1	ABHD14A-ACY1	K14677
ENSG00000121270	-4.631531	-1.658813	0.285714	1	ABCC11	K05671
ENSG00000121577	-4.631531	-1.658813	0.285714	1	POPDC2	_
ENSG00000124449	-4.631531	-1.658813	0.285714	1	IRGC	_
ENSG00000127325	-4.631531	-1.658813	0.285714	1	BEST3	K13880
ENSG00000129654	-4.631531	-1.658813	0.285714	1	FOXJ1	K09402
ENSG00000136918	-4.631531	-1.658813	0.285714	1	WDR38	_
ENSG00000149948	-4.631531	-1.658813	0.285714	1	HMGA2	K09283
ENSG00000152936	-4.631531	-1.658813	0.285714	1	IFLTD1	_
ENSG00000153993	-4.631531	-1.658813	0.285714	1	SEMA3D	K06840
ENSG00000158125	-4.631531	-1.658813	0.285714	1	XDH	K00106
ENSG00000158258	-4.631531	-1.658813	0.285714	1	CLSTN2	K12581
ENSG00000160767	-4.631531	-1.658813	0.285714	1	FAM189B	_
ENSG00000163017	-4.631531	-1.658813	0.285714	1	ACTG2	K12315
ENSG00000163932	-4.631531	-1.658813	0.285714	1	PRKCD	K06068
ENSG00000166920	-4.631531	-1.658813	0.285714	1	C15orf48	_
ENSG00000174885	-4.631531	-1.658813	0.285714	1	NLRP6	_
ENSG00000175315	-4.631531	-1.658813	0.285714	1	CST6	K13902
ENSG00000179388	-4.631531	-1.658813	0.285714	1	EGR3	K12497
ENSG00000183831	-4.631531	-1.658813	0.285714	1	ANKRD45	K14726
ENSG00000185739	-4.631531	-1.658813	0.285714	1	SRL	K17985
ENSG00000187689	-4.631531	-1.658813	0.285714	1	AMTN	_
ENSG00000188175	-4.631531	-1.658813	0.285714	1	HEPACAM2	_
ENSG00000189433	-4.631531	-1.658813	0.285714	1	GJB4	K07623
ENSG00000197635	-4.631531	-1.658813	0.285714	1	DPP4	K01278
ENSG00000198580	-4.631531	-1.658813	0.285714	1	AC073343.1	K09228
ENSG00000198846	-4.631531	-1.658813	0.285714	1	TOX	_
ENSG00000199846	-4.631531	-1.658813	0.285714	1	RNU1-72P	_
ENSG00000204666	-4.631531	-1.658813	0.285714	1	CTD-2126E3.1	_
ENSG00000205959	-4.631531	-1.658813	0.285714	1	RP11-689P11.2	_
ENSG00000206337	-4.631531	-1.658813	0.285714	1	HCP5	_
ENSG00000207151	-4.631531	-1.658813	0.285714	1	Y_RNA	_
ENSG00000207650	-4.631531	-1.658813	0.285714	1	MIR570	_
ENSG00000207973	-4.631531	-1.658813	0.285714	1	MIR589	_
ENSG00000214552	-4.631531	-1.658813	0.285714	1	COPS8P2	K12181
ENSG00000215456	-4.631531	-1.658813	0.285714	1	BCRP4	K08878
ENSG00000218772	-4.631531	-1.658813	0.285714	1	FAM8A6P	_
ENSG00000219027	-4.631531	-1.658813	0.285714	1	RPS3AP2	_
ENSG00000219298	-4.631531	-1.658813	0.285714	1	RP11-472G23.3	K07986
ENSG00000223198	-4.631531	-1.658813	0.285714	1	RNU2-22P	_
ENSG00000224063	-4.631531	-1.658813	0.285714	1	AC007319.1	_
ENSG00000224945	-4.631531	-1.658813	0.285714	1	RP11-82L18.2	_
ENSG00000225028	-4.631531	-1.658813	0.285714	1	RP11-330M19.1	_
ENSG00000225039	-4.631531	-1.658813	0.285714	1	LINC01058	_
ENSG00000225108	-4.631531	-1.658813	0.285714	1	ZBTB45P1	K10516
ENSG00000225450	-4.631531	-1.658813	0.285714	1	RP3-508I15.14	_
ENSG00000228157	-4.631531	-1.658813	0.285714	1	AC007952.5	_
ENSG00000228294	-4.631531	-1.658813	0.285714	1	BMS1P17	_
ENSG00000228485	-4.631531	-1.658813	0.285714	1	GRK5-IT1	_
ENSG00000229127	-4.631531	-1.658813	0.285714	1	AC007038.7	_
ENSG00000229852	-4.631531	-1.658813	0.285714	1	RP11-398K22.12	_
ENSG00000230091	-4.631531	-1.658813	0.285714	1	TMEM254-AS1	_
ENSG00000230712	-4.631531	-1.658813	0.285714	1	AP000354.4	K18592
ENSG00000230812	-4.631531	-1.658813	0.285714	1	RP4-794H19.4	_

ENSG00000231185	-4.631531	-1.658813	0.285714	1	AC005592.2	_
ENSG00000231407	-4.631531	-1.658813	0.285714	1	RP11-576I22.2	_
ENSG00000231842	-4.631531	-1.658813	0.285714	1	RP11-527F13.1	_
ENSG00000231871	-4.631531	-1.658813	0.285714	1	IPO9-AS1	_
ENSG00000232043	-4.631531	-1.658813	0.285714	1	RP4-530I15.9	_
ENSG00000232460	-4.631531	-1.658813	0.285714	1	BMPR1APS2	K04673
ENSG00000233509	-4.631531	-1.658813	0.285714	1	ZNF197-AS1	_
ENSG00000233514	-4.631531	-1.658813	0.285714	1	RP11-570P14.1	K11087
ENSG00000233611	-4.631531	-1.658813	0.285714	1	AC079135.1	_
ENSG00000234175	-4.631531	-1.658813	0.285714	1	RP11-730A19.9	_
ENSG00000235437	-4.631531	-1.658813	0.285714	1	RP11-357C3.3	_
ENSG00000236591	-4.631531	-1.658813	0.285714	1	RP11-162J8.3	_
ENSG00000237683	-4.631531	-1.658813	0.285714	1	AL627309.1	_
ENSG00000238350	-4.631531	-1.658813	0.285714	1	snoU13	_
ENSG00000240106	-4.631531	-1.658813	0.285714	1	RN7SL146P	_
ENSG00000241104	-4.631531	-1.658813	0.285714	1	CEACAMP10	K06500
ENSG00000241218	-4.631531	-1.658813	0.285714	1	RP11-446H18.1	K01647
ENSG00000241769	-4.631531	-1.658813	0.285714	1	LINC00893	_
ENSG00000245573	-4.631531	-1.658813	0.285714	1	BDNF-AS	_
ENSG00000245888	-4.631531	-1.658813	0.285714	1	CTD-3203P2.2	_
ENSG00000248375	-4.631531	-1.658813	0.285714	1	RP11-177B4.1	K06641
ENSG00000249249	-4.631531	-1.658813	0.285714	1	AC010226.4	_
ENSG00000249357	-4.631531	-1.658813	0.285714	1	RP11-432M8.8	K03135
ENSG00000253084	-4.631531	-1.658813	0.285714	1	RNU6-840P	_
ENSG00000253328	-4.631531	-1.658813	0.285714	1	SUMO2P19	K12160
ENSG00000254039	-4.631531	-1.658813	0.285714	1	RP11-304F15.6	_
ENSG00000254290	-4.631531	-1.658813	0.285714	1	RP11-150O12.3	_
ENSG00000255046	-4.631531	-1.658813	0.285714	1	RP11-297N6.4	K04613
ENSG00000255158	-4.631531	-1.658813	0.285714	1	RP11-754B17.1	_
ENSG00000255449	-4.631531	-1.658813	0.285714	1	RP11-91P24.6	_
ENSG00000256316	-4.631531	-1.658813	0.285714	1	HIST1H3F	K11253
ENSG00000256980	-4.631531	-1.658813	0.285714	1	KHDC1L	_
ENSG00000257818	-4.631531	-1.658813	0.285714	1	RP11-1143G9.2	K00731
ENSG00000258414	-4.631531	-1.658813	0.285714	1	RP11-356O9.1	_
ENSG00000258572	-4.631531	-1.658813	0.285714	1	RP11-1070N10.3	_
ENSG00000258658	-4.631531	-1.658813	0.285714	1	RP11-517O13.3	_
ENSG00000259327	-4.631531	-1.658813	0.285714	1	CTD-2184D3.6	_
ENSG00000260118	-4.631531	-1.658813	0.285714	1	RP6-201G10.2	_
ENSG00000260171	-4.631531	-1.658813	0.285714	1	RP11-95H11.1	_
ENSG00000260396	-4.631531	-1.658813	0.285714	1	AC012065.7	_
ENSG00000260583	-4.631531	-1.658813	0.285714	1	LINC00515	_
ENSG00000260765	-4.631531	-1.658813	0.285714	1	CES1P2	K01044
ENSG00000260949	-4.631531	-1.658813	0.285714	1	KB-1836B5.1	_
ENSG00000261215	-4.631531	-1.658813	0.285714	1	RP11-195F19.30	_
ENSG00000261600	-4.631531	-1.658813	0.285714	1	RP11-575H3.1	_
ENSG00000262298	-4.631531	-1.658813	0.285714	1	RP5-1107A17.3	_
ENSG00000262766	-4.631531	-1.658813	0.285714	1	RP11-196G11.4	_
ENSG00000262777	-4.631531	-1.658813	0.285714	1	RP11-818O24.3	_
ENSG00000263321	-4.631531	-1.658813	0.285714	1	RP11-388C12.5	_
ENSG00000263627	-4.631531	-1.658813	0.285714	1	RP11-692N5.1	_
ENSG00000264175	-4.631531	-1.658813	0.285714	1	MIR3189	_
ENSG00000264448	-4.631531	-1.658813	0.285714	1	MIR378D2	_
ENSG00000265800	-4.631531	-1.658813	0.285714	1	RP11-649A18.5	_
ENSG00000266283	-4.631531	-1.658813	0.285714	1	RP11-627G18.1	_
ENSG00000267637	-4.631531	-1.658813	0.285714	1	RP11-619I22.1	_
ENSG00000268069	-4.631531	-1.658813	0.285714	1	AC004466.1	K11408
ENSG00000269019	-4.631531	-1.658813	0.285714	1	AC005932.1	_
ENSG00000269403	-4.631531	-1.658813	0.285714	1	CTD-2616J11.11	K03521

ENSG00000269892	-4.631531	-1.658813	0.285714	1	RP4-761J14.10	_
ENSG00000270069	-4.631531	-1.658813	0.285714	1	RP6-99M1.2	_
ENSG00000270617	-4.631531	-1.658813	0.285714	1	URGCP-MRPS24	K17403
ENSG00000271161	-4.631531	-1.658813	0.285714	1	RP11-708J19.2	_
ENSG00000272201	-4.631531	-1.658813	0.285714	1	U47924.30	_
ENSG00000272360	-4.631531	-1.658813	0.285714	1	RP11-359I18.5	_
ENSG00000272457	-4.631531	-1.658813	0.285714	1	RP11-1070A24.2	_
ENSG00000272854	-4.631531	-1.658813	0.285714	1	RP4-593H12.1	_
ENSG00000272941	-4.631531	-1.658813	0.285714	1	RP11-134L10.1	_
ENSG00000273097	-4.631531	-1.658813	0.285714	1	RP11-219C20.3	_
ENSG00000273264	-4.631531	-1.658813	0.285714	1	RP11-131L23.2	_
ENSG00000273384	-4.631531	-1.658813	0.285714	1	RP5-1098D14.1	_
ENSG00000273389	-4.631531	-1.658813	0.285714	1	RP13-514E23.2	_
ENSG00000273493	-4.631531	-1.658813	0.285714	1	RP11-80H18.4	_
ENSG00000175115	0.704478	6.664569	0.286001	1	PACS1	_
ENSG00000144048	-0.714501	5.087422	0.286049	1	DUSP11	K14165
ENSG00000105427	0.786579	1.868122	0.286292	1	CNFN	_
ENSG00000256982	0.786579	1.868122	0.286292	1	CTD-2555A7.2	_
ENSG00000169242	0.70297	6.845697	0.286757	1	EFNA1	K05462
ENSG00000213865	0.754198	3.178925	0.286807	1	C8orf44	K12581
ENSG00000196696	-0.779767	2.496422	0.28681	1	PDXDC2P	_
ENSG00000173950	-0.721436	4.523365	0.287169	1	XXYLT1	_
ENSG00000162413	0.709913	5.517023	0.28733	1	KLHL21	K10458
ENSG00000255198	0.871451	1.285769	0.287651	1	SNHG9	_
ENSG00000162804	0.725671	3.958529	0.28766	1	SNED1	_
ENSG00000173727	0.741735	2.965498	0.287895	1	AP000769.1	K02983
ENSG00000148444	-0.72779	4.181621	0.287928	1	COMMD3	K11459
ENSG00000152778	-0.762265	2.828262	0.288039	1	IFIT5	K14217
ENSG00000145908	-0.744738	3.605597	0.288089	1	ZNF300	K09228
ENSG00000153558	0.746881	3.068334	0.288796	1	FBXL2	K10268
ENSG00000143353	-0.730848	3.912023	0.288852	1	LYPLAL1	K06999
ENSG00000129028	-0.776704	2.935733	0.289047	1	THAP10	_
ENSG00000113504	0.706207	5.141701	0.289412	1	SLC12A7	K13627
ENSG00000089847	0.90273	1.033447	0.290499	1	ANKRD24	_
ENSG00000229932	0.90273	1.033447	0.290499	1	YWHAZP3	K16197
ENSG00000270223	0.90273	1.033447	0.290499	1	RP13-36G14.4	_
ENSG00000119725	-0.889084	1.131318	0.290499	1	ZNF410	K13949
ENSG00000230615	-0.889084	1.131318	0.290499	1	RP5-1198O20.4	_
ENSG00000135406	0.841766	1.058421	0.290499	1	PRPH	K07607
ENSG00000223891	0.813496	1.562887	0.290856	1	OSER1-AS1	_
ENSG00000131725	0.699989	5.623477	0.29139	1	WDR44	_
ENSG00000188690	-0.705702	5.046975	0.291476	1	UROS	K01719
ENSG00000129657	0.696367	6.361631	0.291832	1	SEC14L1	_
ENSG00000169189	-0.711574	4.778843	0.291972	1	NSMCE1	_
ENSG00000120699	-0.698006	6.403749	0.292084	1	EXOSC8	K12586
ENSG00000145506	0.718145	4.134115	0.292292	1	NKD2	K03213
ENSG00000165929	0.732368	3.140097	0.292459	1	TC2N	K17287
ENSG00000163964	-0.705816	5.025655	0.292611	1	PIGX	K07541
ENSG00000188994	0.707174	4.908222	0.292844	1	ZNF292	_
ENSG00000163104	-0.704576	5.135466	0.29286	1	SMARCAD1	K14439
ENSG00000142046	0.813991	1.818271	0.292915	1	TMEM91	_
ENSG00000238084	-0.806598	1.871983	0.292915	1	RP3-469D22.1	K00237
ENSG00000243749	-0.806598	1.871983	0.292915	1	ZMYM6NB	_
ENSG00000268460	-0.787935	2.366238	0.293234	1	AC006262.6	_
ENSG00000118513	-0.904736	1.458613	0.293828	1	MYB	K09420
ENSG00000143614	0.883172	1.387656	0.293828	1	GATAD2B	_
ENSG00000152763	0.883172	1.387656	0.293828	1	WDR78	_
ENSG00000188277	0.883172	1.387656	0.293828	1	C15orf62	_

ENSG00000186615	0.836125	1.406868	0.293828	1	KTN1-AS1	_
ENSG00000215067	0.836125	1.406868	0.293828	1	AC027763.2	K12417
ENSG00000249199	0.836125	1.406868	0.293828	1	CTD-2139B15.5	_
ENSG00000146426	-0.954791	0.91474	0.293949	1	TIAM2	K16847
ENSG00000172062	-0.954791	0.91474	0.293949	1	SMN1	K13129
ENSG00000203739	0.954703	0.803404	0.293949	1	RP11-296O14.3	_
ENSG00000225573	0.954703	0.803404	0.293949	1	RPL35P5	K02918
ENSG00000229729	0.954703	0.803404	0.293949	1	RP11-159G9.5	_
ENSG00000236992	0.954703	0.803404	0.293949	1	RPL12L3	K02870
ENSG00000164953	-0.714624	4.186325	0.294034	1	TMEM67	K19348
ENSG00000163283	0.691308	7.245061	0.294153	1	ALPP	K01077
ENSG00000181481	-0.716087	4.066115	0.294212	1	RNF135	K16272
ENSG00000064651	-0.714718	4.286117	0.295258	1	SLC12A2	K10951
ENSG00000203875	-0.70106	4.871353	0.295425	1	SNHG5	_
ENSG00000250337	-0.70714	4.504139	0.295507	1	LINC01021	_
ENSG00000118503	-0.703753	4.799376	0.29561	1	TNFAIP3	K11859
ENSG00000131069	-0.693863	5.689487	0.29586	1	ACSS2	K01895
ENSG00000196449	-0.705399	4.808774	0.296154	1	YRDC	_
ENSG00000164850	0.720801	3.688934	0.296425	1	GPER1	K04246
ENSG00000167703	0.694835	5.137977	0.296474	1	SLC43A2	K08229
ENSG00000165655	-0.72195	3.774767	0.296639	1	ZNF503	_
ENSG00000234362	0.79267	1.903188	0.296983	1	AC104654.2	_
ENSG00000161642	0.697844	4.921374	0.297043	1	ZNF385A	_
ENSG00000182871	0.752207	2.660073	0.297256	1	COL18A1	K06823
ENSG00000106610	-0.715534	3.993797	0.297339	1	STAG3L4	K13055
ENSG00000183474	-0.9261	1.269403	0.297457	1	GTF2H2C	K03142
ENSG00000183840	-0.9261	1.269403	0.297457	1	GPR39	K08412
ENSG00000253598	0.857819	1.176412	0.297457	1	SLC10A5	K14343
ENSG00000184470	-0.690959	6.127452	0.297731	1	TXNRD2	K00384
ENSG00000115841	-1.003325	0.627399	0.298081	1	RMDN2	K12756
ENSG00000265962	-1.003325	0.627399	0.298081	1	RP11-674N23.1	_
ENSG00000135338	-0.952996	0.594511	0.298081	1	LCA5	_
ENSG00000186007	-0.952996	0.594511	0.298081	1	LEMD1	_
ENSG00000261690	-0.952996	0.594511	0.298081	1	AC009133.12	_
ENSG00000180834	0.931663	0.478331	0.298081	1	MAP6D1	_
ENSG00000198134	0.931663	0.478331	0.298081	1	RP11-267J23.4	K13784
ENSG00000263412	0.931663	0.478331	0.298081	1	RP5-890E16.2	_
ENSG00000107262	0.683918	8.62852	0.298114	1	BAG1	K09555
ENSG00000167550	-0.714054	3.699164	0.298522	1	RHEBL1	K07849
ENSG00000122035	0.714533	3.906841	0.298984	1	RASL11A	K07852
ENSG00000153832	-0.815924	1.578257	0.299162	1	FBXO36	K10312
ENSG00000203724	-0.815924	1.578257	0.299162	1	C1orf53	_
ENSG00000163386	0.754586	2.566985	0.299211	1	NBPF10	_
ENSG00000172183	-0.708223	4.169351	0.299393	1	ISG20	K12579
ENSG00000140459	-0.712595	3.661583	0.299542	1	CYP11A1	K00498
ENSG00000127663	0.691641	5.093585	0.299594	1	KDM4B	K06709
ENSG00000272645	-0.839744	1.824277	0.300098	1	RP11-504P24.8	_
ENSG00000102878	-0.724181	3.162183	0.300153	1	HSF4	K09417
ENSG00000121988	-0.724181	3.162183	0.300153	1	ZRANB3	K09488
ENSG00000153904	-0.685009	6.401203	0.301059	1	DDAH1	K01482
ENSG00000171346	0.750111	2.747547	0.301579	1	KRT15	K07604
ENSG00000095637	0.732868	2.754552	0.301579	1	SORBS1	K06086
ENSG00000253276	-0.706606	3.988202	0.301752	1	CCDC71L	_
ENSG00000162971	-0.714688	3.644209	0.301763	1	TYW5	K18066
ENSG00000198440	-0.95542	1.051035	0.301975	1	ZNF583	K09228
ENSG00000221995	0.896275	0.969574	0.301975	1	TIAF1	K10362
ENSG00000075651	0.692845	4.845398	0.302166	1	PLD1	K01115
ENSG00000105329	0.680592	6.523364	0.302175	1	TGFB1	K13375

ENSG00000188167	1.163012	0.079269	0.302645	1	TMPPE	_
ENSG00000232630	1.163012	0.079269	0.302645	1	PRPS1P2	K00948
ENSG00000236861	1.163012	0.079269	0.302645	1	AC006378.2	_
ENSG00000269979	1.163012	0.079269	0.302645	1	RP11-498E2.7	_
ENSG00000270133	1.163012	0.079269	0.302645	1	CTC-303L1.2	_
ENSG00000125775	1.027679	0.129644	0.302645	1	SDCBP2	_
ENSG00000180015	1.027679	0.129644	0.302645	1	RP11-756P10.3	K02835
ENSG00000213073	1.027679	0.129644	0.302645	1	RP11-288H12.3	K17610
ENSG00000227533	1.027679	0.129644	0.302645	1	SLC2A1-AS1	_
ENSG00000234281	1.027679	0.129644	0.302645	1	AC007970.1	_
ENSG00000234648	1.027679	0.129644	0.302645	1	AL162151.3	K02925
ENSG00000240891	1.027679	0.129644	0.302645	1	PLCXD2	_
ENSG00000197971	-0.711938	3.900139	0.3027	1	MBP	K17269
ENSG00000171246	0.67668	8.124754	0.303524	1	NPTX1	_
ENSG00000247134	-0.847861	1.680097	0.303817	1	RP11-11N9.4	_
ENSG00000182885	-0.82484	1.665124	0.303817	1	GPR97	K08452
ENSG00000076928	0.820224	1.605921	0.303817	1	ARHGEF1	K12330
ENSG00000237172	0.780877	1.622236	0.303817	1	B3GNT9	K18705
ENSG00000269430	0.756824	2.059312	0.303985	1	LRRC3DN	_
ENSG00000197757	0.713666	3.464674	0.304259	1	HOXC6	K09306
ENSG00000005020	-0.691839	4.783541	0.304259	1	SKAP2	_
ENSG00000226479	-0.696668	4.44506	0.304322	1	TMEM185B	_
ENSG00000023572	-0.693409	4.44303	0.304322	1	GLRX2	K03676
ENSG00000080608	-0.678421	6.711418	0.304436	1	KIAA0020	K14844
ENSG00000185986	-0.725885	3.337181	0.304519	1	SDHAP3	K00234
ENSG00000092978	-0.709255	3.834131	0.304729	1	GPATCH2	K17569
ENSG00000163584	-0.675382	7.602045	0.305117	1	RPL22L1	K02891
ENSG00000079435	1.238158	-0.493694	0.305221	1	LIPE	K07188
ENSG00000104327	1.238158	-0.493694	0.305221	1	CALB1	K14757
ENSG00000138316	1.238158	-0.493694	0.305221	1	ADAMTS14	K08628
ENSG00000147394	1.238158	-0.493694	0.305221	1	ZNF185	_
ENSG00000183682	1.238158	-0.493694	0.305221	1	BMP8A	K16622
ENSG00000197245	1.238158	-0.493694	0.305221	1	FAM110D	_
ENSG00000205086	1.238158	-0.493694	0.305221	1	C2orf91	_
ENSG00000206178	1.238158	-0.493694	0.305221	1	HBZP1	K13826
ENSG00000219626	1.238158	-0.493694	0.305221	1	FAM228B	K10133
ENSG00000221916	1.238158	-0.493694	0.305221	1	C19orf73	_
ENSG00000231313	1.238158	-0.493694	0.305221	1	CLIC1P1	K05021
ENSG00000243587	1.238158	-0.493694	0.305221	1	C6orf183	_
ENSG00000248734	1.238158	-0.493694	0.305221	1	CTD-2260A17.1	_
ENSG00000251593	1.238158	-0.493694	0.305221	1	MSNP1	K05763
ENSG00000260233	1.238158	-0.493694	0.305221	1	SSSCA1-AS1	_
ENSG00000267500	1.238158	-0.493694	0.305221	1	CTD-2006C1.6	K09228
ENSG00000272658	1.238158	-0.493694	0.305221	1	LTB4R2	K04297
ENSG00000272677	1.238158	-0.493694	0.305221	1	RP11-127B20.3	_
ENSG00000257923	0.708641	3.492343	0.305446	1	CUX1	K09313
ENSG00000164442	-0.673957	7.978605	0.305733	1	CITED2	_
ENSG00000105829	-0.681039	5.859534	0.30577	1	BET1	K08504
ENSG00000198406	0.698788	4.01428	0.306074	1	BZW1P2	_
ENSG00000168301	-0.713829	3.546401	0.306592	1	KCTD6	_
ENSG00000099957	-0.759692	2.348112	0.306624	1	P2RX6	K05221
ENSG00000100234	0.674658	6.671819	0.306789	1	TIMP3	K16866
ENSG00000106031	0.723724	2.879192	0.307205	1	HOXA13	K09298
ENSG00000085719	-0.674319	6.771495	0.307216	1	CPNE3	_
ENSG00000164136	-0.708978	3.640623	0.307436	1	IL15	K05433
ENSG00000168389	-0.803187	1.998852	0.307454	1	MFSD2A	_
ENSG00000149313	-0.677049	5.915666	0.30751	1	AASDHPPT	K06133
ENSG00000159263	-0.722798	3.23844	0.307693	1	SIM2	K09100

ENSG00000234494	-0.99817	0.792786	0.307713	1	AC003665.1	_
ENSG00000119636	-0.954005	0.763759	0.307713	1	CCDC176	_
ENSG00000227285	-0.954005	0.763759	0.307713	1	RP3-327A19.5	_
ENSG00000244479	-0.954005	0.763759	0.307713	1	OR2A1-AS1	_
ENSG00000253796	-0.954005	0.763759	0.307713	1	RP11-1084E5.1	_
ENSG00000269386	-0.954005	0.763759	0.307713	1	RAB11B-AS1	_
ENSG00000168795	0.680658	5.414523	0.307957	1	ZBTB5	K10492
ENSG00000090530	-0.673853	6.650069	0.307965	1	LEPREL1	_
ENSG00000223573	0.67541	5.953076	0.308033	1	TINCR	_
ENSG00000074527	-0.678581	5.885657	0.308124	1	NTN4	K06845
ENSG00000231345	-0.858067	1.51964	0.308333	1	RP11-564C4.6	_
ENSG00000068323	0.850133	1.118579	0.308509	1	TFE3	K09105
ENSG00000140876	0.850133	1.118579	0.308509	1	NUDT7	K17879
ENSG00000256092	0.850133	1.118579	0.308509	1	hsa-mir-8072	_
ENSG00000111801	-0.69253	4.198109	0.308709	1	BTN3A3	K06712
ENSG00000169184	0.674951	6.014925	0.308865	1	MN1	_
ENSG00000103485	0.737795	2.370053	0.309113	1	QPRT	K00767
ENSG00000076826	0.681823	4.798796	0.309319	1	CAMSAP3	K17493
ENSG00000135537	-0.747583	2.852405	0.309392	1	LACE1	K18798
ENSG00000165861	0.713412	3.357353	0.309876	1	ZFYVE1	K17603
ENSG00000185033	0.670787	6.516141	0.309998	1	SEMA4B	K06521
ENSG00000173064	0.681073	4.85002	0.310045	1	HECTD4	K17849
ENSG00000197182	0.696549	3.534847	0.310185	1	FLJ27365	_
ENSG00000138640	0.688807	4.497658	0.310477	1	FAM13A	_
ENSG00000130433	0.779649	2.015812	0.310597	1	CACNG6	K04871
ENSG00000243302	0.779649	2.015812	0.310597	1	RP11-274B21.2	_
ENSG00000154328	-0.714529	3.485158	0.310599	1	NEIL2	K10568
ENSG00000134755	-0.691474	4.247306	0.310734	1	DSC2	K07601
ENSG00000124762	0.667551	7.341691	0.310803	1	CDKN1A	K06625
ENSG00000108813	0.682957	4.803524	0.310832	1	DLX4	K09314
ENSG00000171223	-0.665467	9.057819	0.311113	1	JUNB	K09028
ENSG00000230513	-0.741006	2.471631	0.311421	1	THAP7-AS1	_
ENSG00000260257	0.726239	2.428507	0.311421	1	RP5-1085F17.3	_
ENSG00000158825	0.667636	6.330183	0.311716	1	CDA	K01489
ENSG00000140443	0.666615	7.111031	0.311792	1	IGF1R	K05087
ENSG00000006015	-0.673335	5.484057	0.312198	1	C19orf60	_
ENSG00000155850	-0.677427	5.00803	0.31234	1	SLC26A2	K14701
ENSG00000085563	-0.766745	2.30447	0.312343	1	ABCB1	K05658
ENSG00000184584	-0.671969	5.141618	0.313028	1	TMEM173	K12654
ENSG00000060237	0.663087	8.466965	0.313038	1	WNK1	K08867
ENSG00000186603	-0.673683	5.442428	0.313174	1	HPDL	_
ENSG00000163393	-0.703245	3.637028	0.313213	1	SLC22A15	K08211
ENSG00000118600	-0.684661	4.314758	0.313242	1	TMEM5	K02927
ENSG00000170634	-0.758285	2.143977	0.313448	1	ACYP2	K01512
ENSG00000144395	-0.711039	3.396372	0.313889	1	CCDC150	_
ENSG00000133805	0.864902	1.232091	0.313932	1	AMPD3	K01490
ENSG00000180998	0.864902	1.232091	0.313932	1	GPR137C	_
ENSG00000173599	0.663568	6.627086	0.314349	1	PC	K01958
ENSG00000196535	0.669013	5.80829	0.314389	1	MYO18A	K10362
ENSG00000184898	-0.814315	1.942979	0.314789	1	RBM43	_
ENSG00000062370	-0.795248	1.93065	0.314789	1	ZNF112	K09228
ENSG00000270101	-0.722419	3.107248	0.314826	1	RP11-24B19.3	_
ENSG00000163607	-0.680463	4.358038	0.314993	1	GTPBP8	_
ENSG00000141441	-1.066359	0.476647	0.315121	1	GAREM	_
ENSG00000214776	-1.066359	0.476647	0.315121	1	RP11-726G1.1	K04850
ENSG00000242689	-1.066359	0.476647	0.315121	1	CNTF	K05420
ENSG00000160050	1.029385	0.343233	0.315121	1	CCDC28B	_
ENSG00000197558	1.029385	0.343233	0.315121	1	SSPO	_

ENSG00000205869	1.029385	0.343233	0.315121	1	KRTAP5-1	_
ENSG00000249709	1.029385	0.343233	0.315121	1	ZNF564	K09228
ENSG00000258457	1.029385	0.343233	0.315121	1	RP11-298I3.4	_
ENSG00000269982	1.029385	0.343233	0.315121	1	RP11-1020A11.2	_
ENSG00000234618	-1.010147	0.439831	0.315121	1	RPSAP9	K02998
ENSG00000157613	0.923786	0.384744	0.315121	1	CREB3L1	K09048
ENSG00000218226	0.923786	0.384744	0.315121	1	TATDN2P2	K03424
ENSG00000271996	0.923786	0.384744	0.315121	1	RP11-337N6.1	_
ENSG00000139190	0.69035	3.597979	0.315679	1	VAMP1	K08510
ENSG00000083635	-0.679755	4.476738	0.315883	1	NUFIP1	_
ENSG00000143633	-0.678999	4.334368	0.315944	1	C1orf131	K17776
ENSG00000135111	0.661807	6.458256	0.316135	1	TBX3	K10177
ENSG00000137513	-0.672057	4.821086	0.316437	1	NARS2	K01893
ENSG00000117148	0.78826	1.663565	0.316443	1	ACTL8	_
ENSG00000079691	0.663737	5.7604	0.316711	1	LRRC16A	_
ENSG00000182993	-0.74686	2.431623	0.316864	1	C12orf60	K17816
ENSG00000103642	-0.663084	6.035185	0.316988	1	LACTB	K17382
ENSG00000177707	-0.690008	3.962995	0.317354	1	PVRL3	K06592
ENSG00000038002	-0.699767	3.297146	0.317654	1	AGA	K01444
ENSG00000213096	-0.757398	2.029787	0.317704	1	ZNF254	K09228
ENSG00000146109	-0.877854	1.440974	0.318503	1	ABT1	K14785
ENSG00000225135	-0.877854	1.440974	0.318503	1	RP11-361F15.2	_
ENSG00000100084	-0.850461	1.42309	0.318503	1	HIRA	K11293
ENSG00000143951	-0.850461	1.42309	0.318503	1	WDPCP	_
ENSG00000168906	0.654812	9.686542	0.318588	1	MAT2A	K00789
ENSG00000077157	0.691217	3.512013	0.319001	1	PPP1R12B	K12329
ENSG00000151414	-0.657064	6.899761	0.319407	1	NEK7	K08857
ENSG00000129422	0.658422	6.293616	0.319416	1	MTUS1	_
ENSG00000243710	-0.819035	1.810821	0.319771	1	WDR65	K14823
ENSG00000162390	0.801823	1.742931	0.319771	1	ACOT11	K12417
ENSG00000169857	-0.675734	4.109486	0.320085	1	AVEN	_
ENSG00000148814	-0.745361	2.338955	0.320611	1	LRRC27	_
ENSG00000224177	0.720672	2.507779	0.32075	1	LINC00570	_
ENSG00000197121	-0.683584	3.767876	0.320771	1	PGAP1	K05294
ENSG00000183117	-0.707975	2.724333	0.320819	1	CSMD1	K17495
ENSG00000153029	-0.702275	3.274731	0.320849	1	MR1	K17985
ENSG00000205542	-0.651271	10.57076	0.321051	1	TMSB4X	K05764
ENSG00000091428	-0.854459	1.108769	0.321053	1	RAPGEF4	K04351
ENSG00000272438	-0.854459	1.108769	0.321053	1	RP11-54O7.16	_
ENSG00000107829	-0.676465	4.321279	0.321183	1	FBXW4	K10262
ENSG00000108840	0.657644	6.093651	0.321471	1	HDAC5	K11406
ENSG00000158769	0.661505	5.045182	0.322115	1	F11R	K06089
ENSG00000116741	-0.664644	5.021527	0.322159	1	RGS2	K18154
ENSG00000177485	0.798711	1.472899	0.32241	1	ZBTB33	K10507
ENSG00000260361	0.798711	1.472899	0.32241	1	CTD-2313J17.5	_
ENSG00000272686	-0.790907	1.562092	0.32241	1	RP11-390E23.6	_
ENSG00000140450	0.755858	1.490894	0.32241	1	ARRDC4	_
ENSG00000176533	0.755858	1.490894	0.32241	1	GNG7	K04543
ENSG00000175470	-0.737386	2.593053	0.322495	1	PPP2R2D	K04354
ENSG00000116299	0.730371	2.552922	0.322495	1	KIAA1324	_
ENSG00000167658	0.649145	11.44443	0.322506	1	EEF2	K03234
ENSG00000071054	-0.65241	6.688802	0.323269	1	MAP4K4	K04407
ENSG00000179455	-0.705659	3.09668	0.323305	1	MKRN3	K15687
ENSG00000171608	-0.6811	3.849065	0.323685	1	PIK3CD	_
ENSG00000164089	0.93853	0.566432	0.323905	1	ETNPPL	K14286
ENSG00000212670	0.93853	0.566432	0.323905	1	AL161915.1	K12581
ENSG00000242622	0.93853	0.566432	0.323905	1	RP11-18H7.1	_
ENSG00000155957	-0.673356	3.876057	0.324363	1	TMBIM4	K06890

ENSG00000162600	-0.657886	5.158836	0.324378	1	OMA1	_
ENSG00000187017	0.661151	4.28878	0.324427	1	ESPN	_
ENSG00000167972	0.720954	2.404108	0.324489	1	ABCA3	K05643
ENSG00000073756	-0.714575	2.45484	0.324489	1	PTGS2	K11987
ENSG00000152527	-1.192547	0.068768	0.324544	1	PLEKHH2	K10417
ENSG00000168961	-1.192547	0.068768	0.324544	1	LGALS9	K10093
ENSG00000204805	-1.192547	0.068768	0.324544	1	FAM27E4	_
ENSG00000271738	-1.192547	0.068768	0.324544	1	RP11-137H2.6	_
ENSG00000272009	-1.192547	0.068768	0.324544	1	RP1-313I6.12	_
ENSG00000272755	-1.192547	0.068768	0.324544	1	RP11-326G21.1	_
ENSG00000132274	-1.115247	0.018705	0.324544	1	TRIM22	K04257
ENSG00000204536	-1.115247	0.018705	0.324544	1	CCHCR1	K16760
ENSG00000224786	-1.115247	0.018705	0.324544	1	CETN4P	_
ENSG00000235927	-1.115247	0.018705	0.324544	1	NEXN-AS1	_
ENSG00000237773	-1.115247	0.018705	0.324544	1	AC003075.4	_
ENSG00000267473	-1.115247	0.018705	0.324544	1	AC005789.11	_
ENSG00000273226	-1.115247	0.018705	0.324544	1	RP11-513M16.8	_
ENSG00000179284	1.025226	-0.119387	0.324544	1	DAND5	K12581
ENSG00000197358	1.025226	-0.119387	0.324544	1	BNIP3P1	K15464
ENSG00000224023	1.025226	-0.119387	0.324544	1	RP11-383C5.4	_
ENSG00000228084	1.025226	-0.119387	0.324544	1	RP5-884G6.2	_
ENSG00000243885	1.025226	-0.119387	0.324544	1	RP11-278L15.2	_
ENSG00000251359	1.025226	-0.119387	0.324544	1	WWC2-AS2	_
ENSG00000260273	1.025226	-0.119387	0.324544	1	RP11-425D10.10	_
ENSG00000260597	1.025226	-0.119387	0.324544	1	AC012531.25	_
ENSG00000178826	-0.66384	4.824148	0.324691	1	TMEM139	_
ENSG00000135525	0.648994	6.968953	0.324888	1	MAP7	K10433
ENSG00000256073	0.727859	2.200136	0.324905	1	C21orf119	_
ENSG00000122481	-0.676286	4.055248	0.325177	1	RWDD3	_
ENSG00000132326	0.730162	1.929209	0.325362	1	PER2	K02633
ENSG00000141965	0.730162	1.929209	0.325362	1	FEM1A	_
ENSG00000128346	-0.723203	2.69793	0.325652	1	C22orf23	_
ENSG00000160145	0.693068	2.661877	0.325652	1	KALRN	K15048
ENSG00000268879	0.773045	1.579728	0.325787	1	IGFL1P1	_
ENSG00000227543	-0.863593	1.228451	0.325858	1	SPAG5-AS1	_
ENSG00000179630	-0.719811	2.899537	0.326475	1	LACC1	K05810
ENSG00000091127	-0.651973	5.763833	0.326527	1	PUS7	K06176
ENSG00000163171	-0.656387	4.851651	0.326842	1	CDC42EP3	_
ENSG00000215256	-0.751974	2.295015	0.3269	1	DHRS4-AS1	_
ENSG00000117298	0.64543	7.377744	0.326948	1	ECE1	K01415
ENSG00000163346	0.649431	6.130514	0.32709	1	PBXIP1	K13273
ENSG00000174501	0.744097	1.995686	0.327732	1	ANKRD36C	K17299
ENSG00000247095	0.744097	1.995686	0.327732	1	MIR210HG	_
ENSG00000164715	0.656163	4.341815	0.327942	1	LMTK2	K08898
ENSG00000233016	0.646117	6.300296	0.327965	1	SNHG7	_
ENSG00000167220	-0.672398	3.996103	0.328063	1	HDHD2	_
ENSG00000172530	0.652324	5.313086	0.328249	1	BANP	_
ENSG00000136720	0.651903	5.022382	0.328378	1	HS6ST1	K02514
ENSG00000243829	0.694481	2.74915	0.328433	1	CTB-33G10.1	K02997
ENSG00000066557	-0.647011	6.174921	0.32884	1	LRRC40	_
ENSG00000166938	-0.655083	4.691597	0.329271	1	DIS3L	K18681
ENSG00000228998	-0.708793	2.613901	0.329322	1	RP11-697E2.7	K10136
ENSG00000116353	-0.66846	3.873017	0.329491	1	MECR	K07512
ENSG00000198298	-0.724798	2.122607	0.329874	1	ZNF485	K09228
ENSG00000144815	-0.68675	3.19052	0.330151	1	NXPE3	_
ENSG00000166987	0.651914	4.768576	0.330308	1	MBD6	_
ENSG00000230074	-0.872723	0.861179	0.330406	1	RP11-195F19.9	_
ENSG00000078124	0.872624	0.759786	0.330406	1	ACER3	K04711

ENSG00000225663	0.872624	0.759786	0.330406	1	FAM195B	_
ENSG00000260266	0.872624	0.759786	0.330406	1	CTD-2311M21.2	K03767
ENSG00000170442	-0.733335	2.423006	0.330424	1	KRT86	K07605
ENSG00000108846	-0.646944	5.614745	0.330513	1	ABCC3	K05667
ENSG00000170088	-0.662249	4.004205	0.330701	1	TMEM192	K12581
ENSG00000184178	-0.727691	2.663812	0.330723	1	SCFD2	_
ENSG00000169105	-0.662939	4.391656	0.330834	1	CHST14	K08105
ENSG00000185000	0.687893	3.189984	0.331082	1	DGAT1	K11155
ENSG00000169126	-0.766052	1.845856	0.331331	1	ARMC4	_
ENSG00000144468	-0.697206	3.091364	0.332007	1	RHBDD1	K09651
ENSG00000081087	-0.648075	5.12036	0.333176	1	OSTM1	_
ENSG00000224543	-0.80576	1.485673	0.333198	1	SNRPGP15	K11099
ENSG00000260231	0.790565	1.425784	0.333198	1	JHDM1D-AS1	_
ENSG00000267530	-0.704149	2.757072	0.333266	1	AC006273.5	_
ENSG00000131849	0.702841	2.210617	0.333593	1	ZNF132	K09228
ENSG00000078687	1.583971	-0.771526	0.333681	1	TNRC6C	K18412
ENSG00000134940	1.583971	-0.771526	0.333681	1	ACRV1	_
ENSG00000139626	1.583971	-0.771526	0.333681	1	ITGB7	K06590
ENSG00000143479	1.583971	-0.771526	0.333681	1	DYRK3	K18669
ENSG00000168026	1.583971	-0.771526	0.333681	1	TTC21A	_
ENSG00000174945	1.583971	-0.771526	0.333681	1	AMZ1	K06974
ENSG00000185958	1.583971	-0.771526	0.333681	1	FAM186A	_
ENSG00000227288	1.583971	-0.771526	0.333681	1	RP5-837I24.1	_
ENSG00000229348	1.583971	-0.771526	0.333681	1	HYI-AS1	_
ENSG00000232811	1.583971	-0.771526	0.333681	1	RP11-96K19.2	_
ENSG00000234383	1.583971	-0.771526	0.333681	1	CTBP2P8	K04496
ENSG00000236537	1.583971	-0.771526	0.333681	1	RP11-732M18.3	_
ENSG00000247728	1.583971	-0.771526	0.333681	1	RP11-932O9.7	_
ENSG00000247809	1.583971	-0.771526	0.333681	1	NR2F2-AS1	_
ENSG00000249700	1.583971	-0.771526	0.333681	1	SRD5A3-AS1	_
ENSG00000253327	1.583971	-0.771526	0.333681	1	RAD21-AS1	_
ENSG00000258824	1.583971	-0.771526	0.333681	1	CTD-2555O16.2	_
ENSG00000265478	1.583971	-0.771526	0.333681	1	CTD-2145A24.3	_
ENSG00000273113	1.583971	-0.771526	0.333681	1	RP11-367H1.1	_
ENSG00000136514	-1.384585	-0.584421	0.333681	1	RTP4	_
ENSG00000146904	-1.384585	-0.584421	0.333681	1	EPHA1	K05102
ENSG00000151687	-1.384585	-0.584421	0.333681	1	ANKAR	_
ENSG00000162746	-1.384585	-0.584421	0.333681	1	FCRLB	_
ENSG00000167355	-1.384585	-0.584421	0.333681	1	AC104389.28	_
ENSG00000169129	-1.384585	-0.584421	0.333681	1	AFAP1L2	_
ENSG00000196550	-1.384585	-0.584421	0.333681	1	FAM72A	_
ENSG00000197332	-1.384585	-0.584421	0.333681	1	ZNF833P	_
ENSG00000212734	-1.384585	-0.584421	0.333681	1	C17orf100	K15153
ENSG00000213402	-1.384585	-0.584421	0.333681	1	PTPRCAP	K13886
ENSG00000224843	-1.384585	-0.584421	0.333681	1	LINC00240	_
ENSG00000231084	-1.384585	-0.584421	0.333681	1	CTA-253N17.1	K02891
ENSG00000235100	-1.384585	-0.584421	0.333681	1	RP11-80H5.9	_
ENSG00000238334	-1.384585	-0.584421	0.333681	1	snoU13	_
ENSG00000254602	-1.384585	-0.584421	0.333681	1	AP000662.4	_
ENSG00000258701	-1.384585	-0.584421	0.333681	1	LINC00638	_
ENSG00000259803	-1.384585	-0.584421	0.333681	1	SLC22A31	_
ENSG00000267648	-1.384585	-0.584421	0.333681	1	RP11-686D22.3	_
ENSG00000100156	1.273731	-0.679119	0.333681	1	SLC16A8	K08185
ENSG00000129270	1.273731	-0.679119	0.333681	1	MMP28	K08006
ENSG00000145087	1.273731	-0.679119	0.333681	1	STXBP5L	K08518
ENSG00000145949	1.273731	-0.679119	0.333681	1	MYLK4	K00907
ENSG00000160862	1.273731	-0.679119	0.333681	1	AZGP1	_
ENSG00000172752	1.273731	-0.679119	0.333681	1	COL6A5	K06238

ENSG00000197380	1.273731	-0.679119	0.333681	1	DACT3	_
ENSG00000229539	1.273731	-0.679119	0.333681	1	RP11-119B16.2	_
ENSG00000233631	1.273731	-0.679119	0.333681	1	RP11-457M11.2	_
ENSG00000235454	1.273731	-0.679119	0.333681	1	RP11-678B3.2	K16589
ENSG00000238975	1.273731	-0.679119	0.333681	1	snoU13	_
ENSG00000245975	1.273731	-0.679119	0.333681	1	RP11-30K9.6	_
ENSG00000250602	1.273731	-0.679119	0.333681	1	RP11-517I3.1	_
ENSG00000253667	1.273731	-0.679119	0.333681	1	RP11-30L15.4	_
ENSG00000254812	1.273731	-0.679119	0.333681	1	RP11-661A12.12	_
ENSG00000259182	1.273731	-0.679119	0.333681	1	RP11-424I19.2	_
ENSG00000259424	1.273731	-0.679119	0.333681	1	RP11-35O15.1	_
ENSG00000261349	1.273731	-0.679119	0.333681	1	RP3-465N24.5	_
ENSG00000267231	1.273731	-0.679119	0.333681	1	AC005786.5	_
ENSG00000268201	1.273731	-0.679119	0.333681	1	CTD-3138B18.6	_
ENSG00000272800	1.273731	-0.679119	0.333681	1	RP11-438L19.1	_
ENSG00000139800	-0.643223	5.358567	0.334119	1	ZIC5	K09226
ENSG00000221955	0.652325	4.440308	0.334332	1	SLC12A8	K14428
ENSG00000236081	-0.671559	3.349165	0.334427	1	AC074389.9	_
ENSG00000178605	-0.649954	4.468679	0.334686	1	GTPBP6	_
ENSG00000171552	-0.639088	5.911371	0.334736	1	BCL2L1	K04570
ENSG00000188878	-0.694226	3.033567	0.335083	1	FBF1	K16471
ENSG00000138385	-0.63484	7.39235	0.335115	1	SSB	K11090
ENSG00000140691	-0.676633	3.374821	0.33518	1	ARMC5	_
ENSG00000114854	-0.716264	2.320445	0.335217	1	TNNC1	K05865
ENSG00000068650	0.63599	6.392157	0.335237	1	ATP11A	_
ENSG00000157429	-0.881738	1.002749	0.335401	1	ZNF19	K09228
ENSG00000267858	-0.881738	1.002749	0.335401	1	AC016629.8	_
ENSG00000264920	0.822584	0.930337	0.335401	1	RP11-6N17.4	_
ENSG00000182141	-0.739289	2.01821	0.335692	1	ZNF708	K09228
ENSG00000092200	-1.149847	0.308096	0.335879	1	RPGRIP1	K16512
ENSG00000112902	-1.086193	0.266327	0.335879	1	SEMA5A	K06841
ENSG00000213569	-1.086193	0.266327	0.335879	1	RP13-383K5.4	K15203
ENSG00000214212	-1.086193	0.266327	0.335879	1	C19orf38	_
ENSG00000249863	-1.086193	0.266327	0.335879	1	RP11-177C12.1	_
ENSG00000114251	-0.639094	5.985024	0.33591	1	WNT5A	K00444
ENSG00000236499	0.764721	1.535972	0.336064	1	LINC00896	_
ENSG00000141458	0.634418	6.691813	0.336309	1	NPC1	K12385
ENSG00000112081	-0.631267	8.488794	0.336745	1	SRSF3	K12892
ENSG00000165006	0.633919	6.437513	0.336791	1	UBAP1	_
ENSG00000165898	-0.653588	4.05503	0.336837	1	ISCA2	_
ENSG00000172954	-0.659833	3.851851	0.33708	1	LCLAT1	K13513
ENSG00000139133	-0.684524	2.939277	0.337519	1	ALG10	K03850
ENSG00000124107	0.631429	7.30861	0.337569	1	SLPI	_
ENSG00000172493	0.635001	6.228761	0.337783	1	AFF1	K15184
ENSG00000158234	-0.643967	4.684761	0.338193	1	FAIM	_
ENSG00000104765	0.633982	6.163375	0.338449	1	BNIP3L	K15465
ENSG00000138293	0.670802	3.332221	0.338524	1	NCOA4	K09289
ENSG00000120686	-0.633021	6.31336	0.33861	1	UFM1	K12162
ENSG00000137124	-0.63638	5.728819	0.338778	1	ALDH1B1	K00128
ENSG00000160469	0.783274	1.082897	0.339352	1	BRSK1	K08796
ENSG00000243679	-0.70976	2.494239	0.339362	1	RP11-274B21.3	_
ENSG00000099994	-0.637797	5.146508	0.339421	1	SUSD2	_
ENSG00000174405	-0.656486	3.957017	0.339434	1	LIG4	K10777
ENSG00000204634	0.633446	5.822306	0.339494	1	TBC1D8	_
ENSG00000234420	-0.673942	3.049095	0.340145	1	ZNF37BP	K09228
ENSG00000092295	-0.697016	2.8204	0.340336	1	TGM1	K05619
ENSG00000168071	0.656486	3.736734	0.34061	1	CCDC88B	_
ENSG00000111261	-0.776705	1.783484	0.340697	1	MANSC1	K09488

ENSG00000231500	0.75886	1.718941	0.340697	1	RPS18	K02964
ENSG00000178297	0.696207	2.18229	0.340729	1	TMPRSS9	K09640
ENSG00000095015	-0.659563	3.646937	0.341333	1	MAP3K1	K04416
ENSG00000111669	0.709222	2.238412	0.342113	1	TPI1	K01803
ENSG00000218418	0.709222	2.238412	0.342113	1	RP11-296E7.1	_
ENSG00000175197	0.637494	4.56822	0.34232	1	DDIT3	K04452
ENSG00000059804	0.623385	8.719382	0.342535	1	SLC2A3	K08142
ENSG00000166153	-0.811375	1.299814	0.342553	1	DEPDC4	_
ENSG00000142677	0.803714	1.198857	0.342553	1	IL22RA1	K05138
ENSG00000082512	-0.649713	4.106711	0.342608	1	TRAF5	K09849
ENSG00000122497	0.683035	2.620053	0.342671	1	NBPF14	_
ENSG00000147642	-0.659662	3.718532	0.342799	1	SYBU	_
ENSG00000072201	-0.900847	0.560759	0.343208	1	LNX1	K10692
ENSG00000164251	-0.900847	0.560759	0.343208	1	F2RL1	K04234
ENSG00000232807	-0.900847	0.560759	0.343208	1	RP11-536K7.3	_
ENSG00000233868	-0.900847	0.560759	0.343208	1	AC009302.2	_
ENSG00000259562	-0.900847	0.560759	0.343208	1	RP11-762H8.2	_
ENSG00000271888	-0.900847	0.560759	0.343208	1	RP11-560J1.2	_
ENSG00000196227	-0.646556	3.890583	0.343281	1	FAM217B	_
ENSG00000103404	-0.669872	2.989509	0.343714	1	USP31	K11852
ENSG00000109536	-0.747455	1.963024	0.344281	1	FRG1	K13122
ENSG00000171798	0.722687	1.894821	0.344281	1	KNDC1	_
ENSG00000071243	-0.672244	3.255932	0.34436	1	ING3	K11319
ENSG00000142798	0.62318	6.928256	0.344451	1	HSPG2	K06255
ENSG00000256745	0.685096	2.70978	0.344529	1	RP11-680H20.1	K05756
ENSG00000141574	0.624648	6.361428	0.345075	1	SECTM1	_
ENSG00000228106	-0.644178	4.24232	0.345221	1	RP11-452F19.3	_
ENSG00000164976	0.634129	4.80213	0.345264	1	KIAA1161	_
ENSG00000171631	0.626509	5.275	0.345349	1	P2RY6	K04272
ENSG00000105404	0.660994	2.763089	0.345374	1	RABAC1	_
ENSG00000104885	0.624798	6.117215	0.345403	1	DOT1L	K11427
ENSG00000239665	0.677993	2.422072	0.345619	1	RP11-295P9.3	_
ENSG00000156253	-0.628499	5.482099	0.346037	1	RWDD2B	K09513
ENSG00000163170	-0.631543	5.110236	0.346662	1	BOLA3	_
ENSG00000131759	0.630539	4.722773	0.346743	1	RARA	K08527
ENSG00000176788	0.617266	10.45783	0.346758	1	BASP1	K17272
ENSG00000011028	-0.64194	4.216643	0.346877	1	MRC2	K06560
ENSG00000141968	-0.643379	3.618464	0.34714	1	VAV1	K05730
ENSG00000052802	-0.623255	6.017819	0.347157	1	MSMO1	K07750
ENSG00000118181	-0.739531	1.529139	0.347413	1	RPS25	K02975
ENSG00000173928	-0.739531	1.529139	0.347413	1	SWSAP1	K17985
ENSG00000234147	-0.700912	2.569944	0.347535	1	RP3-460G2.2	_
ENSG00000159593	-0.620139	6.660233	0.347695	1	NAE1	K04532
ENSG00000090020	0.638915	4.252515	0.347704	1	SLC9A1	K05742
ENSG00000179855	0.626689	5.181153	0.347985	1	GIPC3	_
ENSG00000198753	0.94457	0.649652	0.348276	1	PLXNB3	K06821
ENSG00000244242	0.94457	0.649652	0.348276	1	IFITM10	_
ENSG00000241014	-0.908444	0.73406	0.348276	1	RP11-244H3.1	_
ENSG00000241316	-0.908444	0.73406	0.348276	1	RP11-81N13.1	_
ENSG00000125744	0.628006	5.07165	0.348527	1	RTN2	_
ENSG00000007264	-0.620999	6.081206	0.348795	1	MATK	K17692
ENSG00000138756	-0.6893	2.676526	0.349193	1	BMP2K	K08854
ENSG00000105708	0.688124	2.641113	0.349193	1	ZNF14	K09228
ENSG00000035499	-0.635404	4.374699	0.34944	1	DEPDC1B	_
ENSG00000026950	-0.624262	5.003818	0.349764	1	BTN3A1	K06712
ENSG00000160190	-0.632103	4.538012	0.350099	1	SLC37A1	K13783
ENSG00000148459	-0.632302	4.305554	0.350357	1	PDSS1	K12504
ENSG00000241990	0.691584	2.275677	0.350467	1	RP6-109B7.3	_

ENSG00000236943	-0.67931	2.775764	0.350643	1	RP11-640M9.1	_
ENSG00000136379	0.616111	6.50729	0.350726	1	ABHD17C	_
ENSG00000138035	-0.616571	6.434138	0.350989	1	PNPT1	K00962
ENSG00000177989	-0.641978	3.724936	0.351916	1	ODF3B	_
ENSG00000173338	0.881335	0.833024	0.351957	1	KCNK7	K04918
ENSG00000156711	0.811518	0.861948	0.351957	1	MAPK13	K04441
ENSG00000245105	0.811518	0.861948	0.351957	1	A2M-AS1	_
ENSG00000177169	0.612653	7.331643	0.352011	1	ULK1	K08269
ENSG00000164631	0.619998	5.318633	0.352138	1	ZNF12	K09228
ENSG00000105849	-0.618932	5.768689	0.352151	1	TWISTNB	K03004
ENSG00000105711	0.693516	2.388479	0.352237	1	SCN1B	K04845
ENSG00000229152	0.693516	2.388479	0.352237	1	ANKRD10-IT1	_
ENSG00000118257	-0.745344	1.832593	0.352333	1	NRP2	K06819
ENSG00000180178	0.731722	1.772222	0.352333	1	FAR2P1	K09228
ENSG00000135148	0.613334	6.566946	0.352428	1	TRAFD1	_
ENSG00000122591	-0.616183	6.133857	0.352518	1	FAM126A	_
ENSG00000159267	-0.640607	3.976445	0.352521	1	HLCS	K01942
ENSG00000161180	-1.352417	-0.160613	0.352672	1	CCDC116	_
ENSG00000167895	-1.352417	-0.160613	0.352672	1	TMC8	_
ENSG00000213888	-1.352417	-0.160613	0.352672	1	AC005003.1	K09228
ENSG00000231177	-1.352417	-0.160613	0.352672	1	LINC00852	_
ENSG00000265452	-1.352417	-0.160613	0.352672	1	MIR3682	_
ENSG00000108854	-1.260308	-0.220081	0.352672	1	SMURF2	K04678
ENSG00000136883	-1.260308	-0.220081	0.352672	1	KIF12	K10399
ENSG00000151882	-1.260308	-0.220081	0.352672	1	CCL28	K05513
ENSG00000170965	-1.260308	-0.220081	0.352672	1	PLAC1	_
ENSG00000181085	-1.260308	-0.220081	0.352672	1	MAPK15	K19603
ENSG00000229474	-1.260308	-0.220081	0.352672	1	PATL2	_
ENSG00000233203	-1.260308	-0.220081	0.352672	1	RP11-67L3.4	_
ENSG00000250802	-1.260308	-0.220081	0.352672	1	ZBED3-AS1	_
ENSG00000251095	-1.260308	-0.220081	0.352672	1	RP11-115D19.1	_
ENSG00000267896	-1.260308	-0.220081	0.352672	1	AC018766.4	_
ENSG00000268166	-1.260308	-0.220081	0.352672	1	CTC-471F3.6	_
ENSG00000062524	1.211955	-0.328686	0.352672	1	LTK	K05118
ENSG00000078900	1.211955	-0.328686	0.352672	1	TP73	K10148
ENSG00000105982	1.211955	-0.328686	0.352672	1	RNF32	_
ENSG00000117643	1.211955	-0.328686	0.352672	1	MAN1C1	K01230
ENSG00000142920	1.211955	-0.328686	0.352672	1	ADC	K01583
ENSG00000170835	1.211955	-0.328686	0.352672	1	CEL	K12298
ENSG00000185710	1.211955	-0.328686	0.352672	1	RP11-645C24.2	K08873
ENSG00000205978	1.211955	-0.328686	0.352672	1	NYNRIN	_
ENSG00000212829	1.211955	-0.328686	0.352672	1	RPS26P3	K02976
ENSG00000214725	1.211955	-0.328686	0.352672	1	CDIPT-AS1	_
ENSG00000215835	1.211955	-0.328686	0.352672	1	RP11-9L18.2	K13209
ENSG00000225693	1.211955	-0.328686	0.352672	1	RP11-54K16.2	K15902
ENSG00000242474	1.211955	-0.328686	0.352672	1	AC093627.9	_
ENSG00000248712	1.211955	-0.328686	0.352672	1	CCDC153	_
ENSG00000272037	1.211955	-0.328686	0.352672	1	KB-431C1.5	_
ENSG00000273472	1.211955	-0.328686	0.352672	1	RP11-102N12.3	_
ENSG00000119630	1.023546	-0.261284	0.352672	1	PGF	K16859
ENSG00000135929	1.023546	-0.261284	0.352672	1	CYP27A1	K00488
ENSG00000179362	1.023546	-0.261284	0.352672	1	HMGN2P46	K11300
ENSG00000180616	1.023546	-0.261284	0.352672	1	SSTR2	K04218
ENSG00000187726	1.023546	-0.261284	0.352672	1	DNAJB13	K09519
ENSG00000204869	1.023546	-0.261284	0.352672	1	IGFL4	_
ENSG00000219682	1.023546	-0.261284	0.352672	1	RP3-425P12.4	K14295
ENSG00000230526	1.023546	-0.261284	0.352672	1	RP11-472G21.2	_
ENSG00000231131	1.023546	-0.261284	0.352672	1	RP11-346D6.6	_

ENSG00000232450	1.023546	-0.261284	0.352672	1	RP4-730K3.3	K02981
ENSG00000232775	1.023546	-0.261284	0.352672	1	AP000525.10	K14569
ENSG00000236345	1.023546	-0.261284	0.352672	1	RP11-59D5__B.2	_
ENSG00000241278	1.023546	-0.261284	0.352672	1	ENPP7P4	K12354
ENSG00000266927	1.023546	-0.261284	0.352672	1	AC006273.7	_
ENSG00000270006	1.023546	-0.261284	0.352672	1	RP11-178L8.7	_
ENSG00000168404	-0.627761	4.611415	0.352781	1	MLKL	K08849
ENSG00000112773	0.612575	6.518355	0.353082	1	FAM46A	_
ENSG00000153574	-0.62784	4.760887	0.353192	1	RPIA	K01807
ENSG00000105339	0.613565	6.018884	0.353456	1	DENND3	_
ENSG00000091592	-0.736489	1.892942	0.353571	1	NLRP1	K11447
ENSG00000013563	0.675044	2.501607	0.353741	1	DNASE1L1	K11995
ENSG00000159055	-0.627267	4.817829	0.353904	1	MIS18A	K11564
ENSG00000125821	-0.618793	4.828616	0.354039	1	DTD1	K07560
ENSG00000145715	-0.619893	5.042812	0.354044	1	RASA1	K04352
ENSG00000271529	0.699896	1.942019	0.354665	1	CICP14	K16941
ENSG00000197157	0.607971	8.347903	0.354731	1	SND1	K15979
ENSG00000230457	-0.818982	1.085816	0.354733	1	PA2G4P4	_
ENSG00000206567	0.771657	1.021373	0.354733	1	AC022007.5	_
ENSG00000169957	-0.615742	5.250098	0.355357	1	ZNF768	K09228
ENSG00000255624	-0.714096	2.060122	0.355639	1	RP11-564D11.3	_
ENSG00000255717	-0.608241	7.304189	0.355754	1	SNHG1	_
ENSG00000171681	-0.631773	4.041592	0.355833	1	ATF7IP	_
ENSG00000198947	-0.611543	5.894603	0.355855	1	DMD	K10366
ENSG00000197982	-0.616921	5.101317	0.355942	1	C1orf122	_
ENSG00000124839	0.623441	4.044657	0.356041	1	RAB17	K07909
ENSG00000221886	0.6393	3.520541	0.356144	1	C5orf54	_
ENSG00000136319	-0.660793	3.199573	0.356189	1	TTC5	_
ENSG00000152256	0.617995	5.022462	0.356388	1	PDK1	K12077
ENSG00000141854	0.701508	2.082485	0.356511	1	hsa-mir-1199	_
ENSG00000254685	-0.627373	4.325297	0.356684	1	FPGT	K00976
ENSG00000143384	-0.605244	8.725838	0.356757	1	MCL1	K02539
ENSG00000175727	0.60896	6.262313	0.356846	1	MLXIP	K09113
ENSG00000010404	-0.831294	1.207475	0.356891	1	IDS	K01136
ENSG00000063601	-0.831294	1.207475	0.356891	1	MTMR1	K18081
ENSG00000146666	-0.831294	1.207475	0.356891	1	LINC00525	_
ENSG00000162894	-0.831294	1.207475	0.356891	1	FAIM3	_
ENSG00000238045	-0.831294	1.207475	0.356891	1	AC009133.14	_
ENSG00000261051	0.793921	1.141998	0.356891	1	RP11-274H2.5	_
ENSG00000257365	-0.69642	2.209862	0.357297	1	FNTB	K05954
ENSG00000123999	0.663845	2.164252	0.357297	1	INHA	K05500
ENSG00000214193	0.638108	3.302015	0.357558	1	SH3D21	_
ENSG00000143061	-0.607675	6.402461	0.357732	1	IGSF3	K06522
ENSG00000115252	0.610409	5.27388	0.357803	1	PDE1A	K13755
ENSG00000215114	-0.624344	4.442602	0.357894	1	UBXN2B	K14012
ENSG00000075624	-0.602892	11.5825	0.357919	1	ACTB	K05692
ENSG00000146776	-0.706729	2.266234	0.358009	1	ATXN7L1	K11318
ENSG00000162545	0.652081	2.927939	0.358416	1	CAMK2N1	_
ENSG00000179218	0.602308	10.77421	0.358443	1	CALR	K08057
ENSG00000141985	0.605592	6.710309	0.358566	1	SH3GL1	K11247
ENSG00000213462	0.762424	1.27481	0.358608	1	ERV3-1	K09228
ENSG00000114200	-0.735371	1.348956	0.358608	1	BCHE	K01050
ENSG00000225518	-0.735371	1.348956	0.358608	1	RP11-396C23.2	_
ENSG00000248092	-0.735371	1.348956	0.358608	1	NNT-AS1	_
ENSG00000030582	0.602362	9.320615	0.358702	1	GRN	_
ENSG00000148200	0.643398	3.453899	0.358972	1	NR6A1	K08561
ENSG00000155034	0.633286	3.458076	0.358972	1	FBXL18	K10284
ENSG00000204147	-0.656259	3.038064	0.35915	1	ASAH2B	K12349

ENSG00000166261	-0.645018	3.503486	0.359222	1	ZNF202	K09229
ENSG00000121931	-0.662889	3.069889	0.359489	1	LRIF1	_
ENSG00000118418	-0.604816	6.510513	0.359669	1	HMGN3	K11301
ENSG00000164855	0.614445	4.996534	0.35991	1	TMEM184A	_
ENSG00000186350	0.606413	5.725857	0.360071	1	RXRA	K08524
ENSG00000081377	-0.625972	3.981843	0.360275	1	CDC14B	K06639
ENSG00000189042	-0.679716	2.516522	0.360288	1	ZNF567	K09228
ENSG00000198363	0.59975	9.543345	0.360708	1	ASPH	K00476
ENSG00000114473	-0.688546	2.56215	0.360748	1	IQCG	_
ENSG00000244026	-0.688546	2.56215	0.360748	1	FAM86DP	_
ENSG00000091317	-0.610015	4.959481	0.360841	1	CMTM6	_
ENSG00000119138	0.602451	6.304636	0.360913	1	KLF9	K09208
ENSG00000141867	0.607323	5.744843	0.360997	1	BRD4	K11722
ENSG00000037965	0.71424	1.50863	0.361157	1	HOXC8	K09308
ENSG00000141012	0.607355	5.486933	0.361579	1	GALNS	K01132
ENSG00000105357	1.026574	0.010275	0.361697	1	MYH14	K10352
ENSG00000117242	1.026574	0.010275	0.361697	1	PINK1-AS	_
ENSG00000222937	1.026574	0.010275	0.361697	1	SNORD63	_
ENSG00000224846	1.026574	0.010275	0.361697	1	RP1-90J20.8	_
ENSG00000229808	1.026574	0.010275	0.361697	1	RP11-456P18.2	K06698
ENSG00000266235	1.026574	0.010275	0.361697	1	MIR3176	_
ENSG00000149922	0.891241	0.063029	0.361697	1	TBX6	K10180
ENSG00000204529	0.891241	0.063029	0.361697	1	GUCY2EP	K12322
ENSG00000268856	0.891241	0.063029	0.361697	1	AP001579.1	_
ENSG00000184985	0.62597	3.823473	0.361942	1	SORCS2	_
ENSG00000159231	-0.627801	3.863555	0.362093	1	CBR3	K00084
ENSG00000163328	0.733698	1.596342	0.362123	1	GPR155	_
ENSG00000244171	-0.621166	4.228196	0.362175	1	PBX2P1	K15609
ENSG00000182551	-0.602044	6.297407	0.362399	1	ADI1	K08967
ENSG00000144355	-0.634598	3.456207	0.362507	1	DLX1	K09314
ENSG00000140263	-0.602869	6.151222	0.362527	1	SORD	K00008
ENSG00000128617	0.644537	2.770003	0.362605	1	OPN1SW	K04252
ENSG00000140297	0.644537	2.770003	0.362605	1	GCNT3	K09662
ENSG00000130518	-0.755064	1.769595	0.362944	1	KIAA1683	_
ENSG00000159885	-0.755064	1.769595	0.362944	1	ZNF222	K09228
ENSG00000223396	-0.755064	1.769595	0.362944	1	RPS10P7	_
ENSG00000111850	-0.658138	2.892127	0.36319	1	SMIM8	_
ENSG00000101928	0.599999	6.599919	0.3634	1	MOSPD1	_
ENSG00000185928	-0.651396	2.97799	0.363707	1	PAGR1	K14973
ENSG00000115392	-0.604835	5.410888	0.363857	1	FANCL	K10606
ENSG00000143437	0.601656	5.977585	0.363953	1	ARNT	K09097
ENSG00000104325	-0.606932	5.082056	0.364183	1	DECR1	K13236
ENSG00000113721	0.635995	3.096772	0.364583	1	PDGFRB	K05089
ENSG00000171105	0.606492	5.181183	0.364746	1	INSR	K04527
ENSG00000163536	0.615904	3.878406	0.365051	1	SERPINI1	_
ENSG00000182378	0.600003	6.116527	0.365291	1	PLCXD1	_
ENSG00000146409	-0.611457	4.424296	0.365331	1	SLC18B1	_
ENSG00000170485	-0.611869	4.808438	0.365466	1	NPAS2	K09026
ENSG00000164070	-0.605941	5.341385	0.365491	1	HSPA4L	K09485
ENSG00000039560	-0.603761	5.394343	0.36573	1	RAI14	_
ENSG00000165115	-0.671488	2.29218	0.366382	1	KIF27	K10395
ENSG00000212994	0.661262	2.258698	0.366382	1	RPS26P6	K02976
ENSG00000129993	0.62326	3.501544	0.366496	1	CBFA2T3	K10053
ENSG00000245532	0.592225	10.25214	0.366567	1	NEAT1	_
ENSG00000164509	-0.951655	0.40193	0.366588	1	IL31RA	K08857
ENSG00000171872	-0.951655	0.40193	0.366588	1	KLF17	K09211
ENSG00000227199	-0.951655	0.40193	0.366588	1	ST7-AS1	_
ENSG00000246985	-0.951655	0.40193	0.366588	1	SOCS2-AS1	_

ENSG00000132881	0.91466	0.284949	0.366588	1	RSG1	_
ENSG00000182580	0.91466	0.284949	0.366588	1	EPHB3	K05112
ENSG00000185055	0.91466	0.284949	0.366588	1	EFCAB10	_
ENSG00000198598	0.91466	0.284949	0.366588	1	MMP17	K07997
ENSG00000260778	0.91466	0.284949	0.366588	1	MIR940	_
ENSG00000267270	0.91466	0.284949	0.366588	1	AC139100.2	_
ENSG00000157600	0.596608	5.98662	0.366602	1	TMEM164	_
ENSG00000105483	-0.62454	3.625307	0.366905	1	CARD8	K12801
ENSG00000173145	-0.599114	5.928051	0.367037	1	NOC3L	K14834
ENSG00000140332	0.594087	7.068752	0.367088	1	TLE3	K04497
ENSG00000231365	-0.627364	3.681033	0.367088	1	RP11-418J17.1	_
ENSG00000167700	-0.599036	5.756625	0.36716	1	MFSD3	_
ENSG00000104976	0.609525	4.389064	0.367174	1	SNAPC2	K15209
ENSG00000139684	-0.631988	3.701434	0.367175	1	ESD	K01070
ENSG00000142556	0.656983	2.430959	0.367202	1	ZNF614	K09228
ENSG00000176845	0.593924	6.801006	0.367679	1	METRNL	_
ENSG00000123106	-0.604718	4.578715	0.367764	1	CCDC91	_
ENSG00000104689	-0.620419	3.904972	0.367842	1	TNFRSF10A	K04722
ENSG00000176994	0.611528	3.948439	0.368027	1	SMCR8	_
ENSG00000138686	-0.615393	4.003729	0.368142	1	BBS7	K16749
ENSG00000166311	0.598341	5.232388	0.368295	1	SMPD1	K12350
ENSG00000150347	0.597733	5.704083	0.36858	1	ARID5B	_
ENSG00000135972	-0.595748	6.021915	0.369282	1	MRPS9	K02996
ENSG00000253948	0.839553	0.515868	0.369464	1	RP11-410L14.2	_
ENSG00000260404	0.839553	0.515868	0.369464	1	RP11-384K6.6	_
ENSG00000272447	0.839553	0.515868	0.369464	1	RP11-182L21.6	_
ENSG00000085185	0.602779	4.722893	0.369772	1	BCORL1	_
ENSG00000106823	-0.622342	3.427787	0.369891	1	ECM2	K08119
ENSG00000002586	0.591965	6.29007	0.37031	1	CD99	K06520
ENSG00000198399	-0.613413	4.223455	0.370948	1	ITSN2	_
ENSG00000067601	-0.829869	0.833535	0.371253	1	PMS2P4	K10858
ENSG00000271936	-0.829869	0.833535	0.371253	1	RP11-443B20.1	_
ENSG00000272476	-0.829869	0.833535	0.371253	1	RP1-191J18.66	_
ENSG00000196741	-0.785704	0.805281	0.371253	1	CXorf24	_
ENSG00000205436	0.785594	0.715067	0.371253	1	EXOC3L4	_
ENSG00000034677	0.588114	7.452955	0.371369	1	RNF19A	K11972
ENSG00000186111	0.597222	5.114226	0.371548	1	PIP5K1C	K00889
ENSG00000082153	0.586478	8.587729	0.371748	1	BZW1	_
ENSG00000272655	-0.843435	0.977906	0.372408	1	POLR2J4	K03008
ENSG00000166575	-0.601169	4.667456	0.372936	1	TMEM135	_
ENSG00000100027	-2.044268	-0.866855	0.372947	1	YPEL1	_
ENSG00000132965	-2.044268	-0.866855	0.372947	1	ALOX5AP	_
ENSG00000137310	-2.044268	-0.866855	0.372947	1	TCF19	_
ENSG00000183690	-2.044268	-0.866855	0.372947	1	EFHC2	_
ENSG00000199753	-2.044268	-0.866855	0.372947	1	SNORD104	_
ENSG00000214770	-2.044268	-0.866855	0.372947	1	RP11-544I20.2	_
ENSG00000224725	-2.044268	-0.866855	0.372947	1	CEP57L1P1	K16762
ENSG00000229447	-2.044268	-0.866855	0.372947	1	RP11-490K7.4	K03123
ENSG00000232600	-2.044268	-0.866855	0.372947	1	AC084125.4	_
ENSG00000238123	-2.044268	-0.866855	0.372947	1	MID1IP1-AS1	_
ENSG00000254343	-2.044268	-0.866855	0.372947	1	RP11-760H22.2	_
ENSG00000261737	-2.044268	-0.866855	0.372947	1	RP4-612B15.3	_
ENSG00000270067	-2.044268	-0.866855	0.372947	1	CTC-487M23.5	_
ENSG00000273030	-2.044268	-0.866855	0.372947	1	RP11-285F16.1	_
ENSG00000101049	-1.877647	-0.968243	0.372947	1	SGK2	K13303
ENSG00000106006	-1.877647	-0.968243	0.372947	1	HOXA6	K09306
ENSG00000126861	-1.877647	-0.968243	0.372947	1	OMG	K16666
ENSG00000163376	-1.877647	-0.968243	0.372947	1	KBTBD8	K10475

ENSG00000168491	-1.877647	-0.968243	0.372947	1	CCDC110	_
ENSG00000172232	-1.877647	-0.968243	0.372947	1	AZU1	K01350
ENSG00000180113	-1.877647	-0.968243	0.372947	1	TDRD6	K18405
ENSG00000183395	-1.877647	-0.968243	0.372947	1	PMCH	K05229
ENSG00000186204	-1.877647	-0.968243	0.372947	1	CYP4F12	K17730
ENSG00000186652	-1.877647	-0.968243	0.372947	1	PRG2	K10786
ENSG00000188092	-1.877647	-0.968243	0.372947	1	GPR89B	_
ENSG00000201772	-1.877647	-0.968243	0.372947	1	SNORA5C	_
ENSG00000222881	-1.877647	-0.968243	0.372947	1	Y_RNA	_
ENSG00000223374	-1.877647	-0.968243	0.372947	1	AC005104.3	_
ENSG00000223508	-1.877647	-0.968243	0.372947	1	RPL23AP53	K02893
ENSG00000224854	-1.877647	-0.968243	0.372947	1	C9orf53	_
ENSG00000226141	-1.877647	-0.968243	0.372947	1	AF277315.13	_
ENSG00000229122	-1.877647	-0.968243	0.372947	1	AGBL5-IT1	_
ENSG00000233560	-1.877647	-0.968243	0.372947	1	KRT8P39	K07605
ENSG00000236066	-1.877647	-0.968243	0.372947	1	RP11-389O22.1	_
ENSG00000250155	-1.877647	-0.968243	0.372947	1	CTD-2353F22.1	_
ENSG00000250410	-1.877647	-0.968243	0.372947	1	RP11-714G18.1	_
ENSG00000254876	-1.877647	-0.968243	0.372947	1	RP11-23J9.5	_
ENSG00000255568	-1.877647	-0.968243	0.372947	1	BRWD1-IT2	_
ENSG00000255992	-1.877647	-0.968243	0.372947	1	RP11-417L19.4	_
ENSG00000258302	-1.877647	-0.968243	0.372947	1	RP11-981P6.1	_
ENSG00000260944	-1.877647	-0.968243	0.372947	1	RP11-463O9.5	_
ENSG00000261468	-1.877647	-0.968243	0.372947	1	RP11-1024P17.1	_
ENSG00000266441	-1.877647	-0.968243	0.372947	1	RP11-91I8.3	_
ENSG00000244879	0.60779	3.820595	0.373425	1	GABPB1-AS1	_
ENSG00000168807	0.590261	5.589119	0.373465	1	SNTB2	K12196
ENSG00000107819	0.586725	6.267401	0.373534	1	SFXN3	_
ENSG00000165046	-0.78046	1.279906	0.37369	1	LETM2	_
ENSG00000231822	-0.78046	1.279906	0.37369	1	AC019097.7	K06669
ENSG00000172785	-0.623465	3.407307	0.373749	1	CBWD1	_
ENSG00000251474	0.62317	3.29338	0.373823	1	RPL32P3	K02912
ENSG00000271870	0.772489	1.326846	0.374041	1	RP11-97C16.1	_
ENSG00000082014	0.627593	3.012496	0.374052	1	SMARCD3	K11650
ENSG00000174093	0.63746	2.934065	0.374117	1	RP11-1407O15.2	K08776
ENSG00000123178	-0.59504	4.978992	0.374127	1	SPRYD7	K11447
ENSG00000136854	0.586657	6.563606	0.374171	1	STXBP1	K15292
ENSG00000151470	-0.63003	2.84333	0.37422	1	C4orf33	_
ENSG00000180596	0.703546	1.462766	0.37428	1	HIST1H2BC	K11252
ENSG00000184702	0.703546	1.462766	0.37428	1	5-Sep	K04557
ENSG00000130940	0.640881	2.614245	0.374367	1	CASZ1	_
ENSG00000156398	-0.645342	2.61203	0.374404	1	SFXN2	K07944
ENSG00000213533	0.724271	1.55313	0.374441	1	TMEM110	_
ENSG00000205060	-0.599551	4.70986	0.374513	1	SLC35B4	K15278
ENSG00000147168	-0.683557	1.982846	0.374665	1	IL2RG	K05070
ENSG00000214226	-0.664491	1.97084	0.374665	1	C17orf67	_
ENSG00000177946	-0.696211	2.048791	0.374666	1	CENPBD1	_
ENSG00000065618	0.650125	1.999939	0.374666	1	COL17A1	K07603
ENSG00000118260	-0.606425	4.378715	0.37509	1	CREB1	K05870
ENSG00000185989	0.5842	7.04493	0.375112	1	RASA3	K12380
ENSG00000115756	0.585789	6.499923	0.375188	1	HPCAL1	K19695
ENSG00000196498	0.583596	7.110993	0.3755	1	NCOR2	K06065
ENSG00000116574	0.590883	4.915878	0.375583	1	RHOU	_
ENSG00000196776	-0.592739	5.408826	0.375613	1	CD47	K06266
ENSG00000116679	-0.583473	7.216447	0.375704	1	IVNS1ABP	K15046
ENSG00000107882	0.596488	4.028867	0.375809	1	SUFU	K06229
ENSG00000145246	-0.605151	4.025292	0.375854	1	ATP10D	K01530
ENSG00000105355	0.58229	7.655382	0.375872	1	PLIN3	_

ENSG00000234072	0.614775	3.687023	0.376702	1	AC074117.10	_
ENSG00000145022	0.610573	3.532444	0.377161	1	TCTA	K09228
ENSG00000065308	0.582766	6.310689	0.377198	1	TRAM2	K12581
ENSG00000179862	-0.582509	6.761389	0.377344	1	CITED4	_
ENSG00000115919	0.579768	8.281173	0.377398	1	KYNU	K01556
ENSG00000128165	-0.603248	4.276275	0.377454	1	ADM2	_
ENSG00000243566	-0.61453	3.443848	0.377523	1	UPK3B	_
ENSG00000096968	-0.615526	3.423595	0.377622	1	JAK2	K04447
ENSG00000161179	-0.584231	6.028653	0.377648	1	YDJC	_
ENSG00000108064	-0.582611	6.557312	0.377762	1	TFAM	K11830
ENSG00000133706	-0.58001	7.766159	0.377797	1	LARS	K01869
ENSG00000115084	-0.586192	5.394335	0.378646	1	SLC35F5	K15289
ENSG00000172262	-0.581187	6.551733	0.379137	1	ZNF131	_
ENSG00000213983	-0.607246	3.737667	0.379736	1	AP1G2	K12391
ENSG00000134152	-0.592704	4.904987	0.379839	1	KATNBL1	_
ENSG00000151233	-0.590278	4.903519	0.379839	1	GXYLT1	K13676
ENSG00000187583	-0.640286	2.849693	0.379901	1	PLEKHN1	_
ENSG00000105290	0.58814	5.130651	0.379904	1	APLP1	K05639
ENSG00000113389	0.580165	6.050346	0.380094	1	NPR3	K12325
ENSG00000148950	-0.610234	3.670517	0.380103	1	IMMP1L	K09647
ENSG00000236753	0.633973	2.731237	0.380326	1	MKLN1-AS	_
ENSG00000164403	-0.615306	3.563539	0.380652	1	SHROOM1	K18625
ENSG00000104205	-0.628852	2.674805	0.380799	1	SGK3	K13304
ENSG00000100330	0.583801	5.161697	0.380924	1	MTMR3	K18082
ENSG00000198198	0.585465	4.696084	0.380966	1	SZT2	K09537
ENSG00000115368	-0.579407	6.382761	0.38102	1	WDR75	K14552
ENSG00000217801	0.594864	3.945776	0.381382	1	RP11-465B22.3	_
ENSG00000053254	-0.589838	4.335902	0.381411	1	FOXN3	K09407
ENSG00000198752	0.577209	6.54533	0.381419	1	CDC42BPB	K16307
ENSG00000169740	-0.587575	4.632539	0.381419	1	ZNF32	_
ENSG00000258429	-0.616551	3.403052	0.381635	1	PDF	K01462
ENSG00000221930	0.601523	3.346548	0.381822	1	FAM45B	_
ENSG00000101160	0.573687	9.225867	0.381922	1	CTSZ	K08568
ENSG00000166250	0.580047	5.830762	0.382139	1	CLMP	K06789
ENSG00000115507	-0.613602	3.313285	0.382218	1	OTX1	K09326
ENSG00000167283	0.611576	3.252744	0.382428	1	ATP5L	K02140
ENSG00000053900	-0.585384	4.709799	0.382553	1	ANAPC4	K03351
ENSG00000164219	-0.590408	4.43229	0.382787	1	PGGT1B	K11713
ENSG00000167549	0.584611	5.036005	0.382981	1	CORO6	K13886
ENSG00000064652	-0.583948	4.861978	0.382988	1	SNX24	K17941
ENSG00000153767	-0.589403	4.84991	0.383068	1	GTF2E1	K03136
ENSG00000124191	-0.628761	3.17971	0.383116	1	TOX2	_
ENSG00000183111	0.610736	3.1576	0.383116	1	ARHGEF37	_
ENSG00000053747	0.581143	5.17515	0.383165	1	LAMA3	K06240
ENSG00000196700	-0.583072	5.157492	0.383364	1	ZNF512B	_
ENSG00000106683	0.638827	2.174998	0.383391	1	LIMK1	K05743
ENSG00000143590	0.599681	3.635159	0.383518	1	EFNA3	K05462
ENSG00000154814	-0.600023	3.628499	0.383709	1	OXNAD1	_
ENSG00000185885	0.573172	7.342882	0.383718	1	IFITM1	K06566
ENSG00000106638	-0.668396	2.140544	0.383825	1	TBL2	_
ENSG00000100442	-0.574377	6.691619	0.384165	1	FKBP3	K09570
ENSG00000273033	0.658652	2.031928	0.384293	1	RP11-67L2.2	_
ENSG00000213160	-0.585851	4.692984	0.384332	1	KLHL23	K10460
ENSG00000171867	-0.573619	6.766252	0.384623	1	PRNP	K05634
ENSG00000085465	0.670251	1.954697	0.384798	1	OVGP1	K17526
ENSG00000160678	0.670251	1.954697	0.384798	1	S100A1	_
ENSG00000108306	0.618115	2.923042	0.38481	1	FBXL20	K10268
ENSG00000183647	0.618115	2.923042	0.38481	1	ZNF530	K09228

ENSG00000168487	0.573594	6.50812	0.384934	1	BMP1	K05502
ENSG00000105971	-0.571242	7.747599	0.38503	1	CAV2	K12958
ENSG00000165060	-0.587682	4.146243	0.38508	1	FXN	K19054
ENSG00000038219	0.574953	5.713312	0.385416	1	BOD1L1	_
ENSG00000135318	-0.579679	5.516906	0.385538	1	NT5E	K01081
ENSG00000175170	0.664869	1.800836	0.385936	1	FAM182B	_
ENSG00000196754	-0.6339	2.780949	0.386264	1	S100A2	_
ENSG00000100911	-0.572785	6.360062	0.386346	1	PSME2	K06697
ENSG00000198886	0.567582	12.86187	0.386508	1	MT-ND4	K03881
ENSG00000204792	0.714577	1.694609	0.386579	1	AC104135.3	_
ENSG00000207547	-0.710785	1.741362	0.386579	1	MIR25	_
ENSG00000262251	0.679101	1.709844	0.386579	1	RP11-199F11.2	_
ENSG00000121417	0.628474	2.711465	0.386681	1	ZNF211	K09228
ENSG00000064199	-0.624931	2.741865	0.386681	1	SPA17	K12581
ENSG00000185238	-0.577018	5.490877	0.386718	1	PRMT3	K11436
ENSG00000151131	-0.58863	4.596827	0.386908	1	C12orf45	_
ENSG00000083838	0.634464	2.663675	0.387124	1	ZNF446	K09229
ENSG00000101384	-0.575676	5.213969	0.387529	1	JAG1	K06052
ENSG00000137210	-0.568773	7.301598	0.387656	1	TMEM14B	_
ENSG00000099860	0.572174	5.912592	0.387935	1	GADD45B	K04402
ENSG00000175274	0.588084	4.296074	0.388008	1	TP53I11	_
ENSG00000107263	0.57264	5.539873	0.388025	1	RAPGEF1	K06277
ENSG00000112365	-0.57906	4.476136	0.388186	1	ZBTB24	K10503
ENSG00000127334	-0.570669	5.819972	0.388288	1	DYRK2	K18669
ENSG00000108984	-0.581689	4.628978	0.388326	1	MAP2K6	K04433
ENSG00000085491	-0.568674	6.902669	0.388431	1	SLC25A24	K14684
ENSG00000149218	-0.582365	4.602209	0.388612	1	ENDOD1	K15049
ENSG00000231638	-0.723561	1.43298	0.388873	1	AC011738.4	_
ENSG00000139117	-0.571436	5.698033	0.388902	1	CPNE8	_
ENSG00000148344	-0.574967	4.802043	0.389546	1	PTGES	K15729
ENSG00000170727	-0.611077	3.010081	0.389575	1	BOP1	K14824
ENSG00000129007	-0.602623	3.394503	0.38968	1	CALML4	_
ENSG00000224411	-0.705105	1.329734	0.389778	1	RP11-1033A18.1	K04079
ENSG00000272316	-0.705105	1.329734	0.389778	1	XXbac-BPGBPG55(	_
ENSG00000108839	0.701237	1.242555	0.389778	1	ALOX12	K00458
ENSG00000255860	-0.798255	1.186151	0.390757	1	RP11-807H22.6	K13649
ENSG00000124693	0.727061	1.106896	0.390757	1	HIST1H3B	K11253
ENSG00000013503	-0.580816	4.251039	0.390891	1	POLR3B	K03021
ENSG00000176907	-0.566366	6.603079	0.391013	1	C8orf4	_
ENSG00000197702	-0.56756	6.4098	0.391415	1	PARVA	K06275
ENSG00000113441	-0.57829	4.771945	0.39147	1	LNPEP	K01257
ENSG00000214194	-0.569282	5.742835	0.391598	1	LINC00998	_
ENSG00000233276	-0.563642	7.515144	0.39163	1	GPX1	K00432
ENSG00000184226	-0.745299	1.038639	0.391806	1	PCDH9	K16498
ENSG00000128886	0.697965	0.98353	0.391806	1	ELL3	K15183
ENSG00000181350	0.697965	0.98353	0.391806	1	FAM211A	_
ENSG00000196313	0.697965	0.98353	0.391806	1	POM121	K14316
ENSG00000228232	0.697965	0.98353	0.391806	1	GAPDHP1	K00134
ENSG00000041353	-0.571771	5.596815	0.391892	1	RAB27B	K07886
ENSG00000067533	-0.571902	5.309724	0.391928	1	RRP15	_
ENSG00000106635	0.646598	2.203426	0.391947	1	BCL7B	_
ENSG00000204842	0.567812	5.51605	0.392016	1	ATXN2	_
ENSG00000175318	0.60279	3.177311	0.392054	1	GRAMD2	_
ENSG00000163644	-0.621365	2.805938	0.392109	1	PPM1K	K17505
ENSG00000078269	0.57638	4.586959	0.392173	1	SYNJ2	K01099
ENSG00000135365	0.565007	6.522991	0.392256	1	PHF21A	_
ENSG00000136010	-0.598718	3.494999	0.392344	1	ALDH1L2	K00289
ENSG00000090534	0.799257	0.790299	0.392904	1	THPO	K06854

ENSG00000205275	0.799257	0.790299	0.392904	1	CSPG4P9	K08115
ENSG00000213312	0.72944	0.820076	0.392904	1	RPL7AP34	K02936
ENSG00000217094	-0.605297	3.139986	0.392996	1	PPIAP31	K03767
ENSG00000156697	-0.564933	6.040701	0.393169	1	UTP14A	K14567
ENSG00000176623	-0.568603	5.505396	0.393212	1	RMDN1	K08142
ENSG00000154914	0.570485	4.632005	0.393306	1	USP43	K11856
ENSG00000163291	-0.591467	3.872128	0.39355	1	PAQR3	K08854
ENSG00000109107	0.565814	5.583619	0.393789	1	ALDOC	K01623
ENSG00000160991	0.85195	0.601643	0.393996	1	ORAI2	K16057
ENSG00000161912	0.85195	0.601643	0.393996	1	ADCY10P1	K11265
ENSG00000224596	0.85195	0.601643	0.393996	1	ZMIZ1-AS1	_
ENSG00000267283	0.85195	0.601643	0.393996	1	AC005306.3	_
ENSG00000122711	0.770273	0.635879	0.393996	1	SPINK4	_
ENSG00000144596	0.770273	0.635879	0.393996	1	GRIP2	_
ENSG00000167476	0.770273	0.635879	0.393996	1	JSRP1	_
ENSG00000271646	0.770273	0.635879	0.393996	1	RP11-326I11.3	_
ENSG00000008277	-0.617187	2.667576	0.394019	1	ADAM22	K16068
ENSG00000204054	0.570763	4.991127	0.394029	1	LINC00963	_
ENSG00000104774	0.565509	5.719992	0.394266	1	MAN2B1	K12311
ENSG00000206573	0.586802	3.592301	0.394301	1	SETD5-AS1	_
ENSG00000068903	0.576887	4.555523	0.394462	1	SIRT2	K11412
ENSG00000153815	0.564829	5.703211	0.394542	1	CMIP	_
ENSG00000187244	-0.579998	4.436272	0.394564	1	BCAM	K06578
ENSG00000056291	-1.03357	-0.033371	0.394643	1	NPFFR2	K08375
ENSG00000176826	-1.03357	-0.033371	0.394643	1	FKBP9L	K09575
ENSG00000213901	-1.03357	-0.033371	0.394643	1	SLC23A3	K14612
ENSG00000243896	-1.03357	-0.033371	0.394643	1	OR2A7	K04257
ENSG00000244513	-1.03357	-0.033371	0.394643	1	CTD-2013N24.2	_
ENSG00000255306	-1.03357	-0.033371	0.394643	1	RP5-901A4.1	_
ENSG00000266402	-1.03357	-0.033371	0.394643	1	SNORA76	_
ENSG00000122861	-0.678102	2.03736	0.394662	1	PLAU	K01348
ENSG00000005483	0.567405	5.184873	0.394821	1	KMT2E	K09189
ENSG00000072501	-0.846743	0.526095	0.394949	1	SMC1A	K06636
ENSG00000150636	-0.846743	0.526095	0.394949	1	CCDC102B	_
ENSG00000177570	-0.846743	0.526095	0.394949	1	SAMD12	_
ENSG00000116663	0.825392	0.424913	0.394949	1	FBXO6	K10100
ENSG00000198689	0.825392	0.424913	0.394949	1	SLC9A6	K12041
ENSG00000238271	0.825392	0.424913	0.394949	1	IFNWP19	K05440
ENSG00000261655	0.825392	0.424913	0.394949	1	CTD-3064M3.3	_
ENSG00000141837	-0.79053	0.490468	0.394949	1	CACNA1A	K04344
ENSG00000213920	-0.79053	0.490468	0.394949	1	MDP1	K17619
ENSG00000236751	-0.79053	0.490468	0.394949	1	RP1-30G7.2	_
ENSG00000253106	-0.79053	0.490468	0.394949	1	RP11-158K1.3	_
ENSG00000106799	0.557927	9.21575	0.395062	1	TGFBR1	K04674
ENSG00000249395	-0.612371	2.982632	0.395175	1	CASC9	_
ENSG00000005175	-0.562051	6.191373	0.395196	1	RPAP3	K04135
ENSG00000151292	-0.565588	5.48893	0.395359	1	CSNK1G3	K08958
ENSG00000158623	-1.019599	0.22316	0.395441	1	COPG2	K17267
ENSG00000231702	-1.019599	0.22316	0.395441	1	RP11-54O7.10	K02924
ENSG00000235912	-1.019599	0.22316	0.395441	1	RP1-159A19.3	K07604
ENSG00000236871	-1.019599	0.22316	0.395441	1	LINC00106	_
ENSG00000271826	-1.019599	0.22316	0.395441	1	RP1-93I3.1	_
ENSG00000011590	0.90396	0.178069	0.395441	1	ZBTB32	K10506
ENSG00000156042	0.90396	0.178069	0.395441	1	TTC18	_
ENSG00000187008	0.90396	0.178069	0.395441	1	PKD1L3	_
ENSG00000213713	0.616285	2.526585	0.395509	1	PIGCP1	K03859
ENSG00000138780	-0.584697	3.74065	0.3956	1	GSTCD	_
ENSG00000082781	-0.559258	7.276187	0.395644	1	ITGB5	K06588

ENSG00000141642	-0.645169	1.958722	0.395739	1	ELAC1	K00784
ENSG00000197461	-0.573323	4.596748	0.395765	1	PDGFA	K04359
ENSG00000074755	0.568029	5.121876	0.395804	1	ZZEF1	K17985
ENSG00000185453	0.591813	3.229944	0.395907	1	C19orf68	_
ENSG00000176978	-0.56179	6.215122	0.39597	1	DPP7	K01276
ENSG00000011143	-0.585614	3.898721	0.39599	1	MKS1	K19332
ENSG00000165416	-0.561212	6.175727	0.396045	1	SUGT1	K12795
ENSG00000158411	-0.56645	5.542475	0.396372	1	MITD1	_
ENSG00000145242	-0.591776	3.69414	0.396374	1	EPHA5	K05106
ENSG00000145604	-0.558615	6.999898	0.396653	1	SKP2	K03875
ENSG00000157259	-0.565544	5.076796	0.396717	1	GATAD1	K17985
ENSG00000115904	0.560617	6.017426	0.396783	1	SOS1	K03099
ENSG00000151881	-0.562408	5.618352	0.396871	1	C5orf28	_
ENSG00000065485	0.559104	6.219747	0.397007	1	PDIA5	K09583
ENSG00000125845	0.557012	7.476454	0.397038	1	BMP2	K04662
ENSG00000159882	-0.643367	2.452533	0.397217	1	ZNF230	K09228
ENSG00000088451	-0.572981	4.614718	0.397236	1	TGDS	K01710
ENSG00000123472	-0.56064	5.908583	0.397375	1	ATPAF1	K07555
ENSG00000132313	-0.562658	5.722273	0.397582	1	MRPL35	K02916
ENSG00000145623	0.555086	8.443555	0.397825	1	OSMR	K08267
ENSG00000106538	0.637023	2.356767	0.398169	1	RARRES2	K10044
ENSG00000156968	-0.632244	2.403142	0.398169	1	MPV17L	K13349
ENSG00000094841	-0.618054	2.394324	0.398169	1	UPRT	K00761
ENSG00000099942	0.555192	7.588511	0.398447	1	CRKL	K04438
ENSG00000168077	-0.560922	5.617462	0.398662	1	SCARA3	_
ENSG00000113140	-0.598773	3.08407	0.398854	1	SPARC	_
ENSG00000164211	-0.558877	5.677675	0.399254	1	STARD4	_
ENSG00000236618	-0.602593	2.728059	0.399458	1	PITPNA-AS1	_
ENSG00000139211	-0.567177	4.376342	0.399704	1	AMIGO2	_
ENSG00000152359	-0.586575	3.673624	0.399976	1	POC5	K16483
ENSG00000152413	-0.586575	3.673624	0.399976	1	HOMER1	K15010
ENSG00000225151	0.599499	2.969045	0.400249	1	GOLGA2P7	_
ENSG00000141551	0.552979	7.608689	0.400299	1	CSNK1D	K08959
ENSG00000172828	-0.59351	3.231266	0.40078	1	CES3	K15743
ENSG00000163006	-0.566997	4.611112	0.400825	1	CCDC138	_
ENSG00000158106	-0.562127	5.353176	0.400871	1	RHPN1	_
ENSG00000099849	-0.562139	5.181769	0.400927	1	RASSF7	K09855
ENSG00000168564	0.553849	6.521041	0.40124	1	CDKN2AIP	_
ENSG00000100997	0.556514	5.926369	0.40126	1	ABHD12	K13704
ENSG00000257815	0.67379	1.526115	0.401305	1	RP11-611E13.2	_
ENSG00000104140	0.552028	7.407144	0.401341	1	RHOV	K07866
ENSG00000115380	-0.551011	8.471113	0.401367	1	EFEMP1	K18262
ENSG00000104312	-0.556883	5.920916	0.40147	1	RIPK2	K08846
ENSG00000204946	-0.580955	3.402435	0.401746	1	ZNF783	K09228
ENSG00000196850	0.554025	5.996906	0.401895	1	PPTC7	K17508
ENSG00000159921	-0.559975	5.389113	0.402234	1	GNE	K12409
ENSG00000198807	-0.608557	2.589201	0.40226	1	PAX9	K09382
ENSG00000185115	-0.57605	3.877916	0.40228	1	NDNL2	_
ENSG00000105866	-0.596568	2.884664	0.402637	1	SP4	K09194
ENSG00000006016	0.560203	5.086099	0.402763	1	CRLF1	_
ENSG00000163521	-0.651356	2.129855	0.402827	1	GLB1L	K11447
ENSG00000226049	0.649266	1.434174	0.403125	1	TLK2P1	K08864
ENSG00000106785	-0.557443	5.484051	0.403225	1	TRIM14	K12004
ENSG00000107798	-0.55198	6.426216	0.403314	1	LIPA	K01052
ENSG00000181035	0.609649	2.503833	0.403327	1	SLC25A42	K15085
ENSG00000114023	0.552818	6.269594	0.403481	1	FAM162A	_
ENSG00000170448	-0.564577	4.563778	0.40361	1	NFXL1	K15683
ENSG00000235043	0.58358	3.066893	0.403614	1	TECRP1	K10258

ENSG00000147601	-0.552371	5.843803	0.403863	1	TERF1	K11110
ENSG00000074582	-0.562541	4.46617	0.403928	1	BCS1L	K08900
ENSG00000177000	0.563942	4.155396	0.404024	1	MTHFR	K00297
ENSG00000140750	0.56223	4.836649	0.404059	1	ARHGAP17	_
ENSG00000033178	-0.554961	5.613896	0.40408	1	UBA6	K10699
ENSG00000213762	0.575652	3.621311	0.404213	1	ZNF134	_
ENSG00000163617	0.622201	2.01207	0.404264	1	KIAA1407	_
ENSG00000196878	0.54777	7.800732	0.404534	1	LAMB3	K06244
ENSG00000211584	-0.557315	5.011814	0.404629	1	SLC48A1	K15380
ENSG00000184508	-0.584963	3.249648	0.405065	1	HDDC3	K01139
ENSG00000244300	0.560377	4.908672	0.405387	1	RP11-475N22.4	_
ENSG00000142627	-0.548771	6.741211	0.405517	1	EPHA2	K05103
ENSG00000044459	-0.576369	3.862953	0.40553	1	CNTLN	K16467
ENSG00000186352	0.631861	1.933823	0.405843	1	ANKRD37	_
ENSG00000198805	-0.548131	6.709797	0.406133	1	PNP	K03783
ENSG00000047578	0.580176	3.355556	0.406252	1	KIAA0556	_
ENSG00000116604	0.550308	5.979585	0.406422	1	MEF2D	K09262
ENSG00000100592	-0.56803	4.056832	0.406511	1	DAAM1	K04512
ENSG00000059588	-0.549625	5.920206	0.406559	1	TARBP1	_
ENSG00000139269	-0.586977	3.178854	0.406612	1	INHBE	K04667
ENSG00000078177	-0.594503	2.942823	0.407071	1	N4BP2	K15720
ENSG00000140287	0.739816	1.16498	0.407523	1	HDC	K01590
ENSG00000175106	-0.716568	1.23914	0.407523	1	TVP23C	K17985
ENSG00000178852	-0.716568	1.23914	0.407523	1	EFCAB13	_
ENSG00000197191	-0.716568	1.23914	0.407523	1	C9orf169	_
ENSG00000258048	-0.716568	1.23914	0.407523	1	RP11-530C5.1	_
ENSG00000246859	0.687668	1.187541	0.407523	1	STARD4-AS1	_
ENSG00000260418	0.687668	1.187541	0.407523	1	RP3-406A7.7	_
ENSG00000187642	-0.669629	1.91366	0.407585	1	C1orf170	_
ENSG00000158715	-0.605425	2.660307	0.407672	1	SLC45A3	K15379
ENSG00000163050	0.550148	5.743146	0.407724	1	ADCK3	_
ENSG00000101680	0.545855	6.632452	0.407884	1	LAMA1	K05637
ENSG00000156958	-0.566	4.194667	0.408103	1	GALK2	K18674
ENSG00000101187	0.543873	7.390956	0.408322	1	SLCO4A1	K14354
ENSG00000048405	-0.561275	4.580317	0.40875	1	ZNF800	_
ENSG00000165832	-0.551722	5.252323	0.408769	1	TRUB1	K03177
ENSG00000148175	0.541813	8.477429	0.409125	1	STOM	K17286
ENSG00000128274	0.553265	4.984245	0.409164	1	A4GALT	K01988
ENSG00000118898	0.543369	7.043425	0.409251	1	PPL	K10386
ENSG00000150455	-0.632904	1.827549	0.409516	1	TIRAP	K05403
ENSG00000235173	-0.632904	1.827549	0.409516	1	FAM203A	_
ENSG00000081913	0.567337	3.908544	0.409952	1	PHLPP1	K16340
ENSG00000237412	0.540318	9.531444	0.409965	1	PRSS56	_
ENSG00000115652	-0.547992	5.154981	0.410102	1	UXS1	K08678
ENSG00000166275	-0.566891	3.393878	0.410106	1	C10orf32	_
ENSG00000174137	0.713165	1.046477	0.410207	1	FAM53A	_
ENSG00000132763	-0.565591	3.901427	0.410501	1	MMACHC	_
ENSG00000173812	-0.540054	8.845609	0.410525	1	EIF1	K03113
ENSG00000086300	-0.561093	4.27369	0.410689	1	SNX10	K17924
ENSG00000101247	-0.569096	3.98954	0.410953	1	NDUFAF5	K18162
ENSG00000185128	0.586317	3.167934	0.411037	1	TBC1D3F	K11837
ENSG00000134830	0.570351	3.471093	0.411516	1	C5AR2	K04171
ENSG00000054179	0.668892	1.669927	0.411671	1	ENTPD2	K01509
ENSG00000164330	0.633416	1.685423	0.411671	1	EBF1	K09103
ENSG00000088727	-0.614116	2.1583	0.411745	1	KIF9	K10397
ENSG00000141519	-0.614116	2.1583	0.411745	1	CCDC40	_
ENSG00000160781	-0.614116	2.1583	0.411745	1	PAQR6	_
ENSG00000124787	-0.553235	4.313798	0.411886	1	RPP40	K14530

ENSG00000151532	-0.556457	4.380477	0.412167	1	VTI1A	K08493
ENSG00000137168	-0.545986	5.761527	0.412262	1	PPIL1	K12733
ENSG00000080822	-0.550464	5.014508	0.412706	1	CLDND1	_
ENSG00000090857	0.561726	3.890949	0.413198	1	PDPR	K17509
ENSG00000163520	0.538698	7.029338	0.413296	1	FBLN2	K17307
ENSG00000149527	0.744925	0.890209	0.413309	1	PLCH2	K19006
ENSG00000183072	0.744925	0.890209	0.413309	1	NKX2-5	K09345
ENSG00000218422	0.744925	0.890209	0.413309	1	AC016773.1	K14283
ENSG00000173227	0.540551	5.908156	0.413337	1	SYT12	_
ENSG00000184863	0.541924	5.856739	0.41334	1	RBM33	_
ENSG00000170946	-0.584335	3.272493	0.413482	1	DNAJC24	K17867
ENSG00000143458	-0.576647	3.267802	0.413482	1	GABPB2	K09454
ENSG00000196312	0.631259	2.043778	0.413612	1	HIATL2	_
ENSG00000120658	-0.56917	3.663047	0.413857	1	ENOX1	_
ENSG00000227764	0.684924	1.570052	0.414088	1	RP11-354K1.1	_
ENSG00000041515	0.646622	1.586746	0.414088	1	MYO16	K17481
ENSG00000109917	-0.544134	5.337018	0.414273	1	ZNF259	K06874
ENSG00000141179	-0.558517	3.528916	0.414278	1	PCTP	K09228
ENSG00000143624	0.55051	4.048057	0.414295	1	INTS3	K13140
ENSG00000077232	-0.537616	7.113961	0.414363	1	DNAJC10	K09530
ENSG00000160712	0.552184	4.42672	0.414706	1	IL6R	K05055
ENSG00000111726	-0.539583	5.836183	0.414849	1	CMAS	K00983
ENSG00000184887	-0.581863	3.047032	0.41505	1	BTBD6	K10478
ENSG00000171793	-0.536227	7.351884	0.415188	1	CTPS1	K01937
ENSG00000088826	0.551264	4.474956	0.415266	1	SMOX	K12259
ENSG00000164603	-0.606951	2.625194	0.415387	1	C7orf60	_
ENSG00000213066	-0.556916	3.723807	0.415539	1	FGFR1OP	K16546
ENSG00000100266	0.535953	6.890598	0.415908	1	PACSIN2	_
ENSG00000087266	0.54126	5.63051	0.415998	1	SH3BP2	K07984
ENSG00000159082	0.551834	4.074364	0.416133	1	SYNJ1	K01099
ENSG00000101004	-0.578764	3.173836	0.416437	1	NINL	K16477
ENSG00000173482	-0.543912	5.182947	0.416628	1	PTPRM	K05693
ENSG00000115464	0.53818	5.919092	0.416703	1	USP34	K11853
ENSG00000123545	-0.54994	4.148378	0.416783	1	NDUFAF4	K18161
ENSG00000100033	0.661929	1.480849	0.416821	1	PRODH	K00318
ENSG00000267040	0.661929	1.480849	0.416821	1	RP11-35G9.3	_
ENSG00000115325	-0.553706	4.222968	0.416853	1	DOK1	K14752
ENSG00000117010	-0.596085	2.581503	0.416911	1	ZNF684	K09228
ENSG00000091831	-0.740143	0.776389	0.416921	1	ESR1	_
ENSG00000219891	-0.740143	0.776389	0.416921	1	ZSCAN12P1	K09229
ENSG00000161547	-0.53324	7.86502	0.417152	1	SRSF2	K12891
ENSG00000089902	0.535428	6.157612	0.417193	1	RCOR1	K11829
ENSG00000176912	0.584599	2.752339	0.417195	1	C18orf56	K00560
ENSG00000187653	-0.543347	4.852786	0.417651	1	TMSB4XP8	K05764
ENSG00000111640	0.530814	12.45609	0.417845	1	GAPDH	K00134
ENSG00000188042	-0.625585	1.94649	0.41793	1	ARL4C	K07945
ENSG00000166016	0.545484	4.785032	0.417996	1	ABTB2	K10521
ENSG00000172965	-0.537735	5.477191	0.418258	1	MIR4435-1HG	_
ENSG00000272565	-0.569749	3.628061	0.41859	1	RP11-485G4.2	_
ENSG00000163482	-0.559808	3.389578	0.418628	1	STK36	K06228
ENSG00000127419	0.564313	3.225831	0.418644	1	TMEM175	_
ENSG00000103876	-0.540852	4.866343	0.418695	1	FAH	K01555
ENSG00000173517	0.553121	4.143112	0.41905	1	PEAK1	K17538
ENSG00000198796	-0.543257	4.806624	0.419081	1	ALPK2	K08868
ENSG00000110172	-0.53229	6.992811	0.4192	1	CHORDC1	K16729
ENSG00000129480	-0.550653	3.907105	0.419207	1	DTD2	K17985
ENSG00000125726	0.535257	6.095319	0.419228	1	CD70	K05470
ENSG00000246705	0.538434	5.329672	0.419783	1	H2AFJ	K11251

ENSG00000196954	-0.538322	5.267155	0.419866	1	CASP4	K04394
ENSG00000241627	-0.695087	1.414924	0.419933	1	UBQLN4P1	K04523
ENSG00000228506	0.679882	1.366596	0.419933	1	RP11-98I9.4	_
ENSG00000138134	-0.541038	4.917916	0.420017	1	STAMBPL1	_
ENSG00000107672	-0.537044	5.621034	0.420097	1	NSMCE4A	_
ENSG00000023516	-0.545889	4.823732	0.420144	1	AKAP11	K16527
ENSG00000120458	-0.603974	2.694582	0.420144	1	MSANTD2	_
ENSG00000164430	-0.537342	5.669118	0.42023	1	MB21D1	K17834
ENSG00000255103	0.588421	2.4334	0.420304	1	KIAA0754	K09228
ENSG00000205047	0.605723	2.1569	0.420439	1	FLJ00104	K17985
ENSG00000135213	0.63257	1.814899	0.420445	1	POM121C	K14316
ENSG00000255248	0.63257	1.814899	0.420445	1	RP11-166D19.1	_
ENSG00000198157	-0.54579	4.253059	0.420582	1	HMGN5	K17045
ENSG00000116273	0.536238	5.367434	0.420722	1	PHF13	_
ENSG00000169100	0.527506	9.135598	0.421228	1	SLC25A6	K05863
ENSG00000182287	-0.542387	4.42385	0.421255	1	AP1S2	K12394
ENSG00000135924	0.539834	4.583492	0.421338	1	DNAJB2	K09508
ENSG00000162623	-0.531025	6.453991	0.421412	1	TYW3	K15450
ENSG00000143952	-0.532439	5.891778	0.421465	1	VPS54	K17600
ENSG00000267673	-0.581612	2.645647	0.421769	1	FDX1L	_
ENSG00000134987	-0.534253	5.960834	0.421961	1	WDR36	K14554
ENSG00000185839	-0.570936	3.144226	0.422031	1	RP4-592A1.2	K00939
ENSG00000113645	0.528462	7.012709	0.422222	1	WWC1	K16685
ENSG00000164815	-0.537071	5.053209	0.42229	1	ORC5	K02607
ENSG00000198369	-0.540062	4.665451	0.422382	1	SPRED2	K04703
ENSG00000244041	0.580411	2.958281	0.422429	1	LINC01011	_
ENSG00000181649	-0.529704	6.410426	0.422477	1	PHLDA2	_
ENSG00000198814	-0.634113	2.119077	0.422712	1	GK	K00864
ENSG00000271335	0.585323	2.56511	0.423507	1	RP11-324I22.4	_
ENSG00000105963	-0.642597	1.290401	0.42351	1	ADAP1	_
ENSG00000177855	-0.642597	1.290401	0.42351	1	CACYBPP2	K04507
ENSG00000114544	-0.558035	3.584736	0.423621	1	SLC41A3	K15122
ENSG00000172273	0.539038	4.404154	0.42365	1	HINFP	K04613
ENSG00000151576	-0.533604	5.158637	0.423709	1	QTRTD1	K15407
ENSG00000076864	0.553021	3.757759	0.423715	1	RAP1GAP	K17700
ENSG00000021826	0.524167	10.05511	0.423902	1	CPS1	K01948
ENSG00000182240	0.52462	8.666107	0.423971	1	BACE2	K07747
ENSG00000250588	-0.553456	3.230471	0.423982	1	IQCJ-SCHIP1	_
ENSG00000261116	-0.559039	3.470843	0.424065	1	RP3-523K23.2	_
ENSG00000177054	-0.555989	3.850623	0.424143	1	ZDHHC13	K18932
ENSG00000116127	-0.550849	3.847523	0.424143	1	ALMS1	K16741
ENSG00000013364	0.526347	6.540446	0.424171	1	MVP	K17266
ENSG00000188910	-0.538837	4.401221	0.424187	1	GJB3	K07622
ENSG00000204406	-0.605539	2.342804	0.424256	1	MBD5	_
ENSG00000214145	0.600197	2.314832	0.424256	1	LINC00887	_
ENSG00000222041	-0.540405	4.749195	0.424408	1	LINC00152	_
ENSG00000111540	0.539382	4.439013	0.424451	1	RAB5B	K07888
ENSG00000076242	-0.534261	5.236142	0.424497	1	MLH1	K08734
ENSG00000163399	0.522851	10.01411	0.425069	1	ATP1A1	K01539
ENSG00000146223	-0.524449	7.535753	0.425215	1	RPL7L1	K17985
ENSG00000136122	-0.532416	5.24655	0.425254	1	BORA	K16831
ENSG00000117461	0.536112	4.412452	0.425527	1	PIK3R3	K02649
ENSG00000169093	0.526367	6.135875	0.425572	1	ASMTL	K05066
ENSG00000155115	-0.528567	5.771191	0.425875	1	GTF3C6	K15203
ENSG00000109066	0.545105	4.220649	0.426009	1	TMEM104	K17985
ENSG00000035687	-0.525267	6.400116	0.426021	1	ADSS	K01939
ENSG00000172476	-0.89069	0.362879	0.426125	1	RAB40A	K07928
ENSG00000188996	-0.89069	0.362879	0.426125	1	HUS1B	K10996

ENSG00000227124	-0.89069	0.362879	0.426125	1	ZNF717	K09228
ENSG00000237350	-0.89069	0.362879	0.426125	1	CDC42P6	K04393
ENSG00000272947	-0.89069	0.362879	0.426125	1	RP11-71H17.9	_
ENSG00000007952	-0.827036	0.322603	0.426125	1	NOX1	K08008
ENSG00000104723	-0.827036	0.322603	0.426125	1	TUSC3	K12669
ENSG00000116885	-0.827036	0.322603	0.426125	1	OSCP1	_
ENSG00000123977	-0.827036	0.322603	0.426125	1	DAW1	K19760
ENSG00000133710	-0.827036	0.322603	0.426125	1	SPINK5	_
ENSG00000155959	-0.827036	0.322603	0.426125	1	VBP1	_
ENSG00000196648	-0.827036	0.322603	0.426125	1	GOLGA6L20	_
ENSG00000227946	-0.827036	0.322603	0.426125	1	AC007383.3	_
ENSG00000268798	-0.827036	0.322603	0.426125	1	AC027307.3	K01368
ENSG00000163319	-0.531795	5.372297	0.426139	1	MRPS18C	K02963
ENSG00000138794	-0.559607	3.410329	0.426189	1	CASP6	K04396
ENSG00000164506	-0.559607	3.410329	0.426189	1	STXBP5	K08518
ENSG00000129514	-0.550517	3.950229	0.426378	1	FOXA1	K09387
ENSG00000149547	-0.524372	6.508369	0.426459	1	EI24	K10134
ENSG00000175137	0.53649	4.810352	0.426642	1	SH3BP5L	_
ENSG00000068079	-0.532893	5.297505	0.426832	1	IFI35	_
ENSG00000069509	-0.535099	4.770174	0.426937	1	FUNDC1	K17986
ENSG00000099377	0.527306	5.596382	0.427281	1	HSD3B7	K12408
ENSG00000164934	-0.523393	6.817371	0.427335	1	DCAF13	K11806
ENSG00000196950	0.523478	6.620359	0.427337	1	SLC39A10	K14716
ENSG00000126391	0.527175	5.660361	0.427489	1	FRMD8	_
ENSG00000213185	-0.729816	1.142407	0.427663	1	FAM24B	_
ENSG00000197381	0.521314	7.352089	0.428007	1	ADARB1	K13194
ENSG00000108465	-0.522687	6.551925	0.428183	1	CDK5RAP3	_
ENSG00000112812	-0.557448	3.65595	0.428188	1	PRSS16	K09649
ENSG00000124193	-0.520856	7.340124	0.428648	1	SRSF6	K12893
ENSG00000133935	-0.526659	5.248847	0.428747	1	C14orf1	K11504
ENSG00000131944	-0.611748	2.206698	0.428915	1	C19orf40	K10898
ENSG00000172270	0.5184	10.47282	0.42892	1	BSG	K06535
ENSG00000125375	-0.574445	2.959354	0.428925	1	ATP5S	K07554
ENSG00000176055	-0.545625	3.631249	0.429177	1	MBLAC2	K14026
ENSG00000053371	-0.529682	5.174469	0.429306	1	AKR7A2	K15303
ENSG00000112234	-0.539235	4.481783	0.429344	1	FBXL4	K10270
ENSG00000180448	0.529307	5.003618	0.429381	1	HMHA1	_
ENSG00000138778	-0.526067	5.465354	0.429817	1	CENPE	K11498
ENSG00000269728	-0.535487	4.419722	0.429839	1	RP11-145M9.4	_
ENSG00000267809	-0.6525	1.555135	0.429983	1	NDUFV2P1	K03943
ENSG00000063660	0.519796	6.803087	0.430216	1	GPC1	K08107
ENSG00000137171	-0.534579	4.302612	0.430312	1	KLC4	K10407
ENSG00000141527	0.526568	5.230137	0.430481	1	CARD14	_
ENSG00000144283	-0.525621	5.653565	0.430627	1	PKP4	K19720
ENSG00000060566	2.438432	-1.595162	0.430642	1	CREB3L3	K09048
ENSG00000063015	2.438432	-1.595162	0.430642	1	SEZ6	_
ENSG00000077092	2.438432	-1.595162	0.430642	1	RARB	K08528
ENSG00000086991	2.438432	-1.595162	0.430642	1	NOX4	_
ENSG00000087085	2.438432	-1.595162	0.430642	1	ACHE	K01049
ENSG00000087116	2.438432	-1.595162	0.430642	1	ADAMTS2	K08618
ENSG00000091656	2.438432	-1.595162	0.430642	1	ZFHX4	K09380
ENSG00000103316	2.438432	-1.595162	0.430642	1	CRYM	K18258
ENSG00000106069	2.438432	-1.595162	0.430642	1	CHN2	K05062
ENSG00000108825	2.438432	-1.595162	0.430642	1	PTGES3L-AARSD1	K07050
ENSG00000111732	2.438432	-1.595162	0.430642	1	AICDA	K10989
ENSG00000113073	2.438432	-1.595162	0.430642	1	SLC4A9	K13860
ENSG00000124232	2.438432	-1.595162	0.430642	1	RBPJL	K06053
ENSG00000129194	2.438432	-1.595162	0.430642	1	SOX15	K09271

ENSG00000130826	2.438432	-1.595162	0.430642	1	DKC1	K11131
ENSG00000130876	2.438432	-1.595162	0.430642	1	SLC7A10	K13782
ENSG00000133246	2.438432	-1.595162	0.430642	1	PRAM1	_
ENSG00000140285	2.438432	-1.595162	0.430642	1	FGF7	K04358
ENSG00000155974	2.438432	-1.595162	0.430642	1	GRIP1	_
ENSG00000159618	2.438432	-1.595162	0.430642	1	GPR114	K08459
ENSG00000162267	2.438432	-1.595162	0.430642	1	ITIH3	_
ENSG00000162618	2.438432	-1.595162	0.430642	1	ELTD1	K04595
ENSG00000162944	2.438432	-1.595162	0.430642	1	RFTN2	_
ENSG00000164675	2.438432	-1.595162	0.430642	1	IQUB	_
ENSG00000165092	2.438432	-1.595162	0.430642	1	ALDH1A1	K07249
ENSG00000169314	2.438432	-1.595162	0.430642	1	C22orf15	_
ENSG00000172247	2.438432	-1.595162	0.430642	1	C1QTNF4	K19470
ENSG00000177335	2.438432	-1.595162	0.430642	1	C8orf31	_
ENSG00000184357	2.438432	-1.595162	0.430642	1	HIST1H1B	K11275
ENSG00000184434	2.438432	-1.595162	0.430642	1	LRRC19	_
ENSG00000185829	2.438432	-1.595162	0.430642	1	ARL17A	K06883
ENSG00000188199	2.438432	-1.595162	0.430642	1	NUTM2B	_
ENSG00000188375	2.438432	-1.595162	0.430642	1	H3F3C	K11253
ENSG00000197140	2.438432	-1.595162	0.430642	1	ADAM32	K16070
ENSG00000197445	2.438432	-1.595162	0.430642	1	C16orf47	_
ENSG00000199038	2.438432	-1.595162	0.430642	1	MIR210	_
ENSG00000204241	2.438432	-1.595162	0.430642	1	RP11-713P17.3	_
ENSG00000207697	2.438432	-1.595162	0.430642	1	MIR573	_
ENSG00000213057	2.438432	-1.595162	0.430642	1	C1orf220	K09228
ENSG00000215187	2.438432	-1.595162	0.430642	1	FAM166B	_
ENSG00000215838	2.438432	-1.595162	0.430642	1	RP11-466F5.6	K01672
ENSG00000217181	2.438432	-1.595162	0.430642	1	RP11-360O19.5	K02889
ENSG00000219410	2.438432	-1.595162	0.430642	1	RP4-761J14.8	_
ENSG00000222489	2.438432	-1.595162	0.430642	1	SNORA79	_
ENSG00000224505	2.438432	-1.595162	0.430642	1	AC002117.1	_
ENSG00000225953	2.438432	-1.595162	0.430642	1	SATB2-AS1	_
ENSG00000226232	2.438432	-1.595162	0.430642	1	RP11-419C5.2	K01896
ENSG00000226321	2.438432	-1.595162	0.430642	1	AC104809.3	_
ENSG00000228261	2.438432	-1.595162	0.430642	1	RP11-127L20.3	_
ENSG00000228915	2.438432	-1.595162	0.430642	1	OR7E128P	K04257
ENSG00000229267	2.438432	-1.595162	0.430642	1	AC072062.1	_
ENSG00000229587	2.438432	-1.595162	0.430642	1	RP11-197P3.5	_
ENSG00000230432	2.438432	-1.595162	0.430642	1	AC114803.3	_
ENSG00000231073	2.438432	-1.595162	0.430642	1	RP11-316M1.3	_
ENSG00000231881	2.438432	-1.595162	0.430642	1	RP5-1120P11.3	_
ENSG00000234268	2.438432	-1.595162	0.430642	1	AP000936.1	K02978
ENSG00000234567	2.438432	-1.595162	0.430642	1	RP1-283K11.2	_
ENSG00000235313	2.438432	-1.595162	0.430642	1	HM13-IT1	_
ENSG00000235772	2.438432	-1.595162	0.430642	1	AP001625.6	_
ENSG00000236754	2.438432	-1.595162	0.430642	1	AC004019.13	_
ENSG00000237021	2.438432	-1.595162	0.430642	1	RP3-486I3.7	_
ENSG00000237596	2.438432	-1.595162	0.430642	1	RP13-143G15.4	_
ENSG00000237766	2.438432	-1.595162	0.430642	1	GGTA2P	K00743
ENSG00000238868	2.438432	-1.595162	0.430642	1	snoU13	_
ENSG00000239149	2.438432	-1.595162	0.430642	1	SNORA59A	_
ENSG00000240032	2.438432	-1.595162	0.430642	1	RP11-274H2.3	_
ENSG00000241547	2.438432	-1.595162	0.430642	1	RP11-4K3__A.5	K05692
ENSG00000245067	2.438432	-1.595162	0.430642	1	IGFBP7-AS1	_
ENSG00000245148	2.438432	-1.595162	0.430642	1	ARAP1-AS2	_
ENSG00000249562	2.438432	-1.595162	0.430642	1	RP11-116O11.2	_
ENSG00000249852	2.438432	-1.595162	0.430642	1	AC145676.2	_
ENSG00000252516	2.438432	-1.595162	0.430642	1	RNA5SP82	_

ENSG00000253463	2.438432	-1.595162	0.430642	1	HMGB1P19	K10802
ENSG00000254122	2.438432	-1.595162	0.430642	1	PCDHGB7	K16496
ENSG00000254710	2.438432	-1.595162	0.430642	1	CTD-2216M2.1	_
ENSG00000254718	2.438432	-1.595162	0.430642	1	CTD-2184C24.2	_
ENSG00000255120	2.438432	-1.595162	0.430642	1	OVOL1-AS1	_
ENSG00000255318	2.438432	-1.595162	0.430642	1	RP11-655M14.13	_
ENSG00000257193	2.438432	-1.595162	0.430642	1	RP11-818F20.4	_
ENSG00000258102	2.438432	-1.595162	0.430642	1	RP11-809C9.2	_
ENSG00000258818	2.438432	-1.595162	0.430642	1	RNASE4	K16632
ENSG00000258890	2.438432	-1.595162	0.430642	1	CEP95	K16544
ENSG00000258891	2.438432	-1.595162	0.430642	1	RP5-1021I20.5	_
ENSG00000258929	2.438432	-1.595162	0.430642	1	RP11-58E21.3	_
ENSG00000259326	2.438432	-1.595162	0.430642	1	RP11-102L12.2	_
ENSG00000259341	2.438432	-1.595162	0.430642	1	RP11-20G13.1	_
ENSG00000260086	2.438432	-1.595162	0.430642	1	RP11-42I10.1	_
ENSG00000260528	2.438432	-1.595162	0.430642	1	FAM157C	_
ENSG00000260592	2.438432	-1.595162	0.430642	1	CTA-363E6.6	_
ENSG00000260884	2.438432	-1.595162	0.430642	1	AC009120.5	_
ENSG00000261546	2.438432	-1.595162	0.430642	1	CTD-2555A7.3	_
ENSG00000261567	2.438432	-1.595162	0.430642	1	RP11-680G10.1	_
ENSG00000262098	2.438432	-1.595162	0.430642	1	CTD-2561B21.10	_
ENSG00000262358	2.438432	-1.595162	0.430642	1	CTD-3195I5.4	_
ENSG00000262601	2.438432	-1.595162	0.430642	1	CTC-786C10.1	_
ENSG00000263105	2.438432	-1.595162	0.430642	1	RP11-95P2.3	_
ENSG00000263916	2.438432	-1.595162	0.430642	1	RP11-110H1.4	_
ENSG00000266573	2.438432	-1.595162	0.430642	1	RP11-449D8.5	_
ENSG00000266891	2.438432	-1.595162	0.430642	1	RP11-692N5.2	K02900
ENSG00000266964	2.438432	-1.595162	0.430642	1	FXYD1	K13360
ENSG00000267033	2.438432	-1.595162	0.430642	1	CTD-2562J15.4	_
ENSG00000267048	2.438432	-1.595162	0.430642	1	RP11-566K11.7	_
ENSG00000267304	2.438432	-1.595162	0.430642	1	AC004637.1	_
ENSG00000267394	2.438432	-1.595162	0.430642	1	CTB-175E5.7	_
ENSG00000267510	2.438432	-1.595162	0.430642	1	CTC-325H20.4	_
ENSG00000267747	2.438432	-1.595162	0.430642	1	RP11-392O1.4	_
ENSG00000267795	2.438432	-1.595162	0.430642	1	SMIM22	_
ENSG00000267964	2.438432	-1.595162	0.430642	1	AC022400.2	K11257
ENSG00000267992	2.438432	-1.595162	0.430642	1	CTB-189B5.3	_
ENSG00000268670	2.438432	-1.595162	0.430642	1	CTD-2622I13.3	_
ENSG00000268931	2.438432	-1.595162	0.430642	1	RP11-886P16.6	_
ENSG00000269889	2.438432	-1.595162	0.430642	1	RP11-816J6.3	_
ENSG00000269903	2.438432	-1.595162	0.430642	1	RP11-571M6.18	_
ENSG00000270265	2.438432	-1.595162	0.430642	1	RP11-731D1.4	_
ENSG00000272006	2.438432	-1.595162	0.430642	1	RP11-583F2.6	K19601
ENSG00000272627	2.438432	-1.595162	0.430642	1	RP11-354E23.5	_
ENSG00000272707	2.438432	-1.595162	0.430642	1	RP11-534C12.1	_
ENSG00000272834	2.438432	-1.595162	0.430642	1	RP5-1042K10.13	_
ENSG00000273013	2.438432	-1.595162	0.430642	1	CTD-2002J20.1	_
ENSG00000273183	2.438432	-1.595162	0.430642	1	RP11-5C23.2	_
ENSG00000023892	1.546197	-1.432825	0.430642	1	DEF6	_
ENSG00000036530	1.546197	-1.432825	0.430642	1	CYP46A1	K07440
ENSG00000079931	1.546197	-1.432825	0.430642	1	MOXD1	_
ENSG00000101470	1.546197	-1.432825	0.430642	1	TNNC2	K12042
ENSG00000104313	1.546197	-1.432825	0.430642	1	EYA1	K15616
ENSG00000124557	1.546197	-1.432825	0.430642	1	BTN1A1	K06712
ENSG00000127957	1.546197	-1.432825	0.430642	1	PMS2P3	K10858
ENSG00000130948	1.546197	-1.432825	0.430642	1	HSD17B3	K10207
ENSG00000133101	1.546197	-1.432825	0.430642	1	CCNA1	K06627
ENSG00000134594	1.546197	-1.432825	0.430642	1	RAB33A	K07919

ENSG00000137270	1.546197	-1.432825	0.430642	1	GCM1	K12581
ENSG00000139767	1.546197	-1.432825	0.430642	1	SRRM4	_
ENSG00000142173	1.546197	-1.432825	0.430642	1	COL6A2	K06238
ENSG00000145934	1.546197	-1.432825	0.430642	1	TENM2	_
ENSG00000157303	1.546197	-1.432825	0.430642	1	SUSD3	_
ENSG00000159166	1.546197	-1.432825	0.430642	1	LAD1	_
ENSG00000159904	1.546197	-1.432825	0.430642	1	ZNF890P	K09228
ENSG00000162630	1.546197	-1.432825	0.430642	1	B3GALT2	K07820
ENSG00000164309	1.546197	-1.432825	0.430642	1	CMYA5	_
ENSG00000164591	1.546197	-1.432825	0.430642	1	MYOZ3	_
ENSG00000164744	1.546197	-1.432825	0.430642	1	SUN3	_
ENSG00000168263	1.546197	-1.432825	0.430642	1	KCNV2	K04935
ENSG00000168955	1.546197	-1.432825	0.430642	1	TM4SF20	K09228
ENSG00000173567	1.546197	-1.432825	0.430642	1	GPR113	K08456
ENSG00000174827	1.546197	-1.432825	0.430642	1	PDZK1	_
ENSG00000174898	1.546197	-1.432825	0.430642	1	CATSPERD	K16895
ENSG00000175189	1.546197	-1.432825	0.430642	1	INHBC	K04667
ENSG00000179772	1.546197	-1.432825	0.430642	1	FOXS1	K09412
ENSG00000184524	1.546197	-1.432825	0.430642	1	CEND1	_
ENSG00000188234	1.546197	-1.432825	0.430642	1	AGAP4	K12491
ENSG00000196193	1.546197	-1.432825	0.430642	1	AC034229.1	_
ENSG00000196503	1.546197	-1.432825	0.430642	1	ARL9	K07957
ENSG00000197727	1.546197	-1.432825	0.430642	1	AL022341.1	_
ENSG00000198374	1.546197	-1.432825	0.430642	1	HIST1H2AL	K11251
ENSG00000198673	1.546197	-1.432825	0.430642	1	FAM19A2	_
ENSG00000203321	1.546197	-1.432825	0.430642	1	RP11-197P3.4	_
ENSG00000204387	1.546197	-1.432825	0.430642	1	C6orf48	_
ENSG00000221184	1.546197	-1.432825	0.430642	1	MIR1254-1	_
ENSG00000224903	1.546197	-1.432825	0.430642	1	AC005534.8	_
ENSG00000225345	1.546197	-1.432825	0.430642	1	SNX18P3	K17299
ENSG00000225580	1.546197	-1.432825	0.430642	1	RP1-159M24.1	_
ENSG00000225883	1.546197	-1.432825	0.430642	1	BMS1P11	K14569
ENSG00000226833	1.546197	-1.432825	0.430642	1	AC097724.3	_
ENSG00000227113	1.546197	-1.432825	0.430642	1	RP11-460N20.4	_
ENSG00000227158	1.546197	-1.432825	0.430642	1	AC073621.2	K10692
ENSG00000228150	1.546197	-1.432825	0.430642	1	RP11-84A14.4	_
ENSG00000228748	1.546197	-1.432825	0.430642	1	RP13-39P12.3	_
ENSG00000228816	1.546197	-1.432825	0.430642	1	AK3P5	K00944
ENSG00000229020	1.546197	-1.432825	0.430642	1	AKR7A2P1	K15303
ENSG00000229425	1.546197	-1.432825	0.430642	1	AJ006998.2	_
ENSG00000229512	1.546197	-1.432825	0.430642	1	AC068580.5	_
ENSG00000232545	1.546197	-1.432825	0.430642	1	KB-318B8.7	_
ENSG00000232814	1.546197	-1.432825	0.430642	1	COL4A2-AS1	_
ENSG00000233654	1.546197	-1.432825	0.430642	1	AC093388.3	_
ENSG00000234084	1.546197	-1.432825	0.430642	1	RP3-388E23.2	_
ENSG00000236266	1.546197	-1.432825	0.430642	1	RP3-467L1.4	_
ENSG00000236527	1.546197	-1.432825	0.430642	1	ARF4P2	K07939
ENSG00000236670	1.546197	-1.432825	0.430642	1	KRT18P5	K07604
ENSG00000237371	1.546197	-1.432825	0.430642	1	RP13-152O15.5	_
ENSG00000238221	1.546197	-1.432825	0.430642	1	RP11-69L16.4	_
ENSG00000241180	1.546197	-1.432825	0.430642	1	RP11-54O7.2	_
ENSG00000241293	1.546197	-1.432825	0.430642	1	PPATP1	K00764
ENSG00000242094	1.546197	-1.432825	0.430642	1	FOXP1-IT1	_
ENSG00000244124	1.546197	-1.432825	0.430642	1	ATP1B3-AS1	_
ENSG00000244952	1.546197	-1.432825	0.430642	1	RP11-1000B6.5	_
ENSG00000245330	1.546197	-1.432825	0.430642	1	KB-1471A8.1	_
ENSG00000245869	1.546197	-1.432825	0.430642	1	RP11-158I9.5	_
ENSG00000246575	1.546197	-1.432825	0.430642	1	AC093162.5	K09516

ENSG00000249207	1.546197	-1.432825	0.430642	1	RP11-360F5.1	_
ENSG00000250021	1.546197	-1.432825	0.430642	1	C15orf38-AP3S2	K12399
ENSG00000250072	1.546197	-1.432825	0.430642	1	CTC-529P8.1	_
ENSG00000251414	1.546197	-1.432825	0.430642	1	RP11-843P14.1	_
ENSG00000251497	1.546197	-1.432825	0.430642	1	RP11-197N18.7	_
ENSG00000252916	1.546197	-1.432825	0.430642	1	RNU6-762P	_
ENSG00000253304	1.546197	-1.432825	0.430642	1	TMEM200B	_
ENSG00000253483	1.546197	-1.432825	0.430642	1	CTC-756D1.1	K09525
ENSG00000253603	1.546197	-1.432825	0.430642	1	CTA-397H3.3	_
ENSG00000254207	1.546197	-1.432825	0.430642	1	RP11-43A14.1	_
ENSG00000254991	1.546197	-1.432825	0.430642	1	RP13-631K18.3	_
ENSG00000258471	1.546197	-1.432825	0.430642	1	RP11-84C10.4	_
ENSG00000258628	1.546197	-1.432825	0.430642	1	RP11-492D6.3	K17985
ENSG00000259659	1.546197	-1.432825	0.430642	1	RP11-151N17.1	_
ENSG00000259797	1.546197	-1.432825	0.430642	1	RP11-96D1.3	_
ENSG00000260277	1.546197	-1.432825	0.430642	1	RP11-101E7.2	_
ENSG00000260735	1.546197	-1.432825	0.430642	1	RP11-72I8.1	_
ENSG00000261766	1.546197	-1.432825	0.430642	1	RP11-22P6.2	_
ENSG00000261821	1.546197	-1.432825	0.430642	1	CTD-2311M21.3	_
ENSG00000262420	1.546197	-1.432825	0.430642	1	RP11-490O6.2	_
ENSG00000263740	1.546197	-1.432825	0.430642	1	RN7SL4P	_
ENSG00000265136	1.546197	-1.432825	0.430642	1	RP13-638C3.6	K16742
ENSG00000265465	1.546197	-1.432825	0.430642	1	MIR4768	_
ENSG00000266578	1.546197	-1.432825	0.430642	1	RP11-838N2.5	_
ENSG00000266643	1.546197	-1.432825	0.430642	1	MIR3677	_
ENSG00000267505	1.546197	-1.432825	0.430642	1	CTC-296K1.3	_
ENSG00000267507	1.546197	-1.432825	0.430642	1	CTD-2587H24.1	_
ENSG00000267633	1.546197	-1.432825	0.430642	1	CTB-5E10.3	_
ENSG00000268199	1.546197	-1.432825	0.430642	1	CTD-3137H5.1	_
ENSG00000268279	1.546197	-1.432825	0.430642	1	RP11-434D12.1	K10838
ENSG00000269887	1.546197	-1.432825	0.430642	1	RP11-477H21.2	_
ENSG00000269924	1.546197	-1.432825	0.430642	1	RP11-697N18.4	_
ENSG00000272377	1.546197	-1.432825	0.430642	1	RP11-682N22.1	_
ENSG00000272382	1.546197	-1.432825	0.430642	1	CTD-2035E11.4	_
ENSG00000272812	1.546197	-1.432825	0.430642	1	RP5-855D21.3	_
ENSG00000272906	1.546197	-1.432825	0.430642	1	RP11-533E19.7	_
ENSG00000272970	1.546197	-1.432825	0.430642	1	RP11-329B9.4	_
ENSG00000169299	-0.525052	5.394308	0.430652	1	PGM2	K15779
ENSG00000140386	0.539414	3.96902	0.430684	1	SCAPER	_
ENSG00000163539	-0.53104	4.634028	0.430696	1	CLASP2	K16578
ENSG00000140451	-0.532223	4.437989	0.431095	1	PIF1	K15255
ENSG00000066827	0.548736	3.368953	0.431105	1	ZFAT	_
ENSG00000129595	0.538406	3.373389	0.431105	1	EPB41L4A	_
ENSG00000254901	0.536652	3.56367	0.431264	1	MEF2BNB	K15696
ENSG00000108852	0.534711	3.809039	0.431429	1	MPP2	_
ENSG00000156639	0.521815	5.828366	0.431503	1	ZFAND3	K08834
ENSG00000198879	0.521878	5.865031	0.431518	1	SFMBT2	K12606
ENSG00000152147	-0.530756	4.607343	0.431759	1	GEMIN6	K13134
ENSG00000197056	-0.5293	4.73443	0.431792	1	ZMYM1	_
ENSG00000164574	-0.52693	4.952204	0.431843	1	GALNT10	K00710
ENSG00000007314	-0.876415	0.132235	0.431875	1	SCN4A	K04837
ENSG00000165406	-0.876415	0.132235	0.431875	1	8-Mar	K10656
ENSG00000225265	-0.876415	0.132235	0.431875	1	RP11-378J18.3	_
ENSG00000240731	-0.876415	0.132235	0.431875	1	RP5-890O3.9	_
ENSG00000069667	0.875871	-0.061516	0.431875	1	RORA	K08532
ENSG00000111291	0.875871	-0.061516	0.431875	1	GPRC5D	K04621
ENSG00000117983	0.875871	-0.061516	0.431875	1	MUC5B	K13908
ENSG00000121104	0.875871	-0.061516	0.431875	1	FAM117A	_

ENSG00000125531	0.875871	-0.061516	0.431875	1	C20orf195	_
ENSG00000164266	0.875871	-0.061516	0.431875	1	SPINK1	_
ENSG00000169885	0.875871	-0.061516	0.431875	1	CALML6	K02183
ENSG00000169962	0.875871	-0.061516	0.431875	1	TAS1R3	K04626
ENSG00000215394	0.875871	-0.061516	0.431875	1	BMS1P18	_
ENSG00000216331	0.875871	-0.061516	0.431875	1	HIST1H1PS1	_
ENSG00000220472	0.875871	-0.061516	0.431875	1	RP11-69L16.5	K02985
ENSG00000229931	0.875871	-0.061516	0.431875	1	RP1-151F17.1	_
ENSG00000232611	0.875871	-0.061516	0.431875	1	RP11-1114A5.4	_
ENSG00000234742	0.875871	-0.061516	0.431875	1	AC144530.1	K02880
ENSG00000236364	0.875871	-0.061516	0.431875	1	RP11-525G13.2	_
ENSG00000248109	0.875871	-0.061516	0.431875	1	CTC-295J13.3	_
ENSG00000161653	-0.799116	0.08425	0.431875	1	NAGS	K11067
ENSG00000163806	-0.799116	0.08425	0.431875	1	SPDYA	K08694
ENSG00000168676	-0.799116	0.08425	0.431875	1	KCTD19	_
ENSG00000230565	-0.799116	0.08425	0.431875	1	ZNF32-AS2	_
ENSG00000235105	-0.799116	0.08425	0.431875	1	RP11-329A14.1	K17532
ENSG00000241634	-0.799116	0.08425	0.431875	1	RP11-381E24.1	_
ENSG00000242220	-0.799116	0.08425	0.431875	1	TCP10L	_
ENSG00000246640	-0.799116	0.08425	0.431875	1	RP11-1094H24.4	_
ENSG00000257773	-0.799116	0.08425	0.431875	1	ST13P3	K09560
ENSG00000273018	-0.799116	0.08425	0.431875	1	CTD-2303H24.2	_
ENSG00000071073	0.740538	-0.00618	0.431875	1	MGAT4A	K00738
ENSG00000159905	0.740538	-0.00618	0.431875	1	ZNF221	K09228
ENSG00000214940	0.740538	-0.00618	0.431875	1	NPIPA8	K04985
ENSG00000228335	0.740538	-0.00618	0.431875	1	AC073063.10	K18732
ENSG00000233885	0.740538	-0.00618	0.431875	1	YEATS2-AS1	_
ENSG00000262154	0.740538	-0.00618	0.431875	1	LA16c-352F10.1	K03113
ENSG00000267871	0.740538	-0.00618	0.431875	1	CTC-444N24.6	_
ENSG00000136051	-0.520439	6.320799	0.432012	1	KIAA1033	K18465
ENSG00000100068	0.552444	2.528735	0.43206	1	LRP5L	K03068
ENSG00000183674	0.56162	2.847399	0.432247	1	LINC00518	_
ENSG00000068971	0.552833	3.230754	0.432343	1	PPP2R5B	K11584
ENSG00000166821	0.533921	4.064072	0.432349	1	PEX11A	K13351
ENSG00000112208	-0.519559	6.507903	0.432464	1	BAG2	K09556
ENSG00000211455	-0.523819	5.630788	0.432489	1	STK38L	K08790
ENSG00000197930	0.516277	7.237882	0.432621	1	ERO1L	K10950
ENSG00000171310	-0.527276	4.789261	0.43277	1	CHST11	K01017
ENSG00000185129	-0.528923	4.446022	0.43293	1	PURA	_
ENSG00000124160	-0.534489	4.291478	0.433011	1	NCOA5	_
ENSG00000197448	-0.516833	6.843918	0.433077	1	GSTK1	K13299
ENSG00000165030	0.518817	6.123931	0.433146	1	NFIL3	K09059
ENSG00000112290	-0.530212	4.814374	0.433233	1	WASF1	K05753
ENSG00000100101	-0.532124	4.427857	0.433555	1	NOL12	K14851
ENSG00000075643	-0.521627	5.397424	0.433823	1	MOCOS	K15631
ENSG00000121578	-0.521807	5.598643	0.433877	1	B4GALT4	K07969
ENSG00000165801	0.54715	2.953474	0.433945	1	ARHGEF40	_
ENSG00000187239	0.523774	5.286412	0.433949	1	FNBP1	_
ENSG00000136273	-0.541006	3.97261	0.434044	1	HUS1	K10903
ENSG00000178694	-0.566455	2.835549	0.434058	1	NSUN3	_
ENSG00000181016	0.635717	1.386	0.43406	1	LSMEM1	_
ENSG00000249626	-0.630941	1.460585	0.43406	1	RP11-496H1.1	K15425
ENSG00000102100	0.592863	1.405105	0.43406	1	SLC35A2	K15272
ENSG00000269473	0.592863	1.405105	0.43406	1	CTD-2619J13.19	_
ENSG00000269896	0.592863	1.405105	0.43406	1	RP4-740C4.6	_
ENSG00000163933	-0.528553	4.41558	0.434192	1	RFT1	K06316
ENSG00000120756	-0.531501	4.36552	0.434262	1	PLS1	K17275
ENSG00000161955	-0.585607	2.49808	0.434301	1	TNFSF13	K05475

ENSG00000167680	0.583873	2.261748	0.434415	1	SEMA6B	K06842
ENSG00000245614	-0.611264	1.814063	0.434448	1	DDX11-AS1	_
ENSG00000223380	0.594808	2.024085	0.43449	1	SEC22B	_
ENSG00000261505	0.594808	2.024085	0.43449	1	LA16c-358B7.3	_
ENSG00000137806	-0.529506	4.495372	0.434673	1	NDUFAF1	K18159
ENSG00000106028	-0.52459	5.103688	0.434981	1	SSBP1	_
ENSG00000136100	-0.519013	5.610335	0.435234	1	VPS36	K12190
ENSG00000099797	0.515892	6.345524	0.435302	1	TECR	K10258
ENSG00000273344	0.547547	3.046656	0.435437	1	PAXIP1-AS1	_
ENSG00000237765	-0.525324	4.648032	0.43549	1	FAM200B	_
ENSG00000186480	-0.517583	5.756709	0.435679	1	INSIG1	_
ENSG00000166401	-0.55612	2.797907	0.435981	1	SERPINB8	K13963
ENSG00000163320	-0.517281	5.871766	0.436095	1	CGGBP1	_
ENSG00000253954	-0.557399	2.630823	0.43632	1	HMGN1P38	K11299
ENSG00000182481	0.525282	4.65683	0.436505	1	KPNA2	K15043
ENSG00000173821	0.51177	7.281181	0.436575	1	RNF213	K03327
ENSG00000261526	0.580867	2.409158	0.436716	1	CTB-31O20.2	_
ENSG00000169679	-0.51145	7.396045	0.436921	1	BUB1	K02178
ENSG00000127481	0.511194	7.344218	0.437005	1	UBR4	K10691
ENSG00000101079	0.515201	5.604531	0.437161	1	NDRG3	K17985
ENSG00000133460	0.552658	2.871806	0.437536	1	SLC2A11	K08148
ENSG00000196159	-0.552891	3.342712	0.437553	1	FAT4	K16669
ENSG00000143507	-0.603303	1.990601	0.437711	1	DUSP10	K04459
ENSG00000180917	-0.532583	4.074787	0.437884	1	CMTR2	K14590
ENSG00000117153	-0.520646	4.712861	0.437903	1	KLHL12	K10450
ENSG00000134321	-1.161914	-0.282393	0.438083	1	RSAD2	K15045
ENSG00000157064	-1.161914	-0.282393	0.438083	1	NMNAT2	K06210
ENSG00000166246	-1.161914	-0.282393	0.438083	1	C16orf71	_
ENSG00000239704	-1.161914	-0.282393	0.438083	1	CDRT4	_
ENSG00000261575	-1.161914	-0.282393	0.438083	1	RP11-259G18.1	_
ENSG00000140678	1.021395	-0.417965	0.438083	1	ITGAX	K06462
ENSG00000167311	1.021395	-0.417965	0.438083	1	ART5	K00775
ENSG00000178429	1.021395	-0.417965	0.438083	1	RPS3AP5	K02984
ENSG00000204956	1.021395	-0.417965	0.438083	1	PCDHGA1	K16495
ENSG00000215126	1.021395	-0.417965	0.438083	1	CBWD7	_
ENSG00000247982	1.021395	-0.417965	0.438083	1	LINC00926	_
ENSG00000271643	1.021395	-0.417965	0.438083	1	RP11-10C24.3	_
ENSG00000121101	0.832987	-0.346466	0.438083	1	TEX14	K17540
ENSG00000143554	0.832987	-0.346466	0.438083	1	SLC27A3	K08772
ENSG00000187994	0.832987	-0.346466	0.438083	1	RINL	_
ENSG00000204282	0.832987	-0.346466	0.438083	1	TNRC6C-AS1	_
ENSG00000225077	0.832987	-0.346466	0.438083	1	LINC00337	_
ENSG00000226281	0.832987	-0.346466	0.438083	1	RP1-80N2.2	_
ENSG00000229951	0.832987	-0.346466	0.438083	1	AC104695.3	_
ENSG00000230979	0.832987	-0.346466	0.438083	1	AC079250.1	K02882
ENSG00000259556	0.832987	-0.346466	0.438083	1	RP11-56B16.2	K15177
ENSG00000260236	0.832987	-0.346466	0.438083	1	RP11-708J19.1	_
ENSG00000260750	0.832987	-0.346466	0.438083	1	RP11-482M8.1	_
ENSG00000264918	0.832987	-0.346466	0.438083	1	AC005229.1	_
ENSG00000270607	0.832987	-0.346466	0.438083	1	RP11-359E10.1	_
ENSG00000173163	-0.539079	3.517175	0.438166	1	COMMD1	_
ENSG00000042317	-0.665103	1.712497	0.438288	1	SPATA7	K19655
ENSG00000116977	-0.665103	1.712497	0.438288	1	LGALS8	K06832
ENSG00000182405	-0.665103	1.712497	0.438288	1	PGBD4	_
ENSG00000232561	0.621713	1.644884	0.438288	1	GTF2IP1	K03121
ENSG00000268885	0.621713	1.644884	0.438288	1	AC026740.1	_
ENSG00000134668	0.712226	0.74652	0.438358	1	SPOCD1	K17596
ENSG00000156345	0.712226	0.74652	0.438358	1	CDK20	K08817

ENSG00000166676	0.712226	0.74652	0.438358	1	TVP23A	_
ENSG00000226803	0.712226	0.74652	0.438358	1	RP11-203B9.4	_
ENSG00000262089	0.712226	0.74652	0.438358	1	RP11-589P10.5	_
ENSG00000130203	0.64241	0.777194	0.438358	1	APOE	K17985
ENSG00000006459	0.525743	4.103025	0.438488	1	KDM7A	K11445
ENSG00000155100	-0.515578	5.270536	0.438495	1	OTUD6B	K18342
ENSG00000110031	0.605558	1.284757	0.438759	1	LPXN	_
ENSG00000075240	0.526995	4.351395	0.438812	1	GRAMD4	_
ENSG00000125703	-0.516099	5.104944	0.438875	1	ATG4C	K08342
ENSG00000166986	-0.508365	8.078617	0.438915	1	MARS	K01874
ENSG00000184500	0.515356	4.786743	0.439035	1	PROS1	K03908
ENSG00000170632	-0.515801	5.526436	0.439214	1	ARMC10	K10279
ENSG00000113594	-0.509732	6.936154	0.439219	1	LIFR	_
ENSG00000268573	0.564964	2.359454	0.439326	1	RP11-158H5.7	_
ENSG00000196123	-0.55358	3.206933	0.439496	1	KIAA0895L	_
ENSG00000107833	-0.511527	6.29096	0.439731	1	NPM3	K11278
ENSG00000118894	-0.530274	3.850316	0.439739	1	FAM86A	_
ENSG00000109762	0.510468	5.562361	0.440152	1	SNX25	K17887
ENSG00000114353	0.571841	2.138603	0.440198	1	GNAI2	_
ENSG00000182704	0.508529	6.719998	0.440411	1	TSKU	K08130
ENSG00000137133	-0.524937	4.621607	0.440454	1	HINT2	_
ENSG00000089048	-0.520637	4.861768	0.440569	1	ESF1	_
ENSG00000124587	0.511223	5.887466	0.440578	1	PEX6	K13339
ENSG00000160746	-0.528799	4.123286	0.440761	1	ANO10	_
ENSG00000132535	0.528361	4.11065	0.440761	1	DLG4	K11828
ENSG00000118246	-0.514938	5.408796	0.440761	1	FASTKD2	K18190
ENSG00000239911	-0.57149	2.528413	0.441081	1	PRKAG2-AS1	_
ENSG00000109458	-0.545527	3.359753	0.441399	1	GAB1	K09593
ENSG00000225031	0.528081	3.864457	0.441404	1	EIF4BP7	K03258
ENSG00000249815	0.528081	3.864457	0.441404	1	RP11-269F21.3	_
ENSG00000104517	0.505851	7.657614	0.441427	1	UBR5	K10593
ENSG00000130803	0.511825	5.149483	0.441672	1	ZNF317	K09228
ENSG00000078237	-0.516467	4.903293	0.44169	1	C12orf5	K14634
ENSG00000066629	0.515351	4.95205	0.441741	1	EML1	K18595
ENSG00000137269	-0.52975	3.804034	0.441876	1	LRRC1	K16175
ENSG00000204856	-0.515692	4.866259	0.441929	1	FAM216A	K06883
ENSG00000140416	-0.507215	6.716616	0.441934	1	TPM1	K10373
ENSG00000166788	-0.527833	4.045875	0.44206	1	SAAL1	_
ENSG00000165948	0.523991	4.138399	0.44213	1	IFI27L1	_
ENSG00000174720	-0.513026	5.543424	0.442312	1	LARP7	K15191
ENSG00000149418	0.528505	3.71191	0.442399	1	ST14	K08670
ENSG00000088543	1.324815	-0.890764	0.442579	1	C3orf18	_
ENSG00000103740	1.324815	-0.890764	0.442579	1	ACSBG1	K15013
ENSG00000106633	1.324815	-0.890764	0.442579	1	GCK	K12407
ENSG00000108950	1.324815	-0.890764	0.442579	1	FAM20A	_
ENSG00000127241	1.324815	-0.890764	0.442579	1	MASP1	K03992
ENSG00000135951	1.324815	-0.890764	0.442579	1	TSGA10	_
ENSG00000156886	1.324815	-0.890764	0.442579	1	ITGAD	K06594
ENSG00000163352	1.324815	-0.890764	0.442579	1	LENEP	_
ENSG00000189431	1.324815	-0.890764	0.442579	1	RASSF10	K09856
ENSG00000196890	1.324815	-0.890764	0.442579	1	HIST3H2BB	K11252
ENSG00000204659	1.324815	-0.890764	0.442579	1	CBY3	_
ENSG00000205838	1.324815	-0.890764	0.442579	1	TTC23L	_
ENSG00000219470	1.324815	-0.890764	0.442579	1	RP3-337H4.6	K02981
ENSG00000224559	1.324815	-0.890764	0.442579	1	LINC01087	_
ENSG00000229525	1.324815	-0.890764	0.442579	1	AC053503.4	_
ENSG00000241269	1.324815	-0.890764	0.442579	1	AC093620.5	_
ENSG00000243789	1.324815	-0.890764	0.442579	1	JMJD7	K16342

ENSG00000250616	1.324815	-0.890764	0.442579	1	RP11-455F5.3	_
ENSG00000251102	1.324815	-0.890764	0.442579	1	CTBP2P4	K04496
ENSG00000251580	1.324815	-0.890764	0.442579	1	RP11-539L10.3	_
ENSG00000260490	1.324815	-0.890764	0.442579	1	RP11-265N6.3	K12757
ENSG00000261441	1.324815	-0.890764	0.442579	1	RP11-217B1.2	_
ENSG00000264548	1.324815	-0.890764	0.442579	1	RP13-516M14.2	_
ENSG00000268225	1.324815	-0.890764	0.442579	1	CTD-2331H12.5	K09228
ENSG00000268379	1.324815	-0.890764	0.442579	1	CTC-360J11.4	K17659
ENSG00000269952	1.324815	-0.890764	0.442579	1	RP11-324I22.3	_
ENSG00000273186	1.324815	-0.890764	0.442579	1	RP11-339B21.10	_
ENSG00000273243	1.324815	-0.890764	0.442579	1	CTA-217C2.2	_
ENSG00000068985	-1.249252	-0.666575	0.442579	1	PAGE1	_
ENSG00000109743	-1.249252	-0.666575	0.442579	1	BST1	K18152
ENSG00000110025	-1.249252	-0.666575	0.442579	1	SNX15	K17927
ENSG00000123201	-1.249252	-0.666575	0.442579	1	GUCY1B2	K12319
ENSG00000134548	-1.249252	-0.666575	0.442579	1	C12orf39	_
ENSG00000135437	-1.249252	-0.666575	0.442579	1	RDH5	K00061
ENSG00000157992	-1.249252	-0.666575	0.442579	1	KRTCAP3	_
ENSG00000169403	-1.249252	-0.666575	0.442579	1	PTAFR	K04279
ENSG00000176054	-1.249252	-0.666575	0.442579	1	RPL23P2	K02894
ENSG00000176681	-1.249252	-0.666575	0.442579	1	LRRC37A	K04613
ENSG00000184730	-1.249252	-0.666575	0.442579	1	APOBR	_
ENSG00000189221	-1.249252	-0.666575	0.442579	1	MAOA	K00274
ENSG00000197620	-1.249252	-0.666575	0.442579	1	CXorf40A	_
ENSG00000204771	-1.249252	-0.666575	0.442579	1	CTD-3232M19.2	_
ENSG00000213976	-1.249252	-0.666575	0.442579	1	CTD-2561J22.2	K09228
ENSG00000220157	-1.249252	-0.666575	0.442579	1	HNRNPA1P12	K12741
ENSG00000225783	-1.249252	-0.666575	0.442579	1	MIAT	_
ENSG00000235385	-1.249252	-0.666575	0.442579	1	GS1-600G8.5	_
ENSG00000237074	-1.249252	-0.666575	0.442579	1	RP11-6J21.2	_
ENSG00000243406	-1.249252	-0.666575	0.442579	1	MRPS31P5	K17410
ENSG00000245156	-1.249252	-0.666575	0.442579	1	RP11-867G23.3	_
ENSG00000253341	-1.249252	-0.666575	0.442579	1	RP11-730G20.2	K13162
ENSG00000256950	-1.249252	-0.666575	0.442579	1	RP11-87C12.2	K06693
ENSG00000258790	-1.249252	-0.666575	0.442579	1	KIAA0391	K17655
ENSG00000259845	-1.249252	-0.666575	0.442579	1	HERC2P10	K10595
ENSG00000260591	-1.249252	-0.666575	0.442579	1	RP11-817O13.6	_
ENSG00000260682	-1.249252	-0.666575	0.442579	1	7SK	_
ENSG00000260920	-1.249252	-0.666575	0.442579	1	RP1-228H13.5	_
ENSG00000263874	-1.249252	-0.666575	0.442579	1	LINC00672	_
ENSG00000269815	-1.249252	-0.666575	0.442579	1	CTD-2278I10.4	_
ENSG00000270589	-1.249252	-0.666575	0.442579	1	RP11-348N5.7	_
ENSG00000271769	-1.249252	-0.666575	0.442579	1	RP11-216P16.2	_
ENSG00000272769	-1.249252	-0.666575	0.442579	1	RP11-725P16.2	_
ENSG00000272788	-1.249252	-0.666575	0.442579	1	RP11-674N23.4	_
ENSG00000273338	-1.249252	-0.666575	0.442579	1	RP11-386I14.4	_
ENSG00000073067	1.014574	-0.790856	0.442579	1	CYP2W1	K07423
ENSG00000080644	1.014574	-0.790856	0.442579	1	CHRNA3	K04805
ENSG00000081181	1.014574	-0.790856	0.442579	1	ARG2	K01476
ENSG00000134042	1.014574	-0.790856	0.442579	1	MRO	_
ENSG00000139329	1.014574	-0.790856	0.442579	1	LUM	K08122
ENSG00000159712	1.014574	-0.790856	0.442579	1	ANKRD18CP	K17299
ENSG00000166482	1.014574	-0.790856	0.442579	1	MFAP4	_
ENSG00000184544	1.014574	-0.790856	0.442579	1	DHRS7C	K11167
ENSG00000185101	1.014574	-0.790856	0.442579	1	ANO9	K19503
ENSG00000186684	1.014574	-0.790856	0.442579	1	CYP27C1	K17951
ENSG00000187260	1.014574	-0.790856	0.442579	1	WDR86	_
ENSG00000213906	1.014574	-0.790856	0.442579	1	LTB4R2	K04297

ENSG00000226029	1.014574	-0.790856	0.442579	1	RP4-798A10.2	_
ENSG00000228492	1.014574	-0.790856	0.442579	1	RAB11FIP1P1	K12484
ENSG00000240668	1.014574	-0.790856	0.442579	1	KRT8P36	K07605
ENSG00000249715	1.014574	-0.790856	0.442579	1	FER1L5	K02927
ENSG00000251994	1.014574	-0.790856	0.442579	1	RNU2-27P	_
ENSG00000255729	1.014574	-0.790856	0.442579	1	AC005618.1	_
ENSG00000255769	1.014574	-0.790856	0.442579	1	RP11-152F13.3	K08622
ENSG00000258759	1.014574	-0.790856	0.442579	1	RP11-1012A1.7	K09529
ENSG00000260495	1.014574	-0.790856	0.442579	1	RP11-55K13.1	_
ENSG00000260808	1.014574	-0.790856	0.442579	1	CTD-2007L18.5	_
ENSG00000261713	1.014574	-0.790856	0.442579	1	SSTR5-AS1	_
ENSG00000266507	1.014574	-0.790856	0.442579	1	MIR4479	_
ENSG00000267141	1.014574	-0.790856	0.442579	1	CTB-31O20.8	_
ENSG00000272482	1.014574	-0.790856	0.442579	1	RP11-474O21.5	_
ENSG00000272896	1.014574	-0.790856	0.442579	1	RP11-216L13.17	_
ENSG00000273343	1.014574	-0.790856	0.442579	1	XXbac-B444P24.13	_
ENSG00000183929	0.634443	1.543356	0.442635	1	DUSP5P1	K04459
ENSG00000112294	-0.621798	1.612621	0.442635	1	ALDH5A1	K00139
ENSG00000101400	0.511372	5.04818	0.44277	1	SNTA1	_
ENSG00000198604	-0.509161	5.987328	0.443229	1	BAZ1A	K11655
ENSG00000185269	0.577985	2.067146	0.443513	1	NOTUM	_
ENSG00000175792	-0.504662	7.04488	0.443567	1	RUVBL1	K04499
ENSG00000198000	-0.506354	6.370864	0.443737	1	NOL8	_
ENSG00000188818	0.520421	4.383377	0.443772	1	ZDHHC11	K18932
ENSG00000048162	-0.50693	6.071719	0.443803	1	NOP16	_
ENSG00000162384	-0.517938	4.213265	0.44387	1	C1orf123	_
ENSG00000054654	0.505956	6.131446	0.443879	1	SYNE2	K19346
ENSG00000104427	-0.522665	4.002775	0.443994	1	ZC2HC1A	_
ENSG00000226381	-0.683529	1.218259	0.444231	1	RP11-119F19.2	_
ENSG00000236360	-0.683529	1.218259	0.444231	1	RP11-334A14.2	K07830
ENSG00000235231	0.672957	1.130437	0.444231	1	AP000351.10	K10028
ENSG00000232233	-0.649716	1.197032	0.444231	1	RP11-573D15.2	_
ENSG00000203761	0.620808	1.153537	0.444231	1	MSTO2P	_
ENSG00000140406	-0.518903	4.618016	0.444307	1	MESDC1	_
ENSG00000116191	-0.512963	5.152465	0.444342	1	RALGPS2	_
ENSG00000105865	-0.531227	3.938642	0.444551	1	DUS4L	K05545
ENSG00000160948	-0.526364	3.93573	0.444551	1	VPS28	K12184
ENSG00000002549	-0.509902	5.480132	0.444596	1	LAP3	K11142
ENSG00000272325	-0.510377	5.289675	0.444624	1	NUDT3	K07766
ENSG00000165506	-0.508628	5.14382	0.4448	1	DNAAF2	K19751
ENSG00000120992	-0.502211	7.432259	0.445168	1	LYPLA1	K06128
ENSG00000008405	-0.508381	5.5453	0.445205	1	CRY1	K02295
ENSG00000119729	0.50341	6.608625	0.445247	1	RHOQ	K07194
ENSG00000139146	-0.502099	7.414672	0.445293	1	FAM60A	_
ENSG00000104998	-0.510295	4.949328	0.445321	1	IL27RA	K19598
ENSG00000094631	-0.812766	0.672515	0.445397	1	HDAC6	K11407
ENSG00000254719	-0.812766	0.672515	0.445397	1	RP11-351I24.3	K04545
ENSG00000087303	-0.762437	0.640598	0.445397	1	NID2	K06826
ENSG00000224699	0.752973	0.552303	0.445397	1	LAMTOR5-AS1	_
ENSG00000129749	0.671297	0.587699	0.445397	1	CHRNA10	K04811
ENSG00000144214	0.671297	0.587699	0.445397	1	LYG1	_
ENSG00000233175	0.671297	0.587699	0.445397	1	CTD-2020K17.3	_
ENSG00000251257	0.671297	0.587699	0.445397	1	CTD-2263F21.1	_
ENSG00000268030	0.671297	0.587699	0.445397	1	AC005253.2	_
ENSG00000272808	0.671297	0.587699	0.445397	1	RP11-66B24.7	_
ENSG00000164077	-0.553709	3.183002	0.445448	1	MON1A	K01557
ENSG00000183718	0.531451	3.578532	0.445569	1	TRIM52	K12025
ENSG00000224259	-0.521409	4.401104	0.44564	1	LINC01133	_

ENSG00000188312	0.530874	3.419387	0.445834	1	CENPP	K11508
ENSG00000148690	-0.510645	4.963424	0.446062	1	FRA10AC1	K13121
ENSG00000153291	-0.520241	4.118175	0.446195	1	SLC25A27	K15112
ENSG00000196155	-0.515894	4.467863	0.446234	1	PLEKHG4	_
ENSG00000075131	-0.513433	4.504948	0.446511	1	TIPIN	K10904
ENSG00000079950	-0.508239	4.869232	0.446542	1	STX7	K08488
ENSG00000173621	-0.501351	6.813959	0.447003	1	LRFN4	K16357
ENSG00000064012	-0.508788	5.149996	0.447028	1	CASP8	K04398
ENSG00000024422	0.50049	6.999597	0.447141	1	EHD2	K12469
ENSG00000135407	0.607648	1.452617	0.447595	1	AVIL	K08017
ENSG00000261254	0.607648	1.452617	0.447595	1	RP4-714D9.5	_
ENSG00000130522	-0.500441	6.865905	0.447719	1	JUND	K04449
ENSG00000183665	-0.528381	3.68988	0.447862	1	TRMT12	K07055
ENSG00000181467	-0.505084	5.593222	0.448433	1	RAP2B	K07838
ENSG00000111666	-0.503547	5.592316	0.448433	1	CHPT1	K00994
ENSG00000187742	-0.511096	4.541104	0.448467	1	SECISBP2	K19539
ENSG00000139131	-0.50296	5.622829	0.448642	1	YARS2	K01866
ENSG00000076201	0.507746	4.789954	0.448718	1	PTPN23	K18040
ENSG00000143669	0.51457	4.008035	0.448855	1	LYST	_
ENSG00000117862	-0.498355	7.070198	0.449222	1	TXNDC12	K05360
ENSG00000140400	-0.504643	5.218584	0.449623	1	MAN2C1	K01191
ENSG00000126814	-0.509716	4.722792	0.449638	1	TRMT5	K15429
ENSG00000128573	-0.559854	2.358445	0.449714	1	FOXP2	K09409
ENSG00000178977	0.59879	1.700729	0.449741	1	LINC00324	_
ENSG00000243716	0.518322	3.761468	0.449889	1	NPIPB5	K08873
ENSG00000173334	-0.501955	5.859028	0.450122	1	TRIB1	K08814
ENSG00000075945	-0.507032	5.161043	0.450341	1	KIFAP3	_
ENSG00000185088	-0.497861	6.663254	0.450497	1	RPS27L	K02978
ENSG00000175745	-0.505054	5.482091	0.450659	1	NR2F1	K08547
ENSG00000270172	-0.535912	2.991889	0.450668	1	RP5-1024G6.8	_
ENSG00000272579	-0.535912	2.991889	0.450668	1	RP11-101E13.5	_
ENSG00000173706	0.49563	7.649046	0.450685	1	HEG1	_
ENSG00000146856	0.639473	1.00929	0.450687	1	AGBL3	_
ENSG00000185504	-0.505679	4.953558	0.450959	1	C17orf70	K10993
ENSG00000163743	-0.513014	3.983427	0.45102	1	RCHY1	K10144
ENSG00000164163	-0.496845	6.349586	0.451096	1	ABCE1	K06174
ENSG00000232445	-0.536478	3.072305	0.45117	1	RP11-132A1.4	_
ENSG00000127954	0.495667	6.999233	0.451519	1	STEAP4	K16467
ENSG00000198890	-0.510903	4.656665	0.451525	1	PRMT6	K11437
ENSG00000182700	0.523684	3.144702	0.451611	1	IGIP	_
ENSG00000164764	-0.556595	2.733388	0.451945	1	SBSPON	K16467
ENSG00000133243	-0.495238	6.498333	0.452247	1	BTBD2	K10477
ENSG00000152620	-0.495377	6.881225	0.452283	1	NADK2	K00858
ENSG00000248835	0.587621	2.097669	0.452285	1	AL357673.1	K12581
ENSG00000223509	-0.579833	2.137088	0.452285	1	RP11-632K20.7	_
ENSG00000143156	-0.509409	4.473745	0.452318	1	NME7	K00940
ENSG00000196923	-0.495988	6.608261	0.452329	1	PDLIM7	_
ENSG00000153162	0.495183	6.672905	0.452566	1	BMP6	K16620
ENSG00000117602	-0.514686	3.84099	0.452929	1	RCAN3	K17905
ENSG00000134056	-0.514686	3.84099	0.452929	1	MRPS36	K17414
ENSG00000173209	-0.49827	5.823158	0.452991	1	AHSA2	_
ENSG00000131844	-0.497211	6.223503	0.453022	1	MCCC2	K01969
ENSG00000172000	0.523628	3.020446	0.45328	1	ZNF556	K09228
ENSG00000176371	0.523628	3.020446	0.45328	1	ZSCAN2	K09488
ENSG00000242588	0.621185	1.33623	0.453309	1	RP11-274B21.1	K08864
ENSG00000254996	0.621185	1.33623	0.453309	1	ANKHD1-EIF4EBP3	K16726
ENSG00000259802	0.621185	1.33623	0.453309	1	CTD-2256P15.2	_
ENSG00000104946	0.503956	4.791556	0.453578	1	TBC1D17	_

ENSG00000164548	-0.496447	6.081466	0.453601	1	TRA2A	K12897
ENSG00000272269	-0.545426	3.128726	0.453613	1	RP11-500C11.3	_
ENSG00000117308	-0.49989	5.192487	0.453688	1	GALE	K01784
ENSG00000185630	-0.501123	5.031884	0.453718	1	PBX1	_
ENSG00000115946	-0.499135	5.549046	0.453909	1	PNO1	K11884
ENSG00000130779	0.492779	7.129871	0.453946	1	CLIP1	K10421
ENSG00000131788	0.809061	0.32812	0.454082	1	PIAS3	K16064
ENSG00000144285	0.809061	0.32812	0.454082	1	SCN1A	K04833
ENSG00000232628	0.809061	0.32812	0.454082	1	RP11-365O16.3	_
ENSG00000272853	0.809061	0.32812	0.454082	1	RP11-398C13.6	_
ENSG00000147650	-0.732038	0.453823	0.454082	1	LRP12	K12581
ENSG00000228519	-0.732038	0.453823	0.454082	1	RP11-298C3.2	K15306
ENSG00000270175	-0.732038	0.453823	0.454082	1	RP11-793H13.11	_
ENSG00000105270	0.710667	0.369847	0.454082	1	CLIP3	K10423
ENSG00000205744	0.710667	0.369847	0.454082	1	DENND1C	_
ENSG00000227630	0.710667	0.369847	0.454082	1	LINC01132	_
ENSG00000250896	0.710667	0.369847	0.454082	1	RNPS1P1	K14325
ENSG00000254416	0.710667	0.369847	0.454082	1	RP11-347E10.1	_
ENSG00000261542	0.710667	0.369847	0.454082	1	RP11-16E18.3	_
ENSG00000272198	0.710667	0.369847	0.454082	1	RP11-309G3.3	_
ENSG00000004399	0.492405	7.248798	0.454114	1	PLXND1	K06822
ENSG00000033867	-0.495703	5.747802	0.454138	1	SLC4A7	K13858
ENSG00000161888	0.497166	5.770729	0.45422	1	SPC24	K11549
ENSG00000064490	-0.49861	5.434314	0.454433	1	RFXANK	K08062
ENSG00000205981	-0.49861	5.434314	0.454433	1	DNAJC19	K09539
ENSG00000152642	-0.502932	5.090862	0.454474	1	GPD1L	K00006
ENSG00000136875	-0.495143	6.163728	0.454687	1	PRPF4	K12662
ENSG00000128266	0.60931	1.603217	0.454799	1	GNAZ	_
ENSG00000168453	0.492065	6.785953	0.455185	1	HR	K00478
ENSG00000267575	-0.535274	2.906702	0.455261	1	CTC-459F4.3	_
ENSG00000115827	-0.516754	3.682914	0.455268	1	DCAF17	_
ENSG00000138641	0.514374	3.671324	0.455268	1	HERC3	K10614
ENSG00000184792	-0.494485	6.086631	0.455337	1	OSBP2	_
ENSG00000164758	-0.504992	4.653162	0.455354	1	MED30	K15143
ENSG00000112996	-0.490185	7.911595	0.455548	1	MRPS30	K17409
ENSG00000137038	-0.498777	5.585043	0.455644	1	TMEM261	_
ENSG00000107862	0.493742	6.248777	0.455679	1	GBF1	K18443
ENSG00000169220	0.527259	3.073437	0.455718	1	RGS14	K17706
ENSG00000253683	-0.608375	1.875361	0.455728	1	CTB-79E8.3	K07604
ENSG00000273108	-0.587365	1.862338	0.455728	1	RP11-416N2.4	_
ENSG00000152952	0.489055	8.634834	0.455939	1	PLOD2	K13645
ENSG00000169439	-0.491595	6.741831	0.456026	1	SDC2	K16336
ENSG00000160271	0.498439	5.011892	0.456063	1	RALGDS	K08732
ENSG00000105755	-0.495097	5.609451	0.456091	1	ETHE1	K17725
ENSG00000070778	-0.530658	3.329283	0.456207	1	PTPN21	K18025
ENSG00000137547	-0.493035	6.414132	0.456208	1	MRPL15	K02876
ENSG00000132436	-0.498536	5.326118	0.456537	1	FIGNL1	K12196
ENSG00000181524	0.513767	3.586142	0.456645	1	RPL24P4	K02896
ENSG00000182973	-0.494623	5.417005	0.456656	1	CNOT10	K12607
ENSG00000198466	0.502822	4.461362	0.456669	1	ZNF587	K09228
ENSG00000126217	0.49096	6.44769	0.456711	1	MCF2L	_
ENSG00000162702	-0.492872	5.816192	0.456759	1	ZNF281	_
ENSG00000145029	-0.561267	2.223748	0.456779	1	NICN1	K16607
ENSG00000104907	-0.49005	7.050894	0.456826	1	TRMT1	K00555
ENSG00000188486	-0.4906	6.751208	0.456915	1	H2AFX	K11251
ENSG00000143224	-0.509351	4.021632	0.457162	1	PPOX	K00231
ENSG00000222011	-0.558335	2.520358	0.457328	1	FAM185A	_
ENSG00000139182	0.491897	5.719091	0.457381	1	CLSTN3	_

ENSG00000179950	0.504636	4.338586	0.457455	1	PUF60	K12838
ENSG00000213923	0.487783	8.043207	0.457497	1	CSNK1E	K08960
ENSG00000163659	-0.490878	6.158234	0.457675	1	TIPARP	K15259
ENSG00000100364	0.488876	6.946367	0.457815	1	KIAA0930	_
ENSG00000160124	-0.493423	5.285176	0.457976	1	CCDC58	K16741
ENSG00000218283	0.489351	6.600446	0.458049	1	MORF4L1P1	K11339
ENSG00000185567	0.48753	7.588175	0.45816	1	AHNAK2	_
ENSG00000196843	0.505647	3.894572	0.458325	1	ARID5A	_
ENSG00000168970	-0.722021	0.90035	0.45842	1	JMJD7-PLA2G4B	K16342
ENSG00000198576	-0.722021	0.90035	0.45842	1	ARC	K15867
ENSG00000120278	0.662847	0.849152	0.45842	1	PLEKHG1	_
ENSG00000252645	0.662847	0.849152	0.45842	1	RNU7-111P	_
ENSG00000142634	0.487499	7.284457	0.458561	1	EFHD2	_
ENSG00000064932	0.487166	7.394611	0.458723	1	SBNO2	_
ENSG00000156162	-0.490794	5.49282	0.45935	1	DPY19L4	_
ENSG00000167552	0.497524	4.232784	0.459416	1	TUBA1A	K07374
ENSG00000064933	-0.495548	4.933619	0.459734	1	PMS1	K10864
ENSG00000226137	-0.512287	3.481879	0.459908	1	BAIAP2-AS1	_
ENSG00000106526	0.637339	1.209696	0.459963	1	ACTR3C	K18584
ENSG00000197409	0.637339	1.209696	0.459963	1	HIST1H3D	K11253
ENSG00000232060	0.637339	1.209696	0.459963	1	RP11-255N24.4	_
ENSG00000163354	-0.610298	1.270272	0.459963	1	DCST2	_
ENSG00000141562	0.488418	6.272837	0.460264	1	NARF	_
ENSG00000113845	-0.4869	6.743074	0.460327	1	TIMMDC1	_
ENSG00000188917	-0.502506	4.227229	0.460463	1	TRMT2B	K15331
ENSG00000271576	0.621478	1.498672	0.460565	1	RP11-486G15.2	_
ENSG00000116406	-0.499221	4.740866	0.46063	1	EDEM3	K10086
ENSG00000162009	0.538904	2.281632	0.460755	1	SSTR5	K04221
ENSG00000135899	-0.566984	1.78666	0.460787	1	SP110	_
ENSG00000183496	-0.566984	1.78666	0.460787	1	MEX3B	K15686
ENSG00000148290	-0.544765	2.16544	0.460817	1	SURF1	K14998
ENSG00000224420	-0.544765	2.16544	0.460817	1	ADM5	_
ENSG00000117620	-0.486452	6.681741	0.460876	1	SLC35A3	K15272
ENSG00000135778	-0.491935	5.29524	0.460966	1	NTPCR	K06928
ENSG00000026103	-0.493989	4.89	0.461088	1	FAS	K04390
ENSG00000125772	0.487757	5.643727	0.461216	1	GPCPD1	K18695
ENSG00000124532	-0.488708	5.992185	0.46126	1	MRS2	K16075
ENSG00000069399	0.489701	5.518922	0.461457	1	BCL3	K09258
ENSG00000153714	0.502823	4.155952	0.461618	1	LURAP1L	_
ENSG00000108561	-0.48289	8.767686	0.461626	1	C1QBP	K15414
ENSG00000151502	0.491948	5.074548	0.461928	1	VPS26B	K18466
ENSG00000182134	0.509051	3.217582	0.462139	1	TDRKH	K18406
ENSG00000122912	0.506269	3.777731	0.462487	1	SLC25A16	K15084
ENSG00000236287	-0.484234	6.821008	0.462613	1	ZBED5	_
ENSG00000163138	-0.516824	2.980364	0.462737	1	PACRGL	K10408
ENSG00000122483	-0.489878	5.336979	0.46283	1	CCDC18	K14825
ENSG00000112033	0.501902	4.095565	0.462871	1	PPARD	K04504
ENSG00000125875	0.494384	4.926325	0.462934	1	TBC1D20	K17985
ENSG00000242539	-0.535044	2.877145	0.462981	1	AC007620.3	_
ENSG00000100209	-0.493759	4.405057	0.463011	1	HSCB	K04082
ENSG00000173846	-0.50609	3.497379	0.463328	1	PLK3	K08862
ENSG00000174791	-0.484607	5.921032	0.463665	1	RIN1	K17638
ENSG00000205403	0.498009	4.123597	0.463909	1	CFI	K01333
ENSG00000032389	-0.492773	4.637362	0.463928	1	TSSC1	_
ENSG00000100307	0.494506	4.035293	0.464235	1	CBX7	K11454
ENSG00000245937	-0.51308	3.206109	0.464307	1	CTC-228N24.3	_
ENSG00000104524	-0.490484	5.045176	0.46439	1	PYCRL	K00286
ENSG00000213123	-0.528166	3.118293	0.464616	1	TCTEX1D2	_

ENSG00000087903	0.507743	3.099713	0.464616	1	RFX2	K09173
ENSG00000168291	-0.482301	6.63497	0.464812	1	PDHB	K00162
ENSG00000185641	0.487247	5.320942	0.464824	1	CTD-2287O16.1	K02975
ENSG00000163029	-0.487429	5.407755	0.464937	1	SMC6	K02159
ENSG00000137871	-0.489529	4.901746	0.464993	1	ZNF280D	_
ENSG00000172803	-0.949783	0.178499	0.465039	1	SNX32	K17920
ENSG00000240399	-0.949783	0.178499	0.465039	1	RP1-228P16.1	K02922
ENSG00000241057	-0.949783	0.178499	0.465039	1	AC004985.12	K17630
ENSG00000162645	0.767521	0.113659	0.465039	1	GBP2	_
ENSG00000221539	0.767521	0.113659	0.465039	1	SNORD99	_
ENSG00000265399	0.767521	0.113659	0.465039	1	RP13-270P17.2	_
ENSG00000272990	0.767521	0.113659	0.465039	1	RP11-305K5.1	_
ENSG00000166479	-0.486027	5.494763	0.46543	1	TMX3	K09585
ENSG00000087253	-0.50054	4.029607	0.465504	1	LPCAT2	K13510
ENSG00000113739	-0.4841	5.328287	0.465789	1	STC2	_
ENSG00000198873	0.485636	5.214886	0.465822	1	GRK5	K08291
ENSG00000172893	-0.489852	5.138828	0.466249	1	DHCR7	K00213
ENSG00000101412	0.480744	6.32638	0.466314	1	E2F1	K17454
ENSG00000162572	-0.617927	1.68297	0.466499	1	SCNN1D	K04826
ENSG00000073711	-0.520383	3.162866	0.466581	1	PPP2R3A	K11583
ENSG00000108375	-0.520383	3.162866	0.466581	1	RNF43	K15694
ENSG00000225830	-0.491226	5.006217	0.46699	1	ERCC6	K10841
ENSG00000124067	0.483493	5.436072	0.467055	1	SLC12A4	K14427
ENSG00000135272	-0.483865	5.32283	0.467287	1	MDFIC	_
ENSG00000141298	0.485122	5.036236	0.467378	1	SSH2	K05766
ENSG00000132423	-0.494868	4.435729	0.467435	1	COQ3	K00591
ENSG00000101311	-0.497761	4.079639	0.467573	1	FERMT1	K17082
ENSG00000210082	0.475482	14.00351	0.467738	1	MT-RNR2	_
ENSG00000186026	-0.694339	1.119961	0.467814	1	ZNF284	K09228
ENSG00000259976	-0.694339	1.119961	0.467814	1	RP11-553L6.5	_
ENSG00000167065	0.656952	1.071081	0.467814	1	DUSP18	K14165
ENSG00000225760	-0.693097	0.74683	0.467852	1	LINC00431	_
ENSG00000230002	-0.693097	0.74683	0.467852	1	ALMS1-IT1	_
ENSG00000242338	-0.693097	0.74683	0.467852	1	BMS1P4	K14569
ENSG00000254860	-0.693097	0.74683	0.467852	1	TMEM9B-AS1	_
ENSG00000258704	-0.693097	0.74683	0.467852	1	RP11-85K15.2	_
ENSG00000125618	0.692974	0.669195	0.467852	1	PAX8	K09293
ENSG00000186765	0.692974	0.669195	0.467852	1	FSCN2	K17455
ENSG00000226823	0.692974	0.669195	0.467852	1	SUGT1P	K12795
ENSG00000254389	0.692974	0.669195	0.467852	1	RHPN1-AS1	_
ENSG00000214357	0.497988	4.024499	0.467852	1	NEURL1B	K01931
ENSG00000259479	-0.507475	3.293531	0.467932	1	CTD-2008A1.2	K00008
ENSG00000065989	0.483211	5.127071	0.467959	1	PDE4A	K13293
ENSG00000125877	-0.482377	5.409813	0.468172	1	ITPA	K01519
ENSG00000172732	-0.485251	5.355266	0.468537	1	MUS81	K08991
ENSG00000173120	0.481302	5.810412	0.468615	1	KDM2A	K10276
ENSG00000112319	0.481559	5.697223	0.468675	1	EYA4	K17622
ENSG00000128791	-0.4855	5.318451	0.468796	1	TWSG1	K17985
ENSG00000027001	-0.4918	4.352441	0.468936	1	MIPEP	K01410
ENSG00000139921	-0.480339	5.932549	0.46911	1	TMX1	_
ENSG00000234160	-0.561804	2.175878	0.469114	1	RP11-613M10.6	_
ENSG00000188153	0.477535	6.509741	0.469494	1	COL4A5	K06237
ENSG00000099364	-0.483306	5.22325	0.469506	1	FBXL19	K10285
ENSG00000261088	-0.508659	3.33757	0.469589	1	RP11-61A14.3	_
ENSG00000163697	-0.482575	4.854093	0.469974	1	APBB2	K04530
ENSG00000253293	-0.50663	3.516655	0.470055	1	HOXA10	K17443
ENSG00000056277	-0.563672	2.021839	0.47017	1	ZNF280C	_
ENSG00000169684	-0.500965	4.042954	0.470182	1	CHRNA5	K04807

ENSG00000152104	0.477447	6.028755	0.470211	1	PTPN14	K18025
ENSG00000226360	0.521582	2.925549	0.470264	1	RPL10AP6	K02865
ENSG00000178425	-0.484043	5.194856	0.470265	1	NT5DC1	K01794
ENSG00000144635	-0.481814	5.097227	0.470499	1	DYNC1LI1	K10416
ENSG00000178878	0.501363	3.432172	0.470579	1	APOLD1	K12581
ENSG00000180376	-0.503744	3.819303	0.470709	1	CCDC66	_
ENSG00000000003	-0.49737	3.619896	0.470904	1	TSPAN6	K17295
ENSG00000100106	-0.490473	4.2201	0.470964	1	TRIOBP	_
ENSG00000178752	0.504311	2.830599	0.471039	1	FAM132B	_
ENSG00000101190	0.480636	5.024143	0.471066	1	TCFL5	K09092
ENSG00000006634	-0.480034	5.730613	0.471074	1	DBF4	K06629
ENSG00000196151	-0.509773	3.3803	0.471154	1	WDSUB1	_
ENSG00000251562	0.472026	10.35697	0.47118	1	MALAT1	_
ENSG00000100983	-0.475537	6.569694	0.471242	1	GSS	K01920
ENSG00000115977	0.552344	2.393629	0.471336	1	AAK1	_
ENSG00000178093	0.531928	2.402679	0.471336	1	TSSK6	K08811
ENSG00000185158	0.531928	2.402679	0.471336	1	LRRC37B	_
ENSG00000205476	0.480015	5.293196	0.471452	1	CCDC85C	_
ENSG00000250317	-0.500245	3.706365	0.471648	1	SMIM20	_
ENSG00000225973	-0.499433	3.846905	0.47182	1	RP11-139H15.1	_
ENSG00000234231	-0.522713	2.745584	0.471923	1	AC093616.4	K03348
ENSG00000260896	0.516515	2.714818	0.471923	1	RP11-314O13.1	_
ENSG00000214717	0.472979	7.284672	0.472015	1	ZBED1	K11170
ENSG00000108175	0.474513	6.120676	0.472052	1	ZMIZ1	_
ENSG00000100292	0.490137	4.082775	0.472077	1	HMOX1	K00510
ENSG00000108176	0.487848	4.292374	0.47226	1	DNAJC12	K09532
ENSG00000166446	0.474593	6.395863	0.472274	1	CDYL2	K00653
ENSG00000151835	-0.475983	5.865834	0.472328	1	SACS	K17592
ENSG00000164332	-0.475472	6.091171	0.472361	1	UBLCP1	K17618
ENSG00000124635	0.529561	2.25476	0.472508	1	HIST1H2BJ	K11252
ENSG00000132680	0.473849	6.531272	0.472535	1	KIAA0907	K17985
ENSG00000124459	-0.503773	3.41758	0.472635	1	ZNF45	K09228
ENSG00000203666	-0.502397	3.587543	0.472743	1	EFCAB2	_
ENSG00000091140	-0.472663	7.07421	0.472869	1	DLD	K00382
ENSG00000079819	0.474182	6.424799	0.47292	1	EPB41L2	K06107
ENSG00000233221	-0.596338	1.596792	0.472999	1	AC133785.1	_
ENSG00000259305	-0.596338	1.596792	0.472999	1	ZHX1-C8ORF76	_
ENSG00000077420	-0.570422	1.580766	0.472999	1	APBB1IP	K17704
ENSG00000102780	-0.483176	4.809527	0.47314	1	DGKH	K00901
ENSG00000179399	0.483747	3.944178	0.473409	1	GPC5	K08111
ENSG00000116044	0.471465	7.218034	0.473523	1	NFE2L2	K05638
ENSG00000120798	-0.47676	5.802947	0.473659	1	NR2C1	K08543
ENSG00000176058	-0.485637	4.607114	0.473732	1	TPRN	_
ENSG00000132510	0.479977	4.42924	0.473772	1	KDM6B	_
ENSG00000144559	-0.562381	2.126352	0.473914	1	TAMM41	K17807
ENSG00000215883	0.552069	2.078668	0.473914	1	CYB5RL	K00326
ENSG00000185684	-0.544716	2.115525	0.473914	1	EP400NL	K11320
ENSG00000267248	-0.544716	2.115525	0.473914	1	CTD-2319I12.4	_
ENSG00000164171	-0.47813	5.392204	0.473945	1	ITGA2	K06481
ENSG00000162976	-0.511812	3.462129	0.474037	1	PQLC3	_
ENSG00000223705	-0.514667	3.275679	0.474084	1	NSUN5P1	K15264
ENSG00000170647	-0.523758	2.809608	0.474154	1	TMEM133	_
ENSG00000162878	0.471682	6.384326	0.474288	1	PKDCC	K17548
ENSG00000070495	0.472361	6.279599	0.474458	1	JMJD6	K11323
ENSG00000136522	-0.472031	6.368847	0.474486	1	MRPL47	K17428
ENSG00000128654	-0.47778	5.456405	0.474619	1	MTX2	K17776
ENSG00000100105	-0.480021	4.959126	0.474889	1	PATZ1	_
ENSG00000204604	-0.496608	3.196238	0.474926	1	ZNF468	K09228

ENSG00000213853	0.469299	7.642549	0.475031	1	EMP2	K19289
ENSG00000120306	0.471379	5.942043	0.475127	1	CYSTM1	_
ENSG00000204936	0.535267	2.343421	0.475519	1	CD177	K06552
ENSG00000171603	0.470326	6.641022	0.475595	1	CLSTN1	_
ENSG00000115364	-0.47177	6.414195	0.475625	1	MRPL19	K02884
ENSG00000008441	0.551873	1.891287	0.475626	1	NFIX	K09171
ENSG00000120370	-0.479044	4.776355	0.475652	1	GORAB	K19748
ENSG00000099308	0.492209	3.738977	0.475653	1	MAST3	K08789
ENSG00000170364	-0.510696	3.107777	0.475864	1	SETMAR	K11433
ENSG00000081760	0.472366	5.792274	0.475876	1	AACS	K01907
ENSG00000103494	-0.496751	3.601961	0.475879	1	RPGRIP1L	K16550
ENSG00000168763	0.477605	5.016024	0.476004	1	CNNM3	K16302
ENSG00000234741	-0.467784	8.292377	0.476007	1	GAS5	_
ENSG00000239900	-0.470094	6.69284	0.476032	1	ADSL	K01756
ENSG00000175602	-0.469792	6.814469	0.476051	1	CCDC85B	K16758
ENSG00000143545	0.469083	6.924742	0.476216	1	RAB13	K06109
ENSG00000257742	-0.506214	3.248099	0.47649	1	RP11-350F4.2	_
ENSG00000247271	-0.53242	2.579545	0.476642	1	ZBED5-AS1	_
ENSG00000005007	0.468805	6.735197	0.476829	1	UPF1	K14326
ENSG00000124151	0.471586	6.056743	0.476841	1	NCOA3	K11256
ENSG00000225259	0.494049	3.348592	0.477012	1	ST13P6	K09560
ENSG00000237517	0.556952	2.178388	0.477186	1	DGCR5	_
ENSG00000198909	0.473838	4.815277	0.477208	1	MAP3K3	K04421
ENSG00000114841	0.681271	0.917837	0.477216	1	DNAH1	K10408
ENSG00000101898	-0.66765	0.989655	0.477216	1	RP3-324O17.4	K07575
ENSG00000257086	-0.66765	0.989655	0.477216	1	RP11-783K16.13	_
ENSG00000073737	-0.627199	0.96444	0.477216	1	DHRS9	K11149
ENSG00000225465	-0.627199	0.96444	0.477216	1	RFPL1S	_
ENSG00000260430	-0.627199	0.96444	0.477216	1	RP11-626G11.4	_
ENSG00000135049	0.469845	5.486939	0.477326	1	AGTPBP1	_
ENSG00000133315	-0.473908	5.268223	0.47739	1	MACROD1	_
ENSG00000198585	-0.512122	2.771234	0.47747	1	NUDT16	K16855
ENSG00000182208	0.487581	4.187775	0.47758	1	MOB2	_
ENSG00000205659	-0.493064	3.812952	0.477733	1	LIN52	K02927
ENSG00000143674	-0.476507	5.075315	0.477735	1	MLK4	K17534
ENSG00000237886	-0.591412	1.735727	0.477755	1	RP11-611D20.2	_
ENSG00000256628	-0.591412	1.735727	0.477755	1	ZBTB11-AS1	_
ENSG00000143499	-0.473297	5.054387	0.478035	1	SMYD2	K11426
ENSG00000067182	0.465774	7.865268	0.478126	1	TNFRSF1A	K03158
ENSG00000171448	0.481056	4.370968	0.478201	1	ZBTB26	K10505
ENSG00000197183	0.490027	3.961129	0.478272	1	C20orf112	_
ENSG00000120662	-0.485708	3.762116	0.478454	1	MTRF1	K02835
ENSG00000116260	0.466537	6.925817	0.478606	1	QSOX1	K10758
ENSG00000170035	-0.471508	5.55558	0.478633	1	UBE2E3	K06689
ENSG00000121753	0.477373	4.706706	0.47868	1	BAI2	K04597
ENSG00000185736	0.480413	4.531572	0.47869	1	ADARB2	K13194
ENSG00000001617	0.468317	6.412966	0.478881	1	SEMA3F	K06840
ENSG00000165275	-0.520948	2.64385	0.478927	1	TRMT10B	K12581
ENSG00000153395	0.464243	8.376095	0.47918	1	LPCAT1	K13510
ENSG00000117407	-0.94699	-0.087628	0.479207	1	ARTN	_
ENSG00000135205	-0.94699	-0.087628	0.479207	1	CCDC146	_
ENSG00000135838	-0.94699	-0.087628	0.479207	1	NPL	K01639
ENSG00000143178	-0.94699	-0.087628	0.479207	1	TBX19	K10184
ENSG00000159588	-0.94699	-0.087628	0.479207	1	CCDC17	_
ENSG00000165985	-0.94699	-0.087628	0.479207	1	C1QL3	_
ENSG00000166947	-0.94699	-0.087628	0.479207	1	EPB42	_
ENSG00000180815	-0.94699	-0.087628	0.479207	1	MAP3K15	K13986
ENSG00000213089	-0.94699	-0.087628	0.479207	1	PDCL3P5	_

ENSG00000236393	-0.94699	-0.087628	0.479207	1	RP11-320G24.1	_
ENSG00000251026	-0.94699	-0.087628	0.479207	1	RP11-138J23.1	_
ENSG00000262712	-0.94699	-0.087628	0.479207	1	RP11-295D4.1	_
ENSG00000272909	-0.94699	-0.087628	0.479207	1	CTD-2555O16.4	_
ENSG00000141576	0.856925	-0.197275	0.479207	1	RNF157	_
ENSG00000178996	0.856925	-0.197275	0.479207	1	SNX18	K17923
ENSG00000181392	0.856925	-0.197275	0.479207	1	SYNE4	_
ENSG00000182950	0.856925	-0.197275	0.479207	1	ODF3L1	_
ENSG00000188659	0.856925	-0.197275	0.479207	1	FAM154B	K07560
ENSG00000229873	0.856925	-0.197275	0.479207	1	OGFR-AS1	_
ENSG00000237437	0.856925	-0.197275	0.479207	1	ASS1P12	K01940
ENSG00000252817	0.856925	-0.197275	0.479207	1	AL590431.1	_
ENSG00000254109	0.856925	-0.197275	0.479207	1	RBPMS-AS1	_
ENSG00000258748	0.856925	-0.197275	0.479207	1	CTD-2223O18.1	_
ENSG00000261731	0.856925	-0.197275	0.479207	1	CTD-2358C21.4	_
ENSG00000261777	0.856925	-0.197275	0.479207	1	RP11-529K1.2	_
ENSG00000029993	-0.854881	-0.144255	0.479207	1	HMGB3	K11296
ENSG00000108405	-0.854881	-0.144255	0.479207	1	P2RX1	K05215
ENSG00000135373	-0.854881	-0.144255	0.479207	1	EHF	K17102
ENSG00000166396	-0.854881	-0.144255	0.479207	1	SERPINB7	K13964
ENSG00000167588	-0.854881	-0.144255	0.479207	1	GPD1	K00006
ENSG00000171189	-0.854881	-0.144255	0.479207	1	GRIK1	K05201
ENSG00000178385	-0.854881	-0.144255	0.479207	1	PLEKHM3	_
ENSG00000230896	-0.854881	-0.144255	0.479207	1	RP11-767N6.7	_
ENSG00000254827	-0.854881	-0.144255	0.479207	1	SLC22A18AS	_
ENSG00000258900	-0.854881	-0.144255	0.479207	1	HNRNPCP1	K12884
ENSG00000265750	-0.854881	-0.144255	0.479207	1	RP11-799B12.4	_
ENSG00000049883	-0.482533	4.304437	0.479224	1	PTCD2	K17711
ENSG00000012660	0.464706	7.603285	0.479393	1	ELOVL5	K10244
ENSG00000155868	-0.487047	3.794057	0.479452	1	MED7	K15148
ENSG00000138758	-0.513613	2.834159	0.479456	1	11-Sep	K16939
ENSG00000168917	0.512647	2.805498	0.479456	1	SLC35G2	K17985
ENSG00000127946	0.733283	0.463827	0.479616	1	HIP1	K04559
ENSG00000183091	0.733283	0.463827	0.479616	1	NEB	K18267
ENSG00000257702	0.733283	0.463827	0.479616	1	LBX2-AS1	_
ENSG00000270039	0.733283	0.463827	0.479616	1	RP11-571M6.17	_
ENSG00000273478	0.733283	0.463827	0.479616	1	RP11-465N4.5	_
ENSG00000196588	0.471544	5.441142	0.479753	1	MKL1	_
ENSG00000054598	-0.466438	6.022164	0.479788	1	FOXC1	K09396
ENSG00000172071	0.473789	4.945053	0.480123	1	EIF2AK3	K08860
ENSG00000221963	-0.467287	5.910913	0.480175	1	APOL6	K14480
ENSG00000163378	-0.502668	3.127804	0.480182	1	EOGT	K18134
ENSG00000237248	0.482543	4.156137	0.480244	1	LINC00987	_
ENSG00000189403	-0.463041	8.527115	0.480313	1	HMGB1	K10802
ENSG00000240682	-0.473651	4.546525	0.480318	1	ISY1	K12870
ENSG00000133059	0.470832	4.587522	0.480404	1	DSTYK	K16288
ENSG00000056972	0.468508	5.124581	0.480439	1	TRAF3IP2	_
ENSG00000198353	0.551246	1.423919	0.480463	1	HOXC4	K09304
ENSG00000169084	0.467075	5.861933	0.480525	1	DHRSX	K11170
ENSG00000176476	-0.495212	3.371597	0.480655	1	CCDC101	K11364
ENSG00000171497	-0.46688	5.812335	0.480662	1	PPID	K05864
ENSG00000111845	-0.464936	6.184405	0.480805	1	PAK1IP1	K14830
ENSG00000267519	0.469629	5.399061	0.480853	1	MIR24-2	_
ENSG00000168569	-0.481327	4.191554	0.480975	1	TMEM223	_
ENSG00000063046	0.461748	9.686585	0.480989	1	EIF4B	K03258
ENSG00000166557	0.463433	7.232683	0.481053	1	TMED3	K14825
ENSG00000242125	-0.464208	6.916551	0.481094	1	SNHG3	_
ENSG00000145743	-0.487976	3.682512	0.481116	1	FBXL17	K10283

ENSG00000170903	-0.487976	3.682512	0.481116	1	MSANTD4	_
ENSG00000093010	-0.464413	6.71594	0.481318	1	COMT	K00545
ENSG00000085840	0.466423	5.484978	0.481809	1	ORC1	K02603
ENSG00000156469	-0.467662	5.85	0.481815	1	MTERFD1	K15032
ENSG00000109534	-0.469602	5.361657	0.482022	1	GAR1	K11128
ENSG00000121486	-0.472678	4.90608	0.482139	1	TRMT1L	_
ENSG00000129757	0.463425	6.594001	0.482185	1	CDKN1C	K09993
ENSG00000004864	-0.467193	5.864345	0.482259	1	SLC25A13	K15105
ENSG00000172115	-0.460658	8.54961	0.482553	1	CYCS	K08738
ENSG00000116786	0.463236	6.034773	0.482901	1	PLEKHM2	K15348
ENSG00000151743	0.51452	2.760869	0.482914	1	AMN1	_
ENSG00000128311	-0.466246	5.364845	0.482944	1	TST	K01011
ENSG00000101473	0.473072	4.580182	0.483008	1	ACOT8	K11992
ENSG00000148358	0.470868	4.390025	0.483622	1	GPR107	_
ENSG00000141543	0.459616	8.208788	0.48367	1	EIF4A3	K13025
ENSG00000103047	-0.474084	4.885501	0.483837	1	TANGO6	_
ENSG00000130508	0.464503	5.724894	0.483875	1	PXDN	K19511
ENSG00000157184	-0.470616	4.715978	0.483957	1	CPT2	K08766
ENSG00000260005	-0.615091	1.175447	0.483994	1	AC027601.1	_
ENSG00000247844	0.460079	7.296962	0.484137	1	CCAT1	_
ENSG00000158792	-0.488736	3.388317	0.484252	1	SPATA2L	K17595
ENSG00000037241	-0.465046	5.63082	0.48446	1	RPL26L1	K02898
ENSG00000140577	0.460151	6.837472	0.484793	1	CRTC3	K16334
ENSG00000198081	-0.487694	3.809766	0.484852	1	ZBTB14	_
ENSG00000233024	0.558829	1.577133	0.484925	1	NPIPA7	K04985
ENSG00000229638	0.473567	4.696591	0.484935	1	RPL4P4	K02930
ENSG00000111981	-0.512492	2.240632	0.48504	1	ULBP1	K07986
ENSG00000156239	-0.512492	2.240632	0.48504	1	N6AMT1	K02493
ENSG00000161082	-0.564398	1.966572	0.485092	1	CELF5	K13207
ENSG00000256433	-0.564398	1.966572	0.485092	1	RP1-102E24.8	_
ENSG00000120669	-0.467573	5.323911	0.48516	1	SOHLH2	_
ENSG00000179111	-0.466435	5.088219	0.485196	1	HES7	K09087
ENSG00000185947	0.477655	3.906668	0.485547	1	ZNF267	K09228
ENSG00000181381	-0.463251	5.518826	0.485697	1	DDX60L	K12581
ENSG00000181472	-0.469371	5.180424	0.485751	1	ZBTB2	K10489
ENSG00000012822	0.463742	5.174115	0.485751	1	CALCOCO1	_
ENSG00000247828	-0.4883	3.191276	0.485767	1	TMEM161B-AS1	_
ENSG00000101255	-0.459745	6.560503	0.486093	1	TRIB3	K19518
ENSG00000196756	-0.464407	5.39428	0.486255	1	SNHG17	_
ENSG00000164535	0.461242	5.791468	0.486342	1	DAGLB	K13806
ENSG00000128487	0.457157	7.779409	0.486361	1	SPECC1	_
ENSG00000043355	-0.463125	5.104842	0.486643	1	ZIC2	K06235
ENSG00000176273	-0.476124	3.787589	0.4867	1	SLC35G1	_
ENSG00000112414	-0.458727	6.409094	0.48677	1	GPR126	K08463
ENSG00000119906	-0.470197	4.050595	0.486849	1	FAM178A	_
ENSG00000175471	-0.472204	4.518141	0.486876	1	MCTP1	_
ENSG00000268516	0.507436	2.522654	0.486983	1	CTD-3138B18.5	_
ENSG00000196968	0.468328	4.3984	0.48718	1	FUT11	K11257
ENSG00000158373	0.483178	3.382862	0.487804	1	HIST1H2BD	K11252
ENSG00000071994	-0.457249	6.809064	0.487896	1	PDCD2	K14801
ENSG00000167280	-0.467915	4.815701	0.487928	1	ENGASE	K01227
ENSG00000164362	-0.544663	2.063805	0.48798	1	TERT	K11126
ENSG00000168661	-0.544663	2.063805	0.48798	1	ZNF30	K09228
ENSG00000134333	0.454082	10.78123	0.488078	1	LDHA	K00016
ENSG00000262477	-0.474433	3.640784	0.488093	1	AC021224.1	_
ENSG00000074356	-0.468651	4.730882	0.488238	1	C17orf85	_
ENSG00000162688	-0.458485	6.001151	0.48826	1	AGL	K01196
ENSG00000082212	-0.458479	5.944004	0.488476	1	ME2	K00027

ENSG00000100271	0.564977	1.715848	0.48859	1	TTLL1	K16599
ENSG00000090971	-0.544324	1.772737	0.48859	1	NAT14	_
ENSG00000151611	-0.544324	1.772737	0.48859	1	MMAA	K07588
ENSG00000182890	0.531938	1.730785	0.48859	1	GLUD2	K00261
ENSG00000156876	-0.458144	6.014076	0.488603	1	SASS6	K16487
ENSG00000162913	0.559998	1.305329	0.489124	1	C1orf145	K17531
ENSG00000166780	0.559998	1.305329	0.489124	1	C16orf45	_
ENSG00000144485	-0.489954	3.01812	0.489153	1	HES6	K09087
ENSG00000133466	0.455547	6.510633	0.489429	1	C1QTNF6	K19470
ENSG00000111875	-0.462352	5.054363	0.489449	1	ASF1A	K10753
ENSG00000126870	0.466404	4.829283	0.489638	1	WDR60	_
ENSG00000182796	-0.51431	2.864616	0.489778	1	TMEM198B	K17692
ENSG00000186666	-0.503831	2.858307	0.489778	1	BCDIN3D	_
ENSG00000130164	0.455784	6.315866	0.489905	1	LDLR	K12473
ENSG00000010704	-0.472721	4.013144	0.490082	1	HFE	K11253
ENSG00000056050	-0.46054	4.906042	0.490166	1	C4orf27	_
ENSG00000113368	-0.454383	6.9126	0.490415	1	LMNB1	K07611
ENSG00000185164	0.459501	5.494757	0.490515	1	NOMO2	_
ENSG00000198276	-0.460709	5.330447	0.490515	1	UCKL1	K00876
ENSG00000141127	-0.46005	5.436327	0.490563	1	PRPSAP2	K17299
ENSG00000082269	-0.479373	3.742531	0.490582	1	FAM135A	_
ENSG00000136824	-0.454492	6.722208	0.490705	1	SMC2	K06674
ENSG00000105514	0.474216	3.640565	0.491067	1	RAB3D	K07884
ENSG00000233762	0.464163	4.621869	0.49118	1	AC007969.5	K02958
ENSG00000183666	-0.505577	2.917613	0.491182	1	GUSBP1	K01195
ENSG00000162994	0.495039	2.897103	0.491182	1	CLHC1	_
ENSG00000115561	-0.461017	4.694119	0.491314	1	CHMP3	K12193
ENSG00000164265	0.509531	2.468223	0.491483	1	SCGB3A2	_
ENSG00000177192	-0.457224	5.980508	0.491571	1	PUS1	K06173
ENSG00000168826	-0.523467	1.883527	0.491653	1	ZBTB49	_
ENSG00000225697	0.457369	5.858136	0.491663	1	SLC26A6	K14704
ENSG00000125089	0.467121	4.52403	0.491764	1	SH3TC1	_
ENSG00000175130	-0.453025	6.731304	0.492083	1	MARCKSL1	K13536
ENSG00000156475	-0.489609	2.757693	0.492084	1	PPP2R2B	K04354
ENSG00000152219	-0.458617	4.953427	0.492401	1	ARL14EP	K17985
ENSG00000162174	0.486581	2.960666	0.492481	1	ASRGL1	K13051
ENSG00000157985	0.459985	5.046968	0.492527	1	AGAP1	K12491
ENSG00000122378	-0.479503	3.34136	0.492967	1	FAM213A	K11447
ENSG00000139160	-0.599226	1.521901	0.49314	1	METTL20	_
ENSG00000229186	-0.599226	1.521901	0.49314	1	ADAM1A	K08607
ENSG00000137601	-0.479685	3.626745	0.493249	1	NEK1	K08857
ENSG00000140396	-0.457781	4.732484	0.493492	1	NCOA2	K11255
ENSG00000112343	-0.461584	4.7505	0.493847	1	TRIM38	K12014
ENSG00000164933	-0.453177	6.171741	0.493851	1	SLC25A32	K15115
ENSG00000139618	-0.481474	3.383996	0.493898	1	BRCA2	K08775
ENSG00000105185	-0.451365	6.53763	0.494123	1	PDCD5	K06875
ENSG00000135127	0.470715	4.17825	0.494132	1	CCDC64	K16756
ENSG00000107105	-0.525284	1.998341	0.494252	1	ELAVL2	K13208
ENSG00000120526	-0.450416	6.753387	0.494528	1	NUDCD1	_
ENSG00000090554	0.790017	0.224692	0.494712	1	FLT3LG	K05454
ENSG00000170153	0.790017	0.224692	0.494712	1	RNF150	K15701
ENSG00000256817	0.790017	0.224692	0.494712	1	TPT1P12	_
ENSG00000115295	-0.760443	0.281025	0.494712	1	CLIP4	K10423
ENSG00000144460	-0.760443	0.281025	0.494712	1	NYAP2	K17985
ENSG00000150672	-0.760443	0.281025	0.494712	1	DLG2	K12075
ENSG00000212232	-0.760443	0.281025	0.494712	1	SNORD17	_
ENSG00000261159	-0.760443	0.281025	0.494712	1	RP11-723O4.9	_
ENSG00000143515	0.471089	3.553545	0.494757	1	ATP8B2	K01530

ENSG00000083828	0.480658	3.246202	0.494797	1	ZNF586	K09228
ENSG00000053702	0.620307	0.944861	0.494848	1	NRIP2	K13215
ENSG00000204524	0.620307	0.944861	0.494848	1	ZNF805	K09228
ENSG00000253958	0.620307	0.944861	0.494848	1	CLDN23	K06087
ENSG00000254639	0.620307	0.944861	0.494848	1	CTD-2589M5.5	_
ENSG00000248019	0.561815	0.971308	0.494848	1	FAM13A-AS1	_
ENSG00000163374	0.452189	5.962592	0.495137	1	YY1AP1	_
ENSG00000183520	-0.451922	6.055475	0.495367	1	UTP11L	K14769
ENSG00000068784	-0.468259	4.172141	0.495677	1	SRBD1	_
ENSG00000111424	-0.466519	3.982933	0.495858	1	VDR	K08539
ENSG00000136068	0.446889	8.041818	0.495948	1	FLNB	K04437
ENSG00000141337	-0.486521	2.934452	0.496003	1	ARSG	K12381
ENSG00000154447	0.457804	4.655414	0.496029	1	SH3RF1	K12171
ENSG00000225892	-0.593748	1.667949	0.496369	1	RP11-384K6.2	_
ENSG00000073670	0.572939	1.619473	0.496369	1	ADAM11	K16067
ENSG00000273329	0.572939	1.619473	0.496369	1	RP11-448A19.1	_
ENSG00000139278	-0.569157	1.652749	0.496369	1	GLIPR1	_
ENSG00000273066	-0.569157	1.652749	0.496369	1	RP11-216L13.19	_
ENSG00000243479	-0.526831	2.104607	0.496484	1	MNX1-AS1	_
ENSG00000157869	-0.460192	4.30205	0.496506	1	RAB28	K07915
ENSG00000180694	-0.450474	5.49282	0.496516	1	TMEM64	_
ENSG00000125870	-0.44926	6.14293	0.497159	1	SNRPB2	K11094
ENSG00000123607	-0.466364	4.061089	0.497452	1	TTC21B	K19673
ENSG00000038382	0.447222	6.649282	0.497496	1	TRIO	K08810
ENSG00000106351	0.450316	5.464225	0.497612	1	AGFG2	_
ENSG00000115548	0.447064	6.089975	0.497677	1	KDM3A	K15601
ENSG00000167513	0.446478	6.881693	0.497762	1	CDT1	K10727
ENSG00000178966	-0.455739	4.944918	0.49785	1	RMI1	K10990
ENSG00000131711	1.018542	-0.59288	0.497906	1	MAP1B	K10429
ENSG00000136383	1.018542	-0.59288	0.497906	1	ALPK3	K08868
ENSG00000138642	1.018542	-0.59288	0.497906	1	HERC6	K10614
ENSG00000162931	1.018542	-0.59288	0.497906	1	TRIM17	K12007
ENSG00000177173	1.018542	-0.59288	0.497906	1	NAP1L4P1	K11282
ENSG00000179168	1.018542	-0.59288	0.497906	1	GGN	_
ENSG00000200090	1.018542	-0.59288	0.497906	1	Y_RNA	_
ENSG00000214784	1.018542	-0.59288	0.497906	1	AC010468.1	K02984
ENSG00000225313	1.018542	-0.59288	0.497906	1	RP11-415J8.3	_
ENSG00000225745	1.018542	-0.59288	0.497906	1	PLAC4	_
ENSG00000225778	1.018542	-0.59288	0.497906	1	PROSER2-AS1	_
ENSG00000227953	1.018542	-0.59288	0.497906	1	RP11-439E19.3	_
ENSG00000233785	1.018542	-0.59288	0.497906	1	RP13-314C10.5	_
ENSG00000235001	1.018542	-0.59288	0.497906	1	EIF4A1P2	K03257
ENSG00000239127	1.018542	-0.59288	0.497906	1	SNORD125	_
ENSG00000242198	1.018542	-0.59288	0.497906	1	CTD-2235C13.1	K02976
ENSG00000245275	1.018542	-0.59288	0.497906	1	SAP30L-AS1	_
ENSG00000248787	1.018542	-0.59288	0.497906	1	RP11-666A20.4	_
ENSG00000251034	1.018542	-0.59288	0.497906	1	RP11-582J16.4	_
ENSG00000260190	1.018542	-0.59288	0.497906	1	RP11-229P13.25	_
ENSG00000263089	1.018542	-0.59288	0.497906	1	RP11-166P13.4	_
ENSG00000267082	1.018542	-0.59288	0.497906	1	CTC-510F12.2	_
ENSG00000267681	1.018542	-0.59288	0.497906	1	CTD-3199J23.6	K11299
ENSG00000268852	1.018542	-0.59288	0.497906	1	AC132872.2	K18047
ENSG00000270049	1.018542	-0.59288	0.497906	1	RP11-297D21.4	_
ENSG00000272430	1.018542	-0.59288	0.497906	1	RP11-38L15.8	_
ENSG00000210196	0.446621	6.730155	0.497909	1	MT-TP	_
ENSG00000141905	0.446704	6.647966	0.497996	1	NFIC	K09170
ENSG00000166012	-0.444911	7.963071	0.498047	1	TAF1D	_
ENSG00000134769	-0.491012	3.044959	0.498147	1	DTNA	_

ENSG00000178075	-0.491012	3.044959	0.498147	1	GRAMD1C	_
ENSG00000140367	-0.473708	3.73918	0.498205	1	UBE2Q2	K10582
ENSG00000080603	0.450498	5.538905	0.498251	1	SRCAP	K11661
ENSG00000184575	-0.444541	8.080741	0.498298	1	XPOT	K14288
ENSG00000013288	0.451771	4.711859	0.498451	1	MAN2B2	K12312
ENSG00000158006	-0.476309	3.274933	0.498608	1	PAFAH2	K01062
ENSG00000153179	-0.450798	5.109898	0.498823	1	RASSF3	K09852
ENSG00000168002	-0.449921	5.82862	0.498857	1	POLR2G	K03015
ENSG00000125257	0.445961	6.506258	0.498888	1	ABCC4	K05673
ENSG00000266967	-0.484089	3.091846	0.499108	1	AARSD1	K07050
ENSG00000243660	0.479535	2.561197	0.499285	1	ZNF487	K05610
ENSG00000197536	-0.577259	1.24982	0.499294	1	C5orf56	_
ENSG00000260766	-0.577259	1.24982	0.499294	1	RP11-226L15.5	_
ENSG00000264058	-0.577259	1.24982	0.499294	1	KRT222	K11651
ENSG00000223820	0.570479	1.176213	0.499294	1	CFL1P1	_
ENSG00000224877	-0.447123	6.156497	0.499375	1	C17orf89	_
ENSG00000169991	0.474472	3.296326	0.499418	1	IFFO2	_
ENSG00000079257	0.447279	5.7779	0.499867	1	LXN	_
ENSG00000143772	-0.529324	2.296025	0.500122	1	ITPKB	K00911
ENSG00000241935	-0.513586	2.286465	0.500122	1	HOGA1	K18123
ENSG00000130600	0.443094	7.336032	0.500329	1	H19	_
ENSG00000143252	-0.44512	6.445244	0.500502	1	SDHC	K00236
ENSG00000248175	0.486646	2.633358	0.500545	1	CTC-428G20.3	_
ENSG00000189362	-0.485396	2.656777	0.500545	1	TMEM194B	_
ENSG00000100344	-0.477874	2.95701	0.500741	1	PNPLA3	K13534
ENSG00000175482	-0.477874	2.95701	0.500741	1	POLD4	K03505
ENSG00000198088	-0.477874	2.95701	0.500741	1	NUP62CL	K14306
ENSG00000198919	-0.463268	3.748848	0.500954	1	DZIP3	K10642
ENSG00000163900	-0.452049	5.335869	0.500959	1	TMEM41A	K12581
ENSG00000158467	0.453696	4.447649	0.50098	1	AHCYL2	K01251
ENSG00000105974	-0.441801	7.894113	0.501113	1	CAV1	K06278
ENSG00000168014	0.451661	4.639667	0.501272	1	C2CD3	K16751
ENSG00000186260	0.464961	3.762187	0.501492	1	MKL2	_
ENSG00000168614	-0.515572	2.373947	0.501624	1	NBPF9	_
ENSG00000162585	-0.448515	4.834164	0.501752	1	C1orf86	K18952
ENSG00000196526	0.442253	6.896507	0.501807	1	AFAP1	K18616
ENSG00000120800	-0.446805	5.389059	0.501843	1	UTP20	K14772
ENSG00000132600	0.442567	6.705508	0.501863	1	PRMT7	K11438
ENSG00000167202	0.456108	4.41093	0.502014	1	TBC1D2B	_
ENSG00000132963	-0.442242	6.859366	0.502164	1	POMP	K11599
ENSG00000065526	0.444032	5.561412	0.502363	1	SPEN	_
ENSG00000181444	0.464138	3.26948	0.502368	1	ZNF467	K09228
ENSG00000139826	-0.448856	4.947737	0.502412	1	ABHD13	K06889
ENSG00000160007	0.44361	6.410827	0.502413	1	ARHGAP35	K05732
ENSG00000100916	-0.473788	3.060419	0.502538	1	BRMS1L	K19196
ENSG00000231419	0.472175	3.043147	0.502538	1	LINC00689	_
ENSG00000178209	0.44639	5.786682	0.502556	1	PLEC	K10388
ENSG00000125731	-0.451801	4.806285	0.502561	1	SH2D3A	_
ENSG00000002746	-0.543775	1.40665	0.502646	1	HECW1	K12167
ENSG00000229953	-0.543775	1.40665	0.502646	1	RP11-284F21.7	_
ENSG00000123213	-0.444219	6.054174	0.502727	1	NLN	K01393
ENSG00000145414	-0.458608	4.004838	0.502809	1	NAF1	K14763
ENSG00000151665	-0.458608	4.004838	0.502809	1	PIGF	K05287
ENSG00000137135	-0.453831	4.479304	0.502966	1	ARHGEF39	K09228
ENSG00000197959	-0.710288	0.607863	0.503	1	DNM3	K01528
ENSG00000246465	-0.710288	0.607863	0.503	1	RP11-57A19.2	_
ENSG00000169955	-0.464409	3.332404	0.503063	1	ZNF747	K09228
ENSG00000131746	0.441812	6.260727	0.503064	1	TNS4	K18080

ENSG00000139726	-0.441761	6.617836	0.503154	1	DENR	K17985
ENSG00000090989	-0.451892	4.921987	0.503171	1	EXOC1	_
ENSG00000170584	-0.4462	5.792757	0.503225	1	NUDCD2	_
ENSG00000166979	0.443301	5.869435	0.503228	1	EVA1C	_
ENSG00000181830	0.440905	6.790926	0.503302	1	SLC35C1	K15279
ENSG00000214510	0.477578	3.095285	0.503343	1	SPINK13	_
ENSG00000154889	0.466793	3.542353	0.503419	1	MPPE1	K12581
ENSG00000124802	-0.442636	6.136116	0.503475	1	EEF1E1	K15439
ENSG00000115540	-0.449524	5.090756	0.503579	1	MOB4	_
ENSG00000186318	0.465138	3.739349	0.503676	1	BACE1	K04521
ENSG00000132879	0.449863	4.730193	0.504031	1	FBXO44	K10103
ENSG00000100284	0.446399	5.120838	0.504148	1	TOM1	_
ENSG00000033327	0.461096	3.775127	0.504154	1	GAB2	K08091
ENSG00000155254	-0.440942	6.52251	0.504177	1	MARVELD1	_
ENSG00000143157	-0.441615	6.150361	0.504185	1	POGK	_
ENSG00000095951	-0.450879	4.752081	0.504272	1	HIVEP1	K12581
ENSG00000015171	-0.444182	5.326076	0.504291	1	ZMYND11	_
ENSG00000196470	-0.462215	3.633564	0.504498	1	SIAH1	K04506
ENSG00000187097	-0.485281	2.905441	0.504583	1	ENTPD5	K01511
ENSG00000004777	0.526609	2.090086	0.504734	1	ARHGAP33	K17933
ENSG00000101361	-0.438316	7.638342	0.50476	1	NOP56	K14564
ENSG00000167721	-0.438988	7.125402	0.504825	1	TSR1	K14799
ENSG00000129055	-0.440531	6.09253	0.504863	1	ANAPC13	K12456
ENSG00000136643	-0.445175	5.177971	0.504864	1	RPS6KC1	_
ENSG00000127948	0.453706	4.516451	0.50516	1	POR	K00327
ENSG00000137710	0.4384	7.119871	0.505171	1	RDX	K05762
ENSG00000003987	0.582131	1.516241	0.505261	1	MTMR7	K18083
ENSG00000046653	0.543829	1.533565	0.505261	1	GPM6B	_
ENSG00000152492	-0.440529	6.461739	0.505577	1	CCDC50	_
ENSG00000113070	0.457467	3.719592	0.505934	1	HBEGF	K08523
ENSG00000132952	-0.451973	4.231482	0.506102	1	USPL1	_
ENSG00000174977	-0.445926	4.973037	0.506171	1	AC026271.5	K14322
ENSG00000072954	0.477786	2.66161	0.506213	1	TMEM38A	K14209
ENSG00000100479	-0.456811	4.117587	0.506515	1	POLE2	K02325
ENSG00000183255	0.43555	8.631091	0.50653	1	PTTG1IP	_
ENSG00000125863	-0.440825	6.111011	0.50665	1	MKKS	K09492
ENSG00000115355	-0.440841	5.877843	0.506897	1	CCDC88A	_
ENSG00000158286	-0.462394	3.824984	0.507191	1	RNF207	_
ENSG00000126768	-0.544236	1.706658	0.507356	1	TIMM17B	K15696
ENSG00000154957	-0.499208	2.321553	0.507365	1	ZNF18	K09229
ENSG00000182324	-0.499208	2.321553	0.507365	1	KCNJ14	K05007
ENSG00000135045	-0.444027	5.446396	0.50761	1	C9orf40	_
ENSG00000131650	-0.443886	4.910365	0.50764	1	KREMEN2	_
ENSG00000181222	0.436234	6.861009	0.5077	1	POLR2A	K03006
ENSG00000175084	0.45833	3.408346	0.507729	1	DES	K07610
ENSG00000091947	0.438799	5.579902	0.507783	1	TMEM101	_
ENSG00000086189	-0.438919	5.90147	0.507798	1	DIMT1	K14191
ENSG00000134480	-0.437838	6.084818	0.507857	1	CCNH	K06634
ENSG00000173890	-0.679167	0.873416	0.508053	1	GPR160	_
ENSG00000180881	-0.679167	0.873416	0.508053	1	CAPS2	_
ENSG00000186523	-0.679167	0.873416	0.508053	1	FAM86B1	K03842
ENSG00000261884	-0.679167	0.873416	0.508053	1	CTC-479C5.12	K09632
ENSG00000160318	-0.635001	0.845902	0.508053	1	CLDND2	_
ENSG00000175591	-0.635001	0.845902	0.508053	1	P2RY2	K04269
ENSG00000257718	-0.635001	0.845902	0.508053	1	RP11-396F22.1	_
ENSG00000105550	0.575816	0.807127	0.508053	1	FGF21	K04358
ENSG00000122367	0.575816	0.807127	0.508053	1	LDB3	K12581
ENSG00000141655	0.575816	0.807127	0.508053	1	TNFRSF11A	K05147

ENSG00000214982	0.575816	0.807127	0.508053	1	PARGP1	K07759
ENSG00000272669	0.575816	0.807127	0.508053	1	RP3-508I15.21	_
ENSG00000206262	0.493548	2.362127	0.50845	1	C3orf72	K11447
ENSG00000270949	0.493548	2.362127	0.50845	1	RP11-288H12.4	_
ENSG00000072840	-0.487379	2.398186	0.50845	1	EVC	K19605
ENSG00000136243	-0.438448	5.86213	0.508506	1	NUPL2	K14321
ENSG00000030110	-0.44154	5.524545	0.508558	1	BAK1	K14021
ENSG00000112039	0.443128	5.077257	0.508645	1	FANCE	K10892
ENSG00000170390	0.464615	3.204308	0.508696	1	DCLK2	K08805
ENSG00000197275	-0.444949	4.445489	0.508811	1	RAD54B	K10877
ENSG00000197258	0.439106	5.503502	0.509138	1	EIF4BP6	K03258
ENSG00000197933	0.480987	2.903351	0.509345	1	ZNF823	K09228
ENSG00000119673	-0.442513	4.957614	0.509348	1	ACOT2	K01068
ENSG00000160752	-0.432915	8.314185	0.509385	1	FDPS	K00787
ENSG00000187123	-0.456047	3.806241	0.5094	1	LYPD6	_
ENSG00000118496	-0.445681	4.502619	0.509485	1	FBXO30	K10307
ENSG00000175548	-0.458976	3.579571	0.509547	1	ALG10B	K03850
ENSG00000130147	0.439578	5.550201	0.50967	1	SH3BP4	_
ENSG00000186866	0.441786	5.266084	0.509828	1	POFUT2	K03691
ENSG00000160336	-0.461163	3.615791	0.509947	1	ZNF761	_
ENSG00000132819	-0.445782	4.677039	0.509971	1	RBM38	_
ENSG00000185730	-0.459831	3.867582	0.510076	1	ZNF696	_
ENSG00000122870	-0.449574	4.559446	0.510109	1	BICC1	K18756
ENSG00000111696	-0.457172	3.64754	0.510327	1	NT5DC3	_
ENSG00000151304	-0.451601	4.102322	0.510436	1	SRFBP1	K12581
ENSG00000107371	-0.436321	5.817039	0.51047	1	EXOSC3	K03681
ENSG00000168899	0.522825	1.904401	0.510498	1	VAMP5	K08514
ENSG00000135315	-0.468002	3.396295	0.510657	1	KIAA1009	K16809
ENSG00000124356	-0.438751	5.611269	0.511015	1	STAMBP	K11866
ENSG00000145781	-0.463178	3.43319	0.51108	1	COMMD10	_
ENSG00000100522	-0.432498	6.494657	0.511118	1	GNPNAT1	K00621
ENSG00000176225	-0.441096	4.527484	0.511283	1	RTTN	K16484
ENSG00000267060	-0.657968	1.097115	0.511408	1	PTGES3L	K07050
ENSG00000137941	0.583261	1.034525	0.511408	1	TTLL7	K16583
ENSG00000176884	0.583261	1.034525	0.511408	1	GRIN1	K05208
ENSG00000109099	0.431068	7.609656	0.511684	1	PMP22	K19289
ENSG00000167799	-0.498726	2.698336	0.511765	1	NUDT8	K18665
ENSG00000126088	-0.43209	6.941107	0.511856	1	UROD	K01599
ENSG00000150756	-0.444452	4.060872	0.512062	1	FAM173B	_
ENSG00000152782	-0.447101	4.272801	0.512112	1	PANK1	K09680
ENSG00000174282	0.432475	6.16902	0.512338	1	ZBTB4	K10491
ENSG00000141738	0.442248	4.487436	0.512511	1	GRB7	_
ENSG00000168228	-0.447437	3.590347	0.51256	1	ZCCHC4	K17985
ENSG00000154822	-0.449748	4.137563	0.512768	1	PLCL2	K15370
ENSG00000124523	-0.445425	4.135033	0.512768	1	SIRT5	K11415
ENSG00000223496	-0.447263	3.903	0.512822	1	EXOSC6	K12587
ENSG00000164654	-0.439005	4.737337	0.51325	1	MIOS	K09228
ENSG00000100889	-0.431886	6.381092	0.513312	1	PCK2	K01596
ENSG00000170537	0.453436	3.30565	0.513345	1	TMC7	_
ENSG00000176422	-0.450417	4.207969	0.513406	1	SPRYD4	K12581
ENSG00000115204	0.434788	4.89584	0.513426	1	MPV17	K13348
ENSG00000105497	0.48179	2.861707	0.51353	1	ZNF175	K09228
ENSG00000182459	0.467459	2.868117	0.51353	1	TEX19	_
ENSG00000258297	0.458516	3.349271	0.513713	1	RP11-658F2.8	_
ENSG00000180747	0.54166	1.25285	0.513776	1	CTD-2547E10.2	K13780
ENSG00000136295	0.431418	6.439426	0.513833	1	TTYH3	_
ENSG00000134440	-0.428622	7.914462	0.513916	1	NARS	K01893
ENSG00000213741	0.429494	7.002044	0.514031	1	RPS29	K02980

ENSG00000188483	-0.432529	5.942626	0.514057	1	IER5L	_
ENSG00000162639	-0.436054	5.240661	0.514166	1	HENMT1	K11847
ENSG00000120533	-0.42956	6.768398	0.514672	1	ENY2	K11368
ENSG00000128536	-0.717439	0.034409	0.514813	1	CDHR3	K16503
ENSG00000134864	-0.717439	0.034409	0.514813	1	GGACT	K19761
ENSG00000188316	-0.717439	0.034409	0.514813	1	ENO4	_
ENSG00000231611	-0.717439	0.034409	0.514813	1	AP006216.11	_
ENSG00000235734	-0.717439	0.034409	0.514813	1	HMGN1P36	K11299
ENSG00000236015	-0.717439	0.034409	0.514813	1	AC011290.5	_
ENSG00000236432	-0.717439	0.034409	0.514813	1	AC097662.2	_
ENSG00000241170	-0.717439	0.034409	0.514813	1	RP11-147I3.1	K02910
ENSG00000259319	-0.717439	0.034409	0.514813	1	RP11-293M10.6	_
ENSG00000263350	-0.717439	0.034409	0.514813	1	RP11-13N13.2	_
ENSG00000176014	0.427615	7.845521	0.514833	1	TUBB6	K07375
ENSG00000138442	-0.433709	5.731481	0.514865	1	WDR12	K14863
ENSG00000128709	0.466028	3.027242	0.514904	1	HOXD9	K09294
ENSG00000123080	0.432644	5.447207	0.514941	1	CDKN2C	K06622
ENSG00000151287	-0.439554	4.949129	0.514968	1	TEX30	_
ENSG00000067955	-0.433721	5.564867	0.515005	1	CBFB	_
ENSG00000227097	0.426889	8.5229	0.515054	1	RP11-742N3.1	K02979
ENSG00000104805	0.42719	7.939628	0.515166	1	NUCB1	_
ENSG00000205664	-0.464291	3.564921	0.515273	1	RP11-706O15.1	K19584
ENSG00000257103	0.459449	3.085404	0.515297	1	LSM14A	K18749
ENSG00000196372	-0.434223	5.511171	0.515373	1	ASB13	K10335
ENSG00000120802	-0.426801	8.240735	0.515422	1	TMPO	_
ENSG00000169241	0.428517	6.687663	0.515558	1	SLC50A1	K15382
ENSG00000078804	0.490465	2.476781	0.515719	1	TP53INP2	K15310
ENSG00000138650	0.490465	2.476781	0.515719	1	PCDH10	K16499
ENSG00000188825	0.490465	2.476781	0.515719	1	LINC00910	_
ENSG00000134709	-0.478694	2.510107	0.515719	1	HOOK1	K16612
ENSG00000180228	-0.440034	4.714179	0.515735	1	PRKRA	_
ENSG00000057019	-0.434937	5.200749	0.515762	1	DCBLD2	_
ENSG00000116641	-0.429455	6.212503	0.516069	1	DOCK7	_
ENSG00000157181	-0.435186	4.953362	0.516141	1	C1orf27	_
ENSG00000272335	0.441361	4.067625	0.516372	1	RP11-53O19.3	_
ENSG00000237499	0.436711	4.404854	0.516698	1	RP11-356I2.4	_
ENSG00000141376	0.458615	3.318803	0.516906	1	BCAS3	_
ENSG00000225828	0.448285	3.323397	0.516906	1	FAM229A	_
ENSG00000143942	-0.438719	4.451465	0.516951	1	CHAC2	_
ENSG00000048392	-0.431342	5.390096	0.516996	1	RRM2B	K10808
ENSG00000137414	0.431618	5.53037	0.517079	1	FAM8A1	_
ENSG00000185022	-0.434888	4.719151	0.517112	1	MAFF	K09037
ENSG00000168283	-0.428313	6.234181	0.517155	1	BMI1	K11459
ENSG00000116209	0.425269	7.553892	0.517429	1	TMEM59	_
ENSG00000050820	0.429593	5.987006	0.517786	1	BCAR1	K05726
ENSG00000112249	-0.432411	5.528345	0.517808	1	ASCC3	K18663
ENSG00000112499	0.517798	1.691597	0.517909	1	SLC22A2	K08199
ENSG00000162946	0.517798	1.691597	0.517909	1	DISC1	K16534
ENSG00000165507	0.517798	1.691597	0.517909	1	C10orf10	_
ENSG00000206535	-0.497907	1.74443	0.517909	1	LNP1	_
ENSG00000271781	-0.497907	1.74443	0.517909	1	CTD-2589H19.6	_
ENSG00000229950	0.484759	1.706785	0.517909	1	TFAP2A-AS1	_
ENSG00000129355	0.467871	2.825443	0.517975	1	CDKN2D	K06623
ENSG00000107863	0.425331	7.072279	0.518001	1	ARHGAP21	_
ENSG00000146232	-0.435551	4.65457	0.518127	1	NFKBIE	K05872
ENSG00000107537	-0.442555	4.034372	0.518226	1	PHYH	K00477
ENSG00000101544	-0.433289	4.958997	0.518471	1	ADNP2	_
ENSG00000104142	0.428305	5.650705	0.518485	1	VPS18	_

ENSG00000052723	-0.430084	5.374352	0.518616	1	SIKE1	K12656
ENSG00000230438	-0.502147	1.870557	0.518754	1	RP11-420G6.4	_
ENSG00000273014	0.496832	1.834	0.518754	1	RP11-225B17.2	_
ENSG00000198420	-0.435608	4.496753	0.518791	1	FAM115A	_
ENSG00000105364	-0.425338	6.675124	0.519012	1	MRPL4	K02926
ENSG00000075142	-0.424445	7.080575	0.519094	1	SRI	_
ENSG00000118369	0.445829	3.736324	0.519161	1	USP35	K11854
ENSG00000105246	-0.5057	1.98643	0.519446	1	EBI3	_
ENSG00000171865	-0.433968	4.297403	0.519528	1	RNASEH1	K03469
ENSG00000269893	-0.435863	4.695696	0.519704	1	SNHG8	_
ENSG00000162104	0.427002	5.792221	0.519821	1	ADCY9	K08049
ENSG00000110013	-0.436781	4.743878	0.519913	1	SIAE	K05970
ENSG00000196961	0.424319	6.549051	0.51997	1	AP2A1	K11824
ENSG00000203780	-0.490383	2.082492	0.520023	1	FANK1	_
ENSG00000110046	0.431381	4.783886	0.52011	1	ATG2A	K17906
ENSG00000147065	0.421392	9.11853	0.520189	1	MSN	_
ENSG00000100354	0.43497	4.455801	0.520229	1	TNRC6B	K17985
ENSG00000159733	0.458718	3.28768	0.520245	1	ZFYVE28	_
ENSG00000145901	-0.423592	6.873693	0.520311	1	TNIP1	_
ENSG00000148925	-0.432794	5.147401	0.520377	1	BTBD10	K10482
ENSG00000168461	0.421417	7.996626	0.5208	1	RAB31	K07891
ENSG00000140836	0.429682	5.25923	0.520812	1	ZFHX3	_
ENSG00000164902	-0.425265	5.897091	0.520829	1	PHAX	K14291
ENSG00000108448	-0.43528	4.164544	0.520883	1	TRIM16L	K09228
ENSG00000102547	-0.497674	2.276833	0.520927	1	CAB39L	K08272
ENSG00000136104	-0.42929	5.566716	0.521026	1	RNASEH2B	K10744
ENSG00000175198	-0.438929	4.212603	0.521049	1	PCCA	K01965
ENSG00000106245	-0.423387	6.528269	0.521277	1	BUD31	K12873
ENSG00000108272	-0.671074	0.416098	0.521385	1	DHRS11	K11447
ENSG00000169621	-0.671074	0.416098	0.521385	1	APLF	K13295
ENSG00000258643	-0.671074	0.416098	0.521385	1	BCL2L2-PABPN1	K14396
ENSG00000269910	-0.671074	0.416098	0.521385	1	RP11-73M18.10	_
ENSG00000272931	-0.671074	0.416098	0.521385	1	RP5-943J3.2	_
ENSG00000147697	0.586024	0.313034	0.521385	1	GSDMC	_
ENSG00000177076	0.586024	0.313034	0.521385	1	ACER2	K01441
ENSG00000228812	0.586024	0.313034	0.521385	1	RP11-157P1.5	_
ENSG00000235316	0.586024	0.313034	0.521385	1	DUSP8P5	K04459
ENSG00000258130	0.586024	0.313034	0.521385	1	RP11-347C12.3	K11364
ENSG00000111897	0.42263	6.644371	0.521427	1	SERINC1	K10408
ENSG00000089351	0.422638	6.6209	0.52147	1	GRAMD1A	_
ENSG00000074047	-0.446185	3.657831	0.521509	1	GLI2	K16798
ENSG00000180257	-0.503277	2.447888	0.5216	1	ZNF816	K09228
ENSG00000269113	0.47236	2.429448	0.5216	1	TRABD2B	_
ENSG00000106415	0.426688	5.775472	0.521697	1	GLCCI1	K03014
ENSG00000058335	-1.689238	-1.077996	0.521739	1	RASGRF1	K04349
ENSG00000084110	-1.689238	-1.077996	0.521739	1	HAL	K01745
ENSG00000101222	-1.689238	-1.077996	0.521739	1	SPEF1	_
ENSG00000102359	-1.689238	-1.077996	0.521739	1	SRPX2	K19408
ENSG00000104804	-1.689238	-1.077996	0.521739	1	TULP2	_
ENSG00000121005	-1.689238	-1.077996	0.521739	1	CRISPLD1	_
ENSG00000123700	-1.689238	-1.077996	0.521739	1	KCNJ2	K04996
ENSG00000129951	-1.689238	-1.077996	0.521739	1	LPPR3	K19582
ENSG00000132915	-1.689238	-1.077996	0.521739	1	PDE6A	K08718
ENSG00000137259	-1.689238	-1.077996	0.521739	1	HIST1H2AB	K11251
ENSG00000143494	-1.689238	-1.077996	0.521739	1	VASH2	_
ENSG00000145536	-1.689238	-1.077996	0.521739	1	ADAMTS16	K08630
ENSG00000146221	-1.689238	-1.077996	0.521739	1	TCTE1	_
ENSG00000150526	-1.689238	-1.077996	0.521739	1	MIA2	_

ENSG00000154608	-1.689238	-1.077996	0.521739	1	CEP170P1	K16463
ENSG00000161040	-1.689238	-1.077996	0.521739	1	FBXL13	K10279
ENSG00000163568	-1.689238	-1.077996	0.521739	1	AIM2	K12966
ENSG00000167483	-1.689238	-1.077996	0.521739	1	FAM129C	_
ENSG00000182853	-1.689238	-1.077996	0.521739	1	VMO1	_
ENSG00000188396	-1.689238	-1.077996	0.521739	1	TCTEX1D4	_
ENSG00000196176	-1.689238	-1.077996	0.521739	1	HIST1H4A	K11254
ENSG00000196275	-1.689238	-1.077996	0.521739	1	GTF2IRD2	K12581
ENSG00000203872	-1.689238	-1.077996	0.521739	1	C6orf163	_
ENSG00000203942	-1.689238	-1.077996	0.521739	1	C10orf62	_
ENSG00000214978	-1.689238	-1.077996	0.521739	1	RP11-477N12.3	K11167
ENSG00000218690	-1.689238	-1.077996	0.521739	1	HIST1H2APS4	K11251
ENSG00000226266	-1.689238	-1.077996	0.521739	1	AC009961.3	_
ENSG00000227252	-1.689238	-1.077996	0.521739	1	AC105760.2	_
ENSG00000227450	-1.689238	-1.077996	0.521739	1	CTB-58E17.5	_
ENSG00000228196	-1.689238	-1.077996	0.521739	1	PTPN2P1	K18026
ENSG00000228218	-1.689238	-1.077996	0.521739	1	ATF4P3	K04374
ENSG00000228802	-1.689238	-1.077996	0.521739	1	AC073641.2	_
ENSG00000231672	-1.689238	-1.077996	0.521739	1	DIRC3	_
ENSG00000233250	-1.689238	-1.077996	0.521739	1	RP3-339A18.6	_
ENSG00000234382	-1.689238	-1.077996	0.521739	1	RP11-40F6.1	K01896
ENSG00000234911	-1.689238	-1.077996	0.521739	1	TEX21P	_
ENSG00000239884	-1.689238	-1.077996	0.521739	1	RN7SL608P	_
ENSG00000242391	-1.689238	-1.077996	0.521739	1	RP4-631H13.2	_
ENSG00000242866	-1.689238	-1.077996	0.521739	1	STRC	_
ENSG00000244441	-1.689238	-1.077996	0.521739	1	RP11-359H3.1	K02915
ENSG00000244607	-1.689238	-1.077996	0.521739	1	CCDC13	_
ENSG00000246731	-1.689238	-1.077996	0.521739	1	CTD-2514K5.2	_
ENSG00000248256	-1.689238	-1.077996	0.521739	1	OCIAD1-AS1	_
ENSG00000251365	-1.689238	-1.077996	0.521739	1	RP11-332J15.3	_
ENSG00000251563	-1.689238	-1.077996	0.521739	1	AF121898.1	K01870
ENSG00000259775	-1.689238	-1.077996	0.521739	1	RP11-45P15.4	_
ENSG00000260814	-1.689238	-1.077996	0.521739	1	RP11-107F6.3	_
ENSG00000267577	-1.689238	-1.077996	0.521739	1	CTD-2587H24.5	_
ENSG00000268863	-1.689238	-1.077996	0.521739	1	AC135048.1	K12408
ENSG00000272368	-1.689238	-1.077996	0.521739	1	RP4-605O3.4	_
ENSG00000272954	-1.689238	-1.077996	0.521739	1	KB-1440D3.13	_
ENSG00000007402	1.404449	-1.13734	0.521739	1	CACNA2D2	K04859
ENSG00000014914	1.404449	-1.13734	0.521739	1	MTMR11	K18085
ENSG00000099769	1.404449	-1.13734	0.521739	1	IGFALS	K17256
ENSG00000108654	1.404449	-1.13734	0.521739	1	DDX5	K12823
ENSG00000129244	1.404449	-1.13734	0.521739	1	ATP1B2	K01540
ENSG00000131779	1.404449	-1.13734	0.521739	1	PEX11B	K13352
ENSG00000136274	1.404449	-1.13734	0.521739	1	NACAD	K03626
ENSG00000140993	1.404449	-1.13734	0.521739	1	TIGD7	_
ENSG00000144785	1.404449	-1.13734	0.521739	1	RP11-977G19.10	K01647
ENSG00000158008	1.404449	-1.13734	0.521739	1	EXTL1	_
ENSG00000160716	1.404449	-1.13734	0.521739	1	CHRNB2	K04813
ENSG00000167191	1.404449	-1.13734	0.521739	1	GPRC5B	K04619
ENSG00000168481	1.404449	-1.13734	0.521739	1	LGI3	_
ENSG00000171766	1.404449	-1.13734	0.521739	1	GATM	K00613
ENSG00000176515	1.404449	-1.13734	0.521739	1	AL033381.1	_
ENSG00000203827	1.404449	-1.13734	0.521739	1	NBPF16	_
ENSG00000205755	1.404449	-1.13734	0.521739	1	CRLF2	K05078
ENSG00000205853	1.404449	-1.13734	0.521739	1	RFPL3S	_
ENSG00000206228	1.404449	-1.13734	0.521739	1	HNRNPA1P4	K12741
ENSG00000222018	1.404449	-1.13734	0.521739	1	C21orf140	_
ENSG00000223510	1.404449	-1.13734	0.521739	1	CDRT15	_

ENSG00000224886	1.404449	-1.13734	0.521739	1	BEND3P3	_
ENSG00000225544	1.404449	-1.13734	0.521739	1	KB-1027C11.4	_
ENSG00000227373	1.404449	-1.13734	0.521739	1	RP11-160H22.5	_
ENSG00000227638	1.404449	-1.13734	0.521739	1	HNRNPA1P14	K12741
ENSG00000228201	1.404449	-1.13734	0.521739	1	AL022341.3	_
ENSG00000228903	1.404449	-1.13734	0.521739	1	RASA4CP	K17630
ENSG00000229472	1.404449	-1.13734	0.521739	1	RP1-64K7.4	_
ENSG00000230107	1.404449	-1.13734	0.521739	1	CTA-126B4.7	_
ENSG00000230567	1.404449	-1.13734	0.521739	1	FAM203B	_
ENSG00000231609	1.404449	-1.13734	0.521739	1	AC009501.4	_
ENSG00000232901	1.404449	-1.13734	0.521739	1	CYCSP10	K08738
ENSG00000233622	1.404449	-1.13734	0.521739	1	CYP2T2P	K07421
ENSG00000234917	1.404449	-1.13734	0.521739	1	RP5-994D16.3	_
ENSG00000235837	1.404449	-1.13734	0.521739	1	AC073333.8	_
ENSG00000238018	1.404449	-1.13734	0.521739	1	AC093110.3	_
ENSG00000240135	1.404449	-1.13734	0.521739	1	PSMD12P	K03035
ENSG00000242337	1.404449	-1.13734	0.521739	1	TFP1	K14736
ENSG00000248588	1.404449	-1.13734	0.521739	1	CTC-458G6.4	_
ENSG00000250900	1.404449	-1.13734	0.521739	1	CTC-338M12.6	_
ENSG00000252981	1.404449	-1.13734	0.521739	1	U3	_
ENSG00000253837	1.404449	-1.13734	0.521739	1	RP11-177H13.2	_
ENSG00000258559	1.404449	-1.13734	0.521739	1	AC005519.4	_
ENSG00000259982	1.404449	-1.13734	0.521739	1	CDC37P1	K09554
ENSG00000260259	1.404449	-1.13734	0.521739	1	RP11-368I7.4	_
ENSG00000260681	1.404449	-1.13734	0.521739	1	CTA-363E6.5	_
ENSG00000262296	1.404449	-1.13734	0.521739	1	RP11-55L4.1	_
ENSG00000263853	1.404449	-1.13734	0.521739	1	AC139530.1	_
ENSG00000263990	1.404449	-1.13734	0.521739	1	CTC-542B22.2	_
ENSG00000267649	1.404449	-1.13734	0.521739	1	CTD-2587H24.10	_
ENSG00000271843	1.404449	-1.13734	0.521739	1	RP11-245J9.5	_
ENSG00000272030	1.404449	-1.13734	0.521739	1	RP1-178F15.4	_
ENSG00000272092	1.404449	-1.13734	0.521739	1	RP11-350N15.5	_
ENSG00000272792	1.404449	-1.13734	0.521739	1	RP11-141C7.4	_
ENSG00000273437	1.404449	-1.13734	0.521739	1	RP11-434H6.7	_
ENSG00000188856	0.428138	4.909083	0.521769	1	RPSAP47	K02998
ENSG00000157224	-0.42633	5.604094	0.521888	1	CLDN12	K13194
ENSG00000163251	0.434773	3.775479	0.521908	1	FZD5	K02375
ENSG00000163946	-0.422043	6.205421	0.522296	1	FAM208A	_
ENSG00000183655	0.468309	2.781473	0.522706	1	KLHL25	K10462
ENSG00000149679	-0.459234	2.801587	0.522706	1	CABLES2	_
ENSG00000184220	-0.42309	6.194701	0.522759	1	CMSS1	_
ENSG00000170786	-0.420529	7.138589	0.522855	1	SDR16C5	K15734
ENSG00000168143	0.446337	2.898402	0.523009	1	FAM83B	_
ENSG00000255135	0.446337	2.898402	0.523009	1	RP11-111M22.3	_
ENSG00000136802	0.426755	5.4462	0.523062	1	LRRC8A	_
ENSG00000205189	0.421755	6.134384	0.523135	1	ZBTB10	K10497
ENSG00000170577	-0.431017	5.016925	0.523152	1	SIX2	K19472
ENSG00000109466	-0.433752	4.514091	0.523533	1	KLHL2	K10443
ENSG00000138050	-0.430418	4.512154	0.523533	1	THUMPD2	_
ENSG00000232119	-0.420507	6.369526	0.523553	1	MCTS1	K07575
ENSG00000183020	0.422845	5.813467	0.523708	1	AP2A2	K11824
ENSG00000008438	-4.235765	-1.844995	0.52381	1	PGLYRP1	K01446
ENSG00000042781	-4.235765	-1.844995	0.52381	1	USH2A	K19636
ENSG00000073282	-4.235765	-1.844995	0.52381	1	TP63	K10149
ENSG00000079263	-4.235765	-1.844995	0.52381	1	SP140	_
ENSG00000084453	-4.235765	-1.844995	0.52381	1	SLCO1A2	K03460
ENSG00000086570	-4.235765	-1.844995	0.52381	1	FAT2	K16506
ENSG00000088538	-4.235765	-1.844995	0.52381	1	DOCK3	K05727

ENSG00000104361	-4.235765	-1.844995	0.52381	1	NIPAL2	_
ENSG00000113578	-4.235765	-1.844995	0.52381	1	FGF1	K18496
ENSG00000115523	-4.235765	-1.844995	0.52381	1	GNLY	_
ENSG00000116194	-4.235765	-1.844995	0.52381	1	ANGPTL1	_
ENSG00000118990	-4.235765	-1.844995	0.52381	1	GLRXP3	K03676
ENSG00000121406	-4.235765	-1.844995	0.52381	1	ZNF549	K09228
ENSG00000123329	-4.235765	-1.844995	0.52381	1	ARHGAP9	_
ENSG00000123575	-4.235765	-1.844995	0.52381	1	FAM199X	_
ENSG00000124257	-4.235765	-1.844995	0.52381	1	NEURL2	K16782
ENSG00000125409	-4.235765	-1.844995	0.52381	1	TEKT3	K18630
ENSG00000126012	-4.235765	-1.844995	0.52381	1	KDM5C	K11446
ENSG00000129009	-4.235765	-1.844995	0.52381	1	ISLR	_
ENSG00000131016	-4.235765	-1.844995	0.52381	1	AKAP12	K16528
ENSG00000139971	-4.235765	-1.844995	0.52381	1	C14orf37	_
ENSG00000140932	-4.235765	-1.844995	0.52381	1	CMTM2	_
ENSG00000141052	-4.235765	-1.844995	0.52381	1	MYOCD	_
ENSG00000141150	-4.235765	-1.844995	0.52381	1	RASL10B	K02927
ENSG00000143355	-4.235765	-1.844995	0.52381	1	LHX9	K09373
ENSG00000145675	-4.235765	-1.844995	0.52381	1	PIK3R1	K02649
ENSG00000145700	-4.235765	-1.844995	0.52381	1	ANKRD31	_
ENSG00000145757	-4.235765	-1.844995	0.52381	1	SPATA9	_
ENSG00000147488	-4.235765	-1.844995	0.52381	1	ST18	_
ENSG00000149927	-4.235765	-1.844995	0.52381	1	DOC2A	_
ENSG00000152128	-4.235765	-1.844995	0.52381	1	TMEM163	_
ENSG00000152661	-4.235765	-1.844995	0.52381	1	GJA1	K07372
ENSG00000153208	-4.235765	-1.844995	0.52381	1	MERTK	K05117
ENSG00000160179	-4.235765	-1.844995	0.52381	1	ABCG1	K05679
ENSG00000161896	-4.235765	-1.844995	0.52381	1	IP6K3	K07756
ENSG00000162438	-4.235765	-1.844995	0.52381	1	CTRC	K01311
ENSG00000162460	-4.235765	-1.844995	0.52381	1	TMEM82	_
ENSG00000162551	-4.235765	-1.844995	0.52381	1	ALPL	K01077
ENSG00000163879	-4.235765	-1.844995	0.52381	1	DNALI1	K10410
ENSG00000164385	-4.235765	-1.844995	0.52381	1	C6orf195	_
ENSG00000164512	-4.235765	-1.844995	0.52381	1	ANKRD55	_
ENSG00000166432	-4.235765	-1.844995	0.52381	1	ZMAT1	_
ENSG00000166667	-4.235765	-1.844995	0.52381	1	SPDYE6	K08694
ENSG00000167178	-4.235765	-1.844995	0.52381	1	ISLR2	_
ENSG00000168060	-4.235765	-1.844995	0.52381	1	NAALADL1	K01301
ENSG00000168367	-4.235765	-1.844995	0.52381	1	LINC00917	_
ENSG00000171121	-4.235765	-1.844995	0.52381	1	KCNMB3	K04939
ENSG00000173811	-4.235765	-1.844995	0.52381	1	CCDC13-AS1	_
ENSG00000174844	-4.235765	-1.844995	0.52381	1	DNAH12	K10408
ENSG00000174992	-4.235765	-1.844995	0.52381	1	ZG16	K12581
ENSG00000175147	-4.235765	-1.844995	0.52381	1	TMEM51-AS1	_
ENSG00000175518	-4.235765	-1.844995	0.52381	1	UBQLNL	K04523
ENSG00000176009	-4.235765	-1.844995	0.52381	1	ASCL3	K09067
ENSG00000176746	-4.235765	-1.844995	0.52381	1	MAGEB6	_
ENSG00000177770	-4.235765	-1.844995	0.52381	1	CDKN2AIPNLP1	_
ENSG00000179133	-4.235765	-1.844995	0.52381	1	C10orf67	_
ENSG00000181819	-4.235765	-1.844995	0.52381	1	KCTD9P2	_
ENSG00000182611	-4.235765	-1.844995	0.52381	1	HIST1H2AJ	K11251
ENSG00000183571	-4.235765	-1.844995	0.52381	1	PGPEP1L	_
ENSG00000183801	-4.235765	-1.844995	0.52381	1	OLFML1	_
ENSG00000184530	-4.235765	-1.844995	0.52381	1	C6orf58	_
ENSG00000184838	-4.235765	-1.844995	0.52381	1	PRR16	K02159
ENSG00000185015	-4.235765	-1.844995	0.52381	1	CA13	K01672
ENSG00000185296	-4.235765	-1.844995	0.52381	1	CTD-2139B15.1	K16830
ENSG00000185990	-4.235765	-1.844995	0.52381	1	F8A3	_

ENSG00000186038	-4.235765	-1.844995	0.52381	1	HTR3E	K04819
ENSG00000188032	-4.235765	-1.844995	0.52381	1	C19orf67	_
ENSG00000196266	-4.235765	-1.844995	0.52381	1	OR10J3	K04257
ENSG00000196302	-4.235765	-1.844995	0.52381	1	RP11-497H16.5	K01195
ENSG00000197497	-4.235765	-1.844995	0.52381	1	ZNF665	K17985
ENSG00000197859	-4.235765	-1.844995	0.52381	1	ADAMTSL2	_
ENSG00000198570	-4.235765	-1.844995	0.52381	1	RD3	K09228
ENSG00000198744	-4.235765	-1.844995	0.52381	1	RP5-857K21.11	K02262
ENSG00000199900	-4.235765	-1.844995	0.52381	1	RNA5SP463	_
ENSG00000200924	-4.235765	-1.844995	0.52381	1	RNU6-1048P	_
ENSG00000202078	-4.235765	-1.844995	0.52381	1	Y_RNA	_
ENSG00000202358	-4.235765	-1.844995	0.52381	1	RNU6-652P	_
ENSG00000203443	-4.235765	-1.844995	0.52381	1	AC092964.1	K16467
ENSG00000203546	-4.235765	-1.844995	0.52381	1	RP11-176H8.1	K12581
ENSG00000203721	-4.235765	-1.844995	0.52381	1	LINC00862	_
ENSG00000203886	-4.235765	-1.844995	0.52381	1	CYP17A1-AS1	_
ENSG00000203910	-4.235765	-1.844995	0.52381	1	C1orf146	K10408
ENSG00000204177	-4.235765	-1.844995	0.52381	1	BMS1P1	K14569
ENSG00000204532	-4.235765	-1.844995	0.52381	1	ZSCAN5C	K09230
ENSG00000205236	-4.235765	-1.844995	0.52381	1	RP11-514P8.6	K17630
ENSG00000205300	-4.235765	-1.844995	0.52381	1	RP11-352D3.2	_
ENSG00000205578	-4.235765	-1.844995	0.52381	1	POM121B	K14316
ENSG00000206633	-4.235765	-1.844995	0.52381	1	SNORA80B	_
ENSG00000206878	-4.235765	-1.844995	0.52381	1	SNORA51	_
ENSG00000207399	-4.235765	-1.844995	0.52381	1	RNU6-1011P	_
ENSG00000207457	-4.235765	-1.844995	0.52381	1	RNU6-476P	_
ENSG00000207468	-4.235765	-1.844995	0.52381	1	SNORA19	_
ENSG00000207944	-4.235765	-1.844995	0.52381	1	MIR574	_
ENSG00000213221	-4.235765	-1.844995	0.52381	1	DNLZ	K17808
ENSG00000213958	-4.235765	-1.844995	0.52381	1	KRT18P29	K07604
ENSG00000213967	-4.235765	-1.844995	0.52381	1	ZNF726	K09228
ENSG00000214694	-4.235765	-1.844995	0.52381	1	ARHGEF33	_
ENSG00000214810	-4.235765	-1.844995	0.52381	1	CYCSP55	K08738
ENSG00000215241	-4.235765	-1.844995	0.52381	1	RP11-266K4.9	_
ENSG00000215527	-4.235765	-1.844995	0.52381	1	AP005482.1	_
ENSG00000216359	-4.235765	-1.844995	0.52381	1	RP11-360O19.1	K00742
ENSG00000217027	-4.235765	-1.844995	0.52381	1	TPT1P4	_
ENSG00000218048	-4.235765	-1.844995	0.52381	1	RP3-407E4.4	_
ENSG00000221241	-4.235765	-1.844995	0.52381	1	SNORD88A	_
ENSG00000221555	-4.235765	-1.844995	0.52381	1	AC068669.1	_
ENSG00000223678	-4.235765	-1.844995	0.52381	1	RP11-311H10.4	_
ENSG00000223703	-4.235765	-1.844995	0.52381	1	AC027612.4	K06522
ENSG00000224451	-4.235765	-1.844995	0.52381	1	ATP5F1P1	K02127
ENSG00000224459	-4.235765	-1.844995	0.52381	1	RP11-169K16.4	_
ENSG00000224595	-4.235765	-1.844995	0.52381	1	AC073626.2	_
ENSG00000224621	-4.235765	-1.844995	0.52381	1	RP11-276H7.3	_
ENSG00000224746	-4.235765	-1.844995	0.52381	1	AC015987.1	_
ENSG00000225419	-4.235765	-1.844995	0.52381	1	RPL21P3	K02889
ENSG00000225434	-4.235765	-1.844995	0.52381	1	RP11-63P12.6	_
ENSG00000225527	-4.235765	-1.844995	0.52381	1	RP11-383B4.4	_
ENSG00000226701	-4.235765	-1.844995	0.52381	1	RP11-570G20.1	K02877
ENSG00000226781	-4.235765	-1.844995	0.52381	1	TBCAP1	K17292
ENSG00000226856	-4.235765	-1.844995	0.52381	1	AC093901.1	_
ENSG00000227558	-4.235765	-1.844995	0.52381	1	PGM5P2	K15636
ENSG00000227591	-4.235765	-1.844995	0.52381	1	RP1-28O10.1	_
ENSG00000227973	-4.235765	-1.844995	0.52381	1	PIN4P1	K09579
ENSG00000228208	-4.235765	-1.844995	0.52381	1	C1orf143	_
ENSG00000228327	-4.235765	-1.844995	0.52381	1	RP11-206L10.2	_

ENSG00000228604	-4.235765	-1.844995	0.52381	1	RP5-872K7.7	_
ENSG00000228707	-4.235765	-1.844995	0.52381	1	RP11-787B4.2	_
ENSG00000228818	-4.235765	-1.844995	0.52381	1	RP11-567G24.1	K03028
ENSG00000229105	-4.235765	-1.844995	0.52381	1	RP11-264C15.2	_
ENSG00000229526	-4.235765	-1.844995	0.52381	1	KRT16P4	K07604
ENSG00000229800	-4.235765	-1.844995	0.52381	1	ATP8A2P2	K14802
ENSG00000229841	-4.235765	-1.844995	0.52381	1	RP11-361K17.2	_
ENSG00000229955	-4.235765	-1.844995	0.52381	1	RP1-5O6.4	_
ENSG00000230533	-4.235765	-1.844995	0.52381	1	RP11-95M15.1	_
ENSG00000230604	-4.235765	-1.844995	0.52381	1	TSEN15P2	K15324
ENSG00000230807	-4.235765	-1.844995	0.52381	1	AC099535.4	K02927
ENSG00000231028	-4.235765	-1.844995	0.52381	1	LINC00271	_
ENSG00000231298	-4.235765	-1.844995	0.52381	1	LINC00704	_
ENSG00000231369	-4.235765	-1.844995	0.52381	1	RP1-40G4P.1	K02877
ENSG00000231724	-4.235765	-1.844995	0.52381	1	RP11-573D15.3	_
ENSG00000232194	-4.235765	-1.844995	0.52381	1	RP1-313L4.3	_
ENSG00000232621	-4.235765	-1.844995	0.52381	1	C4BPAP2	K04002
ENSG00000232684	-4.235765	-1.844995	0.52381	1	ATP11A-AS1	_
ENSG00000232862	-4.235765	-1.844995	0.52381	1	RP4-665N4.4	K19478
ENSG00000233052	-4.235765	-1.844995	0.52381	1	RP11-398B16.2	_
ENSG00000233108	-4.235765	-1.844995	0.52381	1	AC006042.7	_
ENSG00000233776	-4.235765	-1.844995	0.52381	1	RP11-384P7.6	_
ENSG00000233783	-4.235765	-1.844995	0.52381	1	AP001442.2	_
ENSG00000233998	-4.235765	-1.844995	0.52381	1	SETP5	K11290
ENSG00000234141	-4.235765	-1.844995	0.52381	1	AC009473.1	_
ENSG00000234409	-4.235765	-1.844995	0.52381	1	AC006547.14	_
ENSG00000235029	-4.235765	-1.844995	0.52381	1	MNX1-AS2	_
ENSG00000235354	-4.235765	-1.844995	0.52381	1	CTA-276O3.4	K02980
ENSG00000235652	-4.235765	-1.844995	0.52381	1	RP11-545I5.3	_
ENSG00000235703	-4.235765	-1.844995	0.52381	1	LINC00894	_
ENSG00000236013	-4.235765	-1.844995	0.52381	1	RP3-332B22.1	_
ENSG00000236417	-4.235765	-1.844995	0.52381	1	CTSLP1	K01365
ENSG00000236824	-4.235765	-1.844995	0.52381	1	BCYRN1	_
ENSG00000236866	-4.235765	-1.844995	0.52381	1	AL157902.3	_
ENSG00000237232	-4.235765	-1.844995	0.52381	1	ZNF295-AS1	_
ENSG00000237721	-4.235765	-1.844995	0.52381	1	AF064858.11	_
ENSG00000237805	-4.235765	-1.844995	0.52381	1	AC015849.2	_
ENSG00000237848	-4.235765	-1.844995	0.52381	1	RP11-739N20.3	K11098
ENSG00000237877	-4.235765	-1.844995	0.52381	1	AC097500.2	_
ENSG00000238109	-4.235765	-1.844995	0.52381	1	AC004893.10	K11971
ENSG00000238549	-4.235765	-1.844995	0.52381	1	snoU13	_
ENSG00000240429	-4.235765	-1.844995	0.52381	1	LRRFIP1P1	K17785
ENSG00000240718	-4.235765	-1.844995	0.52381	1	RN7SL851P	_
ENSG00000240739	-4.235765	-1.844995	0.52381	1	RP11-69M1.1	_
ENSG00000240823	-4.235765	-1.844995	0.52381	1	RN7SL23P	_
ENSG00000241150	-4.235765	-1.844995	0.52381	1	RP11-308D16.2	K17086
ENSG00000241720	-4.235765	-1.844995	0.52381	1	RP4-735C1.4	_
ENSG00000241933	-4.235765	-1.844995	0.52381	1	RP11-755B10.3	_
ENSG00000243636	-4.235765	-1.844995	0.52381	1	RP11-164O23.7	_
ENSG00000243979	-4.235765	-1.844995	0.52381	1	RP11-347C18.1	K02880
ENSG00000244184	-4.235765	-1.844995	0.52381	1	RP11-314A20.2	_
ENSG00000244291	-4.235765	-1.844995	0.52381	1	C7orf13	_
ENSG00000246740	-4.235765	-1.844995	0.52381	1	CTD-2382E5.1	_
ENSG00000247151	-4.235765	-1.844995	0.52381	1	CSTF3-AS1	_
ENSG00000248884	-4.235765	-1.844995	0.52381	1	CTC-537E7.3	_
ENSG00000248896	-4.235765	-1.844995	0.52381	1	CTD-2135J3.3	_
ENSG00000248908	-4.235765	-1.844995	0.52381	1	RP11-730N24.1	_
ENSG00000248964	-4.235765	-1.844995	0.52381	1	RP11-94H18.1	_

ENSG00000249326	-4.235765	-1.844995	0.52381	1	CTD-2194D22.4	_
ENSG00000250444	-4.235765	-1.844995	0.52381	1	CCT5P1	K09497
ENSG00000251018	-4.235765	-1.844995	0.52381	1	RP11-80G7.1	_
ENSG00000251271	-4.235765	-1.844995	0.52381	1	ALG1L7P	K03842
ENSG00000251821	-4.235765	-1.844995	0.52381	1	RNU6-583P	_
ENSG00000251969	-4.235765	-1.844995	0.52381	1	AL356776.1	_
ENSG00000252579	-4.235765	-1.844995	0.52381	1	RNU7-117P	_
ENSG00000252797	-4.235765	-1.844995	0.52381	1	RN7SKP272	_
ENSG00000252827	-4.235765	-1.844995	0.52381	1	RN7SKP11	_
ENSG00000253273	-4.235765	-1.844995	0.52381	1	RP11-99A14.1	K16449
ENSG00000253595	-4.235765	-1.844995	0.52381	1	CTD-3064M3.1	_
ENSG00000253848	-4.235765	-1.844995	0.52381	1	RP11-10N23.5	_
ENSG00000253910	-4.235765	-1.844995	0.52381	1	PCDHGB2	K16496
ENSG00000253971	-4.235765	-1.844995	0.52381	1	CDC42P3	K04393
ENSG00000254693	-4.235765	-1.844995	0.52381	1	RP11-58K22.5	_
ENSG00000254694	-4.235765	-1.844995	0.52381	1	RP11-50B3.4	_
ENSG00000255085	-4.235765	-1.844995	0.52381	1	AF186192.5	K09228
ENSG00000255439	-4.235765	-1.844995	0.52381	1	RP11-196G11.1	K05357
ENSG00000255553	-4.235765	-1.844995	0.52381	1	RP11-810P12.5	_
ENSG00000255629	-4.235765	-1.844995	0.52381	1	RP11-196H14.3	_
ENSG00000256812	-4.235765	-1.844995	0.52381	1	CAPNS2	K08584
ENSG00000257496	-4.235765	-1.844995	0.52381	1	RP11-474P2.4	_
ENSG00000257790	-4.235765	-1.844995	0.52381	1	RP11-1136G11.6	K03257
ENSG00000257831	-4.235765	-1.844995	0.52381	1	RP11-596D21.1	_
ENSG00000257941	-4.235765	-1.844995	0.52381	1	RP11-290L1.4	_
ENSG00000258044	-4.235765	-1.844995	0.52381	1	RP11-530C5.2	_
ENSG00000258199	-4.235765	-1.844995	0.52381	1	RP11-977G19.5	_
ENSG00000258210	-4.235765	-1.844995	0.52381	1	RP11-478C19.2	_
ENSG00000258458	-4.235765	-1.844995	0.52381	1	CTD-2555K7.2	_
ENSG00000258466	-4.235765	-1.844995	0.52381	1	RP11-1012A1.4	K11152
ENSG00000258526	-4.235765	-1.844995	0.52381	1	RP11-111A21.1	_
ENSG00000258583	-4.235765	-1.844995	0.52381	1	RP11-112J1.2	_
ENSG00000259138	-4.235765	-1.844995	0.52381	1	RP11-950C14.7	_
ENSG00000259151	-4.235765	-1.844995	0.52381	1	CAP2P1	K17261
ENSG00000259364	-4.235765	-1.844995	0.52381	1	RP11-64K12.9	_
ENSG00000259450	-4.235765	-1.844995	0.52381	1	RP11-265N7.1	_
ENSG00000259469	-4.235765	-1.844995	0.52381	1	RP11-227D13.4	_
ENSG00000259472	-4.235765	-1.844995	0.52381	1	RP13-996F3.3	K08622
ENSG00000259642	-4.235765	-1.844995	0.52381	1	C15orf37	K16580
ENSG00000259796	-4.235765	-1.844995	0.52381	1	RP11-355I22.7	_
ENSG00000259931	-4.235765	-1.844995	0.52381	1	CTD-2323K18.2	K11098
ENSG00000260541	-4.235765	-1.844995	0.52381	1	LA16c-429E7.1	_
ENSG00000260588	-4.235765	-1.844995	0.52381	1	RP11-930P14.2	_
ENSG00000260648	-4.235765	-1.844995	0.52381	1	RP11-133K1.7	_
ENSG00000260782	-4.235765	-1.844995	0.52381	1	RP11-480G7.2	K09040
ENSG00000260996	-4.235765	-1.844995	0.52381	1	RP13-122B23.8	_
ENSG00000261072	-4.235765	-1.844995	0.52381	1	RP11-5N19.3	K00022
ENSG00000261357	-4.235765	-1.844995	0.52381	1	RP11-626G11.1	_
ENSG00000261471	-4.235765	-1.844995	0.52381	1	RP11-61F12.1	_
ENSG00000261550	-4.235765	-1.844995	0.52381	1	RP11-467J12.4	_
ENSG00000261864	-4.235765	-1.844995	0.52381	1	RP11-67A1.2	_
ENSG00000262188	-4.235765	-1.844995	0.52381	1	RP11-353N14.4	_
ENSG00000264229	-4.235765	-1.844995	0.52381	1	RNU4ATAC	_
ENSG00000264743	-4.235765	-1.844995	0.52381	1	DPRXP4	_
ENSG00000264829	-4.235765	-1.844995	0.52381	1	RP11-474I11.8	_
ENSG00000264937	-4.235765	-1.844995	0.52381	1	AC100830.5	_
ENSG00000265888	-4.235765	-1.844995	0.52381	1	RP11-408H20.2	_
ENSG00000266347	-4.235765	-1.844995	0.52381	1	AC068641.1	_

ENSG00000266602	-4.235765	-1.844995	0.52381	1	RP11-476K15.1	_
ENSG00000267138	-4.235765	-1.844995	0.52381	1	AC005954.3	_
ENSG00000267150	-4.235765	-1.844995	0.52381	1	RP11-411B10.2	_
ENSG00000267197	-4.235765	-1.844995	0.52381	1	CTC-429L19.3	_
ENSG00000267323	-4.235765	-1.844995	0.52381	1	SLC25A1P5	K15100
ENSG00000267356	-4.235765	-1.844995	0.52381	1	RP11-411B10.3	_
ENSG00000267501	-4.235765	-1.844995	0.52381	1	RP11-108P20.2	_
ENSG00000267959	-4.235765	-1.844995	0.52381	1	MIR3188	_
ENSG00000268047	-4.235765	-1.844995	0.52381	1	AC018766.6	_
ENSG00000268297	-4.235765	-1.844995	0.52381	1	CLEC4GP1	K10061
ENSG00000269161	-4.235765	-1.844995	0.52381	1	CTD-3131K8.3	_
ENSG00000269202	-4.235765	-1.844995	0.52381	1	RP4-614O4.12	_
ENSG00000269296	-4.235765	-1.844995	0.52381	1	AC005614.3	_
ENSG00000269559	-4.235765	-1.844995	0.52381	1	AC093677.1	K13403
ENSG00000269700	-4.235765	-1.844995	0.52381	1	AC069547.2	_
ENSG00000269921	-4.235765	-1.844995	0.52381	1	RP11-646I6.5	_
ENSG00000269980	-4.235765	-1.844995	0.52381	1	RP11-282O18.7	_
ENSG00000270021	-4.235765	-1.844995	0.52381	1	CTC-203F4.2	_
ENSG00000270503	-4.235765	-1.844995	0.52381	1	RP11-407N17.6	_
ENSG00000270571	-4.235765	-1.844995	0.52381	1	RP11-355F16.1	_
ENSG00000270781	-4.235765	-1.844995	0.52381	1	RP11-501C14.9	K02922
ENSG00000270983	-4.235765	-1.844995	0.52381	1	RP11-137J7.3	K10685
ENSG00000271218	-4.235765	-1.844995	0.52381	1	RP3-523E19.2	_
ENSG00000271803	-4.235765	-1.844995	0.52381	1	RP1-63M2.5	_
ENSG00000271816	-4.235765	-1.844995	0.52381	1	RP11-574K11.28	_
ENSG00000271967	-4.235765	-1.844995	0.52381	1	RP11-134K13.4	_
ENSG00000272048	-4.235765	-1.844995	0.52381	1	RP11-458N5.1	_
ENSG00000272071	-4.235765	-1.844995	0.52381	1	RP11-332J15.4	_
ENSG00000272622	-4.235765	-1.844995	0.52381	1	RP11-395N3.2	_
ENSG00000272647	-4.235765	-1.844995	0.52381	1	GS1-259H13.10	K09229
ENSG00000272884	-4.235765	-1.844995	0.52381	1	RP11-104H15.10	_
ENSG00000272894	-4.235765	-1.844995	0.52381	1	RP5-1159O4.1	_
ENSG00000273010	-4.235765	-1.844995	0.52381	1	RP11-96K19.5	_
ENSG00000273073	-4.235765	-1.844995	0.52381	1	RP11-109E12.1	_
ENSG00000273348	-4.235765	-1.844995	0.52381	1	RP11-535A5.1	_
ENSG00000273432	-4.235765	-1.844995	0.52381	1	RP5-1165K10.2	_
ENSG00000116141	-0.441468	3.318857	0.523815	1	MARK1	K08798
ENSG00000072849	-0.424741	5.473245	0.523911	1	DERL2	K13989
ENSG00000153485	-0.452984	3.484814	0.524074	1	TMEM251	_
ENSG00000072756	-0.427982	5.034388	0.524273	1	TRNT1	K00974
ENSG00000018189	-0.437983	3.702146	0.524364	1	RUFY3	K12482
ENSG00000156831	-0.42473	5.148622	0.524402	1	NSMCE2	_
ENSG00000172638	-0.644465	0.71657	0.524474	1	EFEMP2	K08569
ENSG00000213250	-0.644465	0.71657	0.524474	1	RBMS2P1	_
ENSG00000233381	-0.644465	0.71657	0.524474	1	AK4P3	K00939
ENSG00000261139	-0.644465	0.71657	0.524474	1	CTD-2517M14.5	_
ENSG00000168779	-0.594136	0.685575	0.524474	1	SHOX2	K09331
ENSG00000103544	0.593997	0.622115	0.524474	1	C16orf62	_
ENSG00000113555	0.593997	0.622115	0.524474	1	PCDH12	K16499
ENSG00000167771	0.593997	0.622115	0.524474	1	RCOR2	_
ENSG00000237883	0.593997	0.622115	0.524474	1	DGUOK-AS1	_
ENSG00000272097	0.593997	0.622115	0.524474	1	RP11-421M1.8	_
ENSG00000114529	-0.444131	3.858364	0.524546	1	C3orf52	_
ENSG00000197774	-0.438859	3.855277	0.524546	1	EME2	K10883
ENSG00000113318	-0.438039	4.200738	0.524868	1	MSH3	K08736
ENSG00000122417	-0.421595	5.66834	0.524871	1	ODF2L	_
ENSG00000101057	0.416963	8.419088	0.524908	1	MYBL2	K09421
ENSG00000227500	0.431672	4.392392	0.525016	1	SCAMP4	_

ENSG00000125247	-0.436428	4.421116	0.52503	1	TMTC4	_
ENSG00000164294	-0.418016	7.234149	0.525188	1	GPX8	K00432
ENSG00000185624	0.415639	9.985365	0.525641	1	P4HB	K09580
ENSG00000121966	0.418618	6.294647	0.525708	1	CXCR4	K04189
ENSG00000170027	0.415728	8.56266	0.52605	1	YWHAG	K16198
ENSG00000196366	0.599193	0.877561	0.526087	1	C9orf163	_
ENSG00000212123	0.599193	0.877561	0.526087	1	PRR22	_
ENSG00000251151	0.599193	0.877561	0.526087	1	HOXC-AS3	_
ENSG00000253931	0.599193	0.877561	0.526087	1	RP11-909N17.2	_
ENSG00000271533	0.599193	0.877561	0.526087	1	RP3-368A4.6	_
ENSG00000272716	0.599193	0.877561	0.526087	1	RP11-563N4.1	_
ENSG00000231889	-0.585582	0.938716	0.526087	1	TRAF3IP2-AS1	_
ENSG00000245248	-0.585582	0.938716	0.526087	1	USP2-AS1	_
ENSG00000248932	-0.585582	0.938716	0.526087	1	RP11-319G6.1	_
ENSG00000183258	0.418464	6.36617	0.526154	1	DDX41	K13116
ENSG00000152242	0.423393	4.926539	0.526487	1	C18orf25	_
ENSG00000153094	0.423905	4.869065	0.526508	1	BCL2L11	K16341
ENSG00000183605	-0.4252	4.798341	0.526536	1	SFXN4	_
ENSG00000133104	-0.417691	6.652269	0.5266	1	SPG20	K19366
ENSG00000108578	-0.418793	6.277733	0.526629	1	BLMH	K01372
ENSG00000164543	0.416424	7.151336	0.526674	1	STK17A	K08804
ENSG00000101888	-0.431897	3.894011	0.526969	1	NXT2	K14285
ENSG00000163794	0.602847	1.095204	0.526983	1	UCN	_
ENSG00000102796	0.550699	1.118865	0.526983	1	DHRS12	K11168
ENSG00000128510	-0.417497	6.35517	0.527032	1	CPA4	K08637
ENSG00000167232	-0.436881	3.530773	0.527198	1	ZNF91	K09228
ENSG00000124574	0.435483	3.516255	0.527198	1	ABCC10	K05674
ENSG00000181409	0.447899	3.22827	0.527401	1	AATK	K17480
ENSG00000253710	0.453391	3.175703	0.527434	1	ALG11	K03844
ENSG00000265298	-0.575213	1.340041	0.527497	1	RP13-104F24.3	K12854
ENSG00000171951	-0.543621	1.320614	0.527497	1	SCG2	_
ENSG00000188747	-0.445984	3.04392	0.527543	1	NOXA1	_
ENSG00000241058	-0.447956	2.939221	0.527622	1	NSUN6	_
ENSG00000111196	-0.418536	5.616642	0.527752	1	MAGOHB	K12877
ENSG00000189164	-0.54391	1.487781	0.527794	1	ZNF527	K09228
ENSG00000073060	0.413899	8.677318	0.527807	1	SCARB1	K13885
ENSG00000267751	-0.483849	2.621494	0.527843	1	AC009005.2	_
ENSG00000151726	0.453132	2.5987	0.527843	1	ACSL1	K01897
ENSG00000004534	-0.418694	5.984731	0.527916	1	RBM6	K13094
ENSG00000169045	-0.413782	8.803985	0.527931	1	HNRNPH1	K12898
ENSG00000245149	-0.47678	2.470964	0.527935	1	RNF139-AS1	_
ENSG00000118276	-0.544139	1.637368	0.527962	1	B4GALT6	K07553
ENSG00000261455	0.486981	1.610015	0.527962	1	LINC01003	_
ENSG00000104324	-0.467901	2.302707	0.52802	1	CPQ	K01302
ENSG00000163877	0.42645	4.613114	0.528057	1	SNIP1	K13108
ENSG00000183444	-0.474519	2.122826	0.528083	1	OR7E38P	K04257
ENSG00000198951	0.427249	4.541345	0.528162	1	NAGA	K01204
ENSG00000033627	0.419023	5.145923	0.528431	1	ATP6V0A1	K02154
ENSG00000023608	-0.432728	4.328238	0.528512	1	SNAPC1	K15208
ENSG00000197442	0.4263	4.231046	0.528668	1	MAP3K5	K04426
ENSG00000105656	0.433151	4.134251	0.528844	1	ELL	K15183
ENSG00000168710	0.413047	8.195547	0.528944	1	AHCYL1	K01251
ENSG00000164896	-0.415218	6.695575	0.528953	1	FASTK	K08290
ENSG00000170291	-0.418908	5.604097	0.529244	1	ELP5	K17617
ENSG00000156875	-0.414251	6.951119	0.529396	1	HIAT1	_
ENSG00000106355	-0.414231	6.451355	0.529427	1	LSM5	K12624
ENSG00000169851	-0.414905	6.563433	0.529569	1	PCDH7	K16498
ENSG00000184992	-0.418351	5.742166	0.529615	1	BRI3BP	K17985

ENSG00000138185	-0.42444	4.553726	0.529676	1	ENTPD1	K01510
ENSG00000170322	0.420295	4.938059	0.52997	1	NFRKB	K11671
ENSG00000133773	-0.417151	5.831741	0.530028	1	CCDC59	_
ENSG00000171992	0.415595	6.253764	0.5304	1	SYNPO	_
ENSG00000137216	0.414703	6.031992	0.530592	1	TMEM63B	_
ENSG00000144840	-0.43389	4.198319	0.530684	1	RABL3	K07933
ENSG00000196678	-0.447638	3.301299	0.530947	1	ERI2	K18417
ENSG00000165752	0.419712	4.983209	0.530975	1	STK32C	K08793
ENSG00000118985	0.411327	7.765706	0.531011	1	ELL2	K15183
ENSG00000143753	0.410923	8.152791	0.531091	1	DEGS1	K04712
ENSG00000124126	0.420861	4.550784	0.531182	1	PREX1	K12365
ENSG00000097007	0.41185	7.142324	0.531232	1	ABL1	K06619
ENSG00000116717	-0.413239	6.139913	0.531285	1	GADD45A	K04402
ENSG00000172590	-0.416748	5.173082	0.531377	1	MRPL52	K17433
ENSG00000166106	-0.437427	3.161107	0.531402	1	ADAMTS15	K08629
ENSG00000127511	0.412966	6.157939	0.531716	1	SIN3B	K17985
ENSG00000100577	-0.435905	3.882225	0.5318	1	GSTZ1	K01800
ENSG00000241973	0.415339	5.458246	0.532029	1	PI4KA	K00888
ENSG00000188343	0.431567	3.810369	0.532039	1	FAM92A1	_
ENSG00000182054	0.413821	5.910879	0.53219	1	IDH2	K00031
ENSG00000115233	-0.411519	6.773276	0.532462	1	PSMD14	K03030
ENSG00000135999	-0.432086	3.698694	0.53258	1	EPC2	K11322
ENSG00000010165	-0.414086	5.958934	0.53258	1	METTL13	K01528
ENSG00000164405	-0.410197	7.471692	0.532623	1	UQCRQ	K00418
ENSG00000077809	0.428895	3.648268	0.532731	1	GTF2I	K03121
ENSG00000114686	-0.410368	7.218692	0.532809	1	MRPL3	K02906
ENSG00000164970	0.453207	2.74315	0.532868	1	FAM219A	_
ENSG00000133657	-0.410847	6.875236	0.532925	1	ATP13A3	K14951
ENSG00000133119	-0.411191	6.381678	0.533057	1	RFC3	K10756
ENSG00000007047	0.419804	4.766282	0.533092	1	MARK4	K08798
ENSG00000149503	0.411026	6.257777	0.533209	1	INCENP	K11515
ENSG00000184979	-0.460769	2.642046	0.533441	1	USP18	K11846
ENSG00000107290	-0.413117	6.116129	0.533454	1	SETX	K10706
ENSG00000179886	-0.415364	4.914638	0.533628	1	TIGD5	_
ENSG00000183397	0.453103	2.547163	0.533764	1	C19orf71	_
ENSG00000180346	-0.431467	3.948922	0.533861	1	TIGD2	_
ENSG00000198792	0.409312	7.007092	0.53397	1	TMEM184B	_
ENSG00000187609	-0.433001	3.39567	0.534015	1	EXD3	_
ENSG00000147687	-0.41791	5.23018	0.534042	1	TATDN1	K03424
ENSG00000138363	-0.409003	7.284474	0.534072	1	ATIC	K00602
ENSG00000177868	-0.451257	2.493699	0.534116	1	CCDC23	_
ENSG00000164087	-0.415537	4.777703	0.534379	1	POC1A	K16482
ENSG00000122507	-0.46028	2.42205	0.5345	1	BBS9	K19398
ENSG00000110395	0.415677	5.285393	0.534635	1	CBL	K04707
ENSG00000223773	0.427668	4.154174	0.534783	1	CD99P1	_
ENSG00000003436	0.408288	7.109274	0.534835	1	TFPI	K03909
ENSG00000188158	0.413156	5.241938	0.534909	1	NHS	_
ENSG00000179832	0.422386	3.723413	0.535071	1	MROH1	_
ENSG00000100216	-0.409344	6.568995	0.535087	1	TOMM22	K17769
ENSG00000186854	0.408207	6.905927	0.535242	1	TRABD2A	_
ENSG00000084092	0.411136	5.585393	0.535265	1	NOA1	K17985
ENSG00000239569	0.486755	2.209948	0.535383	1	KMT2E-AS1	_
ENSG00000128833	-0.41041	5.670068	0.535464	1	MYO5C	K10357
ENSG00000180329	-0.411104	5.662309	0.53552	1	CCDC43	_
ENSG00000006327	-0.408415	6.711559	0.535688	1	TNFRSF12A	K05149
ENSG00000196205	0.405482	10.43094	0.535699	1	EEF1A1P5	K03231
ENSG00000100302	-0.438806	3.636746	0.53571	1	RASD2	K07844
ENSG00000234912	0.428461	3.623552	0.53571	1	LINC00338	_

ENSG00000263142	0.477	2.112614	0.535895	1	LRRC37A17P	K10128
ENSG00000162004	-0.447831	3.070304	0.535896	1	CCDC78	_
ENSG00000197943	-0.447831	3.070304	0.535896	1	PLCG2	K05859
ENSG00000073150	0.434755	3.059916	0.535896	1	PANX2	K03443
ENSG00000165689	-0.408404	6.621958	0.5359	1	SDCCAG3	_
ENSG00000142875	-0.415796	5.350938	0.536047	1	PRKACB	K04345
ENSG00000090266	-0.408788	6.365318	0.536203	1	NDUFB2	K03958
ENSG00000224831	-0.453578	3.022671	0.536238	1	RP11-651P23.4	_
ENSG00000170854	-0.415196	4.683728	0.536337	1	MINA	_
ENSG00000204611	0.423067	4.207492	0.536388	1	ZNF616	K09228
ENSG00000161243	-0.488203	2.029473	0.536462	1	FBXO27	K10102
ENSG00000180385	0.46575	2.008348	0.536462	1	EMC3-AS1	K04613
ENSG00000160049	-0.409379	6.195332	0.536506	1	DFFA	K02310
ENSG00000171843	0.407649	6.192581	0.536506	1	MLLT3	K15187
ENSG00000198799	0.414148	4.186014	0.536557	1	LRIG2	_
ENSG00000086062	0.404983	8.761382	0.536672	1	B4GALT1	K07966
ENSG00000075151	0.409882	5.948356	0.536698	1	EIF4G3	K03260
ENSG00000205670	-0.419464	4.421004	0.536722	1	SMIM11	K12581
ENSG00000269343	0.418165	3.859768	0.536735	1	ZNF587B	K09228
ENSG00000140474	-0.410483	5.104741	0.536873	1	ULK3	K08269
ENSG00000168944	-0.419433	4.381635	0.53703	1	CEP120	K16459
ENSG00000196636	-0.435342	3.809108	0.537187	1	ACN9	_
ENSG00000175895	-0.414117	5.051619	0.537267	1	PLEKHF2	_
ENSG00000101745	0.420349	4.086053	0.537291	1	ANKRD12	_
ENSG00000139263	-0.416066	4.724048	0.537433	1	LRIG3	_
ENSG00000157933	0.410518	5.372819	0.537596	1	SKI	_
ENSG00000205250	-0.409396	5.982673	0.537608	1	E2F4	K04682
ENSG00000148400	0.413384	5.250042	0.537684	1	NOTCH1	K02599
ENSG00000167264	-0.417111	4.852355	0.537695	1	DUS2	K05543
ENSG00000152457	-0.421455	4.047462	0.537697	1	DCLRE1C	K10887
ENSG00000223482	-0.422345	4.247466	0.538069	1	NUTM2A-AS1	_
ENSG00000165699	-0.412912	4.80614	0.538072	1	TSC1	K07206
ENSG00000162704	-0.403772	8.405599	0.53817	1	ARPC5	K05754
ENSG00000148841	0.407715	6.104651	0.538223	1	ITPRIP	_
ENSG00000169682	-0.4435	2.730187	0.538378	1	SPNS1	_
ENSG00000189077	0.437271	2.703811	0.538378	1	TMEM120A	_
ENSG00000174547	-0.405329	6.856115	0.538475	1	MRPL11	K02867
ENSG00000082516	-0.407879	6.014173	0.538481	1	GEMIN5	K13133
ENSG00000107984	0.409776	5.263834	0.538504	1	DKK1	K02165
ENSG00000024526	-0.404863	7.053895	0.538578	1	DEPDC1	_
ENSG00000267374	-0.520057	1.691873	0.538599	1	LINC00669	_
ENSG00000182359	-0.495466	1.676915	0.538599	1	KBTBD3	K06133
ENSG00000260966	-0.495466	1.676915	0.538599	1	RP11-690D19.3	_
ENSG00000179241	-0.416349	4.566673	0.538736	1	LDLRAD3	_
ENSG00000187676	-0.424929	3.61167	0.538801	1	B3GALTL	K13675
ENSG00000171634	0.411084	4.356537	0.538891	1	BPTF	K11728
ENSG00000214530	0.431639	3.557916	0.539116	1	STARD10	_
ENSG00000131966	-0.408022	5.468308	0.539348	1	ACTR10	K16576
ENSG00000091136	-0.403006	7.89634	0.539348	1	LAMB1	K05636
ENSG00000101849	0.404734	6.550861	0.539409	1	TBL1X	K04508
ENSG00000226314	-0.517148	1.548099	0.539497	1	ZNF192P1	K09229
ENSG00000261609	-0.425699	3.818331	0.53966	1	GAN	K10453
ENSG00000142192	0.401663	9.198146	0.539827	1	APP	K04520
ENSG00000174032	-0.415319	4.254309	0.5399	1	SLC25A30	K15106
ENSG00000069275	-0.402239	8.101517	0.539937	1	NUCKS1	_
ENSG00000151376	-0.417276	4.233525	0.540121	1	ME3	K00029
ENSG00000180229	0.43928	3.445422	0.540161	1	HERC2P3	K10595
ENSG00000186532	0.413934	4.824831	0.540409	1	SMYD4	K16896

ENSG00000228110	0.515832	1.325565	0.54051	1	ST13P19	K09560
ENSG00000127311	-0.513508	1.388154	0.54051	1	HELB	K15254
ENSG00000166473	-0.513508	1.388154	0.54051	1	PKD1L2	K04988
ENSG00000120437	-0.403448	6.778101	0.540518	1	ACAT2	K00626
ENSG00000180921	-0.434632	3.41637	0.540548	1	FAM83H	K19603
ENSG00000213903	-0.434632	3.41637	0.540548	1	LTB4R	K04296
ENSG00000184162	-0.41222	4.682009	0.540569	1	NR2C2AP	K08062
ENSG00000225470	-0.41222	4.682009	0.540569	1	JPX	_
ENSG00000125459	-0.423137	3.725313	0.540577	1	MSTO1	_
ENSG00000151116	-0.414799	4.368943	0.54058	1	UEVLD	_
ENSG00000124766	-0.406808	5.829447	0.540827	1	SOX4	K09268
ENSG00000163412	0.443262	2.41416	0.540855	1	EIF4E3	K04301
ENSG00000109971	-0.400364	10.49407	0.540856	1	HSPA8	K03283
ENSG00000179134	0.403757	6.490079	0.540985	1	SAMD4B	_
ENSG00000213281	-0.401738	7.468029	0.541099	1	NRAS	K07828
ENSG00000196793	-0.435455	3.336207	0.541387	1	ZNF239	K09228
ENSG00000120694	-0.400944	7.878604	0.54144	1	HSPH1	K09485
ENSG00000197989	-0.414467	4.850829	0.541469	1	SNHG12	_
ENSG00000120820	-0.408136	5.326048	0.54163	1	GLT8D2	K14966
ENSG00000265194	-0.543445	1.229035	0.54165	1	RP11-70L8.4	_
ENSG00000267278	-0.50882	1.207906	0.54165	1	MAP3K14-AS1	_
ENSG00000099974	-0.43197	3.292076	0.541844	1	DDTL	K10028
ENSG00000008282	-0.401946	6.859525	0.541862	1	SYPL1	K04264
ENSG00000137497	0.400991	6.379374	0.542185	1	NUMA1	K16808
ENSG00000155621	0.410673	4.645148	0.542304	1	C9orf85	K17985
ENSG00000122678	0.422412	3.535528	0.542411	1	POLM	K03513
ENSG00000007520	-0.401219	6.925114	0.542468	1	TSR3	K09140
ENSG00000139579	-0.402822	6.008074	0.54249	1	NABP2	K09654
ENSG00000187535	0.411014	4.847908	0.542674	1	IFT140	K19672
ENSG00000143443	-0.543007	1.026012	0.542911	1	C1orf56	_
ENSG00000179841	-0.543007	1.026012	0.542911	1	AKAP5	K16522
ENSG00000176971	-0.502557	1.001401	0.542911	1	FIBIN	_
ENSG00000176490	0.405226	5.434062	0.542957	1	DIRAS1	K07840
ENSG00000067798	-1.056316	-0.347833	0.542974	1	NAV3	_
ENSG00000106302	-1.056316	-0.347833	0.542974	1	HYAL4	K01197
ENSG00000143257	-1.056316	-0.347833	0.542974	1	NR1I3	K08541
ENSG00000185338	-1.056316	-0.347833	0.542974	1	SOCS1	K04694
ENSG00000185774	-1.056316	-0.347833	0.542974	1	KCNIP4	_
ENSG00000230267	-1.056316	-0.347833	0.542974	1	HERC2P4	K10595
ENSG00000230337	-1.056316	-0.347833	0.542974	1	RP4-635E18.6	_
ENSG00000231466	-1.056316	-0.347833	0.542974	1	CTA-246H3.11	_
ENSG00000235609	-1.056316	-0.347833	0.542974	1	AF127936.7	_
ENSG00000241418	-1.056316	-0.347833	0.542974	1	RP11-512H23.2	_
ENSG00000248238	-1.056316	-0.347833	0.542974	1	RP11-3J1.1	_
ENSG00000256006	-1.056316	-0.347833	0.542974	1	AC084117.3	_
ENSG00000261423	-1.056316	-0.347833	0.542974	1	RP11-1007O24.3	_
ENSG00000269988	-1.056316	-0.347833	0.542974	1	RP11-395P17.11	_
ENSG00000270018	-1.056316	-0.347833	0.542974	1	RP3-462E2.5	_
ENSG00000271127	-1.056316	-0.347833	0.542974	1	LL22NC03-N64E9.1	_
ENSG00000273237	-1.056316	-0.347833	0.542974	1	CTB-119C2.1	_
ENSG00000047457	-0.942373	-0.416729	0.542974	1	CP	K13624
ENSG00000088340	-0.942373	-0.416729	0.542974	1	FER1L4	K11447
ENSG00000145431	-0.942373	-0.416729	0.542974	1	PDGFC	K05450
ENSG00000165029	-0.942373	-0.416729	0.542974	1	ABCA1	K05641
ENSG00000169245	-0.942373	-0.416729	0.542974	1	CXCL10	K12671
ENSG00000170417	-0.942373	-0.416729	0.542974	1	TMEM182	_
ENSG00000172738	-0.942373	-0.416729	0.542974	1	TMEM217	_
ENSG00000184451	-0.942373	-0.416729	0.542974	1	CCR10	K04185

ENSG00000213412	-0.942373	-0.416729	0.542974	1	HNRNPA1P33	K12741
ENSG00000232346	-0.942373	-0.416729	0.542974	1	SC22CB-1E7.1	K02962
ENSG00000232637	-0.942373	-0.416729	0.542974	1	WI2-3658N16.1	_
ENSG00000244578	-0.942373	-0.416729	0.542974	1	RP11-548O1.3	_
ENSG00000269176	-0.942373	-0.416729	0.542974	1	RP11-727F15.12	_
ENSG00000272402	-0.942373	-0.416729	0.542974	1	RP1-30M3.6	_
ENSG00000273443	-0.942373	-0.416729	0.542974	1	RP11-54O7.18	_
ENSG00000254986	-0.405603	5.355215	0.543108	1	DPP3	K01277
ENSG00000092010	-0.399931	7.285919	0.543167	1	PSME1	K06696
ENSG00000176473	0.41487	4.106648	0.543338	1	WDR25	_
ENSG00000130119	-0.428771	3.156012	0.543393	1	GNL3L	K14538
ENSG00000108511	0.424028	3.136987	0.543393	1	HOXB6	K09306
ENSG00000188315	0.424028	3.136987	0.543393	1	C3orf62	K02927
ENSG00000167397	0.401708	6.10272	0.543503	1	VKORC1	K05357
ENSG00000147592	-0.40821	4.889726	0.54351	1	LACTB2	_
ENSG00000119616	-0.406556	5.309583	0.54363	1	FCF1	K14566
ENSG00000166548	-0.414267	4.242782	0.543797	1	TK2	K00857
ENSG00000118508	-0.401859	5.422087	0.54401	1	RAB32	K07918
ENSG00000023287	0.398523	7.500403	0.544129	1	RB1CC1	K17589
ENSG00000198520	0.619606	0.701639	0.544247	1	C1orf228	_
ENSG00000239332	0.619606	0.701639	0.544247	1	LINC01119	_
ENSG00000100207	0.549789	0.733258	0.544247	1	TCF20	K03950
ENSG00000233006	0.478223	2.040013	0.544556	1	AC034220.3	_
ENSG00000100526	0.404668	5.061638	0.544732	1	CDKN3	K14167
ENSG00000159199	-0.401015	6.417485	0.544765	1	ATP5G1	K02128
ENSG00000100836	-0.402424	5.771328	0.544779	1	PABPN1	K14396
ENSG00000187957	0.410687	4.775973	0.544852	1	DNER	_
ENSG00000181873	0.423847	3.791567	0.544928	1	IBA57	K15696
ENSG00000106080	-0.40656	5.041002	0.545076	1	FKBP14	K09577
ENSG00000079841	0.39814	7.056073	0.545105	1	RIMS1	K15291
ENSG00000213398	0.427175	2.585013	0.545115	1	LCAT	K00650
ENSG00000143924	-0.399864	6.414412	0.54524	1	EML4	K15420
ENSG00000135902	0.44675	2.978475	0.545277	1	CHRND	K04816
ENSG00000062038	0.618558	0.410226	0.545468	1	CDH3	_
ENSG00000261474	0.618558	0.410226	0.545468	1	RP11-452L6.1	_
ENSG00000164880	0.396831	7.603622	0.545724	1	INTS1	K13138
ENSG00000260121	0.466427	1.930224	0.545785	1	RP5-1142A6.9	_
ENSG00000113966	-0.465716	1.962272	0.545785	1	ARL6	K07951
ENSG00000258655	-0.465716	1.962272	0.545785	1	ARHGAP5-AS1	_
ENSG00000116285	0.398346	5.883579	0.545785	1	ERRFI1	_
ENSG00000102805	-0.411087	4.366796	0.545863	1	CLN5	K12390
ENSG00000104714	-0.411087	4.366796	0.545863	1	ERICH1	_
ENSG00000139890	0.616819	0.046836	0.545915	1	REM2	K07848
ENSG00000213700	0.616819	0.046836	0.545915	1	RPL17P50	K02880
ENSG00000229692	0.616819	0.046836	0.545915	1	SOS1-IT1	_
ENSG00000259426	0.616819	0.046836	0.545915	1	RP11-253M7.1	_
ENSG00000105875	0.439634	2.921914	0.545998	1	WDR91	K15696
ENSG00000150967	0.439634	2.921914	0.545998	1	ABCB9	K05656
ENSG00000065615	-0.411004	4.207635	0.546252	1	CYB5R4	K00326
ENSG00000141644	0.398341	5.7906	0.546456	1	MBD1	K11589
ENSG00000182585	-0.410648	4.435184	0.546632	1	EPGN	_
ENSG00000171566	-0.398622	5.943395	0.546797	1	PLRG1	K12862
ENSG00000134363	-0.462728	2.462547	0.547051	1	FST	K04661
ENSG00000197362	-0.448538	2.454076	0.547051	1	ZNF786	K09228
ENSG00000213186	-0.480506	1.857455	0.547186	1	TRIM59	K12028
ENSG00000269044	-0.480506	1.857455	0.547186	1	CTC-429P9.3	_
ENSG00000229619	-0.458536	1.844218	0.547186	1	MBNL1-AS1	_
ENSG00000270804	-0.458536	1.844218	0.547186	1	CTD-2583A14.11	K06688

ENSG00000115183	0.398127	5.785033	0.547197	1	TANC1	_
ENSG00000168765	-0.403041	4.886753	0.547218	1	GSTM4	K00799
ENSG00000172500	-0.397041	6.559786	0.547451	1	FIBP	K16758
ENSG00000166228	-0.402398	5.358418	0.547489	1	PCBD1	K01724
ENSG00000146540	-0.398754	6.086835	0.547492	1	C7orf50	_
ENSG00000223959	-0.41862	3.607986	0.547694	1	AFG3L1P	K08956
ENSG00000129116	0.394953	7.342318	0.547926	1	PALLD	_
ENSG00000101191	0.398445	5.930438	0.548139	1	DIDO1	_
ENSG00000261488	0.463227	2.346148	0.548173	1	RP11-757F18.5	_
ENSG00000232973	-0.457684	2.380372	0.548173	1	CYP1B1-AS1	_
ENSG00000160345	0.442811	2.355501	0.548173	1	C9orf116	_
ENSG00000169231	-0.415329	3.571547	0.548235	1	THBS3	K04659
ENSG00000137094	0.418821	3.736698	0.548323	1	DNAJB5	K09511
ENSG00000107951	-0.401348	5.416972	0.548347	1	MTPAP	K18060
ENSG00000072135	-0.413893	3.911373	0.548388	1	PTPN18	K18024
ENSG00000267350	0.410028	4.044179	0.548438	1	RP1-178F10.3	_
ENSG00000132967	-0.395716	6.654087	0.54857	1	HMGB1P5	K10802
ENSG00000137760	-0.438622	3.064864	0.548684	1	ALKBH8	K10770
ENSG00000115484	-0.392907	9.11014	0.548764	1	CCT4	K09496
ENSG00000101331	-0.474127	1.730038	0.548796	1	CCM2L	_
ENSG00000121236	-0.411464	3.718513	0.548828	1	TRIM6	K11999
ENSG00000197081	0.39426	7.017239	0.549088	1	IGF2R	K06564
ENSG00000130402	0.392581	8.929788	0.549111	1	ACTN4	K05699
ENSG00000089060	-0.399274	5.05554	0.549258	1	SLC8B1	K13754
ENSG00000141569	-0.397611	5.898602	0.549283	1	TRIM65	K12031
ENSG00000109674	-0.404807	4.748639	0.549362	1	NEIL3	K10569
ENSG00000086717	0.45292	2.257815	0.549421	1	PPEF1	K13807
ENSG00000048342	-0.451989	2.293181	0.549421	1	CC2D2A	K19352
ENSG00000135945	-0.398414	5.109798	0.54944	1	REV1	K03515
ENSG00000166689	0.421034	3.256649	0.549454	1	PLEKHA7	_
ENSG00000124207	-0.392439	8.593716	0.549494	1	CSE1L	K18423
ENSG00000080986	-0.395251	6.466343	0.549511	1	NDC80	K11547
ENSG00000179431	-0.44103	2.696659	0.549518	1	FJX1	K16674
ENSG00000168140	0.393719	7.06034	0.549569	1	VASN	_
ENSG00000156983	0.402394	4.950952	0.549733	1	BRPF1	K11348
ENSG00000153187	-0.391806	9.359356	0.549788	1	HNRNPU	K12888
ENSG00000105085	0.405946	3.810701	0.549804	1	MED26	K15169
ENSG00000111961	0.415548	3.441809	0.550034	1	SASH1	K02929
ENSG00000115207	0.396211	6.018463	0.550202	1	GTF3C2	K15200
ENSG00000176407	0.393254	6.944547	0.550225	1	KCMF1	_
ENSG00000152669	-0.409881	4.195725	0.550352	1	CCNO	K10861
ENSG00000196642	0.393769	6.548949	0.55046	1	RABL6	_
ENSG00000124782	0.396053	5.658533	0.550602	1	RREB1	K12581
ENSG00000145416	0.506517	1.55065	0.550667	1	1-Mar	K10656
ENSG00000160588	-0.462549	2.210513	0.550817	1	MPZL3	_
ENSG00000230797	-0.462549	2.210513	0.550817	1	YY2	K09201
ENSG00000269958	-0.445509	2.200316	0.550817	1	RP11-73M18.8	_
ENSG00000259605	0.441197	2.163809	0.550817	1	AC074212.5	_
ENSG00000013275	-0.394226	6.33423	0.550868	1	PSMC4	K03063
ENSG00000165650	0.39613	5.555805	0.550882	1	PDZD8	_
ENSG00000069482	-0.399436	4.946177	0.551083	1	GAL	K05244
ENSG00000116649	-0.395259	6.09391	0.551146	1	SRM	K00797
ENSG00000074696	0.391171	7.909596	0.551168	1	PTPLAD1	K10703
ENSG00000006025	-0.435597	2.904177	0.551321	1	OSBPL7	_
ENSG00000113460	-0.390749	8.386571	0.551341	1	BRIX1	K14820
ENSG00000102287	-0.396821	5.438331	0.551383	1	GABRE	K05185
ENSG00000138081	0.398845	4.987426	0.551447	1	FBXO11	K10297
ENSG00000128578	0.398581	4.847952	0.551469	1	STRIP2	_

ENSG00000101367	-0.391734	7.17431	0.551622	1	MAPRE1	K10436
ENSG00000186567	0.444062	2.52481	0.551786	1	CEACAM19	K16825
ENSG00000168517	-0.429833	2.554084	0.551786	1	HEXIM2	K15189
ENSG00000167088	-0.391891	6.948379	0.551846	1	SNRPD1	K11087
ENSG00000186522	0.391042	7.294963	0.551952	1	10-Sep	K16940
ENSG00000079432	0.393204	5.662874	0.551959	1	CIC	K16941
ENSG00000125733	0.390535	7.478605	0.552243	1	TRIP10	K07196
ENSG00000183260	0.431251	2.820275	0.552341	1	ABHD16B	_
ENSG00000162889	-0.430813	2.844225	0.552341	1	MAPKAPK2	K04443
ENSG00000159873	-0.394158	5.676942	0.552477	1	CCDC117	_
ENSG00000175768	-0.39476	5.669219	0.552635	1	TOMM5	K17773
ENSG00000101417	0.534166	1.375544	0.552865	1	PXMP4	K13350
ENSG00000112379	0.534166	1.375544	0.552865	1	KIAA1244	K17572
ENSG00000171303	0.534166	1.375544	0.552865	1	KCNK3	K04914
ENSG00000053372	-0.392837	6.13996	0.55294	1	MRTO4	K14815
ENSG00000117593	-0.396734	5.48502	0.552999	1	DARS2	K01876
ENSG00000020256	-0.401964	4.69216	0.55312	1	ZFP64	_
ENSG00000143933	-0.388375	9.910759	0.553122	1	CALM2	K02183
ENSG00000243449	-0.402325	4.829387	0.553218	1	C4orf48	_
ENSG00000174010	0.393423	5.848165	0.553261	1	KLHL15	K10452
ENSG00000130255	-0.388702	8.526829	0.553333	1	RPL36	K02920
ENSG00000233184	-0.436895	2.78833	0.553447	1	RP11-421L21.3	_
ENSG00000140199	0.406364	4.11942	0.553562	1	SLC12A6	K14427
ENSG00000106460	-0.393408	5.960377	0.553603	1	TMEM106B	_
ENSG00000169239	-0.427145	3.032806	0.55365	1	CA5B	K01672
ENSG00000135617	0.402357	4.321883	0.553651	1	PRADC1	_
ENSG00000197045	-0.39181	5.795188	0.553688	1	GMFB	_
ENSG00000165704	-0.390138	6.870463	0.553771	1	HPRT1	K00760
ENSG00000176973	0.400129	4.841832	0.55412	1	FAM89B	_
ENSG00000196268	0.479583	1.963591	0.554174	1	ZNF493	K09228
ENSG00000265666	0.479583	1.963591	0.554174	1	CTD-2267D19.2	_
ENSG00000105223	0.389639	6.769139	0.554204	1	PLD3	K16860
ENSG00000007038	-0.389161	7.083312	0.554383	1	PRSS21	K09625
ENSG00000101040	0.392874	5.606279	0.554498	1	ZMYND8	_
ENSG00000203999	-0.430887	2.404537	0.554538	1	RP11-290F20.1	_
ENSG00000009830	-0.406677	4.076161	0.554542	1	POMT2	K00728
ENSG00000119965	-0.409879	3.701755	0.554607	1	C10orf88	_
ENSG00000129646	-0.431739	2.72322	0.554649	1	QRICH2	_
ENSG00000261452	-0.419882	2.716215	0.554649	1	RP11-509E16.1	_
ENSG00000111877	-0.415845	3.194562	0.554671	1	MCM9	K10738
ENSG00000105613	0.41344	3.176577	0.554671	1	MAST1	K08789
ENSG00000110514	0.389755	6.150481	0.554757	1	MADD	_
ENSG00000229644	0.393181	5.043129	0.554905	1	NAMPTL	K03462
ENSG00000138688	-0.408366	4.261121	0.554954	1	KIAA1109	_
ENSG00000129680	-0.397015	5.340158	0.554985	1	MAP7D3	K16807
ENSG00000142089	0.386688	8.759552	0.555187	1	IFITM3	K06566
ENSG00000205208	-0.396494	4.917481	0.555255	1	C4orf46	_
ENSG00000152078	-0.395202	5.233647	0.555273	1	TMEM56	_
ENSG00000108515	0.396004	4.415462	0.555276	1	ENO3	K01689
ENSG00000105197	-0.388582	6.873085	0.555348	1	TIMM50	K17496
ENSG00000180011	-0.394409	5.131186	0.555353	1	ZADH2	K07119
ENSG00000114850	-0.386957	8.088311	0.555426	1	SSR3	K13251
ENSG00000246174	0.521847	1.198481	0.55548	1	KCTD21-AS1	_
ENSG00000260482	0.521847	1.198481	0.55548	1	CTD-2196E14.9	_
ENSG00000143315	-0.414418	3.344468	0.555488	1	PIGM	K05284
ENSG00000171806	-0.414418	3.344468	0.555488	1	METTL18	_
ENSG00000165417	-0.393087	5.118636	0.555609	1	GTF2A1	K03122
ENSG00000249992	-0.411301	3.145764	0.555623	1	TMEM158	K01869

ENSG00000133028	-0.388901	6.429761	0.55565	1	SCO1	K07152
ENSG00000101782	0.389537	6.355929	0.55583	1	RIOK3	K08872
ENSG00000114021	-0.388923	5.907395	0.555866	1	NIT2	K13566
ENSG00000115306	0.386776	7.667853	0.555867	1	SPTBN1	K06115
ENSG00000178950	0.388887	6.194336	0.55591	1	GAK	K08855
ENSG00000197565	0.444749	2.627615	0.555962	1	COL4A6	K06237
ENSG00000171443	0.407499	3.620307	0.55598	1	ZNF524	_
ENSG00000136444	-0.393547	5.644053	0.556032	1	RSAD1	_
ENSG00000172731	0.442321	2.294372	0.556146	1	LRRC20	K04240
ENSG00000203995	-0.403735	4.290473	0.556245	1	ZYG11A	K10350
ENSG00000143258	-0.400572	4.531043	0.556307	1	USP21	K11833
ENSG00000162522	0.386665	7.120906	0.556649	1	KIAA1522	_
ENSG00000153015	-0.391746	5.468306	0.55666	1	CWC27	K12737
ENSG00000161618	-0.391068	5.300711	0.556747	1	ALDH16A1	_
ENSG00000144161	-0.399019	4.166662	0.556893	1	ZC3H8	_
ENSG00000266753	0.438774	2.83857	0.556974	1	RP11-690G19.3	_
ENSG00000054523	0.392128	5.485962	0.557004	1	KIF1B	K10392
ENSG00000173757	0.386891	6.76024	0.557018	1	STAT5B	K11224
ENSG00000165118	-0.39621	4.937622	0.557191	1	C9orf64	K09228
ENSG00000167548	0.386576	6.372454	0.55726	1	KMT2D	K09187
ENSG00000188015	0.415274	3.242929	0.557315	1	S100A3	_
ENSG00000135905	-0.41404	3.260051	0.557315	1	DOCK10	_
ENSG00000084070	0.398013	4.132078	0.557419	1	SMAP2	K12486
ENSG00000115053	-0.384086	10.06844	0.557467	1	NCL	K11294
ENSG00000109189	-0.399839	3.818007	0.557595	1	USP46	K11842
ENSG00000144228	-0.398085	4.033919	0.557663	1	SPOPL	K10523
ENSG00000245694	-0.396764	4.221666	0.55771	1	CRNDE	_
ENSG00000185215	0.385531	6.8574	0.558229	1	TNFAIP2	_
ENSG00000268592	0.408362	3.195969	0.558315	1	RP11-244K5.8	_
ENSG00000188419	-0.392758	4.710631	0.558351	1	CHM	_
ENSG00000110987	-0.393273	4.579425	0.558384	1	BCL7A	_
ENSG00000164182	-0.391427	5.386941	0.558442	1	NDUFAF2	K18160
ENSG00000169418	0.384633	7.323466	0.558446	1	NPR1	K12323
ENSG00000239789	-0.394673	4.274651	0.55847	1	MRPS17	K15120
ENSG00000144959	-0.389863	5.219609	0.5585	1	NCEH1	K14349
ENSG00000153896	-0.582354	1.050156	0.558636	1	ZNF599	K09228
ENSG00000171557	-0.582354	1.050156	0.558636	1	FGG	K03905
ENSG00000259485	0.505602	0.997201	0.558636	1	CTD-2147F2.1	_
ENSG00000260534	0.505602	0.997201	0.558636	1	RP11-1006G14.4	_
ENSG00000130347	-0.39393	4.695508	0.558729	1	RTN4IP1	_
ENSG00000083857	-0.389255	5.548135	0.558911	1	FAT1	K16506
ENSG00000049656	-0.382866	9.187244	0.558971	1	CLPTM1L	_
ENSG00000101098	0.443677	2.47054	0.558985	1	RIMS4	K15299
ENSG00000143970	-0.392127	4.678501	0.559116	1	ASXL2	K11471
ENSG00000072364	0.385022	6.626115	0.559189	1	AFF4	K15185
ENSG00000047849	0.383022	7.92482	0.559467	1	MAP4	K10431
ENSG00000114541	0.398322	3.598871	0.559478	1	FRMD4B	_
ENSG00000234975	0.390681	4.829559	0.55951	1	FTH1P2	K00522
ENSG00000160818	-0.384842	6.361666	0.559801	1	GPATCH4	_
ENSG00000138182	-0.384814	6.559363	0.559859	1	KIF20B	K10402
ENSG00000236552	0.38171	9.587257	0.559982	1	RPL13AP5	K02872
ENSG00000068305	0.386134	5.618847	0.560247	1	MEF2A	K09260
ENSG00000126561	0.387387	5.37918	0.560308	1	STAT5A	K11223
ENSG00000165637	0.382413	7.640128	0.560355	1	VDAC2	K15040
ENSG00000134369	0.392135	5.090318	0.560395	1	NAV1	K16776
ENSG00000070010	0.384752	6.165849	0.560609	1	UFD1L	K14016
ENSG00000166260	-0.390448	5.361609	0.560816	1	COX11	K02258
ENSG00000142185	-0.400401	4.330177	0.560857	1	TRPM2	K04977

ENSG00000185344	-0.393939	4.772821	0.560996	1	ATP6V0A2	K02154
ENSG00000169599	-0.420145	3.40788	0.56102	1	NFU1	_
ENSG00000115806	0.383251	6.610907	0.56103	1	GORASP2	_
ENSG00000175054	-0.386681	5.524552	0.561039	1	ATR	K06640
ENSG00000145020	-0.492763	1.60604	0.561338	1	AMT	K00605
ENSG00000165959	-0.492763	1.60604	0.561338	1	CLMN	K15601
ENSG00000217416	-0.492763	1.60604	0.561338	1	ISCA1P1	K13628
ENSG00000271359	-0.492763	1.60604	0.561338	1	RP11-84C13.1	_
ENSG00000196693	-0.411039	3.653847	0.561347	1	ZNF33B	K09228
ENSG00000166833	0.388578	4.900408	0.561539	1	NAV2	K19483
ENSG00000037637	0.394837	4.501369	0.561591	1	FBXO42	K10317
ENSG00000158747	0.394837	4.501369	0.561591	1	NBL1	K19558
ENSG00000224531	-0.382973	6.603258	0.561636	1	SMIM13	_
ENSG00000137955	-0.380711	8.215149	0.561714	1	RABGGTB	K05956
ENSG00000070785	-0.384255	5.558392	0.561796	1	EIF2B3	K03241
ENSG00000148019	-0.385729	5.31837	0.561878	1	CEP78	K16765
ENSG00000175183	-0.391626	4.894097	0.561902	1	CSRP2	K09377
ENSG00000230844	0.413136	3.354438	0.562029	1	ZNF674-AS1	_
ENSG00000113838	-0.415057	3.499761	0.562128	1	TBCCD1	K16810
ENSG00000112167	-0.468882	2.017823	0.562277	1	SAYSD1	_
ENSG00000185875	-0.401427	3.931466	0.562372	1	THNSL1	K05691
ENSG00000205268	-0.385962	5.183915	0.562395	1	PDE7A	K18436
ENSG00000072832	-0.589441	0.817781	0.562502	1	CRMP1	K07528
ENSG00000130751	-0.589441	0.817781	0.562502	1	NPAS1	K09098
ENSG00000270558	-0.589441	0.817781	0.562502	1	CTD-2124B8.2	K11586
ENSG00000172766	-0.39201	4.595808	0.562593	1	NAA16	K00670
ENSG00000048544	-0.382469	6.259661	0.562805	1	MRPS10	K02946
ENSG00000104953	-0.43285	2.591083	0.562885	1	TLE6	_
ENSG00000116337	0.381511	6.532391	0.562978	1	AMPD2	K01490
ENSG00000115041	0.423533	2.801752	0.562991	1	KCNIP3	_
ENSG00000188811	-0.398472	3.902434	0.563103	1	NHLRC3	K17985
ENSG00000186448	-0.403771	3.805579	0.563107	1	ZNF197	K09229
ENSG00000132688	0.385157	5.220924	0.56331	1	NES	K07609
ENSG00000139832	0.380984	6.477241	0.563322	1	RAB20	K07911
ENSG00000120675	-0.381581	5.926967	0.563337	1	DNAJC15	K09535
ENSG00000118922	-0.389119	4.676776	0.563489	1	KLF12	K09205
ENSG00000152601	-0.380752	6.79129	0.563503	1	MBNL1	K14943
ENSG00000130956	-0.388041	4.559107	0.563534	1	HABP4	K19019
ENSG00000120318	-0.392104	4.139521	0.563723	1	ARAP3	K12490
ENSG00000148218	0.389041	4.910666	0.563775	1	ALAD	K01698
ENSG00000157470	-0.388979	5.080318	0.564015	1	FAM81A	_
ENSG00000169379	-0.388111	4.412595	0.564122	1	ARL13B	K07962
ENSG00000178761	-0.385417	5.265854	0.564174	1	FAM219B	_
ENSG00000135486	-0.377546	9.15692	0.564445	1	HNRNPA1	K12741
ENSG00000090013	0.385458	5.513151	0.564464	1	BLVRB	K05901
ENSG00000101608	-0.378026	8.090771	0.56457	1	MYL12A	K12757
ENSG00000169668	-0.434377	2.756145	0.56459	1	BCRP2	K08878
ENSG00000052795	0.429779	2.730697	0.56459	1	FNIP2	K12581
ENSG00000197744	0.429779	2.730697	0.56459	1	PTMAP2	K13784
ENSG00000147804	-0.515436	1.470373	0.564607	1	SLC39A4	K14710
ENSG00000070756	0.376963	10.94161	0.564628	1	PABPC1	K13126
ENSG00000171236	0.383874	5.5035	0.564721	1	LRG1	_
ENSG00000149054	-0.440304	2.524203	0.564733	1	ZNF215	K09229
ENSG00000160255	0.434733	2.502116	0.564733	1	ITGB2	K06464
ENSG00000182310	0.434733	2.502116	0.564733	1	SPACA6P	_
ENSG00000121039	-0.382413	5.721608	0.564897	1	RDH10	K11151
ENSG00000216895	-0.48329	1.91713	0.564915	1	AC009403.2	_
ENSG00000230562	0.452629	1.896097	0.564915	1	FAM133DP	_

ENSG00000115504	-0.385628	5.126164	0.564947	1	EHBP1	_
ENSG00000104131	-0.377643	7.796243	0.565232	1	EIF3J	K03245
ENSG00000089289	0.378604	6.850917	0.565333	1	IGBP1	K17606
ENSG00000109220	-0.403621	3.232121	0.565439	1	CHIC2	_
ENSG00000175105	0.403421	3.2151	0.565439	1	ZNF654	_
ENSG00000090061	0.3811	5.351475	0.565473	1	CCNK	_
ENSG00000184709	-0.413124	3.074727	0.565683	1	LRRC26	_
ENSG00000139880	0.392957	4.341584	0.56582	1	CDH24	K06814
ENSG00000130193	-0.380735	5.78888	0.565828	1	THEM6	_
ENSG00000177981	-0.391037	4.58672	0.565854	1	ASB8	K10330
ENSG00000122435	-0.388117	4.791891	0.565877	1	TRMT13	K15446
ENSG00000138172	-0.425193	2.898039	0.565977	1	CALHM2	K19739
ENSG00000163513	-0.385361	4.924673	0.56599	1	TGFBR2	K04388
ENSG00000157191	-0.384159	5.36798	0.565994	1	NECAP2	_
ENSG00000122884	0.377438	7.167145	0.566031	1	P4HA1	K00472
ENSG00000235750	-0.389569	4.628028	0.566228	1	KIAA0040	_
ENSG00000139116	-0.380864	5.595086	0.566246	1	KIF21A	K10395
ENSG00000196743	-0.381844	4.956036	0.566284	1	GM2A	K12383
ENSG00000100558	-0.428975	2.689518	0.566334	1	PLEK2	_
ENSG00000135622	-0.428975	2.689518	0.566334	1	SEMA4F	K06521
ENSG00000124444	-0.406772	3.340003	0.566389	1	ZNF576	_
ENSG00000099917	0.37791	6.666872	0.56639	1	MED15	K15157
ENSG00000148396	0.377068	7.0487	0.566586	1	SEC16A	_
ENSG00000135124	-0.396192	3.677797	0.566595	1	P2RX4	K05218
ENSG00000153823	0.42394	2.423052	0.566775	1	PID1	_
ENSG00000171208	-0.384676	4.897017	0.566807	1	NETO2	_
ENSG00000153774	-0.381934	5.300703	0.566998	1	CFDP1	_
ENSG00000203760	-0.38923	4.422847	0.567246	1	CENPW	_
ENSG00000237457	-0.656184	0.574269	0.567307	1	RP11-547I7.2	_
ENSG00000239282	-0.656184	0.574269	0.567307	1	GATSL3	_
ENSG00000273314	-0.656184	0.574269	0.567307	1	RP5-1136G13.2	_
ENSG00000055955	0.646703	0.50156	0.567307	1	ITIH4	_
ENSG00000124743	0.646703	0.50156	0.567307	1	KLHL31	K10467
ENSG00000183323	0.646703	0.50156	0.567307	1	CCDC125	_
ENSG00000198691	0.646703	0.50156	0.567307	1	ABCA4	K05644
ENSG00000214575	0.646703	0.50156	0.567307	1	CPEB1	K02602
ENSG00000222019	0.646703	0.50156	0.567307	1	URAHP	K07127
ENSG00000231249	0.646703	0.50156	0.567307	1	ITPR1-AS1	_
ENSG00000259248	0.646703	0.50156	0.567307	1	USP3-AS1	_
ENSG00000267422	0.646703	0.50156	0.567307	1	CTD-2554C21.1	K19476
ENSG00000106541	-0.599971	0.539768	0.567307	1	AGR2	_
ENSG00000147174	-0.599971	0.539768	0.567307	1	ACRC	_
ENSG00000180264	-0.599971	0.539768	0.567307	1	GPR144	K08466
ENSG00000197406	-0.599971	0.539768	0.567307	1	DIO3	K07754
ENSG00000228560	-0.599971	0.539768	0.567307	1	RP11-550P17.5	_
ENSG00000237781	-0.599971	0.539768	0.567307	1	RP11-54A4.2	_
ENSG00000248690	-0.599971	0.539768	0.567307	1	HAS2-AS1	_
ENSG00000124374	0.565026	0.538185	0.567307	1	PAIP2B	K12581
ENSG00000135423	0.565026	0.538185	0.567307	1	GLS2	K01425
ENSG00000221883	0.565026	0.538185	0.567307	1	ARIH2OS	K17985
ENSG00000253978	0.565026	0.538185	0.567307	1	CTB-178M22.2	_
ENSG00000266835	0.565026	0.538185	0.567307	1	RP11-838N2.4	_
ENSG00000268061	0.565026	0.538185	0.567307	1	NAPA-AS1	_
ENSG00000269374	0.565026	0.538185	0.567307	1	CTB-50E14.5	_
ENSG00000120549	0.452821	2.123728	0.56732	1	KIAA1217	_
ENSG00000157734	-0.441923	2.151513	0.56732	1	SNX22	K17941
ENSG00000261005	0.42904	2.134745	0.56732	1	CTB-58E17.1	_
ENSG00000181523	0.381139	4.955267	0.567359	1	SGSH	K01565

ENSG00000174365	-0.419938	2.837812	0.567621	1	SNHG11	_
ENSG00000108439	-0.391899	4.283652	0.567676	1	PNPO	K00275
ENSG00000119705	-0.380761	5.919755	0.567809	1	SLIRP	K06063
ENSG00000116918	-0.380346	5.59055	0.567893	1	TSNAX	_
ENSG00000066654	-0.384326	5.095755	0.567952	1	THUMPD1	K06963
ENSG00000144134	-0.477464	1.795171	0.567967	1	RABL2A	K07931
ENSG00000166578	0.468046	1.760133	0.567967	1	IQCD	_
ENSG00000183978	-0.38127	5.476195	0.568048	1	COA3	K18175
ENSG00000085415	-0.37964	5.660601	0.568106	1	SEH1L	K14299
ENSG00000173674	-0.374644	7.99193	0.568137	1	EIF1AX	K03236
ENSG00000079134	-0.37864	5.65192	0.568362	1	THOC1	K12878
ENSG00000149809	-0.511321	1.300887	0.568539	1	TM7SF2	K00222
ENSG00000169758	-0.511321	1.300887	0.568539	1	C15orf27	_
ENSG00000250133	-0.511321	1.300887	0.568539	1	HOXC-AS2	_
ENSG00000165804	0.381275	4.990239	0.568664	1	ZNF219	_
ENSG00000154127	-0.378972	5.139877	0.568758	1	UBASH3B	K18993
ENSG00000185324	-0.380262	5.424121	0.568764	1	CDK10	K02449
ENSG00000109180	-0.375874	6.661096	0.568787	1	OCIAD1	_
ENSG00000176390	-0.386598	4.870326	0.568799	1	CRLF3	K17985
ENSG00000186918	0.379127	5.498649	0.568957	1	ZNF395	_
ENSG00000232112	-0.377249	6.074591	0.568984	1	TMA7	K07604
ENSG00000128699	-0.38089	5.414914	0.56907	1	ORMDL1	_
ENSG00000166562	-0.382554	4.982593	0.569075	1	SEC11C	K13280
ENSG00000170855	-0.378975	5.73893	0.569311	1	TRIAP1	K17968
ENSG00000176986	0.37472	6.844417	0.56934	1	SEC24C	K14007
ENSG00000103257	0.372257	12.34702	0.569393	1	SLC7A5	K13780
ENSG00000226278	-0.414205	2.774939	0.569406	1	PSPHP1	K01079
ENSG00000175581	-0.377042	5.756856	0.569438	1	MRPL48	K17429
ENSG00000055609	0.378749	5.305563	0.569529	1	KMT2C	K09188
ENSG00000128915	-0.378605	5.942005	0.569572	1	NARG2	K09228
ENSG00000164209	-0.378605	5.942005	0.569572	1	SLC25A46	K03454
ENSG00000165646	0.392108	3.333231	0.569634	1	SLC18A2	K08155
ENSG00000035141	-0.375545	6.471348	0.569819	1	FAM136A	_
ENSG00000103067	-0.378205	5.597801	0.569964	1	ESRP2	K14947
ENSG00000128609	-0.376967	5.886844	0.570019	1	NDUFA5	K03949
ENSG00000101346	-0.37551	6.393288	0.570067	1	POFUT1	_
ENSG00000179021	-0.387714	4.136994	0.570089	1	C3orf38	_
ENSG00000233237	0.439833	2.020348	0.570106	1	LINC00472	_
ENSG00000177628	-0.404892	3.650273	0.570207	1	GBA	K01201
ENSG00000181027	-0.398718	3.646689	0.570207	1	FKRP	_
ENSG00000148248	0.395702	3.638226	0.570207	1	SURF4	_
ENSG00000018699	-0.376072	5.373266	0.570338	1	TTC27	_
ENSG00000173068	-0.416433	2.546157	0.570342	1	BNC2	_
ENSG00000107816	0.373812	6.709878	0.570524	1	LZTS2	_
ENSG00000149231	-0.37775	5.528353	0.570557	1	CCDC82	_
ENSG00000160685	0.373402	6.914552	0.570578	1	ZBTB7B	K10494
ENSG00000096746	-0.371992	8.262299	0.57066	1	HNRNPH3	K12898
ENSG00000189067	0.3726	7.443611	0.570663	1	LITAF	K19363
ENSG00000173156	0.37581	5.298886	0.57071	1	RHOD	K07530
ENSG00000171150	0.375535	5.777818	0.570777	1	SOCS5	K04698
ENSG00000128641	-0.374521	6.325578	0.570837	1	MYO1B	K10356
ENSG00000233429	-0.384923	4.111856	0.570861	1	HOTAIRM1	_
ENSG00000100345	0.371089	9.334465	0.570999	1	MYH9	K10352
ENSG00000083457	-0.3858	4.566506	0.571115	1	ITGAE	K06524
ENSG00000096872	-0.39268	3.799158	0.571118	1	IFT74	K19679
ENSG00000151445	-0.401359	3.614896	0.571291	1	VIPAS39	_
ENSG00000133056	0.372863	6.818955	0.5713	1	PIK3C2B	K00923
ENSG00000105245	0.379314	5.044493	0.571511	1	NUMBL	K06057

ENSG00000113387	-0.370848	8.758571	0.571539	1	SUB1	K15040
ENSG00000100056	0.379753	4.640026	0.571568	1	DGCR14	K13118
ENSG00000143882	-0.419628	2.273916	0.571582	1	ATP6V1C2	K02148
ENSG00000171960	-0.382428	4.590315	0.57192	1	PPIH	K09567
ENSG00000171791	0.370902	7.787535	0.572063	1	BCL2	K02161
ENSG00000120690	-0.383018	4.882241	0.572189	1	ELF1	K09428
ENSG00000136982	-0.376098	5.837166	0.572251	1	DSCC1	K11271
ENSG00000164920	-0.387199	4.372981	0.572276	1	OSR2	K09215
ENSG00000196663	0.404561	3.247779	0.572349	1	TECPR2	K13182
ENSG00000177105	0.375098	6.049265	0.572496	1	RHOG	K07863
ENSG00000160401	0.433972	2.446985	0.572659	1	C9orf117	K01056
ENSG00000122490	0.376867	5.294414	0.572747	1	PQLC1	_
ENSG00000153721	-0.408857	3.361522	0.572833	1	CNKSR3	K17536
ENSG00000164086	-0.38445	4.782361	0.572852	1	DUSP7	K04459
ENSG00000139719	-0.381647	4.780758	0.572852	1	VPS33A	_
ENSG00000235919	-0.445301	1.950021	0.573297	1	ASH1L-AS1	_
ENSG00000152611	-0.690626	0.238055	0.573302	1	CAPSL	_
ENSG00000152926	-0.690626	0.238055	0.573302	1	ZNF117	K09228
ENSG00000174586	-0.690626	0.238055	0.573302	1	ZNF497	_
ENSG00000237864	-0.690626	0.238055	0.573302	1	LINC00322	_
ENSG00000258315	-0.690626	0.238055	0.573302	1	C17orf49	_
ENSG00000266680	-0.690626	0.238055	0.573302	1	RP5-1148A21.3	_
ENSG00000271133	-0.690626	0.238055	0.573302	1	CTA-293F17.1	_
ENSG00000070729	0.653578	0.16233	0.573302	1	CNGB1	K04952
ENSG00000198569	0.653578	0.16233	0.573302	1	SLC34A3	K14683
ENSG00000205746	0.653578	0.16233	0.573302	1	RP11-1212A22.1	K04985
ENSG00000213999	0.653578	0.16233	0.573302	1	MEF2B	K09261
ENSG00000241741	0.653578	0.16233	0.573302	1	RPL7AP30	K02936
ENSG00000243279	0.653578	0.16233	0.573302	1	PRAF2	_
ENSG00000252447	0.653578	0.16233	0.573302	1	snoU13	_
ENSG00000256391	0.653578	0.16233	0.573302	1	SDIM1	_
ENSG00000273382	0.653578	0.16233	0.573302	1	RP5-1065J22.8	_
ENSG00000109133	-0.371624	6.594716	0.573317	1	TMEM33	_
ENSG00000121858	-0.543243	1.131148	0.573359	1	TNFSF10	K04721
ENSG00000230850	-0.543243	1.131148	0.573359	1	RP11-782C8.1	_
ENSG00000269928	-0.543243	1.131148	0.573359	1	RP11-849F2.8	_
ENSG00000120051	0.529156	1.059255	0.573359	1	CCDC147	_
ENSG00000234857	0.529156	1.059255	0.573359	1	HNRNPUL2-BSCL2	K19365
ENSG00000242861	0.529156	1.059255	0.573359	1	RP11-285F7.2	_
ENSG00000198948	-0.505931	1.108407	0.573359	1	MFAP3L	_
ENSG00000227799	-0.505931	1.108407	0.573359	1	AC012358.4	_
ENSG00000259065	-0.505931	1.108407	0.573359	1	RP5-1021I20.1	_
ENSG00000120093	0.477008	1.083502	0.573359	1	HOXB3	K09303
ENSG00000244625	0.477008	1.083502	0.573359	1	CTA-211A9.5	_
ENSG00000038532	0.3769	5.24546	0.573377	1	CLEC16A	K19513
ENSG00000060762	-0.4136	2.64764	0.573479	1	MPC1	_
ENSG00000072864	0.381169	4.707138	0.573769	1	NDE1	K10352
ENSG00000142686	0.381169	4.707138	0.573769	1	C1orf216	_
ENSG00000159788	0.39256	2.770872	0.574122	1	RGS12	K16449
ENSG00000130762	0.373971	5.486943	0.574297	1	ARHGEF16	K13744
ENSG00000105810	-0.370505	6.645894	0.574358	1	CDK6	K02091
ENSG00000136699	0.370163	6.242663	0.574434	1	SMPD4	K12353
ENSG00000172660	0.385695	4.055037	0.574451	1	TAF15	K14651
ENSG00000013588	0.367515	9.842906	0.574571	1	GPRC5A	K08468
ENSG00000144034	-0.37756	5.091896	0.57459	1	TPRKB	K15901
ENSG00000159023	0.376051	4.952447	0.574641	1	EPB41	K06107
ENSG00000072134	0.373435	5.497677	0.5747	1	EPN2	K12471
ENSG00000213563	-0.376946	4.807569	0.574704	1	C8orf82	_

ENSG00000145388	-0.3772	4.406339	0.574728	1	METTL14		K05925
ENSG00000104375	-0.371306	6.22089	0.574786	1	STK3		K04412
ENSG00000066926	-0.37603	4.843088	0.574816	1	FECH		K01772
ENSG00000233270	-0.384508	4.181092	0.574829	1	SNRPEP4		K11097
ENSG00000035862	0.367721	8.478529	0.574857	1	TIMP2		_
ENSG00000152409	0.373719	5.562322	0.574904	1	JMY		_
ENSG00000099783	-0.367549	8.62469	0.575031	1	HNRNPM		K12887
ENSG00000138623	-0.393104	3.593151	0.575153	1	SEMA7A		K06529
ENSG00000189079	-0.376066	5.281689	0.575184	1	ARID2		K11765
ENSG00000116954	0.37138	5.802424	0.575298	1	RRAGC		K16186
ENSG00000006695	-0.380194	4.622658	0.575383	1	COX10		K02257
ENSG00000162601	-0.371912	5.61934	0.575397	1	MYSM1		K11865
ENSG00000179818	0.400975	3.147443	0.575425	1	PCBP1-AS1		_
ENSG00000164885	-0.378575	4.927533	0.575616	1	CDK5		K02090
ENSG00000100650	-0.367801	7.498048	0.57573	1	SRSF5		K12893
ENSG00000225791	-0.489755	1.531448	0.575777	1	TRAM2-AS1		_
ENSG00000244363	0.471449	1.460711	0.575777	1	RPL7P23		K02937
ENSG00000204775	0.408511	2.541154	0.575816	1	KM-PA-2		K14824
ENSG00000159216	0.372055	5.389704	0.575886	1	RUNX1		K08367
ENSG00000105639	0.403232	3.358911	0.575988	1	JAK3		K11218
ENSG00000105538	-0.367529	7.324544	0.576239	1	RASIP1		_
ENSG00000164073	-0.387144	4.014965	0.576266	1	MFSD8		K12307
ENSG00000119801	0.369452	6.230427	0.576347	1	YPEL5		_
ENSG00000167740	-0.382489	3.552481	0.576412	1	CYB5D2		_
ENSG00000184640	0.366675	7.747392	0.576483	1		9-Sep	K16938
ENSG00000155903	-0.373986	4.668119	0.576785	1	RASA2		K08053
ENSG00000166750	-0.368704	6.039096	0.576903	1	SLFN5		K12581
ENSG00000177873	-0.436227	1.830843	0.576985	1	ZNF619		K09228
ENSG00000185267	-0.436227	1.830843	0.576985	1	CDNF		_
ENSG00000164306	-0.388455	3.854666	0.577008	1	PRIMPOL		_
ENSG00000119041	-0.374232	5.386933	0.577038	1	GTF3C3		K15201
ENSG00000126773	-0.380753	4.437012	0.577078	1	PCNXL4		K02927
ENSG00000100605	0.37031	5.632186	0.577084	1	ITPK1		K00913
ENSG00000100726	-0.367925	6.168905	0.577118	1	TELO2		K11137
ENSG00000163545	-0.399928	3.115478	0.57712	1	NUAK2		K08800
ENSG00000213430	-0.399928	3.115478	0.57712	1	HSPD1P1		K04077
ENSG00000183092	0.369759	5.453249	0.57715	1	BEGAIN		_
ENSG00000174775	-0.376034	4.885184	0.577154	1	HRAS		K02833
ENSG00000119707	-0.366735	7.224171	0.577207	1	RBM25		K12822
ENSG00000170264	-0.395314	3.227278	0.577313	1	FAM161A		K16772
ENSG00000139926	-0.372841	4.610124	0.577344	1	FRMD6		K16822
ENSG00000136146	-0.369089	5.778562	0.577602	1	MED4		K15146
ENSG00000083844	0.427746	2.063358	0.577675	1	ZNF264		K09228
ENSG00000203667	-0.419496	2.089919	0.577675	1	COX20		K18184
ENSG00000111906	-0.369599	6.064176	0.57768	1	HDDC2		K07023
ENSG00000185896	0.364766	8.959137	0.577697	1	LAMP1		K06528
ENSG00000116497	-0.372845	5.316163	0.57774	1	S100PBP		_
ENSG00000183495	0.369534	5.978559	0.577837	1	EP400		K11320
ENSG00000167904	-0.376049	5.060746	0.577903	1	TMEM68		_
ENSG00000187790	-0.385055	3.82402	0.578095	1	FANCM		K10896
ENSG00000076641	0.409959	2.277696	0.578107	1	PAG1		K06115
ENSG00000088256	0.367442	6.149191	0.578243	1	GNA11		K04635
ENSG00000141503	0.365633	7.024897	0.578445	1	MINK1		K04413
ENSG00000148426	-0.368978	5.756054	0.57848	1	PROSER2		_
ENSG00000140950	-0.375981	4.395967	0.578553	1	TLDC1		K12581
ENSG00000169554	-0.426072	2.655003	0.578596	1	ZEB2		K09299
ENSG00000233223	0.411647	2.642775	0.578596	1	AC113189.5		_
ENSG00000115677	0.363832	9.111525	0.578611	1	HDLBP		_

ENSG00000225484	-0.417135	2.806721	0.578742	1	RP11-773D16.1	_
ENSG00000166526	0.370807	5.057762	0.578883	1	ZNF3	K09228
ENSG00000179119	0.370807	5.057762	0.578883	1	SPTY2D1	K15193
ENSG00000223414	0.365203	6.965675	0.578985	1	LINC00473	_
ENSG00000122484	-0.37071	5.428191	0.579003	1	RPAP2	_
ENSG00000065665	0.386508	3.785707	0.579228	1	SEC61A2	K10956
ENSG00000145332	-0.380784	3.99855	0.57927	1	KLHL8	K10446
ENSG00000247626	-0.379965	4.276796	0.579312	1	MARS2	K01874
ENSG00000237493	0.376549	4.317681	0.579318	1	RP11-603J24.7	K06109
ENSG00000130935	-0.365326	6.828035	0.579345	1	NOL11	_
ENSG00000182093	0.371058	4.631235	0.579347	1	WRB	_
ENSG00000226416	-0.542729	0.912462	0.579401	1	MRPL23-AS1	_
ENSG00000167524	0.512162	0.836355	0.579401	1	SGK494	K17529
ENSG00000267395	0.512162	0.836355	0.579401	1	AC074212.6	_
ENSG00000130024	-0.365218	6.688984	0.579741	1	PHF10	_
ENSG00000112308	-0.363394	7.908991	0.579873	1	C6orf62	_
ENSG00000197345	-0.375158	4.877754	0.579917	1	MRPL21	K02888
ENSG00000065802	0.372459	4.65583	0.579993	1	ASB1	K10323
ENSG00000141580	0.363709	7.277667	0.580088	1	WDR45B	_
ENSG00000176101	-0.3657	5.761718	0.580295	1	SSNA1	K16780
ENSG00000011243	0.366256	6.093021	0.580352	1	AKAP8L	K15978
ENSG00000106246	-0.381343	3.836294	0.580373	1	PTCD1	K17710
ENSG00000138036	-0.383525	3.763998	0.580413	1	DYNC2LI1	K10417
ENSG00000154493	-0.756487	-0.20347	0.580436	1	C10orf90	K16773
ENSG00000242282	-0.756487	-0.20347	0.580436	1	AC108488.4	_
ENSG00000245648	-0.756487	-0.20347	0.580436	1	RP11-277P12.20	_
ENSG00000255145	-0.756487	-0.20347	0.580436	1	RP11-60I3.5	_
ENSG00000260669	-0.756487	-0.20347	0.580436	1	AL136419.6	_
ENSG00000264235	-0.756487	-0.20347	0.580436	1	RP13-270P17.1	_
ENSG00000267390	-0.756487	-0.20347	0.580436	1	RP11-635N19.1	_
ENSG00000273190	-0.756487	-0.20347	0.580436	1	RP11-255C15.4	_
ENSG00000273352	-0.756487	-0.20347	0.580436	1	RP11-61L19.3	_
ENSG00000010327	0.666366	-0.278753	0.580436	1	STAB1	K19020
ENSG00000066405	0.666366	-0.278753	0.580436	1	CLDN18	K06087
ENSG00000116176	0.666366	-0.278753	0.580436	1	TPSG1	K09615
ENSG00000148204	0.666366	-0.278753	0.580436	1	CRB2	K16681
ENSG00000185065	0.666366	-0.278753	0.580436	1	AC000068.5	_
ENSG00000197846	0.666366	-0.278753	0.580436	1	HIST1H2BF	K11252
ENSG00000204525	0.666366	-0.278753	0.580436	1	HLA-C	K06751
ENSG00000212998	0.666366	-0.278753	0.580436	1	RBM12B-AS1	_
ENSG00000229092	0.666366	-0.278753	0.580436	1	IGHV3-47	K06856
ENSG00000229867	0.666366	-0.278753	0.580436	1	STEAP3-AS1	_
ENSG00000254554	0.666366	-0.278753	0.580436	1	RP11-351I24.1	_
ENSG00000259705	0.666366	-0.278753	0.580436	1	RP11-227D13.1	_
ENSG00000270562	0.666366	-0.278753	0.580436	1	RP11-154H23.3	_
ENSG00000270959	0.666366	-0.278753	0.580436	1	LPP-AS2	_
ENSG00000272630	0.666366	-0.278753	0.580436	1	RP11-344N10.5	_
ENSG00000189227	-0.38595	3.687886	0.580456	1	C15orf61	_
ENSG00000164307	-0.369335	5.389022	0.580554	1	ERAP1	K09604
ENSG00000156304	0.369255	5.383405	0.580554	1	SCAF4	K13167
ENSG00000091542	0.365374	6.347252	0.580602	1	ALKBH5	K10767
ENSG00000012983	-0.367196	5.697357	0.580651	1	MAP4K5	K08833
ENSG00000081721	-0.368291	5.185092	0.580724	1	DUSP12	K14819
ENSG00000134461	-0.389541	3.245765	0.580735	1	ANKRD16	_
ENSG00000169372	-0.389541	3.245765	0.580735	1	CRADD	K02832
ENSG00000189298	-0.389541	3.245765	0.580735	1	ZKSCAN3	K09229
ENSG00000175395	-0.482593	1.369386	0.580886	1	ZNF25	K09228
ENSG00000135953	-0.451001	1.350337	0.580886	1	MFSD9	_

ENSG00000154240	-0.451001	1.350337	0.580886	1	CEP112	K16767
ENSG00000183426	0.395297	3.001845	0.580886	1	NPIPA1	K04985
ENSG00000215424	0.395297	3.001845	0.580886	1	MCM3AP-AS1	_
ENSG00000170296	0.364823	6.187662	0.580967	1	GABARAP	K08341
ENSG00000166669	-0.404157	2.885675	0.580972	1	ATF7IP2	_
ENSG00000061656	0.390562	2.725257	0.581063	1	SPAG4	_
ENSG00000095139	0.390562	2.725257	0.581063	1	ARCN1	K09228
ENSG00000255508	0.390562	2.725257	0.581063	1	MIR3654	K18709
ENSG00000141084	0.382424	4.112051	0.581157	1	RANBP10	_
ENSG00000273154	0.469024	1.667	0.581299	1	RP4-583P15.15	_
ENSG00000146859	-0.425356	1.700761	0.581299	1	TMEM140	_
ENSG00000154736	-0.425356	1.700761	0.581299	1	ADAMTS5	K08620
ENSG00000170802	-0.373785	4.542211	0.581437	1	FOXN2	K09407
ENSG00000140830	-0.385632	3.734707	0.581651	1	TXNL4B	_
ENSG00000183049	0.371678	5.099357	0.581749	1	CAMK1D	K08794
ENSG00000060656	0.364938	5.225654	0.581992	1	PTPRU	K16662
ENSG00000185305	-0.387467	3.48372	0.582017	1	ARL15	K17201
ENSG00000176531	0.375218	4.142276	0.582036	1	PHLDB3	_
ENSG00000135750	0.362145	7.019136	0.582084	1	KCNK1	K04912
ENSG00000112561	0.373275	4.558529	0.582094	1	TFEB	K15590
ENSG00000116161	-0.36139	7.746025	0.58212	1	CACYBP	K04507
ENSG00000179632	-0.361512	7.532303	0.582229	1	MAF1	_
ENSG00000139746	-0.362277	6.906418	0.582367	1	RBM26	K13192
ENSG00000116586	-0.366845	5.382737	0.582519	1	LAMTOR2	_
ENSG00000171163	-0.367206	5.531197	0.582572	1	ZNF692	_
ENSG00000213339	-0.364872	5.586006	0.582798	1	QTRT1	K00773
ENSG00000149089	-0.365043	5.79837	0.582923	1	APIP	K08964
ENSG00000196705	-0.392548	2.960551	0.582985	1	ZNF431	K04613
ENSG00000116898	-0.361562	6.960858	0.583015	1	MRPS15	K02956
ENSG00000002919	-0.373646	4.461026	0.583034	1	SNX11	K17924
ENSG00000139496	-0.363909	6.070065	0.583058	1	NUPL1	K14307
ENSG00000092621	-0.359482	9.375726	0.583108	1	PHGDH	K00058
ENSG00000122644	-0.363452	5.82642	0.583146	1	ARL4A	K07945
ENSG00000182400	-0.374773	4.41456	0.583148	1	TRAPPC6B	_
ENSG00000185404	-0.39794	2.824892	0.583268	1	SP140L	_
ENSG00000211450	-0.364838	5.512136	0.583332	1	C11orf31	_
ENSG00000198931	-0.361522	6.807871	0.583341	1	APRT	K00759
ENSG00000100422	0.364749	5.466226	0.583453	1	CERK	K04715
ENSG00000129173	-0.404556	2.67512	0.583598	1	E2F8	K09391
ENSG00000183087	-0.404556	2.67512	0.583598	1	GAS6	K05464
ENSG00000155330	-0.368947	4.673253	0.583825	1	C16orf87	_
ENSG00000167333	-0.42665	2.516102	0.583986	1	TRIM68	K12033
ENSG00000172667	0.366426	4.956724	0.584094	1	ZMAT3	K10137
ENSG00000197321	0.366014	5.220949	0.584191	1	SVIL	K10369
ENSG00000039123	-0.360939	6.68262	0.584207	1	SKIV2L2	K12598
ENSG00000117868	0.359576	7.362581	0.584291	1	ESYT2	_
ENSG00000176890	-0.359192	7.810451	0.58435	1	TYMS	K00560
ENSG00000263072	0.394552	3.132658	0.584434	1	RP11-473M20.14	_
ENSG00000253309	-0.439356	2.328153	0.584446	1	SERPINE3	_
ENSG00000177764	-0.423618	2.318798	0.584446	1	ZCCHC3	_
ENSG00000010818	0.372178	4.2385	0.584461	1	HIVEP2	_
ENSG00000147459	0.361697	5.956785	0.584668	1	DOCK5	K17707
ENSG00000161682	0.367133	4.941079	0.584671	1	FAM171A2	_
ENSG00000096696	0.357834	9.247217	0.58483	1	DSP	K10381
ENSG00000158050	0.363249	5.981331	0.584839	1	DUSP2	K04459
ENSG00000156853	-0.382732	3.499226	0.584863	1	ZNF689	K09228
ENSG00000143067	0.380032	3.486184	0.584863	1	ZNF697	K09228
ENSG00000147421	0.380032	3.486184	0.584863	1	HMBOX1	_

ENSG00000160200	-0.358621	7.752957	0.585006	1	CBS	K01697
ENSG00000102384	-0.370812	4.32341	0.585012	1	CENPI	K11501
ENSG00000149357	-0.364165	5.429205	0.585089	1	LAMTOR1	_
ENSG00000163312	-0.385634	3.407266	0.585194	1	HELQ	K19178
ENSG00000068097	-0.371758	4.601125	0.585405	1	HEATR6	_
ENSG00000164221	-0.368564	4.599303	0.585405	1	CCDC112	_
ENSG00000232024	0.382053	3.693763	0.585409	1	LSM12P1	_
ENSG00000126005	0.374325	3.697263	0.585409	1	MMP24-AS1	_
ENSG00000214046	-0.363857	5.242888	0.585432	1	SMIM7	_
ENSG00000050165	-1.099898	-0.754197	0.585507	1	DKK3	_
ENSG00000113319	-1.099898	-0.754197	0.585507	1	RASGRF2	K12326
ENSG00000123965	-1.099898	-0.754197	0.585507	1	PMS2P5	K10858
ENSG00000136327	-1.099898	-0.754197	0.585507	1	NKX2-8	K09347
ENSG00000158423	-1.099898	-0.754197	0.585507	1	RIBC1	_
ENSG00000183054	-1.099898	-0.754197	0.585507	1	RGPD6	K12172
ENSG00000185467	-1.099898	-0.754197	0.585507	1	KPNA7	_
ENSG00000214534	-1.099898	-0.754197	0.585507	1	ZNF705E	K09228
ENSG00000221819	-1.099898	-0.754197	0.585507	1	C16orf3	_
ENSG00000223799	-1.099898	-0.754197	0.585507	1	IL10RB-AS1	_
ENSG00000230397	-1.099898	-0.754197	0.585507	1	SPTLC1P1	K00654
ENSG00000231993	-1.099898	-0.754197	0.585507	1	RP1-85F18.5	_
ENSG00000243970	-1.099898	-0.754197	0.585507	1	PPIEL	K09564
ENSG00000249407	-1.099898	-0.754197	0.585507	1	IL20RB-AS1	_
ENSG00000260938	-1.099898	-0.754197	0.585507	1	RP4-593C16.3	_
ENSG00000265917	-1.099898	-0.754197	0.585507	1	MIR3685	_
ENSG00000269290	-1.099898	-0.754197	0.585507	1	RP11-869B15.1	_
ENSG00000271780	-1.099898	-0.754197	0.585507	1	RP11-1017G21.5	_
ENSG00000272305	-1.099898	-0.754197	0.585507	1	RP11-894J14.5	K16467
ENSG00000272486	-1.099898	-0.754197	0.585507	1	RP11-532M24.1	_
ENSG00000272644	-1.099898	-0.754197	0.585507	1	RP11-33O4.1	_
ENSG00000273156	-1.099898	-0.754197	0.585507	1	RP11-127B20.2	_
ENSG00000104870	1.008684	-1.018953	0.585507	1	FCGRT	_
ENSG00000111275	1.008684	-1.018953	0.585507	1	ALDH2	K00128
ENSG00000112655	1.008684	-1.018953	0.585507	1	PTK7	K05127
ENSG00000131969	1.008684	-1.018953	0.585507	1	ABHD12B	K13705
ENSG00000134962	1.008684	-1.018953	0.585507	1	KLB	K17985
ENSG00000140057	1.008684	-1.018953	0.585507	1	AK7	K00939
ENSG00000154040	1.008684	-1.018953	0.585507	1	CABYR	_
ENSG00000172346	1.008684	-1.018953	0.585507	1	CSDC2	_
ENSG00000174990	1.008684	-1.018953	0.585507	1	CA5A	K01672
ENSG00000181585	1.008684	-1.018953	0.585507	1	TMIE	_
ENSG00000182584	1.008684	-1.018953	0.585507	1	ACTL10	_
ENSG00000184361	1.008684	-1.018953	0.585507	1	SPATA32	K18047
ENSG00000187699	1.008684	-1.018953	0.585507	1	C2orf88	_
ENSG00000204434	1.008684	-1.018953	0.585507	1	POTEKP	K17299
ENSG00000205090	1.008684	-1.018953	0.585507	1	TMEM240	_
ENSG00000215840	1.008684	-1.018953	0.585507	1	RP11-122G18.7	K02981
ENSG00000221625	1.008684	-1.018953	0.585507	1	AC013283.1	_
ENSG00000225096	1.008684	-1.018953	0.585507	1	XXbac-BPG55C20.7	_
ENSG00000229715	1.008684	-1.018953	0.585507	1	EEF1DP3	K15410
ENSG00000232677	1.008684	-1.018953	0.585507	1	LINC00665	_
ENSG00000233081	1.008684	-1.018953	0.585507	1	RP11-440G5.2	_
ENSG00000233588	1.008684	-1.018953	0.585507	1	CYP51A1P2	K05917
ENSG00000235027	1.008684	-1.018953	0.585507	1	AC068580.6	_
ENSG00000235257	1.008684	-1.018953	0.585507	1	AC093415.2	_
ENSG00000236519	1.008684	-1.018953	0.585507	1	AL773604.8	_
ENSG00000246323	1.008684	-1.018953	0.585507	1	RP11-325L7.1	_
ENSG00000251532	1.008684	-1.018953	0.585507	1	CTD-2245E15.3	_

ENSG00000253404	1.008684	-1.018953	0.585507	1	AC034243.1	_
ENSG00000258081	1.008684	-1.018953	0.585507	1	RP11-384J4.2	_
ENSG00000260082	1.008684	-1.018953	0.585507	1	RP11-2C24.5	_
ENSG00000260787	1.008684	-1.018953	0.585507	1	RP11-797A18.4	_
ENSG00000261366	1.008684	-1.018953	0.585507	1	MANEA-AS1	_
ENSG00000261485	1.008684	-1.018953	0.585507	1	PAN3-AS1	_
ENSG00000262482	1.008684	-1.018953	0.585507	1	LA16c-321D4.2	_
ENSG00000263264	1.008684	-1.018953	0.585507	1	CTB-133G6.1	_
ENSG00000267543	1.008684	-1.018953	0.585507	1	RP11-666A8.7	_
ENSG00000270184	1.008684	-1.018953	0.585507	1	RP11-568J23.5	_
ENSG00000101204	-0.933277	-0.848055	0.585507	1	CHRNA4	_
ENSG00000106560	-0.933277	-0.848055	0.585507	1	GIMAP2	K04613
ENSG00000116254	-0.933277	-0.848055	0.585507	1	CHD5	K14435
ENSG00000116652	-0.933277	-0.848055	0.585507	1	DLEU2L	_
ENSG00000120729	-0.933277	-0.848055	0.585507	1	MYOT	_
ENSG00000132622	-0.933277	-0.848055	0.585507	1	HSPA12B	_
ENSG00000133808	-0.933277	-0.848055	0.585507	1	MICALCL	_
ENSG00000149201	-0.933277	-0.848055	0.585507	1	CCDC81	K08857
ENSG00000159224	-0.933277	-0.848055	0.585507	1	GIP	K05258
ENSG00000172771	-0.933277	-0.848055	0.585507	1	EFCAB12	_
ENSG00000186665	-0.933277	-0.848055	0.585507	1	C17orf58	_
ENSG00000186842	-0.933277	-0.848055	0.585507	1	LINC00846	_
ENSG00000197283	-0.933277	-0.848055	0.585507	1	SYNGAP1	K17631
ENSG00000198400	-0.933277	-0.848055	0.585507	1	NTRK1	K03176
ENSG00000203588	-0.933277	-0.848055	0.585507	1	IGBP1-AS1	_
ENSG00000204377	-0.933277	-0.848055	0.585507	1	C1orf134	_
ENSG00000207047	-0.933277	-0.848055	0.585507	1	SNORD51	_
ENSG00000214391	-0.933277	-0.848055	0.585507	1	RP11-121L10.3	K07374
ENSG00000223701	-0.933277	-0.848055	0.585507	1	RAET1E-AS1	_
ENSG00000224066	-0.933277	-0.848055	0.585507	1	RP4-622L5.7	_
ENSG00000224418	-0.933277	-0.848055	0.585507	1	STK24-AS1	_
ENSG00000224997	-0.933277	-0.848055	0.585507	1	AL049840.1	_
ENSG00000228140	-0.933277	-0.848055	0.585507	1	RP3-467K16.4	_
ENSG00000229647	-0.933277	-0.848055	0.585507	1	AC007879.7	_
ENSG00000233025	-0.933277	-0.848055	0.585507	1	CRYZP1	K00344
ENSG00000236581	-0.933277	-0.848055	0.585507	1	STARD13-AS	_
ENSG00000237611	-0.933277	-0.848055	0.585507	1	AL353898.3	K11447
ENSG00000239103	-0.933277	-0.848055	0.585507	1	snoU13	_
ENSG00000246560	-0.933277	-0.848055	0.585507	1	RP11-10L12.4	_
ENSG00000252915	-0.933277	-0.848055	0.585507	1	Y_RNA	_
ENSG00000257660	-0.933277	-0.848055	0.585507	1	RP11-579D7.4	_
ENSG00000258608	-0.933277	-0.848055	0.585507	1	DNAJC19P9	K09539
ENSG00000259529	-0.933277	-0.848055	0.585507	1	RP11-468E2.4	K11974
ENSG00000260081	-0.933277	-0.848055	0.585507	1	RP11-66N11.8	_
ENSG00000261286	-0.933277	-0.848055	0.585507	1	RP11-517C16.2	_
ENSG00000263276	-0.933277	-0.848055	0.585507	1	RP11-96D1.10	_
ENSG00000264350	-0.933277	-0.848055	0.585507	1	SNRPGP2	K11099
ENSG00000265806	-0.933277	-0.848055	0.585507	1	MIR4292	_
ENSG00000266969	-0.933277	-0.848055	0.585507	1	RP11-773H22.4	_
ENSG00000005001	0.698443	-0.91035	0.585507	1	PRSS22	K09626
ENSG00000088854	0.698443	-0.91035	0.585507	1	C20orf194	K10687
ENSG00000102144	0.698443	-0.91035	0.585507	1	PGK1	K00927
ENSG00000124664	0.698443	-0.91035	0.585507	1	SPDEF	K09442
ENSG00000138131	0.698443	-0.91035	0.585507	1	LOXL4	K00280
ENSG00000143001	0.698443	-0.91035	0.585507	1	TMEM61	_
ENSG00000155918	0.698443	-0.91035	0.585507	1	RAET1L	K07987
ENSG00000161298	0.698443	-0.91035	0.585507	1	ZNF382	K09228
ENSG00000165837	0.698443	-0.91035	0.585507	1	FAM194B	_

ENSG00000168124	0.698443	-0.91035	0.585507	1	OR1F1	K04257
ENSG00000170075	0.698443	-0.91035	0.585507	1	GPR37L1	K04244
ENSG00000201457	0.698443	-0.91035	0.585507	1	SNORA55	_
ENSG00000207757	0.698443	-0.91035	0.585507	1	MIR93	_
ENSG00000213590	0.698443	-0.91035	0.585507	1	RP11-229P13.2	K16467
ENSG00000224520	0.698443	-0.91035	0.585507	1	KRT8P45	K07605
ENSG00000228436	0.698443	-0.91035	0.585507	1	RP5-864K19.4	_
ENSG00000231346	0.698443	-0.91035	0.585507	1	LINC01160	_
ENSG00000231563	0.698443	-0.91035	0.585507	1	RP11-245P10.4	_
ENSG00000232295	0.698443	-0.91035	0.585507	1	RP11-154D6.1	_
ENSG00000233452	0.698443	-0.91035	0.585507	1	STXBP5-AS1	_
ENSG00000235111	0.698443	-0.91035	0.585507	1	RP1-29C18.8	_
ENSG00000235285	0.698443	-0.91035	0.585507	1	SMIM2-IT1	_
ENSG00000236184	0.698443	-0.91035	0.585507	1	TCEA1P4	K03145
ENSG00000237813	0.698443	-0.91035	0.585507	1	AC002066.1	_
ENSG00000238528	0.698443	-0.91035	0.585507	1	snoU13	_
ENSG00000243960	0.698443	-0.91035	0.585507	1	RP11-552M11.4	_
ENSG00000250474	0.698443	-0.91035	0.585507	1	WBP1LP2	_
ENSG00000252196	0.698443	-0.91035	0.585507	1	AL590482.1	_
ENSG00000254862	0.698443	-0.91035	0.585507	1	RP11-159H22.2	_
ENSG00000255139	0.698443	-0.91035	0.585507	1	AP000442.1	_
ENSG00000256293	0.698443	-0.91035	0.585507	1	RP11-114F3.2	K02150
ENSG00000257949	0.698443	-0.91035	0.585507	1	TEN1	_
ENSG00000258136	0.698443	-0.91035	0.585507	1	RP11-864J10.4	_
ENSG00000260978	0.698443	-0.91035	0.585507	1	RP11-73C9.1	_
ENSG00000261337	0.698443	-0.91035	0.585507	1	RP11-498D10.5	_
ENSG00000263597	0.698443	-0.91035	0.585507	1	MIR3936	_
ENSG00000268584	0.698443	-0.91035	0.585507	1	RP11-464F9.20	_
ENSG00000269781	0.698443	-0.91035	0.585507	1	FLJ20306	K12581
ENSG00000271857	0.698443	-0.91035	0.585507	1	RP1-244F24.1	_
ENSG00000273188	0.698443	-0.91035	0.585507	1	RP3-402G11.25	_
ENSG00000214765	-0.375914	3.956793	0.585546	1	SEPT7P2	K16944
ENSG00000130517	-0.388954	3.309039	0.585567	1	PGPEP1	K01304
ENSG00000108666	-0.369431	4.557145	0.585577	1	C17orf75	_
ENSG00000175294	0.422905	1.825426	0.585652	1	CATSPER1	K16889
ENSG00000185670	0.422905	1.825426	0.585652	1	ZBTB3	K10490
ENSG00000174456	0.378306	3.620756	0.585727	1	C12orf76	_
ENSG00000147684	-0.357804	7.869876	0.585745	1	NDUFB9	K03965
ENSG00000071655	-0.359789	6.529901	0.585752	1	MBD3	K11591
ENSG00000154059	-0.372045	4.303596	0.585923	1	IMPACT	_
ENSG00000173933	0.367825	4.464215	0.585953	1	RBM4	K13187
ENSG00000143751	0.360276	5.485963	0.585954	1	SDE2	K17985
ENSG00000165338	-0.368696	4.624377	0.585976	1	HECTD2	K12232
ENSG00000013523	-0.375831	3.548635	0.58608	1	ANGEL1	K18729
ENSG00000103187	-0.357642	7.604964	0.586188	1	COTL1	_
ENSG00000213465	-0.363631	5.502509	0.586261	1	ARL2	K07943
ENSG00000196235	0.357948	7.128962	0.586302	1	SUPT5H	K15172
ENSG00000109111	0.358099	6.975103	0.586377	1	SUPT6H	K11292
ENSG00000066230	0.452237	1.523758	0.586413	1	SLC9A3	K12040
ENSG00000249592	0.452237	1.523758	0.586413	1	RP11-440L14.1	_
ENSG00000107864	0.386325	3.081944	0.586478	1	CPEB3	K02602
ENSG00000096717	-0.362231	5.588739	0.586541	1	SIRT1	K11411
ENSG00000175213	0.36657	4.322156	0.586613	1	ZNF408	_
ENSG00000138101	0.368364	3.663104	0.586817	1	DTNB	_
ENSG00000162929	-0.367288	4.564555	0.58694	1	KIAA1841	_
ENSG00000145907	-0.356725	7.778915	0.586963	1	G3BP1	K17265
ENSG00000198911	0.356828	7.45234	0.58706	1	SREBF2	K09107
ENSG00000173715	0.371244	4.22213	0.587131	1	C11orf80	_

ENSG00000072518	0.36846	4.513116	0.587162	1	MARK2	K08798
ENSG00000118690	0.50037	1.142083	0.587167	1	ARMC2	_
ENSG00000242599	0.50037	1.142083	0.587167	1	CSAG4	_
ENSG00000234129	-0.473344	1.18642	0.587167	1	RP11-120D5.1	_
ENSG00000145217	-0.541987	0.653808	0.587191	1	SLC26A1	K14700
ENSG00000150281	-0.541987	0.653808	0.587191	1	CTF1	K05422
ENSG00000180096	-0.541987	0.653808	0.587191	1	1-Sep	K13737
ENSG00000198835	-0.541987	0.653808	0.587191	1	GJC2	K07619
ENSG00000250151	-0.541987	0.653808	0.587191	1	ARPC4-TTLL3	K05755
ENSG00000258659	-0.541987	0.653808	0.587191	1	TRIM34	K11999
ENSG00000261408	-0.541987	0.653808	0.587191	1	TEN1-CDK3	_
ENSG00000204370	-0.359611	5.806223	0.587311	1	SDHD	K00237
ENSG00000168297	-0.381504	3.792706	0.587438	1	PXK	K17543
ENSG00000123136	0.356683	7.17953	0.587555	1	DDX39A	K13182
ENSG00000164904	-0.357335	6.886593	0.587563	1	ALDH7A1	K14085
ENSG00000143228	-0.36009	5.887609	0.587715	1	NUF2	K11548
ENSG00000167085	-0.355674	8.012184	0.587848	1	PHB	K17080
ENSG00000167978	0.355184	8.612218	0.587871	1	SRRM2	K13172
ENSG00000116120	-0.35925	6.216805	0.588015	1	FARSB	K01890
ENSG00000148384	-0.379036	4.060222	0.588066	1	INPP5E	K01099
ENSG00000176124	-0.3652	4.71051	0.588097	1	DLEU1	_
ENSG00000185883	0.360702	5.22331	0.588133	1	ATP6V0C	K02155
ENSG00000067836	0.367089	4.198442	0.588141	1	ROGDI	_
ENSG00000105393	-0.359621	6.02775	0.588207	1	BABAM1	_
ENSG00000112742	-0.357368	6.535112	0.588268	1	TTK	K08866
ENSG00000136932	-0.386289	3.639492	0.588293	1	C9orf156	_
ENSG00000135480	-0.354342	10.07927	0.58831	1	KRT7	K07605
ENSG00000196083	-0.358269	6.07593	0.588416	1	IL1RAP	K04723
ENSG00000106780	0.366657	4.725581	0.58859	1	MEGF9	K17985
ENSG00000166831	-0.383585	3.32652	0.588751	1	RBPMS2	_
ENSG00000205323	0.380582	3.307095	0.588751	1	SARNP	K18732
ENSG00000074964	0.372358	3.922464	0.588826	1	ARHGEF10L	K16727
ENSG00000225921	-0.355148	7.530914	0.588886	1	NOL7	_
ENSG00000105520	0.357087	6.021095	0.588997	1	DKFZP761J1410	K19581
ENSG00000174132	-0.363482	4.843044	0.589052	1	FAM174A	_
ENSG00000256393	0.42631	1.988286	0.589173	1	AC138123.2	K02928
ENSG00000119335	-0.353453	9.541392	0.589376	1	SET	K11290
ENSG00000174109	-0.363451	5.028882	0.589414	1	C16orf91	_
ENSG00000115556	0.398739	2.518731	0.589438	1	PLCD4	K05857
ENSG00000183814	-0.361005	4.862735	0.589489	1	LIN9	_
ENSG00000126522	-0.370021	4.126837	0.589564	1	ASL	K01755
ENSG00000170473	-0.357995	5.590559	0.58961	1	WIBG	K14294
ENSG00000160917	-0.35919	5.191084	0.589699	1	CPSF4	K14404
ENSG00000183340	-0.359895	5.570428	0.589758	1	JRKL	_
ENSG00000077150	-0.353744	7.743615	0.590126	1	NFKB2	_
ENSG00000075539	0.362745	4.841966	0.590165	1	FRYL	_
ENSG00000178105	-0.360416	5.412853	0.590172	1	DDX10	K14776
ENSG00000139239	0.357637	5.993783	0.590212	1	RPL14P1	K02875
ENSG00000267121	0.366797	3.995918	0.590415	1	CTD-2020K17.1	_
ENSG00000167767	0.352919	8.057061	0.590597	1	KRT80	K07605
ENSG00000009780	-0.411961	2.353186	0.590607	1	FAM76A	K08834
ENSG00000186591	0.353478	7.368146	0.590672	1	UBE2H	K10576
ENSG00000177889	-0.354983	6.522792	0.590792	1	UBE2N	K10580
ENSG00000090447	-0.361013	5.094428	0.59089	1	TFAP4	K09108
ENSG00000204922	-0.361013	5.094428	0.59089	1	C11orf83	K15696
ENSG00000138835	-0.39554	2.988523	0.591024	1	RGS3	K07524
ENSG00000135655	0.354155	6.33856	0.591178	1	USP15	K11835
ENSG00000174564	-0.356079	5.748757	0.591321	1	IL20RB	K05137

ENSG00000182919	0.357352	5.150983	0.591345	1	C11orf54	_
ENSG00000185621	-0.388326	2.632789	0.591473	1	LMLN	K13539
ENSG00000066735	0.353922	6.288862	0.591593	1	KIF26A	K10404
ENSG00000102054	0.35128	9.126146	0.591754	1	RBBP7	K11659
ENSG00000144354	-0.354845	6.197855	0.591957	1	CDCA7	_
ENSG00000149499	-0.357526	5.467312	0.592013	1	EML3	K18596
ENSG00000133874	-0.424038	2.140857	0.592079	1	RNF122	K15699
ENSG00000188643	-0.351519	8.063027	0.59217	1	S100A16	_
ENSG00000136810	-0.350952	8.863553	0.592263	1	TXN	K03671
ENSG00000162783	-0.352697	6.919537	0.592362	1	IER5	_
ENSG00000053770	-0.359265	4.707134	0.592513	1	AP5M1	K19023
ENSG00000247708	0.472978	1.345477	0.592573	1	STX18-AS1	_
ENSG00000186806	0.431361	1.365073	0.592573	1	VSIG10L	_
ENSG00000134717	-0.354593	6.006123	0.59271	1	BTF3L4	K01527
ENSG00000184208	-0.372285	3.845354	0.592804	1	C22orf46	_
ENSG00000158195	0.352041	6.823923	0.59298	1	WASF2	K05748
ENSG00000219200	-0.366404	4.428808	0.592998	1	RNASEK	K19770
ENSG00000147044	-0.362794	4.42675	0.592998	1	CASK	K06103
ENSG00000148362	-0.354274	5.755259	0.593101	1	C9orf142	_
ENSG00000165487	-0.355167	5.424113	0.59316	1	MICU2	_
ENSG00000118518	-0.371542	4.139333	0.593184	1	RNF146	_
ENSG00000156381	0.354854	5.456256	0.5932	1	ANKRD9	_
ENSG00000029363	-0.352103	6.789819	0.593232	1	BCLAF1	K13087
ENSG00000135446	-0.351099	7.446351	0.593243	1	CDK4	_
ENSG00000101216	0.357298	5.128557	0.593398	1	GMEB2	_
ENSG00000174669	-0.353757	5.658019	0.593402	1	SLC29A2	K15014
ENSG00000123843	-0.350007	8.492701	0.593458	1	C4BPB	K04003
ENSG00000215158	-0.389434	2.932021	0.593564	1	RP11-1023L17.1	K01195
ENSG00000237190	-0.353377	5.728257	0.593668	1	CDKN2AIPNL	K12581
ENSG00000133816	-0.360653	4.652469	0.593679	1	MICAL2	_
ENSG00000186908	-0.357384	4.713816	0.593726	1	ZDHHC17	K18932
ENSG00000139517	0.365301	4.073617	0.593736	1	LNX2	K10692
ENSG00000160392	0.365301	4.073617	0.593736	1	C19orf47	_
ENSG00000130150	-0.355961	4.880666	0.593845	1	MOSPD2	_
ENSG00000088280	0.361607	4.324387	0.593916	1	ASAP3	K12488
ENSG00000130669	0.351786	6.001334	0.594027	1	PAK4	K05734
ENSG00000163002	-0.357874	4.674917	0.594109	1	NUP35	K14313
ENSG00000062582	0.354125	5.829691	0.594118	1	MRPS24	K17403
ENSG00000121690	0.356962	5.400151	0.594136	1	DEPDC7	_
ENSG00000140455	0.352166	5.961005	0.594176	1	USP3	K11986
ENSG00000127666	0.353751	5.506401	0.594182	1	TICAM1	K05842
ENSG00000239672	-0.35015	7.490363	0.594187	1	NME1	K00940
ENSG00000141526	0.348906	9.208852	0.594228	1	SLC16A3	K08180
ENSG00000154229	0.35349	5.698809	0.594258	1	PRKCA	K02677
ENSG00000100564	-0.363325	4.202478	0.594397	1	PIGH	K03858
ENSG00000088836	0.419815	2.240736	0.594519	1	SLC4A11	K13862
ENSG00000023318	0.349661	7.400208	0.594648	1	ERP44	K17264
ENSG00000162819	-0.352035	5.844837	0.594861	1	BROX	_
ENSG00000157240	-0.405923	2.800129	0.594874	1	FZD1	K02432
ENSG00000165879	0.377789	2.77771	0.594874	1	FRAT1	K03069
ENSG00000173402	0.349022	7.771225	0.594937	1	DAG1	K06265
ENSG00000100578	-0.386037	3.348247	0.594983	1	KIAA0586	_
ENSG00000174943	-0.375818	3.49523	0.59499	1	KCTD13	K15074
ENSG00000149925	0.348011	9.832605	0.595002	1	ALDOA	K01623
ENSG00000007516	0.538229	0.905334	0.595081	1	BAIAP3	K15621
ENSG00000142408	0.538229	0.905334	0.595081	1	CACNG8	K04873
ENSG00000271971	0.538229	0.905334	0.595081	1	CTD-2006H14.2	_
ENSG00000257261	0.479736	0.932498	0.595081	1	RP11-96H19.1	_

ENSG00000105402	0.351704	6.129211	0.59515	1	NAPA	K15296
ENSG00000184857	-0.357513	4.796508	0.595307	1	TMEM186	_
ENSG00000259781	-0.350793	6.473399	0.595307	1	RP11-673C5.1	K10802
ENSG00000140694	-0.353593	5.360521	0.595404	1	PARN	K01148
ENSG00000164751	-0.358784	5.157126	0.595494	1	PEX2	_
ENSG00000130309	-0.34831	8.113243	0.595511	1	COLGALT1	K11703
ENSG00000103512	0.349949	6.625546	0.595553	1	NOMO1	_
ENSG00000105821	-0.350058	6.615926	0.595746	1	DNAJC2	K09522
ENSG00000156030	0.355745	5.169411	0.59578	1	ELMSAN1	K12581
ENSG00000169592	-0.356161	5.49379	0.59582	1	INO80E	K11669
ENSG00000170445	-0.349935	6.2886	0.596063	1	HARS	K01892
ENSG00000119862	-0.361093	4.12173	0.596181	1	LGALSL	_
ENSG00000019485	-0.393523	2.87945	0.596309	1	PRDM11	_
ENSG00000132825	-0.393523	2.87945	0.596309	1	PPP1R3D	K07189
ENSG00000178082	0.382284	2.86443	0.596309	1	TWF1P1	K08870
ENSG00000133812	0.352996	5.527505	0.596483	1	SBF2	K18061
ENSG00000050555	0.350752	5.794506	0.59658	1	LAMC3	K06247
ENSG00000264538	0.366637	3.700754	0.596589	1	SUZ12P	K17985
ENSG00000134815	-0.357628	4.874714	0.596723	1	DHX34	_
ENSG00000213918	0.357796	3.854458	0.597147	1	DNASE1	K11994
ENSG00000148411	0.351798	5.541719	0.597374	1	NACC2	K10486
ENSG00000012211	0.684418	0.269641	0.597593	1	PRICKLE3	K04511
ENSG00000138744	0.684418	0.269641	0.597593	1	NAAA	K13720
ENSG00000186326	0.684418	0.269641	0.597593	1	RGS9BP	_
ENSG00000204209	0.684418	0.269641	0.597593	1	DAXX	K02308
ENSG00000230071	0.684418	0.269641	0.597593	1	RPL4P6	K02930
ENSG00000231503	0.684418	0.269641	0.597593	1	PTMAP4	K13784
ENSG00000243926	0.684418	0.269641	0.597593	1	TIPARP-AS1	_
ENSG00000260806	0.684418	0.269641	0.597593	1	RP11-872J21.3	_
ENSG00000261643	0.684418	0.269641	0.597593	1	RP11-529E10.6	_
ENSG00000269951	0.684418	0.269641	0.597593	1	RP11-797A18.6	_
ENSG00000008323	-0.60742	0.377228	0.597593	1	PLEKHG6	K10408
ENSG00000126953	-0.60742	0.377228	0.597593	1	TIMM8A	K17780
ENSG00000232640	-0.60742	0.377228	0.597593	1	RP1-266L20.2	_
ENSG00000236921	-0.60742	0.377228	0.597593	1	RP11-408N14.1	_
ENSG00000245848	-0.60742	0.377228	0.597593	1	CEBPA	K09055
ENSG00000266501	-0.60742	0.377228	0.597593	1	RP11-766H1.1	K08336
ENSG00000186831	-0.540826	0.33714	0.597593	1	KRT17P2	K07604
ENSG00000246922	-0.540826	0.33714	0.597593	1	UBAP1L	K09488
ENSG00000165572	0.349666	5.365414	0.597708	1	KBTBD6	K10474
ENSG00000183137	-0.354059	4.68855	0.5978	1	CEP57L1	K16762
ENSG00000058804	-0.347101	7.181464	0.59782	1	NDC1	K14315
ENSG00000110315	-0.352724	4.997671	0.597893	1	RNF141	K11447
ENSG00000168405	-0.399377	2.735525	0.597951	1	CMAHP	K08080
ENSG00000148219	-0.38752	2.728579	0.597951	1	ASTN2	_
ENSG00000140092	0.360532	3.786052	0.598011	1	FBLN5	K17340
ENSG00000246067	0.372232	3.342298	0.598032	1	RAB30-AS1	_
ENSG00000155755	-0.353657	4.844534	0.598236	1	TMEM237	_
ENSG00000185917	-0.373835	3.680898	0.598263	1	SETD4	K02927
ENSG00000143878	0.345171	8.731363	0.598365	1	RHOB	K07856
ENSG00000131732	-0.354147	5.182662	0.598397	1	ZCCHC9	K17578
ENSG00000246273	-0.369159	3.93969	0.598406	1	SBF2-AS1	_
ENSG00000121879	-0.350876	4.983894	0.598591	1	PIK3CA	K00922
ENSG00000134744	0.347967	6.214488	0.5987	1	ZCCHC11	K13291
ENSG00000133030	0.345261	7.970173	0.598739	1	MPRIP	_
ENSG00000124733	-0.349291	5.767373	0.598761	1	MEA1	K13980
ENSG00000115761	-0.354433	5.154662	0.598764	1	NOL10	K14788
ENSG00000091039	-0.349135	5.832599	0.598771	1	OSBPL8	_

ENSG00000127955	-0.347045	6.646852	0.598851	1	GNAI1	K04630
ENSG00000150401	0.354611	4.878288	0.598865	1	DCUN1D2	K17822
ENSG00000248370	-0.410814	2.179677	0.598935	1	RP11-366H4.1	_
ENSG00000182858	0.371157	3.710783	0.598964	1	ALG12	K03847
ENSG00000169020	-0.352093	5.268093	0.599015	1	ATP5I	K02129
ENSG00000198265	0.354508	4.688645	0.599017	1	HELZ	_
ENSG00000188725	-0.346633	6.759043	0.599051	1	SMIM15	_
ENSG00000006007	0.345634	7.254755	0.599078	1	GDE1	K19179
ENSG00000236088	-0.386814	2.818384	0.599287	1	COX10-AS1	_
ENSG00000253669	0.38622	2.796521	0.599287	1	KB-1732A1.1	_
ENSG00000136933	-0.349941	5.586011	0.599292	1	RABEPK	_
ENSG00000080819	-0.351472	4.971387	0.599304	1	CPOX	K00228
ENSG00000106404	-0.375843	3.60013	0.599312	1	CLDN15	K06087
ENSG00000125843	0.377264	3.243722	0.599327	1	AP5S1	K19024
ENSG00000113621	-0.350089	5.472252	0.599418	1	TXNDC15	_
ENSG00000130254	0.346821	5.998579	0.599498	1	SAFB2	_
ENSG00000011105	0.354369	4.001547	0.599547	1	TSPAN9	K17350
ENSG00000139372	-0.347163	6.247704	0.599596	1	TDG	K03649
ENSG00000033122	-0.431048	1.767289	0.599598	1	LRRC7	_
ENSG00000092931	-0.362539	3.766942	0.599625	1	MFSD11	K12891
ENSG00000093144	-0.347376	6.207386	0.599735	1	ECHDC1	K18426
ENSG00000135503	0.356014	4.425986	0.599843	1	ACVR1B	K13567
ENSG00000149930	0.350316	5.322152	0.599911	1	TAOK2	K04429
ENSG00000099901	-0.344198	8.029107	0.599925	1	RANBP1	K15306
ENSG00000171551	0.34346	9.166026	0.600002	1	ECEL1	K09610
ENSG00000006118	-0.345431	6.874751	0.600096	1	TMEM132A	K17599
ENSG00000157895	-0.358681	4.166302	0.600247	1	C12orf43	_
ENSG00000160055	-0.378102	3.514569	0.600483	1	TMEM234	K03246
ENSG00000166997	0.35707	4.476172	0.600617	1	CNPY4	_
ENSG00000136463	-0.347718	5.634417	0.600643	1	TACO1	K18189
ENSG00000123552	-0.366855	3.842236	0.600841	1	USP45	K11844
ENSG00000157554	0.351871	4.49969	0.600985	1	ERG	K09435
ENSG00000146476	-0.350994	5.490872	0.601015	1	C6orf211	_
ENSG00000065457	-0.368262	3.377738	0.601038	1	ADAT1	K15440
ENSG00000182979	0.368209	3.359579	0.601038	1	MTA1	K11660
ENSG00000132406	-0.360695	3.373376	0.601038	1	TMEM128	_
ENSG00000031691	-0.347674	5.026159	0.601086	1	CENPQ	K11509
ENSG00000144401	-0.355957	4.681749	0.601114	1	METTL21A	_
ENSG00000174106	-0.352638	4.83689	0.601216	1	LEMD3	K19410
ENSG00000183010	-0.343375	7.570669	0.601247	1	PYCR1	K00286
ENSG00000159403	0.34539	6.092975	0.60138	1	C1R	K01330
ENSG00000007384	0.351072	4.852687	0.601497	1	RHBDF1	_
ENSG00000112304	-0.344793	6.488126	0.601592	1	ACOT13	K17362
ENSG00000105072	-0.365207	3.610769	0.601862	1	C19orf44	_
ENSG00000174231	0.342069	8.287316	0.601895	1	PRPF8	K12856
ENSG00000110318	-0.424592	1.937652	0.602008	1	KIAA1377	_
ENSG00000140465	-0.424592	1.937652	0.602008	1	CYP1A1	K07408
ENSG00000171084	-0.424592	1.937652	0.602008	1	FAM86JP	K03842
ENSG00000134897	-0.350529	4.964388	0.602041	1	BIVM	K10846
ENSG00000147883	0.343237	6.927839	0.60209	1	CDKN2B	K04685
ENSG00000164022	-0.344375	6.402209	0.602255	1	AIMP1	K15437
ENSG00000184489	0.402827	2.374003	0.602421	1	PTP4A3	K18041
ENSG00000063180	0.359404	4.076282	0.602421	1	CA11	_
ENSG00000075089	-0.348357	5.34767	0.602439	1	ACTR6	K11662
ENSG00000139350	-0.348357	5.34767	0.602439	1	NEDD1	K16547
ENSG00000183765	-0.34321	6.863147	0.602466	1	CHEK2	K06641
ENSG00000014824	-0.344645	6.144411	0.602511	1	SLC30A9	K14696
ENSG00000254101	-0.373471	3.475627	0.602513	1	RP11-30J20.1	_

ENSG00000107796	-0.379487	2.754593	0.602528	1	ACTA2	K12313
ENSG00000178537	0.372891	3.262223	0.602535	1	SLC25A20	K15109
ENSG00000166295	-0.34316	6.771935	0.602695	1	ANAPC16	K02927
ENSG00000088682	-0.344177	5.929186	0.602709	1	COQ9	K18587
ENSG00000114904	-0.355665	4.345011	0.602763	1	NEK4	K08857
ENSG00000185697	-0.343586	6.046453	0.602897	1	MYBL1	K09421
ENSG00000135317	-0.345976	5.67355	0.602982	1	SNX14	K17926
ENSG00000187961	-0.348992	4.969984	0.60304	1	KLHL17	K10454
ENSG00000170525	0.349707	5.240854	0.603289	1	PFKFB3	K01103
ENSG00000219201	-0.3818	3.104919	0.603439	1	RP11-181C21.4	_
ENSG00000109320	-0.351319	4.864206	0.603644	1	NFKB1	K02580
ENSG00000003400	-0.486388	1.452725	0.603651	1	CASP10	K04400
ENSG00000162999	-0.486388	1.452725	0.603651	1	DUSP19	K14165
ENSG00000160191	0.352823	4.51321	0.603714	1	PDE9A	K13761
ENSG00000123836	-0.346095	5.501544	0.603786	1	PFKFB2	K19029
ENSG00000259865	-0.39895	2.499749	0.603804	1	RP11-488L18.10	_
ENSG00000104472	-0.342068	6.745249	0.603905	1	CHRAC1	K11656
ENSG00000100288	-0.400678	2.078761	0.603961	1	CHKB	K14156
ENSG00000137218	-0.400678	2.078761	0.603961	1	FRS3	K12461
ENSG00000163067	-0.400678	2.078761	0.603961	1	ZNF2	K09228
ENSG00000143164	0.341948	6.594945	0.604062	1	DCAF6	K11795
ENSG00000013573	-0.342951	6.210356	0.604188	1	DDX11	K11273
ENSG00000152683	-0.343902	5.93564	0.604244	1	SLC30A6	K14693
ENSG00000074266	0.343262	6.285458	0.604379	1	EED	K11462
ENSG00000205213	0.340287	7.517241	0.604431	1	LGR4	K04309
ENSG00000165525	-0.343484	5.800755	0.604596	1	NEMF	_
ENSG00000136044	-0.356089	4.374874	0.604626	1	APPL2	_
ENSG00000120860	-0.353879	3.820531	0.604656	1	CCDC53	K18463
ENSG00000173320	0.349195	4.493871	0.604769	1	STOX2	_
ENSG00000196305	-0.339291	8.516153	0.604787	1	IARS	K01870
ENSG00000132155	0.341043	6.379195	0.6048	1	RAF1	K04366
ENSG00000132639	-0.346248	5.306215	0.604824	1	SNAP25	K18211
ENSG00000127884	-0.339625	7.950419	0.604842	1	ECHS1	K07511
ENSG00000088756	0.355773	3.656345	0.604929	1	ARHGAP28	_
ENSG00000005436	-0.345814	5.121067	0.604971	1	GCFC2	K09061
ENSG00000260852	0.375489	3.210965	0.605003	1	FBXL19-AS1	_
ENSG00000177548	-0.364619	3.231324	0.605003	1	RABEP2	_
ENSG00000143416	0.340823	6.659803	0.605125	1	SELENBP1	K17285
ENSG00000107890	-0.354539	3.863311	0.605127	1	ANKRD26	K17299
ENSG00000187735	-0.339143	8.006837	0.605306	1	TCEA1	K03145
ENSG00000126247	-0.339464	7.61648	0.605344	1	CAPNS1	K08583
ENSG00000151806	-0.352873	4.547721	0.60535	1	GUF1	_
ENSG00000111452	0.520629	0.655605	0.605363	1	GPR133	K08465
ENSG00000128203	0.520629	0.655605	0.605363	1	ASPHD2	_
ENSG00000154310	0.520629	0.655605	0.605363	1	TNIK	K08840
ENSG00000170049	0.520629	0.655605	0.605363	1	KCNAB3	K04884
ENSG00000213213	0.520629	0.655605	0.605363	1	CCDC183	_
ENSG00000225806	0.520629	0.655605	0.605363	1	RP1-309F20.3	_
ENSG00000257093	0.520629	0.655605	0.605363	1	KIAA1147	_
ENSG00000064763	0.450813	0.688218	0.605363	1	FAR2	K13356
ENSG00000106133	0.450813	0.688218	0.605363	1	NSUN5P2	K15264
ENSG00000263327	0.450813	0.688218	0.605363	1	TAPT1-AS1	_
ENSG00000070444	0.34712	5.005449	0.605438	1	MNT	K09115
ENSG00000102048	-0.385636	2.982745	0.605456	1	ASB9	K10331
ENSG00000213782	-0.385636	2.982745	0.605456	1	DDX47	K14777
ENSG00000240849	0.342263	5.742937	0.60557	1	TMEM189	K10704
ENSG00000124466	0.342219	6.263324	0.605641	1	LYPD3	_
ENSG00000104635	0.339166	7.485233	0.605657	1	SLC39A14	K14720

ENSG00000136936	-0.349732	4.067787	0.60567	1	XPA	K10847
ENSG00000134371	-0.343254	5.664084	0.60577	1	CDC73	K15175
ENSG00000061794	-0.339246	7.381715	0.605856	1	MRPS35	K17413
ENSG00000164292	0.339285	7.126995	0.605979	1	RHOBTB3	K07869
ENSG00000075223	-0.342044	6.367105	0.606065	1	SEMA3C	K06840
ENSG00000254726	-0.383712	2.694976	0.606068	1	MEX3A	K15686
ENSG00000152804	-0.350176	4.249182	0.606083	1	HHEX	K08024
ENSG00000136213	0.348012	4.241028	0.606083	1	CHST12	K04742
ENSG00000257218	-0.347812	4.819917	0.606183	1	GATC	K02435
ENSG00000196656	0.33938	6.441668	0.606221	1	AC004057.1	K02976
ENSG00000248049	-0.444988	1.646466	0.606222	1	UBA6-AS1	_
ENSG00000183876	-0.419072	1.630965	0.606222	1	ARSI	K12375
ENSG00000139343	-0.340093	6.570613	0.606372	1	SNRPF	K11098
ENSG00000141098	0.351644	3.96268	0.606401	1	GFOD2	_
ENSG00000152332	-0.339234	6.41357	0.606584	1	UHMK1	K08877
ENSG00000097046	-0.343638	5.636181	0.606632	1	CDC7	K02214
ENSG00000164615	0.346887	5.044568	0.606772	1	CAMLG	K17985
ENSG00000125846	-0.350177	4.006425	0.606785	1	ZNF133	K09228
ENSG00000101846	0.34542	4.961021	0.606795	1	STS	K01131
ENSG00000136159	-0.340508	6.077914	0.606832	1	NUDT15	K03574
ENSG00000136628	-0.337296	8.638336	0.60684	1	EPRS	K14163
ENSG00000197782	0.357256	3.617508	0.606871	1	ZNF780A	K09228
ENSG00000172586	-0.341111	5.658895	0.606873	1	CHCHD1	_
ENSG00000068024	0.349819	4.678412	0.606886	1	HDAC4	K11406
ENSG00000133111	-0.370849	3.398715	0.606922	1	RFXAP	K08063
ENSG00000225190	0.388949	2.287521	0.606931	1	PLEKHM1	K11447
ENSG00000146477	0.346139	4.452368	0.606969	1	SLC22A3	K08200
ENSG00000160785	0.343601	4.800085	0.607067	1	SLC25A44	K15121
ENSG00000120254	-0.33853	7.011717	0.607165	1	MTHFD1L	K13402
ENSG00000131899	0.341977	5.56973	0.607167	1	LLGL1	K06094
ENSG00000165195	-0.341601	5.246326	0.607209	1	PIGA	K03857
ENSG00000059769	-0.346247	4.915878	0.607221	1	DNAJC25	K19371
ENSG00000167863	-0.338432	7.037944	0.607226	1	ATP5H	K02138
ENSG00000168679	0.350948	3.857575	0.607237	1	SLC16A4	K08181
ENSG00000116906	-0.33988	5.650202	0.607371	1	GNPAT	K00649
ENSG00000075407	-0.347234	5.189868	0.607413	1	ZNF37A	K09228
ENSG00000233954	0.338803	6.492311	0.607614	1	RP11-169K16.7	K00416
ENSG00000176731	-0.341102	5.940644	0.607691	1	C8orf59	_
ENSG00000025423	0.35899	3.704235	0.607785	1	HSD17B6	K13369
ENSG00000025156	0.343893	5.14357	0.607795	1	HSF2	K09415
ENSG00000113643	-0.336962	7.790733	0.607823	1	RARS	K01887
ENSG00000205464	0.365914	2.828304	0.607844	1	ATP6AP1L	_
ENSG00000184557	0.340277	5.857969	0.607875	1	SOCS3	K00995
ENSG00000113369	0.34096	5.701333	0.607929	1	ARRDC3	_
ENSG00000124588	-0.337924	6.902862	0.607999	1	NQO2	K08071
ENSG00000102978	-0.340779	6.026445	0.60809	1	POLR2C	K03011
ENSG00000263731	0.437131	1.774551	0.608185	1	RP11-498C9.15	_
ENSG00000010278	0.336231	8.039213	0.608274	1	CD9	K06460
ENSG00000123684	-0.338835	6.350576	0.608425	1	LPGAT1	K13514
ENSG00000138834	0.341172	5.444203	0.608519	1	MAPK8IP3	K04436
ENSG00000095485	-0.34092	5.520747	0.608549	1	CWF19L1	_
ENSG00000163507	-0.339358	6.077268	0.608583	1	KIAA1524	_
ENSG00000149476	-0.339297	5.798401	0.608836	1	DAK	K00863
ENSG00000107929	0.337546	6.440652	0.608942	1	LARP4B	K18763
ENSG00000197548	-0.343657	5.090543	0.608946	1	ATG7	K08337
ENSG00000125037	0.342074	5.175523	0.608958	1	EMC3	K08857
ENSG00000163728	-0.340763	5.266943	0.608969	1	TTC14	_
ENSG00000164190	0.336312	7.246346	0.608979	1	NIPBL	K06672

ENSG00000182831	0.338214	6.168793	0.609046	1	C16orf72	K11504
ENSG00000101166	-0.334877	9.026331	0.609234	1	SLMO2	_
ENSG00000100982	0.341871	5.117242	0.609245	1	PCIF1	K17584
ENSG00000064115	-0.337241	6.623096	0.609282	1	TM7SF3	_
ENSG00000151500	-0.348812	4.44688	0.609527	1	THYN1	_
ENSG00000224837	-0.348275	4.290042	0.609564	1	GCSHP5	K02437
ENSG00000139629	0.336262	6.839286	0.609642	1	GALNT6	K00710
ENSG00000156453	0.338609	5.646268	0.609727	1	PCDH1	K16498
ENSG00000071889	0.370431	1.9348	0.609731	1	FAM3A	_
ENSG00000166598	0.333951	10.99198	0.609764	1	HSP90B1	K09487
ENSG00000111962	0.34266	5.089129	0.609849	1	UST	K03193
ENSG00000140365	-0.338249	6.120709	0.609857	1	COMMD4	_
ENSG00000173226	-0.337982	5.868283	0.609996	1	IQCB1	K16774
ENSG00000130844	0.343255	4.771428	0.610016	1	ZNF331	K09228
ENSG00000141013	0.349824	4.337568	0.610053	1	GAS8	_
ENSG00000142528	0.339129	4.922456	0.61027	1	ZNF473	K09228
ENSG00000172380	-0.334267	8.273307	0.610295	1	GNG12	K04347
ENSG00000023839	0.33578	6.749061	0.610315	1	ABCC2	K05666
ENSG00000183828	0.358064	3.113349	0.610451	1	NUDT14	K08077
ENSG00000112651	-0.337562	6.108704	0.610611	1	MRPL2	K02886
ENSG00000156374	-0.341049	5.382724	0.610708	1	PCGF6	K11470
ENSG00000167363	0.372691	2.685479	0.610715	1	FN3K	K15522
ENSG00000206075	0.335621	6.535556	0.610904	1	SERPINB5	K10139
ENSG00000178821	0.403155	2.07487	0.610979	1	TMEM52	_
ENSG00000125434	0.349409	3.836237	0.611095	1	SLC25A35	K15117
ENSG00000169718	-0.334448	7.258179	0.61113	1	DUS1L	K05542
ENSG00000083937	-0.335478	6.238485	0.611235	1	CHMP2B	K12192
ENSG00000166897	0.340535	5.06178	0.611393	1	ELFN2	K17568
ENSG00000183298	0.355543	3.592281	0.611419	1	RP11-556K13.1	K02998
ENSG00000077942	0.33317	8.152977	0.611457	1	FBLN1	K17307
ENSG00000109445	-0.338252	5.566744	0.611497	1	ZNF330	_
ENSG00000112305	0.351309	3.911083	0.611676	1	SMAP1	K12486
ENSG00000011198	0.344741	3.914077	0.611676	1	ABHD5	K13699
ENSG00000120709	0.334438	6.095523	0.611826	1	FAM53C	K18710
ENSG00000214021	0.35414	3.951343	0.611944	1	TTLL3	K16608
ENSG00000100519	-0.334413	6.338194	0.611987	1	PSMC6	K03064
ENSG00000144810	-0.39833	2.217495	0.612007	1	COL8A1	_
ENSG00000155465	0.70757	-0.136336	0.612094	1	SLC7A7	K13867
ENSG00000198053	0.70757	-0.136336	0.612094	1	SIRPA	K06551
ENSG00000205129	0.70757	-0.136336	0.612094	1	C4orf47	_
ENSG00000214691	0.70757	-0.136336	0.612094	1	AC104654.1	_
ENSG00000260853	0.70757	-0.136336	0.612094	1	RP11-264B17.2	_
ENSG00000272003	0.70757	-0.136336	0.612094	1	RP11-23P13.7	_
ENSG00000174912	-0.630859	-0.017436	0.612094	1	METTL15P1	_
ENSG00000188779	-0.630859	-0.017436	0.612094	1	SKOR1	_
ENSG00000227908	-0.630859	-0.017436	0.612094	1	CTD-2031P19.3	_
ENSG00000253919	-0.630859	-0.017436	0.612094	1	PRKRIRP7	_
ENSG00000256525	-0.630859	-0.017436	0.612094	1	POLG2	K02333
ENSG00000266010	-0.630859	-0.017436	0.612094	1	GATA6-AS1	_
ENSG00000268357	-0.630859	-0.017436	0.612094	1	VN1R81P	_
ENSG00000268707	-0.630859	-0.017436	0.612094	1	RP11-247A12.7	_
ENSG00000272155	-0.630859	-0.017436	0.612094	1	RP11-707M3.3	_
ENSG00000272720	-0.630859	-0.017436	0.612094	1	CTA-228A9.3	_
ENSG00000111863	-0.53875	-0.071453	0.612094	1	ADTRP	_
ENSG00000136286	-0.53875	-0.071453	0.612094	1	MYO1G	K10356
ENSG00000181798	-0.53875	-0.071453	0.612094	1	LINC00471	_
ENSG00000189229	-0.53875	-0.071453	0.612094	1	AC069277.2	_
ENSG00000250116	-0.53875	-0.071453	0.612094	1	RP11-417F21.1	_

ENSG00000254756	-0.53875	-0.071453	0.612094	1	RP11-867G23.12	_
ENSG00000263563	-0.53875	-0.071453	0.612094	1	UBBP4	K04551
ENSG00000268001	-0.53875	-0.071453	0.612094	1	CTC-241F20.3	_
ENSG00000006451	0.334573	6.488877	0.61212	1	RALA	K07834
ENSG00000105732	-0.344588	4.618918	0.612198	1	ZNF574	_
ENSG00000218175	0.342816	4.611754	0.612198	1	AC016739.2	K02942
ENSG00000123219	-0.337827	5.487948	0.612204	1	CENPK	K11503
ENSG00000149084	-0.341936	4.244501	0.612273	1	HSD17B12	_
ENSG00000114503	-0.333677	7.00118	0.612349	1	NCBP2	K12883
ENSG00000261578	-0.478282	1.280849	0.61235	1	RP11-21L23.2	_
ENSG00000265933	-0.478282	1.280849	0.61235	1	LINC00668	_
ENSG00000167011	0.42924	1.241852	0.61235	1	NAT16	_
ENSG00000185561	0.42924	1.241852	0.61235	1	TLCD2	_
ENSG00000213862	0.42924	1.241852	0.61235	1	CTD-2270N23.1	K02936
ENSG00000260466	0.42924	1.241852	0.61235	1	RP4-536B24.2	_
ENSG00000042493	0.33208	8.280495	0.612539	1	CAPG	K10368
ENSG00000110442	0.335789	5.709746	0.612549	1	COMMD9	_
ENSG00000185262	0.334826	5.876771	0.612616	1	UBALD2	_
ENSG00000206562	0.351765	3.766948	0.612671	1	METTL6	K00599
ENSG00000157193	-0.335909	5.671841	0.612849	1	LRP8	_
ENSG00000188266	0.380187	2.323288	0.612869	1	HYKK	K18201
ENSG00000205593	0.380187	2.323288	0.612869	1	DENND6B	_
ENSG00000106105	-0.331651	8.623013	0.612874	1	GARS	K01880
ENSG00000165802	-0.333485	6.768361	0.612978	1	NSMF	_
ENSG00000114026	-0.349324	4.126644	0.61311	1	OGG1	K03660
ENSG00000138448	-0.333185	6.857534	0.613127	1	ITGAV	K06487
ENSG00000117597	-0.337223	5.075023	0.613213	1	DIEXF	K14774
ENSG00000179604	0.333078	6.663246	0.613355	1	CDC42EP4	_
ENSG00000240541	0.375177	2.416643	0.613601	1	TM4SF1-AS1	_
ENSG00000171813	0.33316	5.629505	0.613783	1	PWWP2B	_
ENSG00000071553	0.35233	3.606816	0.613898	1	ATP6AP1	K03662
ENSG00000118193	-0.33494	5.655428	0.613917	1	KIF14	K17915
ENSG00000163840	-0.33494	5.655428	0.613917	1	DTX3L	K06058
ENSG00000254670	-0.433358	1.497479	0.614163	1	CTC-497E21.3	_
ENSG00000103199	0.343071	3.649556	0.614163	1	ZNF500	K09229
ENSG00000182749	0.381073	2.526958	0.61423	1	PAQR7	K04613
ENSG00000239521	0.381073	2.526958	0.61423	1	GATS	K13055
ENSG00000056661	-0.345457	4.560728	0.614281	1	PCGF2	K11460
ENSG00000165175	-0.332241	6.568334	0.614729	1	MID1IP1	_
ENSG00000189114	0.339277	4.645502	0.614787	1	BLOC1S3	_
ENSG00000110497	0.33834	5.216268	0.614899	1	AMBRA1	K17985
ENSG00000187446	0.330694	7.093899	0.615182	1	CHP1	K17610
ENSG00000126456	-0.332192	6.316936	0.615203	1	IRF3	K05411
ENSG00000171425	0.339654	4.773049	0.615439	1	ZNF581	_
ENSG00000079156	-0.361317	3.431414	0.615446	1	OSBPL6	_
ENSG00000185664	0.418543	1.642049	0.615458	1	PMEL	K17304
ENSG00000126945	-0.400338	1.685868	0.615458	1	HNRNPH2	K12898
ENSG00000215644	-0.400338	1.685868	0.615458	1	GCGR	K04583
ENSG00000234062	-0.400338	1.685868	0.615458	1	RP11-308D16.4	_
ENSG00000101310	-0.331957	6.449108	0.6155	1	SEC23B	K14006
ENSG00000197070	-0.33614	5.194642	0.61557	1	ARRDC1	_
ENSG00000133704	-0.331543	6.500796	0.61563	1	IPO8	K10408
ENSG00000118873	-0.33225	5.99041	0.615692	1	RAB3GAP2	_
ENSG00000152465	-0.365	2.890582	0.616059	1	NMT2	K14523
ENSG00000153982	-0.365	2.890582	0.616059	1	GDPD1	K11447
ENSG00000198162	-0.337786	5.150941	0.6162	1	MAN1A2	K01230
ENSG00000132002	-0.329609	7.318426	0.616216	1	DNAJB1	K09507
ENSG00000100417	0.333133	5.403278	0.61652	1	PMM1	K17497

ENSG00000182150	-0.355469	3.670348	0.616537	1	ERCC6L2	_
ENSG00000113119	-0.339441	4.144001	0.616552	1	TMCO6	_
ENSG00000168724	-0.329292	7.273567	0.616617	1	DNAJC21	K09506
ENSG00000102786	-0.330526	5.867544	0.616626	1	INTS6	K13143
ENSG00000083817	-0.372462	3.026146	0.616703	1	ZNF416	K09228
ENSG00000118961	-0.344184	3.710909	0.616716	1	C2orf43	_
ENSG00000146433	-0.333305	5.634425	0.616795	1	TMEM181	K13194
ENSG00000153879	-0.331066	6.44861	0.616866	1	CEBPG	K10049
ENSG00000091986	-0.345204	3.757023	0.616885	1	CCDC80	_
ENSG00000162909	-0.328475	7.723791	0.616955	1	CAPN2	K03853
ENSG00000166783	0.343306	4.284527	0.617144	1	KIAA0430	K17573
ENSG00000159176	-0.328004	8.066426	0.61715	1	CSRP1	K09377
ENSG00000272933	0.385761	1.967986	0.617172	1	RP11-47A8.5	_
ENSG00000226121	0.360382	3.148353	0.61723	1	AHCTF1P1	_
ENSG00000164916	0.329453	6.157125	0.617284	1	FOXK1	K09404
ENSG00000122958	-0.328535	7.367253	0.617302	1	VPS26A	K18466
ENSG00000187634	0.329544	6.469661	0.61753	1	SAMD11	_
ENSG00000135837	0.331406	5.519864	0.617635	1	CEP350	K16768
ENSG00000143028	0.362487	3.267003	0.61767	1	SYPL2	_
ENSG00000142669	0.334851	5.394951	0.617905	1	SH3BGRL3	_
ENSG00000213516	-0.33334	5.411814	0.617947	1	RBMXL1	K12885
ENSG00000100580	-0.338135	4.530684	0.617958	1	TMED8	K17985
ENSG00000108107	0.326571	9.253444	0.617997	1	RPL28	K02903
ENSG00000169918	0.385933	2.223482	0.618242	1	OTUD7A	K11860
ENSG00000130158	0.336909	4.203482	0.618432	1	DOCK6	_
ENSG00000221944	0.336909	4.203482	0.618432	1	TIGD1	K14284
ENSG00000114956	-0.328645	5.966106	0.61845	1	DGUOK	K00904
ENSG00000127989	-0.337798	4.242154	0.618516	1	MTERF	K15031
ENSG00000176108	-0.349867	3.585202	0.61864	1	CHMP6	K12195
ENSG00000124831	0.327456	7.007427	0.618777	1	LRRFIP1	K17785
ENSG00000164142	0.367481	2.449363	0.618968	1	FAM160A1	_
ENSG00000134824	-0.331457	5.266933	0.619013	1	FADS2	K10226
ENSG00000089723	0.493915	0.354975	0.619281	1	OTUB2	K09603
ENSG00000225511	0.493915	0.354975	0.619281	1	LINC00475	_
ENSG00000231043	0.493915	0.354975	0.619281	1	AC007238.1	K13109
ENSG00000266405	0.493915	0.354975	0.619281	1	CBX3P2	_
ENSG00000197724	-0.342597	3.884088	0.619375	1	PHF2	K19414
ENSG00000126756	-0.330099	5.960488	0.619396	1	UXT	_
ENSG00000106018	0.369878	2.637071	0.61949	1	VIPR2	K04590
ENSG00000196739	-0.334493	5.210102	0.619682	1	COL27A1	K19721
ENSG00000007372	0.339739	4.018057	0.619683	1	PAX6	K08031
ENSG00000206530	-0.367726	2.74774	0.6197	1	WDR52	_
ENSG00000117791	-0.334463	4.797994	0.619809	1	2-Mar	_
ENSG00000168672	-0.331947	5.28277	0.619823	1	FAM84B	_
ENSG00000198818	-0.328965	5.311723	0.619876	1	SFT2D1	_
ENSG00000088356	0.328743	5.307827	0.619876	1	PDRG1	_
ENSG00000124120	-0.341202	4.156352	0.619936	1	TTPAL	K17985
ENSG00000214655	0.329795	5.378155	0.62	1	ZSWIM8	_
ENSG00000187840	-0.326397	7.076465	0.620012	1	EIF4EBP1	K07205
ENSG00000127452	-0.338649	4.274207	0.620148	1	FBXL12	K10278
ENSG00000008300	0.360322	2.958262	0.620188	1	CELSR3	K04602
ENSG00000146416	0.360322	2.958262	0.620188	1	AIG1	K16470
ENSG00000148450	-0.339462	4.305562	0.620196	1	MSRB2	K07305
ENSG00000152022	-0.366178	3.047373	0.620316	1	LIX1L	K16673
ENSG00000103707	-0.338037	4.440773	0.620408	1	MTFMT	K00604
ENSG00000158828	0.328491	5.634816	0.620413	1	PINK1	K05688
ENSG00000166451	-0.328615	6.025797	0.620454	1	CENPN	K11506
ENSG00000109501	0.334186	4.582772	0.620617	1	WFS1	K14020

ENSG00000068366	-0.324968	7.815499	0.620629	1	ACSL4	K01897
ENSG00000160783	-0.335156	4.654086	0.620707	1	PMF1	K11546
ENSG00000205758	0.331439	4.649046	0.620707	1	CRYZL1	_
ENSG00000163635	0.337333	4.671595	0.620735	1	ATXN7	K11318
ENSG00000170222	0.356479	3.333925	0.620795	1	ADPRM	K01517
ENSG00000179981	-0.350181	3.347576	0.620795	1	TSHZ1	K09236
ENSG00000151014	-0.351242	3.406651	0.620867	1	CCRN4L	K18764
ENSG00000173214	0.345194	3.385437	0.620867	1	KIAA1919	K08175
ENSG00000165644	-0.329743	5.570437	0.620943	1	COMTD1	_
ENSG00000168438	-0.333612	4.861135	0.620953	1	CDC40	K12816
ENSG00000179546	-0.350511	3.49869	0.620996	1	HTR1D	K04153
ENSG00000087263	-0.328096	6.135134	0.621032	1	OGFOD1	_
ENSG00000143842	0.328488	5.669747	0.621052	1	SOX13	K09269
ENSG00000111252	-0.347445	3.773165	0.621285	1	SH2B3	K12459
ENSG00000139718	0.32709	5.212735	0.621303	1	SETD1B	K11422
ENSG00000140612	0.324248	7.766866	0.621326	1	SEC11A	K13280
ENSG00000136147	-0.338309	3.854053	0.621359	1	PHF11	K18494
ENSG00000114993	-0.339414	3.962021	0.621455	1	RTKN	_
ENSG00000139083	0.336747	4.058176	0.621537	1	ETV6	K03211
ENSG00000040275	-0.324895	6.994707	0.621758	1	SPDL1	_
ENSG00000109929	-0.336763	4.52117	0.621824	1	SC5D	K00227
ENSG00000213380	-0.333349	4.519241	0.621824	1	COG8	_
ENSG00000111615	-0.327231	6.031167	0.621837	1	KRR1	K06961
ENSG00000125827	-0.335607	4.805849	0.621959	1	TMX4	_
ENSG00000165915	-0.32935	4.865635	0.621983	1	SLC39A13	K14719
ENSG00000182952	-0.324764	6.933689	0.622002	1	HMGN4	_
ENSG00000173581	-0.542395	0.789026	0.622031	1	CCDC106	_
ENSG00000187667	-0.542395	0.789026	0.622031	1	WHAMMP3	_
ENSG00000214654	-0.542395	0.789026	0.622031	1	RP11-27I1.4	K03766
ENSG00000223559	-0.542395	0.789026	0.622031	1	RP11-700P18.1	K14288
ENSG00000258727	-0.542395	0.789026	0.622031	1	RP11-66N24.3	_
ENSG00000261204	-0.542395	0.789026	0.622031	1	AC004449.6	_
ENSG00000132026	0.483196	0.764091	0.622031	1	RTBDN	_
ENSG00000188501	0.483196	0.764091	0.622031	1	LCTL	K11447
ENSG00000250451	0.483196	0.764091	0.622031	1	HOXC-AS1	_
ENSG00000123983	-0.324177	7.282338	0.622092	1	ACSL3	K01897
ENSG00000087086	0.322498	9.988412	0.622195	1	FTL	K13625
ENSG00000242485	-0.325622	6.084414	0.622304	1	MRPL20	K02887
ENSG00000111057	-0.322016	11.77047	0.622525	1	KRT18	K07604
ENSG00000181458	0.323911	6.976355	0.622631	1	TMEM45A	_
ENSG00000197498	-0.325529	6.027147	0.622755	1	RPF2	K14847
ENSG00000020426	-0.327543	5.417971	0.622865	1	MNAT1	K10842
ENSG00000155111	0.329012	5.203226	0.622914	1	CDK19	K02208
ENSG00000116198	0.331499	4.444418	0.623149	1	CEP104	K16458
ENSG00000250846	-0.467629	1.085253	0.623263	1	RP11-807H7.1	_
ENSG00000168938	-0.336416	3.973189	0.623355	1	PPIC	K09563
ENSG00000124370	0.337278	3.773579	0.623449	1	MCEE	K05606
ENSG00000115128	-0.323166	7.044659	0.623533	1	SF3B14	K12833
ENSG00000102967	-0.346244	3.514046	0.623594	1	DHODH	K00254
ENSG00000102030	0.344663	3.502369	0.623594	1	NAA10	K00670
ENSG00000105767	0.341609	3.402204	0.623652	1	CADM4	K06783
ENSG00000158636	0.329233	5.237391	0.623698	1	C11orf30	_
ENSG00000160051	-0.352079	3.308328	0.623716	1	IQCC	K11447
ENSG00000267002	0.451934	1.294551	0.623857	1	RP11-242D8.1	_
ENSG00000072818	-0.418701	1.330999	0.623857	1	ACAP1	K12489
ENSG00000119004	-0.418701	1.330999	0.623857	1	CYP20A1	K07435
ENSG00000229656	-0.418701	1.330999	0.623857	1	RP11-462L8.1	_
ENSG00000256294	-0.418701	1.330999	0.623857	1	ZNF225	K09228

ENSG00000145335	-0.358221	2.913876	0.623951	1	SNCA	K04528
ENSG00000136807	0.325636	6.113377	0.624041	1	CDK9	K02211
ENSG00000204947	-0.412097	1.557559	0.624149	1	ZNF425	K09228
ENSG00000232434	0.37824	2.472846	0.624194	1	C9orf172	K01112
ENSG00000247400	0.371855	2.35818	0.624242	1	DNAJC3-AS1	_
ENSG00000162959	0.403685	1.697706	0.624286	1	MEMO1	K06990
ENSG00000198855	0.398175	2.250835	0.624287	1	FICD	_
ENSG00000205903	0.330782	4.617195	0.624297	1	ZNF316	K09228
ENSG00000243452	0.393857	1.839149	0.624339	1	NBPF15	K10653
ENSG00000258947	0.400393	2.000454	0.624344	1	TUBB3	K07375
ENSG00000232472	-0.332953	4.5791	0.624352	1	EEF1B2P3	K03232
ENSG00000110321	-0.320429	9.943166	0.624463	1	EIF4G2	K03260
ENSG00000163683	0.327284	5.151035	0.624611	1	SMIM14	_
ENSG00000168813	-0.328538	5.087939	0.624703	1	ZNF507	_
ENSG00000143643	-0.329455	5.022078	0.624805	1	TTC13	_
ENSG00000111641	-0.321549	7.341327	0.624841	1	NOP2	K14835
ENSG00000084073	-0.322049	6.950084	0.624892	1	ZMPSTE24	K06013
ENSG00000116750	-0.321932	7.000887	0.624932	1	UCHL5	K05610
ENSG00000101152	0.321848	6.335207	0.624944	1	DNAJC5	K09525
ENSG00000104611	-0.323921	6.412074	0.625174	1	SH2D4A	K17577
ENSG00000099385	-0.326847	5.248615	0.62527	1	BCL7C	_
ENSG00000099330	0.328527	5.229219	0.625298	1	OCEL1	_
ENSG00000132463	-0.320602	7.635418	0.625511	1	GRSF1	_
ENSG00000137338	-0.339392	3.826538	0.625512	1	PGBD1	K09230
ENSG00000163611	0.331596	4.577278	0.625591	1	SPICE1	K16490
ENSG00000135637	-0.338164	3.707475	0.625759	1	CCDC142	K17434
ENSG00000182768	-0.32581	5.349804	0.62582	1	NGRN	K13194
ENSG00000272016	-0.333586	4.410169	0.62597	1	RP11-215G15.5	_
ENSG00000159348	0.323499	5.530354	0.626081	1	CYB5R1	K00326
ENSG00000260916	-0.324622	5.485017	0.626203	1	CCPG1	K04613
ENSG00000064309	0.33434	4.238819	0.626378	1	CDON	_
ENSG00000186716	0.321888	5.857943	0.626401	1	BCR	K08878
ENSG00000113732	0.320289	6.958794	0.626557	1	ATP6V0E1	K02153
ENSG00000162368	-0.31948	7.709264	0.626644	1	CMPK1	K13800
ENSG00000129219	0.327885	4.631454	0.626692	1	PLD2	K01115
ENSG00000125814	-0.345874	3.26326	0.626823	1	NAPB	_
ENSG00000107242	0.33937	3.244513	0.626823	1	PIP5K1B	K00889
ENSG00000168872	-0.323878	5.448377	0.62698	1	DDX19A	K18655
ENSG00000100532	-0.34444	3.197848	0.626985	1	CGRRF1	_
ENSG00000232838	-0.351899	3.13452	0.627161	1	PET117	K18188
ENSG00000168209	0.318123	8.560231	0.627385	1	DDIT4	K08270
ENSG00000025770	0.322252	5.87151	0.62739	1	NCAPH2	K11490
ENSG00000105568	0.31834	8.140963	0.627401	1	PPP2R1A	K03456
ENSG00000100281	-0.323896	5.313922	0.627409	1	HMGXB4	K11298
ENSG00000111361	-0.320551	6.485763	0.627447	1	EIF2B1	K03239
ENSG00000188229	-0.317912	8.927824	0.627501	1	TUBB4B	K07375
ENSG00000185278	0.338189	3.83022	0.627573	1	ZBTB37	K10509
ENSG00000204839	0.322229	5.792888	0.627659	1	MROH6	_
ENSG00000131467	-0.318469	7.470411	0.628	1	PSME3	K06698
ENSG00000133678	-0.350186	2.85425	0.628027	1	TMEM254	_
ENSG00000103365	0.321288	6.089701	0.628037	1	GGA2	K12404
ENSG00000135108	0.322189	5.666287	0.628125	1	FBXO21	K10301
ENSG00000104957	-0.331594	4.569901	0.628136	1	CCDC130	K13115
ENSG00000162775	0.320854	5.464243	0.628283	1	RBM15	K13190
ENSG00000118689	0.320194	5.622383	0.6283	1	FOXO3	K09408
ENSG00000101343	-0.327943	4.524928	0.628321	1	CRNKL1	K12869
ENSG00000182810	-0.327943	4.524928	0.628321	1	DDX28	K05543
ENSG00000137203	0.317542	7.913034	0.628433	1	TFAP2A	K09176

ENSG00000134014	-0.322726	5.246304	0.628436	1	ELP3	K07739
ENSG00000116171	-0.319821	6.29483	0.628466	1	SCP2	K08764
ENSG00000041802	-0.321511	5.971712	0.62851	1	LSG1	K14539
ENSG00000178980	-0.323626	5.519795	0.628735	1	SEPW1	_
ENSG00000174891	-0.323999	4.710327	0.628773	1	RSRC1	_
ENSG00000139233	-0.319778	5.780212	0.628811	1	LLPH	K17985
ENSG00000118900	0.319307	6.27975	0.628906	1	UBN1	K17492
ENSG00000182685	0.366862	2.586995	0.62892	1	BRICD5	_
ENSG00000113407	-0.316336	9.559028	0.628983	1	TARS	K01868
ENSG00000101882	-0.323622	5.457375	0.629007	1	NKAP	_
ENSG00000117450	0.316194	9.961135	0.629012	1	PRDX1	K13279
ENSG00000033030	-0.322472	5.29285	0.629016	1	ZCCHC8	K13128
ENSG00000140564	0.318583	6.56363	0.629104	1	FURIN	K01349
ENSG00000152455	-0.320936	5.234718	0.629281	1	SUV39H2	K11419
ENSG00000234743	-0.361555	2.513961	0.629284	1	EIF5AP4	K03263
ENSG00000116478	0.317165	7.399531	0.629337	1	HDAC1	K06067
ENSG00000113456	-0.317589	7.100953	0.629454	1	RAD1	K02830
ENSG00000103260	-0.318108	6.757938	0.629492	1	METRN	K09228
ENSG00000117151	-0.324987	5.007164	0.629493	1	CTBS	K12310
ENSG00000090470	-0.32083	5.460362	0.629678	1	PDCD7	_
ENSG00000108262	0.317794	6.633417	0.629815	1	GIT1	K05737
ENSG00000105726	0.31833	6.396957	0.629843	1	ATP13A1	K14950
ENSG00000167792	-0.317376	6.889712	0.630036	1	NDUFV1	K03942
ENSG00000170832	0.321246	5.378159	0.630074	1	USP32	K11837
ENSG00000157426	-0.340565	3.724182	0.630212	1	AASDH	K00142
ENSG00000156170	-0.325801	4.648812	0.630231	1	NDUFAF6	K18163
ENSG00000105855	-0.321136	5.342264	0.630289	1	ITGB8	K06591
ENSG00000152291	0.315919	7.755079	0.630323	1	TGOLN2	K08857
ENSG00000100129	0.315312	8.48388	0.630468	1	EIF3L	K15029
ENSG00000135829	-0.315795	7.862781	0.630479	1	DHX9	K13184
ENSG00000187193	-0.336555	4.03596	0.630584	1	MT1X	K14739
ENSG00000133318	0.315737	7.541134	0.630727	1	RTN3	_
ENSG00000173726	0.315126	8.361557	0.630738	1	TOMM20	K17770
ENSG00000128595	0.31483	9.038756	0.630741	1	CALU	_
ENSG00000138777	-0.318935	5.998677	0.630754	1	PPA2	K01507
ENSG00000179523	-0.354173	3.088921	0.630776	1	EIF3J-AS1	_
ENSG00000137177	0.316749	6.701671	0.630814	1	KIF13A	K17914
ENSG00000172167	-0.321001	4.921592	0.630905	1	MTBP	_
ENSG00000076706	0.314877	8.431156	0.630968	1	MCAM	K06534
ENSG00000242616	-0.325275	4.718673	0.63101	1	GNG10	K04545
ENSG00000184428	-0.318785	5.954165	0.63101	1	TOP1MT	K03163
ENSG00000104164	-0.318135	6.103662	0.631056	1	BLOC1S6	_
ENSG00000143420	-0.315498	7.531584	0.631139	1	ENSA	_
ENSG00000153006	-0.319555	5.543463	0.631223	1	SREK1IP1	_
ENSG00000171155	-0.319555	5.543463	0.631223	1	C1GALT1C1	K09653
ENSG00000163535	-0.320212	5.301757	0.631261	1	SGOL2	K11581
ENSG00000146556	-0.36229	2.056151	0.631262	1	WASH2P	K18461
ENSG00000136478	0.319549	5.113483	0.631577	1	TEX2	_
ENSG00000095739	-0.326191	4.405993	0.631613	1	BAMBI	K10162
ENSG00000162769	-0.326191	4.405993	0.631613	1	FLVCR1	K08220
ENSG00000136878	0.32317	4.403563	0.631613	1	USP20	K11848
ENSG00000102921	-0.322513	4.961502	0.63164	1	N4BP1	_
ENSG00000123685	-0.382262	1.912552	0.631943	1	BATF3	K09034
ENSG00000214653	-0.382262	1.912552	0.631943	1	HNRNPA3P3	K12741
ENSG00000187156	0.381743	1.887776	0.631943	1	LINC00221	_
ENSG00000189319	-0.324037	4.540046	0.632125	1	FAM53B	_
ENSG00000135940	-0.314648	7.461513	0.632142	1	COX5B	K02265
ENSG00000162642	-0.317166	5.91406	0.632259	1	C1orf52	_

ENSG00000120137	-0.317476	6.24432	0.63226	1	PANK3	K09680
ENSG00000165527	-0.31727	6.016379	0.632527	1	ARF6	K07941
ENSG00000137992	-0.319739	5.432238	0.632601	1	DBT	K09699
ENSG00000259230	-0.407267	1.753118	0.63273	1	CTD-2555C10.3	_
ENSG00000137364	-0.316421	6.052459	0.632736	1	TPMT	K00569
ENSG00000078808	0.314133	7.279134	0.632762	1	SDF4	_
ENSG00000114796	0.324064	4.785904	0.632776	1	KLHL24	K10461
ENSG00000172534	0.34172	2.699991	0.632987	1	HCFC1	K14966
ENSG00000085998	-0.315362	6.357069	0.633006	1	POMGNT1	K09666
ENSG00000089159	0.314373	6.891556	0.633054	1	PXN	K05760
ENSG00000074276	0.759385	-0.698071	0.63315	1	CDHR2	K16502
ENSG00000145965	0.759385	-0.698071	0.63315	1	C6ORF50	_
ENSG00000157315	0.759385	-0.698071	0.63315	1	TMED6	_
ENSG00000162641	0.759385	-0.698071	0.63315	1	AKNAD1	_
ENSG00000166682	0.759385	-0.698071	0.63315	1	TMPRSS5	K09636
ENSG00000168484	0.759385	-0.698071	0.63315	1	SFTPC	_
ENSG00000177236	0.759385	-0.698071	0.63315	1	AP006621.1	_
ENSG00000179761	0.759385	-0.698071	0.63315	1	PIPOX	K00306
ENSG00000180767	0.759385	-0.698071	0.63315	1	CHST13	K07779
ENSG00000183607	0.759385	-0.698071	0.63315	1	GKN2	_
ENSG00000185130	0.759385	-0.698071	0.63315	1	HIST1H2BL	K11252
ENSG00000188152	0.759385	-0.698071	0.63315	1	NUTM2G	_
ENSG00000196622	0.759385	-0.698071	0.63315	1	RIMBP3	K04552
ENSG00000221949	0.759385	-0.698071	0.63315	1	C12orf61	_
ENSG00000223653	0.759385	-0.698071	0.63315	1	RP11-131L23.1	_
ENSG00000227536	0.759385	-0.698071	0.63315	1	SOCS5P4	K04698
ENSG00000231234	0.759385	-0.698071	0.63315	1	SKP1P1	K03094
ENSG00000231652	0.759385	-0.698071	0.63315	1	RP11-553A21.3	_
ENSG00000236204	0.759385	-0.698071	0.63315	1	AC092594.1	_
ENSG00000243708	0.759385	-0.698071	0.63315	1	PLA2G4B	K16342
ENSG00000246339	0.759385	-0.698071	0.63315	1	EXTL3-AS1	_
ENSG00000248027	0.759385	-0.698071	0.63315	1	CTD-2383M3.1	_
ENSG00000248554	0.759385	-0.698071	0.63315	1	RP11-159F24.6	_
ENSG00000259031	0.759385	-0.698071	0.63315	1	CTD-2062F14.3	_
ENSG00000259366	0.759385	-0.698071	0.63315	1	CTD-2647L4.4	_
ENSG00000259826	0.759385	-0.698071	0.63315	1	RP11-467D6.1	_
ENSG00000260176	0.759385	-0.698071	0.63315	1	CTD-3126B10.4	_
ENSG00000262484	0.759385	-0.698071	0.63315	1	CCER2	_
ENSG00000267053	0.759385	-0.698071	0.63315	1	CTD-3162L10.1	_
ENSG00000267419	0.759385	-0.698071	0.63315	1	CTC-559E9.6	_
ENSG00000270640	0.759385	-0.698071	0.63315	1	RP11-373D23.2	_
ENSG00000273374	0.759385	-0.698071	0.63315	1	RP11-383I23.2	_
ENSG00000004478	-0.312645	8.668388	0.633331	1	FKBP4	K09571
ENSG00000166033	0.314628	6.29212	0.633471	1	HTRA1	K08784
ENSG00000196865	-0.323133	4.686708	0.633539	1	NHLRC2	K12581
ENSG00000166716	0.317179	5.072312	0.633625	1	ZNF592	K19325
ENSG00000244560	-0.43949	1.573925	0.633648	1	RP4-800G7.2	K09228
ENSG00000236624	0.415866	1.540924	0.633648	1	CCDC163P	_
ENSG00000156136	-0.322257	5.060628	0.633753	1	DCK	K00893
ENSG00000166266	-0.318091	4.933099	0.633787	1	CUL5	K10612
ENSG00000153944	0.314145	6.300501	0.633788	1	MSI2	K15696
ENSG00000118939	-0.325233	4.307667	0.633791	1	UCHL3	K05609
ENSG00000107938	0.317919	4.910889	0.633931	1	EDRF1	K09228
ENSG00000140264	0.312002	8.615888	0.63399	1	SERF2	_
ENSG00000110958	-0.311742	9.071283	0.634129	1	PTGES3	K15730
ENSG00000167625	0.324122	4.440427	0.634137	1	ZNF526	K17985
ENSG00000086544	0.330088	3.776882	0.634235	1	ITPKC	K00911
ENSG00000186834	0.312913	7.071137	0.634362	1	HEXIM1	K15189

ENSG00000100644	0.312042	7.933989	0.634375	1	HIF1A	K08268
ENSG00000100242	0.315575	6.092266	0.634653	1	SUN2	K19347
ENSG00000177058	-0.328484	3.981447	0.634664	1	SLC38A9	K14995
ENSG00000135926	0.314657	5.977761	0.634694	1	TMBIM1	K06890
ENSG00000167306	0.31749	5.043305	0.634766	1	MYO5B	K10357
ENSG00000114127	-0.320482	4.669588	0.63485	1	XRN1	K12618
ENSG00000092094	-0.33212	3.704032	0.634913	1	OSGEP	K01409
ENSG00000119042	-0.350898	2.434591	0.634991	1	SATB2	_
ENSG00000198498	-0.319385	5.212446	0.635064	1	TMA16	K14860
ENSG00000143549	-0.31083	9.154255	0.635088	1	TPM3	K09290
ENSG00000110888	-0.316466	5.289489	0.635191	1	CAPRIN2	K18744
ENSG00000103274	0.317879	5.095616	0.635202	1	NUBP1	_
ENSG00000132330	-0.345326	2.959378	0.635222	1	SCLY	K01763
ENSG00000155636	-0.335478	3.610317	0.635673	1	RBM45	_
ENSG00000272047	-0.325636	4.424465	0.635696	1	GTF2H5	K10845
ENSG00000253251	0.36824	2.29727	0.635841	1	CTC-534A2.2	_
ENSG00000171723	0.347825	2.306945	0.635841	1	GPHN	K15376
ENSG00000092108	-0.315219	5.65631	0.635941	1	SCFD1	_
ENSG00000111752	-0.436973	1.164565	0.635966	1	PHC1	K11456
ENSG00000248161	-0.436973	1.164565	0.635966	1	RP11-499E18.1	_
ENSG00000105672	0.426679	1.107284	0.635966	1	ETV2	K09432
ENSG00000271976	-0.399661	1.142328	0.635966	1	RP11-884K10.7	_
ENSG00000100814	0.314381	5.88865	0.635979	1	CCNB1IP1	K12581
ENSG00000149823	0.315973	5.361167	0.63601	1	VPS51	K04257
ENSG00000163608	-0.313661	5.861556	0.636247	1	C3orf17	K12756
ENSG00000145220	-0.314558	5.851729	0.636339	1	LYAR	K15263
ENSG00000184634	0.318474	4.861892	0.636378	1	MED12	K15162
ENSG00000099991	0.313602	5.779348	0.63649	1	CABIN1	K17613
ENSG00000103995	-0.312848	6.215156	0.636521	1	CEP152	K16728
ENSG00000223749	-0.345904	2.823477	0.636526	1	MIR503HG	_
ENSG00000151006	0.33436	2.798009	0.636526	1	PRSS53	_
ENSG00000213337	0.327299	3.510388	0.636533	1	ANKRD39	_
ENSG00000158470	-0.31368	6.068867	0.636674	1	B4GALT5	K09905
ENSG00000130449	0.317766	4.717474	0.636694	1	ZSWIM6	_
ENSG00000140280	-0.326912	4.318697	0.636706	1	LYSMD2	_
ENSG00000101844	0.319684	4.311499	0.636706	1	ATG4A	K08342
ENSG00000248275	-0.381087	2.207336	0.636814	1	TRIM52-AS1	_
ENSG00000177917	-0.319688	4.700234	0.636903	1	ARL6IP6	_
ENSG00000197841	-0.333624	3.471004	0.637007	1	ZNF181	K09228
ENSG00000067057	0.309603	7.7073	0.637219	1	PFKP	K00850
ENSG00000089091	-0.498563	0.885656	0.63737	1	DZANK1	K10408
ENSG00000135477	-0.498563	0.885656	0.63737	1	KRT121P	K07605
ENSG00000198150	-0.498563	0.885656	0.63737	1	AC135178.1	K17985
ENSG00000260804	-0.498563	0.885656	0.63737	1	RP11-566E18.3	_
ENSG00000061455	-0.453003	0.858272	0.63737	1	PRDM6	_
ENSG00000106089	-0.453003	0.858272	0.63737	1	STX1A	K04560
ENSG00000111817	-0.453003	0.858272	0.63737	1	DSE	K01794
ENSG00000134245	-0.453003	0.858272	0.63737	1	WNT2B	K00182
ENSG00000143036	-0.453003	0.858272	0.63737	1	SLC44A3	K15282
ENSG00000213385	-0.453003	0.858272	0.63737	1	RP11-577H5.1	K02936
ENSG00000241361	-0.453003	0.858272	0.63737	1	SLC25A24P1	K14684
ENSG00000242715	-0.453003	0.858272	0.63737	1	CCDC169	_
ENSG00000267244	-0.453003	0.858272	0.63737	1	CTB-31O20.4	_
ENSG00000143575	-0.310177	7.15668	0.637398	1	HAX1	K16220
ENSG00000213860	0.315995	5.437131	0.637419	1	RPL21P75	K02889
ENSG00000110851	-0.313543	5.505406	0.637423	1	PRDM4	K12463
ENSG00000060688	-0.310683	6.744551	0.637553	1	SNRNP40	K12857
ENSG00000242086	0.323876	3.869973	0.637561	1	LINC00969	_

ENSG00000123992	-0.313813	4.950188	0.637564	1	DNPEP	K01267
ENSG00000135847	-0.313052	5.720842	0.637656	1	ACBD6	_
ENSG00000143971	-0.311479	5.605946	0.637667	1	ETAA1	_
ENSG00000162236	0.315115	5.187666	0.637716	1	STX5	K08490
ENSG00000134597	-0.317138	4.611629	0.637806	1	RBMX2	K13107
ENSG00000005194	-0.311242	6.320287	0.637824	1	CIAPIN1	_
ENSG00000171262	-0.311423	6.20629	0.63785	1	FAM98B	K15434
ENSG00000186174	0.312708	5.741266	0.637978	1	BCL9L	_
ENSG00000107789	-0.312589	5.509258	0.63806	1	MINPP1	K03103
ENSG00000167595	-0.341123	3.05736	0.63811	1	C19orf55	_
ENSG00000125629	0.321992	4.678544	0.638242	1	INSIG2	_
ENSG00000126803	0.320407	3.792934	0.638377	1	HSPA2	K03283
ENSG00000155393	-0.321317	3.96463	0.638669	1	HEATR3	_
ENSG00000224020	-0.541479	0.504309	0.638759	1	MIR181A2HG	_
ENSG00000227268	-0.541479	0.504309	0.638759	1	KLLN	K17985
ENSG00000148357	0.531978	0.44934	0.638759	1	HMCN2	K17341
ENSG00000163009	0.531978	0.44934	0.638759	1	C2orf48	K11447
ENSG00000241528	0.531978	0.44934	0.638759	1	RP1-130H16.16	_
ENSG00000249485	0.531978	0.44934	0.638759	1	RBBP4P1	K10752
ENSG00000272518	0.531978	0.44934	0.638759	1	RP11-26J3.3	_
ENSG00000083067	0.450301	0.487269	0.638759	1	TRPM3	K04978
ENSG00000122477	0.450301	0.487269	0.638759	1	LRRC39	_
ENSG00000128917	0.450301	0.487269	0.638759	1	DLL4	K06051
ENSG00000269439	0.450301	0.487269	0.638759	1	CTD-3131K8.2	_
ENSG00000158805	-0.317707	4.838297	0.638779	1	ZNF276	_
ENSG00000102753	-0.309941	6.532434	0.638806	1	KPNA3	_
ENSG00000205808	0.334825	2.970155	0.638836	1	PPAPDC2	_
ENSG00000197712	0.311426	5.840406	0.639058	1	FAM114A1	_
ENSG00000125445	-0.309525	6.62186	0.63906	1	MRPS7	K02992
ENSG00000213214	-0.366458	2.552048	0.639076	1	ARHGEF35	_
ENSG00000242028	0.346378	2.543254	0.639076	1	HYPK	_
ENSG00000100296	-0.31041	5.923482	0.639166	1	THOC5	K13174
ENSG00000168393	-0.309687	5.96193	0.639216	1	DTYMK	K00943
ENSG00000174446	-0.312327	5.68213	0.639239	1	SNAPC5	K15211
ENSG00000132294	-0.309112	6.027179	0.639408	1	EFR3A	_
ENSG00000072163	0.31718	4.771534	0.639463	1	LIMS2	_
ENSG00000144827	-0.309883	6.018421	0.639509	1	ABHD10	K13702
ENSG00000163832	-0.319783	4.571679	0.639512	1	ELP6	_
ENSG00000120733	0.308467	6.195335	0.639592	1	KDM3B	K15601
ENSG00000136827	-0.31062	5.946388	0.639894	1	TOR1A	_
ENSG00000119878	-0.317267	4.736821	0.639952	1	CRIPT	_
ENSG00000132434	-0.335784	3.19288	0.639955	1	LANCL2	_
ENSG00000167508	-0.312229	5.898	0.639975	1	MVD	K01597
ENSG00000143756	0.309547	6.157077	0.640055	1	FBXO28	K10306
ENSG00000181577	-0.31349	4.904123	0.640077	1	C6orf223	_
ENSG00000175455	-0.307889	6.254706	0.64012	1	CCDC14	K16541
ENSG00000166147	0.306858	7.785608	0.640136	1	FBN1	K06825
ENSG00000105135	0.31064	5.876735	0.640204	1	ILVBL	K11259
ENSG00000177225	0.313371	5.184049	0.640258	1	PDDC1	_
ENSG00000163956	0.308175	6.603434	0.640294	1	LRPAP1	_
ENSG00000085760	-0.309355	5.909708	0.640342	1	MTIF2	K02519
ENSG00000104133	-0.312984	5.357294	0.640524	1	SPG11	K19026
ENSG00000171316	0.311655	5.80628	0.640545	1	CHD7	K14437
ENSG00000166164	-0.310287	5.929961	0.640588	1	BRD7	K08047
ENSG00000100097	0.305712	8.912046	0.640706	1	LGALS1	K06830
ENSG00000223361	0.389952	1.751272	0.640815	1	FTH1P10	K00522
ENSG00000272654	0.389952	1.751272	0.640815	1	RP11-422P24.11	_
ENSG00000125388	0.359686	1.765755	0.640815	1	GRK4	K08291

ENSG00000174672	0.359686	1.765755	0.640815	1	BRSK2	K08796
ENSG00000181513	0.328846	3.578077	0.640867	1	ACBD4	K02927
ENSG00000214114	-0.310969	5.547216	0.6409	1	MYCBP	_
ENSG00000169228	0.322885	4.074044	0.640965	1	RAB24	K07912
ENSG00000158402	0.315138	4.444526	0.641006	1	CDC25C	K05867
ENSG00000021574	-0.311421	5.124774	0.641043	1	SPAST	K13254
ENSG00000111266	0.308005	5.850331	0.641058	1	DUSP16	K04459
ENSG00000182180	-0.307006	6.987899	0.641108	1	MRPS16	K02959
ENSG00000122565	-0.305393	8.634672	0.641243	1	CBX3	K11586
ENSG00000250479	-0.307798	6.456184	0.64131	1	CHCHD10	_
ENSG00000198668	-0.30614	7.488142	0.641353	1	CALM1	K02183
ENSG00000215246	-0.325149	3.730586	0.641437	1	RP11-43F13.3	_
ENSG00000135631	0.307599	5.810976	0.641569	1	RAB11FIP5	K12484
ENSG00000112877	0.309507	5.5332	0.641672	1	CEP72	K16532
ENSG00000150907	-0.313681	4.764465	0.641817	1	FOXO1	K07201
ENSG00000128513	-0.31193	5.36156	0.641922	1	POT1	K11109
ENSG00000069849	0.304659	8.589408	0.642	1	ATP1B3	K01540
ENSG00000158290	-0.306771	6.618308	0.64206	1	CUL4B	K10609
ENSG00000114491	-0.307906	5.878785	0.642281	1	UMPS	K13421
ENSG00000095794	-0.313818	4.454667	0.64231	1	CREM	K09052
ENSG00000146085	-0.314666	5.02608	0.642359	1	MUT	K01847
ENSG00000110435	-0.305917	6.896758	0.64245	1	PDHX	K13997
ENSG00000173457	-0.308864	5.784998	0.642491	1	PPP1R14B	K17555
ENSG00000198431	0.303947	9.105989	0.642561	1	TXNRD1	K00384
ENSG00000168237	-0.346613	2.673387	0.64257	1	GLYCTK	K15788
ENSG00000139291	-0.316242	4.428477	0.642663	1	TMEM19	K17985
ENSG00000130714	-0.392681	1.615273	0.642691	1	POMT1	K00728
ENSG00000174173	-0.305639	6.82827	0.642876	1	TRMT10C	K17654
ENSG00000069248	-0.3062	6.38566	0.642958	1	NUP133	K14300
ENSG00000050393	-0.30643	6.371448	0.64315	1	MCUR1	K12606
ENSG00000112378	0.303417	8.932311	0.643205	1	PERP	K10136
ENSG00000126351	0.309159	5.462254	0.643328	1	THRA	K05547
ENSG00000171132	-0.330783	2.92477	0.643347	1	PRKCE	K18050
ENSG00000137074	-0.306409	6.043876	0.64335	1	APTX	K10863
ENSG00000111880	-0.314999	4.560559	0.643435	1	RNGTT	K13917
ENSG00000263244	0.308942	5.352599	0.643506	1	RP11-473I1.10	_
ENSG00000130348	-0.308327	5.45338	0.643511	1	QRSL1	K02433
ENSG00000054282	-0.321761	3.743683	0.643563	1	SDCCAG8	K16488
ENSG00000152402	-0.344431	2.573628	0.643788	1	GUCY1A2	K12318
ENSG00000179965	-0.344431	2.573628	0.643788	1	ZNF771	K11504
ENSG00000254967	-0.344431	2.573628	0.643788	1	RP11-680F20.6	_
ENSG00000182307	-0.303853	7.524048	0.643806	1	C8orf33	_
ENSG00000189376	-0.321157	3.871911	0.643852	1	C8orf76	_
ENSG00000026297	-0.315747	3.86885	0.643852	1	RNASET2	K01166
ENSG00000261824	-0.315747	3.86885	0.643852	1	LINC00662	_
ENSG00000146677	-0.320514	3.551493	0.643879	1	AC004453.8	K02912
ENSG00000102078	-0.328472	3.143959	0.643889	1	SLC25A14	K15106
ENSG00000116691	-0.30898	5.467306	0.643973	1	MIIP	_
ENSG00000147894	-0.315669	3.986845	0.644095	1	C9orf72	_
ENSG00000088833	0.307373	5.544535	0.644127	1	NSFL1C	K14012
ENSG00000126070	-0.310795	4.515283	0.644139	1	AGO3	K11593
ENSG00000018610	-0.308226	4.901177	0.644153	1	CXorf56	_
ENSG00000140350	-0.302884	7.923454	0.644467	1	ANP32A	K18646
ENSG00000154719	-0.307321	5.562126	0.644553	1	MRPL39	K17420
ENSG00000116001	-0.304797	6.218709	0.644638	1	TIA1	K13201
ENSG00000025708	-0.303751	6.908845	0.644789	1	TYMP	K00758
ENSG00000220842	0.30413	6.15275	0.644801	1	RP11-572P18.1	K02889
ENSG00000122034	-0.3043	6.594496	0.644801	1	GTF3A	K09191

ENSG00000067829	0.33654	3.067503	0.644831	1	IDH3G	K00030
ENSG00000147852	0.307078	5.204449	0.64493	1	VLDLR	_
ENSG00000254505	-0.426477	1.41668	0.644988	1	CHMP4A	K12194
ENSG00000254761	0.390911	1.384366	0.644988	1	RP11-672A2.1	_
ENSG00000137996	-0.304572	6.268415	0.64508	1	RTCA	K01974
ENSG00000116670	0.304911	6.406785	0.645133	1	MAD2L2	K13728
ENSG00000092203	0.307993	5.561373	0.645155	1	TOX4	_
ENSG00000163785	-0.304135	6.429006	0.645158	1	RYK	K05128
ENSG00000048471	0.349142	2.458014	0.645163	1	SNX29	K17935
ENSG00000168890	0.349142	2.458014	0.645163	1	TMEM150A	_
ENSG00000102763	-0.305605	5.33359	0.645325	1	VWA8	_
ENSG00000136813	-0.302826	7.187084	0.645359	1	KIAA0368	K11886
ENSG00000101337	0.303672	6.48486	0.645438	1	TM9SF4	K17086
ENSG00000152056	-0.328999	3.606626	0.645526	1	AP1S3	K12395
ENSG00000072422	-0.308658	4.716936	0.645927	1	RHOBTB1	K07868
ENSG00000166289	0.307125	5.191288	0.645941	1	PLEKHF1	_
ENSG00000089818	-0.308936	4.783655	0.645971	1	NECAP1	_
ENSG00000130723	0.301576	7.674312	0.646003	1	PRRC2B	_
ENSG00000069011	0.302424	6.891507	0.646047	1	PITX1	K09356
ENSG00000075702	0.304275	5.733024	0.646055	1	WDR62	K11447
ENSG00000160445	0.308025	4.962535	0.646081	1	ZER1	K10350
ENSG00000155324	-0.303159	6.143888	0.646255	1	GRAMD3	_
ENSG00000089639	0.326567	3.552156	0.646264	1	GMIP	_
ENSG00000115685	-0.304126	5.366872	0.646274	1	PPP1R7	K17550
ENSG00000130175	0.300766	8.53015	0.646287	1	PRKCSH	K08288
ENSG00000158220	0.34404	1.947441	0.646292	1	ESYT3	K12581
ENSG00000162670	0.34404	1.947441	0.646292	1	BRINP3	_
ENSG00000008130	0.302565	6.543471	0.646524	1	NADK	K00858
ENSG00000107560	-0.315959	4.27176	0.646542	1	RAB11FIP2	K12484
ENSG00000145354	-0.304498	5.835729	0.646605	1	CISD2	_
ENSG00000242247	0.302592	6.485346	0.646616	1	ARFGAP3	K12493
ENSG00000135916	0.307577	4.804945	0.646658	1	ITM2C	K18265
ENSG00000156414	0.305705	5.381332	0.646733	1	TDRD9	K18408
ENSG00000198961	0.302218	6.550515	0.646889	1	PJA2	K10634
ENSG00000181915	-0.304894	5.610438	0.646947	1	ADO	K10712
ENSG00000103335	0.300173	8.470358	0.646968	1	PIEZO1	_
ENSG00000139197	0.301604	6.863114	0.646989	1	PEX5	K13342
ENSG00000143919	0.331471	2.930524	0.646989	1	CAMKMT	K18826
ENSG00000183475	-0.314593	4.239645	0.64703	1	ASB7	K10329
ENSG00000196670	-0.314581	4.141292	0.647035	1	ZFP62	K09228
ENSG00000131697	0.313096	3.502907	0.647052	1	NPHP4	K16478
ENSG00000138347	0.300723	7.374261	0.647248	1	MYPN	K08834
ENSG00000116539	0.307974	5.199676	0.647481	1	ASH1L	K06101
ENSG00000138071	-0.299764	8.468101	0.64749	1	ACTR2	K17260
ENSG00000187741	0.304108	5.4126	0.64762	1	FANCA	K10888
ENSG00000180573	0.310636	4.870951	0.647631	1	HIST1H2AC	K11251
ENSG00000179598	-0.318122	3.883796	0.64768	1	PLD6	K16862
ENSG00000088812	0.307539	5.08144	0.647809	1	ATRN	_
ENSG00000171604	0.300828	5.968648	0.647835	1	CXXC5	_
ENSG00000225373	0.451738	1.164874	0.647858	1	WASH5P	K18461
ENSG00000259772	0.451738	1.164874	0.647858	1	RP11-16E12.2	_
ENSG00000268218	0.451738	1.164874	0.647858	1	AC137932.1	K17985
ENSG00000242114	0.322364	3.455524	0.647894	1	MTFP1	K17981
ENSG00000011376	-0.307434	5.221849	0.647979	1	LARS2	K01869
ENSG00000163382	-0.300629	6.921919	0.648176	1	APOA1BP	K17759
ENSG00000166908	0.307023	4.952645	0.648197	1	PIP4K2C	K00920
ENSG00000142731	-0.305957	4.958642	0.648197	1	PLK4	K08863
ENSG00000085644	0.324162	2.847866	0.648233	1	ZNF213	K09229

ENSG00000143436	-0.300996	6.660933	0.648266	1	MRPL9	K02939
ENSG00000144848	-0.301048	6.592693	0.648353	1	ATG3	K08343
ENSG00000187837	0.303241	5.525592	0.648384	1	HIST1H1C	K11275
ENSG00000101945	0.364293	2.233701	0.648527	1	SUV39H1	K11419
ENSG00000145075	0.37722	1.802899	0.648562	1	CCDC39	_
ENSG00000172171	-0.315012	4.482373	0.648697	1	TEFM	K17658
ENSG00000101447	0.300513	6.564961	0.648722	1	FAM83D	K16805
ENSG00000152422	-0.306542	4.75311	0.648744	1	XRCC4	K10886
ENSG00000104356	-0.302935	5.450377	0.649115	1	POP1	K01164
ENSG00000130177	-0.300122	6.701057	0.649139	1	CDC16	K03353
ENSG00000241553	-0.300266	5.898019	0.649154	1	ARPC4	K05755
ENSG00000168490	0.311905	3.663943	0.649247	1	PHYHIP	_
ENSG00000172456	-0.327489	3.025077	0.649275	1	FGGY	_
ENSG00000111802	-0.301863	6.317571	0.649291	1	TDP2	K19619
ENSG00000105426	0.299547	6.788791	0.649362	1	PTPRS	K06778
ENSG00000251022	-0.299945	6.683695	0.649368	1	THAP9-AS1	_
ENSG00000198042	-0.306234	5.093035	0.649463	1	MAK16	K14831
ENSG00000198937	0.314864	3.885018	0.649564	1	CCDC167	_
ENSG00000183579	0.312457	4.17967	0.649607	1	ZNRF3	K16273
ENSG00000168038	-0.329859	2.785354	0.649611	1	ULK4	K17545
ENSG00000169598	-0.329859	2.785354	0.649611	1	DFFB	K02311
ENSG00000131238	-0.298392	7.666687	0.649625	1	PPT1	K01074
ENSG00000171475	0.301826	5.258228	0.649673	1	WIPF2	K19475
ENSG00000123427	0.317529	3.351977	0.649763	1	METTL21B	K04460
ENSG00000140320	0.301841	5.543593	0.649981	1	BAHD1	_
ENSG00000263753	-0.318471	3.97578	0.650021	1	LINC00667	_
ENSG00000180806	0.3162	3.968698	0.650021	1	HOXC9	K09294
ENSG00000166582	-0.300858	6.055786	0.650057	1	CENPV	_
ENSG00000168758	0.301814	5.055277	0.650088	1	SEMA4C	K06521
ENSG00000141622	0.338043	3.158713	0.650114	1	RNF165	K17821
ENSG00000109046	0.29887	6.743193	0.650183	1	WSB1	K10341
ENSG00000104856	-0.311995	4.541859	0.650241	1	RELB	K09253
ENSG00000174233	0.298873	6.017594	0.650283	1	ADCY6	K08046
ENSG00000126821	0.298783	5.717245	0.65035	1	SGPP1	K04716
ENSG00000130005	-0.299415	6.44016	0.650484	1	GAMT	K00542
ENSG00000181450	-0.33507	2.953471	0.65056	1	ZNF678	K09228
ENSG00000214389	-0.30115	5.296177	0.650569	1	RPS3AP26	K02984
ENSG00000155367	-0.357155	2.148089	0.650615	1	PPM1J	K17504
ENSG00000154309	-0.307861	4.376643	0.650635	1	DISP1	_
ENSG00000183780	-0.313017	3.580972	0.650944	1	SLC35F3	K15288
ENSG00000111269	0.297667	6.112044	0.651162	1	CREBL2	_
ENSG00000137804	0.29665	7.866819	0.651234	1	NUSAP1	_
ENSG00000157483	0.296829	7.576904	0.651298	1	MYO1E	K10356
ENSG00000102890	-0.374879	1.670799	0.651311	1	ELMO3	K19241
ENSG00000146094	-0.374879	1.670799	0.651311	1	DOK3	_
ENSG00000070404	0.295869	9.382116	0.651327	1	FSTL3	K09171
ENSG00000109654	-0.310973	4.093299	0.651436	1	TRIM2	K11997
ENSG00000111785	-0.306768	4.54746	0.651437	1	RIC8B	_
ENSG00000148843	-0.297832	6.77411	0.651502	1	PDCD11	K14792
ENSG00000161860	0.451198	0.864912	0.651531	1	SYCE2	K19535
ENSG00000224424	0.451198	0.864912	0.651531	1	PRKAR2A-AS1	_
ENSG00000232859	0.451198	0.864912	0.651531	1	LYRM9	_
ENSG00000164989	-0.418086	0.950703	0.651531	1	CCDC171	_
ENSG00000239523	-0.418086	0.950703	0.651531	1	MYLK-AS1	_
ENSG00000166924	0.392706	0.892827	0.651531	1	NYAP1	_
ENSG00000185924	0.392706	0.892827	0.651531	1	RTN4RL1	K16660
ENSG00000196110	0.392706	0.892827	0.651531	1	ZNF699	K09228
ENSG00000196208	0.392706	0.892827	0.651531	1	GREB1	_

ENSG00000214331	0.392706	0.892827	0.651531	1	RP11-252A24.2	K17509
ENSG00000244932	0.392706	0.892827	0.651531	1	RP11-529F4.1	K19656
ENSG00000267161	0.392706	0.892827	0.651531	1	AC005943.5	_
ENSG00000110756	-0.301541	5.554692	0.651775	1	HPS5	_
ENSG00000173011	-0.310671	3.984025	0.651784	1	TADA2B	K15127
ENSG00000169925	0.30387	4.969572	0.651786	1	BRD3	K11721
ENSG00000128159	0.302986	4.760294	0.651845	1	TUBGCP6	K16573
ENSG00000114933	-0.317537	3.532633	0.651876	1	INO80D	K11668
ENSG00000075188	-0.298762	6.283127	0.651907	1	NUP37	K14302
ENSG00000119661	-0.320952	3.248992	0.65193	1	DNAL1	K10411
ENSG00000196152	0.332949	2.376617	0.651939	1	ZNF79	K09228
ENSG00000111602	-0.29782	6.547067	0.651982	1	TIMELESS	K03155
ENSG00000165501	-0.299972	5.223012	0.652024	1	LRR1	K10348
ENSG00000180488	-0.30562	4.810426	0.652031	1	FAM73A	K17985
ENSG00000173598	-0.303341	5.050049	0.652104	1	NUDT4	K07766
ENSG00000176597	-0.308575	4.442597	0.652207	1	B3GNT5	_
ENSG00000197062	-0.305232	4.296409	0.652289	1	RP5-874C20.3	K09230
ENSG00000156026	0.297186	6.588414	0.652316	1	MCU	_
ENSG00000118454	0.298302	6.177824	0.652411	1	ANKRD13C	_
ENSG00000163626	-0.321908	3.046334	0.652626	1	COX18	K17797
ENSG00000104859	0.29795	5.377116	0.652631	1	CLASRP	K13168
ENSG00000138413	-0.295762	7.466814	0.652729	1	IDH1	K00031
ENSG00000133398	-0.297051	6.582232	0.652747	1	MED10	K15151
ENSG00000165685	-0.30188	4.877511	0.652763	1	TMEM52B	_
ENSG00000070961	-0.29579	7.412065	0.652764	1	ATP2B1	K05850
ENSG00000135722	0.350216	2.594815	0.652863	1	FBXL8	K10274
ENSG00000273131	0.350216	2.594815	0.652863	1	RP6-42F4.1	_
ENSG00000127507	0.319205	3.471493	0.652871	1	EMR2	K08443
ENSG00000160293	0.299374	5.70467	0.652915	1	VAV2	K05730
ENSG00000180035	-0.313915	3.826217	0.65299	1	ZNF48	K09228
ENSG00000143776	-0.300207	5.290592	0.652996	1	CDC42BPA	K16307
ENSG00000128951	-0.295422	7.555626	0.653002	1	DUT	K01520
ENSG00000063978	0.296552	6.40676	0.653053	1	RNF4	_
ENSG00000115318	-0.375164	2.013778	0.653067	1	LOXL3	K00280
ENSG00000133627	-0.318314	3.182886	0.653149	1	ACTR3B	K18584
ENSG00000160813	-0.299072	5.549091	0.653213	1	PPP1R35	K17574
ENSG00000126890	-0.295505	7.277963	0.653247	1	CTAG2	_
ENSG00000169410	-0.300078	5.383752	0.6533	1	PTPN9	K18038
ENSG00000149115	0.295575	6.996669	0.653383	1	TNKS1BP1	_
ENSG00000143811	-0.299625	5.340088	0.653497	1	PYCR2	K00286
ENSG00000184922	0.295344	6.304899	0.653743	1	FMNL1	_
ENSG00000179967	-0.302226	5.13102	0.653983	1	PPP1R14BP3	K17555
ENSG00000118046	0.314091	2.959451	0.654062	1	STK11	K07298
ENSG00000011465	-0.297938	5.654583	0.654089	1	DCN	K04660
ENSG00000169230	-0.29483	7.202961	0.654092	1	PRELID1	_
ENSG00000242110	-0.358889	2.280688	0.654113	1	AMACR	K01796
ENSG00000138381	-0.302058	4.864039	0.654122	1	ASNSD1	_
ENSG00000173542	-0.307943	4.478405	0.654189	1	MOB1B	K06685
ENSG00000100764	-0.298737	5.501545	0.654267	1	PSMC1	K03062
ENSG00000163947	0.301792	4.748912	0.654333	1	ARHGEF3	_
ENSG00000032742	-0.333771	3.124098	0.654472	1	IFT88	K16474
ENSG00000170340	-0.301094	5.218315	0.654584	1	B3GNT2	K00741
ENSG00000164823	-0.295629	6.456222	0.654589	1	OSGIN2	_
ENSG00000180447	-0.299457	5.515017	0.654605	1	GAS1	K06232
ENSG00000140521	0.297496	6.055061	0.65461	1	POLG	K02332
ENSG00000178381	0.300219	4.391222	0.654615	1	ZFAND2A	_
ENSG00000203497	-0.374666	1.46258	0.654708	1	PDCD4-AS1	_
ENSG00000238261	-0.374666	1.46258	0.654708	1	RP11-435B5.5	_

ENSG00000241288	-0.374666	1.46258	0.654708	1	RP11-379B18.5	_
ENSG00000247317	-0.374666	1.46258	0.654708	1	RP11-273G15.2	_
ENSG00000117899	-0.296475	6.140183	0.654989	1	MESDC2	_
ENSG00000078902	0.30832	4.300489	0.655095	1	TOLLIP	K05402
ENSG00000153147	-0.294067	7.087918	0.655098	1	SMARCA5	K11654
ENSG00000130304	0.310334	3.908649	0.655102	1	SLC27A1	K08745
ENSG00000005961	0.326481	2.707186	0.655284	1	ITGA2B	K06476
ENSG00000080200	0.326481	2.707186	0.655284	1	CRYBG3	_
ENSG00000117877	-0.318387	3.498154	0.655309	1	CD3EAP	_
ENSG00000146826	-0.318387	3.498154	0.655309	1	C7orf43	_
ENSG00000174953	-0.296303	6.097328	0.655506	1	DHX36	K14442
ENSG00000122783	-0.303184	4.146143	0.655524	1	C7orf49	_
ENSG00000137962	-0.298026	5.720025	0.655566	1	ARHGAP29	K12581
ENSG00000148773	0.293056	7.451526	0.655574	1	MKI67	K17582
ENSG00000167081	0.306993	4.208502	0.655638	1	PBX3	K15610
ENSG00000140563	0.293712	5.984675	0.655747	1	MCTP2	_
ENSG00000181192	-0.294038	6.7015	0.655803	1	DHTKD1	K15791
ENSG00000106723	-0.294287	6.566155	0.655812	1	SPIN1	_
ENSG00000108278	-0.397349	1.865713	0.655991	1	ZNHIT3	K02927
ENSG00000078487	-0.375039	1.852531	0.655991	1	ZCWPW1	_
ENSG00000136231	0.297493	5.696223	0.656061	1	IGF2BP3	K13197
ENSG00000111432	0.301708	3.954774	0.656064	1	FZD10	K02842
ENSG00000085117	0.303193	4.50773	0.656076	1	CD82	K06509
ENSG00000100014	0.303193	4.50773	0.656076	1	SPECC1L	_
ENSG00000103021	-0.313445	3.201967	0.656133	1	CCDC113	K17985
ENSG00000004487	0.292372	7.591528	0.656182	1	KDM1A	K11450
ENSG00000139178	0.293404	6.177812	0.65621	1	C1RL	_
ENSG00000079385	0.487727	0.573765	0.656347	1	CEACAM1	K06499
ENSG00000129991	0.487727	0.573765	0.656347	1	TNNI3	K12044
ENSG00000166922	0.487727	0.573765	0.656347	1	SCG5	_
ENSG00000167968	0.487727	0.573765	0.656347	1	DNASE1L2	K11995
ENSG00000106484	-0.43167	0.587795	0.656347	1	MEST	_
ENSG00000228696	-0.43167	0.587795	0.656347	1	ARL17B	K06883
ENSG00000238278	-0.43167	0.587795	0.656347	1	ALG1L6P	K03842
ENSG00000114054	-0.294942	6.189027	0.65641	1	PCCB	K01966
ENSG00000112130	-0.295375	5.847959	0.656483	1	RNF8	K10667
ENSG00000067704	-0.291678	8.258752	0.656507	1	IARS2	K01870
ENSG00000167920	-0.300621	4.49205	0.656653	1	TMEM99	_
ENSG00000069998	-0.295777	5.538765	0.65673	1	CECR5	_
ENSG00000185432	0.297188	5.381337	0.656924	1	METTL7A	K15696
ENSG00000131242	0.344855	2.401261	0.657004	1	RAB11FIP4	K12485
ENSG00000152942	0.344855	2.401261	0.657004	1	RAD17	_
ENSG00000089195	-0.298703	5.035421	0.657064	1	TRMT6	K03256
ENSG00000171621	0.309052	4.145378	0.657071	1	SPSB1	K10343
ENSG00000091527	-0.291637	7.625398	0.657088	1	CDV3	_
ENSG00000177302	0.29575	5.724736	0.657137	1	TOP3A	K03165
ENSG00000127125	-0.298308	5.178954	0.657147	1	PPCS	K01922
ENSG00000107758	-0.30274	4.466533	0.65725	1	PPP3CB	K04348
ENSG00000162695	-0.296125	5.400438	0.657261	1	SLC30A7	K14692
ENSG00000165283	-0.292068	7.082443	0.657304	1	STOML2	_
ENSG00000132170	-0.300786	4.856494	0.65742	1	PPARG	K08530
ENSG00000105983	-0.295649	5.231192	0.657542	1	LMBR1	K09228
ENSG00000142867	-0.295557	5.357283	0.657576	1	BCL10	K07368
ENSG00000248008	-0.324526	3.271937	0.657634	1	DYNLL1-AS1	_
ENSG00000100523	-0.316536	3.710523	0.657766	1	DDHD1	K13619
ENSG00000147654	-0.298216	4.380787	0.657863	1	EBAG9	_
ENSG00000164258	-0.296491	5.460358	0.657914	1	NDUFS4	K03937
ENSG00000188186	-0.293008	6.072169	0.657922	1	LAMTOR4	_

ENSG00000116455	-0.291822	6.415255	0.657924	1	WDR77		K13221
ENSG00000006625	-0.291812	6.754391	0.658146	1	GGCT		K00682
ENSG00000166532	0.290755	7.314219	0.658261	1	RIMKLB		K18310
ENSG00000114999	-0.291963	6.604024	0.658282	1	TTL		K06047
ENSG00000135776	-0.298425	5.168032	0.658289	1	ABCB10		K05657
ENSG00000019582	1.8725	-1.801828	0.658385	1	CD74		K06505
ENSG00000054356	1.8725	-1.801828	0.658385	1	PTPRN		K07817
ENSG00000071246	1.8725	-1.801828	0.658385	1	VASH1		_
ENSG00000100167	1.8725	-1.801828	0.658385	1		3-Sep	K16938
ENSG00000102317	1.8725	-1.801828	0.658385	1	RBM3		K13186
ENSG00000103472	1.8725	-1.801828	0.658385	1	RRN3P2		K15216
ENSG00000105549	1.8725	-1.801828	0.658385	1	THEG		_
ENSG00000106819	1.8725	-1.801828	0.658385	1	ASPN		K08120
ENSG00000110852	1.8725	-1.801828	0.658385	1	CLEC2B		K10071
ENSG00000111834	1.8725	-1.801828	0.658385	1	RSPH4A		K19756
ENSG00000114124	1.8725	-1.801828	0.658385	1	GRK7		K00909
ENSG00000117152	1.8725	-1.801828	0.658385	1	RGS4		K16449
ENSG00000117971	1.8725	-1.801828	0.658385	1	CHRNB4		K04815
ENSG00000124429	1.8725	-1.801828	0.658385	1	POF1B		_
ENSG00000124529	1.8725	-1.801828	0.658385	1	HIST1H4B		K11254
ENSG00000126860	1.8725	-1.801828	0.658385	1	EVI2A		K16854
ENSG00000128268	1.8725	-1.801828	0.658385	1	MGAT3		K00737
ENSG00000128271	1.8725	-1.801828	0.658385	1	ADORA2A		K04266
ENSG00000130528	1.8725	-1.801828	0.658385	1	HRC		_
ENSG00000134516	1.8725	-1.801828	0.658385	1	DOCK2		K12367
ENSG00000139351	1.8725	-1.801828	0.658385	1	SYCP3		K19528
ENSG00000140481	1.8725	-1.801828	0.658385	1	CCDC33		_
ENSG00000140832	1.8725	-1.801828	0.658385	1	MARVELD3		_
ENSG00000144550	1.8725	-1.801828	0.658385	1	CPNE9		_
ENSG00000146013	1.8725	-1.801828	0.658385	1	GFRA3		_
ENSG00000152487	1.8725	-1.801828	0.658385	1	ARL5B-AS1		_
ENSG00000154330	1.8725	-1.801828	0.658385	1	PGM5		K15636
ENSG00000154451	1.8725	-1.801828	0.658385	1	GBP5		_
ENSG00000157152	1.8725	-1.801828	0.658385	1	SYN2		_
ENSG00000158869	1.8725	-1.801828	0.658385	1	FCER1G		K07983
ENSG00000159197	1.8725	-1.801828	0.658385	1	KCNE2		K04896
ENSG00000159208	1.8725	-1.801828	0.658385	1	C1orf51		K02927
ENSG00000159625	1.8725	-1.801828	0.658385	1	CCDC135		K18643
ENSG00000162188	1.8725	-1.801828	0.658385	1	GNG3		K04540
ENSG00000162595	1.8725	-1.801828	0.658385	1	DIRAS3		K07842
ENSG00000165076	1.8725	-1.801828	0.658385	1	PRSS37		_
ENSG00000166323	1.8725	-1.801828	0.658385	1	C11orf65		_
ENSG00000166923	1.8725	-1.801828	0.658385	1	GREM1		_
ENSG00000170166	1.8725	-1.801828	0.658385	1	HOXD4		K09304
ENSG00000170262	1.8725	-1.801828	0.658385	1	MRAP		_
ENSG00000170419	1.8725	-1.801828	0.658385	1	VSTM2A		_
ENSG00000171657	1.8725	-1.801828	0.658385	1	GPR82		K08420
ENSG00000178660	1.8725	-1.801828	0.658385	1	ARMC10P1		_
ENSG00000179256	1.8725	-1.801828	0.658385	1	SMCO3		_
ENSG00000179403	1.8725	-1.801828	0.658385	1	VWA1		_
ENSG00000180537	1.8725	-1.801828	0.658385	1	RNF182		K11983
ENSG00000180846	1.8725	-1.801828	0.658385	1	CSNK1G2-AS1		_
ENSG00000182162	1.8725	-1.801828	0.658385	1	P2RY8		K08386
ENSG00000182676	1.8725	-1.801828	0.658385	1	PPP1R27		K17566
ENSG00000182866	1.8725	-1.801828	0.658385	1	LCK		K05856
ENSG00000182872	1.8725	-1.801828	0.658385	1	RBM10		K13094
ENSG00000184908	1.8725	-1.801828	0.658385	1	CLCNKB		K05018
ENSG00000188293	1.8725	-1.801828	0.658385	1	IGFL1		_

ENSG00000188981	1.8725	-1.801828	0.658385	1	MSANTD1	_
ENSG00000196132	1.8725	-1.801828	0.658385	1	MYT1	_
ENSG00000197361	1.8725	-1.801828	0.658385	1	FBXL22	K10288
ENSG00000197753	1.8725	-1.801828	0.658385	1	LHFPL5	_
ENSG00000199072	1.8725	-1.801828	0.658385	1	MIRLET7F1	_
ENSG00000200394	1.8725	-1.801828	0.658385	1	SNORA38B	_
ENSG00000200677	1.8725	-1.801828	0.658385	1	SNORD18	_
ENSG00000201121	1.8725	-1.801828	0.658385	1	Y_RNA	_
ENSG00000201264	1.8725	-1.801828	0.658385	1	SNORD73	_
ENSG00000203499	1.8725	-1.801828	0.658385	1	FAM83H-AS1	_
ENSG00000203843	1.8725	-1.801828	0.658385	1	PFN1P2	K05759
ENSG00000204323	1.8725	-1.801828	0.658385	1	SMIM5	_
ENSG00000204581	1.8725	-1.801828	0.658385	1	AC096670.3	_
ENSG00000205622	1.8725	-1.801828	0.658385	1	AF064858.6	_
ENSG00000206044	1.8725	-1.801828	0.658385	1	AC005841.1	_
ENSG00000207110	1.8725	-1.801828	0.658385	1	RNU1-106P	_
ENSG00000207119	1.8725	-1.801828	0.658385	1	U3	_
ENSG00000207234	1.8725	-1.801828	0.658385	1	RNU6-125P	_
ENSG00000207980	1.8725	-1.801828	0.658385	1	MIR23A	_
ENSG00000212327	1.8725	-1.801828	0.658385	1	RNU6-882P	_
ENSG00000212493	1.8725	-1.801828	0.658385	1	SNORD19	_
ENSG00000213260	1.8725	-1.801828	0.658385	1	YWHAZP5	K16197
ENSG00000213279	1.8725	-1.801828	0.658385	1	RP1-29C18.9	_
ENSG00000213612	1.8725	-1.801828	0.658385	1	FAM220CP	_
ENSG00000213939	1.8725	-1.801828	0.658385	1	RP11-314A20.1	K02951
ENSG00000214425	1.8725	-1.801828	0.658385	1	LRRC37A4P	K09228
ENSG00000215156	1.8725	-1.801828	0.658385	1	RP11-1023L17.2	_
ENSG00000221051	1.8725	-1.801828	0.658385	1	AC118344.1	_
ENSG00000223442	1.8725	-1.801828	0.658385	1	AC004041.2	_
ENSG00000223552	1.8725	-1.801828	0.658385	1	RP11-24F11.2	_
ENSG00000223635	1.8725	-1.801828	0.658385	1	RP4-613A2.1	_
ENSG00000223734	1.8725	-1.801828	0.658385	1	RP11-644K8.1	_
ENSG00000223849	1.8725	-1.801828	0.658385	1	RP11-80I15.1	K02976
ENSG00000223922	1.8725	-1.801828	0.658385	1	ASS1P2	K01940
ENSG00000224993	1.8725	-1.801828	0.658385	1	RPL29P12	K02905
ENSG00000226065	1.8725	-1.801828	0.658385	1	ZBTB45P2	K10516
ENSG00000226155	1.8725	-1.801828	0.658385	1	AC124944.3	_
ENSG00000226306	1.8725	-1.801828	0.658385	1	NPY6R	K04208
ENSG00000226352	1.8725	-1.801828	0.658385	1	RP11-523H24.3	_
ENSG00000226771	1.8725	-1.801828	0.658385	1	AP001136.2	_
ENSG00000226874	1.8725	-1.801828	0.658385	1	AC005154.8	K04969
ENSG00000228056	1.8725	-1.801828	0.658385	1	CFL1P3	K05765
ENSG00000228158	1.8725	-1.801828	0.658385	1	TLE1P1	_
ENSG00000228719	1.8725	-1.801828	0.658385	1	RP5-1119A7.14	_
ENSG00000231393	1.8725	-1.801828	0.658385	1	RP11-331F9.3	_
ENSG00000231441	1.8725	-1.801828	0.658385	1	RP11-472M19.2	_
ENSG00000231966	1.8725	-1.801828	0.658385	1	RP11-12M5.4	_
ENSG00000232034	1.8725	-1.801828	0.658385	1	AC092168.2	_
ENSG00000232387	1.8725	-1.801828	0.658385	1	SKA2P1	K10608
ENSG00000232874	1.8725	-1.801828	0.658385	1	RP11-135A1.2	_
ENSG00000234380	1.8725	-1.801828	0.658385	1	AP000330.8	_
ENSG00000234537	1.8725	-1.801828	0.658385	1	RP11-100G15.7	K18036
ENSG00000235066	1.8725	-1.801828	0.658385	1	AC009303.1	_
ENSG00000235070	1.8725	-1.801828	0.658385	1	AC068138.1	_
ENSG00000235138	1.8725	-1.801828	0.658385	1	RP11-374P20.4	_
ENSG00000235545	1.8725	-1.801828	0.658385	1	RP11-230B22.1	_
ENSG00000235660	1.8725	-1.801828	0.658385	1	LINC00345	_
ENSG00000235878	1.8725	-1.801828	0.658385	1	AP001468.1	_

ENSG00000236095	1.8725	-1.801828	0.658385	1	RP11-49O14.2	_
ENSG00000236383	1.8725	-1.801828	0.658385	1	LINC00854	_
ENSG00000236397	1.8725	-1.801828	0.658385	1	DDX11L2	K11273
ENSG00000236833	1.8725	-1.801828	0.658385	1	AC024560.2	_
ENSG00000237301	1.8725	-1.801828	0.658385	1	RP4-680D5.2	_
ENSG00000237575	1.8725	-1.801828	0.658385	1	PYY2	K05233
ENSG00000237742	1.8725	-1.801828	0.658385	1	RP11-624M8.1	_
ENSG00000237819	1.8725	-1.801828	0.658385	1	AC002454.1	_
ENSG00000237828	1.8725	-1.801828	0.658385	1	PA2G4P1	_
ENSG00000237945	1.8725	-1.801828	0.658385	1	LINC00649	_
ENSG00000240905	1.8725	-1.801828	0.658385	1	RN7SL798P	_
ENSG00000241322	1.8725	-1.801828	0.658385	1	CDRT1	K10266
ENSG00000241527	1.8725	-1.801828	0.658385	1	CA15P1	K01672
ENSG00000243243	1.8725	-1.801828	0.658385	1	AC073130.3	_
ENSG00000247372	1.8725	-1.801828	0.658385	1	CTD-2235C13.2	_
ENSG00000247934	1.8725	-1.801828	0.658385	1	RP11-967K21.1	_
ENSG00000248213	1.8725	-1.801828	0.658385	1	CICP16	K16941
ENSG00000248923	1.8725	-1.801828	0.658385	1	MTND5P11	K03883
ENSG00000249141	1.8725	-1.801828	0.658385	1	RP11-514O12.4	K01166
ENSG00000250299	1.8725	-1.801828	0.658385	1	MRPS31P4	K17410
ENSG00000250432	1.8725	-1.801828	0.658385	1	RP11-834C11.5	_
ENSG00000250569	1.8725	-1.801828	0.658385	1	NTAN1P2	K14662
ENSG00000250816	1.8725	-1.801828	0.658385	1	DCAF13P2	K11806
ENSG00000251775	1.8725	-1.801828	0.658385	1	ACA59	_
ENSG00000252311	1.8725	-1.801828	0.658385	1	RNU1-103P	_
ENSG00000252503	1.8725	-1.801828	0.658385	1	RNU6-531P	_
ENSG00000252641	1.8725	-1.801828	0.658385	1	RNU6-678P	_
ENSG00000253047	1.8725	-1.801828	0.658385	1	SNORA40	_
ENSG00000253121	1.8725	-1.801828	0.658385	1	RP11-659E9.4	_
ENSG00000253892	1.8725	-1.801828	0.658385	1	RP11-770E5.2	_
ENSG00000253908	1.8725	-1.801828	0.658385	1	RP11-252I14.2	_
ENSG00000254911	1.8725	-1.801828	0.658385	1	SCARNA9	_
ENSG00000255185	1.8725	-1.801828	0.658385	1	PDXDC2P	_
ENSG00000255507	1.8725	-1.801828	0.658385	1	RP11-535A19.2	_
ENSG00000255509	1.8725	-1.801828	0.658385	1	RP11-445F12.1	_
ENSG00000256360	1.8725	-1.801828	0.658385	1	RP11-707G14.7	_
ENSG00000256897	1.8725	-1.801828	0.658385	1	RP11-17G12.2	_
ENSG00000257058	1.8725	-1.801828	0.658385	1	RP11-864I4.4	_
ENSG00000257150	1.8725	-1.801828	0.658385	1	PGAM1P5	K01834
ENSG00000257221	1.8725	-1.801828	0.658385	1	RP11-689B22.2	_
ENSG00000257527	1.8725	-1.801828	0.658385	1	MIR3179-3	_
ENSG00000257771	1.8725	-1.801828	0.658385	1	RP11-70F11.8	_
ENSG00000258376	1.8725	-1.801828	0.658385	1	RP4-647C14.2	_
ENSG00000259191	1.8725	-1.801828	0.658385	1	RP11-253M7.3	K01557
ENSG00000259209	1.8725	-1.801828	0.658385	1	RP5-991G20.2	_
ENSG00000259294	1.8725	-1.801828	0.658385	1	CTA-339C12.1	_
ENSG00000259323	1.8725	-1.801828	0.658385	1	RP13-608F4.5	K01528
ENSG00000259345	1.8725	-1.801828	0.658385	1	RP11-624L4.1	_
ENSG00000259581	1.8725	-1.801828	0.658385	1	TYRO3P	K05116
ENSG00000259618	1.8725	-1.801828	0.658385	1	RP11-562A8.5	_
ENSG00000259804	1.8725	-1.801828	0.658385	1	CTD-2012K14.7	_
ENSG00000259915	1.8725	-1.801828	0.658385	1	RP11-410E4.1	_
ENSG00000260167	1.8725	-1.801828	0.658385	1	RP11-146F11.5	_
ENSG00000260470	1.8725	-1.801828	0.658385	1	RP11-834C11.11	_
ENSG00000260711	1.8725	-1.801828	0.658385	1	RP11-747H7.3	_
ENSG00000261140	1.8725	-1.801828	0.658385	1	RP11-20I23.6	_
ENSG00000261172	1.8725	-1.801828	0.658385	1	RP11-356C4.5	_
ENSG00000261312	1.8725	-1.801828	0.658385	1	AC002550.5	_

ENSG00000261420	1.8725	-1.801828	0.658385	1	RP1-168L15.5	_
ENSG00000262837	1.8725	-1.801828	0.658385	1	RP11-304F15.7	_
ENSG00000263063	1.8725	-1.801828	0.658385	1	RP11-388C12.1	_
ENSG00000263400	1.8725	-1.801828	0.658385	1	CTC-297N7.5	_
ENSG00000264237	1.8725	-1.801828	0.658385	1	AL137846.1	_
ENSG00000265369	1.8725	-1.801828	0.658385	1	U3	_
ENSG00000265407	1.8725	-1.801828	0.658385	1	MIR4324	_
ENSG00000265802	1.8725	-1.801828	0.658385	1	RN7SL49P	_
ENSG00000266232	1.8725	-1.801828	0.658385	1	MIR3178	_
ENSG00000266924	1.8725	-1.801828	0.658385	1	RP11-154H12.2	_
ENSG00000267003	1.8725	-1.801828	0.658385	1	CTC-507E2.1	_
ENSG00000267159	1.8725	-1.801828	0.658385	1	AC005391.2	_
ENSG00000267425	1.8725	-1.801828	0.658385	1	RP11-128P17.1	_
ENSG00000267481	1.8725	-1.801828	0.658385	1	CTC-559E9.5	_
ENSG00000267706	1.8725	-1.801828	0.658385	1	CTD-2105E13.6	K02267
ENSG00000267848	1.8725	-1.801828	0.658385	1	AL118506.1	_
ENSG00000268536	1.8725	-1.801828	0.658385	1	AC005523.3	_
ENSG00000268650	1.8725	-1.801828	0.658385	1	AC068499.10	_
ENSG00000269305	1.8725	-1.801828	0.658385	1	AL158147.2	_
ENSG00000269358	1.8725	-1.801828	0.658385	1	AC138393.1	K11447
ENSG00000269591	1.8725	-1.801828	0.658385	1	AL354808.2	_
ENSG00000269859	1.8725	-1.801828	0.658385	1	CTD-2537I9.16	_
ENSG00000270107	1.8725	-1.801828	0.658385	1	CTD-2306M10.1	_
ENSG00000270147	1.8725	-1.801828	0.658385	1	RP11-646I6.6	_
ENSG00000270194	1.8725	-1.801828	0.658385	1	RP11-259K5.2	_
ENSG00000270426	1.8725	-1.801828	0.658385	1	RP11-326I11.5	_
ENSG00000270532	1.8725	-1.801828	0.658385	1	PEBP1P2	_
ENSG00000270903	1.8725	-1.801828	0.658385	1	HNRNPA3P9	K12741
ENSG00000270956	1.8725	-1.801828	0.658385	1	RP11-65L3.4	_
ENSG00000271064	1.8725	-1.801828	0.658385	1	RP11-792A8.3	_
ENSG00000271367	1.8725	-1.801828	0.658385	1	RP3-483K16.4	_
ENSG00000271424	1.8725	-1.801828	0.658385	1	RP11-647O20.1	_
ENSG00000271555	1.8725	-1.801828	0.658385	1	CTD-3214K23.1	_
ENSG00000271723	1.8725	-1.801828	0.658385	1	MROH7-TTC4	K01881
ENSG00000271869	1.8725	-1.801828	0.658385	1	RP11-51J9.5	_
ENSG00000271880	1.8725	-1.801828	0.658385	1	RP11-96C23.5	_
ENSG00000272434	1.8725	-1.801828	0.658385	1	RP13-131K19.6	_
ENSG00000272597	1.8725	-1.801828	0.658385	1	RP11-446H18.6	_
ENSG00000272915	1.8725	-1.801828	0.658385	1	RP11-62J1.4	_
ENSG00000272949	1.8725	-1.801828	0.658385	1	RP11-514P8.8	_
ENSG00000273129	1.8725	-1.801828	0.658385	1	RP5-973M2.2	_
ENSG00000273320	1.8725	-1.801828	0.658385	1	RP11-22N19.2	_
ENSG00000273368	1.8725	-1.801828	0.658385	1	RP11-376P6.3	_
ENSG00000005844	0.980265	-1.616597	0.658385	1	ITGAL	K05718
ENSG00000053524	0.980265	-1.616597	0.658385	1	MCF2L2	K04372
ENSG00000092529	0.980265	-1.616597	0.658385	1	CAPN3	K08573
ENSG00000099866	0.980265	-1.616597	0.658385	1	MADCAM1	K06779
ENSG00000100055	0.980265	-1.616597	0.658385	1	CYTH4	K18441
ENSG00000105467	0.980265	-1.616597	0.658385	1	SYNGR4	_
ENSG00000106631	0.980265	-1.616597	0.658385	1	MYL7	K12754
ENSG00000109083	0.980265	-1.616597	0.658385	1	IFT20	K16473
ENSG00000121905	0.980265	-1.616597	0.658385	1	HPCA	K19695
ENSG00000125414	0.980265	-1.616597	0.658385	1	MYH2	K10352
ENSG00000125997	0.980265	-1.616597	0.658385	1	BPIFB9P	_
ENSG00000129675	0.980265	-1.616597	0.658385	1	ARHGEF6	K05729
ENSG00000130822	0.980265	-1.616597	0.658385	1	PNCK	K08795
ENSG00000131409	0.980265	-1.616597	0.658385	1	LRRC4B	K16360
ENSG00000137857	0.980265	-1.616597	0.658385	1	DUOX1	K13411

ENSG00000139209	0.980265	-1.616597	0.658385	1	SLC38A4	K14991
ENSG00000139988	0.980265	-1.616597	0.658385	1	RDH12	K11153
ENSG00000142303	0.980265	-1.616597	0.658385	1	ADAMTS10	K08625
ENSG00000142549	0.980265	-1.616597	0.658385	1	IGLON5	K06773
ENSG00000144063	0.980265	-1.616597	0.658385	1	MALL	_
ENSG00000145113	0.980265	-1.616597	0.658385	1	MUC4	_
ENSG00000148832	0.980265	-1.616597	0.658385	1	PAOX	K00308
ENSG00000155269	0.980265	-1.616597	0.658385	1	GPR78	_
ENSG00000157017	0.980265	-1.616597	0.658385	1	GHRL	K05254
ENSG00000159239	0.980265	-1.616597	0.658385	1	C2orf81	_
ENSG00000159496	0.980265	-1.616597	0.658385	1	RGL4	K08732
ENSG00000159860	0.980265	-1.616597	0.658385	1	FAM115D	_
ENSG00000162510	0.980265	-1.616597	0.658385	1	MATN1	_
ENSG00000164188	0.980265	-1.616597	0.658385	1	RANBP3L	K15304
ENSG00000165171	0.980265	-1.616597	0.658385	1	WBSCR27	_
ENSG00000165807	0.980265	-1.616597	0.658385	1	PPP1R36	K17575
ENSG00000167046	0.980265	-1.616597	0.658385	1	RP11-93B14.6	_
ENSG00000167261	0.980265	-1.616597	0.658385	1	DPEP2	K01273
ENSG00000168675	0.980265	-1.616597	0.658385	1	LDLRAD4	_
ENSG00000171817	0.980265	-1.616597	0.658385	1	ZNF540	K09228
ENSG00000172264	0.980265	-1.616597	0.658385	1	MACROD2	_
ENSG00000172575	0.980265	-1.616597	0.658385	1	RASGRP1	K04350
ENSG00000174428	0.980265	-1.616597	0.658385	1	GTF2IRD2B	K03121
ENSG00000174595	0.980265	-1.616597	0.658385	1	KLF14	K09208
ENSG00000174788	0.980265	-1.616597	0.658385	1	PCP2	_
ENSG00000175262	0.980265	-1.616597	0.658385	1	C1orf127	_
ENSG00000177694	0.980265	-1.616597	0.658385	1	NAALADL2	K01301
ENSG00000178776	0.980265	-1.616597	0.658385	1	C5orf46	_
ENSG00000184083	0.980265	-1.616597	0.658385	1	FAM120C	_
ENSG00000185933	0.980265	-1.616597	0.658385	1	CALHM1	K19738
ENSG00000186354	0.980265	-1.616597	0.658385	1	C9orf47	_
ENSG00000186417	0.980265	-1.616597	0.658385	1	GLDN	K16364
ENSG00000187695	0.980265	-1.616597	0.658385	1	RP11-723O4.6	K15980
ENSG00000187848	0.980265	-1.616597	0.658385	1	P2RX2	K05216
ENSG00000188176	0.980265	-1.616597	0.658385	1	SMTNL2	_
ENSG00000188384	0.980265	-1.616597	0.658385	1	CSPG4P8	K08115
ENSG00000188672	0.980265	-1.616597	0.658385	1	RHCE	K06579
ENSG00000189366	0.980265	-1.616597	0.658385	1	ALG1L	K03842
ENSG00000196507	0.980265	-1.616597	0.658385	1	TCEAL3	_
ENSG00000197238	0.980265	-1.616597	0.658385	1	HIST1H4J	K11253
ENSG00000197847	0.980265	-1.616597	0.658385	1	SLC22A20	K08216
ENSG00000199476	0.980265	-1.616597	0.658385	1	Y_RNA	_
ENSG00000200754	0.980265	-1.616597	0.658385	1	Y_RNA	_
ENSG00000203280	0.980265	-1.616597	0.658385	1	CTA-221G9.11	_
ENSG00000206762	0.980265	-1.616597	0.658385	1	RNU6-418P	_
ENSG00000206992	0.980265	-1.616597	0.658385	1	RNU6-574P	_
ENSG00000211452	0.980265	-1.616597	0.658385	1	DIO1	K01562
ENSG00000212443	0.980265	-1.616597	0.658385	1	SNORA53	_
ENSG00000213077	0.980265	-1.616597	0.658385	1	FAM106A	K14993
ENSG00000213149	0.980265	-1.616597	0.658385	1	CNN2P9	K15411
ENSG00000213290	0.980265	-1.616597	0.658385	1	PGK1P2	K00927
ENSG00000213513	0.980265	-1.616597	0.658385	1	RP13-39P12.2	K00088
ENSG00000213942	0.980265	-1.616597	0.658385	1	RP1-102E24.1	K02910
ENSG00000214353	0.980265	-1.616597	0.658385	1	VAC14-AS1	_
ENSG00000214650	0.980265	-1.616597	0.658385	1	RP11-83B20.1	_
ENSG00000215498	0.980265	-1.616597	0.658385	1	FAM230B	_
ENSG00000220702	0.980265	-1.616597	0.658385	1	RP1-32I10.10	_
ENSG00000221540	0.980265	-1.616597	0.658385	1	MIR1180	_

ENSG00000221946	0.980265	-1.616597	0.658385	1	FXYD7	K13364
ENSG00000222306	0.980265	-1.616597	0.658385	1	Y_RNA	_
ENSG00000223404	0.980265	-1.616597	0.658385	1	LINC00397	_
ENSG00000224383	0.980265	-1.616597	0.658385	1	C17orf72	_
ENSG00000224437	0.980265	-1.616597	0.658385	1	PIGUP1	K05293
ENSG00000224892	0.980265	-1.616597	0.658385	1	RPS4XP16	K02987
ENSG00000224971	0.980265	-1.616597	0.658385	1	SUMO2P3	K12160
ENSG00000225850	0.980265	-1.616597	0.658385	1	RP11-452K12.4	_
ENSG00000226330	0.980265	-1.616597	0.658385	1	RP11-739N20.2	_
ENSG00000227376	0.980265	-1.616597	0.658385	1	FTH1P16	K00522
ENSG00000227470	0.980265	-1.616597	0.658385	1	AC073415.2	_
ENSG00000227495	0.980265	-1.616597	0.658385	1	AC109333.10	_
ENSG00000228446	0.980265	-1.616597	0.658385	1	AC073052.1	_
ENSG00000228463	0.980265	-1.616597	0.658385	1	AP006222.2	_
ENSG00000228570	0.980265	-1.616597	0.658385	1	NUTM2E	_
ENSG00000228792	0.980265	-1.616597	0.658385	1	RP11-354K1.2	_
ENSG00000228917	0.980265	-1.616597	0.658385	1	RP11-544M22.8	_
ENSG00000229487	0.980265	-1.616597	0.658385	1	ALG13-AS1	_
ENSG00000230149	0.980265	-1.616597	0.658385	1	RP3-508I15.19	_
ENSG00000230869	0.980265	-1.616597	0.658385	1	CTGLF10P	K12491
ENSG00000231645	0.980265	-1.616597	0.658385	1	AC025627.9	K07604
ENSG00000231971	0.980265	-1.616597	0.658385	1	RP11-557H15.3	_
ENSG00000232149	0.980265	-1.616597	0.658385	1	FERP1	K08889
ENSG00000232372	0.980265	-1.616597	0.658385	1	RP11-504P24.2	K13051
ENSG00000232491	0.980265	-1.616597	0.658385	1	RP4-756H11.1	_
ENSG00000233469	0.980265	-1.616597	0.658385	1	ST6GALNAC4P1	_
ENSG00000234035	0.980265	-1.616597	0.658385	1	AP001044.2	_
ENSG00000234817	0.980265	-1.616597	0.658385	1	RP3-400B16.1	_
ENSG00000234921	0.980265	-1.616597	0.658385	1	RP11-85O21.5	_
ENSG00000235079	0.980265	-1.616597	0.658385	1	ZRANB2-AS1	_
ENSG00000235852	0.980265	-1.616597	0.658385	1	AC005540.3	_
ENSG00000236975	0.980265	-1.616597	0.658385	1	RP5-1065P14.2	_
ENSG00000236998	0.980265	-1.616597	0.658385	1	RP11-203L2.3	K12398
ENSG00000237903	0.980265	-1.616597	0.658385	1	RP3-404F18.5	_
ENSG00000237950	0.980265	-1.616597	0.658385	1	RP11-7O11.3	_
ENSG00000238386	0.980265	-1.616597	0.658385	1	RNU7-48P	_
ENSG00000238715	0.980265	-1.616597	0.658385	1	snoU13	_
ENSG00000238741	0.980265	-1.616597	0.658385	1	SCARNA7	_
ENSG00000238922	0.980265	-1.616597	0.658385	1	snoU13	_
ENSG00000239335	0.980265	-1.616597	0.658385	1	RP11-745O10.2	_
ENSG00000239470	0.980265	-1.616597	0.658385	1	RP11-16F15.2	K02889
ENSG00000239494	0.980265	-1.616597	0.658385	1	RN7SL333P	_
ENSG00000240005	0.980265	-1.616597	0.658385	1	RP11-293A21.1	_
ENSG00000240449	0.980265	-1.616597	0.658385	1	RP4-584D14.5	_
ENSG00000240591	0.980265	-1.616597	0.658385	1	RP11-247I13.11	_
ENSG00000241313	0.980265	-1.616597	0.658385	1	WWTR1-AS1	_
ENSG00000241975	0.980265	-1.616597	0.658385	1	TCEB1P19	K03872
ENSG00000242325	0.980265	-1.616597	0.658385	1	RPS12P31	_
ENSG00000242775	0.980265	-1.616597	0.658385	1	RP11-875H7.2	_
ENSG00000242791	0.980265	-1.616597	0.658385	1	RP11-651P23.5	_
ENSG00000244515	0.980265	-1.616597	0.658385	1	KRT18P34	K07604
ENSG00000245498	0.980265	-1.616597	0.658385	1	RP11-677M14.7	_
ENSG00000249850	0.980265	-1.616597	0.658385	1	KRT18P31	K07604
ENSG00000250284	0.980265	-1.616597	0.658385	1	CTB-1I21.1	_
ENSG00000250659	0.980265	-1.616597	0.658385	1	RP11-864I4.3	_
ENSG00000250822	0.980265	-1.616597	0.658385	1	CTD-2139B15.4	_
ENSG00000250999	0.980265	-1.616597	0.658385	1	RP11-1379J22.5	_
ENSG00000251013	0.980265	-1.616597	0.658385	1	GAPDHP62	K00134

ENSG00000251459	0.980265	-1.616597	0.658385	1	RP11-319E12.2	_
ENSG00000252071	0.980265	-1.616597	0.658385	1	snoU13	_
ENSG00000253270	0.980265	-1.616597	0.658385	1	RP11-1105O14.1	_
ENSG00000253395	0.980265	-1.616597	0.658385	1	KB-1460A1.1	_
ENSG00000253552	0.980265	-1.616597	0.658385	1	HOXA-AS2	_
ENSG00000253731	0.980265	-1.616597	0.658385	1	PCDHGA6	K16495
ENSG00000254231	0.980265	-1.616597	0.658385	1	CTD-2284J15.1	_
ENSG00000254397	0.980265	-1.616597	0.658385	1	CTD-2371O3.2	_
ENSG00000254449	0.980265	-1.616597	0.658385	1	SF3A3P2	K12827
ENSG00000254459	0.980265	-1.616597	0.658385	1	RP11-91P24.7	_
ENSG00000254593	0.980265	-1.616597	0.658385	1	OR7E126P	K04257
ENSG00000254842	0.980265	-1.616597	0.658385	1	RP11-890B15.2	_
ENSG00000255036	0.980265	-1.616597	0.658385	1	RP11-23J9.4	_
ENSG00000255153	0.980265	-1.616597	0.658385	1	TOLLIP-AS1	_
ENSG00000255471	0.980265	-1.616597	0.658385	1	RP11-736K20.5	_
ENSG00000255989	0.980265	-1.616597	0.658385	1	RP11-711M9.1	_
ENSG00000257243	0.980265	-1.616597	0.658385	1	RP11-133N21.7	K05687
ENSG00000257830	0.980265	-1.616597	0.658385	1	RP11-845M18.7	_
ENSG00000258581	0.980265	-1.616597	0.658385	1	RP11-638I2.10	K03959
ENSG00000258667	0.980265	-1.616597	0.658385	1	HIF1A-AS2	_
ENSG00000258957	0.980265	-1.616597	0.658385	1	RP11-363J20.1	_
ENSG00000259020	0.980265	-1.616597	0.658385	1	RP11-529H20.3	K13784
ENSG00000259088	0.980265	-1.616597	0.658385	1	CTD-2017C7.2	_
ENSG00000259264	0.980265	-1.616597	0.658385	1	RP11-60L3.1	_
ENSG00000259771	0.980265	-1.616597	0.658385	1	RP11-429D19.1	_
ENSG00000259921	0.980265	-1.616597	0.658385	1	AC022819.3	_
ENSG00000260022	0.980265	-1.616597	0.658385	1	LA16c-306A4.1	_
ENSG00000260837	0.980265	-1.616597	0.658385	1	RP11-434B12.1	_
ENSG00000260977	0.980265	-1.616597	0.658385	1	RP11-333I13.1	_
ENSG00000261574	0.980265	-1.616597	0.658385	1	RP1-168P16.2	_
ENSG00000261634	0.980265	-1.616597	0.658385	1	RP11-352D13.6	_
ENSG00000262094	0.980265	-1.616597	0.658385	1	AC139099.5	_
ENSG00000262772	0.980265	-1.616597	0.658385	1	RP11-353N14.2	_
ENSG00000264304	0.980265	-1.616597	0.658385	1	RP11-20B24.7	_
ENSG00000265625	0.980265	-1.616597	0.658385	1	RP11-68I3.11	_
ENSG00000265702	0.980265	-1.616597	0.658385	1	RP11-156L14.1	_
ENSG00000266236	0.980265	-1.616597	0.658385	1	NARF-IT1	_
ENSG00000267255	0.980265	-1.616597	0.658385	1	CTB-50L17.5	_
ENSG00000267811	0.980265	-1.616597	0.658385	1	RP11-727F15.11	_
ENSG00000268678	0.980265	-1.616597	0.658385	1	CTC-444N24.13	_
ENSG00000269487	0.980265	-1.616597	0.658385	1	CTB-174O21.2	_
ENSG00000269737	0.980265	-1.616597	0.658385	1	RP11-345P4.7	_
ENSG00000270048	0.980265	-1.616597	0.658385	1	RP11-214K3.22	_
ENSG00000270104	0.980265	-1.616597	0.658385	1	RP11-245P10.6	_
ENSG00000270926	0.980265	-1.616597	0.658385	1	RP11-551L14.7	K18461
ENSG00000271327	0.980265	-1.616597	0.658385	1	RP11-1109F11.3	_
ENSG00000271553	0.980265	-1.616597	0.658385	1	RP11-274B21.10	_
ENSG00000271741	0.980265	-1.616597	0.658385	1	RP11-244H3.4	_
ENSG00000272606	0.980265	-1.616597	0.658385	1	RP11-554J4.1	_
ENSG00000272662	0.980265	-1.616597	0.658385	1	RP11-190C22.8	_
ENSG00000272836	0.980265	-1.616597	0.658385	1	RP3-402G11.27	_
ENSG00000272918	0.980265	-1.616597	0.658385	1	CTB-152G17.6	_
ENSG00000272945	0.980265	-1.616597	0.658385	1	CTD-3216D2.5	_
ENSG00000272948	0.980265	-1.616597	0.658385	1	AP001412.1	_
ENSG00000273075	0.980265	-1.616597	0.658385	1	RP11-122G18.8	_
ENSG00000273381	0.980265	-1.616597	0.658385	1	RP11-305L7.7	_
ENSG00000150459	0.291199	6.763112	0.658567	1	SAP18	K14324
ENSG00000067225	0.289035	10.80052	0.658602	1	PKM	K00873

ENSG00000178177	0.305549	4.082017	0.658635	1	LCORL	_
ENSG00000198840	-0.28933	9.094036	0.658669	1	MT-ND3	K03880
ENSG00000118197	-0.297492	5.150873	0.65868	1	DDX59	K19466
ENSG00000107738	-0.30783	3.850645	0.658739	1	C10orf54	_
ENSG00000238072	-0.30783	3.850645	0.658739	1	RP11-305M3.2	K03359
ENSG00000178567	-0.303498	3.762933	0.658781	1	EPM2AIP1	_
ENSG00000147853	-0.295809	4.797846	0.658879	1	AK3	K00944
ENSG00000246263	-0.312056	3.458725	0.658934	1	KB-431C1.4	_
ENSG00000232098	-0.314443	3.346903	0.658997	1	CTD-2619J13.14	_
ENSG00000007237	-0.444469	1.260492	0.659012	1	GAS7	K18618
ENSG00000256185	-0.444469	1.260492	0.659012	1	RP11-612B6.2	_
ENSG00000100918	-0.409844	1.239803	0.659012	1	REC8	K13054
ENSG00000130653	-0.409844	1.239803	0.659012	1	PNPLA7	K14676
ENSG00000175764	0.359131	1.209238	0.659012	1	TTLL11	K16604
ENSG00000204954	-0.302261	4.50176	0.659084	1	C12orf73	_
ENSG00000167842	-0.292918	5.804739	0.659153	1	MIS12	_
ENSG00000164168	-0.3028	4.325086	0.659223	1	TMEM184C	_
ENSG00000250571	-0.3028	4.325086	0.659223	1	GLI4	_
ENSG00000146373	-0.299821	4.82129	0.65935	1	RNF217	K11977
ENSG00000174021	-0.289593	7.59084	0.659378	1	GNG5	K04542
ENSG00000121989	-0.339021	2.536101	0.659432	1	ACVR2A	K04670
ENSG00000205309	-0.339021	2.536101	0.659432	1	NT5M	K01081
ENSG00000125637	0.335652	2.520894	0.659432	1	PSD4	K12494
ENSG00000148158	-0.304526	4.05421	0.659471	1	SNX30	K17921
ENSG00000170836	0.30172	4.045301	0.659471	1	PPM1D	K10147
ENSG00000135482	-0.348653	2.316029	0.65953	1	ZC3H10	_
ENSG00000260329	0.353204	1.673282	0.659535	1	RP11-412D9.4	_
ENSG00000124224	0.349916	2.024442	0.659596	1	PPP4R1L	K15424
ENSG00000092208	-0.301527	4.420218	0.659788	1	GEMIN2	K13130
ENSG00000175662	0.300245	4.414057	0.659788	1	TOM1L2	_
ENSG00000167632	0.299472	4.797101	0.659862	1	TRAPPC9	_
ENSG00000198324	-0.308792	3.627747	0.659953	1	FAM109A	_
ENSG00000132142	0.28979	6.060977	0.659987	1	ACACA	K11262
ENSG00000272520	0.30347	4.227407	0.660031	1	CTD-2044J15.2	_
ENSG00000111652	-0.293991	5.656324	0.660105	1	COPS7A	K12180
ENSG00000071205	-0.302874	4.168239	0.660126	1	ARHGAP10	K13736
ENSG00000110063	0.291649	5.470203	0.660397	1	DCPS	K12584
ENSG00000105063	0.28922	6.932298	0.66047	1	PPP6R1	K15499
ENSG00000109436	0.292534	4.969603	0.660526	1	TBC1D9	_
ENSG00000148516	0.290267	5.566011	0.66053	1	ZEB1	K09299
ENSG00000163214	-0.29466	4.933029	0.660598	1	DHX57	K13026
ENSG00000163636	-0.289721	6.648804	0.660656	1	PSMD6	K03037
ENSG00000075426	0.288127	7.66929	0.660787	1	FOSL2	K09030
ENSG00000164051	-0.291405	5.557487	0.660844	1	CCDC51	_
ENSG00000232573	0.287749	7.981202	0.660946	1	RPL3P4	K02925
ENSG00000096092	-0.294711	5.342245	0.660967	1	TMEM14A	_
ENSG00000090776	-0.306358	3.577201	0.661151	1	EFNB1	K05463
ENSG00000105556	0.297037	4.361651	0.661184	1	MIER2	_
ENSG00000112667	-0.290251	6.150715	0.661228	1	DNPH1	_
ENSG00000138190	-0.303076	4.305283	0.661322	1	EXOC6	K09228
ENSG00000087077	-0.288213	7.179012	0.661409	1	TRIP6	K12792
ENSG00000005302	-0.294073	5.297285	0.661413	1	MSL3	K18403
ENSG00000196458	0.311919	2.638918	0.661501	1	ZNF605	K09228
ENSG00000163686	0.299282	4.688902	0.661603	1	ABHD6	K13700
ENSG00000143401	-0.308447	4.103505	0.661813	1	ANP32E	K18648
ENSG00000006282	-0.291474	5.393159	0.66185	1	SPATA20	K06888
ENSG00000085382	-0.303925	3.733592	0.661878	1	HACE1	K12166
ENSG00000092969	-0.297931	3.730212	0.661878	1	TGFB2	K13376

ENSG00000160305	0.291393	5.328771	0.662016	1	DIP2A	K17985
ENSG00000163904	-0.291303	5.838811	0.662017	1	SENP2	K03345
ENSG00000075914	-0.293701	5.056622	0.662035	1	EXOSC7	K17520
ENSG00000239213	0.30428	3.629009	0.662135	1	RP11-85F14.5	_
ENSG00000144036	-0.293971	5.020643	0.662142	1	EXOC6B	_
ENSG00000132256	-0.302282	4.211441	0.662283	1	TRIM5	K10648
ENSG00000162300	0.313113	3.078484	0.662318	1	ZFPL1	_
ENSG00000227467	-0.311243	3.087942	0.662318	1	RP11-169D4.1	_
ENSG00000187051	-0.28876	6.368853	0.66244	1	RPS19BP1	_
ENSG00000151657	-0.300231	4.372365	0.662497	1	KIN	K13102
ENSG00000143390	-0.287848	6.735624	0.662547	1	RFX5	K08061
ENSG00000165678	-0.286119	8.326247	0.662598	1	GHITM	_
ENSG00000145725	-0.617259	0.193598	0.662736	1	PPIP5K2	K13024
ENSG00000177455	-0.617259	0.193598	0.662736	1	CD19	K06465
ENSG00000227039	-0.617259	0.193598	0.662736	1	ITGB2-AS1	_
ENSG00000229160	-0.617259	0.193598	0.662736	1	AC009229.6	_
ENSG00000235217	-0.617259	0.193598	0.662736	1	TSPY26P	K11286
ENSG00000237004	-0.617259	0.193598	0.662736	1	ZNRF2P1	K10694
ENSG00000247373	-0.617259	0.193598	0.662736	1	RP11-486O12.2	_
ENSG00000267041	-0.617259	0.193598	0.662736	1	ZNF850	K09228
ENSG00000267152	-0.617259	0.193598	0.662736	1	CTD-2528L19.6	_
ENSG00000105647	-0.539959	0.147545	0.662736	1	PIK3R2	K02649
ENSG00000126231	-0.539959	0.147545	0.662736	1	PROZ	_
ENSG00000169085	-0.539959	0.147545	0.662736	1	C8orf46	_
ENSG00000226763	-0.539959	0.147545	0.662736	1	SRRM5	K02914
ENSG00000228315	-0.539959	0.147545	0.662736	1	GUSBP11	_
ENSG00000247853	-0.539959	0.147545	0.662736	1	RP5-940J5.6	_
ENSG00000256427	-0.539959	0.147545	0.662736	1	RP11-118B22.4	_
ENSG00000260464	-0.539959	0.147545	0.662736	1	RP4-561L24.3	_
ENSG00000262587	-0.539959	0.147545	0.662736	1	RP11-266L9.4	_
ENSG00000108551	0.502876	0.097717	0.662736	1	RASD1	K07843
ENSG00000138152	0.502876	0.097717	0.662736	1	BTBD16	K10487
ENSG00000144278	0.502876	0.097717	0.662736	1	GALNT13	K00710
ENSG00000160963	0.502876	0.097717	0.662736	1	COL26A1	_
ENSG00000162290	0.502876	0.097717	0.662736	1	DCP1A	K12610
ENSG00000171045	0.502876	0.097717	0.662736	1	TSNARE1	K13814
ENSG00000177675	0.502876	0.097717	0.662736	1	CD163L1	K06545
ENSG00000178460	0.502876	0.097717	0.662736	1	MCMDC2	K11447
ENSG00000183638	0.502876	0.097717	0.662736	1	RP1L1	K19538
ENSG00000213025	0.502876	0.097717	0.662736	1	COX20P1	K18184
ENSG00000216588	0.502876	0.097717	0.662736	1	IGSF23	_
ENSG00000261879	0.502876	0.097717	0.662736	1	RP11-333E1.1	_
ENSG00000157077	0.29662	4.645715	0.66281	1	ZFYVE9	K04679
ENSG00000155256	0.29156	5.237438	0.663014	1	ZFYVE27	K19368
ENSG00000136450	-0.285935	8.018994	0.663022	1	SRSF1	K12890
ENSG00000164144	-0.297777	4.881961	0.663028	1	ARFIP1	_
ENSG00000103061	0.309272	3.677767	0.663048	1	SLC7A6OS	_
ENSG00000066117	-0.287691	6.54147	0.66312	1	SMARCD1	K11650
ENSG00000187498	0.2865	7.174356	0.66313	1	COL4A1	K06237
ENSG00000135900	-0.289764	5.719201	0.66316	1	MRPL44	K17425
ENSG00000171204	-0.289461	6.202187	0.663177	1	TMEM126B	K18165
ENSG00000153214	-0.292962	4.796259	0.66334	1	TMEM87B	_
ENSG00000107372	-0.286069	7.51099	0.663355	1	ZFAND5	_
ENSG00000112941	0.286495	6.972706	0.663416	1	PAPD7	K03514
ENSG00000175756	-0.286976	6.782531	0.663419	1	AURKAIP1	K16830
ENSG00000102531	-0.286535	6.954476	0.663604	1	FNDC3A	_
ENSG00000104093	0.293531	4.963984	0.663618	1	DMXL2	_
ENSG00000102226	-0.28955	5.401472	0.663787	1	USP11	K11835

ENSG00000186470	-0.289122	6.280338	0.663793	1	BTN3A2	K06712
ENSG00000163923	-0.288432	5.672734	0.663869	1	RPL39L	K02924
ENSG00000106366	-0.291664	4.774013	0.663918	1	SERPINE1	K03982
ENSG00000176092	-0.355699	1.524185	0.663922	1	AIM1L	_
ENSG00000100246	-0.330906	2.5658	0.663944	1	DNAL4	K10412
ENSG00000249673	-0.330906	2.5658	0.663944	1	NOP14-AS1	_
ENSG00000070413	-0.290813	5.340082	0.664034	1	DGCR2	_
ENSG00000135185	-0.290813	5.340082	0.664034	1	TMEM243	_
ENSG00000230989	-0.286822	6.549444	0.664061	1	HSBP1	K19765
ENSG00000167964	0.289304	5.68431	0.664083	1	RAB26	K07913
ENSG00000100297	0.285493	7.259643	0.664134	1	MCM5	K15696
ENSG00000126464	0.289773	5.122392	0.664616	1	PRR12	_
ENSG00000264608	0.423524	0.95908	0.66464	1	RP11-192H23.8	_
ENSG00000047230	-0.290544	5.561203	0.664641	1	CTPS2	K01937
ENSG00000165672	-0.284089	8.412208	0.664792	1	PRDX3	K03386
ENSG00000152818	-0.289195	4.993388	0.664819	1	UTRN	_
ENSG00000106571	-0.310622	2.859248	0.664839	1	GLI3	K06230
ENSG00000262814	-0.286104	6.488804	0.664988	1	MRPL12	K02935
ENSG00000186205	-0.285888	6.59456	0.664994	1	1-Mar	_
ENSG00000160199	0.297405	3.914636	0.665017	1	PKNOX1	_
ENSG00000110002	-0.290096	5.479135	0.665027	1	VWA5A	_
ENSG00000058729	-0.28929	5.653719	0.665049	1	RIOK2	K07179
ENSG00000173171	-0.292173	5.108325	0.665091	1	MTX1	K17776
ENSG00000136560	-0.292794	5.013878	0.665146	1	TANK	K12650
ENSG00000131408	0.286561	6.244183	0.665213	1	NR1H2	K08535
ENSG00000142599	0.289456	4.971012	0.665341	1	RERE	K05628
ENSG00000170234	-0.291097	4.871502	0.66542	1	PWWP2A	_
ENSG00000146247	-0.285334	6.683322	0.665423	1	PHIP	K11797
ENSG00000248333	0.294892	4.553373	0.665462	1	CDK11B	K08818
ENSG00000183354	0.298087	4.150434	0.665523	1	KIAA2026	_
ENSG00000066739	0.304347	3.589448	0.665612	1	ATG2B	K17906
ENSG00000157107	-0.29081	5.147158	0.66568	1	FCHO2	_
ENSG00000152795	-0.283144	8.686957	0.665688	1	HNRNPDL	K13044
ENSG00000102882	0.286845	5.564155	0.665785	1	MAPK3	K04371
ENSG00000112578	-0.285738	5.856313	0.665789	1	BYSL	K14797
ENSG00000146648	-0.28411	7.199736	0.665912	1	EGFR	K04361
ENSG00000198682	0.283376	7.656263	0.666054	1	PAPSS2	K13811
ENSG00000166352	-0.324741	2.947538	0.666205	1	C11orf74	_
ENSG00000170684	-0.324741	2.947538	0.666205	1	ZNF296	K17576
ENSG00000110844	0.318551	2.936603	0.666205	1	PRPF40B	K12821
ENSG00000144591	0.28623	5.631252	0.666262	1	GMPPA	K00966
ENSG00000168958	0.283862	6.925428	0.666397	1	MFF	_
ENSG00000138767	-0.295411	4.480291	0.666494	1	CNOT6L	K12603
ENSG00000180354	0.301684	3.790013	0.666616	1	MTURN	K08051
ENSG00000135749	0.319444	2.443049	0.666715	1	PCNXL2	_
ENSG00000211460	-0.283657	6.982813	0.666738	1	TSN	K17934
ENSG00000108379	-0.305958	3.380807	0.666825	1	WNT3	K00312
ENSG00000131174	0.291176	4.418232	0.666833	1	COX7B	K02271
ENSG00000162923	0.283317	7.023531	0.666855	1	WDR26	_
ENSG00000152240	-0.291173	4.857972	0.666926	1	HAUS1	K16584
ENSG00000130299	-0.285109	5.818008	0.666986	1	GTPBP3	K03650
ENSG00000130475	-0.292847	4.589838	0.667004	1	FCHO1	_
ENSG00000135211	-0.288882	4.743257	0.66714	1	TMEM60	_
ENSG00000109265	0.315925	2.760224	0.667152	1	KIAA1211	_
ENSG00000272631	0.372093	1.71278	0.66739	1	RP11-359E3.4	_
ENSG00000082805	0.285378	5.967919	0.667408	1	ERC1	K16072
ENSG00000134779	-0.285464	6.011018	0.667573	1	TPGS2	K16605
ENSG00000176248	-0.287619	5.418981	0.667597	1	ANAPC2	K03349

ENSG00000171103	-0.28725	4.793081	0.667825	1	TRMT61B	K08694
ENSG00000133731	-0.284296	5.941453	0.667826	1	IMPA1	K01092
ENSG00000137492	-0.284825	6.031921	0.667858	1	PRKRIR	_
ENSG00000146731	-0.281025	9.090556	0.667865	1	CCT6A	K09498
ENSG00000175283	-0.296557	4.131158	0.668001	1	DOLK	K00902
ENSG00000126457	-0.281734	7.5021	0.668164	1	PRMT1	K11434
ENSG00000119812	-0.283025	6.570347	0.668208	1	FAM98A	_
ENSG00000169696	0.285509	5.505431	0.668231	1	ASPSCR1	K15627
ENSG00000137500	-0.28553	5.550029	0.668235	1	CCDC90B	K04257
ENSG00000125351	-0.285527	5.973869	0.668265	1	UPF3B	K14328
ENSG00000134146	-0.323134	2.594908	0.668342	1	DPH6	K06927
ENSG00000163558	-0.282349	6.855091	0.668402	1	PRKCI	K06069
ENSG00000145293	0.283759	6.041763	0.668404	1	ENOPH1	K09880
ENSG00000143155	-0.285574	5.524564	0.668427	1	TIPRL	K17607
ENSG00000189339	0.29292	4.454719	0.668488	1	SLC35E2B	K15284
ENSG00000118965	-0.292461	4.059354	0.668511	1	WDR35	K19674
ENSG00000212978	-0.302919	3.320597	0.668514	1	AC016747.3	_
ENSG00000259943	0.298753	3.49539	0.668522	1	RP1-39G22.7	_
ENSG00000234327	0.306819	3.653593	0.668523	1	AC012146.7	_
ENSG00000197912	0.282524	6.463552	0.668713	1	SPG7	K09552
ENSG00000134825	-0.285885	5.779454	0.668741	1	TMEM258	_
ENSG00000177125	0.292759	4.647487	0.668831	1	ZBTB34	K10508
ENSG00000140848	-0.29296	4.573373	0.668855	1	CPNE2	K11341
ENSG00000083168	0.290261	4.300631	0.668946	1	KAT6A	K11305
ENSG00000260027	-0.288946	4.307388	0.668946	1	HOXB7	K09307
ENSG00000083845	0.279767	9.766383	0.669031	1	RPS5	K02989
ENSG00000124541	-0.283603	6.066972	0.669071	1	RRP36	K14572
ENSG00000187866	0.291087	4.595774	0.669213	1	FAM122A	_
ENSG00000177082	-0.289862	4.520894	0.66926	1	WDR73	K06115
ENSG00000162222	0.287236	5.3145	0.669333	1	TTC9C	_
ENSG00000132394	-0.294716	4.340244	0.669372	1	EEFSEC	K03833
ENSG00000129255	-0.283524	6.289901	0.669426	1	MPDU1	K09660
ENSG00000133863	-0.284399	4.777182	0.669444	1	TEX15	_
ENSG00000183793	-0.385662	1.311362	0.669495	1	NPIPA5	K01896
ENSG00000213020	0.385075	1.262982	0.669495	1	ZNF611	K09228
ENSG00000071967	0.282413	5.977005	0.669542	1	CYBRD1	K08370
ENSG00000196187	0.286601	5.330958	0.669543	1	TMEM63A	_
ENSG00000108819	0.281805	6.437947	0.669563	1	PPP1R9B	K17551
ENSG00000170348	0.279992	7.904425	0.669596	1	TMED10	_
ENSG00000095397	0.289289	4.542141	0.669623	1	DFNB31	_
ENSG00000133302	0.281127	6.783946	0.669654	1	ANKRD32	_
ENSG00000121211	-0.288389	4.869996	0.669683	1	MND1	_
ENSG00000143363	-0.283927	5.398357	0.669705	1	PRUNE	K01514
ENSG00000231607	-0.326724	2.693287	0.669732	1	DLEU2	_
ENSG00000101150	-0.280249	7.521784	0.669789	1	TPD52L2	_
ENSG00000215251	0.284193	5.455269	0.669853	1	FASTKD5	_
ENSG00000245849	0.320619	2.35156	0.669885	1	RAD51-AS1	_
ENSG00000114767	-0.289358	4.955775	0.669895	1	RRP9	K14793
ENSG00000132635	-0.285209	5.149619	0.669956	1	PCED1A	_
ENSG00000087470	-0.280013	7.405698	0.670184	1	DNM1L	K17065
ENSG00000144867	-0.281024	6.624183	0.670309	1	SRPRB	K12272
ENSG00000104331	0.279661	7.500301	0.670326	1	IMPAD1	K15759
ENSG00000144061	-0.818653	-0.489463	0.670342	1	NPHP1	K19657
ENSG00000197748	-0.818653	-0.489463	0.670342	1	WDR96	_
ENSG00000205572	-0.818653	-0.489463	0.670342	1	SERF1B	_
ENSG00000228363	-0.818653	-0.489463	0.670342	1	AC015971.2	_
ENSG00000231360	-0.818653	-0.489463	0.670342	1	AL592284.1	K15258
ENSG00000243251	-0.818653	-0.489463	0.670342	1	PGBD3	K10841

ENSG00000245711	-0.818653	-0.489463	0.670342	1	NADK2-AS1	_
ENSG00000260655	-0.818653	-0.489463	0.670342	1	CTA-250D10.23	_
ENSG00000265243	-0.818653	-0.489463	0.670342	1	IGLJCOR18	K06554
ENSG00000272140	-0.818653	-0.489463	0.670342	1	RP11-574K11.29	_
ENSG00000073009	0.801779	-0.512045	0.670342	1	IKBKG	K07210
ENSG00000104901	0.801779	-0.512045	0.670342	1	DKKL1	_
ENSG00000105146	0.801779	-0.512045	0.670342	1	AURKC	K11480
ENSG00000124251	0.801779	-0.512045	0.670342	1	TP53TG5	_
ENSG00000125910	0.801779	-0.512045	0.670342	1	S1PR4	K04293
ENSG00000126460	0.801779	-0.512045	0.670342	1	PRRG2	_
ENSG00000134256	0.801779	-0.512045	0.670342	1	CD101	K06522
ENSG00000151715	0.801779	-0.512045	0.670342	1	TMEM45B	K17776
ENSG00000169067	0.801779	-0.512045	0.670342	1	ACTBL2	K05692
ENSG00000175229	0.801779	-0.512045	0.670342	1	GAL3ST3	K09676
ENSG00000178430	0.801779	-0.512045	0.670342	1	AC006111.1	K12581
ENSG00000186517	0.801779	-0.512045	0.670342	1	ARHGAP30	_
ENSG00000196730	0.801779	-0.512045	0.670342	1	DAPK1	K08803
ENSG00000203832	0.801779	-0.512045	0.670342	1	NBPF20	_
ENSG00000213658	0.801779	-0.512045	0.670342	1	LAT	K07362
ENSG00000214514	0.801779	-0.512045	0.670342	1	KRT42P	K07604
ENSG00000225793	0.801779	-0.512045	0.670342	1	RP1-234P15.4	_
ENSG00000225976	0.801779	-0.512045	0.670342	1	RP11-192N10.2	K02219
ENSG00000229261	0.801779	-0.512045	0.670342	1	RP11-227H15.4	_
ENSG00000235082	0.801779	-0.512045	0.670342	1	SUMO1P3	K12160
ENSG00000240445	0.801779	-0.512045	0.670342	1	FOXO3B	K09408
ENSG00000251000	0.801779	-0.512045	0.670342	1	AC008592.3	K00682
ENSG00000253459	0.801779	-0.512045	0.670342	1	AL139099.1	K10348
ENSG00000259321	0.801779	-0.512045	0.670342	1	RP11-468E2.5	_
ENSG00000260747	0.801779	-0.512045	0.670342	1	RP11-421N8.1	K02127
ENSG00000260872	0.801779	-0.512045	0.670342	1	RP11-680G24.5	_
ENSG00000262692	0.801779	-0.512045	0.670342	1	CTD-3195I5.3	_
ENSG00000266456	0.801779	-0.512045	0.670342	1	RP11-806L2.2	_
ENSG00000268310	0.801779	-0.512045	0.670342	1	AC087645.1	K08731
ENSG00000273017	0.801779	-0.512045	0.670342	1	AP000240.9	_
ENSG00000166359	0.61337	-0.435981	0.670342	1	WDR88	K03231
ENSG00000199804	0.61337	-0.435981	0.670342	1	RNA5SP383	_
ENSG00000224550	0.61337	-0.435981	0.670342	1	RP11-270C12.3	K03948
ENSG00000224728	0.61337	-0.435981	0.670342	1	AC090945.1	K00088
ENSG00000225083	0.61337	-0.435981	0.670342	1	GRTP1-AS1	_
ENSG00000231233	0.61337	-0.435981	0.670342	1	CCDC147-AS1	_
ENSG00000232485	0.61337	-0.435981	0.670342	1	AC098820.3	_
ENSG00000256894	0.61337	-0.435981	0.670342	1	RP11-283G6.3	_
ENSG00000257489	0.61337	-0.435981	0.670342	1	RP11-175P13.3	K16580
ENSG00000258384	0.61337	-0.435981	0.670342	1	AC068831.6	_
ENSG00000260423	0.61337	-0.435981	0.670342	1	RP13-735L24.1	_
ENSG00000265417	0.61337	-0.435981	0.670342	1	RP11-288C17.1	_
ENSG00000267922	0.61337	-0.435981	0.670342	1	AC007193.6	_
ENSG00000272842	0.61337	-0.435981	0.670342	1	RP11-513M16.7	_
ENSG00000273008	0.61337	-0.435981	0.670342	1	RP11-351D16.3	_
ENSG00000178057	-0.283694	5.844931	0.670437	1	NDUFAF3	K09008
ENSG00000167645	-0.281518	6.303834	0.670551	1	YIF1B	K17985
ENSG00000144560	0.286629	4.100235	0.670568	1	VGLL4	_
ENSG00000173992	0.288732	4.629881	0.670629	1	CCS	K04569
ENSG00000153310	-0.279315	7.652271	0.670687	1	FAM49B	_
ENSG00000055147	-0.297969	3.989414	0.670744	1	FAM114A2	_
ENSG00000148803	-0.289703	4.554837	0.670755	1	FUOM	K02431
ENSG00000109756	-0.2904	4.736707	0.670813	1	RAPGEF2	K08018
ENSG00000179085	0.28841	4.730894	0.670813	1	DPM3	K09659

ENSG00000100439	0.291481	4.241503	0.670834	1	ABHD4	K13698
ENSG00000104983	-0.300162	2.797198	0.670933	1	CCDC61	K16755
ENSG00000145569	0.281777	5.72804	0.670946	1	FAM105A	_
ENSG00000138614	-0.285604	5.507335	0.670984	1	VWA9	_
ENSG00000134809	-0.282103	5.479135	0.671235	1	TIMM10	K17778
ENSG00000160221	-0.280191	6.607663	0.671266	1	C21orf33	_
ENSG00000254332	-0.318981	2.489281	0.671374	1	GS1-44D20.1	K10375
ENSG00000059378	-0.280897	5.815682	0.671434	1	PARP12	K15259
ENSG00000164967	-0.289485	4.182667	0.671436	1	RPP25L	K14525
ENSG00000022976	-0.293502	3.909977	0.671463	1	ZNF839	_
ENSG00000126001	0.28187	5.549226	0.671478	1	CEP250	K16464
ENSG00000214753	0.283368	5.448245	0.671496	1	HNRNPUL2	_
ENSG00000159063	-0.281755	5.978051	0.671532	1	ALG8	K03849
ENSG00000176915	-0.278506	7.580636	0.67166	1	ANKLE2	_
ENSG00000100664	0.278059	7.808753	0.67182	1	EIF5	K03262
ENSG00000233608	-0.350197	2.097332	0.67196	1	TWIST2	K09069
ENSG00000168000	0.340585	2.067237	0.67196	1	BSCL2	K19365
ENSG00000187187	0.340585	2.067237	0.67196	1	ZNF546	K09228
ENSG00000177106	-0.280162	6.37364	0.671961	1	EPS8L2	K17277
ENSG00000177283	-0.315524	2.889289	0.671981	1	FZD8	K02375
ENSG00000143870	0.277404	8.359871	0.672169	1	PDIA6	K09584
ENSG00000092148	0.27934	6.446524	0.672275	1	HECTD1	K12231
ENSG00000111490	-0.292435	4.387041	0.672319	1	TBC1D30	_
ENSG00000082458	0.289341	4.3808	0.672319	1	DLG3	K12075
ENSG00000113761	-0.290376	3.997585	0.672398	1	ZNF346	_
ENSG00000143543	-0.277257	8.322608	0.672432	1	JTB	_
ENSG00000105447	-0.280775	5.736565	0.672445	1	GRWD1	K14848
ENSG00000067066	-0.28677	4.967015	0.672452	1	SP100	K15413
ENSG00000137824	0.280738	5.313401	0.672477	1	RMDN3	_
ENSG00000008324	-0.291876	4.15098	0.6725	1	SS18L2	_
ENSG00000183077	0.288474	4.284966	0.672582	1	AFMID	K01432
ENSG00000166436	0.32329	2.581123	0.67263	1	TRIM66	K12032
ENSG00000081307	-0.280096	6.082574	0.672633	1	UBA5	K12164
ENSG00000002016	-0.295309	3.683189	0.672653	1	RAD52	K10873
ENSG00000165730	0.359464	1.513935	0.672668	1	STOX1	_
ENSG00000187796	0.359464	1.513935	0.672668	1	CARD9	K12794
ENSG00000234498	0.359464	1.513935	0.672668	1	RPL13AP20	K02872
ENSG00000130204	-0.277708	7.280865	0.672901	1	TOMM40	K11518
ENSG00000166913	-0.276819	8.277448	0.672949	1	YWHAB	K16197
ENSG00000147677	0.276379	9.041942	0.673002	1	EIF3H	K03247
ENSG00000168778	0.298197	3.263762	0.673043	1	TCTN2	K19361
ENSG00000149554	-0.280638	5.652855	0.673049	1	CHEK1	K02216
ENSG00000137842	-0.287051	4.862482	0.673064	1	TMEM62	K10625
ENSG00000188549	0.284949	5.10592	0.673109	1	C15orf52	_
ENSG00000144741	-0.290778	3.921558	0.673206	1	SLC25A26	K15111
ENSG00000196652	-0.289068	4.698409	0.673283	1	ZKSCAN5	K09229
ENSG00000215030	0.278005	6.641655	0.673368	1	RPL13P12	K02873
ENSG00000166839	0.329596	2.216391	0.673545	1	ANKDD1A	K12581
ENSG00000213689	0.287564	4.507825	0.67356	1	TREX1	K10790
ENSG00000108641	-0.286707	4.393269	0.673575	1	B9D1	K16744
ENSG00000161647	-0.286707	4.393269	0.673575	1	MPP3	_
ENSG00000188985	-0.284978	4.26469	0.673592	1	DHFRP1	K00287
ENSG00000172795	-0.282983	5.306158	0.67364	1	DCP2	K12613
ENSG00000108091	0.277588	6.746797	0.673644	1	CCDC6	K09288
ENSG00000099822	0.280388	5.716394	0.67369	1	HCN2	K04955
ENSG00000183431	-0.276597	7.614118	0.673747	1	SF3A3	K12827
ENSG00000114744	-0.278533	5.73163	0.673812	1	COMMD2	_
ENSG00000141994	-0.282957	4.866979	0.673969	1	DUS3L	K05544

ENSG00000132004	0.292637	4.152954	0.673974	1	FBXW9	K10265
ENSG00000115514	-0.278992	6.294943	0.673994	1	TXNDC9	_
ENSG00000142541	0.274894	11.36943	0.674251	1	RPL13A	K02872
ENSG00000164187	0.282649	5.073691	0.674278	1	LMBRD2	_
ENSG00000101901	-0.28447	4.703441	0.674296	1	ALG13	K07432
ENSG00000100815	-0.281642	4.912774	0.674388	1	TRIP11	_
ENSG00000007341	-0.28871	4.365923	0.674535	1	ST7L	_
ENSG00000138032	-0.279714	5.592407	0.674545	1	PPM1B	K04461
ENSG00000174238	0.276878	6.174127	0.674601	1	PITPNA	_
ENSG00000169871	0.294099	3.684832	0.67468	1	TRIM56	K12756
ENSG00000106992	0.284526	4.082227	0.674773	1	AK1	K00939
ENSG00000076108	0.276484	6.802143	0.674776	1	BAZ2A	K15224
ENSG00000215193	0.278893	5.578007	0.67497	1	PEX26	K13340
ENSG00000137880	0.279685	5.710535	0.675081	1	GCHFR	_
ENSG00000184056	-0.286614	4.430313	0.675096	1	VPS33B	_
ENSG00000151748	-0.278518	5.468292	0.675102	1	SAV1	K16686
ENSG00000138944	0.284116	4.572012	0.675209	1	KIAA1644	_
ENSG00000104884	-0.2775	5.844939	0.675274	1	ERCC2	K10844
ENSG00000105699	0.289226	3.960566	0.675305	1	LSR	_
ENSG00000135736	0.289226	3.960566	0.675305	1	CCDC102A	K16759
ENSG00000166377	-0.29888	2.906509	0.675443	1	ATP9B	K01530
ENSG00000153975	-0.281667	4.938715	0.675446	1	ZUFSP	_
ENSG00000101856	-0.275208	7.462203	0.675458	1	PGRMC1	K17278
ENSG00000167685	0.283165	5.039385	0.675502	1	ZNF444	K09230
ENSG00000166226	-0.27435	8.520352	0.675553	1	CCT2	K09494
ENSG00000116731	0.304943	3.277264	0.675706	1	PRDM2	K11432
ENSG00000087088	-0.277917	5.95706	0.67577	1	BAX	K02159
ENSG00000117505	-0.274988	7.38071	0.675799	1	DR1	_
ENSG00000236859	0.324581	2.498193	0.675907	1	NIFK-AS1	_
ENSG00000265096	0.324581	2.498193	0.675907	1	C1QTNF1-AS1	_
ENSG00000137309	-0.273777	8.746351	0.676077	1	HMGA1	K09282
ENSG00000100865	-0.2814	4.686515	0.6761	1	CINP	_
ENSG00000157741	-0.281749	5.026001	0.676136	1	UBN2	K17492
ENSG00000167670	0.27485	7.033585	0.67624	1	CHAF1A	K10750
ENSG00000064601	0.274019	7.872893	0.676266	1	CTSA	K13289
ENSG00000096433	0.274406	7.388095	0.67629	1	ITPR3	K04960
ENSG00000117222	-0.284758	4.438406	0.676306	1	RBBP5	K14961
ENSG00000140632	0.278113	5.571558	0.676506	1	GLYR1	_
ENSG00000137942	0.275015	6.737352	0.676519	1	FNBP1L	_
ENSG00000243554	-0.36819	1.957939	0.676549	1	AC004967.7	_
ENSG00000177409	-0.34718	1.94562	0.676549	1	SAMD9L	_
ENSG00000225361	-0.34718	1.94562	0.676549	1	PPP1R26-AS1	_
ENSG00000003393	-0.285159	4.136044	0.6766	1	ALS2	K04575
ENSG00000140718	0.29531	3.926257	0.676639	1	FTO	K19469
ENSG00000143815	-0.276889	6.273594	0.676748	1	LBR	K19532
ENSG00000198804	0.272538	13.96935	0.676795	1	MT-CO1	K02256
ENSG00000102053	-0.387832	1.037639	0.676811	1	ZC3H12B	K18668
ENSG00000226471	-0.387832	1.037639	0.676811	1	CTA-292E10.6	_
ENSG00000226800	-0.387832	1.037639	0.676811	1	CACTIN-AS1	_
ENSG00000213079	-0.282941	4.664236	0.676922	1	SCAF8	K13191
ENSG00000185246	-0.281655	4.925803	0.676989	1	PRPF39	K13217
ENSG00000163625	-0.282899	4.211275	0.67715	1	WDFY3	_
ENSG00000126882	0.285674	4.013207	0.677263	1	FAM78A	K15696
ENSG00000104219	-0.285339	4.019311	0.677263	1	ZDHHC2	K18932
ENSG00000171763	-0.277243	5.371096	0.677304	1	SPATA5L1	_
ENSG00000214455	-0.314398	3.158457	0.677357	1	RCN1P2	_
ENSG00000100241	0.273541	6.991408	0.677757	1	SBF1	K18061
ENSG00000134871	0.272368	8.313941	0.677808	1	COL4A2	K06237

ENSG00000119431	0.278186	5.354767	0.677826	1	HDHD3	K02159
ENSG00000107341	0.273909	6.578718	0.67804	1	UBE2R2	K02207
ENSG00000164038	-0.290921	3.895298	0.678041	1	SLC9B2	K00019
ENSG00000119541	-0.275906	5.650244	0.678063	1	VPS4B	K12196
ENSG00000107669	-0.279492	5.095546	0.678137	1	ATE1	K00685
ENSG00000146574	-0.277606	5.205282	0.678141	1	CCZ1B	_
ENSG00000198301	-0.277606	5.205282	0.678141	1	SDAD1	K14856
ENSG00000120837	-0.275263	5.812576	0.678152	1	NFYB	K08065
ENSG00000136840	0.277627	5.222244	0.678257	1	ST6GALNAC4	K03374
ENSG00000129667	-0.280476	4.99474	0.67827	1	RHBDF2	_
ENSG00000227345	-0.285095	4.180214	0.678318	1	PARG	K07759
ENSG00000186300	0.293889	3.646796	0.678336	1	ZNF555	K09228
ENSG00000162409	-0.274647	5.70333	0.678401	1	PRKAA2	K07198
ENSG00000097021	-0.274812	6.175203	0.678457	1	ACOT7	K17360
ENSG00000102900	-0.27314	6.903492	0.678544	1	NUP93	K14309
ENSG00000165813	-0.292954	3.986599	0.678599	1	C10orf118	_
ENSG00000127561	-0.29505	3.713566	0.678664	1	SYNGR3	_
ENSG00000221926	-0.282903	4.253232	0.678767	1	TRIM16	K09228
ENSG00000063854	0.275092	5.728034	0.679053	1	HAGH	K01069
ENSG00000165591	-0.291387	3.81352	0.679253	1	FAAH2	K19176
ENSG00000204852	-0.291387	3.81352	0.679253	1	TCTN1	K19382
ENSG00000175505	-0.297776	3.008105	0.679347	1	CLCF1	K05421
ENSG00000152503	0.409081	1.314887	0.679362	1	TRIM36	K12013
ENSG00000215769	0.409081	1.314887	0.679362	1	hsa-mir-6080	_
ENSG00000175772	0.367463	1.334896	0.679362	1	LINC01106	_
ENSG00000142186	0.271452	7.523596	0.679441	1	SCYL1	K08876
ENSG00000146700	0.292921	3.483769	0.679475	1	SRCRB4D	K16198
ENSG00000042429	-0.27432	5.887	0.679478	1	MED17	K15133
ENSG00000138074	-0.277119	5.082672	0.679548	1	SLC5A6	K14386
ENSG00000100731	0.273062	6.310934	0.679583	1	PCNX	_
ENSG00000226015	-0.305654	2.408414	0.679644	1	CCT8P1	K09500
ENSG00000227081	0.272098	6.785106	0.679686	1	RP11-543P15.1	K02978
ENSG00000138018	-0.273874	5.576893	0.6797	1	EPT1	K00993
ENSG00000114270	-0.285035	4.223071	0.679935	1	COL7A1	K16628
ENSG00000118363	-0.274536	5.808675	0.67994	1	SPCS2	K12947
ENSG00000062598	-0.276869	5.023298	0.680005	1	ELMO2	K18985
ENSG00000073331	-0.282817	3.6131	0.680092	1	ALPK1	K08868
ENSG00000095002	-0.271809	6.81485	0.68018	1	MSH2	K08735
ENSG00000214182	0.289886	2.514816	0.68032	1	PTMAP5	K13784
ENSG00000036549	-0.271856	6.629091	0.680482	1	ZZZ3	_
ENSG00000099331	0.272449	5.832668	0.680519	1	MYO9B	K10360
ENSG00000025772	-0.271661	6.654532	0.680649	1	TOMM34	K17766
ENSG00000183508	0.275112	5.179268	0.680774	1	FAM46C	_
ENSG00000123737	-0.274111	6.150137	0.680796	1	EXOSC9	K03678
ENSG00000050426	0.271021	6.816218	0.680835	1	LETMD1	_
ENSG00000176148	0.280216	3.764693	0.680856	1	TCP11L1	_
ENSG00000127328	-0.281364	4.655564	0.68087	1	RAB3IP	K16779
ENSG00000171490	-0.269494	8.720028	0.680873	1	RSL1D1	K14775
ENSG00000249042	-0.294889	3.316047	0.680906	1	CTD-2015H6.3	_
ENSG00000181240	0.345764	1.574563	0.680978	1	SLC25A41	K14684
ENSG00000226237	0.345764	1.574563	0.680978	1	RP11-276H19.1	_
ENSG00000083123	-0.280655	4.155802	0.680986	1	BCKDHB	K00167
ENSG00000126822	0.278055	5.085387	0.681075	1	PLEKHG3	_
ENSG00000120727	0.27018	7.221535	0.681198	1	PAIP2	_
ENSG00000213593	-0.270157	7.31541	0.681267	1	TMX2	_
ENSG00000182473	-0.272916	5.148363	0.681328	1	EXOC7	K07195
ENSG00000017797	0.270607	6.152053	0.681526	1	RALBP1	K08773
ENSG00000115866	-0.272537	6.163035	0.681565	1	DARS	K01876

ENSG00000123728	-0.270418	6.172787	0.681603	1	RAP2C	K07839
ENSG00000047365	-0.299496	2.852891	0.681793	1	ARAP2	K18440
ENSG00000167637	-0.299496	2.852891	0.681793	1	ZNF283	K09228
ENSG00000213965	-0.275828	5.277076	0.681847	1	NUDT19	K13355
ENSG00000115109	-0.279803	4.662492	0.68189	1	EPB41L5	_
ENSG00000102710	-0.271237	6.068957	0.681925	1	SUPT20H	_
ENSG00000004961	-0.271937	6.357195	0.68199	1	HCCS	K01764
ENSG00000237989	0.330041	1.780073	0.682082	1	AP001046.5	_
ENSG00000151364	-0.303857	2.935592	0.682157	1	KCTD14	K03968
ENSG00000176542	-0.303857	2.935592	0.682157	1	KIAA2018	K07560
ENSG00000092929	-0.272078	5.855576	0.68237	1	UNC13D	K19728
ENSG00000163026	-0.289082	3.679683	0.682371	1	C2orf44	_
ENSG00000114439	-0.272425	5.988454	0.682414	1	BBX	_
ENSG00000100219	-0.26836	8.16632	0.682463	1	XBP1	K09027
ENSG00000105136	-0.292598	3.029622	0.682477	1	ZNF419	K09228
ENSG00000233328	-0.292598	3.029622	0.682477	1	PFN1P1	K05759
ENSG00000107745	0.271633	6.220784	0.682481	1	MICU1	_
ENSG00000104129	-0.285064	4.201802	0.682551	1	DNAJC17	K09537
ENSG00000099940	0.269977	5.922279	0.682605	1	SNAP29	K08509
ENSG00000182518	-0.296817	3.103004	0.682761	1	FAM104B	_
ENSG00000251136	-0.334618	1.990033	0.682879	1	RP11-37B2.1	_
ENSG00000186395	-0.275284	4.954323	0.682902	1	KRT10	K07604
ENSG00000100142	-0.272333	5.59241	0.682938	1	POLR2F	K03014
ENSG00000105220	0.267402	8.853584	0.683101	1	GPI	K01810
ENSG00000011021	-0.299604	3.257754	0.683242	1	CLCN6	K05015
ENSG00000137207	0.269207	6.579161	0.68328	1	YIPF3	_
ENSG00000176563	0.54798	0.209188	0.683327	1	CNTD1	_
ENSG00000235531	0.54798	0.209188	0.683327	1	RP11-383H13.1	K04984
ENSG00000247121	0.54798	0.209188	0.683327	1	CTD-2260A17.2	_
ENSG00000247199	0.54798	0.209188	0.683327	1	RP11-373N22.3	_
ENSG00000261659	0.54798	0.209188	0.683327	1	LA16c-313D11.12	_
ENSG00000272831	0.54798	0.209188	0.683327	1	RP11-792A8.4	_
ENSG00000003147	-0.47101	0.295755	0.683327	1	ICA1	_
ENSG00000139547	-0.47101	0.295755	0.683327	1	RDH16	K11154
ENSG00000142661	-0.47101	0.295755	0.683327	1	MYOM3	_
ENSG00000273466	-0.47101	0.295755	0.683327	1	RP11-548H3.1	_
ENSG00000108797	0.449586	0.254364	0.683327	1	CNTNAP1	K07379
ENSG00000153165	0.449586	0.254364	0.683327	1	RGPD3	K12172
ENSG00000154874	0.449586	0.254364	0.683327	1	CCDC144B	K09537
ENSG00000197506	0.449586	0.254364	0.683327	1	SLC28A3	K11536
ENSG00000232803	0.449586	0.254364	0.683327	1	RP11-93B14.5	_
ENSG00000254910	0.449586	0.254364	0.683327	1	RP11-326C3.7	_
ENSG00000261089	0.449586	0.254364	0.683327	1	RP11-435I10.3	K09554
ENSG00000265437	0.449586	0.254364	0.683327	1	RP11-757O6.4	_
ENSG00000126698	-0.26861	7.066623	0.683338	1	DNAJC8	K09528
ENSG00000060642	-0.290749	3.333468	0.683447	1	PIGV	K07542
ENSG00000234009	0.280156	4.045525	0.683536	1	RPL5P34	K02932
ENSG00000198825	-0.278066	4.051302	0.683536	1	INPP5F	_
ENSG00000117528	0.268473	6.889148	0.683564	1	ABCD3	K05677
ENSG00000226415	0.278839	4.084873	0.683645	1	TPI1P1	K01803
ENSG00000132549	-0.277983	4.589758	0.683704	1	VPS13B	K19526
ENSG00000117697	-0.273843	5.286083	0.683833	1	NSL1	K11545
ENSG00000167699	-0.271179	5.742313	0.683855	1	GLOD4	_
ENSG00000146083	-0.273821	5.56861	0.683935	1	RNF44	K19041
ENSG00000254893	0.335136	2.280655	0.683945	1	RP11-466P24.2	K07836
ENSG00000197208	0.31472	2.290442	0.683945	1	SLC22A4	K08202
ENSG00000125968	-0.266603	8.9404	0.684012	1	ID1	K04680
ENSG00000127993	-0.285006	4.244031	0.684026	1	RBM48	K15696

ENSG00000165312	0.268226	6.769076	0.684034	1	OTUD1	K13716
ENSG00000140471	-0.292506	3.634576	0.68423	1	LINS	_
ENSG00000167395	0.273239	4.820698	0.684265	1	ZNF646	_
ENSG00000105701	0.26657	8.13678	0.684402	1	FKBP8	K09574
ENSG00000151176	0.280444	4.408002	0.684407	1	PLBD2	_
ENSG00000116133	0.266366	8.244948	0.684562	1	DHCR24	K09828
ENSG00000156675	0.271122	4.979438	0.684596	1	RAB11FIP1	K12484
ENSG00000229043	-0.317253	2.557926	0.684618	1	AC091729.9	_
ENSG00000063761	-0.303469	2.550003	0.684618	1	ADCK1	K08869
ENSG00000204650	-0.303469	2.550003	0.684618	1	CRHR1-IT1	K06883
ENSG00000140545	0.288025	3.830862	0.684665	1	MFGE8	K17253
ENSG00000140939	0.278506	4.560926	0.684711	1	NOL3	_
ENSG00000167491	0.26839	6.319193	0.684715	1	GATAD2A	_
ENSG00000110911	-0.268433	6.436721	0.684717	1	SLC11A2	K12347
ENSG00000174485	0.286254	3.876427	0.684756	1	DENND4A	_
ENSG00000116221	0.266308	7.712947	0.685016	1	MRPL37	K17418
ENSG00000100568	-0.269462	5.867633	0.685025	1	VTI1B	K08493
ENSG00000198270	-0.294574	2.76513	0.685079	1	TMEM116	_
ENSG00000204316	-0.282886	4.100707	0.685134	1	MRPL38	K17419
ENSG00000150593	0.26592	7.752171	0.685417	1	PDCD4	K16865
ENSG00000144381	-0.264954	10.32934	0.685497	1	HSPD1	K04077
ENSG00000185379	0.28957	3.031553	0.685548	1	RAD51D	K10871
ENSG00000147133	-0.271661	5.130965	0.685596	1	TAF1	K03125
ENSG00000137100	0.271041	5.276503	0.685775	1	DCTN3	K10425
ENSG00000127837	-0.267297	6.498101	0.685847	1	AAMP	_
ENSG00000178741	-0.265734	7.492239	0.686004	1	COX5A	K02264
ENSG00000187626	0.292344	3.394795	0.686023	1	ZKSCAN4	K09229
ENSG00000121897	-0.286272	3.532124	0.686152	1	LIAS	K03644
ENSG00000085872	0.269938	5.686854	0.686186	1	CHERP	K12841
ENSG00000175556	-0.282822	3.644979	0.686261	1	LONRF3	_
ENSG00000100243	0.265409	7.381483	0.686349	1	CYB5R3	K00326
ENSG00000225241	-0.282871	3.98095	0.686529	1	RP11-640M9.2	K10653
ENSG00000174938	0.26816	5.429013	0.686531	1	SEZ6L2	_
ENSG00000137814	-0.268367	5.523615	0.686587	1	HAUS2	K16585
ENSG00000197694	0.264684	8.009471	0.686602	1	SPTAN1	K06114
ENSG00000070061	-0.266583	6.473668	0.686698	1	IKBKAP	K11373
ENSG00000241506	-0.279219	4.426195	0.686784	1	PSMC1P1	K03062
ENSG00000121964	-0.493762	0.759606	0.686852	1	GTDC1	_
ENSG00000135119	-0.443433	0.729491	0.686852	1	RNFT2	_
ENSG00000166704	-0.443433	0.729491	0.686852	1	ZNF606	K09228
ENSG00000250073	-0.443433	0.729491	0.686852	1	RP11-677M14.3	_
ENSG00000173762	0.38422	0.719999	0.686852	1	CD7	K06457
ENSG00000268262	0.38422	0.719999	0.686852	1	CTC-246B18.8	_
ENSG00000103042	-0.274766	4.604223	0.686864	1	SLC38A7	K14994
ENSG00000166123	-0.271387	5.09681	0.687036	1	GPT2	K00814
ENSG00000162407	-0.269086	5.318274	0.687094	1	PPAP2B	K01080
ENSG00000124562	-0.264846	7.345372	0.68717	1	SNRPC	K11095
ENSG00000114779	-0.268618	5.707533	0.687176	1	ABHD14B	K13706
ENSG00000138750	-0.266929	6.154465	0.687603	1	NUP54	K14308
ENSG00000110104	-0.264978	6.085835	0.687611	1	CCDC86	K14822
ENSG00000135677	0.263626	8.192612	0.687667	1	GNS	K01137
ENSG00000184752	-0.269271	5.614932	0.687678	1	NDUFA12	_
ENSG00000113593	-0.267073	5.484036	0.6877	1	PPWD1	K12736
ENSG00000117481	-0.269516	5.019233	0.687796	1	NSUN4	K17985
ENSG00000135362	0.271501	4.486538	0.68794	1	PRR5L	_
ENSG00000158158	0.275409	4.105455	0.688072	1	CNNM4	K16302
ENSG00000149196	-0.266661	5.859362	0.688079	1	C11orf73	_
ENSG00000143321	-0.262764	9.496896	0.688132	1	HDGF	K16641

ENSG00000226525	-0.361359	1.119694	0.688138	1	RPS7P10	K02993
ENSG00000101997	0.34902	1.071792	0.688138	1	CCDC22	_
ENSG00000148057	0.34902	1.071792	0.688138	1	IDNK	K00851
ENSG00000158321	0.34902	1.071792	0.688138	1	AUTS2	_
ENSG00000168734	0.34902	1.071792	0.688138	1	PKIG	_
ENSG00000273247	0.34902	1.071792	0.688138	1	RP11-83A24.2	_
ENSG00000237296	0.277688	3.891398	0.688157	1	SMG1P1	K08873
ENSG00000123989	-0.265147	6.53542	0.688168	1	CHPF	K00747
ENSG00000130338	0.282424	3.751687	0.688219	1	TULP4	K17985
ENSG00000232559	-0.286124	3.598753	0.688291	1	GS1-124K5.12	K16467
ENSG00000181284	0.274706	3.589919	0.688291	1	TMEM102	_
ENSG00000111321	0.26315	8.012696	0.688321	1	LTBR	K03159
ENSG00000167157	0.288085	3.268531	0.688445	1	PRRX2	K09329
ENSG00000091732	-0.269702	5.163104	0.688472	1	ZC3HC1	_
ENSG00000223803	0.280378	3.203508	0.688482	1	RPS20P14	K02969
ENSG00000248508	0.280378	3.203508	0.688482	1	SRP14-AS1	_
ENSG00000135913	0.294085	3.124915	0.688521	1	USP37	K11850
ENSG00000225648	0.297589	3.047362	0.688562	1	SBDSP1	K14574
ENSG00000117318	0.262717	8.262621	0.688645	1	ID3	K17694
ENSG00000258056	-0.30465	2.88307	0.688653	1	RP11-644F5.11	_
ENSG00000259877	-0.395562	1.398267	0.688667	1	RP11-46C24.7	_
ENSG00000197566	-0.36397	1.379584	0.688667	1	ZNF624	K09228
ENSG00000254614	-0.36397	1.379584	0.688667	1	AP003068.23	K08694
ENSG00000259901	-0.36397	1.379584	0.688667	1	RP5-991G20.4	_
ENSG00000263843	-0.36397	1.379584	0.688667	1	RP11-649A18.12	_
ENSG00000132640	-0.263036	7.849071	0.688678	1	BTBD3	K10478
ENSG00000165997	-0.267059	5.633582	0.6887	1	ARL5B	K07950
ENSG00000104880	0.264783	5.631239	0.6887	1	ARHGEF18	_
ENSG00000268205	-0.274528	4.821197	0.688742	1	CTC-444N24.11	_
ENSG00000197405	-0.30167	2.678901	0.688752	1	C5AR1	K04010
ENSG00000103150	0.291167	2.674328	0.688752	1	MLYCD	K01578
ENSG00000174194	0.312498	2.559346	0.688805	1	AGAP8	K12491
ENSG00000217130	0.312498	2.559346	0.688805	1	RP3-375P9.2	K02947
ENSG00000263002	0.312498	2.559346	0.688805	1	ZNF234	K09228
ENSG00000162341	-0.296209	2.579442	0.688805	1	TPCN2	K14077
ENSG00000134900	-0.264762	6.434709	0.688822	1	TPP2	K01280
ENSG00000154079	-0.312508	2.449422	0.688856	1	C6orf57	K17985
ENSG00000149591	-0.322572	1.640116	0.688885	1	TAGLN	_
ENSG00000186132	-0.306932	2.332025	0.688906	1	C2orf76	_
ENSG00000074054	0.265744	5.391849	0.688926	1	CLASP1	K16578
ENSG00000235453	-0.328085	2.17637	0.688947	1	TOPORS-AS1	_
ENSG00000254837	0.301204	2.159943	0.688947	1	AP001372.2	_
ENSG00000264247	-0.352379	1.839211	0.688962	1	LINC00909	_
ENSG00000165698	0.348173	1.816835	0.688962	1	C9orf9	_
ENSG00000146205	0.319698	1.830619	0.688962	1	ANO7	K19501
ENSG00000181619	0.319698	1.830619	0.688962	1	GPR135	K08427
ENSG00000166816	-0.322852	2.033137	0.688972	1	LDHD	K00102
ENSG00000267010	-0.322852	2.033137	0.688972	1	RP11-108P20.1	_
ENSG00000269293	-0.322852	2.033137	0.688972	1	ZSCAN16-AS1	_
ENSG00000213468	-0.302722	2.021473	0.688972	1	RP11-453F18__B.1	_
ENSG00000043093	-0.265539	5.878848	0.689371	1	DCUN1D1	K17822
ENSG00000180182	0.263957	6.408212	0.689376	1	MED14	K15156
ENSG00000141068	0.271605	4.189693	0.689418	1	KSR1	K14958
ENSG00000125734	0.263291	6.309246	0.689834	1	GPR108	_
ENSG00000221914	0.263218	6.094723	0.689845	1	PPP2R2A	K04354
ENSG00000105248	-0.270483	4.672789	0.689873	1	CCDC94	K15225
ENSG00000112701	-0.263495	6.569953	0.689943	1	SENP6	K08595
ENSG00000100372	-0.266361	5.772281	0.689985	1	SLC25A17	K13354

ENSG00000134324	0.270823	4.584949	0.690013	1	LPIN1	K15728
ENSG00000048140	-0.265753	5.720885	0.690076	1	TSPAN17	K10303
ENSG00000124486	0.261755	7.757841	0.690085	1	USP9X	K11840
ENSG00000196154	0.260931	9.68426	0.690115	1	S100A4	_
ENSG00000121621	-0.2651	5.66759	0.690174	1	KIF18A	K10401
ENSG00000167994	0.267203	4.986391	0.690203	1	RAB3IL1	K16779
ENSG00000103254	-0.263892	5.300595	0.690317	1	FAM173A	_
ENSG00000106305	-0.263815	5.996039	0.69042	1	AIMP2	K15438
ENSG00000171940	-0.266356	5.42102	0.690685	1	ZNF217	_
ENSG00000164329	-0.264648	5.861626	0.69071	1	PAPD4	K14079
ENSG00000001629	0.263642	6.195822	0.690725	1	ANKIB1	K11967
ENSG00000198874	-0.268002	4.731673	0.690736	1	TYW1	K15449
ENSG00000271975	0.270048	4.199297	0.690782	1	RP11-383J24.6	_
ENSG00000117533	-0.269928	4.204049	0.690782	1	VAMP4	K08513
ENSG00000100722	-0.265259	5.61672	0.690794	1	ZC3H14	_
ENSG00000114988	-0.273808	4.705073	0.690795	1	LMAN2L	K10083
ENSG00000132475	-0.260424	9.311981	0.690818	1	H3F3B	K11253
ENSG00000171159	-0.270692	5.013823	0.690942	1	C9orf16	_
ENSG00000116017	-0.280538	4.087858	0.691051	1	ARID3A	K17985
ENSG00000226221	0.270382	4.564687	0.691123	1	AC022431.1	K02898
ENSG00000144579	-0.264566	5.686426	0.69113	1	CTDSP1	K15731
ENSG00000119688	-0.282874	4.005711	0.691256	1	ABCD4	K05678
ENSG00000198915	-0.268962	4.509275	0.691273	1	RASGEF1A	K00746
ENSG00000154222	0.271403	4.412194	0.691523	1	CC2D1B	K18260
ENSG00000112874	-0.277406	3.874373	0.691612	1	NUDT12	K03426
ENSG00000198087	-0.261473	6.826515	0.691717	1	CD2AP	K13738
ENSG00000129187	-0.26496	5.456368	0.691763	1	DCTD	K01493
ENSG00000131653	0.260625	7.256085	0.691859	1	TRAF7	K10646
ENSG00000103657	0.267512	5.196146	0.691909	1	HERC1	K10594
ENSG00000105698	0.262331	6.211902	0.692064	1	USF2	K09106
ENSG00000163110	0.26038	7.290989	0.692094	1	PDLIM5	K17985
ENSG00000033050	-0.261466	6.627357	0.692099	1	ABCF2	K06185
ENSG00000147509	-0.276546	3.672646	0.692202	1	RGS20	K16449
ENSG00000154723	-0.260528	7.038613	0.692433	1	ATP5J	K02131
ENSG00000100239	-0.263419	6.099318	0.692447	1	PPP6R2	K15500
ENSG00000108219	-0.263444	5.021918	0.69249	1	TSPAN14	K17296
ENSG00000158156	-0.295072	2.705857	0.692544	1	XKR8	_
ENSG00000124279	-0.263404	5.537829	0.692625	1	FASTKD3	_
ENSG00000104047	-0.2623	6.140883	0.692669	1	DTWD1	K17934
ENSG00000103024	-0.263642	5.785051	0.692737	1	NME3	K00940
ENSG00000176720	-0.270617	4.688165	0.692803	1	BOK	K02561
ENSG00000110906	0.26223	5.746132	0.692876	1	KCTD10	K15074
ENSG00000165819	-0.262766	5.611355	0.692888	1	METTL3	K05925
ENSG00000003989	0.260932	5.609818	0.692888	1	SLC7A2	K13864
ENSG00000181396	0.263979	5.292283	0.69292	1	OGFOD3	_
ENSG00000174695	-0.259539	7.474646	0.692976	1	TMEM167A	_
ENSG00000115944	0.259877	6.901286	0.693177	1	COX7A2L	K02270
ENSG00000105559	-0.304506	2.480949	0.693272	1	PLEKHA4	_
ENSG00000026508	0.258185	9.086696	0.693371	1	CD44	K06256
ENSG00000182544	-0.261727	5.481096	0.693405	1	MFSD5	_
ENSG00000085274	-0.270789	4.518872	0.693457	1	MYNN	K09228
ENSG00000055044	-0.259301	7.287135	0.693467	1	NOP58	K14565
ENSG00000110696	-0.259234	7.29831	0.693528	1	C11orf58	_
ENSG00000166478	0.266778	4.740874	0.693548	1	ZNF143	_
ENSG00000142252	-0.265768	5.110812	0.693665	1	GEMIN7	K13135
ENSG00000122068	0.261439	5.976269	0.693694	1	FYTTD1	K10408
ENSG00000103196	0.307841	2.326077	0.693717	1	CRISPLD2	_
ENSG00000116883	0.288257	2.335536	0.693717	1	RP11-268J15.5	K17985

ENSG00000198707	-0.266691	4.87142	0.693911	1	CEP290	K16533
ENSG00000134758	-0.265386	5.171629	0.694113	1	RNF138	K10668
ENSG00000197444	-0.300284	2.20414	0.694234	1	OGDHL	K00164
ENSG00000173960	-0.274988	4.329242	0.694292	1	UBXN2A	K04613
ENSG00000132824	0.258681	7.050839	0.694307	1	SERINC3	_
ENSG00000118705	0.257198	9.117451	0.694474	1	RPN2	K12667
ENSG00000084112	0.263985	5.091868	0.694481	1	SSH1	K05766
ENSG00000143322	0.259659	6.117661	0.694581	1	ABL2	K08887
ENSG00000035115	0.325324	2.036267	0.694839	1	SH3YL1	K14404
ENSG00000122862	-0.259098	6.555138	0.694905	1	SRGN	K06849
ENSG00000100749	-0.260625	5.408696	0.694914	1	VRK1	K08816
ENSG00000090097	0.281595	3.503444	0.694932	1	PCBP4	K13162
ENSG00000138175	0.264189	4.987783	0.695	1	ARL3	K07944
ENSG00000179889	0.25809	7.01496	0.695021	1	PDXDC1	_
ENSG00000164576	0.261464	5.782482	0.695058	1	SAP30L	K19202
ENSG00000105258	-0.268743	4.804028	0.695128	1	POLR2I	K03017
ENSG00000115970	-0.268363	4.616743	0.695144	1	THADA	_
ENSG00000159423	0.283718	3.444165	0.695242	1	ALDH4A1	K00294
ENSG00000167779	-0.2718	3.454034	0.695242	1	IGFBP6	_
ENSG00000101000	-0.26153	5.343301	0.695261	1	PROCR	K06557
ENSG00000128294	0.260091	5.212827	0.695286	1	TPST2	K01021
ENSG00000170185	-0.263135	4.589679	0.695294	1	USP38	K11854
ENSG00000088205	-0.257897	7.025235	0.69541	1	DDX18	K13179
ENSG00000136830	0.256439	8.69558	0.695489	1	FAM129B	_
ENSG00000162576	-0.338651	1.886951	0.695557	1	MXRA8	_
ENSG00000251141	-0.338651	1.886951	0.695557	1	RP11-53O19.1	_
ENSG00000233901	-0.316342	1.873957	0.695557	1	RP11-65J3.1	_
ENSG00000105173	-0.264035	4.532148	0.695611	1	CCNE1	K06626
ENSG00000197008	-0.294116	2.822059	0.695707	1	ZNF138	K09228
ENSG00000198718	-0.294116	2.822059	0.695707	1	FAM179B	K09228
ENSG00000160404	-0.273894	3.746301	0.695748	1	TOR2A	K01056
ENSG00000154832	0.258054	6.555042	0.695819	1	CXXC1	K14960
ENSG00000148660	0.25824	6.431782	0.695857	1	CAMK2G	K04515
ENSG00000138382	-0.258763	5.875131	0.695903	1	METTL5	_
ENSG00000179387	-0.270492	4.266851	0.695911	1	ELMOD2	_
ENSG00000198917	-0.262096	5.381633	0.696277	1	C9orf114	K01173
ENSG00000198839	0.260139	5.273112	0.696282	1	ZNF277	_
ENSG00000170791	-0.258226	5.803214	0.696332	1	CHCHD7	_
ENSG00000143457	-0.261462	5.463334	0.696367	1	GOLPH3L	K15620
ENSG00000107036	0.259546	5.363337	0.696378	1	KIAA1432	_
ENSG00000229124	0.333192	1.632724	0.696416	1	VIM-AS1	_
ENSG00000146963	-0.258517	6.047289	0.6965	1	C7orf55-LUC7L2	K13212
ENSG00000172939	-0.259573	5.841912	0.696545	1	OXSR1	K08835
ENSG00000153317	-0.258276	6.318242	0.696552	1	ASAP1	K12488
ENSG00000197586	-0.261768	5.222962	0.696616	1	ENTPD6	K01511
ENSG00000133193	0.262378	5.102134	0.69665	1	FAM104A	_
ENSG00000054690	0.277146	3.575227	0.6967	1	PLEKHH1	_
ENSG00000124171	-0.278291	3.18203	0.696788	1	PARD6B	K06093
ENSG00000226752	-0.278291	3.18203	0.696788	1	PSMD5-AS1	_
ENSG00000159445	-0.25984	5.298367	0.696806	1	THEM4	K16339
ENSG00000131504	0.255726	7.63882	0.696974	1	DIAPH1	K05740
ENSG00000132254	0.256128	7.164457	0.697031	1	ARFIP2	_
ENSG00000082438	-0.272344	3.524298	0.697067	1	COBLL1	_
ENSG00000120925	-0.272344	3.524298	0.697067	1	RNF170	K15707
ENSG00000163703	0.270881	4.069348	0.697075	1	CRELD1	_
ENSG00000123240	0.256581	6.754871	0.697117	1	OPTN	_
ENSG00000055483	0.258962	6.17104	0.697164	1	USP36	K11855
ENSG00000197296	0.290061	3.089374	0.697275	1	FITM2	K12581

ENSG00000172594	0.278671	3.094785	0.697275	1	SMPDL3A	K01128
ENSG00000214826	-0.2649	4.598757	0.697338	1	DDX12P	K11273
ENSG00000152193	-0.265244	4.863913	0.697436	1	RNF219	_
ENSG00000189280	-0.344399	1.444765	0.697454	1	GJB5	K07624
ENSG00000118564	-0.259101	5.189737	0.697531	1	FBXL5	K10271
ENSG00000153107	-0.256939	6.213528	0.697583	1	ANAPC1	K03348
ENSG00000148154	-0.258714	5.987099	0.697687	1	UGCG	K00720
ENSG00000124767	-0.254756	7.970395	0.697895	1	GLO1	K01759
ENSG00000102543	-0.27051	3.699795	0.697928	1	CDADC1	_
ENSG00000077348	0.259381	5.484988	0.69793	1	EXOSC5	K12590
ENSG00000261221	0.257662	5.40331	0.697932	1	ZNF865	K09228
ENSG00000141232	0.254403	8.254689	0.698012	1	TOB1	K14443
ENSG00000047188	-0.258216	5.920678	0.698166	1	YTHDC2	_
ENSG00000169891	0.265443	4.474778	0.698167	1	REPS2	K12581
ENSG00000118217	-0.257121	5.967635	0.698237	1	ATF6	K09054
ENSG00000171503	-0.269574	3.909697	0.698266	1	ETFDH	K00311
ENSG00000004059	-0.255552	6.843802	0.698342	1	ARF5	K07940
ENSG00000154803	0.269194	4.079787	0.698522	1	FLCN	K09594
ENSG00000128731	0.256656	6.034357	0.698522	1	HERC2	K10595
ENSG00000179406	0.378046	1.130619	0.698707	1	LINC00174	_
ENSG00000258586	0.378046	1.130619	0.698707	1	RP5-1021I20.2	_
ENSG00000131471	0.331	1.153525	0.698707	1	AOC3	K00276
ENSG00000240036	0.331	1.153525	0.698707	1	RP11-587D21.1	K02975
ENSG00000272599	0.331	1.153525	0.698707	1	RP11-152N13.16	_
ENSG00000148120	0.265403	4.685652	0.698707	1	C9orf3	K09606
ENSG00000129292	-0.260127	5.597853	0.698743	1	PHF20L1	K18402
ENSG00000113583	-0.255518	6.608199	0.69882	1	C5orf15	_
ENSG00000169223	0.253746	7.964939	0.698943	1	LMAN2	K10082
ENSG00000081870	-0.257212	6.257706	0.69895	1	HSPB11	K19369
ENSG00000065970	0.258693	5.25253	0.699098	1	FOXJ2	K09403
ENSG00000213523	-0.257128	5.139663	0.699336	1	SRA1	_
ENSG00000105948	-0.268431	4.456372	0.699513	1	TTC26	K19685
ENSG00000006432	0.268177	3.949484	0.699514	1	MAP3K9	K04417
ENSG00000007866	0.26676	4.278369	0.699708	1	TEAD3	K09448
ENSG00000084093	-0.259518	5.078752	0.699833	1	REST	K09222
ENSG00000165494	-0.257821	5.820371	0.699847	1	PCF11	K14400
ENSG00000185880	-0.264109	4.253078	0.700009	1	TRIM69	_
ENSG00000197728	-0.257488	5.220608	0.700051	1	RPS26	K02976
ENSG00000032444	0.254395	6.483719	0.700075	1	PNPLA6	K14676
ENSG00000178921	-0.256659	5.942213	0.700091	1	PFAS	K01952
ENSG00000100596	-0.261021	4.854843	0.700156	1	SPTLC2	K00654
ENSG00000174171	0.419542	0.79418	0.700163	1	RP11-23P13.6	_
ENSG00000183208	0.419542	0.79418	0.700163	1	GDPGP1	K15630
ENSG00000198003	0.419542	0.79418	0.700163	1	CCDC151	K17639
ENSG00000213240	0.419542	0.79418	0.700163	1	NOTCH2NL	_
ENSG00000228283	0.419542	0.79418	0.700163	1	KATNBL1P6	_
ENSG00000271361	0.419542	0.79418	0.700163	1	HTATSF1P2	_
ENSG00000120949	-0.405956	0.830285	0.700163	1	TNFRSF8	K05145
ENSG00000229334	-0.405956	0.830285	0.700163	1	AC046143.3	_
ENSG00000156500	0.273985	3.403455	0.700181	1	FAM122C	K17985
ENSG00000135070	-0.256908	5.884063	0.700182	1	ISCA1	K13628
ENSG00000181610	-0.254442	6.531673	0.70019	1	MRPS23	K17402
ENSG00000076351	-0.650888	-0.265519	0.700311	1	SLC46A1	K14613
ENSG00000110944	-0.650888	-0.265519	0.700311	1	IL23A	K05426
ENSG00000135424	-0.650888	-0.265519	0.700311	1	ITGA7	K06583
ENSG00000155984	-0.650888	-0.265519	0.700311	1	TMEM185A	_
ENSG00000164418	-0.650888	-0.265519	0.700311	1	GRIK2	K05202
ENSG00000167971	-0.650888	-0.265519	0.700311	1	CASKIN1	_

ENSG00000169946	-0.650888	-0.265519	0.700311	1	ZFPM2	K17442
ENSG00000208772	-0.650888	-0.265519	0.700311	1	SNORD94	_
ENSG00000212237	-0.650888	-0.265519	0.700311	1	RNA5SP18	_
ENSG00000212673	-0.650888	-0.265519	0.700311	1	AL136115.1	_
ENSG00000228409	-0.650888	-0.265519	0.700311	1	CCT6P1	K09498
ENSG00000240303	-0.650888	-0.265519	0.700311	1	ACAD11	K11730
ENSG00000240906	-0.650888	-0.265519	0.700311	1	AP000356.1	_
ENSG00000251661	-0.650888	-0.265519	0.700311	1	RP11-326C3.11	_
ENSG00000262194	-0.650888	-0.265519	0.700311	1	CTD-3195I5.5	_
ENSG00000264109	-0.650888	-0.265519	0.700311	1	MIR4712	_
ENSG00000271754	-0.650888	-0.265519	0.700311	1	RP3-337H4.9	_
ENSG00000273192	-0.650888	-0.265519	0.700311	1	CITF22-1A6.3	_
ENSG00000168806	-0.267868	4.213509	0.700325	1	LCMT2	K15451
ENSG00000111581	-0.253157	7.294995	0.700377	1	NUP107	K14301
ENSG00000198626	-0.331131	2.086217	0.700494	1	RYR2	K04962
ENSG00000240489	-0.331131	2.086217	0.700494	1	SETP14	K11290
ENSG00000135046	-0.251958	8.945117	0.700499	1	ANXA1	K17091
ENSG00000094975	-0.264612	4.699984	0.700594	1	SUCO	_
ENSG00000253719	-0.257524	5.678756	0.700626	1	ATXN7L3B	K11363
ENSG00000213199	0.269415	3.021282	0.700768	1	ASIC3	K04830
ENSG00000197256	0.257711	5.546405	0.700778	1	KANK2	K15225
ENSG00000147676	-0.252769	7.251963	0.70087	1	MAL2	_
ENSG00000072401	-0.256243	5.651128	0.700874	1	UBE2D1	K06689
ENSG00000234664	-0.264754	4.135854	0.701007	1	HMGN2P5	K11300
ENSG00000175550	-0.2533	6.765853	0.701017	1	DRAP1	_
ENSG00000166225	0.258633	4.750727	0.70104	1	FRS2	K12461
ENSG00000052126	-0.258828	5.10699	0.701085	1	PLEKHA5	_
ENSG00000183856	0.254643	6.002519	0.701155	1	IQGAP3	K05767
ENSG00000197063	0.253161	6.0032	0.701155	1	MAFG	K09037
ENSG00000136045	-0.253268	6.692362	0.701189	1	PWP1	K14791
ENSG00000205269	-0.2593	4.833485	0.701222	1	TMEM170B	_
ENSG00000105516	0.258721	4.492481	0.701426	1	DBP	K09056
ENSG00000187778	-0.254512	6.352429	0.70167	1	MCRS1	K11674
ENSG00000029364	0.254881	5.565073	0.701676	1	SLC39A9	K14715
ENSG00000074695	0.25098	8.627014	0.701687	1	LMAN1	K10080
ENSG00000163528	-0.259922	4.785051	0.701692	1	CHCHD4	K17782
ENSG00000213015	0.257201	4.781389	0.701692	1	ZNF580	_
ENSG00000142227	0.252621	6.68061	0.701705	1	EMP3	_
ENSG00000261705	-0.311052	2.519958	0.701744	1	RP11-61A14.2	_
ENSG00000104154	-0.296862	2.51181	0.701744	1	SLC30A4	K14691
ENSG00000229036	-0.296862	2.51181	0.701744	1	RP1-20N2.6	_
ENSG00000107104	-0.255456	5.110792	0.701791	1	KANK1	_
ENSG00000256967	0.310067	1.879438	0.701882	1	RP11-273B20.1	_
ENSG00000184154	-0.304219	1.920622	0.701882	1	LRTOMT	_
ENSG00000099821	-0.251987	7.15233	0.701886	1	POLRMT	K10908
ENSG00000047634	0.253797	5.961534	0.701962	1	SCML1	K11464
ENSG00000081052	0.258247	5.209274	0.702193	1	COL4A4	K06237
ENSG00000110218	-0.260588	4.734935	0.702194	1	PANX1	K03443
ENSG00000099399	-0.254257	5.270259	0.702283	1	MAGEB2	_
ENSG00000131652	-0.254344	5.324834	0.702365	1	THOC6	K13175
ENSG00000134352	-0.251555	7.145232	0.702383	1	IL6ST	K05060
ENSG00000148300	0.287818	2.842777	0.702412	1	REXO4	K18327
ENSG00000105677	-0.251661	6.988478	0.702493	1	TMEM147	_
ENSG00000164253	-0.252572	6.257718	0.702638	1	WDR41	_
ENSG00000156052	0.252561	6.043697	0.702712	1	GNAQ	K04634
ENSG00000077514	0.252274	6.246986	0.702733	1	POLD3	K03504
ENSG00000160959	-0.252721	5.968342	0.702743	1	LRRC14	K10730
ENSG00000176095	0.254979	5.133756	0.702983	1	IP6K1	K07756

ENSG00000134138	-0.264566	3.932778	0.703076	1	MEIS2	K16670
ENSG00000178982	-0.250164	7.968672	0.703085	1	EIF3K	K15028
ENSG00000080189	-0.257066	5.311661	0.703156	1	SLC35C2	K15280
ENSG00000134250	0.250362	7.500155	0.703162	1	NOTCH2	K02599
ENSG00000178974	0.253253	5.362274	0.703204	1	FBXO34	K10311
ENSG00000109861	-0.250027	7.892002	0.703302	1	CTSC	K01275
ENSG00000196422	-0.254132	5.911981	0.703344	1	PPP1R26	K17565
ENSG00000064687	0.250994	6.316943	0.703625	1	ABCA7	K05645
ENSG00000113273	0.277712	3.037196	0.703665	1	ARSB	K01135
ENSG00000136770	0.252444	6.123324	0.703733	1	DNAJC1	K09521
ENSG00000100567	-0.250852	6.78585	0.703742	1	PSMA3	K02727
ENSG00000156232	-0.282756	3.289153	0.703833	1	WHAMM	_
ENSG00000233966	0.268071	3.278016	0.703833	1	UBE2SP1	K10583
ENSG00000164305	-0.253231	5.057848	0.703863	1	CASP3	K02187
ENSG00000136819	-0.253022	6.041327	0.703932	1	C9orf78	K17985
ENSG00000140553	0.250511	6.768202	0.703938	1	UNC45A	_
ENSG00000106546	-0.254086	5.736591	0.703951	1	AHR	K09093
ENSG00000069974	0.255225	4.89642	0.704057	1	RAB27A	K07885
ENSG00000197037	-0.265212	3.98353	0.704139	1	ZSCAN25	K09229
ENSG00000150779	-0.254375	5.224124	0.704148	1	TIMM8B	K17780
ENSG00000141076	0.254238	4.44291	0.704254	1	CIRH1A	K14548
ENSG00000176834	-0.260353	4.720051	0.704301	1	VSIG10	_
ENSG00000148399	-0.257275	4.797697	0.704313	1	DPH7	K17868
ENSG00000243147	-0.251917	5.739056	0.704405	1	MRPL33	K02913
ENSG00000134602	-0.249267	7.517642	0.704515	1	MST4	K08838
ENSG00000110367	0.280473	2.95467	0.704603	1	DDX6	K12614
ENSG00000213753	-0.260301	4.513065	0.704618	1	CENPBD1P1	K17985
ENSG00000088888	0.259213	4.410215	0.704623	1	MAVS	K12648
ENSG00000196295	0.264053	3.784155	0.70465	1	AC005154.6	_
ENSG00000151348	0.251095	6.097905	0.704779	1	EXT2	K02367
ENSG00000074071	-0.248778	7.786838	0.704806	1	MRPS34	K17412
ENSG00000137522	-0.254511	4.483957	0.704978	1	RNF121	K15698
ENSG00000078246	0.254809	5.262862	0.704996	1	TULP3	K19600
ENSG00000175334	-0.248777	7.585811	0.704999	1	BANF1	_
ENSG00000166189	-0.252309	5.687286	0.705028	1	HPS6	_
ENSG00000083807	-0.260903	4.029824	0.70513	1	SLC27A5	K09229
ENSG00000143622	-0.261341	3.891998	0.705184	1	RIT1	K07832
ENSG00000077312	0.249197	6.804395	0.705365	1	SNRPA	K11091
ENSG00000173744	0.249211	6.620287	0.705663	1	AGFG1	K15044
ENSG00000093000	0.248597	7.046816	0.705686	1	NUP50	K14295
ENSG00000004975	0.250903	5.922971	0.705745	1	DVL2	K02353
ENSG00000100591	-0.248402	7.28494	0.705763	1	AHSA1	_
ENSG00000100290	0.337137	1.468701	0.705766	1	BIK	K18452
ENSG00000109771	0.337137	1.468701	0.705766	1	LRP2BP	K11447
ENSG00000187066	0.337137	1.468701	0.705766	1	TMEM262	_
ENSG00000204758	-0.326651	1.507164	0.705766	1	CTC-308K20.1	_
ENSG00000254429	-0.297007	1.489911	0.705766	1	CTD-2562J17.7	_
ENSG00000259994	-0.297007	1.489911	0.705766	1	RP11-305E6.4	_
ENSG00000114125	-0.251811	5.664148	0.705833	1	RNF7	K10611
ENSG00000173575	0.249878	6.215419	0.705843	1	CHD2	K11367
ENSG00000143369	0.250957	4.666833	0.70587	1	ECM1	_
ENSG00000179361	-0.267244	3.682785	0.705889	1	ARID3B	_
ENSG00000164318	-0.251815	4.808661	0.706036	1	EGFLAM	_
ENSG00000150687	0.246725	9.993797	0.706108	1	PRSS23	K09627
ENSG00000186184	0.248347	6.909297	0.706164	1	POLR1D	K03020
ENSG00000100418	-0.249991	6.188538	0.706202	1	DESI1	_
ENSG00000115840	-0.258327	4.280345	0.706277	1	SLC25A12	K15105
ENSG00000180263	0.250038	5.044793	0.706291	1	FGD6	K05724

ENSG00000089050	-0.259264	4.554666	0.706323	1	RBBP9	K07002
ENSG00000163960	0.248403	6.062862	0.706355	1	UBXN7	_
ENSG00000146535	-0.253289	5.622073	0.706363	1	GNA12	K04346
ENSG00000137409	0.247093	8.054333	0.706408	1	MTCH1	K17885
ENSG00000159720	0.251147	5.720536	0.706543	1	ATP6V0D1	K02146
ENSG00000170310	-0.258647	4.040492	0.706586	1	STX8	K08501
ENSG00000013561	-0.249319	6.25084	0.706768	1	RNF14	K11971
ENSG00000170876	-0.249416	6.36153	0.706894	1	TMEM43	_
ENSG00000137494	0.257191	4.123372	0.706927	1	ANKRD42	K17593
ENSG00000171097	0.25385	4.982266	0.707133	1	CCBL1	K00816
ENSG00000162073	-0.249007	5.944362	0.707173	1	PAQR4	K06633
ENSG00000159388	0.253738	4.782998	0.707222	1	BTG2	K14443
ENSG00000174915	0.248878	6.022908	0.707275	1	PTDSS2	K08730
ENSG00000136868	-0.24785	6.59197	0.707502	1	SLC31A1	K14686
ENSG00000255302	-0.246415	7.613601	0.707646	1	EID1	K17449
ENSG00000174442	-0.2475	6.720903	0.707648	1	ZWILCH	K11579
ENSG00000165304	-0.247158	6.944006	0.707653	1	MELK	K08799
ENSG00000104497	-0.271198	3.380166	0.707692	1	SNX16	K17928
ENSG00000172339	0.270275	3.374202	0.707692	1	ALG14	K07441
ENSG00000119596	-0.253391	4.816462	0.707739	1	YLPM1	K17602
ENSG00000185222	-0.246515	7.376899	0.707789	1	WBP5	_
ENSG00000117751	-0.249353	5.658957	0.707913	1	PPP1R8	K13216
ENSG00000159753	0.301076	1.926646	0.707955	1	RLTPR	_
ENSG00000135378	0.262058	3.796869	0.70807	1	PRRG4	K12581
ENSG00000224597	0.262058	3.796869	0.70807	1	PTCHD3P1	_
ENSG00000116685	0.247197	6.539041	0.708092	1	KIAA2013	_
ENSG00000131153	-0.249096	5.744789	0.708169	1	GINS2	K10733
ENSG00000106993	-0.256745	4.845716	0.708237	1	CDC37L1	_
ENSG00000157800	-0.24935	5.443335	0.708278	1	SLC37A3	K03447
ENSG00000119682	-0.252343	4.896594	0.70838	1	AREL1	_
ENSG00000180370	-0.247022	6.591519	0.708439	1	PAK2	K04410
ENSG00000122966	0.247333	5.616091	0.708505	1	CIT	K16308
ENSG00000033800	0.251419	4.94142	0.708513	1	PIAS1	K04706
ENSG00000163682	-0.245341	8.011937	0.708517	1	RPL9	K02940
ENSG00000168769	-0.258539	3.315342	0.708627	1	TET2	_
ENSG00000065057	-0.250348	5.322643	0.708807	1	NTHL1	K10773
ENSG00000124104	-0.277212	2.894194	0.708834	1	SNX21	K17932
ENSG00000172239	-0.244787	8.251706	0.708979	1	PAIP1	K14322
ENSG00000090615	0.246081	6.713592	0.70904	1	GOLGA3	_
ENSG00000119943	0.301254	2.263874	0.709088	1	PYROXD2	_
ENSG00000180423	0.280838	2.273774	0.709088	1	HARBI1	K12581
ENSG00000237214	0.246712	5.951624	0.709111	1	RP11-12M9.3	K02997
ENSG00000134690	-0.2473	5.606875	0.709199	1	CDCA8	K11514
ENSG00000020577	0.253318	4.502233	0.709211	1	SAMD4A	_
ENSG00000099256	-0.257971	4.259905	0.709234	1	PRTFDC1	_
ENSG00000214087	-0.250818	5.108245	0.709261	1	ARL16	K09228
ENSG00000165792	-0.246878	5.653747	0.709533	1	METTL17	_
ENSG00000175087	-0.255257	3.706309	0.709539	1	PDIK1L	K17539
ENSG00000087460	0.243562	10.32122	0.70965	1	GNAS	K04632
ENSG00000134852	-0.256138	4.476114	0.709671	1	CLOCK	_
ENSG00000132953	-0.249916	5.346527	0.709732	1	XPO4	_
ENSG00000099949	-0.248024	5.499611	0.709738	1	LZTR1	_
ENSG00000162664	-0.24682	6.274807	0.709758	1	ZNF326	K13204
ENSG00000108786	0.284528	2.545346	0.709771	1	HSD17B1	K00044
ENSG00000143995	-0.282556	2.57164	0.709771	1	MEIS1	K15613
ENSG00000174928	-0.268772	2.563791	0.709771	1	C3orf33	K08857
ENSG00000158169	-0.253098	4.802399	0.709795	1	FANCC	K10890
ENSG00000041988	0.253839	4.599601	0.7099	1	THAP3	_

ENSG00000114331	-0.247361	5.980891	0.70992	1	ACAP2	K12489
ENSG00000197798	0.262909	3.909213	0.709934	1	FAM118B	_
ENSG00000089154	0.244056	7.940965	0.709935	1	GCN1L1	_
ENSG00000167778	0.24855	5.108539	0.70995	1	SPRYD3	_
ENSG00000139842	0.243499	8.701106	0.710138	1	CUL4A	K10609
ENSG00000179833	0.245372	6.286314	0.710233	1	SERTAD2	_
ENSG00000132300	-0.244468	7.241039	0.710276	1	PTCD3	K17659
ENSG00000139625	0.261068	3.761721	0.710514	1	MAP3K12	K04423
ENSG00000184014	0.244498	6.879386	0.710568	1	DENND5A	_
ENSG00000148484	-0.248583	5.331373	0.710584	1	RSU1	_
ENSG00000198900	-0.245238	6.505424	0.710637	1	TOP1	K03163
ENSG00000084731	0.257333	3.867791	0.71065	1	KIF3C	K10394
ENSG00000150787	-0.247456	5.825028	0.710682	1	PTS	K01737
ENSG00000079785	-0.243673	7.680911	0.710691	1	DDX1	K13177
ENSG00000106086	-0.251469	4.856323	0.710705	1	PLEKHA8	K08051
ENSG00000145817	-0.244933	6.31828	0.710722	1	YIPF5	K17985
ENSG00000125912	-0.244198	7.109231	0.71076	1	NCLN	_
ENSG00000111331	-0.247346	5.703354	0.710761	1	OAS3	K14216
ENSG00000087995	-0.249387	5.236953	0.710777	1	METTL2A	K15696
ENSG00000176842	-0.269367	3.176999	0.710784	1	IRX5	_
ENSG00000164062	-0.244273	6.990577	0.710849	1	APEH	K01303
ENSG00000138764	0.254154	4.14341	0.71096	1	CCNG2	K10146
ENSG00000170113	0.245008	6.086307	0.710961	1	NIPA1	K19364
ENSG00000177463	0.262377	3.313867	0.710968	1	NR2C2	K08544
ENSG00000169251	-0.243667	7.393942	0.710999	1	NMD3	K07562
ENSG00000145545	-0.245053	6.430714	0.71101	1	SRD5A1	K12343
ENSG00000024862	-0.261007	3.465797	0.711114	1	CCDC28A	_
ENSG00000198538	-0.263024	3.602009	0.711232	1	ZNF28	K09228
ENSG00000111144	-0.243691	7.162799	0.711261	1	LTA4H	K01254
ENSG00000205581	-0.242656	8.459624	0.711277	1	HMGN1	K11299
ENSG00000077238	0.247402	5.472188	0.711283	1	IL4R	K05071
ENSG00000166348	0.24799	4.511918	0.711284	1	USP54	K11447
ENSG00000127191	0.24379	6.868528	0.711387	1	TRAF2	K03173
ENSG00000130396	0.247018	5.554833	0.711593	1	MLLT4	K05702
ENSG00000197780	-0.243897	5.991265	0.711792	1	TAF13	K03127
ENSG00000213463	-0.282599	2.698744	0.711807	1	SYNJ2BP	_
ENSG00000188342	-0.244231	6.003607	0.711981	1	GTF2F2	K03139
ENSG00000177731	-0.242579	7.598556	0.712014	1	FLII	_
ENSG00000139112	0.242214	7.943811	0.712025	1	GABARAPL1	K08341
ENSG00000144158	0.244929	5.763191	0.71213	1	RP11-395L14.17	K11092
ENSG00000174437	0.241852	8.31556	0.7122	1	ATP2A2	K05853
ENSG00000120008	-0.244162	6.344395	0.712206	1	WDR11	_
ENSG00000129566	-0.26387	3.880173	0.712245	1	TEP1	K11127
ENSG00000175066	-0.250954	4.315922	0.712295	1	GK5	K19583
ENSG00000148688	-0.248816	5.341132	0.712341	1	RPP30	K03539
ENSG00000100612	-0.246839	5.34005	0.712341	1	DHRS7	K11165
ENSG00000108771	-0.37392	0.924574	0.71239	1	DHX58	K12649
ENSG00000232093	-0.37392	0.924574	0.71239	1	RP11-307C12.11	_
ENSG00000259250	-0.37392	0.924574	0.71239	1	RP11-50C13.1	_
ENSG00000166455	-0.32836	0.897896	0.71239	1	C16orf46	_
ENSG00000079246	-0.241576	8.625983	0.712418	1	XRCC5	K10885
ENSG00000205544	-0.245965	5.848028	0.712553	1	TMEM256	_
ENSG00000156990	-0.245364	5.625631	0.712585	1	RPUSD3	_
ENSG00000233476	0.242171	7.31178	0.712646	1	EEF1A1P6	K03231
ENSG00000127564	-0.243548	6.458879	0.712651	1	PKMYT1	K06633
ENSG00000083312	-0.241736	7.942958	0.712665	1	TNPO1	K18752
ENSG00000134986	0.2511	4.351273	0.712871	1	NREP	_
ENSG00000117335	0.241022	8.884508	0.712882	1	CD46	K04007

ENSG00000131873	0.243514	5.923682	0.712932	1	CHSY1	K13499
ENSG00000151503	0.242805	6.549844	0.713046	1	NCAPD3	K11491
ENSG00000213300	-0.264702	3.726446	0.713101	1	HNRNPA3P6	K12741
ENSG00000163961	0.247623	4.866659	0.713131	1	RNF168	K12581
ENSG00000156802	0.241305	7.834181	0.71314	1	ATAD2	K06115
ENSG00000109670	0.258779	3.597441	0.713151	1	FBXW7	_
ENSG00000137692	-0.244212	6.174671	0.713169	1	DCUN1D5	K17824
ENSG00000148481	0.247097	4.762145	0.713188	1	FAM188A	_
ENSG00000099984	0.265535	3.174975	0.713345	1	GSTT2	K00799
ENSG00000214293	0.265535	3.174975	0.713345	1	RSBN1L-AS1	_
ENSG00000138613	0.272986	2.99942	0.713429	1	APH1B	K06172
ENSG00000115042	-0.246923	4.994653	0.71344	1	FAHD2A	_
ENSG00000128928	-0.243181	5.987819	0.713466	1	IVD	K00253
ENSG00000129084	-0.241123	7.811463	0.713471	1	PSMA1	K02725
ENSG00000162129	0.246515	4.894973	0.713512	1	CLPB	K03695
ENSG00000172878	0.28269	2.799493	0.713524	1	METAP1D	K01265
ENSG00000175104	-0.271063	2.808958	0.713524	1	TRAF6	K03175
ENSG00000067064	-0.241505	7.325412	0.713535	1	IDI1	K01823
ENSG00000016391	0.295852	2.567317	0.713627	1	CHDH	K00108
ENSG00000174007	0.295852	2.567317	0.713627	1	CEP19	K16801
ENSG00000214029	0.279395	2.575238	0.713627	1	ZNF891	K09228
ENSG00000114648	-0.261628	4.077327	0.713658	1	KLHL18	K10455
ENSG00000168282	-0.290661	2.32267	0.71373	1	MGAT2	K00736
ENSG00000206527	-0.241159	7.497218	0.713733	1	PTPLB	K10703
ENSG00000131848	-0.31391	1.977994	0.713789	1	ZSCAN5A	K11667
ENSG00000117262	-0.2929	1.965842	0.713789	1	GPR89A	_
ENSG00000108651	-0.242083	6.714263	0.713797	1	UTP6	K14557
ENSG00000138430	-0.241294	7.26603	0.713848	1	OLA1	K06942
ENSG00000185252	-0.26544	3.784853	0.713924	1	ZNF74	K09228
ENSG00000235106	-0.26544	3.784853	0.713924	1	LINC00094	_
ENSG00000122512	-0.247813	4.874347	0.713932	1	PMS2	K10858
ENSG00000058056	-0.245744	5.133416	0.714016	1	USP13	K11836
ENSG00000146834	-0.243557	5.978816	0.714035	1	MEPCE	K15190
ENSG00000090273	-0.240848	7.516249	0.714066	1	NUDC	_
ENSG00000148926	-0.241766	6.689861	0.714203	1	ADM	K12333
ENSG00000168286	-0.24382	5.44031	0.714246	1	THAP11	K11512
ENSG00000197324	0.241697	6.33552	0.714255	1	LRP10	K02927
ENSG00000140044	-0.244697	5.404559	0.714291	1	JDP2	K09033
ENSG00000144120	-0.248594	4.532058	0.71434	1	TMEM177	_
ENSG00000041357	-0.239972	8.271857	0.714441	1	PSMA4	K02728
ENSG00000103653	-0.242363	5.604178	0.714529	1	CSK	K05728
ENSG00000119559	-0.253179	4.326896	0.714635	1	C19orf25	K08671
ENSG00000171703	-0.245869	5.094196	0.714752	1	TCEA2	K03145
ENSG00000254999	0.241087	6.201743	0.714759	1	BRK1	K05752
ENSG00000197375	0.246075	5.275396	0.714771	1	SLC22A5	K08202
ENSG00000086065	0.239852	7.762587	0.714851	1	CHMP5	K12198
ENSG00000133858	0.240882	6.684414	0.714986	1	ZFC3H1	_
ENSG00000092140	-0.244144	5.115833	0.715054	1	G2E3	_
ENSG00000213949	-0.240142	7.312523	0.7151	1	ITGA1	K06480
ENSG00000137807	-0.240277	7.186466	0.715106	1	KIF23	K17387
ENSG00000134086	-0.244867	5.544418	0.715127	1	VHL	K03871
ENSG00000198912	-0.253978	4.365674	0.715145	1	C1orf174	K17985
ENSG00000138658	0.247143	4.287547	0.715287	1	C4orf21	_
ENSG00000163950	0.24009	7.069184	0.715305	1	SLBP	K18710
ENSG00000065183	-0.240599	6.80129	0.715327	1	WDR3	K14556
ENSG00000177602	0.250652	4.466953	0.71548	1	GSG2	K16315
ENSG00000137776	-0.24033	6.86243	0.715528	1	SLTM	_
ENSG00000166902	-0.240842	6.547259	0.715531	1	MRPL16	K02878

ENSG00000171612	-0.255599	4.130775	0.71562	1	SLC25A33	K15116
ENSG00000114030	0.240411	6.100443	0.715622	1	KPNA1	K15042
ENSG00000148498	0.240418	6.206505	0.715711	1	PARD3	K04237
ENSG00000167193	0.239812	6.928132	0.715817	1	CRK	K04438
ENSG00000067208	-0.244226	5.076125	0.715828	1	EVI5	_
ENSG00000188613	-0.260963	3.679278	0.715845	1	NANOS1	K18741
ENSG00000059691	-0.247024	4.667515	0.716024	1	PET112	K02434
ENSG00000164081	0.244475	4.663404	0.716024	1	TEX264	_
ENSG00000124177	0.243505	5.429021	0.716169	1	CHD6	K14436
ENSG00000163161	-0.240428	6.459387	0.716188	1	ERCC3	K10843
ENSG00000174099	-0.241819	5.77311	0.716235	1	MSRB3	K07305
ENSG00000241015	-0.291637	2.155305	0.716288	1	TPM3P9	K09290
ENSG00000100311	0.244656	5.007076	0.716354	1	PDGFB	K17386
ENSG00000028203	-0.242494	5.719245	0.716387	1	VEZT	_
ENSG00000180900	-0.244689	4.696518	0.7164	1	SCRIB	K16175
ENSG00000124383	-0.242227	6.061822	0.716423	1	MPHOSPH10	K14559
ENSG00000117625	-0.247318	4.479991	0.716522	1	RCOR3	K11829
ENSG00000125991	0.238304	7.83628	0.716555	1	ERGIC3	_
ENSG00000120685	-0.241466	5.288295	0.716568	1	PROSER1	_
ENSG00000129003	0.242125	5.467235	0.716607	1	VPS13C	K19525
ENSG00000128567	-0.237997	8.406031	0.71661	1	PODXL	K06817
ENSG00000166181	-0.238633	7.42769	0.716682	1	API5	_
ENSG00000164008	-0.265593	2.833633	0.716751	1	C1orf50	_
ENSG00000175634	-0.242164	5.882601	0.716756	1	RPS6KB2	K04688
ENSG00000159202	-0.23881	7.200241	0.716757	1	UBE2Z	K10585
ENSG00000118777	-0.239522	6.665909	0.716795	1	ABCG2	K05681
ENSG00000144589	-0.266189	3.279825	0.716872	1	STK11IP	_
ENSG00000157954	0.239252	6.646345	0.716904	1	WIPI2	K17908
ENSG00000134265	-0.248179	4.514911	0.716937	1	NAPG	_
ENSG00000146066	0.239458	6.263619	0.716968	1	HIGD2A	_
ENSG00000213619	-0.239021	6.841578	0.71705	1	NDUFS3	K03936
ENSG00000143126	0.240294	6.119512	0.717111	1	CELSR2	K04601
ENSG00000136521	-0.239187	6.667635	0.717173	1	NDUFB5	K03961
ENSG00000180979	-0.251562	4.048386	0.717213	1	LRRC57	K02927
ENSG00000142864	-0.23693	10.3048	0.717226	1	SERBP1	K13199
ENSG00000125124	-0.241972	5.439301	0.717244	1	BBS2	K16747
ENSG00000167987	-0.248521	3.803586	0.717256	1	VPS37C	K12185
ENSG00000168036	-0.237416	8.255336	0.717363	1	CTNNB1	K02105
ENSG00000156508	0.236595	13.04456	0.717418	1	EEF1A1	K03231
ENSG00000158092	0.238619	6.724815	0.717489	1	NCK1	K07365
ENSG00000185298	-0.240142	6.238153	0.717526	1	CCDC137	K09228
ENSG00000136156	0.237174	8.264191	0.717557	1	ITM2B	K18264
ENSG00000055130	-0.238151	7.107353	0.717632	1	CUL1	K03347
ENSG00000181625	-0.480515	0.467836	0.717697	1	SLX1B	K15078
ENSG00000225159	-0.480515	0.467836	0.717697	1	NPM1P39	K11276
ENSG00000240889	-0.480515	0.467836	0.717697	1	NDUFB2-AS1	_
ENSG00000130305	0.407335	0.39556	0.717697	1	NSUN5	K15264
ENSG00000149571	0.407335	0.39556	0.717697	1	KIRREL3	_
ENSG00000166813	0.407335	0.39556	0.717697	1	KIF7	K18806
ENSG00000180769	0.407335	0.39556	0.717697	1	WDFY3-AS2	_
ENSG00000187186	0.407335	0.39556	0.717697	1	RP11-195F19.5	K16598
ENSG00000233224	0.407335	0.39556	0.717697	1	HIST1H2AM	K11251
ENSG00000267475	0.407335	0.39556	0.717697	1	CTD-2538C1.2	_
ENSG00000008086	0.325658	0.434874	0.717697	1	CDKL5	K08824
ENSG00000138735	0.325658	0.434874	0.717697	1	PDE5A	K13762
ENSG00000151320	0.325658	0.434874	0.717697	1	AKAP6	K16523
ENSG00000182600	0.325658	0.434874	0.717697	1	C2orf82	_
ENSG00000204710	0.325658	0.434874	0.717697	1	SPDYC	K08694

ENSG00000153560	-0.238582	5.938678	0.717749	1	UBP1	K09275
ENSG00000152404	-0.243819	4.41573	0.717805	1	CWF19L2	_
ENSG00000009790	-0.351849	1.291417	0.717848	1	TRAF3IP3	_
ENSG00000011332	-0.351849	1.291417	0.717848	1	DPF1	K13196
ENSG00000245571	-0.351849	1.291417	0.717848	1	AP001258.4	_
ENSG00000260912	-0.351849	1.291417	0.717848	1	RP11-363E7.4	_
ENSG00000179954	-0.317224	1.271154	0.717848	1	SSC5D	_
ENSG00000246228	-0.317224	1.271154	0.717848	1	CASC8	_
ENSG00000023697	-0.239878	5.460349	0.717979	1	DERA	K01619
ENSG00000101193	0.239514	5.457277	0.717979	1	GID8	_
ENSG00000108557	0.239137	6.14579	0.718028	1	RAI1	K19749
ENSG00000204977	-0.245692	5.041961	0.718057	1	TRIM13	K12003
ENSG00000170190	-0.248251	4.010788	0.718079	1	SLC16A5	K08182
ENSG00000187098	-0.240645	5.425095	0.718113	1	MITF	K09455
ENSG00000142657	-0.238458	6.578773	0.718174	1	PGD	K00033
ENSG00000138069	0.236854	7.838384	0.718206	1	RAB1A	K07874
ENSG00000087302	-0.237061	7.565201	0.718326	1	C14orf166	K15433
ENSG00000132466	0.238258	6.442865	0.718417	1	ANKRD17	K16726
ENSG00000139514	0.237901	6.578133	0.718563	1	SLC7A1	K13863
ENSG00000177426	-0.237747	6.766315	0.718629	1	TGIF1	K19383
ENSG00000196150	0.243095	4.18513	0.718708	1	ZNF250	K09228
ENSG00000197302	-0.288726	2.732324	0.718752	1	ZNF720	K09228
ENSG00000167565	-0.240723	4.767388	0.718807	1	SERTAD3	_
ENSG00000135457	-0.239289	5.761046	0.718893	1	TFCP2	K09275
ENSG00000158161	0.249654	3.958193	0.718997	1	EYA3	K17621
ENSG00000121289	-0.250485	4.105493	0.71908	1	CEP89	K16543
ENSG00000115947	-0.243886	4.878808	0.719144	1	ORC4	K02606
ENSG00000174177	-0.249072	4.234452	0.719148	1	CTU2	K14169
ENSG00000184675	-0.243939	4.350646	0.719205	1	AMER1	K19407
ENSG00000261188	0.250151	3.503981	0.719314	1	CTA-445C9.14	_
ENSG00000102804	-0.235426	8.85235	0.719325	1	TSC22D1	_
ENSG00000118816	-0.236396	7.288363	0.719394	1	CCNI	_
ENSG00000137975	0.267646	1.984617	0.719399	1	CLCA2	K05028
ENSG00000167004	0.235063	9.899185	0.719406	1	PDIA3	K08056
ENSG00000185721	-0.236812	6.930466	0.71942	1	DRG1	K16489
ENSG00000136718	-0.239416	5.857891	0.719484	1	IMP4	K14561
ENSG00000162517	-0.237643	6.009071	0.719518	1	PEF1	_
ENSG00000137054	-0.240145	5.469276	0.71955	1	POLR1E	K03005
ENSG00000136783	-0.25004	4.275762	0.719563	1	NIPSNAP3A	_
ENSG00000159792	-0.248808	4.390918	0.719587	1	PSKH1	K08808
ENSG00000163512	0.246113	4.385316	0.719587	1	AZI2	K12651
ENSG00000109270	-0.244968	4.388801	0.719587	1	LAMTOR3	K04370
ENSG00000108826	-0.238714	5.53689	0.719729	1	MRPL27	K02899
ENSG00000055208	0.237215	6.287978	0.719769	1	TAB2	K04404
ENSG00000133069	0.265785	2.46892	0.719779	1	TMCC2	K16288
ENSG00000186272	0.265785	2.46892	0.719779	1	ZNF17	K09228
ENSG00000037474	-0.234988	8.759578	0.719866	1	NSUN2	K15335
ENSG00000076053	0.240016	5.55109	0.719893	1	RBM7	K13188
ENSG00000205763	0.254348	3.366017	0.719973	1	RP9P	K19604
ENSG00000189159	-0.23687	6.567737	0.720003	1	HN1	_
ENSG00000106244	-0.235664	7.482378	0.720005	1	PDAP1	K05757
ENSG00000068745	0.239594	5.707141	0.720034	1	IP6K2	K07756
ENSG00000158864	-0.236013	7.111803	0.720061	1	NDUFS2	K03935
ENSG00000107874	-0.237078	6.424121	0.720072	1	CUEDC2	_
ENSG00000170260	0.246441	4.353457	0.720325	1	ZNF212	K09228
ENSG00000108423	-0.243761	4.113045	0.720404	1	TUBD1	K10390
ENSG00000257671	0.253282	3.812687	0.720512	1	RP3-416H24.1	_
ENSG00000197451	-0.234755	8.018936	0.720561	1	HNRNPAB	K13044

ENSG00000155876	0.237465	5.976922	0.720609	1	RRAGA	K16185
ENSG00000072042	-0.23605	6.701715	0.720677	1	RDH11	K11152
ENSG00000158435	-0.235946	6.732939	0.720738	1	CNOT11	_
ENSG00000205765	-0.236399	6.4594	0.720766	1	C5orf51	K05080
ENSG00000162980	-0.23671	5.527423	0.72106	1	ARL5A	K07949
ENSG00000167216	-0.338405	1.583299	0.721106	1	KATNAL2	K07767
ENSG00000180884	-0.338405	1.583299	0.721106	1	ZNF792	K09228
ENSG00000197714	-0.338405	1.583299	0.721106	1	ZNF460	K09228
ENSG00000197557	-0.310482	1.567006	0.721106	1	TTC30A	K19683
ENSG00000142235	0.249515	3.705806	0.721136	1	LMTK3	K08899
ENSG00000125834	0.237965	4.659967	0.721144	1	STK35	K08859
ENSG00000095321	0.240695	4.935726	0.721171	1	CRAT	K00624
ENSG00000169919	-0.236507	6.238743	0.721297	1	GUSB	K01195
ENSG00000109113	-0.234524	7.414161	0.72138	1	RAB34	K07921
ENSG00000042088	-0.246162	4.36136	0.721437	1	TDP1	K10862
ENSG00000145730	0.234505	7.21813	0.72148	1	PAM	K00504
ENSG00000161542	-0.237165	5.829671	0.721505	1	PRPSAP1	_
ENSG00000153561	0.235847	6.133328	0.721625	1	RMND5A	_
ENSG00000099999	0.254019	3.765049	0.721645	1	RNF215	_
ENSG00000164054	-0.23679	6.020591	0.721654	1	SHISA5	K10135
ENSG00000162434	0.233351	8.806591	0.721663	1	JAK1	K11217
ENSG00000146414	-0.253592	3.05531	0.721753	1	SHPRH	K15710
ENSG00000187231	0.241904	4.631892	0.721783	1	SESTD1	_
ENSG00000189046	-0.242757	4.838028	0.721801	1	ALKBH2	K10859
ENSG00000089737	0.234365	7.038428	0.721866	1	DDX24	K14805
ENSG00000166851	0.235041	6.537582	0.721893	1	PLK1	K06631
ENSG00000117906	-0.234087	7.264639	0.722056	1	RCN2	_
ENSG00000140326	0.239058	4.434916	0.722062	1	CDAN1	K19531
ENSG00000135870	-0.243024	4.1677	0.722085	1	RC3H1	K15690
ENSG00000156521	-0.236797	5.705043	0.722097	1	TYSND1	K10408
ENSG00000272086	0.267083	2.73557	0.722103	1	CTD-2186M15.3	_
ENSG00000172375	0.261007	3.378602	0.722139	1	C2CD2L	_
ENSG00000106459	-0.239392	4.328974	0.722233	1	NRF1	K11831
ENSG00000064042	0.233304	7.798493	0.722289	1	LIMCH1	_
ENSG00000122203	0.234413	6.712711	0.722298	1	KIAA1191	_
ENSG00000180773	-0.24009	4.476014	0.722351	1	SLC36A4	K14209
ENSG00000068912	-0.235815	6.370598	0.722378	1	ERLEC1	K14008
ENSG00000143569	0.233303	7.617623	0.722442	1	UBAP2L	_
ENSG00000148840	0.234482	6.562413	0.722483	1	PPRC1	K17963
ENSG00000032219	0.240588	4.364197	0.72254	1	ARID4A	K19194
ENSG00000008710	0.234186	6.714384	0.722555	1	PKD1	K04985
ENSG00000137166	0.237292	5.337525	0.722558	1	FOXP4	K09409
ENSG00000125650	0.268935	2.344925	0.722591	1	PSPN	_
ENSG00000196378	0.268935	2.344925	0.722591	1	ZNF34	K09228
ENSG00000132182	0.233165	7.526027	0.722684	1	NUP210	K14314
ENSG00000152749	-0.23977	5.044604	0.722705	1	GPR180	_
ENSG00000083099	-0.242116	4.545217	0.722888	1	LYRM2	_
ENSG00000106333	-0.271526	2.864235	0.722995	1	PCOLCE	_
ENSG00000186230	-0.271526	2.864235	0.722995	1	ZNF749	_
ENSG00000250565	-0.271526	2.864235	0.722995	1	ATP6V1E2	_
ENSG00000108588	0.232771	7.676964	0.722998	1	CCDC47	_
ENSG00000128342	-0.250999	4.128228	0.723017	1	LIF	K05419
ENSG00000089597	0.231987	9.442736	0.723021	1	GANAB	K05546
ENSG00000040341	-0.233987	5.838124	0.723109	1	STAU2	K17597
ENSG00000166889	0.236205	5.902542	0.723218	1	PATL1	K12617
ENSG00000124795	-0.231995	8.894221	0.72323	1	DEK	K17046
ENSG00000138231	-0.235289	5.284922	0.723254	1	DBR1	K18328
ENSG00000179674	-0.305963	1.812149	0.723265	1	ARL14	K17200

ENSG00000231767	-0.305963	1.812149	0.723265	1	MIR4426	K02977
ENSG00000100077	0.272519	1.808228	0.723265	1	ADRBK2	K00910
ENSG00000181061	-0.234197	6.40668	0.723389	1	HIGD1A	_
ENSG00000067248	-0.234754	5.958546	0.723593	1	DHX29	K18995
ENSG00000033011	-0.239621	4.518688	0.723618	1	ALG1	K03842
ENSG00000270143	0.336493	0.92013	0.723661	1	AC004257.3	_
ENSG00000111642	0.231634	8.587496	0.72372	1	CHD4	K11643
ENSG00000187164	-0.235327	5.773117	0.723824	1	KIAA1598	_
ENSG00000169607	-0.234365	5.654626	0.723858	1	CKAP2L	K16769
ENSG00000157349	-0.239301	4.259753	0.723942	1	DDX19B	K18655
ENSG00000206560	-0.234205	6.201136	0.723954	1	ANKRD28	K15502
ENSG00000171055	-0.234859	6.341724	0.724045	1	FEZ2	_
ENSG00000142002	0.235666	6.078512	0.724084	1	DPP9	K08656
ENSG00000151458	-0.242865	4.739786	0.724232	1	ANKRD50	_
ENSG00000112855	-0.23332	5.897389	0.724269	1	HARS2	K01892
ENSG00000159079	-0.23322	5.732498	0.724371	1	C21orf59	_
ENSG00000168936	-0.242153	4.485837	0.724379	1	TMEM129	_
ENSG00000080824	-0.23061	10.72221	0.724399	1	HSP90AA1	K04079
ENSG00000100888	0.236099	5.670542	0.724411	1	CHD8	K04494
ENSG00000139180	-0.234217	5.923608	0.724441	1	NDUFA9	K03953
ENSG00000099810	-0.232261	7.009792	0.724487	1	MTAP	K00772
ENSG00000236801	0.239678	4.515811	0.724604	1	RPL24P8	K02896
ENSG00000139323	-0.238901	4.934296	0.724691	1	POC1B	K16482
ENSG00000085377	-0.234521	6.025311	0.724723	1	PREP	K01322
ENSG00000153044	-0.232723	5.860919	0.724781	1	CENPH	K11500
ENSG00000188760	0.284776	2.016632	0.724796	1	TMEM198	_
ENSG00000185090	-0.251097	3.791011	0.724924	1	MANEAL	_
ENSG00000135968	-0.233075	5.706725	0.725043	1	GCC2	K12172
ENSG00000078814	0.517011	-0.214446	0.725047	1	MYH7B	K10352
ENSG00000171877	0.517011	-0.214446	0.725047	1	FRMD5	_
ENSG00000204169	0.517011	-0.214446	0.725047	1	AGAP7	K12491
ENSG00000233961	0.517011	-0.214446	0.725047	1	RP11-87H9.2	K13122
ENSG00000239246	0.517011	-0.214446	0.725047	1	RP11-464D20.2	K02947
ENSG00000253882	0.517011	-0.214446	0.725047	1	RP11-61L23.2	_
ENSG00000261126	0.517011	-0.214446	0.725047	1	RBFADN	_
ENSG00000267281	0.517011	-0.214446	0.725047	1	RP11-793H13.10	K09045
ENSG00000065621	-0.440356	-0.127832	0.725047	1	GSTO2	K00799
ENSG00000116819	-0.440356	-0.127832	0.725047	1	TFAP2E	K09179
ENSG00000143466	-0.440356	-0.127832	0.725047	1	IKBKE	K07211
ENSG00000187688	-0.440356	-0.127832	0.725047	1	TRPV2	K04971
ENSG00000188004	-0.440356	-0.127832	0.725047	1	C1orf204	_
ENSG00000196569	-0.440356	-0.127832	0.725047	1	LAMA2	K05637
ENSG00000200879	-0.440356	-0.127832	0.725047	1	SNORD14E	_
ENSG00000201512	-0.440356	-0.127832	0.725047	1	SNORA71C	_
ENSG00000214967	-0.440356	-0.127832	0.725047	1	NPIPA7	_
ENSG00000224080	-0.440356	-0.127832	0.725047	1	UBE2FP1	K10687
ENSG00000224195	-0.440356	-0.127832	0.725047	1	RP11-574K11.5	_
ENSG00000226580	-0.440356	-0.127832	0.725047	1	RPL39P40	K02924
ENSG00000230880	-0.440356	-0.127832	0.725047	1	RP11-417J8.3	_
ENSG00000235530	-0.440356	-0.127832	0.725047	1	AC087294.2	_
ENSG00000249855	-0.440356	-0.127832	0.725047	1	EEF1A1P19	K03231
ENSG00000254142	-0.440356	-0.127832	0.725047	1	RP11-53M11.3	_
ENSG00000255090	-0.440356	-0.127832	0.725047	1	RP11-820L6.1	_
ENSG00000257829	-0.440356	-0.127832	0.725047	1	RP11-845M18.6	_
ENSG00000272373	-0.440356	-0.127832	0.725047	1	ZNF33B	_
ENSG00000272902	-0.440356	-0.127832	0.725047	1	RP11-299H21.1	_
ENSG00000151239	-0.231204	7.528924	0.725048	1	TWF1	K08870
ENSG00000023041	-0.236045	5.480113	0.725061	1	ZDHHC6	K18932

ENSG00000141720	-0.233194	6.03937	0.725144	1	PIP4K2B	K00920
ENSG00000127580	0.236221	4.621259	0.725216	1	WDR24	_
ENSG00000108604	-0.233563	5.942253	0.725264	1	SMARCD2	K11650
ENSG00000180104	0.231182	7.202998	0.725293	1	EXOC3	K06110
ENSG00000162139	0.277235	2.754883	0.725376	1	NEU3	K12357
ENSG00000187534	0.277235	2.754883	0.725376	1	CTC-471F3.4	_
ENSG00000085365	0.234347	6.039661	0.725488	1	SCAMP1	_
ENSG00000109103	0.236659	4.985087	0.725718	1	UNC119	_
ENSG00000181744	-0.2392	4.18704	0.725828	1	C3orf58	_
ENSG00000198720	-0.233961	5.619412	0.725841	1	ANKRD13B	_
ENSG00000102181	0.272723	2.209285	0.725938	1	CD99L2	K06520
ENSG00000166197	-0.229612	8.934189	0.725942	1	NOLC1	_
ENSG00000114738	0.232473	5.551089	0.725951	1	MAPKAPK3	K04444
ENSG00000108264	-0.254857	3.332781	0.725968	1	TADA2A	K11314
ENSG00000087842	-0.239943	4.836485	0.726367	1	PIR	K06911
ENSG00000160062	0.240274	3.404079	0.726374	1	ZBTB8A	K10495
ENSG00000145375	-0.239695	4.053552	0.726389	1	SPATA5	K14575
ENSG00000170144	-0.229373	8.337717	0.726504	1	HNRNPA3	K12741
ENSG00000266401	0.342221	1.283759	0.726541	1	RP11-874J12.4	_
ENSG00000272668	0.300604	1.304195	0.726541	1	RP11-190A12.8	_
ENSG00000166845	-0.267438	2.990913	0.726567	1	C18orf54	_
ENSG00000182010	-0.257182	2.985132	0.726567	1	RTKN2	_
ENSG00000115760	0.232347	6.364023	0.726597	1	BIRC6	K10586
ENSG00000101558	0.229638	7.654897	0.7266	1	VAPA	K06096
ENSG00000188070	0.236812	4.717835	0.726603	1	C11orf95	_
ENSG00000160563	-0.241091	4.100302	0.726625	1	MED27	K15170
ENSG00000019991	-1.472476	-1.197592	0.726708	1	HGF	K05460
ENSG00000081923	-1.472476	-1.197592	0.726708	1	ATP8B1	K01530
ENSG00000120055	-1.472476	-1.197592	0.726708	1	C10orf95	_
ENSG00000135549	-1.472476	-1.197592	0.726708	1	PKIB	_
ENSG00000137507	-1.472476	-1.197592	0.726708	1	LRRC32	_
ENSG00000142449	-1.472476	-1.197592	0.726708	1	FBN3	K06825
ENSG00000151023	-1.472476	-1.197592	0.726708	1	ENKUR	_
ENSG00000154263	-1.472476	-1.197592	0.726708	1	ABCA10	K05652
ENSG00000172215	-1.472476	-1.197592	0.726708	1	CXCR6	K04191
ENSG00000172460	-1.472476	-1.197592	0.726708	1	PRSS30P	K09626
ENSG00000172508	-1.472476	-1.197592	0.726708	1	CARNS1	K14755
ENSG00000177640	-1.472476	-1.197592	0.726708	1	CASC2	_
ENSG00000183625	-1.472476	-1.197592	0.726708	1	CCR3	K04178
ENSG00000185105	-1.472476	-1.197592	0.726708	1	MYADML2	_
ENSG00000185862	-1.472476	-1.197592	0.726708	1	EVI2B	K16854
ENSG00000188383	-1.472476	-1.197592	0.726708	1	AC018892.9	K00629
ENSG00000188937	-1.472476	-1.197592	0.726708	1	NYX	K08129
ENSG00000197146	-1.472476	-1.197592	0.726708	1	AL133458.1	K17985
ENSG00000202379	-1.472476	-1.197592	0.726708	1	SNORA70	_
ENSG00000204982	-1.472476	-1.197592	0.726708	1	PRSS3P4	K01312
ENSG00000205791	-1.472476	-1.197592	0.726708	1	LOH12CR2	K17985
ENSG00000205898	-1.472476	-1.197592	0.726708	1	RP11-3B12.4	_
ENSG00000207561	-1.472476	-1.197592	0.726708	1	MIR635	_
ENSG00000208036	-1.472476	-1.197592	0.726708	1	MIR106B	_
ENSG00000209042	-1.472476	-1.197592	0.726708	1	SNORD12C	_
ENSG00000215146	-1.472476	-1.197592	0.726708	1	RP11-313J2.1	K09228
ENSG00000225075	-1.472476	-1.197592	0.726708	1	RP11-426L16.3	_
ENSG00000226887	-1.472476	-1.197592	0.726708	1	ERVMER34-1	_
ENSG00000228137	-1.472476	-1.197592	0.726708	1	AP001469.7	_
ENSG00000231826	-1.472476	-1.197592	0.726708	1	AC016735.2	_
ENSG00000232176	-1.472476	-1.197592	0.726708	1	RP11-146N23.1	K02991
ENSG00000236305	-1.472476	-1.197592	0.726708	1	RP11-126L15.4	_

ENSG00000238099	-1.472476	-1.197592	0.726708	1	RP11-12A2.3	_
ENSG00000240048	-1.472476	-1.197592	0.726708	1	DDX50P2	K13183
ENSG00000243811	-1.472476	-1.197592	0.726708	1	APOBEC3D	K18750
ENSG00000247735	-1.472476	-1.197592	0.726708	1	CTD-2574D22.2	_
ENSG00000248235	-1.472476	-1.197592	0.726708	1	AC037459.4	_
ENSG00000249637	-1.472476	-1.197592	0.726708	1	CTC-329D1.2	_
ENSG00000254634	-1.472476	-1.197592	0.726708	1	RP11-231C14.3	K08873
ENSG00000254815	-1.472476	-1.197592	0.726708	1	RP11-496I9.1	_
ENSG00000258017	-1.472476	-1.197592	0.726708	1	RP11-386G11.10	_
ENSG00000258122	-1.472476	-1.197592	0.726708	1	RP11-61A14.1	_
ENSG00000260075	-1.472476	-1.197592	0.726708	1	NSFP1	K06027
ENSG00000260252	-1.472476	-1.197592	0.726708	1	RP11-384M15.3	_
ENSG00000261147	-1.472476	-1.197592	0.726708	1	RP11-697E2.6	K16582
ENSG00000262904	-1.472476	-1.197592	0.726708	1	RP11-144N1.1	_
ENSG00000265148	-1.472476	-1.197592	0.726708	1	BZRAP1-AS1	_
ENSG00000267049	-1.472476	-1.197592	0.726708	1	AC002398.13	_
ENSG00000267096	-1.472476	-1.197592	0.726708	1	CTD-2537I9.13	_
ENSG00000270742	-1.472476	-1.197592	0.726708	1	RP4-802A10.1	_
ENSG00000272764	-1.472476	-1.197592	0.726708	1	RP11-318E3.9	_
ENSG00000272777	-1.472476	-1.197592	0.726708	1	RP11-571L19.8	_
ENSG00000060558	-1.217286	-1.328937	0.726708	1	GNA15	K04637
ENSG00000107831	-1.217286	-1.328937	0.726708	1	FGF8	K04358
ENSG00000115008	-1.217286	-1.328937	0.726708	1	IL1A	K04383
ENSG00000125538	-1.217286	-1.328937	0.726708	1	IL1B	K04519
ENSG00000128340	-1.217286	-1.328937	0.726708	1	RAC2	K07860
ENSG00000131378	-1.217286	-1.328937	0.726708	1	RFTN1	K07949
ENSG00000137331	-1.217286	-1.328937	0.726708	1	IER3	_
ENSG00000138829	-1.217286	-1.328937	0.726708	1	FBN2	K06825
ENSG00000156689	-1.217286	-1.328937	0.726708	1	GLYATL2	_
ENSG00000161643	-1.217286	-1.328937	0.726708	1	SIGLEC16	K06750
ENSG00000161835	-1.217286	-1.328937	0.726708	1	GRASP	_
ENSG00000162843	-1.217286	-1.328937	0.726708	1	WDR64	_
ENSG00000163596	-1.217286	-1.328937	0.726708	1	ICA1L	_
ENSG00000164867	-1.217286	-1.328937	0.726708	1	NOS3	K13242
ENSG00000166257	-1.217286	-1.328937	0.726708	1	SCN3B	K04847
ENSG00000166763	-1.217286	-1.328937	0.726708	1	STRCP1	_
ENSG00000168939	-1.217286	-1.328937	0.726708	1	SPRY3	K17384
ENSG00000170122	-1.217286	-1.328937	0.726708	1	FOXD4	K09397
ENSG00000173261	-1.217286	-1.328937	0.726708	1	PLAC8L1	_
ENSG00000174527	-1.217286	-1.328937	0.726708	1	MYO1H	K10356
ENSG00000176410	-1.217286	-1.328937	0.726708	1	DNAJC30	K19374
ENSG00000177807	-1.217286	-1.328937	0.726708	1	KCNJ10	K05003
ENSG00000181104	-1.217286	-1.328937	0.726708	1	F2R	K03914
ENSG00000186105	-1.217286	-1.328937	0.726708	1	LRRC70	_
ENSG00000187140	-1.217286	-1.328937	0.726708	1	FOXD3	K09397
ENSG00000196866	-1.217286	-1.328937	0.726708	1	HIST1H2AD	K11251
ENSG00000198542	-1.217286	-1.328937	0.726708	1	ITGBL1	_
ENSG00000199024	-1.217286	-1.328937	0.726708	1	MIR103A2	_
ENSG00000204256	-1.217286	-1.328937	0.726708	1	BRD2	K08871
ENSG00000207770	-1.217286	-1.328937	0.726708	1	MIR568	_
ENSG00000213693	-1.217286	-1.328937	0.726708	1	SEC14L1P1	_
ENSG00000219992	-1.217286	-1.328937	0.726708	1	RP11-420L9.2	K17986
ENSG00000221406	-1.217286	-1.328937	0.726708	1	MIR320B2	_
ENSG00000224272	-1.217286	-1.328937	0.726708	1	AC114730.3	_
ENSG00000225541	-1.217286	-1.328937	0.726708	1	AC002480.5	_
ENSG00000225568	-1.217286	-1.328937	0.726708	1	RP11-384B12.3	K10685
ENSG00000228043	-1.217286	-1.328937	0.726708	1	AC097721.2	_
ENSG00000228906	-1.217286	-1.328937	0.726708	1	RP13-216E22.4	_

ENSG00000229789	-1.217286	-1.328937	0.726708	1	RP11-391M20.1	K13122
ENSG00000230613	-1.217286	-1.328937	0.726708	1	HM13-AS1	_
ENSG00000231256	-1.217286	-1.328937	0.726708	1	C17orf105	_
ENSG00000231760	-1.217286	-1.328937	0.726708	1	RP11-350J20.5	_
ENSG00000232186	-1.217286	-1.328937	0.726708	1	RP11-311P8.2	K11110
ENSG00000232745	-1.217286	-1.328937	0.726708	1	ANKRD20A14P	K17299
ENSG00000233871	-1.217286	-1.328937	0.726708	1	DLG5-AS1	_
ENSG00000234937	-1.217286	-1.328937	0.726708	1	RP11-101O6.2	K03038
ENSG00000235677	-1.217286	-1.328937	0.726708	1	NPM1P26	K11276
ENSG00000235795	-1.217286	-1.328937	0.726708	1	RP11-421L21.2	_
ENSG00000236841	-1.217286	-1.328937	0.726708	1	AC007750.5	_
ENSG00000237037	-1.217286	-1.328937	0.726708	1	NDUFA6-AS1	_
ENSG00000237978	-1.217286	-1.328937	0.726708	1	RP11-385J1.2	_
ENSG00000238961	-1.217286	-1.328937	0.726708	1	SNORA47	_
ENSG00000239122	-1.217286	-1.328937	0.726708	1	RNU2-11P	_
ENSG00000242083	-1.217286	-1.328937	0.726708	1	RPL7AP31	K02936
ENSG00000244945	-1.217286	-1.328937	0.726708	1	RP11-1379J22.2	_
ENSG00000245017	-1.217286	-1.328937	0.726708	1	RP11-181C3.1	K09228
ENSG00000245293	-1.217286	-1.328937	0.726708	1	RP11-286E11.1	_
ENSG00000246982	-1.217286	-1.328937	0.726708	1	RP1-179N16.6	_
ENSG00000248664	-1.217286	-1.328937	0.726708	1	CTC-498J12.3	_
ENSG00000253704	-1.217286	-1.328937	0.726708	1	RP11-267M23.4	_
ENSG00000254409	-1.217286	-1.328937	0.726708	1	RP11-613D13.4	_
ENSG00000255443	-1.217286	-1.328937	0.726708	1	RP1-68D18.4	_
ENSG00000255521	-1.217286	-1.328937	0.726708	1	RP4-607I7.1	_
ENSG00000257137	-1.217286	-1.328937	0.726708	1	C12orf80	_
ENSG00000258065	-1.217286	-1.328937	0.726708	1	RP11-30H9.1	_
ENSG00000258940	-1.217286	-1.328937	0.726708	1	RP11-407N17.5	_
ENSG00000258944	-1.217286	-1.328937	0.726708	1	RP4-647C14.3	_
ENSG00000260625	-1.217286	-1.328937	0.726708	1	RP11-452L6.7	_
ENSG00000261268	-1.217286	-1.328937	0.726708	1	RP11-308D13.3	_
ENSG00000264043	-1.217286	-1.328937	0.726708	1	SNORA40	_
ENSG00000266473	-1.217286	-1.328937	0.726708	1	RP11-401F2.3	_
ENSG00000268891	-1.217286	-1.328937	0.726708	1	AC006014.1	_
ENSG00000271737	-1.217286	-1.328937	0.726708	1	CTB-113I20.2	_
ENSG00000271778	-1.217286	-1.328937	0.726708	1	RP11-379F4.8	_
ENSG00000271782	-1.217286	-1.328937	0.726708	1	RP5-850O15.4	_
ENSG00000271797	-1.217286	-1.328937	0.726708	1	CTC-428G20.6	_
ENSG00000272663	-1.217286	-1.328937	0.726708	1	RP11-191L17.1	_
ENSG00000272829	-1.217286	-1.328937	0.726708	1	XXbac-B135H6.18	_
ENSG00000272892	-1.217286	-1.328937	0.726708	1	RP11-57G10.8	_
ENSG00000272910	-1.217286	-1.328937	0.726708	1	RP11-15L13.4	_
ENSG00000273027	-1.217286	-1.328937	0.726708	1	LL21NC02-1C16.1	_
ENSG00000273350	-1.217286	-1.328937	0.726708	1	RP4-539M6.20	_
ENSG00000273483	-1.217286	-1.328937	0.726708	1	RP4-671G15.2	_
ENSG00000004776	0.999022	-1.288103	0.726708	1	HSPB6	K09545
ENSG00000060140	0.999022	-1.288103	0.726708	1	STYK1	K17510
ENSG00000100583	0.999022	-1.288103	0.726708	1	SAMD15	K09131
ENSG00000124467	0.999022	-1.288103	0.726708	1	PSG8	K06500
ENSG00000126467	0.999022	-1.288103	0.726708	1	TSKS	K16492
ENSG00000127364	0.999022	-1.288103	0.726708	1	TAS2R4	K08474
ENSG00000130307	0.999022	-1.288103	0.726708	1	USHBP1	K12581
ENSG00000130377	0.999022	-1.288103	0.726708	1	ACSBG2	K15013
ENSG00000131097	0.999022	-1.288103	0.726708	1	HIGD1B	_
ENSG00000137077	0.999022	-1.288103	0.726708	1	CCL21	K16062
ENSG00000139044	0.999022	-1.288103	0.726708	1	B4GALNT3	K09656
ENSG00000140009	0.999022	-1.288103	0.726708	1	ESR2	K08551
ENSG00000143786	0.999022	-1.288103	0.726708	1	CNIH3	K09512

ENSG00000145975	0.999022	-1.288103	0.726708	1	FAM217A	_
ENSG00000147382	0.999022	-1.288103	0.726708	1	FAM58A	_
ENSG00000152253	0.999022	-1.288103	0.726708	1	SPC25	K11550
ENSG00000154721	0.999022	-1.288103	0.726708	1	JAM2	K06735
ENSG00000158516	0.999022	-1.288103	0.726708	1	CPA2	K01298
ENSG00000161509	0.999022	-1.288103	0.726708	1	GRIN2C	K05211
ENSG00000162398	0.999022	-1.288103	0.726708	1	C1orf177	_
ENSG00000163803	0.999022	-1.288103	0.726708	1	PLB1	K14621
ENSG00000164037	0.999022	-1.288103	0.726708	1	SLC9B1	_
ENSG00000170689	0.999022	-1.288103	0.726708	1	HOXB9	K09294
ENSG00000173801	0.999022	-1.288103	0.726708	1	JUP	K10056
ENSG00000174807	0.999022	-1.288103	0.726708	1	CD248	K06706
ENSG00000175414	0.999022	-1.288103	0.726708	1	ARL10	K07958
ENSG00000179242	0.999022	-1.288103	0.726708	1	CDH4	K06797
ENSG00000185532	0.999022	-1.288103	0.726708	1	PRKG1	K07376
ENSG00000186940	0.999022	-1.288103	0.726708	1	CHCHD2P9	K00901
ENSG00000196934	0.999022	-1.288103	0.726708	1	RIMBP3B	K04552
ENSG00000212743	0.999022	-1.288103	0.726708	1	DKFZP667F0711	_
ENSG00000213931	0.999022	-1.288103	0.726708	1	HBE1	K13825
ENSG00000214796	0.999022	-1.288103	0.726708	1	RP11-480I12.5	K07374
ENSG00000220937	0.999022	-1.288103	0.726708	1	HNRNPA1P41	K12741
ENSG00000222301	0.999022	-1.288103	0.726708	1	RNY5P8	_
ENSG00000225920	0.999022	-1.288103	0.726708	1	RIMKLBP2	K18310
ENSG00000226167	0.999022	-1.288103	0.726708	1	AP4B1-AS1	_
ENSG00000226179	0.999022	-1.288103	0.726708	1	LINC00685	_
ENSG00000226453	0.999022	-1.288103	0.726708	1	RP11-379B8.1	_
ENSG00000226784	0.999022	-1.288103	0.726708	1	PGAM4	K01834
ENSG00000228217	0.999022	-1.288103	0.726708	1	AL390877.1	K18751
ENSG00000229939	0.999022	-1.288103	0.726708	1	RP11-111F16.2	_
ENSG00000230601	0.999022	-1.288103	0.726708	1	RP11-402G3.3	_
ENSG00000230903	0.999022	-1.288103	0.726708	1	RPL9P8	K02940
ENSG00000235351	0.999022	-1.288103	0.726708	1	AC114730.11	_
ENSG00000237624	0.999022	-1.288103	0.726708	1	OXCT2P1	K01027
ENSG00000237842	0.999022	-1.288103	0.726708	1	RP11-110J1.2	K13111
ENSG00000243517	0.999022	-1.288103	0.726708	1	RP11-627K11.1	K02872
ENSG00000244327	0.999022	-1.288103	0.726708	1	RP11-383G6.3	_
ENSG00000244332	0.999022	-1.288103	0.726708	1	RP11-119K6.6	_
ENSG00000245651	0.999022	-1.288103	0.726708	1	RP11-620J15.2	_
ENSG00000245904	0.999022	-1.288103	0.726708	1	RP11-796E2.4	_
ENSG00000247872	0.999022	-1.288103	0.726708	1	SPCS2P3	K12947
ENSG00000248476	0.999022	-1.288103	0.726708	1	BACH1-IT1	_
ENSG00000249264	0.999022	-1.288103	0.726708	1	EEF1A1P9	K03231
ENSG00000249853	0.999022	-1.288103	0.726708	1	HS3ST5	_
ENSG00000251023	0.999022	-1.288103	0.726708	1	RP11-549J18.1	_
ENSG00000254838	0.999022	-1.288103	0.726708	1	GVINP1	K17985
ENSG00000259910	0.999022	-1.288103	0.726708	1	RP11-616M22.2	_
ENSG00000260046	0.999022	-1.288103	0.726708	1	RP11-454K7.3	_
ENSG00000260891	0.999022	-1.288103	0.726708	1	RP11-96D1.5	_
ENSG00000261187	0.999022	-1.288103	0.726708	1	RP11-1007O24.2	_
ENSG00000262147	0.999022	-1.288103	0.726708	1	RP13-638C3.3	_
ENSG00000262521	0.999022	-1.288103	0.726708	1	AJ003147.8	_
ENSG00000263080	0.999022	-1.288103	0.726708	1	RP11-485G7.5	_
ENSG00000264714	0.999022	-1.288103	0.726708	1	RP11-21G15.1	_
ENSG00000267147	0.999022	-1.288103	0.726708	1	CTC-548K16.1	_
ENSG00000267226	0.999022	-1.288103	0.726708	1	RP11-126O1.5	_
ENSG00000267742	0.999022	-1.288103	0.726708	1	FAM60CP	_
ENSG00000269191	0.999022	-1.288103	0.726708	1	AC005387.2	_
ENSG00000269993	0.999022	-1.288103	0.726708	1	AF003625.3	_

ENSG00000270110	0.999022	-1.288103	0.726708	1	RP5-1139B12.4	_
ENSG00000270277	0.999022	-1.288103	0.726708	1	RP11-65L3.2	_
ENSG00000271871	0.999022	-1.288103	0.726708	1	AC005740.6	_
ENSG00000271966	0.999022	-1.288103	0.726708	1	RP11-7F18.2	_
ENSG00000272070	0.999022	-1.288103	0.726708	1	AC005618.6	_
ENSG00000272263	0.999022	-1.288103	0.726708	1	RP11-767C1.2	_
ENSG00000272420	0.999022	-1.288103	0.726708	1	RP4-740C4.7	_
ENSG00000272620	0.999022	-1.288103	0.726708	1	AFAP1-AS1	_
ENSG00000128463	-0.230516	6.79582	0.726821	1	EMC4	_
ENSG00000272452	0.282231	2.370064	0.726822	1	RP11-391M1.4	_
ENSG00000106004	-0.258945	2.381698	0.726822	1	HOXA5	K09305
ENSG00000151552	-0.246956	4.148098	0.726846	1	QDPR	K00357
ENSG00000186501	0.239431	4.141063	0.726846	1	TMEM222	_
ENSG00000099977	-0.230887	6.539779	0.726877	1	DDT	K10028
ENSG00000025800	0.230121	6.902632	0.726901	1	KPNA6	K15042
ENSG00000162433	0.230695	5.910584	0.726913	1	AK4	K00939
ENSG00000135249	-0.233188	5.474212	0.726992	1	RINT1	K07560
ENSG00000142534	0.228384	10.07904	0.72702	1	RPS11	K02949
ENSG00000179943	-0.239745	4.63258	0.727032	1	FIZ1	_
ENSG00000138617	0.243479	3.549774	0.72707	1	PARP16	K00774
ENSG00000152102	0.231486	6.228313	0.727366	1	FAM168B	_
ENSG00000127526	0.230248	5.846411	0.727368	1	SLC35E1	K15283
ENSG00000172366	-0.230398	6.57237	0.727369	1	FAM195A	_
ENSG00000176393	-0.229626	7.077212	0.7274	1	RNPEP	K01260
ENSG00000196821	0.228951	7.595979	0.727438	1	C6orf106	_
ENSG00000177370	-0.234417	5.347592	0.727458	1	TIMM22	K17790
ENSG00000198355	0.229323	7.134025	0.727503	1	PIM3	K08807
ENSG00000231245	-0.26131	2.525942	0.727525	1	C1DP1	K12592
ENSG00000147475	-0.233025	5.248504	0.727593	1	ERLIN2	_
ENSG00000173692	-0.229458	7.024684	0.727667	1	PSMD1	K04157
ENSG00000160789	0.227913	9.262304	0.727737	1	LMNA	K12641
ENSG00000127980	-0.234048	4.605809	0.7278	1	PEX1	K13338
ENSG00000101974	0.229647	6.67194	0.727806	1	ATP11C	K01530
ENSG00000034152	-0.230572	5.781112	0.727867	1	MAP2K3	K04432
ENSG00000100744	-0.239087	4.110466	0.72792	1	GSKIP	_
ENSG00000094914	-0.23028	6.089775	0.728017	1	AAAS	K14320
ENSG00000106608	0.229918	5.916409	0.72803	1	URGCP	_
ENSG00000138709	-0.235668	4.874306	0.728041	1	LARP1B	K18757
ENSG00000166398	0.243394	4.14847	0.7281	1	KIAA0355	_
ENSG00000175305	0.238045	4.150993	0.7281	1	CCNE2	K06626
ENSG00000128524	0.230808	6.123904	0.728152	1	ATP6V1F	K02151
ENSG00000196597	0.311951	1.591049	0.728199	1	ZNF782	K09228
ENSG00000108187	-0.295689	1.624493	0.728199	1	PBLD	K15601
ENSG00000133997	-0.23473	4.925661	0.728247	1	MED6	K15128
ENSG00000066855	-0.22899	6.979229	0.728269	1	MTFR1	_
ENSG00000198901	0.227864	7.976176	0.728382	1	PRC1	K16732
ENSG00000116678	-0.245344	3.421202	0.728444	1	LEPR	K05062
ENSG00000138095	-0.227435	8.893788	0.728454	1	LRPPRC	K17964
ENSG00000136021	-0.229249	6.046024	0.728506	1	SCYL2	K17541
ENSG00000127337	-0.230687	5.617631	0.728515	1	YEATS4	K11341
ENSG00000205133	-0.243362	3.921009	0.72854	1	TRIQK	_
ENSG00000128626	-0.229363	6.577887	0.728541	1	MRPS12	K02950
ENSG00000182004	-0.230385	5.7562	0.728579	1	SNRPE	K11097
ENSG00000086712	-0.229102	6.697948	0.72861	1	TXLNG	_
ENSG00000116852	-0.254395	3.49708	0.728707	1	KIF21B	K10395
ENSG00000169612	-0.254395	3.49708	0.728707	1	FAM103A1	K18708
ENSG00000116205	-0.247205	3.493071	0.728707	1	TCEANC2	_
ENSG00000215305	-0.237561	4.848692	0.728708	1	VPS16	_

ENSG00000121067	-0.234259	5.254256	0.728769	1	SPOP	K10523
ENSG00000084234	0.227006	8.998344	0.728861	1	APLP2	K08117
ENSG00000108010	-0.228094	7.27517	0.728892	1	GLRX3	K18047
ENSG00000170089	-0.241083	4.018845	0.728894	1	RP11-423H2.1	K12880
ENSG00000087095	0.239866	4.064426	0.729053	1	NLK	K04468
ENSG00000005238	0.232765	5.167168	0.729086	1	FAM214B	_
ENSG00000149932	-0.231064	5.536891	0.72917	1	TMEM219	_
ENSG00000065000	0.227599	7.398837	0.729176	1	AP3D1	K12396
ENSG00000177427	0.238218	4.113446	0.729203	1	MIEF2	K10087
ENSG00000131507	-0.228354	6.840857	0.729216	1	NDFIP1	_
ENSG00000127947	-0.228741	6.561285	0.729284	1	PTPN12	K18024
ENSG00000127527	0.233335	5.234015	0.729289	1	EPS15L1	K12472
ENSG00000075292	-0.227694	7.310652	0.729307	1	ZNF638	_
ENSG00000136261	0.227169	7.796882	0.729311	1	BZW2	_
ENSG00000142945	0.226855	8.145113	0.729429	1	KIF2C	K10393
ENSG00000054277	-0.237202	4.550808	0.729429	1	OPN3	K04256
ENSG00000158711	-0.232748	5.486967	0.7295	1	ELK4	K04376
ENSG00000149761	-0.234889	4.582229	0.729528	1	NUDT22	_
ENSG00000205339	0.226317	9.265721	0.729568	1	IPO7	_
ENSG00000242299	0.228249	6.560067	0.729603	1	RP11-234A1.1	K02964
ENSG00000163634	-0.22855	6.511254	0.729605	1	THOC7	K13176
ENSG00000122026	-0.230364	5.174003	0.729704	1	RPL21	K02889
ENSG00000105011	0.228426	6.401366	0.729709	1	ASF1B	K10753
ENSG00000112245	0.226017	10.07402	0.729737	1	PTP4A1	_
ENSG00000143093	-0.231741	4.986344	0.729848	1	STRIP1	_
ENSG00000242372	-0.226988	7.502371	0.729901	1	EIF6	K03264
ENSG00000189410	-0.292225	2.063695	0.729993	1	SH2D5	_
ENSG00000083097	0.277365	2.059588	0.729993	1	DOPEY1	_
ENSG00000181856	0.277365	2.059588	0.729993	1	SLC2A4	K07191
ENSG00000089280	-0.226222	8.418956	0.73007	1	FUS	K13098
ENSG00000077235	0.22875	6.274974	0.730117	1	GTF3C1	K15199
ENSG00000147679	-0.232522	5.109479	0.730157	1	UTP23	K14773
ENSG00000167258	-0.228967	5.904727	0.730176	1	CDK12	K06115
ENSG00000109787	-0.23087	5.577826	0.73021	1	KLF3	K15605
ENSG00000173566	-0.291119	2.241422	0.730525	1	NUDT18	K17817
ENSG00000230124	-0.291119	2.241422	0.730525	1	RP5-1180C10.2	_
ENSG00000090006	-0.227274	6.358928	0.730582	1	LTBP4	K08023
ENSG00000175193	-0.23116	5.364704	0.730728	1	PARL	K09650
ENSG00000180626	0.233319	3.568553	0.730793	1	ZNF594	K09228
ENSG00000144713	0.225173	9.350541	0.730858	1	RPL32	K02912
ENSG00000177565	-0.226698	6.902584	0.73101	1	TBL1XR1	K04508
ENSG00000198018	0.236813	3.699247	0.731047	1	ENTPD7	K14643
ENSG00000116747	-0.230905	5.247348	0.731053	1	TROVE2	K11089
ENSG00000164924	-0.22473	10.51781	0.731169	1	YWHAZ	K16197
ENSG00000178038	-0.23344	4.489689	0.731195	1	ALS2CL	_
ENSG00000115963	-0.228532	6.119699	0.731293	1	RND3	K07859
ENSG00000114354	0.225156	8.136549	0.731384	1	TFG	K09292
ENSG00000204103	-0.241073	3.932509	0.731442	1	MAFB	K09036
ENSG00000034693	0.234176	4.687504	0.731519	1	PEX3	K13336
ENSG00000077721	-0.22619	6.88119	0.731626	1	UBE2A	K10573
ENSG00000123600	-0.238373	4.087444	0.731667	1	METTL8	K09548
ENSG00000176386	0.231382	4.126135	0.731733	1	CDC26	K03359
ENSG00000141568	0.225604	7.084406	0.731823	1	FOXK2	K09404
ENSG00000087008	0.253536	2.90356	0.731872	1	ACOX3	K00232
ENSG00000114126	-0.22752	5.831223	0.731927	1	TFDP2	K09392
ENSG00000138286	-0.236016	4.346286	0.732005	1	FAM149B1	_
ENSG00000130559	0.229411	5.117456	0.732075	1	CAMSAP1	K17493
ENSG00000247516	-0.238434	4.421839	0.732094	1	MIR4458HG	_

ENSG00000213064	-0.228431	5.991982	0.73211	1	SFT2D2	_
ENSG00000237753	-0.373178	0.553458	0.732196	1	AC079922.3	_
ENSG00000158458	0.373001	0.524082	0.732196	1	NRG2	K05456
ENSG00000186301	0.373001	0.524082	0.732196	1	MST1P2	_
ENSG00000152684	-0.226445	6.386423	0.732221	1	PELO	K06965
ENSG00000082898	-0.224197	8.636581	0.732283	1	XPO1	K14290
ENSG00000171792	-0.226591	6.170463	0.732292	1	RHNO1	_
ENSG00000215492	-0.228125	6.18197	0.732303	1	HNRNPA1P7	K12741
ENSG00000141959	0.224722	7.575619	0.732304	1	PFKL	K00850
ENSG00000091436	0.225989	6.489502	0.73232	1	MLTK	K04424
ENSG00000129472	-0.254055	3.293063	0.73234	1	RAB2B	K07878
ENSG00000103528	-0.245748	3.288419	0.73234	1	SYT17	_
ENSG00000124942	0.223982	8.852563	0.732384	1	AHNAK	_
ENSG00000218510	-0.255615	3.371431	0.732422	1	LINC00339	_
ENSG00000122687	-0.225593	6.044041	0.732499	1	FTSJ2	K02427
ENSG00000147124	0.241747	3.507986	0.732559	1	ZNF41	K09228
ENSG00000104825	-0.232077	4.92421	0.732565	1	NFKBIB	K02581
ENSG00000068028	0.22923	4.998902	0.732624	1	RASSF1	K09850
ENSG00000141480	0.230047	5.073804	0.732676	1	ARRB2	K04439
ENSG00000188372	-0.229172	5.256544	0.732794	1	ZP3	K11447
ENSG00000109390	-0.227854	5.275896	0.732807	1	NDUFC1	K03967
ENSG00000084623	-0.223805	8.329328	0.732895	1	EIF3I	K03246
ENSG00000183155	-0.240355	3.9943	0.732901	1	RABIF	K10653
ENSG00000100300	-0.224151	7.754875	0.732912	1	TSPO	K05770
ENSG00000115844	-0.241786	4.042979	0.732929	1	DLX2	K18488
ENSG00000134802	-0.223568	8.770723	0.732944	1	SLC43A3	K08230
ENSG00000143222	-0.225935	5.735797	0.733045	1	UFC1	K12165
ENSG00000104812	0.224877	6.784524	0.733081	1	GYS1	K00693
ENSG00000090975	0.229143	4.385437	0.7331	1	PITPNM2	_
ENSG00000162377	-0.232846	4.791287	0.733247	1	COA7	K18180
ENSG00000182504	-0.232846	4.791287	0.733247	1	CEP97	K16717
ENSG00000128829	-0.226103	6.353584	0.733264	1	EIF2AK4	K16196
ENSG00000145912	-0.224514	7.029408	0.733321	1	NHP2	K11129
ENSG00000149100	-0.223316	8.506986	0.733359	1	EIF3M	K15030
ENSG00000163866	-0.225904	5.491843	0.733416	1	SMIM12	_
ENSG00000162063	0.227862	5.706291	0.733453	1	CCNF	K10289
ENSG00000172037	0.22671	5.765594	0.733462	1	LAMB2	K06243
ENSG00000018408	0.226814	6.035636	0.733499	1	WWTR1	K16820
ENSG00000115091	-0.223499	7.942523	0.733507	1	ACTR3	K18584
ENSG00000214517	0.224485	6.209434	0.733519	1	PPME1	K13617
ENSG00000143748	0.224533	6.664583	0.733671	1	NVL	K14571
ENSG00000166681	-0.223641	7.567118	0.733673	1	NGFRAP1	K12465
ENSG00000180304	0.224345	6.718938	0.733796	1	OAZ2	K16613
ENSG00000149792	-0.22495	5.869948	0.733853	1	MRPL49	K17430
ENSG00000181852	-0.226744	5.422029	0.733916	1	RNF41	K11981
ENSG00000139668	0.232335	4.701077	0.734059	1	WDFY2	_
ENSG00000142623	0.369419	0.985107	0.734082	1	PADI1	K01481
ENSG00000089094	-0.346214	1.013145	0.734082	1	KDM2B	K10276
ENSG00000236200	-0.346214	1.013145	0.734082	1	KDM4A-AS1	_
ENSG00000244122	-0.346214	1.013145	0.734082	1	UGT1A7	K00699
ENSG00000169375	0.224855	6.287382	0.734091	1	SIN3A	K11644
ENSG00000157326	-0.231586	4.275614	0.734176	1	DHRS4	K11147
ENSG00000178988	0.224179	5.938043	0.734204	1	MRFAP1L1	_
ENSG00000184517	0.23801	3.831502	0.734322	1	ZFP1	K09228
ENSG00000215440	0.231352	3.834667	0.734322	1	NPEPL1	K09611
ENSG00000108592	-0.223454	7.124473	0.734404	1	FTSJ3	K14857
ENSG00000174748	0.22203	9.338027	0.734475	1	RPL15	K02877
ENSG00000186017	-0.246298	3.457585	0.734475	1	ZNF566	K09228

ENSG00000113312	-0.225882	6.005689	0.734523	1	TTC1	_
ENSG00000184281	-0.225478	5.13338	0.734592	1	TSSC4	_
ENSG00000155287	0.230765	5.107301	0.734606	1	SLC25A28	K15113
ENSG00000163702	0.22713	5.035507	0.734652	1	IL17RC	K05166
ENSG00000175287	-0.258066	3.034159	0.734662	1	PHYHD1	K00477
ENSG00000134490	-0.256234	2.934376	0.734708	1	TMEM241	_
ENSG00000237298	-0.331671	1.360622	0.734719	1	TTN-AS1	_
ENSG00000258738	-0.331671	1.360622	0.734719	1	RP11-73E17.2	_
ENSG00000156050	0.327013	1.354589	0.734719	1	FAM161B	K19348
ENSG00000187792	0.327013	1.354589	0.734719	1	ZNF70	K08878
ENSG00000267957	0.327013	1.354589	0.734719	1	RP11-178G16.4	_
ENSG00000115419	-0.223821	6.661213	0.73472	1	GLS	K01425
ENSG00000144909	-0.232534	4.937141	0.734722	1	OSBPL11	_
ENSG00000131061	0.25499	2.812823	0.734757	1	ZNF341	K06115
ENSG00000110400	0.260847	2.688934	0.734808	1	PVRL1	_
ENSG00000136986	0.22331	6.808999	0.734839	1	DERL1	K11519
ENSG00000269990	0.251425	2.561392	0.734862	1	CTD-3074O7.12	_
ENSG00000188321	-0.282503	2.432204	0.734915	1	ZNF559	K09228
ENSG00000189350	0.258151	2.412708	0.734915	1	FAM179A	K12581
ENSG00000221983	0.221654	9.282004	0.734923	1	UBA52	K02927
ENSG00000138041	-0.223529	6.718915	0.73495	1	SMEK2	K17491
ENSG00000132749	-0.282438	2.277776	0.734964	1	MTL5	_
ENSG00000120899	0.266557	2.246923	0.734964	1	PTK2B	K05871
ENSG00000118420	-0.263035	2.093658	0.735	1	UBE3D	_
ENSG00000110074	-0.228921	5.330269	0.735024	1	FOXRED1	K18166
ENSG00000164091	-0.225357	5.702532	0.735143	1	WDR82	K14962
ENSG00000141504	-0.229231	5.113275	0.735238	1	SAT2	K00657
ENSG00000125945	0.231392	4.235033	0.735343	1	ZNF436	K09228
ENSG00000107954	0.225843	5.390822	0.735492	1	NEURL1	K01931
ENSG00000175906	-0.224614	6.049345	0.735497	1	ARL4D	K07945
ENSG00000110660	-0.226307	5.688157	0.735583	1	SLC35F2	K15287
ENSG00000197956	0.221044	9.265475	0.73563	1	S100A6	_
ENSG00000157823	0.239013	3.952656	0.735682	1	AP3S2	K12399
ENSG00000157379	0.234531	3.900762	0.735752	1	DHRS1	K11163
ENSG00000131626	0.22433	5.808522	0.735822	1	PPFIA1	_
ENSG00000168300	-0.225931	5.470263	0.735894	1	PCMTD1	_
ENSG00000140153	0.238227	3.784501	0.735909	1	WDR20	_
ENSG00000163738	-0.236894	3.797144	0.735909	1	MTHFD2L	K13403
ENSG00000181789	0.221151	8.160507	0.735971	1	COPG1	K17267
ENSG00000103066	0.229962	4.993398	0.736056	1	PLA2G15	K06129
ENSG00000172932	0.229289	4.47698	0.736076	1	ANKRD13D	_
ENSG00000165288	-0.227757	4.947103	0.736141	1	BRWD3	K11798
ENSG00000091490	-0.223439	5.577828	0.736196	1	SEL1L3	_
ENSG00000128581	-0.228639	4.407391	0.736199	1	RABL5	K07935
ENSG00000180667	0.222112	6.818361	0.7362	1	YOD1	K13719
ENSG00000125746	0.221787	7.057945	0.736237	1	EML2	K18595
ENSG00000125885	-0.22639	4.808564	0.736383	1	MCM8	K10737
ENSG00000170365	0.248564	3.460823	0.736428	1	SMAD1	K04676
ENSG00000139722	0.223514	5.3761	0.736566	1	VPS37B	K12185
ENSG00000178719	0.231389	4.199639	0.736569	1	GRINA	K06890
ENSG00000125779	-0.227069	5.025869	0.736684	1	PANK2	K09680
ENSG00000115275	-0.221927	6.768387	0.736701	1	MOGS	K01228
ENSG00000088970	0.225657	4.619529	0.736744	1	PLK1S1	_
ENSG00000197860	0.225657	4.619529	0.736744	1	SGTB	K16365
ENSG00000168288	-0.221831	6.799791	0.736758	1	MMADHC	_
ENSG00000253352	0.221259	7.082768	0.736812	1	TUG1	_
ENSG00000136026	-0.22044	8.201089	0.736843	1	CKAP4	K13999
ENSG00000107614	-0.24843	3.228928	0.736872	1	TRDMT1	K15336

ENSG00000117411	-0.222441	6.251487	0.736994	1	B4GALT2	K07967
ENSG00000130707	0.219665	10.24598	0.737018	1	ASS1	K01940
ENSG00000115425	-0.24624	3.142146	0.737053	1	PECR	K07753
ENSG00000095380	-0.221273	7.009839	0.737068	1	NANS	K05304
ENSG00000117614	0.22487	5.741998	0.737156	1	SYF2	K12868
ENSG00000143801	-0.22792	5.078686	0.737229	1	PSEN2	K04522
ENSG00000113597	-0.22553	5.077383	0.737229	1	TRAPPC13	_
ENSG00000145592	0.219399	10.86181	0.737253	1	RPL37	K02922
ENSG00000168803	-0.231103	4.371872	0.737309	1	ADAL	K09228
ENSG00000136144	-0.228972	4.724892	0.737373	1	RCBTB1	K11494
ENSG00000136603	-0.222378	6.199974	0.737417	1	SKIL	_
ENSG00000151876	0.228975	4.287692	0.737511	1	FBXO4	K10291
ENSG00000066044	-0.221085	6.855336	0.737521	1	ELAVL1	K13088
ENSG00000181754	0.238459	3.735917	0.737607	1	AMIGO1	_
ENSG00000162910	-0.224498	5.365763	0.737661	1	MRPL55	K17436
ENSG00000198843	-0.2226	5.985784	0.737666	1	SELT	_
ENSG00000185798	0.229459	4.590651	0.737709	1	WDR53	K10319
ENSG00000167693	0.22131	6.297454	0.737731	1	NXN	K17609
ENSG00000103148	-0.2222	6.06252	0.737791	1	NPRL3	_
ENSG00000145390	-0.232453	4.117993	0.737991	1	USP53	_
ENSG00000115762	0.220477	6.849524	0.73803	1	PLEKHB2	K17985
ENSG00000170004	0.219504	7.901347	0.738037	1	CHD3	K11642
ENSG00000140025	-0.2641	2.718333	0.738042	1	EFCAB11	_
ENSG00000184058	-0.235607	4.07447	0.73813	1	TBX1	K10175
ENSG00000090924	0.234201	4.067105	0.73813	1	PLEKHG2	_
ENSG00000182809	0.237443	3.538589	0.73814	1	CRIP2	_
ENSG00000170502	-0.22409	4.982171	0.738162	1	NUDT9	K13988
ENSG00000173230	0.220008	7.056289	0.738283	1	GOLGB1	_
ENSG00000120910	-0.22712	3.974776	0.738437	1	PPP3CC	K04348
ENSG00000007923	-0.222259	5.446347	0.738452	1	DNAJC11	K09531
ENSG00000008735	-0.248687	3.4048	0.738584	1	MAPK8IP2	K04435
ENSG00000179046	-0.226513	4.302471	0.738605	1	TRIML2	K12039
ENSG00000109685	0.22021	6.615267	0.738716	1	WHSC1	K11424
ENSG00000213442	0.219213	7.460167	0.738743	1	RPL18AP3	K02882
ENSG00000087076	0.271014	2.43672	0.738787	1	HSD17B14	K00826
ENSG00000167604	-0.260188	2.45563	0.738787	1	NFKBID	K14214
ENSG00000123810	0.253129	2.445433	0.738787	1	B9D2	K16745
ENSG00000163710	-0.222912	4.79283	0.738788	1	PCOLCE2	_
ENSG00000182552	-0.223194	5.159354	0.738886	1	RWDD4	_
ENSG00000159200	0.227215	4.492677	0.738958	1	RCAN1	K17901
ENSG00000137449	-0.234457	3.809668	0.738986	1	CPEB2	K02602
ENSG00000168275	-0.224488	5.411773	0.739084	1	COA6	K18179
ENSG00000188130	-0.224488	5.411773	0.739084	1	MAPK12	K04441
ENSG00000138030	0.246409	3.237182	0.739126	1	KHK	K00846
ENSG00000197894	-0.219243	7.205386	0.739135	1	ADH5	K00121
ENSG00000141141	-0.220716	5.941563	0.739224	1	DDX52	K14779
ENSG00000268942	0.260708	2.283598	0.73926	1	CKS1B	K02219
ENSG00000102125	-0.257558	2.303753	0.73926	1	TAZ	K13511
ENSG00000156869	-0.257558	2.303753	0.73926	1	FRRS1	_
ENSG00000160949	-0.220624	6.10706	0.739267	1	TONSL	K09257
ENSG00000138160	-0.219461	6.909919	0.739301	1	KIF11	K10398
ENSG00000198618	0.217597	10.1402	0.739417	1	PPIAP22	K03767
ENSG00000073605	0.239293	3.151072	0.739444	1	GSDMB	_
ENSG00000240990	-0.23311	3.156682	0.739444	1	HOXA11-AS	_
ENSG00000241468	-0.219033	7.145377	0.739454	1	ATP5J2	K02130
ENSG00000101182	-0.218276	7.844171	0.739593	1	PSMA7	K02731
ENSG00000119640	0.233251	3.622993	0.739678	1	ACYP1	K01512
ENSG00000144233	0.220342	5.725494	0.739691	1	AMMECR1L	_

ENSG00000135686	0.227881	4.73446	0.739806	1	KLHL36	K13958
ENSG00000196296	0.270388	2.101294	0.739827	1	ATP2A1	K05853
ENSG00000171853	-0.229111	4.264237	0.739861	1	TRAPPC12	_
ENSG00000135469	0.228707	4.70278	0.739936	1	COQ10A	K18588
ENSG00000134375	-0.218407	7.318951	0.739958	1	TIMM17A	K17795
ENSG00000078061	0.221034	5.759118	0.739965	1	ARAF	K08845
ENSG00000169683	-0.225429	5.187283	0.73999	1	LRRC45	_
ENSG00000198546	-0.223212	5.186077	0.73999	1	ZNF511	_
ENSG00000106948	0.231362	3.924117	0.740011	1	AKNA	K15696
ENSG00000112658	-0.220097	5.99955	0.740043	1	SRF	K04378
ENSG00000163939	-0.225523	4.468024	0.74006	1	PBRM1	K11757
ENSG00000115808	-0.224548	5.165473	0.740088	1	STRN	K17608
ENSG00000179041	-0.218847	6.192814	0.74015	1	RRS1	K14852
ENSG00000113300	-0.221815	5.616743	0.740169	1	CNOT6	K12603
ENSG00000106392	-0.221353	5.142084	0.740188	1	C1GALT1	K00731
ENSG00000151914	0.218574	6.864664	0.740195	1	DST	K10382
ENSG00000161558	0.234286	2.955864	0.740206	1	TMEM143	_
ENSG00000116560	-0.217377	8.322265	0.740297	1	SFPQ	_
ENSG00000120265	0.218152	7.065815	0.740405	1	PCMT1	K00573
ENSG00000196396	0.219572	6.112502	0.740515	1	PTPN1	K05696
ENSG00000115137	0.283184	1.892586	0.740515	1	DNAJC27	K19372
ENSG00000269973	0.283184	1.892586	0.740515	1	RP11-95D17.1	_
ENSG00000101695	-0.237738	3.416967	0.740577	1	RNF125	K12170
ENSG00000094880	-0.219173	5.950094	0.740621	1	CDC23	K03355
ENSG00000134070	-0.260313	2.857896	0.740668	1	IRAK2	K04731
ENSG00000164828	0.217665	7.257607	0.740736	1	SUN1	K19347
ENSG00000099204	0.217978	6.928762	0.740789	1	ABLIM1	K07520
ENSG00000040608	-0.221927	4.508992	0.740845	1	RTN4R	K16659
ENSG00000153250	-0.218062	6.163166	0.740902	1	RBMS1	_
ENSG00000160193	-0.222395	5.552847	0.740935	1	WDR4	K15443
ENSG00000100949	-0.227401	4.273316	0.740967	1	RABGGTA	K14050
ENSG00000103479	0.22157	5.34079	0.740973	1	RBL2	K16332
ENSG00000127585	0.217893	6.315747	0.741002	1	FBXL16	K10282
ENSG00000121741	-0.219919	5.937995	0.741035	1	ZMYM2	_
ENSG00000137055	0.218137	6.624632	0.741081	1	PLAA	K14018
ENSG00000110921	-0.230165	3.988679	0.741136	1	MVK	K00869
ENSG00000089775	0.227032	4.432965	0.74121	1	ZBTB25	K00288
ENSG00000062822	-0.218304	6.551545	0.741263	1	POLD1	K02327
ENSG00000164466	0.217394	7.023585	0.741333	1	SFXN1	K12756
ENSG00000189050	0.228783	4.182843	0.741406	1	RNFT1	_
ENSG00000117155	-0.218305	6.464923	0.741439	1	SSX2IP	K06085
ENSG00000105373	0.216432	8.025303	0.741491	1	GLTSCR2	K14840
ENSG00000104412	-0.219534	6.267507	0.741546	1	EMC2	_
ENSG00000011426	0.216299	8.152324	0.741565	1	ANLN	K18621
ENSG00000081692	-0.224075	4.714894	0.741566	1	JMJD4	K05062
ENSG00000135587	-0.226209	3.885661	0.741686	1	SMPD2	K12351
ENSG00000188566	-0.224088	4.951347	0.741815	1	NDOR1	_
ENSG00000145191	-0.220992	5.539721	0.741942	1	EIF2B5	K03240
ENSG00000105325	0.219311	5.860065	0.742196	1	FZR1	K03364
ENSG00000174444	0.215068	11.5549	0.7422	1	RPL4	K02930
ENSG00000256269	-0.21832	5.645913	0.742244	1	HMBS	K01749
ENSG00000092201	-0.215895	7.926626	0.742271	1	SUPT16H	_
ENSG00000057657	0.234838	3.76208	0.742324	1	PRDM1	_
ENSG00000167986	0.215399	8.627581	0.74236	1	DDB1	K10610
ENSG00000172974	0.216297	7.137712	0.742451	1	AC007318.5	K15040
ENSG00000073578	0.215126	8.963958	0.742545	1	SDHA	K00234
ENSG00000165806	-0.218666	5.156892	0.742596	1	CASP7	K04397
ENSG00000166272	-0.222355	4.813232	0.742616	1	WBP1L	_

ENSG00000134285	0.224186	4.515905	0.742706	1	FKBP11	K09576
ENSG00000126603	0.220012	5.131317	0.742737	1	GLIS2	K09233
ENSG00000140043	0.223641	3.93859	0.742776	1	PTGR2	K13949
ENSG00000136108	-0.215844	7.428818	0.742786	1	CKAP2	K16769
ENSG00000073614	0.217998	6.052252	0.742802	1	KDM5A	K11446
ENSG00000162496	0.221363	5.392915	0.742817	1	DHRS3	K11146
ENSG00000073792	-0.219075	5.00962	0.742858	1	IGF2BP2	K17392
ENSG00000118620	-0.246853	2.979326	0.742872	1	ZNF430	K09228
ENSG00000186193	-0.21704	6.09109	0.742891	1	SAPCD2	_
ENSG00000101574	-0.222611	4.483759	0.742929	1	METTL4	_
ENSG00000104969	0.216437	6.666716	0.742964	1	SGTA	K12581
ENSG00000177469	0.214949	8.267488	0.743056	1	PTRF	K19387
ENSG00000009413	0.223218	4.843996	0.743182	1	REV3L	K02350
ENSG00000103168	-0.220227	5.063026	0.743189	1	TAF1C	K15214
ENSG00000269640	-0.244088	2.875502	0.743528	1	CTD-2521M24.9	_
ENSG00000135845	-0.218107	5.513106	0.743547	1	PIGC	_
ENSG00000235560	-0.322012	1.096649	0.743746	1	AC002310.12	_
ENSG00000109919	-0.215214	7.170246	0.743818	1	MTCH2	K17885
ENSG00000167118	-0.21716	5.671928	0.743826	1	URM1	K12161
ENSG00000121892	-0.215067	7.300186	0.743827	1	PDS5A	K11267
ENSG00000185551	-0.218865	5.209942	0.744052	1	NR2F2	K08548
ENSG00000119778	-0.238389	3.181173	0.7441	1	ATAD2B	_
ENSG00000188493	0.227729	3.716171	0.74428	1	C19orf54	_
ENSG00000271122	0.252608	2.742569	0.744283	1	RP11-379H18.1	_
ENSG00000216490	-0.240865	2.763592	0.744283	1	IFI30	K08059
ENSG00000198026	0.222401	4.559382	0.744287	1	ZNF335	K10653
ENSG00000178927	0.214958	6.23819	0.744332	1	C17orf62	_
ENSG00000154174	-0.215019	6.9266	0.744385	1	TOMM70A	K17768
ENSG00000134684	-0.213509	8.53625	0.744644	1	YARS	K01866
ENSG00000157014	-0.225612	4.378168	0.744683	1	TATDN2	K03424
ENSG00000113272	-0.232789	4.061378	0.744708	1	THG1L	K10761
ENSG00000119655	0.214932	6.636142	0.744749	1	NPC2	K13443
ENSG00000198925	0.21504	6.560952	0.744756	1	ATG9A	K17907
ENSG00000180891	-0.21694	5.259964	0.744856	1	CUEDC1	_
ENSG00000157538	0.215717	6.038287	0.744902	1	DSCR3	K11447
ENSG00000105671	0.214908	6.150067	0.745001	1	DDX49	K14778
ENSG00000179941	-0.226037	4.010552	0.745083	1	BBS10	K19401
ENSG00000087053	-0.214493	6.866669	0.745085	1	MTMR2	K18081
ENSG00000112110	-0.21589	6.287846	0.745113	1	MRPL18	K02881
ENSG00000070718	0.227404	4.156024	0.745203	1	AP3M2	K12398
ENSG00000124713	0.414359	0.60836	0.745262	1	GNMT	K00552
ENSG00000125966	0.414359	0.60836	0.745262	1	MMP24	K08002
ENSG00000143110	0.414359	0.60836	0.745262	1	C1orf162	_
ENSG00000169446	0.414359	0.60836	0.745262	1	MMGT1	_
ENSG00000223797	0.414359	0.60836	0.745262	1	ENTPD3-AS1	_
ENSG00000255100	0.414359	0.60836	0.745262	1	RP11-21L23.3	_
ENSG00000126458	-0.216972	5.34758	0.745281	1	RRAS	K07829
ENSG00000132481	-0.216054	5.524573	0.745392	1	TRIM47	K12023
ENSG00000049449	-0.212545	9.413402	0.745429	1	RCN1	_
ENSG00000155906	-0.221266	4.533792	0.745448	1	RMND1	_
ENSG00000263711	-0.229229	3.963607	0.745484	1	RP11-169F17.1	K17985
ENSG00000120071	0.226796	3.958453	0.745484	1	KANSL1	K18400
ENSG00000143147	-0.224014	3.960737	0.745484	1	GPR161	K08439
ENSG00000084444	-0.232127	2.995552	0.745485	1	KIAA1467	_
ENSG00000135966	-0.218971	4.831801	0.7456	1	TGFBRAP1	_
ENSG00000189306	-0.214785	6.398453	0.745629	1	RRP7A	K14545
ENSG00000168495	0.218165	4.998931	0.745672	1	POLR3D	K03026
ENSG00000136940	-0.219763	4.499324	0.745736	1	PDCL	K09228

ENSG00000111788	-0.22274	4.607548	0.745775	1	RP11-22B23.1	K11273
ENSG00000169026	-0.271579	1.953573	0.745803	1	MFSD7	K12306
ENSG00000077616	0.572238	-0.078186	0.745855	1	NAALAD2	K01301
ENSG00000092068	0.572238	-0.078186	0.745855	1	SLC7A8	K13781
ENSG00000099840	0.572238	-0.078186	0.745855	1	IZUMO4	_
ENSG00000185291	0.572238	-0.078186	0.745855	1	IL3RA	K04737
ENSG00000188263	0.572238	-0.078186	0.745855	1	IL17REL	_
ENSG00000204262	0.572238	-0.078186	0.745855	1	COL5A2	K19721
ENSG00000235387	0.572238	-0.078186	0.745855	1	LINC00961	_
ENSG00000235529	0.572238	-0.078186	0.745855	1	AGAP1-IT1	_
ENSG00000253671	0.572238	-0.078186	0.745855	1	RP11-806O11.1	_
ENSG00000269965	0.572238	-0.078186	0.745855	1	RP11-24B19.4	_
ENSG00000166819	0.448518	-0.022581	0.745855	1	PLIN1	K08768
ENSG00000176933	0.448518	-0.022581	0.745855	1	TOB2P1	K14443
ENSG00000184886	0.448518	-0.022581	0.745855	1	PIGW	K05283
ENSG00000185482	0.448518	-0.022581	0.745855	1	STAC3	_
ENSG00000213252	0.448518	-0.022581	0.745855	1	RP11-819M15.1	_
ENSG00000229994	0.448518	-0.022581	0.745855	1	RPL5P4	K02932
ENSG00000235245	0.448518	-0.022581	0.745855	1	RP11-122K13.12	_
ENSG00000250471	0.448518	-0.022581	0.745855	1	GMPSP1	K01951
ENSG00000260388	0.448518	-0.022581	0.745855	1	LINC00562	_
ENSG00000272417	0.448518	-0.022581	0.745855	1	CTD-2199O4.6	_
ENSG00000272807	0.448518	-0.022581	0.745855	1	RP11-260M2.1	_
ENSG00000273448	0.448518	-0.022581	0.745855	1	RP11-166O4.6	_
ENSG00000010017	0.214604	6.386184	0.745943	1	RANBP9	_
ENSG00000161202	0.214362	6.214146	0.745999	1	DVL3	K02353
ENSG00000167965	-0.213375	6.388555	0.746194	1	MLST8	K08266
ENSG00000020633	0.23109	2.485881	0.746206	1	RUNX3	K09279
ENSG00000136527	-0.212861	7.380746	0.746279	1	TRA2B	K12897
ENSG00000121350	-0.222778	4.646558	0.746345	1	PYROXD1	_
ENSG00000007255	0.223139	3.850069	0.746378	1	TRAPPC6A	_
ENSG00000157510	0.223139	3.850069	0.746378	1	AFAP1L1	_
ENSG00000099864	0.214492	6.251502	0.746558	1	PALM	K16519
ENSG00000172172	-0.213147	6.852717	0.746659	1	MRPL13	K02871
ENSG00000163156	-0.214118	5.689016	0.746707	1	SCNM1	K17985
ENSG00000119787	-0.214277	6.231823	0.74675	1	ATL2	K17339
ENSG00000161203	0.211448	8.976888	0.746792	1	AP2M1	K11826
ENSG00000132383	0.2118	7.913192	0.746915	1	RPA1	K07466
ENSG00000186130	-0.22001	4.582148	0.746931	1	ZBTB6	K10493
ENSG00000105254	-0.214839	5.605091	0.747022	1	TBCB	K17262
ENSG00000115998	-0.231497	3.111699	0.747154	1	C2orf42	_
ENSG00000132432	0.21228	7.059879	0.747179	1	SEC61G	K07342
ENSG00000106689	0.248714	2.768652	0.747295	1	LHX2	K09373
ENSG00000183421	0.290697	1.703753	0.747485	1	RIPK4	K08848
ENSG00000165912	-0.212074	7.127973	0.747495	1	PACSIN3	_
ENSG00000164542	-0.216678	5.208755	0.747643	1	KIAA0895	_
ENSG00000188786	0.224087	4.021996	0.747649	1	MTF1	_
ENSG00000159346	0.211783	7.074933	0.747735	1	ADIPOR1	K07297
ENSG00000064666	-0.212604	6.58891	0.747748	1	CNN2	K15411
ENSG00000105281	-0.211055	8.002418	0.747803	1	SLC1A5	K05616
ENSG00000105953	0.211234	7.60674	0.747809	1	OGDH	K00164
ENSG00000163882	0.212183	6.694517	0.747819	1	POLR2H	K03016
ENSG00000161326	-0.218145	4.903821	0.747966	1	DUSP14	K14165
ENSG00000117016	0.240196	2.99475	0.748046	1	RIMS3	K15298
ENSG00000065427	-0.211117	7.60638	0.74806	1	KARS	K04567
ENSG00000086827	-0.215471	4.81632	0.748072	1	ZW10	K11578
ENSG00000151276	0.219302	3.972584	0.748137	1	MAGI1	K05631
ENSG00000073905	0.224814	3.457254	0.748257	1	VDAC1P1	K05862

ENSG00000137413	-0.222563	4.440017	0.748283	1	TAF8	K14649
ENSG00000198680	-0.222563	4.440017	0.748283	1	TUSC1	_
ENSG00000177425	-0.211224	7.210164	0.74837	1	PAWR	K09228
ENSG00000119686	0.238777	2.648258	0.748426	1	FLVCR2	K08220
ENSG00000234444	-0.230632	3.615881	0.748643	1	ZNF736	_
ENSG00000147471	-0.218218	5.044554	0.748658	1	PROSC	K06997
ENSG00000164338	-0.214396	4.908194	0.74866	1	UTP15	K14549
ENSG00000177030	-0.222052	4.591277	0.748664	1	DEAF1	_
ENSG00000267317	-0.218983	4.481777	0.74885	1	CTB-25B13.12	_
ENSG00000135624	-0.209518	9.391082	0.748937	1	CCT7	K09499
ENSG00000140575	0.209598	8.895884	0.74896	1	IQGAP1	K16848
ENSG00000091409	-0.211334	6.721454	0.748969	1	ITGA6	K06485
ENSG00000174886	-0.212397	5.669351	0.749057	1	NDUFA11	K03956
ENSG00000116288	-0.210273	7.497614	0.749143	1	PARK7	K05687
ENSG00000038274	-0.211106	6.746561	0.749188	1	MAT2B	K00789
ENSG00000132792	-0.213623	5.527426	0.749191	1	CTNNBL1	K12864
ENSG00000109911	-0.228448	3.873779	0.749221	1	ELP4	K11375
ENSG00000130529	-0.217208	4.696392	0.749303	1	TRPM4	K04979
ENSG00000121579	-0.209527	8.305374	0.749373	1	NAA50	_
ENSG00000020129	0.220072	4.517847	0.749386	1	NCDN	_
ENSG00000132740	0.216047	4.0855	0.749633	1	IGHMBP2	K19036
ENSG00000132692	0.27595	1.440512	0.749699	1	BCAN	K06795
ENSG00000254681	0.27595	1.440512	0.749699	1	PKD1P5	K04985
ENSG00000266970	0.27595	1.440512	0.749699	1	RP11-806H10.4	_
ENSG00000272872	0.27595	1.440512	0.749699	1	LL22NC03-N14H11	_
ENSG00000251333	-0.26674	1.472422	0.749699	1	RTN3P1	_
ENSG00000267432	-0.26674	1.472422	0.749699	1	RP11-559N14.5	_
ENSG00000249936	-0.246979	2.517822	0.749762	1	RAC1P2	K04392
ENSG00000011677	0.243748	2.508725	0.749762	1	GABRA3	K05175
ENSG00000172006	0.243748	2.508725	0.749762	1	ZNF554	K09228
ENSG00000135541	0.213428	5.47318	0.749775	1	AHI1	K16740
ENSG00000119979	-0.214381	5.374232	0.749856	1	FAM45A	_
ENSG00000007545	0.216757	4.27651	0.749942	1	CRAMP1L	_
ENSG00000198315	0.213578	5.352662	0.750077	1	ZKSCAN8	K09229
ENSG00000213551	-0.213462	5.355091	0.750077	1	DNAJC9	K09529
ENSG00000196715	-0.211569	6.240548	0.750185	1	VKORC1L1	K05357
ENSG00000034533	-0.247636	2.795726	0.750238	1	ASTE1	K01537
ENSG00000164733	0.208694	8.333447	0.750251	1	CTSB	K01363
ENSG00000167635	-0.20942	7.32611	0.750316	1	ZNF146	K09228
ENSG00000172977	0.213869	4.598003	0.750374	1	KAT5	K11304
ENSG00000110871	-0.212796	5.418955	0.750404	1	COQ5	K06127
ENSG00000138629	-0.212245	6.110888	0.750453	1	UBL7	_
ENSG00000141349	-0.211611	6.362173	0.750465	1	G6PC3	K01084
ENSG00000120697	-0.215011	5.001425	0.750475	1	ALG5	K00729
ENSG00000104866	0.214582	4.734518	0.750531	1	PPP1R37	K17576
ENSG00000115289	-0.219484	4.411453	0.75061	1	PCGF1	K11487
ENSG00000064703	-0.210041	6.321121	0.750675	1	DDX20	K13131
ENSG00000187713	-0.212108	5.882646	0.750695	1	TMEM203	_
ENSG00000171862	0.209416	6.833017	0.750798	1	PTEN	K01110
ENSG00000129911	0.21191	5.625885	0.75084	1	KLF16	K09208
ENSG00000121281	-0.261598	1.997824	0.750881	1	ADCY7	_
ENSG00000176438	-0.261598	1.997824	0.750881	1	SYNE3	K15696
ENSG00000163867	-0.233271	3.396217	0.750954	1	ZMYM6	_
ENSG00000160298	0.215638	4.28555	0.750996	1	C21orf58	_
ENSG00000167646	0.223998	3.69266	0.75119	1	DNAAF3	K19752
ENSG00000090263	-0.210773	5.521715	0.751191	1	MRPS33	K17411
ENSG00000177383	-0.210375	5.901096	0.751228	1	MAGEF1	_
ENSG00000143442	-0.211139	6.065163	0.751322	1	POGZ	K02864

ENSG00000182484	-0.243033	2.372669	0.751363	1	WASH6P	K18461
ENSG00000136002	0.210734	5.855511	0.751379	1	ARHGEF4	K05769
ENSG00000113812	-0.218151	4.053332	0.751405	1	ACTR8	K11673
ENSG00000196367	0.212617	5.230538	0.751535	1	TRRAP	K08874
ENSG00000196517	0.21409	5.48401	0.751627	1	SLC6A9	K05038
ENSG00000144028	0.207547	8.230068	0.751636	1	SNRNP200	K12854
ENSG00000178607	0.240731	2.90975	0.751746	1	ERN1	K08852
ENSG00000116151	0.228039	2.915912	0.751746	1	MORN1	_
ENSG00000205356	0.228039	2.915912	0.751746	1	TECPR1	K17988
ENSG00000081154	-0.20769	7.895913	0.751776	1	PCNP	_
ENSG00000173915	-0.208437	7.067212	0.751778	1	USMG5	K18194
ENSG00000154642	-0.236973	3.136965	0.751833	1	C21orf91	_
ENSG00000260565	-0.236973	3.136965	0.751833	1	ERVK13-1	_
ENSG00000120438	-0.207283	8.558536	0.751837	1	TCP1	K09493
ENSG00000106144	-0.212443	5.467294	0.751853	1	CASP2	K02186
ENSG00000102034	0.212764	5.542633	0.751866	1	ELF4	K09428
ENSG00000065060	-0.21036	5.720106	0.751893	1	UHRF1BP1	K17985
ENSG00000102172	-0.207134	8.736512	0.751928	1	SMS	K00802
ENSG00000107338	-0.225419	3.480972	0.751932	1	SHB	_
ENSG00000138768	-0.208458	6.930942	0.751949	1	USO1	_
ENSG00000143476	0.208335	6.868009	0.751996	1	DTL	K11790
ENSG00000142961	0.224936	3.883141	0.752001	1	MOB3C	_
ENSG00000135452	-0.212376	4.38018	0.752048	1	TSPAN31	K17356
ENSG00000118762	-0.214501	4.462002	0.752053	1	PKD2	K04986
ENSG00000188157	-0.20704	8.649941	0.752075	1	AGRN	K06254
ENSG00000149639	0.210924	5.720499	0.752283	1	SOGA1	K11447
ENSG00000166855	-0.208912	6.420121	0.752349	1	CLPX	K03544
ENSG00000067596	0.208206	6.205828	0.752371	1	DHX8	K12818
ENSG00000198589	0.210289	5.063382	0.752383	1	LRBA	_
ENSG00000110583	0.215374	4.644367	0.752471	1	NAA40	_
ENSG00000166949	0.208952	5.737052	0.752474	1	SMAD3	K04500
ENSG00000116729	0.207314	7.402376	0.752534	1	WLS	_
ENSG00000110651	0.207207	7.522797	0.752542	1	CD81	K06508
ENSG00000113522	-0.210219	6.254965	0.752632	1	RAD50	K10866
ENSG00000113734	-0.226119	3.949458	0.752668	1	BNIP1	K08497
ENSG00000015568	0.300388	1.095694	0.752733	1	RGPD5	K12172
ENSG00000161544	0.300388	1.095694	0.752733	1	CYGB	_
ENSG00000188511	0.300388	1.095694	0.752733	1	C22orf34	_
ENSG00000236682	0.300388	1.095694	0.752733	1	AC068282.3	_
ENSG00000260276	0.300388	1.095694	0.752733	1	RP11-77H9.2	_
ENSG00000267493	0.300388	1.095694	0.752733	1	CIRBP-AS1	_
ENSG00000110200	-0.22148	3.702469	0.752753	1	ANAPC15	_
ENSG00000134504	-0.212589	4.814753	0.752798	1	KCTD1	_
ENSG00000165490	-0.209962	5.741557	0.752858	1	C11orf82	K08857
ENSG00000146833	-0.230091	3.412719	0.752866	1	TRIM4	K11998
ENSG00000112297	-0.227393	3.237816	0.752936	1	AIM1	_
ENSG00000172613	0.214532	4.294533	0.753079	1	RAD9A	K10994
ENSG00000165516	-0.212117	5.287144	0.753134	1	KLHDC2	_
ENSG00000171320	-0.211187	4.786447	0.753183	1	ESCO2	K11268
ENSG00000121957	0.20724	6.921493	0.753187	1	GPSM2	K15837
ENSG00000161813	-0.206843	7.369426	0.753247	1	LARP4	K18763
ENSG00000135632	-0.211815	5.43626	0.753271	1	SMYD5	_
ENSG00000145016	0.221035	4.002994	0.753282	1	KIAA0226	K19330
ENSG00000124357	0.215162	4.005792	0.753282	1	NAGK	K00884
ENSG00000166171	0.215162	4.005792	0.753282	1	DPCD	_
ENSG00000140464	0.209994	5.043556	0.753319	1	PML	K10054
ENSG00000226564	0.257593	2.181229	0.75332	1	FTH1P20	K00522
ENSG00000196924	-0.238117	2.21117	0.75332	1	FLNA	K04437

ENSG00000113048	-0.206351	7.812427	0.753393	1	MRPS27	K17406
ENSG00000167657	0.208916	6.126998	0.753417	1	DAPK3	K08803
ENSG00000152082	-0.208154	5.870721	0.753498	1	MZT2B	K16574
ENSG00000166444	0.208746	5.375051	0.753568	1	ST5	_
ENSG00000144744	-0.208216	6.225997	0.753606	1	UBA3	K10686
ENSG00000134262	-0.220654	4.2596	0.753639	1	AP4B1	K12401
ENSG00000214706	-0.222509	3.496542	0.75373	1	IFRD2	_
ENSG00000175265	-0.207338	6.168074	0.753811	1	GOLGA8A	K10582
ENSG00000112339	0.208241	5.901763	0.754154	1	HBS1L	K14416
ENSG00000169733	-0.207874	5.482073	0.754156	1	RFNG	K05948
ENSG00000078699	0.213882	4.819366	0.754243	1	CBFA2T2	K17985
ENSG00000121749	0.20685	6.518459	0.754281	1	TBC1D15	_
ENSG00000005812	-0.207729	5.782731	0.754333	1	FBXL3	K10269
ENSG00000134253	-0.230277	2.945145	0.754371	1	TRIM45	K12021
ENSG00000225485	-0.219564	2.939164	0.754371	1	ARHGAP23	_
ENSG00000198464	-0.215755	3.575772	0.754502	1	ZNF480	K09228
ENSG00000092199	-0.20466	9.574915	0.754522	1	HNRNPC	K12884
ENSG00000127824	0.205164	7.89132	0.754612	1	TUBA4A	K07374
ENSG00000085982	-0.214332	4.731441	0.754769	1	USP40	K11869
ENSG00000169032	0.206449	6.480125	0.754816	1	MAP2K1	K04368
ENSG00000158552	0.220862	4.168342	0.754862	1	ZFAND2B	_
ENSG00000185009	0.206421	6.463308	0.754879	1	AP3M1	K12398
ENSG00000047249	0.206018	6.718035	0.754902	1	ATP6V1H	K02144
ENSG00000162972	-0.21327	4.8287	0.754959	1	C2orf47	_
ENSG00000196922	-0.211247	4.975188	0.755012	1	ZNF252P	K09228
ENSG00000117036	-0.215088	4.315647	0.755127	1	ETV3	K09433
ENSG00000100387	-0.206459	6.434384	0.755151	1	RBX1	K03868
ENSG00000154710	0.244838	2.388304	0.755155	1	RABGEF1	_
ENSG00000090581	0.20738	5.722159	0.755207	1	GNPTG	K10087
ENSG00000115935	-0.209755	4.699735	0.75522	1	WIPF1	K19475
ENSG00000151553	-0.208177	5.601489	0.755266	1	FAM160B1	_
ENSG00000122008	-0.211517	5.005514	0.755274	1	POLK	K03511
ENSG00000186642	0.204415	8.139705	0.755319	1	PDE2A	K18283
ENSG00000137876	-0.204711	7.679618	0.755407	1	RSL24D1	K02896
ENSG00000269858	0.218126	3.840974	0.755525	1	EGLN2	K09592
ENSG00000100263	-0.2093	5.033873	0.755526	1	RHBDD3	_
ENSG00000117000	0.206187	5.030194	0.755526	1	RLF	_
ENSG00000104863	0.241337	2.819437	0.755926	1	LIN7B	_
ENSG00000267427	0.205389	6.522758	0.755963	1	CTC-503J8.6	_
ENSG00000131149	0.205957	6.341926	0.756157	1	GSE1	_
ENSG00000116830	-0.207557	5.959298	0.75623	1	TTF2	K15173
ENSG00000012963	0.208169	5.187828	0.756301	1	UBR7	K11979
ENSG00000162241	0.22858	3.156242	0.756344	1	SLC25A45	K15123
ENSG00000186283	-0.207319	5.74728	0.756498	1	TOR3A	_
ENSG00000196757	0.215291	4.360114	0.756512	1	ZNF700	K09228
ENSG00000023734	-0.203287	8.423434	0.75654	1	STRAP	K13137
ENSG00000214110	0.300766	1.486524	0.756575	1	LDHAP4	K00016
ENSG00000273162	0.300766	1.486524	0.756575	1	RP11-108L7.15	_
ENSG00000130684	0.265289	1.504097	0.756575	1	ZNF337	K09228
ENSG00000142168	-0.203988	7.338641	0.756589	1	SOD1	K04565
ENSG00000159658	-0.205129	6.418085	0.756723	1	EFCAB14	_
ENSG00000224861	0.221914	3.654868	0.756782	1	YBX1P1	K09276
ENSG00000130021	-0.210794	4.80694	0.75683	1	HDHD1	K17623
ENSG00000111554	-0.219505	3.855619	0.75691	1	MDM1	K17886
ENSG00000031823	0.204792	6.085573	0.756946	1	RANBP3	K15304
ENSG00000154144	-0.222034	4.0321	0.757006	1	TBRG1	K04613
ENSG00000092758	0.218285	4.024757	0.757006	1	COL9A3	K08131
ENSG00000129515	-0.205358	6.269818	0.757036	1	SNX6	K17920

ENSG00000219481	-0.212009	4.744567	0.75704	1	NBPF1	_
ENSG00000183617	-0.219579	4.186867	0.757082	1	MRPL54	K17435
ENSG00000102038	-0.391285	0.698644	0.7571	1	SMARCA1	K11727
ENSG00000114378	0.344543	0.642022	0.7571	1	HYAL1	K01197
ENSG00000165949	0.344543	0.642022	0.7571	1	IFI27	_
ENSG00000198863	-0.209617	5.016381	0.757155	1	RUNDC1	_
ENSG00000102931	-0.205743	5.539724	0.757178	1	ARL2BP	K16742
ENSG00000158062	-0.212213	4.452011	0.757193	1	UBXN11	_
ENSG00000143183	0.204226	6.581688	0.757204	1	TMCO1	K01489
ENSG00000123636	-0.226817	3.515903	0.75725	1	BAZ2B	_
ENSG00000117713	0.207696	5.310149	0.757257	1	ARID1A	K11653
ENSG00000115750	-0.219713	3.920734	0.757385	1	TAF1B	K15213
ENSG00000205571	-0.215432	4.236495	0.757458	1	SMN2	K13129
ENSG00000106348	-0.203758	6.867463	0.757484	1	IMPDH1	K00088
ENSG00000178229	-0.273667	2.231417	0.757515	1	ZNF543	K09228
ENSG00000099250	0.210703	4.711265	0.757535	1	NRP1	K06724
ENSG00000065150	-0.202184	8.710316	0.757683	1	IPO5	_
ENSG00000229809	-0.251627	2.711281	0.757734	1	ZNF688	K09228
ENSG00000126749	-0.239045	2.70419	0.757734	1	EMG1	K14568
ENSG00000237523	-0.229778	3.065264	0.757802	1	LINC00857	_
ENSG00000064547	0.219843	3.065052	0.757802	1	LPAR2	K04291
ENSG00000163486	0.219843	3.065052	0.757802	1	SRGAP2	K07526
ENSG00000116752	-0.204147	6.441971	0.757808	1	BCAS2	K12861
ENSG00000141448	-0.215734	4.286757	0.757809	1	GATA6	K17897
ENSG00000174080	0.207306	4.8743	0.75801	1	CTSF	K01373
ENSG00000165916	-0.202453	7.659616	0.758044	1	PSMC3	K03065
ENSG00000136938	-0.202017	8.347859	0.758057	1	ANP32B	K18647
ENSG00000167601	0.202398	7.399755	0.758233	1	AXL	K05115
ENSG00000164284	-0.203339	5.741563	0.75825	1	GRPEL2	K03687
ENSG00000163389	-0.217314	3.867651	0.758279	1	POGLUT1	K13667
ENSG00000121691	0.204284	5.466247	0.758364	1	CAT	K03781
ENSG00000198455	-0.207429	5.274743	0.758458	1	ZXDB	_
ENSG00000226054	-0.251861	2.414755	0.758834	1	MEMO1P1	K06990
ENSG00000118855	-0.206762	5.182437	0.758876	1	MFSD1	_
ENSG00000206417	-0.282182	1.7984	0.758903	1	H1FX-AS1	_
ENSG00000095209	-0.203775	5.947994	0.758939	1	TMEM38B	_
ENSG00000142453	-0.203843	6.174163	0.758945	1	CARM1	K05931
ENSG00000090487	-0.202236	6.980036	0.759079	1	SPG21	K19367
ENSG00000112144	-0.228989	3.279081	0.759179	1	ICK	K08828
ENSG00000175048	0.214012	3.270053	0.759179	1	ZDHHC14	K16675
ENSG00000105926	-0.205012	5.661587	0.759209	1	MPP6	_
ENSG00000173848	-0.20322	5.890806	0.759216	1	NET1	_
ENSG00000165219	0.202577	6.444266	0.759364	1	GAPVD1	K14209
ENSG00000086015	0.202421	6.515089	0.759411	1	MAST2	K08789
ENSG00000198551	-0.217284	3.608507	0.759458	1	ZNF627	K09228
ENSG00000102241	-0.201706	7.148238	0.759465	1	HTATSF1	K13093
ENSG00000161714	0.201402	7.258678	0.759535	1	PLCD3	K05857
ENSG00000143653	-0.202319	6.116605	0.759675	1	SCCPDH	_
ENSG00000254093	-0.220774	3.370781	0.759739	1	PINX1	K11135
ENSG00000145779	0.201946	6.625464	0.759769	1	TNFAIP8	_
ENSG00000248131	-0.23489	2.577468	0.759863	1	CT49	_
ENSG00000087206	0.203539	5.455283	0.760048	1	UIMC1	K12581
ENSG00000183955	-0.20225	5.53784	0.760067	1	SETD8	K11428
ENSG00000186063	-0.202421	5.479129	0.760165	1	AIDA	_
ENSG00000183530	0.204016	5.138841	0.760241	1	PRR14L	K01613
ENSG00000108384	-0.203837	5.668496	0.76039	1	RAD51C	K10870
ENSG00000162194	-0.201216	5.866983	0.760392	1	C11orf48	_
ENSG00000261355	-0.272371	2.052285	0.76046	1	RP11-698N11.4	_

ENSG00000228782	-0.252241	2.040772	0.76046	1	MRPL45P2	K17426
ENSG00000143379	0.201442	5.896604	0.760559	1	SETDB1	K11421
ENSG00000228253	0.202343	6.18364	0.7606	1	MT-ATP8	K02125
ENSG00000204520	0.223099	3.531256	0.760673	1	MICA	K07985
ENSG00000003249	0.214933	3.535182	0.760673	1	DBNDD1	_
ENSG00000047617	-0.233778	2.730716	0.760688	1	ANO2	K19497
ENSG00000257167	-0.233778	2.730716	0.760688	1	TMPO-AS1	_
ENSG00000262049	-0.233778	2.730716	0.760688	1	RP13-1032I1.7	_
ENSG00000147403	0.199899	7.908385	0.760712	1	RPL10	K02866
ENSG00000176946	-0.203024	6.002319	0.760782	1	THAP4	_
ENSG00000056586	-0.204822	5.52267	0.760822	1	RC3H2	K15690
ENSG00000144746	0.200932	6.688529	0.760841	1	ARL6IP5	_
ENSG00000117592	-0.199306	9.16115	0.760861	1	PRDX6	K11188
ENSG00000153914	-0.201051	6.69763	0.760902	1	SREK1	K13165
ENSG00000090861	-0.199503	8.356455	0.760973	1	AARS	K01872
ENSG00000144362	-0.337996	1.197386	0.761112	1	PHOSPHO2	K13248
ENSG00000250959	-0.337996	1.197386	0.761112	1	GLUD1P3	K00261
ENSG00000267680	-0.337996	1.197386	0.761112	1	ZNF224	K17985
ENSG00000213401	0.28544	1.176017	0.761112	1	MAGEA12	_
ENSG00000139154	-0.200447	6.237096	0.761122	1	AEBP2	K17452
ENSG00000212664	0.207508	4.156209	0.761163	1	RP11-592N21.1	K02880
ENSG00000112624	0.211069	3.686046	0.76142	1	GLTSCR1L	_
ENSG00000101972	0.199327	7.640072	0.761581	1	STAG2	K06671
ENSG00000108961	-0.217973	3.75855	0.761741	1	RANGRF	K15117
ENSG00000141446	0.202877	5.148775	0.761921	1	ESCO1	K12900
ENSG00000138078	-0.203322	5.58698	0.761943	1	PREPL	_
ENSG00000152904	-0.201705	6.194054	0.761962	1	GGPS1	K00804
ENSG00000185813	-0.203552	4.90231	0.762037	1	PCYT2	K00967
ENSG00000188428	-0.208976	4.742918	0.762039	1	BLOC1S5	K04613
ENSG00000142892	-0.199896	6.817077	0.762041	1	PIGK	K05290
ENSG00000105193	0.19787	10.02053	0.762305	1	RPS16	K02960
ENSG00000133624	0.217207	3.085928	0.762406	1	ZNF767	K09228
ENSG00000120075	0.240042	2.421573	0.762406	1	HOXB5	K09305
ENSG00000261740	0.222157	2.430377	0.762406	1	RP11-345J4.5	K08873
ENSG00000074842	0.198801	7.394529	0.762415	1	C19orf10	_
ENSG00000173898	-0.201569	5.800142	0.762449	1	SPTBN2	K06115
ENSG00000124596	-0.204384	4.844005	0.76245	1	OARD1	_
ENSG00000116698	0.199561	6.64664	0.762499	1	SMG7	K14409
ENSG00000198954	-0.199377	6.134185	0.762526	1	KIAA1279	K10734
ENSG00000125730	0.198785	7.271792	0.76256	1	C3	K03990
ENSG00000086061	-0.197963	8.767964	0.762567	1	DNAJA1	K09502
ENSG00000125454	-0.210323	4.48564	0.762568	1	SLC25A19	K15108
ENSG00000268006	0.20923	4.262919	0.762653	1	PTOV1-AS1	_
ENSG00000167528	-0.207196	4.526123	0.762724	1	ZNF641	K09228
ENSG00000254004	-0.204816	4.747802	0.762773	1	ZNF260	K09228
ENSG00000160214	-0.20128	5.971232	0.762778	1	RRP1	K14849
ENSG00000111843	-0.197833	8.39068	0.762897	1	TMEM14C	_
ENSG00000173267	0.198596	7.144785	0.762923	1	SNCG	_
ENSG00000084693	0.205171	5.128837	0.762935	1	AGBL5	_
ENSG00000164050	0.205387	4.811604	0.76302	1	PLXNB1	K06821
ENSG00000164713	-0.200561	5.958606	0.76304	1	BRI3	_
ENSG00000157870	0.247035	2.590936	0.763054	1	FAM213B	K15717
ENSG00000180953	0.247035	2.590936	0.763054	1	ST20	K02162
ENSG00000184949	0.247035	2.590936	0.763054	1	FAM227A	_
ENSG00000135535	0.198014	7.679356	0.763076	1	CD164	K06546
ENSG00000270015	-0.282008	1.550502	0.763086	1	RP11-540B6.6	_
ENSG00000143376	0.201489	5.325561	0.763114	1	SNX27	K17936
ENSG00000119231	0.199879	5.897338	0.76315	1	SENP5	K08594

ENSG00000119383	-0.198907	6.841738	0.76315	1	PPP2R4	K17605
ENSG00000129484	-0.203535	5.349722	0.763194	1	PARP2	K10798
ENSG00000106609	0.198675	6.832204	0.76324	1	TMEM248	_
ENSG00000119326	-0.197688	8.0774	0.763256	1	CTNNAL1	_
ENSG00000161638	0.197997	7.466281	0.763282	1	ITGA5	K06484
ENSG00000166925	-0.201661	5.67108	0.763424	1	TSC22D4	_
ENSG00000155508	0.198369	6.948719	0.763432	1	CNOT8	K12581
ENSG00000175938	0.217629	3.383631	0.763482	1	ORAI3	K16058
ENSG00000137145	0.203709	5.436147	0.763489	1	DENND4C	_
ENSG00000075234	-0.201067	5.43929	0.763489	1	TTC38	_
ENSG00000188706	0.201414	5.456284	0.763557	1	ZDHHC9	K16675
ENSG00000169570	-0.228708	2.756766	0.763571	1	DTWD2	_
ENSG00000116525	0.227552	2.730161	0.763571	1	TRIM62	K12030
ENSG00000134780	0.213221	2.737183	0.763571	1	DAGLA	K13806
ENSG00000179715	0.213221	2.737183	0.763571	1	PCED1B	_
ENSG00000131019	-0.458282	0.099778	0.763627	1	ULBP3	K07986
ENSG00000138378	-0.458282	0.099778	0.763627	1	STAT4	K11222
ENSG00000197165	-0.458282	0.099778	0.763627	1	SULT1A2	K01014
ENSG00000211454	-0.458282	0.099778	0.763627	1	AKR7L	K15303
ENSG00000213236	-0.458282	0.099778	0.763627	1	YWHAZP2	K16197
ENSG00000250041	-0.458282	0.099778	0.763627	1	CTD-2003C8.2	_
ENSG00000255050	-0.458282	0.099778	0.763627	1	RP11-661A12.9	_
ENSG00000269018	-0.458282	0.099778	0.763627	1	AP001362.1	K04613
ENSG00000271153	-0.458282	0.099778	0.763627	1	ABC7-43041300I9.1	K02893
ENSG00000000460	-0.208945	4.273168	0.763666	1	C1orf112	K17542
ENSG00000129696	-0.218083	3.476914	0.763757	1	TTI2	K10408
ENSG00000184047	-0.206299	3.963351	0.763798	1	DIABLO	K10522
ENSG00000059758	-0.20266	5.262234	0.763887	1	CDK17	K15595
ENSG00000106012	0.201805	5.283354	0.763957	1	IQCE	_
ENSG00000180340	-0.20176	5.483053	0.764063	1	FZD2	K02235
ENSG00000067900	0.197843	5.992791	0.764073	1	ROCK1	K04514
ENSG00000059728	-0.206566	4.413422	0.764074	1	MXD1	K09114
ENSG00000131558	-0.200351	5.778754	0.764117	1	EXOC4	K06111
ENSG00000197343	-0.20209	5.504446	0.764121	1	ZNF655	K09229
ENSG00000135956	0.199212	5.793532	0.764164	1	TMEM127	K15696
ENSG00000188986	-0.198203	6.719841	0.764168	1	NELFB	K15180
ENSG00000135090	-0.207106	4.457972	0.764195	1	TAOK3	K04429
ENSG00000155085	-0.247265	1.834161	0.764237	1	AK9	K18533
ENSG00000145386	-0.197467	7.246171	0.764266	1	CCNA2	K06627
ENSG00000171222	0.202855	5.212906	0.764267	1	SCAND1	_
ENSG00000148700	0.198254	6.4558	0.764353	1	ADD3	K18622
ENSG00000146067	-0.201801	4.822476	0.764408	1	FAM193B	_
ENSG00000157827	-0.198907	5.907698	0.764464	1	FMNL2	_
ENSG00000036257	0.198124	6.051548	0.764527	1	CUL3	K03869
ENSG00000044090	0.199061	5.422908	0.764792	1	CUL7	K10613
ENSG00000160211	0.202799	4.721319	0.764872	1	G6PD	K00036
ENSG00000138594	-0.196577	7.655523	0.764877	1	TMOD3	K12581
ENSG00000064313	-0.199725	5.955081	0.764896	1	TAF2	K03128
ENSG00000164169	-0.222145	3.214277	0.764898	1	PRMT10	K19737
ENSG00000125386	0.210752	3.901048	0.764905	1	FAM193A	_
ENSG00000068697	0.196166	8.129349	0.764914	1	LAPTM4A	K12387
ENSG00000172086	-0.203425	5.036532	0.764925	1	KRCC1	_
ENSG00000181191	0.25397	2.07109	0.76498	1	PJA1	K10633
ENSG00000120519	-0.243451	2.08249	0.76498	1	SLC10A7	K14347
ENSG00000213995	0.199134	5.863809	0.765015	1	CARKD	K17757
ENSG00000162852	-0.200194	4.794321	0.765025	1	CNST	K17581
ENSG00000250462	0.208661	3.967102	0.765041	1	LRRC37BP1	_
ENSG00000178188	0.203948	4.82406	0.765097	1	SH2B1	K05725

ENSG00000198715	0.200651	4.825621	0.765097	1	C1orf85	_
ENSG00000151693	0.208023	4.019464	0.765166	1	ASAP2	K12488
ENSG00000128191	0.199951	5.196245	0.765266	1	DGCR8	K18419
ENSG00000100360	-0.214675	3.404182	0.765311	1	IFT27	K07934
ENSG00000162520	-0.214675	3.404182	0.765311	1	SYNC	K10377
ENSG00000136270	-0.196506	7.273053	0.765352	1	TBRG4	_
ENSG00000125977	-0.195745	8.227194	0.765422	1	EIF2S2	K03238
ENSG00000166188	-0.207949	3.488505	0.765481	1	ZNF319	K09228
ENSG00000235272	0.226011	2.266878	0.765497	1	FAM103A2P	K18708
ENSG00000122085	-0.203485	4.696329	0.765543	1	MTERFD2	K15032
ENSG00000167378	0.202042	4.727963	0.765607	1	IRGQ	_
ENSG00000140650	0.203654	5.120031	0.765665	1	PMM2	K17497
ENSG00000158850	-0.199797	5.494761	0.765805	1	B4GALT3	K07968
ENSG00000133114	-0.199512	5.225224	0.765838	1	GPALPP1	_
ENSG00000112763	-0.199947	5.250768	0.765877	1	BTN2A1	K06712
ENSG00000144674	0.196175	7.012996	0.765899	1	GOLGA4	_
ENSG00000140157	-0.197605	6.034772	0.765953	1	NIPA2	_
ENSG00000155252	0.19777	5.591696	0.765963	1	PI4K2A	K13711
ENSG00000165724	-0.198533	5.855681	0.765994	1	ZMYND19	K17868
ENSG00000116095	-0.20628	4.508898	0.766023	1	PLEKHA3	_
ENSG00000100462	-0.196683	6.629359	0.766093	1	PRMT5	K02516
ENSG00000092020	-0.214323	3.917774	0.766188	1	PPP2R3C	K11583
ENSG00000173638	-0.199805	5.596974	0.766366	1	SLC19A1	K14609
ENSG00000159322	-0.199393	5.904775	0.76639	1	ADPGK	K08074
ENSG00000083520	-0.196246	6.736792	0.766412	1	DIS3	K12585
ENSG00000180628	-0.196729	6.209543	0.766468	1	PCGF5	K11489
ENSG00000203993	0.204533	4.138903	0.766484	1	C9orf37	_
ENSG00000133606	0.19527	7.431344	0.766488	1	MKRN1	K15687
ENSG00000164176	-0.195934	6.910015	0.766499	1	EDIL3	_
ENSG00000100285	0.206025	4.290121	0.766653	1	NEFH	K04574
ENSG00000152117	0.206025	4.290121	0.766653	1	AC093838.4	K12353
ENSG00000147419	-0.199516	5.519808	0.766688	1	CCDC25	K14571
ENSG00000109790	-0.196944	6.186265	0.766705	1	KLHL5	K10442
ENSG00000144199	-0.202075	5.076035	0.766724	1	FAHD2B	_
ENSG00000140382	-0.198493	5.560301	0.766729	1	HMG20A	_
ENSG00000249353	0.194994	7.489169	0.766755	1	NPM1P27	K11276
ENSG00000120696	0.205505	4.510251	0.766871	1	KBTBD7	K10474
ENSG00000141452	-0.202048	4.556207	0.766908	1	C18orf8	_
ENSG00000087191	-0.195094	7.295547	0.766969	1	PSMC5	K03066
ENSG00000168818	-0.204971	4.667313	0.767005	1	STX18	K08492
ENSG00000166439	0.201291	4.734576	0.767062	1	RNF169	_
ENSG00000117724	0.194221	8.195369	0.767142	1	CENPF	K11499
ENSG00000079387	-0.196927	5.106869	0.767314	1	SENP1	K08592
ENSG00000173653	-0.201881	5.211102	0.767374	1	RCE1	K08658
ENSG00000146463	-0.196462	6.113457	0.767467	1	ZMYM4	_
ENSG00000162714	-0.199635	5.39832	0.767473	1	ZNF496	K09229
ENSG00000114416	0.193905	8.195517	0.767511	1	FXR1	K15516
ENSG00000085871	-0.203362	4.045239	0.767518	1	MGST2	K00799
ENSG00000147535	-0.203362	4.045239	0.767518	1	PPAPDC1B	_
ENSG00000139531	-0.207276	4.201124	0.767592	1	SUOX	K00387
ENSG00000012174	-0.19552	6.535181	0.767621	1	MBTPS2	K07765
ENSG00000161010	0.2077	4.387688	0.767668	1	C5orf45	_
ENSG00000154743	-0.198613	4.435815	0.767684	1	TSEN2	K15322
ENSG00000123908	0.199057	4.632111	0.767751	1	AGO2	_
ENSG00000121774	-0.193812	8.042449	0.767794	1	KHDRBS1	K13198
ENSG00000141499	-0.200379	4.938475	0.767835	1	WRAP53	_
ENSG00000121851	-0.357324	0.801667	0.767877	1	POLR3GL	K03024
ENSG00000203813	-0.357324	0.801667	0.767877	1	HIST1H3H	K11253

ENSG00000272277	-0.357324	0.801667	0.767877	1	RP1-40E16.12	_
ENSG00000103044	0.320565	0.750992	0.767877	1	HAS3	K00752
ENSG00000224687	0.320565	0.750992	0.767877	1	RASAL2-AS1	_
ENSG00000235016	0.320565	0.750992	0.767877	1	RP11-493K19.3	_
ENSG00000181751	-0.306995	0.772389	0.767877	1	C5orf30	_
ENSG00000149658	-0.195693	6.24464	0.768039	1	YTHDF1	_
ENSG00000133275	-0.194876	6.683228	0.768096	1	CSNK1G2	K08958
ENSG00000126777	0.193494	7.989523	0.768114	1	KTN1	K17975
ENSG00000221978	-0.195015	6.5614	0.768157	1	CCNL2	_
ENSG00000105137	0.194794	6.55809	0.768187	1	SYDE1	_
ENSG00000177963	-0.19514	6.471925	0.768188	1	RIC8A	_
ENSG00000165732	-0.193105	8.749074	0.768237	1	DDX21	K16911
ENSG00000137161	0.194851	6.020097	0.768314	1	CNPY3	_
ENSG00000188243	-0.200195	5.290495	0.768403	1	COMMD6	_
ENSG00000162736	0.194569	6.568764	0.768429	1	NCSTN	K06171
ENSG00000132780	-0.193144	8.223256	0.768457	1	NASP	K11291
ENSG00000180964	-0.199958	4.814705	0.768486	1	TCEAL8	_
ENSG00000169905	0.194219	6.786792	0.768487	1	TOR1AIP2	K17985
ENSG00000139631	-0.202053	4.642951	0.768523	1	CSAD	K01594
ENSG00000103111	-0.20424	4.565502	0.768541	1	MON1B	_
ENSG00000158292	0.214426	3.65846	0.7688	1	GPR153	K08437
ENSG00000167105	0.214426	3.65846	0.7688	1	TMEM92	_
ENSG00000110108	-0.193483	7.252813	0.768895	1	TMEM109	_
ENSG00000176142	-0.198113	5.219354	0.768931	1	TMEM39A	_
ENSG00000204764	-0.223853	2.916219	0.769086	1	RANBP17	_
ENSG00000044446	-0.197572	5.705917	0.769088	1	PHKA2	K07190
ENSG00000151327	-0.195553	5.633623	0.769135	1	FAM177A1	_
ENSG00000137872	0.220745	2.782276	0.769137	1	SEMA6D	K06842
ENSG00000102981	-0.240373	1.59266	0.76926	1	PARD6A	K06093
ENSG00000135747	0.240854	2.293346	0.769296	1	ZNF670	K09228
ENSG00000231584	0.240854	2.293346	0.769296	1	FAHD2CP	_
ENSG00000213881	0.264382	1.857739	0.769341	1	NPM1P6	K11276
ENSG00000215068	-0.237279	1.882184	0.769341	1	AC025171.1	_
ENSG00000166797	-0.19385	6.64127	0.769364	1	FAM96A	_
ENSG00000039319	-0.193527	5.761096	0.76941	1	ZFYVE16	K04679
ENSG00000152382	-0.201714	4.489493	0.769414	1	TADA1	K11317
ENSG00000044115	0.191923	9.094584	0.769448	1	CTNNA1	K05691
ENSG00000132849	0.194296	6.012637	0.769512	1	INADL	K06092
ENSG00000168061	-0.19533	5.955795	0.769567	1	SAC3D1	K16734
ENSG00000164023	-0.195043	5.558443	0.769595	1	SGMS2	_
ENSG00000120896	-0.195719	5.909162	0.769611	1	SORBS3	_
ENSG00000109065	-0.19702	5.009564	0.769646	1	NAT9	_
ENSG00000147586	-0.193818	6.152792	0.769661	1	MRPS28	K17407
ENSG00000174373	-0.198706	4.164857	0.769711	1	RALGAPA1	_
ENSG00000105641	-0.744868	-0.94909	0.769726	1	SLC5A5	K14385
ENSG00000118557	-0.744868	-0.94909	0.769726	1	PMFBP1	_
ENSG00000137573	-0.744868	-0.94909	0.769726	1	SULF1	K14607
ENSG00000144452	-0.744868	-0.94909	0.769726	1	ABCA12	K05646
ENSG00000145002	-0.744868	-0.94909	0.769726	1	FAM86B2	K12354
ENSG00000155265	-0.744868	-0.94909	0.769726	1	GOLGA7B	_
ENSG00000164949	-0.744868	-0.94909	0.769726	1	GEM	K07846
ENSG00000165555	-0.744868	-0.94909	0.769726	1	NOXRED1	_
ENSG00000168743	-0.744868	-0.94909	0.769726	1	NPNT	K06824
ENSG00000171126	-0.744868	-0.94909	0.769726	1	KCNG3	K04902
ENSG00000175600	-0.744868	-0.94909	0.769726	1	SUGCT	K18703
ENSG00000178078	-0.744868	-0.94909	0.769726	1	STAP2	K17985
ENSG00000185634	-0.744868	-0.94909	0.769726	1	SHC4	K17449
ENSG00000199568	-0.744868	-0.94909	0.769726	1	RNU5A-1	_

ENSG00000213062	-0.744868	-0.94909	0.769726	1	AL021068.1	_
ENSG00000221843	-0.744868	-0.94909	0.769726	1	C2orf16	_
ENSG00000223756	-0.744868	-0.94909	0.769726	1	TSSC2	K03842
ENSG00000224959	-0.744868	-0.94909	0.769726	1	AC017002.2	_
ENSG00000226686	-0.744868	-0.94909	0.769726	1	AC012309.5	_
ENSG00000226706	-0.744868	-0.94909	0.769726	1	RP11-426A6.5	_
ENSG00000227653	-0.744868	-0.94909	0.769726	1	ISCA1P6	K13628
ENSG00000236209	-0.744868	-0.94909	0.769726	1	AC104135.2	_
ENSG00000236308	-0.744868	-0.94909	0.769726	1	RP11-316M21.7	_
ENSG00000238048	-0.744868	-0.94909	0.769726	1	AP005019.4	_
ENSG00000243742	-0.744868	-0.94909	0.769726	1	RPLP0P2	K02941
ENSG00000244119	-0.744868	-0.94909	0.769726	1	PDCL3P4	_
ENSG00000248278	-0.744868	-0.94909	0.769726	1	SUMO2P17	K12160
ENSG00000250404	-0.744868	-0.94909	0.769726	1	AC090587.2	_
ENSG00000252481	-0.744868	-0.94909	0.769726	1	SCARNA13	_
ENSG00000253878	-0.744868	-0.94909	0.769726	1	RP11-347C18.3	_
ENSG00000254331	-0.744868	-0.94909	0.769726	1	CKS1BP7	K02219
ENSG00000256196	-0.744868	-0.94909	0.769726	1	RP11-881M11.4	_
ENSG00000257052	-0.744868	-0.94909	0.769726	1	RP11-881M11.2	_
ENSG00000257410	-0.744868	-0.94909	0.769726	1	RP11-2H8.2	_
ENSG00000259086	-0.744868	-0.94909	0.769726	1	RP11-134E15.2	_
ENSG00000259918	-0.744868	-0.94909	0.769726	1	NDUFA5P11	K03949
ENSG00000263006	-0.744868	-0.94909	0.769726	1	ROCK1P1	K04514
ENSG00000265393	-0.744868	-0.94909	0.769726	1	CTD-2517M22.17	_
ENSG00000266446	-0.744868	-0.94909	0.769726	1	RP11-149I2.4	_
ENSG00000271828	-0.744868	-0.94909	0.769726	1	CTD-2310F14.1	_
ENSG00000271964	-0.744868	-0.94909	0.769726	1	RP11-415F23.2	_
ENSG00000272112	-0.744868	-0.94909	0.769726	1	CTB-113P19.5	_
ENSG00000272129	-0.744868	-0.94909	0.769726	1	RP11-250B2.6	_
ENSG00000273366	-0.744868	-0.94909	0.769726	1	CTA-989H11.1	_
ENSG00000273488	-0.744868	-0.94909	0.769726	1	RP11-114I8.4	_
ENSG00000122692	-0.193122	6.890955	0.769799	1	SMU1	K13111
ENSG00000149150	0.200214	4.684252	0.770006	1	SLC43A1	K08228
ENSG00000087448	-0.196033	5.641545	0.770292	1	KLHL42	_
ENSG00000165233	0.201329	4.406481	0.770385	1	C9orf89	_
ENSG00000088899	-0.203811	3.601093	0.770392	1	LZTS3	_
ENSG00000163930	-0.193434	5.976127	0.770469	1	BAP1	K08588
ENSG00000169689	0.193065	6.390311	0.770506	1	STRA13	K15360
ENSG00000116871	0.192025	7.16539	0.770546	1	MAP7D1	K16806
ENSG00000197226	0.19228	6.876935	0.770614	1	TBC1D9B	_
ENSG00000100629	-0.208856	3.436831	0.770634	1	CEP128	K16460
ENSG00000149657	-0.195947	5.128324	0.770635	1	LSM14B	K18749
ENSG00000164983	-0.193267	5.869245	0.770645	1	TMEM65	K04257
ENSG00000198938	-0.190543	12.20716	0.770682	1	MT-CO3	K02262
ENSG00000165322	-0.194667	5.606001	0.770741	1	ARHGAP12	_
ENSG00000154556	0.192278	6.285605	0.770771	1	SORBS2	_
ENSG00000090889	-0.192823	6.498345	0.770829	1	KIF4A	K10395
ENSG00000124181	0.192958	5.716314	0.770916	1	PLCG1	K01116
ENSG00000110429	0.195689	5.256053	0.770929	1	FBXO3	K10290
ENSG00000171492	-0.192469	6.644344	0.770965	1	LRRC8D	_
ENSG00000157881	0.198939	4.895128	0.771055	1	PANK4	K09680
ENSG00000228929	0.209741	3.146792	0.771114	1	RP11-159C21.4	K02953
ENSG00000100403	-0.194267	6.336429	0.771162	1	ZC3H7B	K18047
ENSG00000114650	0.193102	6.212909	0.771163	1	SCAP	_
ENSG00000070814	-0.190946	7.775791	0.771336	1	TCOF1	K14562
ENSG00000198467	0.190286	9.07167	0.771367	1	TPM2	K10374
ENSG00000186187	-0.200604	4.373768	0.771371	1	ZNRF1	K10694
ENSG00000136048	-0.191239	7.336535	0.771414	1	DRAM1	_

ENSG00000134108	0.191932	6.096491	0.771416	1	ARL8B	K07955
ENSG00000126705	0.197381	4.961439	0.771548	1	AHDC1	_
ENSG00000188064	0.193972	5.73786	0.771598	1	WNT7B	K00572
ENSG00000177189	-0.191794	6.712781	0.771631	1	RPS6KA3	K04373
ENSG00000197024	-0.194537	4.62349	0.771648	1	ZNF398	K09228
ENSG00000137944	-0.193031	5.512148	0.771759	1	CCBL2	K00816
ENSG00000115993	0.195142	5.104822	0.771787	1	TRAK2	K15374
ENSG00000186812	-0.219113	2.807509	0.771823	1	ZNF397	K09230
ENSG00000112531	-0.192887	5.926561	0.772141	1	QKI	K14945
ENSG00000100201	0.189646	8.674705	0.772254	1	DDX17	K13178
ENSG00000197386	0.193491	5.614272	0.772301	1	HTT	K04533
ENSG00000099899	-0.193362	5.710949	0.77241	1	TRMT2A	K15332
ENSG00000158604	0.190446	7.066349	0.772504	1	TMED4	_
ENSG00000109814	0.192511	5.796686	0.772507	1	UGDH	K00012
ENSG00000179922	0.21405	2.494278	0.772519	1	ZNF784	_
ENSG00000154370	0.192733	5.881033	0.772593	1	TRIM11	K10650
ENSG00000126214	0.19429	4.7379	0.772819	1	KLC1	K10407
ENSG00000117305	0.200041	4.178262	0.772851	1	HMGCL	K01640
ENSG00000051620	-0.194965	4.706397	0.772932	1	HEBP2	_
ENSG00000188060	-0.196165	4.911052	0.772955	1	RAB42	K07929
ENSG00000135476	0.190851	6.299042	0.772957	1	ESPL1	K02365
ENSG00000114520	-0.193609	5.819678	0.77313	1	SNX4	K17919
ENSG00000160072	-0.193539	5.344332	0.773146	1	ATAD3B	K17681
ENSG00000012232	0.193554	5.579813	0.773204	1	EXTL3	_
ENSG00000072736	0.200944	4.334344	0.773343	1	NFATC3	K17333
ENSG00000147164	0.19277	5.852449	0.773348	1	SNX12	K17918
ENSG00000108100	0.190557	6.009226	0.773419	1	CCNY	_
ENSG00000197747	0.188785	8.322541	0.773421	1	S100A10	K17274
ENSG00000087299	-0.208046	3.619107	0.773461	1	L2HGDH	K00109
ENSG00000136451	-0.19079	6.068475	0.7735	1	VEZF1	_
ENSG00000144182	-0.254244	2.133891	0.773533	1	LIPT1	K10105
ENSG00000231025	-0.254244	2.133891	0.773533	1	RP11-175O19.4	_
ENSG00000260868	-0.254244	2.133891	0.773533	1	RP11-394I13.1	_
ENSG00000140398	0.247728	2.112445	0.773533	1	NEIL1	K10567
ENSG00000189223	0.247728	2.112445	0.773533	1	PAX8-AS1	_
ENSG00000170915	0.225418	2.123499	0.773533	1	PAQR8	_
ENSG00000187775	0.225418	2.123499	0.773533	1	DNAH17	K10408
ENSG00000182318	0.211078	3.049455	0.773552	1	ZSCAN22	K09230
ENSG00000226950	0.197312	4.514147	0.773592	1	DANCR	_
ENSG00000046651	-0.192624	5.662461	0.773696	1	OFD1	K16480
ENSG00000103540	-0.191785	4.883096	0.773698	1	CCP110	K16453
ENSG00000134308	0.188315	8.924134	0.773719	1	YWHAQ	K16197
ENSG00000108106	0.18844	8.516127	0.773731	1	UBE2S	K10583
ENSG00000108960	-0.195094	5.037847	0.773734	1	MMD	_
ENSG00000134057	-0.188492	8.424412	0.773776	1	CCNB1	K05868
ENSG00000236698	-0.194997	5.149464	0.773864	1	EIF1AXP1	K03236
ENSG00000080345	-0.19027	6.465013	0.773866	1	RIF1	K11138
ENSG00000131368	-0.191032	6.099479	0.773951	1	MRPS25	K17404
ENSG00000124275	-0.189041	7.340066	0.773975	1	MTRR	K00597
ENSG00000122642	-0.190093	6.503192	0.773995	1	FKBP9	K09575
ENSG00000118412	0.192835	4.781647	0.774081	1	CASP8AP2	_
ENSG00000137486	0.199711	4.383545	0.774084	1	ARRB1	K04439
ENSG00000120159	-0.193121	5.306078	0.774264	1	CAAP1	_
ENSG00000177084	0.189041	6.935472	0.774309	1	POLE	K02324
ENSG00000151725	-0.191164	6.074338	0.774342	1	CENPU	K11513
ENSG00000176399	0.210617	3.371098	0.7744	1	DMRTA1	K19491
ENSG00000198930	-0.192662	5.376331	0.774435	1	CSAG1	_
ENSG00000107959	-0.190674	5.828191	0.774464	1	PITRM1	K06972

ENSG00000175426	-0.20019	4.031872	0.774538	1	PCSK1	K01359
ENSG00000107521	-0.199384	4.479691	0.774587	1	HPS1	_
ENSG00000113716	0.189156	6.261196	0.774601	1	HMGXB3	_
ENSG00000141570	-0.192133	4.823988	0.774611	1	CBX8	K11455
ENSG00000123388	-0.200261	3.705531	0.77464	1	HOXC11	K09296
ENSG00000065613	-0.18865	7.155998	0.774647	1	SLK	K08836
ENSG00000169972	0.195526	4.786416	0.77476	1	PUSL1	_
ENSG00000112984	0.188625	6.960005	0.774762	1	KIF20A	K10402
ENSG00000100150	0.204242	3.274794	0.774791	1	DEPDC5	_
ENSG00000140104	0.204242	3.274794	0.774791	1	C14orf79	_
ENSG00000158882	-0.204372	3.974525	0.774793	1	TOMM40L	K11518
ENSG00000114120	-0.188415	7.169151	0.774905	1	SLC25A36	K15116
ENSG00000180008	0.190542	5.942988	0.774922	1	SOCS4	K04697
ENSG00000135063	0.278912	1.607319	0.775122	1	FAM189A2	_
ENSG00000186564	0.278912	1.607319	0.775122	1	FOXD2	K09397
ENSG00000225439	0.278912	1.607319	0.775122	1	BOLA3-AS1	_
ENSG00000196357	0.246612	1.623381	0.775122	1	ZNF565	K09228
ENSG00000165502	0.188052	7.044891	0.775324	1	RPL36AL	K02929
ENSG00000182173	-0.189616	5.472235	0.775344	1	TSEN54	K15326
ENSG00000167460	-0.186725	9.102814	0.775562	1	TPM4	K10375
ENSG00000075975	0.190824	5.2364	0.77557	1	MKRN2	K15687
ENSG00000105204	0.226907	2.516985	0.775704	1	DYRK1B	K08825
ENSG00000138002	-0.20635	3.075158	0.775744	1	IFT172	K19676
ENSG00000173221	0.189875	5.28899	0.775806	1	GLRX	K03676
ENSG00000117410	0.187296	7.332096	0.775886	1	ATP6V0B	K03661
ENSG00000107164	-0.188633	6.404752	0.775896	1	FUBP3	K13210
ENSG00000100852	0.188409	6.384529	0.775938	1	ARHGAP5	K13709
ENSG00000173085	-0.19032	5.162973	0.775967	1	COQ2	K06125
ENSG00000122140	-0.187992	6.767713	0.77597	1	MRPS2	K02967
ENSG00000145782	0.187598	6.319513	0.776044	1	ATG12	K08336
ENSG00000143033	-0.187612	6.309078	0.776108	1	MTF2	K11485
ENSG00000104643	-0.217688	3.387578	0.77617	1	MTMR9	K18084
ENSG00000139433	-0.193223	4.653393	0.776171	1	GLTP	K12581
ENSG00000140943	-0.187864	6.726929	0.776187	1	MBTPS1	K08653
ENSG00000103222	0.187672	5.87206	0.776254	1	ABCC1	K05665
ENSG00000179335	0.188051	6.427998	0.776272	1	CLK3	K08823
ENSG00000133687	0.190833	5.498647	0.776484	1	TMTC1	_
ENSG00000170734	0.196765	4.44732	0.776485	1	POLH	K03509
ENSG00000198039	-0.226943	2.363576	0.776539	1	ZNF273	K09228
ENSG00000166167	0.190257	5.052887	0.776567	1	BTRC	K03362
ENSG00000087884	0.195637	4.260744	0.77662	1	AAMDC	K09228
ENSG00000169398	0.186407	7.641105	0.776644	1	PTK2	K05725
ENSG00000040487	-0.200452	3.858408	0.776648	1	PQLC2	_
ENSG00000023330	0.187595	6.509269	0.776655	1	ALAS1	K00643
ENSG00000175467	0.187342	6.211118	0.776659	1	SART1	K11984
ENSG00000075711	0.188653	5.891417	0.776796	1	DLG1	K12076
ENSG00000150093	0.185458	9.979429	0.776812	1	ITGB1	K05719
ENSG00000169174	-0.186176	7.808435	0.776882	1	PCSK9	K13050
ENSG00000107362	0.189411	5.702047	0.776892	1	ABHD17B	K12581
ENSG00000134313	-0.195226	4.215245	0.776909	1	KIDINS220	K12460
ENSG00000120688	-0.189889	5.086365	0.776957	1	WBP4	K13220
ENSG00000128185	-0.191807	5.169078	0.777004	1	DGCR6L	K06262
ENSG00000137478	-0.205212	3.793572	0.777032	1	FCHSD2	_
ENSG00000147813	-0.186037	7.595901	0.777226	1	NAPRT1	K00763
ENSG00000100196	0.188242	5.647048	0.777228	1	KDELR3	K10949
ENSG00000100139	-0.187334	5.629208	0.777345	1	MICALL1	_
ENSG00000101115	-0.200613	3.484469	0.777371	1	SALL4	_
ENSG00000164048	-0.200613	3.484469	0.777371	1	ZNF589	K09228

ENSG00000173451	-0.200613	3.484469	0.777371	1	THAP2	_
ENSG00000175826	-0.186477	7.000095	0.777389	1	CTDNEP1	K17617
ENSG00000111445	-0.187895	5.796222	0.777394	1	RFC5	K10756
ENSG00000186106	-0.192925	4.69797	0.777407	1	ANKRD46	_
ENSG00000104442	-0.186539	6.32993	0.777441	1	ARMC1	K17306
ENSG00000167302	0.200684	3.716556	0.777453	1	ENTHD2	_
ENSG00000260267	-0.245952	2.173042	0.777582	1	RP11-452L6.5	_
ENSG00000108785	0.241839	2.15264	0.777582	1	HSD17B1P1	K00044
ENSG00000227540	0.241839	2.15264	0.777582	1	RP11-152N13.5	_
ENSG00000249279	0.358578	0.823559	0.77773	1	CTC-436P18.3	_
ENSG00000260442	0.358578	0.823559	0.77773	1	RP11-22P6.3	_
ENSG00000263320	0.358578	0.823559	0.77773	1	RP11-498D10.6	_
ENSG00000178199	0.300086	0.852261	0.77773	1	ZC3H12D	K18668
ENSG00000214192	0.300086	0.852261	0.77773	1	UBE2V1P2	K10704
ENSG00000255517	0.300086	0.852261	0.77773	1	CTD-3074O7.5	_
ENSG00000224858	-0.281314	0.870644	0.77773	1	RPL29P11	K02905
ENSG00000267801	-0.281314	0.870644	0.77773	1	RP11-552F3.9	_
ENSG00000185340	-0.189871	5.899661	0.777733	1	GAS2L1	_
ENSG00000143013	-0.185763	7.398124	0.777741	1	LMO4	_
ENSG00000257698	-0.226353	2.697061	0.777827	1	RP11-620J15.3	_
ENSG00000188021	-0.186949	5.781959	0.77784	1	UBQLN2	K04523
ENSG00000143702	-0.190679	5.727586	0.777842	1	CEP170	K16463
ENSG00000101003	-0.192014	4.843961	0.777874	1	GINS1	K10732
ENSG00000157212	-0.192646	4.741211	0.777879	1	PAXIP1	K14972
ENSG00000184307	-0.195505	4.503047	0.777889	1	ZDHHC23	K18932
ENSG00000204272	-0.190248	5.468281	0.77799	1	RP11-622K12.1	_
ENSG00000178951	0.188051	5.327759	0.778009	1	ZBTB7A	K10494
ENSG00000113448	-0.185503	7.352201	0.778094	1	PDE4D	K13293
ENSG00000140905	-0.189014	4.881601	0.778291	1	GCSH	K02437
ENSG00000160014	0.18526	7.281354	0.778317	1	CALM3	K02183
ENSG00000197785	-0.186901	5.802517	0.778394	1	ATAD3A	K17681
ENSG00000127022	0.183922	10.08345	0.778596	1	CANX	K08054
ENSG00000197355	0.192302	4.628601	0.778629	1	UAP1L1	K00972
ENSG00000122674	-0.18854	5.010903	0.778634	1	CCZ1	_
ENSG00000148908	0.189594	5.491835	0.778672	1	RGS10	K16449
ENSG00000162542	-0.199566	3.9349	0.778709	1	TMCO4	_
ENSG00000101557	-0.185102	7.090006	0.778875	1	USP14	K11843
ENSG00000204178	-0.193682	4.224639	0.778884	1	TMEM57	_
ENSG00000162148	-0.397642	0.252986	0.779001	1	PPP1R32	K17571
ENSG00000163113	-0.397642	0.252986	0.779001	1	OTUD7B	K11860
ENSG00000176809	-0.397642	0.252986	0.779001	1	LRRC37A3	_
ENSG00000177842	-0.397642	0.252986	0.779001	1	ZNF620	K09228
ENSG00000235034	-0.397642	0.252986	0.779001	1	C19orf81	_
ENSG00000256028	-0.397642	0.252986	0.779001	1	RP11-197N18.2	_
ENSG00000124140	0.397277	0.146629	0.779001	1	SLC12A5	K14427
ENSG00000150540	0.397277	0.146629	0.779001	1	HNMT	K00546
ENSG00000196873	0.397277	0.146629	0.779001	1	CBWD3	K17985
ENSG00000212789	0.397277	0.146629	0.779001	1	ST13P5	K09560
ENSG00000217275	0.397277	0.146629	0.779001	1	RP1-34B20.4	K02947
ENSG00000228395	0.397277	0.146629	0.779001	1	RP11-216B9.6	_
ENSG00000231305	0.397277	0.146629	0.779001	1	RP11-723O4.2	_
ENSG00000232110	0.397277	0.146629	0.779001	1	RP11-149I23.3	_
ENSG00000251867	0.397277	0.146629	0.779001	1	Y_RNA	_
ENSG00000266490	0.397277	0.146629	0.779001	1	CTD-2349P21.9	_
ENSG00000272374	0.397277	0.146629	0.779001	1	RP3-329A5.8	_
ENSG00000101162	-0.320343	0.208735	0.779001	1	TUBB1	K07375
ENSG00000232818	-0.320343	0.208735	0.779001	1	RPS2P32	K02981
ENSG00000250838	-0.320343	0.208735	0.779001	1	RP11-501C14.5	_

ENSG00000258920	-0.320343	0.208735	0.779001	1	FOXN3-AS1	_
ENSG00000267216	-0.320343	0.208735	0.779001	1	AC010642.1	K09228
ENSG00000269940	-0.320343	0.208735	0.779001	1	RP11-73M18.7	_
ENSG00000139998	0.298884	0.19372	0.779001	1	RAB15	K07908
ENSG00000156265	0.298884	0.19372	0.779001	1	MAP3K7CL	_
ENSG00000185900	0.298884	0.19372	0.779001	1	POMK	K17547
ENSG00000214432	0.298884	0.19372	0.779001	1	AC068831.10	_
ENSG00000226824	0.298884	0.19372	0.779001	1	RP4-756H11.3	_
ENSG00000228705	0.298884	0.19372	0.779001	1	LINC00659	_
ENSG00000231007	0.298884	0.19372	0.779001	1	CDC20P1	K03363
ENSG00000231789	0.298884	0.19372	0.779001	1	RP11-558F24.4	_
ENSG00000244161	0.298884	0.19372	0.779001	1	FLNB-AS1	_
ENSG00000250506	0.298884	0.19372	0.779001	1	CDK3	K02088
ENSG00000260461	0.298884	0.19372	0.779001	1	RP11-541N10.3	_
ENSG00000270820	0.298884	0.19372	0.779001	1	RP11-355B11.2	_
ENSG00000164961	-0.18634	6.063251	0.779037	1	KIAA0196	K18464
ENSG00000108518	-0.183584	9.681656	0.779087	1	PFN1	K05759
ENSG00000164109	-0.185052	6.941772	0.779136	1	MAD2L1	K02537
ENSG00000171295	0.202876	3.205182	0.779213	1	ZNF440	K09228
ENSG00000144791	0.189777	5.154971	0.779245	1	LIMD1	K16682
ENSG00000122779	-0.186366	5.825881	0.779257	1	TRIM24	K08881
ENSG00000168615	0.184118	7.706017	0.77927	1	ADAM9	K06834
ENSG00000126709	-0.18887	4.919742	0.779297	1	IFI6	_
ENSG00000136436	-0.185903	6.305763	0.779314	1	CALCOCO2	_
ENSG00000120742	-0.183839	7.962197	0.779502	1	SERP1	_
ENSG00000163444	-0.185556	6.307978	0.779548	1	TMEM183A	_
ENSG00000179611	0.206617	3.404701	0.779629	1	DGKZP1	K00901
ENSG00000174839	-0.200737	4.122731	0.779664	1	DENND6A	_
ENSG00000103253	-0.18658	5.721789	0.779718	1	HAGHL	K01069
ENSG00000134001	-0.183759	7.778027	0.779735	1	EIF2S1	K03237
ENSG00000196504	-0.183956	7.512938	0.779739	1	PRPF40A	K12821
ENSG00000082258	-0.190056	4.85611	0.779788	1	CCNT2	K15188
ENSG00000181555	0.188272	5.663596	0.779794	1	SETD2	K11423
ENSG00000143162	-0.184884	6.619185	0.779853	1	CREG1	_
ENSG00000158985	0.185133	6.022106	0.779928	1	CDC42SE2	_
ENSG00000005100	-0.184889	5.864752	0.779963	1	DHX33	K17820
ENSG00000255284	0.231051	2.363498	0.779995	1	AP006621.5	_
ENSG00000179163	0.206081	3.091346	0.780003	1	FUCA1	K01206
ENSG00000075399	0.19504	3.096741	0.780003	1	VPS9D1	_
ENSG00000156504	0.184459	6.665302	0.780052	1	FAM122B	_
ENSG00000134508	0.187321	5.722141	0.780067	1	CABLES1	_
ENSG00000130429	-0.183511	7.674803	0.780112	1	ARPC1B	K05757
ENSG00000102893	-0.187028	5.525528	0.780131	1	PHKB	K17985
ENSG00000196141	-0.189223	5.244989	0.780141	1	SPATS2L	_
ENSG00000130159	0.185123	5.607982	0.780254	1	ECSIT	K04405
ENSG00000131446	-0.18469	6.512839	0.780276	1	MGAT1	K00726
ENSG00000162616	-0.184234	6.801903	0.780303	1	DNAJB4	K09510
ENSG00000123595	0.183568	7.25285	0.780327	1	RAB9A	K07899
ENSG00000148606	-0.188594	5.219341	0.780347	1	POLR3A	K03018
ENSG00000184545	0.195837	3.874549	0.780399	1	DUSP8	K04459
ENSG00000182612	-0.208724	2.716693	0.780565	1	TSPAN10	K17351
ENSG00000142409	0.187312	5.568756	0.78058	1	ZNF787	K09228
ENSG00000144306	0.195665	3.50104	0.780595	1	SCRN3	K14358
ENSG00000173039	0.183617	6.114956	0.7806	1	RELA	K04735
ENSG00000153531	-0.282103	1.679802	0.780694	1	ADPRHL1	_
ENSG00000172671	-0.255712	1.664619	0.780694	1	ZFAND4	K12163
ENSG00000148824	-0.194378	4.130198	0.780706	1	MTG1	_
ENSG00000112029	-0.185738	5.593354	0.780795	1	FBXO5	K10292

ENSG00000104889	-0.183323	7.201686	0.780816	1	RNASEH2A	K10743
ENSG00000196586	-0.18566	5.222861	0.780838	1	MYO6	K10358
ENSG00000136848	0.186272	5.485971	0.780883	1	DAB2IP	K17631
ENSG00000166130	-0.183162	7.292811	0.780898	1	IKBIP	K17985
ENSG00000130675	0.21916	2.984236	0.780915	1	MNX1	K08025
ENSG00000130772	0.207204	2.990069	0.780915	1	MED18	K15135
ENSG00000170265	0.187616	5.529384	0.780925	1	ZNF282	K09228
ENSG00000168994	-0.18474	6.030782	0.781053	1	PXDC1	_
ENSG00000148337	-0.183777	6.620526	0.781142	1	CIZ1	_
ENSG00000143198	0.182662	7.424655	0.781215	1	MGST3	K00799
ENSG00000241127	-0.191808	4.293288	0.781342	1	YAE1D1	_
ENSG00000204138	-0.188941	4.900798	0.78138	1	PHACTR4	K17585
ENSG00000101347	-0.205858	3.751894	0.781543	1	SAMHD1	K17985
ENSG00000175573	-0.190285	4.999993	0.781725	1	C11orf68	_
ENSG00000142694	0.195015	4.134002	0.781739	1	EVA1B	_
ENSG00000228305	-0.192746	4.140159	0.781739	1	AC016734.2	_
ENSG00000156709	-0.182453	7.271702	0.781751	1	AIFM1	K04727
ENSG00000154265	-0.185731	5.176358	0.781794	1	ABCA5	K05648
ENSG00000179588	0.20369	3.111841	0.782074	1	ZFPM1	K17441
ENSG00000257727	-0.18395	5.880459	0.782151	1	CNPY2	K01647
ENSG00000103248	-0.202606	3.519328	0.782172	1	MTHFSD	_
ENSG00000213390	-0.195417	3.51538	0.782172	1	ARHGAP19	_
ENSG00000167110	0.182621	6.631163	0.782255	1	GOLGA2	_
ENSG00000104728	0.185165	5.175771	0.782296	1	ARHGEF10	K16727
ENSG00000162066	0.18686	5.229402	0.782297	1	AMDHD2	K01443
ENSG00000188313	-0.184445	5.828199	0.782305	1	PLSCR1	_
ENSG00000136205	-0.184363	5.962154	0.782306	1	TNS3	K18080
ENSG00000124783	-0.181108	8.664421	0.782309	1	SSR1	K13249
ENSG00000100813	0.181842	7.169008	0.782438	1	ACIN1	K12875
ENSG00000068724	0.183018	5.946538	0.782453	1	TTC7A	_
ENSG00000136891	-0.183988	5.759489	0.78264	1	TEX10	K14827
ENSG00000115274	0.191597	4.531365	0.782666	1	INO80B	K11666
ENSG00000021355	0.181677	7.101255	0.782708	1	SERPINB1	K13963
ENSG00000171914	-0.19347	4.254794	0.782734	1	TLN2	_
ENSG00000058272	-0.182591	6.488652	0.782775	1	PPP1R12A	K06270
ENSG00000181991	-0.182441	5.705091	0.782827	1	MRPS11	K02948
ENSG00000168090	-0.181398	7.385622	0.782862	1	COPS6	K12179
ENSG00000185122	0.195925	3.425462	0.782984	1	HSF1	K09414
ENSG00000114107	-0.186311	5.263357	0.783008	1	CEP70	_
ENSG00000116062	0.180729	8.020212	0.783023	1	MSH6	K08737
ENSG00000068137	0.182626	6.174505	0.783064	1	PLEKHH3	_
ENSG00000156261	-0.180204	9.55709	0.78308	1	CCT8	K09500
ENSG00000111142	-0.180768	7.949861	0.783109	1	METAP2	K01265
ENSG00000141447	0.181384	6.059433	0.783125	1	OSBPL1A	K04613
ENSG00000169902	-0.217756	3.011604	0.783139	1	TPST1	K01021
ENSG00000256069	-0.298631	1.341373	0.783156	1	A2MP1	_
ENSG00000203879	0.260153	1.324302	0.783156	1	GDI1	K17255
ENSG00000225695	0.260153	1.324302	0.783156	1	HNRNPA1P35	K12741
ENSG00000267288	0.260153	1.324302	0.783156	1	RP13-890H12.2	_
ENSG00000167770	-0.184794	5.968465	0.783179	1	OTUB1	K09602
ENSG00000162772	0.184169	5.935107	0.783198	1	ATF3	K09032
ENSG00000182220	0.180984	7.383969	0.783219	1	ATP6AP2	K19514
ENSG00000103227	0.226499	2.397327	0.783346	1	LMF1	_
ENSG00000198246	0.226499	2.397327	0.783346	1	SLC29A3	K15014
ENSG00000102554	0.180846	7.380021	0.783385	1	KLF5	K09206
ENSG00000102984	0.224682	1.964351	0.783418	1	ZNF821	K02981
ENSG00000236017	-0.219267	1.973729	0.783418	1	ASMTL-AS1	_
ENSG00000185608	-0.186892	5.317106	0.783439	1	MRPL40	K17421

ENSG00000184903	0.188115	3.832142	0.78348	1	IMMP2L	K09648
ENSG00000115241	0.180274	8.146867	0.783481	1	PPM1G	K17499
ENSG00000185187	0.185751	4.878855	0.783519	1	SIGIRR	K05169
ENSG00000243317	-0.183482	5.63098	0.783588	1	C7orf73	K14444
ENSG00000166734	-0.18126	6.919518	0.783602	1	CASC4	K02927
ENSG00000179295	-0.180582	7.461426	0.78374	1	PTPN11	_
ENSG00000181817	-0.185021	5.133307	0.783848	1	LSM10	_
ENSG00000158417	-0.179867	8.480007	0.783851	1	EIF5B	K03243
ENSG00000132471	0.181219	6.645259	0.783872	1	WBP2	_
ENSG00000121210	-0.188276	4.499132	0.783874	1	KIAA0922	_
ENSG00000151332	-0.188276	4.499132	0.783874	1	MBIP	_
ENSG00000108270	-0.180856	7.058156	0.783883	1	AATF	K14782
ENSG00000104381	-0.19256	3.905871	0.784027	1	GDAP1	_
ENSG00000145214	-0.19256	3.905871	0.784027	1	DGKQ	K00901
ENSG00000177951	0.181862	5.692736	0.784061	1	BET1L	K08504
ENSG00000156671	0.182555	6.020749	0.784129	1	SAMD8	K12581
ENSG00000183691	-0.189427	3.771064	0.784189	1	NOG	K04658
ENSG00000160741	0.181885	5.957879	0.784209	1	CRTC2	K16333
ENSG00000151465	-0.180181	7.420832	0.784251	1	CDC123	_
ENSG00000154864	0.180011	7.430761	0.784316	1	PIEZO2	_
ENSG00000160993	0.196697	3.972837	0.784525	1	ALKBH4	K10766
ENSG00000130748	-0.182517	5.810353	0.784578	1	TMEM160	_
ENSG00000090432	0.19079	4.502616	0.784665	1	MUL1	K15688
ENSG00000213694	0.219065	2.57724	0.784749	1	S1PR3	K04290
ENSG00000090612	0.203153	2.585091	0.784749	1	ZNF268	K09228
ENSG00000204514	0.203153	2.585091	0.784749	1	ZNF814	K09228
ENSG00000106367	-0.180166	6.931419	0.784866	1	AP1S1	K12394
ENSG00000164587	0.178548	9.912461	0.78493	1	RPS14	K02955
ENSG00000198856	-0.180376	6.704865	0.784972	1	OSTC	K14572
ENSG00000042286	0.186647	4.747768	0.784978	1	AIFM2	K11504
ENSG00000156411	-0.181787	5.996881	0.785017	1	C14orf2	K18193
ENSG00000168802	-0.181095	5.829746	0.785026	1	CHTF8	K11270
ENSG00000077458	-0.186275	4.860628	0.7851	1	FAM76B	_
ENSG00000185219	0.191988	3.910057	0.785211	1	ZNF445	K09229
ENSG00000109689	-0.190668	3.917498	0.785211	1	STIM2	K18196
ENSG00000262468	0.215263	2.202164	0.785265	1	RP11-95P2.1	_
ENSG00000073417	0.183389	5.776798	0.785291	1	PDE8A	K18437
ENSG00000159335	-0.179016	7.764211	0.785304	1	PTMS	_
ENSG00000172301	-0.18578	5.144501	0.785379	1	COPRS	_
ENSG00000253738	-0.187038	4.931272	0.785564	1	GS1-251I9.4	_
ENSG00000125967	0.181104	5.517933	0.785672	1	NECAB3	_
ENSG00000121058	-0.182867	5.477161	0.785776	1	COIL	K13150
ENSG00000127870	-0.18103	5.476177	0.785776	1	RNF6	_
ENSG00000007168	0.17883	7.351537	0.785776	1	PAFAH1B1	K16794
ENSG00000048707	0.186038	4.961472	0.785779	1	VPS13D	K19527
ENSG00000071794	0.178708	7.454472	0.785821	1	HLTF	K15711
ENSG00000227671	-0.192293	3.625984	0.785824	1	MIR3916	K09228
ENSG00000204923	-0.244122	1.718494	0.785998	1	FBXO48	K17985
ENSG00000238103	-0.244122	1.718494	0.785998	1	RPL9P7	K02940
ENSG00000099365	0.238385	1.679497	0.785998	1	STX1B	K08486
ENSG00000115239	0.208119	1.694709	0.785998	1	GPR75-ASB3	K10325
ENSG00000116711	-0.182414	4.721369	0.786065	1	PLA2G4A	K16342
ENSG00000119414	-0.179924	6.386563	0.786099	1	PPP6C	K15498
ENSG00000139637	-0.181484	5.765143	0.786154	1	C12orf10	_
ENSG00000132275	-0.183469	5.206337	0.786237	1	RRP8	K14850
ENSG00000137200	-0.183469	5.206337	0.786237	1	CMTR1	K14589
ENSG00000115902	-0.188131	4.868157	0.786327	1	SLC1A4	K05615
ENSG00000064999	0.185462	4.967097	0.78635	1	ANKS1A	_

ENSG00000150990	0.180991	5.310173	0.786417	1	DHX37	K14780
ENSG00000154813	-0.185754	4.563547	0.786495	1	DPH3	K15455
ENSG00000115884	-0.180896	5.975453	0.786506	1	SDC1	K06257
ENSG00000185163	-0.184509	4.686064	0.78651	1	DDX51	K14807
ENSG00000047621	-0.183453	4.798986	0.786523	1	C12orf4	_
ENSG00000169371	-0.182546	4.903708	0.786534	1	SNUPN	K13151
ENSG00000142444	0.184999	4.999017	0.786543	1	C19orf52	K00779
ENSG00000113758	0.178213	7.181141	0.786677	1	DBN1	_
ENSG00000127990	-0.180962	5.437264	0.786712	1	SGCE	_
ENSG00000124098	0.186612	4.166213	0.786761	1	FAM210B	_
ENSG00000149016	0.186612	4.166213	0.786761	1	TUT1	K18709
ENSG00000196683	-0.178173	7.254607	0.786786	1	TOMM7	K17771
ENSG00000104064	-0.184125	5.389977	0.786855	1	GABPB1	K09454
ENSG00000112146	-0.182788	5.314903	0.786865	1	FBXO9	K10295
ENSG00000072803	0.178871	6.438129	0.786976	1	FBXW11	K03362
ENSG00000083720	-0.177323	8.176707	0.787002	1	OXCT1	K01027
ENSG00000110697	0.179168	6.159837	0.787024	1	PITPNM1	_
ENSG00000160208	-0.178433	6.814049	0.787071	1	RRP1B	K14849
ENSG00000105640	0.176712	9.385186	0.787194	1	RPL18A	K02882
ENSG00000171298	0.18178	5.209381	0.7872	1	GAA	K12316
ENSG00000076924	0.178744	6.397054	0.787201	1	XAB2	K12867
ENSG00000103495	0.179771	6.102226	0.787212	1	MAZ	_
ENSG00000167716	0.184013	5.03561	0.787268	1	WDR81	K17601
ENSG00000161021	0.181537	5.391889	0.787279	1	MAML1	K06061
ENSG00000120053	-0.178161	6.348894	0.787345	1	GOT1	K14454
ENSG00000151353	0.188858	4.281258	0.787363	1	TMEM18	_
ENSG00000243364	0.188858	4.281258	0.787363	1	EFNA4	K05462
ENSG00000140990	-0.177755	7.146182	0.787405	1	NDUFB10	K03966
ENSG00000121060	-0.178361	6.650078	0.787425	1	TRIM25	_
ENSG00000178913	-0.177597	7.199925	0.787525	1	TAF7	K03132
ENSG00000171861	-0.182224	5.442312	0.787532	1	RNMTL1	_
ENSG00000107902	-0.194196	3.931969	0.787539	1	LHPP	K11725
ENSG00000164438	-0.194196	3.931969	0.787539	1	TLX3	K15607
ENSG00000131023	-0.18067	4.877107	0.78754	1	LATS1	K08791
ENSG00000118242	0.215384	2.606444	0.787606	1	MREG	_
ENSG00000111247	-0.179629	6.273291	0.787613	1	RAD51AP1	_
ENSG00000135974	0.182327	5.130133	0.787739	1	C2orf49	K15432
ENSG00000061938	0.179741	5.829458	0.787745	1	TNK2	K08886
ENSG00000132017	0.180121	5.318996	0.787756	1	DCAF15	K11791
ENSG00000138279	-0.177384	7.193354	0.787783	1	ANXA7	K17095
ENSG00000245060	-0.203299	3.468762	0.787831	1	LINC00847	_
ENSG00000100938	0.178553	6.178742	0.787841	1	GMPR2	K00364
ENSG00000132591	-0.178183	6.523855	0.787862	1	ERAL1	_
ENSG00000050327	0.188417	3.997865	0.787871	1	ARHGEF5	_
ENSG00000141295	-0.18169	4.946969	0.787884	1	SCRN2	K14358
ENSG00000241837	-0.1768	7.770859	0.7879	1	ATP5O	K02137
ENSG00000179409	-0.178173	6.439025	0.78804	1	GEMIN4	K13132
ENSG00000147121	-0.191284	3.799698	0.78804	1	KRBOX4	K09228
ENSG00000156011	-0.191284	3.799698	0.78804	1	PSD3	K12494
ENSG00000119523	-0.178943	5.903344	0.788091	1	ALG2	K03843
ENSG00000182534	0.178213	6.078387	0.788102	1	MXRA7	_
ENSG00000139405	-0.179247	5.85192	0.788143	1	RITA1	_
ENSG00000028528	0.177643	6.512124	0.788281	1	SNX1	K17917
ENSG00000117266	0.182175	4.914231	0.788303	1	CDK18	K15596
ENSG00000134255	-0.179468	5.350779	0.788317	1	CEPT1	K13644
ENSG00000160803	-0.17797	6.385521	0.788387	1	UBQLN4	K04523
ENSG00000254535	0.196117	2.770194	0.788394	1	PABPC4L	K13126
ENSG00000130227	0.176888	6.292859	0.788588	1	XPO7	K18460

ENSG00000181026	-0.178047	6.021382	0.78862	1	AEN	K18340
ENSG00000173276	-0.182291	5.518854	0.788656	1	ZBTB21	_
ENSG00000205643	0.18343	3.936192	0.788685	1	CDPF1	_
ENSG00000198815	0.177992	6.05349	0.788756	1	FOXJ3	K09403
ENSG00000260077	-0.230704	2.248325	0.788912	1	RP11-254F7.2	_
ENSG00000103175	-0.213038	2.238356	0.788912	1	WFDC1	_
ENSG00000129493	0.178839	5.83869	0.788987	1	HEATR5A	_
ENSG00000015133	-0.197897	2.927122	0.789028	1	CCDC88C	_
ENSG00000100711	-0.181287	4.662016	0.78908	1	ZFYVE21	_
ENSG00000135912	-0.181287	4.662016	0.78908	1	TTLL4	K16601
ENSG00000247077	-0.177005	5.876746	0.789132	1	PGAM5	K15637
ENSG00000205730	-0.17648	6.951046	0.789158	1	ITPRIPL2	K17985
ENSG00000143079	-0.180884	4.988971	0.789159	1	CTTNBP2NL	K12795
ENSG00000164818	0.17646	6.297329	0.789264	1	HEATR2	K19759
ENSG00000114770	-0.178904	5.38264	0.789279	1	ABCC5	K05668
ENSG00000198952	0.176284	6.790692	0.789432	1	SMG5	K11125
ENSG00000152223	0.185434	4.129086	0.789451	1	EPG5	K17985
ENSG00000173218	-0.178144	5.808014	0.789466	1	VANGL1	K04510
ENSG00000235363	-0.210037	3.054281	0.789549	1	SNRPGP10	K11099
ENSG00000164053	-0.190862	3.879291	0.78957	1	ATRIP	K10905
ENSG00000100316	0.174439	11.20149	0.78962	1	RPL3	K02925
ENSG00000147100	0.287666	1.373975	0.789628	1	SLC16A2	K08231
ENSG00000231841	0.287666	1.373975	0.789628	1	RP11-206F17.2	_
ENSG00000235374	0.287666	1.373975	0.789628	1	SSR4P1	K04571
ENSG00000261353	0.287666	1.373975	0.789628	1	CTA-14H9.5	_
ENSG00000109680	-0.281892	1.408368	0.789628	1	TBC1D19	_
ENSG00000130066	0.175338	7.550298	0.789693	1	SAT1	K00657
ENSG00000138463	0.184931	4.665424	0.789771	1	DIRC2	K15381
ENSG00000133027	-0.180948	5.024411	0.789827	1	PEMT	K00551
ENSG00000108587	-0.178079	5.654663	0.789894	1	GOSR1	K08495
ENSG00000119421	-0.177422	6.24988	0.789979	1	NDUFA8	K03952
ENSG00000225210	-0.189442	3.175269	0.789984	1	AL589743.1	_
ENSG00000123146	0.175284	7.20542	0.790087	1	CD97	K08446
ENSG00000235655	0.175791	6.692261	0.790157	1	H3F3AP4	K11253
ENSG00000131747	0.174235	9.180653	0.790159	1	TOP2A	K03164
ENSG00000161011	0.174644	7.953749	0.790209	1	SQSTM1	K14381
ENSG00000198663	0.176061	5.88324	0.790328	1	C6orf89	K12733
ENSG00000170540	0.17467	7.662434	0.790386	1	ARL6IP1	_
ENSG00000110171	0.183697	4.711387	0.79058	1	TRIM3	K11997
ENSG00000198727	0.173621	11.03795	0.790596	1	MT-CYB	K00412
ENSG00000125656	-0.175985	6.440042	0.790599	1	CLPP	K01358
ENSG00000130699	-0.183418	4.19853	0.790617	1	TAF4	K03129
ENSG00000239264	-0.178097	4.932681	0.790618	1	TXNDC5	K13984
ENSG00000101773	-0.176788	6.020709	0.790733	1	RBBP8	_
ENSG00000197903	0.178127	5.937964	0.790775	1	HIST1H2BK	K11252
ENSG00000166326	-0.177908	5.870766	0.790872	1	TRIM44	K12020
ENSG00000174028	-0.207256	2.800896	0.790883	1	FAM3C2	_
ENSG00000266208	-0.207256	2.800896	0.790883	1	CTD-2267D19.3	K07627
ENSG00000002330	-0.184807	4.308796	0.790928	1	BAD	K02158
ENSG00000198171	-0.180203	5.064248	0.790985	1	DDRGK1	_
ENSG00000034713	0.175239	5.922845	0.791015	1	GABARAPL2	K08341
ENSG00000169914	0.180802	4.360367	0.791067	1	OTUD3	K13717
ENSG00000086200	-0.176948	6.055386	0.791087	1	IPO11	_
ENSG00000146872	-0.177309	5.600601	0.79112	1	TLK2	K08864
ENSG00000129932	-0.182379	4.677517	0.791141	1	DOHH	K06072
ENSG00000156931	-0.182379	4.677517	0.791141	1	VPS8	_
ENSG00000145740	-0.175167	6.61207	0.791234	1	SLC30A5	K14692
ENSG00000086475	-0.175419	6.450604	0.791242	1	SEPHS1	K01008

ENSG00000161980	-0.17574	6.132367	0.791282	1	POLR3K	K03019
ENSG00000155506	0.173669	8.026752	0.79131	1	LARP1	K18757
ENSG00000164889	0.173729	7.667836	0.791489	1	SLC4A2	K13855
ENSG00000185722	0.174133	6.009194	0.791503	1	ANKFY1	_
ENSG00000085788	0.177796	5.098465	0.791577	1	DDHD2	K16545
ENSG00000111653	0.177696	4.594569	0.791637	1	ING4	K11346
ENSG00000104979	0.174025	7.127826	0.791652	1	C19orf53	_
ENSG00000198791	-0.175115	6.397534	0.791705	1	CNOT7	K12581
ENSG00000103197	0.176984	5.707926	0.791714	1	TSC2	K07207
ENSG00000161204	0.175469	6.247937	0.791785	1	ABCF3	K06158
ENSG00000065491	0.181384	4.310364	0.791801	1	TBC1D22B	_
ENSG00000129968	0.173775	6.027476	0.791808	1	ABHD17A	_
ENSG00000197601	-0.174331	6.851735	0.791823	1	FAR1	K13356
ENSG00000110422	0.177714	5.785571	0.79188	1	HIPK3	K08826
ENSG00000115468	0.218876	2.008805	0.791892	1	EFHD1	_
ENSG00000198561	-0.174106	6.853252	0.792085	1	CTNND1	K05690
ENSG00000151338	-0.197361	3.300863	0.792102	1	MIPOL1	_
ENSG00000088448	0.173528	7.236596	0.792116	1	ANKRD10	_
ENSG00000165271	0.174101	6.70114	0.792125	1	NOL6	K14544
ENSG00000213977	-0.183405	4.034374	0.792176	1	TAX1BP3	_
ENSG00000164045	-0.178136	5.16173	0.792196	1	CDC25A	K06645
ENSG00000196352	0.17223	11.12913	0.792228	1	CD55	K04006
ENSG00000142178	-0.172521	9.123003	0.792232	1	SIK1	K19008
ENSG00000160446	-0.180172	4.646346	0.792424	1	ZDHHC12	K18932
ENSG00000050438	0.357477	0.297976	0.792443	1	SLC4A8	K13859
ENSG00000129282	0.357477	0.297976	0.792443	1	MRM1	K15507
ENSG00000223802	0.357477	0.297976	0.792443	1	CERS1	K04710
ENSG00000231113	0.357477	0.297976	0.792443	1	RP3-475N16.1	_
ENSG00000231443	0.357477	0.297976	0.792443	1	AC024937.6	K15425
ENSG00000188554	-0.173153	7.477779	0.792444	1	NBR1	K17987
ENSG00000142675	0.180727	3.493526	0.792468	1	CNKSR1	_
ENSG00000090520	-0.173523	7.058516	0.792483	1	DNAJB11	K09517
ENSG00000147162	0.172731	7.876838	0.792511	1	OGT	K09667
ENSG00000122882	-0.177359	5.612293	0.792552	1	ECD	_
ENSG00000198089	-0.179542	4.803686	0.792677	1	SFI1	K17985
ENSG00000130313	-0.174062	6.525773	0.792688	1	PGLS	K01057
ENSG00000143761	0.172135	8.649463	0.792808	1	ARF1	K07937
ENSG00000196456	-0.223738	2.47029	0.792819	1	ZNF775	_
ENSG00000187147	-0.175528	6.020037	0.79285	1	RNF220	_
ENSG00000102580	-0.175931	5.657272	0.792962	1	DNAJC3	K09523
ENSG00000184117	-0.172529	7.534664	0.793123	1	NIPSNAP1	_
ENSG00000213625	-0.173187	6.822999	0.79321	1	LEPROT	K17985
ENSG00000160796	-0.176041	5.57233	0.793211	1	NBEAL2	_
ENSG00000169139	-0.172432	7.502643	0.793267	1	UBE2V2	K10704
ENSG00000140395	-0.173543	6.204857	0.793323	1	WDR61	K12602
ENSG00000130023	-0.204779	2.955843	0.793482	1	ERMARD	_
ENSG00000183808	0.17547	5.595323	0.793594	1	RBM12B	_
ENSG00000166471	-0.173435	5.959359	0.793594	1	TMEM41B	_
ENSG00000142494	-0.193066	3.090377	0.793618	1	SLC47A1	K03327
ENSG00000092871	-0.183296	3.084993	0.793618	1	RFFL	K10871
ENSG00000132323	-0.179753	4.477602	0.793658	1	ILKAP	K17500
ENSG00000066135	-0.174723	5.72263	0.793697	1	KDM4A	K06709
ENSG00000135316	-0.17166	7.963317	0.7938	1	SYNCRIP	K13160
ENSG00000111907	0.172989	6.44669	0.793853	1	TPD52L1	_
ENSG00000149782	-0.174853	5.744047	0.794036	1	PLCB3	K05858
ENSG00000213799	-0.185217	3.91453	0.794203	1	ZNF845	K09228
ENSG00000119013	-0.175825	5.416892	0.794231	1	NDUFB3	K03959
ENSG00000099290	-0.186725	4.04772	0.794264	1	FAM21A	K18462

ENSG00000138785	-0.182169	4.435706	0.794411	1	INTS12	K13149
ENSG00000196712	-0.173914	5.965683	0.794426	1	NF1	K08052
ENSG00000173875	0.177428	4.479278	0.794426	1	ZNF791	K09228
ENSG00000140284	0.172355	6.527413	0.794455	1	SLC27A2	K08746
ENSG00000027847	-0.178043	4.660268	0.794478	1	B4GALT7	K00733
ENSG00000197961	0.17336	6.294527	0.794512	1	ZNF121	K09228
ENSG00000196199	-0.175211	5.420989	0.794633	1	MPHOSPH8	_
ENSG00000021762	0.174935	5.581641	0.794656	1	OSBPL5	_
ENSG00000130312	-0.173279	6.269905	0.794729	1	MRPL34	K02914
ENSG00000143314	-0.172456	6.387109	0.794844	1	MRPL24	K02895
ENSG00000066279	-0.171566	6.940759	0.79494	1	ASPM	K16743
ENSG00000077147	-0.170726	7.880204	0.794959	1	TM9SF3	K17087
ENSG00000228300	-0.173624	5.986567	0.794975	1	C19orf24	_
ENSG00000162385	-0.175362	5.447341	0.795031	1	MAGOH	K12877
ENSG00000158186	-0.175381	5.212248	0.795062	1	MRAS	K07831
ENSG00000109381	0.17239	5.181849	0.795066	1	ELF2	K09428
ENSG00000104218	-0.175488	5.095336	0.795079	1	CSPP1	K16771
ENSG00000196214	-0.175409	4.931237	0.795106	1	ZNF766	K09228
ENSG00000230330	-0.303361	0.988168	0.795106	1	HMGN2P3	K11300
ENSG00000267534	-0.303361	0.988168	0.795106	1	S1PR2	K04292
ENSG00000010295	0.282388	0.946849	0.795106	1	IFFO1	_
ENSG00000164620	0.282388	0.946849	0.795106	1	RELL2	_
ENSG00000213928	0.282388	0.946849	0.795106	1	IRF9	K04693
ENSG00000140511	0.23024	0.973007	0.795106	1	HAPLN3	K06852
ENSG00000185437	0.23024	0.973007	0.795106	1	SH3BGR	_
ENSG00000269963	0.23024	0.973007	0.795106	1	RP11-73M18.9	_
ENSG00000170515	-0.170242	8.578375	0.795122	1	PA2G4	_
ENSG00000042753	-0.170974	7.362958	0.795122	1	AP2S1	K11827
ENSG00000142784	0.1783	4.661993	0.795153	1	WDTC1	K11807
ENSG00000140941	0.171839	6.286673	0.795171	1	MAP1LC3B	K14230
ENSG00000230055	-0.172148	6.389203	0.795201	1	CISD3	K11460
ENSG00000160218	0.172738	5.918494	0.795265	1	TRAPPC10	_
ENSG00000221838	-0.185131	3.988188	0.795311	1	AP4M1	K12402
ENSG00000157911	0.183462	3.686449	0.795396	1	PEX10	K13346
ENSG00000119321	-0.175648	5.498643	0.795405	1	FKBP15	K17478
ENSG00000008382	0.185811	3.520454	0.79545	1	MPND	_
ENSG00000184967	-0.174514	5.066853	0.795596	1	NOC4L	K14771
ENSG00000177042	0.190598	2.820875	0.795689	1	TMEM80	_
ENSG00000248569	-0.235825	1.454688	0.795724	1	CTC-512J14.5	K15170
ENSG00000258985	-0.235825	1.454688	0.795724	1	RP11-368P15.3	_
ENSG00000137574	-0.174163	5.547247	0.795755	1	TGS1	K14292
ENSG00000125898	0.206157	2.276739	0.795849	1	FAM110A	K05467
ENSG00000188931	0.206157	2.276739	0.795849	1	C1orf192	_
ENSG00000186487	0.252219	1.771325	0.795875	1	MYT1L	_
ENSG00000228672	0.252219	1.771325	0.795875	1	PROB1	_
ENSG00000182185	-0.233411	1.770449	0.795875	1	RAD51B	K10869
ENSG00000138660	-0.171943	5.328045	0.795901	1	AP1AR	_
ENSG00000164172	-0.172582	5.132035	0.796053	1	MOCS2	K03635
ENSG00000172340	0.17168	5.976104	0.796068	1	SUCLG2	K01900
ENSG00000123143	-0.17004	7.437409	0.796145	1	PKN1	K06071
ENSG00000077454	-0.181969	4.238538	0.79615	1	LRCH4	_
ENSG00000058600	0.173148	6.090665	0.796228	1	POLR3E	K14721
ENSG00000150753	-0.168893	10.46264	0.796238	1	CCT5	K09497
ENSG00000114745	0.178046	4.865393	0.79629	1	GORASP1	_
ENSG00000070269	-0.183403	3.999175	0.796375	1	TMEM260	_
ENSG00000137834	0.171932	5.927174	0.796393	1	SMAD6	K04677
ENSG00000167889	0.176628	4.714766	0.796447	1	MGAT5B	K09661
ENSG00000114784	-0.177772	4.591039	0.796589	1	EIF1B	K03113

ENSG00000096060	0.170025	5.963496	0.796631	1	FKBP5	K09571
ENSG00000108582	0.169625	7.303678	0.796638	1	CPD	K07752
ENSG00000114346	-0.169335	7.71839	0.796722	1	ECT2	_
ENSG00000165688	-0.16979	7.175008	0.796725	1	PMPCA	K01412
ENSG00000177663	0.181079	4.4004	0.796823	1	IL17RA	K05164
ENSG00000091483	-0.169472	7.427264	0.796824	1	FH	K01679
ENSG00000251322	0.175131	4.831893	0.796922	1	SHANK3	K15009
ENSG00000100325	0.170629	5.685069	0.797009	1	ASCC2	K18667
ENSG00000213326	0.178954	4.242451	0.797044	1	RPS7P11	K02993
ENSG00000117676	0.170603	6.3001	0.797135	1	RPS6KA1	K04373
ENSG00000132024	0.171321	5.785563	0.797175	1	CC2D1A	K18260
ENSG00000167291	0.171321	5.785563	0.797175	1	TBC1D16	_
ENSG00000159579	-0.171641	5.013576	0.797216	1	RSPRY1	K17866
ENSG00000198242	-0.168497	8.497154	0.797216	1	RPL23A	K02893
ENSG00000123416	-0.168191	9.564997	0.797218	1	TUBA1B	K07374
ENSG00000141552	0.172016	5.862262	0.797351	1	ANAPC11	K03358
ENSG00000257621	-0.179944	4.552351	0.797366	1	RP11-349A22.5	_
ENSG00000187953	-0.181605	3.937556	0.797547	1	PMS2CL	K10858
ENSG00000004700	0.169372	6.706596	0.797672	1	RECQL	K10899
ENSG00000111832	0.169422	6.649157	0.797702	1	RWDD1	_
ENSG00000146909	-0.171375	5.628332	0.797792	1	NOM1	K17583
ENSG00000148356	0.175521	4.982478	0.797806	1	LRSAM1	K10641
ENSG00000072071	0.171084	5.583471	0.797882	1	LPHN1	K04592
ENSG00000164040	-0.168397	7.572701	0.797952	1	PGRMC2	K17278
ENSG00000166441	0.167229	10.91177	0.798143	1	RPL27A	K02900
ENSG00000132670	-0.178639	4.510655	0.798186	1	PTPRA	K18032
ENSG00000125901	-0.170464	5.870028	0.798223	1	MRPS26	K17405
ENSG00000119285	-0.17109	5.834374	0.798309	1	HEATR1	K14550
ENSG00000151690	-0.213548	2.689893	0.798318	1	MFSD6	_
ENSG00000074416	0.168715	6.77752	0.798341	1	MGLL	K01054
ENSG00000073169	0.170447	5.795079	0.7984	1	SELO	_
ENSG00000166128	-0.177021	4.415276	0.79841	1	RAB8B	K07902
ENSG00000163714	-0.167573	8.226769	0.798448	1	U2SURP	K12842
ENSG00000215252	-0.172021	5.890115	0.798467	1	GOLGA8B	K10582
ENSG00000160216	-0.170027	5.873023	0.798512	1	AGPAT3	K13523
ENSG00000075790	-0.168627	5.872277	0.798512	1	BCAP29	K05545
ENSG00000198824	-0.197807	2.523821	0.798691	1	CHAMP1	_
ENSG00000132470	0.19721	2.50262	0.798691	1	ITGB4	K06525
ENSG00000099814	0.170939	5.544506	0.798702	1	CEP170B	K16463
ENSG00000122952	-0.169443	6.200107	0.798706	1	ZWINT	_
ENSG00000233830	0.170914	4.810172	0.798788	1	EIF4HP1	_
ENSG00000130726	0.167096	8.361641	0.798872	1	TRIM28	K08882
ENSG00000162613	0.167622	7.415098	0.79889	1	FUBP1	K13210
ENSG00000140598	-0.170538	5.322578	0.798909	1	EFTUD1	K14536
ENSG00000137815	0.168536	6.5274	0.798941	1	RTF1	K15178
ENSG00000104853	0.168375	6.64126	0.798944	1	CLPTM1	_
ENSG00000096063	-0.167608	7.295398	0.799157	1	SRPK1	K15409
ENSG00000164930	-0.169054	6.160836	0.799297	1	FZD6	K02376
ENSG00000140995	0.170594	5.548263	0.799422	1	DEF8	_
ENSG00000185950	-0.168416	6.464091	0.799433	1	IRS2	K07187
ENSG00000113391	-0.186467	3.13084	0.799437	1	FAM172A	_
ENSG00000167130	0.175742	4.014389	0.799504	1	DOLPP1	K07252
ENSG00000125812	0.172051	4.314853	0.799506	1	GZF1	_
ENSG00000172845	0.167807	6.686277	0.799543	1	SP3	K09193
ENSG00000129933	0.172818	5.503493	0.799568	1	MAU2	K11266
ENSG00000133131	-0.170018	4.928345	0.799612	1	MORC4	_
ENSG00000188971	0.171257	4.736354	0.79963	1	RP11-427H3.3	_
ENSG00000047056	0.182279	3.947627	0.799717	1	WDR37	_

ENSG00000167515	0.169122	5.860748	0.799721	1	TRAPPC2L	_
ENSG00000224660	0.230948	2.082488	0.799731	1	SH3BP5-AS1	_
ENSG00000064726	-0.167051	7.30707	0.799801	1	BTBD1	K10477
ENSG00000184708	0.173748	5.030272	0.799808	1	EIF4ENIF1	K18728
ENSG00000134970	-0.167681	6.734021	0.79982	1	TMED7	K14825
ENSG00000130590	-0.19717	3.000181	0.799824	1	SAMD10	_
ENSG00000179051	-0.16678	7.580744	0.79985	1	RCC2	_
ENSG00000073910	-0.533966	-0.648321	0.7999	1	FRY	_
ENSG00000102904	-0.533966	-0.648321	0.7999	1	TSNAXIP1	_
ENSG00000120217	-0.533966	-0.648321	0.7999	1	CD274	K06745
ENSG00000129226	-0.533966	-0.648321	0.7999	1	CD68	K06501
ENSG00000135083	-0.533966	-0.648321	0.7999	1	CCNJL	_
ENSG00000136367	-0.533966	-0.648321	0.7999	1	ZFHX2	K09379
ENSG00000140403	-0.533966	-0.648321	0.7999	1	DNAJA4	K09505
ENSG00000174125	-0.533966	-0.648321	0.7999	1	TLR1	_
ENSG00000178096	-0.533966	-0.648321	0.7999	1	BOLA1	K05527
ENSG00000178665	-0.533966	-0.648321	0.7999	1	ZNF713	_
ENSG00000197914	-0.533966	-0.648321	0.7999	1	HIST1H4K	K11254
ENSG00000198156	-0.533966	-0.648321	0.7999	1	NPIPB6	K01896
ENSG00000198750	-0.533966	-0.648321	0.7999	1	GATSL2	_
ENSG00000205143	-0.533966	-0.648321	0.7999	1	ARID3C	_
ENSG00000214402	-0.533966	-0.648321	0.7999	1	LCNL1	_
ENSG00000224094	-0.533966	-0.648321	0.7999	1	RPS24P8	K02974
ENSG00000227256	-0.533966	-0.648321	0.7999	1	MIS18A-AS1	_
ENSG00000227606	-0.533966	-0.648321	0.7999	1	AC005392.13	_
ENSG00000232936	-0.533966	-0.648321	0.7999	1	RP11-80H5.2	_
ENSG00000233325	-0.533966	-0.648321	0.7999	1	MIPEPP3	K01410
ENSG00000236137	-0.533966	-0.648321	0.7999	1	RP11-27K13.3	_
ENSG00000237118	-0.533966	-0.648321	0.7999	1	CYP2F2P	K07416
ENSG00000237522	-0.533966	-0.648321	0.7999	1	NONOP2	K13214
ENSG00000253608	-0.533966	-0.648321	0.7999	1	RP11-770E5.1	_
ENSG00000255725	-0.533966	-0.648321	0.7999	1	RP11-443N24.1	K03649
ENSG00000267342	-0.533966	-0.648321	0.7999	1	RP11-552F3.10	_
ENSG00000267523	-0.533966	-0.648321	0.7999	1	CTD-2537I9.12	_
ENSG00000267904	-0.533966	-0.648321	0.7999	1	CTC-429P9.5	_
ENSG00000269680	-0.533966	-0.648321	0.7999	1	CTD-3128G10.6	_
ENSG00000269843	-0.533966	-0.648321	0.7999	1	CTC-490E21.10	_
ENSG00000272195	-0.533966	-0.648321	0.7999	1	RP11-156E8.1	_
ENSG00000272205	-0.533966	-0.648321	0.7999	1	RP11-277B15.3	_
ENSG00000272556	-0.533966	-0.648321	0.7999	1	RP11-638I8.1	_
ENSG00000272960	-0.533966	-0.648321	0.7999	1	RP11-339B21.15	_
ENSG00000105808	0.443254	-0.810055	0.7999	1	RASA4	K17630
ENSG00000127533	0.443254	-0.810055	0.7999	1	F2RL3	K04236
ENSG00000135636	0.443254	-0.810055	0.7999	1	DYSF	K18261
ENSG00000139656	0.443254	-0.810055	0.7999	1	SMIM2	K02889
ENSG00000140986	0.443254	-0.810055	0.7999	1	RPL3L	K02925
ENSG00000141505	0.443254	-0.810055	0.7999	1	ASGR1	K10063
ENSG00000178150	0.443254	-0.810055	0.7999	1	ZNF114	K09228
ENSG00000189149	0.443254	-0.810055	0.7999	1	CRYM-AS1	_
ENSG00000204099	0.443254	-0.810055	0.7999	1	NEU4	K12357
ENSG00000224165	0.443254	-0.810055	0.7999	1	DNAJC27-AS1	_
ENSG00000225022	0.443254	-0.810055	0.7999	1	UBE2D3P1	K06689
ENSG00000228060	0.443254	-0.810055	0.7999	1	RP11-69E11.8	_
ENSG00000230641	0.443254	-0.810055	0.7999	1	USP12-AS2	_
ENSG00000231628	0.443254	-0.810055	0.7999	1	RP3-355L5.4	_
ENSG00000231851	0.443254	-0.810055	0.7999	1	AC104655.2	_
ENSG00000234297	0.443254	-0.810055	0.7999	1	RP11-54D18.4	_
ENSG00000234962	0.443254	-0.810055	0.7999	1	LINC00700	_

ENSG00000235398	0.443254	-0.810055	0.7999	1	LINC00623	_
ENSG00000235513	0.443254	-0.810055	0.7999	1	RP4-756G23.5	_
ENSG00000237748	0.443254	-0.810055	0.7999	1	UQCRBP1	K00417
ENSG00000243389	0.443254	-0.810055	0.7999	1	AC012442.5	_
ENSG00000243415	0.443254	-0.810055	0.7999	1	RP11-274H2.2	_
ENSG00000247950	0.443254	-0.810055	0.7999	1	SEC24B-AS1	_
ENSG00000249456	0.443254	-0.810055	0.7999	1	RP11-298J20.4	_
ENSG00000251485	0.443254	-0.810055	0.7999	1	AC068134.10	K18758
ENSG00000254143	0.443254	-0.810055	0.7999	1	RP11-470M17.2	_
ENSG00000258038	0.443254	-0.810055	0.7999	1	CTD-2384A14.1	_
ENSG00000260279	0.443254	-0.810055	0.7999	1	AC137932.5	_
ENSG00000265500	0.443254	-0.810055	0.7999	1	SENP3-EIF4A1	_
ENSG00000266824	0.443254	-0.810055	0.7999	1	RP11-599B13.7	_
ENSG00000266930	0.443254	-0.810055	0.7999	1	CTD-2085J24.4	_
ENSG00000267160	0.443254	-0.810055	0.7999	1	RP11-1072C15.4	_
ENSG00000268179	0.443254	-0.810055	0.7999	1	AL645608.1	_
ENSG00000269501	0.443254	-0.810055	0.7999	1	RP11-353N4.6	K15258
ENSG00000273261	0.443254	-0.810055	0.7999	1	RP11-531F16.4	_
ENSG00000139908	-0.367345	-0.735611	0.7999	1	TSSK4	K08811
ENSG00000149531	-0.367345	-0.735611	0.7999	1	FRG1B	K13122
ENSG00000157950	-0.367345	-0.735611	0.7999	1	SSX2B	K15625
ENSG00000159915	-0.367345	-0.735611	0.7999	1	ZNF233	K09228
ENSG00000159958	-0.367345	-0.735611	0.7999	1	TNFRSF13C	K05151
ENSG00000160973	-0.367345	-0.735611	0.7999	1	FOXH1	K09400
ENSG00000160999	-0.367345	-0.735611	0.7999	1	SH2B2	K07193
ENSG00000161270	-0.367345	-0.735611	0.7999	1	NPHS1	_
ENSG00000165757	-0.367345	-0.735611	0.7999	1	KIAA1462	_
ENSG00000179044	-0.367345	-0.735611	0.7999	1	EXOC3L1	_
ENSG00000182264	-0.367345	-0.735611	0.7999	1	IZUMO1	K17985
ENSG00000197210	-0.367345	-0.735611	0.7999	1	KB-1592A4.15	_
ENSG00000206622	-0.367345	-0.735611	0.7999	1	SNORA69	_
ENSG00000213793	-0.367345	-0.735611	0.7999	1	ZNF888	_
ENSG00000213885	-0.367345	-0.735611	0.7999	1	RPL13AP7	K02872
ENSG00000224358	-0.367345	-0.735611	0.7999	1	RP11-466F5.8	_
ENSG00000231925	-0.367345	-0.735611	0.7999	1	TAPBP	K08058
ENSG00000232774	-0.367345	-0.735611	0.7999	1	RP11-47I22.3	_
ENSG00000234460	-0.367345	-0.735611	0.7999	1	XXyac-YM21GA2.4	_
ENSG00000236651	-0.367345	-0.735611	0.7999	1	AC104801.1	_
ENSG00000237101	-0.367345	-0.735611	0.7999	1	RP11-365O16.6	_
ENSG00000250295	-0.367345	-0.735611	0.7999	1	RP11-434I12.2	_
ENSG00000250764	-0.367345	-0.735611	0.7999	1	CTD-2152M20.2	_
ENSG00000253051	-0.367345	-0.735611	0.7999	1	SNORA31	_
ENSG00000254317	-0.367345	-0.735611	0.7999	1	RP11-473O4.5	_
ENSG00000258922	-0.367345	-0.735611	0.7999	1	CTD-2313J17.1	_
ENSG00000259024	-0.367345	-0.735611	0.7999	1	TVP23C-CDRT4	_
ENSG00000260136	-0.367345	-0.735611	0.7999	1	CTD-2270L9.4	_
ENSG00000260526	-0.367345	-0.735611	0.7999	1	RP11-73K9.2	_
ENSG00000261428	-0.367345	-0.735611	0.7999	1	RP11-16P6.1	_
ENSG00000264647	-0.367345	-0.735611	0.7999	1	RP11-68I3.7	_
ENSG00000267185	-0.367345	-0.735611	0.7999	1	PTP4A2P1	K18041
ENSG00000270041	-0.367345	-0.735611	0.7999	1	LL22NC03-N27C7.1	_
ENSG00000270344	-0.367345	-0.735611	0.7999	1	RP11-734K2.4	_
ENSG00000271404	-0.367345	-0.735611	0.7999	1	RP11-365F18.5	_
ENSG00000272153	-0.367345	-0.735611	0.7999	1	RP1-286D6.5	_
ENSG00000273212	-0.367345	-0.735611	0.7999	1	AC000068.10	_
ENSG00000128335	-0.172921	5.275838	0.799931	1	APOL2	K14480
ENSG00000134470	-0.177816	3.885078	0.799951	1	IL15RA	K05074
ENSG00000130713	-0.168751	6.308579	0.799952	1	EXOSC2	K03679

ENSG00000076382	0.166768	7.272777	0.800034	1	SPAG5	_
ENSG00000172466	-0.169331	6.150385	0.800055	1	ZNF24	K09230
ENSG00000230733	-0.175708	4.152377	0.800069	1	AC092171.4	_
ENSG00000072310	0.166617	7.306162	0.800178	1	SREBF1	K07197
ENSG00000163818	0.173008	4.52577	0.800377	1	LZTFL1	K19400
ENSG00000204217	0.176283	4.270031	0.800544	1	BMPR2	K04671
ENSG00000245910	0.166411	7.170462	0.800562	1	SNHG6	_
ENSG00000198625	-0.169543	4.970888	0.800572	1	MDM4	K10127
ENSG00000013583	0.168577	5.866776	0.800584	1	HEBP1	_
ENSG00000003402	-0.171293	5.070763	0.800698	1	CFLAR	K04724
ENSG00000215012	0.16949	5.752564	0.800738	1	C22orf29	_
ENSG00000198125	-0.181178	3.65046	0.800811	1	MB	_
ENSG00000183628	0.188694	3.247664	0.800837	1	DGCR6	K06262
ENSG00000131475	-0.167815	6.186337	0.800873	1	VPS25	K08867
ENSG00000106537	-0.167091	6.343	0.800896	1	TSPAN13	K17356
ENSG00000167470	-0.166129	6.231365	0.800921	1	MIDN	_
ENSG00000113269	0.17178	5.135157	0.800931	1	RNF130	K15701
ENSG00000186298	-0.165457	8.231308	0.800942	1	PPP1CC	K06269
ENSG00000198055	-0.168765	5.694141	0.800987	1	GRK6	K08291
ENSG00000003509	-0.169461	5.417915	0.801069	1	NDUFAF7	K06070
ENSG00000128272	-0.164887	9.575165	0.801117	1	ATF4	K04374
ENSG00000133422	0.169233	5.563192	0.8012	1	MORC2	_
ENSG00000197019	-0.178612	4.099694	0.801258	1	SERTAD1	K12581
ENSG00000164597	-0.169717	5.444323	0.801348	1	COG5	_
ENSG00000144655	0.17745	3.813676	0.801383	1	CSRNP1	K17494
ENSG00000133393	-0.168785	5.14075	0.801404	1	FOPNL	K16535
ENSG00000173409	-0.173439	4.905142	0.801405	1	ARV1	_
ENSG00000234797	-0.166509	6.584055	0.801452	1	RPS3AP6	K02984
ENSG00000173545	-0.165772	7.168949	0.801464	1	ZNF622	K14816
ENSG00000224138	0.239579	1.45868	0.801477	1	AC000123.4	_
ENSG00000228274	0.239579	1.45868	0.801477	1	RP3-508I15.9	_
ENSG00000197102	0.165008	7.966387	0.801552	1	DYNC1H1	K10413
ENSG00000101464	-0.171341	4.977863	0.801631	1	PIGU	K05293
ENSG00000186432	-0.16602	6.785385	0.801693	1	KPNA4	_
ENSG00000075413	0.166735	6.161035	0.801697	1	MARK3	K08798
ENSG00000103160	-0.170506	4.539162	0.801811	1	HSDL1	_
ENSG00000182827	0.165902	6.659167	0.801827	1	ACBD3	_
ENSG00000100124	-0.171926	5.048426	0.801835	1	ANKRD54	_
ENSG00000114735	-0.183285	3.016193	0.801853	1	HEMK1	K02493
ENSG00000005882	0.173992	4.670656	0.801869	1	PDK2	K00898
ENSG00000136816	-0.16782	5.498643	0.801876	1	TOR1B	K08857
ENSG00000111110	-0.167533	5.23336	0.801909	1	PPM1H	K17503
ENSG00000166483	-0.165802	6.747142	0.80201	1	WEE1	K06632
ENSG00000160633	0.165115	7.212493	0.802045	1	SAFB	K05090
ENSG00000186312	-0.173366	4.759043	0.802106	1	CA5BP1	K01672
ENSG00000173113	-0.166119	6.452136	0.802157	1	TRMT112	K15448
ENSG00000010270	-0.174991	4.284185	0.802249	1	STARD3NL	_
ENSG00000198160	-0.165198	6.325627	0.802259	1	MIER1	_
ENSG00000185728	-0.165379	6.850252	0.80235	1	YTHDF3	_
ENSG00000178307	0.16511	5.971203	0.802386	1	TMEM11	_
ENSG00000134046	0.165481	6.613728	0.802394	1	MBD2	K11590
ENSG00000164898	0.185453	2.869829	0.802502	1	C7orf55	K13212
ENSG00000173905	0.165601	6.461684	0.802508	1	GOLIM4	_
ENSG00000006715	-0.173656	4.800522	0.802511	1	VPS41	_
ENSG00000125817	0.164846	7.073423	0.802516	1	CENPB	K11496
ENSG00000115350	-0.168503	4.677451	0.802516	1	POLE4	K03506
ENSG00000125743	-0.16432	7.848457	0.802533	1	SNRPD2	K11096
ENSG00000119912	-0.165965	5.991411	0.802561	1	IDE	K01408

ENSG00000111229	-0.164179	7.846746	0.802701	1	ARPC3	K05756
ENSG00000164031	-0.16904	5.239175	0.802787	1	DNAJB14	K09520
ENSG00000126775	0.169635	4.341092	0.802799	1	ATG14	K17889
ENSG00000188185	-0.281561	1.073177	0.802807	1	LINC00265	_
ENSG00000234764	-0.281561	1.073177	0.802807	1	AC008132.12	K09390
ENSG00000273203	-0.281561	1.073177	0.802807	1	AC006946.16	_
ENSG00000171658	0.266942	1.035582	0.802807	1	RP11-443P15.2	_
ENSG00000224888	0.266942	1.035582	0.802807	1	RP5-1142A6.2	_
ENSG00000184100	-0.239944	1.049262	0.802807	1	BRD7P2	K11723
ENSG00000245317	-0.239944	1.049262	0.802807	1	CTC-241N9.1	_
ENSG00000005889	0.165252	5.707076	0.802904	1	ZFX	_
ENSG00000144567	0.165045	6.109203	0.802952	1	FAM134A	_
ENSG00000172890	0.16568	5.118841	0.802975	1	NADSYN1	K01950
ENSG00000138674	0.16413	7.443095	0.802983	1	SEC31A	K14005
ENSG00000198899	0.163082	11.09586	0.80303	1	MT-ATP6	K02126
ENSG00000213740	-0.166294	5.791508	0.803078	1	SERBP1P1	K13199
ENSG00000075391	-0.167735	4.883016	0.803255	1	RASAL2	K17633
ENSG00000132842	-0.165182	6.451638	0.803261	1	AP3B1	K12397
ENSG00000165675	-0.174253	3.908576	0.803271	1	ENOX2	_
ENSG00000130703	0.164704	5.507357	0.803336	1	OSBPL2	_
ENSG00000214160	0.164161	7.058681	0.80334	1	ALG3	K03845
ENSG00000163132	0.173598	4.63754	0.803399	1	MSX1	K09341
ENSG00000231312	-0.235177	2.123048	0.803434	1	AC007246.3	_
ENSG00000130827	-0.215855	2.112113	0.803434	1	PLXNA3	K06820
ENSG00000155592	0.208288	2.093783	0.803434	1	ZKSCAN2	K09229
ENSG00000263272	0.208288	2.093783	0.803434	1	CTC-524C5.2	_
ENSG00000197162	0.185978	2.10498	0.803434	1	ZNF785	K09228
ENSG00000198373	-0.170164	4.050198	0.80353	1	WWP2	K05630
ENSG00000070423	0.164738	6.437072	0.803567	1	RNF126	K11982
ENSG00000116199	-0.164897	6.45364	0.803591	1	FAM20B	_
ENSG00000164096	0.169927	4.920079	0.803597	1	C4orf3	_
ENSG00000179820	-0.166165	6.349467	0.803621	1	MYADM	_
ENSG00000143374	-0.166409	6.162067	0.80363	1	TARS2	K01868
ENSG00000196262	0.162599	10.5685	0.803646	1	PPIA	K03767
ENSG00000167862	-0.16784	5.385782	0.80368	1	ICT1	K15033
ENSG00000155363	0.163815	7.055054	0.803752	1	MOV10	_
ENSG00000179627	0.181383	3.023453	0.803841	1	ZBTB42	_
ENSG00000076944	-0.168132	5.41278	0.803915	1	STXBP2	K15300
ENSG00000008083	-0.166432	5.674542	0.804105	1	JARID2	K11478
ENSG00000269378	0.164372	5.717122	0.804162	1	ITGB1P1	K05719
ENSG00000105968	0.163126	7.401382	0.804205	1	H2AFV	K11251
ENSG00000204899	-0.164051	6.651874	0.804228	1	MZT1	K18633
ENSG00000248873	-0.41686	0.43029	0.804303	1	SERBP1P6	K13199
ENSG00000268402	-0.41686	0.43029	0.804303	1	AL031320.1	_
ENSG00000075043	-0.350267	0.391604	0.804303	1	KCNQ2	K04927
ENSG00000155066	-0.350267	0.391604	0.804303	1	PROM2	K15602
ENSG00000197463	-0.350267	0.391604	0.804303	1	CSAG3	_
ENSG00000254978	-0.350267	0.391604	0.804303	1	ALG1L9P	K03842
ENSG00000256591	-0.350267	0.391604	0.804303	1	RP11-286N22.8	K17571
ENSG00000100376	0.170046	4.598239	0.80433	1	FAM118A	K12581
ENSG00000177666	0.164033	6.445152	0.804383	1	PNPLA2	K16816
ENSG00000108349	0.164586	6.226381	0.804389	1	CASC3	K14323
ENSG00000184916	-0.164672	6.257953	0.804422	1	JAG2	K06052
ENSG00000061676	-0.162883	7.565807	0.804461	1	NCKAP1	K05750
ENSG00000105771	0.16749	5.098486	0.80459	1	SMG9	K18735
ENSG00000134574	-0.16657	6.006485	0.804626	1	DDB2	K10140
ENSG00000157110	0.177558	3.676276	0.804673	1	RBPMS	_
ENSG00000214174	0.195701	2.887438	0.804673	1	AMZ2P1	K06974

ENSG00000214595	-0.190729	2.897808	0.804673	1	EML6	_
ENSG00000212802	0.164179	6.244438	0.804731	1	RPL15P3	K02877
ENSG00000168116	-0.176496	4.304301	0.804754	1	KIAA1586	_
ENSG00000009307	0.161803	9.377854	0.804779	1	CSDE1	_
ENSG00000164663	-0.177657	3.845984	0.804825	1	USP49	K11834
ENSG00000136908	0.163665	6.414093	0.804871	1	DPM2	K09658
ENSG00000136143	0.163422	6.574655	0.804882	1	SUCLA2	K01900
ENSG00000176896	0.217203	1.835765	0.804884	1	TCEANC	K11341
ENSG00000261788	0.217203	1.835765	0.804884	1	RP11-480G7.1	_
ENSG00000119681	-0.190863	1.855902	0.804884	1	LTBP2	K08023
ENSG00000168395	-0.173067	3.99357	0.804947	1	ING5	K11345
ENSG00000162298	0.16745	5.312395	0.804992	1	SYVN1	K10601
ENSG00000112210	-0.1693	4.127455	0.805046	1	RAB23	K06234
ENSG00000169641	0.165368	5.521758	0.805131	1	LUZP1	_
ENSG00000169756	-0.163054	5.661616	0.805214	1	LIMS1	K17985
ENSG00000173442	0.161837	8.038754	0.805254	1	EHBP1L1	_
ENSG00000185418	0.173447	4.56152	0.80531	1	TARSL2	K01868
ENSG00000184076	0.164193	6.334752	0.805416	1	UQCR10	K00419
ENSG00000125249	-0.167071	4.823895	0.805431	1	RAP2A	K07837
ENSG00000180581	0.197947	2.34763	0.805445	1	SRP9P1	K03109
ENSG00000229180	0.197947	2.34763	0.805445	1	GS1-124K5.11	_
ENSG00000136754	0.166625	5.571521	0.805504	1	ABI1	_
ENSG00000105928	-0.162993	6.63434	0.805505	1	DFNA5	K18047
ENSG00000143106	-0.161524	8.161112	0.805628	1	PSMA5	K02729
ENSG00000143303	-0.171898	4.701247	0.805689	1	RRNAD1	_
ENSG00000150938	-0.161699	7.766316	0.80569	1	CRIM1	_
ENSG00000074657	0.167693	5.007273	0.805757	1	ZNF532	_
ENSG00000120256	0.162057	7.105636	0.805768	1	LRP11	_
ENSG00000229212	0.190637	3.039319	0.805791	1	RP11-561C5.4	_
ENSG00000188636	-0.164772	5.710137	0.805863	1	LDOC1L	_
ENSG00000122386	0.16641	4.469477	0.805991	1	ZNF205	K09228
ENSG00000184402	0.170652	4.742928	0.806002	1	SS18L1	_
ENSG00000145911	-0.163855	5.175111	0.806012	1	N4BP3	_
ENSG00000178449	0.163562	6.224619	0.806022	1	COX14	K18181
ENSG00000164346	-0.161966	7.088821	0.80605	1	NSA2	K14842
ENSG00000160867	-0.164893	5.834383	0.806163	1	FGFR4	K05095
ENSG00000166450	-0.166654	4.869583	0.806283	1	PRTG	_
ENSG00000123124	0.162666	6.327115	0.806327	1	WWP1	K05633
ENSG00000240857	-0.172744	4.524016	0.806346	1	RDH14	K11162
ENSG00000108506	-0.172011	4.073834	0.806447	1	INTS2	K13139
ENSG00000111912	0.162038	6.611922	0.806453	1	NCOA7	K17985
ENSG00000224470	0.165631	5.175802	0.806461	1	ATXN1L	_
ENSG00000062725	-0.1671	4.908044	0.806547	1	APPBP2	_
ENSG00000100038	0.165372	4.905524	0.806547	1	TOP3B	K03165
ENSG00000141627	0.165372	4.905524	0.806547	1	DYM	_
ENSG00000169221	0.163627	5.380354	0.806601	1	TBC1D10B	K11447
ENSG00000126243	0.181821	3.401013	0.806635	1	LRFN3	_
ENSG00000205707	0.164337	4.662062	0.806638	1	LYRM5	_
ENSG00000147854	0.163156	6.229305	0.806674	1	UHRF2	K15713
ENSG00000177888	-0.170249	4.375664	0.806767	1	ZBTB41	K10513
ENSG00000101294	0.161191	7.115794	0.806779	1	HM13	K09595
ENSG00000089123	-0.175899	2.914968	0.806798	1	TASP1	K08657
ENSG00000118263	-0.25296	1.53378	0.806915	1	KLF7	K09207
ENSG00000170469	0.230664	1.521426	0.806915	1	SPATA24	_
ENSG00000151929	0.161816	6.482436	0.806929	1	BAG3	K09557
ENSG00000109586	-0.161659	6.723715	0.806929	1	GALNT7	K00710
ENSG00000188603	-0.176578	4.007301	0.806964	1	CLN3	K12389
ENSG00000163902	0.159986	9.132891	0.80699	1	RPN1	K12666

ENSG00000171700	0.168997	4.527698	0.807046	1	RGS19	K16449
ENSG00000175324	-0.163528	4.912406	0.807086	1	LSM1	K12620
ENSG00000100350	-0.165302	4.836215	0.807141	1	FOXRED2	_
ENSG00000141646	-0.165911	5.538789	0.807244	1	SMAD4	K04501
ENSG00000031003	0.16672	5.018151	0.80726	1	FAM13B	_
ENSG00000120029	0.16672	5.018151	0.80726	1	C10orf76	_
ENSG00000198793	-0.163046	5.698371	0.807274	1	MTOR	K07203
ENSG00000214941	-0.171334	3.518807	0.807302	1	ZSWIM7	K18169
ENSG00000090905	-0.168598	4.877066	0.807388	1	TNRC6A	K18412
ENSG00000171735	-0.165436	5.056335	0.807475	1	CAMTA1	_
ENSG00000134453	-0.160797	7.025989	0.807509	1	RBM17	K12840
ENSG00000124496	-0.169123	3.943124	0.80753	1	TRERF1	_
ENSG00000196323	-0.167126	4.987554	0.807544	1	ZBTB44	K10515
ENSG00000101084	0.160918	6.726595	0.8076	1	C20orf24	_
ENSG00000100554	0.162166	6.113006	0.807671	1	ATP6V1D	K02149
ENSG00000158941	-0.162075	5.959379	0.807725	1	CCAR2	_
ENSG00000146842	-0.165677	5.566796	0.807736	1	TMEM209	K09654
ENSG00000136997	-0.159455	8.834589	0.807757	1	MYC	K04377
ENSG00000133121	-0.199609	2.768854	0.80801	1	STARD13	_
ENSG00000151689	-0.199609	2.768854	0.80801	1	INPP1	K01107
ENSG00000121022	-0.160083	7.235044	0.8081	1	COPS5	K09613
ENSG00000198898	0.159754	7.476726	0.80812	1	CAPZA2	K10364
ENSG00000175782	0.180172	3.417561	0.808126	1	SLC35E3	K15285
ENSG00000112893	-0.162735	5.944512	0.80814	1	MAN2A1	K01231
ENSG00000162441	-0.173999	3.870121	0.808151	1	LZIC	_
ENSG00000101213	-0.16543	4.91967	0.808153	1	PTK6	K08894
ENSG00000168067	0.165015	4.485247	0.808157	1	MAP4K2	K04414
ENSG00000068793	0.159927	7.203564	0.808177	1	CYFIP1	K05749
ENSG00000167196	-0.162253	6.084122	0.808186	1	FBXO22	K10302
ENSG00000163516	0.16402	5.470218	0.808218	1	ANKZF1	_
ENSG00000172572	0.160777	6.257109	0.808263	1	PDE3A	K19021
ENSG00000147130	-0.160101	5.890134	0.808358	1	ZMYM3	K06641
ENSG00000231770	-0.204642	2.388133	0.808448	1	TMEM44-AS1	_
ENSG00000163795	0.194137	2.381805	0.808448	1	ZNF513	K09228
ENSG00000171444	0.194137	2.381805	0.808448	1	MCC	_
ENSG00000163754	-0.160856	5.312681	0.808472	1	GYG1	K00750
ENSG00000183751	-0.160663	6.440595	0.808603	1	TBL3	K14555
ENSG00000260336	0.163057	5.548261	0.808627	1	RP11-395B7.7	_
ENSG00000125107	0.159333	7.378071	0.808706	1	CNOT1	K12604
ENSG00000143727	0.159675	6.984352	0.808722	1	ACP1	K14394
ENSG00000128590	0.17043	4.332399	0.808792	1	DNAJB9	K09515
ENSG00000102908	-0.165252	4.49511	0.808868	1	NFAT5	K17335
ENSG00000170464	0.180645	2.91717	0.808878	1	DNAJC18	K09538
ENSG00000138821	-0.160147	6.30639	0.808911	1	SLC39A8	K14714
ENSG00000272405	-0.163941	5.06683	0.808918	1	RP11-284F21.10	_
ENSG00000143382	-0.159085	7.500775	0.809007	1	ADAMTSL4	_
ENSG00000155463	0.159262	7.100835	0.809074	1	OXA1L	K03217
ENSG00000068394	0.237499	1.871083	0.809098	1	GPKOW	K13101
ENSG00000267855	0.237499	1.871083	0.809098	1	NDUFA7	K03951
ENSG00000123643	0.211109	1.884288	0.809098	1	SLC36A1	K14209
ENSG00000205609	0.166135	4.680996	0.809154	1	EIF3CL	K03252
ENSG00000126883	0.163466	5.668764	0.80922	1	NUP214	K14317
ENSG00000010292	0.158521	7.807335	0.809322	1	NCAPD2	K06677
ENSG00000163590	0.162022	4.728139	0.809365	1	PPM1L	K17506
ENSG00000198176	-0.158635	7.68007	0.809381	1	TFDP1	K04683
ENSG00000151366	-0.164289	4.968076	0.809387	1	NDUFC2	K03968
ENSG00000149136	-0.158045	8.815173	0.809432	1	SSRP1	K09272
ENSG00000117408	-0.16606	5.106809	0.809552	1	IPO13	_

ENSG00000196812	0.177175	3.066073	0.809577	1	ZSCAN16	K09230
ENSG00000185753	0.173667	3.71694	0.809596	1	CXorf38	K17985
ENSG00000119718	0.16244	5.319011	0.809678	1	EIF2B2	K03754
ENSG00000132507	-0.157647	9.23547	0.809767	1	EIF5A	K03263
ENSG00000011523	-0.163337	4.689366	0.809773	1	CEP68	K16764
ENSG00000134153	0.159016	6.758961	0.809797	1	EMC7	_
ENSG00000104783	0.160525	5.992031	0.809862	1	KCNN4	K04945
ENSG00000123066	0.160525	5.992031	0.809862	1	MED13L	K15164
ENSG00000172269	-0.160028	5.606917	0.809865	1	DPAGT1	K01001
ENSG00000087365	0.157798	8.321404	0.80988	1	SF3B2	K12829
ENSG00000134107	-0.159052	6.736522	0.809984	1	BHLHE40	K03729
ENSG00000244187	-0.16143	5.008113	0.810052	1	TMEM141	_
ENSG00000213347	0.164327	4.447534	0.81006	1	MXD3	K07912
ENSG00000115170	-0.174336	3.208532	0.810152	1	ACVR1	K04675
ENSG00000178585	-0.174336	3.208532	0.810152	1	CTNNBIP1	K04493
ENSG00000066322	-0.158867	6.331643	0.810382	1	ELOVL1	K10247
ENSG00000104936	-0.160283	6.1349	0.810436	1	DMPK	K08788
ENSG00000168566	-0.158946	6.538136	0.810445	1	SNRNP48	K13156
ENSG00000130045	0.220198	2.16337	0.810446	1	NXNL2	K17609
ENSG00000198888	0.156802	10.52253	0.810517	1	MT-ND1	K03878
ENSG00000183779	0.164324	4.400518	0.81058	1	ZNF703	_
ENSG00000175854	-0.162292	5.27016	0.810651	1	SWI5	_
ENSG00000006114	-0.160998	4.605424	0.810661	1	SYNRG	_
ENSG00000138698	-0.163601	4.781486	0.810723	1	RAP1GDS1	_
ENSG00000088986	-0.157384	7.848875	0.81073	1	DYNLL1	K10418
ENSG00000140743	0.160546	5.907548	0.810773	1	CDR2	_
ENSG00000163938	-0.157482	7.637573	0.81078	1	GNL3	K14538
ENSG00000049167	-0.162636	3.728336	0.810789	1	ERCC8	K10570
ENSG00000132341	-0.156601	9.718025	0.810892	1	RAN	K07936
ENSG00000172009	-0.157472	7.307945	0.811106	1	THOP1	K01392
ENSG00000173207	-0.157298	7.433928	0.811183	1	CKS1B	K02219
ENSG00000021776	-0.16159	5.444321	0.811257	1	AQR	K12874
ENSG00000117133	-0.15819	6.577675	0.811267	1	RPF1	K14846
ENSG00000214367	-0.162702	4.787843	0.811297	1	HAUS3	K16586
ENSG00000106462	0.158103	6.455672	0.811354	1	EZH2	K11430
ENSG00000247228	0.190505	2.415185	0.81136	1	RP11-296I10.3	_
ENSG00000143578	-0.170463	3.893862	0.811366	1	CREB3L4	K09048
ENSG00000137575	-0.156793	7.910829	0.811386	1	SDCBP	K17254
ENSG00000176401	0.186204	3.081486	0.811415	1	EID2B	K17985
ENSG00000185480	-0.159284	5.896035	0.811504	1	PARPBP	_
ENSG00000165119	-0.156149	9.084877	0.811587	1	HNRNPK	K12886
ENSG00000163820	0.163311	3.967613	0.81161	1	FYCO1	_
ENSG00000107821	-0.160065	5.219302	0.811695	1	KAZALD1	_
ENSG00000140548	0.166744	3.641704	0.811699	1	ZNF710	_
ENSG00000132467	-0.159382	5.81817	0.811803	1	UTP3	K14767
ENSG00000226419	-0.171506	3.227323	0.811828	1	SLC16A1-AS1	_
ENSG00000260054	-0.165166	4.047498	0.81183	1	RP11-611L7.1	_
ENSG00000010318	-0.211899	1.576439	0.812063	1	PHF7	_
ENSG00000092445	0.158943	5.818585	0.812083	1	TYRO3	K05116
ENSG00000119408	0.160665	5.056916	0.812111	1	NEK6	K08857
ENSG00000181827	-0.160491	4.709594	0.812209	1	RFX7	_
ENSG00000077097	-0.156597	7.22191	0.812235	1	TOP2B	K03164
ENSG00000141873	-0.158604	5.800981	0.812309	1	SLC39A3	K14709
ENSG00000160113	-0.155957	7.977555	0.81233	1	NR2F6	K08549
ENSG00000117479	0.158571	5.312403	0.81236	1	SLC19A2	K14610
ENSG00000137076	0.156292	7.30999	0.812368	1	TLN1	K06271
ENSG00000255737	0.168483	3.454256	0.812467	1	AGAP2-AS1	K17848
ENSG00000198832	0.157166	6.442609	0.812483	1	SELM	_

ENSG00000250182	0.193308	2.795758	0.812517	1	EEF1A1P13	K03231
ENSG00000241945	-0.157031	6.545199	0.812692	1	PWP2	K14558
ENSG00000184939	-0.179724	2.966473	0.812907	1	ZFP90	K09228
ENSG00000119711	-0.164565	4.1249	0.812929	1	ALDH6A1	K00140
ENSG00000165055	-0.158765	5.228681	0.81296	1	METTL2B	K17985
ENSG00000100601	0.165157	4.18583	0.813069	1	ALKBH1	K10765
ENSG00000101104	-0.161553	4.840797	0.813086	1	PABPC1L	K13126
ENSG00000170631	0.171192	3.743425	0.81313	1	ZNF16	K09228
ENSG00000139168	-0.156427	6.662756	0.813203	1	ZCRB1	K13154
ENSG00000061936	0.157992	5.848597	0.813237	1	SFSWAP	_
ENSG00000162869	-0.189189	3.237024	0.813475	1	PPP1R21	K17562
ENSG00000260539	0.162178	4.425254	0.813524	1	RP11-252A24.7	_
ENSG00000065243	-0.155516	7.203598	0.813533	1	PKN2	K06071
ENSG00000160075	-0.156085	6.658859	0.813613	1	SSU72	K15544
ENSG00000135775	-0.157981	5.380527	0.81364	1	COG2	_
ENSG00000177352	0.164997	4.529623	0.813704	1	CCDC71	_
ENSG00000115415	-0.156379	6.412073	0.813712	1	STAT1	K11220
ENSG00000126934	0.15481	7.719047	0.813778	1	MAP2K2	K04369
ENSG00000106682	-0.170892	3.4682	0.81387	1	EIF4H	_
ENSG00000163166	0.155007	7.305274	0.813892	1	IWS1	K17498
ENSG00000126787	-0.155647	6.808677	0.813895	1	DLGAP5	K16804
ENSG00000136877	-0.156288	6.301964	0.813995	1	FPGS	K01930
ENSG00000205726	0.157211	6.095772	0.814133	1	ITSN1	_
ENSG00000120253	-0.158709	5.493789	0.814163	1	NUP43	K14305
ENSG00000048028	0.158009	5.517931	0.814197	1	USP28	K11849
ENSG00000100767	-0.160484	4.891876	0.814223	1	PAPLN	K12581
ENSG00000167553	-0.153862	9.326223	0.81423	1	TUBA1C	K07374
ENSG00000005206	0.157749	5.795063	0.814244	1	SPPL2B	_
ENSG00000141380	-0.155445	6.707896	0.814289	1	SS18	K15623
ENSG00000168159	-0.155843	6.400709	0.814367	1	RNF187	K15709
ENSG00000159352	-0.154393	7.686063	0.814398	1	PSMD4	K03029
ENSG00000170881	-0.162468	5.077249	0.814446	1	RNF139	K15703
ENSG00000173418	-0.157856	5.840524	0.814569	1	NAA20	K17972
ENSG00000108256	0.153774	8.479992	0.814576	1	NUFIP2	K06115
ENSG00000170100	-0.191143	2.819209	0.814693	1	ZNF778	K09228
ENSG00000119487	0.156341	5.975378	0.814704	1	MAPKAP1	_
ENSG00000088766	0.154866	5.689316	0.814706	1	CRLS1	K08744
ENSG00000136485	0.155193	6.491223	0.814728	1	DCAF7	K11805
ENSG00000137266	0.158698	4.261049	0.81482	1	SLC22A23	K08215
ENSG00000198646	0.156064	5.470219	0.814833	1	NCOA6	_
ENSG00000145107	-0.312214	0.518168	0.814851	1	TM4SF19	_
ENSG00000224817	-0.312214	0.518168	0.814851	1	RP11-190J1.3	_
ENSG00000251144	-0.312214	0.518168	0.814851	1	CTD-2532K18.2	_
ENSG00000161664	0.248359	0.472995	0.814851	1	ASB16	K10338
ENSG00000168256	0.248359	0.472995	0.814851	1	NKIRAS2	K17197
ENSG00000187990	0.248359	0.472995	0.814851	1	HIST1H2BG	K11252
ENSG00000197021	0.248359	0.472995	0.814851	1	CXorf40B	_
ENSG00000233225	0.248359	0.472995	0.814851	1	AC004987.9	_
ENSG00000188677	-0.158173	5.497674	0.814855	1	PARVB	K06275
ENSG00000143434	0.176144	2.963001	0.814858	1	SEMA6C	K06842
ENSG00000028839	-0.165061	4.381947	0.814919	1	TBPL1	K03120
ENSG00000113552	-0.157023	6.016016	0.814979	1	GNPDA1	K02564
ENSG00000136003	-0.155282	6.404854	0.815022	1	ISCU	_
ENSG00000138600	0.158034	5.446261	0.815166	1	SPPL2A	K09596
ENSG00000075218	0.15593	5.447268	0.815166	1	GTSE1	K10129
ENSG00000100304	-0.15407	7.257872	0.815185	1	TTLL12	K16609
ENSG00000205352	-0.153545	7.739337	0.815359	1	PRR13	_
ENSG00000185236	-0.154786	6.091227	0.815489	1	RAB11B	K07905

ENSG00000165355	-0.165375	3.928761	0.815491	1	FBXO33	K10310
ENSG00000139437	-0.167143	4.210275	0.8156	1	TCHP	K16811
ENSG00000137185	-0.162011	4.59821	0.815716	1	ZSCAN9	K09230
ENSG00000147669	-0.154067	6.837709	0.81572	1	POLR2K	K03009
ENSG00000156502	-0.154755	6.361808	0.81573	1	SUPV3L1	K17675
ENSG00000115020	-0.157753	5.253019	0.815847	1	PIKFYVE	K00921
ENSG00000160877	-0.153202	7.387591	0.816077	1	NACC1	K10486
ENSG00000054267	0.154772	5.534122	0.81622	1	ARID4B	K19195
ENSG00000116584	-0.154156	6.458666	0.816247	1	ARHGEF2	K12791
ENSG00000106153	-0.152109	9.14342	0.816361	1	CHCHD2	K00901
ENSG00000165424	0.162655	4.214499	0.816428	1	ZCCHC24	_
ENSG00000170275	0.152231	8.406999	0.816439	1	CRTAP	K19606
ENSG00000124570	0.152044	8.611807	0.816575	1	SERPINB6	K13963
ENSG00000196876	-0.262382	1.153501	0.816583	1	SCN8A	_
ENSG00000214900	-0.262382	1.153501	0.816583	1	C14orf182	K02927
ENSG00000137709	0.253341	1.119146	0.816583	1	POU2F3	K09364
ENSG00000150527	0.253341	1.119146	0.816583	1	CTAGE5	_
ENSG00000155666	0.253341	1.119146	0.816583	1	KDM8	K10277
ENSG00000173540	0.253341	1.119146	0.816583	1	GMPPB	K00966
ENSG00000250903	0.253341	1.119146	0.816583	1	GMDS-AS1	_
ENSG00000255031	0.253341	1.119146	0.816583	1	RP11-802E16.3	_
ENSG00000180611	-0.175979	3.487959	0.816616	1	MB21D2	_
ENSG00000001630	-0.166064	3.264189	0.816685	1	CYP51A1	K05917
ENSG00000161800	0.152505	7.493348	0.816686	1	RACGAP1	K16733
ENSG00000146729	-0.153747	5.936678	0.816689	1	GBAS	_
ENSG00000162231	0.154203	5.876499	0.816715	1	NXF1	K14284
ENSG00000113108	-0.176286	2.988658	0.81677	1	APBB3	_
ENSG00000233927	-0.176286	2.988658	0.81677	1	RPS28	K02979
ENSG00000070770	0.152662	6.167731	0.816812	1	CSNK2A2	K03097
ENSG00000184068	0.177321	2.827443	0.816819	1	RP5-821D11.7	_
ENSG00000071127	0.152519	7.236499	0.816907	1	WDR1	_
ENSG00000174130	0.18704	2.447807	0.816928	1	TLR6	K10169
ENSG00000118058	0.222509	1.581542	0.816944	1	KMT2A	K09186
ENSG00000236255	0.222509	1.581542	0.816944	1	AC009404.2	_
ENSG00000219529	0.19021	1.597888	0.816944	1	AP000580.1	K02969
ENSG00000198182	-0.195154	2.22831	0.816978	1	ZNF607	K09228
ENSG00000213839	-0.195154	2.22831	0.816978	1	RP11-3J10.4	_
ENSG00000159674	0.174139	2.222759	0.816978	1	SPON2	_
ENSG00000228797	0.205418	1.931223	0.817004	1	FAM207BP	_
ENSG00000169252	-0.197627	1.96151	0.817004	1	ADRB2	K04142
ENSG00000183161	-0.197627	1.96151	0.817004	1	FANCF	K10893
ENSG00000160233	0.1804	1.943844	0.817004	1	LRRC3	K17985
ENSG00000186907	0.152626	6.994339	0.817046	1	RTN4RL2	K16661
ENSG00000133961	0.155612	5.615149	0.817158	1	NUMB	K06057
ENSG00000102854	-0.151613	8.253814	0.817313	1	MSLN	_
ENSG00000101266	0.15279	6.648659	0.817318	1	CSNK2A1	K03097
ENSG00000240972	-0.151983	7.477147	0.817435	1	MIF	K07253
ENSG00000104763	-0.153547	5.883478	0.817499	1	ASAH1	K12348
ENSG00000143740	0.156007	5.518889	0.817571	1	SNAP47	K05062
ENSG00000100804	-0.151765	7.554951	0.817623	1	PSMB5	K02737
ENSG00000113013	-0.150797	10.17059	0.817702	1	HSPA9	K04043
ENSG00000243199	0.159428	4.457588	0.817775	1	RP11-408P14.1	K02934
ENSG00000128228	0.153709	5.844777	0.817807	1	SDF2L1	_
ENSG00000172354	0.151462	7.537241	0.817886	1	GNB2	K04537
ENSG00000140829	0.15432	5.73782	0.81794	1	DHX38	K12815
ENSG00000184661	-0.154003	5.470255	0.817972	1	CDCA2	K17590
ENSG00000111639	-0.151617	7.359587	0.817984	1	MRPL51	K17432
ENSG00000198783	-0.157211	4.757356	0.818037	1	ZNF830	K13104

ENSG00000243927	-0.154152	4.755722	0.818037	1	MRPS6	K02990
ENSG00000078369	0.151587	7.187958	0.81806	1	GNB1	K04536
ENSG00000134463	-0.16498	3.394962	0.818094	1	ECHDC3	_
ENSG00000145555	0.151147	7.739092	0.818105	1	MYO10	K12559
ENSG00000137656	-0.163591	4.16698	0.818151	1	BUD13	K13106
ENSG00000187118	-0.158976	4.164495	0.818151	1	CMC1	K18171
ENSG00000083812	0.177068	3.270812	0.818249	1	ZNF324	K09228
ENSG00000131013	-0.158519	4.88447	0.818393	1	PPIL4	K12735
ENSG00000100714	-0.151392	7.21664	0.818402	1	MTHFD1	K00288
ENSG00000228794	-0.183307	3.150657	0.81843	1	LINC01128	_
ENSG00000176209	0.169463	3.1477	0.81843	1	SMIM19	_
ENSG00000241852	0.169463	3.1477	0.81843	1	C8orf58	_
ENSG00000247498	0.169463	3.1477	0.81843	1	RP11-392P7.6	_
ENSG00000130733	0.152729	5.950039	0.818437	1	YIPF2	_
ENSG00000154102	-0.167468	3.954459	0.818469	1	C16orf74	_
ENSG00000126524	-0.15126	7.185969	0.818593	1	SBDS	K14574
ENSG00000170175	-0.172956	3.01051	0.818643	1	CHRNB1	K04812
ENSG00000004779	-0.15171	6.691467	0.818733	1	NDUFAB1	K03955
ENSG00000152377	-0.15176	6.645367	0.818749	1	SPOCK1	K08136
ENSG00000011638	-0.163939	4.297532	0.818756	1	TMEM159	_
ENSG00000143774	0.150627	7.636075	0.818798	1	GUK1	K00942
ENSG00000111596	-0.15375	6.117978	0.818848	1	CNOT2	K12605
ENSG00000110931	-0.152236	5.555662	0.81885	1	CAMKK2	K07359
ENSG00000170191	0.161041	4.2333	0.81887	1	NANP	K01097
ENSG00000237036	0.187981	2.845561	0.818898	1	ZEB1-AS1	_
ENSG00000234545	-0.166465	3.701685	0.818899	1	FAM133B	_
ENSG00000167182	0.161893	3.69695	0.818899	1	SP2	K09192
ENSG00000113328	-0.150959	7.197362	0.818936	1	CCNG1	K10145
ENSG00000189057	0.155277	4.847293	0.818987	1	FAM111B	_
ENSG00000077254	0.150509	7.4163	0.819119	1	USP33	K11848
ENSG00000111711	-0.151023	6.960332	0.819153	1	GOLT1B	_
ENSG00000129518	0.152787	5.214158	0.819169	1	EAPP	_
ENSG00000049541	0.193533	2.6705	0.819207	1	RFC2	K10755
ENSG00000128713	-0.187592	2.675438	0.819207	1	HOXD11	K09296
ENSG00000169629	0.178762	2.677829	0.819207	1	RGPD8	K12172
ENSG00000113615	0.151213	5.506391	0.819285	1	SEC24A	K14007
ENSG00000166454	-0.152826	5.99625	0.819336	1	ATMIN	_
ENSG00000170775	-0.159462	4.36073	0.819389	1	GPR37	K04243
ENSG00000185838	0.15835	3.95923	0.81944	1	GNB1L	_
ENSG00000140374	0.150102	7.346554	0.819665	1	ETFA	K03522
ENSG00000117054	0.149831	7.685729	0.819706	1	ACADM	K00249
ENSG00000234851	0.150138	7.252118	0.819713	1	RP11-3P17.3	K02893
ENSG00000133884	0.151216	6.347167	0.819771	1	DPF2	K13196
ENSG00000187630	-0.172001	3.286947	0.819787	1	DHRS4L2	K11147
ENSG00000152767	0.152801	5.797427	0.819815	1	FARP1	_
ENSG00000128694	-0.162212	4.176694	0.819829	1	OSGEPL1	K01409
ENSG00000134419	0.149045	9.34724	0.819913	1	RPS15A	K02957
ENSG00000111412	0.159535	4.677601	0.819946	1	C12orf49	_
ENSG00000137274	-0.161229	4.112252	0.820002	1	BPHL	K18399
ENSG00000134287	-0.149924	7.314603	0.820036	1	ARF3	K07938
ENSG00000184164	0.152266	5.588945	0.820069	1	CRELD2	K16467
ENSG00000197170	-0.150346	6.871427	0.820077	1	PSMD12	K03035
ENSG00000059915	-0.171317	2.255213	0.820079	1	PSD	K12494
ENSG00000122705	-0.149422	7.97411	0.820081	1	CLTA	K04644
ENSG00000099904	-0.153729	5.159238	0.820162	1	ZDHHC8	K18932
ENSG00000168792	-0.155118	4.042083	0.820192	1	ABHD15	K13707
ENSG00000112312	-0.149837	7.19677	0.820268	1	GMNN	K10749
ENSG00000166888	-0.15007	6.956812	0.820287	1	STAT6	K11225

ENSG00000132694	0.155387	4.244966	0.820463	1	ARHGEF11	K12331
ENSG00000115541	-0.14927	7.652939	0.820498	1	HSPE1	K04078
ENSG00000117118	-0.149673	7.108381	0.820565	1	SDHB	K00235
ENSG00000167522	0.149506	7.127402	0.820592	1	ANKRD11	_
ENSG00000135336	-0.15267	5.465303	0.820779	1	ORC3	K02605
ENSG00000132676	-0.149128	7.491357	0.820808	1	DAP3	K17408
ENSG00000100227	0.14976	6.709461	0.820815	1	POLDIP3	_
ENSG00000148187	-0.154912	5.32366	0.820881	1	MRRF	K02838
ENSG00000144747	0.15097	5.717109	0.820912	1	TMF1	_
ENSG00000100028	-0.148677	8.00105	0.820947	1	SNRPD3	K11088
ENSG00000103496	-0.15223	5.293804	0.82098	1	STX4	K13502
ENSG00000171574	-0.161432	4.315235	0.82104	1	ZNF584	_
ENSG00000114902	0.149458	6.777445	0.821077	1	SPCS1	K12946
ENSG00000135679	0.149966	6.390703	0.821085	1	MDM2	K06643
ENSG00000034510	-0.147867	10.16905	0.821184	1	TMSB10	K13785
ENSG00000126878	-0.155928	5.23218	0.821192	1	AIF1L	K18617
ENSG00000075785	0.148315	8.117592	0.821231	1	RAB7A	K07897
ENSG00000198721	-0.148616	7.45735	0.821447	1	ECI2	K13239
ENSG00000139675	-0.155177	4.59455	0.821453	1	HNRNPA1L2	K12741
ENSG00000188002	0.155039	4.190735	0.821475	1	RP11-43F13.1	_
ENSG00000103510	0.152567	5.024942	0.821496	1	KAT8	K11308
ENSG00000155744	0.168017	3.627555	0.821505	1	FAM126B	_
ENSG00000159685	-0.161094	4.379815	0.821567	1	CHCHD6	K17564
ENSG00000134575	-0.15261	5.700067	0.821568	1	ACP2	K14410
ENSG00000165914	-0.228829	1.649252	0.821578	1	TTC7B	_
ENSG00000254285	-0.228829	1.649252	0.821578	1	KRT8P3	K07605
ENSG00000228801	0.21502	1.63924	0.821578	1	RP11-110G21.1	_
ENSG00000196576	0.147787	8.344484	0.821744	1	PLXNB2	_
ENSG00000185043	0.148441	7.197544	0.821781	1	CIB1	K17259
ENSG00000129625	-0.148407	7.342962	0.821807	1	REEP5	K17279
ENSG00000141933	0.161586	3.532283	0.821874	1	TPGS1	K16581
ENSG00000131165	0.149068	6.507212	0.821947	1	CHMP1A	K12197
ENSG00000089685	-0.148155	7.476181	0.821976	1	BIRC5	K08731
ENSG00000162614	-0.156974	4.439584	0.822051	1	NEXN	_
ENSG00000125648	-0.149207	6.458183	0.822101	1	SLC25A23	K14684
ENSG00000112406	0.150463	4.789772	0.822447	1	HECA	K12581
ENSG00000018280	-0.68332	-0.566483	0.822492	1	SLC11A1	K12347
ENSG00000100399	-0.68332	-0.566483	0.822492	1	CHADL	_
ENSG00000117586	-0.68332	-0.566483	0.822492	1	TNFSF4	K05469
ENSG00000123009	-0.68332	-0.566483	0.822492	1	NME2P1	K00940
ENSG00000124116	-0.68332	-0.566483	0.822492	1	WFDC3	K08857
ENSG00000136114	-0.68332	-0.566483	0.822492	1	THSD1	_
ENSG00000137877	-0.68332	-0.566483	0.822492	1	SPTBN5	K06115
ENSG00000152705	-0.68332	-0.566483	0.822492	1	CATSPER3	K16891
ENSG00000166596	-0.68332	-0.566483	0.822492	1	WDR16	_
ENSG00000171160	-0.68332	-0.566483	0.822492	1	MORN4	K12581
ENSG00000176076	-0.68332	-0.566483	0.822492	1	KCNE1L	K04895
ENSG00000185860	-0.68332	-0.566483	0.822492	1	C1orf110	_
ENSG00000188766	-0.68332	-0.566483	0.822492	1	SPRED3	K04703
ENSG00000203301	-0.68332	-0.566483	0.822492	1	AL590822.1	K17985
ENSG00000225205	-0.68332	-0.566483	0.822492	1	AC093818.1	_
ENSG00000226124	-0.68332	-0.566483	0.822492	1	FTCDNL1	_
ENSG00000232671	-0.68332	-0.566483	0.822492	1	RP11-126K1.2	_
ENSG00000233110	-0.68332	-0.566483	0.822492	1	RP11-301L8.2	_
ENSG00000237015	-0.68332	-0.566483	0.822492	1	CTA-984G1.5	_
ENSG00000238465	-0.68332	-0.566483	0.822492	1	snoU13	_
ENSG00000241978	-0.68332	-0.566483	0.822492	1	AKAP2	K16519
ENSG00000247627	-0.68332	-0.566483	0.822492	1	MTND4P12	K03881

ENSG00000254369	-0.68332	-0.566483	0.822492	1	HOXA-AS3	_
ENSG00000257681	-0.68332	-0.566483	0.822492	1	RP11-341G23.4	_
ENSG00000259514	-0.68332	-0.566483	0.822492	1	RP11-685G9.2	_
ENSG00000259768	-0.68332	-0.566483	0.822492	1	RP5-991G20.1	_
ENSG00000260018	-0.68332	-0.566483	0.822492	1	RP11-505K9.1	_
ENSG00000260400	-0.68332	-0.566483	0.822492	1	RP11-119F7.5	_
ENSG00000263823	-0.68332	-0.566483	0.822492	1	RP11-326K13.4	_
ENSG00000266708	-0.68332	-0.566483	0.822492	1	RP11-661O13.1	_
ENSG00000267547	-0.68332	-0.566483	0.822492	1	RP11-686D22.4	_
ENSG00000269427	-0.68332	-0.566483	0.822492	1	CTC-429P9.1	_
ENSG00000270091	-0.68332	-0.566483	0.822492	1	RP11-78O7.2	_
ENSG00000270130	-0.68332	-0.566483	0.822492	1	RP11-214K3.23	_
ENSG00000081985	0.542622	-0.611472	0.822492	1	IL12RB2	K05064
ENSG00000084628	0.542622	-0.611472	0.822492	1	NKAIN1	_
ENSG00000133392	0.542622	-0.611472	0.822492	1	MYH11	K10352
ENSG00000133943	0.542622	-0.611472	0.822492	1	C14orf159	_
ENSG00000142959	0.542622	-0.611472	0.822492	1	BEST4	K13881
ENSG00000161381	0.542622	-0.611472	0.822492	1	PLXDC1	K09228
ENSG00000173275	0.542622	-0.611472	0.822492	1	ZNF449	K09230
ENSG00000178201	0.542622	-0.611472	0.822492	1	VN1R1	K04614
ENSG00000185275	0.542622	-0.611472	0.822492	1	CD24P4	K06469
ENSG00000215022	0.542622	-0.611472	0.822492	1	RP1-257A7.4	_
ENSG00000215210	0.542622	-0.611472	0.822492	1	RBMXP2	K12885
ENSG00000234322	0.542622	-0.611472	0.822492	1	ST13P18	K09560
ENSG00000234616	0.542622	-0.611472	0.822492	1	JRK	_
ENSG00000234814	0.542622	-0.611472	0.822492	1	SVILP1	K10369
ENSG00000236233	0.542622	-0.611472	0.822492	1	RP11-12A20.7	_
ENSG00000236947	0.542622	-0.611472	0.822492	1	RP11-98G7.1	_
ENSG00000237595	0.542622	-0.611472	0.822492	1	RP11-112L6.3	_
ENSG00000237923	0.542622	-0.611472	0.822492	1	XXbac-BPG27H4.8	_
ENSG00000241549	0.542622	-0.611472	0.822492	1	GUSBP2	K01195
ENSG00000243759	0.542622	-0.611472	0.822492	1	ST13P15	K09560
ENSG00000245680	0.542622	-0.611472	0.822492	1	ZNF585B	K09228
ENSG00000258741	0.542622	-0.611472	0.822492	1	RP11-386M24.4	K11251
ENSG00000261604	0.542622	-0.611472	0.822492	1	CTD-2636A23.2	_
ENSG00000261614	0.542622	-0.611472	0.822492	1	YBX3P1	K06099
ENSG00000262434	0.542622	-0.611472	0.822492	1	RP11-676J12.8	_
ENSG00000263069	0.542622	-0.611472	0.822492	1	CTD-2047H16.4	_
ENSG00000263847	0.542622	-0.611472	0.822492	1	RP11-143J12.3	_
ENSG00000265458	0.542622	-0.611472	0.822492	1	RP13-20L14.6	_
ENSG00000266852	0.542622	-0.611472	0.822492	1	MIR4482-1	_
ENSG00000272369	0.542622	-0.611472	0.822492	1	RP11-446N19.1	_
ENSG00000272659	0.542622	-0.611472	0.822492	1	AP000295.10	_
ENSG00000272758	0.542622	-0.611472	0.822492	1	RP11-299J3.8	_
ENSG00000112599	0.354214	-0.530294	0.822492	1	GUCA1B	K08328
ENSG00000122432	0.354214	-0.530294	0.822492	1	SPATA1	_
ENSG00000136352	0.354214	-0.530294	0.822492	1	NKX2-1	K09342
ENSG00000147144	0.354214	-0.530294	0.822492	1	CCDC120	K15696
ENSG00000152439	0.354214	-0.530294	0.822492	1	ZNF773	K09228
ENSG00000163687	0.354214	-0.530294	0.822492	1	DNASE1L3	K11995
ENSG00000165828	0.354214	-0.530294	0.822492	1	PRAP1	K06115
ENSG00000177374	0.354214	-0.530294	0.822492	1	HIC1	_
ENSG00000182902	0.354214	-0.530294	0.822492	1	SLC25A18	K15107
ENSG00000186510	0.354214	-0.530294	0.822492	1	CLCNKA	K05017
ENSG00000197816	0.354214	-0.530294	0.822492	1	CCDC180	_
ENSG00000204219	0.354214	-0.530294	0.822492	1	TCEA3	_
ENSG00000205704	0.354214	-0.530294	0.822492	1	LINC00634	_
ENSG00000214954	0.354214	-0.530294	0.822492	1	LRRC69	_

ENSG00000215032	0.354214	-0.530294	0.822492	1	GNL3LP1	K10570
ENSG00000227775	0.354214	-0.530294	0.822492	1	RP1-283E3.4	K02993
ENSG00000232303	0.354214	-0.530294	0.822492	1	RP11-205M20.7	K02311
ENSG00000236700	0.354214	-0.530294	0.822492	1	LINC01010	_
ENSG00000236829	0.354214	-0.530294	0.822492	1	Z97634.3	K02893
ENSG00000248677	0.354214	-0.530294	0.822492	1	CTD-2044J15.1	_
ENSG00000248905	0.354214	-0.530294	0.822492	1	FMN1	K10367
ENSG00000255874	0.354214	-0.530294	0.822492	1	LINC00346	_
ENSG00000257285	0.354214	-0.530294	0.822492	1	RP11-298I3.1	_
ENSG00000259298	0.354214	-0.530294	0.822492	1	RP11-562A8.4	_
ENSG00000260103	0.354214	-0.530294	0.822492	1	RP11-10O17.1	_
ENSG00000260751	0.354214	-0.530294	0.822492	1	CTD-2196E14.6	_
ENSG00000264859	0.354214	-0.530294	0.822492	1	RP11-75N4.2	_
ENSG00000265982	0.354214	-0.530294	0.822492	1	RP11-927P21.4	_
ENSG00000266053	0.354214	-0.530294	0.822492	1	RP11-143J12.2	_
ENSG00000266993	0.354214	-0.530294	0.822492	1	RP4-657D16.3	_
ENSG00000268093	0.354214	-0.530294	0.822492	1	AC022154.7	_
ENSG00000269275	0.354214	-0.530294	0.822492	1	CTD-2105E13.15	_
ENSG00000269549	0.354214	-0.530294	0.822492	1	AL031663.2	K17985
ENSG00000272345	0.354214	-0.530294	0.822492	1	RP1-30M3.5	_
ENSG00000272696	0.354214	-0.530294	0.822492	1	RP11-339B21.13	_
ENSG00000165684	-0.154049	5.211036	0.822523	1	SNAPC4	K09453
ENSG00000177051	0.153163	5.209421	0.822523	1	FBXO46	K10320
ENSG00000167702	0.149139	5.533174	0.822567	1	KIFC2	K10406
ENSG00000162191	0.147846	7.047373	0.82265	1	UBXN1	_
ENSG00000144647	-0.167952	3.643263	0.822693	1	POMGNT2	K18207
ENSG00000145945	-0.245376	1.229628	0.82277	1	FAM50B	K13119
ENSG00000172382	-0.245376	1.229628	0.82277	1	PRSS27	K09628
ENSG00000196659	-0.245376	1.229628	0.82277	1	TTC30B	K19683
ENSG00000213190	-0.245376	1.229628	0.82277	1	MLLT11	_
ENSG00000260793	-0.245376	1.229628	0.82277	1	RP5-882C2.2	_
ENSG00000171889	0.241274	1.19811	0.82277	1	MIR31HG	_
ENSG00000106852	-0.208064	1.208343	0.82277	1	LHX6	K09375
ENSG00000261351	-0.208064	1.208343	0.82277	1	CTD-3185P2.1	_
ENSG00000265688	-0.208064	1.208343	0.82277	1	MAFG-AS1	_
ENSG00000167977	0.151319	5.754985	0.822793	1	KCTD5	_
ENSG00000198826	-0.148223	6.730798	0.8228	1	ARHGAP11A	_
ENSG00000146858	-0.179517	2.891613	0.822917	1	ZC3HAV1L	K10734
ENSG00000107223	-0.147004	8.006012	0.822931	1	EDF1	K03627
ENSG00000171467	-0.152703	5.110593	0.822978	1	ZNF318	_
ENSG00000122786	-0.147913	6.789423	0.823078	1	CALD1	K12327
ENSG00000135404	0.146417	9.119377	0.823094	1	CD63	K06497
ENSG00000125971	0.148536	6.23627	0.823157	1	DYNLRB1	K10419
ENSG00000197077	0.149874	5.665299	0.823224	1	KIAA1671	K11447
ENSG00000198736	0.152867	4.332533	0.823266	1	MSRB1	K07305
ENSG00000004897	-0.147484	6.992711	0.823304	1	CDC27	K03350
ENSG00000186185	-0.149403	5.761951	0.823344	1	KIF18B	K10401
ENSG00000123091	0.147991	6.361106	0.823477	1	RNF11	K11980
ENSG00000132286	-0.151175	4.802007	0.823517	1	TIMM10B	K17779
ENSG00000179958	-0.149807	5.669397	0.823517	1	DCTPP1	K16904
ENSG00000131668	-0.148449	6.319103	0.823542	1	BARX1	K09361
ENSG00000129636	-0.150672	5.403484	0.823544	1	ITFG1	K17257
ENSG00000161970	0.14589	9.5525	0.82362	1	RPL26	K02898
ENSG00000144554	0.14823	5.569668	0.823714	1	FANCD2	K10891
ENSG00000184363	-0.148477	6.15782	0.82377	1	PKP3	_
ENSG00000176396	0.152572	4.842722	0.823822	1	EID2	_
ENSG00000009954	-0.159326	3.653622	0.823867	1	BAZ1B	K11658
ENSG00000127589	-0.147412	6.644945	0.823898	1	TUBBP1	K07375

ENSG00000108443	-0.150973	5.673695	0.8241	1	RPS6KB1	K04688
ENSG00000006831	0.146528	7.116677	0.824136	1	ADIPOR2	K07297
ENSG00000139651	0.14814	5.916275	0.824136	1	ZNF740	_
ENSG00000197948	-0.161102	3.998933	0.824147	1	FCHSD1	_
ENSG00000100575	-0.150715	5.288205	0.824165	1	TIMM9	K17777
ENSG00000174718	-0.150715	5.288205	0.824165	1	KIAA1551	_
ENSG00000147526	0.15386	4.144353	0.824245	1	TACC1	K14281
ENSG00000138297	-0.175073	3.32688	0.82425	1	TIMM23	K17794
ENSG00000248487	-0.211117	2.017431	0.824282	1	ABHD14A	K13706
ENSG00000105479	0.299634	0.559845	0.824298	1	CCDC114	_
ENSG00000143429	0.299634	0.559845	0.824298	1	AC027612.6	K14957
ENSG00000175018	0.299634	0.559845	0.824298	1	TEX36	_
ENSG00000196998	0.299634	0.559845	0.824298	1	WDR45	_
ENSG00000263013	0.299634	0.559845	0.824298	1	RP11-876N24.5	_
ENSG00000269089	0.299634	0.559845	0.824298	1	AP003733.1	K06115
ENSG00000118096	-0.280968	0.634607	0.824298	1	IFT46	K19682
ENSG00000135100	-0.280968	0.634607	0.824298	1	HNF1A	K08036
ENSG00000139899	-0.280968	0.634607	0.824298	1	CBLN3	_
ENSG00000244490	-0.280968	0.634607	0.824298	1	RWDD4P1	_
ENSG00000112425	-0.222476	0.601334	0.824298	1	EPM2A	K14165
ENSG00000136867	-0.222476	0.601334	0.824298	1	SLC31A2	K14687
ENSG00000213742	-0.222476	0.601334	0.824298	1	ZNF337-AS1	_
ENSG00000227906	-0.222476	0.601334	0.824298	1	SNAP25-AS1	_
ENSG00000237289	-0.222476	0.601334	0.824298	1	CKMT1B	_
ENSG00000122406	0.145151	10.5663	0.824353	1	RPL5	K02932
ENSG00000115641	-0.145833	7.879035	0.824409	1	FHL2	K14380
ENSG00000204070	0.150878	4.514427	0.824447	1	SYS1	K17776
ENSG00000126581	-0.148518	5.496703	0.824457	1	BECN1	K08334
ENSG00000089248	0.145529	8.26695	0.824465	1	ERP29	K09586
ENSG00000148110	0.146937	6.483859	0.824511	1	HIATL1	_
ENSG00000171161	-0.152367	4.719574	0.824537	1	ZNF672	K09228
ENSG00000126653	-0.14958	5.748155	0.824563	1	NSRP1	K13206
ENSG00000066933	0.154256	4.075747	0.824633	1	MYO9A	K10360
ENSG00000070915	0.154256	4.075747	0.824633	1	SLC12A3	K14426
ENSG00000081026	0.154256	4.075747	0.824633	1	MAGI3	K06112
ENSG00000100784	0.164107	2.519144	0.824692	1	RPS6KA5	K04445
ENSG00000168310	0.151069	4.461631	0.824754	1	IRF2	K10153
ENSG00000103351	-0.152415	4.768635	0.824827	1	CLUAP1	K19684
ENSG00000116922	-0.150452	4.854457	0.824845	1	C1orf109	_
ENSG00000213683	-0.164772	2.908848	0.82486	1	AC002056.3	K11092
ENSG00000105287	0.148302	4.936002	0.824861	1	PRKD2	K06070
ENSG00000197771	-0.145881	7.276686	0.824873	1	MCMBP	_
ENSG00000159842	0.148624	5.354852	0.824928	1	ABR	K08878
ENSG00000175595	-0.165662	3.448164	0.824994	1	ERCC4	K10848
ENSG00000185596	0.15816	3.659303	0.825024	1	WASH3P	K18461
ENSG00000136937	-0.146454	6.617984	0.825079	1	NCBP1	K12882
ENSG00000153406	-0.146411	6.561567	0.825226	1	NMRAL1	K05610
ENSG00000158104	-0.146377	6.479959	0.825412	1	HPD	K00457
ENSG00000070761	-0.148615	5.450345	0.825446	1	C16orf80	_
ENSG00000270081	-0.155591	4.224314	0.825465	1	RP5-935K16.1	_
ENSG00000072415	-0.146546	6.350598	0.825476	1	MPP5	K06091
ENSG00000175063	-0.145965	6.72253	0.82549	1	UBE2C	K06688
ENSG00000079332	0.144714	8.132037	0.825503	1	SAR1A	K07953
ENSG00000165169	0.146247	6.355752	0.825552	1	DYNLT3	K10420
ENSG00000137073	-0.146868	5.479125	0.825604	1	UBAP2	_
ENSG00000066084	0.146129	6.173199	0.825625	1	DIP2B	_
ENSG00000186076	0.166884	3.558499	0.825625	1	RP11-887P2.3	K01834
ENSG00000071894	-0.163556	3.074158	0.825769	1	CPSF1	K14401

ENSG00000198742	-0.149898	4.948297	0.825816	1	SMURF1	K04678
ENSG00000054611	0.147984	4.862483	0.825856	1	TBC1D22A	_
ENSG00000105612	-0.145306	6.998901	0.825881	1	DNASE2	K01158
ENSG00000125868	0.144201	8.564553	0.825913	1	DSTN	K10363
ENSG00000175322	0.186573	2.286524	0.825977	1	ZNF519	K09228
ENSG00000261799	0.186573	2.286524	0.825977	1	RP11-283I3.6	_
ENSG00000159556	-0.183129	2.30092	0.825977	1	ISL2	K18492
ENSG00000124549	0.167252	2.296235	0.825977	1	BTN2A3P	K06712
ENSG00000112541	0.149655	5.062204	0.825982	1	PDE10A	K18438
ENSG00000011295	-0.148634	5.665085	0.825982	1	TTC19	K18169
ENSG00000118804	-0.218205	1.703729	0.825983	1	FAM47E-STBD1	_
ENSG00000228989	-0.218205	1.703729	0.825983	1	AC133528.2	_
ENSG00000261706	-0.218205	1.703729	0.825983	1	AP001505.9	_
ENSG00000049759	0.147972	5.363406	0.826	1	NEDD4L	K13305
ENSG00000258289	0.145159	6.01254	0.826027	1	CHURC1	K09228
ENSG00000102189	-0.147404	4.988882	0.826028	1	EEA1	K12478
ENSG00000011304	0.144104	8.540239	0.826038	1	PTBP1	K13218
ENSG00000154473	-0.144278	8.146745	0.826087	1	BUB3	K02180
ENSG00000156787	-0.148081	5.642452	0.826144	1	TBC1D31	_
ENSG00000140307	-0.145275	6.828133	0.82615	1	GTF2A2	K03123
ENSG00000060339	-0.144758	7.16569	0.826329	1	CCAR1	K11978
ENSG00000203791	-0.150708	4.781434	0.826432	1	METTL10	_
ENSG00000130958	-0.155357	4.421278	0.826452	1	SLC35D2	K15281
ENSG00000267100	0.154247	3.939394	0.826478	1	ILF3-AS1	_
ENSG00000197892	0.164221	3.206016	0.826502	1	KIF13B	K17914
ENSG00000063176	-0.153602	3.21755	0.826502	1	SPHK2	K04718
ENSG00000244398	0.143706	8.555364	0.826506	1	RP11-466H18.1	K02929
ENSG00000155561	-0.144332	7.486746	0.826507	1	NUP205	K14310
ENSG00000103275	-0.144181	7.588393	0.826596	1	UBE2I	K10577
ENSG00000128534	-0.144591	6.314716	0.826692	1	NAA38	K12627
ENSG00000038358	-0.148607	5.371022	0.826708	1	EDC4	K12616
ENSG00000141424	-0.144954	6.684265	0.826749	1	SLC39A6	K14712
ENSG00000169188	-0.146921	5.596091	0.826786	1	APEX2	K10772
ENSG00000116138	-0.162622	3.578602	0.826841	1	DNAJC16	K09536
ENSG00000135473	-0.148075	4.872515	0.826855	1	PAN2	K12571
ENSG00000138119	0.143607	8.008465	0.826889	1	MYOF	K18261
ENSG00000100065	-0.146434	5.857268	0.826894	1	CARD10	_
ENSG00000130758	-0.149284	4.995772	0.826945	1	MAP3K10	K04418
ENSG00000135842	0.146306	5.570591	0.826969	1	FAM129A	_
ENSG00000113282	-0.144393	6.956836	0.827019	1	CLINT1	K12471
ENSG00000066651	-0.150309	5.10932	0.827021	1	TRMT11	K15430
ENSG00000188352	0.144615	5.516972	0.827038	1	FOCAD	_
ENSG00000084733	0.144087	7.035028	0.827125	1	RAB10	K07903
ENSG00000157036	-0.1784	2.545889	0.827137	1	EXOG	K15050
ENSG00000086598	-0.143166	8.618892	0.827177	1	TMED2	K14809
ENSG00000116396	-0.150537	4.487223	0.827376	1	KCNC4	_
ENSG00000161013	-0.1439	7.098527	0.827427	1	MGAT4B	K00738
ENSG00000130363	0.166049	3.071566	0.827465	1	RSPH3	K17985
ENSG00000198837	-0.14538	5.703442	0.827518	1	DENND4B	_
ENSG00000121064	0.146334	5.493785	0.827559	1	SCPEP1	K09646
ENSG00000152464	-0.148709	5.041735	0.827561	1	RPP38	K14523
ENSG00000072210	-0.144526	6.478983	0.827608	1	ALDH3A2	K00128
ENSG00000175348	-0.14485	5.797062	0.827632	1	TMEM9B	_
ENSG00000166025	0.145978	5.925687	0.827659	1	AMOTL1	K06104
ENSG00000075336	-0.152891	4.83617	0.82767	1	TIMM21	K17796
ENSG00000110717	-0.144199	6.656298	0.82769	1	NDUFS8	K03941
ENSG00000179348	0.143843	6.755251	0.827766	1	GATA2	K17894
ENSG00000102024	-0.153064	4.242932	0.827774	1	PLS3	K17336

ENSG00000145495	0.142591	8.488139	0.82786	1		6-Mar	K10661
ENSG00000226742	-0.145522	5.606924	0.827985	1	HSBP1L1		_
ENSG00000203965	-0.153097	4.31076	0.828025	1	EFCAB7		_
ENSG00000100429	0.15779	3.577145	0.82804	1	HDAC10		K18671
ENSG00000174579	0.143549	6.141218	0.828042	1	MSL2		K13164
ENSG00000116675	-0.144687	5.190766	0.828129	1	DNAJC6		K09526
ENSG00000145494	-0.142694	7.88611	0.828136	1	NDUFS6		K03939
ENSG00000185085	0.146159	4.964399	0.828158	1	INTS5		K13142
ENSG00000143799	-0.142192	9.059068	0.828178	1	PARP1		K10798
ENSG00000120314	0.147393	5.580714	0.828188	1	WDR55		_
ENSG00000134291	0.142439	8.070829	0.828243	1	TMEM106C		_
ENSG00000145780	0.147115	5.257258	0.828373	1	FEM1C		_
ENSG00000104067	-0.144302	6.071835	0.828406	1	TJP1		K05701
ENSG00000198876	0.144492	6.162207	0.828513	1	DCAF12		K11803
ENSG00000148985	-0.146573	5.418932	0.828555	1	PGAP2		_
ENSG00000133488	0.168279	2.923291	0.828623	1	SEC14L4		K17981
ENSG00000115657	0.156019	2.929384	0.828623	1	ABCB6		K05661
ENSG00000090863	0.14293	6.896023	0.828666	1	GLG1		K06816
ENSG00000182446	0.142443	7.392166	0.828718	1	NPLOC4		K14015
ENSG00000099326	0.147237	4.703153	0.82875	1	MZF1		K09221
ENSG00000131797	0.182882	2.322151	0.828783	1	CLUHP3		K03255
ENSG00000151572	-0.177569	2.335909	0.828783	1	ANO4		K19499
ENSG00000145194	-0.145699	5.36039	0.829049	1	ECE2		K01415
ENSG00000158773	-0.145699	5.36039	0.829049	1	USF1		K09106
ENSG00000103035	-0.142344	7.160503	0.829203	1	PSMD7		K03038
ENSG00000230291	0.156576	3.591794	0.829222	1	RP11-244J10.1		K02898
ENSG00000233461	0.156576	3.591794	0.829222	1	RP11-295G20.2		_
ENSG00000167674	0.14258	6.783805	0.829227	1	HDGFRP2		_
ENSG00000139505	0.148823	4.890861	0.829304	1	MTMR6		K18083
ENSG00000038210	-0.147169	4.758934	0.829433	1	PI4K2B		_
ENSG00000078304	0.14264	6.581015	0.829449	1	PPP2R5C		K11584
ENSG00000105723	0.144544	6.002306	0.829485	1	GSK3A		K08822
ENSG00000168993	-0.159626	2.567642	0.829515	1	CPLX1		K15294
ENSG00000184990	0.142184	6.322128	0.829543	1	SIVA1		_
ENSG00000107175	-0.146113	5.671121	0.829597	1	CREB3		K09048
ENSG00000114857	-0.14449	5.670261	0.829597	1	NKTR		K12740
ENSG00000146242	-0.142458	6.743555	0.829619	1	TPBG		_
ENSG00000074800	0.140588	11.44076	0.829726	1	ENO1		K01689
ENSG00000151247	-0.143689	5.621242	0.829753	1	EIF4E		K03259
ENSG00000185818	0.146088	5.270962	0.829869	1	NAT8L		K18309
ENSG00000092098	0.152802	3.884022	0.829976	1	RNF31		K11974
ENSG00000140853	0.144913	4.981172	0.829987	1	NLRC5		_
ENSG00000173473	0.141953	6.770197	0.829991	1	SMARCC1		K11649
ENSG00000119402	-0.142665	6.294763	0.830007	1	FBXW2		K10261
ENSG00000155760	-0.158826	2.774106	0.830044	1	FZD7		K02432
ENSG00000227063	0.141125	7.693973	0.830046	1	RPL41P1		K02928
ENSG00000162144	0.143815	5.74601	0.830052	1	CYB561A3		K16295
ENSG00000106628	-0.141137	7.765821	0.830075	1	POLD2		K02328
ENSG00000116251	0.140606	8.670649	0.830153	1	RPL22		K02891
ENSG00000103415	-0.142065	5.818969	0.830209	1	HMOX2		K00510
ENSG00000062716	0.140813	7.960276	0.830243	1	VMP1		_
ENSG00000103264	0.1453	4.898249	0.830264	1	FBXO31		K10308
ENSG00000214063	-0.142422	6.357575	0.830334	1	TSPAN4		K17294
ENSG00000198728	0.146626	5.489885	0.830353	1	LDB1		K15617
ENSG00000184271	-0.151683	3.603891	0.830389	1	POU6F1		K09368
ENSG00000138668	-0.140555	8.293389	0.830437	1	HNRNPD		K13044
ENSG00000134049	0.14195	6.471038	0.830446	1	IER3IP1		_
ENSG00000060069	0.146101	5.208244	0.830474	1	CTDP1		K15732

ENSG00000126461	-0.14205	6.498512	0.830509	1	SCAF1	_
ENSG00000244687	0.154775	3.700432	0.830594	1	UBE2V1	K10704
ENSG00000156858	-0.153687	4.203037	0.830635	1	PRR14	_
ENSG00000072062	0.145286	4.85796	0.83066	1	PRKACA	K04345
ENSG00000198786	0.139806	11.15989	0.830675	1	MT-ND5	K03883
ENSG00000076604	-0.140855	7.418721	0.830705	1	TRAF4	K09848
ENSG00000056736	-0.15774	3.11509	0.830759	1	IL17RB	K05165
ENSG00000025434	0.144504	4.98672	0.830887	1	NR1H3	K08536
ENSG00000142546	-0.141568	6.562975	0.830968	1	NOSIP	K13125
ENSG00000143889	-0.141764	5.996271	0.83101	1	HNRNPLL	K13159
ENSG00000106330	0.151184	3.248448	0.831012	1	MOSPD3	_
ENSG00000119471	-0.141276	6.746419	0.831018	1	HSDL2	_
ENSG00000117758	-0.146899	3.979457	0.831068	1	STX12	K13813
ENSG00000167566	-0.145723	5.144431	0.831136	1	NCKAP5L	K17985
ENSG00000137221	0.143529	5.143922	0.831136	1	TJAP1	K06105
ENSG00000124788	0.146867	4.51637	0.831166	1	ATXN1	_
ENSG00000168887	0.145747	5.180681	0.83119	1	C2orf68	K17985
ENSG00000172663	-0.152259	4.063185	0.831191	1	TMEM134	_
ENSG00000181218	-0.149053	3.386307	0.831219	1	HIST3H2A	K11251
ENSG00000154920	-0.14413	5.250702	0.831292	1	EME1	K10882
ENSG00000118680	0.139548	8.992823	0.83131	1	MYL12B	K12757
ENSG00000104980	-0.140686	6.058753	0.831318	1	TIMM44	K17804
ENSG00000113649	-0.140507	7.218457	0.831323	1	TCERG1	K12824
ENSG00000057294	-0.146687	4.994391	0.831334	1	PKP2	K12642
ENSG00000174684	-0.146687	4.994391	0.831334	1	B3GNT1	K00741
ENSG00000006453	-0.14155	6.372995	0.831339	1	BAIAP2L1	K01557
ENSG00000179532	0.141868	5.554806	0.831379	1	DNHD1	_
ENSG00000114859	-0.149438	3.506918	0.831393	1	CLCN2	K05011
ENSG00000273290	-0.149764	3.618213	0.83154	1	CTC-297N7.8	_
ENSG00000129810	-0.144362	4.343449	0.831574	1	SGOL1	K11580
ENSG00000013375	0.140974	6.442558	0.831653	1	PGM3	K01836
ENSG00000196453	0.153689	3.713885	0.831666	1	ZNF777	K09228
ENSG00000180992	-0.141375	6.174923	0.831863	1	MRPL14	K02874
ENSG00000185475	0.145688	3.898891	0.831872	1	TMEM179B	K03131
ENSG00000004866	0.145745	5.113818	0.831899	1	ST7	_
ENSG00000141577	-0.141818	5.115638	0.831899	1	AZI1	K16540
ENSG00000152558	-0.139197	8.216211	0.832096	1	TMEM123	_
ENSG00000196411	-0.140152	6.886734	0.832152	1	EPHB4	K05113
ENSG00000168591	0.146581	4.958764	0.832188	1	TMUB2	_
ENSG00000162636	0.141591	5.966255	0.832312	1	FAM102B	K11339
ENSG00000110880	0.139275	7.601861	0.832316	1	CORO1C	K13886
ENSG00000076984	-0.143057	5.695008	0.832373	1	MAP2K7	K04431
ENSG00000130706	0.139769	6.898955	0.832419	1	ADRM1	_
ENSG00000196455	-0.141193	5.83825	0.832463	1	PIK3R4	K08333
ENSG00000122566	-0.138345	10.35055	0.832503	1	HNRNPA2B1	K13158
ENSG00000182247	0.147632	4.785016	0.832549	1	UBE2E2	K06689
ENSG00000158545	0.139744	6.069182	0.832591	1	ZC3H18	K13092
ENSG00000128692	-0.145022	4.963815	0.83264	1	EIF2S2P4	K03238
ENSG00000091009	-0.142646	5.298257	0.832784	1	RBM27	K13193
ENSG00000099817	0.139009	7.403858	0.832795	1	POLR2E	K03013
ENSG00000144136	-0.139026	7.505706	0.832801	1	SLC20A1	K14640
ENSG00000171953	0.150938	3.907626	0.832804	1	ATPAF2	K07556
ENSG00000117425	-0.33718	0.667029	0.832809	1	PTCH2	K11101
ENSG00000138380	-0.33718	0.667029	0.832809	1	CARF	_
ENSG00000146021	-0.33718	0.667029	0.832809	1	KLHL3	K10443
ENSG00000166823	-0.33718	0.667029	0.832809	1	MESP1	K09076
ENSG00000180673	-0.33718	0.667029	0.832809	1	EXOC5P1	_
ENSG00000205918	-0.33718	0.667029	0.832809	1	PDPK2	K06276

ENSG00000227258	-0.33718	0.667029	0.832809	1	SMIM2-AS1	_
ENSG00000260456	-0.33718	0.667029	0.832809	1	C16orf95	_
ENSG00000271980	-0.33718	0.667029	0.832809	1	CTD-2256P15.4	_
ENSG00000099219	0.139247	7.084348	0.832824	1	ERMP1	K04590
ENSG00000076067	-0.155907	3.996133	0.832837	1	RBMS2	_
ENSG00000131943	-0.155907	3.996133	0.832837	1	C19orf12	_
ENSG00000172992	-0.151012	4.149681	0.832891	1	DCAKD	_
ENSG00000227232	-0.151012	4.149681	0.832891	1	WASH7P	K18461
ENSG00000125354	-0.141559	5.623912	0.832895	1	6-Sep	K16939
ENSG00000108468	-0.139527	6.873715	0.832913	1	CBX1	K11585
ENSG00000165943	0.147816	4.221702	0.832914	1	MOAP1	K12581
ENSG00000170382	0.147816	4.221702	0.832914	1	LRRN2	_
ENSG00000178149	0.150629	4.415084	0.83297	1	DALRD3	_
ENSG00000185361	-0.14451	4.533344	0.833	1	TNFAIP8L1	K17985
ENSG00000160606	-0.145495	4.694371	0.833037	1	TLCD1	_
ENSG00000137831	-0.140968	6.272263	0.833053	1	UACA	_
ENSG00000148308	0.139539	6.491171	0.833275	1	GTF3C5	K15202
ENSG00000105229	0.144326	5.199914	0.833509	1	PIAS4	K16065
ENSG00000187531	0.141841	5.201111	0.833509	1	SIRT7	K11417
ENSG00000139835	0.140551	4.794563	0.833562	1	GRTP1	_
ENSG00000166333	-0.141431	4.699422	0.833577	1	ILK	K06272
ENSG00000181029	-0.145045	4.157016	0.833675	1	TRAPPC5	K10840
ENSG00000034063	-0.139072	6.690241	0.833724	1	UHRF1	_
ENSG00000169499	-0.139522	5.682264	0.833734	1	PLEKHA2	K17985
ENSG00000116990	0.144759	3.829615	0.833747	1	MYCL	K09110
ENSG00000179294	-0.139791	6.244788	0.833791	1	C17orf96	_
ENSG00000100802	0.160529	3.631215	0.833795	1	C14orf93	_
ENSG00000188997	-0.162604	3.411495	0.833855	1	KCTD21	_
ENSG00000243667	-0.162604	3.411495	0.833855	1	WDR92	K17985
ENSG00000144029	-0.138497	7.090212	0.833861	1	MRPS5	K02988
ENSG00000149600	-0.143124	5.240303	0.833876	1	COMMD7	_
ENSG00000118200	-0.142354	5.829027	0.833943	1	CAMSAP2	K17493
ENSG00000179562	-0.14415	5.056287	0.833948	1	GCC1	_
ENSG00000213904	-0.264818	1.321827	0.833964	1	LIPE-AS1	_
ENSG00000270362	-0.264818	1.321827	0.833964	1	HMGN3-AS1	_
ENSG00000270055	-0.230193	1.301974	0.833964	1	CTD-3092A11.2	_
ENSG00000272593	0.190044	1.293491	0.833964	1	RP11-339B21.11	_
ENSG00000159433	0.15233	2.974814	0.833976	1	STARD9	K16491
ENSG00000100811	0.137926	7.485216	0.834015	1	YY1	K09201
ENSG00000113811	-0.145756	4.890351	0.834021	1	SELK	K09228
ENSG00000259032	0.166383	2.809102	0.834026	1	ENSAP2	_
ENSG00000147912	-0.183207	2.612126	0.83408	1	FBXO10	K10296
ENSG00000204745	-0.183207	2.612126	0.83408	1	AC083899.3	K03348
ENSG00000267796	0.171846	2.600651	0.83408	1	LIN37	_
ENSG00000078618	0.137675	7.761239	0.834107	1	NRD1	K01411
ENSG00000100031	0.140816	5.40336	0.834149	1	GGT1	K18592
ENSG00000184983	0.201738	1.748114	0.834171	1	NDUFA6	K03950
ENSG00000091157	-0.196271	2.101084	0.834179	1	WDR7	_
ENSG00000147955	0.137588	7.756579	0.834214	1	SIGMAR1	_
ENSG00000143153	-0.137377	8.169948	0.834292	1	ATP1B1	K01540
ENSG00000167962	0.13786	7.194502	0.834359	1	ZNF598	_
ENSG00000196419	-0.136894	9.546268	0.834369	1	XRCC6	K10884
ENSG00000078403	-0.142961	5.554733	0.834369	1	MLLT10	K06630
ENSG00000141971	-0.142747	5.526485	0.83439	1	MVB12A	K12186
ENSG00000091164	-0.13843	5.810409	0.834453	1	TXNL1	_
ENSG00000058453	-0.143245	4.091655	0.834507	1	CROCC	K16469
ENSG00000240344	0.143011	4.847336	0.834526	1	PPIL3	K12734
ENSG00000119685	0.148961	4.00651	0.834569	1	TTLL5	K16602

ENSG00000150977	-0.147803	4.015148	0.834569	1	RILPL2	_
ENSG00000173852	0.140387	5.922787	0.8346	1	DPY19L1	_
ENSG00000105321	0.143724	4.704853	0.834647	1	CCDC9	_
ENSG00000244716	0.137047	7.994386	0.834709	1	RP11-20O24.4	K02880
ENSG00000213866	-0.147035	3.842547	0.834716	1	YBX1P10	K09276
ENSG00000067606	0.144696	3.743794	0.834802	1	PRKCZ	K18952
ENSG00000184349	0.144696	3.743794	0.834802	1	EFNA5	K05462
ENSG00000174227	-0.145822	4.493045	0.834852	1	PIGG	K05310
ENSG00000093167	0.140313	5.903122	0.834853	1	LRRFIP2	_
ENSG00000009335	0.137795	6.79529	0.8349	1	UBE3C	K10589
ENSG00000065548	-0.137813	6.899649	0.834916	1	ZC3H15	_
ENSG00000125484	-0.139387	6.219721	0.834934	1	GTF3C4	K11310
ENSG00000206195	-0.152613	3.541268	0.835013	1	AP000525.9	_
ENSG00000165209	-0.137509	5.928094	0.835059	1	STRBP	K13200
ENSG00000142544	-0.152513	3.423622	0.835143	1	CTU1	K14168
ENSG00000255150	0.146881	3.418171	0.835143	1	EID3	_
ENSG00000015475	-0.142911	4.714532	0.835177	1	BID	K04726
ENSG00000132561	0.13937	5.616035	0.835187	1	MATN2	_
ENSG00000133983	-0.145284	5.068096	0.835199	1	COX16	K18182
ENSG00000173083	-0.142517	4.663558	0.835241	1	HPSE	K07964
ENSG00000128283	0.136999	7.324483	0.835259	1	CDC42EP1	_
ENSG00000109805	-0.137438	6.86623	0.835407	1	NCAPG	K06678
ENSG00000137504	-0.136847	7.45116	0.835446	1	CREBZF	_
ENSG00000141664	-0.14482	4.500804	0.835465	1	ZCCHC2	_
ENSG00000196363	-0.138032	6.375647	0.835509	1	WDR5	K14963
ENSG00000061273	-0.137516	6.723391	0.835523	1	HDAC7	K11408
ENSG00000005073	-0.152072	2.999068	0.835687	1	HOXA11	K09296
ENSG00000011009	-0.140839	5.725978	0.83585	1	LYPLA2	K06130
ENSG00000184731	-0.163706	2.830827	0.835948	1	FAM110C	K13194
ENSG00000115159	-0.13936	5.777227	0.83602	1	GPD2	K00111
ENSG00000140992	0.14399	4.502903	0.836073	1	PDPK1	K06276
ENSG00000243646	0.140962	5.035712	0.836096	1	IL10RB	K05135
ENSG00000065135	-0.135968	7.931554	0.836116	1	GNAI3	K04630
ENSG00000063245	-0.136489	7.137679	0.836195	1	EPN1	K12471
ENSG00000171148	-0.137329	6.042893	0.836211	1	TADA3	K11315
ENSG00000115107	0.139176	5.512172	0.836245	1	STEAP3	K10142
ENSG00000132846	-0.151495	4.028679	0.836267	1	ZBED3	_
ENSG00000101751	0.169174	2.629362	0.836273	1	POLI	K03510
ENSG00000224930	-0.144902	4.672191	0.836327	1	LINC00162	_
ENSG00000082213	-0.137294	6.373008	0.836391	1	C5orf22	_
ENSG00000122042	-0.138885	5.572342	0.836426	1	UBL3	_
ENSG00000008869	0.148253	3.942321	0.836433	1	HEATR5B	_
ENSG00000070501	-0.144571	4.619671	0.836436	1	POLB	K02330
ENSG00000113068	-0.137251	6.198399	0.836495	1	PFDN1	K09548
ENSG00000228589	-0.139546	5.193156	0.836581	1	SPCS2P4	K12947
ENSG00000133265	-0.137322	5.629237	0.836593	1	HSPBP1	K09562
ENSG00000226562	0.176028	2.39086	0.836688	1	CYP4F26P	_
ENSG00000106617	-0.167247	2.403458	0.836688	1	PRKAG2	K07200
ENSG00000173480	0.158143	2.399853	0.836688	1	ZNF417	K09228
ENSG00000185627	-0.136514	6.676176	0.836787	1	PSMD13	K03039
ENSG00000180233	0.143922	4.449678	0.836813	1	ZNRF2	K10694
ENSG00000100625	-0.140778	4.67732	0.836865	1	SIX4	K15615
ENSG00000137288	-0.137104	5.232156	0.83687	1	UQCC2	K17682
ENSG00000100906	-0.136747	6.182799	0.836914	1	NFKBIA	K04734
ENSG00000111276	0.136578	6.386997	0.83697	1	CDKN1B	K06624
ENSG00000114554	-0.137246	5.988017	0.836988	1	PLXNA1	K06820
ENSG00000101911	-0.136671	6.403876	0.837071	1	PRPS2	K00948
ENSG00000189423	-0.17017	2.130374	0.837235	1	USP32P3	K11837

ENSG00000110723	0.164008	2.116078	0.837235	1	EXPH5	_
ENSG00000131470	0.139695	5.197531	0.83733	1	PSMC3IP	K06695
ENSG00000137802	0.140673	4.188459	0.837485	1	MAPKBP1	_
ENSG00000181045	0.140673	4.188459	0.837485	1	SLC26A11	K14708
ENSG00000155189	-0.139089	5.272405	0.8375	1	AGPAT5	K19007
ENSG00000211456	-0.139089	5.272405	0.8375	1	SACM1L	_
ENSG00000007392	-0.137785	5.789959	0.837522	1	LUC7L	_
ENSG00000141027	-0.13611	6.491242	0.837578	1	NCOR1	K04650
ENSG00000127445	0.13558	5.971848	0.837698	1	PIN1	K09578
ENSG00000136897	-0.136482	6.016733	0.837789	1	MRPL50	K17431
ENSG00000214975	0.15113	2.840478	0.837826	1	PPIAP29	K03767
ENSG00000116863	0.136227	5.498647	0.837831	1	ADPRHL2	K11687
ENSG00000110066	0.138878	4.520248	0.837872	1	SUV420H1	K11429
ENSG00000148730	0.136428	6.154199	0.837972	1	EIF4EBP2	K18644
ENSG00000171790	-0.199305	1.806924	0.837983	1	SLFNL1	_
ENSG00000006530	0.195822	1.799603	0.837983	1	AGK	K09881
ENSG00000177542	-0.137796	5.865573	0.838035	1	SLC25A22	K15107
ENSG00000170037	-0.139551	5.092697	0.838046	1	CNTROB	K16468
ENSG00000089157	0.133604	11.28863	0.838072	1	RPLP0	K02941
ENSG00000107581	0.133823	8.770056	0.83821	1	EIF3A	K03254
ENSG00000197323	0.134948	6.837332	0.838233	1	TRIM33	K08883
ENSG00000171302	0.13539	6.373295	0.838408	1	CANT1	K12304
ENSG00000154582	-0.134845	6.905888	0.83844	1	TCEB1	K03872
ENSG00000090661	-0.158886	3.179452	0.838473	1	CERS4	K04710
ENSG00000005059	0.157338	3.172505	0.838473	1	CCDC109B	_
ENSG00000106701	-0.148626	3.57102	0.838483	1	FSD1L	_
ENSG00000137103	-0.148626	3.57102	0.838483	1	TMEM8B	_
ENSG00000168894	0.138492	5.798198	0.838509	1	RNF181	_
ENSG00000123374	-0.134288	7.430488	0.838515	1	CDK2	K02206
ENSG00000131941	0.136338	5.280036	0.838557	1	RHPN2	_
ENSG00000101596	-0.134798	6.785904	0.838663	1	SMCHD1	_
ENSG00000139546	-0.13742	5.507342	0.838734	1	TARBP2	K18420
ENSG00000104886	-0.137059	5.871575	0.838736	1	PLEKHJ1	_
ENSG00000082068	-0.135191	6.407499	0.838824	1	WDR70	_
ENSG00000122696	0.1529	3.446507	0.83889	1	SLC25A51	K17675
ENSG00000176463	-0.138912	4.205285	0.838962	1	SLCO3A1	K14353
ENSG00000138107	0.134122	7.043659	0.838971	1	ACTR1A	K16575
ENSG00000142327	0.135845	5.352714	0.838994	1	RNPEPL1	K09605
ENSG00000166233	0.134608	6.563463	0.839023	1	ARIH1	K11968
ENSG00000151746	-0.139847	4.936865	0.839043	1	BICD1	K18739
ENSG00000122547	0.220806	1.344091	0.839045	1	EEPD1	_
ENSG00000250320	0.220806	1.344091	0.839045	1	CTD-2269F5.1	_
ENSG00000125864	0.182504	1.36357	0.839045	1	BFSP1	K10378
ENSG00000128563	0.182504	1.36357	0.839045	1	PRKRIP1	K09228
ENSG00000010256	0.133237	8.38027	0.83906	1	UQCRC1	K00414
ENSG00000122965	-0.134853	5.022951	0.839145	1	RBM19	K14787
ENSG00000010810	0.133517	7.634	0.839155	1	FYN	K05703
ENSG00000133401	0.172839	2.424021	0.839163	1	PDZD2	_
ENSG00000141391	-0.146709	2.427398	0.839163	1	SLMO1	_
ENSG00000160799	0.142185	4.473588	0.839278	1	CCDC12	K12871
ENSG00000198408	0.133418	7.559422	0.83933	1	MGEA5	K15719
ENSG00000172244	-0.135411	5.94527	0.839385	1	C5orf34	_
ENSG00000103266	-0.133896	7.03047	0.83941	1	STUB1	K09561
ENSG00000015532	-0.135645	5.628352	0.839443	1	XYLT2	K00771
ENSG00000105171	-0.135479	5.390998	0.83952	1	POP4	K03538
ENSG00000248527	0.13273	8.500373	0.839616	1	MTATP6P1	K02126
ENSG00000226084	0.138544	5.107482	0.83963	1	RP4-706A16.3	K02880
ENSG00000095319	-0.134195	6.622927	0.839631	1	NUP188	K14311

ENSG00000040633	-0.134594	6.35117	0.839643	1	PHF23	_
ENSG00000177156	0.133313	7.330476	0.839648	1	TALDO1	K00616
ENSG00000174151	-0.171779	2.861545	0.839662	1	CYB561D1	_
ENSG00000197879	0.132843	7.87998	0.839793	1	MYO1C	K10356
ENSG00000103363	0.13308	7.446217	0.839824	1	TCEB2	K03873
ENSG00000106211	0.132209	9.53767	0.839939	1	HSPB1	K04455
ENSG00000136715	0.135427	5.293527	0.839944	1	SAP130	K19192
ENSG00000181938	-0.140584	4.70615	0.840019	1	GINS3	_
ENSG00000100979	-0.536946	-0.330685	0.840166	1	PLTP	K08761
ENSG00000114670	-0.536946	-0.330685	0.840166	1	NEK11	K08857
ENSG00000130711	-0.536946	-0.330685	0.840166	1	PRDM12	_
ENSG00000159714	-0.536946	-0.330685	0.840166	1	ZDHHC1	K18932
ENSG00000170379	-0.536946	-0.330685	0.840166	1	FAM115C	_
ENSG00000172478	-0.536946	-0.330685	0.840166	1	C2orf54	K00757
ENSG00000176472	-0.536946	-0.330685	0.840166	1	ZNF575	K17725
ENSG00000177483	-0.536946	-0.330685	0.840166	1	RBM44	_
ENSG00000179528	-0.536946	-0.330685	0.840166	1	LBX2	K09353
ENSG00000203836	-0.536946	-0.330685	0.840166	1	NBPF24	_
ENSG00000213225	-0.536946	-0.330685	0.840166	1	AC018804.7	K14833
ENSG00000227496	-0.536946	-0.330685	0.840166	1	RP11-145A3.1	_
ENSG00000228307	-0.536946	-0.330685	0.840166	1	OR2S1P	K04257
ENSG00000234290	-0.536946	-0.330685	0.840166	1	AC116366.6	_
ENSG00000242445	-0.536946	-0.330685	0.840166	1	RPL7AP11	K02936
ENSG00000256885	-0.536946	-0.330685	0.840166	1	AP001877.1	_
ENSG00000258568	-0.536946	-0.330685	0.840166	1	RHOQP1	K07194
ENSG00000259715	-0.536946	-0.330685	0.840166	1	CTD-3110H11.1	_
ENSG00000261533	-0.536946	-0.330685	0.840166	1	RP11-15N24.4	_
ENSG00000261889	-0.536946	-0.330685	0.840166	1	RP11-473M20.16	_
ENSG00000266992	-0.536946	-0.330685	0.840166	1	DHX40P1	K18711
ENSG00000267913	-0.536946	-0.330685	0.840166	1	AP001055.1	_
ENSG00000100100	0.446749	-0.364163	0.840166	1	PIK3IP1	K12581
ENSG00000115602	0.446749	-0.364163	0.840166	1	IL1RL1	K05171
ENSG00000164532	0.446749	-0.364163	0.840166	1	TBX20	K10185
ENSG00000180764	0.446749	-0.364163	0.840166	1	PIPSL	K00889
ENSG00000205639	0.446749	-0.364163	0.840166	1	MFSD2B	_
ENSG00000205871	0.446749	-0.364163	0.840166	1	RPS3AP47	K02984
ENSG00000231806	0.446749	-0.364163	0.840166	1	PCAT7	_
ENSG00000232648	0.446749	-0.364163	0.840166	1	RP11-367N14.2	_
ENSG00000234936	0.446749	-0.364163	0.840166	1	AC010883.5	_
ENSG00000238054	0.446749	-0.364163	0.840166	1	RP11-316I3.2	_
ENSG00000243696	0.446749	-0.364163	0.840166	1	RP5-966M1.6	_
ENSG00000259515	0.446749	-0.364163	0.840166	1	RP11-365N19.2	_
ENSG00000261840	0.446749	-0.364163	0.840166	1	RP11-146F11.1	_
ENSG00000049249	-0.413226	-0.399295	0.840166	1	TNFRSF9	K05146
ENSG00000105650	-0.413226	-0.399295	0.840166	1	PDE4C	K13293
ENSG00000126500	-0.413226	-0.399295	0.840166	1	FLRT1	K16362
ENSG00000136040	-0.413226	-0.399295	0.840166	1	PLXNC1	K06572
ENSG00000144642	-0.413226	-0.399295	0.840166	1	RBMS3	_
ENSG00000157306	-0.413226	-0.399295	0.840166	1	RP11-66N24.4	_
ENSG00000179304	-0.413226	-0.399295	0.840166	1	FAM156B	_
ENSG00000185044	-0.413226	-0.399295	0.840166	1	RP11-435B5.4	_
ENSG00000198326	-0.413226	-0.399295	0.840166	1	TMEM239	_
ENSG00000223947	-0.413226	-0.399295	0.840166	1	AC016738.4	_
ENSG00000224046	-0.413226	-0.399295	0.840166	1	AC005076.5	_
ENSG00000228486	-0.413226	-0.399295	0.840166	1	LINC01125	_
ENSG00000260633	-0.413226	-0.399295	0.840166	1	RP11-375I20.6	_
ENSG00000260833	-0.413226	-0.399295	0.840166	1	AC124789.1	_
ENSG00000272899	-0.413226	-0.399295	0.840166	1	RP11-309L24.9	_

ENSG00000273055	-0.413226	-0.399295	0.840166	1	CTB-13F3.1	_
ENSG00000138336	-0.164276	2.169694	0.840181	1	TET1	K13097
ENSG00000188283	-0.164276	2.169694	0.840181	1	ZNF383	K09228
ENSG00000110344	0.160487	2.156094	0.840181	1	UBE4A	K10596
ENSG00000232389	0.160487	2.156094	0.840181	1	RP11-134K13.2	K00802
ENSG00000167720	-0.146472	3.893575	0.840299	1	SRR	K12235
ENSG00000186815	-0.135242	5.886473	0.84035	1	TPCN1	K16896
ENSG00000110237	0.138955	4.94603	0.84036	1	ARHGEF17	_
ENSG00000135392	0.135996	4.947462	0.84036	1	DNAJC14	K09534
ENSG00000109323	-0.136951	5.716844	0.84042	1	MANBA	K01192
ENSG00000177595	-0.14217	4.360604	0.840451	1	PIDD	K10130
ENSG00000169738	-0.136374	5.864823	0.840458	1	DCXR	K03331
ENSG00000237438	0.151836	2.664905	0.840489	1	CECR7	_
ENSG00000213139	-0.254846	0.742418	0.84052	1	CRYGS	K16810
ENSG00000123411	0.214295	0.706746	0.84052	1	IKZF4	K09220
ENSG00000198780	0.214295	0.706746	0.84052	1	FAM169A	_
ENSG00000236242	0.214295	0.706746	0.84052	1	MYO16-AS1	_
ENSG00000257605	0.214295	0.706746	0.84052	1	RP11-680A11.5	_
ENSG00000271862	0.214295	0.706746	0.84052	1	RP11-343L5.2	_
ENSG00000065882	-0.137959	4.90503	0.84057	1	TBC1D1	K18341
ENSG00000184009	-0.133145	6.759454	0.84067	1	ACTG1	K05692
ENSG00000119899	0.134114	5.718761	0.840676	1	SLC17A5	K12301
ENSG00000166971	0.144336	3.338065	0.84068	1	AKTIP	_
ENSG00000145882	-0.145989	3.802252	0.840739	1	PCYOX1L	K05906
ENSG00000161677	-0.146323	4.147166	0.840766	1	JOSD2	K15235
ENSG00000166224	-0.135947	4.660063	0.840787	1	SGPL1	K01634
ENSG00000156256	-0.135081	5.526486	0.840802	1	USP16	K11844
ENSG00000107331	0.132752	6.859034	0.840821	1	ABCA2	K05642
ENSG00000141258	0.133604	5.959204	0.840913	1	SGSM2	_
ENSG00000154639	0.133579	5.980905	0.841018	1	CXADR	K06788
ENSG00000106346	0.13598	4.81499	0.841029	1	USP42	K11855
ENSG00000161533	0.136268	4.714947	0.841044	1	ACOX1	K00232
ENSG00000112592	-0.139413	4.768582	0.841282	1	TBP	K03120
ENSG00000227615	-0.153631	3.475803	0.841295	1	RP11-864N7.2	K02951
ENSG00000165102	0.136788	4.550536	0.841354	1	HGSNAT	K10532
ENSG00000196417	-0.156827	2.879136	0.841457	1	ZNF765	K04614
ENSG00000111319	0.143487	4.149404	0.841515	1	SCNN1A	K04824
ENSG00000014919	-0.135485	5.920881	0.841583	1	COX15	K02259
ENSG00000198892	0.168429	1.813508	0.841624	1	SHISA4	_
ENSG00000133103	-0.1397	4.960988	0.841656	1	COG6	_
ENSG00000125611	-0.141093	4.369105	0.841746	1	CHCHD5	_
ENSG00000163755	-0.132807	6.133716	0.841861	1	HPS3	_
ENSG00000119953	-0.132854	5.83058	0.841869	1	SMNDC1	K12839
ENSG00000104450	-0.134944	4.724479	0.842055	1	SPAG1	_
ENSG00000131188	0.141733	4.304025	0.842124	1	PRR7	_
ENSG00000169180	-0.131735	6.901141	0.842151	1	XPO6	_
ENSG00000001036	-0.13149	7.12174	0.842171	1	FUCA2	K01206
ENSG00000104835	0.156735	3.056053	0.842194	1	SARS2	K01875
ENSG00000101444	-0.130793	7.935705	0.84229	1	AHCY	K01251
ENSG00000090054	-0.132275	6.388277	0.84233	1	SPTLC1	K00654
ENSG00000118482	0.131733	6.5933	0.842401	1	PHF3	_
ENSG00000185946	-0.14404	4.001488	0.842421	1	RNPC3	K13157
ENSG00000203880	-0.138338	4.828424	0.842442	1	PCMTD2	_
ENSG00000164494	0.14212	3.482464	0.842471	1	PDSS2	K12505
ENSG00000108828	0.130469	8.158616	0.842472	1	VAT1	_
ENSG00000163798	-0.134947	5.211009	0.842611	1	SLC4A1AP	K01368
ENSG00000164252	-0.134661	5.514071	0.842715	1	AGGF1	_
ENSG00000106009	-0.131702	6.55184	0.842718	1	BRAT1	_

ENSG00000113810	0.130703	7.328553	0.842763	1	SMC4	K06675
ENSG00000105705	0.13756	4.924521	0.842779	1	SUGP1	K13096
ENSG00000125633	-0.1335	5.389952	0.842885	1	CCDC93	_
ENSG00000019549	-0.134271	5.485007	0.842903	1	SNAI2	K05706
ENSG00000185800	0.137931	4.83203	0.842908	1	DMWD	_
ENSG00000173559	-0.137576	4.834579	0.842908	1	NABP1	_
ENSG00000172046	-0.134136	5.739994	0.842945	1	USP19	K11847
ENSG00000138593	-0.135072	5.359316	0.843094	1	SECISBP2L	_
ENSG00000145916	-0.135072	5.359316	0.843094	1	RMND5B	K15696
ENSG00000124164	0.133818	5.934304	0.843116	1	VAPB	K10707
ENSG00000155304	-0.131533	6.385661	0.843218	1	HSPA13	K09491
ENSG00000182923	-0.14341	4.449559	0.843228	1	CEP63	K16763
ENSG00000111885	-0.138696	4.245125	0.843234	1	MAN1A1	K01230
ENSG00000163517	-0.151308	3.369478	0.843243	1	HDAC11	K11418
ENSG00000132669	-0.134645	5.326924	0.843313	1	RIN2	_
ENSG00000143158	-0.132047	5.937431	0.843333	1	MPC2	_
ENSG00000083444	0.129685	8.237209	0.84337	1	PLOD1	K00473
ENSG00000141522	-0.130553	6.989682	0.843446	1	ARHGDIA	K12462
ENSG00000162430	-0.135721	5.144414	0.843457	1	SEPN1	K12581
ENSG00000037749	-0.131429	5.692477	0.843536	1	MFAP3	_
ENSG00000129071	-0.131287	6.369849	0.84354	1	MBD4	K10801
ENSG00000197563	0.133512	5.18191	0.843571	1	PIGN	K05285
ENSG00000117143	0.130129	7.145096	0.843625	1	UAP1	K00972
ENSG00000108848	0.129349	8.448915	0.843676	1	LUC7L3	_
ENSG00000132823	0.131961	5.984372	0.843677	1	OSER1	_
ENSG00000005075	0.133596	5.104944	0.843721	1	POLR2J	K03008
ENSG00000122729	-0.131037	6.424961	0.843734	1	ACO1	K01681
ENSG00000119203	-0.13103	5.871584	0.843794	1	CPSF3	K14403
ENSG00000163479	0.129558	7.722243	0.843819	1	SSR2	K13250
ENSG00000130768	-0.204233	1.436702	0.843823	1	SMPDL3B	K01128
ENSG00000127124	-0.171934	1.418457	0.843823	1	HIVEP3	K09239
ENSG00000230487	-0.171934	1.418457	0.843823	1	PSMG3-AS1	_
ENSG00000125166	-0.12972	7.551328	0.843858	1	GOT2	K14455
ENSG00000168827	-0.130377	5.987339	0.843885	1	GFM1	K02355
ENSG00000134058	0.152757	2.465002	0.843897	1	CDK7	K02202
ENSG00000146530	0.152757	2.465002	0.843897	1	VWDE	_
ENSG00000115942	-0.130619	5.670272	0.843976	1	ORC2	K02604
ENSG00000065600	-0.134631	4.519974	0.843993	1	TMEM206	_
ENSG00000269609	0.142825	3.234711	0.844158	1	RP11-18I14.10	_
ENSG00000162599	0.131957	5.776739	0.84424	1	NFIA	K09168
ENSG00000115520	0.137088	4.844306	0.844288	1	COQ10B	K18588
ENSG00000160691	0.129334	7.425416	0.844313	1	SHC1	K06279
ENSG00000219545	-0.135022	4.696014	0.844411	1	RPA3-AS1	_
ENSG00000121644	0.130318	6.36957	0.844449	1	DESI2	_
ENSG00000179933	0.130147	5.969039	0.844458	1	C14orf119	K12875
ENSG00000142396	0.138309	3.832781	0.844467	1	ERVK3-1	_
ENSG00000169258	0.1349	4.522183	0.844563	1	GPRIN1	_
ENSG00000174903	0.129203	7.281936	0.844596	1	RAB1B	K07875
ENSG00000204991	0.131747	5.754964	0.844665	1	SPIRE2	K02098
ENSG00000090621	-0.128669	8.076484	0.844741	1	PABPC4	K13126
ENSG00000177706	-0.13479	5.27013	0.844806	1	FAM20C	_
ENSG00000126602	-0.128427	8.407567	0.844857	1	TRAP1	K09488
ENSG00000124789	-0.129177	7.173069	0.844873	1	NUP153	K14296
ENSG00000151849	-0.130493	5.306022	0.844877	1	CENPJ	K11502
ENSG00000103194	-0.129642	6.697895	0.844937	1	USP10	K11841
ENSG00000230234	0.136552	4.104264	0.845016	1	RP1-276N6.2	_
ENSG00000224287	-0.135822	4.109469	0.845016	1	MSL3P1	K18403
ENSG00000131748	0.130077	5.408539	0.84507	1	STARD3	_

ENSG00000117748	-0.129857	5.734246	0.845103	1	RPA2	K10739
ENSG00000086666	0.129436	6.556934	0.845193	1	ZFAND6	K01555
ENSG00000136152	-0.135182	4.903557	0.845276	1	COG3	_
ENSG00000170949	-0.136146	4.339062	0.845403	1	ZNF160	_
ENSG00000160410	0.129468	6.185872	0.845433	1	SHKBP1	_
ENSG00000150457	0.12989	5.274385	0.845483	1	LATS2	K08791
ENSG00000164520	0.132633	5.03974	0.845489	1	RAET1E	_
ENSG00000178718	-0.129864	6.148009	0.845556	1	RPP25	K14525
ENSG00000177479	-0.129276	6.556512	0.8456	1	ARIH2	K17985
ENSG00000164118	-0.139914	4.033919	0.84565	1	CEP44	K16761
ENSG00000174744	-0.13173	5.890917	0.845799	1	BRMS1	K19196
ENSG00000168175	-0.128978	6.652001	0.8458	1	MAPK1IP1L	_
ENSG00000130513	-0.128726	6.336619	0.845812	1	GDF15	K05504
ENSG00000088298	0.133901	5.34949	0.845907	1	EDEM2	K10085
ENSG00000173436	-0.135307	4.272572	0.84596	1	MINOS1	K17784
ENSG00000172428	0.134606	4.406944	0.846118	1	MYEOV2	_
ENSG00000120334	-0.131929	4.477301	0.846186	1	CENPL	K11504
ENSG00000176340	-0.128487	6.722181	0.846277	1	COX8A	K02273
ENSG00000124299	0.129531	5.046402	0.846278	1	PEPD	K14213
ENSG00000100902	-0.130018	5.942432	0.846283	1	PSMA6	K02730
ENSG00000108239	0.136338	3.948155	0.846357	1	TBC1D12	K17985
ENSG00000197372	0.136338	3.948155	0.846357	1	ZNF675	K09228
ENSG00000102595	-0.128791	5.798658	0.846371	1	UGGT2	K11718
ENSG00000170919	0.147576	2.71931	0.846424	1	TPT1-AS1	_
ENSG00000186638	0.136085	4.033691	0.846437	1	KIF24	K10393
ENSG00000129460	-0.133099	5.321452	0.846515	1	NGDN	K14765
ENSG00000262944	-0.1504	2.925889	0.846609	1	RP11-473I1.9	_
ENSG00000139636	0.130144	4.274948	0.846627	1	LMBR1L	_
ENSG00000169018	-0.129889	6.179191	0.846668	1	FEM1B	K10349
ENSG00000182752	-0.13918	4.349874	0.846677	1	PAPPA	K07762
ENSG00000125657	0.12734	7.441147	0.846682	1	TNFSF9	K05472
ENSG00000170677	-0.129321	5.923782	0.84669	1	SOCS6	K10322
ENSG00000152990	-0.132168	5.173847	0.84675	1	GPR125	K08462
ENSG00000112679	0.137302	4.543023	0.846803	1	DUSP22	K14165
ENSG00000153071	0.127277	7.294839	0.846885	1	DAB2	K12475
ENSG00000102309	-0.135754	4.717835	0.8469	1	PIN4	K09579
ENSG00000183696	-0.129378	5.972061	0.846927	1	UPP1	K00757
ENSG00000073111	0.12688	7.798126	0.846969	1	MCM2	K02540
ENSG00000148229	-0.127252	7.205214	0.847136	1	POLE3	K02326
ENSG00000107438	-0.127183	7.23141	0.847191	1	PDLIM1	_
ENSG00000106290	0.128784	5.429056	0.847195	1	TAF6	K03131
ENSG00000128607	0.130691	5.493786	0.847215	1	KLHDC10	_
ENSG00000096070	0.140918	4.041655	0.847216	1	BRPF3	K11350
ENSG00000255529	-0.129222	5.527438	0.847225	1	POLR2M	_
ENSG00000109118	0.130504	5.556669	0.847234	1	PHF12	_
ENSG00000105835	0.12661	7.83453	0.847269	1	NAMPT	K03462
ENSG00000174292	-0.138491	4.283894	0.847288	1	TNK1	K08885
ENSG00000151422	-0.134046	4.35627	0.847306	1	FER	K08889
ENSG00000117475	-0.135103	4.666977	0.847377	1	BLZF1	_
ENSG00000065978	-0.125902	10.12611	0.847409	1	YBX1	K09276
ENSG00000164164	-0.13344	5.100371	0.847452	1	OTUD4	K13718
ENSG00000164985	-0.126328	8.199384	0.847472	1	PSIP1	_
ENSG00000197756	0.125943	9.292929	0.847488	1	RPL37A	K02921
ENSG00000180357	0.128505	5.528425	0.847504	1	ZNF609	K15696
ENSG00000160539	0.259601	0.781234	0.84754	1	PPAPDC3	_
ENSG00000185442	0.259601	0.781234	0.84754	1	FAM174B	_
ENSG00000223477	0.259601	0.781234	0.84754	1	LINC00842	_
ENSG00000241764	0.259601	0.781234	0.84754	1	AC002467.7	_

ENSG00000251196	0.259601	0.781234	0.84754	1	RP11-54F2.1	_
ENSG00000258708	0.259601	0.781234	0.84754	1	SLC25A21-AS1	K15110
ENSG00000272142	-0.232681	0.842792	0.84754	1	RP11-428J1.5	_
ENSG00000273311	-0.232681	0.842792	0.84754	1	DGCR11	_
ENSG00000250159	0.201109	0.810762	0.84754	1	RP11-381K20.2	_
ENSG00000264469	0.201109	0.810762	0.84754	1	RP11-173M1.8	_
ENSG00000127720	-0.182352	0.814313	0.84754	1	METTL25	_
ENSG00000171100	-0.182352	0.814313	0.84754	1	MTM1	K01108
ENSG00000186275	-0.182352	0.814313	0.84754	1	NBPF12	_
ENSG00000229645	-0.182352	0.814313	0.84754	1	LINC00341	_
ENSG00000238197	-0.182352	0.814313	0.84754	1	PAXBP1-AS1	_
ENSG00000259330	-0.182352	0.814313	0.84754	1	LINC00984	_
ENSG00000108298	0.125692	10.75845	0.847577	1	RPL19	K02885
ENSG00000182154	-0.127234	6.149251	0.847738	1	MRPL41	K17422
ENSG00000214113	-0.12787	5.885743	0.847755	1	LYRM4	_
ENSG00000070882	-0.128812	5.811982	0.847761	1	OSBPL3	_
ENSG00000158201	-0.129834	5.705976	0.847769	1	ABHD3	K13696
ENSG00000145365	0.128916	4.72497	0.847874	1	TIFA	_
ENSG00000249115	-0.134267	4.673858	0.847881	1	HAUS5	K16588
ENSG00000141556	0.127296	6.451066	0.847913	1	TBCD	K15522
ENSG00000157796	-0.133901	4.427258	0.847919	1	WDR19	K19671
ENSG00000100504	-0.126527	7.242165	0.847963	1	PYGL	K00688
ENSG00000104529	0.125502	9.487036	0.847974	1	EEF1D	K15410
ENSG00000139793	0.12652	7.101648	0.847979	1	MBNL2	K14943
ENSG00000173698	0.128277	5.437181	0.848088	1	GPR64	K08451
ENSG00000163694	0.128299	5.225965	0.848156	1	RBM47	K04613
ENSG00000101181	0.130697	5.187945	0.848169	1	MTG2	K03979
ENSG00000117650	0.126365	7.082996	0.848183	1	NEK2	K08857
ENSG00000138801	-0.127395	5.108005	0.848198	1	PAPSS1	K13811
ENSG00000078140	-0.126763	6.800164	0.848221	1	UBE2K	K04649
ENSG00000146830	-0.127174	6.233216	0.848249	1	GIGYF1	K18730
ENSG00000172336	-0.12712	6.213269	0.848257	1	POP7	K14527
ENSG00000154001	0.128045	5.708722	0.848261	1	PPP2R5E	K11584
ENSG00000164941	-0.126727	6.174958	0.848273	1	INTS8	K13145
ENSG00000221990	-0.16251	2.748333	0.848305	1	C5orf55	K17985
ENSG00000180776	0.15948	2.738791	0.848305	1	ZDHHC20	K18932
ENSG00000162032	0.154008	2.232932	0.848413	1	SPSB3	K10345
ENSG00000173141	-0.131021	5.375242	0.848418	1	MRP63	K17985
ENSG00000128039	0.132976	4.431565	0.84851	1	SRD5A3	K12345
ENSG00000143337	-0.126591	6.031569	0.848535	1	TOR1AIP1	_
ENSG00000072786	-0.131009	4.938269	0.848685	1	STK10	K08837
ENSG00000101624	-0.136898	4.142121	0.848693	1	CEP76	K16457
ENSG00000101220	-0.128657	5.716018	0.848749	1	C20orf27	_
ENSG00000173511	-0.130508	5.343208	0.848752	1	VEGFB	K16858
ENSG00000138346	-0.130252	4.790884	0.848794	1	DNA2	K10742
ENSG00000012061	-0.128094	5.631896	0.848815	1	ERCC1	K10849
ENSG00000112282	-0.136297	3.97377	0.848816	1	MED23	K15166
ENSG00000144231	-0.127784	5.847433	0.848878	1	POLR2D	K03012
ENSG00000161057	-0.126112	6.879365	0.848889	1	PSMC2	K03061
ENSG00000113638	0.125921	5.770314	0.848944	1	TTC33	_
ENSG00000175029	0.125777	6.981779	0.848997	1	CTBP2	K04496
ENSG00000179918	-0.126581	5.922342	0.84903	1	SEPHS2	K01008
ENSG00000181220	-0.136508	4.50462	0.849036	1	ZNF746	K09228
ENSG00000145833	0.125292	7.491263	0.84909	1	DDX46	K12811
ENSG00000131669	0.127464	5.797396	0.849151	1	NINJ1	_
ENSG00000104897	-0.128698	5.482068	0.849228	1	SF3A2	K12826
ENSG00000134533	0.138276	3.422426	0.849306	1	RERG	K07855
ENSG00000008853	-0.128852	5.578779	0.849398	1	RHOBTB2	K07868

ENSG00000141542	0.133843	4.144541	0.849407	1	RAB40B	K17985
ENSG00000108679	0.124278	9.384267	0.849463	1	LGALS3BP	K17300
ENSG00000196792	0.128085	4.569189	0.849514	1	STRN3	K17608
ENSG00000103591	-0.126116	6.467202	0.849522	1	AAGAB	_
ENSG00000092820	0.124433	8.103806	0.849719	1	EZR	K08007
ENSG00000197114	0.154145	2.952278	0.849863	1	ZGPAT	_
ENSG00000188827	0.142189	2.958242	0.849863	1	SLX4	K10484
ENSG00000169118	-0.139855	4.235859	0.849989	1	CSNK1G1	K08958
ENSG00000108469	-0.132401	4.902083	0.85	1	RECQL5	K10902
ENSG00000083535	-0.126957	5.355034	0.850001	1	PIBF1	K16538
ENSG00000140391	0.124273	7.747961	0.850121	1	TSPAN3	K17293
ENSG00000001461	-0.14646	2.767321	0.850141	1	NIPAL3	K04613
ENSG00000126767	0.126876	5.554802	0.850247	1	ELK1	K04375
ENSG00000155090	0.124645	7.05719	0.850266	1	KLF10	K09209
ENSG00000171109	-0.125675	6.3442	0.850275	1	MFN1	K06030
ENSG00000139641	0.124619	7.035278	0.850321	1	ESYT1	_
ENSG00000063438	0.125072	6.551783	0.850407	1	AHRR	K09094
ENSG00000108474	0.134066	3.981894	0.850412	1	PIGL	K03434
ENSG00000100359	-0.124784	6.254046	0.85044	1	SGSM3	_
ENSG00000130635	0.123677	8.455522	0.850462	1	COL5A1	K19721
ENSG00000159461	0.124123	6.234458	0.850476	1	AMFR	K10636
ENSG00000090686	0.125151	6.436441	0.850495	1	USP48	K11858
ENSG00000134899	-0.128849	4.855895	0.850515	1	ERCC5	K10846
ENSG00000159086	-0.12495	6.292577	0.850531	1	PAXBP1	K13211
ENSG00000130340	0.125123	6.402607	0.850585	1	SNX9	K17923
ENSG00000197312	-0.129063	5.002572	0.850647	1	DDI2	K11885
ENSG00000107937	0.123668	8.033694	0.850673	1	GTPBP4	K06943
ENSG00000166741	0.123627	7.909433	0.850792	1	NNMT	K00541
ENSG00000167925	-0.139141	4.164314	0.850816	1	GHDC	_
ENSG00000101052	0.132544	4.164265	0.850816	1	IFT52	K19681
ENSG00000110075	0.124966	6.370084	0.850827	1	PPP6R3	K15501
ENSG00000022277	-0.125088	6.386728	0.850894	1	RTFDC1	_
ENSG00000154727	-0.129126	5.048327	0.850939	1	GABPA	K09441
ENSG00000225137	0.170605	2.259966	0.850972	1	DYNC1I2P1	K10415
ENSG00000169062	-0.124925	6.406498	0.851052	1	UPF3A	K14328
ENSG00000158526	0.128887	5.216554	0.85127	1	TSR2	K14800
ENSG00000162458	0.135718	3.573821	0.851289	1	FBLIM1	K17985
ENSG00000010072	0.131911	4.247476	0.851315	1	SPRTN	_
ENSG00000056097	-0.123553	7.42162	0.851343	1	ZFR	K13203
ENSG00000196505	-0.131011	5.009397	0.85143	1	GDAP2	_
ENSG00000249565	-0.131011	5.009397	0.85143	1	SERBP1P5	K13199
ENSG00000049245	-0.124299	6.575496	0.851504	1	VAMP3	K13505
ENSG00000119772	-0.175657	1.949173	0.851626	1	DNMT3A	K17398
ENSG00000113384	0.12261	8.330774	0.851793	1	GOLPH3	K15620
ENSG00000150433	-0.150006	3.31322	0.851976	1	TMEM218	_
ENSG00000148834	-0.123487	6.915406	0.851977	1	GSTO1	K00799
ENSG00000151093	-0.139016	3.701293	0.85198	1	OXSM	K09458
ENSG00000126790	-0.132356	3.910997	0.852251	1	L3HYPDH	K18384
ENSG00000145919	-0.123462	6.737068	0.852253	1	BOD1	_
ENSG00000135763	-0.132588	4.716154	0.852273	1	URB2	K14862
ENSG00000125826	-0.124328	6.152958	0.852275	1	RBCK1	K10630
ENSG00000167487	-0.146694	3.58567	0.852323	1	KLHL26	K10463
ENSG00000183011	0.124075	5.952825	0.85233	1	LSMD1	_
ENSG00000132589	-0.12278	7.280183	0.852404	1	FLOT2	K07192
ENSG00000132646	-0.122266	8.05789	0.852413	1	PCNA	K04802
ENSG00000105552	-0.122881	6.154808	0.852451	1	BCAT2	K00826
ENSG00000112514	0.137253	4.094017	0.852457	1	CUTA	K03926
ENSG00000165480	-0.125822	5.696714	0.852495	1	SKA3	_

ENSG00000172164	-0.138423	3.81155	0.852565	1	SNTB1	_
ENSG00000166507	-0.193049	1.499661	0.852572	1	NDST2	K02577
ENSG00000260193	-0.193049	1.499661	0.852572	1	RP11-83N9.5	_
ENSG00000266903	-0.193049	1.499661	0.852572	1	CTB-171A8.1	_
ENSG00000111727	0.161444	2.549503	0.852577	1	HCFC2	K14966
ENSG00000135643	0.136043	3.454833	0.852725	1	KCNMB4	K04941
ENSG00000163918	-0.122614	7.105003	0.85279	1	RFC4	K10755
ENSG00000116857	0.122521	7.070954	0.852791	1	TMEM9	_
ENSG00000155329	-0.125875	4.828378	0.852831	1	ZCCHC10	_
ENSG00000176105	0.1223	7.27427	0.852854	1	YES1	K05705
ENSG00000107854	0.123912	6.276953	0.852891	1	TNKS2	K10799
ENSG00000106100	-0.13095	3.711254	0.852925	1	NOD1	K08727
ENSG00000103043	-0.124947	5.235635	0.852938	1	VAC14	K15305
ENSG00000163001	0.125353	5.484995	0.85296	1	CCDC104	K04613
ENSG00000133195	-0.141516	2.99331	0.852982	1	SLC39A11	K14717
ENSG00000139990	0.129878	4.479579	0.853079	1	DCAF5	K11800
ENSG00000170871	0.125843	4.605703	0.853153	1	KIAA0232	_
ENSG00000122545	-0.122044	7.412138	0.853157	1	7-Sep	K16944
ENSG00000141956	0.127797	5.156307	0.853229	1	PRDM15	_
ENSG00000159479	-0.123541	6.223873	0.853265	1	MED8	K15129
ENSG00000117523	0.122015	7.188124	0.853277	1	PRRC2C	_
ENSG00000114166	0.127121	3.592262	0.853342	1	KAT2B	K06062
ENSG00000100395	-0.122741	5.647723	0.853432	1	L3MBTL2	K17985
ENSG00000168137	0.123483	6.121767	0.853484	1	SETD5	_
ENSG00000164754	0.120898	9.385887	0.853514	1	RAD21	K06670
ENSG00000121542	-0.13555	4.193325	0.853558	1	SEC22A	K08520
ENSG00000177337	0.133627	3.710825	0.853859	1	DLGAP1-AS1	_
ENSG00000162396	0.126647	3.714271	0.853859	1	PARS2	K01881
ENSG00000147536	0.12595	4.109461	0.853889	1	GINS4	K10735
ENSG00000125347	0.125237	4.674332	0.853911	1	IRF1	K09444
ENSG00000171606	-0.124218	4.78769	0.853914	1	ZNF274	K12458
ENSG00000134278	0.128096	4.88945	0.853916	1	SPIRE1	K02098
ENSG00000136870	-0.124696	4.89176	0.853916	1	ZNF189	K09228
ENSG00000103326	0.12203	6.634483	0.853916	1	CAPN15	K08582
ENSG00000134283	-0.122097	6.704662	0.853932	1	PPHLN1	_
ENSG00000214309	0.243873	0.880319	0.85396	1	MBLAC1	_
ENSG00000257576	0.243873	0.880319	0.85396	1	RP11-153M3.1	K04077
ENSG00000268362	0.243873	0.880319	0.85396	1	CTD-2017D11.1	_
ENSG00000160688	-0.122354	6.465231	0.854014	1	FLAD1	K06641
ENSG00000196591	-0.120833	8.24809	0.854026	1	HDAC2	K06067
ENSG00000167315	-0.122394	6.391965	0.854099	1	ACAA2	K07508
ENSG00000176208	0.124902	4.941795	0.854118	1	ATAD5	_
ENSG00000143319	-0.122649	5.768428	0.854153	1	ISG20L2	K18329
ENSG00000183337	0.121587	6.842259	0.85417	1	BCOR	_
ENSG00000149269	-0.122563	5.466291	0.854204	1	PAK1	K04409
ENSG00000165264	-0.124881	5.564958	0.854317	1	NDUFB6	K03962
ENSG00000081665	-0.334757	-0.186789	0.854437	1	ZNF506	K09228
ENSG00000130997	-0.334757	-0.186789	0.854437	1	POLN	K16618
ENSG00000142621	-0.334757	-0.186789	0.854437	1	FHAD1	K12581
ENSG00000158023	-0.334757	-0.186789	0.854437	1	WDR66	_
ENSG00000170425	-0.334757	-0.186789	0.854437	1	ADORA2B	K04267
ENSG00000181722	-0.334757	-0.186789	0.854437	1	ZBTB20	_
ENSG00000185245	-0.334757	-0.186789	0.854437	1	GP1BA	K06261
ENSG00000228933	-0.334757	-0.186789	0.854437	1	RP11-268G12.1	_
ENSG00000256304	-0.334757	-0.186789	0.854437	1	RP11-512M8.3	K10421
ENSG00000260219	-0.334757	-0.186789	0.854437	1	RP11-347C12.10	_
ENSG00000261801	-0.334757	-0.186789	0.854437	1	LOXL1-AS1	_
ENSG00000082196	0.297395	-0.296143	0.854437	1	C1QTNF3	_

ENSG00000169203	0.297395	-0.296143	0.854437	1	RP11-231C14.4	_
ENSG00000178301	0.297395	-0.296143	0.854437	1	AQP11	K09870
ENSG00000179899	0.297395	-0.296143	0.854437	1	PHC1P1	K11456
ENSG00000184348	0.297395	-0.296143	0.854437	1	HIST1H2AK	K11251
ENSG00000213228	0.297395	-0.296143	0.854437	1	RPL12P38	K02870
ENSG00000225335	0.297395	-0.296143	0.854437	1	XXbac-B476C20.9	_
ENSG00000228925	0.297395	-0.296143	0.854437	1	AC016722.4	_
ENSG00000232480	0.297395	-0.296143	0.854437	1	RP11-224O19.2	_
ENSG00000246889	0.297395	-0.296143	0.854437	1	AP000487.5	_
ENSG00000252419	0.297395	-0.296143	0.854437	1	RMRPP2	_
ENSG00000261662	0.297395	-0.296143	0.854437	1	RP5-1042I8.7	_
ENSG00000263934	0.297395	-0.296143	0.854437	1	SNORD3A	_
ENSG00000272509	0.297395	-0.296143	0.854437	1	RP11-347C18.5	_
ENSG00000116667	-0.134206	4.281607	0.854529	1	C1orf21	_
ENSG00000149182	-0.122509	6.090655	0.854564	1	ARFGAP2	K12493
ENSG00000198695	0.119944	9.864819	0.854571	1	MT-ND6	K03884
ENSG00000133226	0.121423	6.645042	0.854628	1	SRRM1	K13171
ENSG00000139132	-0.135701	3.72796	0.854781	1	FGD4	K05723
ENSG00000101665	0.127831	4.794613	0.854801	1	SMAD7	K19631
ENSG00000136518	-0.121194	6.828615	0.854833	1	ACTL6A	K11340
ENSG00000156110	-0.121113	6.843195	0.85491	1	ADK	K00856
ENSG00000157450	0.121621	4.948925	0.854915	1	RNF111	K10635
ENSG00000152127	0.124374	5.172221	0.854946	1	MGAT5	K00744
ENSG00000136856	-0.125704	4.56321	0.854948	1	SLC2A8	K08145
ENSG00000107651	-0.120869	6.11679	0.854999	1	SEC23IP	_
ENSG00000104299	0.136898	3.182652	0.855112	1	INTS9	K13146
ENSG00000111450	-0.123357	4.903519	0.855152	1	STX2	K08486
ENSG00000239306	0.120564	5.750883	0.855189	1	RBM14	_
ENSG00000039650	-0.122329	5.258713	0.855213	1	PNKP	K08073
ENSG00000198841	-0.129151	4.500708	0.855263	1	KTI12	K15456
ENSG00000150991	0.119396	9.351226	0.855323	1	UBC	K08770
ENSG00000105379	0.121319	6.282593	0.85536	1	ETFB	K03521
ENSG00000198208	-0.139892	2.817778	0.855393	1	RPS6KL1	K12606
ENSG00000119684	-0.128248	3.945504	0.855468	1	MLH3	K08739
ENSG00000171823	-0.128248	3.945504	0.855468	1	FBXL14	K10280
ENSG00000099246	0.120452	6.848741	0.855519	1	RAB18	K07910
ENSG00000104369	0.123791	5.47812	0.855586	1	JPH1	K19530
ENSG00000139173	0.135244	3.342573	0.855599	1	TMEM117	_
ENSG00000143537	-0.119513	8.043856	0.85572	1	ADAM15	K06836
ENSG00000107742	0.148185	2.305872	0.85584	1	SPOCK2	K08136
ENSG00000119522	0.123661	5.264152	0.85585	1	DENND1A	_
ENSG00000110955	-0.11871	10.73013	0.855965	1	ATP5B	K02133
ENSG00000111790	-0.120834	5.551003	0.856036	1	FGFR1OP2	_
ENSG00000239305	0.127054	4.807209	0.856112	1	RNF103	K15695
ENSG00000254635	-0.125208	5.012102	0.856214	1	WAC-AS1	_
ENSG00000155313	-0.121121	6.234978	0.856315	1	USP25	K11849
ENSG00000147439	0.132805	3.617413	0.856318	1	BIN3	_
ENSG00000142230	-0.119061	7.918118	0.85634	1	SAE1	K10684
ENSG00000087502	0.119942	6.671107	0.856361	1	ERGIC2	_
ENSG00000130244	-0.135474	3.207706	0.856418	1	FAM98C	_
ENSG00000197713	-0.120543	6.153583	0.856453	1	RPE	K01783
ENSG00000060749	-0.120411	6.390927	0.856468	1	QSER1	_
ENSG00000114209	-0.12028	6.438702	0.856538	1	PDCD10	K18269
ENSG00000104671	0.121308	5.553867	0.856554	1	DCTN6	K10428
ENSG00000263535	0.143323	2.587063	0.856566	1	AK4P1	K00939
ENSG00000119242	0.127921	2.594846	0.856566	1	CCDC92	K16452
ENSG00000196497	0.127921	2.594846	0.856566	1	IPO4	_
ENSG00000184007	0.118428	8.610036	0.856695	1	PTP4A2	K18041

ENSG00000260032	0.118587	7.959978	0.856801	1	LINC00657	_
ENSG00000153936	0.119761	6.495004	0.856837	1	HS2ST1	K02513
ENSG00000164323	-0.12031	6.199635	0.856879	1	KIAA1430	_
ENSG00000162402	0.11939	6.77011	0.856889	1	USP24	K11840
ENSG00000137819	0.12111	5.65225	0.856997	1	PAQR5	_
ENSG00000113971	-0.132312	3.959707	0.857024	1	NPHP3	K19360
ENSG00000054793	0.120258	4.870101	0.857074	1	ATP9A	K01530
ENSG00000049618	0.126925	4.646563	0.857103	1	ARID1B	K11653
ENSG00000006744	-0.11907	6.961522	0.8572	1	ELAC2	K00784
ENSG00000067560	0.11797	8.697612	0.857213	1	RHOA	K04513
ENSG00000135930	-0.119375	6.322476	0.857259	1	EIF4E2	K03259
ENSG00000101442	0.131873	3.631655	0.857284	1	ACTR5	K11672
ENSG00000153066	0.12092	5.685883	0.857289	1	TXNDC11	K09228
ENSG00000024048	-0.121942	5.716025	0.857338	1	UBR2	K10626
ENSG00000123353	0.120928	5.715412	0.857338	1	ORMDL2	_
ENSG00000072274	-0.11779	9.017622	0.857369	1	TFRC	K06503
ENSG00000121775	-0.126461	4.523742	0.857382	1	TMEM39B	_
ENSG00000160299	0.119974	6.225092	0.857407	1	PCNT	K16481
ENSG00000092531	-0.119683	5.627475	0.857428	1	SNAP23	K08508
ENSG00000122550	-0.119683	5.627475	0.857428	1	KLHL7	K10445
ENSG00000119328	0.122952	4.92601	0.857563	1	FAM206A	K11373
ENSG00000088247	-0.118127	7.746887	0.857576	1	KHSRP	K13210
ENSG00000023909	-0.120165	5.396204	0.857594	1	GCLM	K11205
ENSG00000197417	-0.190107	2.005599	0.85762	1	SHPK	K11214
ENSG00000178458	0.152131	2.000963	0.85762	1	H3F3AP6	K11253
ENSG00000187764	0.152131	2.000963	0.85762	1	SEMA4D	K06521
ENSG00000167114	-0.121939	5.282557	0.857728	1	SLC27A4	K08745
ENSG00000169609	0.129696	3.962373	0.85779	1	C15orf40	K09131
ENSG00000138303	-0.122654	5.632783	0.857915	1	ASCC1	K18666
ENSG00000184779	-0.125119	4.156832	0.858024	1	RPS17	K02962
ENSG00000164032	-0.117357	8.556505	0.858052	1	H2AFZ	K11251
ENSG00000138439	-0.132775	3.514331	0.858093	1	FAM117B	_
ENSG00000155657	0.164065	2.331611	0.858156	1	TTN	K12567
ENSG00000160602	0.145489	2.341	0.858156	1	NEK8	K08857
ENSG00000165521	0.145489	2.341	0.858156	1	EML5	K18597
ENSG00000136271	-0.118028	7.146451	0.858232	1	DDX56	K14810
ENSG00000161671	0.119208	6.061274	0.858378	1	EMC10	_
ENSG00000224183	-0.151555	2.625407	0.85848	1	SDHDP6	K00237
ENSG00000170606	-0.116822	8.760184	0.858616	1	HSPA4	K09489
ENSG00000197530	-0.124945	4.781331	0.858623	1	MIB2	K10645
ENSG00000136141	-0.129731	4.073409	0.858624	1	LRCH1	_
ENSG00000141449	-0.129731	4.073409	0.858624	1	GREB1L	_
ENSG00000164610	-0.129808	4.159331	0.858691	1	RP9	K19604
ENSG00000137714	-0.120013	6.042264	0.858721	1	FDX1	K12022
ENSG00000127483	0.117702	6.898128	0.858751	1	HP1BP3	_
ENSG00000106603	-0.118499	6.369888	0.858791	1	COA1	K18173
ENSG00000140479	-0.133162	3.226518	0.858962	1	PCSK6	K08672
ENSG00000134077	-0.118485	5.373121	0.858993	1	THUMPD3	_
ENSG00000122707	-0.120797	5.482068	0.859045	1	RECK	K17461
ENSG00000172775	-0.117264	6.114902	0.859113	1	FAM192A	_
ENSG00000133250	0.123879	3.521497	0.85912	1	ZNF414	_
ENSG00000155380	0.116429	8.469165	0.859146	1	SLC16A1	K08179
ENSG00000244754	-0.118991	6.243687	0.859164	1	N4BP2L2	_
ENSG00000102781	-0.137604	3.656778	0.859177	1	KATNAL1	K07767
ENSG00000138796	-0.12001	5.82055	0.859186	1	HADH	K00022
ENSG00000125449	-0.123856	3.881453	0.859263	1	ARMC7	_
ENSG00000100461	-0.120553	5.448333	0.859302	1	RBM23	K13091
ENSG00000166165	0.116028	9.149331	0.859412	1	CKB	K00933

ENSG00000169660	-0.123857	4.546415	0.859441	1	HEXDC	K14459
ENSG00000101138	-0.120927	5.740007	0.859601	1	CSTF1	K14406
ENSG00000141378	0.117357	6.430323	0.859814	1	PTRH2	K04794
ENSG00000167543	-0.118895	5.682281	0.859827	1	TP53I13	K13707
ENSG00000229927	-0.259195	0.962689	0.859853	1	RHEBP1	K07208
ENSG00000231066	-0.259195	0.962689	0.859853	1	NPM1P9	K11276
ENSG00000188107	-0.213635	0.936686	0.859853	1	EYS	K19601
ENSG00000250539	-0.213635	0.936686	0.859853	1	KRT8P33	K07605
ENSG00000157045	0.121667	4.734923	0.859921	1	NTAN1	K14662
ENSG00000183763	-0.127709	4.258837	0.859992	1	TRAIP	K11985
ENSG00000198833	-0.117091	5.744924	0.860045	1	UBE2J1	K10578
ENSG00000179029	0.12262	3.887359	0.860058	1	TMEM107	_
ENSG00000111799	-0.115892	7.893277	0.860156	1	COL12A1	K08132
ENSG00000129158	-0.13891	3.398645	0.860164	1	SERGEF	K15421
ENSG00000197766	0.116681	6.699237	0.860224	1	CFD	K01334
ENSG00000104756	0.11865	5.097303	0.860225	1	KCTD9	_
ENSG00000043514	0.117775	5.829375	0.860235	1	TRIT1	K00791
ENSG00000168246	0.121295	4.954597	0.860276	1	UBTD2	_
ENSG00000185909	0.14922	2.864815	0.860291	1	KLHDC8B	_
ENSG00000251432	-0.133774	2.866539	0.860291	1	RP11-420A23.1	_
ENSG00000146425	0.119068	5.625833	0.860313	1	DYNLT1	K10420
ENSG00000169951	-0.134236	2.646039	0.860345	1	ZNF764	K09228
ENSG00000175567	-0.116908	6.335025	0.860378	1	UCP2	K15103
ENSG00000250251	-0.172282	2.370076	0.8604	1	PKD1P6	K04985
ENSG00000109519	-0.117547	6.23557	0.860425	1	GRPEL1	K03687
ENSG00000143294	0.116757	6.496452	0.860427	1	PRCC	K13105
ENSG00000116688	0.116498	6.702611	0.860437	1	MFN2	K06030
ENSG00000111012	-0.162343	2.03682	0.86044	1	CYP27B1	K07438
ENSG00000179115	-0.115966	7.377001	0.86047	1	FARSA	K01889
ENSG00000116350	-0.118094	6.107291	0.860491	1	SRSF4	K12893
ENSG00000132199	0.117644	5.914043	0.860601	1	ENOSF1	K18334
ENSG00000167747	-0.115853	7.254897	0.860724	1	C19orf48	_
ENSG00000229117	-0.115294	8.094791	0.860751	1	RPL41	K02928
ENSG00000095066	0.120797	5.31354	0.860752	1	HOOK2	K16611
ENSG00000128011	-0.122463	4.74582	0.860803	1	LRFN1	K16354
ENSG00000133316	-0.117321	5.599724	0.860814	1	WDR74	K14841
ENSG00000138073	0.117286	5.780718	0.860901	1	PREB	K14003
ENSG00000156384	0.128574	3.789146	0.860926	1	SFR1	K17985
ENSG00000085721	-0.119253	5.725171	0.860949	1	RRN3	K15216
ENSG00000105669	0.115844	6.91894	0.860951	1	COPE	K17268
ENSG00000029725	-0.115985	6.903771	0.860966	1	RABEP1	K12480
ENSG00000137040	-0.116879	5.944591	0.860969	1	RANBP6	_
ENSG00000100697	-0.12234	4.963751	0.861033	1	DICER1	K11592
ENSG00000188010	-0.129137	3.544645	0.86113	1	MORN2	_
ENSG00000166199	0.121271	4.270481	0.861212	1	ALKBH3	K10860
ENSG00000178467	0.121271	4.270481	0.861212	1	P4HTM	K06711
ENSG00000112335	0.11536	7.211079	0.861225	1	SNX3	K17918
ENSG00000100483	-0.128948	3.410882	0.861261	1	VCPKMT	_
ENSG00000107295	0.11798	5.062275	0.861301	1	SH3GL2	K11247
ENSG00000172057	0.12065	4.965875	0.861408	1	ORMDL3	_
ENSG00000158863	0.124697	4.563652	0.861442	1	FAM160B2	_
ENSG00000070047	0.118279	5.434142	0.861493	1	PHRF1	K17586
ENSG00000160613	0.116187	5.435157	0.861493	1	PCSK7	K08673
ENSG00000164609	0.116421	6.247203	0.861517	1	SLU7	K12819
ENSG00000164877	-0.121695	5.157958	0.861535	1	MICALL2	_
ENSG00000106591	-0.116161	6.361923	0.8616	1	MRPL32	K02911
ENSG00000101199	0.115029	7.228445	0.861604	1	ARFGAP1	K12492
ENSG00000196196	-0.140865	3.084006	0.86161	1	HRCT1	_

ENSG00000066248	-0.126778	3.908014	0.861621	1	NGEF	K07525
ENSG00000123131	0.114387	8.402507	0.861624	1	PRDX4	K03386
ENSG00000151923	-0.115381	6.943132	0.86164	1	TIAL1	K13201
ENSG00000104881	0.116937	5.844726	0.861677	1	PPP1R13L	K17985
ENSG00000125944	-0.114469	8.178574	0.861695	1	HNRNPR	K13161
ENSG00000105438	0.114479	7.944007	0.861739	1	KDELR1	K10949
ENSG00000262758	-0.126459	3.805136	0.861762	1	CTD-3195I5.1	_
ENSG00000048991	0.117973	5.402333	0.861815	1	R3HDM1	K05054
ENSG00000184232	0.116386	5.674755	0.861923	1	OAF	_
ENSG00000140259	0.115349	6.558284	0.862017	1	MFAP1	K13110
ENSG00000171241	-0.116443	6.122474	0.86202	1	SHCBP1	_
ENSG00000231991	0.115148	6.680103	0.862084	1	ANXA2P2	K17092
ENSG00000166974	-0.147761	2.653451	0.862161	1	MAPRE2	K10436
ENSG00000152556	-0.114446	7.509201	0.862175	1	PFKM	K00850
ENSG00000196839	0.114325	6.078898	0.862266	1	ADA	K01488
ENSG00000122873	-0.115061	6.715156	0.862332	1	CISD1	_
ENSG00000215301	-0.113563	8.936772	0.862471	1	DDX3X	K11594
ENSG00000184381	0.122265	3.262827	0.862614	1	PLA2G6	K16343
ENSG00000184445	0.11467	6.639747	0.862712	1	KNTC1	_
ENSG00000110721	-0.120379	4.876904	0.862724	1	CHKA	K14156
ENSG00000121152	-0.1154	6.108568	0.862735	1	NCAPH	K06676
ENSG00000143977	-0.117209	4.984655	0.862889	1	SNRPG	K11099
ENSG00000169884	-0.11939	4.022957	0.86293	1	WNT10B	K01357
ENSG00000129351	0.113084	8.722416	0.863072	1	ILF3	K13090
ENSG00000121073	-0.117103	5.861086	0.86309	1	SLC35B1	K15275
ENSG00000107077	0.125103	4.205344	0.863189	1	KDM4C	K06709
ENSG00000117682	0.119813	4.373572	0.863392	1	DHDDS	K11778
ENSG00000124380	-0.115722	5.52839	0.863446	1	SNRNP27	K12846
ENSG00000090372	-0.1143	6.556564	0.863486	1	STRN4	K17608
ENSG00000138760	0.113141	7.625698	0.863546	1	SCARB2	K12384
ENSG00000127540	-0.113597	7.093535	0.863599	1	UQCR11	K00420
ENSG00000148737	-0.118079	3.70436	0.863697	1	TCF7L2	K04491
ENSG00000107140	-0.118839	4.943941	0.863752	1	TESK1	K08841
ENSG00000124203	0.115584	5.812295	0.863838	1	ZNF831	_
ENSG00000140859	-0.124103	3.931158	0.863902	1	KIFC3	K10406
ENSG00000159214	0.137214	2.672259	0.86393	1	CCDC24	K05038
ENSG00000197385	0.137214	2.672259	0.86393	1	ZNF860	_
ENSG00000103202	0.113304	6.939809	0.86397	1	NME4	K00940
ENSG00000106077	-0.201436	1.633698	0.863995	1	ABHD11	K13703
ENSG00000165181	-0.173513	1.617951	0.863995	1	C9orf84	_
ENSG00000186162	0.158618	1.614023	0.863995	1	CIDECP	_
ENSG00000215154	0.158618	1.614023	0.863995	1	AC141586.5	_
ENSG00000117859	0.11306	7.114312	0.864076	1	OSBPL9	_
ENSG00000189091	-0.112568	7.640223	0.864323	1	SF3B3	K12830
ENSG00000180817	0.111955	8.469386	0.86452	1	PPA1	K01507
ENSG00000100116	-0.115728	5.144379	0.86457	1	GCAT	K00639
ENSG00000168374	-0.112236	7.839006	0.864579	1	ARF4	K07939
ENSG00000087111	0.113405	6.367395	0.864645	1	PIGS	K05291
ENSG00000240087	0.140478	2.408782	0.864681	1	RP11-254B13.1	K02998
ENSG00000175073	0.113997	5.982258	0.864698	1	VCPIP1	K11861
ENSG00000140181	-0.115645	5.190722	0.864742	1	HERC2P2	K10595
ENSG00000183735	0.116149	5.358087	0.864778	1	TBK1	K05410
ENSG00000109775	-0.121536	4.463393	0.864782	1	UFSP2	_
ENSG00000133142	-0.11295	6.76599	0.864787	1	TCEAL4	_
ENSG00000198554	-0.114711	5.70515	0.86484	1	WDHD1	K11274
ENSG00000103423	-0.112715	6.835573	0.864973	1	DNAJA3	K09504
ENSG00000173041	-0.122053	4.226371	0.86503	1	ZNF680	K09228
ENSG00000163162	-0.113019	6.54206	0.865044	1	RNF149	K15704

ENSG00000165156	-0.112989	6.200846	0.865139	1	ZHX1	_
ENSG00000169692	0.112457	6.795191	0.865158	1	AGPAT2	K13509
ENSG00000164465	0.114066	5.475164	0.865186	1	DCBLD1	_
ENSG00000137817	-0.116933	4.73596	0.865261	1	PARP6	K15258
ENSG00000047579	-0.113787	4.734301	0.865261	1	DTNBP1	_
ENSG00000272750	-0.19709	1.024885	0.865284	1	RP11-378J18.8	_
ENSG00000106976	0.179911	0.998629	0.865284	1	DNM1	K01528
ENSG00000206145	0.179911	0.998629	0.865284	1	P2RX6P	K05221
ENSG00000170266	0.112297	6.015192	0.865326	1	GLB1	K12309
ENSG00000166860	-0.121522	4.144458	0.865347	1	ZBTB39	K10511
ENSG00000125676	0.112038	7.032209	0.865387	1	THOC2	K12879
ENSG00000157764	-0.116682	4.542616	0.86553	1	BRAF	K04365
ENSG00000196387	-0.135741	3.124679	0.865556	1	ZNF140	K09228
ENSG00000101197	0.149665	2.692365	0.865655	1	BIRC7	K16061
ENSG00000181638	0.149665	2.692365	0.865655	1	ZFP41	_
ENSG00000167733	-0.176418	2.08996	0.865756	1	HSD11B1L	_
ENSG00000169627	-0.176418	2.08996	0.865756	1	BOLA2B	_
ENSG00000178401	-0.176418	2.08996	0.865756	1	DNAJC22	K19370
ENSG00000166435	0.167739	2.074916	0.865756	1	XRRA1	_
ENSG00000262879	-0.156287	2.078738	0.865756	1	RP11-156P1.3	_
ENSG00000167447	-0.114488	5.33018	0.865767	1	SMG8	K18734
ENSG00000155275	0.119554	3.596014	0.865915	1	TRMT44	K15447
ENSG00000092853	-0.119626	4.680642	0.865934	1	CLSPN	_
ENSG00000119771	0.12452	3.953964	0.866109	1	KLHL29	K10465
ENSG00000144824	-0.110917	7.768797	0.866211	1	PHLDB2	_
ENSG00000085514	0.381678	-0.15322	0.866239	1	PILRA	K15411
ENSG00000142619	0.381678	-0.15322	0.866239	1	PADI3	K01481
ENSG00000172404	0.381678	-0.15322	0.866239	1	DNAJB7	K09513
ENSG00000230439	0.381678	-0.15322	0.866239	1	RP11-488P3.1	_
ENSG00000234424	0.381678	-0.15322	0.866239	1	RP11-213G2.1	K10395
ENSG00000247679	0.381678	-0.15322	0.866239	1	RP11-1277A3.1	_
ENSG00000264919	0.381678	-0.15322	0.866239	1	MIR4697	_
ENSG00000272078	0.381678	-0.15322	0.866239	1	RP4-734G22.3	_
ENSG00000272744	0.381678	-0.15322	0.866239	1	RP11-367N14.3	_
ENSG00000102174	0.257959	-0.094796	0.866239	1	PHEX	K08636
ENSG00000120784	0.257959	-0.094796	0.866239	1	ZFP30	K09228
ENSG00000135740	0.257959	-0.094796	0.866239	1	SLC9A5	K14723
ENSG00000138080	0.257959	-0.094796	0.866239	1	EMILIN1	_
ENSG00000163959	0.257959	-0.094796	0.866239	1	SLC51A	K17985
ENSG00000164651	0.257959	-0.094796	0.866239	1	SP8	K09198
ENSG00000164845	0.257959	-0.094796	0.866239	1	FAM86FP	K12354
ENSG00000177788	0.257959	-0.094796	0.866239	1	RP5-1061H20.4	_
ENSG00000178104	0.257959	-0.094796	0.866239	1	PDE4DIP	K16549
ENSG00000196747	0.257959	-0.094796	0.866239	1	HIST1H2AI	K11251
ENSG00000198785	0.257959	-0.094796	0.866239	1	GRIN3A	K05213
ENSG00000213621	0.257959	-0.094796	0.866239	1	RPSAP54	K02998
ENSG00000253854	0.257959	-0.094796	0.866239	1	RP11-10N23.2	_
ENSG00000272046	0.257959	-0.094796	0.866239	1	RP11-471M2.3	_
ENSG00000272288	0.257959	-0.094796	0.866239	1	RP11-140K17.3	_
ENSG00000272702	0.257959	-0.094796	0.866239	1	RP11-44N22.3	_
ENSG00000146757	0.125832	3.727591	0.866273	1	ZNF92	K09228
ENSG00000176692	-0.121499	3.734364	0.866273	1	FOXC2	K09396
ENSG00000104613	0.11139	6.861968	0.866355	1	INTS10	K13147
ENSG00000168538	0.11524	5.375103	0.866433	1	TRAPPC11	_
ENSG00000064419	-0.113533	6.05288	0.866441	1	TNPO3	K15436
ENSG00000139613	0.111768	6.05203	0.866441	1	SMARCC2	K11649
ENSG00000108671	-0.111326	6.892028	0.866563	1	PSMD11	K03036
ENSG00000081320	-0.115273	4.408676	0.866643	1	STK17B	K08804

ENSG00000131508	-0.110885	7.178911	0.866759	1	UBE2D2	K06689
ENSG00000165244	0.11104	5.900139	0.866785	1	ZNF367	K09228
ENSG00000225630	0.111076	5.928513	0.866846	1	MTND2P28	K03879
ENSG00000164975	-0.113528	5.457339	0.866879	1	SNAPC3	K15210
ENSG00000005810	-0.115352	5.215708	0.8669	1	MYCBP2	K10693
ENSG00000003096	0.11754	4.749695	0.866949	1	KLHL13	K10447
ENSG00000170759	-0.110049	8.189	0.866999	1	KIF5B	K10396
ENSG00000166477	0.112037	6.264988	0.867073	1	LEO1	K15177
ENSG00000062194	-0.110933	6.837881	0.867105	1	GPBP1	_
ENSG00000175793	-0.110372	7.395109	0.867163	1	SFN	K06644
ENSG00000112640	0.110863	6.697103	0.867195	1	PPP2R5D	K11584
ENSG00000224546	0.119169	3.31671	0.8672	1	EIF4BP3	K03258
ENSG00000070950	-0.117494	5.081044	0.867246	1	RAD18	K10627
ENSG00000087274	0.111004	5.986411	0.867318	1	ADD1	_
ENSG00000076003	0.110262	7.218146	0.867333	1	MCM6	K02542
ENSG00000142507	-0.11052	7.002472	0.867394	1	PSMB6	K02738
ENSG00000173914	-0.118944	4.254178	0.86741	1	RBM4B	K13187
ENSG00000187650	0.184105	1.654901	0.867417	1	VMAC	K17985
ENSG00000223478	0.184105	1.654901	0.867417	1	RP11-545E17.3	_
ENSG00000197587	-0.164932	1.673673	0.867417	1	DMBX1	K11447
ENSG00000231711	-0.164932	1.673673	0.867417	1	LINC00899	_
ENSG00000131791	0.153839	1.670369	0.867417	1	PRKAB2	K07199
ENSG00000215447	0.153839	1.670369	0.867417	1	BX322557.10	_
ENSG00000054118	0.110371	6.983795	0.867439	1	THRAP3	K13112
ENSG00000100412	0.11093	6.395284	0.867561	1	ACO2	K01681
ENSG00000118579	-0.114423	5.133179	0.867658	1	MED28	K15141
ENSG00000144645	0.113024	5.70619	0.867798	1	OSBPL10	_
ENSG00000169021	-0.10978	7.46971	0.867809	1	UQCRFS1	K00411
ENSG00000234518	-0.12628	3.750807	0.867937	1	PTGES3P1	K15730
ENSG00000100897	-0.113866	5.184706	0.868041	1	DCAF11	K11801
ENSG00000132405	0.117778	3.866515	0.868104	1	TBC1D14	_
ENSG00000197535	-0.116608	4.99018	0.868105	1	MYO5A	K10357
ENSG00000175215	0.109579	7.183546	0.868186	1	CTDSP2	K15731
ENSG00000101546	-0.117414	4.427147	0.868211	1	RBFA	_
ENSG00000197150	-0.111701	5.314842	0.868214	1	ABCB8	K05655
ENSG00000010244	-0.109427	7.347731	0.868342	1	ZNF207	_
ENSG00000106771	0.109442	7.097413	0.868435	1	TMEM245	_
ENSG00000099956	-0.110222	6.485957	0.868488	1	SMARCB1	K11648
ENSG00000174652	0.113946	5.359157	0.868537	1	ZNF266	K09228
ENSG00000188735	-0.116502	4.770095	0.868596	1	TMEM120B	_
ENSG00000174996	-0.111479	5.514073	0.868687	1	KLC2	K10407
ENSG00000152217	-0.142464	2.459514	0.868708	1	SETBP1	_
ENSG00000170242	0.109219	6.978929	0.868826	1	USP47	K11857
ENSG00000130717	0.113385	5.049092	0.868846	1	UCK1	K00876
ENSG00000060971	-0.112693	4.57973	0.868852	1	ACAA1	K07513
ENSG00000180879	-0.123068	3.990269	0.868943	1	SSR4	K04571
ENSG00000188177	-0.137236	2.73447	0.868977	1	ZC3H6	K12581
ENSG00000169752	0.133523	2.726376	0.868977	1	NRG4	K05458
ENSG00000183741	-0.109978	6.369913	0.868984	1	CBX6	K11453
ENSG00000156928	-0.109774	6.471212	0.869049	1	MALSU1	_
ENSG00000103507	-0.111643	5.980449	0.869054	1	BCKDK	K00905
ENSG00000128059	-0.111349	5.721029	0.869074	1	PPAT	K00764
ENSG00000124198	-0.11172	6.057498	0.869105	1	ARFGEF2	K18442
ENSG00000182986	-0.116937	4.272423	0.869141	1	ZNF320	K09228
ENSG00000082996	-0.109499	5.754707	0.8693	1	RNF13	K15692
ENSG00000203485	-0.108661	7.26323	0.869343	1	INF2	_
ENSG00000181588	-0.112429	5.369941	0.869346	1	MEX3D	K15686
ENSG00000198740	0.115874	4.895399	0.869453	1	ZNF652	_

ENSG00000116459	-0.107985	8.232217	0.869458	1	ATP5F1	K02127
ENSG00000102910	-0.114617	5.20029	0.869561	1	LONP2	K01338
ENSG00000172725	-0.10929	6.485471	0.869605	1	CORO1B	K13886
ENSG00000135441	-0.115651	4.443345	0.869744	1	BLOC1S1	_
ENSG00000196465	-0.109765	6.113654	0.869777	1	MYL6B	K12751
ENSG00000092330	0.112486	4.720057	0.869795	1	TINF2	K11112
ENSG00000141030	-0.1082	7.23383	0.869926	1	COPS3	K12177
ENSG00000007968	0.110313	5.160023	0.870164	1	E2F2	K09389
ENSG00000175203	0.107976	7.032862	0.87026	1	DCTN2	K10424
ENSG00000160161	0.218309	1.060075	0.870304	1	CILP2	_
ENSG00000176714	0.218309	1.060075	0.870304	1	CCDC121	K06883
ENSG00000260686	0.218309	1.060075	0.870304	1	CTB-36H16.2	_
ENSG00000270102	0.218309	1.060075	0.870304	1	RP11-498E2.8	_
ENSG00000119547	-0.122507	3.893287	0.870335	1	ONECUT2	_
ENSG00000204237	-0.109749	5.637201	0.870347	1	OXLD1	_
ENSG00000106266	0.109504	5.602537	0.870354	1	SNX8	K17922
ENSG00000173681	-0.126809	3.356228	0.870444	1	CXorf23	K17985
ENSG00000078319	-0.128582	3.183627	0.870482	1	PMS2P1	K10858
ENSG00000177943	-0.130882	2.987527	0.870527	1	MAMDC4	_
ENSG00000245556	-0.120169	2.981718	0.870527	1	CTD-2037K23.2	_
ENSG00000113712	0.107811	6.96459	0.870532	1	CSNK1A1	K08957
ENSG00000172465	0.112206	4.850492	0.870576	1	TCEAL1	_
ENSG00000250067	-0.133987	2.760502	0.870578	1	YJEFN3	K11353
ENSG00000117543	-0.108595	6.393572	0.870597	1	DPH5	K00586
ENSG00000135605	-0.138409	2.490936	0.870633	1	TEC	K07364
ENSG00000154146	-0.138409	2.490936	0.870633	1	NRGN	_
ENSG00000228638	-0.138409	2.490936	0.870633	1	FCF1P2	K14566
ENSG00000148341	0.108153	6.552661	0.870645	1	SH3GLB2	K11248
ENSG00000100320	-0.108131	6.700928	0.870657	1	RBFOX2	K14946
ENSG00000185808	0.111704	4.598552	0.870679	1	PIGP	K03861
ENSG00000176387	-0.14521	2.159113	0.870682	1	HSD11B2	_
ENSG00000214300	0.142368	2.12708	0.870682	1	SPDYE3	K08694
ENSG00000145088	-0.125888	2.148444	0.870682	1	EAF2	K15186
ENSG00000048649	0.109549	6.14796	0.870717	1	RSF1	K11657
ENSG00000147224	0.107118	7.703722	0.870727	1	PRPS1	K00948
ENSG00000100528	-0.108146	6.570949	0.870836	1	CNIH1	_
ENSG00000260359	0.11577	4.200662	0.870876	1	RP11-4F5.2	_
ENSG00000099624	-0.10709	7.629002	0.870912	1	ATP5D	K02134
ENSG00000163527	0.106742	8.072099	0.870966	1	STT3B	K07151
ENSG00000171858	0.106573	8.435789	0.871004	1	RPS21	K02971
ENSG00000142856	-0.108241	6.386779	0.871033	1	ITGB3BP	K11510
ENSG00000198382	0.109828	5.169808	0.87108	1	UVRAG	_
ENSG00000131171	0.116092	3.782952	0.871144	1	SH3BGRL	_
ENSG00000103994	-0.107957	6.45981	0.871246	1	ZNF106	_
ENSG00000106524	0.108879	5.577946	0.871283	1	ANKMY2	_
ENSG00000119333	-0.107068	7.198459	0.871321	1	WDR34	_
ENSG00000118640	-0.108916	5.952413	0.871354	1	VAMP8	K08512
ENSG00000213445	0.108481	5.952089	0.871354	1	SIPA1	K08013
ENSG00000076513	0.11209	5.173457	0.871382	1	ANKRD13A	_
ENSG00000156482	0.105932	10.04848	0.871391	1	RPL30	K02908
ENSG00000122376	-0.107587	6.032947	0.87145	1	FAM35A	_
ENSG00000136717	0.107003	6.996207	0.871468	1	BIN1	K12562
ENSG00000046604	-0.10678	7.37889	0.871493	1	DSG2	K07597
ENSG00000175970	-0.110055	5.309324	0.871539	1	UNC119B	_
ENSG00000065559	-0.110046	5.509269	0.871586	1	MAP2K4	K04430
ENSG00000154945	0.107908	5.395056	0.871714	1	ANKRD40	K09488
ENSG00000111237	-0.106805	7.012029	0.871842	1	VPS29	K18467
ENSG00000110060	-0.114394	3.798691	0.871922	1	PUS3	K01855

ENSG00000134759	0.107246	6.384809	0.871993	1	ELP2	K11374
ENSG00000135390	-0.105909	8.059788	0.872048	1	ATP5G2	K02128
ENSG00000146676	0.106996	6.322028	0.872158	1	PURB	K11251
ENSG00000135269	0.106344	7.067288	0.872184	1	TES	_
ENSG00000171456	0.106695	6.677502	0.87222	1	ASXL1	K11471
ENSG00000198920	0.111405	4.981263	0.872267	1	KIAA0753	_
ENSG00000138326	0.105115	10.83653	0.872296	1	RPS24	K02974
ENSG00000197885	0.114544	4.392534	0.872339	1	NKIRAS1	K17197
ENSG00000135932	-0.108033	6.259822	0.872397	1	CAB39	K08272
ENSG00000070214	0.106421	6.788832	0.872406	1	SLC44A1	_
ENSG00000047410	-0.105619	7.960865	0.872454	1	TPR	K09291
ENSG00000154767	-0.111123	5.233288	0.872499	1	XPC	K10838
ENSG00000105717	0.133785	2.50482	0.872503	1	PBX4	K15611
ENSG00000114737	0.133785	2.50482	0.872503	1	CISH	K04701
ENSG00000198252	-0.115867	4.550116	0.872573	1	STYX	K18042
ENSG00000135052	-0.105625	7.599027	0.872696	1	GOLM1	_
ENSG00000164078	-0.108557	5.280289	0.872704	1	MST1R	K05100
ENSG00000164626	0.12561	3.206849	0.872812	1	KCNK5	K04916
ENSG00000162521	0.105658	7.253349	0.87283	1	RBBP4	K10752
ENSG00000178074	-0.111573	4.62484	0.872891	1	C2orf69	_
ENSG00000139645	-0.105965	6.972439	0.872897	1	ANKRD52	K15504
ENSG00000110048	0.106145	6.595027	0.872994	1	OSBP	_
ENSG00000100348	-0.106225	6.310965	0.873011	1	TXN2	K03671
ENSG00000132773	0.111167	5.238826	0.873071	1	TOE1	K13202
ENSG00000133639	-0.106304	6.552394	0.873075	1	BTG1	K14443
ENSG00000110090	0.108735	5.886849	0.873093	1	CPT1A	K08765
ENSG00000130303	-0.104734	8.795606	0.873137	1	BST2	K06731
ENSG00000099992	0.114591	3.925489	0.873196	1	TBC1D10A	_
ENSG00000135506	0.10518	7.439016	0.873249	1	OS9	K10088
ENSG00000165782	0.110592	4.821308	0.873302	1	TMEM55B	K13084
ENSG00000163807	-0.110466	4.826778	0.873302	1	KIAA1143	K17985
ENSG00000160223	-0.10716	4.56125	0.873485	1	ICOSLG	K06710
ENSG00000161904	-0.106542	6.082305	0.873522	1	LEMD2	_
ENSG00000184602	0.115381	3.385554	0.873533	1	SNN	_
ENSG00000018510	0.105878	6.413914	0.873586	1	AGPS	K00803
ENSG00000168496	0.104395	8.317113	0.873675	1	FEN1	K04799
ENSG00000104365	-0.109853	5.104152	0.87369	1	IKBKB	K07209
ENSG00000136758	-0.104636	7.818804	0.873727	1	YME1L1	K08955
ENSG00000135720	-0.107459	5.92454	0.873745	1	DYNC1LI2	K10416
ENSG00000100058	0.109911	4.634335	0.873755	1	CRYBB2P1	K00543
ENSG00000166024	0.118587	4.235993	0.873756	1	R3HCC1L	_
ENSG00000006652	0.104868	7.270498	0.873765	1	IFRD1	_
ENSG00000133433	-0.182934	1.741872	0.873769	1	GSTT2B	K00799
ENSG00000227008	-0.182934	1.741872	0.873769	1	RP3-417G15.1	K02974
ENSG00000167815	0.104441	7.991278	0.873776	1	PRDX2	K03386
ENSG00000108312	-0.104866	7.355535	0.873814	1	UBTF	K09273
ENSG00000151694	0.106851	4.767568	0.873833	1	ADAM17	K06059
ENSG00000125691	0.103907	9.490747	0.873918	1	RPL23	K02894
ENSG00000102858	-0.106895	5.107964	0.874	1	MGRN1	K10604
ENSG00000134297	0.122094	3.551768	0.874102	1	PLEKHA8P1	_
ENSG00000218739	-0.105326	6.60783	0.87416	1	CEBPZ-AS1	K14832
ENSG00000067365	-0.111726	3.823642	0.8742	1	METTL22	_
ENSG00000100109	0.110093	4.892467	0.874256	1	TFIP11	K13103
ENSG00000147996	-0.149303	2.529808	0.874319	1	CBWD5	_
ENSG00000114391	-0.103636	9.168002	0.874348	1	RPL24	K02896
ENSG00000163597	0.108777	5.54167	0.874392	1	SNHG16	_
ENSG00000166710	0.103328	11.35762	0.874417	1	B2M	K08055
ENSG00000135521	-0.106397	5.753085	0.874621	1	LTV1	K14798

ENSG00000110107	-0.103833	7.815115	0.874696	1	PRPF19	K10599
ENSG00000177683	-0.106033	5.34858	0.874749	1	THAP5	_
ENSG00000116991	0.106521	4.841358	0.874797	1	SIPA1L2	K17702
ENSG00000134318	0.104287	6.91744	0.874817	1	ROCK2	K17388
ENSG00000115145	-0.10918	4.577877	0.874828	1	STAM2	K04705
ENSG00000100425	0.107725	5.213038	0.874903	1	BRD1	K11349
ENSG00000105323	0.10356	7.841885	0.874921	1	HNRNPUL1	K15047
ENSG00000117480	-0.223035	1.130975	0.87496	1	FAAH	K15528
ENSG00000151651	-0.223035	1.130975	0.87496	1	ADAM8	K06540
ENSG00000185803	-0.223035	1.130975	0.87496	1	SLC52A2	_
ENSG00000245146	-0.223035	1.130975	0.87496	1	LINC01024	_
ENSG00000167005	-0.104216	6.975933	0.874994	1	NUDT21	K14397
ENSG00000227372	0.108232	4.15713	0.875181	1	TP73-AS1	_
ENSG00000230311	-0.158705	2.207979	0.87526	1	AC004074.3	K17770
ENSG00000143845	0.157159	2.195027	0.87526	1	ETNK2	K00894
ENSG00000056558	0.136744	2.205406	0.87526	1	TRAF1	K03172
ENSG00000224051	-0.108132	4.907858	0.87532	1	GLTPD1	_
ENSG00000173327	0.103707	6.024641	0.875359	1	MAP3K11	K04419
ENSG00000214223	-0.103873	5.762001	0.875406	1	HNRNPA1P10	K12741
ENSG00000125753	-0.103957	6.861473	0.875447	1	VASP	K06274
ENSG00000111481	-0.103087	8.017631	0.875468	1	COPZ1	K05867
ENSG00000160256	0.10583	5.885361	0.875522	1	FAM207A	_
ENSG00000100796	-0.105438	6.106047	0.875551	1	SMEK1	K17491
ENSG00000174371	0.10369	6.881792	0.875575	1	EXO1	K10746
ENSG00000175611	-0.11299	3.046675	0.875731	1	LINC00476	_
ENSG00000229119	0.107759	4.166937	0.875766	1	CTB-63M22.1	K02941
ENSG00000138029	-0.103979	6.230944	0.87583	1	HADHB	K07509
ENSG00000160194	-0.106499	5.948163	0.87583	1	NDUFV3	K03944
ENSG00000176102	0.105232	5.798945	0.875865	1	CSTF3	K14408
ENSG00000108406	0.104171	6.184	0.87587	1	DHX40	K18711
ENSG00000156171	0.103867	6.479791	0.875896	1	DRAM2	_
ENSG00000224631	0.10273	7.772538	0.875962	1	RP11-51O6.1	K02977
ENSG00000166886	-0.107052	5.081022	0.876028	1	NAB2	_
ENSG00000196436	-0.13082	2.551809	0.876084	1	NPIPB15	_
ENSG00000006712	0.103485	6.302138	0.87611	1	PAF1	K15174
ENSG00000111605	-0.103182	7.048265	0.876152	1	CPSF6	K14398
ENSG00000029534	-0.371702	0.050164	0.876186	1	ANK1	K10380
ENSG00000119737	-0.371702	0.050164	0.876186	1	GPR75	K08417
ENSG00000139865	-0.371702	0.050164	0.876186	1	TTC6	_
ENSG00000150667	-0.371702	0.050164	0.876186	1	FSIP1	_
ENSG00000167555	-0.371702	0.050164	0.876186	1	ZNF528	K09228
ENSG00000184923	-0.371702	0.050164	0.876186	1	NUTM2A	_
ENSG00000262691	-0.371702	0.050164	0.876186	1	CTC-277H1.7	_
ENSG00000266040	-0.371702	0.050164	0.876186	1	CTB-58E17.3	_
ENSG00000272768	-0.371702	0.050164	0.876186	1	RP4-673M15.1	_
ENSG00000172426	0.334575	0.030691	0.876186	1	RSPH9	K19757
ENSG00000186792	0.334575	0.030691	0.876186	1	HYAL3	K01197
ENSG00000226403	0.334575	0.030691	0.876186	1	RP11-31F19.1	_
ENSG00000231731	0.334575	0.030691	0.876186	1	AC010976.2	_
ENSG00000232457	0.334575	0.030691	0.876186	1	SLC16A6P1	K08183
ENSG00000271789	0.334575	0.030691	0.876186	1	RP5-1112D6.7	_
ENSG00000272087	0.334575	0.030691	0.876186	1	RP11-379F4.7	_
ENSG00000100385	-0.279593	-0.001446	0.876186	1	IL2RB	K05069
ENSG00000162620	-0.279593	-0.001446	0.876186	1	LRRIQ3	_
ENSG00000162814	-0.279593	-0.001446	0.876186	1	SPATA17	_
ENSG00000166317	-0.279593	-0.001446	0.876186	1	SYNPO2L	K17985
ENSG00000182368	-0.279593	-0.001446	0.876186	1	FAM27A	_
ENSG00000186715	-0.279593	-0.001446	0.876186	1	MST1L	_

ENSG00000231240	-0.279593	-0.001446	0.876186	1	KLF2P1	K09537
ENSG00000236404	-0.279593	-0.001446	0.876186	1	RP11-125B21.2	_
ENSG00000259684	-0.279593	-0.001446	0.876186	1	RP11-120K9.2	_
ENSG00000260349	-0.279593	-0.001446	0.876186	1	RP11-473I1.5	_
ENSG00000260805	-0.279593	-0.001446	0.876186	1	RP11-61J19.4	_
ENSG00000260879	-0.279593	-0.001446	0.876186	1	RP11-483I13.5	_
ENSG00000262888	-0.279593	-0.001446	0.876186	1	RP11-462G12.2	_
ENSG00000181038	0.104057	5.562244	0.876195	1	METTL23	_
ENSG00000151092	0.106861	5.180742	0.876225	1	NGLY1	K01456
ENSG00000105607	-0.107217	4.666842	0.876279	1	GCDH	K00252
ENSG00000125741	-0.103906	4.6651	0.876279	1	OPA3	_
ENSG00000173269	0.112307	4.174205	0.876345	1	MMRN2	_
ENSG00000159217	0.103439	6.444959	0.876463	1	IGF2BP1	K17391
ENSG00000121940	-0.106604	4.979077	0.876467	1	CLCC1	_
ENSG00000141279	0.102533	7.245838	0.876595	1	NPEPPS	K08776
ENSG00000059145	0.108203	4.66925	0.87669	1	UNKL	_
ENSG00000137845	-0.102982	6.801043	0.876697	1	ADAM10	K06704
ENSG00000164104	-0.102145	7.836185	0.876714	1	HMGB2	K11295
ENSG00000182179	-0.149696	1.779092	0.876722	1	UBA7	K10698
ENSG00000189190	-0.149696	1.779092	0.876722	1	ZNF600	_
ENSG00000087338	0.10422	5.237672	0.87687	1	GMCL1	K10485
ENSG00000073350	0.103628	6.196018	0.876878	1	LLGL2	K06094
ENSG00000128245	-0.102653	6.963348	0.876887	1	YWHAH	K16198
ENSG00000196976	0.112786	3.721138	0.876943	1	LAGE3	K15902
ENSG00000213080	0.124874	3.05154	0.876967	1	RP11-312J18.5	K00802
ENSG00000087152	0.10369	5.932098	0.876977	1	ATXN7L3	K11363
ENSG00000111328	-0.103273	6.373109	0.877014	1	CDK2AP1	_
ENSG00000167840	-0.107292	4.276875	0.877048	1	ZNF232	K09230
ENSG00000067334	-0.10191	7.636538	0.877137	1	DNTTIP2	_
ENSG00000100934	-0.102765	6.660354	0.877155	1	SEC23A	K14006
ENSG00000142552	0.102633	6.612608	0.877185	1	RCN3	_
ENSG00000178338	-0.119902	3.258672	0.877231	1	ZNF354B	K09228
ENSG00000182957	-0.119902	3.258672	0.877231	1	SPATA13	K05769
ENSG00000124593	-0.113653	3.973518	0.877259	1	PRICKLE4	K17772
ENSG00000105053	0.107627	4.6076	0.877431	1	VRK3	K08816
ENSG00000163463	-0.105484	4.990151	0.877454	1	KRTCAP2	_
ENSG00000015153	-0.102898	5.29152	0.877468	1	YAF2	K11468
ENSG00000240230	-0.105821	4.680576	0.877503	1	COX19	K18183
ENSG00000162512	0.104138	5.338709	0.877769	1	SDC3	K16337
ENSG00000176953	-0.104646	5.383651	0.877804	1	NFATC2IP	_
ENSG00000156970	0.101657	7.069556	0.877815	1	BUB1B	K06637
ENSG00000164366	-0.10433	5.724356	0.877843	1	CCDC127	K16467
ENSG00000114395	0.106407	4.089176	0.877986	1	CYB561D2	K08371
ENSG00000154217	0.104482	5.468244	0.8781	1	PITPNC1	_
ENSG00000138031	0.103049	5.946398	0.878163	1	ADCY3	K08043
ENSG00000066427	-0.130893	3.078591	0.878179	1	ATXN3	K11863
ENSG00000272711	-0.120851	3.073155	0.878179	1	RP11-259N19.1	_
ENSG00000132478	-0.104147	5.301559	0.878259	1	UNK	_
ENSG00000058673	-0.101028	7.557071	0.87826	1	ZC3H11A	_
ENSG00000164074	-0.117871	3.276841	0.878288	1	C4orf29	K12307
ENSG00000134910	0.101116	7.224865	0.878318	1	STT3A	K07151
ENSG00000134202	-0.101477	6.946393	0.878321	1	GSTM3	K00799
ENSG00000151718	0.101817	6.43586	0.878424	1	WWC2	_
ENSG00000092036	-0.107	5.002515	0.878427	1	HAUS4	K16587
ENSG00000084072	-0.102136	6.341028	0.878436	1	PPIE	K09564
ENSG00000102699	-0.10331	6.007971	0.878515	1	PARP4	K10798
ENSG00000073008	-0.101833	6.471238	0.878572	1	PVR	K06539
ENSG00000159111	0.104605	5.590748	0.878593	1	MRPL10	K02864

ENSG00000104518	-0.109065	4.387844	0.878649	1	GSDMD	_
ENSG00000189060	0.107336	4.888037	0.878732	1	H1F0	K11275
ENSG00000100151	0.103552	5.351662	0.878785	1	PICK1	_
ENSG00000146587	0.104176	5.517926	0.8788	1	RBAK	K09228
ENSG00000089693	0.100063	8.347445	0.87888	1	MLF2	_
ENSG00000074603	0.102317	5.559455	0.879021	1	DPP8	K08655
ENSG00000117399	-0.100281	7.73242	0.879029	1	CDC20	K03363
ENSG00000177954	0.099669	9.387917	0.879047	1	RPS27	K02978
ENSG00000051825	0.104729	5.063617	0.879061	1	MPHOSPH9	K16775
ENSG00000118707	0.107278	4.950387	0.879072	1	TGIF2	K19553
ENSG00000172331	-0.107922	4.553808	0.879116	1	BPGM	K01837
ENSG00000100485	-0.104565	4.303739	0.879149	1	SOS2	K03099
ENSG00000186625	0.105928	4.205513	0.879162	1	KATNA1	K07767
ENSG00000167705	0.110945	3.88156	0.879214	1	RILP	K13883
ENSG00000181274	-0.100861	5.484027	0.879256	1	FRAT2	K03096
ENSG00000075618	-0.099606	8.906303	0.87928	1	FSCN1	K17455
ENSG00000078399	0.163615	1.164769	0.87929	1	HOXA9	K09294
ENSG00000161940	0.163615	1.164769	0.87929	1	BCL6B	_
ENSG00000167711	0.163615	1.164769	0.87929	1	SERPINF2	K03983
ENSG00000173013	0.163615	1.164769	0.87929	1	CCDC96	_
ENSG00000229833	0.163615	1.164769	0.87929	1	PET100	K18186
ENSG00000231064	0.163615	1.164769	0.87929	1	RP11-263K19.4	_
ENSG00000234769	0.163615	1.164769	0.87929	1	WASH4P	K18461
ENSG00000251669	0.163615	1.164769	0.87929	1	FAM86EP	_
ENSG00000185619	0.105077	5.218944	0.879323	1	PCGF3	K11488
ENSG00000168255	-0.122108	2.860194	0.879416	1	POLR2J3	K03008
ENSG00000125995	0.100704	5.638209	0.879429	1	ROMO1	_
ENSG00000151135	-0.10021	7.308493	0.879458	1	C12orf23	K15031
ENSG00000100478	-0.142903	1.829066	0.879539	1	AP4S1	K12403
ENSG00000035928	0.100547	6.720137	0.879547	1	RFC1	K10754
ENSG00000101871	-0.102134	5.712703	0.8796	1	MID1	K08285
ENSG00000176624	0.102323	5.173473	0.87962	1	MEX3C	K15686
ENSG00000184216	0.110233	4.394652	0.879621	1	IRAK1	K04730
ENSG00000124786	-0.10536	5.123135	0.879644	1	SLC35B3	K15277
ENSG00000121680	0.104104	4.957474	0.87973	1	PEX16	K13335
ENSG00000053501	-0.10443	4.114237	0.879765	1	USE1	K08507
ENSG00000123159	0.100139	6.89511	0.879827	1	GIPC1	_
ENSG00000197976	0.10026	6.325295	0.879879	1	AKAP17A	K13169
ENSG00000124217	-0.111154	3.887237	0.879898	1	MOCS3	K11996
ENSG00000131931	-0.111154	3.887237	0.879898	1	THAP1	_
ENSG00000125520	-0.100191	5.997723	0.879962	1	SLC2A4RG	_
ENSG00000087157	0.104251	5.074076	0.879973	1	PGS1	K10408
ENSG00000196531	-0.098856	9.667174	0.880001	1	NACA	K03626
ENSG00000146410	-0.103372	4.402388	0.880101	1	MTFR2	_
ENSG00000164978	-0.10443	5.22978	0.880141	1	NUDT2	K01518
ENSG00000186660	0.099788	6.971216	0.880165	1	ZFP91	_
ENSG00000130787	0.10184	5.941407	0.88017	1	HIP1R	K04559
ENSG00000154358	0.100262	6.494939	0.880201	1	OBSCN	K17531
ENSG00000138495	0.106259	4.710062	0.880268	1	COX17	K02260
ENSG00000141002	0.099947	6.569362	0.880471	1	TCF25	_
ENSG00000138246	-0.102744	5.723528	0.880568	1	DNAJC13	K09533
ENSG00000184924	0.101288	4.407176	0.880578	1	PTRHD1	_
ENSG00000181924	-0.100203	6.404506	0.880643	1	COA4	_
ENSG00000156973	0.105946	4.716753	0.880653	1	PDE6D	K13758
ENSG00000101421	-0.099381	7.053241	0.880717	1	CHMP4B	K12194
ENSG00000181004	-0.11939	2.884082	0.880773	1	BBS12	K19402
ENSG00000124228	-0.100304	6.314842	0.880922	1	DDX27	K13181
ENSG00000067113	-0.113065	4.129428	0.880922	1	PPAP2A	K01080

ENSG00000119537	-0.10037	6.065398	0.880991	1	KDSR	K04708
ENSG00000108294	-0.098626	7.675029	0.881062	1	PSMB3	K02735
ENSG00000132801	-0.123855	2.610232	0.881095	1	ZSWIM3	K17604
ENSG00000119599	0.118491	3.478946	0.881097	1	DCAF4	K11799
ENSG00000124172	0.098272	8.207648	0.8811	1	ATP5E	K02135
ENSG00000158109	0.102831	4.85508	0.881204	1	TPRG1L	_
ENSG00000159131	-0.098467	7.712295	0.881228	1	GART	K11787
ENSG00000132781	-0.103076	3.907733	0.881243	1	MUTYH	K03575
ENSG00000164068	0.104618	4.233781	0.881326	1	RNF123	K12169
ENSG00000189266	-0.113962	3.31251	0.881353	1	PNRC2	K18751
ENSG00000141741	0.101552	5.698596	0.881389	1	MIEN1	K07401
ENSG00000198763	-0.097576	10.93147	0.881392	1	MT-ND2	K03879
ENSG00000117984	0.098342	7.48134	0.881444	1	CTSD	K01379
ENSG00000172197	-0.107222	4.136868	0.881492	1	MBOAT1	K13517
ENSG00000175416	0.098948	6.677902	0.881524	1	CLTB	K04645
ENSG00000144597	0.105293	4.657143	0.881537	1	EAF1	K15186
ENSG00000102886	0.151021	2.269865	0.881549	1	GDPD3	_
ENSG00000182196	0.151021	2.269865	0.881549	1	ARL6IP4	_
ENSG00000119396	-0.098763	6.899442	0.881641	1	RAB14	K07881
ENSG00000159363	-0.104919	4.583349	0.881671	1	ATP13A2	K13526
ENSG00000223768	-0.116267	3.114144	0.881677	1	LINC00205	_
ENSG00000144476	0.109866	3.642569	0.881687	1	ACKR3	K04304
ENSG00000129245	0.098925	6.571666	0.881696	1	FXR2	K15516
ENSG00000230202	0.0979	7.867632	0.881723	1	RP11-632C17__A.1	K02905
ENSG00000114942	-0.097529	8.51385	0.881924	1	EEF1B2	K03232
ENSG00000170921	0.101858	5.389836	0.881973	1	TANC2	_
ENSG00000077152	-0.098796	6.581547	0.882042	1	UBE2T	K13960
ENSG00000112787	-0.099212	5.809677	0.882124	1	FBRSL1	_
ENSG00000114062	0.098766	6.411317	0.882126	1	UBE3A	K10587
ENSG00000166170	0.10015	6.105874	0.882129	1	BAG5	K09559
ENSG00000109686	-0.101171	5.520773	0.882162	1	SH3D19	_
ENSG00000198522	-0.101171	5.520773	0.882162	1	GPN1	K06883
ENSG00000110536	-0.100049	6.13504	0.882215	1	PTPMT1	K14165
ENSG00000258472	0.115155	1.840865	0.88223	1	RP11-192H23.4	K17529
ENSG00000130640	0.099231	6.1864	0.88224	1	TUBGCP2	K16569
ENSG00000116212	-0.098462	6.713549	0.882249	1	LRRC42	_
ENSG00000156172	-0.112079	3.33002	0.882341	1	C8orf37	K17985
ENSG00000086504	-0.097922	7.161929	0.882356	1	MRPL28	K02902
ENSG00000156795	-0.105694	4.247159	0.882379	1	WDYHV1	_
ENSG00000198836	-0.098036	6.836016	0.882597	1	OPA1	K17079
ENSG00000143621	-0.09679	8.993788	0.88265	1	ILF2	K13089
ENSG00000074621	-0.106799	2.631086	0.882677	1	SLC24A1	K13749
ENSG00000196418	-0.106799	2.631086	0.882677	1	ZNF124	K09228
ENSG00000133895	0.1025	5.054409	0.882767	1	MEN1	K14970
ENSG00000106853	0.097175	7.521768	0.88282	1	PTGR1	K13948
ENSG00000196810	-0.11799	3.506387	0.882849	1	CTBP1-AS2	_
ENSG00000185420	0.109546	3.498638	0.882849	1	SMYD3	K11426
ENSG00000115687	0.103772	4.252324	0.882898	1	PASK	K08801
ENSG00000060491	0.097867	6.597266	0.882932	1	OGFR	K08131
ENSG00000146670	0.097722	6.686869	0.882987	1	CDCA5	K17390
ENSG00000177410	0.097313	7.095756	0.883016	1	ZFAS1	_
ENSG00000163848	0.097606	6.196601	0.88306	1	ZNF148	_
ENSG00000136942	-0.096307	9.449544	0.88312	1	RPL35	K02918
ENSG00000141524	-0.108504	4.351795	0.883143	1	TMC6	K02355
ENSG00000120451	0.103916	4.348064	0.883143	1	SNX19	K17930
ENSG00000196247	0.108621	3.928443	0.883205	1	ZNF107	K09228
ENSG00000178397	0.101769	4.879138	0.88325	1	FAM220A	_
ENSG00000042445	0.09784	6.383205	0.883283	1	RETSAT	K09516

ENSG00000138162	0.098462	5.649615	0.883318	1	TACC2	K14282
ENSG00000141140	0.198421	1.21982	0.883327	1	MYO19	_
ENSG00000176024	-0.158345	1.26131	0.883327	1	ZNF613	_
ENSG00000235636	-0.158345	1.26131	0.883327	1	NUS1P1	K19177
ENSG00000188599	0.156803	1.241159	0.883327	1	NPIPP1	_
ENSG00000149806	-0.096828	7.542117	0.883329	1	FAU	K02983
ENSG00000168883	0.097306	6.797894	0.883348	1	USP39	K12847
ENSG00000052841	-0.097607	6.631925	0.883395	1	TTC17	_
ENSG00000161265	0.122535	2.901207	0.883398	1	U2AF1L4	K12836
ENSG00000260671	0.122535	2.901207	0.883398	1	RP11-278A23.2	_
ENSG00000212694	-0.116761	2.907583	0.883398	1	AC084018.1	_
ENSG00000213189	-0.116761	2.907583	0.883398	1	BTF3L4P2	K01527
ENSG00000246695	-0.116761	2.907583	0.883398	1	RASSF8-AS1	_
ENSG00000103245	0.099811	5.116439	0.883489	1	NARFL	_
ENSG00000005700	-0.097294	6.827552	0.8835	1	IBTK	_
ENSG00000203772	-0.126446	2.307636	0.883507	1	SPRN	_
ENSG00000163481	-0.102975	4.527409	0.88355	1	RNF25	K10640
ENSG00000168653	-0.096854	7.242593	0.883561	1	NDUFS5	K03938
ENSG00000089335	-0.107029	3.808016	0.883576	1	ZNF302	K09228
ENSG00000143418	0.096562	7.439227	0.883621	1	CERS2	K04710
ENSG00000171988	0.096998	5.954901	0.883646	1	JMJD1C	K11449
ENSG00000185049	0.102035	5.064934	0.883658	1	NELFA	K15179
ENSG00000120509	-0.099866	5.322521	0.883665	1	PDZD11	_
ENSG00000160310	-0.10295	5.121872	0.883773	1	PRMT2	K11435
ENSG00000101654	0.097267	5.498646	0.8838	1	RNMT	K00565
ENSG00000112983	-0.097982	6.304348	0.883807	1	BRD8	K11321
ENSG00000003056	-0.097348	6.527931	0.883866	1	M6PR	K10089
ENSG00000125356	-0.096886	6.856214	0.883953	1	NDUFA1	K03945
ENSG00000197852	0.107899	4.057893	0.884071	1	FAM212B	_
ENSG00000159128	-0.110242	3.347322	0.884268	1	IFNGR2	K05133
ENSG00000260708	-0.110242	3.347322	0.884268	1	CTA-29F11.1	_
ENSG00000115216	0.096683	6.648456	0.884286	1	NRBP1	K08875
ENSG00000105778	-0.101033	5.377336	0.884474	1	AVL9	K14404
ENSG00000079482	-0.110631	3.948145	0.884478	1	OPHN1	_
ENSG00000169087	-0.110631	3.948145	0.884478	1	HSPBAP1	K19375
ENSG00000108296	-0.100367	4.834443	0.884478	1	CWC25	_
ENSG00000100294	0.101578	5.075383	0.884534	1	MCAT	K00645
ENSG00000130816	-0.09594	7.341863	0.884569	1	DNMT1	K00558
ENSG00000117500	-0.096385	6.812425	0.884613	1	TMED5	K14825
ENSG00000123415	-0.099065	5.187092	0.884691	1	SMUG1	K10800
ENSG00000109511	-0.238666	0.162897	0.884696	1	ANXA10	K17097
ENSG00000213433	-0.238666	0.162897	0.884696	1	RP11-54C4.1	K02942
ENSG00000234694	-0.238666	0.162897	0.884696	1	RP1-92O14.3	_
ENSG00000272449	-0.238666	0.162897	0.884696	1	RP3-395M20.12	_
ENSG00000105887	0.096996	6.05662	0.88472	1	MTPN	_
ENSG00000196547	-0.101278	5.23793	0.884761	1	MAN2A2	K01231
ENSG00000123505	-0.09547	7.800093	0.884779	1	AMD1	K01611
ENSG00000215271	-0.103249	4.176162	0.884802	1	HOMEZ	_
ENSG00000249641	-0.159977	1.890397	0.884803	1	HOXC13-AS	_
ENSG00000137075	-0.105092	4.544429	0.884814	1	RNF38	K19041
ENSG00000167880	-0.099627	5.631913	0.884829	1	EVPL	K10383
ENSG00000176871	0.098561	5.8447	0.884915	1	WSB2	K10342
ENSG00000204859	0.102544	4.279697	0.884931	1	ZBTB48	K10519
ENSG00000150760	0.099217	5.189196	0.884959	1	DOCK1	K13708
ENSG00000083799	0.099945	4.699998	0.885005	1	CYLD	K08601
ENSG00000111775	0.095074	8.094842	0.885009	1	COX6A1	K02266
ENSG00000072110	-0.095681	7.153035	0.885064	1	ACTN1	K05699
ENSG00000115307	-0.095827	6.975968	0.885082	1	AUP1	K19716

ENSG00000010539	-0.099825	4.774839	0.885087	1	ZNF200	K09228
ENSG00000132485	-0.094915	8.476816	0.885092	1	ZRANB2	_
ENSG00000159692	0.096015	6.598165	0.885157	1	CTBP1	K04496
ENSG00000125304	-0.095161	7.764855	0.885175	1	TM9SF2	K17086
ENSG00000184785	-0.09556	5.781268	0.885185	1	SMIM10	_
ENSG00000104852	0.094889	8.195257	0.885186	1	SNRNP70	K11093
ENSG00000108395	-0.096748	5.975608	0.88522	1	TRIM37	K10608
ENSG00000153575	0.102004	4.071064	0.885229	1	TUBGCP5	K16572
ENSG00000090060	-0.095233	7.513382	0.885274	1	PAPOLA	K14376
ENSG00000168078	0.095398	7.095427	0.885321	1	PBK	K08865
ENSG00000168172	-0.10229	4.185822	0.885336	1	HOOK3	K16536
ENSG00000161016	0.094243	11.3352	0.885383	1	RPL8	K02938
ENSG00000155640	0.110792	2.324932	0.885401	1	C10orf12	_
ENSG00000107897	-0.097262	5.638089	0.885418	1	ACBD5	_
ENSG00000121310	-0.106372	4.285886	0.885429	1	ECHDC2	_
ENSG00000060138	0.094414	9.04839	0.885466	1	YBX3	K06099
ENSG00000116266	-0.095081	5.948183	0.885505	1	STXBP3	K15301
ENSG00000006534	0.096192	6.201972	0.885563	1	ALDH3B1	K00129
ENSG00000132912	0.095442	6.095635	0.885619	1	DCTN4	K10426
ENSG00000133740	-0.130839	2.673698	0.885717	1	E2F5	K04682
ENSG00000108559	0.095165	6.92753	0.885776	1	NUP88	K14318
ENSG00000104660	-0.098611	5.605146	0.885784	1	LEPROTL1	_
ENSG00000189308	-0.097722	5.250647	0.885788	1	LIN54	_
ENSG00000172315	-0.100101	4.4771	0.886014	1	TP53RK	K08851
ENSG00000184831	-0.097704	5.792382	0.886067	1	APOO	_
ENSG00000168096	-0.102444	4.714351	0.886117	1	ANKS3	K06588
ENSG00000175216	0.094146	8.042712	0.886155	1	CKAP5	K16803
ENSG00000272888	-0.096488	5.56959	0.886167	1	AC013394.2	_
ENSG00000156642	-0.095222	6.694218	0.886173	1	NPTN	_
ENSG00000084463	0.094381	7.443255	0.886246	1	WBP11	K12866
ENSG00000164080	0.107175	3.697743	0.886257	1	RAD54L2	_
ENSG00000154122	0.095208	6.448957	0.886344	1	ANKH	K09488
ENSG00000091651	-0.096366	5.489891	0.886352	1	ORC6	K02608
ENSG00000218891	0.098047	5.572421	0.886371	1	ZNF579	_
ENSG00000141510	-0.097924	5.612333	0.886379	1	TP53	K04451
ENSG00000002822	0.096332	5.259608	0.886546	1	MAD1L1	K06638
ENSG00000165733	-0.094951	6.650817	0.886562	1	BMS1	K14569
ENSG00000082641	0.093977	7.683519	0.886565	1	NFE2L1	_
ENSG00000164327	0.094334	7.021027	0.886678	1	RICTOR	K08267
ENSG00000124201	0.094216	6.191199	0.886743	1	ZNFX1	K17985
ENSG00000138443	-0.096154	5.361426	0.88679	1	ABI2	K05751
ENSG00000124422	0.093724	7.723302	0.886845	1	USP22	K11366
ENSG00000076662	-0.098589	4.572301	0.886863	1	ICAM3	K06486
ENSG00000104408	0.093111	9.663153	0.886914	1	EIF3E	K03250
ENSG00000131187	0.095805	4.094428	0.886924	1	F12	K01328
ENSG00000154065	-0.107137	3.981794	0.886942	1	ANKRD29	_
ENSG00000213859	-0.103735	3.710867	0.886985	1	KCTD11	_
ENSG00000135047	0.093439	8.078532	0.88699	1	CTSL	K01365
ENSG00000123395	0.095625	6.245991	0.886995	1	C12orf44	_
ENSG00000130702	0.09315	8.883427	0.887036	1	LAMA5	K06240
ENSG00000183405	-0.1067	3.381317	0.887044	1	RPS7P1	K02993
ENSG00000221823	-0.09599	5.998417	0.887053	1	PPP3R1	K06268
ENSG00000235706	-0.194716	1.281806	0.8871	1	DICER1-AS1	_
ENSG00000272077	-0.194716	1.281806	0.8871	1	RP11-348P10.2	_
ENSG00000186468	0.092863	10.34306	0.887124	1	RPS23	K02973
ENSG00000175741	-0.122716	2.959402	0.887127	1	RWDD4P2	_
ENSG00000133619	0.107992	2.953473	0.887127	1	KRBA1	_
ENSG00000183060	0.107992	2.953473	0.887127	1	LYSMD4	_

ENSG00000102898	-0.09436	6.730589	0.88716	1	NUTF2	_
ENSG00000104679	-0.101264	4.656421	0.887234	1	R3HCC1	_
ENSG00000041982	0.12715	2.350321	0.887234	1	TNC	K06252
ENSG00000100092	0.159733	1.910288	0.887266	1	SH3BP1	K12482
ENSG00000167525	0.159733	1.910288	0.887266	1	PROCA1	_
ENSG00000162924	0.134715	1.92309	0.887266	1	REL	K09254
ENSG00000132204	-0.130687	1.924134	0.887266	1	LINC00470	_
ENSG00000164066	-0.130687	1.924134	0.887266	1	INTU	K17985
ENSG00000178917	-0.130687	1.924134	0.887266	1	ZNF852	K09229
ENSG00000185347	-0.130687	1.924134	0.887266	1	C14orf80	_
ENSG00000182963	-0.095898	5.220392	0.887316	1	GJC1	K07616
ENSG00000177733	-0.093184	8.047651	0.887387	1	HNRNPA0	K12894
ENSG00000115163	0.099791	5.055735	0.887392	1	CENPA	K11495
ENSG00000173273	0.098541	4.733375	0.887566	1	TNKS	K10799
ENSG00000085978	0.097836	5.222484	0.887572	1	ATG16L1	K17890
ENSG00000182197	-0.093525	6.30436	0.887593	1	EXT1	K02366
ENSG00000124225	0.097207	5.547308	0.887601	1	PMEPA1	_
ENSG00000076554	0.092664	8.911121	0.887614	1	TPD52	_
ENSG00000161996	-0.094749	6.203868	0.887659	1	WDR90	_
ENSG00000185201	0.094192	6.3684	0.887691	1	IFITM2	K06566
ENSG00000171314	-0.093011	7.805407	0.887741	1	PGAM1	K01834
ENSG00000117640	0.097041	4.409271	0.887781	1	MTFR1L	K16800
ENSG00000188785	-0.111448	3.566729	0.887809	1	ZNF548	K09228
ENSG00000176903	-0.095345	5.175002	0.887869	1	PNMA1	_
ENSG00000163875	-0.09347	6.097137	0.887875	1	MEAF6	K11344
ENSG00000175376	-0.098118	5.119343	0.887914	1	EIF1AD	K15025
ENSG00000100600	-0.094045	6.458858	0.88795	1	LGMN	K01369
ENSG00000134884	-0.092976	7.522244	0.887988	1	ARGLU1	K13173
ENSG00000080546	-0.099551	4.111656	0.888027	1	SESN1	K10141
ENSG00000104823	0.092981	7.313555	0.888038	1	ECH1	K12663
ENSG00000182199	-0.092341	8.679669	0.888122	1	SHMT2	K00600
ENSG00000113141	0.09286	7.388525	0.888123	1	IK	K13109
ENSG00000217128	0.097246	4.50319	0.888136	1	FNIP1	_
ENSG00000100225	0.092959	7.223067	0.888142	1	FBXO7	K10293
ENSG00000144895	0.093028	7.124006	0.88815	1	EIF2A	K15026
ENSG00000169926	0.093006	7.020345	0.888278	1	KLF13	_
ENSG00000185825	0.100598	4.324196	0.888325	1	BCAP31	K14009
ENSG00000108639	-0.093321	6.70561	0.888444	1	SYNGR2	_
ENSG00000125755	0.093098	6.762349	0.888456	1	SYMPK	K06100
ENSG00000037757	-0.098606	5.335606	0.88846	1	MRI1	K08963
ENSG00000171307	0.094829	5.822383	0.888463	1	ZDHHC16	K18932
ENSG00000137970	0.091968	8.340241	0.888649	1	RPL7P9	K02937
ENSG00000158987	0.105884	3.406565	0.888822	1	RAPGEF6	K08020
ENSG00000129250	0.092059	7.665901	0.88889	1	KIF1C	K10392
ENSG00000099910	0.099176	3.87574	0.8889	1	KLHL22	K07604
ENSG00000074370	0.095404	4.229189	0.88894	1	ATP2A3	K05853
ENSG00000178295	-0.095469	4.752226	0.888978	1	GEN1	K15338
ENSG00000147454	0.094729	5.86441	0.889074	1	SLC25A37	K15113
ENSG00000120805	-0.092836	6.664286	0.889087	1	ARL1	K07942
ENSG00000176170	-0.093248	6.343215	0.889108	1	SPHK1	K04718
ENSG00000147251	0.10587	3.216742	0.889108	1	DOCK11	_
ENSG00000050405	-0.091504	8.581474	0.889169	1	LIMA1	_
ENSG00000133065	0.094295	5.191607	0.889171	1	SLC41A1	K15122
ENSG00000188612	-0.09174	7.954416	0.889183	1	SUMO2	K12160
ENSG00000114573	0.093522	5.934235	0.889279	1	ATP6V1A	K02145
ENSG00000078043	-0.098569	4.011907	0.889303	1	PIAS2	K16063
ENSG00000123144	0.091985	7.100855	0.889429	1	C19orf43	_
ENSG00000158710	0.091067	9.312168	0.889448	1	TAGLN2	_

ENSG00000103126	0.092711	6.379505	0.889454	1	AXIN1	K02157
ENSG00000137764	0.100226	4.431785	0.889535	1	MAP2K5	K04463
ENSG00000153113	0.091116	8.655634	0.889562	1	CAST	K04281
ENSG00000122359	0.091826	7.156348	0.889568	1	ANXA11	K17095
ENSG00000137275	-0.092619	5.487939	0.88957	1	RIPK1	K02861
ENSG00000007376	-0.092589	6.12064	0.889659	1	RPUSD1	_
ENSG00000157778	0.092391	6.496858	0.88966	1	PSMG3	K11877
ENSG00000073803	0.099852	4.341617	0.889719	1	MAP3K13	K04422
ENSG00000055211	-0.09537	5.695048	0.889744	1	GINM1	_
ENSG00000167900	0.091661	7.092198	0.889828	1	TK1	K00857
ENSG00000155660	-0.091025	8.216041	0.889905	1	PDIA4	K09582
ENSG00000111206	-0.091758	7.04234	0.889907	1	FOXM1	K09406
ENSG00000084652	-0.091967	6.792017	0.889958	1	TXLNA	_
ENSG00000177646	-0.092456	6.398306	0.889963	1	ACAD9	K15980
ENSG00000100258	0.0919	6.659331	0.890028	1	LMF2	_
ENSG00000116903	0.09563	5.0935	0.890191	1	EXOC8	_
ENSG00000088387	0.093717	5.810704	0.890196	1	DOCK9	_
ENSG00000153037	-0.092794	5.776481	0.890281	1	SRP19	K03105
ENSG00000089006	-0.091711	6.704776	0.890383	1	SNX5	K17920
ENSG00000223855	0.096661	3.427268	0.890544	1	AC147651.3	_
ENSG00000123975	-0.091135	7.092672	0.890604	1	CKS2	K02219
ENSG00000092439	-0.091854	6.438792	0.890618	1	TRPM7	K04982
ENSG00000152270	0.10585	2.998124	0.890624	1	PDE3B	K13296
ENSG00000163083	-0.18274	1.351736	0.890635	1	INHBB	K04667
ENSG00000179476	-0.18274	1.351736	0.890635	1	C14orf28	_
ENSG00000235944	0.150697	1.313696	0.890635	1	ZNF815P	K09228
ENSG00000176927	-0.148115	1.332281	0.890635	1	EFCAB5	_
ENSG00000224818	-0.148115	1.332281	0.890635	1	RP11-134G8.8	_
ENSG00000069696	0.112395	1.33358	0.890635	1	DRD4	K04147
ENSG00000070371	0.112395	1.33358	0.890635	1	CLTCL1	K04646
ENSG00000149564	0.112395	1.33358	0.890635	1	ESAM	K06787
ENSG00000198380	-0.09077	7.432479	0.890722	1	GFPT1	K00820
ENSG00000126249	-0.097224	4.624766	0.890749	1	PDCD2L	K14801
ENSG00000022840	0.090776	7.21835	0.890778	1	RNF10	_
ENSG00000085733	0.090569	7.519345	0.890788	1	CTTN	K06106
ENSG00000159377	-0.090234	8.371186	0.890789	1	PSMB4	K02736
ENSG00000140987	0.097106	4.845976	0.890833	1	ZSCAN32	K09228
ENSG00000186416	-0.092515	5.631917	0.890854	1	NKRF	_
ENSG00000108179	-0.090545	7.559449	0.890891	1	PPIF	K09565
ENSG00000112200	-0.091514	6.486507	0.890949	1	ZNF451	_
ENSG00000151893	0.090899	6.885084	0.890961	1	CACUL1	_
ENSG00000127080	0.098368	4.031409	0.89101	1	IPPK	K10572
ENSG00000198732	-0.090967	6.803055	0.891148	1	SMOC1	_
ENSG00000107968	0.096213	4.708431	0.891252	1	MAP3K8	K04415
ENSG00000165124	-0.091305	6.444845	0.891269	1	SVEP1	K17495
ENSG00000106263	0.089628	8.867149	0.891294	1	EIF3B	K03253
ENSG00000172809	-0.089678	8.798161	0.891298	1	RPL38	K12581
ENSG00000237686	-0.108713	2.746755	0.891349	1	RP5-1120P11.1	_
ENSG00000182372	0.105823	2.740394	0.891349	1	CLN8	_
ENSG00000172830	-0.097147	4.548136	0.891373	1	SSH3	K05766
ENSG00000172765	0.103719	3.90819	0.891384	1	TMCC1	_
ENSG00000073584	0.091522	6.025291	0.891488	1	SMARCE1	K11651
ENSG00000125835	-0.089804	7.979829	0.891505	1	SNRPB	K11086
ENSG00000153827	0.091225	5.99603	0.891588	1	TRIP12	K10590
ENSG00000013306	-0.089514	8.506683	0.891602	1	SLC25A39	K15119
ENSG00000126746	0.090696	5.932795	0.89165	1	ZNF384	_
ENSG00000078674	0.090737	6.433289	0.891746	1	PCM1	K16537
ENSG00000079313	0.093296	5.278951	0.891754	1	REXO1	K14570

ENSG00000104915	-0.091197	6.176865	0.891813	1	STX10	K08498
ENSG00000214176	-0.147146	1.981602	0.891888	1	PLEKHM1P	_
ENSG00000106479	0.107849	1.980967	0.891888	1	ZNF862	_
ENSG00000154429	0.092525	4.716813	0.891952	1	CCSAP	K16454
ENSG00000163734	0.206774	0.23912	0.89207	1	CXCL3	K05505
ENSG00000234949	0.206774	0.23912	0.89207	1	AC104667.3	K15696
ENSG00000252690	0.206774	0.23912	0.89207	1	SCARNA15	_
ENSG00000266904	0.206774	0.23912	0.89207	1	LINC00663	_
ENSG00000270441	0.206774	0.23912	0.89207	1	RP11-694I15.7	K06243
ENSG00000271833	0.206774	0.23912	0.89207	1	RP11-356B19.11	_
ENSG00000271895	0.206774	0.23912	0.89207	1	RP4-635E18.8	_
ENSG00000272787	0.206774	0.23912	0.89207	1	KB-226F1.2	_
ENSG00000154511	-0.100617	4.046831	0.89212	1	FAM69A	_
ENSG00000135723	-0.093135	5.524589	0.892224	1	FHOD1	_
ENSG00000105676	0.092433	5.523659	0.892224	1	ARMC6	_
ENSG00000146281	-0.093065	5.062745	0.892282	1	PM20D2	K17985
ENSG00000118515	0.088622	9.642412	0.892342	1	SGK1	K13302
ENSG00000183291	-0.088864	8.559348	0.892366	1	15-Sep	_
ENSG00000166452	0.09142	6.10011	0.892514	1	AKIP1	_
ENSG00000183513	0.094668	5.18197	0.892629	1	COA5	K18178
ENSG00000108784	-0.091922	5.237918	0.892662	1	NAGLU	K01205
ENSG00000179750	0.089466	4.801073	0.892687	1	APOBEC3B	K18750
ENSG00000137509	0.088686	7.712647	0.89293	1	PRCP	K01285
ENSG00000164919	-0.08857	8.084318	0.892937	1	COX6C	K02268
ENSG00000102870	-0.10911	3.272131	0.89295	1	ZNF629	K09228
ENSG00000160058	0.092324	5.847742	0.892954	1	BSDC1	_
ENSG00000180574	-0.094572	4.652907	0.892955	1	EIF2S3L	K03242
ENSG00000095203	-0.09679	4.806504	0.893012	1	EPB41L4B	_
ENSG00000129473	0.095894	4.876173	0.893036	1	BCL2L2	K02163
ENSG00000233913	0.091679	5.1303	0.893113	1	CTC-575D19.1	K02866
ENSG00000108523	0.08902	6.958526	0.893146	1	RNF167	K15706
ENSG00000134186	-0.089021	7.012785	0.89324	1	PRPF38B	K12850
ENSG00000129347	0.090283	6.200168	0.893567	1	KRI1	K14786
ENSG00000213553	0.088367	7.33516	0.893586	1	RPLP0P6	K02941
ENSG00000089486	0.092967	4.951847	0.893644	1	CDIP1	_
ENSG00000214078	-0.089394	6.385788	0.893668	1	CPNE1	_
ENSG00000116985	0.094598	4.662402	0.893671	1	BMP8B	K16622
ENSG00000117569	0.094256	4.578588	0.89368	1	PTBP2	K14948
ENSG00000153814	-0.094836	4.490781	0.893689	1	JAZF1	K19495
ENSG00000113580	0.088671	6.818998	0.893721	1	NR3C1	K05771
ENSG00000172757	-0.087512	9.553188	0.893726	1	CFL1	K05765
ENSG00000122218	0.087779	8.244946	0.893748	1	COPA	K05236
ENSG00000011454	0.090693	5.670435	0.893771	1	RABGAP1	K09488
ENSG00000172840	-0.100984	3.804803	0.893781	1	PDP2	K01102
ENSG00000146938	-0.102598	3.652775	0.893806	1	NLGN4X	K07378
ENSG00000126107	0.092288	5.305821	0.893844	1	HECTD3	K12233
ENSG00000152433	0.145192	1.382752	0.893954	1	ZNF547	K09228
ENSG00000224281	0.145192	1.382752	0.893954	1	SLC25A5-AS1	_
ENSG00000224738	-0.138895	1.399945	0.893954	1	AC099850.1	_
ENSG00000226287	-0.138895	1.399945	0.893954	1	TMEM191A	_
ENSG00000215788	-0.11849	2.779346	0.893961	1	TNFRSF25	K05160
ENSG00000261115	-0.11849	2.779346	0.893961	1	TMEM178B	_
ENSG00000109736	-0.091066	5.547265	0.893987	1	MFSD10	_
ENSG00000106615	0.088216	7.079912	0.893994	1	RHEB	K07208
ENSG00000089820	-0.111157	2.442361	0.894017	1	ARHGAP4	K07526
ENSG00000196074	-0.111157	2.442361	0.894017	1	SYCP2	K19529
ENSG00000235477	-0.111157	2.442361	0.894017	1	RP11-122G18.5	_
ENSG00000130487	0.128736	2.012935	0.894059	1	KLHDC7B	_

ENSG00000101019	-0.09061	5.593394	0.89416	1	UQCC1	K17662
ENSG00000131828	-0.08723	9.058254	0.89418	1	PDHA1	K00161
ENSG00000102572	0.08794	7.179821	0.894233	1	STK24	K08838
ENSG00000141560	-0.090181	5.638093	0.894323	1	FN3KRP	K15523
ENSG00000143367	-0.088542	5.596116	0.894346	1	TUFT1	K12581
ENSG00000103429	0.088874	6.240181	0.894406	1	BFAR	K15684
ENSG00000123815	0.090233	4.587762	0.894427	1	ADCK4	K08869
ENSG00000144524	-0.091281	5.367807	0.894489	1	COPS7B	K12180
ENSG00000162244	0.087006	8.724855	0.894506	1	RPL29	K02905
ENSG00000213614	-0.088358	6.067387	0.89452	1	HEXA	K12373
ENSG00000124688	0.089708	5.315771	0.894523	1	MAD2L1BP	_
ENSG00000165392	-0.098584	4.408561	0.894546	1	WRN	K10900
ENSG00000113658	0.089356	6.157778	0.894586	1	SMAD5	K16790
ENSG00000232388	0.094065	4.676199	0.894727	1	LINC00493	_
ENSG00000259917	-0.107236	3.290136	0.894774	1	HNRNPLP2	K13159
ENSG00000176715	0.088938	5.514089	0.894791	1	ACSF3	K18660
ENSG00000186594	0.096217	4.084114	0.894797	1	MIR22HG	_
ENSG00000131375	0.090202	5.684152	0.894825	1	CAPN7	K08576
ENSG00000114098	-0.090897	5.372047	0.894922	1	ARMC8	_
ENSG00000142949	-0.086737	8.13776	0.895124	1	PTPRF	K05695
ENSG00000071859	-0.101224	3.666631	0.895206	1	FAM50A	K13119
ENSG00000134698	-0.115679	2.804598	0.895221	1	AGO4	K11593
ENSG00000113558	0.08646	8.548713	0.895224	1	SKP1	K03094
ENSG00000144659	-0.088618	5.274617	0.895255	1	SLC25A38	K15118
ENSG00000221968	-0.08741	6.919545	0.89529	1	FADS3	K10225
ENSG00000171680	-0.093079	4.422901	0.895379	1	PLEKHG5	K19464
ENSG00000112149	-0.087672	5.477153	0.895434	1	CD83	K06510
ENSG00000069424	0.089716	4.602287	0.895529	1	KCNAB2	K04883
ENSG00000204682	-0.088203	4.605039	0.895529	1	CASC10	K09228
ENSG00000164897	0.0866	6.051316	0.895625	1	TMUB1	_
ENSG00000182095	-0.087296	6.735561	0.89566	1	TNRC18	_
ENSG00000183726	-0.08709	6.931447	0.895662	1	TMEM50A	_
ENSG00000179454	0.110907	3.303608	0.895662	1	KLHL28	K10464
ENSG00000166912	-0.096804	3.303315	0.895662	1	MTMR10	K18085
ENSG00000100908	0.094899	3.830256	0.895664	1	EMC9	_
ENSG00000136536	0.087556	6.375796	0.895664	1	7-Mar	K10662
ENSG00000148335	-0.087462	6.232154	0.89586	1	NTMT1	K16219
ENSG00000185651	0.086312	7.546566	0.895907	1	UBE2L3	K04552
ENSG00000224578	-0.08744	6.442843	0.895924	1	HNRNPA1P48	K12741
ENSG00000071082	0.085661	9.618369	0.895928	1	RPL31	K02910
ENSG00000203950	-0.088796	5.530292	0.895953	1	FAM127B	_
ENSG00000110619	-0.08671	7.046679	0.89599	1	CARS	K01883
ENSG00000168522	-0.090071	5.619488	0.895993	1	FNTA	K05955
ENSG00000120948	-0.086005	8.015186	0.896074	1	TARDBP	_
ENSG00000158019	-0.086992	5.439261	0.896112	1	BRE	K12173
ENSG00000143570	-0.085895	8.136674	0.896142	1	SLC39A1	K14709
ENSG00000178952	-0.085776	8.403937	0.896162	1	TUFM	K02358
ENSG00000169583	-0.094068	3.509837	0.896176	1	CLIC3	K05023
ENSG00000148943	-0.08767	5.705173	0.896198	1	LIN7C	K04613
ENSG00000177885	0.087061	6.390512	0.896237	1	GRB2	K04364
ENSG00000145241	-0.09738	3.841921	0.896277	1	CENPC	K11497
ENSG00000166136	-0.091944	4.923782	0.896324	1	NDUFB8	K03964
ENSG00000121274	0.090166	4.107267	0.896341	1	PAPD5	K03514
ENSG00000170846	0.090166	4.107267	0.896341	1	AC093323.3	_
ENSG00000134996	0.089192	5.055759	0.896351	1	OSTF1	_
ENSG00000203896	0.102288	2.817166	0.896452	1	LIME1	_
ENSG00000179262	-0.085825	7.663898	0.896513	1	RAD23A	K10839
ENSG00000142197	-0.095123	3.320941	0.896536	1	DOPEY2	K12581

ENSG00000169992	0.086736	6.431245	0.896566	1	NLGN2	K07378
ENSG00000134222	-0.089049	5.670302	0.89662	1	PSRC1	_
ENSG00000143771	-0.088041	6.157351	0.896621	1	CNIH4	_
ENSG00000121766	0.086661	6.127342	0.896684	1	ZCCHC17	K11723
ENSG00000134644	-0.086371	6.774126	0.896724	1	PUM1	K17943
ENSG00000129562	0.08662	6.37209	0.896797	1	DAD1	K12668
ENSG00000168003	0.084776	10.84336	0.896862	1	SLC3A2	K06519
ENSG00000123064	0.085813	7.093779	0.896879	1	DDX54	K14808
ENSG00000239857	0.100843	3.518061	0.896934	1	GET4	_
ENSG00000133872	-0.08572	7.283456	0.896947	1	TMEM66	_
ENSG00000198231	0.085503	7.389013	0.897	1	DDX42	K12835
ENSG00000072121	0.090606	4.231567	0.897061	1	ZFYVE26	K19027
ENSG00000186086	0.175681	1.430391	0.897076	1	NBPF6	_
ENSG00000228109	0.175681	1.430391	0.897076	1	MFI2-AS1	_
ENSG00000186010	-0.088326	5.352865	0.897155	1	NDUFA13	K11353
ENSG00000138433	0.088259	4.537653	0.897171	1	CIR1	K06066
ENSG00000105583	0.086309	6.349131	0.897208	1	WDR83OS	_
ENSG00000118655	0.087103	5.190417	0.897337	1	DCLRE1B	K15341
ENSG00000104341	-0.084416	9.187273	0.897551	1	LAPTM4B	K12387
ENSG00000135597	-0.091327	4.797052	0.897558	1	REPS1	_
ENSG00000011478	0.085095	6.015138	0.897642	1	QPCTL	K00683
ENSG00000108773	-0.124922	2.835906	0.897654	1	KAT2A	K06062
ENSG00000169169	-0.124922	2.835906	0.897654	1	CPT1C	K19524
ENSG00000144040	-0.112966	2.829417	0.897654	1	SFXN5	K12484
ENSG00000183891	-0.107351	3.532969	0.89768	1	TTC32	_
ENSG00000079150	-0.100015	3.529055	0.89768	1	FKBP7	K09573
ENSG00000169764	-0.084966	7.349731	0.897799	1	UGP2	K00963
ENSG00000177239	-0.086148	6.260453	0.897829	1	MAN1B1	K01230
ENSG00000147533	-0.089155	5.414807	0.897855	1	GOLGA7	_
ENSG00000120889	0.085143	6.87911	0.897908	1	TNFRSF10B	K04722
ENSG00000169490	0.086334	5.645231	0.897937	1	TM2D2	_
ENSG00000147010	-0.088592	5.140608	0.897984	1	SH3KBP1	K12470
ENSG00000104221	0.101036	3.104047	0.898	1	BRF2	K15197
ENSG00000048828	-0.084468	7.871525	0.898016	1	FAM120A	_
ENSG00000163249	-0.092602	4.94813	0.898034	1	CCNYL1	_
ENSG00000173889	0.090271	4.947596	0.898034	1	PHC3	K11458
ENSG00000133789	-0.084997	7.066446	0.898035	1	SWAP70	_
ENSG00000176894	0.090666	4.354611	0.898093	1	PXMP2	K13347
ENSG00000260917	0.126018	2.055837	0.89815	1	RP11-57H14.4	_
ENSG00000153234	0.088474	4.004435	0.898213	1	NR4A2	K08558
ENSG00000131724	0.084449	7.454006	0.898224	1	IL13RA1	K05076
ENSG00000028310	0.085012	6.601745	0.898402	1	BRD9	K11723
ENSG00000119723	-0.098545	3.544144	0.898416	1	COQ6	K06126
ENSG00000162062	-0.090973	4.729252	0.898427	1	C16orf59	_
ENSG00000100053	-0.280451	0.351706	0.898528	1	CRYBB3	_
ENSG00000136630	-0.280451	0.351706	0.898528	1	HLX	K09339
ENSG00000163666	-0.280451	0.351706	0.898528	1	HESX1	K09354
ENSG00000188897	-0.280451	0.351706	0.898528	1	CTD-3088G3.8	_
ENSG00000232882	-0.280451	0.351706	0.898528	1	PHKA1P1	K07190
ENSG00000266947	-0.280451	0.351706	0.898528	1	RP11-799D4.4	_
ENSG00000268358	-0.280451	0.351706	0.898528	1	EPB41L4A-AS2	_
ENSG00000162526	0.270897	0.340128	0.898528	1	TSSK3	K08811
ENSG00000187942	0.270897	0.340128	0.898528	1	LDLRAD2	_
ENSG00000233554	0.270897	0.340128	0.898528	1	RP11-326F20.5	_
ENSG00000253320	0.270897	0.340128	0.898528	1	KB-1507C5.2	_
ENSG00000152939	0.18922	0.380917	0.898528	1	MARVELD2	K17291
ENSG00000229980	0.18922	0.380917	0.898528	1	TOB1-AS1	_
ENSG00000198700	0.084337	7.235308	0.898533	1	IPO9	_

ENSG00000081177	0.09962	3.714657	0.898551	1	EXD2	K12606
ENSG00000172059	0.090326	5.023746	0.898637	1	KLF11	K09209
ENSG00000078668	-0.084931	6.614181	0.898681	1	VDAC3	K15041
ENSG00000170248	-0.084553	6.910475	0.898747	1	PDCD6IP	K12200
ENSG00000120063	-0.085356	5.894678	0.898768	1	GNA13	K04639
ENSG00000177830	0.08683	5.268752	0.898781	1	CHID1	K17525
ENSG00000183684	-0.084609	6.812861	0.898798	1	ALYREF	K12881
ENSG00000186577	0.088499	2.848329	0.898828	1	C6orf1	_
ENSG00000117691	-0.089134	5.526492	0.898909	1	NENF	_
ENSG00000145391	0.084869	6.360385	0.898923	1	SETD7	K11431
ENSG00000103852	-0.104369	3.128989	0.898973	1	TTC23	_
ENSG00000011260	-0.08425	7.028673	0.898977	1	UTP18	K14553
ENSG00000178802	0.085823	5.430087	0.899063	1	MPI	K01809
ENSG00000091640	0.085197	5.968957	0.899082	1	SPAG7	_
ENSG00000185658	0.084408	6.036683	0.899104	1	BRWD1	K11798
ENSG00000011566	0.086343	4.892544	0.899122	1	MAP4K3	K04406
ENSG00000176783	-0.086329	5.621273	0.899124	1	RUFY1	K12482
ENSG00000139697	0.086207	6.102654	0.899249	1	SBNO1	_
ENSG00000182841	0.088747	4.147443	0.899296	1	RRP7B	K14545
ENSG00000007202	0.083582	7.400215	0.899312	1	KIAA0100	_
ENSG00000106789	0.090053	4.371669	0.899338	1	CORO2A	K13887
ENSG00000269190	-0.089072	4.822056	0.899401	1	FBXO17	K10101
ENSG00000077713	-0.088216	5.27914	0.899446	1	SLC25A43	K15120
ENSG00000128309	0.083705	6.924596	0.899594	1	MPST	K01011
ENSG00000150867	-0.089199	4.748943	0.899708	1	PIP4K2A	K00920
ENSG00000073849	-0.090815	4.575938	0.899725	1	ST6GAL1	K00778
ENSG00000198677	-0.083613	7.004296	0.899773	1	TTC37	K12600
ENSG00000132768	-0.08494	5.755544	0.899796	1	DPH2	K17866
ENSG00000089022	-0.084424	5.491841	0.899842	1	MAPKAPK5	K04442
ENSG00000100221	-0.083752	6.75762	0.899904	1	JOSD1	K15235
ENSG00000100162	0.08431	5.042519	0.899942	1	CENPM	K11505
ENSG00000118162	-0.096151	2.871522	0.899976	1	KPTN	_
ENSG00000187908	-0.162134	1.48225	0.900018	1	DMBT1	K13912
ENSG00000143450	0.105579	1.466637	0.900018	1	OAZ3	K16614
ENSG00000171403	0.105579	1.466637	0.900018	1	KRT9	K07604
ENSG00000188559	-0.116198	2.54382	0.90003	1	RALGAPA2	_
ENSG00000124006	-0.086638	5.396188	0.900065	1	OBSL1	K19574
ENSG00000111860	-0.130733	2.108496	0.900079	1	CEP85L	K16766
ENSG00000119280	-0.087306	4.486829	0.900113	1	C1orf198	_
ENSG00000188529	-0.092135	4.387724	0.900151	1	SRSF10	_
ENSG00000115486	0.083898	6.32251	0.900154	1	GGCX	K10106
ENSG00000100632	-0.082991	7.347588	0.900185	1	ERH	_
ENSG00000197299	-0.087827	5.497674	0.90022	1	BLM	K10901
ENSG00000161036	-0.086874	4.980413	0.900229	1	LRWD1	_
ENSG00000100220	-0.083189	7.001222	0.900288	1	RTCB	K14415
ENSG00000167536	0.098503	3.741159	0.900458	1	DHRS13	K11169
ENSG00000198612	-0.084745	5.918793	0.900479	1	COPS8	K12181
ENSG00000150054	-0.084955	5.882097	0.900503	1	MPP7	_
ENSG00000125457	-0.084081	4.913612	0.900542	1	MIF4GD	_
ENSG00000137273	0.083942	5.550117	0.900561	1	FOXF2	K09399
ENSG00000166848	-0.084259	5.685719	0.900639	1	TERF2IP	K11113
ENSG00000110711	0.08645	5.64172	0.900671	1	AIP	K17767
ENSG00000152234	0.08171	9.214755	0.900786	1	ATP5A1	K02132
ENSG00000168264	-0.08257	7.190791	0.900839	1	IRF2BP2	_
ENSG00000198492	-0.082928	6.767314	0.900885	1	YTHDF2	_
ENSG00000176046	0.083578	6.063169	0.900905	1	NUPR1	K15626
ENSG00000115459	0.08612	3.905475	0.900932	1	ELMOD3	_
ENSG00000223764	0.086499	5.057083	0.900962	1	RP11-54O7.3	_

ENSG00000160209	0.081698	8.444865	0.901022	1	PDXK	K00868
ENSG00000125482	-0.088118	4.685612	0.901039	1	TTF1	K15225
ENSG00000100138	-0.081898	7.932207	0.901086	1	NHP2L1	K12845
ENSG00000242294	0.111473	2.883788	0.901097	1	STAG3L5P	K13055
ENSG00000149929	0.087733	4.176856	0.901175	1	HIRIP3	_
ENSG00000140105	0.08155	8.278926	0.901265	1	WARS	K01867
ENSG00000188242	-0.083595	5.652985	0.901344	1	CTD-2228K2.5	_
ENSG00000175387	-0.082594	6.722769	0.901347	1	SMAD2	K04500
ENSG00000134330	-0.096322	3.91639	0.901485	1	IAH1	K17985
ENSG00000140740	-0.081743	7.580947	0.901505	1	UQCRC2	K00415
ENSG00000157593	0.082059	6.978465	0.901524	1	SLC35B2	K15276
ENSG00000136709	0.083474	5.9335	0.901588	1	WDR33	K15542
ENSG00000123297	-0.082521	6.534144	0.901706	1	TSFM	K02357
ENSG00000251381	0.083706	5.365576	0.90173	1	LINC00958	_
ENSG00000116213	-0.085893	4.410536	0.901738	1	WRAP73	_
ENSG00000188976	-0.081372	7.899351	0.901741	1	NOC2L	K14833
ENSG00000163348	0.082804	6.17061	0.901759	1	PYGO2	_
ENSG00000112306	-0.080872	9.55853	0.90176	1	RPS12	K02951
ENSG00000162928	0.082515	5.471217	0.901773	1	PEX13	K13344
ENSG00000011485	-0.082506	6.500161	0.901776	1	PPP5C	K04460
ENSG00000046647	0.098466	3.164054	0.901785	1	GEMIN8	K13136
ENSG00000112367	0.092058	4.05542	0.901815	1	FIG4	_
ENSG00000106397	-0.081894	7.024648	0.901826	1	PLOD3	K13646
ENSG00000143398	0.081541	7.191244	0.901949	1	PIP5K1A	K00889
ENSG00000198169	-0.083881	5.475183	0.901961	1	ZNF251	K09228
ENSG00000154781	-0.089652	3.924997	0.902032	1	CCDC174	_
ENSG00000174574	0.081588	6.92278	0.902151	1	AKIRIN1	_
ENSG00000162735	0.083998	5.663522	0.902172	1	PEX19	K13337
ENSG00000138592	-0.082043	6.539811	0.902273	1	USP8	K11839
ENSG00000106003	0.090799	3.770491	0.902294	1	LFNG	K05948
ENSG00000087510	0.087737	4.313303	0.902318	1	TFAP2C	K09177
ENSG00000100104	-0.088297	4.519698	0.902323	1	SRRD	_
ENSG00000103502	-0.082163	5.982585	0.902337	1	CDIPT	K00999
ENSG00000100380	0.080475	8.77064	0.902394	1	ST13	K09560
ENSG00000114742	-0.088851	4.789136	0.902478	1	WDR48	K15361
ENSG00000138092	-0.08349	4.61757	0.902493	1	CENPO	K11507
ENSG00000145685	0.082329	5.266479	0.902549	1	LHFPL2	_
ENSG00000134317	-0.092961	3.602976	0.90262	1	GRHL1	K09275
ENSG00000145216	0.081678	5.432122	0.902623	1	FIP1L1	K14405
ENSG00000139977	-0.089567	4.707597	0.902643	1	NAA30	K00670
ENSG00000087301	0.107698	3.178456	0.902688	1	TXNDC16	_
ENSG00000115524	0.08029	8.423548	0.902734	1	SF3B1	K12828
ENSG00000079462	-0.084254	5.272346	0.902763	1	PAFAH1B3	K16795
ENSG00000072195	0.083432	4.791623	0.902776	1	SPEG	K08809
ENSG00000138678	-0.127264	2.581315	0.902788	1	AGPAT9	K13506
ENSG00000166262	-0.127264	2.581315	0.902788	1	FAM227B	_
ENSG00000255857	-0.127264	2.581315	0.902788	1	PXN-AS1	_
ENSG00000126106	-0.112933	2.573496	0.902788	1	TMEM53	K13194
ENSG00000197147	-0.112933	2.573496	0.902788	1	LRRC8B	K09488
ENSG00000104892	0.135663	1.511653	0.902795	1	KLC3	K10407
ENSG00000160117	0.135663	1.511653	0.902795	1	ANKLE1	_
ENSG00000247796	-0.12294	1.526493	0.902795	1	CTD-2366F13.1	_
ENSG00000066697	-0.087714	5.150514	0.9028	1	MSANTD3	_
ENSG00000197965	0.080704	7.298804	0.90287	1	MPZL1	K06770
ENSG00000138434	0.080426	7.602915	0.902981	1	SSFA2	_
ENSG00000075275	0.081374	6.405581	0.903066	1	CELSR1	K04600
ENSG00000134954	0.083236	4.797946	0.903072	1	ETS1	K02678
ENSG00000151466	-0.089308	4.323463	0.903142	1	SCLT1	_

ENSG00000167775	0.0809	6.736202	0.903192	1	CD320	K06734
ENSG00000184840	0.080037	7.987518	0.903229	1	TMED9	_
ENSG00000107872	-0.08212	5.157891	0.903263	1	FBXL15	K10281
ENSG00000175866	-0.08212	5.157891	0.903263	1	BAIAP2	K05627
ENSG00000101365	-0.080985	6.681515	0.903344	1	IDH3B	K00030
ENSG00000184743	0.081158	6.225578	0.903395	1	ATL3	K17339
ENSG00000170312	-0.079823	8.080485	0.903515	1	CDK1	K02087
ENSG00000135250	0.08171	6.166337	0.903555	1	SRPK2	K08831
ENSG00000189171	-0.082529	6.104825	0.903729	1	S100A13	_
ENSG00000079459	-0.079851	7.66803	0.90373	1	FDFT1	K00801
ENSG00000006576	-0.081867	6.002553	0.903765	1	PHTF2	_
ENSG00000108479	0.081355	6.003552	0.903765	1	GALK1	K00849
ENSG00000178999	-0.080769	6.50934	0.903857	1	AURKB	K11479
ENSG00000104147	-0.084275	5.399307	0.903909	1	OIP5	K11565
ENSG00000180198	-0.080367	6.807409	0.903924	1	RCC1	K11493
ENSG00000163041	-0.081297	5.16768	0.903949	1	H3F3A	K11253
ENSG00000116815	-0.083361	5.228584	0.903992	1	CD58	K06492
ENSG00000218426	0.079727	7.313492	0.904038	1	RP11-475C16.1	K02900
ENSG00000001460	-0.109803	2.602577	0.904111	1	STPG1	_
ENSG00000011422	-0.080389	6.646938	0.904111	1	PLAUR	K03985
ENSG00000144935	-0.248559	0.48187	0.904233	1	TRPC1	K04964
ENSG00000161103	-0.248559	0.48187	0.904233	1	AC008132.13	_
ENSG00000175536	-0.248559	0.48187	0.904233	1	LIPT2	K03801
ENSG00000203362	-0.248559	0.48187	0.904233	1	RP3-337H4.8	_
ENSG00000231125	-0.248559	0.48187	0.904233	1	AF129075.5	_
ENSG00000267249	-0.248559	0.48187	0.904233	1	RP11-973H7.3	_
ENSG00000272168	-0.248559	0.48187	0.904233	1	CASC15	_
ENSG00000225067	-0.181966	0.444504	0.904233	1	RPL23AP2	K02893
ENSG00000237758	-0.181966	0.444504	0.904233	1	BANF1P3	_
ENSG00000250397	-0.181966	0.444504	0.904233	1	RP11-1391J7.1	_
ENSG00000259972	-0.181966	0.444504	0.904233	1	AC009120.6	_
ENSG00000272578	-0.181966	0.444504	0.904233	1	AP000347.2	K01195
ENSG00000196781	-0.090666	4.096271	0.904236	1	TLE1	_
ENSG00000039523	0.080842	5.973843	0.904256	1	FAM65A	_
ENSG00000070540	0.086131	4.224583	0.9043	1	WIPI1	K17908
ENSG00000159147	-0.080484	6.441352	0.904308	1	DONSON	K11876
ENSG00000105419	0.08242	4.341749	0.904353	1	MEIS3	K16671
ENSG00000183527	0.079318	7.49347	0.904397	1	PSMG1	K11875
ENSG00000183671	0.096867	3.202713	0.904445	1	GPR1	K04310
ENSG00000163811	-0.079711	7.034723	0.904452	1	WDR43	K14546
ENSG00000164402	-0.086083	4.820494	0.904528	1	8-Sep	K16939
ENSG00000183624	0.081929	6.117254	0.904556	1	HMCES	_
ENSG00000243943	0.083758	4.972981	0.904573	1	ZNF512	_
ENSG00000143553	0.081385	5.298046	0.904653	1	SNAPIN	_
ENSG00000173818	0.08426	3.962888	0.904677	1	ENDOV	_
ENSG00000170619	-0.080738	5.465293	0.904688	1	COMMD5	_
ENSG00000123473	0.082445	5.657449	0.904723	1	STIL	K16724
ENSG00000087586	-0.07912	7.480702	0.904753	1	AURKA	K11481
ENSG00000148634	-0.079033	7.619146	0.904754	1	HERC4	K10615
ENSG00000169826	-0.088579	4.559287	0.904781	1	CSGALNACT2	K00746
ENSG00000154845	-0.080138	6.383192	0.904822	1	PPP4R1	K15424
ENSG00000108055	0.078675	7.922632	0.904911	1	SMC3	K06669
ENSG00000125952	-0.081787	5.24717	0.905067	1	MAX	K04453
ENSG00000146282	-0.082666	5.121834	0.905076	1	RARS2	K01887
ENSG00000105058	0.079682	6.392058	0.905127	1	FAM32A	K13120
ENSG00000214135	0.089638	3.971243	0.905189	1	AC024560.3	K00234
ENSG00000145979	-0.085617	3.970411	0.905189	1	TBC1D7	_
ENSG00000115317	0.080475	5.665249	0.905203	1	HTRA2	K08669

ENSG00000099812	-0.078747	7.467141	0.905215	1	MISP	_
ENSG00000225855	0.0877	3.447674	0.905264	1	RUSC1-AS1	_
ENSG00000081791	0.078389	6.060531	0.905268	1	KIAA0141	_
ENSG00000149480	-0.078998	7.075602	0.90527	1	MTA2	K11660
ENSG00000151729	0.07945	6.472319	0.905289	1	SLC25A4	K05863
ENSG00000129235	0.079665	5.915435	0.9053	1	TXNDC17	_
ENSG00000089053	-0.079028	6.993347	0.905321	1	ANAPC5	K03352
ENSG00000177034	-0.111758	2.953472	0.905344	1	MTX3	K17776
ENSG00000215375	-0.111758	2.953472	0.905344	1	MYL5	K12753
ENSG00000130638	-0.078589	7.522793	0.905362	1	ATXN10	K19323
ENSG00000113719	0.078041	8.655991	0.905377	1	ERGIC1	_
ENSG00000243725	0.097601	2.610272	0.905398	1	TTC4	K01881
ENSG00000100490	0.163812	1.55538	0.905421	1	CDKL1	K08824
ENSG00000253982	0.131512	1.572019	0.905421	1	CTD-2336O2.1	_
ENSG00000163681	0.078543	5.955575	0.905421	1	SLMAP	_
ENSG00000113163	-0.086624	4.663287	0.905431	1	COL4A3BP	K08283
ENSG00000136492	-0.086624	4.663287	0.905431	1	BRIP1	K15362
ENSG00000234449	-0.121312	2.187322	0.90545	1	RP11-706O15.3	_
ENSG00000246582	0.098353	2.187874	0.90545	1	RP11-1149O23.3	_
ENSG00000214485	0.077896	8.813283	0.905505	1	RPL7P1	K02937
ENSG00000189180	0.079207	5.12656	0.905538	1	ZNF33A	K09228
ENSG00000135823	-0.079336	5.529344	0.905576	1	STX6	K08498
ENSG00000002834	-0.077953	8.467038	0.905601	1	LASP1	K15494
ENSG00000059122	-0.078403	5.960233	0.90568	1	FLYWCH1	_
ENSG00000122970	0.087511	4.665898	0.905747	1	IFT81	K19677
ENSG00000175874	-0.079404	5.130614	0.905767	1	CREG2	K05750
ENSG00000163510	-0.082498	5.062721	0.905804	1	CWC22	K13100
ENSG00000149716	0.088189	3.653395	0.905866	1	ORAOV1	K10408
ENSG00000116489	-0.077683	8.160818	0.906064	1	CAPZA1	K10364
ENSG00000157916	-0.078502	6.885875	0.90608	1	RER1	_
ENSG00000180758	-0.087933	4.126681	0.906089	1	GPR157	K08467
ENSG00000204160	-0.08067	4.372906	0.906305	1	ZDHHC18	K16675
ENSG00000130856	-0.098501	2.970022	0.90635	1	ZNF236	_
ENSG00000174951	-0.098501	2.970022	0.90635	1	FUT1	K00718
ENSG00000266173	-0.098501	2.970022	0.90635	1	STRADA	K08271
ENSG00000075826	0.084565	2.965353	0.90635	1	SEC31B	K14005
ENSG00000132612	-0.079238	6.113723	0.906429	1	VPS4A	K12196
ENSG00000111011	-0.078569	6.536048	0.90647	1	RSRC2	_
ENSG00000136235	0.09483	3.663717	0.906489	1	GPNMB	K02927
ENSG00000221909	0.09483	3.663717	0.906489	1	FAM200A	K09229
ENSG00000083223	-0.084126	5.074482	0.906517	1	ZCCHC6	K13291
ENSG00000169359	-0.079876	5.270071	0.906533	1	SLC33A1	K03372
ENSG00000107021	-0.08118	5.078374	0.906752	1	TBC1D13	_
ENSG00000116754	-0.076883	8.822166	0.906773	1	SRSF11	K12899
ENSG00000107949	-0.077943	6.846419	0.906803	1	BCCIP	K15262
ENSG00000101189	0.078958	5.777478	0.906808	1	MRGBP	K11343
ENSG00000111364	-0.079721	5.978424	0.906829	1	DDX55	K14809
ENSG00000116237	-0.078196	6.597551	0.906829	1	ICMT	K00587
ENSG00000129353	0.077941	6.661015	0.906856	1	SLC44A2	K15377
ENSG00000125970	0.07751	7.115419	0.906888	1	RALY	K12895
ENSG00000095787	0.077107	7.769188	0.906894	1	WAC	_
ENSG00000127554	-0.082954	5.148035	0.906896	1	GFER	K17783
ENSG00000090339	-0.077773	6.903928	0.906939	1	ICAM1	K06490
ENSG00000165714	-0.102109	3.249111	0.906965	1	LOH12CR1	_
ENSG00000183506	-0.102109	3.249111	0.906965	1	PI4KAP2	K00888
ENSG00000150165	0.085777	3.245196	0.906965	1	ANXA8L1	K17096
ENSG00000119403	0.078139	5.863632	0.906981	1	PHF19	K11486
ENSG00000051382	-0.078602	5.392015	0.906984	1	PIK3CB	K00922

ENSG00000151779	0.081144	4.384393	0.907064	1	NBAS	_
ENSG00000234608	0.081144	4.384393	0.907064	1	MAPKAPK5-AS1	_
ENSG00000124145	-0.077682	6.868349	0.907092	1	SDC4	K16338
ENSG00000148719	0.0812	5.448289	0.907092	1	DNAJB12	K09518
ENSG00000124702	0.077376	7.047728	0.907113	1	KLHDC3	_
ENSG00000112357	-0.116984	2.225185	0.907115	1	PEX7	K13341
ENSG00000163453	0.077794	6.532318	0.907202	1	IGFBP7	_
ENSG00000174307	-0.078452	6.092038	0.907282	1	PHLDA3	_
ENSG00000159592	0.077229	6.976704	0.907361	1	GPBP1L1	_
ENSG00000177150	0.081785	5.284602	0.907553	1	FAM210A	_
ENSG00000185127	-0.080395	4.695762	0.907596	1	C6orf120	_
ENSG00000187605	0.085628	4.501325	0.907614	1	TET3	_
ENSG00000127418	0.077692	5.703635	0.907681	1	FGFRL1	_
ENSG00000165282	0.081743	4.946198	0.907703	1	PIGO	K05288
ENSG00000104872	0.077243	6.621457	0.907749	1	PIH1D1	_
ENSG00000126858	0.078517	5.793386	0.90781	1	RHOT1	K07870
ENSG00000092964	-0.080491	5.022818	0.907812	1	DPYSL2	K07528
ENSG00000124508	0.109776	2.659299	0.907871	1	BTN2A2	K06712
ENSG00000262454	-0.145039	1.602006	0.907908	1	RP11-65J21.3	_
ENSG00000009844	-0.079684	5.995703	0.90795	1	VTA1	K12199
ENSG00000127922	0.077128	6.485101	0.908071	1	SHFM1	K10881
ENSG00000105227	-0.095526	3.494382	0.908084	1	PRX	_
ENSG00000135116	-0.095526	3.494382	0.908084	1	HRK	_
ENSG00000136710	-0.095526	3.494382	0.908084	1	CCDC115	K14561
ENSG00000136098	-0.087959	3.490356	0.908084	1	NEK3	K08857
ENSG00000187189	-0.087959	3.490356	0.908084	1	TSPYL4	K11287
ENSG00000101474	-0.077412	6.104837	0.908086	1	APMAP	_
ENSG00000101224	0.076232	7.536625	0.908099	1	CDC25B	K05866
ENSG00000100034	0.07803	5.92196	0.908106	1	PPM1F	_
ENSG00000166133	-0.081394	4.87528	0.908117	1	RPUSD2	_
ENSG00000107679	-0.077883	6.034346	0.908117	1	PLEKHA1	K17985
ENSG00000084774	0.076952	6.598551	0.908131	1	CAD	K11540
ENSG00000068383	0.082542	4.018101	0.908149	1	INPP5A	K01106
ENSG00000134590	0.078102	6.071009	0.90816	1	FAM127A	_
ENSG00000178035	-0.076288	7.491476	0.90817	1	IMPDH2	K00088
ENSG00000010803	-0.086617	4.402272	0.90818	1	SCMH1	K11461
ENSG00000080371	-0.077573	5.843691	0.908279	1	RAB21	K07890
ENSG00000158716	0.079602	5.57057	0.908282	1	DUSP23	K14165
ENSG00000136950	0.076742	6.673529	0.908288	1	ARPC5L	K05754
ENSG00000041880	0.093765	3.691161	0.908311	1	PARP3	K10798
ENSG00000144224	-0.076874	6.66866	0.908324	1	UBXN4	_
ENSG00000159596	-0.077297	6.325879	0.908345	1	TMEM69	K03327
ENSG00000057935	-0.076592	5.965157	0.908403	1	MTA3	K11660
ENSG00000171206	-0.077326	5.846742	0.908415	1	TRIM8	K12001
ENSG00000130332	-0.077546	6.209847	0.908416	1	LSM7	K12626
ENSG00000124641	0.084636	4.407291	0.908546	1	MED20	K13528
ENSG00000168924	0.078901	6.042008	0.908593	1	LETM1	K17800
ENSG00000188026	-0.080917	4.025244	0.908625	1	RILPL1	_
ENSG00000141934	-0.076192	7.110296	0.908625	1	PPAP2C	K01080
ENSG00000040531	-0.084605	4.517763	0.908639	1	CTNS	K12386
ENSG00000125534	-0.076079	7.07951	0.908793	1	PPDPF	_
ENSG00000196290	-0.07908	5.307083	0.908943	1	NIF3L1	_
ENSG00000123191	-0.088178	3.875055	0.90901	1	ATP7B	K17686
ENSG00000129534	-0.078609	5.974945	0.909024	1	MIS18BP1	_
ENSG00000139410	-0.101142	2.686476	0.90906	1	SDSL	K17989
ENSG00000147408	-0.078051	5.85661	0.909088	1	CSGALNACT1	_
ENSG00000101246	0.079841	4.805823	0.909128	1	ARFRP1	K07952
ENSG00000187145	-0.076595	6.348115	0.909154	1	MRPS21	K02970

ENSG00000130165	0.075691	5.427034	0.909192	1	ELOF1	_
ENSG00000184465	0.079221	4.300069	0.909221	1	WDR27	_
ENSG00000167981	-0.094274	3.014014	0.909242	1	ZNF597	K09228
ENSG00000170456	-0.077654	6.053615	0.909297	1	DENND5B	_
ENSG00000164300	0.174991	0.509994	0.909315	1	SERINC5	_
ENSG00000185189	0.174991	0.509994	0.909315	1	NRBP2	K08875
ENSG00000238273	0.174991	0.509994	0.909315	1	AC012360.6	_
ENSG00000267309	0.174991	0.509994	0.909315	1	CTD-2630F21.1	_
ENSG00000126562	-0.161511	0.567163	0.909315	1	WNK4	K08867
ENSG00000147576	-0.161511	0.567163	0.909315	1	ADHFE1	K11173
ENSG00000163467	-0.161511	0.567163	0.909315	1	TSACC	K06580
ENSG00000165609	0.075365	7.289274	0.909332	1	NUDT5	K13987
ENSG00000115825	0.075363	7.197805	0.909411	1	PRKD3	K06070
ENSG00000247556	0.075104	7.524995	0.909472	1	OIP5-AS1	_
ENSG00000157637	-0.075664	6.911968	0.909479	1	SLC38A10	K14996
ENSG00000167325	0.07462	8.425303	0.909599	1	RRM1	K10807
ENSG00000249915	0.074633	8.350797	0.909611	1	PDCD6	_
ENSG00000119915	0.084113	4.423741	0.909626	1	ELOVL3	K10248
ENSG00000110841	-0.075387	6.130099	0.909654	1	PPFIBP1	_
ENSG00000130764	0.078057	5.866643	0.909752	1	LRRC47	K04613
ENSG00000157456	-0.075124	7.253078	0.909779	1	CCNB2	K05868
ENSG00000172531	-0.075086	7.303626	0.90978	1	PPP1CA	K06269
ENSG00000089876	-0.076069	6.327521	0.909824	1	DHX32	K18994
ENSG00000143373	0.077518	5.646101	0.909894	1	ZNF687	_
ENSG00000074319	0.076526	5.546365	0.909899	1	TSG101	K12183
ENSG00000135976	0.085091	3.525963	0.910091	1	ANKRD36	K17299
ENSG00000178026	0.094217	2.694072	0.910218	1	FAM211B	_
ENSG00000172780	-0.137539	1.658374	0.910267	1	RAB43	K07930
ENSG00000173535	0.127703	1.629955	0.910267	1	TNFRSF10C	K04722
ENSG00000144730	-0.109615	1.642889	0.910267	1	IL17RD	K05167
ENSG00000204519	0.097436	1.645686	0.910267	1	ZNF551	K09228
ENSG00000261342	0.097436	1.645686	0.910267	1	AC006538.1	_
ENSG00000163322	0.095249	2.262976	0.910273	1	FAM175A	K04613
ENSG00000234311	0.095249	2.262976	0.910273	1	RP11-432J24.3	_
ENSG00000108799	0.080548	4.54147	0.910297	1	EZH1	K17451
ENSG00000175582	0.074674	7.02263	0.910404	1	RAB6A	K07893
ENSG00000138180	0.074506	7.232056	0.910419	1	CEP55	K16456
ENSG00000106554	0.073898	8.534866	0.910435	1	CHCHD3	K17563
ENSG00000138398	0.076636	5.919785	0.910517	1	PPIG	K09566
ENSG00000133835	-0.075136	6.5903	0.910533	1	HSD17B4	K12405
ENSG00000187109	-0.073532	9.497598	0.910667	1	NAP1L1	K11279
ENSG00000100036	-0.086569	3.316378	0.910882	1	SLC35E4	K15286
ENSG00000127586	-0.074827	6.315464	0.911028	1	CHTF18	K11269
ENSG00000104228	-0.085059	3.910437	0.911049	1	TRIM35	K12012
ENSG00000077684	-0.076859	5.713562	0.911063	1	JADE1	K11347
ENSG00000181666	0.092797	3.047017	0.911073	1	HKR1	K09228
ENSG00000112159	-0.07875	5.513116	0.911078	1	MDN1	K14572
ENSG00000073050	0.078192	4.749862	0.911087	1	XRCC1	K10803
ENSG00000182158	0.074287	6.596276	0.911352	1	CREB3L2	K09048
ENSG00000161791	0.084602	3.541123	0.911381	1	FMNL3	_
ENSG00000149212	0.076873	3.545014	0.911381	1	SESN3	K10141
ENSG00000128908	0.074775	5.463284	0.911469	1	INO80	K11665
ENSG00000136935	0.079025	4.458111	0.911712	1	GOLGA1	_
ENSG00000104522	-0.073995	6.683248	0.911781	1	TSTA3	K02377
ENSG00000177697	0.073077	7.626825	0.911859	1	CD151	K06537
ENSG00000054967	0.078004	4.343944	0.911865	1	RELT	K05156
ENSG00000114302	0.073253	5.525562	0.9119	1	PRKAR2A	K04739
ENSG00000148153	-0.078791	4.673593	0.912055	1	INIP	K17985

ENSG00000104626	-0.076929	5.360347	0.912153	1	ERI1	K18416
ENSG00000177700	-0.074135	6.324247	0.912163	1	POLR2L	K03007
ENSG00000145996	-0.077953	4.353715	0.91223	1	CDKAL1	K15865
ENSG00000103152	0.07546	5.908872	0.912314	1	MPG	K03652
ENSG00000159069	0.0732	6.879061	0.912332	1	FBXW5	K10263
ENSG00000079277	-0.078229	4.680446	0.912346	1	MKNK1	K04372
ENSG00000132793	0.082911	3.334922	0.912358	1	LPIN3	K15728
ENSG00000157500	-0.076334	5.78129	0.912405	1	APPL1	K08733
ENSG00000176853	-0.073594	6.551104	0.912452	1	FAM91A1	_
ENSG00000183458	0.124194	1.685649	0.912508	1	RP11-958N24.1	K04985
ENSG00000259583	0.124194	1.685649	0.912508	1	RP11-66B24.4	_
ENSG00000247137	-0.103744	1.697763	0.912508	1	RP11-727A23.5	_
ENSG00000185716	-0.088619	3.936485	0.912518	1	C16orf52	_
ENSG00000166847	-0.077899	5.426063	0.912585	1	DCTN5	K10427
ENSG00000198853	-0.076942	5.305974	0.912629	1	RUSC2	_
ENSG00000139687	-0.0738	6.035023	0.912694	1	RB1	K06618
ENSG00000137154	0.071693	10.80038	0.912723	1	RPS6	K02991
ENSG00000101639	0.079338	5.029157	0.912746	1	CEP192	K16725
ENSG00000273015	-0.088399	3.077594	0.912832	1	LINC00938	_
ENSG00000012048	-0.072522	6.182354	0.91284	1	BRCA1	K10605
ENSG00000176022	0.077538	4.361126	0.912952	1	B3GALT6	K00734
ENSG00000126267	0.072321	7.275168	0.913026	1	COX6B1	K02267
ENSG00000105819	-0.073013	6.617352	0.913059	1	PMPCB	K17732
ENSG00000138111	0.091248	3.347743	0.913078	1	TMEM180	K04613
ENSG00000264364	-0.073175	6.043025	0.913146	1	DYNLL2	K10418
ENSG00000164151	0.071888	7.581042	0.913329	1	KIAA0947	_
ENSG00000122126	-0.07507	5.797147	0.913345	1	OCRL	K01099
ENSG00000102974	-0.073756	5.31811	0.913375	1	CTCF	_
ENSG00000112685	-0.073301	5.799513	0.913478	1	EXOC2	K17637
ENSG00000111737	0.073434	6.047313	0.913482	1	RAB35	K07876
ENSG00000259758	0.072562	6.512255	0.913546	1	CASC7	_
ENSG00000142684	0.09123	3.088893	0.913685	1	ZNF593	K14821
ENSG00000172889	-0.07329	6.09205	0.913826	1	EGFL7	_
ENSG00000146592	0.229817	0.594615	0.913871	1	CREB5	K09047
ENSG00000171962	0.229817	0.594615	0.913871	1	LRRC48	_
ENSG00000196331	0.229817	0.594615	0.913871	1	HIST1H2BO	K11252
ENSG00000198225	0.229817	0.594615	0.913871	1	FKBP1C	K09568
ENSG00000230590	0.229817	0.594615	0.913871	1	FTX	_
ENSG00000173212	0.163224	0.628449	0.913871	1	MAB21L3	_
ENSG00000269947	0.163224	0.628449	0.913871	1	RP11-849F2.9	_
ENSG00000164117	-0.086602	3.959191	0.913941	1	FBXO8	K10294
ENSG00000173153	-0.073767	6.018211	0.914032	1	ESRRA	K08552
ENSG00000235552	0.071222	7.669245	0.914079	1	RPL6P27	K02934
ENSG00000127399	0.075374	4.971606	0.91408	1	LRRC61	_
ENSG00000130921	0.07453	5.390896	0.914112	1	C12orf65	_
ENSG00000072778	-0.07085	8.277914	0.914287	1	ACADVL	K09479
ENSG00000120963	-0.071693	6.877724	0.91432	1	ZNF706	_
ENSG00000114316	0.076858	4.386522	0.91436	1	USP4	K11835
ENSG00000169976	0.072206	6.099427	0.914362	1	SF3B5	K12832
ENSG00000132613	-0.076564	4.260704	0.914372	1	MTSS1L	_
ENSG00000119977	-0.07391	5.398263	0.914459	1	TCTN3	K19382
ENSG00000004660	0.073233	5.05843	0.914525	1	CAMKK1	K07359
ENSG00000184384	-0.106017	2.772582	0.914571	1	MAML2	K06061
ENSG00000233493	-0.105299	2.333186	0.914627	1	TMEM238	_
ENSG00000239415	-0.105299	2.333186	0.914627	1	AP001469.9	_
ENSG00000175701	0.092453	2.334356	0.914627	1	LINC00116	_
ENSG00000231742	-0.157018	1.727338	0.914639	1	RP11-112L6.4	_
ENSG00000247240	-0.157018	1.727338	0.914639	1	UBL7-AS1	_

ENSG00000169057	-0.130627	1.712637	0.914639	1	MECP2	K11588
ENSG00000178184	-0.130627	1.712637	0.914639	1	PARD6G	K06093
ENSG00000097033	-0.071113	7.191143	0.91469	1	SH3GLB1	K11248
ENSG00000139624	0.07155	5.676438	0.914701	1	CERS5	K04710
ENSG00000155368	-0.070992	7.18483	0.914843	1	DBI	K08762
ENSG00000131845	0.078196	3.976954	0.914863	1	ZNF304	K09228
ENSG00000171469	-0.074967	4.720893	0.914882	1	ZNF561	K09228
ENSG00000125149	0.083401	3.799233	0.914941	1	C16orf70	_
ENSG00000149308	0.077518	4.51295	0.914984	1	NPAT	_
ENSG00000134905	0.071002	6.877187	0.914992	1	CARS2	K01883
ENSG00000134030	-0.072136	4.990062	0.914999	1	CTIF	_
ENSG00000070831	-0.070872	7.154379	0.915017	1	CDC42	K04393
ENSG00000267194	-0.084688	3.613633	0.915039	1	RP1-193H18.2	_
ENSG00000130724	-0.071386	6.603448	0.915045	1	CHMP2A	K12191
ENSG00000163913	-0.078583	4.398063	0.915047	1	IFT122	K19656
ENSG00000160703	0.079988	4.273089	0.915121	1	NLRX1	K12653
ENSG00000025796	0.070443	7.429968	0.915182	1	SEC63	K09540
ENSG00000128881	0.07408	4.135151	0.91521	1	TTBK2	K08815
ENSG00000228223	0.074814	4.993807	0.915226	1	HCG11	_
ENSG00000162408	0.077312	4.520617	0.915298	1	NOL9	K06947
ENSG00000131323	0.075369	4.731826	0.915426	1	TRAF3	K03174
ENSG00000122643	-0.07117	5.962357	0.915435	1	NT5C3A	K01081
ENSG00000157693	-0.079481	4.281167	0.915491	1	C9orf91	_
ENSG00000075239	-0.070224	7.408009	0.915577	1	ACAT1	K00626
ENSG00000174327	-0.091052	2.791284	0.915594	1	SLC16A13	K08189
ENSG00000103550	-0.076561	4.141555	0.915615	1	KNOP1	_
ENSG00000225733	-0.07275	6.194976	0.915652	1	FGD5-AS1	_
ENSG00000165934	-0.071594	6.19438	0.915652	1	CPSF2	K14402
ENSG00000124535	-0.070524	6.912354	0.915692	1	WRNIP1	K07478
ENSG00000164105	-0.077106	4.735788	0.915695	1	SAP30	K19202
ENSG00000126062	0.070996	5.155173	0.915734	1	TMEM115	_
ENSG00000105202	0.069716	7.968495	0.915739	1	FBL	K14563
ENSG00000115966	-0.07191	4.91938	0.915761	1	ATF2	K04450
ENSG00000141367	0.069402	8.941282	0.915767	1	CLTC	K04646
ENSG00000129197	-0.072495	5.591584	0.915792	1	RPAIN	_
ENSG00000217555	0.077883	4.636263	0.915799	1	CKLF	_
ENSG00000240342	-0.069454	8.612176	0.915847	1	RPS2P5	K02981
ENSG00000149262	-0.075587	4.834353	0.915856	1	INTS4	K13141
ENSG00000182512	-0.072024	5.795573	0.91598	1	GLRX5	K07390
ENSG00000103005	-0.071045	6.201548	0.916043	1	USB1	_
ENSG00000117362	0.089751	3.129587	0.916148	1	APH1A	K06172
ENSG00000040933	0.075037	4.744976	0.916229	1	INPP4A	K01109
ENSG00000175110	-0.070103	5.976354	0.916237	1	MRPS22	K17401
ENSG00000134294	-0.06886	10.06907	0.916246	1	SLC38A2	K14207
ENSG00000166340	-0.07034	6.628925	0.916273	1	TPP1	K01279
ENSG00000023191	-0.069923	6.958208	0.916368	1	RNH1	K16634
ENSG00000105401	0.069078	8.06273	0.916467	1	CDC37	K09554
ENSG00000139734	-0.070255	6.50793	0.916551	1	DIAPH3	K05745
ENSG00000131876	0.069586	7.007685	0.916574	1	SNRPA1	K11092
ENSG00000135547	0.077233	2.805384	0.916593	1	HEY2	K09091
ENSG00000120333	0.073946	4.652072	0.916651	1	MRPS14	K02954
ENSG00000100147	0.120952	1.739268	0.916668	1	CCDC134	_
ENSG00000166762	0.120952	1.739268	0.916668	1	CATSPER2	K16890
ENSG00000012124	0.093029	1.753778	0.916668	1	CD22	K06467
ENSG00000026036	0.093029	1.753778	0.916668	1	RTEL1-TNFRSF6B	K11136
ENSG00000111325	0.093029	1.753778	0.916668	1	OGFOD2	_
ENSG00000116984	0.073474	4.933358	0.916696	1	MTR	K00548
ENSG00000095261	-0.070227	6.429735	0.916706	1	PSMD5	K06692

ENSG00000127914	0.072023	5.495732	0.91671	1	AKAP9	K16551
ENSG00000142687	0.072023	5.495732	0.91671	1	KIAA0319L	_
ENSG00000145990	0.071222	6.027269	0.91684	1	GFOD1	K12581
ENSG00000160932	-0.068628	8.559119	0.916869	1	LY6E	K06846
ENSG00000131373	-0.07337	5.715235	0.916916	1	HACL1	K12261
ENSG00000040199	0.075971	3.83972	0.916943	1	PHLPP2	K16340
ENSG00000169016	0.071742	4.012792	0.917086	1	E2F6	K09390
ENSG00000140265	0.072914	5.178374	0.917119	1	ZSCAN29	K09230
ENSG00000084090	-0.068803	7.551169	0.917187	1	STARD7	_
ENSG00000119048	-0.06959	6.59123	0.917233	1	UBE2B	K10574
ENSG00000125450	0.069275	6.700748	0.917279	1	NUP85	K14304
ENSG00000144580	-0.072372	5.909361	0.917307	1	RQCD1	K09228
ENSG00000124659	-0.072699	5.183437	0.917313	1	TBCC	K18034
ENSG00000099341	0.068891	7.103307	0.917325	1	PSMD8	K03031
ENSG00000164808	-0.070926	5.564048	0.917367	1	SPIDR	_
ENSG00000128585	-0.068873	5.955313	0.917447	1	MKLN1	_
ENSG00000063241	-0.069316	6.594864	0.917558	1	ISOC2	_
ENSG00000140688	-0.070267	4.863241	0.917575	1	C16orf58	_
ENSG00000139218	-0.0688	7.000941	0.917681	1	SCAF11	_
ENSG00000132664	-0.074622	4.4471	0.917689	1	POLR3F	K03025
ENSG00000205809	0.078859	3.154673	0.917714	1	KLRC2	K06541
ENSG00000167967	0.070921	5.778268	0.917733	1	E4F1	K09193
ENSG00000136381	0.069286	6.231397	0.917761	1	IREB2	_
ENSG00000123094	0.069237	6.340445	0.917811	1	RASSF8	K09855
ENSG00000167695	0.072573	5.19284	0.91789	1	FAM57A	_
ENSG00000238227	-0.069352	6.004627	0.917907	1	C9orf69	_
ENSG00000142676	0.067485	9.69901	0.917946	1	RPL11	K02868
ENSG00000218227	-0.200742	0.711721	0.917982	1	RP11-889L3.1	K02885
ENSG00000105700	0.070687	6.051282	0.918232	1	KXD1	_
ENSG00000169967	0.070687	6.051282	0.918232	1	MAP3K2	K04420
ENSG00000012171	-0.077859	4.457136	0.918324	1	SEMA3B	_
ENSG00000102007	0.075346	3.864033	0.918384	1	PLP2	_
ENSG00000171227	-0.070703	3.865846	0.918384	1	TMEM37	_
ENSG00000068400	-0.079681	3.177725	0.918475	1	GRIPAP1	_
ENSG00000135604	0.078312	3.174252	0.918475	1	STX11	K08487
ENSG00000125871	-0.072751	5.052167	0.918489	1	MGME1	K19465
ENSG00000159210	-0.068264	6.843025	0.918508	1	SNF8	K12188
ENSG00000069702	0.06849	6.475738	0.918518	1	TGFBR3	K05843
ENSG00000020181	-0.086534	2.840975	0.918522	1	GPR124	K08461
ENSG00000174738	0.070645	5.639072	0.918528	1	NR1D2	K08531
ENSG00000143190	-0.069915	4.785886	0.918549	1	POU2F1	K09364
ENSG00000071539	0.067193	8.514577	0.918574	1	TRIP13	K17985
ENSG00000249471	0.089921	2.402366	0.918577	1	ZNF324B	K09228
ENSG00000236814	-0.093294	1.801652	0.918602	1	RP11-446E9.1	K03064
ENSG00000140988	-0.06682	11.30587	0.918617	1	RPS2	K02981
ENSG00000135297	0.069447	5.694339	0.918649	1	MTO1	K03495
ENSG00000145439	-0.075161	4.334264	0.918678	1	CBR4	K11539
ENSG00000184110	0.067835	5.410629	0.91875	1	EIF3C	K03252
ENSG00000198189	-0.069309	5.347478	0.918775	1	HSD17B11	_
ENSG00000198964	0.069375	5.697724	0.918783	1	SGMS1	_
ENSG00000179151	-0.067853	6.179949	0.918902	1	EDC3	K12615
ENSG00000214022	0.067508	7.201649	0.918915	1	REPIN1	K09228
ENSG00000101752	0.068553	6.025918	0.91909	1	MIB1	K10645
ENSG00000153786	-0.069474	5.984687	0.919116	1	ZDHHC7	K18932
ENSG00000197483	-0.073141	4.054485	0.919196	1	ZNF628	K09229
ENSG00000182944	-0.066779	8.343517	0.919196	1	EWSR1	K13209
ENSG00000103121	-0.068035	6.498755	0.919247	1	CMC2	K18172
ENSG00000185386	-0.068513	5.757184	0.919275	1	MAPK11	K04441

ENSG00000143207	-0.070735	5.357138	0.919282	1	RFWD2	K10143
ENSG00000163344	-0.076575	4.700811	0.919372	1	PMVK	K13273
ENSG00000156976	-0.066325	9.445651	0.919422	1	EIF4A2	K03257
ENSG00000089177	-0.08439	2.865196	0.919453	1	KIF16B	K17916
ENSG00000177311	0.067468	6.706634	0.919457	1	ZBTB38	K10510
ENSG00000096384	-0.066034	11.28796	0.919571	1	HSP90AB1	K04079
ENSG00000147905	0.071803	5.226039	0.919757	1	ZCCHC7	K12597
ENSG00000174529	-0.095245	2.433699	0.919814	1	TMEM81	_
ENSG00000234602	-0.095245	2.433699	0.919814	1	MCIDAS	K10749
ENSG00000105655	0.088743	2.435204	0.919814	1	ISYNA1	K01858
ENSG00000164010	0.088743	2.435204	0.919814	1	ERMAP	K06712
ENSG00000196787	0.088743	2.435204	0.919814	1	HIST1H2AG	K11251
ENSG00000107281	0.066681	6.119745	0.919837	1	NPDC1	K01509
ENSG00000037042	-0.08421	3.700506	0.919994	1	TUBG2	K10389
ENSG00000069345	-0.067551	6.338964	0.920087	1	DNAJA2	K09503
ENSG00000171863	0.065838	8.679864	0.920165	1	RPS7	K02993
ENSG00000154978	-0.068286	5.670318	0.920166	1	VOPP1	_
ENSG00000087269	0.067205	6.387835	0.920196	1	NOP14	K14766
ENSG00000163602	0.070886	5.160089	0.920219	1	RYBP	K11469
ENSG00000099785	0.080465	3.896735	0.92023	1	2-Mar	K10657
ENSG00000258634	0.074445	3.899749	0.92023	1	RP4-773N10.4	_
ENSG00000160183	-0.070046	5.500582	0.920257	1	TMPRSS3	K09634
ENSG00000196584	-0.070046	5.500582	0.920257	1	XRCC2	K10879
ENSG00000092470	0.067265	5.439221	0.920345	1	WDR76	K02159
ENSG00000177519	0.067265	5.439221	0.920345	1	RPRM	K10128
ENSG00000144320	0.068851	4.365453	0.920346	1	KIAA1715	_
ENSG00000153046	0.066611	6.832832	0.920352	1	CDYL	K00653
ENSG00000115129	0.087659	2.872507	0.920363	1	TP53I3	K10133
ENSG00000083093	-0.071508	4.075425	0.920411	1	PALB2	K10897
ENSG00000161999	-0.070428	5.443293	0.920501	1	JMJD8	K09561
ENSG00000001631	0.070008	4.823011	0.920506	1	KRIT1	K17705
ENSG00000157353	-0.074298	4.230641	0.920555	1	FUK	K05305
ENSG00000183770	-0.074298	4.230641	0.920555	1	FOXL2	K09405
ENSG00000152061	0.071784	4.231728	0.920555	1	RABGAP1L	_
ENSG00000147874	0.066183	7.042697	0.92066	1	HAUS6	K16589
ENSG00000070159	0.068994	5.007525	0.920662	1	PTPN3	K18027
ENSG00000116793	-0.073091	4.826639	0.920744	1	PHTF1	_
ENSG00000196943	-0.070096	4.825082	0.920744	1	NOP9	K14790
ENSG00000078070	-0.06905	4.237585	0.920911	1	MCCC1	K01968
ENSG00000256223	0.104264	2.458769	0.921013	1	ZNF10	K09228
ENSG00000168268	-0.066471	6.061537	0.921055	1	NT5DC2	_
ENSG00000131462	-0.066029	6.98235	0.921055	1	TUBG1	K10389
ENSG00000226328	0.074642	4.623878	0.921123	1	CTA-217C2.1	_
ENSG00000085662	0.065442	7.582383	0.921138	1	AKR1B1	K00011
ENSG00000137936	-0.065756	7.156862	0.921208	1	BCAR3	_
ENSG00000138193	-0.093891	2.895236	0.921253	1	PLCE1	K05860
ENSG00000001084	-0.068235	4.929476	0.921254	1	GCLC	K11204
ENSG00000206538	-0.067849	6.146919	0.921286	1	VGLL3	_
ENSG00000175220	-0.070363	5.182226	0.921342	1	ARHGAP1	K18470
ENSG00000176619	-0.065486	7.37302	0.921345	1	LMNB2	K07611
ENSG00000141198	-0.068408	5.691681	0.921352	1	TOM1L1	K02258
ENSG00000100614	-0.069564	5.255213	0.921366	1	PPM1A	K04457
ENSG00000122335	0.086396	3.22656	0.921383	1	SERAC1	K04135
ENSG00000172216	0.064977	8.141877	0.921405	1	CEBPB	K10048
ENSG00000047932	-0.066043	6.663086	0.921422	1	GOPC	K05088
ENSG00000091073	-0.070223	4.739045	0.921423	1	DTX2	K06058
ENSG00000242259	-0.070223	4.739045	0.921423	1	C22orf39	K17675
ENSG00000167523	0.0861	3.495156	0.921464	1	SPATA33	K02927

ENSG00000184207	-0.06609	6.551129	0.921521	1	PGP	K19269
ENSG00000005339	0.06881	5.462291	0.921576	1	CREBBP	K04498
ENSG00000088992	0.07136	4.250291	0.921614	1	TESC	K17612
ENSG00000112695	-0.065275	7.32379	0.921641	1	COX7A2	K02270
ENSG00000147437	0.153331	0.737896	0.92171	1	GNRH1	K05252
ENSG00000181135	0.153331	0.737896	0.92171	1	ZNF707	K09228
ENSG00000183032	0.153331	0.737896	0.92171	1	SLC25A21	K15110
ENSG00000236850	0.153331	0.737896	0.92171	1	BMS1P20	_
ENSG00000111837	-0.130204	0.785177	0.92171	1	MAK	K08829
ENSG00000167984	-0.130204	0.785177	0.92171	1	NLRC3	_
ENSG00000205534	-0.130204	0.785177	0.92171	1	RP11-345J4.8	K08873
ENSG00000108958	0.094839	0.768291	0.92171	1	AC016292.3	K00236
ENSG00000105127	0.068152	5.587087	0.921734	1	AKAP8	K16525
ENSG00000134851	0.065367	6.816572	0.921875	1	TMEM165	_
ENSG00000112659	0.068391	4.748272	0.92192	1	CUL9	K11970
ENSG00000175354	0.067389	5.532213	0.922019	1	PTPN2	K18026
ENSG00000115310	0.064513	7.925265	0.922069	1	RTN4	_
ENSG00000204588	-0.084094	3.239481	0.922078	1	LINC01123	_
ENSG00000066136	-0.067899	5.652124	0.922135	1	NFYC	K08066
ENSG00000122257	0.065358	6.122893	0.92214	1	RBBP6	K10624
ENSG00000144426	0.067262	5.952724	0.922181	1	NBEAL1	_
ENSG00000155158	-0.112802	1.864224	0.922212	1	TTC39B	_
ENSG00000157578	-0.112802	1.864224	0.922212	1	LCA5L	_
ENSG00000234585	-0.112802	1.864224	0.922212	1	CCT6P3	K09498
ENSG00000049246	0.089239	1.854325	0.922212	1	PER3	K02633
ENSG00000122557	-0.072275	4.527227	0.922224	1	HERPUD2	K17985
ENSG00000179091	-0.06429	8.278657	0.922244	1	CYC1	K00413
ENSG00000150776	-0.067223	5.346399	0.922375	1	C11orf57	_
ENSG00000125503	0.067038	5.344147	0.922375	1	PPP1R12C	K17457
ENSG00000147050	0.06774	3.937536	0.922422	1	KDM6A	K11447
ENSG00000168273	0.06774	3.937536	0.922422	1	SMIM4	_
ENSG00000163870	0.06495	6.752102	0.922452	1	TPRA1	_
ENSG00000213178	0.065419	5.81535	0.922456	1	RP11-641D5.1	K02891
ENSG00000178127	-0.069243	4.410421	0.922571	1	NDUFV2	K03943
ENSG00000138621	-0.077782	3.517241	0.922613	1	PPCDC	K01598
ENSG00000151090	-0.077782	3.517241	0.922613	1	THRB	K08362
ENSG00000189334	0.069837	3.518584	0.922613	1	S100A14	_
ENSG00000089234	0.06687	5.352761	0.922699	1	BRAP	K10632
ENSG00000196502	0.06942	4.117614	0.922735	1	SULT1A1	K01014
ENSG00000100926	-0.082502	3.257902	0.922761	1	TM9SF1	K17085
ENSG00000171202	-0.066127	4.957961	0.922762	1	TMEM126A	K18157
ENSG00000104904	0.063566	9.337174	0.922765	1	OAZ1	K16548
ENSG00000063322	0.064624	5.771852	0.922842	1	MED29	K15142
ENSG00000160294	0.064932	6.453873	0.922849	1	MCM3AP	_
ENSG00000228716	0.066671	6.053257	0.92286	1	DHFR	K00287
ENSG00000178971	0.078341	3.751246	0.922988	1	CTC1	_
ENSG00000183207	0.063816	7.722314	0.923025	1	RUVBL2	K11338
ENSG00000130813	0.074328	4.125128	0.923109	1	C19orf66	_
ENSG00000123739	0.067993	4.767729	0.923135	1	PLA2G12A	K01047
ENSG00000116580	-0.068439	4.965004	0.923182	1	GON4L	_
ENSG00000005893	-0.063959	7.303679	0.923253	1	LAMP2	K06528
ENSG00000115421	-0.075638	3.953417	0.923266	1	PAPOLG	K14376
ENSG00000162961	0.0646	6.415843	0.923304	1	DPY30	K14965
ENSG00000163191	0.063158	9.053161	0.92332	1	S100A11	_
ENSG00000088832	0.063518	7.832653	0.923324	1	FKBP1A	K09568
ENSG00000027697	0.065231	6.260862	0.923329	1	IFNGR1	K05132
ENSG00000113360	-0.06419	6.9091	0.923363	1	DROSHA	K03685
ENSG00000166411	-0.066427	5.930372	0.923392	1	IDH3A	K00030

ENSG00000241258	-0.068579	5.140572	0.923403	1	CRCP	K02927
ENSG00000111725	0.067654	4.423853	0.923487	1	PRKAB1	K07199
ENSG00000189241	-0.064488	6.520898	0.923503	1	TSPYL1	K11284
ENSG00000165629	-0.063269	8.215904	0.92351	1	ATP5C1	K02136
ENSG00000163349	0.064164	6.189321	0.923589	1	HIPK1	K08826
ENSG00000130731	-0.063664	7.260298	0.923648	1	C16orf13	_
ENSG00000105376	0.065946	5.787029	0.923676	1	ICAM5	K06769
ENSG00000135821	-0.074736	3.964684	0.923681	1	GLUL	K01915
ENSG00000213699	-0.068357	5.301526	0.923745	1	SLC35F6	K17985
ENSG00000085552	-0.089591	2.941531	0.923807	1	IGSF9	_
ENSG00000057608	-0.062945	8.429598	0.923813	1	GDI2	K17255
ENSG00000143612	-0.062871	8.521469	0.923869	1	C1orf43	_
ENSG00000179979	0.112554	1.889096	0.923899	1	CRIPAK	K11835
ENSG00000227354	0.112554	1.889096	0.923899	1	RBM26-AS1	_
ENSG00000270145	0.112554	1.889096	0.923899	1	CTD-2267D19.6	_
ENSG00000175711	-0.107666	1.911428	0.923899	1	B3GNTL1	_
ENSG00000177096	-0.107666	1.911428	0.923899	1	FAM109B	K01204
ENSG00000189120	0.087536	1.902085	0.923899	1	SP6	K09196
ENSG00000232931	-0.084271	1.898594	0.923899	1	LINC00342	_
ENSG00000014138	0.064769	6.155259	0.923912	1	POLA2	K02321
ENSG00000135048	0.063593	6.87342	0.923963	1	TMEM2	K17429
ENSG00000126003	0.066757	4.981384	0.924009	1	PLAGL2	K19486
ENSG00000177119	0.063582	6.829737	0.924023	1	ANO6	K19500
ENSG00000145012	0.064721	5.574252	0.924251	1	LPP	K16676
ENSG00000105186	-0.064482	6.12448	0.924343	1	ANKRD27	_
ENSG00000004766	-0.070878	4.894534	0.924368	1	CCDC132	_
ENSG00000070367	0.066515	5.577013	0.924386	1	EXOC5	_
ENSG00000099617	0.085513	2.529454	0.924406	1	EFNA2	K05462
ENSG00000187514	0.062068	10.34993	0.924447	1	PTMA	K13784
ENSG00000140299	-0.063558	6.649185	0.924448	1	BNIP2	K18448
ENSG00000069812	0.069651	4.688276	0.924498	1	HES2	K09087
ENSG00000167107	0.066949	5.455309	0.924612	1	ACSF2	K00666
ENSG00000069020	-0.076503	2.958222	0.924622	1	MAST4	K08789
ENSG00000125485	0.064431	5.243524	0.924699	1	DDX31	K14806
ENSG00000051180	0.063415	6.08849	0.9247	1	RAD51	K04482
ENSG00000143412	0.075319	3.28645	0.924739	1	ANXA9	K17090
ENSG00000272734	0.066111	3.291125	0.924739	1	ADIRF-AS1	_
ENSG00000171720	-0.063667	6.332461	0.924791	1	HDAC3	K11404
ENSG00000226976	-0.074126	3.562421	0.924813	1	COX6A1P2	K02266
ENSG00000136280	-0.065976	4.9983	0.924819	1	CCM2	_
ENSG00000136874	-0.076548	3.791876	0.924863	1	STX17	K08491
ENSG00000153339	-0.065447	5.248304	0.924868	1	TRAPPC8	K14572
ENSG00000146007	-0.062886	6.967726	0.924876	1	ZMAT2	K12848
ENSG00000113194	-0.063342	6.175114	0.924902	1	FAF2	K18726
ENSG00000186001	0.069959	4.313441	0.924949	1	LRCH3	_
ENSG00000189343	0.062726	6.92487	0.92496	1	RPS2P46	K02981
ENSG00000178691	0.062957	6.567385	0.925086	1	SUZ12	K02927
ENSG00000008256	-0.066034	5.174956	0.925207	1	CYTH3	K18441
ENSG00000115816	-0.062439	7.160253	0.925224	1	CEBPZ	K14832
ENSG00000142937	0.061362	11.03012	0.925255	1	RPS8	K02995
ENSG00000175224	0.063075	6.319707	0.92529	1	ATG13	K08331
ENSG00000085511	-0.072958	3.577173	0.925343	1	MAP3K4	K04428
ENSG00000085276	-0.069309	3.307204	0.925376	1	MECOM	K04462
ENSG00000093009	0.06276	6.522757	0.925383	1	CDC45	K06628
ENSG00000170043	0.063549	5.920488	0.925386	1	TRAPPC1	_
ENSG00000143409	0.072992	2.971253	0.925419	1	FAM63A	_
ENSG00000164296	0.072992	2.971253	0.925419	1	TIGD6	_
ENSG00000006062	-0.066288	4.916462	0.925434	1	MAP3K14	_

ENSG00000163945	0.110123	1.935764	0.925514	1	UVSSA	_
ENSG00000224914	0.110123	1.935764	0.925514	1	LINC00863	_
ENSG00000100393	0.065549	5.182015	0.925558	1	EP300	K04498
ENSG00000100023	-0.065329	5.184631	0.925558	1	PPIL2	K10598
ENSG00000140262	-0.06336	5.97288	0.925581	1	TCF12	K15603
ENSG00000176261	0.063732	5.408566	0.925647	1	ZBTB8OS	K17985
ENSG00000167969	-0.062301	6.883715	0.925678	1	ECI1	K13238
ENSG00000149541	0.06546	5.186841	0.925732	1	B3GAT3	K10158
ENSG00000057757	-0.065085	5.541635	0.925738	1	PITHD1	_
ENSG00000169727	0.061874	7.180609	0.925758	1	GPS1	K12175
ENSG00000149483	-0.062994	5.544456	0.925873	1	TMEM138	K17985
ENSG00000139436	-0.070939	4.332057	0.925895	1	GIT2	K12487
ENSG00000146828	-0.06498	5.189445	0.925905	1	SLC12A9	K14429
ENSG00000101126	-0.062343	6.653986	0.925911	1	ADNP	_
ENSG00000177045	-0.07017	4.60314	0.926029	1	SIX5	K19474
ENSG00000204843	-0.061727	7.204922	0.926045	1	DCTN1	K04648
ENSG00000140983	-0.061833	6.952589	0.926168	1	RHOT2	K07871
ENSG00000171453	-0.064702	5.672041	0.926187	1	POLR1C	K03027
ENSG00000171729	0.083852	2.987659	0.9262	1	TMEM51	_
ENSG00000121454	0.075708	3.589438	0.926382	1	LHX4	K09374
ENSG00000103343	-0.063909	4.482766	0.926389	1	ZNF174	K09230
ENSG00000178252	0.063592	6.078138	0.926401	1	WDR6	_
ENSG00000131269	0.06395	5.617784	0.926486	1	ABCB7	K05662
ENSG00000116030	0.060908	7.801516	0.926508	1	SUMO1	K12160
ENSG00000243989	-0.098458	2.56563	0.92651	1	ACY1	K14677
ENSG00000180185	-0.063151	5.280243	0.926515	1	FAHD1	K01557
ENSG00000100991	0.061427	6.915447	0.926543	1	TRPC4AP	K11796
ENSG00000110719	0.061544	6.124771	0.926544	1	TCIRG1	K02154
ENSG00000108094	0.062671	6.206646	0.926552	1	CUL2	K03870
ENSG00000131100	0.06095	7.635393	0.926553	1	ATP6V1E1	K02150
ENSG00000172831	-0.069163	4.8374	0.926563	1	CES2	K03927
ENSG00000150961	0.062238	6.081378	0.926586	1	SEC24D	K14007
ENSG00000050130	-0.062131	5.845239	0.926624	1	JKAMP	_
ENSG00000196233	-0.073549	3.829334	0.926649	1	LCOR	_
ENSG00000239382	-0.073549	3.829334	0.926649	1	ALKBH6	K10768
ENSG00000139620	0.063713	5.993225	0.926678	1	KANSL2	K18401
ENSG00000132305	-0.060901	7.654701	0.926695	1	IMMT	K17785
ENSG00000156471	0.060866	7.52063	0.926727	1	PTDSS1	K08729
ENSG00000126261	-0.060711	7.900197	0.926775	1	UBA2	K10685
ENSG00000110713	0.060849	7.457638	0.92679	1	NUP98	K14297
ENSG00000073536	-0.065012	5.285875	0.926836	1	NLE1	K14855
ENSG00000117632	-0.060407	8.570283	0.926843	1	STMN1	K04381
ENSG00000100426	0.062191	5.500585	0.926884	1	ZBED4	_
ENSG00000174917	0.066574	5.127839	0.927032	1	C19orf70	_
ENSG00000140543	-0.102866	1.957145	0.927062	1	DET1	K10571
ENSG00000076650	0.069983	3.830577	0.927082	1	GPATCH1	K13123
ENSG00000001497	-0.060831	7.138954	0.927193	1	LAS1L	K16912
ENSG00000178935	-0.075124	3.346646	0.927225	1	ZNF552	K09228
ENSG00000177200	0.067414	4.627469	0.927299	1	CHD9	K14438
ENSG00000115365	0.060406	7.453887	0.927331	1	LANCL1	_
ENSG00000148835	0.06552	5.046551	0.927335	1	TAF5	K03130
ENSG00000038295	0.061417	5.135357	0.927385	1	TLL1	K09608
ENSG00000175390	-0.060083	8.19767	0.927386	1	EIF3F	K03249
ENSG00000167461	0.062858	5.575172	0.927464	1	RAB8A	K07901
ENSG00000159256	0.064437	4.954761	0.927477	1	MORC3	_
ENSG00000226085	-0.095613	2.594877	0.927519	1	UQCRFS1P1	K00411
ENSG00000198105	-0.068799	4.041185	0.927597	1	ZNF248	K09228
ENSG00000272068	-0.07122	3.024268	0.927714	1	RP11-284F21.9	_

ENSG00000089327	-0.060198	7.301753	0.927814	1	FXYD5	K13362
ENSG00000119760	-0.06286	5.919553	0.927816	1	SUPT7L	K11316
ENSG00000124074	0.073564	3.356674	0.927821	1	ENKD1	_
ENSG00000119777	0.060891	6.388863	0.927828	1	TMEM214	_
ENSG00000108344	0.060116	7.176664	0.927893	1	PSMD3	K03033
ENSG00000165671	-0.061898	5.870189	0.927909	1	NSD1	K15588
ENSG00000180818	0.061784	6.109605	0.927949	1	HOXC10	K17444
ENSG00000242071	0.059794	7.623646	0.927962	1	RPL7AP6	K02936
ENSG00000120647	0.062171	6.237206	0.928049	1	CCDC77	K16757
ENSG00000140319	-0.059491	8.278543	0.92807	1	SRP14	K03104
ENSG00000196072	0.061184	5.150257	0.928082	1	BLOC1S2	K16750
ENSG00000152475	0.189768	0.907761	0.928218	1	ZNF837	K09228
ENSG00000176919	0.189768	0.907761	0.928218	1	C8G	K03999
ENSG00000227855	0.189768	0.907761	0.928218	1	DPY19L2P3	_
ENSG00000260401	0.189768	0.907761	0.928218	1	RP11-800A3.4	_
ENSG00000148296	-0.166588	0.910136	0.928218	1	SURF6	_
ENSG00000221817	-0.166588	0.910136	0.928218	1	RP11-137L10.6	_
ENSG00000268403	-0.166588	0.910136	0.928218	1	AC132192.1	_
ENSG00000136378	0.13762	0.934613	0.928218	1	ADAMTS7	K08622
ENSG00000187838	0.13762	0.934613	0.928218	1	TMEM256-PLSCR3	_
ENSG00000259172	0.13762	0.934613	0.928218	1	RP11-299G20.2	_
ENSG00000064607	0.060593	6.338253	0.928263	1	SUGP2	K13166
ENSG00000057663	-0.059408	6.282095	0.92829	1	ATG5	K08339
ENSG00000213930	-0.068517	3.634721	0.928376	1	GALT	K00965
ENSG00000267080	-0.082061	3.368171	0.928407	1	ASB16-AS1	_
ENSG00000123200	0.059723	6.245318	0.928455	1	ZC3H13	_
ENSG00000214944	-0.064902	4.974787	0.928458	1	ARHGEF28	K16529
ENSG00000163468	-0.058866	9.276941	0.928518	1	CCT3	K09495
ENSG00000175606	-0.061953	6.0317	0.928593	1	TMEM70	K17966
ENSG00000170852	-0.062296	5.885844	0.928638	1	KBTBD2	K10470
ENSG00000135862	0.059319	7.424306	0.92867	1	LAMC1	K05635
ENSG00000163159	-0.059936	6.079816	0.92867	1	VPS72	K11664
ENSG00000142733	-0.067215	4.062319	0.928695	1	MAP3K6	K04425
ENSG00000160714	-0.0598	6.821125	0.92878	1	UBE2Q1	K10582
ENSG00000123268	-0.059723	6.86692	0.92882	1	ATF1	K09053
ENSG00000106049	0.059697	6.720399	0.928847	1	HIBADH	K00020
ENSG00000147604	-0.058459	10.15858	0.92887	1	RPL7	K02937
ENSG00000123352	-0.06021	6.350852	0.928941	1	SPATS2	_
ENSG00000083642	-0.060936	5.404482	0.928964	1	PDS5B	K11267
ENSG00000183853	0.059854	6.458354	0.928985	1	KIRREL	_
ENSG00000149311	-0.06397	5.33123	0.929005	1	ATM	K04728
ENSG00000130520	-0.058679	8.339374	0.92903	1	LSM4	K12623
ENSG00000095906	0.059734	6.533678	0.929031	1	NUBP2	K03593
ENSG00000068796	-0.060652	5.729364	0.929037	1	KIF2A	K10393
ENSG00000142166	-0.061367	5.670323	0.929073	1	IFNAR1	K05130
ENSG00000187555	0.059421	6.796167	0.929098	1	USP7	K11838
ENSG00000130511	0.059599	6.607065	0.929101	1	SSBP4	_
ENSG00000206053	0.058787	7.694814	0.929142	1	HN1L	_
ENSG00000182718	0.058131	10.5232	0.929219	1	ANXA2	K17092
ENSG00000177674	0.063212	4.664287	0.929243	1	AGTRAP	_
ENSG00000138942	0.063266	5.173535	0.929269	1	RNF185	K10666
ENSG00000099381	0.061081	5.792573	0.929333	1	SETD1A	K11422
ENSG00000162227	-0.074278	3.652351	0.929333	1	TAF6L	K03131
ENSG00000198816	0.060847	6.00009	0.929369	1	ZNF358	K09228
ENSG00000161217	-0.062463	5.551952	0.929411	1	PCYT1A	K00968
ENSG00000170421	0.057862	11.46544	0.929489	1	KRT8	K07604
ENSG00000184182	-0.064728	4.897459	0.929498	1	UBE2F	K10687
ENSG00000148672	-0.058483	7.833731	0.929518	1	GLUD1	K00261

ENSG00000167671	0.059278	6.56552	0.929542	1	UBXN6	K14011
ENSG00000175575	-0.066363	4.790632	0.929598	1	PAAF1	K11887
ENSG00000125352	-0.062923	5.002401	0.929786	1	RNF113A	K13127
ENSG00000084676	-0.066427	3.662658	0.929802	1	NCOA1	K09101
ENSG00000131503	-0.067959	3.066683	0.929872	1	ANKHD1	K16726
ENSG00000144736	0.071152	4.259484	0.9299	1	SHQ1	K14764
ENSG00000108352	0.105715	2.024795	0.929971	1	RAPGEFL1	K08019
ENSG00000135164	-0.059946	5.69084	0.930011	1	DMTF1	_
ENSG00000138468	-0.079232	3.401698	0.93011	1	SENP7	K08596
ENSG00000232187	-0.079232	3.401698	0.93011	1	FTH1P7	K00522
ENSG00000149273	-0.057277	10.59998	0.930264	1	RPS3	K02985
ENSG00000176087	-0.059254	6.284347	0.930338	1	SLC35A4	K15273
ENSG00000106636	0.058369	6.815376	0.930352	1	YKT6	K08516
ENSG00000145331	0.081806	2.646237	0.930385	1	TRMT10A	K15445
ENSG00000127329	-0.076478	2.644226	0.930385	1	PTPRB	K05694
ENSG00000196204	0.066197	4.689993	0.930407	1	RNF216P1	K11976
ENSG00000108946	-0.057865	7.608287	0.93041	1	PRKAR1A	K04739
ENSG00000140463	0.066318	4.27093	0.930514	1	BBS4	K16531
ENSG00000160953	-0.058918	5.976374	0.93056	1	MUM1	_
ENSG00000158615	-0.057214	8.972371	0.930599	1	PPP1R15B	K17558
ENSG00000271270	0.072355	3.407184	0.93066	1	TMCC1-AS1	_
ENSG00000108669	-0.058388	6.121348	0.930685	1	CYTH1	K18441
ENSG00000153207	0.057815	7.166918	0.930691	1	AHCTF1	_
ENSG00000133612	-0.058481	6.566502	0.930706	1	AGAP3	K12491
ENSG00000120942	-0.069146	4.106278	0.930794	1	UBIAD1	K18623
ENSG00000127314	-0.058189	6.689267	0.930894	1	RAP1B	K07836
ENSG00000171466	0.059098	5.291362	0.930904	1	ZNF562	K09228
ENSG00000089220	-0.057242	8.053967	0.930906	1	PEBP1	K04257
ENSG00000159259	-0.057978	5.7685	0.930927	1	CHAF1B	K10751
ENSG00000261040	0.059112	4.929088	0.931073	1	CTD-2319I12.1	_
ENSG00000130962	-0.152925	1.000028	0.931075	1	PRRG1	K00522
ENSG00000158859	-0.152925	1.000028	0.931075	1	ADAMTS4	K07764
ENSG00000175003	-0.152925	1.000028	0.931075	1	SLC22A1	K08198
ENSG00000212766	-0.152925	1.000028	0.931075	1	LINC00277	_
ENSG00000254531	-0.152925	1.000028	0.931075	1	AP001816.1	_
ENSG00000134882	-0.057738	6.960725	0.931122	1	UBAC2	_
ENSG00000166619	0.059871	5.651337	0.931156	1	BLCAP	_
ENSG00000104960	0.057658	6.868899	0.931158	1	PTOV1	K15168
ENSG00000000457	-0.060605	3.426044	0.931201	1	SCYL3	K17542
ENSG00000149179	0.06023	5.213104	0.931216	1	C11orf49	_
ENSG00000163565	0.058116	6.360839	0.931226	1	IFI16	_
ENSG00000261461	-0.074217	2.671952	0.931291	1	UBE2MP1	K10579
ENSG00000105486	-0.059317	5.990924	0.931306	1	LIG1	K10747
ENSG00000066777	0.057911	5.378303	0.931333	1	ARFGEF1	K18442
ENSG00000147274	-0.056842	8.108682	0.931364	1	RBMX	K12885
ENSG00000078142	-0.061835	4.938063	0.931455	1	PIK3C3	K00914
ENSG00000165113	-0.062731	4.123928	0.931466	1	GKAP1	_
ENSG00000183576	0.057662	6.225516	0.931494	1	SETD3	K19199
ENSG00000148572	0.062468	5.221356	0.93153	1	NRBF2	_
ENSG00000187954	0.057779	6.386234	0.931597	1	CYHR1	_
ENSG00000100029	-0.056804	7.696115	0.931641	1	PES1	K14843
ENSG00000112697	0.056728	7.689926	0.931644	1	TMEM30A	_
ENSG00000256053	-0.064348	4.301202	0.93171	1	APOPT1	K10407
ENSG00000161920	0.061278	4.300211	0.93171	1	MED11	K15131
ENSG00000035403	0.056626	7.627849	0.931805	1	VCL	K05700
ENSG00000147649	0.056324	8.284562	0.931853	1	MTDH	_
ENSG00000127324	0.061339	5.140341	0.931894	1	TSPAN8	K17349
ENSG00000230383	0.056666	6.004871	0.931945	1	AC009245.3	K02934

ENSG00000174780	-0.05661	7.558459	0.931969	1	SRP72	K03108
ENSG00000186787	0.066779	2.681306	0.932173	1	SPIN2B	_
ENSG00000015479	-0.05588	9.013342	0.932209	1	MATR3	K13213
ENSG00000228630	0.056253	5.147793	0.932221	1	HOTAIR	_
ENSG00000082701	0.057285	6.342033	0.932259	1	GSK3B	K03083
ENSG00000164331	-0.067373	3.44642	0.932259	1	ANKRA2	_
ENSG00000156509	-0.06371	4.310066	0.932292	1	FBXO43	K10318
ENSG00000158796	0.057231	4.847643	0.9323	1	DEDD	K12165
ENSG00000166685	0.057231	4.847643	0.9323	1	COG1	_
ENSG00000036054	-0.057372	6.344939	0.932384	1	TBC1D23	_
ENSG00000163684	-0.06064	4.73407	0.932407	1	RPP14	K14529
ENSG00000143256	0.056942	6.529401	0.932416	1	PFDN2	K09549
ENSG00000100211	-0.06132	4.143894	0.93245	1	CBY1	_
ENSG00000117385	0.056483	6.961912	0.932489	1	LEPRE1	K08134
ENSG00000183386	-0.061906	4.852629	0.932499	1	FHL3	_
ENSG00000162813	-0.058723	5.244831	0.932607	1	BPNT1	K01082
ENSG00000172661	-0.090187	2.086237	0.932657	1	FAM21C	K18462
ENSG00000148225	0.081739	2.078715	0.932657	1	WDR31	K16467
ENSG00000136279	0.057829	6.020501	0.932662	1	DBNL	_
ENSG00000167881	-0.056437	6.948358	0.932713	1	SRP68	K03107
ENSG00000049860	-0.055616	8.206479	0.932807	1	HEXB	K12373
ENSG00000156535	-0.055862	7.658421	0.932809	1	CD109	K06530
ENSG00000134109	-0.058466	5.066598	0.932817	1	EDEM1	K10084
ENSG00000144118	0.056067	6.745132	0.933218	1	RALB	K07835
ENSG00000067167	0.055255	7.976276	0.933283	1	TRAM1	K14010
ENSG00000106400	0.055881	6.176621	0.933306	1	ZNHIT1	K11663
ENSG00000205629	-0.064714	4.166077	0.933407	1	LCMT1	K18203
ENSG00000125458	-0.05925	5.70099	0.933416	1	NT5C	K01081
ENSG00000121057	0.056785	5.880081	0.933532	1	AKAP1	K16518
ENSG00000112739	-0.05541	7.356844	0.933577	1	PRPF4B	_
ENSG00000101439	-0.05571	6.847651	0.933706	1	CST3	K13899
ENSG00000171169	0.064992	4.34188	0.933706	1	NAIF1	K11447
ENSG00000230195	0.131279	1.023736	0.933711	1	AC118138.2	_
ENSG00000263470	0.131279	1.023736	0.933711	1	RP11-160O5.1	_
ENSG00000269352	0.131279	1.023736	0.933711	1	AC018766.5	_
ENSG00000135245	0.061375	3.974353	0.933737	1	HILPDA	K17985
ENSG00000119927	-0.073375	3.75008	0.933758	1	GPAM	K00629
ENSG00000114626	0.055844	5.18203	0.933811	1	ABTB1	K10520
ENSG00000119392	-0.056945	6.2334	0.933845	1	GLE1	K18723
ENSG00000135931	-0.08645	2.126834	0.933924	1	ARMC9	_
ENSG00000084710	-0.06632	2.115892	0.933924	1	EFR3B	_
ENSG00000213420	-0.06632	2.115892	0.933924	1	GPC2	K08108
ENSG00000125844	0.054885	7.409137	0.934061	1	RRBP1	K14000
ENSG00000099804	0.055556	6.484051	0.934153	1	CDC34	K02207
ENSG00000205517	0.060472	4.352689	0.934255	1	RGL3	K17639
ENSG00000149091	-0.061086	4.774682	0.934295	1	DGKZ	K00901
ENSG00000114450	0.060879	5.000711	0.93437	1	GNB4	K04538
ENSG00000163125	-0.058461	5.282493	0.934377	1	RPRD2	_
ENSG00000236565	-0.070169	3.172662	0.934438	1	HNRNPA3P5	K12741
ENSG00000256060	-0.060594	3.167579	0.934438	1	TRAPPC2P1	K09228
ENSG00000069956	0.055307	6.41838	0.934544	1	MAPK6	K06855
ENSG00000214413	0.063085	4.189154	0.934641	1	BBIP1	K19399
ENSG00000143862	0.059319	5.106365	0.934643	1	ARL8A	K07955
ENSG00000171067	-0.055706	5.107864	0.934643	1	C11orf24	K11504
ENSG00000174136	0.061267	4.78377	0.934701	1	RGMB	K06847
ENSG00000167173	0.055629	5.850734	0.934711	1	C15orf39	_
ENSG00000179526	-0.055393	6.374839	0.93473	1	SHARPIN	_
ENSG00000088305	0.056555	4.901476	0.934803	1	DNMT3B	K17399

ENSG00000198918	0.055131	6.340945	0.934868	1	RPL39	K02924
ENSG00000166004	-0.056877	5.855127	0.934903	1	KIAA1731	_
ENSG00000186871	0.058057	4.522642	0.934919	1	ERCC6L	_
ENSG00000168556	0.054123	5.113995	0.934964	1	ING2	K19198
ENSG00000112977	0.053848	7.836637	0.935061	1	DAP	_
ENSG00000064995	-0.054813	5.863438	0.935286	1	TAF11	K03135
ENSG00000266066	0.078645	2.754263	0.935487	1	POLRMTP1	K10908
ENSG00000177380	0.056341	4.918982	0.935536	1	PPFIA3	_
ENSG00000111271	0.057949	4.210681	0.935537	1	ACAD10	K11729
ENSG00000181704	0.054497	6.398236	0.935553	1	YIPF6	_
ENSG00000070614	0.053389	6.182667	0.935652	1	NDST1	K02576
ENSG00000101457	0.05462	4.805921	0.935693	1	DNTTIP1	K08707
ENSG00000119820	-0.056276	5.931108	0.935766	1	YIPF4	K17985
ENSG00000166579	0.053841	6.138518	0.935797	1	NDEL1	K16739
ENSG00000145337	-0.062381	4.389606	0.93585	1	PYURF	K11001
ENSG00000134748	0.054343	6.313076	0.935862	1	PRPF38A	K12849
ENSG00000179195	0.053694	6.959082	0.935874	1	ZNF664	K09228
ENSG00000005156	0.057711	4.810606	0.935888	1	LIG3	K10776
ENSG00000080815	0.055292	5.229573	0.935915	1	PSEN1	K04505
ENSG00000101843	-0.054231	6.471392	0.935989	1	PSMD10	K06694
ENSG00000119318	-0.052873	8.482834	0.936028	1	RAD23B	K10839
ENSG00000213341	-0.053703	5.75963	0.93604	1	CHUK	K04467
ENSG00000006837	0.171263	1.084096	0.93615	1	CDKL3	K08824
ENSG00000183199	0.171263	1.084096	0.93615	1	HSP90AB3P	K04079
ENSG00000272473	0.171263	1.084096	0.93615	1	AC006273.4	_
ENSG00000149292	-0.140967	1.08468	0.93615	1	TTC12	_
ENSG00000204044	-0.140967	1.08468	0.93615	1	RP11-465L10.10	_
ENSG00000269906	-0.140967	1.08468	0.93615	1	RP11-248J18.2	_
ENSG00000259256	0.077926	2.78005	0.936266	1	RP11-838N2.3	_
ENSG00000172922	0.055215	5.886767	0.936314	1	RNASEH2C	K10745
ENSG00000178764	-0.102246	2.176867	0.936319	1	ZHX2	_
ENSG00000186807	0.079329	2.159526	0.936319	1	ANXA8L2	K17096
ENSG00000127423	0.057657	4.552861	0.936328	1	AUNIP	K16800
ENSG00000249859	0.055604	5.564086	0.936393	1	PVT1	_
ENSG00000172409	-0.055887	4.940903	0.93643	1	CLP1	K14399
ENSG00000131437	-0.058389	4.035739	0.936466	1	KIF3A	K10394
ENSG00000103249	0.053231	6.8606	0.936533	1	CLCN7	K05016
ENSG00000137193	0.054499	5.492813	0.936535	1	PIM1	K04702
ENSG00000167257	0.057772	3.812117	0.93654	1	RNF214	_
ENSG00000141759	-0.053832	6.414961	0.936558	1	TXNL4A	K12859
ENSG00000164237	-0.05266	7.77892	0.936621	1	CMBL	K01061
ENSG00000057252	-0.053622	6.540849	0.936626	1	SOAT1	K00637
ENSG00000106399	-0.054962	5.951091	0.936654	1	RPA3	K10740
ENSG00000132382	0.052998	6.958021	0.936719	1	MYBBP1A	K02331
ENSG00000156273	0.054131	6.110857	0.936743	1	BACH1	K09042
ENSG00000197217	-0.058277	4.94803	0.93678	1	ENTPD4	K12305
ENSG00000167384	0.054958	4.042554	0.936791	1	ZNF180	K09228
ENSG00000115211	0.055475	5.57517	0.936849	1	EIF2B4	K03680
ENSG00000162733	0.052747	7.121307	0.936877	1	DDR2	K05125
ENSG00000145425	-0.051835	10.43768	0.936898	1	RPS3A	K02984
ENSG00000167996	0.051746	11.81586	0.936917	1	FTH1	K00522
ENSG00000102996	0.053016	6.722036	0.936941	1	MMP15	K07995
ENSG00000165238	-0.05934	4.242145	0.936977	1	WNK2	K08867
ENSG00000101391	-0.055811	5.649514	0.937024	1	CDK5RAP1	_
ENSG00000171421	0.052653	6.919764	0.937174	1	MRPL36	K02919
ENSG00000152520	-0.055082	4.717475	0.937328	1	PAN3	K12572
ENSG00000162337	-0.053808	6.073983	0.937331	1	LRP5	K03068
ENSG00000109618	-0.075002	3.23464	0.937352	1	SEPSECS	K03341

ENSG00000008394	0.05185	8.079598	0.937373	1	MGST1	K00799
ENSG00000162607	0.051697	8.502561	0.937399	1	USP1	K11832
ENSG00000172361	-0.079591	2.20477	0.937453	1	CCDC11	_
ENSG00000127054	-0.054002	6.22464	0.937491	1	CPSF3L	K13148
ENSG00000158042	-0.052381	7.074093	0.937502	1	MRPL17	K02879
ENSG00000070366	0.055568	4.721974	0.93753	1	SMG6	K11124
ENSG00000108433	0.055568	4.721974	0.93753	1	GOSR2	K08496
ENSG00000136240	0.05149	8.705015	0.93759	1	KDELR2	K10949
ENSG00000008283	0.053919	6.131026	0.937592	1	CYB561	K08360
ENSG00000132522	0.057447	3.83689	0.937662	1	GPS2	K15307
ENSG00000184897	0.051925	7.334713	0.937706	1	H1FX	K11275
ENSG00000186654	0.059251	4.43612	0.937859	1	PRR5	_
ENSG00000080503	0.05463	5.802031	0.937901	1	SMARCA2	K11647
ENSG00000180398	-0.051623	7.717757	0.937918	1	MCFD2	_
ENSG00000008294	0.051901	7.074553	0.937944	1	SPAG9	_
ENSG00000168701	0.054453	5.08327	0.937996	1	TMEM208	_
ENSG00000186951	0.057289	3.849118	0.938027	1	PPARA	K07294
ENSG00000100994	-0.051974	6.238067	0.938058	1	PYGB	K00688
ENSG00000132581	0.052749	5.984916	0.938107	1	SDF2	_
ENSG00000148334	-0.052106	6.80752	0.938121	1	PTGES2	K05309
ENSG00000183943	-0.055198	4.861682	0.938137	1	PRKX	K19584
ENSG00000065328	-0.052535	6.386424	0.938172	1	MCM10	K10736
ENSG00000107960	0.057105	4.275542	0.938354	1	OBFC1	_
ENSG00000165661	-0.052454	6.319925	0.938376	1	QSOX2	K10758
ENSG00000169330	-0.130415	1.164667	0.938413	1	KIAA1024	_
ENSG00000174607	-0.130415	1.164667	0.938413	1	UGT8	K04628
ENSG00000214783	-0.130415	1.164667	0.938413	1	POLR2J4	_
ENSG00000254612	0.063871	2.836788	0.938492	1	RP11-676M6.1	K09512
ENSG00000167468	0.050663	8.596322	0.938627	1	GPX4	K05361
ENSG00000062282	-0.054877	4.088205	0.938675	1	DGAT2	K11160
ENSG00000005022	-0.050465	9.324954	0.938724	1	SLC25A5	K05863
ENSG00000136943	0.058911	4.460236	0.938818	1	CTSV	K01375
ENSG00000185019	-0.072082	3.271377	0.938979	1	UBOX5	K10600
ENSG00000257337	-0.072082	3.271377	0.938979	1	RP11-983P16.4	_
ENSG00000070610	-0.054799	5.303735	0.939097	1	GBA2	K17108
ENSG00000089057	0.063076	3.870197	0.939098	1	SLC23A2	K14611
ENSG00000058262	-0.05043	8.203834	0.939112	1	SEC61A1	K10956
ENSG00000187079	-0.050298	7.960287	0.939389	1	TEAD1	K09448
ENSG00000196459	0.056765	4.302471	0.939413	1	TRAPPC2	K12581
ENSG00000197363	-0.061863	3.284731	0.939503	1	ZNF517	K09228
ENSG00000170604	-0.051437	5.48696	0.939583	1	IRF2BP1	_
ENSG00000113575	-0.049902	8.341048	0.939696	1	PPP2CA	K04382
ENSG00000131263	0.05041	6.928064	0.939887	1	RLIM	K16271
ENSG00000113231	-0.052558	5.232048	0.939981	1	PDE8B	K18437
ENSG00000174851	-0.05035	7.054115	0.939986	1	YIF1A	_
ENSG00000096401	-0.050993	6.394269	0.940027	1	CDC5L	K12860
ENSG00000128973	0.05362	5.576089	0.940054	1	CLN6	K12359
ENSG00000148429	0.053531	4.908805	0.940064	1	USP6NL	K12581
ENSG00000172007	0.060409	3.625661	0.940088	1	RAB33B	K07920
ENSG00000105048	0.050111	7.053235	0.940134	1	TNNT1	K10372
ENSG00000160753	0.051933	5.920468	0.94028	1	RUSC1	_
ENSG00000137818	-0.048927	10.53509	0.940429	1	RPLP1	K02942
ENSG00000076770	0.059016	4.132796	0.940451	1	MBNL3	K14943
ENSG00000149577	-0.057141	4.1314	0.940451	1	SIDT2	_
ENSG00000110768	0.049444	5.862837	0.940453	1	GTF2H1	K03141
ENSG00000170270	0.060197	3.639809	0.940492	1	C14orf142	_
ENSG00000149177	0.053195	5.426021	0.940498	1	PTPRJ	K05698
ENSG00000198586	0.053195	5.426021	0.940498	1	TLK1	K08864

ENSG00000164236	0.120762	1.186972	0.94052	1	ANKRD33B	_
ENSG00000188959	0.120762	1.186972	0.94052	1	C9orf152	_
ENSG00000171017	-0.082731	1.219292	0.94052	1	LRRC8E	_
ENSG00000176749	-0.082731	1.219292	0.94052	1	CDK5R1	K11716
ENSG00000126216	-0.051694	5.864948	0.940541	1	TUBGCP3	K16570
ENSG00000224126	0.075293	2.878819	0.940568	1	UBE2SP2	K10583
ENSG00000115257	-0.070653	2.882772	0.940568	1	PCSK4	K08671
ENSG00000130544	-0.070653	2.882772	0.940568	1	ZNF557	K09228
ENSG00000064102	-0.049741	7.206165	0.940584	1	ASUN	_
ENSG00000140525	-0.049536	7.489903	0.940607	1	FANCI	K10895
ENSG00000250920	0.076183	2.272835	0.940623	1	RP11-297P16.4	_
ENSG00000272093	-0.073444	2.278727	0.940623	1	CTC-365E16.1	_
ENSG00000254858	0.051876	5.343077	0.940759	1	MPV17L2	K13348
ENSG00000180822	-0.051374	5.518868	0.940932	1	PSMG4	K11878
ENSG00000150712	-0.05015	5.936856	0.940947	1	MTMR12	K18085
ENSG00000149485	0.04925	7.328437	0.940959	1	FADS1	K10224
ENSG00000151461	-0.051152	6.053671	0.940975	1	UPF2	K14327
ENSG00000212747	-0.067973	3.324791	0.941022	1	FAM127C	_
ENSG00000198015	0.049625	6.73031	0.941043	1	MRPL42	K17423
ENSG00000062650	-0.049756	6.645736	0.941169	1	WAPAL	_
ENSG00000168439	0.048699	8.113079	0.941189	1	STIP1	K09553
ENSG00000131591	0.049244	4.671258	0.941211	1	C1orf159	_
ENSG00000114739	0.059988	3.65382	0.941285	1	ACVR2B	K13596
ENSG00000167644	0.049168	6.899684	0.941422	1	C19orf33	_
ENSG00000115694	0.049094	6.273349	0.941478	1	STK25	K08838
ENSG00000169564	0.048315	8.34553	0.941565	1	PCBP1	K12889
ENSG00000206149	-0.051939	3.669352	0.941674	1	HERC2P9	K10595
ENSG00000162734	0.04929	6.535063	0.941678	1	PEA15	_
ENSG00000166140	0.051625	5.95482	0.941679	1	ZFYVE19	_
ENSG00000084754	-0.04837	7.927715	0.941751	1	HADHA	K07515
ENSG00000106070	-0.04904	6.129534	0.941817	1	GRB10	_
ENSG00000168385	0.04804	8.544263	0.941834	1	2-Sep	K16942
ENSG00000197905	0.04876	5.959052	0.941839	1	TEAD4	K09448
ENSG00000241360	0.066564	3.677913	0.942057	1	PDXP	K07758
ENSG00000213024	-0.048764	6.910258	0.94206	1	NUP62	K14306
ENSG00000155858	-0.064589	3.947619	0.942098	1	LSM11	K17985
ENSG00000065029	0.050335	3.9463	0.942098	1	ZNF76	_
ENSG00000138604	0.048867	6.136013	0.942101	1	GLCE	K01793
ENSG00000125818	-0.050753	5.466281	0.942107	1	PSMF1	K06700
ENSG00000131116	-0.059275	4.173336	0.942126	1	ZNF428	K02914
ENSG00000142751	0.055884	4.69513	0.942175	1	GPN2	K06883
ENSG00000102901	0.048541	5.185662	0.942206	1	CENPT	K11512
ENSG00000205302	-0.048783	6.75816	0.942209	1	SNX2	K17917
ENSG00000182272	0.048746	6.612417	0.942244	1	B4GALNT4	K09657
ENSG00000116704	0.053956	4.547265	0.942373	1	SLC35D1	K15281
ENSG00000139266	-0.05476	4.376945	0.942383	1	9-Mar	K10659
ENSG00000168092	0.0486	6.633777	0.942395	1	PAFAH1B2	K16795
ENSG00000101407	-0.049034	5.556621	0.942432	1	TTI1	_
ENSG00000182899	-0.047351	9.716844	0.942445	1	RPL35A	K02917
ENSG00000174796	0.064803	3.361117	0.942466	1	THAP6	_
ENSG00000141699	0.050891	5.194071	0.942475	1	FAM134C	_
ENSG00000273143	0.116353	1.26214	0.942485	1	RP11-525A16.4	_
ENSG00000174840	0.051843	5.084568	0.94249	1	PDE12	K19612
ENSG00000005448	-0.088056	2.323796	0.942563	1	WDR54	K17985
ENSG00000262246	-0.088056	2.323796	0.942563	1	CORO7	K18619
ENSG00000099995	0.048176	6.823492	0.942702	1	SF3A1	K12825
ENSG00000173918	0.061552	3.963145	0.942725	1	C1QTNF1	K19470
ENSG00000081014	0.052335	4.710307	0.942738	1	AP4E1	K09131

ENSG00000111716	-0.046993	11.14531	0.94274	1	LDHB	K00016
ENSG00000089682	-0.05494	4.557235	0.942788	1	RBM41	_
ENSG00000197568	0.050107	4.556637	0.942788	1	HHLA3	_
ENSG00000164291	-0.056842	3.700113	0.942809	1	ARSK	K12376
ENSG00000108510	-0.05036	6.045066	0.942911	1	MED13	K15164
ENSG00000155545	-0.050856	5.399292	0.942917	1	MIER3	_
ENSG00000170315	-0.046984	9.050998	0.943021	1	UBB	K04551
ENSG00000173272	0.048847	5.724501	0.943067	1	MZT2A	K16574
ENSG00000100883	-0.048252	6.582181	0.943077	1	SRP54	K03106
ENSG00000258839	0.07354	2.948694	0.943128	1	MC1R	K04199
ENSG00000119720	-0.065377	2.952282	0.943128	1	NRDE2	_
ENSG00000169981	-0.065377	2.952282	0.943128	1	ZNF35	K09228
ENSG00000119125	-0.047172	7.816779	0.943269	1	GDA	K01487
ENSG00000104964	0.046782	8.757116	0.943302	1	AES	K04497
ENSG00000068615	0.051411	4.399231	0.94331	1	REEP1	K17338
ENSG00000101109	-0.047239	5.318088	0.943446	1	STK4	K04411
ENSG00000198056	0.04832	5.219021	0.943528	1	PRIM1	K02684
ENSG00000108883	-0.046955	7.705019	0.943598	1	EFTUD2	K12852
ENSG00000068120	0.046851	7.7074	0.943633	1	COASY	K02318
ENSG00000182606	0.047657	5.323496	0.943687	1	TRAK1	K15369
ENSG00000107815	-0.050289	5.813642	0.943723	1	C10orf2	K17680
ENSG00000118007	-0.049463	4.584874	0.943801	1	STAG1	K06671
ENSG00000125319	0.052088	4.736872	0.943833	1	C17orf53	_
ENSG00000174243	0.046759	7.476411	0.943884	1	DDX23	K12858
ENSG00000143742	-0.046517	8.142958	0.943897	1	SRP9	K03109
ENSG00000011007	0.050579	5.231915	0.94404	1	TCEB3	K15076
ENSG00000163872	0.048486	5.515046	0.944052	1	YEATS2	K12581
ENSG00000115286	-0.047331	6.671352	0.944076	1	NDUFS7	K03940
ENSG00000075568	0.050778	4.886742	0.944182	1	TMEM131	_
ENSG00000165185	0.055334	4.421892	0.944207	1	KIAA1958	K11447
ENSG00000103018	-0.046627	7.39594	0.94421	1	CYB5B	_
ENSG00000150403	0.046849	6.91974	0.944216	1	TMCO3	_
ENSG00000185485	0.058819	3.735139	0.944257	1	SDHAP1	K00234
ENSG00000197044	-0.112638	1.312521	0.944323	1	ZNF441	K09228
ENSG00000217716	0.061512	2.977126	0.944326	1	RPS10P3	K02947
ENSG00000155846	0.055326	4.01025	0.944528	1	PPARGC1B	K17962
ENSG00000130766	0.051909	4.75648	0.94454	1	SESN2	K10141
ENSG00000089009	-0.045607	9.765373	0.944561	1	RPL6	K02934
ENSG00000168040	0.051127	4.432116	0.944645	1	FADD	K02373
ENSG00000108061	0.047894	6.091032	0.944702	1	SHOC2	K19613
ENSG00000237506	0.049664	5.027885	0.944759	1	RPSAP15	K02998
ENSG00000146143	-0.055148	4.610259	0.944779	1	PRIM2	K02685
ENSG00000126226	0.046029	7.316913	0.94488	1	PCID2	_
ENSG00000005955	-0.072927	3.002586	0.944907	1	GGNBP2	_
ENSG00000160207	-0.062133	2.996835	0.944907	1	HSF2BP	_
ENSG00000181544	-0.05971	3.756396	0.944953	1	FANCB	K10889
ENSG00000109854	0.046613	6.503975	0.944962	1	HTATIP2	K17290
ENSG00000170545	-0.054208	4.249036	0.944966	1	SMAGP	_
ENSG00000074219	-0.047975	5.150446	0.944975	1	TEAD2	K09448
ENSG00000141384	0.04973	4.023856	0.945104	1	TAF4B	K03129
ENSG00000226944	0.063615	3.427869	0.945153	1	RP1-120G22.11	_
ENSG00000122033	-0.048181	4.774629	0.945229	1	MTIF3	K02520
ENSG00000169714	0.04509	8.988796	0.945304	1	CNBP	K09250
ENSG00000109475	-0.045181	8.513437	0.945373	1	RPL34	K02915
ENSG00000051009	0.048505	4.777438	0.945398	1	FAM160A2	_
ENSG00000076248	-0.045894	7.028503	0.94542	1	UNG	K03648
ENSG00000198642	-0.047442	5.551956	0.945477	1	KLHL9	K10447
ENSG00000116957	-0.04655	5.715259	0.945514	1	TBCE	_

ENSG00000100401	-0.045258	7.962074	0.945516	1	RANGAP1	K14319
ENSG00000137693	-0.045235	7.83649	0.945612	1	YAP1	K16687
ENSG00000161654	-0.050508	4.788984	0.945734	1	LSM12	_
ENSG00000215041	-0.046002	5.054747	0.94575	1	NEURL4	K16777
ENSG00000119969	-0.048198	5.796392	0.945781	1	HELLS	K19001
ENSG00000109881	-0.047405	6.176972	0.945791	1	CCDC34	K16753
ENSG00000131236	0.044895	8.113058	0.945816	1	CAP1	K17261
ENSG00000105879	-0.046506	5.174925	0.945884	1	CBLL1	K15685
ENSG00000118495	-0.04702	5.941149	0.946011	1	PLAGL1	K19485
ENSG00000111087	-0.105081	1.381158	0.946047	1	GLI1	K16797
ENSG00000185332	-0.105081	1.381158	0.946047	1	TMEM105	_
ENSG00000154654	0.075083	1.353153	0.946047	1	NCAM2	K06491
ENSG00000232995	0.075083	1.353153	0.946047	1	RP11-267N12.3	_
ENSG00000251603	0.075083	1.353153	0.946047	1	RP11-164P12.4	_
ENSG00000166086	-0.070456	1.362087	0.946047	1	JAM3	K06785
ENSG00000066455	0.04628	5.568711	0.946066	1	GOLGA5	_
ENSG00000261189	-0.079155	2.424978	0.946086	1	RP3-512B11.3	_
ENSG00000241878	-0.047304	5.388864	0.946097	1	PISD	_
ENSG00000170471	-0.047628	5.805829	0.946112	1	RALGAPB	_
ENSG00000140526	0.050351	4.939159	0.946195	1	ABHD2	_
ENSG00000114853	0.049489	4.055859	0.94622	1	ZBTB47	K10518
ENSG00000242612	0.048336	4.802805	0.946231	1	DECR2	K13237
ENSG00000116005	0.045065	6.995254	0.946312	1	PCYOX1	K05906
ENSG00000174013	0.047103	5.738535	0.946386	1	FBXO45	K10319
ENSG00000185359	-0.045708	6.41293	0.946401	1	HGS	K12182
ENSG00000197622	-0.046343	5.952523	0.946458	1	CDC42SE1	_
ENSG00000163428	0.044821	7.053212	0.946557	1	LRRC58	K17985
ENSG00000254598	-0.069565	3.045631	0.946578	1	CSNK2A3	K03097
ENSG00000204104	-0.046923	4.29214	0.946632	1	TRAF3IP1	K19680
ENSG00000247596	0.047402	4.951979	0.946639	1	TWF2	K08870
ENSG00000205531	-0.044566	7.359034	0.946743	1	NAP1L4	K11282
ENSG00000178531	-0.049522	5.083489	0.946841	1	CTXN1	_
ENSG00000215421	-0.07646	2.457194	0.9469	1	ZNF407	_
ENSG00000121848	-0.060548	2.448619	0.9469	1	RNF115	K11982
ENSG00000094804	-0.044232	7.715711	0.946902	1	CDC6	K02213
ENSG00000164611	0.043984	8.101441	0.946928	1	PTTG1	K06635
ENSG00000141026	0.050595	4.49572	0.946936	1	MED9	K15149
ENSG00000157540	-0.045816	5.755586	0.94698	1	DYRK1A	K08825
ENSG00000102225	0.044271	7.344966	0.946996	1	CDK16	_
ENSG00000121390	-0.046006	5.512158	0.947191	1	PSPC1	K13219
ENSG00000130695	0.048173	4.827734	0.947197	1	CEP85	K16766
ENSG00000149636	0.045348	5.762164	0.94723	1	DSN1	K11544
ENSG00000270504	-0.055415	3.819816	0.947259	1	RP11-420L9.5	_
ENSG00000197329	-0.049319	3.816629	0.947259	1	PELI1	K11964
ENSG00000140948	0.047684	5.319107	0.947268	1	ZCCHC14	_
ENSG00000132964	0.046629	4.505433	0.947332	1	CDK8	K02208
ENSG00000100347	0.044199	6.043267	0.947404	1	SAMM50	K07277
ENSG00000168216	0.049126	5.101283	0.9475	1	LMBRD1	K14617
ENSG00000185834	0.047726	4.315819	0.947539	1	RPL12P4	K02870
ENSG00000031698	0.043288	8.482416	0.947635	1	SARS	K01875
ENSG00000124784	-0.045186	6.173337	0.947719	1	RIOK1	K07178
ENSG00000133597	-0.044627	5.434188	0.9478	1	ADCK2	K08869
ENSG00000251369	0.049185	4.097454	0.947813	1	ZNF550	K09228
ENSG00000198356	0.044111	6.651759	0.947819	1	ASNA1	K01551
ENSG00000186020	0.062275	3.507185	0.947986	1	ZNF529	K17985
ENSG00000020922	-0.044904	5.92825	0.947991	1	MRE11A	K10865
ENSG00000138386	-0.045511	5.704369	0.947997	1	NAB1	_
ENSG00000161526	-0.045939	6.181212	0.948027	1	SAP30BP	_

ENSG00000161956	-0.046848	5.623074	0.948029	1	SENP3	K08593
ENSG00000066027	-0.044918	6.000571	0.948175	1	PPP2R5A	K11584
ENSG00000181690	0.05122	3.840036	0.948189	1	PLAG1	_
ENSG00000225138	-0.04518	5.23786	0.948208	1	CTD-2228K2.7	_
ENSG00000126453	-0.046749	5.711917	0.948252	1	BCL2L12	_
ENSG00000160551	-0.045083	6.066825	0.948276	1	TAOK1	K04429
ENSG00000162604	-0.043764	5.790864	0.948289	1	TM2D1	K10734
ENSG00000130311	0.043884	6.131003	0.948368	1	DDA1	K11792
ENSG00000239887	-0.073885	2.488709	0.948457	1	C1orf226	K16513
ENSG00000119509	-0.048569	4.538453	0.948486	1	INVS	K19626
ENSG00000189043	-0.043185	7.227125	0.948529	1	NDUFA4	K03948
ENSG00000108509	0.043878	5.13414	0.948647	1	CAMTA2	_
ENSG00000003756	0.043859	5.723667	0.94867	1	RBM5	K13094
ENSG00000144566	-0.043745	5.948273	0.948712	1	RAB5A	K03283
ENSG00000145741	-0.042218	9.58533	0.948716	1	BTF3	K01527
ENSG00000111786	-0.042976	7.210051	0.948796	1	SRSF9	K12890
ENSG00000109819	-0.052245	4.128849	0.948823	1	PPARGC1A	_
ENSG00000148090	-0.048166	4.720774	0.948872	1	AUH	K05607
ENSG00000044574	0.04199	10.36923	0.948879	1	HSPA5	K09490
ENSG00000132604	-0.042952	5.01461	0.948889	1	TERF2	K11111
ENSG00000170860	-0.044799	5.954649	0.948924	1	LSM3	K12622
ENSG00000170638	-0.043047	6.920815	0.948986	1	TRABD	_
ENSG00000100503	-0.046072	5.262058	0.949014	1	NIN	K16476
ENSG00000153487	-0.043841	5.260912	0.949014	1	ING1	K19197
ENSG00000167113	-0.043841	5.260912	0.949014	1	COQ4	K18586
ENSG00000142330	-0.051303	4.555351	0.949045	1	CAPN10	K08579
ENSG00000221890	-0.046731	4.136296	0.949069	1	NPTXR	K12581
ENSG00000173486	0.04482	5.470232	0.949106	1	FKBP2	K09569
ENSG00000244509	-0.045703	5.889572	0.949143	1	APOBEC3C	K18750
ENSG00000154917	-0.130542	1.464597	0.949189	1	RAB6B	K07894
ENSG00000174500	-0.130542	1.464597	0.949189	1	GCSAM	_
ENSG00000176678	-0.098243	1.446694	0.949189	1	FOXL1	K09405
ENSG00000250509	-0.098243	1.446694	0.949189	1	CTC-573N18.1	_
ENSG00000198496	0.054541	2.515249	0.949201	1	NBR2	K04135
ENSG00000233937	0.054541	2.515249	0.949201	1	CTC-338M12.4	_
ENSG00000089063	0.043546	5.816879	0.94923	1	TMEM230	_
ENSG00000204130	-0.051478	3.880571	0.94938	1	RUFY2	K12898
ENSG00000017260	-0.042327	7.351279	0.94947	1	ATP2C1	K01537
ENSG00000198755	0.041482	9.771359	0.949561	1	RPL10A	K02865
ENSG00000236439	-0.063415	3.128061	0.949634	1	RP11-175B9.3	K02978
ENSG00000223745	0.059267	3.125264	0.949634	1	RP4-717I23.3	_
ENSG00000185920	0.046366	3.560465	0.949837	1	PTCH1	K06225
ENSG00000135451	-0.042505	6.73984	0.949847	1	TROAP	_
ENSG00000114698	-0.05073	3.892421	0.949955	1	PLSCR4	_
ENSG00000014216	0.043033	5.984205	0.94996	1	CAPN1	K01367
ENSG00000135372	-0.042527	6.569792	0.950034	1	NAT10	K14521
ENSG00000064961	0.041273	8.650652	0.950037	1	HMG20B	_
ENSG00000112419	-0.054524	4.170861	0.950267	1	PHACTR2	_
ENSG00000157106	-0.042511	6.396378	0.950301	1	SMG1	K08873
ENSG00000136153	-0.040944	9.12813	0.950356	1	LMO7	K06084
ENSG00000076356	0.047213	4.401337	0.950486	1	PLXNA2	_
ENSG00000122971	0.048626	4.177211	0.9505	1	ACADS	K00248
ENSG00000180739	0.061073	3.582365	0.950541	1	S1PR5	K04295
ENSG00000241839	0.053624	3.586141	0.950541	1	PLEKHO2	_
ENSG00000129559	0.042135	6.334399	0.950618	1	NEDD8	K12158
ENSG00000115414	-0.092025	1.509396	0.950626	1	FN1	K05717
ENSG00000223546	-0.092025	1.509396	0.950626	1	LINC00630	_
ENSG00000144357	0.046121	4.60252	0.95065	1	UBR3	K11978

ENSG00000079739	0.040855	8.274733	0.950668	1	PGM1	K01835
ENSG00000095383	0.043294	5.312503	0.950767	1	TBC1D2	_
ENSG00000119314	0.04124	7.211033	0.950777	1	PTBP3	K17844
ENSG00000105221	-0.041761	6.708741	0.950787	1	AKT2	K04456
ENSG00000086758	0.046067	5.196472	0.950797	1	HUWE1	K10592
ENSG00000160226	0.044672	3.926309	0.951067	1	C21orf2	_
ENSG00000111077	0.045474	5.320207	0.951082	1	TENC1	K18080
ENSG00000164111	0.040274	8.967193	0.951173	1	ANXA5	K16646
ENSG00000105854	-0.040522	7.977666	0.951269	1	PON2	K01045
ENSG00000105519	0.053886	2.575363	0.95131	1	CAPS	_
ENSG00000128000	0.053886	2.575363	0.95131	1	ZNF780B	K09228
ENSG00000230699	0.053886	2.575363	0.95131	1	RP11-54O7.1	_
ENSG00000134248	-0.041031	7.01909	0.951335	1	LAMTOR5	K16344
ENSG00000147140	-0.04013	9.052279	0.951365	1	NONO	K13214
ENSG00000078967	0.053246	4.203597	0.951409	1	UBE2D4	K06689
ENSG00000134909	0.053327	3.615071	0.951561	1	ARHGAP32	_
ENSG00000145349	0.053327	3.615071	0.951561	1	CAMK2D	K04515
ENSG00000145014	0.042413	4.632912	0.951665	1	TMEM44	_
ENSG00000126804	0.041417	6.107015	0.951843	1	ZBTB1	K10488
ENSG00000173065	-0.051446	4.218718	0.951852	1	FAM222B	_
ENSG00000008513	-0.052723	3.953156	0.951868	1	ST3GAL1	_
ENSG00000237973	0.041535	6.041931	0.95191	1	hsa-mir-6723	K02256
ENSG00000025293	-0.040684	6.857198	0.951924	1	PHF20	K18402
ENSG00000166135	0.040424	5.81843	0.951935	1	HIF1AN	K18055
ENSG00000005884	-0.039861	8.280772	0.951936	1	ITGA3	K06482
ENSG00000169964	-0.06464	2.60833	0.951975	1	TMEM42	_
ENSG00000259431	-0.050309	2.600655	0.951975	1	THTPA	K05307
ENSG00000130829	0.102624	1.528825	0.951983	1	DUSP9	K18498
ENSG00000132386	0.102624	1.528825	0.951983	1	SERPINF1	K19614
ENSG00000272106	0.102624	1.528825	0.951983	1	RP11-345P4.9	_
ENSG00000214719	0.070325	1.545766	0.951983	1	AC005562.1	_
ENSG00000159228	0.040709	6.576529	0.952033	1	CBR1	K00079
ENSG00000198034	0.039334	10.89049	0.952077	1	RPS4X	K02987
ENSG00000205485	-0.046489	3.961554	0.95213	1	AC004980.7	K09228
ENSG00000162851	0.043537	5.235422	0.952133	1	TFB2M	K17653
ENSG00000103932	-0.043154	5.561273	0.952145	1	RPAP1	K09228
ENSG00000132424	0.040297	6.943073	0.95215	1	PNISR	K13170
ENSG00000146918	0.040192	7.052861	0.952179	1	NCAPG2	K11492
ENSG00000104343	-0.041387	5.656495	0.952202	1	UBE2W	K10688
ENSG00000228474	0.041395	6.119687	0.952258	1	OST4	_
ENSG00000180957	0.045874	4.652212	0.952318	1	PITPNB	_
ENSG00000104687	-0.040467	6.732032	0.952329	1	GSR	K00383
ENSG00000156599	-0.040626	6.122648	0.952375	1	ZDHHC5	_
ENSG00000197111	-0.039366	8.496574	0.952458	1	PCBP2	K13162
ENSG00000166166	-0.045174	4.656145	0.952479	1	TRMT61A	K07442
ENSG00000130810	-0.053733	3.64154	0.95254	1	PPAN	K14859
ENSG00000163466	-0.039564	7.613763	0.952642	1	ARPC2	K05758
ENSG00000182362	0.042328	4.667844	0.952797	1	YBEY	_
ENSG00000177600	0.038633	9.523316	0.953067	1	RPLP2	K02943
ENSG00000075303	0.041615	4.999391	0.953098	1	SLC25A40	K15119
ENSG00000204186	-0.045155	3.665801	0.95317	1	ZDBF2	K11447
ENSG00000011451	-0.043317	5.270031	0.953192	1	WIZ	_
ENSG00000104290	-0.046245	3.23869	0.953203	1	FZD3	K02329
ENSG00000141425	0.039871	6.452291	0.953203	1	RPRD1A	K15559
ENSG00000164951	-0.040535	6.2129	0.953228	1	PDP1	_
ENSG00000214756	-0.115991	1.585857	0.953267	1	METTL12	K12606
ENSG00000262050	-0.115991	1.585857	0.953267	1	RP11-74E22.3	_
ENSG00000268713	-0.115991	1.585857	0.953267	1	CTC-444N24.8	_

ENSG00000237846	0.09992	1.58844	0.953267	1	RP11-216M21.1	_
ENSG00000205937	-0.039128	7.474505	0.953267	1	RNPS1	K14325
ENSG00000113648	-0.038841	8.045962	0.953281	1	H2AFY	K11251
ENSG00000131043	-0.043151	5.147966	0.953319	1	AAR2	K13205
ENSG00000213639	-0.038777	7.925064	0.95342	1	PPP1CB	K06269
ENSG00000107779	0.040563	5.695161	0.953464	1	BMPR1A	K04673
ENSG00000069966	0.043391	5.281261	0.953603	1	GNB5	K04539
ENSG00000146054	-0.049227	4.008655	0.953641	1	TRIM7	K12000
ENSG00000135387	0.038174	8.974175	0.95373	1	CAPRIN1	K18743
ENSG00000104731	0.040943	5.403403	0.953748	1	KLHDC4	_
ENSG00000100083	0.043776	5.16381	0.953874	1	GGA1	K12404
ENSG00000136111	0.040281	5.957626	0.95391	1	TBC1D4	K17902
ENSG00000107643	-0.047463	4.283163	0.953949	1	MAPK8	K04440
ENSG00000167600	-0.047463	4.283163	0.953949	1	CYP2S1	K07420
ENSG00000164162	-0.054179	3.261897	0.954017	1	ANAPC10	K03357
ENSG00000186814	0.048099	3.264358	0.954017	1	ZSCAN30	K09230
ENSG00000259494	-0.042411	4.704022	0.954028	1	MRPL46	K17427
ENSG00000096093	0.038842	3.691961	0.954085	1	EFHC1	K11545
ENSG00000130881	0.038842	3.691961	0.954085	1	LRP3	_
ENSG00000008952	-0.038517	7.280298	0.954158	1	SEC62	K12275
ENSG00000120656	-0.042031	4.710734	0.954178	1	TAF12	K03126
ENSG00000114388	-0.047903	4.030272	0.954361	1	NPRL2	_
ENSG00000176974	-0.039246	6.299511	0.954409	1	SHMT1	K00600
ENSG00000220785	0.047982	3.282504	0.954414	1	MTMR9LP	K18084
ENSG00000162076	0.038241	7.127216	0.954488	1	FLYWCH2	_
ENSG00000011132	-0.046389	4.30106	0.954542	1	APBA3	_
ENSG00000112584	0.049648	4.034603	0.954597	1	FAM120B	_
ENSG00000112699	-0.039124	5.980584	0.954598	1	GMDS	K01711
ENSG00000138138	0.038204	7.04457	0.954602	1	ATAD1	_
ENSG00000182108	-0.055943	3.713533	0.954676	1	DEXI	_
ENSG00000204822	-0.043024	3.293325	0.954804	1	MRPL53	K17434
ENSG00000250899	-0.043024	3.293325	0.954804	1	RP11-253E3.3	_
ENSG00000170412	0.04126	5.19887	0.954946	1	GPRC5C	K04620
ENSG00000069329	-0.037904	7.205031	0.954964	1	VPS35	K18468
ENSG00000171174	0.052727	2.688559	0.955043	1	RBKS	K00852
ENSG00000065518	-0.038045	6.835488	0.955152	1	NDUFB4	K03960
ENSG00000090565	0.040404	5.836966	0.95518	1	RAB11FIP3	K12485
ENSG00000184674	-0.03796	5.839158	0.955247	1	GSTT1	K00799
ENSG00000169249	-0.045988	4.062102	0.955288	1	ZRSR2	_
ENSG00000167186	-0.04083	4.059421	0.955288	1	COQ7	K06134
ENSG00000131351	0.042616	4.552947	0.955327	1	HAUS8	K16591
ENSG00000124226	0.03796	6.596148	0.955346	1	RNF114	K15697
ENSG00000119401	-0.039634	5.210876	0.95536	1	TRIM32	K10607
ENSG00000136930	-0.037193	7.879062	0.955372	1	PSMB7	K02739
ENSG00000136238	0.036822	8.420482	0.955527	1	RAC1	K04392
ENSG00000114423	0.052129	3.738523	0.955534	1	CBLB	K04707
ENSG00000177879	0.037087	7.621526	0.955555	1	AP3S1	K12399
ENSG00000066422	0.038866	5.932706	0.95557	1	ZBTB11	K10498
ENSG00000170779	0.038865	5.668673	0.9556	1	CDCA4	_
ENSG00000105722	0.037868	6.457286	0.955627	1	ERF	K09434
ENSG00000134982	0.038864	5.572401	0.955693	1	APC	K02085
ENSG00000128050	-0.036591	8.924934	0.955708	1	PAICS	K01587
ENSG00000122299	-0.038279	6.296728	0.955827	1	ZC3H7A	_
ENSG00000188807	-0.043817	4.344856	0.95587	1	TMEM201	_
ENSG00000204628	0.036116	10.66463	0.956015	1	GNB2L1	K14753
ENSG00000103769	-0.036825	7.535819	0.956025	1	RAB11A	K07904
ENSG00000131381	-0.038497	4.943704	0.956089	1	ZFYVE20	K12481
ENSG00000143947	-0.036116	9.962841	0.956096	1	RPS27A	K02977

ENSG00000144021	-0.037234	6.850768	0.956121	1	CIAO1	_
ENSG00000170142	-0.037419	6.646656	0.956133	1	UBE2E1	K06689
ENSG00000121716	0.065737	2.735018	0.956163	1	PILRB	K15411
ENSG00000065325	0.038864	5.587075	0.956165	1	GLP2R	K04582
ENSG00000141252	0.042081	4.774262	0.956172	1	VPS53	K02927
ENSG00000130770	0.036878	7.047883	0.95621	1	ATPIF1	_
ENSG00000070081	-0.037589	6.330359	0.956368	1	NUCB2	_
ENSG00000175309	-0.042831	4.36201	0.956416	1	PHYKPL	K18202
ENSG00000175166	0.035949	8.608986	0.956532	1	PSMD2	K03028
ENSG00000100324	0.038858	4.788629	0.956576	1	TAB1	K04403
ENSG00000101350	0.038865	5.70276	0.95662	1	KIF3B	K10394
ENSG00000166173	-0.04541	3.775541	0.956628	1	LARP6	K18733
ENSG00000176700	-0.056995	3.354895	0.956658	1	SCAND2P	K09230
ENSG00000213638	-0.056995	3.354895	0.956658	1	ADAT3	K15442
ENSG00000169762	-0.048542	3.35044	0.956658	1	TAPT1	_
ENSG00000095564	0.036941	6.510694	0.956685	1	BTAF1	K15192
ENSG00000152894	-0.036826	6.049072	0.95671	1	PTPRK	K06776
ENSG00000152944	0.037669	6.197646	0.956843	1	MED21	K15152
ENSG00000163719	-0.044609	4.604807	0.956888	1	MTMR14	K18086
ENSG00000171848	-0.036252	7.2475	0.956937	1	RRM2	K10808
ENSG00000250208	-0.045994	4.381097	0.956948	1	FZD10-AS1	_
ENSG00000108963	0.038858	4.80127	0.956974	1	DPH1	K07561
ENSG00000081386	0.047439	3.369959	0.95701	1	ZNF510	K09228
ENSG00000142459	-0.039119	5.13054	0.957093	1	EVI5L	K09228
ENSG00000156467	0.035466	8.495069	0.957155	1	UQCRB	K00417
ENSG00000103091	-0.03942	4.978894	0.957157	1	WDR59	_
ENSG00000100335	-0.036473	6.647972	0.95728	1	MIEF1	_
ENSG00000100441	0.038861	5.136645	0.957301	1	KHNYN	_
ENSG00000198113	0.037309	5.81998	0.957336	1	TOR4A	K15696
ENSG00000132356	0.035714	7.383264	0.957372	1	PRKAA1	K07198
ENSG00000156860	-0.041009	5.141765	0.957405	1	FBRS	_
ENSG00000022567	0.038864	5.633761	0.957523	1	SLC45A4	K15378
ENSG00000110801	0.038863	5.412701	0.957602	1	PSMD9	K06693
ENSG00000174516	0.043865	4.140421	0.957652	1	PELI3	K11964
ENSG00000051341	-0.039122	5.532196	0.957677	1	POLQ	K02349
ENSG00000052749	0.036275	6.08391	0.957748	1	RRP12	K14794
ENSG00000173786	-0.09832	1.750637	0.957778	1	CNP	K01121
ENSG00000251795	-0.09832	1.750637	0.957778	1	SNORA66	_
ENSG00000123104	0.03886	5.000769	0.95784	1	ITPR2	K04959
ENSG00000159459	0.03886	5.006238	0.957951	1	UBR1	K10625
ENSG00000196639	-0.053642	3.405371	0.958034	1	HRH1	K04149
ENSG00000101158	0.035081	7.53429	0.958046	1	NELFCD	K15181
ENSG00000050748	-0.036113	6.358424	0.958115	1	MAPK9	K04440
ENSG00000103353	0.035022	6.099984	0.958222	1	UBFD1	_
ENSG00000185787	-0.03482	7.783798	0.958312	1	MORF4L1	K11339
ENSG00000164347	-0.035671	6.504186	0.958442	1	GFM2	K02355
ENSG00000102098	-0.037981	4.659584	0.958484	1	SCML2	K11465
ENSG00000162437	-0.036116	6.183651	0.958485	1	RAVER2	_
ENSG00000169895	-0.03503	7.143729	0.958511	1	SYAP1	K12581
ENSG00000138085	0.036183	6.033909	0.958585	1	ATRAID	_
ENSG00000198690	-0.041098	3.850358	0.958663	1	FAN1	K15363
ENSG00000166529	-0.050059	2.82147	0.958733	1	ZSCAN21	K09230
ENSG00000198142	-0.034617	7.466121	0.95876	1	SOWAHC	K12581
ENSG00000179743	-0.067834	1.78772	0.958772	1	RP11-169K16.9	_
ENSG00000183569	-0.067834	1.78772	0.958772	1	SERHL2	K04613
ENSG00000268471	-0.067834	1.78772	0.958772	1	MIR4453	_
ENSG00000197951	0.065636	1.768122	0.958772	1	ZNF71	K09228
ENSG00000234753	0.065636	1.768122	0.958772	1	RP11-328M4.2	_

ENSG00000165891	-0.039108	4.182843	0.958839	1	E2F7	K09391
ENSG00000105976	-0.03419	8.264499	0.958848	1	MET	K05099
ENSG00000100353	0.034046	8.272427	0.958959	1	EIF3D	K03251
ENSG00000004455	-0.034047	8.416994	0.958963	1	AK2	K00939
ENSG00000130725	0.034799	6.754019	0.959011	1	UBE2M	K10579
ENSG00000108599	-0.038327	5.581561	0.959112	1	AKAP10	K16526
ENSG00000146576	-0.046342	3.865545	0.959143	1	C7orf26	_
ENSG00000117419	0.035301	6.208357	0.959206	1	ERI3	K18418
ENSG00000136448	0.034348	7.087113	0.959252	1	NMT1	_
ENSG00000136811	-0.035194	5.975722	0.959262	1	ODF2	K16479
ENSG00000062485	0.033893	7.947482	0.959277	1	CS	K01647
ENSG00000197603	-0.033984	5.79562	0.959292	1	C5orf42	K16480
ENSG00000006757	-0.036164	4.693864	0.959439	1	PNPLA4	K11157
ENSG00000151148	-0.038473	4.889999	0.959449	1	UBE3B	K10588
ENSG00000076043	-0.034653	6.579021	0.959577	1	REXO2	K13288
ENSG00000105327	0.038846	3.882443	0.959611	1	BBC3	K10132
ENSG00000156463	0.038836	3.456559	0.959637	1	SH3RF2	K12171
ENSG00000164938	0.038836	3.456559	0.959637	1	TP53INP1	K15310
ENSG00000166415	0.091065	1.804928	0.95972	1	WDR72	_
ENSG00000213963	0.091065	1.804928	0.95972	1	AC074286.1	_
ENSG00000182195	0.064674	1.81874	0.95972	1	LDOC1	_
ENSG00000153922	-0.036454	6.153784	0.959729	1	CHD1	K11367
ENSG00000172053	0.033759	7.272718	0.959826	1	QARS	K01886
ENSG00000108292	-0.033946	7.103168	0.959865	1	MLLT6	_
ENSG00000109920	0.033903	6.95193	0.959909	1	FNBP4	_
ENSG00000166946	0.03496	5.49476	0.959931	1	CCNDBP1	_
ENSG00000114861	-0.037056	4.220947	0.959961	1	FOXP1	_
ENSG00000155096	0.033497	7.489829	0.96	1	AZIN1	_
ENSG00000197958	0.03281	9.89973	0.960114	1	RPL12	K02870
ENSG00000271912	-0.058892	2.876495	0.960136	1	RP11-661A12.14	_
ENSG00000233426	-0.043346	4.496301	0.96019	1	EIF3FP3	K03249
ENSG00000180098	0.034942	4.495817	0.96019	1	TRNAU1AP	_
ENSG00000117448	0.033507	7.033543	0.96032	1	AKR1A1	K00002
ENSG00000122778	-0.035667	5.63195	0.960521	1	KIAA1549	K17985
ENSG00000196646	-0.045598	2.893945	0.960582	1	ZNF136	K09228
ENSG00000132128	0.033681	6.404399	0.960775	1	LRRC41	K10347
ENSG00000166747	0.034289	5.849183	0.960801	1	AP1G1	K12391
ENSG00000131473	0.038837	3.503722	0.960834	1	ACLY	K01648
ENSG00000103978	0.034067	6.194646	0.960859	1	TMEM87A	_
ENSG00000149187	0.03298	7.068273	0.960931	1	CELF1	K13207
ENSG00000124222	-0.033617	6.487185	0.960958	1	STX16	K08489
ENSG00000079616	-0.033455	6.60268	0.961001	1	KIF22	K10403
ENSG00000177614	0.050677	2.914809	0.961019	1	PGBD5	_
ENSG00000236144	0.050677	2.914809	0.961019	1	AD000090.2	_
ENSG00000132005	-0.039609	4.2604	0.961024	1	RFX1	K09173
ENSG00000101935	-0.036406	5.27116	0.961073	1	AMMECR1	_
ENSG00000138376	-0.042236	3.935948	0.961169	1	BARD1	K10683
ENSG00000196544	-0.037462	4.532716	0.961212	1	C17orf59	_
ENSG00000143149	0.033155	6.527896	0.961259	1	ALDH9A1	K00149
ENSG00000182628	-0.033422	5.867981	0.96126	1	SKA2	_
ENSG00000111846	0.03463	5.960436	0.961262	1	GCNT2	K00742
ENSG00000023228	-0.033047	6.731647	0.96134	1	NDUFS1	K03934
ENSG00000136682	0.043422	4.273387	0.961365	1	CBWD2	_
ENSG00000049239	0.038852	4.275691	0.961365	1	H6PD	K13937
ENSG00000164830	0.034033	5.771015	0.961379	1	OXR1	_
ENSG00000106605	-0.03338	6.370142	0.961413	1	BLVRA	K00214
ENSG00000111670	-0.033179	6.534311	0.961425	1	GNPTAB	K08239
ENSG00000073969	-0.037586	4.960699	0.961445	1	NSF	K06027

ENSG00000198339	0.063779	1.867642	0.961491	1	HIST1H4I	K11254
ENSG00000155438	-0.033245	6.307855	0.961671	1	NIFK	K14838
ENSG00000071462	0.038852	4.284721	0.961701	1	WBSCR22	K19306
ENSG00000253729	0.031457	9.518973	0.961814	1	PRKDC	K06642
ENSG00000211459	0.031322	11.31324	0.961824	1	MT-RNR1	_
ENSG00000089127	-0.034857	5.681492	0.961836	1	OAS1	K14216
ENSG00000221829	0.034097	5.790165	0.961857	1	FANCG	K10894
ENSG00000144504	0.03882	2.943906	0.961863	1	ANKMY1	K14165
ENSG00000120832	0.032463	4.785477	0.961942	1	MTERFD3	K15031
ENSG00000051596	-0.032674	5.690862	0.962089	1	THOC3	K12880
ENSG00000173020	-0.034343	5.99235	0.962095	1	ADRBK1	K00910
ENSG00000133112	0.030988	11.13426	0.96224	1	TPT1	_
ENSG00000055070	-0.032285	6.644047	0.962369	1	SZRD1	_
ENSG00000196365	0.031196	8.42224	0.962379	1	LONP1	K08675
ENSG00000051108	-0.032144	6.766303	0.962396	1	HERPUD1	K14027
ENSG00000143324	0.032404	6.350049	0.962397	1	XPR1	_
ENSG00000147889	-0.031139	8.592661	0.962446	1	CDKN2A	K06621
ENSG00000115234	-0.032297	6.258882	0.962565	1	SNX17	K17929
ENSG00000197818	0.038839	3.579055	0.962766	1	SLC9A8	K14724
ENSG00000173465	0.031634	5.605207	0.962884	1	SSSCA1	_
ENSG00000141385	-0.031671	6.77594	0.962961	1	AFG3L2	K08956
ENSG00000146701	-0.035754	4.826501	0.96297	1	MDH2	K00026
ENSG00000168612	0.046213	3.589906	0.963026	1	ZSWIM1	K17985
ENSG00000181896	-0.035098	3.587552	0.963026	1	ZNF101	K09228
ENSG00000110628	-0.032413	4.831117	0.963081	1	SLC22A18	K08214
ENSG00000179010	0.030569	8.531177	0.96311	1	MRFAP1	_
ENSG00000146166	0.085944	1.948312	0.963112	1	LGSN	_
ENSG00000115255	-0.033148	5.197784	0.963131	1	REEP6	K17279
ENSG00000168876	-0.033148	5.197784	0.963131	1	ANKRD49	_
ENSG00000242802	-0.033148	5.197784	0.963131	1	AP5Z1	K19025
ENSG00000055917	0.031632	6.409562	0.963254	1	PUM2	K17943
ENSG00000244462	-0.031243	6.983191	0.963263	1	RBM12	_
ENSG00000164134	-0.031434	6.747656	0.96328	1	NAA15	K00670
ENSG00000092847	0.033489	5.035969	0.963349	1	AGO1	K11593
ENSG00000088179	-0.040281	4.617344	0.963363	1	PTPN4	K18037
ENSG00000172985	0.035256	4.615161	0.963363	1	SH3RF3	K12171
ENSG00000188846	-0.030173	9.602993	0.963391	1	RPL14	K02875
ENSG00000216285	0.049949	3.004997	0.963447	1	RP11-490H24.5	K01834
ENSG00000184436	-0.033271	5.745827	0.963479	1	THAP7	_
ENSG00000165632	-0.04855	3.609484	0.963536	1	TAF3	K14650
ENSG00000113595	0.03884	3.608121	0.963536	1	TRIM23	K07963
ENSG00000166803	-0.030547	7.605502	0.963618	1	KIAA0101	_
ENSG00000164944	-0.03125	6.610787	0.963667	1	KIAA1429	_
ENSG00000198730	-0.030784	6.14329	0.963704	1	CTR9	K15176
ENSG00000076685	0.032495	5.374093	0.963718	1	NT5C2	K01081
ENSG00000058063	0.032786	4.859866	0.963838	1	ATP11B	K01530
ENSG00000176485	0.03184	5.967458	0.963855	1	PLA2G16	K16817
ENSG00000104213	-0.076402	1.989458	0.963872	1	PDGFRL	_
ENSG00000204271	-0.054092	1.977338	0.963872	1	SPIN3	_
ENSG00000197579	-0.030878	5.761272	0.963882	1	TOPORS	K10631
ENSG00000111530	-0.030032	8.083837	0.963989	1	CAND1	K17263
ENSG00000130939	0.033587	5.062463	0.964004	1	UBE4B	K10597
ENSG00000130414	-0.031207	6.392764	0.964016	1	NDUFA10	K03954
ENSG00000116809	0.033518	4.053384	0.964133	1	ZBTB17	K10500
ENSG00000137770	0.030504	5.979323	0.964149	1	CTDSPL2	K17616
ENSG00000116874	0.030987	5.069017	0.964187	1	WARS2	K01867
ENSG00000138411	-0.048529	3.034458	0.964191	1	HECW2	K12168
ENSG00000168575	0.038824	3.03233	0.964191	1	SLC20A2	_

ENSG00000162302	-0.032121	5.398244	0.964306	1	RPS6KA4	K16510
ENSG00000119185	-0.03043	5.987527	0.964343	1	ITGB1BP1	_
ENSG00000213246	0.030812	6.255037	0.964435	1	SUPT4H1	K15171
ENSG00000204120	-0.030329	5.668622	0.964444	1	GIGYF2	K18730
ENSG00000149743	0.045924	3.647046	0.964516	1	TRPT1	K10669
ENSG00000165886	0.038841	3.650649	0.964516	1	UBTD1	_
ENSG00000099795	0.030364	6.177164	0.964562	1	NDUFB7	K03963
ENSG00000139370	-0.036398	4.401922	0.964623	1	SLC15A4	K14638
ENSG00000184203	-0.029987	7.132233	0.964653	1	PPP1R2	K16833
ENSG00000112062	-0.030845	6.007431	0.964817	1	MAPK14	K04441
ENSG00000197265	0.030645	5.420918	0.964875	1	GTF2E2	K03137
ENSG00000168502	0.031201	6.104444	0.964924	1	SOGA2	_
ENSG00000068078	-0.032911	5.424007	0.964945	1	FGFR3	K05094
ENSG00000006607	0.032985	4.90741	0.964968	1	FARP2	K06082
ENSG00000070476	-0.039107	4.090625	0.965023	1	ZXDC	_
ENSG00000108590	0.038849	4.092423	0.965023	1	MED31	K15153
ENSG00000175182	0.038849	4.092423	0.965023	1	FAM131A	_
ENSG00000069943	0.035428	4.68503	0.965058	1	PIGB	K05286
ENSG00000134444	0.032006	4.686751	0.965058	1	KIAA1468	_
ENSG00000166169	-0.034365	4.912	0.965067	1	POLL	K03512
ENSG00000124214	0.029558	7.044864	0.965111	1	STAU1	K17597
ENSG00000102265	0.02899	7.958431	0.965242	1	TIMP1	K16451
ENSG00000205423	-0.056216	3.082025	0.965251	1	CNEP1R1	_
ENSG00000229320	-0.056216	3.082025	0.965251	1	KRT8P12	K07605
ENSG00000110925	0.033032	4.919055	0.965264	1	CSRNP2	K17494
ENSG00000168056	-0.029432	7.169874	0.965296	1	LTBP3	K08023
ENSG00000167785	-0.094154	2.04447	0.965301	1	ZNF558	K09228
ENSG00000131480	0.060745	2.048187	0.965301	1	AOC2	K00276
ENSG00000232150	0.060745	2.048187	0.965301	1	ST13P4	K09560
ENSG00000013374	0.029522	6.817208	0.965359	1	NUB1	_
ENSG00000165526	-0.030658	4.927922	0.965458	1	RPUSD4	_
ENSG00000198060	-0.030728	6.037783	0.965551	1	5-Mar	K10660
ENSG00000197050	-0.044732	3.097211	0.965591	1	ZNF420	K09228
ENSG00000130202	0.029283	6.655626	0.965814	1	PVRL2	K06531
ENSG00000011405	0.030322	5.948404	0.96582	1	PIK3C2A	K00923
ENSG00000105993	0.028912	7.140603	0.96582	1	DNAJB6	K09512
ENSG00000013392	0.033782	4.125517	0.96587	1	RWDD2A	K11447
ENSG00000136636	-0.029352	6.631728	0.965947	1	KCTD3	K04613
ENSG00000114646	-0.069479	2.074961	0.965974	1	CSPG5	K08116
ENSG00000155097	-0.028786	7.294938	0.965979	1	ATP6V1C1	K02148
ENSG00000105738	0.030821	4.458425	0.965989	1	SIPA1L3	K17703
ENSG00000058799	0.031395	5.14536	0.966	1	YIPF1	_
ENSG00000065054	0.028033	5.607007	0.966005	1	SLC9A3R2	K13358
ENSG00000159840	0.028327	7.642706	0.966203	1	ZYX	K06273
ENSG00000153443	0.03096	5.477143	0.966221	1	UBALD1	_
ENSG00000109089	0.028376	6.066982	0.966254	1	CDR2L	_
ENSG00000010322	-0.029974	5.484023	0.96635	1	NISCH	K18047
ENSG00000100413	-0.029974	5.484023	0.96635	1	POLR3H	K03022
ENSG00000092841	-0.027789	9.238746	0.966364	1	MYL6	K12751
ENSG00000099968	-0.029041	6.254876	0.966391	1	BCL2L13	K15485
ENSG00000181163	0.027569	11.0805	0.966412	1	NPM1	K11276
ENSG00000166794	-0.027847	8.730685	0.966416	1	PPIB	K03768
ENSG00000158669	-0.030641	5.62575	0.966418	1	AGPAT6	K13506
ENSG00000125630	-0.028966	6.074688	0.966426	1	POLR1B	K03002
ENSG00000137947	0.029749	6.076766	0.966468	1	GTF2B	K03124
ENSG00000198258	-0.028853	6.642304	0.96654	1	UBL5	K13113
ENSG00000134247	-0.028819	6.624193	0.966603	1	PTGFRN	K06729
ENSG00000011275	-0.030049	5.875474	0.966618	1	RNF216	K11976

ENSG00000196466	0.060098	2.090001	0.966621	1	ZNF799	K09228
ENSG00000230629	0.060098	2.090001	0.966621	1	RPS23P8	K02973
ENSG00000161914	0.038778	2.101189	0.966621	1	ZNF653	_
ENSG00000235859	0.038778	2.101189	0.966621	1	AC006978.6	K03066
ENSG00000168476	-0.031856	5.637248	0.966706	1	REEP4	K17338
ENSG00000169855	0.028526	6.087768	0.966722	1	ROBO1	K06753
ENSG00000157978	0.029116	5.176018	0.966723	1	LDLRAP1	K12474
ENSG00000181885	0.038843	3.745264	0.966748	1	CLDN7	K06087
ENSG00000108424	-0.027601	8.580592	0.966761	1	KPNB1	K14293
ENSG00000163605	-0.028816	5.99236	0.96683	1	PPP4R2	K15425
ENSG00000132388	0.028415	6.656058	0.966868	1	UBE2G1	K10575
ENSG00000147457	-0.030755	5.508311	0.966919	1	CHMP7	K15053
ENSG00000131469	-0.027291	9.423976	0.966936	1	RPL27	K02901
ENSG00000180530	-0.02891	5.185781	0.966957	1	NRIP1	K17965
ENSG00000129315	0.028274	6.636792	0.96706	1	CCNT1	K15188
ENSG00000153201	0.027659	7.42874	0.967142	1	RANBP2	K12172
ENSG00000265681	0.027938	6.008227	0.967183	1	RPL17	K02880
ENSG00000132361	-0.027613	7.610462	0.967187	1	CLUH	K03255
ENSG00000163541	0.02786	6.902899	0.967299	1	SUCLG1	K01899
ENSG00000177556	-0.029194	5.796415	0.967454	1	ATOX1	K07213
ENSG00000177406	0.038828	3.175123	0.967496	1	RP11-218M22.1	_
ENSG00000160570	0.032491	4.791825	0.967526	1	DEDD2	_
ENSG00000082515	0.031272	5.535048	0.967528	1	MRPL22	K02890
ENSG00000243678	-0.026921	8.273638	0.967698	1	NME1-NME2	K00940
ENSG00000157625	0.027884	5.809056	0.967751	1	TAB3	K12793
ENSG00000107566	-0.027729	6.703334	0.967832	1	ERLIN1	_
ENSG00000183250	-0.06334	2.155698	0.967844	1	C21orf67	_
ENSG00000136247	-0.031738	5.226175	0.967861	1	ZDHHC4	K18932
ENSG00000244005	-0.027884	6.525788	0.967865	1	NFS1	K04487
ENSG00000174306	0.032574	4.810702	0.96792	1	ZHX3	_
ENSG00000218336	0.027126	5.229609	0.967934	1	TENM3	_
ENSG00000129925	0.028201	6.043234	0.967992	1	TMEM8A	_
ENSG00000165458	-0.026747	7.974258	0.968042	1	INPPL1	K15909
ENSG00000122515	-0.028007	6.149487	0.968126	1	ZMIZ2	_
ENSG00000155229	-0.02752	6.594103	0.968219	1	MMS19	K15075
ENSG00000064545	0.02753	6.413671	0.968228	1	TMEM161A	_
ENSG00000115839	-0.028393	5.709421	0.968337	1	RAB3GAP1	K18270
ENSG00000127804	-0.030311	5.838402	0.968378	1	METTL16	_
ENSG00000114982	0.027783	5.57424	0.968402	1	KANSL3	K16719
ENSG00000185519	-0.060525	2.194439	0.968423	1	FAM131C	_
ENSG00000103671	0.031403	4.566032	0.968426	1	TRIP4	K09228
ENSG00000196182	0.029519	4.243712	0.968466	1	STK40	K16312
ENSG00000047315	0.026696	7.098646	0.968548	1	POLR2B	K03010
ENSG00000129128	-0.028068	6.174594	0.968655	1	SPCS3	K12948
ENSG00000102316	-0.026599	6.9317	0.968959	1	MAGED2	_
ENSG00000129103	-0.026098	7.672614	0.968993	1	SUMF2	K10408
ENSG00000128923	-0.030436	4.597505	0.969091	1	FAM63B	K12581
ENSG00000100603	-0.026796	6.590015	0.969103	1	SNW1	K06063
ENSG00000143393	0.026729	6.440682	0.969166	1	PI4KB	K00888
ENSG00000119397	-0.045143	3.257132	0.969202	1	CNTRL	K16770
ENSG00000166484	0.038845	3.858452	0.969263	1	MAPK7	K04464
ENSG00000132003	-0.030505	5.100164	0.969324	1	ZSWIM4	_
ENSG00000168434	0.034325	4.287006	0.969424	1	COG7	_
ENSG00000168010	-0.03386	3.871395	0.96944	1	ATG16L2	K17890
ENSG00000111203	0.029622	3.273837	0.969469	1	ITFG2	_
ENSG00000150477	0.057854	2.246098	0.969519	1	KIAA1328	K12581
ENSG00000101161	0.02606	6.777765	0.969604	1	PRPF6	K12855
ENSG00000198894	0.032801	3.873567	0.969616	1	CIPC	_

ENSG00000100823	-0.025723	7.385328	0.969638	1	APEX1	K10771
ENSG00000160094	0.038832	3.28719	0.969732	1	ZNF362	_
ENSG00000141034	-0.032002	5.121738	0.969775	1	GID4	K01368
ENSG00000109184	-0.029787	5.487936	0.9698	1	DCUN1D4	K17824
ENSG00000161981	-0.026944	5.910165	0.969908	1	SNRNP25	K13153
ENSG00000117139	0.024862	6.23478	0.969938	1	KDM5B	K11446
ENSG00000037897	-0.03237	4.312031	0.969945	1	METTL1	K03439
ENSG00000112031	0.029979	4.315956	0.969945	1	MTRF1L	K02835
ENSG00000108861	-0.02622	6.4774	0.96995	1	DUSP3	K17614
ENSG00000153885	-0.026033	5.64866	0.969963	1	KCTD15	_
ENSG00000140534	0.027878	5.324607	0.969982	1	TICRR	_
ENSG00000071537	0.027184	5.498645	0.970028	1	SEL1L	K14026
ENSG00000141858	0.026784	6.03322	0.970064	1	SAMD1	_
ENSG00000102471	-0.025618	7.015433	0.970072	1	NDFIP2	_
ENSG00000197136	-0.025861	6.688537	0.970119	1	PCNXL3	_
ENSG00000119689	-0.025715	6.672721	0.970315	1	DLST	K00658
ENSG00000125447	-0.027828	4.926472	0.970349	1	GGA3	K12404
ENSG00000112992	-0.032353	3.324092	0.970492	1	NNT	K00323
ENSG00000105372	0.057365	2.282619	0.970542	1	RPS19	K02966
ENSG00000104695	0.025233	6.77496	0.970612	1	PPP2CB	K04382
ENSG00000167112	-0.026321	5.680644	0.970614	1	TRUB2	_
ENSG00000117280	-0.026012	5.687468	0.970761	1	RAB7L1	K07916
ENSG00000115266	0.033045	3.932204	0.970791	1	APC2	K02085
ENSG00000165476	0.025531	6.30525	0.970796	1	REEP3	K17338
ENSG00000105464	0.034542	4.357168	0.970816	1	GRIN2D	K05212
ENSG00000129691	-0.029559	4.950785	0.97084	1	ASH2L	K14964
ENSG00000131795	-0.070604	2.314337	0.971028	1	RBM8A	K12876
ENSG00000171840	-0.070604	2.314337	0.971028	1	NINJ2	_
ENSG00000157837	-0.026758	5.378349	0.971031	1	SPPL3	K09598
ENSG00000135341	0.027639	5.555709	0.971172	1	MAP3K7	K04427
ENSG00000170325	-0.030638	3.358671	0.971216	1	PRDM10	_
ENSG00000102302	-0.029018	4.380976	0.971292	1	FGD1	K05720
ENSG00000168411	0.024849	6.55471	0.971312	1	RFWD3	K15691
ENSG00000214026	0.027697	5.56315	0.971329	1	MRPL23	K02892
ENSG00000085449	-0.024425	5.563135	0.971329	1	WDFY1	_
ENSG00000132359	0.024813	6.550973	0.971361	1	RAP1GAP2	K17708
ENSG00000183597	0.030396	4.384755	0.971408	1	TANGO2	_
ENSG00000128708	0.0245	6.859729	0.971423	1	HAT1	K11303
ENSG00000008988	-0.023565	9.766823	0.971426	1	RPS20	K02969
ENSG00000123154	0.038834	3.374358	0.971449	1	WDR83	K13124
ENSG00000065154	-0.025911	5.571458	0.971483	1	OAT	K00819
ENSG00000086232	-0.023884	8.030868	0.971502	1	EIF2AK1	K16194
ENSG00000170581	-0.025463	5.208486	0.971519	1	STAT2	K11221
ENSG00000172432	-0.024832	6.530075	0.971564	1	GTPBP2	_
ENSG00000131495	0.025856	6.215434	0.97157	1	NDUFA2	K03946
ENSG00000181929	0.024498	6.699315	0.971582	1	PRKAG1	K07200
ENSG00000198862	0.025321	5.868818	0.971628	1	LTN1	_
ENSG00000172175	0.024696	6.439665	0.971636	1	MALT1	K07369
ENSG00000183283	-0.023845	7.824855	0.971651	1	DAZAP2	_
ENSG00000167123	-0.024578	6.698287	0.971666	1	CERCAM	_
ENSG00000125686	-0.02712	5.873984	0.97171	1	MED1	K15144
ENSG00000185591	0.024396	6.689149	0.971716	1	SP1	K04684
ENSG00000086589	-0.024158	6.004031	0.971794	1	RBM22	K12872
ENSG00000095574	0.03327	3.98855	0.971879	1	IKZF5	K09220
ENSG00000160285	0.025039	6.127826	0.971934	1	LSS	K01852
ENSG00000099625	-0.027708	3.997482	0.972028	1	C19orf26	_
ENSG00000137106	0.02379	7.126837	0.972061	1	GRHPR	K00049
ENSG00000167272	-0.026267	5.755607	0.972103	1	POP5	K03537

ENSG00000104368	-0.036381	3.413309	0.972127	1	PLAT	K01343
ENSG00000172869	0.024881	5.240095	0.972162	1	DMXL1	_
ENSG00000197223	0.026547	4.428213	0.972198	1	C1D	K12592
ENSG00000229132	-0.032569	4.011198	0.972321	1	EIF4A1P10	K03257
ENSG00000114631	-0.023411	7.47261	0.972393	1	PODXL2	K06818
ENSG00000014641	-0.023036	8.367597	0.972397	1	MDH1	K00025
ENSG00000001167	0.029146	4.767889	0.972471	1	NFYA	K08064
ENSG00000127838	0.02506	5.258544	0.972473	1	PNKD	_
ENSG00000132153	0.023604	6.846446	0.972525	1	DHX30	K13185
ENSG00000241685	-0.023714	6.864058	0.972536	1	ARPC1A	K05757
ENSG00000148303	-0.031497	4.03278	0.972608	1	RPL7A	K02936
ENSG00000131051	-0.022883	8.301448	0.972609	1	RBM39	K13091
ENSG00000151498	0.028048	4.03483	0.972749	1	ACAD8	K11538
ENSG00000102606	0.026514	5.639925	0.972804	1	ARHGEF7	K13710
ENSG00000160284	-0.046428	2.407357	0.97282	1	SPATC1L	_
ENSG00000213118	-0.046428	2.407357	0.97282	1	CHCHD2P4	K00901
ENSG00000183048	-0.026864	5.280205	0.972896	1	SLC25A10	K13577
ENSG00000165280	-0.022509	8.655175	0.972945	1	VCP	K13525
ENSG00000136731	0.023273	6.815627	0.972955	1	UGGT1	K11718
ENSG00000159110	-0.024314	5.065214	0.973068	1	IFNAR2	K05131
ENSG00000167323	0.023187	6.072195	0.973087	1	STIM1	K16059
ENSG00000119669	-0.025951	5.485981	0.973129	1	IRF2BPL	_
ENSG00000111911	-0.029208	5.073062	0.973205	1	HINT3	K12581
ENSG00000132661	-0.027207	4.809367	0.973224	1	NXT1	K14285
ENSG00000148297	0.038798	2.428882	0.973234	1	MED22	K15139
ENSG00000198468	0.038798	2.428882	0.973234	1	FLVCR1-AS1	_
ENSG00000129083	0.022172	8.93276	0.973242	1	COPB1	K17301
ENSG00000013810	-0.022951	7.045631	0.973288	1	TACC3	K14283
ENSG00000198887	0.025029	5.668663	0.973352	1	SMC5	_
ENSG00000163812	0.023308	5.669524	0.973352	1	ZDHHC3	K18932
ENSG00000136504	-0.02347	5.305926	0.973364	1	KAT7	K11307
ENSG00000104231	-0.023186	6.671441	0.973389	1	ZFAND1	K07059
ENSG00000135093	-0.033047	3.47765	0.973391	1	USP30	K11851
ENSG00000179912	-0.029447	4.075001	0.973434	1	R3HDM2	_
ENSG00000108591	-0.024794	5.676364	0.973486	1	DRG2	K06944
ENSG00000175643	-0.026353	4.82801	0.973546	1	RMI2	K15365
ENSG00000122482	0.023502	6.216608	0.973615	1	ZNF644	_
ENSG00000112715	-0.022371	7.390058	0.973716	1	VEGFA	K05448
ENSG00000119929	0.023572	5.982782	0.973777	1	CUTC	K06201
ENSG00000168101	0.028513	4.097659	0.973829	1	NUDT16L1	K16867
ENSG00000185963	-0.023528	5.986849	0.973848	1	BICD2	K18739
ENSG00000154518	-0.022052	7.676713	0.973918	1	ATP5G3	K02128
ENSG00000130479	-0.025403	5.701019	0.973922	1	MAP1S	K10429
ENSG00000155366	0.022087	7.43151	0.973924	1	RHOC	K17504
ENSG00000104691	-0.022953	4.103278	0.973958	1	UBXN8	_
ENSG00000162065	-0.022953	4.103278	0.973958	1	TBC1D24	_
ENSG00000169504	0.021832	7.919608	0.973978	1	CLIC4	K05024
ENSG00000154305	-0.022872	6.237563	0.974004	1	MIA3	_
ENSG00000187824	-0.058316	2.48033	0.974025	1	TMEM220	_
ENSG00000147862	0.02376	5.542594	0.974163	1	NFIB	K09169
ENSG00000122863	0.023847	6.249264	0.974209	1	CHST3	K01020
ENSG00000142655	-0.027513	4.116023	0.974212	1	PEX14	K02310
ENSG00000166002	0.027456	4.538009	0.974231	1	SMCO4	_
ENSG00000120705	-0.021516	8.327216	0.974266	1	ETF1	K03265
ENSG00000170917	0.038801	2.492601	0.974404	1	NUDT6	_
ENSG00000167380	-0.029996	3.539249	0.974544	1	ZNF226	K09228
ENSG00000147416	0.026438	5.147845	0.974559	1	ATP6V1B2	K02147
ENSG00000135334	-0.023262	5.147931	0.974559	1	AKIRIN2	_

ENSG00000111647	-0.031499	4.138644	0.974584	1	UHRF1BP1L	_
ENSG00000160201	-0.021715	7.290216	0.974588	1	U2AF1	K12836
ENSG00000130734	-0.028578	4.558947	0.974602	1	ATG4D	K08342
ENSG00000181090	-0.023153	5.896249	0.974669	1	EHMT1	K11420
ENSG00000135018	0.021316	7.781507	0.97467	1	UBQLN1	K04523
ENSG00000103174	-0.02823	4.566367	0.974693	1	NAGPA	K01125
ENSG00000160447	0.022633	5.897057	0.974706	1	PKN3	K06071
ENSG00000163281	-0.024257	4.571887	0.974784	1	GNPDA2	K02564
ENSG00000138757	-0.021519	7.212518	0.974888	1	G3BP2	_
ENSG00000075415	0.020664	9.643682	0.974959	1	SLC25A3	K15102
ENSG00000115073	0.024158	4.905975	0.974973	1	ACTR1B	K16575
ENSG00000110057	0.02214	5.396138	0.974985	1	UNC93B1	_
ENSG00000182022	0.023902	3.571937	0.975085	1	CHST15	K08106
ENSG00000171311	-0.023586	5.402394	0.975086	1	EXOSC1	K07573
ENSG00000134452	0.021897	6.372426	0.975118	1	FBXO18	K10300
ENSG00000253368	-0.05414	2.541743	0.975128	1	TRNP1	_
ENSG00000081019	0.024215	4.591765	0.97514	1	RSBN1	_
ENSG00000131143	-0.020736	8.230525	0.975253	1	COX4I1	K02263
ENSG00000096654	-0.027873	3.583785	0.97526	1	ZNF184	K09228
ENSG00000167394	0.024212	4.179855	0.975297	1	ZNF668	_
ENSG00000167526	-0.020262	10.59691	0.97537	1	RPL13	K02873
ENSG00000182117	-0.021426	6.809597	0.975374	1	NOP10	K11130
ENSG00000099800	-0.021537	6.562924	0.975525	1	TIMM13	K17781
ENSG00000148331	0.024509	4.940665	0.975529	1	ASB6	K10328
ENSG00000176454	-0.021666	6.210582	0.975545	1	LPCAT4	K13512
ENSG00000165138	0.02321	5.949101	0.975582	1	ANKS6	_
ENSG00000144677	0.022242	5.213156	0.975671	1	CTDSPL	K15731
ENSG00000105429	0.024622	4.952045	0.975732	1	MEGF8	_
ENSG00000121903	0.038805	2.583197	0.975812	1	ZSCAN20	K09230
ENSG00000149257	-0.019983	9.157273	0.975899	1	SERPINH1	K09501
ENSG00000094916	0.020323	7.528288	0.976013	1	CBX5	K11587
ENSG00000130749	0.024787	4.648824	0.976068	1	ZC3H4	_
ENSG00000123562	-0.019816	9.213165	0.976092	1	MORF4L2	K11342
ENSG00000170653	0.024898	4.980109	0.976191	1	ATF7	K09045
ENSG00000127774	0.021376	4.657556	0.97623	1	EMC6	_
ENSG00000162885	-0.031045	4.246534	0.9764	1	B3GALNT2	K09654
ENSG00000203644	-0.048509	2.629236	0.97646	1	AC083799.1	_
ENSG00000065911	-0.019532	8.457808	0.976638	1	MTHFD2	K13403
ENSG00000145868	-0.022619	5.273414	0.976639	1	FBXO38	K10313
ENSG00000114978	-0.02002	7.220192	0.976705	1	MOB1A	K06685
ENSG00000008128	-0.032864	2.649857	0.976771	1	CDK11A	K08818
ENSG00000172345	-0.032864	2.649857	0.976771	1	STARD5	K10373
ENSG00000122390	0.024477	2.64805	0.976771	1	NAA60	_
ENSG00000213145	0.024477	2.64805	0.976771	1	CRIP1	_
ENSG00000116514	0.025188	4.690185	0.976778	1	RNF19B	K11973
ENSG00000158480	-0.023393	3.682655	0.976883	1	SPATA2	K17595
ENSG00000111371	0.019152	8.999219	0.976911	1	SLC38A1	K14990
ENSG00000130741	-0.018983	9.580846	0.977044	1	EIF2S3	K03242
ENSG00000158163	0.038809	2.668441	0.977074	1	DZIP1L	K16470
ENSG00000167578	0.038809	2.668441	0.977074	1	RAB4B	K07880
ENSG00000116096	0.022533	5.888198	0.977087	1	SPR	K00072
ENSG00000167393	-0.023663	5.301485	0.977094	1	PPP2R3B	K11583
ENSG00000065361	-0.029491	3.699719	0.977183	1	ERBB3	K05084
ENSG00000145860	-0.020092	6.619772	0.97721	1	RNF145	_
ENSG00000115649	-0.024046	4.298658	0.977214	1	CNPPD1	_
ENSG00000168066	0.019613	6.774538	0.977444	1	SF1	K13095
ENSG00000136861	-0.020103	6.40837	0.977471	1	CDK5RAP2	K16542
ENSG00000204084	-0.023272	4.316369	0.977505	1	INPP5B	K01099

ENSG00000230409	-0.019252	5.552897	0.977536	1	TCEA1P2	K03145
ENSG00000126067	0.019084	7.435048	0.977578	1	PSMB2	K02734
ENSG00000177732	-0.020933	5.55756	0.977617	1	SOX12	K09268
ENSG00000247572	-0.030121	2.704624	0.977656	1	CKMT2-AS1	_
ENSG00000185513	0.025027	2.702945	0.977656	1	L3MBTL1	_
ENSG00000141428	0.021268	4.329175	0.977694	1	C18orf21	_
ENSG00000043143	0.021556	5.929802	0.97773	1	JADE2	K11347
ENSG00000229944	0.025724	4.335673	0.977788	1	AC004797.1	K03259
ENSG00000104738	0.018522	8.534777	0.977793	1	MCM4	K02212
ENSG00000070018	-0.019357	5.765322	0.977821	1	LRP6	K03068
ENSG00000123485	0.020235	5.939123	0.977885	1	HJURP	_
ENSG00000011114	-0.026995	3.752698	0.977899	1	BTBD7	K10479
ENSG00000253716	0.025678	3.751973	0.977899	1	RP13-582O9.5	_
ENSG00000243701	0.038812	2.722596	0.977937	1	LINC00883	_
ENSG00000158856	0.026029	3.790513	0.978571	1	DMTN	_
ENSG00000129195	0.022188	5.400298	0.978622	1	FAM64A	_
ENSG00000157617	0.021379	5.140412	0.978669	1	C2CD2	_
ENSG00000145982	-0.021034	5.14173	0.978721	1	FARS2	K01889
ENSG00000184900	0.018448	6.911994	0.978735	1	SUMO3	K12160
ENSG00000101966	-0.020525	4.818695	0.978739	1	XIAP	K04725
ENSG00000178028	0.021253	5.642558	0.978882	1	DMAP1	K11324
ENSG00000103549	0.020412	5.837705	0.978916	1	RNF40	K10696
ENSG00000125652	-0.022837	5.157791	0.978925	1	ALKBH7	K10769
ENSG00000217930	-0.023047	4.832622	0.97893	1	PAM16	K17805
ENSG00000197006	0.019241	6.163128	0.978938	1	METTL9	_
ENSG00000148339	-0.027457	4.414252	0.978939	1	SLC25A25	K14684
ENSG00000148343	0.022279	4.41391	0.978939	1	FAM73B	_
ENSG00000155827	-0.020894	5.843006	0.978979	1	RNF20	K10696
ENSG00000171408	0.038815	2.800198	0.978991	1	PDE7B	K18436
ENSG00000102871	-0.023575	3.828693	0.97908	1	TRADD	K03171
ENSG00000138867	0.019373	6.172888	0.979088	1	GUCD1	_
ENSG00000138814	-0.022153	5.657377	0.979097	1	PPP3CA	K04348
ENSG00000188938	-0.019816	5.171236	0.979124	1	FAM120AOS	_
ENSG00000103145	-0.020301	5.659983	0.979132	1	HCFC1R1	_
ENSG00000008018	-0.017586	8.112233	0.979141	1	PSMB1	K02732
ENSG00000077549	0.01783	6.968418	0.979439	1	CAPZB	K10365
ENSG00000099260	0.020794	4.871941	0.979482	1	PALMD	K16519
ENSG00000168028	0.016896	10.16209	0.979499	1	RPSA	K02998
ENSG00000142632	-0.021241	4.875076	0.979542	1	ARHGEF19	_
ENSG00000088876	-0.027382	3.880276	0.9798	1	ZNF343	K09228
ENSG00000198712	-0.016519	13.01993	0.979848	1	MT-CO2	K02261
ENSG00000122741	0.018322	5.905124	0.979879	1	DCAF10	K11802
ENSG00000117616	-0.020016	5.224988	0.979887	1	C1orf63	K09654
ENSG00000165895	0.020936	3.891554	0.979915	1	ARHGAP42	_
ENSG00000112576	0.018074	6.079986	0.979928	1	CCND3	K10152
ENSG00000030066	-0.016999	7.946316	0.979932	1	NUP160	K14303
ENSG00000143793	-0.022123	5.230841	0.979978	1	C1orf35	_
ENSG00000141582	-0.017858	6.538142	0.980026	1	CBX4	K11452
ENSG00000105176	-0.017356	6.98325	0.980151	1	URI1	K17560
ENSG00000137563	0.017241	6.928646	0.980189	1	GGH	K01307
ENSG00000065357	-0.025763	3.915557	0.980252	1	DGKA	K00901
ENSG00000103037	-0.021905	4.929335	0.980286	1	SETD6	K05302
ENSG00000113240	0.027172	3.923627	0.980363	1	CLK4	K08823
ENSG00000171530	0.016742	7.511782	0.980383	1	TBCA	K17292
ENSG00000177576	0.026863	2.903768	0.980395	1	C18orf32	_
ENSG00000162591	0.018729	4.937823	0.980395	1	MEGF6	_
ENSG00000180901	0.018729	4.937823	0.980395	1	KCTD2	K02138
ENSG00000160408	0.019602	4.519051	0.980421	1	ST6GALNAC6	K03376

ENSG00000152700	-0.020172	5.530302	0.980442	1	SAR1B	K07953
ENSG00000100099	0.021656	4.942101	0.98045	1	HPS4	_
ENSG00000139644	0.016086	9.910783	0.980513	1	TMBIM6	_
ENSG00000158717	0.018888	4.949225	0.980558	1	RNF166	K17044
ENSG00000184432	-0.016263	8.659123	0.98056	1	COPB2	K17302
ENSG00000262001	-0.024727	3.938608	0.980579	1	DLGAP1-AS2	_
ENSG00000171824	-0.017584	6.366473	0.980589	1	EXOSC10	K12591
ENSG00000169131	0.03882	2.920926	0.980611	1	ZNF354A	K09228
ENSG00000146457	-0.017312	6.575868	0.980642	1	WTAP	_
ENSG00000111707	-0.019403	5.549156	0.980692	1	SUDS3	K19201
ENSG00000196935	-0.021872	4.540183	0.980707	1	SRGAP1	K07526
ENSG00000167930	-0.017171	6.14146	0.980757	1	ITFG3	K17258
ENSG00000109332	-0.016315	7.917407	0.980779	1	UBE2D3	K06689
ENSG00000071564	-0.016549	7.350328	0.980831	1	TCF3	K09063
ENSG00000019995	0.016551	5.786175	0.980879	1	ZRANB1	K11862
ENSG00000183309	0.0165	5.299222	0.98089	1	ZNF623	K09228
ENSG00000235954	-0.023724	3.961296	0.980895	1	TTC28-AS1	_
ENSG00000128891	0.027248	2.949893	0.981029	1	C15orf57	_
ENSG00000107771	-0.016974	4.568154	0.981053	1	CCSER2	K12022
ENSG00000177508	0.019575	4.999448	0.981231	1	IRX3	_
ENSG00000171135	0.017211	6.191006	0.981411	1	JAGN1	K05168
ENSG00000230897	0.016018	6.191603	0.981411	1	RPS18P12	K02964
ENSG00000133794	0.019865	5.021221	0.981477	1	ARNTL	K02296
ENSG00000008838	0.017319	6.198192	0.981497	1	MED24	K15167
ENSG00000099622	0.016002	6.030517	0.981539	1	CIRBP	K13195
ENSG00000100154	0.020762	4.608061	0.981581	1	TTC28	_
ENSG00000158691	0.022433	4.016037	0.981594	1	ZSCAN12	K09230
ENSG00000184588	0.022433	4.016037	0.981594	1	PDE4B	K13293
ENSG00000105662	0.027609	2.99459	0.981624	1	CRTC1	K15309
ENSG00000261061	0.027609	2.99459	0.981624	1	RP11-303E16.2	_
ENSG00000157350	-0.018835	4.613654	0.981645	1	ST3GAL2	K03368
ENSG00000014164	-0.016866	5.360309	0.981685	1	ZC3H3	_
ENSG00000138663	0.016537	6.298554	0.981721	1	COPS4	K12178
ENSG00000127603	0.015788	7.13712	0.98179	1	MACF1	_
ENSG00000121864	0.017675	5.860527	0.981836	1	ZNF639	K12581
ENSG00000175061	0.015059	9.031318	0.981898	1	FAM211A-AS1	_
ENSG00000007541	-0.017159	6.234669	0.98196	1	PIGQ	K03860
ENSG00000166887	-0.019015	5.648664	0.981976	1	VPS39	K09228
ENSG00000196510	-0.015929	6.81001	0.982058	1	ANAPC7	K03354
ENSG00000085999	0.016177	5.655664	0.982067	1	RAD54L	K10875
ENSG00000163655	-0.015263	7.86721	0.982086	1	GMPS	K01951
ENSG00000138375	0.017882	5.070352	0.982089	1	SMARCAL1	K02159
ENSG00000108389	0.018033	5.080757	0.982225	1	MTMR4	K18082
ENSG00000198001	-0.024307	4.072345	0.982243	1	IRAK4	K04733
ENSG00000140006	0.023033	4.069255	0.982243	1	WDR89	K00757
ENSG00000124571	0.015589	6.822588	0.982292	1	XPO5	K14289
ENSG00000125651	0.015954	6.398638	0.982302	1	GTF2F1	K03138
ENSG00000131115	0.028111	3.059144	0.98236	1	ZNF227	K09228
ENSG00000133026	0.028111	3.059144	0.98236	1	MYH10	K10352
ENSG00000168904	0.028111	3.059144	0.98236	1	LRRC28	_
ENSG00000185761	-0.020436	5.095021	0.982402	1	ADAMTSL5	K15686
ENSG00000096080	0.014966	5.687501	0.982454	1	MRPS18A	K02963
ENSG00000135766	-0.015874	6.464084	0.982531	1	EGLN1	K09592
ENSG00000254166	0.038825	3.07459	0.982535	1	CASC19	_
ENSG00000110011	-0.035631	3.071142	0.982535	1	DNAJC4	K09524
ENSG00000101132	-0.01634	5.919631	0.982556	1	PFDN4	K09550
ENSG00000138802	0.016564	5.440245	0.982658	1	SEC24B	K14007
ENSG00000174197	-0.017313	5.931892	0.982702	1	MGA	_

ENSG00000115268	0.014178	10.00887	0.982831	1	RPS15	K02958
ENSG00000139725	-0.018099	4.718976	0.982893	1	RHOF	K07872
ENSG00000130589	0.018781	4.720475	0.982948	1	HELZ2	_
ENSG00000124209	0.016934	5.464287	0.98296	1	RAB22A	K07891
ENSG00000110047	0.015733	5.73439	0.983018	1	EHD1	K12483
ENSG00000138639	0.02857	3.120933	0.98304	1	ARHGAP24	_
ENSG00000051523	0.01439	7.86455	0.98307	1	CYBA	K08009
ENSG00000128789	-0.015448	5.740114	0.983072	1	PSMG2	K11876
ENSG00000120868	-0.021236	4.143518	0.983095	1	APAF1	K02084
ENSG00000164011	0.023908	4.150522	0.983176	1	ZNF691	_
ENSG00000112759	-0.015159	6.584126	0.983248	1	SLC29A1	K15014
ENSG00000220205	-0.025685	4.155913	0.983256	1	VAMP2	K13504
ENSG00000130489	-0.015016	5.168792	0.983279	1	SCO2	K07152
ENSG00000140497	-0.015867	5.492814	0.983283	1	SCAMP2	_
ENSG00000108829	0.014032	8.464723	0.983289	1	LRRC59	_
ENSG00000183864	0.016631	5.983463	0.983311	1	TOB2	K14443
ENSG00000147324	0.019325	4.759858	0.983377	1	MFHAS1	_
ENSG00000135241	0.019413	4.766318	0.983481	1	PNPLA8	K16815
ENSG00000154114	0.028855	3.160704	0.983517	1	TBCEL	K18273
ENSG00000141404	-0.019223	4.772908	0.983532	1	GNAL	K04633
ENSG00000105298	0.017173	5.191723	0.983552	1	CACTIN	K17985
ENSG00000257267	-0.019612	4.182667	0.983568	1	ZNF271	K09230
ENSG00000135414	-0.015812	4.777699	0.983583	1	GDF11	K05497
ENSG00000163975	0.015847	5.519831	0.983595	1	MFI2	K06569
ENSG00000131148	0.015908	5.523649	0.983625	1	EMC8	_
ENSG00000182670	0.014169	7.222114	0.983699	1	TTC3	K15712
ENSG00000154640	-0.015573	6.214737	0.983733	1	BTG3	K14443
ENSG00000147548	-0.016387	6.027765	0.983815	1	WHSC1L1	K11425
ENSG00000156603	-0.020783	4.812467	0.983981	1	MED19	K15137
ENSG00000167985	-0.017459	4.817129	0.98403	1	SDHAF2	K18168
ENSG00000075420	-0.014278	6.747314	0.984132	1	FNDC3B	_
ENSG00000124614	-0.013445	8.222073	0.984144	1	RPS10	K02947
ENSG00000153989	-0.014062	6.93999	0.984198	1	NUS1	K19177
ENSG00000178234	-0.016618	5.251703	0.984215	1	GALNT11	K00710
ENSG00000107625	-0.014763	5.843015	0.984233	1	DDX50	K13183
ENSG00000109572	0.017264	4.837085	0.984267	1	CLCN3	K05012
ENSG00000006194	-0.016149	5.846828	0.98428	1	ZNF263	K09229
ENSG00000158122	0.015632	4.250757	0.9843	1	AAED1	_
ENSG00000105397	-0.015769	6.078615	0.984368	1	TYK2	K11219
ENSG00000068654	0.014571	5.855987	0.984373	1	POLR1A	K02999
ENSG00000073712	-0.013786	7.059874	0.984426	1	FERMT2	K17083
ENSG00000244038	-0.014107	6.636639	0.984463	1	DDOST	K12670
ENSG00000204673	-0.014314	6.396985	0.984517	1	AKT1S1	K16184
ENSG00000120158	-0.01557	4.866062	0.984543	1	RCL1	K11222
ENSG00000163781	-0.013966	6.687731	0.984577	1	TOPBP1	K10728
ENSG00000000419	0.013913	6.516378	0.984641	1	DPM1	K00721
ENSG00000184677	0.025227	4.282578	0.984643	1	ZBTB40	K10512
ENSG00000215717	0.013494	5.88076	0.984646	1	TMEM167B	_
ENSG00000168005	-0.015117	4.878041	0.984678	1	C11orf84	_
ENSG00000206418	0.016548	5.885224	0.98469	1	RAB12	K07907
ENSG00000221869	-0.013925	6.592789	0.984737	1	CEBPD	K10050
ENSG00000113161	0.013867	6.457209	0.984766	1	HMGCR	K00021
ENSG00000198556	0.020472	3.278551	0.984798	1	ZNF789	K11447
ENSG00000262580	0.020472	3.278551	0.984798	1	RP11-334C17.5	_
ENSG00000109171	0.016774	5.316934	0.984892	1	SLAIN2	K16580
ENSG00000137713	-0.013339	5.659988	0.985094	1	PPP2R1B	K03456
ENSG00000132716	-0.01466	5.671218	0.985219	1	DCAF8	K11804
ENSG00000256663	0.021374	4.337876	0.985222	1	RP11-424C20.2	K10638

ENSG00000061987	0.014738	5.673819	0.985243	1	MON2	_
ENSG00000093183	0.015784	4.933532	0.985275	1	SEC22C	K08520
ENSG00000100280	0.012964	6.970847	0.985355	1	AP1B1	K12392
ENSG00000147316	-0.02106	4.368234	0.985526	1	MCPH1	K19403
ENSG00000159884	0.021363	3.349093	0.985547	1	CCDC107	_
ENSG00000105443	0.016325	4.967571	0.985596	1	CYTH2	K18441
ENSG00000186575	-0.014938	5.709433	0.985605	1	NF2	K16684
ENSG00000197969	-0.012237	5.992392	0.985781	1	VPS13A	K19525
ENSG00000165097	0.016181	5.415795	0.98589	1	KDM1B	K19413
ENSG00000166337	-0.013194	6.004053	0.985895	1	TAF10	K03134
ENSG00000188215	0.021977	3.399827	0.986006	1	DCUN1D3	K17823
ENSG00000152443	0.03048	3.412083	0.986117	1	ZNF776	K09228
ENSG00000076321	0.022172	3.416349	0.986117	1	KLHL20	K10457
ENSG00000168214	0.014649	5.767776	0.98617	1	RBPJ	K06053
ENSG00000112118	0.01166	8.389801	0.986203	1	MCM3	K02541
ENSG00000176155	0.014689	5.449308	0.986221	1	CCDC57	_
ENSG00000183648	-0.014761	5.038755	0.98627	1	NDUFB1	K03957
ENSG00000160087	0.013335	5.784581	0.98632	1	UBE2J2	K04554
ENSG00000088325	-0.011502	8.422886	0.98644	1	TPX2	K16812
ENSG00000104824	0.011748	7.47599	0.986487	1	HNRNPL	K13159
ENSG00000099203	-0.020334	4.484452	0.986625	1	TMED1	_
ENSG00000152133	-0.016463	4.482467	0.986625	1	GPATCH11	K12581
ENSG00000198924	-0.016162	4.490291	0.986675	1	DCLRE1A	_
ENSG00000071189	-0.015767	5.082124	0.986685	1	SNX13	K17925
ENSG00000163257	0.015451	5.082058	0.986685	1	DCAF16	K02927
ENSG00000147799	0.015406	4.49788	0.986725	1	ARHGAP39	_
ENSG00000077380	-0.012473	6.097958	0.986777	1	DYNC1I2	K10415
ENSG00000197857	0.023084	3.496238	0.986843	1	ZNF44	K09228
ENSG00000143179	-0.011635	7.289704	0.986859	1	UCK2	K00876
ENSG00000100299	-0.01461	5.112861	0.98698	1	ARSA	K01134
ENSG00000177971	0.012124	6.360169	0.987	1	IMP3	K12756
ENSG00000270170	-0.01175	5.1217	0.987044	1	NCBP2-AS2	_
ENSG00000136888	0.011274	7.424581	0.987094	1	ATP6V1G1	K02152
ENSG00000169635	0.023422	3.526996	0.987131	1	HIC2	_
ENSG00000180902	0.023422	3.526996	0.987131	1	D2HGDH	_
ENSG00000079999	-0.013538	5.876246	0.987158	1	KEAP1	K10456
ENSG00000138399	-0.017852	4.545803	0.987161	1	FASTKD1	_
ENSG00000108788	0.013598	5.886702	0.987251	1	MLX	K09113
ENSG00000166965	-0.011544	5.890365	0.987288	1	RCCD1	_
ENSG00000130560	0.011723	6.501925	0.987319	1	UBAC1	K12174
ENSG00000120725	-0.011068	6.173421	0.987452	1	SIL1	K14001
ENSG00000170385	0.010898	7.405045	0.987564	1	SLC30A1	K14688
ENSG00000103342	-0.010423	8.780303	0.98765	1	GSPT1	K03267
ENSG00000107404	-0.011312	6.807681	0.987674	1	DVL1	K02353
ENSG00000182149	-0.011298	6.76235	0.987738	1	IST1	K19476
ENSG00000100075	0.024205	3.601139	0.987757	1	SLC25A1	K15100
ENSG00000142208	0.011515	6.327773	0.98778	1	AKT1	K04456
ENSG00000254087	0.01214	5.96602	0.987932	1	LYN	K05854
ENSG00000186635	0.011348	6.31901	0.987994	1	ARAP1	K18439
ENSG00000177738	-0.025876	3.626989	0.988008	1	CTD-2201E18.3	_
ENSG00000141101	0.011442	6.002733	0.988238	1	NOB1	K11883
ENSG00000182541	0.017796	3.661403	0.988248	1	LIMK2	K05744
ENSG00000164244	-0.010923	6.701712	0.98826	1	PRRC1	K12581
ENSG00000111231	0.011584	5.689199	0.988322	1	GPN3	K06883
ENSG00000016864	-0.012802	6.014945	0.988331	1	GLT8D1	_
ENSG00000160710	0.009824	8.253441	0.988485	1	ADAR	K12968
ENSG00000172819	-0.010848	6.57635	0.988497	1	RARG	K08529
ENSG00000115596	-0.011399	5.714456	0.988512	1	WNT6	K00445

ENSG00000126215	-0.015061	4.710612	0.988539	1	XRCC3	K10880
ENSG00000106803	0.010756	6.399142	0.988614	1	SEC61B	K09481
ENSG00000130052	-0.015563	3.706253	0.988627	1	STARD8	_
ENSG00000156531	-0.010845	6.46559	0.988638	1	PHF6	_
ENSG00000230453	0.018586	3.715812	0.9887	1	ANKRD18B	K17299
ENSG00000171970	-0.021652	3.723042	0.988772	1	ZNF57	K09228
ENSG00000198858	0.011141	5.75321	0.988822	1	R3HDM4	_
ENSG00000187522	0.011588	6.08062	0.988848	1	HSPA14	_
ENSG00000177303	0.009392	4.751783	0.988865	1	CASKIN2	_
ENSG00000130584	-0.020006	3.76233	0.989051	1	ZBTB46	K10517
ENSG00000183723	0.011308	5.376218	0.989123	1	CMTM4	_
ENSG00000168397	0.010427	5.798829	0.989165	1	ATG4B	K08342
ENSG00000173611	0.019493	3.781061	0.989186	1	SCAI	_
ENSG00000198722	0.016792	4.806066	0.989272	1	UNC13B	K15293
ENSG00000166037	0.009602	7.170052	0.989295	1	CEP57	K16762
ENSG00000106299	-0.010538	6.153231	0.989399	1	WASL	K05747
ENSG00000078747	0.011765	6.156977	0.989424	1	ITCH	K05632
ENSG00000006125	-0.010206	6.455134	0.989429	1	AP2B1	K11825
ENSG00000103966	-0.012472	5.421956	0.98947	1	EHD4	K12477
ENSG00000123933	-0.012472	5.421956	0.98947	1	MXD4	K09114
ENSG00000131871	-0.0109	6.178274	0.989574	1	VIMP	K14025
ENSG00000179152	-0.013739	5.443279	0.989615	1	TCAIM	_
ENSG00000106344	-0.011009	5.862748	0.989628	1	RBM28	K14573
ENSG00000163013	0.010856	5.459312	0.989736	1	FBXO41	_
ENSG00000154237	0.020634	3.867721	0.989811	1	LRRK1	K08843
ENSG00000110700	-0.008657	8.492012	0.989888	1	RPS13	K02953
ENSG00000204253	0.008872	3.885779	0.989928	1	HNRNPCP2	K12884
ENSG00000154380	-0.008926	7.631941	0.989934	1	ENAH	K05746
ENSG00000100410	0.009725	6.292952	0.989999	1	PHF5A	K12834
ENSG00000178464	-0.009749	6.377615	0.990087	1	CTD-2192J16.15	K02866
ENSG00000172888	-0.014229	3.909596	0.990098	1	ZNF621	K09228
ENSG00000167136	-0.013799	3.921217	0.990153	1	ENDOG	K01173
ENSG00000054148	0.010211	6.260713	0.990157	1	PHPT1	K01112
ENSG00000169813	-0.008378	8.694001	0.990168	1	HNRNPF	K12898
ENSG00000168303	-0.01115	5.943351	0.990191	1	MPLKIP	K17985
ENSG00000196981	0.021371	3.926583	0.990208	1	WDR5B	K14963
ENSG00000154124	0.009327	6.479483	0.990255	1	FAM105B	K18343
ENSG00000075856	0.009404	6.277204	0.990265	1	SART3	_
ENSG00000158793	-0.012349	4.953568	0.990305	1	NIT1	K05750
ENSG00000119574	0.015778	3.940997	0.990316	1	ZBTB45	K10516
ENSG00000163155	0.010068	3.943916	0.990316	1	LYSMD1	_
ENSG00000185104	0.008913	6.909403	0.990335	1	FAF1	_
ENSG00000160957	0.008762	7.129278	0.990347	1	RECQL4	K10730
ENSG00000253797	-0.011948	4.964845	0.990384	1	UTP14C	K14567
ENSG00000107186	0.013532	4.96898	0.99041	1	MPDZ	K06095
ENSG00000123364	-0.014303	4.977434	0.990462	1	HOXC13	K09298
ENSG00000198746	0.016142	3.963659	0.990473	1	GPATCH3	_
ENSG00000134901	-0.010973	4.992657	0.990564	1	KDELC1	_
ENSG00000116473	-0.009306	6.421765	0.990566	1	RAP1A	K04353
ENSG00000101290	0.011283	4.998098	0.99059	1	CDS2	K00981
ENSG00000196428	-0.010782	4.998156	0.99059	1	TSC22D2	_
ENSG00000109606	-0.00832	7.676784	0.990646	1	DHX15	K12820
ENSG00000080839	-0.02181	4.005632	0.990724	1	RBL1	K04681
ENSG00000106052	-0.008133	7.721313	0.99085	1	TAX1BP1	_
ENSG00000063244	0.008209	7.375755	0.990857	1	U2AF2	K12837
ENSG00000123130	0.008685	6.628766	0.990875	1	ACOT9	K17361
ENSG00000137822	0.012493	5.06251	0.990998	1	TUBGCP4	K16571
ENSG00000087087	-0.008533	6.844029	0.991017	1	SRRT	_

ENSG00000144711	-0.019575	4.059204	0.991053	1	IQSEC1	K12495
ENSG00000141219	0.012685	5.072971	0.991067	1	C17orf80	_
ENSG00000137812	-0.009566	5.668638	0.991116	1	CASC5	_
ENSG00000033100	0.008724	6.351054	0.99114	1	CHPF2	K03419
ENSG00000253626	-0.018312	4.090418	0.991232	1	EIF5AL1	K03263
ENSG00000181788	0.010033	5.696828	0.991277	1	SIAH2	K08742
ENSG00000108953	0.007234	9.482741	0.99137	1	YWHAE	K06630
ENSG00000182934	0.007845	7.144853	0.991452	1	SRPR	K13431
ENSG00000160360	0.008705	4.138264	0.991527	1	GPSM1	K15839
ENSG00000077463	-0.012786	5.16268	0.991599	1	SIRT6	K11416
ENSG00000224870	-0.015942	4.150894	0.991608	1	RP4-758J18.2	_
ENSG00000067369	-0.008672	6.172211	0.991625	1	TP53BP1	_
ENSG00000051128	-0.008366	6.450132	0.99167	1	HOMER3	K15010
ENSG00000105643	0.009625	5.176049	0.991679	1	ARRDC2	_
ENSG00000119638	-0.008353	6.181909	0.991684	1	NEK9	K08857
ENSG00000071626	-0.007381	7.878478	0.991686	1	DAZAP1	K14411
ENSG00000127463	-0.008195	6.186734	0.991713	1	EMC1	_
ENSG00000164649	-0.01192	5.186959	0.991737	1	CDCA7L	K10408
ENSG00000253570	-0.01192	5.186959	0.991737	1	RNF5P1	K10666
ENSG00000164180	-0.011083	5.210836	0.991871	1	TMEM161B	_
ENSG00000147789	0.010489	5.801977	0.991893	1	ZNF7	K09228
ENSG00000112851	-0.007753	6.890976	0.99192	1	ERBB2IP	K12796
ENSG00000144455	-0.013415	4.218389	0.991989	1	SUMF1	K13444
ENSG00000235174	0.00667	9.457258	0.992062	1	RPL39P3	K02924
ENSG00000105576	0.00794	6.314047	0.992139	1	TNPO2	K18727
ENSG00000133703	-0.008074	6.330989	0.992187	1	KRAS	K07827
ENSG00000104447	0.01121	4.262267	0.992202	1	TRPS1	_
ENSG00000173660	-0.006943	7.808411	0.992254	1	UQCRH	K00416
ENSG00000141736	-0.007548	5.876255	0.992295	1	ERBB2	K05083
ENSG00000244313	0.007149	7.141437	0.992302	1	RP11-425L10.1	K02947
ENSG00000113742	-0.015227	4.294124	0.992371	1	CPEB4	K02602
ENSG00000127184	0.006531	8.195206	0.99252	1	COX7C	K02272
ENSG00000149573	0.012394	4.324873	0.992533	1	MPZL2	_
ENSG00000074855	0.012555	4.333599	0.992595	1	ANO8	K19502
ENSG00000065268	0.008649	5.933373	0.992597	1	WDR18	K14829
ENSG00000169710	-0.006186	9.433081	0.992683	1	FASN	K00665
ENSG00000113569	-0.006509	7.809782	0.992783	1	NUP155	K14312
ENSG00000151151	-0.009677	5.382551	0.992786	1	IPMK	K00915
ENSG00000100030	0.006692	7.203155	0.992816	1	MAPK1	K04371
ENSG00000143514	-0.009395	5.390937	0.99283	1	TP53BP2	K16823
ENSG00000146063	-0.009395	5.390937	0.99283	1	TRIM41	K12017
ENSG00000090316	-0.007063	5.981338	0.992837	1	MAEA	_
ENSG00000125170	0.009555	5.39406	0.992845	1	DOK4	_
ENSG00000163935	-0.011748	4.389247	0.992865	1	SFMBT1	_
ENSG00000135040	-0.008703	5.411689	0.992931	1	NAA35	_
ENSG00000166595	0.009974	5.41477	0.992945	1	FAM96B	_
ENSG00000149532	-0.007273	6.483846	0.992954	1	CPSF7	K14398
ENSG00000130811	0.006451	7.320021	0.993033	1	EIF3G	K03248
ENSG00000126254	0.006889	6.627873	0.993058	1	RBM42	_
ENSG00000110274	-0.01031	4.430497	0.993062	1	CEP164	K16462
ENSG00000269131	-0.013736	4.448708	0.993143	1	AC004447.2	_
ENSG00000100941	0.006082	7.590752	0.993326	1	PNN	K13114
ENSG00000166200	-0.006131	7.607877	0.993354	1	COPS2	K12176
ENSG00000104388	0.005982	7.77383	0.993358	1	RAB2A	K07877
ENSG00000123349	-0.005807	8.331533	0.993418	1	PFDN5	K04797
ENSG00000069869	0.008174	5.51983	0.993431	1	NEDD4	K10591
ENSG00000068489	-0.008556	5.535986	0.993504	1	PRR11	_
ENSG00000167797	0.012114	4.530527	0.993521	1	CDK2AP2	_

ENSG00000100416	0.007575	6.134044	0.993554	1	TRMU	K00566
ENSG00000068878	0.006412	6.684399	0.993582	1	PSME4	K06699
ENSG00000164039	0.012533	4.553119	0.993615	1	BDH2	K00019
ENSG00000169567	-0.00563	8.227687	0.993671	1	HINT1	K02503
ENSG00000196236	-0.009404	4.571804	0.993706	1	XPNPEP3	K01262
ENSG00000165434	0.009274	4.577222	0.993729	1	PGM2L1	K11809
ENSG00000160908	0.009574	4.591844	0.993795	1	ZNF394	K09229
ENSG00000179271	0.005717	7.471025	0.993836	1	GADD45GIP1	_
ENSG00000181904	-0.006968	6.224762	0.993945	1	C5orf24	K12581
ENSG00000088930	0.00589	6.905006	0.994017	1	XRN2	K12619
ENSG00000124608	0.007238	4.650658	0.994026	1	AARS2	K01872
ENSG00000198131	0.005824	5.660868	0.994042	1	ZNF544	K09228
ENSG00000103855	-0.005583	7.457558	0.994113	1	CD276	K06746
ENSG00000124155	-0.005591	7.285522	0.994222	1	PIGT	K05292
ENSG00000059573	0.005369	7.539572	0.994222	1	ALDH18A1	K12657
ENSG00000156873	0.011646	4.697027	0.994222	1	PHKG2	K00871
ENSG00000168488	0.006197	6.285653	0.994291	1	ATXN2L	_
ENSG00000106261	0.007272	5.725291	0.994302	1	ZKSCAN1	K09229
ENSG00000085832	0.007344	5.728603	0.994312	1	EPS15	K12472
ENSG00000198146	0.007417	5.731908	0.99433	1	ZNF770	K17985
ENSG00000113851	-0.007775	4.727232	0.994334	1	CRBN	K11793
ENSG00000161847	0.009122	4.738702	0.994388	1	RAVER1	_
ENSG00000205155	0.009122	4.738702	0.994388	1	PSENEN	K06170
ENSG00000120833	-0.006929	4.753479	0.994441	1	SOCS2	K04695
ENSG00000163808	-0.008847	4.79357	0.994595	1	KIF15	K10400
ENSG00000011258	0.008238	4.847819	0.994787	1	MBTD1	_
ENSG00000187266	-0.010034	4.850926	0.994802	1	EPOR	K05079
ENSG00000108021	0.005705	6.312938	0.994854	1	FAM208B	_
ENSG00000214253	-0.005249	5.902156	0.994955	1	FIS1	K17969
ENSG00000054116	0.005419	6.522564	0.994956	1	TRAPPC3	_
ENSG00000101146	-0.007669	5.918195	0.995012	1	RAE1	K14298
ENSG00000114867	0.004327	8.876277	0.995022	1	EIF4G1	K03260
ENSG00000134686	-0.004628	5.922545	0.995026	1	PHC2	K11457
ENSG00000142039	-0.007481	4.927851	0.995064	1	CCDC97	_
ENSG00000110917	-0.004789	7.46726	0.995075	1	MLEC	_
ENSG00000224032	0.008784	5.010403	0.995338	1	EPB41L4A-AS1	_
ENSG00000188290	-0.005455	6.345091	0.995348	1	HES4	K09089
ENSG00000164024	0.005231	6.339717	0.995396	1	METAP1	K01265
ENSG00000165669	0.009344	5.037363	0.995423	1	FAM204A	_
ENSG00000135801	0.009562	5.048007	0.995458	1	TAF5L	K03130
ENSG00000174165	0.009562	5.048007	0.995458	1	ZDHHC24	K18932
ENSG00000015676	-0.006091	6.055085	0.995462	1	NUDCD3	_
ENSG00000197043	0.00445	7.209085	0.995541	1	ANXA6	K17094
ENSG00000138496	0.005046	5.08208	0.995561	1	PARP9	K15260
ENSG00000160972	-0.004732	6.099255	0.995598	1	PPP1R16A	K17458
ENSG00000055950	0.006853	6.101199	0.995603	1	MRPL43	K17424
ENSG00000127616	0.004269	7.409405	0.995636	1	SMARCA4	K11647
ENSG00000159140	-0.003928	7.990338	0.995843	1	SON	_
ENSG00000112078	0.004695	6.491704	0.99587	1	KCTD20	_
ENSG00000101577	-0.006853	5.194157	0.995893	1	LPIN2	K15728
ENSG00000149798	-0.006701	5.19894	0.995902	1	CDC42EP2	_
ENSG00000128989	-0.004093	7.47394	0.995918	1	ARPP19	_
ENSG00000174939	-0.008901	5.204901	0.995921	1	ASPHD1	_
ENSG00000233137	-0.004652	6.223595	0.995961	1	RP11-220I1.1	_
ENSG00000112242	-0.004468	6.26809	0.996082	1	E2F3	K06620
ENSG00000095059	-0.004848	6.329363	0.996106	1	DHPS	K00809
ENSG00000168884	-0.006355	5.283566	0.996136	1	TNIP2	_
ENSG00000119139	0.004924	5.282428	0.996136	1	TJP2	_

ENSG00000115446	-0.007419	5.320251	0.996235	1	UNC50	_
ENSG00000182224	-0.007278	5.324635	0.996243	1	CYB5D1	K11642
ENSG00000196975	0.004338	6.517304	0.996276	1	ANXA4	K17093
ENSG00000160131	-0.004512	6.510075	0.996276	1	VMA21	_
ENSG00000063177	-0.003238	9.339806	0.996296	1	RPL18	K02883
ENSG00000112079	-0.00449	6.478455	0.996343	1	STK38	K08790
ENSG00000166508	0.003321	8.493421	0.996344	1	MCM7	K02210
ENSG00000131584	0.005023	5.379386	0.996382	1	ACAP3	K12489
ENSG00000120539	0.004295	6.30019	0.996583	1	MASTL	K16309
ENSG00000101868	-0.004048	6.733819	0.996585	1	POLA1	K02320
ENSG00000169217	-0.004788	5.471227	0.996605	1	CD2BP2	K13099
ENSG00000143493	0.005478	5.485979	0.996637	1	INTS7	K13144
ENSG00000111300	0.004106	6.338088	0.996764	1	NAA25	K17973
ENSG00000065883	-0.005407	5.576068	0.996842	1	CDK13	K08819
ENSG00000166881	-0.00393	6.622503	0.996851	1	TMEM194A	_
ENSG00000074201	0.003222	7.387827	0.996922	1	CLNS1A	K05019
ENSG00000119844	0.004755	5.615071	0.996925	1	AFTPH	_
ENSG00000177613	-0.004558	5.662593	0.997024	1	CSTF2T	K14407
ENSG00000149923	0.003027	5.879999	0.997439	1	PPP4C	K15423
ENSG00000055332	-0.003506	6.511521	0.997498	1	EIF2AK2	K16195
ENSG00000173456	0.003966	5.918215	0.997504	1	RNF26	K15693
ENSG00000128944	-0.002841	7.161443	0.997663	1	KNSTRN	_
ENSG00000124333	-0.003468	6.098618	0.997797	1	VAMP7	K08515
ENSG00000198218	-0.004577	6.104364	0.997806	1	QRICH1	_
ENSG00000007080	-0.003049	6.652459	0.997888	1	CCDC124	K12581
ENSG00000171928	0.003474	6.266403	0.998038	1	TVP23B	_
ENSG00000188895	0.002379	7.074335	0.998156	1	MSL1	K13163
ENSG00000110330	0.002523	6.827564	0.99818	1	BIRC2	K16060
ENSG00000213585	0.001721	8.905273	0.998197	1	VDAC1	K05862
ENSG00000182087	0.001842	7.753019	0.998414	1	TMEM259	_
ENSG00000065809	-0.002308	6.900207	0.998536	1	FAM107B	_
ENSG00000160213	-0.002374	6.73465	0.99862	1	CSTB	K13907
ENSG00000157020	0.001398	7.294522	0.999202	1	SEC13	K14004
ENSG00000160679	-0.001669	6.948233	0.999269	1	CHTOP	K09228
ENSG00000205913	0.038659	0.986652	1	1	SRRM2-AS1	_
ENSG00000103599	-0.028133	1.474493	1	1	IQCH	_
ENSG00000067221	-0.023356	2.134172	1	1	STOML1	_
ENSG00000177721	0.018365	2.152261	1	1	ANXA2R	_
ENSG00000272462	0.018365	2.152261	1	1	U91328.19	_
ENSG00000234771	0.017768	2.112278	1	1	RP11-395P17.3	_
ENSG00000169814	-0.005977	3.572418	1	1	BTD	K01435
ENSG00000260114	-0.117956	0.883016	1	1	CTD-2574D22.4	_
ENSG00000262185	-0.117956	0.883016	1	1	RP11-462G12.1	_
ENSG00000142347	-0.11215	0.406004	1	1	MYO1F	K10356
ENSG00000145736	-0.11215	0.406004	1	1	GTF2H2	K08857
ENSG00000187736	-0.11215	0.406004	1	1	NHEJ1	K10980
ENSG00000241043	-0.11215	0.406004	1	1	GVQW1	K02311
ENSG00000259153	-0.11215	0.406004	1	1	RP6-65G23.3	_
ENSG00000261589	-0.11215	0.406004	1	1	CTC-462L7.1	_
ENSG00000100890	0.11192	0.420428	1	1	KIAA0391	K17655
ENSG00000102385	0.11192	0.420428	1	1	DRP2	_
ENSG00000151474	0.11192	0.420428	1	1	FRMD4A	_
ENSG00000164707	0.11192	0.420428	1	1	SLC13A4	K14444
ENSG00000198518	0.11192	0.420428	1	1	HIST1H4E	K11254
ENSG00000227986	0.11192	0.420428	1	1	TRIM60P18	K12029
ENSG00000070087	0.105175	0.545937	1	1	PFN2	K05759
ENSG00000070388	0.105175	0.545937	1	1	FGF22	K04358
ENSG00000080298	0.105175	0.545937	1	1	RFX3	K09173

ENSG00000253616	0.105175	0.545937	1	1	RP11-875O11.3	_
ENSG00000254979	0.105175	0.545937	1	1	RP11-872D17.8	K08230
ENSG00000255242	0.105175	0.545937	1	1	C14orf169	_
ENSG00000258366	0.105175	0.545937	1	1	RTEL1	K11136
ENSG00000269486	0.105175	0.545937	1	1	CTC-360G5.9	_
ENSG00000269867	0.105175	0.545937	1	1	CTD-2583A14.8	_
ENSG00000273270	0.105175	0.545937	1	1	RP11-212P7.2	_
ENSG00000187105	0.090792	0.86781	1	1	HEATR4	_
ENSG00000213846	0.090792	0.86781	1	1	AC098614.2	K10375
ENSG00000232284	0.090792	0.86781	1	1	GNG12-AS1	_
ENSG00000185046	-0.076099	0.755352	1	1	ANKS1B	_
ENSG00000234684	-0.076099	0.755352	1	1	SDCBP2-AS1	_
ENSG00000261761	-0.076099	0.755352	1	1	RP11-103J17.2	_
ENSG00000272604	-0.076099	0.755352	1	1	RP11-251G23.5	_
ENSG00000186153	-0.067627	0.8553	1	1	WWOX	K19329
ENSG00000261971	-0.067627	0.8553	1	1	RP11-473M20.7	_
ENSG00000172458	0.038643	0.895389	1	1	IL17D	_
ENSG00000181826	0.038581	0.580883	1	1	RELL1	_
ENSG00000198929	0.038581	0.580883	1	1	NOS1AP	K16513
ENSG00000254469	0.038581	0.580883	1	1	RP11-849H4.2	K04965
ENSG00000260083	0.038581	0.580883	1	1	MIR4519	_
ENSG00000099889	-0.016081	3.692759	1	1	ARVCF	_
ENSG00000165121	-0.011731	2.422547	1	1	RP11-213G2.3	K10395
ENSG00000077782	0.011383	3.69236	1	1	FGFR1	K04362
ENSG00000167642	-0.009794	3.675608	1	1	SPINT2	_
ENSG00000068976	0.162062	-0.231544	1	1	PYGM	K00688
ENSG00000116128	0.162062	-0.231544	1	1	BCL9	_
ENSG00000135835	0.162062	-0.231544	1	1	KIAA1614	_
ENSG00000143552	0.162062	-0.231544	1	1	NUP210L	K14314
ENSG00000147003	0.162062	-0.231544	1	1	TMEM27	_
ENSG00000180425	0.162062	-0.231544	1	1	C11orf71	_
ENSG00000197122	0.162062	-0.231544	1	1	SRC	K05704
ENSG00000224536	0.162062	-0.231544	1	1	RP11-134G8.7	_
ENSG00000248866	0.162062	-0.231544	1	1	USP46-AS1	_
ENSG00000255867	0.162062	-0.231544	1	1	DENND5B-AS1	_
ENSG00000257557	0.162062	-0.231544	1	1	RP11-84G21.1	_
ENSG00000272301	0.162062	-0.231544	1	1	RP11-111M22.4	_
ENSG00000124920	0.038796	2.395936	1	1	MYRF	_
ENSG00000245213	0.038765	1.927668	1	1	RP11-10K16.1	_
ENSG00000135925	0.038342	-0.170038	1	1	WNT10A	K13194
ENSG00000142530	0.038342	-0.170038	1	1	FAM71E1	_
ENSG00000151150	0.038342	-0.170038	1	1	ANK3	K10380
ENSG00000167037	0.038342	-0.170038	1	1	SGSM1	_
ENSG00000183889	0.038342	-0.170038	1	1	NPIPA7	K04985
ENSG00000187024	0.038342	-0.170038	1	1	PTRH1	K01056
ENSG00000187984	0.038342	-0.170038	1	1	ANKRD19P	K17299
ENSG00000197837	0.038342	-0.170038	1	1	HIST4H4	_
ENSG00000204882	0.038342	-0.170038	1	1	GPR20	K04317
ENSG00000204920	0.038342	-0.170038	1	1	ZNF155	_
ENSG00000233058	0.038342	-0.170038	1	1	LINC00884	_
ENSG00000254206	0.038342	-0.170038	1	1	NPIPB11	K01896
ENSG00000257740	0.038342	-0.170038	1	1	RP11-977G19.12	_
ENSG00000268087	0.038342	-0.170038	1	1	CTC-429P9.2	_
ENSG00000270964	0.038342	-0.170038	1	1	RP11-502I4.3	_
ENSG00000273117	0.038342	-0.170038	1	1	AC144652.1	_
ENSG00000137821	-0.034979	1.342723	1	1	LRRC49	K16606
ENSG00000148291	-0.034979	1.342723	1	1	SURF2	_
ENSG00000158406	-0.034979	1.342723	1	1	HIST1H4H	K11254

ENSG00000186141	-0.034979	1.342723	1	1	POLR3C	K03023
ENSG00000108733	0.028269	3.080035	1	1	PEX12	K13345
ENSG00000137267	0.019485	3.204383	1	1	TUBB2A	K07375
ENSG00000234171	-0.013963	3.081031	1	1	RNASEH1-AS1	_
ENSG00000143375	0.009518	3.190255	1	1	CGN	K06102
ENSG00000089916	-0.008912	4.586479	1	1	GPATCH2L	K17569
ENSG00000172113	-0.006566	3.963915	1	1	NME6	K00940
ENSG00000115282	0.006202	4.024319	1	1	TTC31	_
ENSG00000184277	0.005511	4.405773	1	1	TM2D3	_
ENSG00000197429	0.005442	3.006105	1	1	IPP	K13956
ENSG00000244165	0.005442	3.006105	1	1	P2RY11	K08387
ENSG00000257732	0.005442	3.006105	1	1	RP11-818F20.5	_
ENSG00000108528	-0.00333	6.059043	1	1	SLC25A11	K15104
ENSG00000112182	-0.144529	0.68026	1	1	BACH2	K09042
ENSG00000120049	-0.144529	0.68026	1	1	KCNIP2	_
ENSG00000121653	-0.144529	0.68026	1	1	MAPK8IP1	K04434
ENSG00000141028	-0.144529	0.68026	1	1	CDRT15P1	_
ENSG00000142065	-0.144529	0.68026	1	1	ZFP14	K09228
ENSG00000175449	-0.144529	0.68026	1	1	RFESD	_
ENSG00000213261	-0.144529	0.68026	1	1	AC093106.7	K03232
ENSG00000249550	-0.144529	0.68026	1	1	RP11-438N16.1	_
ENSG00000250222	-0.144529	0.68026	1	1	CTC-338M12.5	_
ENSG00000273137	-0.144529	0.68026	1	1	RP3-402G11.28	_
ENSG00000125378	0.09957	0.661394	1	1	BMP4	K04662
ENSG00000127366	0.09957	0.661394	1	1	TAS2R5	K08474
ENSG00000157150	0.09957	0.661394	1	1	TIMP4	_
ENSG00000175730	0.09957	0.661394	1	1	BAK1P1	K14021
ENSG00000187492	0.09957	0.661394	1	1	CDHR4	K16504
ENSG00000241343	0.09957	0.661394	1	1	RPL36A	K02929
ENSG00000196421	-0.086037	0.647996	1	1	LINC00176	_
ENSG00000248121	-0.086037	0.647996	1	1	SMURF2P1	K04678
ENSG00000254198	-0.086037	0.647996	1	1	RP11-598P20.3	_
ENSG00000256673	-0.086037	0.647996	1	1	RP11-599J14.2	_
ENSG00000268189	-0.086037	0.647996	1	1	AC005785.2	_
ENSG00000128564	-0.042578	2.471897	1	1	VGF	_
ENSG00000168152	-0.042578	2.471897	1	1	THAP9	K12606
ENSG00000156510	-0.039043	2.222042	1	1	HKDC1	K00844
ENSG00000019169	0.022528	2.469609	1	1	MARCO	K13884
ENSG00000131979	-0.019324	3.421207	1	1	GCH1	K01495
ENSG00000265415	0.013622	3.40413	1	1	CTD-2510F5.4	_
ENSG00000266962	-0.013585	3.759006	1	1	RP11-400F19.6	_
ENSG00000136052	-0.008429	4.361758	1	1	SLC41A2	K15122
ENSG00000174749	0.007456	4.300636	1	1	C4orf32	K12581
ENSG00000149596	0.006286	4.608138	1	1	JPH2	K19530
ENSG00000162378	-0.003847	4.507671	1	1	ZYG11B	K10350
ENSG00000173264	0.003247	4.48038	1	1	GPR137	_
ENSG00000107185	-0.001655	5.441259	1	1	RGP1	_
ENSG00000004939	3.724485	-2.294189	1	1	SLC4A1	K06573
ENSG00000005469	3.724485	-2.294189	1	1	CROT	K05940
ENSG00000006555	3.724485	-2.294189	1	1	TTC22	_
ENSG00000009709	3.724485	-2.294189	1	1	PAX7	K09381
ENSG00000010626	3.724485	-2.294189	1	1	LRRC23	_
ENSG00000018869	3.724485	-2.294189	1	1	ZNF582	K09228
ENSG00000026559	3.724485	-2.294189	1	1	KCNG1	K04900
ENSG00000028137	3.724485	-2.294189	1	1	TNFRSF1B	K05141
ENSG00000048545	3.724485	-2.294189	1	1	GUCA1A	K08328
ENSG00000052850	3.724485	-2.294189	1	1	ALX4	K09451
ENSG00000055732	3.724485	-2.294189	1	1	MCOLN3	K04994

ENSG00000058404	3.724485	-2.294189	1	1	CAMK2B	K04515
ENSG00000060709	3.724485	-2.294189	1	1	RIMBP2	K17591
ENSG00000064886	3.724485	-2.294189	1	1	CHI3L2	K17523
ENSG00000066032	3.724485	-2.294189	1	1	CTNNA2	K05691
ENSG00000066056	3.724485	-2.294189	1	1	TIE1	K05120
ENSG00000067715	3.724485	-2.294189	1	1	SYT1	K15290
ENSG00000071282	3.724485	-2.294189	1	1	LMCD1	_
ENSG00000074935	3.724485	-2.294189	1	1	TUBE1	K10391
ENSG00000075884	3.724485	-2.294189	1	1	ARHGAP15	_
ENSG00000078018	3.724485	-2.294189	1	1	MAP2	K10430
ENSG00000080561	3.724485	-2.294189	1	1	MID2	K10647
ENSG00000081138	3.724485	-2.294189	1	1	CDH7	K06799
ENSG00000081148	3.724485	-2.294189	1	1	IMPG2	K19017
ENSG00000081248	3.724485	-2.294189	1	1	CACNA1S	K04857
ENSG00000083290	3.724485	-2.294189	1	1	ULK2	K08269
ENSG00000083814	3.724485	-2.294189	1	1	ZNF671	K09228
ENSG00000099937	3.724485	-2.294189	1	1	SERPIND1	K03912
ENSG00000099954	3.724485	-2.294189	1	1	CECR2	_
ENSG00000100362	3.724485	-2.294189	1	1	PVALB	_
ENSG00000100427	3.724485	-2.294189	1	1	MLC1	_
ENSG00000100628	3.724485	-2.294189	1	1	ASB2	K10324
ENSG00000100652	3.724485	-2.294189	1	1	SLC10A1	K14341
ENSG00000101082	3.724485	-2.294189	1	1	SLA2	K11447
ENSG00000101203	3.724485	-2.294189	1	1	COL20A1	_
ENSG00000101336	3.724485	-2.294189	1	1	HCK	K08893
ENSG00000101443	3.724485	-2.294189	1	1	WFDC2	_
ENSG00000101542	3.724485	-2.294189	1	1	CDH20	K06807
ENSG00000102466	3.724485	-2.294189	1	1	FGF14	K04358
ENSG00000102678	3.724485	-2.294189	1	1	FGF9	K04358
ENSG00000102802	3.724485	-2.294189	1	1	MEDAG	_
ENSG00000102837	3.724485	-2.294189	1	1	OLFM4	K04592
ENSG00000103490	3.724485	-2.294189	1	1	PYCARD	K12799
ENSG00000104848	3.724485	-2.294189	1	1	KCNA7	K04880
ENSG00000104921	3.724485	-2.294189	1	1	FCER2	K06468
ENSG00000105374	3.724485	-2.294189	1	1	NKG7	_
ENSG00000105409	3.724485	-2.294189	1	1	ATP1A3	K01539
ENSG00000105605	3.724485	-2.294189	1	1	CACNG7	K04872
ENSG00000105784	3.724485	-2.294189	1	1	RUNDC3B	K05659
ENSG00000106327	3.724485	-2.294189	1	1	TFR2	_
ENSG00000106927	3.724485	-2.294189	1	1	AMBP	_
ENSG00000107485	3.724485	-2.294189	1	1	GATA3	K17895
ENSG00000108452	3.724485	-2.294189	1	1	ZNF29P	K09228
ENSG00000110328	3.724485	-2.294189	1	1	GALNT18	K00710
ENSG00000111218	3.724485	-2.294189	1	1	PRMT8	K11439
ENSG00000111305	3.724485	-2.294189	1	1	GSG1	_
ENSG00000111665	3.724485	-2.294189	1	1	CDCA3	K07825
ENSG00000112299	3.724485	-2.294189	1	1	VNN1	K08069
ENSG00000114547	3.724485	-2.294189	1	1	ROPN1B	_
ENSG00000115386	3.724485	-2.294189	1	1	REG1A	_
ENSG00000115461	3.724485	-2.294189	1	1	IGFBP5	K10138
ENSG00000116157	3.724485	-2.294189	1	1	GPX7	K00432
ENSG00000116544	3.724485	-2.294189	1	1	DLGAP3	K15008
ENSG00000116771	3.724485	-2.294189	1	1	AGMAT	K01480
ENSG00000117215	3.724485	-2.294189	1	1	PLA2G2D	K01047
ENSG00000118004	3.724485	-2.294189	1	1	COLEC11	K10066
ENSG00000118017	3.724485	-2.294189	1	1	A4GNT	K08251
ENSG00000118473	3.724485	-2.294189	1	1	SGIP1	_
ENSG00000118514	3.724485	-2.294189	1	1	ALDH8A1	_

ENSG00000118785	3.724485	-2.294189	1	1	SPP1	K06250
ENSG00000119283	3.724485	-2.294189	1	1	TRIM67	K10649
ENSG00000120337	3.724485	-2.294189	1	1	TNFSF18	K05479
ENSG00000122718	3.724485	-2.294189	1	1	OR2S2	K04257
ENSG00000124194	3.724485	-2.294189	1	1	GDAP1L1	_
ENSG00000124701	3.724485	-2.294189	1	1	APOBEC2	K18772
ENSG00000124731	3.724485	-2.294189	1	1	TREM1	K14362
ENSG00000124935	3.724485	-2.294189	1	1	SCGB1D2	_
ENSG00000125245	3.724485	-2.294189	1	1	GPR18	K08410
ENSG00000125337	3.724485	-2.294189	1	1	KIF25	K18627
ENSG00000125735	3.724485	-2.294189	1	1	TNFSF14	K05477
ENSG00000126952	3.724485	-2.294189	1	1	NXF5	K14284
ENSG00000127083	3.724485	-2.294189	1	1	OMD	K08124
ENSG00000127362	3.724485	-2.294189	1	1	TAS2R3	K08474
ENSG00000128594	3.724485	-2.294189	1	1	LRRC4	K16351
ENSG00000128604	3.724485	-2.294189	1	1	IRF5	K09446
ENSG00000129538	3.724485	-2.294189	1	1	RNASE1	K01168
ENSG00000129673	3.724485	-2.294189	1	1	AANAT	K00669
ENSG00000130055	3.724485	-2.294189	1	1	GDPD2	K01124
ENSG00000130300	3.724485	-2.294189	1	1	PLVAP	K17309
ENSG00000130649	3.724485	-2.294189	1	1	CYP2E1	K07415
ENSG00000131379	3.724485	-2.294189	1	1	C3orf20	_
ENSG00000131620	3.724485	-2.294189	1	1	ANO1	K19496
ENSG00000131781	3.724485	-2.294189	1	1	FMO5	K00485
ENSG00000132164	3.724485	-2.294189	1	1	SLC6A11	K05039
ENSG00000132855	3.724485	-2.294189	1	1	ANGPTL3	_
ENSG00000132874	3.724485	-2.294189	1	1	SLC14A2	K08716
ENSG00000133110	3.724485	-2.294189	1	1	POSTN	_
ENSG00000133138	3.724485	-2.294189	1	1	TBC1D8B	_
ENSG00000133739	3.724485	-2.294189	1	1	LRRCC1	K16475
ENSG00000134160	3.724485	-2.294189	1	1	TRPM1	K04976
ENSG00000134398	3.724485	-2.294189	1	1	ERN2	K11715
ENSG00000134757	3.724485	-2.294189	1	1	DSG3	K07598
ENSG00000134874	3.724485	-2.294189	1	1	DZIP1	K16470
ENSG00000134955	3.724485	-2.294189	1	1	SLC37A2	K13783
ENSG00000135226	3.724485	-2.294189	1	1	UGT2B28	K00699
ENSG00000135363	3.724485	-2.294189	1	1	LMO2	K15612
ENSG00000136193	3.724485	-2.294189	1	1	SCRN1	K14358
ENSG00000136275	3.724485	-2.294189	1	1	C7orf69	_
ENSG00000136541	3.724485	-2.294189	1	1	ERMN	_
ENSG00000136689	3.724485	-2.294189	1	1	IL1RN	K05481
ENSG00000136872	3.724485	-2.294189	1	1	ALDOB	K01623
ENSG00000138039	3.724485	-2.294189	1	1	LHCGR	K04248
ENSG00000138759	3.724485	-2.294189	1	1	FRAS1	_
ENSG00000138964	3.724485	-2.294189	1	1	PARVG	K06275
ENSG00000139219	3.724485	-2.294189	1	1	COL2A1	K19719
ENSG00000139679	3.724485	-2.294189	1	1	LPAR6	_
ENSG00000139874	3.724485	-2.294189	1	1	SSTR1	K04217
ENSG00000140107	3.724485	-2.294189	1	1	SLC25A47	K15123
ENSG00000140505	3.724485	-2.294189	1	1	CYP1A2	K07409
ENSG00000140557	3.724485	-2.294189	1	1	ST8SIA2	K06612
ENSG00000141086	3.724485	-2.294189	1	1	CTRL	K09632
ENSG00000141497	3.724485	-2.294189	1	1	ZMYND15	_
ENSG00000142224	3.724485	-2.294189	1	1	IL19	K05444
ENSG00000142609	3.724485	-2.294189	1	1	C1orf222	_
ENSG00000142615	3.724485	-2.294189	1	1	CELA2A	K01346
ENSG00000143452	3.724485	-2.294189	1	1	HORMAD1	_
ENSG00000143631	3.724485	-2.294189	1	1	FLG	K10384

ENSG00000143839	3.724485	-2.294189	1	1	REN	K01380
ENSG00000144035	3.724485	-2.294189	1	1	NAT8	_
ENSG00000144366	3.724485	-2.294189	1	1	GULP1	_
ENSG00000144837	3.724485	-2.294189	1	1	PLA1A	K13618
ENSG00000145284	3.724485	-2.294189	1	1	SCD5	K00507
ENSG00000146411	3.724485	-2.294189	1	1	SLC2A12	K08149
ENSG00000147099	3.724485	-2.294189	1	1	HDAC8	K11405
ENSG00000147145	3.724485	-2.294189	1	1	LPAR4	_
ENSG00000147202	3.724485	-2.294189	1	1	DIAPH2	K05741
ENSG00000147234	3.724485	-2.294189	1	1	FRMPD3	_
ENSG00000149488	3.724485	-2.294189	1	1	TMC2	K12581
ENSG00000149575	3.724485	-2.294189	1	1	SCN2B	K04846
ENSG00000150471	3.724485	-2.294189	1	1	LPHN3	K04594
ENSG00000150510	3.724485	-2.294189	1	1	FAM124A	_
ENSG00000150551	3.724485	-2.294189	1	1	LYPD1	_
ENSG00000150783	3.724485	-2.294189	1	1	TEX12	K19537
ENSG00000151360	3.724485	-2.294189	1	1	ALLC	K01477
ENSG00000151468	3.724485	-2.294189	1	1	CCDC3	_
ENSG00000151575	3.724485	-2.294189	1	1	TEX9	_
ENSG00000152086	3.724485	-2.294189	1	1	TUBA3E	K07374
ENSG00000152154	3.724485	-2.294189	1	1	TMEM178A	_
ENSG00000152229	3.724485	-2.294189	1	1	PSTPIP2	K17985
ENSG00000152430	3.724485	-2.294189	1	1	BOLL	K17985
ENSG00000152760	3.724485	-2.294189	1	1	TCTEX1D1	_
ENSG00000152932	3.724485	-2.294189	1	1	RAB3C	K07883
ENSG00000152977	3.724485	-2.294189	1	1	ZIC1	_
ENSG00000153086	3.724485	-2.294189	1	1	ACMSD	K03392
ENSG00000153446	3.724485	-2.294189	1	1	C16orf89	K12022
ENSG00000153495	3.724485	-2.294189	1	1	TEX29	_
ENSG00000154133	3.724485	-2.294189	1	1	ROBO4	K06784
ENSG00000154342	3.724485	-2.294189	1	1	WNT3A	K00312
ENSG00000155980	3.724485	-2.294189	1	1	KIF5A	K10396
ENSG00000156574	3.724485	-2.294189	1	1	NODAL	K04666
ENSG00000156575	3.724485	-2.294189	1	1	PRG3	_
ENSG00000156920	3.724485	-2.294189	1	1	GPR112	K08455
ENSG00000157680	3.724485	-2.294189	1	1	DGKI	K00901
ENSG00000158445	3.724485	-2.294189	1	1	KCNB1	K04885
ENSG00000158714	3.724485	-2.294189	1	1	SLAMF8	K16853
ENSG00000159708	3.724485	-2.294189	1	1	LRRC36	K16594
ENSG00000160321	3.724485	-2.294189	1	1	ZNF208	K09228
ENSG00000160801	3.724485	-2.294189	1	1	PTH1R	K04585
ENSG00000160868	3.724485	-2.294189	1	1	CYP3A4	K17689
ENSG00000162753	3.724485	-2.294189	1	1	SLC9C2	K14726
ENSG00000162771	3.724485	-2.294189	1	1	FAM71A	_
ENSG00000162873	3.724485	-2.294189	1	1	KLHDC8A	_
ENSG00000163154	3.724485	-2.294189	1	1	TNFAIP8L2	_
ENSG00000163297	3.724485	-2.294189	1	1	ANTXR2	_
ENSG00000163497	3.724485	-2.294189	1	1	FEV	K09437
ENSG00000163618	3.724485	-2.294189	1	1	CADPS	_
ENSG00000163632	3.724485	-2.294189	1	1	C3orf49	K12581
ENSG00000163817	3.724485	-2.294189	1	1	SLC6A20	K05048
ENSG00000164007	3.724485	-2.294189	1	1	CLDN19	K06087
ENSG00000164303	3.724485	-2.294189	1	1	ENPP6	K08743
ENSG00000164458	3.724485	-2.294189	1	1	T	K10172
ENSG00000164841	3.724485	-2.294189	1	1	TMEM74	_
ENSG00000164946	3.724485	-2.294189	1	1	FREM1	_
ENSG00000165457	3.724485	-2.294189	1	1	FOLR2	K13649
ENSG00000165917	3.724485	-2.294189	1	1	RAPSN	_

ENSG00000166856	3.724485	-2.294189	1	1	GPR182	K04242
ENSG00000167104	3.724485	-2.294189	1	1	BPIFB6	_
ENSG00000167165	3.724485	-2.294189	1	1	UGT1A6	K09509
ENSG00000167183	3.724485	-2.294189	1	1	PRR15L	_
ENSG00000167414	3.724485	-2.294189	1	1	GNG8	K04544
ENSG00000167619	3.724485	-2.294189	1	1	TMEM145	K17985
ENSG00000167754	3.724485	-2.294189	1	1	KLK5	K09617
ENSG00000167941	3.724485	-2.294189	1	1	SOST	K16834
ENSG00000168032	3.724485	-2.294189	1	1	ENTPD3	K01510
ENSG00000168148	3.724485	-2.294189	1	1	HIST3H3	K11253
ENSG00000168269	3.724485	-2.294189	1	1	FOXI1	K09401
ENSG00000168386	3.724485	-2.294189	1	1	FILIP1L	_
ENSG00000168754	3.724485	-2.294189	1	1	FAM178B	_
ENSG00000168878	3.724485	-2.294189	1	1	SFTPB	K12581
ENSG00000168913	3.724485	-2.294189	1	1	ENHO	_
ENSG00000169989	3.724485	-2.294189	1	1	TIGD4	_
ENSG00000170044	3.724485	-2.294189	1	1	ZPLD1	_
ENSG00000170289	3.724485	-2.294189	1	1	CNGB3	K04953
ENSG00000170290	3.724485	-2.294189	1	1	SLN	_
ENSG00000170498	3.724485	-2.294189	1	1	KISS1	_
ENSG00000170627	3.724485	-2.294189	1	1	GTSF1	_
ENSG00000170629	3.724485	-2.294189	1	1	DPY19L2P2	K09131
ENSG00000170962	3.724485	-2.294189	1	1	PDGFD	K05450
ENSG00000171051	3.724485	-2.294189	1	1	FPR1	K04172
ENSG00000171502	3.724485	-2.294189	1	1	COL24A1	K06236
ENSG00000171533	3.724485	-2.294189	1	1	MAP6	K10432
ENSG00000171855	3.724485	-2.294189	1	1	IFNB1	K05415
ENSG00000171873	3.724485	-2.294189	1	1	ADRA1D	K04137
ENSG00000172058	3.724485	-2.294189	1	1	SERF1A	K12022
ENSG00000172349	3.724485	-2.294189	1	1	IL16	_
ENSG00000172367	3.724485	-2.294189	1	1	PDZD3	_
ENSG00000172403	3.724485	-2.294189	1	1	SYNPO2	_
ENSG00000172955	3.724485	-2.294189	1	1	ADH6	K13952
ENSG00000173124	3.724485	-2.294189	1	1	C10orf129	K01896
ENSG00000173673	3.724485	-2.294189	1	1	HES3	K09088
ENSG00000174111	3.724485	-2.294189	1	1	SOCS7	K04699
ENSG00000174358	3.724485	-2.294189	1	1	SLC6A19	K05334
ENSG00000174448	3.724485	-2.294189	1	1	STARD6	_
ENSG00000174562	3.724485	-2.294189	1	1	KLK15	K09623
ENSG00000174576	3.724485	-2.294189	1	1	NPAS4	_
ENSG00000175206	3.724485	-2.294189	1	1	NPPA	K12334
ENSG00000175538	3.724485	-2.294189	1	1	KCNE3	K04897
ENSG00000175773	3.724485	-2.294189	1	1	RP11-121M22.1	_
ENSG00000175806	3.724485	-2.294189	1	1	MSRA	K07304
ENSG00000176244	3.724485	-2.294189	1	1	ACBD7	K12581
ENSG00000176601	3.724485	-2.294189	1	1	MAP3K19	K17533
ENSG00000176774	3.724485	-2.294189	1	1	MAGEB18	_
ENSG00000176945	3.724485	-2.294189	1	1	MUC20	K17985
ENSG00000177197	3.724485	-2.294189	1	1	PCNPP5	_
ENSG00000177202	3.724485	-2.294189	1	1	SPACA4	_
ENSG00000177238	3.724485	-2.294189	1	1	TRIM72	K12036
ENSG00000177432	3.724485	-2.294189	1	1	NAP1L5	K11283
ENSG00000177464	3.724485	-2.294189	1	1	GPR4	_
ENSG00000177669	3.724485	-2.294189	1	1	MBOAT4	_
ENSG00000177990	3.724485	-2.294189	1	1	DPY19L2	_
ENSG00000178162	3.724485	-2.294189	1	1	FAR2P2	_
ENSG00000178723	3.724485	-2.294189	1	1	GLULP4	K01915
ENSG00000178772	3.724485	-2.294189	1	1	CPN2	K13023

ENSG00000178860	3.724485	-2.294189	1	1	MSC	K09072
ENSG00000178882	3.724485	-2.294189	1	1	FAM101A	_
ENSG00000178896	3.724485	-2.294189	1	1	EXOSC4	K11600
ENSG00000178972	3.724485	-2.294189	1	1	AC097658.1	K17985
ENSG00000179381	3.724485	-2.294189	1	1	OR4K11P	K04257
ENSG00000179452	3.724485	-2.294189	1	1	RP11-380B22.1	_
ENSG00000180279	3.724485	-2.294189	1	1	CTD-2568A17.5	_
ENSG00000180999	3.724485	-2.294189	1	1	C1orf105	_
ENSG00000181097	3.724485	-2.294189	1	1	RP11-429J17.2	_
ENSG00000181260	3.724485	-2.294189	1	1	MTHFD2P7	K13403
ENSG00000181355	3.724485	-2.294189	1	1	OFCC1	_
ENSG00000181541	3.724485	-2.294189	1	1	MAB21L2	_
ENSG00000181908	3.724485	-2.294189	1	1	AP003774.4	K10408
ENSG00000182040	3.724485	-2.294189	1	1	USH1G	_
ENSG00000182230	3.724485	-2.294189	1	1	FAM153B	_
ENSG00000182333	3.724485	-2.294189	1	1	LIPF	K14452
ENSG00000182625	3.724485	-2.294189	1	1	RP11-123C21.1	K04545
ENSG00000183150	3.724485	-2.294189	1	1	GPR19	_
ENSG00000183169	3.724485	-2.294189	1	1	POM121L1P	_
ENSG00000183185	3.724485	-2.294189	1	1	GABRR3	K05190
ENSG00000183379	3.724485	-2.294189	1	1	SYNDIG1L	_
ENSG00000183813	3.724485	-2.294189	1	1	CCR4	K04179
ENSG00000184033	3.724485	-2.294189	1	1	CTAG1B	_
ENSG00000184224	3.724485	-2.294189	1	1	C11orf72	_
ENSG00000184347	3.724485	-2.294189	1	1	SLIT3	K06850
ENSG00000184486	3.724485	-2.294189	1	1	POU3F2	K09365
ENSG00000184490	3.724485	-2.294189	1	1	AP000351.9	K00799
ENSG00000184635	3.724485	-2.294189	1	1	ZNF93	K09228
ENSG00000185775	3.724485	-2.294189	1	1	SPATA31A6	_
ENSG00000186150	3.724485	-2.294189	1	1	UBL4B	_
ENSG00000186160	3.724485	-2.294189	1	1	CYP4Z1	K07429
ENSG00000186166	3.724485	-2.294189	1	1	CCDC84	_
ENSG00000186197	3.724485	-2.294189	1	1	EDARADD	_
ENSG00000186198	3.724485	-2.294189	1	1	SLC51B	K14361
ENSG00000186790	3.724485	-2.294189	1	1	FOXE3	K09398
ENSG00000186919	3.724485	-2.294189	1	1	ZACN	K16888
ENSG00000186976	3.724485	-2.294189	1	1	EFCAB6	_
ENSG00000186977	3.724485	-2.294189	1	1	KRTAP19-5	_
ENSG00000187122	3.724485	-2.294189	1	1	SLIT1	_
ENSG00000187416	3.724485	-2.294189	1	1	LHFPL3	_
ENSG00000187456	3.724485	-2.294189	1	1	RDM1	K10874
ENSG00000187479	3.724485	-2.294189	1	1	C11orf96	_
ENSG00000187486	3.724485	-2.294189	1	1	KCNJ11	K05004
ENSG00000187513	3.724485	-2.294189	1	1	GJA4	K07613
ENSG00000187601	3.724485	-2.294189	1	1	MAGEH1	_
ENSG00000187791	3.724485	-2.294189	1	1	FAM205CP	_
ENSG00000187867	3.724485	-2.294189	1	1	PALM3	_
ENSG00000188257	3.724485	-2.294189	1	1	PLA2G2A	K01047
ENSG00000188770	3.724485	-2.294189	1	1	OPTC	K08128
ENSG00000189001	3.724485	-2.294189	1	1	SBSN	_
ENSG00000189099	3.724485	-2.294189	1	1	PRSS48	_
ENSG00000189275	3.724485	-2.294189	1	1	AL450307.1	_
ENSG00000196361	3.724485	-2.294189	1	1	ELAVL3	K13208
ENSG00000196689	3.724485	-2.294189	1	1	TRPV1	K05222
ENSG00000196704	3.724485	-2.294189	1	1	AMZ2	K06974
ENSG00000196814	3.724485	-2.294189	1	1	MVB12B	K12186
ENSG00000196927	3.724485	-2.294189	1	1	AC009093.1	K12581
ENSG00000196960	3.724485	-2.294189	1	1	RP11-192P3.5	_

ENSG00000197284	3.724485	-2.294189	1	1	RP11-589F5.3	_
ENSG00000197360	3.724485	-2.294189	1	1	ZNF98	K09228
ENSG00000197416	3.724485	-2.294189	1	1	FABP12	_
ENSG00000197632	3.724485	-2.294189	1	1	SERPINB2	K13963
ENSG00000197815	3.724485	-2.294189	1	1	AC122129.1	K17985
ENSG00000197888	3.724485	-2.294189	1	1	UGT2B17	K00699
ENSG00000198211	3.724485	-2.294189	1	1	TUBB3	K07375
ENSG00000198221	3.724485	-2.294189	1	1	MLLT4-AS1	_
ENSG00000198331	3.724485	-2.294189	1	1	HYLS1	K16472
ENSG00000198384	3.724485	-2.294189	1	1	TPTE2P3	K18079
ENSG00000198471	3.724485	-2.294189	1	1	RTP2	_
ENSG00000198523	3.724485	-2.294189	1	1	PLN	K05852
ENSG00000198547	3.724485	-2.294189	1	1	C20orf203	_
ENSG00000198574	3.724485	-2.294189	1	1	SH2D1B	K07989
ENSG00000198632	3.724485	-2.294189	1	1	AC004878.3	K10858
ENSG00000198739	3.724485	-2.294189	1	1	LRRTM3	_
ENSG00000198758	3.724485	-2.294189	1	1	EPS8L3	K17277
ENSG00000198822	3.724485	-2.294189	1	1	GRM3	K04605
ENSG00000198870	3.724485	-2.294189	1	1	C9orf96	K17546
ENSG00000199047	3.724485	-2.294189	1	1	MIR378A	_
ENSG00000199082	3.724485	-2.294189	1	1	MIR342	_
ENSG00000199132	3.724485	-2.294189	1	1	MIR450A1	_
ENSG00000199545	3.724485	-2.294189	1	1	RNA5SP195	_
ENSG00000199875	3.724485	-2.294189	1	1	Y_RNA	_
ENSG00000200033	3.724485	-2.294189	1	1	RNU6-403P	_
ENSG00000200115	3.724485	-2.294189	1	1	RNA5SP440	_
ENSG00000200142	3.724485	-2.294189	1	1	Y_RNA	_
ENSG00000200181	3.724485	-2.294189	1	1	SNORD85	_
ENSG00000200294	3.724485	-2.294189	1	1	SNORA36	_
ENSG00000200301	3.724485	-2.294189	1	1	RNA5SP483	_
ENSG00000200397	3.724485	-2.294189	1	1	Y_RNA	_
ENSG00000200403	3.724485	-2.294189	1	1	RNU6-1099P	_
ENSG00000200437	3.724485	-2.294189	1	1	RNU6-799P	_
ENSG00000200652	3.724485	-2.294189	1	1	SNORA25	_
ENSG00000200788	3.724485	-2.294189	1	1	Y_RNA	_
ENSG00000200834	3.724485	-2.294189	1	1	Y_RNA	_
ENSG00000200842	3.724485	-2.294189	1	1	Y_RNA	_
ENSG00000200997	3.724485	-2.294189	1	1	RNU1-85P	_
ENSG00000201071	3.724485	-2.294189	1	1	Y_RNA	_
ENSG00000201076	3.724485	-2.294189	1	1	RNU4-51P	_
ENSG00000201185	3.724485	-2.294189	1	1	RNA5SP202	_
ENSG00000201441	3.724485	-2.294189	1	1	RNU6-646P	_
ENSG00000201547	3.724485	-2.294189	1	1	Y_RNA	_
ENSG00000201616	3.724485	-2.294189	1	1	RNU1-91P	_
ENSG00000201619	3.724485	-2.294189	1	1	SNORA67	_
ENSG00000201898	3.724485	-2.294189	1	1	SNORA72	_
ENSG00000202107	3.724485	-2.294189	1	1	SNORD103B	_
ENSG00000202119	3.724485	-2.294189	1	1	RNU6-302P	_
ENSG00000202222	3.724485	-2.294189	1	1	Y_RNA	_
ENSG00000202347	3.724485	-2.294189	1	1	RNU1-16P	_
ENSG00000202472	3.724485	-2.294189	1	1	RNA5SP248	_
ENSG00000202487	3.724485	-2.294189	1	1	Y_RNA	_
ENSG00000202522	3.724485	-2.294189	1	1	Y_RNA	_
ENSG00000202538	3.724485	-2.294189	1	1	RNU4-2	_
ENSG00000203306	3.724485	-2.294189	1	1	AP001007.1	_
ENSG00000203325	3.724485	-2.294189	1	1	RP11-277A4.4	_
ENSG00000203441	3.724485	-2.294189	1	1	LINC00449	_
ENSG00000203446	3.724485	-2.294189	1	1	AC004988.1	_

ENSG00000203542	3.724485	-2.294189	1	1	AL353671.1	K02927
ENSG00000203615	3.724485	-2.294189	1	1	AC069200.1	K09131
ENSG00000203697	3.724485	-2.294189	1	1	CAPN8	K08577
ENSG00000203849	3.724485	-2.294189	1	1	RP11-417J8.6	_
ENSG00000203852	3.724485	-2.294189	1	1	HIST2H3A	K11253
ENSG00000203863	3.724485	-2.294189	1	1	AL079342.1	K10408
ENSG00000204113	3.724485	-2.294189	1	1	BMP2KL	K08854
ENSG00000204150	3.724485	-2.294189	1	1	CTGLF11P	K12491
ENSG00000204304	3.724485	-2.294189	1	1	PBX2	K15609
ENSG00000204396	3.724485	-2.294189	1	1	VWA7	_
ENSG00000204913	3.724485	-2.294189	1	1	LRRC3C	_
ENSG00000205015	3.724485	-2.294189	1	1	RP11-46C24.3	_
ENSG00000205108	3.724485	-2.294189	1	1	FAM205A	_
ENSG00000205334	3.724485	-2.294189	1	1	AC074091.13	K12581
ENSG00000205537	3.724485	-2.294189	1	1	RP11-89H19.1	_
ENSG00000205832	3.724485	-2.294189	1	1	C16orf96	_
ENSG00000205867	3.724485	-2.294189	1	1	KRTAP5-2	_
ENSG00000206013	3.724485	-2.294189	1	1	IFITM5	K06566
ENSG00000206028	3.724485	-2.294189	1	1	CTA-373H7.7	_
ENSG00000206082	3.724485	-2.294189	1	1	LINC01002	_
ENSG00000206090	3.724485	-2.294189	1	1	AP000350.7	K00799
ENSG00000206549	3.724485	-2.294189	1	1	PRSS50	_
ENSG00000206605	3.724485	-2.294189	1	1	RNU6-946P	_
ENSG00000206646	3.724485	-2.294189	1	1	Y_RNA	_
ENSG00000206712	3.724485	-2.294189	1	1	RNU6-26P	_
ENSG00000206713	3.724485	-2.294189	1	1	Y_RNA	_
ENSG00000206768	3.724485	-2.294189	1	1	Y_RNA	_
ENSG00000206775	3.724485	-2.294189	1	1	SNORD37	_
ENSG00000206786	3.724485	-2.294189	1	1	RNU6-701P	_
ENSG00000206859	3.724485	-2.294189	1	1	RNU6-767P	_
ENSG00000206887	3.724485	-2.294189	1	1	RNU6-1008P	_
ENSG00000206989	3.724485	-2.294189	1	1	SNORD63	_
ENSG00000207003	3.724485	-2.294189	1	1	RNU6-611P	_
ENSG00000207008	3.724485	-2.294189	1	1	SNORA54	_
ENSG00000207009	3.724485	-2.294189	1	1	Y_RNA	_
ENSG00000207021	3.724485	-2.294189	1	1	Y_RNA	_
ENSG00000207042	3.724485	-2.294189	1	1	RNU6-196P	_
ENSG00000207175	3.724485	-2.294189	1	1	RNU1-67P	_
ENSG00000207186	3.724485	-2.294189	1	1	RNA5SP122	_
ENSG00000207281	3.724485	-2.294189	1	1	Y_RNA	_
ENSG00000207370	3.724485	-2.294189	1	1	Y_RNA	_
ENSG00000207467	3.724485	-2.294189	1	1	Y_RNA	_
ENSG00000207631	3.724485	-2.294189	1	1	MIR641	_
ENSG00000207696	3.724485	-2.294189	1	1	MIR659	_
ENSG00000207924	3.724485	-2.294189	1	1	MIR196A2	_
ENSG00000207988	3.724485	-2.294189	1	1	MIR576	_
ENSG00000207997	3.724485	-2.294189	1	1	MIR644A	_
ENSG00000211581	3.724485	-2.294189	1	1	MIR765	_
ENSG00000211644	3.724485	-2.294189	1	1	IGLV1-51	_
ENSG00000211959	3.724485	-2.294189	1	1	IGHV4-39	K06856
ENSG00000212242	3.724485	-2.294189	1	1	RNA5SP219	_
ENSG00000212342	3.724485	-2.294189	1	1	SNORA12	_
ENSG00000212378	3.724485	-2.294189	1	1	SNORD78	_
ENSG00000212440	3.724485	-2.294189	1	1	SNORA75	_
ENSG00000212466	3.724485	-2.294189	1	1	RNU6-952P	_
ENSG00000212526	3.724485	-2.294189	1	1	RNU6-466P	_
ENSG00000212533	3.724485	-2.294189	1	1	SNORA75	_
ENSG00000212536	3.724485	-2.294189	1	1	RNA5SP474	_

ENSG00000212542	3.724485	-2.294189	1	1	RNA5SP496	_
ENSG00000212939	3.724485	-2.294189	1	1	RP1-29C18.10	_
ENSG00000213058	3.724485	-2.294189	1	1	RP4-765C7.2	K02955
ENSG00000213131	3.724485	-2.294189	1	1	YWHAZP4	K16197
ENSG00000213212	3.724485	-2.294189	1	1	NCLP1	K11294
ENSG00000213216	3.724485	-2.294189	1	1	RP11-355O1.7	K02975
ENSG00000213315	3.724485	-2.294189	1	1	CTD-3035D6.1	K02964
ENSG00000213559	3.724485	-2.294189	1	1	HNRNPA1P64	K12741
ENSG00000213598	3.724485	-2.294189	1	1	RP11-112J1.1	K02910
ENSG00000213673	3.724485	-2.294189	1	1	SLC25A5P3	K05863
ENSG00000213703	3.724485	-2.294189	1	1	RP5-849H19.2	K02872
ENSG00000213731	3.724485	-2.294189	1	1	RAB5CP1	K07889
ENSG00000213757	3.724485	-2.294189	1	1	CTC-451P13.1	K02984
ENSG00000213857	3.724485	-2.294189	1	1	RP11-12M9.4	K05692
ENSG00000213863	3.724485	-2.294189	1	1	XX-C2158C12.2	_
ENSG00000213871	3.724485	-2.294189	1	1	TAF9BP1	K03133
ENSG00000213872	3.724485	-2.294189	1	1	AC092798.2	K02912
ENSG00000213985	3.724485	-2.294189	1	1	AC078899.1	K17260
ENSG00000214018	3.724485	-2.294189	1	1	RRM2P3	K10808
ENSG00000214027	3.724485	-2.294189	1	1	ARPC3P5	K05756
ENSG00000214124	3.724485	-2.294189	1	1	SNRPEP9	K11097
ENSG00000214264	3.724485	-2.294189	1	1	RP11-356J5.5	_
ENSG00000214289	3.724485	-2.294189	1	1	RPL39P5	K02924
ENSG00000214435	3.724485	-2.294189	1	1	AS3MT	K07755
ENSG00000214456	3.724485	-2.294189	1	1	PLIN5	K17985
ENSG00000214544	3.724485	-2.294189	1	1	GTF2IRD2P1	K03121
ENSG00000214688	3.724485	-2.294189	1	1	C10orf105	K06813
ENSG00000214886	3.724485	-2.294189	1	1	FAM177A1P1	_
ENSG00000214946	3.724485	-2.294189	1	1	TBC1D26	_
ENSG00000214998	3.724485	-2.294189	1	1	AC006465.4	K05868
ENSG00000215065	3.724485	-2.294189	1	1	DUSP8P4	K04459
ENSG00000215347	3.724485	-2.294189	1	1	SLC25A5P1	K05863
ENSG00000215483	3.724485	-2.294189	1	1	LINC00598	_
ENSG00000215529	3.724485	-2.294189	1	1	EFCAB8	_
ENSG00000215845	3.724485	-2.294189	1	1	TSTD1	_
ENSG00000215910	3.724485	-2.294189	1	1	C1orf167	K12831
ENSG00000215973	3.724485	-2.294189	1	1	MIR933	_
ENSG00000216193	3.724485	-2.294189	1	1	AC009336.1	_
ENSG00000217372	3.724485	-2.294189	1	1	TUBB4BP7	K07375
ENSG00000217648	3.724485	-2.294189	1	1	RP1-95L4.4	K09650
ENSG00000217746	3.724485	-2.294189	1	1	RP1-303A1.1	_
ENSG00000217950	3.724485	-2.294189	1	1	AC073869.20	K14833
ENSG00000218018	3.724485	-2.294189	1	1	RP4-800J21.3	_
ENSG00000218069	3.724485	-2.294189	1	1	RSL24D1P1	K02896
ENSG00000218793	3.724485	-2.294189	1	1	RP3-382I10.3	K01611
ENSG00000219085	3.724485	-2.294189	1	1	NPM1P37	K11276
ENSG00000219163	3.724485	-2.294189	1	1	HMGB1P20	K10802
ENSG00000219355	3.724485	-2.294189	1	1	RPL31P52	K02910
ENSG00000219435	3.724485	-2.294189	1	1	TEX40	_
ENSG00000219712	3.724485	-2.294189	1	1	RP11-532F6.2	_
ENSG00000219736	3.724485	-2.294189	1	1	RP11-560O20.1	K14295
ENSG00000219932	3.724485	-2.294189	1	1	RPL12P8	K02870
ENSG00000220583	3.724485	-2.294189	1	1	RPL35P2	K02918
ENSG00000220660	3.724485	-2.294189	1	1	RP3-406A7.3	_
ENSG00000220693	3.724485	-2.294189	1	1	AC011498.1	K17985
ENSG00000220695	3.724485	-2.294189	1	1	RP1-121G13.3	K17822
ENSG00000221169	3.724485	-2.294189	1	1	AC013444.2	_
ENSG00000221203	3.724485	-2.294189	1	1	MIR1262	_

ENSG00000221204	3.724485	-2.294189	1	1	AC106795.1	_
ENSG00000221230	3.724485	-2.294189	1	1	MIR548L	_
ENSG00000221272	3.724485	-2.294189	1	1	AC067742.1	_
ENSG00000221429	3.724485	-2.294189	1	1	AC018635.1	_
ENSG00000221878	3.724485	-2.294189	1	1	PSG7	K06500
ENSG00000222005	3.724485	-2.294189	1	1	LINC01118	_
ENSG00000222035	3.724485	-2.294189	1	1	AC079354.3	_
ENSG00000222044	3.724485	-2.294189	1	1	RP5-1039K5.16	_
ENSG00000222069	3.724485	-2.294189	1	1	RN7SKP285	_
ENSG00000222100	3.724485	-2.294189	1	1	RNA5SP316	_
ENSG00000222121	3.724485	-2.294189	1	1	AL512290.1	_
ENSG00000222190	3.724485	-2.294189	1	1	MIR1910	_
ENSG00000222282	3.724485	-2.294189	1	1	RNU6-584P	_
ENSG00000222370	3.724485	-2.294189	1	1	SNORA36B	_
ENSG00000222371	3.724485	-2.294189	1	1	RN7SKP202	_
ENSG00000222610	3.724485	-2.294189	1	1	RNU6-402P	_
ENSG00000222808	3.724485	-2.294189	1	1	RNU4-47P	_
ENSG00000223187	3.724485	-2.294189	1	1	Y_RNA	_
ENSG00000223188	3.724485	-2.294189	1	1	Y_RNA	_
ENSG00000223300	3.724485	-2.294189	1	1	Y_RNA	_
ENSG00000223432	3.724485	-2.294189	1	1	RP11-62L18.3	_
ENSG00000223462	3.724485	-2.294189	1	1	RP11-285G1.9	_
ENSG00000223511	3.724485	-2.294189	1	1	RP13-297E16.4	_
ENSG00000223704	3.724485	-2.294189	1	1	RP1-90L6.2	_
ENSG00000223787	3.724485	-2.294189	1	1	RP4-593M8.1	_
ENSG00000223825	3.724485	-2.294189	1	1	DAZAP2P1	_
ENSG00000223867	3.724485	-2.294189	1	1	AC098592.7	_
ENSG00000223878	3.724485	-2.294189	1	1	AC005517.3	K03767
ENSG00000223979	3.724485	-2.294189	1	1	SMCR2	_
ENSG00000223984	3.724485	-2.294189	1	1	HNRNPRP1	K13161
ENSG00000224077	3.724485	-2.294189	1	1	AP000936.4	_
ENSG00000224078	3.724485	-2.294189	1	1	SNHG14	_
ENSG00000224081	3.724485	-2.294189	1	1	LINC01057	_
ENSG00000224100	3.724485	-2.294189	1	1	AP001630.5	_
ENSG00000224155	3.724485	-2.294189	1	1	RP11-700P18.2	K11584
ENSG00000224232	3.724485	-2.294189	1	1	AC110299.5	_
ENSG00000224238	3.724485	-2.294189	1	1	WARS2-IT1	_
ENSG00000224276	3.724485	-2.294189	1	1	RP11-336K24.5	_
ENSG00000224324	3.724485	-2.294189	1	1	THAP5P1	_
ENSG00000224349	3.724485	-2.294189	1	1	RP11-462G2.2	_
ENSG00000224460	3.724485	-2.294189	1	1	RP11-439L18.2	_
ENSG00000224533	3.724485	-2.294189	1	1	TMLHE-AS1	_
ENSG00000224563	3.724485	-2.294189	1	1	LPP-AS1	_
ENSG00000224629	3.724485	-2.294189	1	1	RP5-1142J19.2	K02929
ENSG00000224682	3.724485	-2.294189	1	1	SOCS5P2	K04698
ENSG00000224707	3.724485	-2.294189	1	1	E2F3-IT1	_
ENSG00000224794	3.724485	-2.294189	1	1	RP3-333H23.8	_
ENSG00000224800	3.724485	-2.294189	1	1	RP11-235D19.2	K02977
ENSG00000224839	3.724485	-2.294189	1	1	AC093698.2	K02870
ENSG00000224899	3.724485	-2.294189	1	1	RP11-3B12.5	_
ENSG00000224910	3.724485	-2.294189	1	1	AC002115.5	_
ENSG00000224928	3.724485	-2.294189	1	1	KRT8P30	K07605
ENSG00000225002	3.724485	-2.294189	1	1	RP11-6F6.1	_
ENSG00000225030	3.724485	-2.294189	1	1	RP4-784A16.3	_
ENSG00000225053	3.724485	-2.294189	1	1	PRPF38AP1	K12849
ENSG00000225078	3.724485	-2.294189	1	1	RP11-123N4.4	_
ENSG00000225092	3.724485	-2.294189	1	1	RP11-405O10.2	_
ENSG00000225111	3.724485	-2.294189	1	1	AC083900.1	_

ENSG00000225123	3.724485	-2.294189	1	1	RP11-255A11.4	_
ENSG00000225146	3.724485	-2.294189	1	1	AC073957.15	_
ENSG00000225169	3.724485	-2.294189	1	1	BRI3P1	K09699
ENSG00000225263	3.724485	-2.294189	1	1	PCDH9-AS3	_
ENSG00000225315	3.724485	-2.294189	1	1	RP11-293P20.2	_
ENSG00000225331	3.724485	-2.294189	1	1	AP001055.6	_
ENSG00000225442	3.724485	-2.294189	1	1	MPRIP-AS1	_
ENSG00000225561	3.724485	-2.294189	1	1	RP11-412H9.2	_
ENSG00000225632	3.724485	-2.294189	1	1	RP5-997D24.3	_
ENSG00000225639	3.724485	-2.294189	1	1	PGAM1P2	K01834
ENSG00000225715	3.724485	-2.294189	1	1	RP11-325E14.5	K02725
ENSG00000225731	3.724485	-2.294189	1	1	AP001627.1	_
ENSG00000225756	3.724485	-2.294189	1	1	DBH-AS1	_
ENSG00000225768	3.724485	-2.294189	1	1	RP11-127O4.3	_
ENSG00000225831	3.724485	-2.294189	1	1	RPS18P1	_
ENSG00000225912	3.724485	-2.294189	1	1	RP13-258O15.1	K02898
ENSG00000225951	3.724485	-2.294189	1	1	ODF2-AS1	_
ENSG00000225969	3.724485	-2.294189	1	1	LINC00035	_
ENSG00000225972	3.724485	-2.294189	1	1	MTND1P23	K03878
ENSG00000226017	3.724485	-2.294189	1	1	PRICKLE2-AS3	_
ENSG00000226040	3.724485	-2.294189	1	1	AC005740.3	K02437
ENSG00000226074	3.724485	-2.294189	1	1	PRSS44	_
ENSG00000226220	3.724485	-2.294189	1	1	CICP22	K16941
ENSG00000226241	3.724485	-2.294189	1	1	LL0XNC01-221F2.2	_
ENSG00000226251	3.724485	-2.294189	1	1	RP11-15I11.3	_
ENSG00000226359	3.724485	-2.294189	1	1	AC055811.5	K05692
ENSG00000226364	3.724485	-2.294189	1	1	AC102948.2	_
ENSG00000226423	3.724485	-2.294189	1	1	AC093642.4	_
ENSG00000226469	3.724485	-2.294189	1	1	ADAM1B	K09537
ENSG00000226493	3.724485	-2.294189	1	1	RP11-524O24.2	K02898
ENSG00000226497	3.724485	-2.294189	1	1	RP11-406O16.1	_
ENSG00000226604	3.724485	-2.294189	1	1	PAPPA-AS2	_
ENSG00000226789	3.724485	-2.294189	1	1	AC110926.4	K12866
ENSG00000226808	3.724485	-2.294189	1	1	LINC00840	_
ENSG00000226812	3.724485	-2.294189	1	1	RP5-881L22.5	_
ENSG00000226872	3.724485	-2.294189	1	1	AC002472.11	_
ENSG00000226877	3.724485	-2.294189	1	1	RP11-575L7.2	_
ENSG00000226913	3.724485	-2.294189	1	1	BSN-AS2	_
ENSG00000227038	3.724485	-2.294189	1	1	AC005077.12	K15696
ENSG00000227076	3.724485	-2.294189	1	1	RP11-4C20.4	_
ENSG00000227116	3.724485	-2.294189	1	1	RP3-471C18.1	_
ENSG00000227141	3.724485	-2.294189	1	1	RP11-545A16.3	K12491
ENSG00000227172	3.724485	-2.294189	1	1	AC011290.4	_
ENSG00000227290	3.724485	-2.294189	1	1	RP4-544H6.2	_
ENSG00000227413	3.724485	-2.294189	1	1	RP5-1042K10.12	_
ENSG00000227431	3.724485	-2.294189	1	1	RP1-155G6.4	_
ENSG00000227463	3.724485	-2.294189	1	1	RP11-158D2.2	_
ENSG00000227581	3.724485	-2.294189	1	1	RP13-140E4.1	K17597
ENSG00000227582	3.724485	-2.294189	1	1	RP11-262H14.7	K08458
ENSG00000227692	3.724485	-2.294189	1	1	MED28P3	K15141
ENSG00000227719	3.724485	-2.294189	1	1	AC006042.6	_
ENSG00000227725	3.724485	-2.294189	1	1	GCOM2	_
ENSG00000227838	3.724485	-2.294189	1	1	RP1-213J1P__B.1	_
ENSG00000227857	3.724485	-2.294189	1	1	RP4-533D7.5	_
ENSG00000227948	3.724485	-2.294189	1	1	AC003989.4	K01940
ENSG00000228008	3.724485	-2.294189	1	1	CTD-2330K9.3	_
ENSG00000228032	3.724485	-2.294189	1	1	C14orf144	_
ENSG00000228040	3.724485	-2.294189	1	1	RP3-522D1.2	K02924

ENSG00000228072	3.724485	-2.294189	1	1	RP11-255A11.2	_
ENSG00000228259	3.724485	-2.294189	1	1	AC136604.1	_
ENSG00000228278	3.724485	-2.294189	1	1	ORM2	K17308
ENSG00000228470	3.724485	-2.294189	1	1	RP11-176D17.3	_
ENSG00000228477	3.724485	-2.294189	1	1	RP3-342P20.2	K02981
ENSG00000228507	3.724485	-2.294189	1	1	DAP3P2	K17408
ENSG00000228525	3.724485	-2.294189	1	1	RP5-1087E8.2	_
ENSG00000228612	3.724485	-2.294189	1	1	HK2P1	K00844
ENSG00000228620	3.724485	-2.294189	1	1	RP3-434P1.6	_
ENSG00000228677	3.724485	-2.294189	1	1	TTC3-AS1	_
ENSG00000228679	3.724485	-2.294189	1	1	RP4-676J13.2	_
ENSG00000228702	3.724485	-2.294189	1	1	RP11-671E7.1	K18708
ENSG00000228718	3.724485	-2.294189	1	1	RP11-145H9.3	_
ENSG00000228810	3.724485	-2.294189	1	1	PABPC1P11	K12581
ENSG00000228829	3.724485	-2.294189	1	1	AC005077.5	K03121
ENSG00000228834	3.724485	-2.294189	1	1	RP11-249L21.4	K02130
ENSG00000228887	3.724485	-2.294189	1	1	EEF1DP1	K15410
ENSG00000228919	3.724485	-2.294189	1	1	AC097381.1	_
ENSG00000228949	3.724485	-2.294189	1	1	UGT1A12P	K00699
ENSG00000228976	3.724485	-2.294189	1	1	SUMO2P8	K12160
ENSG00000228983	3.724485	-2.294189	1	1	AC025627.7	K03327
ENSG00000229162	3.724485	-2.294189	1	1	RP11-84D1.1	_
ENSG00000229168	3.724485	-2.294189	1	1	RPL19P20	K02885
ENSG00000229191	3.724485	-2.294189	1	1	RP11-168O16.1	_
ENSG00000229282	3.724485	-2.294189	1	1	RP1-40E16.2	_
ENSG00000229344	3.724485	-2.294189	1	1	RP5-857K21.7	K02261
ENSG00000229497	3.724485	-2.294189	1	1	AC005189.6	_
ENSG00000229519	3.724485	-2.294189	1	1	RP11-58A11.2	K02915
ENSG00000229523	3.724485	-2.294189	1	1	RP11-7G23.4	_
ENSG00000229605	3.724485	-2.294189	1	1	RP11-492M23.2	K02889
ENSG00000229628	3.724485	-2.294189	1	1	AC073115.7	_
ENSG00000229648	3.724485	-2.294189	1	1	AC016753.7	K02998
ENSG00000229659	3.724485	-2.294189	1	1	RP11-345K20.2	K02898
ENSG00000229660	3.724485	-2.294189	1	1	RP5-1142J19.1	_
ENSG00000229676	3.724485	-2.294189	1	1	ZNF492	K09228
ENSG00000229821	3.724485	-2.294189	1	1	RP11-567E21.3	_
ENSG00000229915	3.724485	-2.294189	1	1	AC016999.2	_
ENSG00000229925	3.724485	-2.294189	1	1	AP001043.1	_
ENSG00000229990	3.724485	-2.294189	1	1	RP11-574K11.8	_
ENSG00000230005	3.724485	-2.294189	1	1	SNAP47-AS1	_
ENSG00000230020	3.724485	-2.294189	1	1	NHS-AS1	_
ENSG00000230047	3.724485	-2.294189	1	1	AC017104.4	_
ENSG00000230054	3.724485	-2.294189	1	1	RP11-402G3.5	_
ENSG00000230321	3.724485	-2.294189	1	1	CTD-3105H18.9	_
ENSG00000230394	3.724485	-2.294189	1	1	RP11-692P14.1	_
ENSG00000230395	3.724485	-2.294189	1	1	AC092651.1	K03348
ENSG00000230444	3.724485	-2.294189	1	1	TFAMP1	K11830
ENSG00000230459	3.724485	-2.294189	1	1	RP11-741G21.1	K11300
ENSG00000230468	3.724485	-2.294189	1	1	CLUHP5	_
ENSG00000230495	3.724485	-2.294189	1	1	RP11-462D18.2	K07863
ENSG00000230500	3.724485	-2.294189	1	1	RP11-360I20.2	_
ENSG00000230516	3.724485	-2.294189	1	1	RP11-555J4.3	_
ENSG00000230550	3.724485	-2.294189	1	1	RP11-74C13.3	_
ENSG00000230587	3.724485	-2.294189	1	1	AC093609.1	_
ENSG00000230684	3.724485	-2.294189	1	1	RP11-409K20.6	_
ENSG00000230694	3.724485	-2.294189	1	1	RP11-62F24.1	_
ENSG00000230747	3.724485	-2.294189	1	1	AC021188.4	_
ENSG00000230758	3.724485	-2.294189	1	1	SNAP23P	K08508

ENSG00000230832	3.724485	-2.294189	1	1	RP11-325P15.2	K11090
ENSG00000230872	3.724485	-2.294189	1	1	AC092338.5	_
ENSG00000230914	3.724485	-2.294189	1	1	AC004840.8	_
ENSG00000230927	3.724485	-2.294189	1	1	TMBIM7P	_
ENSG00000231027	3.724485	-2.294189	1	1	AC079325.6	K06719
ENSG00000231104	3.724485	-2.294189	1	1	RP11-354M20.3	_
ENSG00000231170	3.724485	-2.294189	1	1	AC002451.3	_
ENSG00000231187	3.724485	-2.294189	1	1	RP11-38L15.3	_
ENSG00000231193	3.724485	-2.294189	1	1	RP11-462B18.2	_
ENSG00000231335	3.724485	-2.294189	1	1	AC107072.2	_
ENSG00000231403	3.724485	-2.294189	1	1	AC099344.3	_
ENSG00000231518	3.724485	-2.294189	1	1	RP11-327L3.3	_
ENSG00000231544	3.724485	-2.294189	1	1	RSL24D1P11	K02896
ENSG00000231564	3.724485	-2.294189	1	1	EIF4A1P11	K03257
ENSG00000231686	3.724485	-2.294189	1	1	RP1-203P18.1	_
ENSG00000231726	3.724485	-2.294189	1	1	HMGN2P38	K11300
ENSG00000231804	3.724485	-2.294189	1	1	RP11-317B17.3	K02889
ENSG00000231868	3.724485	-2.294189	1	1	RP1-202O8.2	_
ENSG00000231992	3.724485	-2.294189	1	1	RP11-57H12.2	_
ENSG00000232002	3.724485	-2.294189	1	1	AC093642.6	_
ENSG00000232046	3.724485	-2.294189	1	1	AC007392.3	_
ENSG00000232129	3.724485	-2.294189	1	1	AC011385.2	K12581
ENSG00000232131	3.724485	-2.294189	1	1	NCOA7-AS1	_
ENSG00000232133	3.724485	-2.294189	1	1	IMPDH1P10	K00088
ENSG00000232166	3.724485	-2.294189	1	1	RP4-799P18.5	K02940
ENSG00000232224	3.724485	-2.294189	1	1	LINC00202-1	_
ENSG00000232228	3.724485	-2.294189	1	1	RP11-77O7.1	K02929
ENSG00000232229	3.724485	-2.294189	1	1	LINC00865	_
ENSG00000232316	3.724485	-2.294189	1	1	RP1-124C6.1	_
ENSG00000232362	3.724485	-2.294189	1	1	ATP5LP2	K02140
ENSG00000232396	3.724485	-2.294189	1	1	RP1-76B20.11	_
ENSG00000232448	3.724485	-2.294189	1	1	RP11-416N4.1	_
ENSG00000232479	3.724485	-2.294189	1	1	AC010900.2	K07605
ENSG00000232487	3.724485	-2.294189	1	1	RASA3-IT1	_
ENSG00000232526	3.724485	-2.294189	1	1	AC025750.6	K05862
ENSG00000232568	3.724485	-2.294189	1	1	RPL23AP35	K02893
ENSG00000232586	3.724485	-2.294189	1	1	RP11-46A10.4	_
ENSG00000232623	3.724485	-2.294189	1	1	AP000266.7	_
ENSG00000232646	3.724485	-2.294189	1	1	RP11-344N10.4	_
ENSG00000232699	3.724485	-2.294189	1	1	BDH2P1	K00019
ENSG00000232777	3.724485	-2.294189	1	1	MRPL51P2	K17432
ENSG00000232926	3.724485	-2.294189	1	1	AC000078.5	_
ENSG00000232949	3.724485	-2.294189	1	1	AC002480.4	_
ENSG00000233125	3.724485	-2.294189	1	1	ACTBP12	K05692
ENSG00000233332	3.724485	-2.294189	1	1	RP4-799P18.2	_
ENSG00000233343	3.724485	-2.294189	1	1	ATP6V1G1P4	_
ENSG00000233369	3.724485	-2.294189	1	1	RP11-396K3.1	K03121
ENSG00000233392	3.724485	-2.294189	1	1	AC104809.4	_
ENSG00000233431	3.724485	-2.294189	1	1	RP11-63N8.3	_
ENSG00000233518	3.724485	-2.294189	1	1	RPL7P35	K02937
ENSG00000233521	3.724485	-2.294189	1	1	RP5-1172A22.1	_
ENSG00000233593	3.724485	-2.294189	1	1	RP4-665J23.1	_
ENSG00000233635	3.724485	-2.294189	1	1	AC037445.1	_
ENSG00000233684	3.724485	-2.294189	1	1	AC079779.6	_
ENSG00000233690	3.724485	-2.294189	1	1	EBAG9P1	_
ENSG00000233694	3.724485	-2.294189	1	1	AC007365.1	_
ENSG00000233725	3.724485	-2.294189	1	1	LINC00284	_
ENSG00000233821	3.724485	-2.294189	1	1	ENOX1-AS1	_

ENSG00000233827	3.724485	-2.294189	1	1	FAM58BP	_
ENSG00000233877	3.724485	-2.294189	1	1	RP11-182I10.2	_
ENSG00000233963	3.724485	-2.294189	1	1	ATP8A2P3	K14802
ENSG00000233978	3.724485	-2.294189	1	1	AC016735.3	_
ENSG00000234017	3.724485	-2.294189	1	1	RP11-214N15.5	K03231
ENSG00000234136	3.724485	-2.294189	1	1	AC055764.1	_
ENSG00000234148	3.724485	-2.294189	1	1	RP11-395L14.3	_
ENSG00000234324	3.724485	-2.294189	1	1	RPL9P2	K02940
ENSG00000234371	3.724485	-2.294189	1	1	RP11-315J22.5	K02998
ENSG00000234393	3.724485	-2.294189	1	1	RP11-211N11.5	_
ENSG00000234423	3.724485	-2.294189	1	1	AC019118.2	_
ENSG00000234471	3.724485	-2.294189	1	1	AC147651.1	_
ENSG00000234562	3.724485	-2.294189	1	1	TPMTP2	K00569
ENSG00000234698	3.724485	-2.294189	1	1	RP11-112L6.2	_
ENSG00000234709	3.724485	-2.294189	1	1	UPF3AP3	K14328
ENSG00000234718	3.724485	-2.294189	1	1	AC007161.5	_
ENSG00000234826	3.724485	-2.294189	1	1	AC003084.2	_
ENSG00000234919	3.724485	-2.294189	1	1	AC064834.3	_
ENSG00000234925	3.724485	-2.294189	1	1	RP11-254B13.3	K02138
ENSG00000234973	3.724485	-2.294189	1	1	RP11-272J7.4	_
ENSG00000234986	3.724485	-2.294189	1	1	XX-C2158C12.1	_
ENSG00000235084	3.724485	-2.294189	1	1	CHCHD2P6	K00901
ENSG00000235284	3.724485	-2.294189	1	1	SNORD62A	_
ENSG00000235330	3.724485	-2.294189	1	1	RP11-165F24.2	K02870
ENSG00000235422	3.724485	-2.294189	1	1	KATNBL1P3	_
ENSG00000235565	3.724485	-2.294189	1	1	RP11-86H7.7	_
ENSG00000235643	3.724485	-2.294189	1	1	RP1-69M21.2	_
ENSG00000235664	3.724485	-2.294189	1	1	AC005682.8	_
ENSG00000235704	3.724485	-2.294189	1	1	AC023490.2	_
ENSG00000235746	3.724485	-2.294189	1	1	CTBP2P2	K04496
ENSG00000235760	3.724485	-2.294189	1	1	AC138655.4	_
ENSG00000235957	3.724485	-2.294189	1	1	COX7CP1	K02272
ENSG00000235990	3.724485	-2.294189	1	1	RP11-323D18.5	K02893
ENSG00000236049	3.724485	-2.294189	1	1	AC104777.2	_
ENSG00000236055	3.724485	-2.294189	1	1	RP11-423O2.2	_
ENSG00000236120	3.724485	-2.294189	1	1	RP11-733O18.1	_
ENSG00000236155	3.724485	-2.294189	1	1	RP11-231P20.2	_
ENSG00000236226	3.724485	-2.294189	1	1	RP11-645N11.3	_
ENSG00000236318	3.724485	-2.294189	1	1	AC019117.1	_
ENSG00000236430	3.724485	-2.294189	1	1	KRT8P29	K07605
ENSG00000236463	3.724485	-2.294189	1	1	LINC00427	_
ENSG00000236480	3.724485	-2.294189	1	1	PKMP1	K00873
ENSG00000236483	3.724485	-2.294189	1	1	CTD-3105H18.8	_
ENSG00000236512	3.724485	-2.294189	1	1	RP3-336K20__B.2	K02905
ENSG00000236534	3.724485	-2.294189	1	1	H3F3BP1	K11253
ENSG00000236546	3.724485	-2.294189	1	1	RP1-118J21.5	_
ENSG00000236549	3.724485	-2.294189	1	1	AC079807.3	K02977
ENSG00000236567	3.724485	-2.294189	1	1	RP11-39K24.7	K09063
ENSG00000236583	3.724485	-2.294189	1	1	AP004290.1	K17392
ENSG00000236616	3.724485	-2.294189	1	1	BAK1P2	K14021
ENSG00000236678	3.724485	-2.294189	1	1	LINC00347	_
ENSG00000236756	3.724485	-2.294189	1	1	DNAJC9-AS1	_
ENSG00000236942	3.724485	-2.294189	1	1	RP11-6B6.3	K08341
ENSG00000236977	3.724485	-2.294189	1	1	ANKRD44-IT1	_
ENSG00000237088	3.724485	-2.294189	1	1	RP11-73E6.2	K02936
ENSG00000237136	3.724485	-2.294189	1	1	C4orf51	_
ENSG00000237139	3.724485	-2.294189	1	1	RP11-137L10.5	K02976
ENSG00000237271	3.724485	-2.294189	1	1	AC009498.1	_

ENSG00000237328	3.724485	-2.294189	1	1	RAI1-AS1	_
ENSG00000237377	3.724485	-2.294189	1	1	AC005703.2	_
ENSG00000237416	3.724485	-2.294189	1	1	RP11-465K1.2	_
ENSG00000237483	3.724485	-2.294189	1	1	RP11-423E7.1	K02977
ENSG00000237512	3.724485	-2.294189	1	1	UNC5B-AS1	_
ENSG00000237588	3.724485	-2.294189	1	1	RP11-66D17.3	_
ENSG00000237663	3.724485	-2.294189	1	1	DNAJC19P7	K09539
ENSG00000237669	3.724485	-2.294189	1	1	HCG4P3	K06751
ENSG00000237691	3.724485	-2.294189	1	1	IFNWP2	K05440
ENSG00000237887	3.724485	-2.294189	1	1	RPL23AP32	K02893
ENSG00000237934	3.724485	-2.294189	1	1	RP11-467D18.2	_
ENSG00000237972	3.724485	-2.294189	1	1	TUBG1P	K10389
ENSG00000238000	3.724485	-2.294189	1	1	RP11-274E7.2	K06697
ENSG00000238140	3.724485	-2.294189	1	1	RP11-253A20.1	_
ENSG00000238164	3.724485	-2.294189	1	1	RP3-395M20.8	_
ENSG00000238222	3.724485	-2.294189	1	1	MKRN4P	K15687
ENSG00000238231	3.724485	-2.294189	1	1	RP11-460I13.6	K06566
ENSG00000238272	3.724485	-2.294189	1	1	RP3-436N22.3	_
ENSG00000238280	3.724485	-2.294189	1	1	RP11-436D10.3	_
ENSG00000238311	3.724485	-2.294189	1	1	snoU13	_
ENSG00000238322	3.724485	-2.294189	1	1	snoU13	_
ENSG00000238403	3.724485	-2.294189	1	1	snoU13	_
ENSG00000238475	3.724485	-2.294189	1	1	snoU13	_
ENSG00000238533	3.724485	-2.294189	1	1	snoU13	_
ENSG00000238534	3.724485	-2.294189	1	1	Y_RNA	_
ENSG00000238711	3.724485	-2.294189	1	1	RNY4P25	_
ENSG00000238754	3.724485	-2.294189	1	1	snoU109	_
ENSG00000238791	3.724485	-2.294189	1	1	snoU13	_
ENSG00000238831	3.724485	-2.294189	1	1	RNU7-189P	_
ENSG00000238834	3.724485	-2.294189	1	1	snoU13	_
ENSG00000238857	3.724485	-2.294189	1	1	snoU13	_
ENSG00000238920	3.724485	-2.294189	1	1	snoU13	_
ENSG00000238934	3.724485	-2.294189	1	1	ACA64	_
ENSG00000238948	3.724485	-2.294189	1	1	snoU13	_
ENSG00000238995	3.724485	-2.294189	1	1	snoU13	_
ENSG00000239079	3.724485	-2.294189	1	1	snoU13	_
ENSG00000239125	3.724485	-2.294189	1	1	snoU13	_
ENSG00000239183	3.724485	-2.294189	1	1	SNORA84	_
ENSG00000239198	3.724485	-2.294189	1	1	RPL5P22	K02932
ENSG00000239254	3.724485	-2.294189	1	1	RP11-365F18.3	K18403
ENSG00000239351	3.724485	-2.294189	1	1	NPM1P29	K11276
ENSG00000239377	3.724485	-2.294189	1	1	RP4-584D14.6	_
ENSG00000239602	3.724485	-2.294189	1	1	RP11-449H3.1	K02917
ENSG00000239614	3.724485	-2.294189	1	1	HMGN1P7	K11299
ENSG00000239774	3.724485	-2.294189	1	1	RP11-496B10.3	_
ENSG00000239821	3.724485	-2.294189	1	1	RN7SL513P	_
ENSG00000239883	3.724485	-2.294189	1	1	RP11-175I17.2	K07759
ENSG00000239899	3.724485	-2.294189	1	1	RN7SL674P	_
ENSG00000239919	3.724485	-2.294189	1	1	SNRPGP3	K11099
ENSG00000240021	3.724485	-2.294189	1	1	TEX35	_
ENSG00000240093	3.724485	-2.294189	1	1	AC093627.12	_
ENSG00000240219	3.724485	-2.294189	1	1	RP11-430C7.5	_
ENSG00000240305	3.724485	-2.294189	1	1	RP11-1072C15.1	K02934
ENSG00000240322	3.724485	-2.294189	1	1	RN7SL481P	_
ENSG00000240338	3.724485	-2.294189	1	1	RP11-331F4.4	K01310
ENSG00000240385	3.724485	-2.294189	1	1	RPS29P20	_
ENSG00000240401	3.724485	-2.294189	1	1	AC012358.8	_
ENSG00000240687	3.724485	-2.294189	1	1	RP11-521D12.1	_

ENSG00000240694	3.724485	-2.294189	1	1	PNMA2	_
ENSG00000240710	3.724485	-2.294189	1	1	RP11-430C7.4	_
ENSG00000240720	3.724485	-2.294189	1	1	LRRD1	_
ENSG00000240723	3.724485	-2.294189	1	1	RN7SL382P	_
ENSG00000240882	3.724485	-2.294189	1	1	RP11-174O3.1	K15168
ENSG00000240991	3.724485	-2.294189	1	1	RPL23AP67	K02893
ENSG00000241007	3.724485	-2.294189	1	1	SEPT7P6	K16944
ENSG00000241095	3.724485	-2.294189	1	1	CYP51A1P1	K05917
ENSG00000241362	3.724485	-2.294189	1	1	RPL36AP43	K02929
ENSG00000241369	3.724485	-2.294189	1	1	CT64	_
ENSG00000241413	3.724485	-2.294189	1	1	RN7SL441P	_
ENSG00000241416	3.724485	-2.294189	1	1	KRT8P13	K07605
ENSG00000241535	3.724485	-2.294189	1	1	CBX5P1	K11587
ENSG00000241568	3.724485	-2.294189	1	1	RN7SL338P	_
ENSG00000241593	3.724485	-2.294189	1	1	RP11-520D19.2	_
ENSG00000241631	3.724485	-2.294189	1	1	RN7SL316P	_
ENSG00000241735	3.724485	-2.294189	1	1	FABP5P3	K08754
ENSG00000241738	3.724485	-2.294189	1	1	ZNF90P1	K09228
ENSG00000241772	3.724485	-2.294189	1	1	AC092620.2	_
ENSG00000241811	3.724485	-2.294189	1	1	RN7SL651P	_
ENSG00000241879	3.724485	-2.294189	1	1	KLF3P2	_
ENSG00000242058	3.724485	-2.294189	1	1	RPS4XP19	K02987
ENSG00000242173	3.724485	-2.294189	1	1	ARHGDIG	K12462
ENSG00000242398	3.724485	-2.294189	1	1	RN7SL800P	_
ENSG00000242477	3.724485	-2.294189	1	1	CTD-2161E19.1	K02865
ENSG00000242551	3.724485	-2.294189	1	1	POU5F1P6	K09367
ENSG00000242748	3.724485	-2.294189	1	1	RPL23AP81	K02893
ENSG00000242882	3.724485	-2.294189	1	1	RPL5P11	K02932
ENSG00000242888	3.724485	-2.294189	1	1	RP11-436M15.1	K02981
ENSG00000242941	3.724485	-2.294189	1	1	CTD-2325P2.2	K02870
ENSG00000243018	3.724485	-2.294189	1	1	RP5-1070G24.2	_
ENSG00000243088	3.724485	-2.294189	1	1	RN7SL539P	_
ENSG00000243089	3.724485	-2.294189	1	1	RP11-779P15.2	_
ENSG00000243130	3.724485	-2.294189	1	1	PSG11	K06500
ENSG00000243307	3.724485	-2.294189	1	1	RP11-239L20.3	_
ENSG00000243385	3.724485	-2.294189	1	1	CTD-2110K23.1	_
ENSG00000243388	3.724485	-2.294189	1	1	RPL3P3	K02925
ENSG00000243402	3.724485	-2.294189	1	1	RP11-473O4.1	K02877
ENSG00000243433	3.724485	-2.294189	1	1	RP11-148K1.10	_
ENSG00000243533	3.724485	-2.294189	1	1	TBCE-AS1	_
ENSG00000243871	3.724485	-2.294189	1	1	RN7SL487P	_
ENSG00000243910	3.724485	-2.294189	1	1	TUBA4B	K07374
ENSG00000243959	3.724485	-2.294189	1	1	RN7SL684P	_
ENSG00000244045	3.724485	-2.294189	1	1	TMEM199	_
ENSG00000244061	3.724485	-2.294189	1	1	RP11-389C8.1	K02865
ENSG00000244063	3.724485	-2.294189	1	1	AC024704.2	_
ENSG00000244131	3.724485	-2.294189	1	1	RPSAP51	K02998
ENSG00000244134	3.724485	-2.294189	1	1	RPS12P20	K02951
ENSG00000244236	3.724485	-2.294189	1	1	RN7SL604P	_
ENSG00000244280	3.724485	-2.294189	1	1	ECEL1P2	K09610
ENSG00000244378	3.724485	-2.294189	1	1	RPS2P45	K02981
ENSG00000244677	3.724485	-2.294189	1	1	RN7SL706P	_
ENSG00000244691	3.724485	-2.294189	1	1	RP11-600F24.2	K02865
ENSG00000244701	3.724485	-2.294189	1	1	RP5-894A10.2	_
ENSG00000244921	3.724485	-2.294189	1	1	CTB-36O1.7	K00412
ENSG00000245205	3.724485	-2.294189	1	1	EEF1A1P4	K03231
ENSG00000245311	3.724485	-2.294189	1	1	RP11-165P7.1	_
ENSG00000245954	3.724485	-2.294189	1	1	RP11-18H21.1	_

ENSG00000246863	3.724485	-2.294189	1	1	RP11-325N19.3	_
ENSG00000247311	3.724485	-2.294189	1	1	CTC-441N14.2	_
ENSG00000247324	3.724485	-2.294189	1	1	RP11-510M2.2	_
ENSG00000247925	3.724485	-2.294189	1	1	RP3-510L9.1	_
ENSG00000248394	3.724485	-2.294189	1	1	FOSL1P1	K04502
ENSG00000248416	3.724485	-2.294189	1	1	RP11-1191J2.4	_
ENSG00000248521	3.724485	-2.294189	1	1	RP11-174F8.1	K10568
ENSG00000248568	3.724485	-2.294189	1	1	KRT8P48	K07605
ENSG00000248714	3.724485	-2.294189	1	1	RP11-1079K10.3	_
ENSG00000248794	3.724485	-2.294189	1	1	CTD-2248H3.1	K02951
ENSG00000248795	3.724485	-2.294189	1	1	RP11-173E2.1	K11883
ENSG00000248863	3.724485	-2.294189	1	1	RP11-83A24.1	_
ENSG00000248909	3.724485	-2.294189	1	1	HMGB1P21	K10802
ENSG00000248916	3.724485	-2.294189	1	1	NUP210P3	K14314
ENSG00000249053	3.724485	-2.294189	1	1	RP11-419C19.1	_
ENSG00000249096	3.724485	-2.294189	1	1	RP11-290F5.1	_
ENSG00000249119	3.724485	-2.294189	1	1	MTND6P4	K03884
ENSG00000249176	3.724485	-2.294189	1	1	RP11-304F15.5	K14815
ENSG00000249242	3.724485	-2.294189	1	1	TMEM150C	_
ENSG00000249428	3.724485	-2.294189	1	1	RP11-503N18.3	_
ENSG00000249437	3.724485	-2.294189	1	1	NAIP	K12807
ENSG00000249441	3.724485	-2.294189	1	1	RP11-94H6.1	_
ENSG00000249610	3.724485	-2.294189	1	1	CTC-441N14.1	_
ENSG00000249680	3.724485	-2.294189	1	1	TUBA3GP	K07374
ENSG00000249784	3.724485	-2.294189	1	1	SCARNA22	_
ENSG00000249889	3.724485	-2.294189	1	1	ALG1L11P	K03842
ENSG00000249923	3.724485	-2.294189	1	1	XXbac-B444P24.8	_
ENSG00000250140	3.724485	-2.294189	1	1	CTD-2517O10.5	K02898
ENSG00000250220	3.724485	-2.294189	1	1	RP11-692D12.1	_
ENSG00000250261	3.724485	-2.294189	1	1	AC007050.18	_
ENSG00000250280	3.724485	-2.294189	1	1	RP11-305O6.3	_
ENSG00000250282	3.724485	-2.294189	1	1	RP5-875H18.4	_
ENSG00000250332	3.724485	-2.294189	1	1	CTD-2272G21.2	K07293
ENSG00000250508	3.724485	-2.294189	1	1	RP11-757G1.6	_
ENSG00000250600	3.724485	-2.294189	1	1	ROPN1L-AS1	_
ENSG00000250677	3.724485	-2.294189	1	1	RP11-576N17.2	_
ENSG00000250749	3.724485	-2.294189	1	1	RP11-451H23.1	_
ENSG00000250756	3.724485	-2.294189	1	1	RP11-225B17.1	K08376
ENSG00000250882	3.724485	-2.294189	1	1	CTC-459M5.1	_
ENSG00000250986	3.724485	-2.294189	1	1	AC141928.1	_
ENSG00000251148	3.724485	-2.294189	1	1	RP11-478C1.7	_
ENSG00000251171	3.724485	-2.294189	1	1	RP11-327O17.2	_
ENSG00000251187	3.724485	-2.294189	1	1	CTC-459M5.2	_
ENSG00000251314	3.724485	-2.294189	1	1	CTD-2337A12.1	_
ENSG00000251350	3.724485	-2.294189	1	1	RP11-328N19.1	_
ENSG00000251455	3.724485	-2.294189	1	1	RP11-164P12.3	_
ENSG00000251461	3.724485	-2.294189	1	1	RP11-501C14.6	_
ENSG00000251484	3.724485	-2.294189	1	1	RP5-1065J22.4	K12889
ENSG00000251574	3.724485	-2.294189	1	1	RP11-6N13.1	_
ENSG00000251590	3.724485	-2.294189	1	1	NIFKP7	K14838
ENSG00000251596	3.724485	-2.294189	1	1	HADHAP1	K07515
ENSG00000251685	3.724485	-2.294189	1	1	RP11-813N20.1	K00699
ENSG00000251692	3.724485	-2.294189	1	1	PTX4	_
ENSG00000251695	3.724485	-2.294189	1	1	AC124890.1	_
ENSG00000251738	3.724485	-2.294189	1	1	AC073215.1	_
ENSG00000251791	3.724485	-2.294189	1	1	SCARNA6	_
ENSG00000251869	3.724485	-2.294189	1	1	SCARNA23	_
ENSG00000251990	3.724485	-2.294189	1	1	RNA5SP180	_

ENSG00000251992	3.724485	-2.294189	1	1	SCARNA17	_
ENSG00000252010	3.724485	-2.294189	1	1	SCARNA5	_
ENSG00000252021	3.724485	-2.294189	1	1	Y_RNA	_
ENSG00000252070	3.724485	-2.294189	1	1	RNA5SP341	_
ENSG00000252118	3.724485	-2.294189	1	1	RNU6ATAC39P	_
ENSG00000252200	3.724485	-2.294189	1	1	snoZ6	_
ENSG00000252234	3.724485	-2.294189	1	1	AC006557.1	_
ENSG00000252355	3.724485	-2.294189	1	1	RN7SKP287	_
ENSG00000252469	3.724485	-2.294189	1	1	RNU7-160P	_
ENSG00000252543	3.724485	-2.294189	1	1	U3	_
ENSG00000252549	3.724485	-2.294189	1	1	RNU6-759P	_
ENSG00000252552	3.724485	-2.294189	1	1	RNU6-307P	_
ENSG00000252590	3.724485	-2.294189	1	1	RNU7-143P	_
ENSG00000252636	3.724485	-2.294189	1	1	RNU6-826P	_
ENSG00000252637	3.724485	-2.294189	1	1	RNA5SP268	_
ENSG00000252707	3.724485	-2.294189	1	1	RNU11-2P	_
ENSG00000252839	3.724485	-2.294189	1	1	RNU6-419P	_
ENSG00000252905	3.724485	-2.294189	1	1	RNA5SP330	_
ENSG00000252908	3.724485	-2.294189	1	1	RNU6-1003P	_
ENSG00000252914	3.724485	-2.294189	1	1	RNU6-789P	_
ENSG00000252949	3.724485	-2.294189	1	1	AL136303.1	_
ENSG00000252971	3.724485	-2.294189	1	1	RNU6-1057P	_
ENSG00000252987	3.724485	-2.294189	1	1	RNA5SP66	_
ENSG00000253140	3.724485	-2.294189	1	1	RP11-567J20.3	_
ENSG00000253153	3.724485	-2.294189	1	1	KB-1562D12.3	K02896
ENSG00000253180	3.724485	-2.294189	1	1	RP11-410L14.1	K10704
ENSG00000253228	3.724485	-2.294189	1	1	NRBF2P4	_
ENSG00000253295	3.724485	-2.294189	1	1	RP11-779O18.1	_
ENSG00000253356	3.724485	-2.294189	1	1	RP11-90P5.2	_
ENSG00000253397	3.724485	-2.294189	1	1	RP11-597M17.1	_
ENSG00000253456	3.724485	-2.294189	1	1	CTD-2363C16.2	_
ENSG00000253539	3.724485	-2.294189	1	1	RP11-402L5.1	_
ENSG00000253582	3.724485	-2.294189	1	1	RP11-649G15.2	_
ENSG00000253708	3.724485	-2.294189	1	1	RP11-51J9.4	_
ENSG00000253739	3.724485	-2.294189	1	1	RP11-90P5.7	_
ENSG00000253768	3.724485	-2.294189	1	1	CTB-33O18.1	_
ENSG00000253802	3.724485	-2.294189	1	1	CTA-392C11.1	_
ENSG00000253844	3.724485	-2.294189	1	1	RP11-546K22.1	_
ENSG00000253911	3.724485	-2.294189	1	1	KB-1205A7.2	_
ENSG00000253983	3.724485	-2.294189	1	1	RP1-16A9.1	_
ENSG00000254038	3.724485	-2.294189	1	1	RP11-419C23.1	_
ENSG00000254244	3.724485	-2.294189	1	1	PAICSP4	K01587
ENSG00000254353	3.724485	-2.294189	1	1	RP11-195E2.4	_
ENSG00000254362	3.724485	-2.294189	1	1	RP11-14I17.3	_
ENSG00000254477	3.724485	-2.294189	1	1	AP000640.10	_
ENSG00000254577	3.724485	-2.294189	1	1	RP11-484D2.4	_
ENSG00000254648	3.724485	-2.294189	1	1	RP11-713P17.4	_
ENSG00000254672	3.724485	-2.294189	1	1	RP5-916O11.3	K06632
ENSG00000254747	3.724485	-2.294189	1	1	RP11-664H7.1	_
ENSG00000254791	3.724485	-2.294189	1	1	FAR1-IT1	_
ENSG00000254854	3.724485	-2.294189	1	1	CTD-2523D13.2	_
ENSG00000254879	3.724485	-2.294189	1	1	AC103828.1	_
ENSG00000254884	3.724485	-2.294189	1	1	RP11-682B13.2	_
ENSG00000254973	3.724485	-2.294189	1	1	RP11-429J17.7	_
ENSG00000254982	3.724485	-2.294189	1	1	HMGB1P24	K10802
ENSG00000255115	3.724485	-2.294189	1	1	RP11-91P24.3	_
ENSG00000255156	3.724485	-2.294189	1	1	KLHL22-IT1	_
ENSG00000255186	3.724485	-2.294189	1	1	RP11-514F3.5	_

ENSG00000255250	3.724485	-2.294189	1	1	CTD-2005H7.2	_
ENSG00000255272	3.724485	-2.294189	1	1	RP11-646J21.6	_
ENSG00000255362	3.724485	-2.294189	1	1	RP11-619A14.3	_
ENSG00000255382	3.724485	-2.294189	1	1	RP11-718B12.2	_
ENSG00000255420	3.724485	-2.294189	1	1	RP11-236J17.3	_
ENSG00000255434	3.724485	-2.294189	1	1	CTD-2530H12.8	_
ENSG00000255670	3.724485	-2.294189	1	1	RP11-253I19.3	_
ENSG00000255693	3.724485	-2.294189	1	1	RP11-766N7.3	_
ENSG00000255776	3.724485	-2.294189	1	1	RP11-436I9.3	K15040
ENSG00000255817	3.724485	-2.294189	1	1	RP11-196H14.4	_
ENSG00000255967	3.724485	-2.294189	1	1	RP11-438L7.3	K07515
ENSG00000256008	3.724485	-2.294189	1	1	RP11-728G15.1	_
ENSG00000256011	3.724485	-2.294189	1	1	RP11-392P7.7	_
ENSG00000256116	3.724485	-2.294189	1	1	RP11-783K16.14	_
ENSG00000256139	3.724485	-2.294189	1	1	RP11-968O1.5	_
ENSG00000256195	3.724485	-2.294189	1	1	RP11-64D24.4	_
ENSG00000256299	3.724485	-2.294189	1	1	RP11-989F5.3	_
ENSG00000256731	3.724485	-2.294189	1	1	AL591069.1	_
ENSG00000256937	3.724485	-2.294189	1	1	RP11-436I9.5	K07604
ENSG00000256981	3.724485	-2.294189	1	1	TAS2R18	K08474
ENSG00000257241	3.724485	-2.294189	1	1	RP11-611E13.3	_
ENSG00000257272	3.724485	-2.294189	1	1	RP11-317N8.3	_
ENSG00000257289	3.724485	-2.294189	1	1	RP11-611O2.6	K04631
ENSG00000257302	3.724485	-2.294189	1	1	FAHD2P1	_
ENSG00000257384	3.724485	-2.294189	1	1	RP11-644F5.12	_
ENSG00000257456	3.724485	-2.294189	1	1	RP11-310I24.1	_
ENSG00000257474	3.724485	-2.294189	1	1	RP11-359M6.1	_
ENSG00000257529	3.724485	-2.294189	1	1	RPL36A-HNRNPH2	K12898
ENSG00000257803	3.724485	-2.294189	1	1	RP11-575G13.2	K03857
ENSG00000257894	3.724485	-2.294189	1	1	RP1-78O14.1	_
ENSG00000257947	3.724485	-2.294189	1	1	RP11-46I1.1	_
ENSG00000257999	3.724485	-2.294189	1	1	RP11-61E11.2	_
ENSG00000258177	3.724485	-2.294189	1	1	RP11-394J1.2	_
ENSG00000258205	3.724485	-2.294189	1	1	RP11-248E9.1	_
ENSG00000258240	3.724485	-2.294189	1	1	RP1-46F2.3	_
ENSG00000258380	3.724485	-2.294189	1	1	RP11-33N16.2	_
ENSG00000258425	3.724485	-2.294189	1	1	CTD-2207P18.1	_
ENSG00000258428	3.724485	-2.294189	1	1	RP11-1085N6.2	_
ENSG00000258553	3.724485	-2.294189	1	1	RP11-16B13.1	_
ENSG00000258571	3.724485	-2.294189	1	1	PTTG4P	K06635
ENSG00000258615	3.724485	-2.294189	1	1	RP11-725G5.3	_
ENSG00000258632	3.724485	-2.294189	1	1	RP11-354B3.1	K02898
ENSG00000258679	3.724485	-2.294189	1	1	RP11-74M13.4	K16197
ENSG00000258717	3.724485	-2.294189	1	1	RP11-566J3.2	_
ENSG00000258789	3.724485	-2.294189	1	1	RP11-507K2.3	_
ENSG00000258815	3.724485	-2.294189	1	1	RP11-408B11.2	_
ENSG00000258823	3.724485	-2.294189	1	1	CTD-2555K7.3	_
ENSG00000258845	3.724485	-2.294189	1	1	RP11-945F5.1	_
ENSG00000258917	3.724485	-2.294189	1	1	ZMYND19P1	_
ENSG00000259002	3.724485	-2.294189	1	1	RP11-671J11.7	_
ENSG00000259078	3.724485	-2.294189	1	1	PTBP1P	K13218
ENSG00000259215	3.724485	-2.294189	1	1	RP11-253M7.4	_
ENSG00000259232	3.724485	-2.294189	1	1	RP11-349G13.1	K13202
ENSG00000259268	3.724485	-2.294189	1	1	AC007950.1	_
ENSG00000259335	3.724485	-2.294189	1	1	HNRNPMP1	K12887
ENSG00000259351	3.724485	-2.294189	1	1	RP11-111E14.1	_
ENSG00000259378	3.724485	-2.294189	1	1	DCAF13P3	K11806
ENSG00000259405	3.724485	-2.294189	1	1	ISCA1P4	K13628

ENSG00000259410	3.724485	-2.294189	1	1	RP11-34F13.3	_
ENSG00000259411	3.724485	-2.294189	1	1	HNRNPA1P45	K12741
ENSG00000259414	3.724485	-2.294189	1	1	HERC2P7	K10595
ENSG00000259429	3.724485	-2.294189	1	1	UBE2Q2P3	K10582
ENSG00000259473	3.724485	-2.294189	1	1	RP11-96C21.1	_
ENSG00000259474	3.724485	-2.294189	1	1	RP11-650L12.1	_
ENSG00000259504	3.724485	-2.294189	1	1	RP11-352D13.5	_
ENSG00000259536	3.724485	-2.294189	1	1	RP11-111A22.1	_
ENSG00000259563	3.724485	-2.294189	1	1	CTD-2329K10.1	_
ENSG00000259570	3.724485	-2.294189	1	1	RP11-671M22.4	_
ENSG00000259645	3.724485	-2.294189	1	1	RP11-253M7.6	_
ENSG00000259730	3.724485	-2.294189	1	1	CTD-2007H18.1	_
ENSG00000259735	3.724485	-2.294189	1	1	RP11-356M20.3	_
ENSG00000259754	3.724485	-2.294189	1	1	RP11-208K4.1	_
ENSG00000259876	3.724485	-2.294189	1	1	CTD-3037G24.4	_
ENSG00000259924	3.724485	-2.294189	1	1	RP11-16E23.4	K09520
ENSG00000259928	3.724485	-2.294189	1	1	RP11-218M11.1	_
ENSG00000259964	3.724485	-2.294189	1	1	RP11-673C5.2	_
ENSG00000260013	3.724485	-2.294189	1	1	APOOP5	_
ENSG00000260038	3.724485	-2.294189	1	1	RP11-407G23.4	_
ENSG00000260047	3.724485	-2.294189	1	1	RP11-989E6.2	K14009
ENSG00000260185	3.724485	-2.294189	1	1	RP11-432I5.6	_
ENSG00000260239	3.724485	-2.294189	1	1	RP11-274H24.1	_
ENSG00000260347	3.724485	-2.294189	1	1	MOCS1P1	K03639
ENSG00000260369	3.724485	-2.294189	1	1	CTD-2526A2.2	_
ENSG00000260488	3.724485	-2.294189	1	1	RP11-166B2.7	_
ENSG00000260602	3.724485	-2.294189	1	1	HMGN2P40	K11300
ENSG00000260619	3.724485	-2.294189	1	1	RP11-775C24.3	_
ENSG00000260631	3.724485	-2.294189	1	1	RBM22P12	K12872
ENSG00000260657	3.724485	-2.294189	1	1	RP11-315D16.4	_
ENSG00000260685	3.724485	-2.294189	1	1	RP11-326L17.1	_
ENSG00000260738	3.724485	-2.294189	1	1	LINC00554	_
ENSG00000260776	3.724485	-2.294189	1	1	RP11-114H24.2	K10595
ENSG00000260865	3.724485	-2.294189	1	1	CTA-331F8.1	_
ENSG00000260870	3.724485	-2.294189	1	1	RP11-358M11.4	K03966
ENSG00000260895	3.724485	-2.294189	1	1	RP11-554A11.7	_
ENSG00000260903	3.724485	-2.294189	1	1	XKR7	_
ENSG00000260971	3.724485	-2.294189	1	1	RP11-504A18.1	_
ENSG00000261024	3.724485	-2.294189	1	1	GS1-279B7.1	_
ENSG00000261044	3.724485	-2.294189	1	1	AP006547.3	_
ENSG00000261092	3.724485	-2.294189	1	1	RP11-21B23.2	_
ENSG00000261096	3.724485	-2.294189	1	1	RP11-690I21.2	_
ENSG00000261124	3.724485	-2.294189	1	1	AC135050.5	_
ENSG00000261131	3.724485	-2.294189	1	1	RP11-93O14.2	_
ENSG00000261186	3.724485	-2.294189	1	1	RP11-341N2.1	_
ENSG00000261242	3.724485	-2.294189	1	1	CTD-2302E22.4	_
ENSG00000261320	3.724485	-2.294189	1	1	RP11-297D21.2	_
ENSG00000261341	3.724485	-2.294189	1	1	CTD-2568A17.1	_
ENSG00000261407	3.724485	-2.294189	1	1	RP11-326A19.3	_
ENSG00000261433	3.724485	-2.294189	1	1	CTC-297N7.1	_
ENSG00000261439	3.724485	-2.294189	1	1	CTD-2050B12.2	_
ENSG00000261512	3.724485	-2.294189	1	1	RP11-46D6.1	_
ENSG00000261524	3.724485	-2.294189	1	1	ABCB10P3	K05657
ENSG00000261620	3.724485	-2.294189	1	1	HMGN2P41	K11300
ENSG00000261644	3.724485	-2.294189	1	1	RP11-327F22.2	_
ENSG00000261656	3.724485	-2.294189	1	1	CTD-2258A20.5	_
ENSG00000261687	3.724485	-2.294189	1	1	RP11-473C18.3	_
ENSG00000261774	3.724485	-2.294189	1	1	RP11-328J14.2	_

ENSG00000261790	3.724485	-2.294189	1	1	AC005606.14	_
ENSG00000261803	3.724485	-2.294189	1	1	RP11-434E6.2	_
ENSG00000261925	3.724485	-2.294189	1	1	RP11-388M20.9	_
ENSG00000261959	3.724485	-2.294189	1	1	RP11-893F2.14	_
ENSG00000261963	3.724485	-2.294189	1	1	RP11-74E22.4	K16452
ENSG00000262133	3.724485	-2.294189	1	1	RP11-676J12.6	_
ENSG00000262155	3.724485	-2.294189	1	1	RP11-266L9.5	_
ENSG00000262179	3.724485	-2.294189	1	1	RP1-302G2.5	_
ENSG00000262370	3.724485	-2.294189	1	1	RP11-473M20.9	_
ENSG00000262480	3.724485	-2.294189	1	1	SAMD11P1	_
ENSG00000262514	3.724485	-2.294189	1	1	RP11-96D1.9	_
ENSG00000262662	3.724485	-2.294189	1	1	CTD-2561B21.3	_
ENSG00000262759	3.724485	-2.294189	1	1	MRPS21P9	_
ENSG00000262870	3.724485	-2.294189	1	1	CYCSP40	_
ENSG00000262874	3.724485	-2.294189	1	1	C19orf84	_
ENSG00000262905	3.724485	-2.294189	1	1	RP5-1029F21.2	_
ENSG00000262943	3.724485	-2.294189	1	1	ALOX12P2	K00458
ENSG00000262983	3.724485	-2.294189	1	1	AF001550.7	_
ENSG00000263017	3.724485	-2.294189	1	1	RP11-356I18.1	_
ENSG00000263164	3.724485	-2.294189	1	1	RP11-333E1.2	_
ENSG00000263279	3.724485	-2.294189	1	1	RP11-109M19.1	_
ENSG00000263338	3.724485	-2.294189	1	1	RP11-235E17.3	_
ENSG00000263383	3.724485	-2.294189	1	1	AC005549.1	_
ENSG00000263389	3.724485	-2.294189	1	1	MIR3973	_
ENSG00000263466	3.724485	-2.294189	1	1	RP1-56K13.2	_
ENSG00000263584	3.724485	-2.294189	1	1	MIR4480	_
ENSG00000263607	3.724485	-2.294189	1	1	RN7SL80P	_
ENSG00000263648	3.724485	-2.294189	1	1	RP11-471L13.3	K10420
ENSG00000263762	3.724485	-2.294189	1	1	MIR4286	_
ENSG00000263887	3.724485	-2.294189	1	1	RP11-51L5.5	K11837
ENSG00000263913	3.724485	-2.294189	1	1	AC004812.1	_
ENSG00000263975	3.724485	-2.294189	1	1	CTB-58E17.9	_
ENSG00000263995	3.724485	-2.294189	1	1	AC011499.1	_
ENSG00000264017	3.724485	-2.294189	1	1	RN7SL336P	_
ENSG00000264127	3.724485	-2.294189	1	1	SCML2P1	_
ENSG00000264213	3.724485	-2.294189	1	1	RN7SL324P	_
ENSG00000264216	3.724485	-2.294189	1	1	NOS2P1	K13241
ENSG00000264224	3.724485	-2.294189	1	1	AC004655.1	_
ENSG00000264256	3.724485	-2.294189	1	1	AC025166.1	_
ENSG00000264308	3.724485	-2.294189	1	1	AL121897.1	_
ENSG00000264317	3.724485	-2.294189	1	1	RN7SL154P	_
ENSG00000264408	3.724485	-2.294189	1	1	MIR4470	_
ENSG00000264627	3.724485	-2.294189	1	1	RN7SL820P	_
ENSG00000264735	3.724485	-2.294189	1	1	RP11-498C9.17	_
ENSG00000264843	3.724485	-2.294189	1	1	RP11-856M7.2	_
ENSG00000264853	3.724485	-2.294189	1	1	RP11-16C1.2	_
ENSG00000264895	3.724485	-2.294189	1	1	RP11-421E14.2	_
ENSG00000264947	3.724485	-2.294189	1	1	hsa-mir-3181	_
ENSG00000264994	3.724485	-2.294189	1	1	SNORD92	_
ENSG00000265068	3.724485	-2.294189	1	1	AL591377.1	_
ENSG00000265089	3.724485	-2.294189	1	1	MIR4655	_
ENSG00000265218	3.724485	-2.294189	1	1	RP11-927P21.1	_
ENSG00000265261	3.724485	-2.294189	1	1	RP11-162A12.3	K14288
ENSG00000265289	3.724485	-2.294189	1	1	AC104984.4	_
ENSG00000265314	3.724485	-2.294189	1	1	MIR548AH	_
ENSG00000265342	3.724485	-2.294189	1	1	RP11-16C1.1	_
ENSG00000265445	3.724485	-2.294189	1	1	RP11-849N15.1	_
ENSG00000265450	3.724485	-2.294189	1	1	MIR4502	_

ENSG00000265462	3.724485	-2.294189	1	1	MIR3680-1	_
ENSG00000265496	3.724485	-2.294189	1	1	MIR1539	_
ENSG00000265648	3.724485	-2.294189	1	1	RN7SL279P	_
ENSG00000265654	3.724485	-2.294189	1	1	AL513547.1	_
ENSG00000265660	3.724485	-2.294189	1	1	MIR4664	_
ENSG00000265713	3.724485	-2.294189	1	1	RP11-82O19.2	_
ENSG00000265715	3.724485	-2.294189	1	1	MIR3118-1	_
ENSG00000265798	3.724485	-2.294189	1	1	RP11-271K11.5	_
ENSG00000265820	3.724485	-2.294189	1	1	MIR3177	_
ENSG00000265907	3.724485	-2.294189	1	1	RP11-737O24.2	_
ENSG00000265908	3.724485	-2.294189	1	1	RP11-20B24.6	_
ENSG00000265939	3.724485	-2.294189	1	1	UBE2CP2	_
ENSG00000265944	3.724485	-2.294189	1	1	C18orf64	_
ENSG00000265948	3.724485	-2.294189	1	1	RP11-296E23.2	_
ENSG00000266017	3.724485	-2.294189	1	1	MIR4477A	_
ENSG00000266097	3.724485	-2.294189	1	1	MIR5192	_
ENSG00000266155	3.724485	-2.294189	1	1	RP11-927P21.6	_
ENSG00000266315	3.724485	-2.294189	1	1	MIR4668	_
ENSG00000266465	3.724485	-2.294189	1	1	AC025165.1	_
ENSG00000266467	3.724485	-2.294189	1	1	RN7SL220P	_
ENSG00000266538	3.724485	-2.294189	1	1	RP11-385D13.3	_
ENSG00000266654	3.724485	-2.294189	1	1	RP11-1376P16.1	_
ENSG00000266657	3.724485	-2.294189	1	1	AL583842.3	_
ENSG00000266728	3.724485	-2.294189	1	1	AC015688.3	K10093
ENSG00000266783	3.724485	-2.294189	1	1	RP11-715F3.2	_
ENSG00000266801	3.724485	-2.294189	1	1	RP11-521L9.2	_
ENSG00000266832	3.724485	-2.294189	1	1	AL445464.1	_
ENSG00000266895	3.724485	-2.294189	1	1	CTC-268N12.2	_
ENSG00000266897	3.724485	-2.294189	1	1	AC005546.2	_
ENSG00000266913	3.724485	-2.294189	1	1	CTC-548K16.2	_
ENSG00000266953	3.724485	-2.294189	1	1	RP11-618P17.4	K14801
ENSG00000266957	3.724485	-2.294189	1	1	RP11-49K24.4	_
ENSG00000266959	3.724485	-2.294189	1	1	AC005786.3	K02993
ENSG00000266990	3.724485	-2.294189	1	1	AC004528.4	_
ENSG00000267006	3.724485	-2.294189	1	1	CTC-448F2.4	_
ENSG00000267009	3.724485	-2.294189	1	1	RP11-120M18.2	_
ENSG00000267011	3.724485	-2.294189	1	1	CTB-50L17.16	_
ENSG00000267025	3.724485	-2.294189	1	1	AC015849.19	_
ENSG00000267051	3.724485	-2.294189	1	1	RP11-128P17.2	_
ENSG00000267052	3.724485	-2.294189	1	1	CTB-30L5.1	_
ENSG00000267069	3.724485	-2.294189	1	1	RP11-64C12.8	_
ENSG00000267078	3.724485	-2.294189	1	1	RP11-666A8.9	_
ENSG00000267122	3.724485	-2.294189	1	1	AC004490.1	_
ENSG00000267127	3.724485	-2.294189	1	1	RP11-795F19.5	_
ENSG00000267179	3.724485	-2.294189	1	1	ZNF763	K09228
ENSG00000267218	3.724485	-2.294189	1	1	AC005336.5	K10908
ENSG00000267274	3.724485	-2.294189	1	1	CTD-2006C1.12	_
ENSG00000267296	3.724485	-2.294189	1	1	CEBPA-AS1	_
ENSG00000267319	3.724485	-2.294189	1	1	CTD-2528L19.3	K09228
ENSG00000267322	3.724485	-2.294189	1	1	SCARNA17	_
ENSG00000267334	3.724485	-2.294189	1	1	CTD-2534I21.8	_
ENSG00000267346	3.724485	-2.294189	1	1	EIF5AP3	K03263
ENSG00000267405	3.724485	-2.294189	1	1	CTC-296K1.4	_
ENSG00000267406	3.724485	-2.294189	1	1	CTD-2189E23.2	_
ENSG00000267455	3.724485	-2.294189	1	1	RP11-78A19.2	_
ENSG00000267491	3.724485	-2.294189	1	1	CTD-2373H9.5	_
ENSG00000267504	3.724485	-2.294189	1	1	RP11-845C23.2	_
ENSG00000267539	3.724485	-2.294189	1	1	RP11-138H8.7	_

ENSG00000267545	3.724485	-2.294189	1	1	AC005779.2	_
ENSG00000267583	3.724485	-2.294189	1	1	RP11-322E11.5	_
ENSG00000267587	3.724485	-2.294189	1	1	RP11-687F6.4	K00384
ENSG00000267620	3.724485	-2.294189	1	1	RP11-795H16.2	K03141
ENSG00000267703	3.724485	-2.294189	1	1	CTD-2013N17.4	_
ENSG00000267735	3.724485	-2.294189	1	1	CTD-2265O21.7	_
ENSG00000267786	3.724485	-2.294189	1	1	AF038458.3	_
ENSG00000267788	3.724485	-2.294189	1	1	CTD-2534I21.9	_
ENSG00000267889	3.724485	-2.294189	1	1	AC079354.2	_
ENSG00000267892	3.724485	-2.294189	1	1	CTD-2540F13.2	_
ENSG00000268041	3.724485	-2.294189	1	1	CTD-2575K13.6	K09434
ENSG00000268049	3.724485	-2.294189	1	1	CTD-2619J13.9	_
ENSG00000268157	3.724485	-2.294189	1	1	AC010524.4	_
ENSG00000268175	3.724485	-2.294189	1	1	AC117395.1	_
ENSG00000268230	3.724485	-2.294189	1	1	CTD-2619J13.8	_
ENSG00000268397	3.724485	-2.294189	1	1	AC008443.1	K12025
ENSG00000268400	3.724485	-2.294189	1	1	CTD-3214H19.4	K15300
ENSG00000268401	3.724485	-2.294189	1	1	CTC-344H19.4	_
ENSG00000268434	3.724485	-2.294189	1	1	AC011530.4	K08788
ENSG00000268510	3.724485	-2.294189	1	1	IFNL3P1	K05447
ENSG00000268530	3.724485	-2.294189	1	1	CTC-273B12.5	_
ENSG00000268688	3.724485	-2.294189	1	1	AC007382.1	_
ENSG00000268743	3.724485	-2.294189	1	1	CTD-2538G9.5	_
ENSG00000268746	3.724485	-2.294189	1	1	CTD-2571L23.8	_
ENSG00000268747	3.724485	-2.294189	1	1	CTD-3030D20.1	K18182
ENSG00000268791	3.724485	-2.294189	1	1	AC093323.1	_
ENSG00000268805	3.724485	-2.294189	1	1	PRED57	_
ENSG00000268846	3.724485	-2.294189	1	1	AC018867.2	_
ENSG00000268849	3.724485	-2.294189	1	1	SIGLEC22P	K06473
ENSG00000268965	3.724485	-2.294189	1	1	AC061992.1	K10408
ENSG00000269068	3.724485	-2.294189	1	1	RP11-256I23.2	_
ENSG00000269102	3.724485	-2.294189	1	1	CTD-2525I3.5	_
ENSG00000269282	3.724485	-2.294189	1	1	AC024257.1	_
ENSG00000269321	3.724485	-2.294189	1	1	CTC-453G23.4	_
ENSG00000269425	3.724485	-2.294189	1	1	AC007292.7	_
ENSG00000269584	3.724485	-2.294189	1	1	TDGF1P7	_
ENSG00000269600	3.724485	-2.294189	1	1	AC016629.3	_
ENSG00000269895	3.724485	-2.294189	1	1	AP000654.4	_
ENSG00000269898	3.724485	-2.294189	1	1	RP11-568J23.4	_
ENSG00000269955	3.724485	-2.294189	1	1	LUC7L2	K13212
ENSG00000269962	3.724485	-2.294189	1	1	RP13-238F13.5	_
ENSG00000270016	3.724485	-2.294189	1	1	RP11-932O9.8	_
ENSG00000270031	3.724485	-2.294189	1	1	RP3-426I6.6	_
ENSG00000270040	3.724485	-2.294189	1	1	RP11-416A14.1	_
ENSG00000270071	3.724485	-2.294189	1	1	AP001172.2	_
ENSG00000270115	3.724485	-2.294189	1	1	RP11-415J8.7	_
ENSG00000270116	3.724485	-2.294189	1	1	AP001429.1	_
ENSG00000270124	3.724485	-2.294189	1	1	RP11-118F19.1	_
ENSG00000270136	3.724485	-2.294189	1	1	MINOS1-NBL1	K19558
ENSG00000270257	3.724485	-2.294189	1	1	RP11-17P16.2	_
ENSG00000270296	3.724485	-2.294189	1	1	RP11-59H1.4	K08501
ENSG00000270314	3.724485	-2.294189	1	1	GS1-165B14.2	_
ENSG00000270322	3.724485	-2.294189	1	1	RP11-697E2.10	K11644
ENSG00000270409	3.724485	-2.294189	1	1	RP11-44D5.1	_
ENSG00000270638	3.724485	-2.294189	1	1	RP3-466P17.1	_
ENSG00000270697	3.724485	-2.294189	1	1	RP11-381K20.4	_
ENSG00000270755	3.724485	-2.294189	1	1	RP11-138E2.1	_
ENSG00000270775	3.724485	-2.294189	1	1	AP000436.4	K03231

ENSG00000270823	3.724485	-2.294189	1	1	hsa-mir-335	_
ENSG00000270846	3.724485	-2.294189	1	1	RP11-674I16.1	_
ENSG00000270857	3.724485	-2.294189	1	1	RP11-757G14.3	K10704
ENSG00000270937	3.724485	-2.294189	1	1	RP5-856G1.1	_
ENSG00000270948	3.724485	-2.294189	1	1	RP11-460N20.7	_
ENSG00000271100	3.724485	-2.294189	1	1	RP11-697H9.5	K02147
ENSG00000271199	3.724485	-2.294189	1	1	RP13-216E22.5	_
ENSG00000271228	3.724485	-2.294189	1	1	CTC-336P14.1	_
ENSG00000271236	3.724485	-2.294189	1	1	SUMO2P15	K12160
ENSG00000271252	3.724485	-2.294189	1	1	RP11-286B14.2	_
ENSG00000271314	3.724485	-2.294189	1	1	RP11-435O5.6	_
ENSG00000271370	3.724485	-2.294189	1	1	RP11-253I19.4	K12399
ENSG00000271410	3.724485	-2.294189	1	1	RP11-506H20.2	K08808
ENSG00000271437	3.724485	-2.294189	1	1	RP11-295B17.6	K15413
ENSG00000271484	3.724485	-2.294189	1	1	RP11-132J14.3	_
ENSG00000271511	3.724485	-2.294189	1	1	RP11-712C19.1	K17772
ENSG00000271554	3.724485	-2.294189	1	1	RP4-665N4.8	_
ENSG00000271586	3.724485	-2.294189	1	1	RP11-52M17.1	_
ENSG00000271607	3.724485	-2.294189	1	1	RP3-418A9.3	_
ENSG00000271635	3.724485	-2.294189	1	1	RP11-68E19.1	K06629
ENSG00000271647	3.724485	-2.294189	1	1	KRT8P47	K07605
ENSG00000271730	3.724485	-2.294189	1	1	RP11-787I22.3	_
ENSG00000271732	3.724485	-2.294189	1	1	RP5-1182A14.5	_
ENSG00000271761	3.724485	-2.294189	1	1	XXbac-BPGBPG55(	_
ENSG00000271835	3.724485	-2.294189	1	1	RP11-129K12.4	_
ENSG00000271868	3.724485	-2.294189	1	1	RP11-1293J14.1	_
ENSG00000272109	3.724485	-2.294189	1	1	CTD-2260A17.3	_
ENSG00000272181	3.724485	-2.294189	1	1	RP11-245J9.6	_
ENSG00000272211	3.724485	-2.294189	1	1	RP11-347P5.1	_
ENSG00000272265	3.724485	-2.294189	1	1	CTD-2287O16.4	_
ENSG00000272283	3.724485	-2.294189	1	1	Metazoa_SRP	_
ENSG00000272324	3.724485	-2.294189	1	1	CTD-2154B17.4	_
ENSG00000272381	3.724485	-2.294189	1	1	RP11-192P3.4	_
ENSG00000272735	3.724485	-2.294189	1	1	RP11-467P9.1	_
ENSG00000272950	3.724485	-2.294189	1	1	RP11-307C18.1	_
ENSG00000272963	3.724485	-2.294189	1	1	OR7A19P	K04257
ENSG00000273003	3.724485	-2.294189	1	1	RP11-399J13.3	K17927
ENSG00000273090	3.724485	-2.294189	1	1	RP11-78I14.1	_
ENSG00000273196	3.724485	-2.294189	1	1	RP11-717A5.2	_
ENSG00000273287	3.724485	-2.294189	1	1	CTA-268H5.14	_
ENSG00000273308	3.724485	-2.294189	1	1	RP11-496H1.2	_
ENSG00000273328	3.724485	-2.294189	1	1	RP11-141M3.5	_
ENSG00000273428	3.724485	-2.294189	1	1	RP4-539M6.22	_
ENSG00000273444	3.724485	-2.294189	1	1	RP5-1147A1.2	K01265
ENSG00000273464	3.724485	-2.294189	1	1	RP11-313P22.1	_
ENSG00000273476	3.724485	-2.294189	1	1	RP11-108L7.14	_
ENSG00000001626	-3.68859	-2.060656	1	1	CFTR	_
ENSG00000006210	-3.68859	-2.060656	1	1	CX3CL1	K05508
ENSG00000010932	-3.68859	-2.060656	1	1	FMO1	K00485
ENSG00000015413	-3.68859	-2.060656	1	1	DPEP1	K01273
ENSG00000017483	-3.68859	-2.060656	1	1	SLC38A5	K14992
ENSG00000048540	-3.68859	-2.060656	1	1	LMO3	_
ENSG00000054219	-3.68859	-2.060656	1	1	LY75	K06559
ENSG00000064225	-3.68859	-2.060656	1	1	ST3GAL6	K03792
ENSG00000068354	-3.68859	-2.060656	1	1	TBC1D25	_
ENSG00000068781	-3.68859	-2.060656	1	1	STON1-GTF2A1L	K03122
ENSG00000069206	-3.68859	-2.060656	1	1	ADAM7	K16071
ENSG00000077327	-3.68859	-2.060656	1	1	SPAG6	_

ENSG00000077522	-3.68859	-2.060656	1	1	ACTN2	K05699
ENSG00000078589	-3.68859	-2.060656	1	1	P2RY10	K04274
ENSG00000079974	-3.68859	-2.060656	1	1	RABL2B	K07931
ENSG00000081237	-3.68859	-2.060656	1	1	PTPRC	K06478
ENSG00000091482	-3.68859	-2.060656	1	1	SMPX	_
ENSG00000091513	-3.68859	-2.060656	1	1	TF	K14736
ENSG00000100473	-3.68859	-2.060656	1	1	COCH	_
ENSG00000100678	-3.68859	-2.060656	1	1	SLC8A3	K05849
ENSG00000101278	-3.68859	-2.060656	1	1	RPS10L	K02947
ENSG00000101280	-3.68859	-2.060656	1	1	ANGPT4	K05467
ENSG00000101460	-3.68859	-2.060656	1	1	MAP1LC3A	K10435
ENSG00000101670	-3.68859	-2.060656	1	1	LIPG	K01046
ENSG00000101842	-3.68859	-2.060656	1	1	VSIG1	_
ENSG00000102312	-3.68859	-2.060656	1	1	PORCN	K00181
ENSG00000104814	-3.68859	-2.060656	1	1	MAP4K1	K04408
ENSG00000105131	-3.68859	-2.060656	1	1	EPHX3	_
ENSG00000105219	-3.68859	-2.060656	1	1	CNTD2	_
ENSG00000106034	-3.68859	-2.060656	1	1	CPED1	_
ENSG00000106483	-3.68859	-2.060656	1	1	SFRP4	K02185
ENSG00000108255	-3.68859	-2.060656	1	1	CRYBA1	_
ENSG00000109047	-3.68859	-2.060656	1	1	RCVRN	K13764
ENSG00000109163	-3.68859	-2.060656	1	1	GNRHR	K04280
ENSG00000109625	-3.68859	-2.060656	1	1	CPZ	K13022
ENSG00000109758	-3.68859	-2.060656	1	1	HGFAC	K09631
ENSG00000112053	-3.68859	-2.060656	1	1	SLC26A8	K14705
ENSG00000113263	-3.68859	-2.060656	1	1	ITK	K07363
ENSG00000113946	-3.68859	-2.060656	1	1	CLDN16	K06087
ENSG00000114638	-3.68859	-2.060656	1	1	UPK1B	K17347
ENSG00000115085	-3.68859	-2.060656	1	1	ZAP70	K07360
ENSG00000115221	-3.68859	-2.060656	1	1	ITGB6	_
ENSG00000116774	-3.68859	-2.060656	1	1	OLFML3	_
ENSG00000118520	-3.68859	-2.060656	1	1	ARG1	K01476
ENSG00000119608	-3.68859	-2.060656	1	1	PROX2	_
ENSG00000120328	-3.68859	-2.060656	1	1	PCDHB12	K16494
ENSG00000121764	-3.68859	-2.060656	1	1	HCRTR1	K04238
ENSG00000121904	-3.68859	-2.060656	1	1	CSMD2	K17495
ENSG00000122188	-3.68859	-2.060656	1	1	LAX1	K12022
ENSG00000123500	-3.68859	-2.060656	1	1	COL10A1	K19479
ENSG00000124215	-3.68859	-2.060656	1	1	CDH26	_
ENSG00000125571	-3.68859	-2.060656	1	1	IL37	K05485
ENSG00000125895	-3.68859	-2.060656	1	1	TMEM74B	_
ENSG00000128250	-3.68859	-2.060656	1	1	RFPL1	_
ENSG00000130201	-3.68859	-2.060656	1	1	EXOC3L2	K09228
ENSG00000130413	-3.68859	-2.060656	1	1	STK33	K08813
ENSG00000130701	-3.68859	-2.060656	1	1	RBBP8NL	_
ENSG00000130755	-3.68859	-2.060656	1	1	GMFG	_
ENSG00000130943	-3.68859	-2.060656	1	1	PKDREJ	_
ENSG00000131095	-3.68859	-2.060656	1	1	GFAP	K05640
ENSG00000131773	-3.68859	-2.060656	1	1	KHDRBS3	K14942
ENSG00000133477	-3.68859	-2.060656	1	1	FAM83F	K12581
ENSG00000135517	-3.68859	-2.060656	1	1	MIP	K09863
ENSG00000135702	-3.68859	-2.060656	1	1	CHST5	K09670
ENSG00000135828	-3.68859	-2.060656	1	1	RNASEL	K01165
ENSG00000135914	-3.68859	-2.060656	1	1	HTR2B	K04157
ENSG00000136059	-3.68859	-2.060656	1	1	VILL	_
ENSG00000136160	-3.68859	-2.060656	1	1	EDNRB	K04198
ENSG00000137691	-3.68859	-2.060656	1	1	C11orf70	_
ENSG00000137948	-3.68859	-2.060656	1	1	BRDT	K11724

ENSG00000137960	-3.68859	-2.060656	1	1	GIPC2	_
ENSG00000138315	-3.68859	-2.060656	1	1	OIT3	_
ENSG00000139292	-3.68859	-2.060656	1	1	LGR5	K04308
ENSG00000140519	-3.68859	-2.060656	1	1	RHCG	K06580
ENSG00000140873	-3.68859	-2.060656	1	1	ADAMTS18	K08632
ENSG00000141469	-3.68859	-2.060656	1	1	SLC14A1	K08716
ENSG00000141698	-3.68859	-2.060656	1	1	NT5C3B	K01081
ENSG00000143140	-3.68859	-2.060656	1	1	GJA5	K07614
ENSG00000143368	-3.68859	-2.060656	1	1	SF3B4	K12831
ENSG00000144712	-3.68859	-2.060656	1	1	CAND2	K17263
ENSG00000144821	-3.68859	-2.060656	1	1	MYH15	K10352
ENSG00000145063	-3.68859	-2.060656	1	1	AC062028.1	_
ENSG00000146006	-3.68859	-2.060656	1	1	LRRTM2	K16665
ENSG00000147485	-3.68859	-2.060656	1	1	PXDNL	K19511
ENSG00000147896	-3.68859	-2.060656	1	1	IFNK	K05441
ENSG00000149050	-3.68859	-2.060656	1	1	ZNF214	K09228
ENSG00000149633	-3.68859	-2.060656	1	1	KIAA1755	_
ENSG00000149656	-3.68859	-2.060656	1	1	LINC00266-1	_
ENSG00000151062	-3.68859	-2.060656	1	1	CACNA2D4	K04861
ENSG00000152578	-3.68859	-2.060656	1	1	GRIA4	K05200
ENSG00000153064	-3.68859	-2.060656	1	1	BANK1	_
ENSG00000153253	-3.68859	-2.060656	1	1	SCN3A	K04836
ENSG00000154485	-3.68859	-2.060656	1	1	MMP21	K08000
ENSG00000154589	-3.68859	-2.060656	1	1	LY96	K05400
ENSG00000154839	-3.68859	-2.060656	1	1	SKA1	_
ENSG00000155016	-3.68859	-2.060656	1	1	CYP2U1	K07422
ENSG00000155428	-3.68859	-2.060656	1	1	TRIM74	K12024
ENSG00000158077	-3.68859	-2.060656	1	1	NLRP14	_
ENSG00000158113	-3.68859	-2.060656	1	1	LRRC43	_
ENSG00000160185	-3.68859	-2.060656	1	1	UBASH3A	K18993
ENSG00000160323	-3.68859	-2.060656	1	1	ADAMTS13	K08627
ENSG00000160838	-3.68859	-2.060656	1	1	LRRC71	_
ENSG00000161610	-3.68859	-2.060656	1	1	HCRT	K05246
ENSG00000162426	-3.68859	-2.060656	1	1	SLC45A1	K15378
ENSG00000162825	-3.68859	-2.060656	1	1	NBPF8	_
ENSG00000162882	-3.68859	-2.060656	1	1	HAAO	K00452
ENSG00000162891	-3.68859	-2.060656	1	1	IL20	K05444
ENSG00000162998	-3.68859	-2.060656	1	1	FRZB	K02185
ENSG00000163121	-3.68859	-2.060656	1	1	NEURL3	K15689
ENSG00000163263	-3.68859	-2.060656	1	1	C1orf189	_
ENSG00000163689	-3.68859	-2.060656	1	1	C3orf67	_
ENSG00000163810	-3.68859	-2.060656	1	1	TGM4	_
ENSG00000163823	-3.68859	-2.060656	1	1	CCR1	K04176
ENSG00000163915	-3.68859	-2.060656	1	1	C3orf65	_
ENSG00000164175	-3.68859	-2.060656	1	1	SLC45A2	K15378
ENSG00000164451	-3.68859	-2.060656	1	1	FAM26D	_
ENSG00000164530	-3.68859	-2.060656	1	1	PI16	_
ENSG00000164742	-3.68859	-2.060656	1	1	ADCY1	K08041
ENSG00000165084	-3.68859	-2.060656	1	1	C8orf34	_
ENSG00000165246	-3.68859	-2.060656	1	1	NLGN4Y	K07378
ENSG00000165805	-3.68859	-2.060656	1	1	C12orf50	K08857
ENSG00000165899	-3.68859	-2.060656	1	1	OTOGL	_
ENSG00000165972	-3.68859	-2.060656	1	1	CCDC38	_
ENSG00000166183	-3.68859	-2.060656	1	1	ASPG	K13278
ENSG00000166402	-3.68859	-2.060656	1	1	TUB	_
ENSG00000166825	-3.68859	-2.060656	1	1	ANPEP	K11140
ENSG00000166927	-3.68859	-2.060656	1	1	MS4A7	_
ENSG00000167383	-3.68859	-2.060656	1	1	ZNF229	K09228

ENSG00000167780	-3.68859	-2.060656	1	1	SOAT2	K00637
ENSG00000167874	-3.68859	-2.060656	1	1	TMEM88	K11448
ENSG00000168306	-3.68859	-2.060656	1	1	ACOX2	K10214
ENSG00000168356	-3.68859	-2.060656	1	1	SCN11A	K04843
ENSG00000168454	-3.68859	-2.060656	1	1	TXNDC2	_
ENSG00000168748	-3.68859	-2.060656	1	1	CA7	K01672
ENSG00000170162	-3.68859	-2.060656	1	1	VGLL2	_
ENSG00000170231	-3.68859	-2.060656	1	1	FABP6	K08755
ENSG00000170801	-3.68859	-2.060656	1	1	HTRA3	K08785
ENSG00000171115	-3.68859	-2.060656	1	1	GIMAP8	_
ENSG00000172073	-3.68859	-2.060656	1	1	TEX37	_
ENSG00000172243	-3.68859	-2.060656	1	1	CLEC7A	K10074
ENSG00000172543	-3.68859	-2.060656	1	1	CTSW	K08569
ENSG00000172717	-3.68859	-2.060656	1	1	FAM71D	_
ENSG00000173080	-3.68859	-2.060656	1	1	RXFP4	K08398
ENSG00000173114	-3.68859	-2.060656	1	1	LRRN3	_
ENSG00000173198	-3.68859	-2.060656	1	1	CYSLTR1	K04322
ENSG00000173421	-3.68859	-2.060656	1	1	CCDC36	K12581
ENSG00000175445	-3.68859	-2.060656	1	1	LPL	K01059
ENSG00000176840	-3.68859	-2.060656	1	1	MIR7-3HG	_
ENSG00000177453	-3.68859	-2.060656	1	1	NIM1K	_
ENSG00000177627	-3.68859	-2.060656	1	1	C12orf54	_
ENSG00000177710	-3.68859	-2.060656	1	1	SLC35G5	_
ENSG00000178084	-3.68859	-2.060656	1	1	HTR3C	K04819
ENSG00000179766	-3.68859	-2.060656	1	1	ATP8B5P	K01555
ENSG00000180071	-3.68859	-2.060656	1	1	ANKRD18A	K17299
ENSG00000180878	-3.68859	-2.060656	1	1	C11orf42	_
ENSG00000181323	-3.68859	-2.060656	1	1	SPEM1	_
ENSG00000181358	-3.68859	-2.060656	1	1	CTAGE10P	_
ENSG00000182326	-3.68859	-2.060656	1	1	C1S	K01331
ENSG00000182557	-3.68859	-2.060656	1	1	SPNS3	_
ENSG00000182983	-3.68859	-2.060656	1	1	ZNF662	K09228
ENSG00000183016	-3.68859	-2.060656	1	1	IQCA1P1	_
ENSG00000183396	-3.68859	-2.060656	1	1	TMEM89	_
ENSG00000183678	-3.68859	-2.060656	1	1	CTAG1A	_
ENSG00000183773	-3.68859	-2.060656	1	1	AIFM3	_
ENSG00000184084	-3.68859	-2.060656	1	1	CTD-2372A4.1	_
ENSG00000184206	-3.68859	-2.060656	1	1	GOLGA6L4	K10595
ENSG00000184571	-3.68859	-2.060656	1	1	PIWIL3	K02156
ENSG00000184750	-3.68859	-2.060656	1	1	MAGEA2	_
ENSG00000185162	-3.68859	-2.060656	1	1	AP001324.1	_
ENSG00000185198	-3.68859	-2.060656	1	1	PRSS57	_
ENSG00000185523	-3.68859	-2.060656	1	1	C1orf227	_
ENSG00000187621	-3.68859	-2.060656	1	1	TCL6	_
ENSG00000187763	-3.68859	-2.060656	1	1	OR2B7P	K04257
ENSG00000187905	-3.68859	-2.060656	1	1	AC002472.13	K07374
ENSG00000188133	-3.68859	-2.060656	1	1	TMEM215	_
ENSG00000188365	-3.68859	-2.060656	1	1	AC092171.2	_
ENSG00000188732	-3.68859	-2.060656	1	1	FAM221A	_
ENSG00000188761	-3.68859	-2.060656	1	1	BCL2L15	_
ENSG00000189292	-3.68859	-2.060656	1	1	FAM150B	_
ENSG00000189375	-3.68859	-2.060656	1	1	TBC1D28	K05150
ENSG00000196374	-3.68859	-2.060656	1	1	HIST1H2BM	K11252
ENSG00000196408	-3.68859	-2.060656	1	1	NOXO1	K17934
ENSG00000197180	-3.68859	-2.060656	1	1	BX936347.1	_
ENSG00000197629	-3.68859	-2.060656	1	1	MPEG1	_
ENSG00000197769	-3.68859	-2.060656	1	1	MAP1LC3C	K10435
ENSG00000197977	-3.68859	-2.060656	1	1	ELOVL2	K10205

ENSG00000198121	-3.68859	-2.060656	1	1	LPAR1	K04289
ENSG00000198312	-3.68859	-2.060656	1	1	BMS1P9	K14569
ENSG00000198857	-3.68859	-2.060656	1	1	HSD3BP5	K00070
ENSG00000199331	-3.68859	-2.060656	1	1	Y_RNA	_
ENSG00000199832	-3.68859	-2.060656	1	1	Y_RNA	_
ENSG00000199890	-3.68859	-2.060656	1	1	Y_RNA	_
ENSG00000199933	-3.68859	-2.060656	1	1	Y_RNA	_
ENSG00000199936	-3.68859	-2.060656	1	1	Y_RNA	_
ENSG00000200253	-3.68859	-2.060656	1	1	RNU6-529P	_
ENSG00000200344	-3.68859	-2.060656	1	1	Y_RNA	_
ENSG00000200376	-3.68859	-2.060656	1	1	RNU5E-10P	_
ENSG00000200502	-3.68859	-2.060656	1	1	Y_RNA	_
ENSG00000200674	-3.68859	-2.060656	1	1	RN7SKP160	_
ENSG00000200922	-3.68859	-2.060656	1	1	Y_RNA	_
ENSG00000201592	-3.68859	-2.060656	1	1	snoU2_19	_
ENSG00000201724	-3.68859	-2.060656	1	1	Y_RNA	_
ENSG00000202343	-3.68859	-2.060656	1	1	SNORA2	_
ENSG00000202515	-3.68859	-2.060656	1	1	VTRNA1-3	_
ENSG00000203356	-3.68859	-2.060656	1	1	RP11-296A18.3	_
ENSG00000203426	-3.68859	-2.060656	1	1	AC140061.7	K17985
ENSG00000203785	-3.68859	-2.060656	1	1	SPRR2E	_
ENSG00000203900	-3.68859	-2.060656	1	1	RP11-261N11.8	_
ENSG00000204010	-3.68859	-2.060656	1	1	IFIT1B	K14217
ENSG00000204152	-3.68859	-2.060656	1	1	TIMM23B	K17794
ENSG00000204652	-3.68859	-2.060656	1	1	RPS26P8	K02976
ENSG00000204653	-3.68859	-2.060656	1	1	ASPDH	K16360
ENSG00000204837	-3.68859	-2.060656	1	1	RP11-204M4.2	_
ENSG00000205089	-3.68859	-2.060656	1	1	CCNI2	_
ENSG00000205147	-3.68859	-2.060656	1	1	AC016586.1	K12581
ENSG00000205209	-3.68859	-2.060656	1	1	SCGB2B2	_
ENSG00000205420	-3.68859	-2.060656	1	1	KRT6A	K07605
ENSG00000205784	-3.68859	-2.060656	1	1	ARRDC5	K12756
ENSG00000205890	-3.68859	-2.060656	1	1	RP11-473M20.5	_
ENSG00000205922	-3.68859	-2.060656	1	1	ONECUT3	K08026
ENSG00000206142	-3.68859	-2.060656	1	1	FAM230C	_
ENSG00000206612	-3.68859	-2.060656	1	1	SNORA2A	_
ENSG00000206640	-3.68859	-2.060656	1	1	Y_RNA	_
ENSG00000206651	-3.68859	-2.060656	1	1	Y_RNA	_
ENSG00000206747	-3.68859	-2.060656	1	1	RNU6-245P	_
ENSG00000206808	-3.68859	-2.060656	1	1	Y_RNA	_
ENSG00000206832	-3.68859	-2.060656	1	1	RNU6V	_
ENSG00000206913	-3.68859	-2.060656	1	1	SNORA7	_
ENSG00000207217	-3.68859	-2.060656	1	1	SNORA42	_
ENSG00000207313	-3.68859	-2.060656	1	1	SNORA2B	_
ENSG00000207342	-3.68859	-2.060656	1	1	Y_RNA	_
ENSG00000207419	-3.68859	-2.060656	1	1	SNORA67	_
ENSG00000207438	-3.68859	-2.060656	1	1	Y_RNA	_
ENSG00000207450	-3.68859	-2.060656	1	1	Y_RNA	_
ENSG00000207568	-3.68859	-2.060656	1	1	MIR203	_
ENSG00000207698	-3.68859	-2.060656	1	1	MIR32	_
ENSG00000207975	-3.68859	-2.060656	1	1	MIR181B1	_
ENSG00000211451	-3.68859	-2.060656	1	1	GNRHR2	K04280
ENSG00000212402	-3.68859	-2.060656	1	1	SNORA74B	_
ENSG00000212710	-3.68859	-2.060656	1	1	CTAGE1	_
ENSG00000213014	-3.68859	-2.060656	1	1	VN2R17P	_
ENSG00000213471	-3.68859	-2.060656	1	1	TTLL13	K16582
ENSG00000213763	-3.68859	-2.060656	1	1	ACTBP2	K05692
ENSG00000214433	-3.68859	-2.060656	1	1	AC091167.3	K10595

ENSG00000214559	-3.68859	-2.060656	1	1	RP11-323J4.1	_
ENSG00000215102	-3.68859	-2.060656	1	1	TERF1P4	K11110
ENSG00000215199	-3.68859	-2.060656	1	1	YWHAZP6	K16197
ENSG00000215630	-3.68859	-2.060656	1	1	GUSBP9	K01195
ENSG00000215808	-3.68859	-2.060656	1	1	LINC01139	_
ENSG00000215912	-3.68859	-2.060656	1	1	TTC34	_
ENSG00000216412	-3.68859	-2.060656	1	1	RPL12P2	K02870
ENSG00000216867	-3.68859	-2.060656	1	1	AC114776.1	K02891
ENSG00000216921	-3.68859	-2.060656	1	1	AC131097.4	_
ENSG00000217644	-3.68859	-2.060656	1	1	RP4-803A2.1	K02882
ENSG00000217769	-3.68859	-2.060656	1	1	RP11-76H14.2	K09560
ENSG00000218416	-3.68859	-2.060656	1	1	AC110619.2	_
ENSG00000218631	-3.68859	-2.060656	1	1	RP3-395C13.1	_
ENSG00000218732	-3.68859	-2.060656	1	1	RP5-1046G13.2	K09400
ENSG00000219433	-3.68859	-2.060656	1	1	RP11-350J20.4	K10482
ENSG00000219553	-3.68859	-2.060656	1	1	RP1-281H8.3	_
ENSG00000219747	-3.68859	-2.060656	1	1	RP1-292B18.1	K02912
ENSG00000219755	-3.68859	-2.060656	1	1	RP1-199J3.5	K02985
ENSG00000220925	-3.68859	-2.060656	1	1	IGBP1-AS2	_
ENSG00000221338	-3.68859	-2.060656	1	1	AC108456.1	_
ENSG00000221487	-3.68859	-2.060656	1	1	AC069294.1	_
ENSG00000221716	-3.68859	-2.060656	1	1	SNORA11	_
ENSG00000222378	-3.68859	-2.060656	1	1	RNA5SP44	_
ENSG00000222448	-3.68859	-2.060656	1	1	RN7SKP150	_
ENSG00000223485	-3.68859	-2.060656	1	1	RP11-417E7.1	_
ENSG00000223528	-3.68859	-2.060656	1	1	RP11-223P11.3	_
ENSG00000223571	-3.68859	-2.060656	1	1	DHRSX-IT1	_
ENSG00000223617	-3.68859	-2.060656	1	1	LINC00370	_
ENSG00000223669	-3.68859	-2.060656	1	1	RP11-93B14.4	_
ENSG00000223722	-3.68859	-2.060656	1	1	RP11-467L13.5	K06566
ENSG00000223935	-3.68859	-2.060656	1	1	AC008074.3	_
ENSG00000224014	-3.68859	-2.060656	1	1	RP11-488L18.6	K12160
ENSG00000224376	-3.68859	-2.060656	1	1	AC017104.6	_
ENSG00000224431	-3.68859	-2.060656	1	1	AC063976.7	K17985
ENSG00000224479	-3.68859	-2.060656	1	1	AC136289.1	_
ENSG00000224745	-3.68859	-2.060656	1	1	RP11-380G5.2	_
ENSG00000224965	-3.68859	-2.060656	1	1	KCNC4-AS1	_
ENSG00000224984	-3.68859	-2.060656	1	1	RP11-524H19.2	_
ENSG00000224985	-3.68859	-2.060656	1	1	RP11-297K8.2	_
ENSG00000225195	-3.68859	-2.060656	1	1	RP11-338E21.2	_
ENSG00000225362	-3.68859	-2.060656	1	1	CT62	_
ENSG00000225392	-3.68859	-2.060656	1	1	AC016700.4	K02919
ENSG00000225555	-3.68859	-2.060656	1	1	AP000320.6	_
ENSG00000225572	-3.68859	-2.060656	1	1	DOCK4-AS1	_
ENSG00000225946	-3.68859	-2.060656	1	1	RP11-395B7.2	_
ENSG00000225964	-3.68859	-2.060656	1	1	AC017076.5	_
ENSG00000226650	-3.68859	-2.060656	1	1	KIF4B	K10395
ENSG00000226694	-3.68859	-2.060656	1	1	RP11-69C17.2	_
ENSG00000226859	-3.68859	-2.060656	1	1	RP11-48F14.2	_
ENSG00000226965	-3.68859	-2.060656	1	1	AC003088.1	_
ENSG00000227082	-3.68859	-2.060656	1	1	AL592494.5	_
ENSG00000227163	-3.68859	-2.060656	1	1	RP5-1198O20.5	K12581
ENSG00000227197	-3.68859	-2.060656	1	1	AC009518.8	_
ENSG00000227237	-3.68859	-2.060656	1	1	AL672294.1	_
ENSG00000227347	-3.68859	-2.060656	1	1	HNRNPKP2	K12886
ENSG00000227459	-3.68859	-2.060656	1	1	AC079612.2	_
ENSG00000227479	-3.68859	-2.060656	1	1	AC124861.1	_
ENSG00000227934	-3.68859	-2.060656	1	1	RP11-423F24.3	_

ENSG00000227999	-3.68859	-2.060656	1	1	MTND5P1	_
ENSG00000228010	-3.68859	-2.060656	1	1	AC073343.13	_
ENSG00000228366	-3.68859	-2.060656	1	1	RP11-18B3.3	K12900
ENSG00000228369	-3.68859	-2.060656	1	1	TXNDC12-AS1	_
ENSG00000228420	-3.68859	-2.060656	1	1	RP4-735C1.6	_
ENSG00000228717	-3.68859	-2.060656	1	1	AF013593.1	_
ENSG00000228766	-3.68859	-2.060656	1	1	RPL7L1P8	_
ENSG00000228793	-3.68859	-2.060656	1	1	RP1-223B1.1	_
ENSG00000228798	-3.68859	-2.060656	1	1	AP000473.5	_
ENSG00000229082	-3.68859	-2.060656	1	1	OR5AW1P	K04257
ENSG00000229204	-3.68859	-2.060656	1	1	PTGES3P3	K15730
ENSG00000229278	-3.68859	-2.060656	1	1	RP11-483F11.7	_
ENSG00000229298	-3.68859	-2.060656	1	1	TUBB8P1	K07375
ENSG00000229382	-3.68859	-2.060656	1	1	BX322557.13	_
ENSG00000229563	-3.68859	-2.060656	1	1	RP11-245M24.1	_
ENSG00000229567	-3.68859	-2.060656	1	1	RP4-717I23.2	K02924
ENSG00000229598	-3.68859	-2.060656	1	1	PRDX3P1	K03386
ENSG00000229750	-3.68859	-2.060656	1	1	AC096649.2	_
ENSG00000229862	-3.68859	-2.060656	1	1	RP11-505P4.7	_
ENSG00000229983	-3.68859	-2.060656	1	1	RP11-15I11.2	_
ENSG00000229992	-3.68859	-2.060656	1	1	HMGB3P9	K11296
ENSG00000230037	-3.68859	-2.060656	1	1	UBBP1	K04551
ENSG00000230109	-3.68859	-2.060656	1	1	RP11-275N1.1	_
ENSG00000230204	-3.68859	-2.060656	1	1	FTH1P5	K00522
ENSG00000230251	-3.68859	-2.060656	1	1	PHBP4	K17080
ENSG00000230257	-3.68859	-2.060656	1	1	NFE4	_
ENSG00000230260	-3.68859	-2.060656	1	1	RP11-75C23.1	_
ENSG00000230358	-3.68859	-2.060656	1	1	RP11-166O4.1	K08694
ENSG00000230479	-3.68859	-2.060656	1	1	AP000695.6	_
ENSG00000230778	-3.68859	-2.060656	1	1	ANKRD63	_
ENSG00000230793	-3.68859	-2.060656	1	1	SMARCE1P5	K11651
ENSG00000230882	-3.68859	-2.060656	1	1	AC005077.14	_
ENSG00000230943	-3.68859	-2.060656	1	1	RP11-367G18.1	_
ENSG00000230946	-3.68859	-2.060656	1	1	HNRNPA1P68	K12741
ENSG00000231134	-3.68859	-2.060656	1	1	TCF7L1-IT1	_
ENSG00000231333	-3.68859	-2.060656	1	1	RPL34P6	K02915
ENSG00000231459	-3.68859	-2.060656	1	1	LINC00032	_
ENSG00000231665	-3.68859	-2.060656	1	1	OGFOD1P1	_
ENSG00000231845	-3.68859	-2.060656	1	1	HMGB3P14	K11296
ENSG00000232022	-3.68859	-2.060656	1	1	RP5-1109J22.1	K15528
ENSG00000232124	-3.68859	-2.060656	1	1	AP001057.1	_
ENSG00000232352	-3.68859	-2.060656	1	1	SEMA3B-AS1	_
ENSG00000232451	-3.68859	-2.060656	1	1	AC016768.1	_
ENSG00000232456	-3.68859	-2.060656	1	1	RP11-5P18.10	_
ENSG00000232519	-3.68859	-2.060656	1	1	RP11-29H23.4	_
ENSG00000232608	-3.68859	-2.060656	1	1	TIMM9P2	K17777
ENSG00000232645	-3.68859	-2.060656	1	1	RP3-322G13.5	_
ENSG00000232650	-3.68859	-2.060656	1	1	RP5-834N19.1	_
ENSG00000232693	-3.68859	-2.060656	1	1	AC012370.2	_
ENSG00000232768	-3.68859	-2.060656	1	1	RP11-201O14.2	K02980
ENSG00000233117	-3.68859	-2.060656	1	1	LINC00702	_
ENSG00000233186	-3.68859	-2.060656	1	1	RP11-307I14.3	K17846
ENSG00000233205	-3.68859	-2.060656	1	1	AC108479.2	_
ENSG00000233219	-3.68859	-2.060656	1	1	RP11-89N17.3	_
ENSG00000233347	-3.68859	-2.060656	1	1	ERP29P1	K09586
ENSG00000233478	-3.68859	-2.060656	1	1	RP1-187B23.1	_
ENSG00000233558	-3.68859	-2.060656	1	1	RP3-486I3.4	K02989
ENSG00000233602	-3.68859	-2.060656	1	1	ERI3-IT1	_

ENSG00000233956	-3.68859	-2.060656	1	1	BTF3P6	K01527
ENSG00000234203	-3.68859	-2.060656	1	1	RP5-1050D4.2	_
ENSG00000234245	-3.68859	-2.060656	1	1	RP11-94I2.4	_
ENSG00000234319	-3.68859	-2.060656	1	1	RPS12P2	K02951
ENSG00000234336	-3.68859	-2.060656	1	1	JAZF1-AS1	_
ENSG00000234384	-3.68859	-2.060656	1	1	LINC01049	_
ENSG00000234405	-3.68859	-2.060656	1	1	LL0XNC01-250H12	_
ENSG00000234523	-3.68859	-2.060656	1	1	NDUFB1P2	K03957
ENSG00000234699	-3.68859	-2.060656	1	1	RP11-225H22.4	_
ENSG00000234772	-3.68859	-2.060656	1	1	LINC00412	_
ENSG00000234801	-3.68859	-2.060656	1	1	MORF4	K11339
ENSG00000235062	-3.68859	-2.060656	1	1	BCRP5	K08878
ENSG00000235123	-3.68859	-2.060656	1	1	DSCAM-AS1	_
ENSG00000235156	-3.68859	-2.060656	1	1	TMEM30C	_
ENSG00000235180	-3.68859	-2.060656	1	1	LINC00601	_
ENSG00000235376	-3.68859	-2.060656	1	1	RPEL1	K01783
ENSG00000235426	-3.68859	-2.060656	1	1	RP11-342M3.5	_
ENSG00000235574	-3.68859	-2.060656	1	1	AC073150.6	K14822
ENSG00000235605	-3.68859	-2.060656	1	1	RP5-827C21.1	K02958
ENSG00000235701	-3.68859	-2.060656	1	1	PCBP2P1	K13162
ENSG00000235740	-3.68859	-2.060656	1	1	RP11-436I24.1	_
ENSG00000235786	-3.68859	-2.060656	1	1	ZNRF3-IT1	_
ENSG00000235907	-3.68859	-2.060656	1	1	RP4-580O19.2	K02727
ENSG00000235908	-3.68859	-2.060656	1	1	RHOA-IT1	_
ENSG00000236024	-3.68859	-2.060656	1	1	RP11-483H20.6	_
ENSG00000236048	-3.68859	-2.060656	1	1	AC013470.6	K11090
ENSG00000236327	-3.68859	-2.060656	1	1	RP11-759F5.1	_
ENSG00000236545	-3.68859	-2.060656	1	1	AP001619.3	_
ENSG00000236554	-3.68859	-2.060656	1	1	ASNSP3	K01953
ENSG00000236655	-3.68859	-2.060656	1	1	AC023347.2	K05038
ENSG00000237233	-3.68859	-2.060656	1	1	RP11-809M12.1	_
ENSG00000237418	-3.68859	-2.060656	1	1	AC069257.6	K02924
ENSG00000237609	-3.68859	-2.060656	1	1	AF064858.10	_
ENSG00000237714	-3.68859	-2.060656	1	1	P4HA2-AS1	_
ENSG00000237870	-3.68859	-2.060656	1	1	AC073130.1	_
ENSG00000238055	-3.68859	-2.060656	1	1	RP1-128O3.6	K02929
ENSG00000238059	-3.68859	-2.060656	1	1	HSPE1P21	K04078
ENSG00000238171	-3.68859	-2.060656	1	1	AC068196.1	_
ENSG00000238251	-3.68859	-2.060656	1	1	RP11-172F4.2	K14842
ENSG00000238258	-3.68859	-2.060656	1	1	RP11-342D11.2	_
ENSG00000238583	-3.68859	-2.060656	1	1	snoU13	_
ENSG00000238665	-3.68859	-2.060656	1	1	snoU13	_
ENSG00000238824	-3.68859	-2.060656	1	1	snoU13	_
ENSG00000239300	-3.68859	-2.060656	1	1	RP11-400L8.2	_
ENSG00000239393	-3.68859	-2.060656	1	1	CTD-2301A4.1	K02969
ENSG00000239912	-3.68859	-2.060656	1	1	RPL39P36	K02924
ENSG00000239948	-3.68859	-2.060656	1	1	RN7SL368P	_
ENSG00000240455	-3.68859	-2.060656	1	1	RN7SL671P	_
ENSG00000240463	-3.68859	-2.060656	1	1	RPS19P3	K02966
ENSG00000240497	-3.68859	-2.060656	1	1	RP11-185E8.1	_
ENSG00000240522	-3.68859	-2.060656	1	1	RPL7AP10	K02936
ENSG00000240724	-3.68859	-2.060656	1	1	RPS12P15	_
ENSG00000241217	-3.68859	-2.060656	1	1	RN7SL809P	_
ENSG00000241352	-3.68859	-2.060656	1	1	RP11-392P7.1	K02991
ENSG00000241464	-3.68859	-2.060656	1	1	RPL39P38	K02924
ENSG00000241520	-3.68859	-2.060656	1	1	AC098820.4	_
ENSG00000241556	-3.68859	-2.060656	1	1	RP11-490G8.1	K02905
ENSG00000241635	-3.68859	-2.060656	1	1	UGT1A8	K00699

ENSG00000241745	-3.68859	-2.060656	1	1	RN7SL788P	_
ENSG00000241770	-3.68859	-2.060656	1	1	RP11-555M1.3	_
ENSG00000241818	-3.68859	-2.060656	1	1	RP11-1000B6.2	_
ENSG00000242095	-3.68859	-2.060656	1	1	RN7SL113P	_
ENSG00000242375	-3.68859	-2.060656	1	1	RP11-498P14.3	_
ENSG00000242412	-3.68859	-2.060656	1	1	DBIL5P2	_
ENSG00000242951	-3.68859	-2.060656	1	1	RP11-507E23.1	K02974
ENSG00000242992	-3.68859	-2.060656	1	1	FTH1P4	K00522
ENSG00000243339	-3.68859	-2.060656	1	1	RN7SL738P	_
ENSG00000243637	-3.68859	-2.060656	1	1	AC019221.4	_
ENSG00000243672	-3.68859	-2.060656	1	1	RPS3AP40	K02984
ENSG00000244040	-3.68859	-2.060656	1	1	IL12A-AS1	_
ENSG00000244137	-3.68859	-2.060656	1	1	RP11-99J16__A.2	_
ENSG00000246016	-3.68859	-2.060656	1	1	RP11-1C1.7	_
ENSG00000247774	-3.68859	-2.060656	1	1	PCED1B-AS1	_
ENSG00000248234	-3.68859	-2.060656	1	1	CTD-2108O9.4	K02901
ENSG00000248254	-3.68859	-2.060656	1	1	AC092597.3	_
ENSG00000248515	-3.68859	-2.060656	1	1	RP11-608O21.1	_
ENSG00000248590	-3.68859	-2.060656	1	1	GLDCP1	K00281
ENSG00000248696	-3.68859	-2.060656	1	1	AC011406.2	_
ENSG00000248713	-3.68859	-2.060656	1	1	RP11-766F14.2	_
ENSG00000248746	-3.68859	-2.060656	1	1	ACTN3	K05699
ENSG00000248996	-3.68859	-2.060656	1	1	RP11-1334A24.6	_
ENSG00000249006	-3.68859	-2.060656	1	1	RP11-317B7.2	K14416
ENSG00000249085	-3.68859	-2.060656	1	1	CTD-2631K10.1	_
ENSG00000249140	-3.68859	-2.060656	1	1	PRDX2P3	K03386
ENSG00000249212	-3.68859	-2.060656	1	1	ATP1B1P1	K01540
ENSG00000249240	-3.68859	-2.060656	1	1	AC069368.3	_
ENSG00000249286	-3.68859	-2.060656	1	1	CTD-2210P15.2	K01611
ENSG00000249304	-3.68859	-2.060656	1	1	RP11-26P13.2	_
ENSG00000249378	-3.68859	-2.060656	1	1	LINC01060	_
ENSG00000249526	-3.68859	-2.060656	1	1	CTB-35F21.1	_
ENSG00000249532	-3.68859	-2.060656	1	1	MIR302B	_
ENSG00000249593	-3.68859	-2.060656	1	1	CTB-46B19.2	_
ENSG00000249602	-3.68859	-2.060656	1	1	RP11-98D18.3	_
ENSG00000249655	-3.68859	-2.060656	1	1	CTC-325J23.2	_
ENSG00000249691	-3.68859	-2.060656	1	1	RP11-445J14.1	_
ENSG00000249898	-3.68859	-2.060656	1	1	CTD-2541M15.1	_
ENSG00000250734	-3.68859	-2.060656	1	1	RP11-404E16.1	_
ENSG00000251131	-3.68859	-2.060656	1	1	CTD-2035E11.3	_
ENSG00000251155	-3.68859	-2.060656	1	1	RP11-384K6.4	K16941
ENSG00000251537	-3.68859	-2.060656	1	1	RP11-385D13.1	K10266
ENSG00000251556	-3.68859	-2.060656	1	1	RP11-118M9.3	_
ENSG00000251576	-3.68859	-2.060656	1	1	RP11-536I6.1	_
ENSG00000251598	-3.68859	-2.060656	1	1	RP11-789C2.1	_
ENSG00000251667	-3.68859	-2.060656	1	1	RP11-844P9.3	K11864
ENSG00000251739	-3.68859	-2.060656	1	1	RNU6-1053P	_
ENSG00000251806	-3.68859	-2.060656	1	1	SNORD119	_
ENSG00000252106	-3.68859	-2.060656	1	1	Y_RNA	_
ENSG00000252334	-3.68859	-2.060656	1	1	RNU6-1337P	_
ENSG00000252390	-3.68859	-2.060656	1	1	RNU5F-4P	_
ENSG00000252464	-3.68859	-2.060656	1	1	RN7SKP70	_
ENSG00000252498	-3.68859	-2.060656	1	1	RNU6-1016P	_
ENSG00000252643	-3.68859	-2.060656	1	1	RNU6-1136P	_
ENSG00000252896	-3.68859	-2.060656	1	1	AC067957.1	_
ENSG00000252933	-3.68859	-2.060656	1	1	RNU6-1223P	_
ENSG00000252942	-3.68859	-2.060656	1	1	RNA5SP290	_
ENSG00000253054	-3.68859	-2.060656	1	1	RNU7-77P	_

ENSG00000253154	-3.68859	-2.060656	1	1	CTA-392E5.1	_
ENSG00000253516	-3.68859	-2.060656	1	1	HMGB1P41	K10802
ENSG00000253774	-3.68859	-2.060656	1	1	CTD-3023L14.1	_
ENSG00000253898	-3.68859	-2.060656	1	1	RP11-51M18.1	_
ENSG00000253972	-3.68859	-2.060656	1	1	RP11-4K16.2	_
ENSG00000254063	-3.68859	-2.060656	1	1	DUXAP2	_
ENSG00000254226	-3.68859	-2.060656	1	1	CTB-12O2.1	_
ENSG00000254339	-3.68859	-2.060656	1	1	RP11-267L5.1	_
ENSG00000254503	-3.68859	-2.060656	1	1	CTD-2521M24.4	K12581
ENSG00000254671	-3.68859	-2.060656	1	1	STT3A-AS1	_
ENSG00000254739	-3.68859	-2.060656	1	1	RP13-46H24.1	_
ENSG00000254852	-3.68859	-2.060656	1	1	NPIPA2	K01896
ENSG00000254985	-3.68859	-2.060656	1	1	RSF1-IT2	_
ENSG00000255108	-3.68859	-2.060656	1	1	AP006621.8	_
ENSG00000255124	-3.68859	-2.060656	1	1	RP11-680F20.5	_
ENSG00000255169	-3.68859	-2.060656	1	1	RP11-646J21.3	_
ENSG00000255237	-3.68859	-2.060656	1	1	RP13-317D12.3	_
ENSG00000255322	-3.68859	-2.060656	1	1	RP11-22P4.1	_
ENSG00000255477	-3.68859	-2.060656	1	1	RP11-63D14.1	_
ENSG00000255572	-3.68859	-2.060656	1	1	RP11-273B20.3	_
ENSG00000255647	-3.68859	-2.060656	1	1	AC093510.1	_
ENSG00000255748	-3.68859	-2.060656	1	1	AC226150.4	_
ENSG00000255823	-3.68859	-2.060656	1	1	MTRNR2L8	K01982
ENSG00000255836	-3.68859	-2.060656	1	1	RP11-157G21.2	_
ENSG00000255933	-3.68859	-2.060656	1	1	RP11-495K9.5	_
ENSG00000255970	-3.68859	-2.060656	1	1	RP11-43N5.1	_
ENSG00000256039	-3.68859	-2.060656	1	1	RP11-291B21.2	_
ENSG00000256155	-3.68859	-2.060656	1	1	RP11-277P12.9	_
ENSG00000256356	-3.68859	-2.060656	1	1	HSPA8P5	K03283
ENSG00000256538	-3.68859	-2.060656	1	1	RP11-847H18.3	_
ENSG00000257184	-3.68859	-2.060656	1	1	RP1-170O19.20	K09294
ENSG00000257501	-3.68859	-2.060656	1	1	RP11-1016B18.1	_
ENSG00000257569	-3.68859	-2.060656	1	1	GSTP1P1	K00799
ENSG00000257624	-3.68859	-2.060656	1	1	RP1-128M12.3	_
ENSG00000257735	-3.68859	-2.060656	1	1	RP11-370I10.6	_
ENSG00000258128	-3.68859	-2.060656	1	1	MKRN9P	K15687
ENSG00000258343	-3.68859	-2.060656	1	1	RP11-536G4.2	_
ENSG00000258365	-3.68859	-2.060656	1	1	RP11-1105G2.3	_
ENSG00000258684	-3.68859	-2.060656	1	1	BMS1P16	K14569
ENSG00000258733	-3.68859	-2.060656	1	1	CTD-2341M24.1	_
ENSG00000258760	-3.68859	-2.060656	1	1	CTD-2509G16.5	_
ENSG00000258938	-3.68859	-2.060656	1	1	RP11-317N8.5	_
ENSG00000258959	-3.68859	-2.060656	1	1	RP11-1017G21.4	_
ENSG00000259079	-3.68859	-2.060656	1	1	RP1-261D10.1	_
ENSG00000259083	-3.68859	-2.060656	1	1	RP11-407N17.4	_
ENSG00000259262	-3.68859	-2.060656	1	1	NDUFA3P4	K03947
ENSG00000259348	-3.68859	-2.060656	1	1	RP11-4G2.1	_
ENSG00000259358	-3.68859	-2.060656	1	1	RP11-456J20.1	_
ENSG00000259371	-3.68859	-2.060656	1	1	RP11-468E2.6	K11801
ENSG00000259402	-3.68859	-2.060656	1	1	RP11-30K9.4	_
ENSG00000259407	-3.68859	-2.060656	1	1	RP11-158M2.3	_
ENSG00000259415	-3.68859	-2.060656	1	1	RP11-7M10.2	_
ENSG00000259441	-3.68859	-2.060656	1	1	MRPL15P1	K02876
ENSG00000259467	-3.68859	-2.060656	1	1	NDUFAF4P1	K18161
ENSG00000259480	-3.68859	-2.060656	1	1	RP11-26F2.1	K12200
ENSG00000259532	-3.68859	-2.060656	1	1	RP11-138H8.2	_
ENSG00000259780	-3.68859	-2.060656	1	1	RP11-304L19.12	_
ENSG00000259882	-3.68859	-2.060656	1	1	RP11-293B20.2	_

ENSG00000259985	-3.68859	-2.060656	1	1	RP11-549B18.1	_
ENSG00000260035	-3.68859	-2.060656	1	1	CTD-2651B20.6	_
ENSG00000260198	-3.68859	-2.060656	1	1	RP11-441F2.2	_
ENSG00000260281	-3.68859	-2.060656	1	1	RP11-329J18.2	_
ENSG00000260350	-3.68859	-2.060656	1	1	RP11-152P23.2	_
ENSG00000260439	-3.68859	-2.060656	1	1	LA16c-366D3.1	_
ENSG00000260475	-3.68859	-2.060656	1	1	RP11-85A1.3	_
ENSG00000260509	-3.68859	-2.060656	1	1	RP11-271M24.2	_
ENSG00000260729	-3.68859	-2.060656	1	1	RP11-106M3.2	_
ENSG00000260871	-3.68859	-2.060656	1	1	CTD-2373J6.1	_
ENSG00000261019	-3.68859	-2.060656	1	1	RP11-111K18.2	_
ENSG00000261064	-3.68859	-2.060656	1	1	RP11-1000B6.3	_
ENSG00000261141	-3.68859	-2.060656	1	1	RP11-303E16.5	_
ENSG00000261191	-3.68859	-2.060656	1	1	RP11-16L14.2	_
ENSG00000261240	-3.68859	-2.060656	1	1	RP11-304L19.4	_
ENSG00000261317	-3.68859	-2.060656	1	1	RP11-566K11.5	_
ENSG00000261465	-3.68859	-2.060656	1	1	RP11-626G11.5	_
ENSG00000261469	-3.68859	-2.060656	1	1	RP11-96D1.6	_
ENSG00000261476	-3.68859	-2.060656	1	1	RP11-77K12.3	_
ENSG00000261481	-3.68859	-2.060656	1	1	RP11-77H9.6	_
ENSG00000261654	-3.68859	-2.060656	1	1	RP11-96K19.4	_
ENSG00000261783	-3.68859	-2.060656	1	1	RP11-252K23.2	_
ENSG00000261827	-3.68859	-2.060656	1	1	RP11-340F14.5	_
ENSG00000262165	-3.68859	-2.060656	1	1	RP11-81A22.5	_
ENSG00000262223	-3.68859	-2.060656	1	1	RP11-1055B8.3	_
ENSG00000262372	-3.68859	-2.060656	1	1	RP11-669E14.6	_
ENSG00000262583	-3.68859	-2.060656	1	1	RP11-77K12.5	K19362
ENSG00000262732	-3.68859	-2.060656	1	1	CTD-2135D7.2	_
ENSG00000263051	-3.68859	-2.060656	1	1	RP11-565F19.3	_
ENSG00000263220	-3.68859	-2.060656	1	1	RP11-420A6.2	_
ENSG00000263660	-3.68859	-2.060656	1	1	AC087793.1	_
ENSG00000263931	-3.68859	-2.060656	1	1	RP11-180P8.1	_
ENSG00000264067	-3.68859	-2.060656	1	1	RP11-401O9.3	_
ENSG00000264176	-3.68859	-2.060656	1	1	MAGOH2	K12877
ENSG00000264265	-3.68859	-2.060656	1	1	RP11-14P20.1	_
ENSG00000264324	-3.68859	-2.060656	1	1	RP11-287D1.3	K13857
ENSG00000264349	-3.68859	-2.060656	1	1	MIR4258	_
ENSG00000264508	-3.68859	-2.060656	1	1	RN7SL473P	_
ENSG00000264520	-3.68859	-2.060656	1	1	RP4-777O23.2	_
ENSG00000264812	-3.68859	-2.060656	1	1	RP13-20L14.4	_
ENSG00000264931	-3.68859	-2.060656	1	1	MIR3138	_
ENSG00000265321	-3.68859	-2.060656	1	1	MIR4263	_
ENSG00000265403	-3.68859	-2.060656	1	1	AC015884.1	_
ENSG00000265528	-3.68859	-2.060656	1	1	AC092316.2	_
ENSG00000265574	-3.68859	-2.060656	1	1	CTD-2095E4.1	_
ENSG00000265943	-3.68859	-2.060656	1	1	RP11-739L10.1	_
ENSG00000266098	-3.68859	-2.060656	1	1	AL137127.1	_
ENSG00000266126	-3.68859	-2.060656	1	1	RP11-209D14.4	_
ENSG00000266164	-3.68859	-2.060656	1	1	RN7SL758P	_
ENSG00000266542	-3.68859	-2.060656	1	1	MIR5572	_
ENSG00000266843	-3.68859	-2.060656	1	1	AL360007.1	_
ENSG00000266941	-3.68859	-2.060656	1	1	AC104532.3	_
ENSG00000266942	-3.68859	-2.060656	1	1	RP11-378E13.3	_
ENSG00000266980	-3.68859	-2.060656	1	1	RP11-552F3.4	_
ENSG00000267092	-3.68859	-2.060656	1	1	CTB-25B13.9	_
ENSG00000267117	-3.68859	-2.060656	1	1	AC010525.4	_
ENSG00000267302	-3.68859	-2.060656	1	1	RP11-178C3.2	_
ENSG00000267385	-3.68859	-2.060656	1	1	CTB-50L17.14	K04613

ENSG00000267470	-3.68859	-2.060656	1	1	ZNF571-AS1	_
ENSG00000267476	-3.68859	-2.060656	1	1	RP11-126O1.4	_
ENSG00000267551	-3.68859	-2.060656	1	1	AC005264.2	_
ENSG00000267605	-3.68859	-2.060656	1	1	CTD-3220F14.1	_
ENSG00000267654	-3.68859	-2.060656	1	1	RP11-973H7.4	_
ENSG00000267711	-3.68859	-2.060656	1	1	RP11-686D22.5	_
ENSG00000267852	-3.68859	-2.060656	1	1	CTB-133G6.2	_
ENSG00000267868	-3.68859	-2.060656	1	1	RP11-120K24.3	_
ENSG00000267874	-3.68859	-2.060656	1	1	CTD-2527I21.9	_
ENSG00000267934	-3.68859	-2.060656	1	1	CTB-176F20.3	_
ENSG00000268173	-3.68859	-2.060656	1	1	PIK3R2	K02649
ENSG00000268302	-3.68859	-2.060656	1	1	AL035252.1	_
ENSG00000268466	-3.68859	-2.060656	1	1	AL132989.1	_
ENSG00000268550	-3.68859	-2.060656	1	1	AC090427.1	_
ENSG00000268751	-3.68859	-2.060656	1	1	SCGB1B2P	_
ENSG00000268788	-3.68859	-2.060656	1	1	AC110615.1	K14856
ENSG00000268838	-3.68859	-2.060656	1	1	AC027228.1	_
ENSG00000269097	-3.68859	-2.060656	1	1	AC003682.17	_
ENSG00000269153	-3.68859	-2.060656	1	1	CTD-3193O13.10	K06130
ENSG00000269431	-3.68859	-2.060656	1	1	CTB-175P5.1	K15464
ENSG00000269534	-3.68859	-2.060656	1	1	CTC-453G23.5	_
ENSG00000269652	-3.68859	-2.060656	1	1	CTD-2195B23.3	_
ENSG00000269890	-3.68859	-2.060656	1	1	RP5-1139B12.2	_
ENSG00000269919	-3.68859	-2.060656	1	1	RP1-134E15.3	_
ENSG00000269938	-3.68859	-2.060656	1	1	RP11-214K3.20	_
ENSG00000270127	-3.68859	-2.060656	1	1	RP11-526I2.5	_
ENSG00000270154	-3.68859	-2.060656	1	1	RP11-419I17.1	_
ENSG00000270540	-3.68859	-2.060656	1	1	RP11-785G17.1	_
ENSG00000270554	-3.68859	-2.060656	1	1	RP11-225N10.3	_
ENSG00000270772	-3.68859	-2.060656	1	1	RP11-332H21.2	K00161
ENSG00000270911	-3.68859	-2.060656	1	1	RP11-272L13.4	_
ENSG00000270917	-3.68859	-2.060656	1	1	RP11-27I1.6	_
ENSG00000270951	-3.68859	-2.060656	1	1	RP1-309F20.4	_
ENSG00000271011	-3.68859	-2.060656	1	1	RP11-171I2.5	_
ENSG00000271018	-3.68859	-2.060656	1	1	RP11-60E8.2	K17784
ENSG00000271237	-3.68859	-2.060656	1	1	RP11-140C5.3	_
ENSG00000271324	-3.68859	-2.060656	1	1	RP11-10C24.2	_
ENSG00000271387	-3.68859	-2.060656	1	1	RP11-382D12.2	_
ENSG00000271557	-3.68859	-2.060656	1	1	RP11-692M12.4	_
ENSG00000271806	-3.68859	-2.060656	1	1	RP5-892K4.1	_
ENSG00000271849	-3.68859	-2.060656	1	1	CTC-332L22.1	_
ENSG00000271924	-3.68859	-2.060656	1	1	5S_rRNA	_
ENSG00000271926	-3.68859	-2.060656	1	1	CTD-2376I4.1	_
ENSG00000271970	-3.68859	-2.060656	1	1	RP3-337H4.10	_
ENSG00000271973	-3.68859	-2.060656	1	1	RP11-572O6.1	_
ENSG00000272248	-3.68859	-2.060656	1	1	RP3-406P24.4	_
ENSG00000272334	-3.68859	-2.060656	1	1	RP11-129K12.1	_
ENSG00000272366	-3.68859	-2.060656	1	1	RP11-573G6.10	_
ENSG00000272389	-3.68859	-2.060656	1	1	CTC-487M23.7	_
ENSG00000272507	-3.68859	-2.060656	1	1	U6	_
ENSG00000272562	-3.68859	-2.060656	1	1	RP11-396C23.4	_
ENSG00000272582	-3.68859	-2.060656	1	1	RP5-1039K5.17	_
ENSG00000272589	-3.68859	-2.060656	1	1	ZSWIM8-AS1	_
ENSG00000272810	-3.68859	-2.060656	1	1	U91328.22	_
ENSG00000272817	-3.68859	-2.060656	1	1	RP11-402D21.2	_
ENSG00000272828	-3.68859	-2.060656	1	1	CTD-2555O16.3	_
ENSG00000273062	-3.68859	-2.060656	1	1	RP11-428K3.1	_
ENSG00000273096	-3.68859	-2.060656	1	1	RP3-508I15.20	_

ENSG00000273309	-3.68859	-2.060656	1	1	AC004410.3	_
ENSG00000273367	-3.68859	-2.060656	1	1	RP5-827C21.6	_
ENSG00000273398	-3.68859	-2.060656	1	1	RP11-474G23.1	K06268
ENSG00000273450	-3.68859	-2.060656	1	1	RP11-76P2.4	_
ENSG00000273492	-3.68859	-2.060656	1	1	AP000230.1	_
ENSG00000004809	-2.796355	-2.316672	1	1	SLC22A16	K08212
ENSG00000006075	-2.796355	-2.316672	1	1	CCL3	K05408
ENSG00000008056	-2.796355	-2.316672	1	1	SYN1	_
ENSG00000009694	-2.796355	-2.316672	1	1	TENM1	_
ENSG00000009765	-2.796355	-2.316672	1	1	IYD	K17231
ENSG00000010379	-2.796355	-2.316672	1	1	SLC6A13	K05039
ENSG00000018625	-2.796355	-2.316672	1	1	ATP1A2	K01539
ENSG00000019102	-2.796355	-2.316672	1	1	VSIG2	_
ENSG00000019505	-2.796355	-2.316672	1	1	SYT13	K12022
ENSG00000023902	-2.796355	-2.316672	1	1	PLEKHO1	_
ENSG00000026652	-2.796355	-2.316672	1	1	AGPAT4	K13523
ENSG00000042832	-2.796355	-2.316672	1	1	TG	K10809
ENSG00000042980	-2.796355	-2.316672	1	1	ADAM28	K08614
ENSG00000049769	-2.796355	-2.316672	1	1	PPP1R3F	K17453
ENSG00000064989	-2.796355	-2.316672	1	1	CALCRL	_
ENSG00000067191	-2.796355	-2.316672	1	1	CACNB1	K04862
ENSG00000069493	-2.796355	-2.316672	1	1	CLEC2D	K10072
ENSG00000070526	-2.796355	-2.316672	1	1	ST6GALNAC1	K03479
ENSG00000071051	-2.796355	-2.316672	1	1	NCK2	K07365
ENSG00000072609	-2.796355	-2.316672	1	1	CHFR	K11447
ENSG00000072858	-2.796355	-2.316672	1	1	SIDT1	_
ENSG00000073146	-2.796355	-2.316672	1	1	MOV10L1	K13983
ENSG00000073598	-2.796355	-2.316672	1	1	FNDC8	_
ENSG00000077063	-2.796355	-2.316672	1	1	CTTNBP2	_
ENSG00000077080	-2.796355	-2.316672	1	1	ACTL6B	K11652
ENSG00000080166	-2.796355	-2.316672	1	1	DCT	K01827
ENSG00000081853	-2.796355	-2.316672	1	1	PCDHGA2	K16495
ENSG00000082126	-2.796355	-2.316672	1	1	MPP4	_
ENSG00000082293	-2.796355	-2.316672	1	1	COL19A1	_
ENSG00000083307	-2.796355	-2.316672	1	1	GRHL2	K09275
ENSG00000087245	-2.796355	-2.316672	1	1	MMP2	K01398
ENSG00000087589	-2.796355	-2.316672	1	1	CASS4	_
ENSG00000089101	-2.796355	-2.316672	1	1	C20orf26	K12869
ENSG00000090376	-2.796355	-2.316672	1	1	IRAK3	K04732
ENSG00000091972	-2.796355	-2.316672	1	1	CD200	K06556
ENSG00000092067	-2.796355	-2.316672	1	1	CEBPE	K10051
ENSG00000092850	-2.796355	-2.316672	1	1	TEKT2	K18629
ENSG00000093134	-2.796355	-2.316672	1	1	VNN3	K08069
ENSG00000095587	-2.796355	-2.316672	1	1	TLL2	K13047
ENSG00000095596	-2.796355	-2.316672	1	1	CYP26A1	K07437
ENSG00000100197	-2.796355	-2.316672	1	1	CYP2D6	K17712
ENSG00000100351	-2.796355	-2.316672	1	1	GRAP2	K07366
ENSG00000100368	-2.796355	-2.316672	1	1	CSF2RB	K04738
ENSG00000100505	-2.796355	-2.316672	1	1	TRIM9	K10649
ENSG00000100842	-2.796355	-2.316672	1	1	EFS	_
ENSG00000101638	-2.796355	-2.316672	1	1	ST8SIA5	K03369
ENSG00000101890	-2.796355	-2.316672	1	1	GUCY2F	K12321
ENSG00000101916	-2.796355	-2.316672	1	1	TLR8	K10170
ENSG00000102010	-2.796355	-2.316672	1	1	BMX	K08896
ENSG00000102104	-2.796355	-2.316672	1	1	RS1	K08824
ENSG00000102119	-2.796355	-2.316672	1	1	EMD	K12569
ENSG00000102539	-2.796355	-2.316672	1	1	MLNR	K05266
ENSG00000102970	-2.796355	-2.316672	1	1	CCL17	K05514

ENSG00000103241	-2.796355	-2.316672	1	1	FOXF1	K09399
ENSG00000103522	-2.796355	-2.316672	1	1	IL21R	K05075
ENSG00000104177	-2.796355	-2.316672	1	1	MYEF2	K13753
ENSG00000104321	-2.796355	-2.316672	1	1	TRPA1	K04984
ENSG00000104941	-2.796355	-2.316672	1	1	RSPH6A	_
ENSG00000105122	-2.796355	-2.316672	1	1	RASAL3	K17634
ENSG00000105278	-2.796355	-2.316672	1	1	ZFR2	_
ENSG00000105366	-2.796355	-2.316672	1	1	SIGLEC8	K06748
ENSG00000105370	-2.796355	-2.316672	1	1	LIM2	_
ENSG00000105509	-2.796355	-2.316672	1	1	HAS1	K00752
ENSG00000105664	-2.796355	-2.316672	1	1	COMP	K04659
ENSG00000105697	-2.796355	-2.316672	1	1	HAMP	_
ENSG00000105988	-2.796355	-2.316672	1	1	NHP2P1	K11129
ENSG00000106436	-2.796355	-2.316672	1	1	MYL10	K12756
ENSG00000106686	-2.796355	-2.316672	1	1	SPATA6L	_
ENSG00000108849	-2.796355	-2.316672	1	1	PPY	K05234
ENSG00000109084	-2.796355	-2.316672	1	1	TMEM97	K04264
ENSG00000109610	-2.796355	-2.316672	1	1	SOD3	K16627
ENSG00000110243	-2.796355	-2.316672	1	1	APOA5	K09025
ENSG00000110455	-2.796355	-2.316672	1	1	ACCS	_
ENSG00000110887	-2.796355	-2.316672	1	1	DAO	K00273
ENSG00000111254	-2.796355	-2.316672	1	1	AKAP3	_
ENSG00000111676	-2.796355	-2.316672	1	1	ATN1	K05626
ENSG00000112183	-2.796355	-2.316672	1	1	RBM24	_
ENSG00000112212	-2.796355	-2.316672	1	1	TSPO2	_
ENSG00000112246	-2.796355	-2.316672	1	1	SIM1	K09100
ENSG00000112303	-2.796355	-2.316672	1	1	VNN2	K08069
ENSG00000112619	-2.796355	-2.316672	1	1	PRPH2	K17343
ENSG00000113100	-2.796355	-2.316672	1	1	CDH9	K06801
ENSG00000113520	-2.796355	-2.316672	1	1	IL4	K05430
ENSG00000113657	-2.796355	-2.316672	1	1	DPYSL3	K07528
ENSG00000113889	-2.796355	-2.316672	1	1	KNG1	K03898
ENSG00000114812	-2.796355	-2.316672	1	1	VIPR1	_
ENSG00000115112	-2.796355	-2.316672	1	1	TFCP2L1	K09275
ENSG00000115297	-2.796355	-2.316672	1	1	TLX2	K15606
ENSG00000115353	-2.796355	-2.316672	1	1	TACR1	K04222
ENSG00000115705	-2.796355	-2.316672	1	1	TPO	K00431
ENSG00000116521	-2.796355	-2.316672	1	1	SCAMP3	_
ENSG00000116785	-2.796355	-2.316672	1	1	CFHR3	K04004
ENSG00000117114	-2.796355	-2.316672	1	1	LPHN2	K04593
ENSG00000118156	-2.796355	-2.316672	1	1	ZNF541	_
ENSG00000118292	-2.796355	-2.316672	1	1	C1orf54	_
ENSG00000118526	-2.796355	-2.316672	1	1	TCF21	K09072
ENSG00000119411	-2.796355	-2.316672	1	1	BSPRY	_
ENSG00000120215	-2.796355	-2.316672	1	1	MLANA	K17303
ENSG00000120242	-2.796355	-2.316672	1	1	IFNA8	K05414
ENSG00000120937	-2.796355	-2.316672	1	1	NPPB	K12335
ENSG00000121898	-2.796355	-2.316672	1	1	CPXM2	K08639
ENSG00000122787	-2.796355	-2.316672	1	1	AKR1D1	K00251
ENSG00000123453	-2.796355	-2.316672	1	1	SARDH	K00314
ENSG00000123892	-2.796355	-2.316672	1	1	RAB38	K07923
ENSG00000124092	-2.796355	-2.316672	1	1	CTCFL	K11447
ENSG00000124205	-2.796355	-2.316672	1	1	EDN3	K05227
ENSG00000124491	-2.796355	-2.316672	1	1	F13A1	K03917
ENSG00000124835	-2.796355	-2.316672	1	1	AC105760.3	_
ENSG00000125207	-2.796355	-2.316672	1	1	PIWIL1	K02156
ENSG00000125675	-2.796355	-2.316672	1	1	GRIA3	K05199
ENSG00000125787	-2.796355	-2.316672	1	1	GNRH2	K05252

ENSG00000126010	-2.796355	-2.316672	1	1	GRPR	K04169
ENSG00000126895	-2.796355	-2.316672	1	1	AVPR2	K04228
ENSG00000126950	-2.796355	-2.316672	1	1	TMEM35	_
ENSG00000127249	-2.796355	-2.316672	1	1	ATP13A4	K14951
ENSG00000128254	-2.796355	-2.316672	1	1	C22orf24	_
ENSG00000128276	-2.796355	-2.316672	1	1	RFPL3	_
ENSG00000128285	-2.796355	-2.316672	1	1	MCHR1	K04320
ENSG00000128313	-2.796355	-2.316672	1	1	APOL5	K14480
ENSG00000128849	-2.796355	-2.316672	1	1	CGNL1	_
ENSG00000129151	-2.796355	-2.316672	1	1	BBOX1	_
ENSG00000130173	-2.796355	-2.316672	1	1	C19orf80	_
ENSG00000130592	-2.796355	-2.316672	1	1	LSP1	K14957
ENSG00000130957	-2.796355	-2.316672	1	1	FBP2	K03841
ENSG00000131015	-2.796355	-2.316672	1	1	ULBP2	K07986
ENSG00000131096	-2.796355	-2.316672	1	1	PYY	K05233
ENSG00000131355	-2.796355	-2.316672	1	1	EMR3	K08444
ENSG00000131386	-2.796355	-2.316672	1	1	GALNT15	K00710
ENSG00000131435	-2.796355	-2.316672	1	1	PDLIM4	_
ENSG00000131484	-2.796355	-2.316672	1	1	RP11-798G7.5	_
ENSG00000131771	-2.796355	-2.316672	1	1	PPP1R1B	K15494
ENSG00000132911	-2.796355	-2.316672	1	1	NMUR2	K05053
ENSG00000133256	-2.796355	-2.316672	1	1	PDE6B	K13756
ENSG00000133636	-2.796355	-2.316672	1	1	NTS	K05235
ENSG00000133962	-2.796355	-2.316672	1	1	CATSPERB	K16893
ENSG00000134072	-2.796355	-2.316672	1	1	CAMK1	K08794
ENSG00000134198	-2.796355	-2.316672	1	1	TSPAN2	K17354
ENSG00000134326	-2.796355	-2.316672	1	1	CMPK2	K13809
ENSG00000134817	-2.796355	-2.316672	1	1	APLNR	K04174
ENSG00000135077	-2.796355	-2.316672	1	1	HAVCR2	_
ENSG00000135175	-2.796355	-2.316672	1	1	OCM2	_
ENSG00000135443	-2.796355	-2.316672	1	1	KRT85	K07605
ENSG00000135472	-2.796355	-2.316672	1	1	FAIM2	_
ENSG00000135625	-2.796355	-2.316672	1	1	EGR4	K12498
ENSG00000136014	-2.796355	-2.316672	1	1	USP44	K11834
ENSG00000136267	-2.796355	-2.316672	1	1	DGKB	K00901
ENSG00000136574	-2.796355	-2.316672	1	1	GATA4	K09183
ENSG00000137033	-2.796355	-2.316672	1	1	IL33	K12967
ENSG00000138028	-2.796355	-2.316672	1	1	CGREF1	K00846
ENSG00000138075	-2.796355	-2.316672	1	1	ABCG5	K05683
ENSG00000138400	-2.796355	-2.316672	1	1	MDH1B	K15048
ENSG00000138483	-2.796355	-2.316672	1	1	CCDC54	_
ENSG00000138823	-2.796355	-2.316672	1	1	MTTP	K14463
ENSG00000138892	-2.796355	-2.316672	1	1	TTLL8	K16608
ENSG00000139508	-2.796355	-2.316672	1	1	SLC46A3	K14613
ENSG00000139549	-2.796355	-2.316672	1	1	DHH	K11990
ENSG00000139572	-2.796355	-2.316672	1	1	GPR84	K08421
ENSG00000139973	-2.796355	-2.316672	1	1	SYT16	K19328
ENSG00000140368	-2.796355	-2.316672	1	1	PSTPIP1	K12804
ENSG00000140527	-2.796355	-2.316672	1	1	WDR93	K12581
ENSG00000140749	-2.796355	-2.316672	1	1	IGSF6	K11447
ENSG00000140839	-2.796355	-2.316672	1	1	CLEC18B	_
ENSG00000141434	-2.796355	-2.316672	1	1	MEP1B	K08606
ENSG00000141748	-2.796355	-2.316672	1	1	ARL5C	K07960
ENSG00000141756	-2.796355	-2.316672	1	1	FKBP10	K09575
ENSG00000142405	-2.796355	-2.316672	1	1	NLRP12	K01353
ENSG00000142583	-2.796355	-2.316672	1	1	SLC2A5	K08143
ENSG00000143185	-2.796355	-2.316672	1	1	XCL2	K05507
ENSG00000143603	-2.796355	-2.316672	1	1	KCNN3	K04944

ENSG00000144229	-2.796355	-2.316672	1	1	THSD7B	_
ENSG00000144671	-2.796355	-2.316672	1	1	SLC22A14	K08210
ENSG00000144771	-2.796355	-2.316672	1	1	LRTM1	_
ENSG00000144792	-2.796355	-2.316672	1	1	ZNF660	_
ENSG00000144852	-2.796355	-2.316672	1	1	NR1I2	K08540
ENSG00000144893	-2.796355	-2.316672	1	1	MED12L	K15162
ENSG00000145050	-2.796355	-2.316672	1	1	MANF	_
ENSG00000145681	-2.796355	-2.316672	1	1	HAPLN1	K06848
ENSG00000145879	-2.796355	-2.316672	1	1	SPINK7	_
ENSG00000145936	-2.796355	-2.316672	1	1	KCNMB1	K04937
ENSG00000146216	-2.796355	-2.316672	1	1	TTBK1	K08815
ENSG00000146276	-2.796355	-2.316672	1	1	GABRR1	K05190
ENSG00000146839	-2.796355	-2.316672	1	1	ZAN	_
ENSG00000146955	-2.796355	-2.316672	1	1	RAB19	K17047
ENSG00000147082	-2.796355	-2.316672	1	1	CCNB3	K05868
ENSG00000147183	-2.796355	-2.316672	1	1	CPXCR1	_
ENSG00000147206	-2.796355	-2.316672	1	1	NXF3	K14284
ENSG00000147402	-2.796355	-2.316672	1	1	GABRQ	K05192
ENSG00000147588	-2.796355	-2.316672	1	1	PMP2	K08753
ENSG00000147614	-2.796355	-2.316672	1	1	ATP6V0D2	K02146
ENSG00000147724	-2.796355	-2.316672	1	1	FAM135B	_
ENSG00000148377	-2.796355	-2.316672	1	1	IDI2	K01823
ENSG00000148735	-2.796355	-2.316672	1	1	PLEKHS1	_
ENSG00000149428	-2.796355	-2.316672	1	1	HYOU1	K09486
ENSG00000149506	-2.796355	-2.316672	1	1	ZP1	_
ENSG00000149599	-2.796355	-2.316672	1	1	DUSP15	K14165
ENSG00000150676	-2.796355	-2.316672	1	1	CCDC83	_
ENSG00000150681	-2.796355	-2.316672	1	1	RGS18	K16449
ENSG00000151240	-2.796355	-2.316672	1	1	DIP2C	K09228
ENSG00000151303	-2.796355	-2.316672	1	1	AGAP11	_
ENSG00000151617	-2.796355	-2.316672	1	1	EDNRA	K04197
ENSG00000151640	-2.796355	-2.316672	1	1	DPYSL4	K07528
ENSG00000151963	-2.796355	-2.316672	1	1	RP11-775A3.1	K09228
ENSG00000152766	-2.796355	-2.316672	1	1	ANKRD22	_
ENSG00000152822	-2.796355	-2.316672	1	1	GRM1	K04603
ENSG00000153976	-2.796355	-2.316672	1	1	HS3ST3A1	K07809
ENSG00000154016	-2.796355	-2.316672	1	1	GRAP	K04364
ENSG00000154118	-2.796355	-2.316672	1	1	JPH3	K19530
ENSG00000154134	-2.796355	-2.316672	1	1	ROBO3	K06755
ENSG00000154277	-2.796355	-2.316672	1	1	UCHL1	K05611
ENSG00000155099	-2.796355	-2.316672	1	1	TMEM55A	K13084
ENSG00000155749	-2.796355	-2.316672	1	1	ALS2CR12	_
ENSG00000156049	-2.796355	-2.316672	1	1	GNA14	K04636
ENSG00000156219	-2.796355	-2.316672	1	1	ART3	K00775
ENSG00000156299	-2.796355	-2.316672	1	1	TIAM1	K05731
ENSG00000156427	-2.796355	-2.316672	1	1	FGF18	K04358
ENSG00000157005	-2.796355	-2.316672	1	1	SST	K05237
ENSG00000157423	-2.796355	-2.316672	1	1	HYDIN	K17570
ENSG00000157851	-2.796355	-2.316672	1	1	DPYSL5	K07529
ENSG00000157856	-2.796355	-2.316672	1	1	DRC1	K19754
ENSG00000158055	-2.796355	-2.316672	1	1	GRHL3	K09275
ENSG00000158525	-2.796355	-2.316672	1	1	CPA5	K08781
ENSG00000158560	-2.796355	-2.316672	1	1	DYNC1I1	K10415
ENSG00000158806	-2.796355	-2.316672	1	1	NPM2	K11277
ENSG00000158813	-2.796355	-2.316672	1	1	EDA	K05480
ENSG00000158865	-2.796355	-2.316672	1	1	SLC5A11	K14391
ENSG00000159212	-2.796355	-2.316672	1	1	CLIC6	K05026
ENSG00000159251	-2.796355	-2.316672	1	1	ACTC1	K12314

ENSG00000159339	-2.796355	-2.316672	1	1	PADI4	K01481
ENSG00000159713	-2.796355	-2.316672	1	1	TPPP3	_
ENSG00000160181	-2.796355	-2.316672	1	1	TFF2	_
ENSG00000160188	-2.796355	-2.316672	1	1	RSPH1	K19755
ENSG00000160339	-2.796355	-2.316672	1	1	FCN2	K10104
ENSG00000160472	-2.796355	-2.316672	1	1	TMEM190	_
ENSG00000160886	-2.796355	-2.316672	1	1	LY6K	_
ENSG00000161133	-2.796355	-2.316672	1	1	USP41	K11846
ENSG00000162627	-2.796355	-2.316672	1	1	SNX7	K17921
ENSG00000162654	-2.796355	-2.316672	1	1	GBP4	_
ENSG00000162687	-2.796355	-2.316672	1	1	KCNT2	K04947
ENSG00000162836	-2.796355	-2.316672	1	1	ACP6	K14395
ENSG00000162897	-2.796355	-2.316672	1	1	FCAMR	K16851
ENSG00000163053	-2.796355	-2.316672	1	1	SLC16A14	K08190
ENSG00000163072	-2.796355	-2.316672	1	1	NOSTRIN	_
ENSG00000163216	-2.796355	-2.316672	1	1	SPRR2D	_
ENSG00000163221	-2.796355	-2.316672	1	1	S100A12	_
ENSG00000163359	-2.796355	-2.316672	1	1	COL6A3	K06238
ENSG00000163362	-2.796355	-2.316672	1	1	C1orf106	_
ENSG00000163380	-2.796355	-2.316672	1	1	LMOD3	K12581
ENSG00000163762	-2.796355	-2.316672	1	1	TM4SF18	_
ENSG00000163814	-2.796355	-2.316672	1	1	CDCP1	K06732
ENSG00000164049	-2.796355	-2.316672	1	1	FBXW12	K10267
ENSG00000164076	-2.796355	-2.316672	1	1	CAMKV	K08812
ENSG00000164181	-2.796355	-2.316672	1	1	ELOVL7	K10250
ENSG00000164185	-2.796355	-2.316672	1	1	ZNF474	_
ENSG00000164659	-2.796355	-2.316672	1	1	KIAA1324L	_
ENSG00000164691	-2.796355	-2.316672	1	1	TAGAP	_
ENSG00000164741	-2.796355	-2.316672	1	1	DLC1	_
ENSG00000164749	-2.796355	-2.316672	1	1	HNF4G	K08037
ENSG00000164761	-2.796355	-2.316672	1	1	TNFRSF11B	K05148
ENSG00000165120	-2.796355	-2.316672	1	1	SSMEM1	_
ENSG00000165125	-2.796355	-2.316672	1	1	TRPV6	K04975
ENSG00000165194	-2.796355	-2.316672	1	1	PCDH19	K16499
ENSG00000165325	-2.796355	-2.316672	1	1	CCDC67	_
ENSG00000165548	-2.796355	-2.316672	1	1	TMEM63C	_
ENSG00000165702	-2.796355	-2.316672	1	1	GFI1B	K09223
ENSG00000165923	-2.796355	-2.316672	1	1	AGBL2	_
ENSG00000165966	-2.796355	-2.316672	1	1	PDZRN4	K15682
ENSG00000166143	-2.796355	-2.316672	1	1	PPP1R14D	K17557
ENSG00000166159	-2.796355	-2.316672	1	1	LRTM2	_
ENSG00000166341	-2.796355	-2.316672	1	1	DCHS1	K16507
ENSG00000166349	-2.796355	-2.316672	1	1	RAG1	K10628
ENSG00000166428	-2.796355	-2.316672	1	1	PLD4	K16860
ENSG00000166670	-2.796355	-2.316672	1	1	MMP10	K01396
ENSG00000167034	-2.796355	-2.316672	1	1	NKX3-1	K09348
ENSG00000167083	-2.796355	-2.316672	1	1	GNGT2	K04549
ENSG00000167100	-2.796355	-2.316672	1	1	SAMD14	K17551
ENSG00000167131	-2.796355	-2.316672	1	1	CCDC103	_
ENSG00000167230	-2.796355	-2.316672	1	1	C17orf78	_
ENSG00000167332	-2.796355	-2.316672	1	1	OR51E2	K04257
ENSG00000167531	-2.796355	-2.316672	1	1	LALBA	K00704
ENSG00000167617	-2.796355	-2.316672	1	1	CDC42EP5	_
ENSG00000167723	-2.796355	-2.316672	1	1	TRPV3	K04972
ENSG00000168242	-2.796355	-2.316672	1	1	HIST1H2BI	K11252
ENSG00000168243	-2.796355	-2.316672	1	1	GNG4	K04541
ENSG00000168843	-2.796355	-2.316672	1	1	FSTL5	K06826
ENSG00000169508	-2.796355	-2.316672	1	1	GPR183	K04305

ENSG00000169777	-2.796355	-2.316672	1	1	TAS2R1	K08474
ENSG00000170454	-2.796355	-2.316672	1	1	KRT75	K07605
ENSG00000170477	-2.796355	-2.316672	1	1	KRT4	K07605
ENSG00000170927	-2.796355	-2.316672	1	1	PKHD1	_
ENSG00000170935	-2.796355	-2.316672	1	1	NCBP2L	K12883
ENSG00000171291	-2.796355	-2.316672	1	1	ZNF439	K09228
ENSG00000171357	-2.796355	-2.316672	1	1	LURAP1	K12479
ENSG00000171451	-2.796355	-2.316672	1	1	DSEL	K17985
ENSG00000171532	-2.796355	-2.316672	1	1	NEUROD2	K09078
ENSG00000171570	-2.796355	-2.316672	1	1	RAB4B-EGLN2	K09592
ENSG00000171659	-2.796355	-2.316672	1	1	GPR34	K08383
ENSG00000171847	-2.796355	-2.316672	1	1	FAM90A1	_
ENSG00000173262	-2.796355	-2.316672	1	1	SLC2A14	K08142
ENSG00000173728	-2.796355	-2.316672	1	1	C1orf100	_
ENSG00000174498	-2.796355	-2.316672	1	1	IGDCC3	_
ENSG00000174640	-2.796355	-2.316672	1	1	SLCO2A1	K14345
ENSG00000174715	-2.796355	-2.316672	1	1	RP11-79L9.2	K03767
ENSG00000175121	-2.796355	-2.316672	1	1	WFDC5	_
ENSG00000175749	-2.796355	-2.316672	1	1	EIF3KP1	K15028
ENSG00000175841	-2.796355	-2.316672	1	1	FAM172BP	K02927
ENSG00000176153	-2.796355	-2.316672	1	1	GPX2	K00432
ENSG00000176354	-2.796355	-2.316672	1	1	AC097359.1	_
ENSG00000176406	-2.796355	-2.316672	1	1	RIMS2	K15297
ENSG00000176571	-2.796355	-2.316672	1	1	CNBD1	_
ENSG00000176658	-2.796355	-2.316672	1	1	MYO1D	K10356
ENSG00000177291	-2.796355	-2.316672	1	1	GJD4	K07630
ENSG00000177452	-2.796355	-2.316672	1	1	RP4-597J3.1	K02885
ENSG00000177459	-2.796355	-2.316672	1	1	C8orf47	_
ENSG00000178125	-2.796355	-2.316672	1	1	PPP1R42	K09613
ENSG00000178171	-2.796355	-2.316672	1	1	AMER3	K04372
ENSG00000178233	-2.796355	-2.316672	1	1	TMEM151B	K01872
ENSG00000178597	-2.796355	-2.316672	1	1	PSAPL1	K12382
ENSG00000178631	-2.796355	-2.316672	1	1	ACTG1P1	K05692
ENSG00000178997	-2.796355	-2.316672	1	1	EXD1	K18740
ENSG00000179023	-2.796355	-2.316672	1	1	KLHDC7A	_
ENSG00000179057	-2.796355	-2.316672	1	1	IGSF22	_
ENSG00000179058	-2.796355	-2.316672	1	1	C9orf50	_
ENSG00000179082	-2.796355	-2.316672	1	1	C9orf106	_
ENSG00000179101	-2.796355	-2.316672	1	1	RP11-349N19.2	K11253
ENSG00000179292	-2.796355	-2.316672	1	1	TMEM151A	_
ENSG00000179407	-2.796355	-2.316672	1	1	DNAJB8	K09514
ENSG00000179915	-2.796355	-2.316672	1	1	NRXN1	K07377
ENSG00000180105	-2.796355	-2.316672	1	1	AC017081.2	K01441
ENSG00000180139	-2.796355	-2.316672	1	1	ACTA2-AS1	_
ENSG00000180189	-2.796355	-2.316672	1	1	HMGB1P14	K10802
ENSG00000180245	-2.796355	-2.316672	1	1	RRH	K04253
ENSG00000180353	-2.796355	-2.316672	1	1	HCLS1	K06106
ENSG00000180549	-2.796355	-2.316672	1	1	FUT7	K07635
ENSG00000180610	-2.796355	-2.316672	1	1	ZBTB12P1	K10499
ENSG00000180697	-2.796355	-2.316672	1	1	C3orf22	_
ENSG00000180867	-2.796355	-2.316672	1	1	PDIA3P1	K08056
ENSG00000181039	-2.796355	-2.316672	1	1	ANKRD34A	K03024
ENSG00000181741	-2.796355	-2.316672	1	1	FDX1P1	_
ENSG00000181781	-2.796355	-2.316672	1	1	ODF3L2	_
ENSG00000182389	-2.796355	-2.316672	1	1	CACNB4	K04865
ENSG00000182500	-2.796355	-2.316672	1	1	ORAI1	K16056
ENSG00000182545	-2.796355	-2.316672	1	1	RNASE10	_
ENSG00000182646	-2.796355	-2.316672	1	1	FAM156A	_

ENSG00000182774	-2.796355	-2.316672	1	1	RPS17L	K02962
ENSG00000182814	-2.796355	-2.316672	1	1	FUNDC2P2	_
ENSG00000182824	-2.796355	-2.316672	1	1	AC008132.1	_
ENSG00000182870	-2.796355	-2.316672	1	1	GALNT9	K00710
ENSG00000183196	-2.796355	-2.316672	1	1	CHST6	K09671
ENSG00000183230	-2.796355	-2.316672	1	1	CTNNA3	K05691
ENSG00000183305	-2.796355	-2.316672	1	1	MAGEA2B	_
ENSG00000183324	-2.796355	-2.316672	1	1	C15orf60	_
ENSG00000183580	-2.796355	-2.316672	1	1	FBXL7	K10273
ENSG00000183775	-2.796355	-2.316672	1	1	KCTD16	_
ENSG00000183844	-2.796355	-2.316672	1	1	FAM3B	_
ENSG00000183979	-2.796355	-2.316672	1	1	NPB	K05267
ENSG00000184111	-2.796355	-2.316672	1	1	RP11-366I13.3	K02868
ENSG00000184113	-2.796355	-2.316672	1	1	CLDN5	K06087
ENSG00000184185	-2.796355	-2.316672	1	1	KCNJ12	K19468
ENSG00000184343	-2.796355	-2.316672	1	1	SRPK3	K08832
ENSG00000184371	-2.796355	-2.316672	1	1	CSF1	K05453
ENSG00000184507	-2.796355	-2.316672	1	1	NUTM1	_
ENSG00000184735	-2.796355	-2.316672	1	1	DDX53	K17043
ENSG00000184984	-2.796355	-2.316672	1	1	CHRM5	K04133
ENSG00000185013	-2.796355	-2.316672	1	1	NT5C1B	K01081
ENSG00000185070	-2.796355	-2.316672	1	1	FLRT2	_
ENSG00000185247	-2.796355	-2.316672	1	1	MAGEA11	_
ENSG00000185306	-2.796355	-2.316672	1	1	C12orf56	_
ENSG00000185339	-2.796355	-2.316672	1	1	TCN2	K14619
ENSG00000185433	-2.796355	-2.316672	1	1	LINC00158	_
ENSG00000185899	-2.796355	-2.316672	1	1	TAS2R60	K08474
ENSG00000186442	-2.796355	-2.316672	1	1	KRT3	K07605
ENSG00000186466	-2.796355	-2.316672	1	1	AQP7P1	K08771
ENSG00000186529	-2.796355	-2.316672	1	1	CYP4F3	K17726
ENSG00000186628	-2.796355	-2.316672	1	1	FSD2	_
ENSG00000186998	-2.796355	-2.316672	1	1	EMID1	_
ENSG00000187045	-2.796355	-2.316672	1	1	TMPRSS6	K09637
ENSG00000187144	-2.796355	-2.316672	1	1	SPATA21	_
ENSG00000187166	-2.796355	-2.316672	1	1	H1FNT	_
ENSG00000187229	-2.796355	-2.316672	1	1	RP11-161I2.1	_
ENSG00000187268	-2.796355	-2.316672	1	1	FAM9C	K17985
ENSG00000187288	-2.796355	-2.316672	1	1	CIDEC	_
ENSG00000187474	-2.796355	-2.316672	1	1	FPR3	K04173
ENSG00000187616	-2.796355	-2.316672	1	1	TMEM8C	_
ENSG00000187762	-2.796355	-2.316672	1	1	HSPE1P11	K04078
ENSG00000187862	-2.796355	-2.316672	1	1	TTC24	_
ENSG00000187902	-2.796355	-2.316672	1	1	SHISA7	_
ENSG00000187912	-2.796355	-2.316672	1	1	CLEC17A	K17513
ENSG00000187955	-2.796355	-2.316672	1	1	COL14A1	K08133
ENSG00000187987	-2.796355	-2.316672	1	1	ZSCAN23	K09230
ENSG00000188037	-2.796355	-2.316672	1	1	CLCN1	K05010
ENSG00000188073	-2.796355	-2.316672	1	1	PMS2P10	K10858
ENSG00000188089	-2.796355	-2.316672	1	1	PLA2G4E	K16342
ENSG00000188124	-2.796355	-2.316672	1	1	OR2AG2	K04257
ENSG00000188162	-2.796355	-2.316672	1	1	OTOG	_
ENSG00000188305	-2.796355	-2.316672	1	1	C19orf35	_
ENSG00000188424	-2.796355	-2.316672	1	1	XXbac-B33L19.3	_
ENSG00000188451	-2.796355	-2.316672	1	1	SRP72P2	K03108
ENSG00000188624	-2.796355	-2.316672	1	1	IGFL3	_
ENSG00000188626	-2.796355	-2.316672	1	1	GOLGA8M	K10595
ENSG00000188676	-2.796355	-2.316672	1	1	IDO2	K00463
ENSG00000188686	-2.796355	-2.316672	1	1	SCXA	K09070

ENSG00000188693	-2.796355	-2.316672	1	1	CTB-161K23.1	_
ENSG00000188869	-2.796355	-2.316672	1	1	TMC3	_
ENSG00000188958	-2.796355	-2.316672	1	1	UTS2B	K05248
ENSG00000189058	-2.796355	-2.316672	1	1	APOD	K03098
ENSG00000189320	-2.796355	-2.316672	1	1	FAM180A	_
ENSG00000189377	-2.796355	-2.316672	1	1	CXCL17	_
ENSG00000196267	-2.796355	-2.316672	1	1	ZNF836	K09228
ENSG00000196289	-2.796355	-2.316672	1	1	BECN1P1	K08334
ENSG00000196369	-2.796355	-2.316672	1	1	SRGAP2B	K07526
ENSG00000196566	-2.796355	-2.316672	1	1	RP11-57C13.3	_
ENSG00000196570	-2.796355	-2.316672	1	1	PFN3	K05759
ENSG00000196593	-2.796355	-2.316672	1	1	ANKRD20A19P	K17299
ENSG00000196634	-2.796355	-2.316672	1	1	RP3-337D23.3	_
ENSG00000196666	-2.796355	-2.316672	1	1	FAM180B	_
ENSG00000196674	-2.796355	-2.316672	1	1	Z98881.1	_
ENSG00000196867	-2.796355	-2.316672	1	1	ZFP28	K09228
ENSG00000196900	-2.796355	-2.316672	1	1	C5orf48	_
ENSG00000196917	-2.796355	-2.316672	1	1	HCAR1	K08401
ENSG00000197047	-2.796355	-2.316672	1	1	Z73979.1	_
ENSG00000197168	-2.796355	-2.316672	1	1	NEK5	K08857
ENSG00000197376	-2.796355	-2.316672	1	1	OR8S1	K04257
ENSG00000197830	-2.796355	-2.316672	1	1	AC011385.1	K11447
ENSG00000197882	-2.796355	-2.316672	1	1	OR7E13P	K04257
ENSG00000197915	-2.796355	-2.316672	1	1	HRNR	_
ENSG00000198336	-2.796355	-2.316672	1	1	MYL4	K12750
ENSG00000198416	-2.796355	-2.316672	1	1	CTD-2173L22.4	K09228
ENSG00000198535	-2.796355	-2.316672	1	1	C2CD4A	K10734
ENSG00000198566	-2.796355	-2.316672	1	1	ZNF658B	K09228
ENSG00000198768	-2.796355	-2.316672	1	1	APCDD1L	_
ENSG00000198945	-2.796355	-2.316672	1	1	L3MBTL3	_
ENSG00000199226	-2.796355	-2.316672	1	1	RNU6-50P	_
ENSG00000199415	-2.796355	-2.316672	1	1	RNA5SP370	_
ENSG00000199466	-2.796355	-2.316672	1	1	Y_RNA	_
ENSG00000199471	-2.796355	-2.316672	1	1	Y_RNA	_
ENSG00000199550	-2.796355	-2.316672	1	1	Y_RNA	_
ENSG00000199664	-2.796355	-2.316672	1	1	RNU6-1266P	_
ENSG00000199668	-2.796355	-2.316672	1	1	Y_RNA	_
ENSG00000199676	-2.796355	-2.316672	1	1	Y_RNA	_
ENSG00000199732	-2.796355	-2.316672	1	1	Y_RNA	_
ENSG00000199755	-2.796355	-2.316672	1	1	Y_RNA	_
ENSG00000199806	-2.796355	-2.316672	1	1	RNA5SP491	_
ENSG00000199961	-2.796355	-2.316672	1	1	SNORD1B	_
ENSG00000200059	-2.796355	-2.316672	1	1	Y_RNA	_
ENSG00000200063	-2.796355	-2.316672	1	1	SNORA30	_
ENSG00000200201	-2.796355	-2.316672	1	1	Y_RNA	_
ENSG00000200209	-2.796355	-2.316672	1	1	Y_RNA	_
ENSG00000200237	-2.796355	-2.316672	1	1	SNORA70	_
ENSG00000200252	-2.796355	-2.316672	1	1	Y_RNA	_
ENSG00000200354	-2.796355	-2.316672	1	1	SNORA71D	_
ENSG00000200525	-2.796355	-2.316672	1	1	RNU6-1209P	_
ENSG00000200579	-2.796355	-2.316672	1	1	Y_RNA	_
ENSG00000200759	-2.796355	-2.316672	1	1	RNU6-884P	_
ENSG00000200785	-2.796355	-2.316672	1	1	SNORD8	_
ENSG00000200847	-2.796355	-2.316672	1	1	Y_RNA	_
ENSG00000200888	-2.796355	-2.316672	1	1	Y_RNA	_
ENSG00000200914	-2.796355	-2.316672	1	1	RNA5SP435	_
ENSG00000200972	-2.796355	-2.316672	1	1	RNU5A-8P	_
ENSG00000200985	-2.796355	-2.316672	1	1	RNA5SP493	_

ENSG00000201025	-2.796355	-2.316672	1	1	SNORD74	_
ENSG00000201077	-2.796355	-2.316672	1	1	RNU6-188P	_
ENSG00000201113	-2.796355	-2.316672	1	1	RNU6-647P	_
ENSG00000201217	-2.796355	-2.316672	1	1	Y_RNA	_
ENSG00000201239	-2.796355	-2.316672	1	1	Y_RNA	_
ENSG00000201407	-2.796355	-2.316672	1	1	SNORA68	_
ENSG00000201469	-2.796355	-2.316672	1	1	RNA5SP379	_
ENSG00000201470	-2.796355	-2.316672	1	1	RNY4P7	_
ENSG00000201492	-2.796355	-2.316672	1	1	RNA5SP78	_
ENSG00000201499	-2.796355	-2.316672	1	1	RNU6-312P	_
ENSG00000201573	-2.796355	-2.316672	1	1	Y_RNA	_
ENSG00000201622	-2.796355	-2.316672	1	1	RNU6-621P	_
ENSG00000201761	-2.796355	-2.316672	1	1	RNU6-336P	_
ENSG00000202031	-2.796355	-2.316672	1	1	SNORD38A	_
ENSG00000202272	-2.796355	-2.316672	1	1	Y_RNA	_
ENSG00000202279	-2.796355	-2.316672	1	1	Y_RNA	_
ENSG00000202318	-2.796355	-2.316672	1	1	Y_RNA	_
ENSG00000202537	-2.796355	-2.316672	1	1	U8	_
ENSG00000203315	-2.796355	-2.316672	1	1	AC015815.2	K15696
ENSG00000203403	-2.796355	-2.316672	1	1	AC020907.2	K09228
ENSG00000203428	-2.796355	-2.316672	1	1	AC140061.9	K14443
ENSG00000203563	-2.796355	-2.316672	1	1	AC138951.1	K13194
ENSG00000203632	-2.796355	-2.316672	1	1	AC007690.1	K05255
ENSG00000203722	-2.796355	-2.316672	1	1	RAET1G	K07987
ENSG00000203734	-2.796355	-2.316672	1	1	ECT2L	_
ENSG00000203814	-2.796355	-2.316672	1	1	HIST2H2BF	K11252
ENSG00000203930	-2.796355	-2.316672	1	1	LINC00632	_
ENSG00000204001	-2.796355	-2.316672	1	1	LCN8	_
ENSG00000204165	-2.796355	-2.316672	1	1	CXorf65	_
ENSG00000204195	-2.796355	-2.316672	1	1	AWAT1	K11171
ENSG00000204344	-2.796355	-2.316672	1	1	STK19	K08880
ENSG00000204446	-2.796355	-2.316672	1	1	C9orf170	_
ENSG00000204463	-2.796355	-2.316672	1	1	BAG6	_
ENSG00000204464	-2.796355	-2.316672	1	1	C1orf195	_
ENSG00000204533	-2.796355	-2.316672	1	1	AC024580.1	_
ENSG00000204711	-2.796355	-2.316672	1	1	C9orf135	K02981
ENSG00000204793	-2.796355	-2.316672	1	1	FOXD4L6	K09397
ENSG00000204928	-2.796355	-2.316672	1	1	GRXCR2	_
ENSG00000205041	-2.796355	-2.316672	1	1	CTC-425O23.2	_
ENSG00000205085	-2.796355	-2.316672	1	1	FAM71F2	_
ENSG00000205111	-2.796355	-2.316672	1	1	CDKL4	K08824
ENSG00000205181	-2.796355	-2.316672	1	1	LINC00654	_
ENSG00000205361	-2.796355	-2.316672	1	1	MT1DP	K14739
ENSG00000205482	-2.796355	-2.316672	1	1	AC007000.12	K08694
ENSG00000205500	-2.796355	-2.316672	1	1	AC013472.3	_
ENSG00000205592	-2.796355	-2.316672	1	1	MUC19	_
ENSG00000205595	-2.796355	-2.316672	1	1	AREGB	K09782
ENSG00000205644	-2.796355	-2.316672	1	1	CTD-2375G15.1	K02981
ENSG00000206127	-2.796355	-2.316672	1	1	GOLGA8O	K10595
ENSG00000206168	-2.796355	-2.316672	1	1	Z69890.1	_
ENSG00000206190	-2.796355	-2.316672	1	1	ATP10A	K01530
ENSG00000206384	-2.796355	-2.316672	1	1	COL6A6	K06238
ENSG00000206503	-2.796355	-2.316672	1	1	HLA-A	K06751
ENSG00000206645	-2.796355	-2.316672	1	1	Y_RNA	_
ENSG00000206647	-2.796355	-2.316672	1	1	SNORA2	_
ENSG00000206650	-2.796355	-2.316672	1	1	SNORA70G	_
ENSG00000206679	-2.796355	-2.316672	1	1	Y_RNA	_
ENSG00000206698	-2.796355	-2.316672	1	1	RNU1-73P	_

ENSG00000206739	-2.796355	-2.316672	1	1	Y_RNA	_
ENSG00000206814	-2.796355	-2.316672	1	1	Y_RNA	_
ENSG00000206824	-2.796355	-2.316672	1	1	Y_RNA	_
ENSG00000206838	-2.796355	-2.316672	1	1	SNORA5A	_
ENSG00000206897	-2.796355	-2.316672	1	1	SNORA9	_
ENSG00000206899	-2.796355	-2.316672	1	1	RNU6-36P	_
ENSG00000206921	-2.796355	-2.316672	1	1	RNU6-481P	_
ENSG00000206922	-2.796355	-2.316672	1	1	RNU6-80P	_
ENSG00000206937	-2.796355	-2.316672	1	1	SNORA70B	_
ENSG00000206952	-2.796355	-2.316672	1	1	SNORA50	_
ENSG00000206962	-2.796355	-2.316672	1	1	RNU6-74P	_
ENSG00000206969	-2.796355	-2.316672	1	1	RNU6-1316P	_
ENSG00000206995	-2.796355	-2.316672	1	1	Y_RNA	_
ENSG00000207036	-2.796355	-2.316672	1	1	Y_RNA	_
ENSG00000207052	-2.796355	-2.316672	1	1	RNU6-378P	_
ENSG00000207088	-2.796355	-2.316672	1	1	SNORA7B	_
ENSG00000207127	-2.796355	-2.316672	1	1	Y_RNA	_
ENSG00000207129	-2.796355	-2.316672	1	1	RNA5SP187	_
ENSG00000207171	-2.796355	-2.316672	1	1	SNORA51	_
ENSG00000207218	-2.796355	-2.316672	1	1	Y_RNA	_
ENSG00000207233	-2.796355	-2.316672	1	1	SNORA37	_
ENSG00000207240	-2.796355	-2.316672	1	1	Y_RNA	_
ENSG00000207297	-2.796355	-2.316672	1	1	SNORD7	_
ENSG00000207357	-2.796355	-2.316672	1	1	RNU6-2	_
ENSG00000207455	-2.796355	-2.316672	1	1	RNU6-331P	_
ENSG00000207512	-2.796355	-2.316672	1	1	Y_RNA	_
ENSG00000207609	-2.796355	-2.316672	1	1	MIR491	_
ENSG00000207612	-2.796355	-2.316672	1	1	MIR604	_
ENSG00000207614	-2.796355	-2.316672	1	1	MIR193A	_
ENSG00000207635	-2.796355	-2.316672	1	1	MIR499A	_
ENSG00000207693	-2.796355	-2.316672	1	1	MIR602	_
ENSG00000207709	-2.796355	-2.316672	1	1	MIR197	_
ENSG00000207736	-2.796355	-2.316672	1	1	MIR657	_
ENSG00000207737	-2.796355	-2.316672	1	1	MIR181B2	_
ENSG00000207751	-2.796355	-2.316672	1	1	MIR130B	_
ENSG00000207755	-2.796355	-2.316672	1	1	MIR450A2	_
ENSG00000207771	-2.796355	-2.316672	1	1	MIR550A1	_
ENSG00000207808	-2.796355	-2.316672	1	1	MIR27A	_
ENSG00000207814	-2.796355	-2.316672	1	1	MIR147A	_
ENSG00000207815	-2.796355	-2.316672	1	1	MIR563	_
ENSG00000207874	-2.796355	-2.316672	1	1	MIR610	_
ENSG00000207956	-2.796355	-2.316672	1	1	MIR579	_
ENSG00000208018	-2.796355	-2.316672	1	1	MIR645	_
ENSG00000211491	-2.796355	-2.316672	1	1	MIR320D1	_
ENSG00000211513	-2.796355	-2.316672	1	1	MIR320E	_
ENSG00000211892	-2.796355	-2.316672	1	1	IGHG4	K06856
ENSG00000211937	-2.796355	-2.316672	1	1	IGHV2-5	_
ENSG00000211997	-2.796355	-2.316672	1	1	MIR708	_
ENSG00000212432	-2.796355	-2.316672	1	1	SNORA75	_
ENSG00000212464	-2.796355	-2.316672	1	1	SNORA12	_
ENSG00000212569	-2.796355	-2.316672	1	1	Y_RNA	_
ENSG00000212572	-2.796355	-2.316672	1	1	RNU6-903P	_
ENSG00000212605	-2.796355	-2.316672	1	1	RNU1-56P	_
ENSG00000212643	-2.796355	-2.316672	1	1	ZRSR1	_
ENSG00000212930	-2.796355	-2.316672	1	1	RP11-414H23.2	_
ENSG00000213013	-2.796355	-2.316672	1	1	RPS15AP36	K02957
ENSG00000213041	-2.796355	-2.316672	1	1	RP11-383G10.3	K02880
ENSG00000213049	-2.796355	-2.316672	1	1	HNRNPA1P34	K12741

ENSG00000213055	-2.796355	-2.316672	1	1	AC064852.5	K03232
ENSG00000213090	-2.796355	-2.316672	1	1	AC007256.5	K17541
ENSG00000213104	-2.796355	-2.316672	1	1	NPM1P46	K11276
ENSG00000213111	-2.796355	-2.316672	1	1	COX5BP2	_
ENSG00000213169	-2.796355	-2.316672	1	1	RP11-393N4.2	K02938
ENSG00000213188	-2.796355	-2.316672	1	1	YBX1P4	K09276
ENSG00000213239	-2.796355	-2.316672	1	1	NPM1P32	K11276
ENSG00000213262	-2.796355	-2.316672	1	1	AL365331.2	K15040
ENSG00000213293	-2.796355	-2.316672	1	1	CTD-2666L21.3	K02903
ENSG00000213352	-2.796355	-2.316672	1	1	GAPDHP44	K00134
ENSG00000213434	-2.796355	-2.316672	1	1	VTI1BP2	K08493
ENSG00000213438	-2.796355	-2.316672	1	1	YBX2P1	K09277
ENSG00000213529	-2.796355	-2.316672	1	1	RP11-432F4.2	K02912
ENSG00000213540	-2.796355	-2.316672	1	1	RP11-486G15.1	K03671
ENSG00000213556	-2.796355	-2.316672	1	1	RP11-3J11.1	K02130
ENSG00000213592	-2.796355	-2.316672	1	1	AP000662.9	K03767
ENSG00000213671	-2.796355	-2.316672	1	1	OLA1P2	K06942
ENSG00000213750	-2.796355	-2.316672	1	1	KB-1683C8.1	K02981
ENSG00000213771	-2.796355	-2.316672	1	1	KRT8P37	K07605
ENSG00000213801	-2.796355	-2.316672	1	1	ZNF816-ZNF321P	K09228
ENSG00000213830	-2.796355	-2.316672	1	1	CFL1P5	K05765
ENSG00000213953	-2.796355	-2.316672	1	1	AC018867.1	_
ENSG00000214006	-2.796355	-2.316672	1	1	AL353671.4	K09228
ENSG00000214019	-2.796355	-2.316672	1	1	RP6-218J18.2	K13091
ENSG00000214081	-2.796355	-2.316672	1	1	CYP4F30P	K17726
ENSG00000214248	-2.796355	-2.316672	1	1	AC010336.1	K12581
ENSG00000214380	-2.796355	-2.316672	1	1	RP11-457K10.2	K08065
ENSG00000214401	-2.796355	-2.316672	1	1	KANSL1-AS1	_
ENSG00000214535	-2.796355	-2.316672	1	1	RPS15AP1	K02957
ENSG00000214597	-2.796355	-2.316672	1	1	TMEM249	_
ENSG00000214695	-2.796355	-2.316672	1	1	NPAP1P2	K08857
ENSG00000214733	-2.796355	-2.316672	1	1	RP11-429J17.8	_
ENSG00000214822	-2.796355	-2.316672	1	1	KRT16P3	K07604
ENSG00000214860	-2.796355	-2.316672	1	1	EVPLL	K10383
ENSG00000214871	-2.796355	-2.316672	1	1	AC005082.1	K12581
ENSG00000215009	-2.796355	-2.316672	1	1	ACSM4	K01896
ENSG00000215283	-2.796355	-2.316672	1	1	HMGB3P24	K11296
ENSG00000215284	-2.796355	-2.316672	1	1	RP11-198M15.1	K12198
ENSG00000215482	-2.796355	-2.316672	1	1	CALM2P3	K02183
ENSG00000215515	-2.796355	-2.316672	1	1	IFIT1P1	K14217
ENSG00000215533	-2.796355	-2.316672	1	1	LINC00189	_
ENSG00000215841	-2.796355	-2.316672	1	1	RP11-800A3.2	_
ENSG00000215866	-2.796355	-2.316672	1	1	RP11-426L16.8	_
ENSG00000215902	-2.796355	-2.316672	1	1	AL353354.1	_
ENSG00000215914	-2.796355	-2.316672	1	1	MMP23A	K08001
ENSG00000216115	-2.796355	-2.316672	1	1	AC017083.1	_
ENSG00000216141	-2.796355	-2.316672	1	1	MIR941-2	_
ENSG00000216475	-2.796355	-2.316672	1	1	RP1-91J24.1	K03111
ENSG00000217075	-2.796355	-2.316672	1	1	AC007401.2	_
ENSG00000217078	-2.796355	-2.316672	1	1	RP1-13D10.3	_
ENSG00000217195	-2.796355	-2.316672	1	1	RP11-277I20.2	_
ENSG00000217258	-2.796355	-2.316672	1	1	AC007249.3	_
ENSG00000217289	-2.796355	-2.316672	1	1	AC018865.5	_
ENSG00000217684	-2.796355	-2.316672	1	1	RPS3AP24	K02984
ENSG00000217862	-2.796355	-2.316672	1	1	HIST1H4PS1	_
ENSG00000217874	-2.796355	-2.316672	1	1	OR4F7P	K04257
ENSG00000218073	-2.796355	-2.316672	1	1	RP1-13D10.2	K10136
ENSG00000218274	-2.796355	-2.316672	1	1	RP3-407E4.3	_

ENSG00000218520	-2.796355	-2.316672	1	1	RP11-184C23.4	_
ENSG00000218682	-2.796355	-2.316672	1	1	AC010150.1	K01689
ENSG00000218896	-2.796355	-2.316672	1	1	TUBB8P2	K07375
ENSG00000219391	-2.796355	-2.316672	1	1	AC019129.1	K13126
ENSG00000219451	-2.796355	-2.316672	1	1	RPL23P8	K02894
ENSG00000219565	-2.796355	-2.316672	1	1	ZNF259P1	K06874
ENSG00000219608	-2.796355	-2.316672	1	1	HIGD1AP16	_
ENSG00000219699	-2.796355	-2.316672	1	1	RP11-325O24.2	K04508
ENSG00000219770	-2.796355	-2.316672	1	1	VN1R11P	K04614
ENSG00000219986	-2.796355	-2.316672	1	1	BTF3P7	K01527
ENSG00000220392	-2.796355	-2.316672	1	1	FCF1P5	_
ENSG00000220506	-2.796355	-2.316672	1	1	RP3-415N12.1	_
ENSG00000220563	-2.796355	-2.316672	1	1	PKMP3	K00873
ENSG00000220773	-2.796355	-2.316672	1	1	RP3-334F4.1	K02998
ENSG00000221020	-2.796355	-2.316672	1	1	AC107993.1	_
ENSG00000221044	-2.796355	-2.316672	1	1	U3	_
ENSG00000221063	-2.796355	-2.316672	1	1	MIR1296	_
ENSG00000221159	-2.796355	-2.316672	1	1	AL356741.1	_
ENSG00000221261	-2.796355	-2.316672	1	1	MIR1208	_
ENSG00000221439	-2.796355	-2.316672	1	1	RNU4ATAC16P	_
ENSG00000221520	-2.796355	-2.316672	1	1	MIR1285-1	_
ENSG00000221598	-2.796355	-2.316672	1	1	MIR1249	_
ENSG00000221681	-2.796355	-2.316672	1	1	AC092902.1	_
ENSG00000221740	-2.796355	-2.316672	1	1	SNORD93	_
ENSG00000221937	-2.796355	-2.316672	1	1	TAS2R40	K08474
ENSG00000222047	-2.796355	-2.316672	1	1	C10orf55	K01348
ENSG00000222092	-2.796355	-2.316672	1	1	RNU6-908P	_
ENSG00000222460	-2.796355	-2.316672	1	1	RN7SKP271	_
ENSG00000222705	-2.796355	-2.316672	1	1	RN7SKP39	_
ENSG00000222724	-2.796355	-2.316672	1	1	RNU2-63P	_
ENSG00000223003	-2.796355	-2.316672	1	1	RNA5SP184	_
ENSG00000223113	-2.796355	-2.316672	1	1	RNA5SP247	_
ENSG00000223138	-2.796355	-2.316672	1	1	RNA5SP450	_
ENSG00000223263	-2.796355	-2.316672	1	1	RNU6-387P	_
ENSG00000223284	-2.796355	-2.316672	1	1	RNU6-195P	_
ENSG00000223492	-2.796355	-2.316672	1	1	RP11-15M15.1	_
ENSG00000223505	-2.796355	-2.316672	1	1	RP11-397P13.7	K02917
ENSG00000223575	-2.796355	-2.316672	1	1	RBMX2P3	K13107
ENSG00000223634	-2.796355	-2.316672	1	1	AC012506.3	_
ENSG00000223718	-2.796355	-2.316672	1	1	AC093107.7	K02877
ENSG00000223724	-2.796355	-2.316672	1	1	RAD17P2	K06662
ENSG00000223806	-2.796355	-2.316672	1	1	LINC00114	_
ENSG00000223821	-2.796355	-2.316672	1	1	RP11-505P4.6	_
ENSG00000223829	-2.796355	-2.316672	1	1	AC004870.4	_
ENSG00000223864	-2.796355	-2.316672	1	1	NPM1P19	K11276
ENSG00000223873	-2.796355	-2.316672	1	1	AC013733.5	K14324
ENSG00000223956	-2.796355	-2.316672	1	1	RP4-710M16.2	_
ENSG00000223972	-2.796355	-2.316672	1	1	DDX11L1	K11273
ENSG00000224001	-2.796355	-2.316672	1	1	RP11-270F18.2	K07955
ENSG00000224049	-2.796355	-2.316672	1	1	RP11-30O15.1	_
ENSG00000224057	-2.796355	-2.316672	1	1	EGFR-AS1	_
ENSG00000224079	-2.796355	-2.316672	1	1	AC091729.7	_
ENSG00000224083	-2.796355	-2.316672	1	1	RP11-39K24.4	K02256
ENSG00000224085	-2.796355	-2.316672	1	1	RP11-4H14.1	K02893
ENSG00000224132	-2.796355	-2.316672	1	1	AC112715.2	_
ENSG00000224221	-2.796355	-2.316672	1	1	RPS6P8	_
ENSG00000224315	-2.796355	-2.316672	1	1	RPL7P7	K02937
ENSG00000224356	-2.796355	-2.316672	1	1	RP11-151A6.4	_

ENSG00000224429	-2.796355	-2.316672	1	1	LINC00422	_
ENSG00000224473	-2.796355	-2.316672	1	1	RP11-449I17.5	K10152
ENSG00000224514	-2.796355	-2.316672	1	1	LINC00620	_
ENSG00000224541	-2.796355	-2.316672	1	1	AP001439.2	_
ENSG00000224569	-2.796355	-2.316672	1	1	RP11-341G5.2	_
ENSG00000224648	-2.796355	-2.316672	1	1	RP11-397D12.4	_
ENSG00000224652	-2.796355	-2.316672	1	1	LINC00885	_
ENSG00000224729	-2.796355	-2.316672	1	1	PCOLCE-AS1	_
ENSG00000224733	-2.796355	-2.316672	1	1	RP1-86D1.5	_
ENSG00000224739	-2.796355	-2.316672	1	1	AC016735.1	_
ENSG00000224763	-2.796355	-2.316672	1	1	FDPSP8	K00787
ENSG00000224799	-2.796355	-2.316672	1	1	RP11-445O16.3	_
ENSG00000224820	-2.796355	-2.316672	1	1	BTF3P4	K01527
ENSG00000224838	-2.796355	-2.316672	1	1	FKSG52	_
ENSG00000224853	-2.796355	-2.316672	1	1	LINC00393	_
ENSG00000224940	-2.796355	-2.316672	1	1	PRRT4	_
ENSG00000224960	-2.796355	-2.316672	1	1	SMEK3P	K17491
ENSG00000225010	-2.796355	-2.316672	1	1	RP11-435B5.1	_
ENSG00000225062	-2.796355	-2.316672	1	1	AC021016.6	_
ENSG00000225098	-2.796355	-2.316672	1	1	BCRP1	K08878
ENSG00000225119	-2.796355	-2.316672	1	1	LINC00999	K18047
ENSG00000225131	-2.796355	-2.316672	1	1	PSME2P2	K06697
ENSG00000225133	-2.796355	-2.316672	1	1	MORF4L1P4	_
ENSG00000225163	-2.796355	-2.316672	1	1	LINC00618	_
ENSG00000225166	-2.796355	-2.316672	1	1	AC012462.2	_
ENSG00000225230	-2.796355	-2.316672	1	1	AC008937.3	_
ENSG00000225280	-2.796355	-2.316672	1	1	RP11-227D2.3	_
ENSG00000225364	-2.796355	-2.316672	1	1	ATP6V0E1P1	_
ENSG00000225401	-2.796355	-2.316672	1	1	TGIF2P1	K19553
ENSG00000225408	-2.796355	-2.316672	1	1	RP11-207C16.4	_
ENSG00000225420	-2.796355	-2.316672	1	1	AC104134.2	_
ENSG00000225486	-2.796355	-2.316672	1	1	RP11-301G21.1	K14394
ENSG00000225514	-2.796355	-2.316672	1	1	MTND1P34	_
ENSG00000225535	-2.796355	-2.316672	1	1	AC068610.3	_
ENSG00000225591	-2.796355	-2.316672	1	1	RP11-360D2.2	K02978
ENSG00000225667	-2.796355	-2.316672	1	1	LINC00505	_
ENSG00000225718	-2.796355	-2.316672	1	1	RP11-3P22.2	_
ENSG00000225744	-2.796355	-2.316672	1	1	AC017002.4	_
ENSG00000225751	-2.796355	-2.316672	1	1	AC087501.1	_
ENSG00000225792	-2.796355	-2.316672	1	1	AC004540.4	_
ENSG00000225798	-2.796355	-2.316672	1	1	AC025918.2	_
ENSG00000225854	-2.796355	-2.316672	1	1	RP11-569G9.7	K02915
ENSG00000225877	-2.796355	-2.316672	1	1	AC004603.4	_
ENSG00000225945	-2.796355	-2.316672	1	1	RP3-441A12.1	_
ENSG00000225963	-2.796355	-2.316672	1	1	AC009950.2	_
ENSG00000226008	-2.796355	-2.316672	1	1	AC073135.7	K16941
ENSG00000226026	-2.796355	-2.316672	1	1	RP11-57H12.3	_
ENSG00000226058	-2.796355	-2.316672	1	1	AC092570.1	K00237
ENSG00000226153	-2.796355	-2.316672	1	1	RP3-389A20.5	K17610
ENSG00000226156	-2.796355	-2.316672	1	1	RPL23AP76	_
ENSG00000226253	-2.796355	-2.316672	1	1	MRPL35P3	K02916
ENSG00000226258	-2.796355	-2.316672	1	1	GRM7-AS3	_
ENSG00000226262	-2.796355	-2.316672	1	1	PHKBP1	K07190
ENSG00000226291	-2.796355	-2.316672	1	1	AC091729.8	_
ENSG00000226428	-2.796355	-2.316672	1	1	RP11-106M7.4	K02877
ENSG00000226450	-2.796355	-2.316672	1	1	CYP2D8P	K17712
ENSG00000226465	-2.796355	-2.316672	1	1	RP13-401N8.1	_
ENSG00000226499	-2.796355	-2.316672	1	1	RP5-882O7.1	K02981

ENSG00000226505	-2.796355	-2.316672	1	1	AC016700.3	_
ENSG00000226520	-2.796355	-2.316672	1	1	KIRREL-IT1	_
ENSG00000226524	-2.796355	-2.316672	1	1	RP1-102H19.8	K02927
ENSG00000226549	-2.796355	-2.316672	1	1	SCDP1	K00507
ENSG00000226666	-2.796355	-2.316672	1	1	HSPA9P1	K04043
ENSG00000226673	-2.796355	-2.316672	1	1	LINC01108	_
ENSG00000226705	-2.796355	-2.316672	1	1	SDCBPP1	K17254
ENSG00000226813	-2.796355	-2.316672	1	1	AC114813.1	_
ENSG00000226820	-2.796355	-2.316672	1	1	RP11-747I9.1	K04210
ENSG00000226869	-2.796355	-2.316672	1	1	LHFPL3-AS1	_
ENSG00000226878	-2.796355	-2.316672	1	1	GSTM3P2	K00799
ENSG00000226889	-2.796355	-2.316672	1	1	RP11-474I16.8	_
ENSG00000226919	-2.796355	-2.316672	1	1	RP11-80B9.1	_
ENSG00000226926	-2.796355	-2.316672	1	1	AC010423.1	_
ENSG00000226948	-2.796355	-2.316672	1	1	RPS4XP2	K02987
ENSG00000226957	-2.796355	-2.316672	1	1	RP4-533D7.4	_
ENSG00000226964	-2.796355	-2.316672	1	1	RHEBP2	K07208
ENSG00000226973	-2.796355	-2.316672	1	1	RP4-663N10.2	K01380
ENSG00000226987	-2.796355	-2.316672	1	1	RP11-544A12.5	K02872
ENSG00000226991	-2.796355	-2.316672	1	1	AC112229.6	K09228
ENSG00000227045	-2.796355	-2.316672	1	1	RP11-98D18.1	_
ENSG00000227150	-2.796355	-2.316672	1	1	RP11-473E2.3	_
ENSG00000227155	-2.796355	-2.316672	1	1	RP11-165F24.3	_
ENSG00000227201	-2.796355	-2.316672	1	1	CNN2P1	_
ENSG00000227234	-2.796355	-2.316672	1	1	SPANXB2	_
ENSG00000227368	-2.796355	-2.316672	1	1	AC079753.5	K02208
ENSG00000227502	-2.796355	-2.316672	1	1	RP1-249H1.4	_
ENSG00000227527	-2.796355	-2.316672	1	1	RP11-223A3.1	_
ENSG00000227542	-2.796355	-2.316672	1	1	AC092614.2	_
ENSG00000227659	-2.796355	-2.316672	1	1	CLYBL-AS2	_
ENSG00000227676	-2.796355	-2.316672	1	1	LINC01068	_
ENSG00000227712	-2.796355	-2.316672	1	1	RP11-418J17.3	_
ENSG00000227729	-2.796355	-2.316672	1	1	RD3L	_
ENSG00000227744	-2.796355	-2.316672	1	1	AC114788.2	_
ENSG00000227765	-2.796355	-2.316672	1	1	RP11-162O12.2	K12757
ENSG00000227821	-2.796355	-2.316672	1	1	AL392086.1	_
ENSG00000227845	-2.796355	-2.316672	1	1	RP11-184B22.2	K09535
ENSG00000227852	-2.796355	-2.316672	1	1	RP11-156G14.6	K02980
ENSG00000227877	-2.796355	-2.316672	1	1	LINC00948	_
ENSG00000227879	-2.796355	-2.316672	1	1	PSPC1P1	_
ENSG00000227910	-2.796355	-2.316672	1	1	RP11-73B2.6	_
ENSG00000227962	-2.796355	-2.316672	1	1	RP11-382D8.3	K10575
ENSG00000228033	-2.796355	-2.316672	1	1	AC010967.2	_
ENSG00000228063	-2.796355	-2.316672	1	1	RP11-135J2.4	_
ENSG00000228069	-2.796355	-2.316672	1	1	RP11-100G15.3	K02262
ENSG00000228079	-2.796355	-2.316672	1	1	AC012368.1	_
ENSG00000228086	-2.796355	-2.316672	1	1	RP5-837M10.4	_
ENSG00000228089	-2.796355	-2.316672	1	1	RP1-197O17.2	_
ENSG00000228100	-2.796355	-2.316672	1	1	AC016912.3	_
ENSG00000228107	-2.796355	-2.316672	1	1	AP000692.9	_
ENSG00000228113	-2.796355	-2.316672	1	1	AC003991.3	_
ENSG00000228125	-2.796355	-2.316672	1	1	RP11-516A11.3	_
ENSG00000228149	-2.796355	-2.316672	1	1	RPL3P1	K02925
ENSG00000228153	-2.796355	-2.316672	1	1	RP11-23I7.1	_
ENSG00000228169	-2.796355	-2.316672	1	1	PPIAP19	K03767
ENSG00000228221	-2.796355	-2.316672	1	1	LINC00578	_
ENSG00000228242	-2.796355	-2.316672	1	1	AC093495.4	_
ENSG00000228292	-2.796355	-2.316672	1	1	RP11-220I1.4	K02889

ENSG00000228313	-2.796355	-2.316672	1	1	CTD-2555K7.4	_
ENSG00000228317	-2.796355	-2.316672	1	1	RP11-235C23.5	_
ENSG00000228330	-2.796355	-2.316672	1	1	RP11-730A19.5	K02257
ENSG00000228404	-2.796355	-2.316672	1	1	AP001468.58	_
ENSG00000228414	-2.796355	-2.316672	1	1	AC010733.4	_
ENSG00000228422	-2.796355	-2.316672	1	1	LINC00687	_
ENSG00000228546	-2.796355	-2.316672	1	1	CTA-313A17.3	K10858
ENSG00000228549	-2.796355	-2.316672	1	1	U1	_
ENSG00000228614	-2.796355	-2.316672	1	1	RP13-476E20.1	_
ENSG00000228625	-2.796355	-2.316672	1	1	RP11-1B20.1	_
ENSG00000228700	-2.796355	-2.316672	1	1	RP11-155G14.1	K02922
ENSG00000228729	-2.796355	-2.316672	1	1	RP11-211A18.2	_
ENSG00000228759	-2.796355	-2.316672	1	1	FAF2P1	K18726
ENSG00000228768	-2.796355	-2.316672	1	1	AC003101.1	_
ENSG00000228791	-2.796355	-2.316672	1	1	THRB-AS1	_
ENSG00000228808	-2.796355	-2.316672	1	1	HMGB3P4	_
ENSG00000228838	-2.796355	-2.316672	1	1	RP4-784A16.2	_
ENSG00000228972	-2.796355	-2.316672	1	1	RP1-86D1.2	_
ENSG00000229007	-2.796355	-2.316672	1	1	EXOSC3P1	K03681
ENSG00000229016	-2.796355	-2.316672	1	1	RP11-224O19.4	_
ENSG00000229018	-2.796355	-2.316672	1	1	RP11-313P13.3	K10858
ENSG00000229028	-2.796355	-2.316672	1	1	KRT223P	K07604
ENSG00000229044	-2.796355	-2.316672	1	1	RP11-266K22.2	_
ENSG00000229048	-2.796355	-2.316672	1	1	DUTP1	K01520
ENSG00000229081	-2.796355	-2.316672	1	1	LINC01165	_
ENSG00000229107	-2.796355	-2.316672	1	1	ABHD17AP4	_
ENSG00000229161	-2.796355	-2.316672	1	1	TCP1P1	K09493
ENSG00000229221	-2.796355	-2.316672	1	1	HNRNPA1P66	K12741
ENSG00000229228	-2.796355	-2.316672	1	1	LINC00582	_
ENSG00000229251	-2.796355	-2.316672	1	1	HNRNPA1P8	K12741
ENSG00000229256	-2.796355	-2.316672	1	1	ST13P13	K09560
ENSG00000229356	-2.796355	-2.316672	1	1	LRRC3-AS1	_
ENSG00000229368	-2.796355	-2.316672	1	1	AC090587.4	_
ENSG00000229372	-2.796355	-2.316672	1	1	SZT2-AS1	_
ENSG00000229393	-2.796355	-2.316672	1	1	RP3-395M20.3	_
ENSG00000229409	-2.796355	-2.316672	1	1	RP11-494O16.3	_
ENSG00000229414	-2.796355	-2.316672	1	1	KCNQ1-AS1	_
ENSG00000229427	-2.796355	-2.316672	1	1	ANKRD26P4	_
ENSG00000229473	-2.796355	-2.316672	1	1	RGS17P1	K16449
ENSG00000229491	-2.796355	-2.316672	1	1	RP11-342D14.1	_
ENSG00000229530	-2.796355	-2.316672	1	1	RP11-62C3.8	K08120
ENSG00000229569	-2.796355	-2.316672	1	1	RP11-481G8.2	_
ENSG00000229635	-2.796355	-2.316672	1	1	RP4-713B5.2	K03362
ENSG00000229637	-2.796355	-2.316672	1	1	PRAC2	_
ENSG00000229683	-2.796355	-2.316672	1	1	ASNSP5	K01953
ENSG00000229703	-2.796355	-2.316672	1	1	XX-CR54.1	_
ENSG00000229758	-2.796355	-2.316672	1	1	DYNLT3P2	K10420
ENSG00000229780	-2.796355	-2.316672	1	1	UBE2Q1-AS1	_
ENSG00000229786	-2.796355	-2.316672	1	1	SNRPFP2	K11098
ENSG00000229795	-2.796355	-2.316672	1	1	RPS21P1	K02971
ENSG00000229832	-2.796355	-2.316672	1	1	RP11-384C4.2	_
ENSG00000229893	-2.796355	-2.316672	1	1	AC004549.6	_
ENSG00000229894	-2.796355	-2.316672	1	1	GK3P	K00864
ENSG00000229920	-2.796355	-2.316672	1	1	RPS4XP5	K02987
ENSG00000229926	-2.796355	-2.316672	1	1	RP11-424E7.3	K05073
ENSG00000229954	-2.796355	-2.316672	1	1	MTND2P2	_
ENSG00000229964	-2.796355	-2.316672	1	1	RP11-135A1.3	_
ENSG00000229999	-2.796355	-2.316672	1	1	RP5-1042K10.10	_

ENSG00000230021	-2.796355	-2.316672	1	1	RP5-857K21.4	_
ENSG00000230058	-2.796355	-2.316672	1	1	RP11-172E9.2	_
ENSG00000230068	-2.796355	-2.316672	1	1	CDC42-IT1	_
ENSG00000230084	-2.796355	-2.316672	1	1	RP4-613B23.1	_
ENSG00000230092	-2.796355	-2.316672	1	1	RP11-206L10.8	K10305
ENSG00000230098	-2.796355	-2.316672	1	1	TCERG1L-AS1	_
ENSG00000230133	-2.796355	-2.316672	1	1	RP5-1100I6.2	_
ENSG00000230201	-2.796355	-2.316672	1	1	ATP6V0CP1	K02155
ENSG00000230207	-2.796355	-2.316672	1	1	RPL4P5	K02930
ENSG00000230289	-2.796355	-2.316672	1	1	RP11-334J6.6	_
ENSG00000230304	-2.796355	-2.316672	1	1	CICP6	K16941
ENSG00000230305	-2.796355	-2.316672	1	1	AC004980.9	K10858
ENSG00000230310	-2.796355	-2.316672	1	1	CTD-2192J16.11	_
ENSG00000230319	-2.796355	-2.316672	1	1	AL022476.2	_
ENSG00000230325	-2.796355	-2.316672	1	1	RP11-385F5.4	_
ENSG00000230359	-2.796355	-2.316672	1	1	TPI1P2	K01803
ENSG00000230368	-2.796355	-2.316672	1	1	FAM41C	_
ENSG00000230399	-2.796355	-2.316672	1	1	RBBP8P1	_
ENSG00000230426	-2.796355	-2.316672	1	1	ERVMER61-1	_
ENSG00000230454	-2.796355	-2.316672	1	1	U73166.2	_
ENSG00000230530	-2.796355	-2.316672	1	1	LIMD1-AS1	_
ENSG00000230561	-2.796355	-2.316672	1	1	CTC-228N24.1	_
ENSG00000230570	-2.796355	-2.316672	1	1	RP11-69L16.6	_
ENSG00000230585	-2.796355	-2.316672	1	1	PHBP12	K17080
ENSG00000230592	-2.796355	-2.316672	1	1	RPSAP8	K02998
ENSG00000230593	-2.796355	-2.316672	1	1	AC090804.1	K03767
ENSG00000230631	-2.796355	-2.316672	1	1	RP11-359N11.1	_
ENSG00000230676	-2.796355	-2.316672	1	1	RP11-65J3.3	_
ENSG00000230697	-2.796355	-2.316672	1	1	AC097635.4	K14324
ENSG00000230734	-2.796355	-2.316672	1	1	RPL10P3	K02866
ENSG00000230753	-2.796355	-2.316672	1	1	RP4-553F4.6	_
ENSG00000230773	-2.796355	-2.316672	1	1	AC079807.4	_
ENSG00000230787	-2.796355	-2.316672	1	1	PSAT1P3	K00831
ENSG00000230835	-2.796355	-2.316672	1	1	AP001187.11	_
ENSG00000230849	-2.796355	-2.316672	1	1	GOT2P2	K14455
ENSG00000230894	-2.796355	-2.316672	1	1	RP11-67K19.3	_
ENSG00000230902	-2.796355	-2.316672	1	1	FAM204CP	_
ENSG00000230939	-2.796355	-2.316672	1	1	RP11-314C16.1	_
ENSG00000231006	-2.796355	-2.316672	1	1	RPL7P32	K02937
ENSG00000231035	-2.796355	-2.316672	1	1	RPL7L1P9	_
ENSG00000231105	-2.796355	-2.316672	1	1	RP5-1071N3.1	_
ENSG00000231171	-2.796355	-2.316672	1	1	LINC01098	K11504
ENSG00000231172	-2.796355	-2.316672	1	1	AC007099.1	_
ENSG00000231173	-2.796355	-2.316672	1	1	AC116609.3	_
ENSG00000231181	-2.796355	-2.316672	1	1	RP13-15M17.1	K02940
ENSG00000231289	-2.796355	-2.316672	1	1	RP11-782C8.6	K02256
ENSG00000231341	-2.796355	-2.316672	1	1	VDAC1P6	K05862
ENSG00000231344	-2.796355	-2.316672	1	1	RP3-426I6.2	_
ENSG00000231367	-2.796355	-2.316672	1	1	AC016995.3	_
ENSG00000231383	-2.796355	-2.316672	1	1	FGF12-AS1	_
ENSG00000231416	-2.796355	-2.316672	1	1	RP11-422P24.9	K02915
ENSG00000231435	-2.796355	-2.316672	1	1	AC011747.3	_
ENSG00000231464	-2.796355	-2.316672	1	1	AC024937.4	K15425
ENSG00000231536	-2.796355	-2.316672	1	1	AC123886.2	_
ENSG00000231651	-2.796355	-2.316672	1	1	DLG3-AS1	_
ENSG00000231709	-2.796355	-2.316672	1	1	RP5-857K21.1	_
ENSG00000231816	-2.796355	-2.316672	1	1	RP4-782L23.1	_
ENSG00000231888	-2.796355	-2.316672	1	1	MTND5P15	K03883

ENSG00000231905	-2.796355	-2.316672	1	1	SETP10	K11290
ENSG00000231920	-2.796355	-2.316672	1	1	NEBL-AS1	_
ENSG00000231929	-2.796355	-2.316672	1	1	HMGB3P31	K11296
ENSG00000232063	-2.796355	-2.316672	1	1	RP11-307E17.8	_
ENSG00000232075	-2.796355	-2.316672	1	1	MRPL35P2	K02916
ENSG00000232218	-2.796355	-2.316672	1	1	RP1-149A16.16	_
ENSG00000232259	-2.796355	-2.316672	1	1	RP11-4C20.3	_
ENSG00000232260	-2.796355	-2.316672	1	1	BTF3L4P1	K01527
ENSG00000232270	-2.796355	-2.316672	1	1	INTS4L2	K13141
ENSG00000232439	-2.796355	-2.316672	1	1	RPL18AP7	K02882
ENSG00000232454	-2.796355	-2.316672	1	1	RP11-3J10.7	K02946
ENSG00000232504	-2.796355	-2.316672	1	1	AC105053.4	_
ENSG00000232536	-2.796355	-2.316672	1	1	RP11-74C1.4	_
ENSG00000232540	-2.796355	-2.316672	1	1	RPL36P19	_
ENSG00000232553	-2.796355	-2.316672	1	1	AC006026.10	K08823
ENSG00000232555	-2.796355	-2.316672	1	1	AC104088.1	_
ENSG00000232560	-2.796355	-2.316672	1	1	C21orf37	_
ENSG00000232571	-2.796355	-2.316672	1	1	RP11-435B5.2	K02256
ENSG00000232587	-2.796355	-2.316672	1	1	EEF1A1P3	K03231
ENSG00000232709	-2.796355	-2.316672	1	1	MARK2P9	_
ENSG00000232724	-2.796355	-2.316672	1	1	AC111186.1	_
ENSG00000232792	-2.796355	-2.316672	1	1	FTH1P25	K18495
ENSG00000232909	-2.796355	-2.316672	1	1	RP3-510O8.4	_
ENSG00000232938	-2.796355	-2.316672	1	1	AC139099.3	K02893
ENSG00000233010	-2.796355	-2.316672	1	1	RPEP4	K01783
ENSG00000233040	-2.796355	-2.316672	1	1	FAM204BP	_
ENSG00000233068	-2.796355	-2.316672	1	1	RP1-140K8.1	_
ENSG00000233072	-2.796355	-2.316672	1	1	RPS15AP6	K02957
ENSG00000233096	-2.796355	-2.316672	1	1	RP11-520A21.1	_
ENSG00000233220	-2.796355	-2.316672	1	1	LINC00167	_
ENSG00000233319	-2.796355	-2.316672	1	1	PPIAP32	K03767
ENSG00000233327	-2.796355	-2.316672	1	1	USP32P2	K11837
ENSG00000233330	-2.796355	-2.316672	1	1	RP1-12G14.7	_
ENSG00000233401	-2.796355	-2.316672	1	1	PRKAR1AP	K04739
ENSG00000233455	-2.796355	-2.316672	1	1	RP4-667H12.4	K03469
ENSG00000233590	-2.796355	-2.316672	1	1	RP11-153K11.3	_
ENSG00000233601	-2.796355	-2.316672	1	1	NCOR1P3	K04650
ENSG00000233614	-2.796355	-2.316672	1	1	DDX11L10	K11273
ENSG00000233647	-2.796355	-2.316672	1	1	RP11-8J9.1	_
ENSG00000233671	-2.796355	-2.316672	1	1	AC083899.1	K11506
ENSG00000233775	-2.796355	-2.316672	1	1	RP4-811H24.9	_
ENSG00000233845	-2.796355	-2.316672	1	1	AC093732.1	_
ENSG00000233929	-2.796355	-2.316672	1	1	MT1XP1	K14739
ENSG00000233996	-2.796355	-2.316672	1	1	AC013439.4	K15601
ENSG00000234004	-2.796355	-2.316672	1	1	RP11-543B16.1	K02975
ENSG00000234030	-2.796355	-2.316672	1	1	TMEM97P1	_
ENSG00000234041	-2.796355	-2.316672	1	1	RP11-345I18.1	_
ENSG00000234065	-2.796355	-2.316672	1	1	MTND4P26	_
ENSG00000234093	-2.796355	-2.316672	1	1	RPS15AP11	K02957
ENSG00000234185	-2.796355	-2.316672	1	1	RP5-1132H15.1	_
ENSG00000234193	-2.796355	-2.316672	1	1	AC097461.4	_
ENSG00000234197	-2.796355	-2.316672	1	1	ETV5-AS1	_
ENSG00000234222	-2.796355	-2.316672	1	1	RP11-315I20.1	_
ENSG00000234252	-2.796355	-2.316672	1	1	AC007308.6	_
ENSG00000234263	-2.796355	-2.316672	1	1	RP3-325F22.3	_
ENSG00000234332	-2.796355	-2.316672	1	1	BCAS2P2	K12861
ENSG00000234377	-2.796355	-2.316672	1	1	RNF219-AS1	_
ENSG00000234428	-2.796355	-2.316672	1	1	RP11-666F17.1	_

ENSG00000234465	-2.796355	-2.316672	1	1	PINLYP	_
ENSG00000234520	-2.796355	-2.316672	1	1	AC018464.3	_
ENSG00000234536	-2.796355	-2.316672	1	1	AC096582.7	K13051
ENSG00000234548	-2.796355	-2.316672	1	1	RP11-78H24.1	_
ENSG00000234556	-2.796355	-2.316672	1	1	LINC00701	_
ENSG00000234589	-2.796355	-2.316672	1	1	RP11-253E3.1	K02872
ENSG00000234610	-2.796355	-2.316672	1	1	LINC00624	_
ENSG00000234650	-2.796355	-2.316672	1	1	PCCA-AS1	_
ENSG00000234693	-2.796355	-2.316672	1	1	RP4-530I15.6	_
ENSG00000234711	-2.796355	-2.316672	1	1	TUBB8P11	K07375
ENSG00000234719	-2.796355	-2.316672	1	1	RP11-166B2.1	_
ENSG00000234782	-2.796355	-2.316672	1	1	TPT1P9	_
ENSG00000234788	-2.796355	-2.316672	1	1	HSPA8P3	_
ENSG00000234793	-2.796355	-2.316672	1	1	AC114730.7	_
ENSG00000234805	-2.796355	-2.316672	1	1	AC090505.5	_
ENSG00000234806	-2.796355	-2.316672	1	1	RP11-9E13.4	K02898
ENSG00000234832	-2.796355	-2.316672	1	1	RP3-322G13.7	_
ENSG00000234999	-2.796355	-2.316672	1	1	AC004016.2	K11095
ENSG00000235008	-2.796355	-2.316672	1	1	RP3-497J21.1	K13197
ENSG00000235021	-2.796355	-2.316672	1	1	RP11-439E19.7	_
ENSG00000235028	-2.796355	-2.316672	1	1	HMGN1P30	K11299
ENSG00000235045	-2.796355	-2.316672	1	1	RPL7P8	K02937
ENSG00000235065	-2.796355	-2.316672	1	1	RPL24P2	K02896
ENSG00000235072	-2.796355	-2.316672	1	1	AC012074.2	_
ENSG00000235085	-2.796355	-2.316672	1	1	AC091153.4	_
ENSG00000235129	-2.796355	-2.316672	1	1	FABP7P2	K08756
ENSG00000235185	-2.796355	-2.316672	1	1	RP5-1056L3.1	_
ENSG00000235213	-2.796355	-2.316672	1	1	OR6E1P	K04257
ENSG00000235263	-2.796355	-2.316672	1	1	RP13-392I16.1	_
ENSG00000235297	-2.796355	-2.316672	1	1	FAUP1	K02983
ENSG00000235321	-2.796355	-2.316672	1	1	AC007556.3	_
ENSG00000235328	-2.796355	-2.316672	1	1	AC006946.12	K02910
ENSG00000235411	-2.796355	-2.316672	1	1	AC007041.2	_
ENSG00000235413	-2.796355	-2.316672	1	1	KRT18P63	K07604
ENSG00000235480	-2.796355	-2.316672	1	1	RP11-363D14.1	_
ENSG00000235613	-2.796355	-2.316672	1	1	NSRP1P1	K13206
ENSG00000235674	-2.796355	-2.316672	1	1	LDHAP2	K00016
ENSG00000235700	-2.796355	-2.316672	1	1	CYCSP52	K08738
ENSG00000235733	-2.796355	-2.316672	1	1	RP3-522P13.3	_
ENSG00000235776	-2.796355	-2.316672	1	1	AC000089.3	K02936
ENSG00000235840	-2.796355	-2.316672	1	1	AC012363.13	_
ENSG00000235843	-2.796355	-2.316672	1	1	RP13-16H11.5	_
ENSG00000235848	-2.796355	-2.316672	1	1	RMDN2-AS1	_
ENSG00000235861	-2.796355	-2.316672	1	1	AC005237.2	_
ENSG00000235862	-2.796355	-2.316672	1	1	RP11-338C15.5	_
ENSG00000235884	-2.796355	-2.316672	1	1	LINC00941	_
ENSG00000235885	-2.796355	-2.316672	1	1	AC023115.2	_
ENSG00000235903	-2.796355	-2.316672	1	1	CPB2-AS1	_
ENSG00000236060	-2.796355	-2.316672	1	1	HSPB1P1	K04455
ENSG00000236086	-2.796355	-2.316672	1	1	HMGN2P28	K11300
ENSG00000236110	-2.796355	-2.316672	1	1	OR2AM1P	K17461
ENSG00000236119	-2.796355	-2.316672	1	1	AP000688.15	_
ENSG00000236159	-2.796355	-2.316672	1	1	RP3-343K2.4	_
ENSG00000236229	-2.796355	-2.316672	1	1	VEZF1P1	_
ENSG00000236238	-2.796355	-2.316672	1	1	AC093106.4	K02955
ENSG00000236254	-2.796355	-2.316672	1	1	MTND4P14	K03881
ENSG00000236296	-2.796355	-2.316672	1	1	GUSBP5	K01195
ENSG00000236297	-2.796355	-2.316672	1	1	RP11-175P19.2	K03231

ENSG00000236299	-2.796355	-2.316672	1	1	RP11-340I6.7	_
ENSG00000236317	-2.796355	-2.316672	1	1	RP11-348H3.5	K02979
ENSG00000236324	-2.796355	-2.316672	1	1	RP3-403L10.3	_
ENSG00000236391	-2.796355	-2.316672	1	1	AC092573.2	K02271
ENSG00000236427	-2.796355	-2.316672	1	1	RP11-107M16.2	_
ENSG00000236438	-2.796355	-2.316672	1	1	FAM157A	_
ENSG00000236456	-2.796355	-2.316672	1	1	RP4-785G19.2	_
ENSG00000236489	-2.796355	-2.316672	1	1	RP11-537I16.2	K02915
ENSG00000236542	-2.796355	-2.316672	1	1	MED28P7	K15141
ENSG00000236548	-2.796355	-2.316672	1	1	RP11-510H23.1	_
ENSG00000236609	-2.796355	-2.316672	1	1	ZNF853	K09228
ENSG00000236677	-2.796355	-2.316672	1	1	AP000688.11	_
ENSG00000236780	-2.796355	-2.316672	1	1	AC078941.1	_
ENSG00000236804	-2.796355	-2.316672	1	1	RPS3AP12	K02984
ENSG00000236811	-2.796355	-2.316672	1	1	GAPDHP2	K00134
ENSG00000236856	-2.796355	-2.316672	1	1	AC105393.1	_
ENSG00000236869	-2.796355	-2.316672	1	1	RP11-944L7.4	_
ENSG00000236876	-2.796355	-2.316672	1	1	TMSB4XP1	K05764
ENSG00000236892	-2.796355	-2.316672	1	1	RP11-482E14.2	_
ENSG00000236896	-2.796355	-2.316672	1	1	RP11-535C21.3	_
ENSG00000236905	-2.796355	-2.316672	1	1	RP11-348H3.2	K02989
ENSG00000236953	-2.796355	-2.316672	1	1	ZDHHC20-IT1	_
ENSG00000237119	-2.796355	-2.316672	1	1	LINC01056	_
ENSG00000237166	-2.796355	-2.316672	1	1	AC007163.3	_
ENSG00000237174	-2.796355	-2.316672	1	1	RP11-415D17.3	_
ENSG00000237178	-2.796355	-2.316672	1	1	AC019080.1	K11253
ENSG00000237200	-2.796355	-2.316672	1	1	ZBTB40-IT1	_
ENSG00000237212	-2.796355	-2.316672	1	1	RP11-569G13.2	_
ENSG00000237260	-2.796355	-2.316672	1	1	AC073069.2	K03767
ENSG00000237280	-2.796355	-2.316672	1	1	RP11-96C23.9	_
ENSG00000237281	-2.796355	-2.316672	1	1	AC021016.8	_
ENSG00000237283	-2.796355	-2.316672	1	1	RP11-316I3.1	_
ENSG00000237291	-2.796355	-2.316672	1	1	RP11-782C8.4	_
ENSG00000237331	-2.796355	-2.316672	1	1	XIAP-AS1	_
ENSG00000237404	-2.796355	-2.316672	1	1	RP3-471C18.2	_
ENSG00000237543	-2.796355	-2.316672	1	1	RP11-58A12.3	_
ENSG00000237551	-2.796355	-2.316672	1	1	AC096775.2	K02889
ENSG00000237641	-2.796355	-2.316672	1	1	RP11-690I21.1	_
ENSG00000237664	-2.796355	-2.316672	1	1	LINC00316	_
ENSG00000237668	-2.796355	-2.316672	1	1	RPS15AP38	K02957
ENSG00000237729	-2.796355	-2.316672	1	1	AC002075.4	K02978
ENSG00000237732	-2.796355	-2.316672	1	1	AC010980.2	K09381
ENSG00000237768	-2.796355	-2.316672	1	1	RP11-344N10.2	_
ENSG00000237772	-2.796355	-2.316672	1	1	AC092620.3	_
ENSG00000237827	-2.796355	-2.316672	1	1	RP11-332O19.2	K02957
ENSG00000237840	-2.796355	-2.316672	1	1	FAM21FP	K18462
ENSG00000237852	-2.796355	-2.316672	1	1	RP4-630A11.3	_
ENSG00000237868	-2.796355	-2.316672	1	1	AC097533.1	K10666
ENSG00000237899	-2.796355	-2.316672	1	1	RP4-739H11.3	_
ENSG00000237927	-2.796355	-2.316672	1	1	RP3-393E18.2	_
ENSG00000237982	-2.796355	-2.316672	1	1	RP11-815I22.1	_
ENSG00000238009	-2.796355	-2.316672	1	1	RP11-34P13.7	_
ENSG00000238015	-2.796355	-2.316672	1	1	RP4-641G12.3	K02894
ENSG00000238043	-2.796355	-2.316672	1	1	RP11-699L21.2	_
ENSG00000238049	-2.796355	-2.316672	1	1	LL0XNC01-105G4.3	_
ENSG00000238097	-2.796355	-2.316672	1	1	RP11-513G11.3	_
ENSG00000238113	-2.796355	-2.316672	1	1	RP11-262H14.1	_
ENSG00000238122	-2.796355	-2.316672	1	1	RP11-483I13.2	_

ENSG00000238160	-2.796355	-2.316672	1	1	AC116366.5	_
ENSG00000238165	-2.796355	-2.316672	1	1	AC007560.1	_
ENSG00000238178	-2.796355	-2.316672	1	1	RP11-431J24.2	_
ENSG00000238181	-2.796355	-2.316672	1	1	AHCYP2	K01251
ENSG00000238199	-2.796355	-2.316672	1	1	UBE2V2P3	K10704
ENSG00000238220	-2.796355	-2.316672	1	1	AP000275.64	_
ENSG00000238244	-2.796355	-2.316672	1	1	GABARAPL3	K08341
ENSG00000238329	-2.796355	-2.316672	1	1	snoU13	_
ENSG00000238349	-2.796355	-2.316672	1	1	snoU13	_
ENSG00000238364	-2.796355	-2.316672	1	1	RNU7-140P	_
ENSG00000238541	-2.796355	-2.316672	1	1	snoU13	_
ENSG00000238569	-2.796355	-2.316672	1	1	snoU13	_
ENSG00000238683	-2.796355	-2.316672	1	1	snoU13	_
ENSG00000238777	-2.796355	-2.316672	1	1	RNU7-141P	_
ENSG00000238829	-2.796355	-2.316672	1	1	RNU7-45P	_
ENSG00000238835	-2.796355	-2.316672	1	1	SCARNA18	_
ENSG00000238886	-2.796355	-2.316672	1	1	SNORD121A	_
ENSG00000239035	-2.796355	-2.316672	1	1	snoU13	_
ENSG00000239043	-2.796355	-2.316672	1	1	SNORD127	_
ENSG00000239052	-2.796355	-2.316672	1	1	snoU13	_
ENSG00000239059	-2.796355	-2.316672	1	1	snoU13	_
ENSG00000239096	-2.796355	-2.316672	1	1	snoU13	_
ENSG00000239133	-2.796355	-2.316672	1	1	snoU13	_
ENSG00000239218	-2.796355	-2.316672	1	1	RPS20P22	K02969
ENSG00000239247	-2.796355	-2.316672	1	1	RN7SL589P	_
ENSG00000239474	-2.796355	-2.316672	1	1	KLHL41	K10473
ENSG00000239524	-2.796355	-2.316672	1	1	RPL32P34	K02912
ENSG00000239593	-2.796355	-2.316672	1	1	RP11-477J21.6	_
ENSG00000239627	-2.796355	-2.316672	1	1	RP11-124A7.1	K02870
ENSG00000239670	-2.796355	-2.316672	1	1	RP4-803A2.2	K02981
ENSG00000239791	-2.796355	-2.316672	1	1	AC002310.7	_
ENSG00000239830	-2.796355	-2.316672	1	1	RPS4XP22	K02987
ENSG00000239868	-2.796355	-2.316672	1	1	RN7SL34P	_
ENSG00000239908	-2.796355	-2.316672	1	1	RN7SL75P	_
ENSG00000240074	-2.796355	-2.316672	1	1	RPL9P30	K02940
ENSG00000240137	-2.796355	-2.316672	1	1	RP11-103G8.2	_
ENSG00000240233	-2.796355	-2.316672	1	1	RN7SL587P	_
ENSG00000240280	-2.796355	-2.316672	1	1	TCAM1P	_
ENSG00000240366	-2.796355	-2.316672	1	1	RPL36AP45	K02929
ENSG00000240418	-2.796355	-2.316672	1	1	CTD-2013N17.1	K02981
ENSG00000240436	-2.796355	-2.316672	1	1	RP11-95I19.1	K02917
ENSG00000240476	-2.796355	-2.316672	1	1	LINC00973	_
ENSG00000240549	-2.796355	-2.316672	1	1	THOC7-AS1	_
ENSG00000240775	-2.796355	-2.316672	1	1	RP11-170N16.2	K02976
ENSG00000240793	-2.796355	-2.316672	1	1	UBA52P8	K02927
ENSG00000240877	-2.796355	-2.316672	1	1	RN7SL521P	_
ENSG00000240915	-2.796355	-2.316672	1	1	RP11-659E9.2	_
ENSG00000240950	-2.796355	-2.316672	1	1	RP11-372E1.1	K02938
ENSG00000240966	-2.796355	-2.316672	1	1	RN7SL681P	_
ENSG00000240974	-2.796355	-2.316672	1	1	CTD-2024I7.1	K02977
ENSG00000241011	-2.796355	-2.316672	1	1	RP11-713C19.1	_
ENSG00000241026	-2.796355	-2.316672	1	1	CTD-2036J7.1	K02889
ENSG00000241035	-2.796355	-2.316672	1	1	RP11-689J19.1	K02927
ENSG00000241084	-2.796355	-2.316672	1	1	RP11-344B5.3	_
ENSG00000241111	-2.796355	-2.316672	1	1	RP11-129B22.1	_
ENSG00000241456	-2.796355	-2.316672	1	1	RP4-555L14.4	_
ENSG00000241489	-2.796355	-2.316672	1	1	IDS	K01136
ENSG00000241494	-2.796355	-2.316672	1	1	RP11-796G6.1	K02898

ENSG00000241570	-2.796355	-2.316672	1	1	RP11-372E1.6	_
ENSG00000241641	-2.796355	-2.316672	1	1	RPS23P6	_
ENSG00000241671	-2.796355	-2.316672	1	1	RP11-529G21.3	_
ENSG00000241765	-2.796355	-2.316672	1	1	RP11-159A18.1	K02976
ENSG00000241947	-2.796355	-2.316672	1	1	HNRNPA1P24	K12741
ENSG00000241959	-2.796355	-2.316672	1	1	RN7SL76P	_
ENSG00000241991	-2.796355	-2.316672	1	1	RP11-95I19.2	_
ENSG00000242104	-2.796355	-2.316672	1	1	RP11-340E6.1	_
ENSG00000242180	-2.796355	-2.316672	1	1	OR51B5	K04257
ENSG00000242258	-2.796355	-2.316672	1	1	LINC00996	_
ENSG00000242399	-2.796355	-2.316672	1	1	RPS20P23	_
ENSG00000242593	-2.796355	-2.316672	1	1	RP5-921G16.1	_
ENSG00000242607	-2.796355	-2.316672	1	1	RPS3AP34	K02984
ENSG00000242737	-2.796355	-2.316672	1	1	RP11-562A8.1	K02969
ENSG00000243004	-2.796355	-2.316672	1	1	AC005062.2	_
ENSG00000243116	-2.796355	-2.316672	1	1	RP11-564C24.1	_
ENSG00000243225	-2.796355	-2.316672	1	1	RP11-7F17.1	K02942
ENSG00000243333	-2.796355	-2.316672	1	1	RN7SL174P	_
ENSG00000243414	-2.796355	-2.316672	1	1	TICAM2	K14825
ENSG00000243508	-2.796355	-2.316672	1	1	RP11-775J23.2	K09512
ENSG00000243607	-2.796355	-2.316672	1	1	RPL35AP26	K02917
ENSG00000243649	-2.796355	-2.316672	1	1	CFB	K01335
ENSG00000243746	-2.796355	-2.316672	1	1	EEF1A1P10	K03231
ENSG00000243795	-2.796355	-2.316672	1	1	RP11-572M11.3	_
ENSG00000243797	-2.796355	-2.316672	1	1	CTB-111H14.1	_
ENSG00000243859	-2.796355	-2.316672	1	1	RPL5P17	K02932
ENSG00000243977	-2.796355	-2.316672	1	1	RP11-473P24.2	K11253
ENSG00000244104	-2.796355	-2.316672	1	1	RN7SL659P	_
ENSG00000244128	-2.796355	-2.316672	1	1	RP11-85M11.2	_
ENSG00000244307	-2.796355	-2.316672	1	1	RN7SL395P	_
ENSG00000244468	-2.796355	-2.316672	1	1	RP11-206M11.7	_
ENSG00000244491	-2.796355	-2.316672	1	1	RP3-508I15.18	_
ENSG00000244527	-2.796355	-2.316672	1	1	RP11-254G11.1	K02889
ENSG00000244588	-2.796355	-2.316672	1	1	RAD21L1	_
ENSG00000244618	-2.796355	-2.316672	1	1	RN7SL334P	_
ENSG00000244998	-2.796355	-2.316672	1	1	CTD-3064M3.4	_
ENSG00000245025	-2.796355	-2.316672	1	1	RP11-875O11.1	_
ENSG00000245598	-2.796355	-2.316672	1	1	DACT3-AS1	_
ENSG00000245719	-2.796355	-2.316672	1	1	RP11-34F13.2	_
ENSG00000246211	-2.796355	-2.316672	1	1	RP11-632K5.3	_
ENSG00000246763	-2.796355	-2.316672	1	1	RGMB-AS1	_
ENSG00000248079	-2.796355	-2.316672	1	1	DPH6-AS1	_
ENSG00000248156	-2.796355	-2.316672	1	1	RP11-12P19.1	K14443
ENSG00000248172	-2.796355	-2.316672	1	1	RP11-1094H24.3	_
ENSG00000248196	-2.796355	-2.316672	1	1	RP11-476C8.2	K03648
ENSG00000248334	-2.796355	-2.316672	1	1	WHAMMP2	K09488
ENSG00000248346	-2.796355	-2.316672	1	1	RP11-292D4.1	_
ENSG00000248405	-2.796355	-2.316672	1	1	PRR5-ARHGAP8	K18470
ENSG00000248491	-2.796355	-2.316672	1	1	RP11-125O18.1	_
ENSG00000248514	-2.796355	-2.316672	1	1	CTC-338M12.1	_
ENSG00000248546	-2.796355	-2.316672	1	1	ANP32C	K18646
ENSG00000248571	-2.796355	-2.316672	1	1	RP11-768B22.2	_
ENSG00000248656	-2.796355	-2.316672	1	1	RP11-255I10.2	_
ENSG00000248663	-2.796355	-2.316672	1	1	LINC00992	_
ENSG00000248729	-2.796355	-2.316672	1	1	RP11-531A21.3	_
ENSG00000248752	-2.796355	-2.316672	1	1	RP11-114J13.1	_
ENSG00000248753	-2.796355	-2.316672	1	1	CTC-276P9.2	_
ENSG00000248757	-2.796355	-2.316672	1	1	CTD-2193G5.1	_

ENSG00000248925	-2.796355	-2.316672	1	1	CTD-2083E4.6	_
ENSG00000248936	-2.796355	-2.316672	1	1	RP11-36B15.1	_
ENSG00000248950	-2.796355	-2.316672	1	1	LDHBP3	_
ENSG00000249012	-2.796355	-2.316672	1	1	RP11-731D1.1	_
ENSG00000249020	-2.796355	-2.316672	1	1	SNORA58	_
ENSG00000249031	-2.796355	-2.316672	1	1	SUMO2P6	K12160
ENSG00000249047	-2.796355	-2.316672	1	1	COX6B1P5	K02267
ENSG00000249068	-2.796355	-2.316672	1	1	CTC-287O8.1	K01231
ENSG00000249201	-2.796355	-2.316672	1	1	CTD-3080P12.3	_
ENSG00000249230	-2.796355	-2.316672	1	1	CDH12P2	K06804
ENSG00000249346	-2.796355	-2.316672	1	1	LINC01016	_
ENSG00000249359	-2.796355	-2.316672	1	1	RP11-374A4.1	_
ENSG00000249388	-2.796355	-2.316672	1	1	RP11-834C11.6	_
ENSG00000249396	-2.796355	-2.316672	1	1	RP11-1C1.4	_
ENSG00000249412	-2.796355	-2.316672	1	1	CTC-563A5.2	_
ENSG00000249465	-2.796355	-2.316672	1	1	RBMXP4	K12885
ENSG00000249503	-2.796355	-2.316672	1	1	HMGN1P16	_
ENSG00000249540	-2.796355	-2.316672	1	1	RP11-789L4.1	K12834
ENSG00000249609	-2.796355	-2.316672	1	1	RP11-635L1.3	_
ENSG00000249624	-2.796355	-2.316672	1	1	AP000295.9	K05135
ENSG00000249678	-2.796355	-2.316672	1	1	RP11-619J20.1	_
ENSG00000249753	-2.796355	-2.316672	1	1	RP11-415I12.3	_
ENSG00000249771	-2.796355	-2.316672	1	1	RP11-457P14.5	_
ENSG00000249831	-2.796355	-2.316672	1	1	RP11-4O3.2	_
ENSG00000249870	-2.796355	-2.316672	1	1	RP11-481C4.1	_
ENSG00000249934	-2.796355	-2.316672	1	1	RP11-466G12.3	K09289
ENSG00000249986	-2.796355	-2.316672	1	1	YWHAQP6	K16197
ENSG00000250050	-2.796355	-2.316672	1	1	MTND4P9	_
ENSG00000250075	-2.796355	-2.316672	1	1	RP11-584P21.2	_
ENSG00000250106	-2.796355	-2.316672	1	1	ANKRD33B-AS1	_
ENSG00000250111	-2.796355	-2.316672	1	1	AC107982.4	K17299
ENSG00000250241	-2.796355	-2.316672	1	1	RP11-9G1.3	_
ENSG00000250326	-2.796355	-2.316672	1	1	RP11-284M14.1	_
ENSG00000250330	-2.796355	-2.316672	1	1	CTC-422A18.2	K08341
ENSG00000250360	-2.796355	-2.316672	1	1	CTD-2089N3.1	_
ENSG00000250496	-2.796355	-2.316672	1	1	ABT1P1	K14785
ENSG00000250568	-2.796355	-2.316672	1	1	RP11-333E13.2	K13126
ENSG00000250599	-2.796355	-2.316672	1	1	AC064853.3	K14610
ENSG00000250624	-2.796355	-2.316672	1	1	RP11-93M12.1	_
ENSG00000250748	-2.796355	-2.316672	1	1	RP11-230G5.2	_
ENSG00000250762	-2.796355	-2.316672	1	1	RP1-313L4.4	_
ENSG00000250769	-2.796355	-2.316672	1	1	RP11-241F15.3	_
ENSG00000250869	-2.796355	-2.316672	1	1	RP11-453N18.1	K17985
ENSG00000250878	-2.796355	-2.316672	1	1	METTL21EP	_
ENSG00000250950	-2.796355	-2.316672	1	1	RP11-33B1.3	_
ENSG00000250961	-2.796355	-2.316672	1	1	CTD-2023N9.1	_
ENSG00000250978	-2.796355	-2.316672	1	1	RP11-357D18.1	_
ENSG00000250990	-2.796355	-2.316672	1	1	AC073635.5	_
ENSG00000251055	-2.796355	-2.316672	1	1	RP11-257A22.1	_
ENSG00000251164	-2.796355	-2.316672	1	1	HULC	_
ENSG00000251168	-2.796355	-2.316672	1	1	CTD-2072I24.1	_
ENSG00000251203	-2.796355	-2.316672	1	1	RP11-14I17.1	K09512
ENSG00000251205	-2.796355	-2.316672	1	1	CTD-2050E21.2	_
ENSG00000251258	-2.796355	-2.316672	1	1	RFPL4B	_
ENSG00000251357	-2.796355	-2.316672	1	1	AP000350.10	K08148
ENSG00000251361	-2.796355	-2.316672	1	1	CTD-2091N23.1	_
ENSG00000251435	-2.796355	-2.316672	1	1	RP11-365H8.2	K00731
ENSG00000251441	-2.796355	-2.316672	1	1	RTEL1P1	K11136

ENSG00000251451	-2.796355	-2.316672	1	1	GS1-124K5.6	K03121
ENSG00000251467	-2.796355	-2.316672	1	1	CTC-250P20.2	K17617
ENSG00000251533	-2.796355	-2.316672	1	1	LINC00605	_
ENSG00000251544	-2.796355	-2.316672	1	1	MTND5P12	K03883
ENSG00000251620	-2.796355	-2.316672	1	1	STPG2-AS1	_
ENSG00000251634	-2.796355	-2.316672	1	1	RP11-1280N14.3	K12807
ENSG00000251648	-2.796355	-2.316672	1	1	CTD-2353N24.1	_
ENSG00000251679	-2.796355	-2.316672	1	1	RP11-669M16.2	_
ENSG00000251827	-2.796355	-2.316672	1	1	RN7SKP215	_
ENSG00000251985	-2.796355	-2.316672	1	1	RNU6-1161P	_
ENSG00000251991	-2.796355	-2.316672	1	1	RNU7-49P	_
ENSG00000252157	-2.796355	-2.316672	1	1	RNU6-479P	_
ENSG00000252174	-2.796355	-2.316672	1	1	RNU7-18P	_
ENSG00000252213	-2.796355	-2.316672	1	1	SNORA74	_
ENSG00000252241	-2.796355	-2.316672	1	1	U3	_
ENSG00000252361	-2.796355	-2.316672	1	1	RNU6-118P	_
ENSG00000252408	-2.796355	-2.316672	1	1	SNORD38	_
ENSG00000252414	-2.796355	-2.316672	1	1	RNU6-100P	_
ENSG00000252494	-2.796355	-2.316672	1	1	RNU6-126P	_
ENSG00000252515	-2.796355	-2.316672	1	1	RNU6-1171P	_
ENSG00000252585	-2.796355	-2.316672	1	1	Y_RNA	_
ENSG00000252599	-2.796355	-2.316672	1	1	RNU6-566P	_
ENSG00000252602	-2.796355	-2.316672	1	1	U8	_
ENSG00000252614	-2.796355	-2.316672	1	1	RNU6-807P	_
ENSG00000252618	-2.796355	-2.316672	1	1	RNA5SP441	_
ENSG00000252620	-2.796355	-2.316672	1	1	RNU6ATAC24P	_
ENSG00000252782	-2.796355	-2.316672	1	1	RNU6-341P	_
ENSG00000252808	-2.796355	-2.316672	1	1	SCARNA4	_
ENSG00000252821	-2.796355	-2.316672	1	1	RNU6-388P	_
ENSG00000252834	-2.796355	-2.316672	1	1	snoR442	_
ENSG00000252859	-2.796355	-2.316672	1	1	RNU6-375P	_
ENSG00000252865	-2.796355	-2.316672	1	1	RNU6-594P	_
ENSG00000252970	-2.796355	-2.316672	1	1	RNA5SP159	_
ENSG00000252972	-2.796355	-2.316672	1	1	RNA5SP398	_
ENSG00000252992	-2.796355	-2.316672	1	1	SCARNA11	_
ENSG00000253112	-2.796355	-2.316672	1	1	CTD-2373N4.5	_
ENSG00000253175	-2.796355	-2.316672	1	1	RP11-267M23.6	K02910
ENSG00000253176	-2.796355	-2.316672	1	1	RP11-1149M3.2	K14535
ENSG00000253197	-2.796355	-2.316672	1	1	CTD-3239E11.2	_
ENSG00000253229	-2.796355	-2.316672	1	1	HIGD1AP6	_
ENSG00000253284	-2.796355	-2.316672	1	1	RP11-282K24.3	_
ENSG00000253302	-2.796355	-2.316672	1	1	STAU2-AS1	_
ENSG00000253321	-2.796355	-2.316672	1	1	RP11-61G23.2	_
ENSG00000253334	-2.796355	-2.316672	1	1	RP13-923O23.6	_
ENSG00000253384	-2.796355	-2.316672	1	1	CTD-2547L16.3	K04135
ENSG00000253390	-2.796355	-2.316672	1	1	CTC-756D1.2	_
ENSG00000253433	-2.796355	-2.316672	1	1	AC083843.3	_
ENSG00000253457	-2.796355	-2.316672	1	1	SMIM18	_
ENSG00000253485	-2.796355	-2.316672	1	1	PCDHGA5	K16495
ENSG00000253499	-2.796355	-2.316672	1	1	RP11-393K19.1	K18164
ENSG00000253515	-2.796355	-2.316672	1	1	RP11-417F21.2	_
ENSG00000253537	-2.796355	-2.316672	1	1	PCDHGA7	K16495
ENSG00000253567	-2.796355	-2.316672	1	1	RP11-181B11.2	_
ENSG00000253620	-2.796355	-2.316672	1	1	AC144568.4	_
ENSG00000253628	-2.796355	-2.316672	1	1	RP11-536N17.1	_
ENSG00000253736	-2.796355	-2.316672	1	1	RP11-779O18.3	_
ENSG00000253764	-2.796355	-2.316672	1	1	RP11-439C15.4	_
ENSG00000253808	-2.796355	-2.316672	1	1	IGHVII-46-1	_

ENSG00000253832	-2.796355	-2.316672	1	1	CTD-2168K21.1	_
ENSG00000253865	-2.796355	-2.316672	1	1	RP11-394O4.3	_
ENSG00000253899	-2.796355	-2.316672	1	1	RP11-613H2.2	_
ENSG00000253917	-2.796355	-2.316672	1	1	AC226119.5	_
ENSG00000253965	-2.796355	-2.316672	1	1	CTC-329D1.3	_
ENSG00000254028	-2.796355	-2.316672	1	1	AC083843.2	_
ENSG00000254034	-2.796355	-2.316672	1	1	RP11-662B19.2	_
ENSG00000254084	-2.796355	-2.316672	1	1	KB-1930G5.4	_
ENSG00000254088	-2.796355	-2.316672	1	1	SLC2A3P4	K08142
ENSG00000254197	-2.796355	-2.316672	1	1	RP11-10J21.5	_
ENSG00000254254	-2.796355	-2.316672	1	1	RP11-17A4.2	_
ENSG00000254273	-2.796355	-2.316672	1	1	RP11-181D18.4	_
ENSG00000254275	-2.796355	-2.316672	1	1	RP11-89M16.1	_
ENSG00000254300	-2.796355	-2.316672	1	1	LINC01111	_
ENSG00000254328	-2.796355	-2.316672	1	1	CTC-308K20.4	K13504
ENSG00000254340	-2.796355	-2.316672	1	1	RP11-10A14.3	_
ENSG00000254370	-2.796355	-2.316672	1	1	RP11-181B11.1	_
ENSG00000254414	-2.796355	-2.316672	1	1	RP11-182J1.1	_
ENSG00000254432	-2.796355	-2.316672	1	1	RP11-33I11.2	_
ENSG00000254460	-2.796355	-2.316672	1	1	RP11-939C17.4	_
ENSG00000254463	-2.796355	-2.316672	1	1	RP11-484D2.3	K03767
ENSG00000254478	-2.796355	-2.316672	1	1	RP11-158I9.1	K02272
ENSG00000254479	-2.796355	-2.316672	1	1	SLC25A1P1	K15100
ENSG00000254512	-2.796355	-2.316672	1	1	RP11-472I20.2	K17080
ENSG00000254536	-2.796355	-2.316672	1	1	RP11-108K14.8	K00308
ENSG00000254538	-2.796355	-2.316672	1	1	RP11-463D19.1	_
ENSG00000254544	-2.796355	-2.316672	1	1	PCNAP4	K04802
ENSG00000254548	-2.796355	-2.316672	1	1	RP11-429J17.5	_
ENSG00000254584	-2.796355	-2.316672	1	1	RP1-17K7.2	_
ENSG00000254604	-2.796355	-2.316672	1	1	AP000487.6	_
ENSG00000254664	-2.796355	-2.316672	1	1	CTD-2560E9.3	_
ENSG00000254686	-2.796355	-2.316672	1	1	RP1-276E15.1	_
ENSG00000254690	-2.796355	-2.316672	1	1	GS1-393G12.12	_
ENSG00000254691	-2.796355	-2.316672	1	1	RP11-91P24.5	_
ENSG00000254701	-2.796355	-2.316672	1	1	RP11-1415C14.4	K01195
ENSG00000254730	-2.796355	-2.316672	1	1	RP11-801G16.2	_
ENSG00000254777	-2.796355	-2.316672	1	1	AC022182.1	_
ENSG00000254826	-2.796355	-2.316672	1	1	CTD-2530H12.2	_
ENSG00000254833	-2.796355	-2.316672	1	1	RP11-50B3.2	_
ENSG00000254847	-2.796355	-2.316672	1	1	RP11-51B23.3	_
ENSG00000254898	-2.796355	-2.316672	1	1	RP11-513D5.2	_
ENSG00000254941	-2.796355	-2.316672	1	1	RP11-677M14.5	_
ENSG00000254964	-2.796355	-2.316672	1	1	RP11-831H9.3	_
ENSG00000254981	-2.796355	-2.316672	1	1	RP11-350N15.3	_
ENSG00000254984	-2.796355	-2.316672	1	1	FTLP6	K17985
ENSG00000255015	-2.796355	-2.316672	1	1	RP11-716H6.1	_
ENSG00000255038	-2.796355	-2.316672	1	1	RP11-1167A19.2	_
ENSG00000255094	-2.796355	-2.316672	1	1	RP11-406D1.2	_
ENSG00000255141	-2.796355	-2.316672	1	1	HNRNPA1P76	K12741
ENSG00000255160	-2.796355	-2.316672	1	1	RP11-428C19.5	_
ENSG00000255197	-2.796355	-2.316672	1	1	RP11-750H9.5	_
ENSG00000255201	-2.796355	-2.316672	1	1	RP11-350N15.4	_
ENSG00000255203	-2.796355	-2.316672	1	1	OR7E2P	K04257
ENSG00000255240	-2.796355	-2.316672	1	1	RP11-142C4.6	_
ENSG00000255308	-2.796355	-2.316672	1	1	RP11-428C19.4	_
ENSG00000255319	-2.796355	-2.316672	1	1	ENPP7P8	_
ENSG00000255325	-2.796355	-2.316672	1	1	RP11-96B2.1	_
ENSG00000255328	-2.796355	-2.316672	1	1	RP11-326C3.12	_

ENSG00000255375	-2.796355	-2.316672	1	1	RP1-65P5.5	_
ENSG00000255397	-2.796355	-2.316672	1	1	AC022182.2	K11291
ENSG00000255400	-2.796355	-2.316672	1	1	RP13-631K18.5	_
ENSG00000255409	-2.796355	-2.316672	1	1	RSF1-IT1	_
ENSG00000255421	-2.796355	-2.316672	1	1	CTD-2011F17.2	_
ENSG00000255427	-2.796355	-2.316672	1	1	RP11-350D17.2	_
ENSG00000255474	-2.796355	-2.316672	1	1	RP11-234B24.2	_
ENSG00000255557	-2.796355	-2.316672	1	1	RP11-770G2.2	_
ENSG00000255565	-2.796355	-2.316672	1	1	RP11-6B19.1	_
ENSG00000255746	-2.796355	-2.316672	1	1	RP11-283I3.4	_
ENSG00000255808	-2.796355	-2.316672	1	1	RP11-31L22.3	_
ENSG00000255829	-2.796355	-2.316672	1	1	RP11-20D14.3	_
ENSG00000255839	-2.796355	-2.316672	1	1	RP11-338K17.8	_
ENSG00000255847	-2.796355	-2.316672	1	1	RP11-167N4.2	_
ENSG00000255871	-2.796355	-2.316672	1	1	RP11-69C13.1	_
ENSG00000255883	-2.796355	-2.316672	1	1	AC099522.2	_
ENSG00000255910	-2.796355	-2.316672	1	1	RP11-405A12.2	_
ENSG00000255968	-2.796355	-2.316672	1	1	RP11-513G19.1	_
ENSG00000256159	-2.796355	-2.316672	1	1	RP11-820K3.4	K02907
ENSG00000256234	-2.796355	-2.316672	1	1	RP11-283G6.4	_
ENSG00000256238	-2.796355	-2.316672	1	1	RP11-473N11.2	_
ENSG00000256263	-2.796355	-2.316672	1	1	DDX11L8	K11273
ENSG00000256278	-2.796355	-2.316672	1	1	RP11-182J1.5	_
ENSG00000256288	-2.796355	-2.316672	1	1	RP11-277P12.10	_
ENSG00000256325	-2.796355	-2.316672	1	1	RP11-611O2.1	_
ENSG00000256351	-2.796355	-2.316672	1	1	RP1-7G5.5	_
ENSG00000256361	-2.796355	-2.316672	1	1	RP11-613F22.6	_
ENSG00000256381	-2.796355	-2.316672	1	1	RP11-500M8.4	_
ENSG00000256407	-2.796355	-2.316672	1	1	RP11-446E24.4	_
ENSG00000256448	-2.796355	-2.316672	1	1	RP11-809N8.4	_
ENSG00000256499	-2.796355	-2.316672	1	1	CTC-465D4.1	_
ENSG00000256564	-2.796355	-2.316672	1	1	RP11-424M22.3	_
ENSG00000256588	-2.796355	-2.316672	1	1	RP11-613F22.8	_
ENSG00000256612	-2.796355	-2.316672	1	1	CYP2B7P	K17709
ENSG00000256650	-2.796355	-2.316672	1	1	RERG-IT1	_
ENSG00000256671	-2.796355	-2.316672	1	1	LIMS3L	_
ENSG00000256739	-2.796355	-2.316672	1	1	RP11-45F15.1	_
ENSG00000256746	-2.796355	-2.316672	1	1	RP11-17G12.3	_
ENSG00000256771	-2.796355	-2.316672	1	1	ZNF253	K09228
ENSG00000256898	-2.796355	-2.316672	1	1	RP11-707G14.8	K05756
ENSG00000256916	-2.796355	-2.316672	1	1	RP11-817J15.2	_
ENSG00000256944	-2.796355	-2.316672	1	1	RP11-881M11.1	_
ENSG00000256966	-2.796355	-2.316672	1	1	RP11-613M10.8	K10296
ENSG00000257069	-2.796355	-2.316672	1	1	RP11-783K16.10	_
ENSG00000257207	-2.796355	-2.316672	1	1	LIMS3	_
ENSG00000257246	-2.796355	-2.316672	1	1	RP11-416A17.6	K17080
ENSG00000257256	-2.796355	-2.316672	1	1	RP3-405J10.4	_
ENSG00000257283	-2.796355	-2.316672	1	1	RP11-1060G2.1	_
ENSG00000257336	-2.796355	-2.316672	1	1	PRELID2P1	_
ENSG00000257403	-2.796355	-2.316672	1	1	CTD-2547E10.3	_
ENSG00000257438	-2.796355	-2.316672	1	1	RP11-114F10.3	_
ENSG00000257488	-2.796355	-2.316672	1	1	RP5-1057I20.2	_
ENSG00000257507	-2.796355	-2.316672	1	1	RP11-956E11.1	_
ENSG00000257512	-2.796355	-2.316672	1	1	RP11-486A14.1	K03357
ENSG00000257594	-2.796355	-2.316672	1	1	GALNT4	K00710
ENSG00000257663	-2.796355	-2.316672	1	1	RP11-1100L3.7	_
ENSG00000257674	-2.796355	-2.316672	1	1	RP11-76E16.2	K17392
ENSG00000257726	-2.796355	-2.316672	1	1	RP11-116D17.1	_

ENSG00000257859	-2.796355	-2.316672	1	1	CASC18	_
ENSG00000257886	-2.796355	-2.316672	1	1	RP11-61E11.1	_
ENSG00000257890	-2.796355	-2.316672	1	1	RP11-114F10.2	_
ENSG00000257910	-2.796355	-2.316672	1	1	RP11-461F16.3	_
ENSG00000257918	-2.796355	-2.316672	1	1	RP11-482D24.3	_
ENSG00000257924	-2.796355	-2.316672	1	1	RP11-493L12.5	_
ENSG00000257940	-2.796355	-2.316672	1	1	RP11-248E9.6	_
ENSG00000257956	-2.796355	-2.316672	1	1	RP11-359M6.3	K14564
ENSG00000257966	-2.796355	-2.316672	1	1	OLA1P3	K06942
ENSG00000258034	-2.796355	-2.316672	1	1	RP11-493P1.2	_
ENSG00000258088	-2.796355	-2.316672	1	1	RP11-114H23.2	_
ENSG00000258137	-2.796355	-2.316672	1	1	RP11-753H16.3	_
ENSG00000258171	-2.796355	-2.316672	1	1	RP11-511B23.3	_
ENSG00000258204	-2.796355	-2.316672	1	1	RP11-90E9.1	K02896
ENSG00000258260	-2.796355	-2.316672	1	1	RP11-977G19.14	_
ENSG00000258284	-2.796355	-2.316672	1	1	POLR2KP1	K03009
ENSG00000258317	-2.796355	-2.316672	1	1	RP11-603J24.5	_
ENSG00000258352	-2.796355	-2.316672	1	1	RP11-23J18.1	K06566
ENSG00000258355	-2.796355	-2.316672	1	1	RP11-651L5.2	_
ENSG00000258404	-2.796355	-2.316672	1	1	RP11-1029J19.5	_
ENSG00000258439	-2.796355	-2.316672	1	1	RP11-173A8.2	_
ENSG00000258452	-2.796355	-2.316672	1	1	RP11-757H14.2	K11300
ENSG00000258462	-2.796355	-2.316672	1	1	RP11-831F12.4	_
ENSG00000258488	-2.796355	-2.316672	1	1	RP11-467L19.8	K10595
ENSG00000258591	-2.796355	-2.316672	1	1	RP11-545M17.3	K03767
ENSG00000258599	-2.796355	-2.316672	1	1	RP11-84C10.1	_
ENSG00000258601	-2.796355	-2.316672	1	1	RP11-81F13.2	_
ENSG00000258675	-2.796355	-2.316672	1	1	RP11-299L17.3	_
ENSG00000258745	-2.796355	-2.316672	1	1	RP11-218E20.5	_
ENSG00000258777	-2.796355	-2.316672	1	1	HIF1A-AS1	_
ENSG00000258802	-2.796355	-2.316672	1	1	RP11-589M4.2	_
ENSG00000258809	-2.796355	-2.316672	1	1	CTD-2555O16.1	_
ENSG00000258851	-2.796355	-2.316672	1	1	RP11-894P9.2	_
ENSG00000258896	-2.796355	-2.316672	1	1	SCOCP1	_
ENSG00000258982	-2.796355	-2.316672	1	1	RP11-638I2.4	_
ENSG00000259005	-2.796355	-2.316672	1	1	RP3-449M8.6	_
ENSG00000259040	-2.796355	-2.316672	1	1	BLOC1S5-TXNDC5	K13984
ENSG00000259048	-2.796355	-2.316672	1	1	CTD-2058B24.2	_
ENSG00000259051	-2.796355	-2.316672	1	1	HNRNPUP1	K12888
ENSG00000259070	-2.796355	-2.316672	1	1	LINC00639	_
ENSG00000259096	-2.796355	-2.316672	1	1	FAM35CP	_
ENSG00000259113	-2.796355	-2.316672	1	1	RP11-406H23.2	_
ENSG00000259222	-2.796355	-2.316672	1	1	RP11-809H16.5	_
ENSG00000259224	-2.796355	-2.316672	1	1	SLC35G6	_
ENSG00000259244	-2.796355	-2.316672	1	1	RP11-182J1.12	_
ENSG00000259274	-2.796355	-2.316672	1	1	CTD-2501E16.2	_
ENSG00000259286	-2.796355	-2.316672	1	1	RP11-696L21.2	_
ENSG00000259399	-2.796355	-2.316672	1	1	TGIF2-C20orf24	K19553
ENSG00000259416	-2.796355	-2.316672	1	1	RP11-158M2.5	_
ENSG00000259442	-2.796355	-2.316672	1	1	RP11-752G15.8	_
ENSG00000259462	-2.796355	-2.316672	1	1	RP11-752G15.3	_
ENSG00000259501	-2.796355	-2.316672	1	1	RP11-467N20.1	_
ENSG00000259604	-2.796355	-2.316672	1	1	RP11-66B24.1	_
ENSG00000259617	-2.796355	-2.316672	1	1	RP11-540O11.6	_
ENSG00000259649	-2.796355	-2.316672	1	1	RP11-351M8.1	_
ENSG00000259664	-2.796355	-2.316672	1	1	CTD-2147F2.2	_
ENSG00000259757	-2.796355	-2.316672	1	1	RP11-129I19.2	_
ENSG00000259790	-2.796355	-2.316672	1	1	ANP32BP1	K18647

ENSG00000259871	-2.796355	-2.316672	1	1	CTA-363E6.1	_
ENSG00000259883	-2.796355	-2.316672	1	1	CTD-2382E5.4	_
ENSG00000259922	-2.796355	-2.316672	1	1	RP11-322D14.1	_
ENSG00000259932	-2.796355	-2.316672	1	1	CTD-2651B20.7	_
ENSG00000259935	-2.796355	-2.316672	1	1	RP11-519C12.1	_
ENSG00000259942	-2.796355	-2.316672	1	1	RP11-292F22.7	_
ENSG00000260011	-2.796355	-2.316672	1	1	RP13-20L14.1	_
ENSG00000260012	-2.796355	-2.316672	1	1	RP11-329J18.4	K02737
ENSG00000260017	-2.796355	-2.316672	1	1	RP11-1035H13.2	_
ENSG00000260034	-2.796355	-2.316672	1	1	RP11-266L9.2	_
ENSG00000260036	-2.796355	-2.316672	1	1	RP11-178D12.2	K16339
ENSG00000260091	-2.796355	-2.316672	1	1	RP11-33B1.4	_
ENSG00000260105	-2.796355	-2.316672	1	1	AOC4P	K00276
ENSG00000260115	-2.796355	-2.316672	1	1	CTC-420A11.2	_
ENSG00000260133	-2.796355	-2.316672	1	1	CA5AP1	K01672
ENSG00000260156	-2.796355	-2.316672	1	1	RP11-394B2.6	_
ENSG00000260253	-2.796355	-2.316672	1	1	RP4-676L2.1	_
ENSG00000260286	-2.796355	-2.316672	1	1	C6orf229	_
ENSG00000260293	-2.796355	-2.316672	1	1	RP11-715J22.6	_
ENSG00000260318	-2.796355	-2.316672	1	1	COX6CP1	K02268
ENSG00000260417	-2.796355	-2.316672	1	1	CTD-2542L18.1	_
ENSG00000260441	-2.796355	-2.316672	1	1	RP11-96D1.7	_
ENSG00000260496	-2.796355	-2.316672	1	1	RP11-161M6.3	_
ENSG00000260515	-2.796355	-2.316672	1	1	CTD-2199O4.3	_
ENSG00000260545	-2.796355	-2.316672	1	1	CTB-134F13.1	_
ENSG00000260581	-2.796355	-2.316672	1	1	CTB-113P19.4	_
ENSG00000260608	-2.796355	-2.316672	1	1	RP11-382A20.7	_
ENSG00000260613	-2.796355	-2.316672	1	1	RP3-522J7.6	_
ENSG00000260644	-2.796355	-2.316672	1	1	HERC2P5	K10595
ENSG00000260647	-2.796355	-2.316672	1	1	RP1-178F10.1	_
ENSG00000260651	-2.796355	-2.316672	1	1	AF213884.2	_
ENSG00000260660	-2.796355	-2.316672	1	1	RP11-69H7.2	_
ENSG00000260698	-2.796355	-2.316672	1	1	RP11-439E19.9	_
ENSG00000260809	-2.796355	-2.316672	1	1	CTD-2144E22.10	K18468
ENSG00000260818	-2.796355	-2.316672	1	1	UNGP1	K03648
ENSG00000260851	-2.796355	-2.316672	1	1	RP11-403P17.5	K00857
ENSG00000260860	-2.796355	-2.316672	1	1	RP11-615I2.3	_
ENSG00000260880	-2.796355	-2.316672	1	1	HCCAT5	_
ENSG00000260914	-2.796355	-2.316672	1	1	RP11-343C2.11	K12196
ENSG00000260997	-2.796355	-2.316672	1	1	RP4-647J21.1	_
ENSG00000261026	-2.796355	-2.316672	1	1	CTD-3247F14.2	_
ENSG00000261037	-2.796355	-2.316672	1	1	CTC-471C19.1	_
ENSG00000261056	-2.796355	-2.316672	1	1	RP11-454F8.2	K09228
ENSG00000261067	-2.796355	-2.316672	1	1	RP11-264B17.3	_
ENSG00000261202	-2.796355	-2.316672	1	1	RP3-496C20.1	_
ENSG00000261243	-2.796355	-2.316672	1	1	RP11-517C16.4	_
ENSG00000261269	-2.796355	-2.316672	1	1	RP11-389C8.2	_
ENSG00000261291	-2.796355	-2.316672	1	1	RP11-295M3.2	_
ENSG00000261359	-2.796355	-2.316672	1	1	C16orf98	K12799
ENSG00000261392	-2.796355	-2.316672	1	1	RP11-473I1.6	_
ENSG00000261399	-2.796355	-2.316672	1	1	LA16c-329F2.1	_
ENSG00000261431	-2.796355	-2.316672	1	1	RP4-616B8.4	_
ENSG00000261435	-2.796355	-2.316672	1	1	MIR1587	_
ENSG00000261519	-2.796355	-2.316672	1	1	Y_RNA	_
ENSG00000261568	-2.796355	-2.316672	1	1	RP11-524C21.2	_
ENSG00000261592	-2.796355	-2.316672	1	1	RP11-178L8.3	_
ENSG00000261599	-2.796355	-2.316672	1	1	HERC2P8	K10595
ENSG00000261610	-2.796355	-2.316672	1	1	AP000265.1	_

ENSG00000261616	-2.796355	-2.316672	1	1	RP11-6O2.3	_
ENSG00000261669	-2.796355	-2.316672	1	1	CTD-2515A14.1	_
ENSG00000261673	-2.796355	-2.316672	1	1	RP11-328J14.1	_
ENSG00000261729	-2.796355	-2.316672	1	1	GS1-204I12.4	_
ENSG00000261789	-2.796355	-2.316672	1	1	RP11-709D24.5	_
ENSG00000261796	-2.796355	-2.316672	1	1	ISY1-RAB43	K12870
ENSG00000261839	-2.796355	-2.316672	1	1	RP1-265C24.8	_
ENSG00000261872	-2.796355	-2.316672	1	1	RP5-867C24.5	_
ENSG00000261916	-2.796355	-2.316672	1	1	RP11-235E17.4	_
ENSG00000262026	-2.796355	-2.316672	1	1	RP11-2C24.6	_
ENSG00000262052	-2.796355	-2.316672	1	1	RP11-763E3.1	_
ENSG00000262115	-2.796355	-2.316672	1	1	RP11-455O6.2	_
ENSG00000262136	-2.796355	-2.316672	1	1	CTD-2033A16.3	_
ENSG00000262227	-2.796355	-2.316672	1	1	RP5-1050D4.5	_
ENSG00000262248	-2.796355	-2.316672	1	1	RP11-235E17.5	K01724
ENSG00000262380	-2.796355	-2.316672	1	1	CTB-193M12.3	_
ENSG00000262412	-2.796355	-2.316672	1	1	RP11-85G18.6	_
ENSG00000262429	-2.796355	-2.316672	1	1	RP5-1050D4.3	_
ENSG00000262471	-2.796355	-2.316672	1	1	RP11-95P2.4	K08762
ENSG00000262488	-2.796355	-2.316672	1	1	RP11-876N24.1	_
ENSG00000262526	-2.796355	-2.316672	1	1	CTD-2545G14.7	K08341
ENSG00000262623	-2.796355	-2.316672	1	1	RP5-1107A17.2	K02957
ENSG00000262668	-2.796355	-2.316672	1	1	AJ003147.9	_
ENSG00000262730	-2.796355	-2.316672	1	1	RP11-1099M24.7	K04884
ENSG00000262810	-2.796355	-2.316672	1	1	RP11-667K14.5	_
ENSG00000262999	-2.796355	-2.316672	1	1	CTD-3088G3.6	_
ENSG00000263045	-2.796355	-2.316672	1	1	RP11-28B23.1	_
ENSG00000263201	-2.796355	-2.316672	1	1	CTC-479C5.6	_
ENSG00000263311	-2.796355	-2.316672	1	1	RP11-498D10.8	_
ENSG00000263393	-2.796355	-2.316672	1	1	RP11-53I6.4	_
ENSG00000263406	-2.796355	-2.316672	1	1	RN7SL821P	_
ENSG00000263407	-2.796355	-2.316672	1	1	MIR4660	_
ENSG00000263477	-2.796355	-2.316672	1	1	RP11-338L22.2	_
ENSG00000263499	-2.796355	-2.316672	1	1	RP11-118E18.4	_
ENSG00000263539	-2.796355	-2.316672	1	1	AC024475.1	_
ENSG00000263657	-2.796355	-2.316672	1	1	RP11-82O19.1	_
ENSG00000263680	-2.796355	-2.316672	1	1	RP11-57A1.1	_
ENSG00000263742	-2.796355	-2.316672	1	1	MIR3165	_
ENSG00000263813	-2.796355	-2.316672	1	1	MIR3679	_
ENSG00000264012	-2.796355	-2.316672	1	1	RP11-627G18.2	_
ENSG00000264171	-2.796355	-2.316672	1	1	MIR4305	_
ENSG00000264190	-2.796355	-2.316672	1	1	MIR5006	_
ENSG00000264273	-2.796355	-2.316672	1	1	CTD-2145A24.4	_
ENSG00000264279	-2.796355	-2.316672	1	1	MIR4786	_
ENSG00000264384	-2.796355	-2.316672	1	1	RN7SL431P	_
ENSG00000264435	-2.796355	-2.316672	1	1	RP11-338L22.3	K00940
ENSG00000264496	-2.796355	-2.316672	1	1	AC108218.1	_
ENSG00000264572	-2.796355	-2.316672	1	1	MIR4296	_
ENSG00000264584	-2.796355	-2.316672	1	1	RN7SL600P	_
ENSG00000264666	-2.796355	-2.316672	1	1	RP11-524F11.2	_
ENSG00000264671	-2.796355	-2.316672	1	1	AP006477.1	_
ENSG00000264756	-2.796355	-2.316672	1	1	RN7SL805P	_
ENSG00000264771	-2.796355	-2.316672	1	1	AC022819.1	_
ENSG00000264792	-2.796355	-2.316672	1	1	MIR4637	_
ENSG00000264857	-2.796355	-2.316672	1	1	MIR5696	_
ENSG00000264860	-2.796355	-2.316672	1	1	RP11-143K11.5	_
ENSG00000264864	-2.796355	-2.316672	1	1	MIR3613	_
ENSG00000264878	-2.796355	-2.316672	1	1	Z85986.1	_

ENSG00000264961	-2.796355	-2.316672	1	1	MIR4730	_
ENSG00000264964	-2.796355	-2.316672	1	1	RP11-888D10.3	_
ENSG00000265008	-2.796355	-2.316672	1	1	RP11-53I6.3	_
ENSG00000265069	-2.796355	-2.316672	1	1	RP11-781P6.1	_
ENSG00000265083	-2.796355	-2.316672	1	1	MIR3691	_
ENSG00000265123	-2.796355	-2.316672	1	1	RN7SL200P	_
ENSG00000265126	-2.796355	-2.316672	1	1	CTB-187M2.2	_
ENSG00000265128	-2.796355	-2.316672	1	1	RP11-15F12.3	_
ENSG00000265139	-2.796355	-2.316672	1	1	RP11-227G15.2	_
ENSG00000265195	-2.796355	-2.316672	1	1	MIR4312	_
ENSG00000265264	-2.796355	-2.316672	1	1	TIMM10B	K17779
ENSG00000265282	-2.796355	-2.316672	1	1	RP11-269G24.4	_
ENSG00000265287	-2.796355	-2.316672	1	1	RP11-192H23.6	_
ENSG00000265317	-2.796355	-2.316672	1	1	AC103740.1	_
ENSG00000265345	-2.796355	-2.316672	1	1	MIR5188	_
ENSG00000265386	-2.796355	-2.316672	1	1	RN7SL219P	_
ENSG00000265394	-2.796355	-2.316672	1	1	RP11-1148O4.2	_
ENSG00000265425	-2.796355	-2.316672	1	1	RP11-128D14.1	_
ENSG00000265463	-2.796355	-2.316672	1	1	AC012391.1	_
ENSG00000265480	-2.796355	-2.316672	1	1	KRT18P55	K07604
ENSG00000265485	-2.796355	-2.316672	1	1	RP11-449D8.1	_
ENSG00000265507	-2.796355	-2.316672	1	1	MIR4435-1	_
ENSG00000265527	-2.796355	-2.316672	1	1	MIR5690	_
ENSG00000265611	-2.796355	-2.316672	1	1	MIR5010	_
ENSG00000265638	-2.796355	-2.316672	1	1	AC005702.3	_
ENSG00000265647	-2.796355	-2.316672	1	1	AL358815.1	_
ENSG00000265809	-2.796355	-2.316672	1	1	AC007114.1	_
ENSG00000265843	-2.796355	-2.316672	1	1	LINC01029	_
ENSG00000265932	-2.796355	-2.316672	1	1	MIR3146	_
ENSG00000265987	-2.796355	-2.316672	1	1	RP11-16C1.3	_
ENSG00000266047	-2.796355	-2.316672	1	1	AL139377.1	_
ENSG00000266063	-2.796355	-2.316672	1	1	MIR4771-2	_
ENSG00000266075	-2.796355	-2.316672	1	1	RN7SL574P	_
ENSG00000266111	-2.796355	-2.316672	1	1	RP11-296K13.4	_
ENSG00000266296	-2.796355	-2.316672	1	1	ARIH2P1	K11969
ENSG00000266311	-2.796355	-2.316672	1	1	AC007952.4	_
ENSG00000266340	-2.796355	-2.316672	1	1	RP11-848P1.4	_
ENSG00000266385	-2.796355	-2.316672	1	1	RP11-227G15.8	_
ENSG00000266397	-2.796355	-2.316672	1	1	RP11-737O24.1	_
ENSG00000266423	-2.796355	-2.316672	1	1	MIR3163	_
ENSG00000266428	-2.796355	-2.316672	1	1	RN7SL663P	_
ENSG00000266521	-2.796355	-2.316672	1	1	RP11-650P15.1	_
ENSG00000266541	-2.796355	-2.316672	1	1	RP11-881L2.1	_
ENSG00000266594	-2.796355	-2.316672	1	1	MIR4766	_
ENSG00000266649	-2.796355	-2.316672	1	1	MIR3126	_
ENSG00000266677	-2.796355	-2.316672	1	1	RP11-258F1.1	_
ENSG00000266721	-2.796355	-2.316672	1	1	MIR5695	_
ENSG00000266811	-2.796355	-2.316672	1	1	MIR3118-3	_
ENSG00000266844	-2.796355	-2.316672	1	1	RP11-862L9.3	_
ENSG00000266872	-2.796355	-2.316672	1	1	RP11-19P22.8	_
ENSG00000266918	-2.796355	-2.316672	1	1	RP11-798G7.8	_
ENSG00000266922	-2.796355	-2.316672	1	1	CTC-499B15.6	_
ENSG00000266934	-2.796355	-2.316672	1	1	RP11-332H18.3	_
ENSG00000266936	-2.796355	-2.316672	1	1	CTC-215O4.4	_
ENSG00000266944	-2.796355	-2.316672	1	1	AC005262.4	K02918
ENSG00000266987	-2.796355	-2.316672	1	1	RP11-354P11.8	_
ENSG00000266994	-2.796355	-2.316672	1	1	RP11-127I20.7	_
ENSG00000267032	-2.796355	-2.316672	1	1	RP11-807E13.2	_

ENSG00000267036	-2.796355	-2.316672	1	1	AC005559.3	K04955
ENSG00000267064	-2.796355	-2.316672	1	1	UXT-AS1	_
ENSG00000267088	-2.796355	-2.316672	1	1	RP11-115N12.1	_
ENSG00000267108	-2.796355	-2.316672	1	1	RP11-861E21.1	_
ENSG00000267110	-2.796355	-2.316672	1	1	CTD-2587H24.4	K12044
ENSG00000267112	-2.796355	-2.316672	1	1	RP11-839G9.1	_
ENSG00000267114	-2.796355	-2.316672	1	1	CTB-129P6.11	K12900
ENSG00000267131	-2.796355	-2.316672	1	1	RP11-332H18.5	_
ENSG00000267157	-2.796355	-2.316672	1	1	CTB-54O9.9	K05544
ENSG00000267198	-2.796355	-2.316672	1	1	RP11-798G7.6	_
ENSG00000267211	-2.796355	-2.316672	1	1	RP5-1067M6.3	_
ENSG00000267230	-2.796355	-2.316672	1	1	RP11-376M2.2	_
ENSG00000267257	-2.796355	-2.316672	1	1	RP11-1151B14.4	_
ENSG00000267275	-2.796355	-2.316672	1	1	CTD-2562J15.6	_
ENSG00000267282	-2.796355	-2.316672	1	1	CTB-129P6.4	_
ENSG00000267289	-2.796355	-2.316672	1	1	CTD-2623N2.11	_
ENSG00000267299	-2.796355	-2.316672	1	1	CTB-66B24.1	_
ENSG00000267518	-2.796355	-2.316672	1	1	RP11-794C22.2	K11300
ENSG00000267533	-2.796355	-2.316672	1	1	RP11-815J4.7	_
ENSG00000267541	-2.796355	-2.316672	1	1	RP11-380M21.2	K02261
ENSG00000267557	-2.796355	-2.316672	1	1	CTC-379B2.4	_
ENSG00000267562	-2.796355	-2.316672	1	1	CTC-421K24.1	K02896
ENSG00000267573	-2.796355	-2.316672	1	1	KRT8P5	K07605
ENSG00000267614	-2.796355	-2.316672	1	1	AC138472.6	_
ENSG00000267647	-2.796355	-2.316672	1	1	MAP1LC3P	_
ENSG00000267657	-2.796355	-2.316672	1	1	CTB-186H2.2	_
ENSG00000267692	-2.796355	-2.316672	1	1	TAF9P3	K18532
ENSG00000267701	-2.796355	-2.316672	1	1	RP11-28F1.2	_
ENSG00000267729	-2.796355	-2.316672	1	1	AC010761.14	_
ENSG00000267741	-2.796355	-2.316672	1	1	UBE2L4	K04552
ENSG00000267743	-2.796355	-2.316672	1	1	RP11-718I15.1	_
ENSG00000267744	-2.796355	-2.316672	1	1	RP11-799D4.1	K09440
ENSG00000267765	-2.796355	-2.316672	1	1	CTD-3193K9.3	_
ENSG00000267800	-2.796355	-2.316672	1	1	RP11-49K24.5	K12160
ENSG00000267885	-2.796355	-2.316672	1	1	FKSG48	_
ENSG00000267976	-2.796355	-2.316672	1	1	AP000695.1	K03029
ENSG00000268015	-2.796355	-2.316672	1	1	CTD-2525I3.3	_
ENSG00000268034	-2.796355	-2.316672	1	1	AC005795.1	K09290
ENSG00000268058	-2.796355	-2.316672	1	1	CTD-2017D11.3	_
ENSG00000268076	-2.796355	-2.316672	1	1	AC087239.1	_
ENSG00000268098	-2.796355	-2.316672	1	1	AP000688.1	_
ENSG00000268155	-2.796355	-2.316672	1	1	AL161450.1	_
ENSG00000268162	-2.796355	-2.316672	1	1	HBCBP	_
ENSG00000268275	-2.796355	-2.316672	1	1	AL035588.1	_
ENSG00000268307	-2.796355	-2.316672	1	1	CTD-2619J13.13	_
ENSG00000268328	-2.796355	-2.316672	1	1	AC019206.1	_
ENSG00000268367	-2.796355	-2.316672	1	1	AC126614.1	K02927
ENSG00000268396	-2.796355	-2.316672	1	1	AC012485.1	_
ENSG00000268423	-2.796355	-2.316672	1	1	AC011551.3	K06115
ENSG00000268457	-2.796355	-2.316672	1	1	AL160175.1	_
ENSG00000268615	-2.796355	-2.316672	1	1	RP11-65J3.15	_
ENSG00000268649	-2.796355	-2.316672	1	1	MIR296	_
ENSG00000268656	-2.796355	-2.316672	1	1	AC110084.1	_
ENSG00000268686	-2.796355	-2.316672	1	1	AC010524.2	_
ENSG00000268758	-2.796355	-2.316672	1	1	EMR4P	K08445
ENSG00000268764	-2.796355	-2.316672	1	1	CTC-450M9.1	_
ENSG00000268836	-2.796355	-2.316672	1	1	LA16c-OS12.2	_
ENSG00000268913	-2.796355	-2.316672	1	1	AC026806.2	_

ENSG00000268938	-2.796355	-2.316672	1	1	AC005387.3	_
ENSG00000268996	-2.796355	-2.316672	1	1	AL807752.1	_
ENSG00000269148	-2.796355	-2.316672	1	1	AC092301.3	_
ENSG00000269172	-2.796355	-2.316672	1	1	CTC-218B8.3	_
ENSG00000269227	-2.796355	-2.316672	1	1	RP11-345P4.6	K02993
ENSG00000269279	-2.796355	-2.316672	1	1	AL136376.1	K01539
ENSG00000269304	-2.796355	-2.316672	1	1	AC010522.1	K09568
ENSG00000269363	-2.796355	-2.316672	1	1	MGC4771	_
ENSG00000269388	-2.796355	-2.316672	1	1	AC018755.16	K14168
ENSG00000269397	-2.796355	-2.316672	1	1	CTB-92J24.2	_
ENSG00000269402	-2.796355	-2.316672	1	1	AC116407.2	K12173
ENSG00000269407	-2.796355	-2.316672	1	1	AL096677.1	_
ENSG00000269466	-2.796355	-2.316672	1	1	RP11-432M8.17	K11253
ENSG00000269526	-2.796355	-2.316672	1	1	ERVV-1	_
ENSG00000269570	-2.796355	-2.316672	1	1	AP001350.1	_
ENSG00000269653	-2.796355	-2.316672	1	1	CTB-102L5.7	_
ENSG00000269736	-2.796355	-2.316672	1	1	CTD-2521M24.11	_
ENSG00000269746	-2.796355	-2.316672	1	1	AC009060.1	_
ENSG00000269832	-2.796355	-2.316672	1	1	AL627171.2	_
ENSG00000269913	-2.796355	-2.316672	1	1	CTC-1337H24.1	_
ENSG00000269925	-2.796355	-2.316672	1	1	RP3-467L1.6	_
ENSG00000269934	-2.796355	-2.316672	1	1	RP5-1139B12.3	_
ENSG00000269935	-2.796355	-2.316672	1	1	RP11-482M8.3	_
ENSG00000269976	-2.796355	-2.316672	1	1	RP11-130L8.2	_
ENSG00000269996	-2.796355	-2.316672	1	1	RP11-343N15.5	_
ENSG00000270002	-2.796355	-2.316672	1	1	RP11-93H12.4	_
ENSG00000270011	-2.796355	-2.316672	1	1	ZNF559-ZNF177	K09228
ENSG00000270036	-2.796355	-2.316672	1	1	RP11-685G9.4	_
ENSG00000270095	-2.796355	-2.316672	1	1	RP11-214K3.18	_
ENSG00000270103	-2.796355	-2.316672	1	1	RNU11	_
ENSG00000270137	-2.796355	-2.316672	1	1	CTC-137K3.1	_
ENSG00000270248	-2.796355	-2.316672	1	1	CTC-471J1.10	_
ENSG00000270255	-2.796355	-2.316672	1	1	RP11-420B22.2	_
ENSG00000270299	-2.796355	-2.316672	1	1	RP5-850E9.3	K12260
ENSG00000270324	-2.796355	-2.316672	1	1	RP11-180P8.4	_
ENSG00000270356	-2.796355	-2.316672	1	1	IGHV1OR15-4	K06856
ENSG00000270399	-2.796355	-2.316672	1	1	RP11-1407O15.3	K13241
ENSG00000270490	-2.796355	-2.316672	1	1	RP11-622C24.1	K17800
ENSG00000270577	-2.796355	-2.316672	1	1	RP5-1147A1.1	_
ENSG00000270591	-2.796355	-2.316672	1	1	RP11-73M18.11	K11584
ENSG00000270777	-2.796355	-2.316672	1	1	RP11-103J8.2	_
ENSG00000270849	-2.796355	-2.316672	1	1	CHCHD2P3	_
ENSG00000270876	-2.796355	-2.316672	1	1	CTC-523E23.8	_
ENSG00000270972	-2.796355	-2.316672	1	1	RP11-326C3.15	_
ENSG00000271002	-2.796355	-2.316672	1	1	RP11-599B13.8	K02868
ENSG00000271141	-2.796355	-2.316672	1	1	RP11-171I2.4	_
ENSG00000271214	-2.796355	-2.316672	1	1	RP11-9M16.3	_
ENSG00000271225	-2.796355	-2.316672	1	1	RP11-460N11.3	K15464
ENSG00000271227	-2.796355	-2.316672	1	1	RP1-152L7.8	_
ENSG00000271337	-2.796355	-2.316672	1	1	NDE1P2	K04257
ENSG00000271414	-2.796355	-2.316672	1	1	RP11-751K21.1	_
ENSG00000271415	-2.796355	-2.316672	1	1	RP11-305E17.7	_
ENSG00000271490	-2.796355	-2.316672	1	1	RP11-60E8.4	_
ENSG00000271532	-2.796355	-2.316672	1	1	RP11-426K3.1	K19399
ENSG00000271537	-2.796355	-2.316672	1	1	RP11-365F18.6	K14566
ENSG00000271600	-2.796355	-2.316672	1	1	RP11-864G5.8	_
ENSG00000271626	-2.796355	-2.316672	1	1	RP11-3K24.2	K11253
ENSG00000271639	-2.796355	-2.316672	1	1	RP11-289A15.1	_

ENSG00000271670	-2.796355	-2.316672	1	1	RP11-95I16.4	_
ENSG00000271710	-2.796355	-2.316672	1	1	RP11-312P12.3	_
ENSG00000271757	-2.796355	-2.316672	1	1	RP11-111M22.5	_
ENSG00000271795	-2.796355	-2.316672	1	1	CTC-251D13.1	_
ENSG00000271798	-2.796355	-2.316672	1	1	SNORA51	_
ENSG00000271819	-2.796355	-2.316672	1	1	U6	_
ENSG00000271889	-2.796355	-2.316672	1	1	RP11-493E12.1	_
ENSG00000271930	-2.796355	-2.316672	1	1	RP11-44N12.5	_
ENSG00000271937	-2.796355	-2.316672	1	1	RP11-424N24.2	_
ENSG00000271977	-2.796355	-2.316672	1	1	AC226119.4	_
ENSG00000271991	-2.796355	-2.316672	1	1	RP11-79O8.1	_
ENSG00000272004	-2.796355	-2.316672	1	1	RP11-345P4.10	_
ENSG00000272055	-2.796355	-2.316672	1	1	RNU6-6P	_
ENSG00000272123	-2.796355	-2.316672	1	1	CTD-2366F13.2	_
ENSG00000272170	-2.796355	-2.316672	1	1	RP11-387M24.5	_
ENSG00000272267	-2.796355	-2.316672	1	1	RP11-375N15.2	_
ENSG00000272274	-2.796355	-2.316672	1	1	LINC00551	_
ENSG00000272281	-2.796355	-2.316672	1	1	TPTE2P2	K18079
ENSG00000272308	-2.796355	-2.316672	1	1	RP11-231G3.1	_
ENSG00000272312	-2.796355	-2.316672	1	1	RP11-239L20.6	_
ENSG00000272333	-2.796355	-2.316672	1	1	KMT2B	K14959
ENSG00000272354	-2.796355	-2.316672	1	1	RP11-307L14.2	_
ENSG00000272359	-2.796355	-2.316672	1	1	U4	_
ENSG00000272361	-2.796355	-2.316672	1	1	GS1-166A23.2	_
ENSG00000272403	-2.796355	-2.316672	1	1	RP1-93H18.7	_
ENSG00000272439	-2.796355	-2.316672	1	1	U6	_
ENSG00000272472	-2.796355	-2.316672	1	1	RP11-95G17.2	_
ENSG00000272506	-2.796355	-2.316672	1	1	RP4-535B20.4	_
ENSG00000272508	-2.796355	-2.316672	1	1	RP11-96C23.14	_
ENSG00000272516	-2.796355	-2.316672	1	1	RP11-573G6.9	_
ENSG00000272533	-2.796355	-2.316672	1	1	SNORA28	_
ENSG00000272736	-2.796355	-2.316672	1	1	RP11-799N11.1	_
ENSG00000272745	-2.796355	-2.316672	1	1	RP5-1007H16.1	_
ENSG00000272772	-2.796355	-2.316672	1	1	CTD-2410N18.5	K03094
ENSG00000272827	-2.796355	-2.316672	1	1	RP11-445P19.3	_
ENSG00000272832	-2.796355	-2.316672	1	1	RP11-91K8.5	_
ENSG00000272856	-2.796355	-2.316672	1	1	RP11-710E1.2	_
ENSG00000272859	-2.796355	-2.316672	1	1	RP11-352E8.2	_
ENSG00000272861	-2.796355	-2.316672	1	1	RP11-332H14.1	_
ENSG00000272895	-2.796355	-2.316672	1	1	RP11-98C1.2	_
ENSG00000272921	-2.796355	-2.316672	1	1	RP11-500M8.7	K03953
ENSG00000272922	-2.796355	-2.316672	1	1	RP11-329B9.5	_
ENSG00000272968	-2.796355	-2.316672	1	1	RBAK-RBAKDN	K09228
ENSG00000272979	-2.796355	-2.316672	1	1	RP11-647K16.1	_
ENSG00000273009	-2.796355	-2.316672	1	1	RP11-352G9.1	_
ENSG00000273019	-2.796355	-2.316672	1	1	RP11-508N22.13	_
ENSG00000273044	-2.796355	-2.316672	1	1	RP4-569D19.8	_
ENSG00000273063	-2.796355	-2.316672	1	1	RP11-334G22.1	_
ENSG00000273091	-2.796355	-2.316672	1	1	AP000255.6	_
ENSG00000273093	-2.796355	-2.316672	1	1	RP11-532L16.3	_
ENSG00000273184	-2.796355	-2.316672	1	1	RP11-212P7.3	_
ENSG00000273210	-2.796355	-2.316672	1	1	AP001437.1	_
ENSG00000273216	-2.796355	-2.316672	1	1	AC002059.10	_
ENSG00000273245	-2.796355	-2.316672	1	1	RP11-434P11.2	_
ENSG00000273293	-2.796355	-2.316672	1	1	RP11-445N20.3	_
ENSG00000273321	-2.796355	-2.316672	1	1	RP11-621L6.3	_
ENSG00000273361	-2.796355	-2.316672	1	1	RP11-378A13.2	_
ENSG00000273396	-2.796355	-2.316672	1	1	RP11-326I19.3	_

ENSG00000273419	-2.796355	-2.316672	1	1	RP4-751H13.7	_
ENSG00000273454	-2.796355	-2.316672	1	1	RP11-797D24.3	_
ENSG00000012223	0.92813	-2.038698	1	1	LTF	K17283
ENSG00000021300	0.92813	-2.038698	1	1	PLEKHB1	_
ENSG00000049283	0.92813	-2.038698	1	1	EPN3	K12471
ENSG00000064655	0.92813	-2.038698	1	1	EYA2	K17620
ENSG00000072952	0.92813	-2.038698	1	1	MRVI1	K12337
ENSG00000076344	0.92813	-2.038698	1	1	RGS11	K16449
ENSG00000080618	0.92813	-2.038698	1	1	CPB2	K01300
ENSG00000100604	0.92813	-2.038698	1	1	CHGA	_
ENSG00000101017	0.92813	-2.038698	1	1	CD40	K03160
ENSG00000101955	0.92813	-2.038698	1	1	SRPX	_
ENSG00000102081	0.92813	-2.038698	1	1	FMR1	K15516
ENSG00000103710	0.92813	-2.038698	1	1	RASL12	K07854
ENSG00000103742	0.92813	-2.038698	1	1	IGDCC4	_
ENSG00000103832	0.92813	-2.038698	1	1	GOLGA8UP	K10595
ENSG00000104537	0.92813	-2.038698	1	1	ANXA13	_
ENSG00000104879	0.92813	-2.038698	1	1	CKM	K00933
ENSG00000105679	0.92813	-2.038698	1	1	GAPDHS	K10705
ENSG00000106804	0.92813	-2.038698	1	1	C5	K03994
ENSG00000108556	0.92813	-2.038698	1	1	CHRNE	K04817
ENSG00000109927	0.92813	-2.038698	1	1	TECTA	K18273
ENSG00000110693	0.92813	-2.038698	1	1	SOX6	_
ENSG00000110900	0.92813	-2.038698	1	1	TSPAN11	K17352
ENSG00000111405	0.92813	-2.038698	1	1	ENDOU	K14648
ENSG00000111729	0.92813	-2.038698	1	1	CLEC4A	K10057
ENSG00000113722	0.92813	-2.038698	1	1	CDX1	K09312
ENSG00000114013	0.92813	-2.038698	1	1	CD86	K05413
ENSG00000114948	0.92813	-2.038698	1	1	ADAM23	K06837
ENSG00000117020	0.92813	-2.038698	1	1	AKT3	K04456
ENSG00000120332	0.92813	-2.038698	1	1	TNN	K06252
ENSG00000120500	0.92813	-2.038698	1	1	ARR3	K13801
ENSG00000120664	0.92813	-2.038698	1	1	SPG20OS	_
ENSG00000121053	0.92813	-2.038698	1	1	EPX	K10788
ENSG00000124399	0.92813	-2.038698	1	1	RP11-663P9.2	K03960
ENSG00000124882	0.92813	-2.038698	1	1	EREG	K09784
ENSG00000125931	0.92813	-2.038698	1	1	CITED1	_
ENSG00000126264	0.92813	-2.038698	1	1	HCST	K07988
ENSG00000128591	0.92813	-2.038698	1	1	FLNC	K04437
ENSG00000129990	0.92813	-2.038698	1	1	SYT5	_
ENSG00000130612	0.92813	-2.038698	1	1	CYP2G1P	K07417
ENSG00000131044	0.92813	-2.038698	1	1	TTLL9	K16603
ENSG00000131067	0.92813	-2.038698	1	1	GGT7	K00681
ENSG00000132437	0.92813	-2.038698	1	1	DDC	K01593
ENSG00000133063	0.92813	-2.038698	1	1	CHIT1	K01183
ENSG00000134775	0.92813	-2.038698	1	1	FHOD3	_
ENSG00000134827	0.92813	-2.038698	1	1	TCN1	_
ENSG00000135248	0.92813	-2.038698	1	1	FAM71F1	_
ENSG00000135454	0.92813	-2.038698	1	1	B4GALNT1	K00725
ENSG00000136931	0.92813	-2.038698	1	1	NR5A1	K08560
ENSG00000137463	0.92813	-2.038698	1	1	MGARP	_
ENSG00000137869	0.92813	-2.038698	1	1	CYP19A1	K07434
ENSG00000142538	0.92813	-2.038698	1	1	PTH2	_
ENSG00000143858	0.92813	-2.038698	1	1	SYT2	K15290
ENSG00000145248	0.92813	-2.038698	1	1	SLC10A4	K14344
ENSG00000145703	0.92813	-2.038698	1	1	IQGAP2	K05767
ENSG00000147465	0.92813	-2.038698	1	1	STAR	K16931
ENSG00000153060	0.92813	-2.038698	1	1	TEKT5	K18632

ENSG00000153093	0.92813	-2.038698	1	1	ACOXL	K04613
ENSG00000153930	0.92813	-2.038698	1	1	ANKFN1	_
ENSG00000154537	0.92813	-2.038698	1	1	FAM27C	_
ENSG00000154645	0.92813	-2.038698	1	1	CHODL	K10078
ENSG00000157211	0.92813	-2.038698	1	1	CDCP2	_
ENSG00000157551	0.92813	-2.038698	1	1	KCNJ15	K05008
ENSG00000158683	0.92813	-2.038698	1	1	PKD1L1	K04987
ENSG00000159261	0.92813	-2.038698	1	1	CLDN14	K06087
ENSG00000160951	0.92813	-2.038698	1	1	PTGER1	K04258
ENSG00000161798	0.92813	-2.038698	1	1	AQP5	K09867
ENSG00000162069	0.92813	-2.038698	1	1	CCDC64B	K16756
ENSG00000162490	0.92813	-2.038698	1	1	DRAXIN	K17985
ENSG00000162552	0.92813	-2.038698	1	1	WNT4	K00408
ENSG00000162777	0.92813	-2.038698	1	1	DENND2D	_
ENSG00000162888	0.92813	-2.038698	1	1	C1orf147	K07211
ENSG00000163106	0.92813	-2.038698	1	1	HPGDS	K01830
ENSG00000163508	0.92813	-2.038698	1	1	EOMES	K10173
ENSG00000163576	0.92813	-2.038698	1	1	EFHB	_
ENSG00000163815	0.92813	-2.038698	1	1	CLEC3B	K17520
ENSG00000164485	0.92813	-2.038698	1	1	IL22RA2	K05139
ENSG00000164647	0.92813	-2.038698	1	1	STEAP1	K14737
ENSG00000165462	0.92813	-2.038698	1	1	PHOX2A	K09330
ENSG00000166387	0.92813	-2.038698	1	1	PPFIBP2	_
ENSG00000166492	0.92813	-2.038698	1	1	FAM86GP	_
ENSG00000167654	0.92813	-2.038698	1	1	ATCAY	K08857
ENSG00000167774	0.92813	-2.038698	1	1	NDUFA7	K03951
ENSG00000168918	0.92813	-2.038698	1	1	INPP5D	K03084
ENSG00000169896	0.92813	-2.038698	1	1	ITGAM	K06461
ENSG00000170748	0.92813	-2.038698	1	1	RBMXL2	K12885
ENSG00000171385	0.92813	-2.038698	1	1	KCND3	K04893
ENSG00000171812	0.92813	-2.038698	1	1	COL8A2	_
ENSG00000171931	0.92813	-2.038698	1	1	FBXW10	K10266
ENSG00000172901	0.92813	-2.038698	1	1	AQPEP	K13724
ENSG00000173626	0.92813	-2.038698	1	1	TRAPPC3L	_
ENSG00000173678	0.92813	-2.038698	1	1	SPDYE2B	K08694
ENSG00000174004	0.92813	-2.038698	1	1	NRROS	_
ENSG00000174502	0.92813	-2.038698	1	1	SLC26A9	K14706
ENSG00000175356	0.92813	-2.038698	1	1	SCUBE2	_
ENSG00000176236	0.92813	-2.038698	1	1	C10orf111	K17985
ENSG00000176349	0.92813	-2.038698	1	1	AC110781.3	_
ENSG00000176444	0.92813	-2.038698	1	1	CLK2	K08823
ENSG00000176510	0.92813	-2.038698	1	1	OR10AC1P	K04257
ENSG00000177182	0.92813	-2.038698	1	1	CLVS1	K16467
ENSG00000177359	0.92813	-2.038698	1	1	RP11-551L14.1	_
ENSG00000177993	0.92813	-2.038698	1	1	ZNRF3-AS1	_
ENSG00000178193	0.92813	-2.038698	1	1	RP4-682C21.5	_
ENSG00000178412	0.92813	-2.038698	1	1	RP11-567M16.3	_
ENSG00000179061	0.92813	-2.038698	1	1	AC012074.1	_
ENSG00000179165	0.92813	-2.038698	1	1	PXT1	_
ENSG00000179219	0.92813	-2.038698	1	1	LINC00311	_
ENSG00000179776	0.92813	-2.038698	1	1	CDH5	K06533
ENSG00000180210	0.92813	-2.038698	1	1	F2	K01313
ENSG00000180211	0.92813	-2.038698	1	1	RP1-278E11.3	K02894
ENSG00000182218	0.92813	-2.038698	1	1	HHIPL1	_
ENSG00000183484	0.92813	-2.038698	1	1	GPR132	K08426
ENSG00000183535	0.92813	-2.038698	1	1	COL18A1-AS1	_
ENSG00000184040	0.92813	-2.038698	1	1	TMEM236	K14404
ENSG00000184492	0.92813	-2.038698	1	1	FOXD4L1	K09397

ENSG00000185038	0.92813	-2.038698	1	1	MROH2A	_
ENSG00000186207	0.92813	-2.038698	1	1	LCE5A	_
ENSG00000186393	0.92813	-2.038698	1	1	KRT26	K07604
ENSG00000186471	0.92813	-2.038698	1	1	AKAP14	K16530
ENSG00000187627	0.92813	-2.038698	1	1	RGPD1	K12172
ENSG00000188112	0.92813	-2.038698	1	1	C6orf132	K17985
ENSG00000188163	0.92813	-2.038698	1	1	FAM166A	_
ENSG00000188783	0.92813	-2.038698	1	1	PRELP	K08125
ENSG00000188916	0.92813	-2.038698	1	1	FAM196A	_
ENSG00000189051	0.92813	-2.038698	1	1	RNF222	K17985
ENSG00000189167	0.92813	-2.038698	1	1	ZAR1L	K18762
ENSG00000189212	0.92813	-2.038698	1	1	DPY19L2P1	_
ENSG00000196183	0.92813	-2.038698	1	1	RPS2P4	K02981
ENSG00000196189	0.92813	-2.038698	1	1	SEMA4A	K06521
ENSG00000196532	0.92813	-2.038698	1	1	HIST1H3C	K11253
ENSG00000196611	0.92813	-2.038698	1	1	MMP1	K01388
ENSG00000196993	0.92813	-2.038698	1	1	NPIPB9	_
ENSG00000197446	0.92813	-2.038698	1	1	CYP2F1	K07416
ENSG00000197457	0.92813	-2.038698	1	1	STMN3	_
ENSG00000197697	0.92813	-2.038698	1	1	HIST1H2BE	K11252
ENSG00000198771	0.92813	-2.038698	1	1	RCSD1	K09488
ENSG00000199509	0.92813	-2.038698	1	1	RNA5SP477	_
ENSG00000200135	0.92813	-2.038698	1	1	Y_RNA	_
ENSG00000200170	0.92813	-2.038698	1	1	Y_RNA	_
ENSG00000200309	0.92813	-2.038698	1	1	Y_RNA	_
ENSG00000200815	0.92813	-2.038698	1	1	RNU6-1091P	_
ENSG00000201151	0.92813	-2.038698	1	1	SNORD56	_
ENSG00000201944	0.92813	-2.038698	1	1	SNORA72	_
ENSG00000202260	0.92813	-2.038698	1	1	RN7SKP69	_
ENSG00000202434	0.92813	-2.038698	1	1	SNORA4	_
ENSG00000202461	0.92813	-2.038698	1	1	Y_RNA	_
ENSG00000203589	0.92813	-2.038698	1	1	RP5-886K2.1	K17985
ENSG00000203630	0.92813	-2.038698	1	1	AL136531.1	K04613
ENSG00000204055	0.92813	-2.038698	1	1	RP11-247A12.2	_
ENSG00000204388	0.92813	-2.038698	1	1	HSPA1B	K03283
ENSG00000204583	0.92813	-2.038698	1	1	LRCOL1	_
ENSG00000205018	0.92813	-2.038698	1	1	RP11-830F9.6	_
ENSG00000205022	0.92813	-2.038698	1	1	PABPN1L	K14396
ENSG00000205217	0.92813	-2.038698	1	1	FAM106B	_
ENSG00000206603	0.92813	-2.038698	1	1	SNORA22	_
ENSG00000206634	0.92813	-2.038698	1	1	SNORA22	_
ENSG00000206728	0.92813	-2.038698	1	1	Y_RNA	_
ENSG00000206848	0.92813	-2.038698	1	1	RNU6-890P	_
ENSG00000207588	0.92813	-2.038698	1	1	MIR593	_
ENSG00000213394	0.92813	-2.038698	1	1	RP4-747G18.5	K02998
ENSG00000213432	0.92813	-2.038698	1	1	RP11-624L12.1	K02880
ENSG00000213613	0.92813	-2.038698	1	1	RP11-380G5.3	K02868
ENSG00000213714	0.92813	-2.038698	1	1	FAM209B	_
ENSG00000214359	0.92813	-2.038698	1	1	RPL18P10	K02883
ENSG00000214546	0.92813	-2.038698	1	1	AC087491.2	_
ENSG00000214548	0.92813	-2.038698	1	1	MEG3	_
ENSG00000214875	0.92813	-2.038698	1	1	MED28P1	K15141
ENSG00000215045	0.92813	-2.038698	1	1	GRID2IP	_
ENSG00000215811	0.92813	-2.038698	1	1	BTNL10	_
ENSG00000215915	0.92813	-2.038698	1	1	ATAD3C	K17681
ENSG00000216624	0.92813	-2.038698	1	1	GAPDHP72	K00134
ENSG00000216863	0.92813	-2.038698	1	1	LY86-AS1	_
ENSG00000217442	0.92813	-2.038698	1	1	SYCE3	K19536

ENSG00000219392	0.92813	-2.038698	1	1	RP1-265C24.5	K09228
ENSG00000220305	0.92813	-2.038698	1	1	HNRNPH1P1	K12898
ENSG00000221340	0.92813	-2.038698	1	1	RNU6ATAC18P	_
ENSG00000222020	0.92813	-2.038698	1	1	AC062017.1	_
ENSG00000222040	0.92813	-2.038698	1	1	ADRA2B	K04139
ENSG00000222389	0.92813	-2.038698	1	1	RNU2-28P	_
ENSG00000222800	0.92813	-2.038698	1	1	RNU2-62P	_
ENSG00000223060	0.92813	-2.038698	1	1	Y_RNA	_
ENSG00000223117	0.92813	-2.038698	1	1	RN7SKP296	_
ENSG00000223390	0.92813	-2.038698	1	1	RP11-91A18.4	_
ENSG00000223791	0.92813	-2.038698	1	1	UBE2E1-AS1	_
ENSG00000223923	0.92813	-2.038698	1	1	AC010136.2	_
ENSG00000224124	0.92813	-2.038698	1	1	POM121L10P	_
ENSG00000224269	0.92813	-2.038698	1	1	AP000697.6	_
ENSG00000224644	0.92813	-2.038698	1	1	RP11-16L21.7	_
ENSG00000224677	0.92813	-2.038698	1	1	PDIA3P2	K08056
ENSG00000224715	0.92813	-2.038698	1	1	CITF22-49D8.1	_
ENSG00000224750	0.92813	-2.038698	1	1	RP11-94M14.2	_
ENSG00000224885	0.92813	-2.038698	1	1	TSSC1-IT1	_
ENSG00000225093	0.92813	-2.038698	1	1	RPL3P7	K02925
ENSG00000225099	0.92813	-2.038698	1	1	ATP6V1E1P1	K02150
ENSG00000225278	0.92813	-2.038698	1	1	RP11-782C8.5	_
ENSG00000225353	0.92813	-2.038698	1	1	RP11-292F9.1	_
ENSG00000225643	0.92813	-2.038698	1	1	RP11-70P17.1	_
ENSG00000225805	0.92813	-2.038698	1	1	CTD-2313N18.7	K10803
ENSG00000226002	0.92813	-2.038698	1	1	RP11-460N20.5	K03121
ENSG00000226104	0.92813	-2.038698	1	1	AC011475.1	K17557
ENSG00000226245	0.92813	-2.038698	1	1	ZNF32-AS1	_
ENSG00000226247	0.92813	-2.038698	1	1	AC005034.3	K15171
ENSG00000226310	0.92813	-2.038698	1	1	RP3-323P24.3	_
ENSG00000226438	0.92813	-2.038698	1	1	RP11-420K8.1	_
ENSG00000226652	0.92813	-2.038698	1	1	PSMD10P2	K06694
ENSG00000226851	0.92813	-2.038698	1	1	AC004112.4	_
ENSG00000226963	0.92813	-2.038698	1	1	AC078883.4	_
ENSG00000227057	0.92813	-2.038698	1	1	WDR46	K14768
ENSG00000227145	0.92813	-2.038698	1	1	IL21-AS1	_
ENSG00000227203	0.92813	-2.038698	1	1	SUB1P1	_
ENSG00000227321	0.92813	-2.038698	1	1	MTND4P15	_
ENSG00000227473	0.92813	-2.038698	1	1	TSSK5P1	_
ENSG00000227620	0.92813	-2.038698	1	1	ALG1L8P	K03842
ENSG00000227684	0.92813	-2.038698	1	1	CROCCP4	K16469
ENSG00000227694	0.92813	-2.038698	1	1	AC005884.1	K02893
ENSG00000228028	0.92813	-2.038698	1	1	AC069257.8	_
ENSG00000228037	0.92813	-2.038698	1	1	RP3-395M20.9	_
ENSG00000228187	0.92813	-2.038698	1	1	RP11-363H12.1	K07050
ENSG00000228264	0.92813	-2.038698	1	1	RP11-350G8.7	K03031
ENSG00000228389	0.92813	-2.038698	1	1	AC068039.4	_
ENSG00000228434	0.92813	-2.038698	1	1	AC004951.6	_
ENSG00000228478	0.92813	-2.038698	1	1	RP1-290I10.3	_
ENSG00000228540	0.92813	-2.038698	1	1	AC073326.3	_
ENSG00000228960	0.92813	-2.038698	1	1	OR2A9P	K04257
ENSG00000229153	0.92813	-2.038698	1	1	EPHA1-AS1	_
ENSG00000229184	0.92813	-2.038698	1	1	RP11-255J3.3	K02138
ENSG00000229327	0.92813	-2.038698	1	1	RP11-135A24.2	_
ENSG00000229930	0.92813	-2.038698	1	1	RP11-504P24.5	K10305
ENSG00000229956	0.92813	-2.038698	1	1	ZRANB2-AS2	_
ENSG00000230148	0.92813	-2.038698	1	1	HOXB-AS1	_
ENSG00000230183	0.92813	-2.038698	1	1	CNOT6LP1	K12603

ENSG00000230415	0.92813	-2.038698	1	1	RP5-902P8.10	_
ENSG00000230433	0.92813	-2.038698	1	1	RP1-20B11.2	_
ENSG00000230510	0.92813	-2.038698	1	1	PPP5D1	K04460
ENSG00000230581	0.92813	-2.038698	1	1	RP11-390F4.8	K05692
ENSG00000230626	0.92813	-2.038698	1	1	RP11-286H14.4	K04369
ENSG00000230825	0.92813	-2.038698	1	1	AC005532.5	_
ENSG00000230912	0.92813	-2.038698	1	1	RP3-508I15.10	_
ENSG00000230952	0.92813	-2.038698	1	1	AC099344.2	_
ENSG00000230953	0.92813	-2.038698	1	1	RP4-631H13.6	K07604
ENSG00000231057	0.92813	-2.038698	1	1	RP11-122M14.1	_
ENSG00000231460	0.92813	-2.038698	1	1	RP11-321L2.1	_
ENSG00000231528	0.92813	-2.038698	1	1	FAM225A	_
ENSG00000231705	0.92813	-2.038698	1	1	RP11-432J24.2	_
ENSG00000231747	0.92813	-2.038698	1	1	AC079922.2	K02969
ENSG00000231831	0.92813	-2.038698	1	1	MTHFD1P1	K00288
ENSG00000232019	0.92813	-2.038698	1	1	AC074183.4	_
ENSG00000232057	0.92813	-2.038698	1	1	AC093390.1	_
ENSG00000232059	0.92813	-2.038698	1	1	RP11-173A6.2	K02922
ENSG00000232104	0.92813	-2.038698	1	1	RP11-509J21.1	_
ENSG00000232118	0.92813	-2.038698	1	1	BACH1-AS1	_
ENSG00000232134	0.92813	-2.038698	1	1	RPS15AP12	K02957
ENSG00000232162	0.92813	-2.038698	1	1	USP12-AS1	_
ENSG00000232546	0.92813	-2.038698	1	1	RP11-458F8.1	K03121
ENSG00000232618	0.92813	-2.038698	1	1	RP11-439L18.1	_
ENSG00000232627	0.92813	-2.038698	1	1	AC021876.4	K02910
ENSG00000232662	0.92813	-2.038698	1	1	LDHBP1	K00016
ENSG00000233045	0.92813	-2.038698	1	1	AC097523.1	K02877
ENSG00000233098	0.92813	-2.038698	1	1	RP11-344E13.3	_
ENSG00000233232	0.92813	-2.038698	1	1	NPIPB7	_
ENSG00000233297	0.92813	-2.038698	1	1	RASA4DP	K17630
ENSG00000233334	0.92813	-2.038698	1	1	FAM53B-AS1	_
ENSG00000233578	0.92813	-2.038698	1	1	RP5-1022P6.3	K03259
ENSG00000233673	0.92813	-2.038698	1	1	ANAPC1P1	K03348
ENSG00000233838	0.92813	-2.038698	1	1	DPH3P1	K15455
ENSG00000233851	0.92813	-2.038698	1	1	LATS2-AS1	_
ENSG00000233912	0.92813	-2.038698	1	1	AC026202.3	_
ENSG00000233994	0.92813	-2.038698	1	1	GDI2P2	K17255
ENSG00000234003	0.92813	-2.038698	1	1	CTD-3105H18.11	_
ENSG00000234396	0.92813	-2.038698	1	1	RP11-181G12.4	_
ENSG00000234969	0.92813	-2.038698	1	1	RP13-33H18.1	K07050
ENSG00000235044	0.92813	-2.038698	1	1	PPIAP3	K03767
ENSG00000235236	0.92813	-2.038698	1	1	RP13-131K19.1	_
ENSG00000235488	0.92813	-2.038698	1	1	JARID2-AS1	_
ENSG00000235590	0.92813	-2.038698	1	1	GNAS-AS1	_
ENSG00000235724	0.92813	-2.038698	1	1	AC009299.2	_
ENSG00000235790	0.92813	-2.038698	1	1	RP11-73M7.6	_
ENSG00000235834	0.92813	-2.038698	1	1	RP1-60N8.1	_
ENSG00000235847	0.92813	-2.038698	1	1	LDHAP7	K00016
ENSG00000235904	0.92813	-2.038698	1	1	RBMS3-AS3	_
ENSG00000236045	0.92813	-2.038698	1	1	RP3-467K16.7	_
ENSG00000236375	0.92813	-2.038698	1	1	POU5F1P5	K09367
ENSG00000236577	0.92813	-2.038698	1	1	SNRPGP14	_
ENSG00000236627	0.92813	-2.038698	1	1	RP11-252P19.1	_
ENSG00000236733	0.92813	-2.038698	1	1	RP11-203L2.4	_
ENSG00000236844	0.92813	-2.038698	1	1	AC091633.2	K02896
ENSG00000236932	0.92813	-2.038698	1	1	CEACAMP8	K06499
ENSG00000236933	0.92813	-2.038698	1	1	RP11-439A17.7	_
ENSG00000237135	0.92813	-2.038698	1	1	DDX10P1	_

ENSG00000237471	0.92813	-2.038698	1	1	AC073115.6	_
ENSG00000237567	0.92813	-2.038698	1	1	RP3-359N14.2	_
ENSG00000237704	0.92813	-2.038698	1	1	AP004289.2	K18403
ENSG00000238001	0.92813	-2.038698	1	1	RP11-555J4.2	K11095
ENSG00000238098	0.92813	-2.038698	1	1	ABCA17P	K05643
ENSG00000239063	0.92813	-2.038698	1	1	snoU13	_
ENSG00000239636	0.92813	-2.038698	1	1	RP4-728D4.2	_
ENSG00000239828	0.92813	-2.038698	1	1	RP11-446H18.5	_
ENSG00000240007	0.92813	-2.038698	1	1	RP6-206I17.4	_
ENSG00000240132	0.92813	-2.038698	1	1	ETF1P2	K03265
ENSG00000240216	0.92813	-2.038698	1	1	CPHL1P	K13624
ENSG00000240373	0.92813	-2.038698	1	1	SEC62-AS1	_
ENSG00000240695	0.92813	-2.038698	1	1	RP11-102M11.1	K09528
ENSG00000240751	0.92813	-2.038698	1	1	RP11-553D4.2	K05956
ENSG00000240771	0.92813	-2.038698	1	1	ARHGEF25	_
ENSG00000240964	0.92813	-2.038698	1	1	RN7SL751P	_
ENSG00000241475	0.92813	-2.038698	1	1	RP4-781K5.5	_
ENSG00000241484	0.92813	-2.038698	1	1	ARHGAP8	K18034
ENSG00000242602	0.92813	-2.038698	1	1	CTD-2339M3.1	K02917
ENSG00000242797	0.92813	-2.038698	1	1	GLYCTK-AS1	_
ENSG00000242876	0.92813	-2.038698	1	1	RN7SL812P	_
ENSG00000243107	0.92813	-2.038698	1	1	AC000120.7	_
ENSG00000243504	0.92813	-2.038698	1	1	RPS23P1	K02973
ENSG00000243550	0.92813	-2.038698	1	1	RP11-483E7.1	_
ENSG00000243650	0.92813	-2.038698	1	1	RN7SL834P	_
ENSG00000243961	0.92813	-2.038698	1	1	RP5-839B4.8	_
ENSG00000243978	0.92813	-2.038698	1	1	RGAG1	_
ENSG00000244219	0.92813	-2.038698	1	1	GS1-259H13.2	_
ENSG00000244265	0.92813	-2.038698	1	1	SIAH2-AS1	_
ENSG00000244535	0.92813	-2.038698	1	1	RP1-181J22.1	K02915
ENSG00000244752	0.92813	-2.038698	1	1	CRYBB2	_
ENSG00000245552	0.92813	-2.038698	1	1	RP11-712B9.2	_
ENSG00000245748	0.92813	-2.038698	1	1	RP11-367J11.2	_
ENSG00000248318	0.92813	-2.038698	1	1	RP11-713M15.1	_
ENSG00000248377	0.92813	-2.038698	1	1	HMGN1P9	K11299
ENSG00000248445	0.92813	-2.038698	1	1	CTB-118N6.3	_
ENSG00000248599	0.92813	-2.038698	1	1	RP11-302J23.1	_
ENSG00000248643	0.92813	-2.038698	1	1	RBM14-RBM4	K13187
ENSG00000248648	0.92813	-2.038698	1	1	RP11-485M7.1	K03948
ENSG00000248774	0.92813	-2.038698	1	1	RP11-798M19.3	_
ENSG00000249084	0.92813	-2.038698	1	1	RP11-376O6.2	_
ENSG00000249188	0.92813	-2.038698	1	1	ENPP7P1	K12354
ENSG00000249502	0.92813	-2.038698	1	1	AC006160.5	_
ENSG00000249623	0.92813	-2.038698	1	1	RP11-610J23.1	_
ENSG00000249690	0.92813	-2.038698	1	1	RP11-1336O20.2	_
ENSG00000249911	0.92813	-2.038698	1	1	RP11-122C5.1	_
ENSG00000250130	0.92813	-2.038698	1	1	RP11-616K22.1	K00134
ENSG00000250131	0.92813	-2.038698	1	1	RP11-130F10.1	_
ENSG00000250192	0.92813	-2.038698	1	1	RP11-300M6.1	_
ENSG00000250556	0.92813	-2.038698	1	1	CCDC34P1	_
ENSG00000250917	0.92813	-2.038698	1	1	RP4-785G19.5	_
ENSG00000251050	0.92813	-2.038698	1	1	RP11-168A11.4	_
ENSG00000251127	0.92813	-2.038698	1	1	RP11-280G9.1	_
ENSG00000251448	0.92813	-2.038698	1	1	RP11-71E19.2	_
ENSG00000251520	0.92813	-2.038698	1	1	RP11-46A10.6	_
ENSG00000251548	0.92813	-2.038698	1	1	RP11-215G15.4	_
ENSG00000251665	0.92813	-2.038698	1	1	RP11-700H6.2	_
ENSG00000251720	0.92813	-2.038698	1	1	RNU7-123P	_

ENSG00000251749	0.92813	-2.038698	1	1	U3	_
ENSG00000252847	0.92813	-2.038698	1	1	RNU2-46P	_
ENSG00000252910	0.92813	-2.038698	1	1	RNU7-177P	_
ENSG00000253194	0.92813	-2.038698	1	1	RP11-351A11.1	_
ENSG00000253214	0.92813	-2.038698	1	1	RP11-1149M10.2	_
ENSG00000253618	0.92813	-2.038698	1	1	GRPEL2-AS1	_
ENSG00000253754	0.92813	-2.038698	1	1	RP11-35G22.1	_
ENSG00000254420	0.92813	-2.038698	1	1	RP11-452H21.1	_
ENSG00000254431	0.92813	-2.038698	1	1	RP11-550A5.2	_
ENSG00000254501	0.92813	-2.038698	1	1	AP003068.9	_
ENSG00000254510	0.92813	-2.038698	1	1	RP11-867G23.10	_
ENSG00000254662	0.92813	-2.038698	1	1	RP11-872D17.4	_
ENSG00000254688	0.92813	-2.038698	1	1	RP11-47J17.3	_
ENSG00000254740	0.92813	-2.038698	1	1	RP11-334E6.3	_
ENSG00000254741	0.92813	-2.038698	1	1	RP11-661A12.7	_
ENSG00000255478	0.92813	-2.038698	1	1	RP11-867O8.5	_
ENSG00000255524	0.92813	-2.038698	1	1	NPIPB8	K01896
ENSG00000255526	0.92813	-2.038698	1	1	NEDD8-MDP1	K17619
ENSG00000255651	0.92813	-2.038698	1	1	RP11-466C23.4	_
ENSG00000255753	0.92813	-2.038698	1	1	RP11-22B23.2	K18461
ENSG00000255843	0.92813	-2.038698	1	1	AP000593.7	_
ENSG00000256037	0.92813	-2.038698	1	1	MRPL40P1	_
ENSG00000256268	0.92813	-2.038698	1	1	RP11-221N13.3	_
ENSG00000257443	0.92813	-2.038698	1	1	RP11-150C16.1	_
ENSG00000257509	0.92813	-2.038698	1	1	RP11-762I7.4	_
ENSG00000257545	0.92813	-2.038698	1	1	RP11-144F15.1	K03021
ENSG00000257752	0.92813	-2.038698	1	1	RP11-474L23.3	_
ENSG00000257817	0.92813	-2.038698	1	1	RP4-601P9.2	_
ENSG00000257893	0.92813	-2.038698	1	1	RP11-587P21.2	_
ENSG00000257896	0.92813	-2.038698	1	1	RP11-210N13.1	_
ENSG00000258082	0.92813	-2.038698	1	1	RP11-443B7.3	_
ENSG00000258303	0.92813	-2.038698	1	1	RP11-887P2.6	_
ENSG00000258446	0.92813	-2.038698	1	1	RP11-895M11.2	K18171
ENSG00000258461	0.92813	-2.038698	1	1	RP11-164J13.1	_
ENSG00000258485	0.92813	-2.038698	1	1	SRMP2	K00797
ENSG00000258527	0.92813	-2.038698	1	1	ASB9P1	K10331
ENSG00000258593	0.92813	-2.038698	1	1	CTD-3051D23.4	_
ENSG00000258623	0.92813	-2.038698	1	1	CTD-2325P2.3	_
ENSG00000258673	0.92813	-2.038698	1	1	RP4-773N10.6	_
ENSG00000259120	0.92813	-2.038698	1	1	SMIM6	_
ENSG00000259177	0.92813	-2.038698	1	1	RP11-154B12.3	_
ENSG00000259322	0.92813	-2.038698	1	1	RP11-762H8.1	_
ENSG00000259417	0.92813	-2.038698	1	1	RP11-2E17.1	_
ENSG00000259422	0.92813	-2.038698	1	1	RP11-593F23.1	_
ENSG00000259453	0.92813	-2.038698	1	1	RP11-815J21.1	_
ENSG00000259652	0.92813	-2.038698	1	1	RP11-797A18.3	_
ENSG00000260101	0.92813	-2.038698	1	1	RP11-568N6.1	_
ENSG00000260179	0.92813	-2.038698	1	1	RP5-902P8.12	_
ENSG00000260229	0.92813	-2.038698	1	1	RP11-391L3.5	K03767
ENSG00000260339	0.92813	-2.038698	1	1	HEXA-AS1	_
ENSG00000260381	0.92813	-2.038698	1	1	RP11-429P3.5	_
ENSG00000260799	0.92813	-2.038698	1	1	KRT8P50	K07605
ENSG00000260874	0.92813	-2.038698	1	1	RP11-715J22.4	_
ENSG00000260934	0.92813	-2.038698	1	1	CTA-363E6.7	_
ENSG00000261002	0.92813	-2.038698	1	1	RP11-546B15.1	_
ENSG00000261079	0.92813	-2.038698	1	1	RP11-252A24.3	_
ENSG00000261143	0.92813	-2.038698	1	1	ADAMTS7P3	K08622
ENSG00000261229	0.92813	-2.038698	1	1	RP11-38G5.4	_

ENSG00000261266	0.92813	-2.038698	1	1	CTD-2196E14.5	_
ENSG00000261302	0.92813	-2.038698	1	1	RP11-343H19.1	_
ENSG00000261521	0.92813	-2.038698	1	1	RP11-408H20.1	_
ENSG00000261529	0.92813	-2.038698	1	1	RP13-487P22.1	_
ENSG00000261566	0.92813	-2.038698	1	1	CTD-2144E22.6	_
ENSG00000261707	0.92813	-2.038698	1	1	RP11-264M12.2	_
ENSG00000261723	0.92813	-2.038698	1	1	CTD-2196E14.3	_
ENSG00000261744	0.92813	-2.038698	1	1	RP11-21B21.4	_
ENSG00000261775	0.92813	-2.038698	1	1	RP11-10O17.3	_
ENSG00000262172	0.92813	-2.038698	1	1	CTD-2529O21.1	_
ENSG00000262304	0.92813	-2.038698	1	1	SHPK	K11214
ENSG00000262362	0.92813	-2.038698	1	1	LA16c-380H5.2	_
ENSG00000262636	0.92813	-2.038698	1	1	CTD-3088G3.4	K02881
ENSG00000262681	0.92813	-2.038698	1	1	RP11-311F12.1	_
ENSG00000263361	0.92813	-2.038698	1	1	MIR378H	_
ENSG00000263764	0.92813	-2.038698	1	1	SNORD43	_
ENSG00000263809	0.92813	-2.038698	1	1	RP11-849F2.7	K02898
ENSG00000264057	0.92813	-2.038698	1	1	RP11-583F2.1	K11728
ENSG00000264377	0.92813	-2.038698	1	1	MIR4671	_
ENSG00000264424	0.92813	-2.038698	1	1	MYH4	K10352
ENSG00000264475	0.92813	-2.038698	1	1	RP11-76K13.3	_
ENSG00000264590	0.92813	-2.038698	1	1	RN7SL512P	_
ENSG00000264653	0.92813	-2.038698	1	1	MIR5194	_
ENSG00000264801	0.92813	-2.038698	1	1	RP11-149I2.5	K00772
ENSG00000265137	0.92813	-2.038698	1	1	MIR3192	_
ENSG00000265451	0.92813	-2.038698	1	1	RP11-204L24.2	_
ENSG00000265677	0.92813	-2.038698	1	1	AL137229.1	_
ENSG00000265879	0.92813	-2.038698	1	1	MIR4748	_
ENSG00000265984	0.92813	-2.038698	1	1	RP11-699A5.2	_
ENSG00000266289	0.92813	-2.038698	1	1	RP11-1C8.6	_
ENSG00000266494	0.92813	-2.038698	1	1	MIR4641	_
ENSG00000266537	0.92813	-2.038698	1	1	CTD-2510F5.2	K08694
ENSG00000266644	0.92813	-2.038698	1	1	RP11-927P21.2	_
ENSG00000266850	0.92813	-2.038698	1	1	RP11-370A5.1	_
ENSG00000266933	0.92813	-2.038698	1	1	AC005775.2	_
ENSG00000266975	0.92813	-2.038698	1	1	CTC-425F1.2	_
ENSG00000267026	0.92813	-2.038698	1	1	RP11-92C4.3	_
ENSG00000267183	0.92813	-2.038698	1	1	AC010525.6	_
ENSG00000267228	0.92813	-2.038698	1	1	IER3IP1	_
ENSG00000267345	0.92813	-2.038698	1	1	CTD-2293H3.2	_
ENSG00000267462	0.92813	-2.038698	1	1	RP11-866E20.3	_
ENSG00000267549	0.92813	-2.038698	1	1	AC006116.17	_
ENSG00000267561	0.92813	-2.038698	1	1	RP5-1052I5.2	K02513
ENSG00000267567	0.92813	-2.038698	1	1	CTD-2538C1.3	_
ENSG00000267568	0.92813	-2.038698	1	1	RP11-87G24.3	_
ENSG00000267666	0.92813	-2.038698	1	1	AC004156.3	_
ENSG00000267677	0.92813	-2.038698	1	1	RP11-27G24.1	_
ENSG00000267780	0.92813	-2.038698	1	1	RP11-154H12.3	_
ENSG00000267804	0.92813	-2.038698	1	1	RP11-665N17.4	_
ENSG00000268223	0.92813	-2.038698	1	1	ARL14EPL	_
ENSG00000268288	0.92813	-2.038698	1	1	RP11-98D18.16	_
ENSG00000268412	0.92813	-2.038698	1	1	AC013449.1	K15448
ENSG00000268520	0.92813	-2.038698	1	1	CTD-2616J11.10	_
ENSG00000268521	0.92813	-2.038698	1	1	VN1R83P	_
ENSG00000268578	0.92813	-2.038698	1	1	AL354718.1	_
ENSG00000268583	0.92813	-2.038698	1	1	CTC-453G23.8	_
ENSG00000268603	0.92813	-2.038698	1	1	RP11-316O14.1	_
ENSG00000269066	0.92813	-2.038698	1	1	CTD-2528A14.5	_

ENSG00000269489	0.92813	-2.038698	1	1	RP11-98D18.17	_
ENSG00000269624	0.92813	-2.038698	1	1	AL138847.1	K09488
ENSG00000269753	0.92813	-2.038698	1	1	AL589739.1	_
ENSG00000269846	0.92813	-2.038698	1	1	RBL1	K04681
ENSG00000269989	0.92813	-2.038698	1	1	RP11-635N19.3	_
ENSG00000270024	0.92813	-2.038698	1	1	C8orf44-SGK3	K13304
ENSG00000270169	0.92813	-2.038698	1	1	CTC-1337H24.2	_
ENSG00000270178	0.92813	-2.038698	1	1	RP11-494H4.3	_
ENSG00000270210	0.92813	-2.038698	1	1	RP11-373D23.3	_
ENSG00000270269	0.92813	-2.038698	1	1	RP11-697H9.4	K09647
ENSG00000270285	0.92813	-2.038698	1	1	RP11-1029M24.4	K02978
ENSG00000270429	0.92813	-2.038698	1	1	KNOP1P2	_
ENSG00000270720	0.92813	-2.038698	1	1	RP11-84C13.2	_
ENSG00000270878	0.92813	-2.038698	1	1	CTD-2302E22.5	K00639
ENSG00000270919	0.92813	-2.038698	1	1	RP11-379K22.3	_
ENSG00000270986	0.92813	-2.038698	1	1	RP11-123C21.2	K10802
ENSG00000270987	0.92813	-2.038698	1	1	RP3-467N11.2	_
ENSG00000271151	0.92813	-2.038698	1	1	RP11-394I13.2	_
ENSG00000271200	0.92813	-2.038698	1	1	RP11-430G17.3	_
ENSG00000271631	0.92813	-2.038698	1	1	RP11-408O19.5	_
ENSG00000271727	0.92813	-2.038698	1	1	RP11-532F6.4	_
ENSG00000271748	0.92813	-2.038698	1	1	MIR92B	_
ENSG00000271840	0.92813	-2.038698	1	1	RP1-224A6.9	_
ENSG00000271847	0.92813	-2.038698	1	1	RP4-740C4.8	_
ENSG00000271893	0.92813	-2.038698	1	1	RP11-762E8.1	_
ENSG00000271913	0.92813	-2.038698	1	1	RP1-111C20.4	_
ENSG00000272100	0.92813	-2.038698	1	1	RP11-155O18.6	_
ENSG00000272110	0.92813	-2.038698	1	1	Y_RNA	_
ENSG00000272138	0.92813	-2.038698	1	1	RP11-27N21.3	_
ENSG00000272146	0.92813	-2.038698	1	1	RP11-755B10.4	_
ENSG00000272192	0.92813	-2.038698	1	1	CTD-2532N20.1	_
ENSG00000272375	0.92813	-2.038698	1	1	RP11-51J9.6	_
ENSG00000272529	0.92813	-2.038698	1	1	RP11-415F23.4	_
ENSG00000272682	0.92813	-2.038698	1	1	AC004471.10	_
ENSG00000272691	0.92813	-2.038698	1	1	RP11-290M5.4	_
ENSG00000272715	0.92813	-2.038698	1	1	RP4-753F5.1	_
ENSG00000273035	0.92813	-2.038698	1	1	RP11-449G16.1	_
ENSG00000273076	0.92813	-2.038698	1	1	RP3-508I15.22	_
ENSG00000273107	0.92813	-2.038698	1	1	RP11-165A20.3	_
ENSG00000273125	0.92813	-2.038698	1	1	RP11-115H18.1	_
ENSG00000273240	0.92813	-2.038698	1	1	RP11-455J20.3	_
ENSG00000273297	0.92813	-2.038698	1	1	RP11-38M8.1	_
ENSG00000004139	-0.907046	-1.474542	1	1	SARM1	_
ENSG00000047597	-0.907046	-1.474542	1	1	XK	K19522
ENSG00000070190	-0.907046	-1.474542	1	1	DAPP1	K12229
ENSG00000074181	-0.907046	-1.474542	1	1	NOTCH3	K02599
ENSG00000102032	-0.907046	-1.474542	1	1	RENBP	K01787
ENSG00000105967	-0.907046	-1.474542	1	1	TFEC	K15591
ENSG00000117013	-0.907046	-1.474542	1	1	KCNQ4	K04929
ENSG00000118407	-0.907046	-1.474542	1	1	FILIP1	K09228
ENSG00000123901	-0.907046	-1.474542	1	1	GPR83	K04210
ENSG00000124493	-0.907046	-1.474542	1	1	GRM4	K04607
ENSG00000129214	-0.907046	-1.474542	1	1	SHBG	_
ENSG00000129682	-0.907046	-1.474542	1	1	FGF13	K04358
ENSG00000134183	-0.907046	-1.474542	1	1	GNAT2	K04631
ENSG00000140675	-0.907046	-1.474542	1	1	SLC5A2	K14382
ENSG00000142700	-0.907046	-1.474542	1	1	DMRTA2	K19491
ENSG00000145451	-0.907046	-1.474542	1	1	GLRA3	K05195

ENSG00000155926	-0.907046	-1.474542	1	1	SLA	_
ENSG00000156140	-0.907046	-1.474542	1	1	ADAMTS3	K08619
ENSG00000160870	-0.907046	-1.474542	1	1	CYP3A7	K17691
ENSG00000164669	-0.907046	-1.474542	1	1	INTS4L1	K13141
ENSG00000167765	-0.907046	-1.474542	1	1	AC018755.1	K04613
ENSG00000169605	-0.907046	-1.474542	1	1	GKN1	_
ENSG00000171016	-0.907046	-1.474542	1	1	PYGO1	K11447
ENSG00000171827	-0.907046	-1.474542	1	1	ZNF570	K09228
ENSG00000173401	-0.907046	-1.474542	1	1	GLIPR1L1	_
ENSG00000176732	-0.907046	-1.474542	1	1	PFN4	K05759
ENSG00000180481	-0.907046	-1.474542	1	1	GLIPR1L2	_
ENSG00000184261	-0.907046	-1.474542	1	1	KCNK12	K04921
ENSG00000185304	-0.907046	-1.474542	1	1	RGPD2	K12172
ENSG00000186451	-0.907046	-1.474542	1	1	SPATA12	_
ENSG00000187753	-0.907046	-1.474542	1	1	C9orf153	_
ENSG00000188921	-0.907046	-1.474542	1	1	PTPLAD2	K10703
ENSG00000197584	-0.907046	-1.474542	1	1	KCNMB2	K04938
ENSG00000198286	-0.907046	-1.474542	1	1	CARD11	K07367
ENSG00000198366	-0.907046	-1.474542	1	1	HIST1H3A	K11253
ENSG00000198478	-0.907046	-1.474542	1	1	SH3BGRL2	_
ENSG00000200550	-0.907046	-1.474542	1	1	RNU6-137P	_
ENSG00000202220	-0.907046	-1.474542	1	1	Y_RNA	_
ENSG00000202290	-0.907046	-1.474542	1	1	RNA5SP37	_
ENSG00000207062	-0.907046	-1.474542	1	1	SNORA15	_
ENSG00000211445	-0.907046	-1.474542	1	1	GPX3	K00432
ENSG00000212396	-0.907046	-1.474542	1	1	RNA5SP323	_
ENSG00000213018	-0.907046	-1.474542	1	1	AL590762.11	K14396
ENSG00000213144	-0.907046	-1.474542	1	1	RP11-64B16.2	_
ENSG00000214700	-0.907046	-1.474542	1	1	C12orf71	_
ENSG00000215086	-0.907046	-1.474542	1	1	NPM1P24	K11276
ENSG00000216901	-0.907046	-1.474542	1	1	AL022393.7	_
ENSG00000217236	-0.907046	-1.474542	1	1	SP9	K09199
ENSG00000223298	-0.907046	-1.474542	1	1	RNY3P8	_
ENSG00000223697	-0.907046	-1.474542	1	1	AF230666.2	_
ENSG00000223711	-0.907046	-1.474542	1	1	AC091633.3	_
ENSG00000224209	-0.907046	-1.474542	1	1	LINC00466	_
ENSG00000224261	-0.907046	-1.474542	1	1	RP11-179G5.1	K02998
ENSG00000226243	-0.907046	-1.474542	1	1	RPL37AP1	K02921
ENSG00000226478	-0.907046	-1.474542	1	1	UPF3AP1	K14328
ENSG00000227477	-0.907046	-1.474542	1	1	STK4-AS1	_
ENSG00000227632	-0.907046	-1.474542	1	1	AC018804.6	K16307
ENSG00000227811	-0.907046	-1.474542	1	1	FAM212B-AS1	_
ENSG00000227868	-0.907046	-1.474542	1	1	C1orf234	_
ENSG00000227941	-0.907046	-1.474542	1	1	UQCRBP2	K00417
ENSG00000228407	-0.907046	-1.474542	1	1	RP4-800M22.1	K00121
ENSG00000228510	-0.907046	-1.474542	1	1	RP11-339B21.8	K12617
ENSG00000228737	-0.907046	-1.474542	1	1	AC008781.7	_
ENSG00000229781	-0.907046	-1.474542	1	1	AC013444.1	K15216
ENSG00000229896	-0.907046	-1.474542	1	1	RP11-456H18.2	_
ENSG00000230499	-0.907046	-1.474542	1	1	AC108463.1	_
ENSG00000230606	-0.907046	-1.474542	1	1	AC159540.1	_
ENSG00000230870	-0.907046	-1.474542	1	1	FBXW11P1	K03362
ENSG00000231574	-0.907046	-1.474542	1	1	RP11-91K9.1	_
ENSG00000231595	-0.907046	-1.474542	1	1	AC005224.2	_
ENSG00000231768	-0.907046	-1.474542	1	1	RP5-855F14.1	_
ENSG00000231952	-0.907046	-1.474542	1	1	DPY19L1P2	_
ENSG00000232398	-0.907046	-1.474542	1	1	TMPRSS11CP	K14359
ENSG00000232527	-0.907046	-1.474542	1	1	RP11-14N7.2	_

ENSG00000233695	-0.907046	-1.474542	1	1	GAS6-AS1	_
ENSG00000234705	-0.907046	-1.474542	1	1	HMGA1P4	_
ENSG00000235159	-0.907046	-1.474542	1	1	RP6-109B7.4	_
ENSG00000235295	-0.907046	-1.474542	1	1	XXbac-B476C20.17	_
ENSG00000236056	-0.907046	-1.474542	1	1	GAPDHP14	K00134
ENSG00000237548	-0.907046	-1.474542	1	1	TTLL11-IT1	_
ENSG00000238317	-0.907046	-1.474542	1	1	SNORD11	_
ENSG00000238369	-0.907046	-1.474542	1	1	snoU13	_
ENSG00000240364	-0.907046	-1.474542	1	1	RPL31P59	K02910
ENSG00000240395	-0.907046	-1.474542	1	1	RPL5P23	K02932
ENSG00000242154	-0.907046	-1.474542	1	1	RP4-778K6.3	_
ENSG00000243284	-0.907046	-1.474542	1	1	VSIG8	_
ENSG00000243697	-0.907046	-1.474542	1	1	RP11-463O9.1	K02936
ENSG00000244218	-0.907046	-1.474542	1	1	RN7SL81P	_
ENSG00000244257	-0.907046	-1.474542	1	1	PKD1P1	K04985
ENSG00000245812	-0.907046	-1.474542	1	1	RP11-175K6.1	_
ENSG00000248473	-0.907046	-1.474542	1	1	CTC-338M12.2	_
ENSG00000248693	-0.907046	-1.474542	1	1	CTD-2023M8.1	_
ENSG00000249216	-0.907046	-1.474542	1	1	RP11-227F19.5	_
ENSG00000249319	-0.907046	-1.474542	1	1	AC068533.7	K01755
ENSG00000249937	-0.907046	-1.474542	1	1	RP11-454P21.1	_
ENSG00000250853	-0.907046	-1.474542	1	1	RNF138P1	K10668
ENSG00000251429	-0.907046	-1.474542	1	1	RP11-597D13.7	_
ENSG00000253227	-0.907046	-1.474542	1	1	RP11-383J24.1	_
ENSG00000253986	-0.907046	-1.474542	1	1	CTC-756D1.3	_
ENSG00000254373	-0.907046	-1.474542	1	1	RP11-34P1.2	K00417
ENSG00000254433	-0.907046	-1.474542	1	1	RP11-677I18.3	_
ENSG00000255458	-0.907046	-1.474542	1	1	RP11-539G18.2	_
ENSG00000255621	-0.907046	-1.474542	1	1	RP11-377D9.3	_
ENSG00000255893	-0.907046	-1.474542	1	1	RP11-685N10.1	_
ENSG00000256720	-0.907046	-1.474542	1	1	RP11-436I9.6	K14443
ENSG00000257135	-0.907046	-1.474542	1	1	RP11-320M2.1	_
ENSG00000257175	-0.907046	-1.474542	1	1	RP11-536C10.12	K11841
ENSG00000257524	-0.907046	-1.474542	1	1	RP11-203J24.9	_
ENSG00000258512	-0.907046	-1.474542	1	1	RP11-796G6.2	_
ENSG00000258646	-0.907046	-1.474542	1	1	RP11-950C14.3	_
ENSG00000258695	-0.907046	-1.474542	1	1	RP3-414A15.2	_
ENSG00000258857	-0.907046	-1.474542	1	1	RP11-247L20.3	_
ENSG00000259488	-0.907046	-1.474542	1	1	RP11-154J22.1	_
ENSG00000259727	-0.907046	-1.474542	1	1	RP11-1069G10.2	_
ENSG00000259762	-0.907046	-1.474542	1	1	RP11-158M2.4	_
ENSG00000260360	-0.907046	-1.474542	1	1	RP11-533E19.5	_
ENSG00000260777	-0.907046	-1.474542	1	1	CTD-2008P7.1	_
ENSG00000261123	-0.907046	-1.474542	1	1	RP11-304L19.3	_
ENSG00000261997	-0.907046	-1.474542	1	1	RP11-212I21.4	_
ENSG00000262152	-0.907046	-1.474542	1	1	LINC00514	_
ENSG00000262410	-0.907046	-1.474542	1	1	RP11-388C12.8	_
ENSG00000262678	-0.907046	-1.474542	1	1	RP5-1050D4.4	_
ENSG00000263606	-0.907046	-1.474542	1	1	RP11-737O24.3	K16729
ENSG00000263727	-0.907046	-1.474542	1	1	RP11-806L2.5	_
ENSG00000264655	-0.907046	-1.474542	1	1	CTD-2267D19.4	K03767
ENSG00000265010	-0.907046	-1.474542	1	1	RP11-661C3.2	_
ENSG00000266304	-0.907046	-1.474542	1	1	RP11-484N16.1	_
ENSG00000267016	-0.907046	-1.474542	1	1	RP11-75C10.9	_
ENSG00000267163	-0.907046	-1.474542	1	1	AC084219.3	_
ENSG00000267769	-0.907046	-1.474542	1	1	CTB-50L17.9	_
ENSG00000267778	-0.907046	-1.474542	1	1	AC004221.2	_
ENSG00000267798	-0.907046	-1.474542	1	1	RP11-333J10.3	_

ENSG00000268366	-0.907046	-1.474542	1	1	CTC-492K19.4	_
ENSG00000269814	-0.907046	-1.474542	1	1	CTC-273B12.10	_
ENSG00000270009	-0.907046	-1.474542	1	1	RP1-127C7.6	_
ENSG00000271155	-0.907046	-1.474542	1	1	RP11-435O5.5	_
ENSG00000272161	-0.907046	-1.474542	1	1	RP4-713A8.1	_
ENSG00000272386	-0.907046	-1.474542	1	1	RP11-666A8.12	_
ENSG00000272703	-0.907046	-1.474542	1	1	RP11-78A19.4	_
ENSG00000272717	-0.907046	-1.474542	1	1	RP11-342I1.2	_
ENSG00000272869	-0.907046	-1.474542	1	1	CTC-487M23.8	K03105
ENSG00000272973	-0.907046	-1.474542	1	1	KB-1125A3.11	_
ENSG00000273001	-0.907046	-1.474542	1	1	RP11-118K6.3	_
ENSG00000273253	-0.907046	-1.474542	1	1	RP3-402G11.26	_
ENSG00000000938	0.603256	-1.157594	1	1	FGR	K08891
ENSG00000064270	0.603256	-1.157594	1	1	ATP2C2	K01537
ENSG00000065923	0.603256	-1.157594	1	1	SLC9A7	K12041
ENSG00000068438	0.603256	-1.157594	1	1	FTSJ1	K14864
ENSG00000087237	0.603256	-1.157594	1	1	CETP	K16835
ENSG00000088882	0.603256	-1.157594	1	1	CPXM1	K08638
ENSG00000103811	0.603256	-1.157594	1	1	CTSH	K01366
ENSG00000107147	0.603256	-1.157594	1	1	KCNT1	K04946
ENSG00000114757	0.603256	-1.157594	1	1	PEX5L	K13342
ENSG00000123610	0.603256	-1.157594	1	1	TNFAIP6	K19018
ENSG00000126353	0.603256	-1.157594	1	1	CCR7	K04182
ENSG00000137474	0.603256	-1.157594	1	1	MYO7A	_
ENSG00000139597	0.603256	-1.157594	1	1	N4BP2L1	_
ENSG00000139914	0.603256	-1.157594	1	1	FITM1	_
ENSG00000142233	0.603256	-1.157594	1	1	NTN5	_
ENSG00000154438	0.603256	-1.157594	1	1	ASZ1	K18410
ENSG00000168126	0.603256	-1.157594	1	1	OR2W6P	K04257
ENSG00000168646	0.603256	-1.157594	1	1	AXIN2	K04385
ENSG00000169894	0.603256	-1.157594	1	1	MUC3A	_
ENSG00000170468	0.603256	-1.157594	1	1	AC005280.1	K16914
ENSG00000170509	0.603256	-1.157594	1	1	HSD17B13	K17985
ENSG00000181652	0.603256	-1.157594	1	1	ATG9B	K17907
ENSG00000182511	0.603256	-1.157594	1	1	FES	K07527
ENSG00000183935	0.603256	-1.157594	1	1	HTR7P1	K04163
ENSG00000186314	0.603256	-1.157594	1	1	PRELID2	K01368
ENSG00000205238	0.603256	-1.157594	1	1	SPDYE2	K08694
ENSG00000213033	0.603256	-1.157594	1	1	AURKAPS1	K11481
ENSG00000218027	0.603256	-1.157594	1	1	RP11-157J24.1	K09428
ENSG00000223804	0.603256	-1.157594	1	1	RP6-206I17.1	_
ENSG00000223886	0.603256	-1.157594	1	1	RP11-251G23.2	_
ENSG00000224067	0.603256	-1.157594	1	1	RP11-430K21.2	_
ENSG00000225472	0.603256	-1.157594	1	1	RP11-120J1.1	_
ENSG00000226849	0.603256	-1.157594	1	1	RP4-635E18.7	_
ENSG00000230306	0.603256	-1.157594	1	1	BANF1P2	_
ENSG00000231519	0.603256	-1.157594	1	1	AC007285.7	_
ENSG00000232027	0.603256	-1.157594	1	1	RP11-275F13.3	K03038
ENSG00000232702	0.603256	-1.157594	1	1	RP3-437C15.1	_
ENSG00000232742	0.603256	-1.157594	1	1	RHOQP2	K07194
ENSG00000233396	0.603256	-1.157594	1	1	RP11-458D21.1	_
ENSG00000233818	0.603256	-1.157594	1	1	AP000695.4	_
ENSG00000234329	0.603256	-1.157594	1	1	RP11-767N6.2	K02936
ENSG00000234902	0.603256	-1.157594	1	1	AC007879.2	_
ENSG00000235023	0.603256	-1.157594	1	1	AP001626.2	_
ENSG00000235527	0.603256	-1.157594	1	1	RP5-1073O3.7	_
ENSG00000236423	0.603256	-1.157594	1	1	LINC01134	_
ENSG00000237379	0.603256	-1.157594	1	1	CBX1P3	K11585

ENSG00000239557	0.603256	-1.157594	1	1	RP11-168J18.6	K11584
ENSG00000244476	0.603256	-1.157594	1	1	ERVFRD-1	_
ENSG00000244723	0.603256	-1.157594	1	1	ASLP1	K01755
ENSG00000246308	0.603256	-1.157594	1	1	RP11-685M7.3	_
ENSG00000246777	0.603256	-1.157594	1	1	RP11-61A14.4	_
ENSG00000248124	0.603256	-1.157594	1	1	RRN3P1	K15216
ENSG00000248971	0.603256	-1.157594	1	1	KRT8P46	K07605
ENSG00000249375	0.603256	-1.157594	1	1	CASC11	_
ENSG00000250012	0.603256	-1.157594	1	1	RP11-124N2.1	_
ENSG00000250247	0.603256	-1.157594	1	1	SEPHS2P1	K01008
ENSG00000252075	0.603256	-1.157594	1	1	RNU7-109P	_
ENSG00000254806	0.603256	-1.157594	1	1	SYS1-DBNDD2	_
ENSG00000255236	0.603256	-1.157594	1	1	CTD-2655K5.1	_
ENSG00000255337	0.603256	-1.157594	1	1	RP11-315O6.1	_
ENSG00000255641	0.603256	-1.157594	1	1	NKG2-E	K06541
ENSG00000255966	0.603256	-1.157594	1	1	RP5-940J5.3	_
ENSG00000257531	0.603256	-1.157594	1	1	RP3-405J10.2	K02976
ENSG00000260007	0.603256	-1.157594	1	1	RP11-315D16.2	K12359
ENSG00000260572	0.603256	-1.157594	1	1	RP11-16N11.2	_
ENSG00000260743	0.603256	-1.157594	1	1	RP11-255C15.3	_
ENSG00000260910	0.603256	-1.157594	1	1	LINC00565	_
ENSG00000260992	0.603256	-1.157594	1	1	DOCK9-AS2	_
ENSG00000261335	0.603256	-1.157594	1	1	RP11-318A15.2	_
ENSG00000262877	0.603256	-1.157594	1	1	RP11-1055B8.4	_
ENSG00000263766	0.603256	-1.157594	1	1	RP11-580I16.2	_
ENSG00000267759	0.603256	-1.157594	1	1	CTB-25B13.13	_
ENSG00000268059	0.603256	-1.157594	1	1	AL441883.1	_
ENSG00000271983	0.603256	-1.157594	1	1	RP11-28H5.2	_
ENSG00000272156	0.603256	-1.157594	1	1	RP11-477N3.1	_
ENSG00000100985	-0.528106	-1.058472	1	1	MMP9	K01403
ENSG00000101298	-0.528106	-1.058472	1	1	SNPH	_
ENSG00000108387	-0.528106	-1.058472	1	1	4-Sep	K16943
ENSG00000111913	-0.528106	-1.058472	1	1	FAM65B	K01940
ENSG00000116833	-0.528106	-1.058472	1	1	NR5A2	K08027
ENSG00000130643	-0.528106	-1.058472	1	1	CALY	K15493
ENSG00000133216	-0.528106	-1.058472	1	1	EPHB2	K05111
ENSG00000142513	-0.528106	-1.058472	1	1	ACPT	K19284
ENSG00000150316	-0.528106	-1.058472	1	1	CWC15	K12863
ENSG00000157388	-0.528106	-1.058472	1	1	CACNA1D	K04851
ENSG00000157873	-0.528106	-1.058472	1	1	TNFRSF14	K05152
ENSG00000163449	-0.528106	-1.058472	1	1	TMEM169	_
ENSG00000166220	-0.528106	-1.058472	1	1	TBATA	_
ENSG00000166963	-0.528106	-1.058472	1	1	MAP1A	K10429
ENSG00000179593	-0.528106	-1.058472	1	1	ALOX15B	K08022
ENSG00000182687	-0.528106	-1.058472	1	1	GALR2	K04231
ENSG00000183134	-0.528106	-1.058472	1	1	PTGDR2	K06715
ENSG00000183977	-0.528106	-1.058472	1	1	PP2D1	_
ENSG00000197646	-0.528106	-1.058472	1	1	PDCD1LG2	K06708
ENSG00000200714	-0.528106	-1.058472	1	1	Y_RNA	_
ENSG00000205277	-0.528106	-1.058472	1	1	MUC12	_
ENSG00000205683	-0.528106	-1.058472	1	1	DPF3	_
ENSG00000206920	-0.528106	-1.058472	1	1	RNY1P6	_
ENSG00000215270	-0.528106	-1.058472	1	1	LA16c-60H5.7	K11518
ENSG00000217733	-0.528106	-1.058472	1	1	CCT7P1	K09499
ENSG00000220685	-0.528106	-1.058472	1	1	RP11-530A18.1	K17653
ENSG00000220875	-0.528106	-1.058472	1	1	HIST1H3PS1	K11253
ENSG00000226440	-0.528106	-1.058472	1	1	RP11-506B6.6	_
ENSG00000226754	-0.528106	-1.058472	1	1	RP5-1024G6.5	_

ENSG00000227070	-0.528106	-1.058472	1	1	RP11-191G24.1	_
ENSG00000227666	-0.528106	-1.058472	1	1	CYCSP24	K08738
ENSG00000228044	-0.528106	-1.058472	1	1	RP4-781K5.4	_
ENSG00000228192	-0.528106	-1.058472	1	1	RP11-342M1.3	_
ENSG00000231784	-0.528106	-1.058472	1	1	DBIL5P	_
ENSG00000234369	-0.528106	-1.058472	1	1	TATDN1P1	K03424
ENSG00000235831	-0.528106	-1.058472	1	1	BHLHE40-AS1	_
ENSG00000236123	-0.528106	-1.058472	1	1	CEACAMP11	K06500
ENSG00000238336	-0.528106	-1.058472	1	1	snoU13	_
ENSG00000244256	-0.528106	-1.058472	1	1	RN7SL130P	_
ENSG00000248607	-0.528106	-1.058472	1	1	RP11-158I23.1	_
ENSG00000251143	-0.528106	-1.058472	1	1	RP11-849H4.4	_
ENSG00000251161	-0.528106	-1.058472	1	1	RP11-540O11.1	_
ENSG00000253660	-0.528106	-1.058472	1	1	CTC-353G13.1	_
ENSG00000253819	-0.528106	-1.058472	1	1	LINC01151	_
ENSG00000253966	-0.528106	-1.058472	1	1	CTC-455F18.3	K10391
ENSG00000257222	-0.528106	-1.058472	1	1	RP11-554E23.4	_
ENSG00000257613	-0.528106	-1.058472	1	1	RP11-320P7.1	_
ENSG00000258515	-0.528106	-1.058472	1	1	RP11-203M5.7	_
ENSG00000258811	-0.528106	-1.058472	1	1	CTD-3051D23.1	_
ENSG00000259238	-0.528106	-1.058472	1	1	RP11-361D15.2	_
ENSG00000261216	-0.528106	-1.058472	1	1	RP11-166B2.5	_
ENSG00000261490	-0.528106	-1.058472	1	1	RP11-448G15.3	_
ENSG00000265112	-0.528106	-1.058472	1	1	MIR3153	_
ENSG00000267219	-0.528106	-1.058472	1	1	AC010504.2	_
ENSG00000267595	-0.528106	-1.058472	1	1	RP11-242D8.2	K10605
ENSG00000270020	-0.528106	-1.058472	1	1	RP11-463O9.9	_
ENSG00000271032	-0.528106	-1.058472	1	1	CTD-2527I21.14	_
ENSG00000271321	-0.528106	-1.058472	1	1	CTAGE6	_
ENSG00000272148	-0.528106	-1.058472	1	1	RP11-195B17.1	_
ENSG00000272428	-0.528106	-1.058472	1	1	RP11-704J17.5	_
ENSG00000272432	-0.528106	-1.058472	1	1	RP3-465N24.6	_
ENSG00000272791	-0.528106	-1.058472	1	1	RP11-464F9.22	_
ENSG00000273081	-0.528106	-1.058472	1	1	RP4-813F11.4	_
ENSG00000013297	-0.51128	-1.637813	1	1	CLDN11	K06087
ENSG00000044524	-0.51128	-1.637813	1	1	EPHA3	K05104
ENSG00000064393	-0.51128	-1.637813	1	1	HIPK2	K08826
ENSG00000067177	-0.51128	-1.637813	1	1	PHKA1	K07190
ENSG00000069712	-0.51128	-1.637813	1	1	KIAA1107	K10480
ENSG00000080007	-0.51128	-1.637813	1	1	DDX43	K17043
ENSG00000101353	-0.51128	-1.637813	1	1	MROH8	K14443
ENSG00000103313	-0.51128	-1.637813	1	1	MEFV	K12803
ENSG00000105255	-0.51128	-1.637813	1	1	FSD1	_
ENSG00000107165	-0.51128	-1.637813	1	1	TYRP1	K00506
ENSG00000111780	-0.51128	-1.637813	1	1	AL021546.6	K02435
ENSG00000117245	-0.51128	-1.637813	1	1	KIF17	K10394
ENSG00000122733	-0.51128	-1.637813	1	1	KIAA1045	_
ENSG00000124772	-0.51128	-1.637813	1	1	CPNE5	_
ENSG00000128652	-0.51128	-1.637813	1	1	HOXD3	K09303
ENSG00000129744	-0.51128	-1.637813	1	1	ART1	K06716
ENSG00000131152	-0.51128	-1.637813	1	1	RP11-178L8.4	K10308
ENSG00000139200	-0.51128	-1.637813	1	1	PIANP	_
ENSG00000139540	-0.51128	-1.637813	1	1	SLC39A5	_
ENSG00000140623	-0.51128	-1.637813	1	1	12-Sep	K16938
ENSG00000142319	-0.51128	-1.637813	1	1	SLC6A3	K05036
ENSG00000142611	-0.51128	-1.637813	1	1	PRDM16	K04462
ENSG00000148848	-0.51128	-1.637813	1	1	ADAM12	K06835
ENSG00000151655	-0.51128	-1.637813	1	1	ITIH2	K19015

ENSG00000153237	-0.51128	-1.637813	1	1	CCDC148	_
ENSG00000155761	-0.51128	-1.637813	1	1	SPAG17	_
ENSG00000157168	-0.51128	-1.637813	1	1	NRG1	K05455
ENSG00000159374	-0.51128	-1.637813	1	1	M1AP	_
ENSG00000161149	-0.51128	-1.637813	1	1	TUBA3FP	K07374
ENSG00000162676	-0.51128	-1.637813	1	1	GFI1	K09223
ENSG00000163239	-0.51128	-1.637813	1	1	TDRD10	_
ENSG00000163749	-0.51128	-1.637813	1	1	CCDC158	_
ENSG00000163884	-0.51128	-1.637813	1	1	KLF15	K09210
ENSG00000163931	-0.51128	-1.637813	1	1	TKT	K00615
ENSG00000166501	-0.51128	-1.637813	1	1	PRKCB	K02677
ENSG00000168350	-0.51128	-1.637813	1	1	DEGS2	K04712
ENSG00000168589	-0.51128	-1.637813	1	1	DYNLRB2	K10419
ENSG00000169903	-0.51128	-1.637813	1	1	TM4SF4	_
ENSG00000170667	-0.51128	-1.637813	1	1	RASA4B	K17630
ENSG00000171401	-0.51128	-1.637813	1	1	KRT13	K07604
ENSG00000171649	-0.51128	-1.637813	1	1	ZIK1	K09228
ENSG00000172936	-0.51128	-1.637813	1	1	MYD88	K04729
ENSG00000173088	-0.51128	-1.637813	1	1	C10orf131	K19352
ENSG00000175077	-0.51128	-1.637813	1	1	RTP1	_
ENSG00000175463	-0.51128	-1.637813	1	1	TBC1D10C	_
ENSG00000177191	-0.51128	-1.637813	1	1	B3GNT8	K09665
ENSG00000178623	-0.51128	-1.637813	1	1	GPR35	K04276
ENSG00000179580	-0.51128	-1.637813	1	1	RNF151	K11981
ENSG00000180787	-0.51128	-1.637813	1	1	ZFP3	K09228
ENSG00000182533	-0.51128	-1.637813	1	1	CAV3	K12959
ENSG00000183785	-0.51128	-1.637813	1	1	TUBA8	K07374
ENSG00000184324	-0.51128	-1.637813	1	1	CSAG2	_
ENSG00000184599	-0.51128	-1.637813	1	1	FAM19A3	_
ENSG00000184697	-0.51128	-1.637813	1	1	CLDN6	K06087
ENSG00000185615	-0.51128	-1.637813	1	1	PDIA2	K09581
ENSG00000185837	-0.51128	-1.637813	1	1	CECR5-AS1	_
ENSG00000188763	-0.51128	-1.637813	1	1	FZD9	K02842
ENSG00000188833	-0.51128	-1.637813	1	1	ENTPD8	K01510
ENSG00000188859	-0.51128	-1.637813	1	1	FAM78B	_
ENSG00000189337	-0.51128	-1.637813	1	1	KAZN	_
ENSG00000196420	-0.51128	-1.637813	1	1	S100A5	_
ENSG00000196860	-0.51128	-1.637813	1	1	TOMM20L	K17770
ENSG00000198829	-0.51128	-1.637813	1	1	SUCNR1	K10042
ENSG00000200169	-0.51128	-1.637813	1	1	RNU5D-1	_
ENSG00000200769	-0.51128	-1.637813	1	1	Y_RNA	_
ENSG00000201388	-0.51128	-1.637813	1	1	SNORA68	_
ENSG00000203327	-0.51128	-1.637813	1	1	AC012358.7	_
ENSG00000203581	-0.51128	-1.637813	1	1	OR1F2P	K04257
ENSG00000204310	-0.51128	-1.637813	1	1	AGPAT1	K13509
ENSG00000205693	-0.51128	-1.637813	1	1	MANSC4	_
ENSG00000206658	-0.51128	-1.637813	1	1	RNU6-1039P	_
ENSG00000212452	-0.51128	-1.637813	1	1	SNORD69	_
ENSG00000212541	-0.51128	-1.637813	1	1	RNU6-510P	_
ENSG00000212993	-0.51128	-1.637813	1	1	POU5F1B	K09367
ENSG00000213994	-0.51128	-1.637813	1	1	RP11-414H17.5	_
ENSG00000214108	-0.51128	-1.637813	1	1	TPT1P5	_
ENSG00000214146	-0.51128	-1.637813	1	1	RP11-699L21.1	_
ENSG00000214237	-0.51128	-1.637813	1	1	FAM188B2	K08742
ENSG00000215018	-0.51128	-1.637813	1	1	COL28A1	_
ENSG00000215548	-0.51128	-1.637813	1	1	RP11-764K9.4	K13122
ENSG00000215795	-0.51128	-1.637813	1	1	RP11-488L18.3	K13342
ENSG00000218996	-0.51128	-1.637813	1	1	RP1-99E18.2	K07945

ENSG00000219507	-0.51128	-1.637813	1	1	FTH1P8	K00522
ENSG00000220793	-0.51128	-1.637813	1	1	RPL21P119	K02889
ENSG00000221743	-0.51128	-1.637813	1	1	Z95152.1	_
ENSG00000222046	-0.51128	-1.637813	1	1	DCDC2B	_
ENSG00000223254	-0.51128	-1.637813	1	1	Y_RNA	_
ENSG00000223313	-0.51128	-1.637813	1	1	RNU6-516P	_
ENSG00000223461	-0.51128	-1.637813	1	1	AC004471.9	_
ENSG00000223519	-0.51128	-1.637813	1	1	KIF28P	_
ENSG00000223901	-0.51128	-1.637813	1	1	AP001469.5	_
ENSG00000224043	-0.51128	-1.637813	1	1	CCNT2-AS1	_
ENSG00000224093	-0.51128	-1.637813	1	1	RP5-1033H22.2	_
ENSG00000224468	-0.51128	-1.637813	1	1	RP11-181K3.4	_
ENSG00000224490	-0.51128	-1.637813	1	1	TTC21B-AS1	_
ENSG00000224790	-0.51128	-1.637813	1	1	AP000704.5	_
ENSG00000224855	-0.51128	-1.637813	1	1	OPA1-AS1	_
ENSG00000225206	-0.51128	-1.637813	1	1	MIR137HG	_
ENSG00000225385	-0.51128	-1.637813	1	1	RP11-350E12.4	_
ENSG00000225447	-0.51128	-1.637813	1	1	RPS15AP10	K02957
ENSG00000225826	-0.51128	-1.637813	1	1	LINC00626	_
ENSG00000226055	-0.51128	-1.637813	1	1	PAICSP1	K01587
ENSG00000226203	-0.51128	-1.637813	1	1	RP4-760C5.5	_
ENSG00000226472	-0.51128	-1.637813	1	1	RP11-551L14.4	_
ENSG00000226544	-0.51128	-1.637813	1	1	RPL7P22	_
ENSG00000226548	-0.51128	-1.637813	1	1	AC016722.3	_
ENSG00000226690	-0.51128	-1.637813	1	1	AC005281.1	_
ENSG00000227071	-0.51128	-1.637813	1	1	RP11-4E23.2	_
ENSG00000227210	-0.51128	-1.637813	1	1	AC079145.4	_
ENSG00000228502	-0.51128	-1.637813	1	1	EEF1A1P11	K03231
ENSG00000228554	-0.51128	-1.637813	1	1	AC004837.5	_
ENSG00000228701	-0.51128	-1.637813	1	1	TNKS2-AS1	_
ENSG00000228824	-0.51128	-1.637813	1	1	MIR4500HG	_
ENSG00000229401	-0.51128	-1.637813	1	1	RP1-290I10.7	_
ENSG00000229989	-0.51128	-1.637813	1	1	MIR181A1HG	_
ENSG00000230069	-0.51128	-1.637813	1	1	LRRC37A15P	K01192
ENSG00000230280	-0.51128	-1.637813	1	1	HNRNPA1P59	K12741
ENSG00000230637	-0.51128	-1.637813	1	1	CTA-246H3.8	_
ENSG00000230724	-0.51128	-1.637813	1	1	LINC01001	_
ENSG00000231530	-0.51128	-1.637813	1	1	RP11-187A9.3	_
ENSG00000231587	-0.51128	-1.637813	1	1	SNORD62B	_
ENSG00000231856	-0.51128	-1.637813	1	1	RP11-327P2.5	_
ENSG00000232727	-0.51128	-1.637813	1	1	YWHAEP1	K06630
ENSG00000233170	-0.51128	-1.637813	1	1	RP11-863K10.4	_
ENSG00000233626	-0.51128	-1.637813	1	1	RP11-565J7.1	K09579
ENSG00000234183	-0.51128	-1.637813	1	1	AC004854.4	_
ENSG00000234754	-0.51128	-1.637813	1	1	RP11-421L10.1	_
ENSG00000235121	-0.51128	-1.637813	1	1	RP11-90L20.2	_
ENSG00000236269	-0.51128	-1.637813	1	1	ENO1-IT1	_
ENSG00000236409	-0.51128	-1.637813	1	1	NRADDP	_
ENSG00000236641	-0.51128	-1.637813	1	1	CTA-221G9.7	_
ENSG00000236937	-0.51128	-1.637813	1	1	PTGES3P4	K15730
ENSG00000236991	-0.51128	-1.637813	1	1	EDRF1-AS1	_
ENSG00000237263	-0.51128	-1.637813	1	1	MAPK6PS3	K06855
ENSG00000237268	-0.51128	-1.637813	1	1	RP13-492C18.2	K17299
ENSG00000239280	-0.51128	-1.637813	1	1	RP11-271C24.3	K09699
ENSG00000239405	-0.51128	-1.637813	1	1	TMED10P2	_
ENSG00000241255	-0.51128	-1.637813	1	1	RP1-89D4.1	K02974
ENSG00000241458	-0.51128	-1.637813	1	1	RPL7P19	K02937
ENSG00000243404	-0.51128	-1.637813	1	1	RPL35AP32	K02917

ENSG00000243849	-0.51128	-1.637813	1	1	WDR52-AS1	_
ENSG00000244926	-0.51128	-1.637813	1	1	ALKBH3-AS1	_
ENSG00000246430	-0.51128	-1.637813	1	1	LINC00968	_
ENSG00000247345	-0.51128	-1.637813	1	1	RP11-266N13.2	_
ENSG00000248206	-0.51128	-1.637813	1	1	RP11-739P1.2	_
ENSG00000248898	-0.51128	-1.637813	1	1	CTD-2288O8.1	_
ENSG00000249055	-0.51128	-1.637813	1	1	TBCAP3	K17292
ENSG00000249379	-0.51128	-1.637813	1	1	RP1-27K12.4	_
ENSG00000249650	-0.51128	-1.637813	1	1	RP11-310P5.1	_
ENSG00000250033	-0.51128	-1.637813	1	1	SLC7A11-AS1	_
ENSG00000250362	-0.51128	-1.637813	1	1	AC008592.5	_
ENSG00000250492	-0.51128	-1.637813	1	1	INTS6P1	K13143
ENSG00000250656	-0.51128	-1.637813	1	1	ST3GAL1P1	K00780
ENSG00000250697	-0.51128	-1.637813	1	1	CTD-2066L21.3	_
ENSG00000251323	-0.51128	-1.637813	1	1	RP11-452H21.4	_
ENSG00000251963	-0.51128	-1.637813	1	1	AC090420.1	_
ENSG00000252147	-0.51128	-1.637813	1	1	Y_RNA	_
ENSG00000252202	-0.51128	-1.637813	1	1	Y_RNA	_
ENSG00000252245	-0.51128	-1.637813	1	1	RNU6-736P	_
ENSG00000253203	-0.51128	-1.637813	1	1	GUSBP3	K01195
ENSG00000253258	-0.51128	-1.637813	1	1	RP11-539E17.5	_
ENSG00000253476	-0.51128	-1.637813	1	1	RP11-395I14.2	_
ENSG00000254162	-0.51128	-1.637813	1	1	RP11-48B3.3	_
ENSG00000254286	-0.51128	-1.637813	1	1	RP11-89K10.1	_
ENSG00000254509	-0.51128	-1.637813	1	1	RP11-677M14.6	K06787
ENSG00000254731	-0.51128	-1.637813	1	1	CTD-2005H7.1	_
ENSG00000255008	-0.51128	-1.637813	1	1	AP000442.4	_
ENSG00000257097	-0.51128	-1.637813	1	1	RP11-450K4.1	_
ENSG00000257230	-0.51128	-1.637813	1	1	RP11-272B17.2	_
ENSG00000258092	-0.51128	-1.637813	1	1	RP4-816N1.7	_
ENSG00000258096	-0.51128	-1.637813	1	1	RP11-474P2.2	_
ENSG00000258334	-0.51128	-1.637813	1	1	RP11-161H23.9	_
ENSG00000258427	-0.51128	-1.637813	1	1	RBM8B	K12876
ENSG00000259129	-0.51128	-1.637813	1	1	LINC00648	_
ENSG00000259265	-0.51128	-1.637813	1	1	RP11-809H16.4	_
ENSG00000259295	-0.51128	-1.637813	1	1	CSPG4P12	K08115
ENSG00000259314	-0.51128	-1.637813	1	1	CTD-3065B20.3	_
ENSG00000259521	-0.51128	-1.637813	1	1	RP11-540O11.4	_
ENSG00000259630	-0.51128	-1.637813	1	1	CTD-2262B20.1	K08754
ENSG00000259736	-0.51128	-1.637813	1	1	RP11-387D10.2	_
ENSG00000260088	-0.51128	-1.637813	1	1	RP11-92G12.3	_
ENSG00000260272	-0.51128	-1.637813	1	1	RP11-20I23.1	K02155
ENSG00000260366	-0.51128	-1.637813	1	1	CTD-2639E6.4	_
ENSG00000260467	-0.51128	-1.637813	1	1	RP11-405F3.4	_
ENSG00000260911	-0.51128	-1.637813	1	1	RP11-196G11.2	_
ENSG00000260924	-0.51128	-1.637813	1	1	AC004463.6	_
ENSG00000260927	-0.51128	-1.637813	1	1	RP11-459F6.3	_
ENSG00000261173	-0.51128	-1.637813	1	1	RP11-169E6.1	_
ENSG00000261886	-0.51128	-1.637813	1	1	RP11-63A1.1	_
ENSG00000261949	-0.51128	-1.637813	1	1	GFY	_
ENSG00000262006	-0.51128	-1.637813	1	1	RP11-700H6.4	_
ENSG00000262202	-0.51128	-1.637813	1	1	SNORD3D	_
ENSG00000262979	-0.51128	-1.637813	1	1	CTD-2047H16.2	_
ENSG00000265656	-0.51128	-1.637813	1	1	RP11-13N13.6	_
ENSG00000266920	-0.51128	-1.637813	1	1	ACTBP9	K05692
ENSG00000267059	-0.51128	-1.637813	1	1	UQCR11	K11591
ENSG00000267715	-0.51128	-1.637813	1	1	CTB-25B13.6	_
ENSG00000267750	-0.51128	-1.637813	1	1	AC003102.3	_

ENSG00000267758	-0.51128	-1.637813	1	1	RP11-358B23.5	_
ENSG00000267857	-0.51128	-1.637813	1	1	PRED58	_
ENSG00000268211	-0.51128	-1.637813	1	1	AL359091.2	K17985
ENSG00000268518	-0.51128	-1.637813	1	1	CTD-2545M3.8	_
ENSG00000268632	-0.51128	-1.637813	1	1	AC073657.1	K18047
ENSG00000269054	-0.51128	-1.637813	1	1	CTD-2619J13.3	_
ENSG00000269337	-0.51128	-1.637813	1	1	AL591479.1	_
ENSG00000269656	-0.51128	-1.637813	1	1	CTD-2571L23.6	_
ENSG00000269984	-0.51128	-1.637813	1	1	RP11-362K14.5	_
ENSG00000269994	-0.51128	-1.637813	1	1	RP11-276H19.2	_
ENSG00000270030	-0.51128	-1.637813	1	1	RP11-326C3.13	_
ENSG00000270062	-0.51128	-1.637813	1	1	RP11-248J18.3	_
ENSG00000270108	-0.51128	-1.637813	1	1	RP11-73M18.6	_
ENSG00000270681	-0.51128	-1.637813	1	1	RP11-372K14.2	_
ENSG00000271092	-0.51128	-1.637813	1	1	TMEM56-RWDD3	_
ENSG00000271427	-0.51128	-1.637813	1	1	RP11-188D8.1	_
ENSG00000271664	-0.51128	-1.637813	1	1	RP4-800G7.3	K19539
ENSG00000271984	-0.51128	-1.637813	1	1	RP3-337O18.9	_
ENSG00000272008	-0.51128	-1.637813	1	1	RP11-393I2.4	_
ENSG00000272053	-0.51128	-1.637813	1	1	RP11-367G6.3	_
ENSG00000272056	-0.51128	-1.637813	1	1	RP11-503P10.1	_
ENSG00000272128	-0.51128	-1.637813	1	1	KB-1836B5.4	_
ENSG00000272130	-0.51128	-1.637813	1	1	RP11-360I2.1	_
ENSG00000272189	-0.51128	-1.637813	1	1	RP3-325F22.5	_
ENSG00000272275	-0.51128	-1.637813	1	1	RP11-791G15.2	_
ENSG00000272416	-0.51128	-1.637813	1	1	CTD-2081C10.7	_
ENSG00000272498	-0.51128	-1.637813	1	1	RP11-415F23.3	_
ENSG00000272567	-0.51128	-1.637813	1	1	RP11-73K9.3	_
ENSG00000272732	-0.51128	-1.637813	1	1	RP5-1159O4.2	_
ENSG00000272799	-0.51128	-1.637813	1	1	RP11-474N24.6	_
ENSG00000273058	-0.51128	-1.637813	1	1	RP11-385F5.5	_
ENSG00000273061	-0.51128	-1.637813	1	1	RP11-6J24.6	_
ENSG00000273080	-0.51128	-1.637813	1	1	RP11-301O19.1	_
ENSG00000273102	-0.51128	-1.637813	1	1	AP000569.9	_
ENSG00000273331	-0.51128	-1.637813	1	1	TM4SF19	_
ENSG00000273353	-0.51128	-1.637813	1	1	CTA-268H5.12	_
ENSG00000273369	-0.51128	-1.637813	1	1	RP11-700J17.1	_
ENSG00000037280	0.43309	-1.453784	1	1	FLT4	K05097
ENSG00000057468	0.43309	-1.453784	1	1	MSH4	K08740
ENSG00000064489	0.43309	-1.453784	1	1	MEF2BNB-MEF2B	K09261
ENSG00000069188	0.43309	-1.453784	1	1	SDK2	K16353
ENSG00000095303	0.43309	-1.453784	1	1	PTGS1	K00509
ENSG00000099282	0.43309	-1.453784	1	1	TSPAN15	K17297
ENSG00000099337	0.43309	-1.453784	1	1	KCNK6	K17985
ENSG00000101335	0.43309	-1.453784	1	1	MYL9	K12755
ENSG00000110076	0.43309	-1.453784	1	1	NRXN2	K07377
ENSG00000111728	0.43309	-1.453784	1	1	ST8SIA1	K03371
ENSG00000112706	0.43309	-1.453784	1	1	IMPG1	K19016
ENSG00000118849	0.43309	-1.453784	1	1	RARRES1	_
ENSG00000122735	0.43309	-1.453784	1	1	DNAI1	K10409
ENSG00000124091	0.43309	-1.453784	1	1	GCNT7	_
ENSG00000131080	0.43309	-1.453784	1	1	EDA2R	K05163
ENSG00000132207	0.43309	-1.453784	1	1	SLX1A	K15078
ENSG00000133106	0.43309	-1.453784	1	1	EPSTI1	_
ENSG00000134339	0.43309	-1.453784	1	1	SAA2	K17310
ENSG00000135299	0.43309	-1.453784	1	1	ANKRD6	_
ENSG00000136011	0.43309	-1.453784	1	1	STAB2	K19013
ENSG00000143184	0.43309	-1.453784	1	1	XCL1	K05507

ENSG00000143318	0.43309	-1.453784	1	1	CASQ1	K17985
ENSG00000146005	0.43309	-1.453784	1	1	PSD2	K12494
ENSG00000148795	0.43309	-1.453784	1	1	CYP17A1	K00512
ENSG00000152580	0.43309	-1.453784	1	1	IGSF10	_
ENSG00000152689	0.43309	-1.453784	1	1	RASGRP3	K12362
ENSG00000159899	0.43309	-1.453784	1	1	NPR2	K12324
ENSG00000162391	0.43309	-1.453784	1	1	FAM151A	_
ENSG00000165197	0.43309	-1.453784	1	1	FIGF	K05449
ENSG00000171388	0.43309	-1.453784	1	1	APLN	K05225
ENSG00000173258	0.43309	-1.453784	1	1	ZNF483	K09229
ENSG00000178814	0.43309	-1.453784	1	1	OPLAH	K01469
ENSG00000181264	0.43309	-1.453784	1	1	TMEM136	_
ENSG00000183066	0.43309	-1.453784	1	1	WBP2NL	_
ENSG00000184988	0.43309	-1.453784	1	1	TMEM106A	_
ENSG00000185559	0.43309	-1.453784	1	1	DLK1	_
ENSG00000188100	0.43309	-1.453784	1	1	FAM25A	_
ENSG00000188487	0.43309	-1.453784	1	1	INSC	_
ENSG00000197696	0.43309	-1.453784	1	1	NMB	K05223
ENSG00000198393	0.43309	-1.453784	1	1	ZNF26	K09228
ENSG00000202058	0.43309	-1.453784	1	1	RN7SKP80	_
ENSG00000203276	0.43309	-1.453784	1	1	AL353898.2	K11447
ENSG00000203684	0.43309	-1.453784	1	1	IBA57-AS1	_
ENSG00000204173	0.43309	-1.453784	1	1	LRRC37A5P	_
ENSG00000206172	0.43309	-1.453784	1	1	HBA1	K13822
ENSG00000206630	0.43309	-1.453784	1	1	SNORD60	_
ENSG00000212371	0.43309	-1.453784	1	1	SNORA46	_
ENSG00000213130	0.43309	-1.453784	1	1	EEF1DP5	K15410
ENSG00000213132	0.43309	-1.453784	1	1	AC022498.1	_
ENSG00000213135	0.43309	-1.453784	1	1	AC015815.6	K09228
ENSG00000213514	0.43309	-1.453784	1	1	RP11-428P16.2	_
ENSG00000214973	0.43309	-1.453784	1	1	CHCHD3P3	K17563
ENSG00000214999	0.43309	-1.453784	1	1	AC129492.6	K08021
ENSG00000215472	0.43309	-1.453784	1	1	RPL17-C18orf32	K02880
ENSG00000215863	0.43309	-1.453784	1	1	LINC01138	_
ENSG00000217325	0.43309	-1.453784	1	1	PRELID1P1	_
ENSG00000219395	0.43309	-1.453784	1	1	HSPA8P15	_
ENSG00000220920	0.43309	-1.453784	1	1	RP3-525L6.2	_
ENSG00000222043	0.43309	-1.453784	1	1	AC079305.10	_
ENSG00000222071	0.43309	-1.453784	1	1	MIR1915	_
ENSG00000222533	0.43309	-1.453784	1	1	RNU6-705P	_
ENSG00000223714	0.43309	-1.453784	1	1	RP5-1172N10.2	_
ENSG00000224452	0.43309	-1.453784	1	1	RSL24D1P6	K02896
ENSG00000224905	0.43309	-1.453784	1	1	AP001347.6	_
ENSG00000224969	0.43309	-1.453784	1	1	RP11-54O7.11	_
ENSG00000225113	0.43309	-1.453784	1	1	RP5-1160K1.3	_
ENSG00000225400	0.43309	-1.453784	1	1	RP11-1114A5.5	K07915
ENSG00000225531	0.43309	-1.453784	1	1	RP11-196I18.3	K08964
ENSG00000226891	0.43309	-1.453784	1	1	RP11-182I10.3	_
ENSG00000227959	0.43309	-1.453784	1	1	RP11-276H7.2	_
ENSG00000228444	0.43309	-1.453784	1	1	RP11-173B14.4	_
ENSG00000229207	0.43309	-1.453784	1	1	SERPINH1P1	K09501
ENSG00000229222	0.43309	-1.453784	1	1	KRT18P4	K07604
ENSG00000229388	0.43309	-1.453784	1	1	RP11-442N24__B.1	_
ENSG00000229901	0.43309	-1.453784	1	1	RP11-399E6.4	_
ENSG00000230528	0.43309	-1.453784	1	1	NOS2P3	K13241
ENSG00000231119	0.43309	-1.453784	1	1	RP4-569M23.2	_
ENSG00000232470	0.43309	-1.453784	1	1	RP11-313D6.3	_
ENSG00000232876	0.43309	-1.453784	1	1	CTA-212D2.2	_

ENSG00000232907	0.43309	-1.453784	1	1	RP5-977B1.7	_
ENSG00000233231	0.43309	-1.453784	1	1	HNRNPA1P49	K12741
ENSG00000233436	0.43309	-1.453784	1	1	BTBD18	_
ENSG00000233967	0.43309	-1.453784	1	1	RP11-250B2.3	_
ENSG00000234264	0.43309	-1.453784	1	1	RP4-694A7.4	_
ENSG00000234289	0.43309	-1.453784	1	1	H2BFS	K11252
ENSG00000234337	0.43309	-1.453784	1	1	RP11-297L17.6	K09290
ENSG00000235408	0.43309	-1.453784	1	1	SNORA71B	_
ENSG00000235958	0.43309	-1.453784	1	1	UBOX5-AS1	_
ENSG00000236197	0.43309	-1.453784	1	1	AC002429.5	_
ENSG00000236662	0.43309	-1.453784	1	1	RP11-108L7.4	_
ENSG00000236969	0.43309	-1.453784	1	1	GGT8P	K18592
ENSG00000238260	0.43309	-1.453784	1	1	RP11-46F15.2	_
ENSG00000239033	0.43309	-1.453784	1	1	snoU13	_
ENSG00000239556	0.43309	-1.453784	1	1	AC004951.5	_
ENSG00000240163	0.43309	-1.453784	1	1	RP11-745A24.1	K02929
ENSG00000240571	0.43309	-1.453784	1	1	RP11-775D22.3	_
ENSG00000241889	0.43309	-1.453784	1	1	RP11-435F17.3	K17617
ENSG00000242853	0.43309	-1.453784	1	1	RN7SL749P	_
ENSG00000242960	0.43309	-1.453784	1	1	FTH1P23	K00522
ENSG00000243766	0.43309	-1.453784	1	1	HOTTIP	_
ENSG00000243964	0.43309	-1.453784	1	1	RP11-16F15.1	K02893
ENSG00000247675	0.43309	-1.453784	1	1	LRP4-AS1	_
ENSG00000249661	0.43309	-1.453784	1	1	TNRC18P1	_
ENSG00000249685	0.43309	-1.453784	1	1	RP11-360F5.3	_
ENSG00000250068	0.43309	-1.453784	1	1	RP11-576C12.1	_
ENSG00000250069	0.43309	-1.453784	1	1	CTB-131B5.2	_
ENSG00000250365	0.43309	-1.453784	1	1	CTD-2213F21.2	_
ENSG00000251307	0.43309	-1.453784	1	1	RP11-506H20.1	_
ENSG00000253666	0.43309	-1.453784	1	1	KB-1615E4.2	_
ENSG00000254294	0.43309	-1.453784	1	1	IMPDH1P6	K00088
ENSG00000254419	0.43309	-1.453784	1	1	RP11-261P9.4	_
ENSG00000255062	0.43309	-1.453784	1	1	RP11-712L6.5	_
ENSG00000255445	0.43309	-1.453784	1	1	RP11-573M3.3	_
ENSG00000257298	0.43309	-1.453784	1	1	RP3-405J10.3	_
ENSG00000257534	0.43309	-1.453784	1	1	RP11-834C11.10	_
ENSG00000257921	0.43309	-1.453784	1	1	RP11-571M6.15	K02357
ENSG00000258273	0.43309	-1.453784	1	1	RP11-370I10.4	K08789
ENSG00000258344	0.43309	-1.453784	1	1	RP11-968A15.8	_
ENSG00000258359	0.43309	-1.453784	1	1	PCNPP1	_
ENSG00000258819	0.43309	-1.453784	1	1	RP11-7F17.3	_
ENSG00000258984	0.43309	-1.453784	1	1	UBE2F-SCLY	K01763
ENSG00000259056	0.43309	-1.453784	1	1	DUXAP1	_
ENSG00000259315	0.43309	-1.453784	1	1	RP11-752G15.9	K05692
ENSG00000259381	0.43309	-1.453784	1	1	RP11-192M23.1	K14863
ENSG00000259498	0.43309	-1.453784	1	1	RP11-244F12.3	_
ENSG00000260037	0.43309	-1.453784	1	1	CTD-2524L6.3	_
ENSG00000260425	0.43309	-1.453784	1	1	LA16c-316G12.2	_
ENSG00000261386	0.43309	-1.453784	1	1	CTD-2012K14.6	_
ENSG00000261487	0.43309	-1.453784	1	1	AC135048.13	_
ENSG00000261759	0.43309	-1.453784	1	1	RP11-626G11.3	_
ENSG00000261773	0.43309	-1.453784	1	1	WI2-89031B12.1	_
ENSG00000263214	0.43309	-1.453784	1	1	ZNF205-AS1	_
ENSG00000263968	0.43309	-1.453784	1	1	RN7SL381P	_
ENSG00000264007	0.43309	-1.453784	1	1	RP11-68I3.10	_
ENSG00000264091	0.43309	-1.453784	1	1	AP002495.1	_
ENSG00000264554	0.43309	-1.453784	1	1	RN7SL793P	_
ENSG00000264808	0.43309	-1.453784	1	1	MIR4523	_

ENSG00000265661	0.43309	-1.453784	1	1	MIR3118-2	_
ENSG00000266910	0.43309	-1.453784	1	1	CTC-448F2.6	K14535
ENSG00000267167	0.43309	-1.453784	1	1	RP11-687F6.1	_
ENSG00000267474	0.43309	-1.453784	1	1	CTC-548K16.6	_
ENSG00000267532	0.43309	-1.453784	1	1	MIR497HG	_
ENSG00000267571	0.43309	-1.453784	1	1	AC104532.4	_
ENSG00000267710	0.43309	-1.453784	1	1	AC006116.20	_
ENSG00000267898	0.43309	-1.453784	1	1	CTD-2639E6.9	_
ENSG00000269243	0.43309	-1.453784	1	1	CTD-2231E14.8	_
ENSG00000269946	0.43309	-1.453784	1	1	RP11-2B6.3	_
ENSG00000270800	0.43309	-1.453784	1	1	RPS10-NUDT3	K02947
ENSG00000271580	0.43309	-1.453784	1	1	RP11-536L3.4	_
ENSG00000271788	0.43309	-1.453784	1	1	CTD-2201E18.5	_
ENSG00000272183	0.43309	-1.453784	1	1	RP11-523H20.3	_
ENSG00000272975	0.43309	-1.453784	1	1	CTC-297N7.11	_
ENSG00000273064	0.43309	-1.453784	1	1	RP11-474G23.3	_
ENSG00000273218	0.43309	-1.453784	1	1	LLNLR-246C6.1	_
ENSG00000273249	0.43309	-1.453784	1	1	LL09NC01-251B2.3	_
ENSG00000002079	0.293016	-1.038791	1	1	MYH16	K17751
ENSG00000006116	0.293016	-1.038791	1	1	CACNG3	K04868
ENSG00000047648	0.293016	-1.038791	1	1	ARHGAP6	_
ENSG00000079101	0.293016	-1.038791	1	1	CLUL1	_
ENSG00000099338	0.293016	-1.038791	1	1	CATSPERG	K16894
ENSG00000108924	0.293016	-1.038791	1	1	HLF	K09057
ENSG00000109101	0.293016	-1.038791	1	1	FOXN1	K09407
ENSG00000133640	0.293016	-1.038791	1	1	LRRIQ1	_
ENSG00000135253	0.293016	-1.038791	1	1	KCP	_
ENSG00000139946	0.293016	-1.038791	1	1	PELI2	K11964
ENSG00000146469	0.293016	-1.038791	1	1	VIP	K05264
ENSG00000162039	0.293016	-1.038791	1	1	MEIOB	K01557
ENSG00000162745	0.293016	-1.038791	1	1	OLFML2B	_
ENSG00000162997	0.293016	-1.038791	1	1	PRORSD1P	K12581
ENSG00000164404	0.293016	-1.038791	1	1	GDF9	K05498
ENSG00000171060	0.293016	-1.038791	1	1	C8orf74	_
ENSG00000171786	0.293016	-1.038791	1	1	NHLH1	K09075
ENSG00000172139	0.293016	-1.038791	1	1	SLC9C1	K14726
ENSG00000175868	0.293016	-1.038791	1	1	CALCB	K12332
ENSG00000182791	0.293016	-1.038791	1	1	CCDC87	_
ENSG00000184956	0.293016	-1.038791	1	1	MUC6	_
ENSG00000188596	0.293016	-1.038791	1	1	C12orf55	_
ENSG00000189269	0.293016	-1.038791	1	1	C22orf43	_
ENSG00000196218	0.293016	-1.038791	1	1	RYR1	K04961
ENSG00000198590	0.293016	-1.038791	1	1	C3orf35	_
ENSG00000199266	0.293016	-1.038791	1	1	SNORA60	_
ENSG00000211689	0.293016	-1.038791	1	1	TRGC1	_
ENSG00000214093	0.293016	-1.038791	1	1	RP11-247I13.3	K02964
ENSG00000214832	0.293016	-1.038791	1	1	UPF3AP2	K14328
ENSG00000214851	0.293016	-1.038791	1	1	LINC00612	_
ENSG00000215784	0.293016	-1.038791	1	1	FAM72D	_
ENSG00000224271	0.293016	-1.038791	1	1	RP11-191L9.4	_
ENSG00000224616	0.293016	-1.038791	1	1	RP11-305E17.6	_
ENSG00000226180	0.293016	-1.038791	1	1	AC010536.1	K19530
ENSG00000227060	0.293016	-1.038791	1	1	LINC00629	_
ENSG00000229677	0.293016	-1.038791	1	1	RP11-383F6.1	K02880
ENSG00000232310	0.293016	-1.038791	1	1	RP11-557H15.4	_
ENSG00000232888	0.293016	-1.038791	1	1	RPS11P5	K02949
ENSG00000234807	0.293016	-1.038791	1	1	LINC01135	_
ENSG00000237054	0.293016	-1.038791	1	1	PRMT5-AS1	_

ENSG00000238035	0.293016	-1.038791	1	1	AC138035.2	_
ENSG00000239763	0.293016	-1.038791	1	1	AC009120.3	K02889
ENSG00000242349	0.293016	-1.038791	1	1	NPPA-AS1	_
ENSG00000243824	0.293016	-1.038791	1	1	RP11-434O22.1	K02870
ENSG00000246523	0.293016	-1.038791	1	1	RP11-736K20.6	_
ENSG00000247595	0.293016	-1.038791	1	1	SPTY2D1-AS1	_
ENSG00000248559	0.293016	-1.038791	1	1	RP11-215P8.3	_
ENSG00000249839	0.293016	-1.038791	1	1	AC011330.5	K13024
ENSG00000251417	0.293016	-1.038791	1	1	RP11-1348G14.4	_
ENSG00000253170	0.293016	-1.038791	1	1	RP11-431J17.1	_
ENSG00000254851	0.293016	-1.038791	1	1	RP11-109L13.1	K08673
ENSG00000254859	0.293016	-1.038791	1	1	RP11-661A12.5	_
ENSG00000256383	0.293016	-1.038791	1	1	AC020910.2	_
ENSG00000257038	0.293016	-1.038791	1	1	RP11-800A3.7	_
ENSG00000259577	0.293016	-1.038791	1	1	RP11-430B1.2	_
ENSG00000260274	0.293016	-1.038791	1	1	RP11-817O13.8	_
ENSG00000264329	0.293016	-1.038791	1	1	MIR3911	_
ENSG00000265474	0.293016	-1.038791	1	1	AC010761.9	_
ENSG00000266921	0.293016	-1.038791	1	1	RP11-15A1.7	_
ENSG00000267544	0.293016	-1.038791	1	1	AC007229.3	_
ENSG00000268364	0.293016	-1.038791	1	1	SMC5-AS1	_
ENSG00000269318	0.293016	-1.038791	1	1	AC007292.3	_
ENSG00000269560	0.293016	-1.038791	1	1	CTD-2192J16.21	_
ENSG00000269657	0.293016	-1.038791	1	1	AC079210.1	K12581
ENSG00000269881	0.293016	-1.038791	1	1	ITFG3	K17258
ENSG00000270890	0.293016	-1.038791	1	1	RP3-468K18.6	_
ENSG00000271020	0.293016	-1.038791	1	1	RP11-10C24.1	_
ENSG00000271914	0.293016	-1.038791	1	1	RP4-789D17.5	_
ENSG00000272609	0.293016	-1.038791	1	1	RP11-461M2.2	_
ENSG00000272864	0.293016	-1.038791	1	1	RP11-17E13.2	_
ENSG00000101306	-0.277893	-0.471731	1	1	MYLK2	K00907
ENSG00000119703	-0.277893	-0.471731	1	1	ZC2HC1C	_
ENSG00000140323	-0.277893	-0.471731	1	1	DISP2	_
ENSG00000151846	-0.277893	-0.471731	1	1	PABPC3	K13126
ENSG00000157343	-0.277893	-0.471731	1	1	ARMC12	_
ENSG00000166343	-0.277893	-0.471731	1	1	MSS51	K17656
ENSG00000175564	-0.277893	-0.471731	1	1	UCP3	K15103
ENSG00000187952	-0.277893	-0.471731	1	1	HS6ST1P1	K02514
ENSG00000188801	-0.277893	-0.471731	1	1	ZNF322P1	K09228
ENSG00000196559	-0.277893	-0.471731	1	1	LINC00610	_
ENSG00000207383	-0.277893	-0.471731	1	1	Y_RNA	_
ENSG00000207554	-0.277893	-0.471731	1	1	MIR647	_
ENSG00000218350	-0.277893	-0.471731	1	1	LYPLA1P3	K06128
ENSG00000221299	-0.277893	-0.471731	1	1	Z83826.1	_
ENSG00000223572	-0.277893	-0.471731	1	1	CKMT1A	K00933
ENSG00000227295	-0.277893	-0.471731	1	1	ELL2P1	K15183
ENSG00000230537	-0.277893	-0.471731	1	1	RP11-305L7.1	_
ENSG00000233337	-0.277893	-0.471731	1	1	UBE2FP3	K10687
ENSG00000238279	-0.277893	-0.471731	1	1	BX470102.3	_
ENSG00000248592	-0.277893	-0.471731	1	1	TMEM110-MUSTN	_
ENSG00000250081	-0.277893	-0.471731	1	1	CTD-2116N20.1	_
ENSG00000250855	-0.277893	-0.471731	1	1	RP11-269F21.1	_
ENSG00000255026	-0.277893	-0.471731	1	1	RP11-326C3.2	_
ENSG00000261730	-0.277893	-0.471731	1	1	RP4-668J24.2	_
ENSG00000261732	-0.277893	-0.471731	1	1	LA16c-431H6.6	K17985
ENSG00000262703	-0.277893	-0.471731	1	1	RP11-485G7.6	_
ENSG00000013619	-0.272916	-1.177678	1	1	MAMLD1	K19512
ENSG00000089692	-0.272916	-1.177678	1	1	LAG3	K06565

ENSG00000103184	-0.272916	-1.177678	1	1	SEC14L5	_
ENSG00000108622	-0.272916	-1.177678	1	1	ICAM2	K06523
ENSG00000109339	-0.272916	-1.177678	1	1	MAPK10	K04440
ENSG00000112175	-0.272916	-1.177678	1	1	BMP5	K04663
ENSG00000124406	-0.272916	-1.177678	1	1	ATP8A1	K14802
ENSG00000128310	-0.272916	-1.177678	1	1	GALR3	K04232
ENSG00000129048	-0.272916	-1.177678	1	1	ACKR4	K04186
ENSG00000131885	-0.272916	-1.177678	1	1	KRT17P1	K07604
ENSG00000132141	-0.272916	-1.177678	1	1	CCT6B	K09498
ENSG00000145040	-0.272916	-1.177678	1	1	UCN2	K05257
ENSG00000146966	-0.272916	-1.177678	1	1	DENND2A	_
ENSG00000160224	-0.272916	-1.177678	1	1	AIRE	K10603
ENSG00000164304	-0.272916	-1.177678	1	1	CAGE1	_
ENSG00000170523	-0.272916	-1.177678	1	1	KRT83	K07605
ENSG00000173838	-0.272916	-1.177678	1	1	10-Mar	K10665
ENSG00000174514	-0.272916	-1.177678	1	1	MFSD4	_
ENSG00000179846	-0.272916	-1.177678	1	1	NKPD1	_
ENSG00000183153	-0.272916	-1.177678	1	1	GJD3	K07627
ENSG00000185689	-0.272916	-1.177678	1	1	C6orf201	K13239
ENSG00000186767	-0.272916	-1.177678	1	1	SPIN4	_
ENSG00000187556	-0.272916	-1.177678	1	1	NANOS3	K18761
ENSG00000197153	-0.272916	-1.177678	1	1	HIST1H3J	K11253
ENSG00000197670	-0.272916	-1.177678	1	1	RP4-724E16.2	_
ENSG00000197978	-0.272916	-1.177678	1	1	GOLGA6L9	_
ENSG00000198106	-0.272916	-1.177678	1	1	SNX29P2	_
ENSG00000200154	-0.272916	-1.177678	1	1	SNORD103A	_
ENSG00000204580	-0.272916	-1.177678	1	1	DDR1	K05124
ENSG00000205702	-0.272916	-1.177678	1	1	CYP2D7P	K07414
ENSG00000207181	-0.272916	-1.177678	1	1	SNORA14B	_
ENSG00000212175	-0.272916	-1.177678	1	1	SNORA12	_
ENSG00000214429	-0.272916	-1.177678	1	1	CYCSP6	K08738
ENSG00000225091	-0.272916	-1.177678	1	1	SNORA71A	_
ENSG00000225822	-0.272916	-1.177678	1	1	UBXN7-AS1	_
ENSG00000226312	-0.272916	-1.177678	1	1	CFLAR-AS1	_
ENSG00000227230	-0.272916	-1.177678	1	1	RP11-261C10.5	_
ENSG00000227848	-0.272916	-1.177678	1	1	SUCLA2-AS1	_
ENSG00000229528	-0.272916	-1.177678	1	1	RP5-1066H13.4	_
ENSG00000230623	-0.272916	-1.177678	1	1	RP11-469A15.2	_
ENSG00000230736	-0.272916	-1.177678	1	1	RP1-149A16.3	_
ENSG00000230836	-0.272916	-1.177678	1	1	AC104135.4	_
ENSG00000231050	-0.272916	-1.177678	1	1	RP1-140A9.1	_
ENSG00000232160	-0.272916	-1.177678	1	1	RAP2C-AS1	_
ENSG00000233491	-0.272916	-1.177678	1	1	AC010091.1	_
ENSG00000233859	-0.272916	-1.177678	1	1	ADH5P4	K00121
ENSG00000234155	-0.272916	-1.177678	1	1	RP11-30P6.6	_
ENSG00000234335	-0.272916	-1.177678	1	1	RPS4XP11	K02987
ENSG00000235092	-0.272916	-1.177678	1	1	AC011747.7	_
ENSG00000236307	-0.272916	-1.177678	1	1	AC104651.1	K15439
ENSG00000237238	-0.272916	-1.177678	1	1	BMS1P10	K14569
ENSG00000243244	-0.272916	-1.177678	1	1	STON1	_
ENSG00000244055	-0.272916	-1.177678	1	1	AC007566.10	_
ENSG00000248671	-0.272916	-1.177678	1	1	CTD-2313N18.5	_
ENSG00000249196	-0.272916	-1.177678	1	1	RP11-669N7.2	_
ENSG00000249786	-0.272916	-1.177678	1	1	EAF1-AS1	_
ENSG00000250848	-0.272916	-1.177678	1	1	CTD-2083E4.5	K15425
ENSG00000252965	-0.272916	-1.177678	1	1	Y_RNA	_
ENSG00000253366	-0.272916	-1.177678	1	1	RP11-589F5.4	K01195
ENSG00000254995	-0.272916	-1.177678	1	1	STX16-NPEPL1	K08489

ENSG00000255774	-0.272916	-1.177678	1	1	AP000439.3	_
ENSG00000256879	-0.272916	-1.177678	1	1	RP11-284H19.1	_
ENSG00000257543	-0.272916	-1.177678	1	1	RP11-321F8.4	_
ENSG00000259867	-0.272916	-1.177678	1	1	RP11-525K10.3	_
ENSG00000260941	-0.272916	-1.177678	1	1	LINC00622	_
ENSG00000261253	-0.272916	-1.177678	1	1	AC137932.6	_
ENSG00000261305	-0.272916	-1.177678	1	1	RP4-584D14.7	_
ENSG00000261898	-0.272916	-1.177678	1	1	RP11-314A20.5	_
ENSG00000262003	-0.272916	-1.177678	1	1	RP11-676J12.7	K04613
ENSG00000263422	-0.272916	-1.177678	1	1	AC005702.1	_
ENSG00000263786	-0.272916	-1.177678	1	1	RP11-649A18.4	_
ENSG00000265413	-0.272916	-1.177678	1	1	RP11-789C17.1	_
ENSG00000266926	-0.272916	-1.177678	1	1	AC005943.4	_
ENSG00000267416	-0.272916	-1.177678	1	1	CTD-2319I12.2	_
ENSG00000267980	-0.272916	-1.177678	1	1	AC007292.6	_
ENSG00000268129	-0.272916	-1.177678	1	1	RP11-91G21.1	_
ENSG00000268568	-0.272916	-1.177678	1	1	AC007228.9	_
ENSG00000268945	-0.272916	-1.177678	1	1	CTD-2192J16.26	_
ENSG00000270022	-0.272916	-1.177678	1	1	RNU12	_
ENSG00000272065	-0.272916	-1.177678	1	1	U91328.20	_
ENSG00000272081	-0.272916	-1.177678	1	1	CTD-2376I4.2	_
ENSG00000272692	-0.272916	-1.177678	1	1	RP11-399K21.14	_
ENSG00000272721	-0.272916	-1.177678	1	1	RP11-778D9.12	_
ENSG00000272761	-0.272916	-1.177678	1	1	RP11-572C15.6	_
ENSG00000273004	-0.272916	-1.177678	1	1	GS1-279B7.2	_
ENSG00000004838	0.228977	0.081819	1	1	ZMYND10	_
ENSG00000182795	0.228977	0.081819	1	1	C1orf116	_
ENSG00000185614	0.228977	0.081819	1	1	FAM212A	_
ENSG00000214140	0.228977	0.081819	1	1	PRCD	K19637
ENSG00000225218	0.228977	0.081819	1	1	AP001628.6	_
ENSG00000234869	0.228977	0.081819	1	1	RP3-439F8.1	_
ENSG00000268912	0.228977	0.081819	1	1	CTD-2619J13.17	_
ENSG00000079393	0.226491	-0.716902	1	1	DUSP13	K14165
ENSG00000129535	0.226491	-0.716902	1	1	NRL	K09038
ENSG00000143344	0.226491	-0.716902	1	1	RGL1	K17635
ENSG00000149328	0.226491	-0.716902	1	1	GLB1L2	_
ENSG00000149346	0.226491	-0.716902	1	1	SLX4IP	_
ENSG00000159784	0.226491	-0.716902	1	1	FAM131B	_
ENSG00000179296	0.226491	-0.716902	1	1	CTGLF12P	K12491
ENSG00000180672	0.226491	-0.716902	1	1	AC007362.1	_
ENSG00000183542	0.226491	-0.716902	1	1	KLRC4	K06541
ENSG00000185565	0.226491	-0.716902	1	1	LSAMP	K06772
ENSG00000188610	0.226491	-0.716902	1	1	FAM72B	_
ENSG00000213943	0.226491	-0.716902	1	1	KRT18P17	K07604
ENSG00000215414	0.226491	-0.716902	1	1	PSMA6P1	K02730
ENSG00000215493	0.226491	-0.716902	1	1	XXbac-B562F10.11	K10450
ENSG00000223551	0.226491	-0.716902	1	1	TMSB4XP4	K05764
ENSG00000227440	0.226491	-0.716902	1	1	ATP5G1P4	K02128
ENSG00000227742	0.226491	-0.716902	1	1	CALR4P	K08057
ENSG00000230387	0.226491	-0.716902	1	1	RP4-737E23.2	_
ENSG00000231351	0.226491	-0.716902	1	1	AC111200.7	_
ENSG00000235448	0.226491	-0.716902	1	1	RP11-3L8.3	_
ENSG00000235897	0.226491	-0.716902	1	1	TM4SF19-AS1	_
ENSG00000238298	0.226491	-0.716902	1	1	snoU13	_
ENSG00000242252	0.226491	-0.716902	1	1	BGLAP	K11546
ENSG00000242968	0.226491	-0.716902	1	1	RP11-731C17.1	_
ENSG00000243015	0.226491	-0.716902	1	1	RN7SL737P	_
ENSG00000251179	0.226491	-0.716902	1	1	RP11-893F2.9	_

ENSG00000253824	0.226491	-0.716902	1	1	KB-173C10.2	_
ENSG00000255819	0.226491	-0.716902	1	1	KLRC4-KLRK1	K06541
ENSG00000256210	0.226491	-0.716902	1	1	AC005255.3	_
ENSG00000259015	0.226491	-0.716902	1	1	RP11-109N23.6	_
ENSG00000259291	0.226491	-0.716902	1	1	RP11-617F23.1	_
ENSG00000259746	0.226491	-0.716902	1	1	HSPE1P3	K04078
ENSG00000260063	0.226491	-0.716902	1	1	RP5-968P14.2	_
ENSG00000260213	0.226491	-0.716902	1	1	RP11-303E16.3	_
ENSG00000260290	0.226491	-0.716902	1	1	CTD-2033A16.2	K13214
ENSG00000261168	0.226491	-0.716902	1	1	RP11-68I18.10	_
ENSG00000261338	0.226491	-0.716902	1	1	RP11-378A13.1	_
ENSG00000263818	0.226491	-0.716902	1	1	CTD-2206N4.4	K09228
ENSG00000270060	0.226491	-0.716902	1	1	RP11-390K5.6	_
ENSG00000272084	0.226491	-0.716902	1	1	RP5-1126H10.2	_
ENSG00000272572	0.226491	-0.716902	1	1	RP11-179B2.2	_
ENSG00000105642	-0.220814	-0.248569	1	1	KCNN1	_
ENSG00000112309	-0.220814	-0.248569	1	1	B3GAT2	K10157
ENSG00000153294	-0.220814	-0.248569	1	1	GPR115	K08457
ENSG00000168542	-0.220814	-0.248569	1	1	COL3A1	K19720
ENSG00000176029	-0.220814	-0.248569	1	1	C11orf16	_
ENSG00000188033	-0.220814	-0.248569	1	1	ZNF490	K09228
ENSG00000188095	-0.220814	-0.248569	1	1	MESP2	K09076
ENSG00000206559	-0.220814	-0.248569	1	1	ZCWPW2	_
ENSG00000214558	-0.220814	-0.248569	1	1	RP11-74E24.2	_
ENSG00000227619	-0.220814	-0.248569	1	1	RP11-492E3.2	_
ENSG00000254165	-0.220814	-0.248569	1	1	RP11-503E24.2	_
ENSG00000256576	-0.220814	-0.248569	1	1	RP13-977J11.2	_
ENSG00000259354	-0.220814	-0.248569	1	1	RP11-519G16.3	_
ENSG00000265511	-0.220814	-0.248569	1	1	RP11-524F11.1	_
ENSG00000267213	-0.220814	-0.248569	1	1	AC007773.2	_
ENSG00000267242	-0.220814	-0.248569	1	1	AC069278.4	_
ENSG00000273265	-0.220814	-0.248569	1	1	RP11-353K11.1	_
ENSG00000095752	-0.207083	0.310517	1	1	IL11	K05417
ENSG00000152380	-0.207083	0.310517	1	1	FAM151B	_
ENSG00000152726	-0.207083	0.310517	1	1	FAM21B	K18462
ENSG00000214562	-0.207083	0.310517	1	1	NUTM2D	_
ENSG00000229431	-0.207083	0.310517	1	1	RP1-92O14.6	_
ENSG00000245522	-0.207083	0.310517	1	1	RP11-540A21.2	_
ENSG00000251550	-0.207083	0.310517	1	1	RP11-501C14.7	K18171
ENSG00000255468	-0.207083	0.310517	1	1	RP11-867G23.8	K15014
ENSG00000261888	-0.207083	0.310517	1	1	AC144831.1	_
ENSG00000273145	-0.207083	0.310517	1	1	CITF22-92A6.1	_
ENSG00000085231	0.187593	-0.453904	1	1	TAF9	K14535
ENSG00000105737	0.187593	-0.453904	1	1	GRIK5	K05205
ENSG00000163126	0.187593	-0.453904	1	1	ANKRD23	_
ENSG00000166145	0.187593	-0.453904	1	1	SPINT1	K15619
ENSG00000176909	0.187593	-0.453904	1	1	MAMSTR	_
ENSG00000204164	0.187593	-0.453904	1	1	BMS1P5	K12491
ENSG00000207864	0.187593	-0.453904	1	1	MIR27B	_
ENSG00000224680	0.187593	-0.453904	1	1	PLA2G12AP1	K01047
ENSG00000225171	0.187593	-0.453904	1	1	DUTP6	K01520
ENSG00000226009	0.187593	-0.453904	1	1	KCNIP2-AS1	_
ENSG00000226329	0.187593	-0.453904	1	1	AC005682.6	K14685
ENSG00000228452	0.187593	-0.453904	1	1	RP5-994D16.9	_
ENSG00000229589	0.187593	-0.453904	1	1	ACVR2B-AS1	_
ENSG00000236671	0.187593	-0.453904	1	1	PRKG1-AS1	_
ENSG00000237436	0.187593	-0.453904	1	1	RP11-312B8.1	_
ENSG00000238083	0.187593	-0.453904	1	1	LRRC37A2	_

ENSG00000254485	0.187593	-0.453904	1	1	RP11-380O24.1	_
ENSG00000259706	0.187593	-0.453904	1	1	HSP90B2P	K09487
ENSG00000260781	0.187593	-0.453904	1	1	ARHGAP23P1	_
ENSG00000267439	0.187593	-0.453904	1	1	AC002398.11	_
ENSG00000273313	0.187593	-0.453904	1	1	RBAKDN	_
ENSG00000006740	-0.181199	-0.05522	1	1	ARHGAP44	_
ENSG00000019144	-0.181199	-0.05522	1	1	PHLDB1	_
ENSG00000091106	-0.181199	-0.05522	1	1	NLRC4	K12805
ENSG00000136197	-0.181199	-0.05522	1	1	C7orf25	_
ENSG00000173110	-0.181199	-0.05522	1	1	HSPA6	K03283
ENSG00000180336	-0.181199	-0.05522	1	1	C17orf104	K13349
ENSG00000215033	-0.181199	-0.05522	1	1	AL603965.1	_
ENSG00000221164	-0.181199	-0.05522	1	1	SNORA11	_
ENSG00000228439	-0.181199	-0.05522	1	1	TSTD3	_
ENSG00000230798	-0.181199	-0.05522	1	1	RP4-792G4.2	_
ENSG00000233056	-0.181199	-0.05522	1	1	ERVH48-1	_
ENSG00000237977	-0.181199	-0.05522	1	1	EIF4HP2	_
ENSG00000244693	-0.181199	-0.05522	1	1	CTAGE8	_
ENSG00000258101	-0.181199	-0.05522	1	1	RP11-977B10.2	_
ENSG00000263004	-0.181199	-0.05522	1	1	RP11-166P13.3	_
ENSG00000267653	-0.181199	-0.05522	1	1	RP1-193H18.3	_
ENSG00000002745	-0.178936	-0.829121	1	1	WNT16	K01558
ENSG00000087916	-0.178936	-0.829121	1	1	SLC6A14	K05038
ENSG00000110427	-0.178936	-0.829121	1	1	KIAA1549L	_
ENSG00000128596	-0.178936	-0.829121	1	1	CCDC136	_
ENSG00000130545	-0.178936	-0.829121	1	1	CRB3	K06090
ENSG00000132702	-0.178936	-0.829121	1	1	HAPLN2	K06851
ENSG00000143891	-0.178936	-0.829121	1	1	GALM	K01785
ENSG00000143994	-0.178936	-0.829121	1	1	ABHD1	K13696
ENSG00000157890	-0.178936	-0.829121	1	1	MEGF11	_
ENSG00000162892	-0.178936	-0.829121	1	1	IL24	K05444
ENSG00000164849	-0.178936	-0.829121	1	1	GPR146	_
ENSG00000177144	-0.178936	-0.829121	1	1	NUDT4P1	K07766
ENSG00000189136	-0.178936	-0.829121	1	1	UBE2Q2P1	K10582
ENSG00000189144	-0.178936	-0.829121	1	1	ZNF573	K09228
ENSG00000204149	-0.178936	-0.829121	1	1	AGAP6	K12491
ENSG00000218305	-0.178936	-0.829121	1	1	AC006024.4	K06639
ENSG00000225764	-0.178936	-0.829121	1	1	LEPREL1-AS1	_
ENSG00000226853	-0.178936	-0.829121	1	1	AC010894.3	_
ENSG00000228817	-0.178936	-0.829121	1	1	BACH1-IT2	_
ENSG00000230176	-0.178936	-0.829121	1	1	RP4-779E11.3	_
ENSG00000232216	-0.178936	-0.829121	1	1	IGHV3-43	K06856
ENSG00000232713	-0.178936	-0.829121	1	1	AC010733.5	K02951
ENSG00000232860	-0.178936	-0.829121	1	1	SMG7-AS1	_
ENSG00000233246	-0.178936	-0.829121	1	1	RP11-415J8.5	_
ENSG00000235314	-0.178936	-0.829121	1	1	LINC00957	_
ENSG00000236526	-0.178936	-0.829121	1	1	RP4-742J24.2	_
ENSG00000238041	-0.178936	-0.829121	1	1	RP11-59K5.1	_
ENSG00000240207	-0.178936	-0.829121	1	1	RP11-379F4.4	_
ENSG00000240350	-0.178936	-0.829121	1	1	AC017002.1	_
ENSG00000243224	-0.178936	-0.829121	1	1	RP5-1157M23.2	_
ENSG00000248257	-0.178936	-0.829121	1	1	PSG10P	K06500
ENSG00000249007	-0.178936	-0.829121	1	1	RP11-510N19.5	_
ENSG00000249628	-0.178936	-0.829121	1	1	LINC00942	_
ENSG00000251259	-0.178936	-0.829121	1	1	AC004069.2	_
ENSG00000251396	-0.178936	-0.829121	1	1	RP11-163N6.2	_
ENSG00000253508	-0.178936	-0.829121	1	1	RP1-170O19.14	_
ENSG00000253636	-0.178936	-0.829121	1	1	RP11-531A24.5	_

ENSG00000256633	-0.178936	-0.829121	1	1	RP11-169D4.2	_
ENSG00000258179	-0.178936	-0.829121	1	1	RP11-248E9.7	K11087
ENSG00000263335	-0.178936	-0.829121	1	1	AF001548.5	_
ENSG00000268947	-0.178936	-0.829121	1	1	AD000684.2	_
ENSG00000271882	-0.178936	-0.829121	1	1	KB-1410C5.5	_
ENSG00000272010	-0.178936	-0.829121	1	1	CTD-3025N20.3	_
ENSG00000273045	-0.178936	-0.829121	1	1	C2ORF15	_
ENSG00000273356	-0.178936	-0.829121	1	1	RP11-804H8.6	_
ENSG00000175911	-0.152086	0.115352	1	1	AC127496.1	K09228
ENSG00000184925	-0.152086	0.115352	1	1	LCN12	_
ENSG00000205771	-0.152086	0.115352	1	1	CATSPER2P1	K16890
ENSG00000249684	-0.152086	0.115352	1	1	RP11-423H2.3	_
ENSG00000103034	0.144016	-0.038928	1	1	NDRG4	_
ENSG00000160111	0.144016	-0.038928	1	1	CPAMD8	_
ENSG00000162068	0.144016	-0.038928	1	1	NTN3	K06844
ENSG00000182057	0.144016	-0.038928	1	1	Z83851.3	_
ENSG00000186409	0.144016	-0.038928	1	1	CCDC30	_
ENSG00000186714	0.144016	-0.038928	1	1	CCDC73	K15030
ENSG00000197813	0.144016	-0.038928	1	1	CTC-301O7.4	_
ENSG00000206140	0.144016	-0.038928	1	1	TMEM191C	_
ENSG00000233198	0.144016	-0.038928	1	1	RNF224	_
ENSG00000241962	0.144016	-0.038928	1	1	C2orf15	K02907
ENSG00000243477	0.144016	-0.038928	1	1	NAT6	_
ENSG00000253210	0.144016	-0.038928	1	1	RP11-809O17.1	_
ENSG00000263345	0.144016	-0.038928	1	1	RP1-59D14.5	_
ENSG00000268543	0.144016	-0.038928	1	1	CTD-2619J13.16	_
ENSG00000136490	0.130583	0.130969	1	1	LIMD2	_
ENSG00000163357	0.130583	0.130969	1	1	DCST1	K10653
ENSG00000163406	0.130583	0.130969	1	1	SLC15A2	K14637
ENSG00000176920	0.130583	0.130969	1	1	FUT2	K00718
ENSG00000184378	0.130583	0.130969	1	1	ACTRT3	_
ENSG00000224635	0.130583	0.130969	1	1	RP4-564F22.5	_
ENSG00000225507	0.130583	0.130969	1	1	AC069282.6	K08866
ENSG00000230163	0.130583	0.130969	1	1	RP5-997D16.2	_
ENSG00000235298	0.130583	0.130969	1	1	RP11-575L7.8	_
ENSG00000258469	0.130583	0.130969	1	1	CHMP4BP1	K12194
ENSG00000261597	0.130583	0.130969	1	1	RP11-353B9.1	_
ENSG00000267280	0.130583	0.130969	1	1	RP11-332H18.4	_
ENSG00000269423	0.130583	0.130969	1	1	CTC-273B12.6	_
ENSG00000272468	0.130583	0.130969	1	1	RP1-86C11.7	_
ENSG00000125508	-0.129783	0.26795	1	1	SRMS	K08895
ENSG00000147255	-0.129783	0.26795	1	1	IGSF1	_
ENSG00000186704	-0.129783	0.26795	1	1	DTX2P1	K06058
ENSG00000245534	-0.129783	0.26795	1	1	RP11-219B17.1	_
ENSG00000254595	-0.129783	0.26795	1	1	CTD-2010I16.1	K05862
ENSG00000260448	-0.129783	0.26795	1	1	RP11-449H11.1	_
ENSG00000260727	-0.129783	0.26795	1	1	SLC7A5P1	K13780
ENSG00000262222	-0.129783	0.26795	1	1	RP11-876N24.4	_
ENSG00000090920	-0.128539	-0.54844	1	1	FCGBP	_
ENSG00000100206	-0.128539	-0.54844	1	1	DMC1	K10872
ENSG00000105695	-0.128539	-0.54844	1	1	MAG	K06771
ENSG00000114923	-0.128539	-0.54844	1	1	SLC4A3	K13856
ENSG00000139985	-0.128539	-0.54844	1	1	ADAM21	K08610
ENSG00000154252	-0.128539	-0.54844	1	1	GAL3ST2	K09675
ENSG00000197540	-0.128539	-0.54844	1	1	GZMM	K08649
ENSG00000213648	-0.128539	-0.54844	1	1	SULT1A4	K01014
ENSG00000216906	-0.128539	-0.54844	1	1	RP11-350J20.9	_
ENSG00000217165	-0.128539	-0.54844	1	1	ANKRD18EP	_

ENSG00000230185	-0.128539	-0.54844	1	1	C9orf147	_
ENSG00000230224	-0.128539	-0.54844	1	1	PHBP9	K17080
ENSG00000240006	-0.128539	-0.54844	1	1	RP11-200A1.1	_
ENSG00000257636	-0.128539	-0.54844	1	1	RP11-1103G16.1	_
ENSG00000260645	-0.128539	-0.54844	1	1	RP11-250B2.5	_
ENSG00000261071	-0.128539	-0.54844	1	1	RP1-223E5.4	_
ENSG00000263280	-0.128539	-0.54844	1	1	LA16c-325D7.2	_
ENSG00000269942	-0.128539	-0.54844	1	1	RP11-29B2.5	_
ENSG00000273000	-0.128539	-0.54844	1	1	KB-1572G7.2	_
ENSG00000273007	-0.128539	-0.54844	1	1	RP11-170N16.3	_
ENSG00000132016	0.120194	0.282947	1	1	C19orf57	_
ENSG00000154175	0.120194	0.282947	1	1	ABI3BP	K08464
ENSG00000163141	0.120194	0.282947	1	1	BNIPL	K18448
ENSG00000231663	0.120194	0.282947	1	1	RP5-827C21.4	_
ENSG00000239388	0.120194	0.282947	1	1	ASB14	K10336
ENSG00000254473	0.120194	0.282947	1	1	RP11-522I20.3	_
ENSG00000257390	0.120194	0.282947	1	1	RP11-762I7.5	K09534
ENSG00000269069	0.120194	0.282947	1	1	CTC-471F3.5	K12617
ENSG00000269974	0.120194	0.282947	1	1	RP11-932O9.10	_
ENSG00000272079	0.120194	0.282947	1	1	LA16c-380H5.5	_
ENSG00000272977	0.120194	0.282947	1	1	CTA-390C10.10	_
ENSG00000167377	-0.098114	1.060879	1	1	ZNF23	K09228
ENSG00000221821	-0.098114	1.060879	1	1	C6orf226	_
ENSG00000185414	-0.097857	0.532045	1	1	MRPL30	K02907
ENSG00000198838	-0.097857	0.532045	1	1	RYR3	K04963
ENSG00000215817	-0.097857	0.532045	1	1	ZC3H11B	_
ENSG00000226920	-0.097857	0.532045	1	1	RP5-1068B5.3	_
ENSG00000248432	-0.097857	0.532045	1	1	RP11-269F21.2	_
ENSG00000273306	-0.097857	0.532045	1	1	RP11-527J8.1	_
ENSG00000008516	-0.097095	-0.313455	1	1	MMP25	K08003
ENSG00000089505	-0.097095	-0.313455	1	1	CMTM1	_
ENSG00000124657	-0.097095	-0.313455	1	1	OR2B6	K04257
ENSG00000137673	-0.097095	-0.313455	1	1	MMP7	K01397
ENSG00000151117	-0.097095	-0.313455	1	1	TMEM86A	_
ENSG00000163430	-0.097095	-0.313455	1	1	FSTL1	_
ENSG00000163462	-0.097095	-0.313455	1	1	TRIM46	K12022
ENSG00000178386	-0.097095	-0.313455	1	1	ZNF223	K09228
ENSG00000179148	-0.097095	-0.313455	1	1	ALOXE3	K18684
ENSG00000187801	-0.097095	-0.313455	1	1	ZFP69B	K09228
ENSG00000212607	-0.097095	-0.313455	1	1	SNORA45	_
ENSG00000228878	-0.097095	-0.313455	1	1	AC007551.3	_
ENSG00000233930	-0.097095	-0.313455	1	1	KRTAP5-AS1	_
ENSG00000238120	-0.097095	-0.313455	1	1	CTA-941F9.9	_
ENSG00000243155	-0.097095	-0.313455	1	1	RP11-46A10.5	_
ENSG00000254676	-0.097095	-0.313455	1	1	RP11-727A23.4	_
ENSG00000257800	-0.097095	-0.313455	1	1	FNBP1P1	K09488
ENSG00000260269	-0.097095	-0.313455	1	1	CTD-2323K18.1	_
ENSG00000261438	-0.097095	-0.313455	1	1	RP11-399O19.9	_
ENSG00000264107	-0.097095	-0.313455	1	1	RP11-848P1.5	_
ENSG00000271550	-0.097095	-0.313455	1	1	RP11-561N12.7	K09228
ENSG00000271978	-0.097095	-0.313455	1	1	RP11-428J1.4	_
ENSG00000272370	-0.097095	-0.313455	1	1	RP11-307L14.1	_
ENSG00000170633	-0.089965	1.142248	1	1	RNF34	K10276
ENSG00000212864	-0.089965	1.142248	1	1	RNF208	_
ENSG00000231982	-0.089965	1.142248	1	1	RP11-573D15.1	_
ENSG00000260065	-0.089965	1.142248	1	1	CTA-445C9.15	_
ENSG00000267106	-0.089965	1.142248	1	1	C19orf82	K04613
ENSG00000224186	0.087291	0.960903	1	1	C5orf66	_

ENSG00000102103	-0.086347	1.5695	1	1	PQBP1	K12865
ENSG00000214106	-0.086347	1.5695	1	1	PAXIP1-AS2	K06115
ENSG00000129038	0.084232	1.04835	1	1	LOXL1	K14678
ENSG00000166192	0.084232	1.04835	1	1	SENP8	K08597
ENSG00000130818	0.081537	1.130798	1	1	ZNF426	K09228
ENSG00000273142	0.081537	1.130798	1	1	RP11-458F8.4	_
ENSG00000159917	-0.076267	1.292447	1	1	ZNF235	K09228
ENSG00000178226	-0.076267	1.292447	1	1	PRSS36	K09630
ENSG00000179913	-0.076267	1.292447	1	1	B3GNT3	K07970
ENSG00000217241	-0.076267	1.292447	1	1	CBX3P9	K11586
ENSG00000258057	-0.076267	1.292447	1	1	BCDIN3D-AS1	_
ENSG00000117009	-0.075601	-0.111345	1	1	KMO	K00486
ENSG00000147119	-0.075601	-0.111345	1	1	CHST7	K04743
ENSG00000153790	-0.075601	-0.111345	1	1	C7orf31	_
ENSG00000174521	-0.075601	-0.111345	1	1	TTC9B	_
ENSG00000213542	-0.075601	-0.111345	1	1	RP11-467H10.1	K02936
ENSG00000213707	-0.075601	-0.111345	1	1	HMGB1P10	K10802
ENSG00000228624	-0.075601	-0.111345	1	1	RP3-399L15.3	_
ENSG00000231989	-0.075601	-0.111345	1	1	PPP1R2P3	K16833
ENSG00000237126	-0.075601	-0.111345	1	1	AC073254.1	_
ENSG00000243902	-0.075601	-0.111345	1	1	RP1-63G5.5	_
ENSG00000247363	-0.075601	-0.111345	1	1	RP11-637A17.2	_
ENSG00000265242	-0.075601	-0.111345	1	1	RP11-649A18.7	_
ENSG00000267440	-0.075601	-0.111345	1	1	CTC-501O10.1	_
ENSG00000269899	-0.075601	-0.111345	1	1	RP11-589N15.2	_
ENSG00000270728	-0.075601	-0.111345	1	1	RP4-657E11.10	_
ENSG00000272144	-0.075601	-0.111345	1	1	CTD-2035E11.5	_
ENSG00000273026	-0.075601	-0.111345	1	1	RP11-422P24.10	_
ENSG00000250988	0.069005	1.60465	1	1	RP11-752G15.6	_
ENSG00000273038	0.066672	1.715663	1	1	RP11-479G22.8	_
ENSG00000144026	-0.065203	1.428533	1	1	ZNF514	K09228
ENSG00000182109	-0.065203	1.428533	1	1	RP11-69E11.4	_
ENSG00000114383	-0.060318	0.948796	1	1	TUSC2	_
ENSG00000196118	-0.060318	0.948796	1	1	C16orf93	K10696
ENSG00000232499	-0.060318	0.948796	1	1	RP11-512F24.1	K12741
ENSG00000143248	-0.059977	0.065968	1	1	RGS5	K16449
ENSG00000152042	-0.059977	0.065968	1	1	NBPF11	_
ENSG00000196388	-0.059977	0.065968	1	1	INCA1	_
ENSG00000246526	-0.059977	0.065968	1	1	RP11-539L10.2	_
ENSG00000253187	-0.059977	0.065968	1	1	HOXA10-AS	_
ENSG00000271992	-0.059977	0.065968	1	1	RP11-42O15.3	_
ENSG00000171119	-0.05608	1.552928	1	1	NRTN	_
ENSG00000271430	-0.05608	1.552928	1	1	RP3-368A4.5	_
ENSG00000027075	-0.053948	1.03662	1	1	PRKCH	K18051
ENSG00000240875	-0.053948	1.03662	1	1	LINC00886	_
ENSG00000173825	-0.052093	1.611337	1	1	TIGD3	_
ENSG00000215513	-0.052093	1.611337	1	1	PI4KAP1	K00888
ENSG00000237149	-0.052093	1.611337	1	1	ZNF503-AS2	_
ENSG00000235169	-0.048347	1.119423	1	1	SMIM1	_
ENSG00000124159	-0.048106	0.22391	1	1	MATN4	K06053
ENSG00000215302	-0.048106	0.22391	1	1	CTD-3092A11.1	K14559
ENSG00000228302	-0.048106	0.22391	1	1	RP11-186N15.3	_
ENSG00000240291	-0.048106	0.22391	1	1	RP11-499P20.2	_
ENSG00000259806	-0.048106	0.22391	1	1	CTD-2196E14.4	_
ENSG00000267123	-0.048106	0.22391	1	1	CTD-2357A8.3	_
ENSG00000179456	-0.045047	1.72153	1	1	ZBTB18	_
ENSG00000172250	-0.043384	1.197745	1	1	SERHL	_
ENSG00000197128	-0.043384	1.197745	1	1	ZNF772	K09228

ENSG00000270605	-0.043384	1.197745	1	1	RP5-1092A3.4	_
ENSG00000137496	-0.041918	1.773633	1	1	IL18BP	_
ENSG00000176125	-0.039013	1.823926	1	1	UFSP1	_
ENSG00000188368	-0.038955	1.272048	1	1	PRR19	K17985
ENSG00000216937	-0.038955	1.272048	1	1	CCDC7	_
ENSG00000236264	-0.038782	0.366298	1	1	RPL26P30	K02898
ENSG00000248099	-0.038782	0.366298	1	1	INSL3	K05255
ENSG00000249129	-0.038782	0.366298	1	1	SUDS3P1	K19201
ENSG00000267169	-0.038782	0.366298	1	1	CTB-55O6.12	_
ENSG00000090932	0.038769	1.973042	1	1	DLL3	K06051
ENSG00000180855	0.038769	1.973042	1	1	ZNF443	K09228
ENSG00000237264	0.038769	1.973042	1	1	FTH1P11	K00522
ENSG00000162415	0.038753	1.782306	1	1	ZSWIM5	_
ENSG00000154153	0.038738	1.620653	1	1	FAM134B	_
ENSG00000129204	0.038733	1.562481	1	1	USP6	K11837
ENSG00000180938	0.038733	1.562481	1	1	ZNF572	K09228
ENSG00000253200	0.038733	1.562481	1	1	RP11-582J16.5	_
ENSG00000089163	0.038726	1.501867	1	1	SIRT4	K11414
ENSG00000109991	0.038719	1.438596	1	1	P2RX3	K05217
ENSG00000272821	0.038719	1.438596	1	1	CTA-384D8.36	_
ENSG00000234476	0.038712	1.372426	1	1	RP11-496N12.6	_
ENSG00000263126	0.038703	1.303077	1	1	CTC-479C5.10	_
ENSG00000074660	0.038694	1.23023	1	1	SCARF1	_
ENSG00000115363	0.038694	1.23023	1	1	EVA1A	_
ENSG00000185250	0.038694	1.23023	1	1	PPIL6	K12739
ENSG00000068001	0.038684	1.153513	1	1	HYAL2	K01197
ENSG00000244151	0.038684	1.153513	1	1	RP11-148K1.12	_
ENSG00000273486	0.038684	1.153513	1	1	RP11-731C17.2	_
ENSG00000266714	0.038672	1.072491	1	1	MYO15B	K10361
ENSG00000160828	0.038626	0.797968	1	1	STAG3L2	K13055
ENSG00000243547	0.038626	0.797968	1	1	HNRNPKP4	K12886
ENSG00000258645	0.038626	0.797968	1	1	HSPE1P2	K04078
ENSG00000261663	0.038626	0.797968	1	1	RP11-304L19.11	_
ENSG00000265817	0.038626	0.797968	1	1	FSBP	K10877
ENSG00000267372	0.038626	0.797968	1	1	AC005330.2	_
ENSG00000227398	0.038606	0.693499	1	1	KIF9-AS1	_
ENSG00000258311	0.038606	0.693499	1	1	RP11-644F5.10	K00061
ENSG00000261460	0.038606	0.693499	1	1	RP11-106M3.3	_
ENSG00000010310	0.038553	0.458739	1	1	GIPR	K04580
ENSG00000132010	0.038553	0.458739	1	1	ZNF20	K17985
ENSG00000154898	0.038553	0.458739	1	1	CCDC144CP	K11837
ENSG00000175920	0.038553	0.458739	1	1	DOK7	_
ENSG00000187010	0.038553	0.458739	1	1	RHD	K06579
ENSG00000188687	0.038553	0.458739	1	1	SLC4A5	K13857
ENSG00000196220	0.038553	0.458739	1	1	SRGAP3	K07526
ENSG00000198723	0.038553	0.458739	1	1	C19orf45	_
ENSG00000204866	0.038553	0.458739	1	1	IGFL2	_
ENSG00000213085	0.038553	0.458739	1	1	CCDC19	_
ENSG00000057704	0.038517	0.325308	1	1	TMCC3	_
ENSG00000236054	0.038517	0.325308	1	1	LL22NC03-104C7.1	_
ENSG00000264575	0.038517	0.325308	1	1	LINC00526	_
ENSG00000267073	0.038517	0.325308	1	1	AC005256.1	_
ENSG00000138271	0.038473	0.178289	1	1	GPR87	K08389
ENSG00000170458	0.038473	0.178289	1	1	CD14	K04391
ENSG00000178726	0.038473	0.178289	1	1	THBD	K03907
ENSG00000201678	0.038473	0.178289	1	1	Y_RNA	_
ENSG00000230667	0.038473	0.178289	1	1	SETSIP	K11290
ENSG00000231160	0.038473	0.178289	1	1	RP11-617D20.1	_

ENSG00000248265	0.038473	0.178289	1	1	RP11-834C11.3	_
ENSG00000256050	0.038473	0.178289	1	1	RP11-982M15.6	_
ENSG00000109063	0.038417	0.014597	1	1	MYH3	K10352
ENSG00000182308	0.038417	0.014597	1	1	DCAF4L1	K11799
ENSG00000182327	0.038417	0.014597	1	1	GLTPD2	_
ENSG00000198035	0.038417	0.014597	1	1	AGAP9	K12491
ENSG00000205810	0.038417	0.014597	1	1	KLRC3	K06541
ENSG00000230082	0.038417	0.014597	1	1	PRRT3-AS1	_
ENSG00000236493	0.038417	0.014597	1	1	EIF2S2P3	K03238
ENSG00000255346	0.038417	0.014597	1	1	NOX5	_
ENSG00000256001	0.038417	0.014597	1	1	RP11-575F12.2	_
ENSG00000259732	0.038417	0.014597	1	1	RP11-59H7.3	_
ENSG00000260051	0.038417	0.014597	1	1	LA16c-390E6.4	_
ENSG00000267655	0.038417	0.014597	1	1	CTD-2286N8.2	_
ENSG00000269688	0.038417	0.014597	1	1	AC008982.2	_
ENSG00000272040	0.038417	0.014597	1	1	CTC-366B18.4	_
ENSG00000105877	0.038238	-0.381773	1	1	DNAH11	K10408
ENSG00000106336	0.038238	-0.381773	1	1	FBXO24	K10304
ENSG00000110203	0.038238	-0.381773	1	1	FOLR3	K13649
ENSG00000115423	0.038238	-0.381773	1	1	DNAH6	K10408
ENSG00000154479	0.038238	-0.381773	1	1	CCDC173	_
ENSG00000163704	0.038238	-0.381773	1	1	PRRT3	_
ENSG00000171224	0.038238	-0.381773	1	1	C10orf35	_
ENSG00000171757	0.038238	-0.381773	1	1	LRRC34	_
ENSG00000173926	0.038238	-0.381773	1	1	3-Mar	K10658
ENSG00000188681	0.038238	-0.381773	1	1	TEKT4P2	K18631
ENSG00000204540	0.038238	-0.381773	1	1	PSORS1C1	_
ENSG00000228271	0.038238	-0.381773	1	1	RP11-513G11.2	_
ENSG00000230658	0.038238	-0.381773	1	1	KLHL7-AS1	_
ENSG00000235381	0.038238	-0.381773	1	1	RP11-477D19.2	_
ENSG00000246792	0.038238	-0.381773	1	1	RP11-68L18.1	_
ENSG00000248103	0.038238	-0.381773	1	1	CTC-338M12.9	_
ENSG00000260742	0.038238	-0.381773	1	1	RP11-366L5.1	_
ENSG00000260757	0.038238	-0.381773	1	1	RP11-120K18.2	_
ENSG00000263050	0.038238	-0.381773	1	1	RP11-667K14.3	_
ENSG00000264456	0.038238	-0.381773	1	1	RP11-848P1.2	_
ENSG00000273148	0.038238	-0.381773	1	1	RP5-1068E13.7	_
ENSG00000049089	0.038083	-0.629953	1	1	COL9A2	K08131
ENSG00000081059	0.038083	-0.629953	1	1	TCF7	K02620
ENSG00000108370	0.038083	-0.629953	1	1	RGS9	K13765
ENSG00000108691	0.038083	-0.629953	1	1	CCL2	K14624
ENSG00000119632	0.038083	-0.629953	1	1	IFI27L2	_
ENSG00000134365	0.038083	-0.629953	1	1	CFHR4	K04004
ENSG00000145041	0.038083	-0.629953	1	1	VPRBP	K11789
ENSG00000145244	0.038083	-0.629953	1	1	CORIN	K09614
ENSG00000148680	0.038083	-0.629953	1	1	HTR7	K04163
ENSG00000154099	0.038083	-0.629953	1	1	DNAAF1	K19750
ENSG00000165140	0.038083	-0.629953	1	1	FBP1	K03841
ENSG00000166546	0.038083	-0.629953	1	1	BEAN1	K19324
ENSG00000177984	0.038083	-0.629953	1	1	LCN15	_
ENSG00000182257	0.038083	-0.629953	1	1	C22orf26	K12581
ENSG00000186364	0.038083	-0.629953	1	1	NUDT17	_
ENSG00000196371	0.038083	-0.629953	1	1	FUT4	K07632
ENSG00000197093	0.038083	-0.629953	1	1	GAL3ST4	K09677
ENSG00000225264	0.038083	-0.629953	1	1	ZNRF2P2	K10694
ENSG00000230303	0.038083	-0.629953	1	1	RP11-213G2.2	_
ENSG00000234043	0.038083	-0.629953	1	1	RP11-56M3.1	K13988
ENSG00000236675	0.038083	-0.629953	1	1	MTX1P1	K17776

ENSG00000236830	0.038083	-0.629953	1	1	CBR3-AS1	_
ENSG00000236924	0.038083	-0.629953	1	1	RP11-390F4.6	_
ENSG00000239407	0.038083	-0.629953	1	1	LL0XNC01-237H1.2	_
ENSG00000240996	0.038083	-0.629953	1	1	RP11-80H5.7	_
ENSG00000244459	0.038083	-0.629953	1	1	RP11-1398P2.1	_
ENSG00000247081	0.038083	-0.629953	1	1	RP11-318M2.2	_
ENSG00000249332	0.038083	-0.629953	1	1	RP1-251I12.1	_
ENSG00000251611	0.038083	-0.629953	1	1	RP11-610P16.1	_
ENSG00000251615	0.038083	-0.629953	1	1	RP11-774O3.3	_
ENSG00000255200	0.038083	-0.629953	1	1	AP003068.18	K01834
ENSG00000259953	0.038083	-0.629953	1	1	RP11-4O1.2	_
ENSG00000266258	0.038083	-0.629953	1	1	RP11-41O4.1	_
ENSG00000270580	0.038083	-0.629953	1	1	RP11-1186N24.5	_
ENSG00000271452	0.038083	-0.629953	1	1	RP11-342K6.2	_
ENSG00000272367	0.038083	-0.629953	1	1	CTC-428H11.2	_
ENSG00000053438	0.037826	-0.929792	1	1	NNAT	_
ENSG00000101342	0.037826	-0.929792	1	1	TLDC2	_
ENSG00000111816	0.037826	-0.929792	1	1	FRK	K08892
ENSG00000126583	0.037826	-0.929792	1	1	PRKCG	K19663
ENSG00000132530	0.037826	-0.929792	1	1	XAF1	_
ENSG00000154096	0.037826	-0.929792	1	1	THY1	K06514
ENSG00000170161	0.037826	-0.929792	1	1	RP11-262H14.4	_
ENSG00000177494	0.037826	-0.929792	1	1	ZBED2	K06517
ENSG00000179673	0.037826	-0.929792	1	1	RPRML	K10128
ENSG00000181227	0.037826	-0.929792	1	1	RP4-682C21.2	K00658
ENSG00000181315	0.037826	-0.929792	1	1	ZNF322	_
ENSG00000183248	0.037826	-0.929792	1	1	CTD-3193O13.9	_
ENSG00000185495	0.037826	-0.929792	1	1	RP11-504P24.4	_
ENSG00000188710	0.037826	-0.929792	1	1	QRFP	_
ENSG00000196966	0.037826	-0.929792	1	1	HIST1H3E	K11253
ENSG00000204019	0.037826	-0.929792	1	1	CT83	_
ENSG00000205790	0.037826	-0.929792	1	1	AC005594.3	_
ENSG00000212719	0.037826	-0.929792	1	1	C17orf51	K10408
ENSG00000213492	0.037826	-0.929792	1	1	NT5C3AP1	K01081
ENSG00000221845	0.037826	-0.929792	1	1	C7orf65	K04613
ENSG00000226986	0.037826	-0.929792	1	1	RP11-543B16.2	_
ENSG00000227073	0.037826	-0.929792	1	1	SDHDP2	K00237
ENSG00000229021	0.037826	-0.929792	1	1	NBPF18P	_
ENSG00000230701	0.037826	-0.929792	1	1	FBXW4P1	K10262
ENSG00000232453	0.037826	-0.929792	1	1	RP4-794H19.1	_
ENSG00000232872	0.037826	-0.929792	1	1	CTAGE3P	_
ENSG00000235204	0.037826	-0.929792	1	1	RP11-121A14.2	_
ENSG00000235748	0.037826	-0.929792	1	1	RP11-32D17.4	K16941
ENSG00000236349	0.037826	-0.929792	1	1	SUCLG2P2	K01900
ENSG00000236710	0.037826	-0.929792	1	1	AC108448.2	_
ENSG00000237685	0.037826	-0.929792	1	1	RP11-360O19.4	_
ENSG00000242852	0.037826	-0.929792	1	1	ZNF709	K09228
ENSG00000250794	0.037826	-0.929792	1	1	ALG1L12P	K03842
ENSG00000252412	0.037826	-0.929792	1	1	Y_RNA	_
ENSG00000253540	0.037826	-0.929792	1	1	FAM86HP	K12354
ENSG00000254461	0.037826	-0.929792	1	1	RP11-755F10.3	_
ENSG00000254783	0.037826	-0.929792	1	1	RP11-320L11.2	_
ENSG00000255182	0.037826	-0.929792	1	1	CTD-2517M22.14	_
ENSG00000259582	0.037826	-0.929792	1	1	RP11-461F11.3	_
ENSG00000259668	0.037826	-0.929792	1	1	RP11-707P17.2	_
ENSG00000262180	0.037826	-0.929792	1	1	OCLM	_
ENSG00000262951	0.037826	-0.929792	1	1	RP11-670E13.2	_
ENSG00000264219	0.037826	-0.929792	1	1	MIR4442	_

ENSG00000265095	0.037826	-0.929792	1	1	FTLP12	_
ENSG00000267414	0.037826	-0.929792	1	1	RP11-456K23.1	_
ENSG00000267730	0.037826	-0.929792	1	1	CTD-2537I9.5	_
ENSG00000267737	0.037826	-0.929792	1	1	AC061992.2	_
ENSG00000268340	0.037826	-0.929792	1	1	RP3-477O4.16	_
ENSG00000268812	0.037826	-0.929792	1	1	RP1-102K2.8	_
ENSG00000269693	0.037826	-0.929792	1	1	CTD-2192J16.20	K09228
ENSG00000271662	0.037826	-0.929792	1	1	RP11-141C7.3	K15425
ENSG00000271927	0.037826	-0.929792	1	1	CTD-2541M15.4	_
ENSG00000272172	0.037826	-0.929792	1	1	RP13-582O9.7	_
ENSG00000010361	0.037324	-1.308612	1	1	FUZ	_
ENSG00000028277	0.037324	-1.308612	1	1	POU2F2	K09364
ENSG00000042062	0.037324	-1.308612	1	1	FAM65C	_
ENSG00000047662	0.037324	-1.308612	1	1	FAM184B	K09228
ENSG00000074966	0.037324	-1.308612	1	1	TXK	K08016
ENSG00000088881	0.037324	-1.308612	1	1	EBF4	K09103
ENSG00000095464	0.037324	-1.308612	1	1	PDE6C	K13757
ENSG00000102003	0.037324	-1.308612	1	1	SYP	_
ENSG00000104490	0.037324	-1.308612	1	1	NCALD	K19695
ENSG00000106125	0.037324	-1.308612	1	1	FAM188B	K01875
ENSG00000126903	0.037324	-1.308612	1	1	SLC10A3	K14343
ENSG00000141696	0.037324	-1.308612	1	1	LEPREL4	_
ENSG00000144488	0.037324	-1.308612	1	1	ESPNL	_
ENSG00000157782	0.037324	-1.308612	1	1	CABP1	_
ENSG00000160097	0.037324	-1.308612	1	1	FNDC5	_
ENSG00000164619	0.037324	-1.308612	1	1	BMPER	_
ENSG00000164621	0.037324	-1.308612	1	1	SMAD5-AS1	_
ENSG00000165028	0.037324	-1.308612	1	1	NIPSNAP3B	_
ENSG00000165188	0.037324	-1.308612	1	1	RNF183	_
ENSG00000165935	0.037324	-1.308612	1	1	SMCO2	_
ENSG00000167945	0.037324	-1.308612	1	1	PRR25	_
ENSG00000168062	0.037324	-1.308612	1	1	BATF2	K09034
ENSG00000171345	0.037324	-1.308612	1	1	KRT19	K07604
ENSG00000171365	0.037324	-1.308612	1	1	CLCN5	K05012
ENSG00000171916	0.037324	-1.308612	1	1	LGALS9C	K10093
ENSG00000172789	0.037324	-1.308612	1	1	HOXC5	K09305
ENSG00000174236	0.037324	-1.308612	1	1	REP15	_
ENSG00000174326	0.037324	-1.308612	1	1	SLC16A11	K08188
ENSG00000177340	0.037324	-1.308612	1	1	AC024940.1	_
ENSG00000182217	0.037324	-1.308612	1	1	HIST2H4B	K11254
ENSG00000184060	0.037324	-1.308612	1	1	ADAP2	_
ENSG00000185666	0.037324	-1.308612	1	1	SYN3	_
ENSG00000186340	0.037324	-1.308612	1	1	THBS2	K04659
ENSG00000187151	0.037324	-1.308612	1	1	ANGPTL5	_
ENSG00000187944	0.037324	-1.308612	1	1	C2orf66	_
ENSG00000188385	0.037324	-1.308612	1	1	JAKMIP3	_
ENSG00000196562	0.037324	-1.308612	1	1	SULF2	K14607
ENSG00000197858	0.037324	-1.308612	1	1	GPAA1	K05289
ENSG00000198658	0.037324	-1.308612	1	1	ABHD17AP2	_
ENSG00000204584	0.037324	-1.308612	1	1	RP11-304F15.3	_
ENSG00000207721	0.037324	-1.308612	1	1	MIR186	_
ENSG00000213280	0.037324	-1.308612	1	1	RP11-212P7.1	K02947
ENSG00000213809	0.037324	-1.308612	1	1	KLRK1	K06728
ENSG00000224549	0.037324	-1.308612	1	1	RP11-370B11.3	_
ENSG00000224956	0.037324	-1.308612	1	1	RP11-206L10.1	_
ENSG00000225913	0.037324	-1.308612	1	1	RP11-57C13.6	_
ENSG00000226239	0.037324	-1.308612	1	1	RP1-310O13.7	_
ENSG00000226259	0.037324	-1.308612	1	1	GTF2H2B	K03142

ENSG00000227492	0.037324	-1.308612	1	1	RP11-316M21.6	_
ENSG00000227741	0.037324	-1.308612	1	1	RP11-536C5.7	_
ENSG00000228212	0.037324	-1.308612	1	1	OFD1P17	K16480
ENSG00000229325	0.037324	-1.308612	1	1	ACAP2-IT1	_
ENSG00000229728	0.037324	-1.308612	1	1	RP11-314N13.3	_
ENSG00000231167	0.037324	-1.308612	1	1	YBX1P2	K09276
ENSG00000231940	0.037324	-1.308612	1	1	RPS7P3	K02993
ENSG00000232013	0.037324	-1.308612	1	1	AC107021.1	_
ENSG00000232725	0.037324	-1.308612	1	1	U52111.14	_
ENSG00000233547	0.037324	-1.308612	1	1	RP11-57H14.2	_
ENSG00000234740	0.037324	-1.308612	1	1	RP11-272P10.2	_
ENSG00000234945	0.037324	-1.308612	1	1	AC109828.1	_
ENSG00000235489	0.037324	-1.308612	1	1	DBF4P1	K06629
ENSG00000236643	0.037324	-1.308612	1	1	RP11-175D17.3	_
ENSG00000237821	0.037324	-1.308612	1	1	AC083873.4	K09560
ENSG00000238924	0.037324	-1.308612	1	1	RNU7-163P	_
ENSG00000241081	0.037324	-1.308612	1	1	RP11-488C13.1	K02891
ENSG00000242288	0.037324	-1.308612	1	1	RP11-464F9.1	_
ENSG00000242756	0.037324	-1.308612	1	1	RHOT1P3	K07870
ENSG00000246090	0.037324	-1.308612	1	1	RP11-696N14.1	_
ENSG00000246366	0.037324	-1.308612	1	1	RP11-382J12.1	_
ENSG00000248187	0.037324	-1.308612	1	1	RP11-184M15.1	_
ENSG00000248489	0.037324	-1.308612	1	1	CTD-2007H13.3	_
ENSG00000248668	0.037324	-1.308612	1	1	OXCT1-AS1	_
ENSG00000248791	0.037324	-1.308612	1	1	CTD-2165H16.3	_
ENSG00000249755	0.037324	-1.308612	1	1	RP11-466G12.2	K06489
ENSG00000249868	0.037324	-1.308612	1	1	RP11-63E5.6	_
ENSG00000251279	0.037324	-1.308612	1	1	CTC-436P18.1	_
ENSG00000253392	0.037324	-1.308612	1	1	AC006277.2	_
ENSG00000255010	0.037324	-1.308612	1	1	RP11-113K21.3	_
ENSG00000258028	0.037324	-1.308612	1	1	RP11-148E17.1	_
ENSG00000258354	0.037324	-1.308612	1	1	MIR3180-1	_
ENSG00000258654	0.037324	-1.308612	1	1	RP11-509A17.3	_
ENSG00000258768	0.037324	-1.308612	1	1	CTD-2292M16.8	_
ENSG00000259071	0.037324	-1.308612	1	1	RP11-247L20.4	_
ENSG00000259726	0.037324	-1.308612	1	1	CSPG4P11	K08115
ENSG00000259728	0.037324	-1.308612	1	1	LINC00933	_
ENSG00000259895	0.037324	-1.308612	1	1	RP11-715J22.2	_
ENSG00000260989	0.037324	-1.308612	1	1	LA16c-395F10.2	_
ENSG00000261105	0.037324	-1.308612	1	1	RP11-173B14.5	_
ENSG00000261267	0.037324	-1.308612	1	1	RP11-44I10.3	_
ENSG00000264425	0.037324	-1.308612	1	1	MIR4653	_
ENSG00000265683	0.037324	-1.308612	1	1	RP11-173M1.5	_
ENSG00000266145	0.037324	-1.308612	1	1	RHOT1P1	K07870
ENSG00000266313	0.037324	-1.308612	1	1	RP11-173M1.4	_
ENSG00000267498	0.037324	-1.308612	1	1	CTB-32O4.2	_
ENSG00000268593	0.037324	-1.308612	1	1	CTD-2611O12.6	_
ENSG00000269894	0.037324	-1.308612	1	1	RP11-1020A11.1	_
ENSG00000269967	0.037324	-1.308612	1	1	RP11-84A19.4	_
ENSG00000270228	0.037324	-1.308612	1	1	RP11-453E17.3	K19759
ENSG00000271551	0.037324	-1.308612	1	1	RP11-230C9.2	_
ENSG00000271815	0.037324	-1.308612	1	1	CTD-2235C13.3	_
ENSG00000271825	0.037324	-1.308612	1	1	RP11-337N6.2	_
ENSG00000272017	0.037324	-1.308612	1	1	RP1-199J3.7	_
ENSG00000272905	0.037324	-1.308612	1	1	RP11-265E18.1	_
ENSG00000273254	0.037324	-1.308612	1	1	RP1-100J12.1	_
ENSG00000273456	0.037324	-1.308612	1	1	RP11-686O6.2	_
ENSG00000085224	-0.036311	1.872531	1	1	ATRX	K10779

ENSG00000017621	0.035895	-1.82353	1	1	MAGIX	K12581
ENSG00000039987	0.035895	-1.82353	1	1	BEST2	K13879
ENSG00000050628	0.035895	-1.82353	1	1	PTGER3	K04260
ENSG00000064201	0.035895	-1.82353	1	1	TSPAN32	K17359
ENSG00000072182	0.035895	-1.82353	1	1	ASIC4	K04831
ENSG00000082074	0.035895	-1.82353	1	1	FYB	K17698
ENSG00000085831	0.035895	-1.82353	1	1	TTC39A	_
ENSG00000086730	0.035895	-1.82353	1	1	LAT2	_
ENSG00000091137	0.035895	-1.82353	1	1	SLC26A4	K14702
ENSG00000091536	0.035895	-1.82353	1	1	MYO15A	K10361
ENSG00000092421	0.035895	-1.82353	1	1	SEMA6A	K06842
ENSG00000099960	0.035895	-1.82353	1	1	SLC7A4	K13866
ENSG00000100276	0.035895	-1.82353	1	1	RASL10A	K07850
ENSG00000100739	0.035895	-1.82353	1	1	BDKRB1	K03915
ENSG00000101440	0.035895	-1.82353	1	1	ASIP	K08725
ENSG00000101850	0.035895	-1.82353	1	1	GPR143	K08470
ENSG00000102218	0.035895	-1.82353	1	1	RP2	K18272
ENSG00000105251	0.035895	-1.82353	1	1	SHD	_
ENSG00000106384	0.035895	-1.82353	1	1	MOGAT3	K14456
ENSG00000106624	0.035895	-1.82353	1	1	AEBP1	_
ENSG00000109072	0.035895	-1.82353	1	1	VTN	K06251
ENSG00000111215	0.035895	-1.82353	1	1	PRR4	K08474
ENSG00000111339	0.035895	-1.82353	1	1	ART4	K06717
ENSG00000111644	0.035895	-1.82353	1	1	ACRBP	_
ENSG00000118307	0.035895	-1.82353	1	1	CASC1	K17580
ENSG00000122986	0.035895	-1.82353	1	1	HVCN1	_
ENSG00000125462	0.035895	-1.82353	1	1	C1orf61	_
ENSG00000128218	0.035895	-1.82353	1	1	VPREB3	K06553
ENSG00000130287	0.035895	-1.82353	1	1	NCAN	K06794
ENSG00000133067	0.035895	-1.82353	1	1	LGR6	K08399
ENSG00000134258	0.035895	-1.82353	1	1	VTCN1	K06747
ENSG00000135218	0.035895	-1.82353	1	1	CD36	K06259
ENSG00000136881	0.035895	-1.82353	1	1	BAAT	K00659
ENSG00000137098	0.035895	-1.82353	1	1	SPAG8	_
ENSG00000137976	0.035895	-1.82353	1	1	DNASE2B	K01158
ENSG00000138798	0.035895	-1.82353	1	1	EGF	K04357
ENSG00000141294	0.035895	-1.82353	1	1	LRRC46	_
ENSG00000141579	0.035895	-1.82353	1	1	ZNF750	_
ENSG00000143107	0.035895	-1.82353	1	1	FNDC7	_
ENSG00000143632	0.035895	-1.82353	1	1	ACTA1	K10354
ENSG00000146243	0.035895	-1.82353	1	1	IRAK1BP1	K17776
ENSG00000150076	0.035895	-1.82353	1	1	C10ORF68	_
ENSG00000150768	0.035895	-1.82353	1	1	DLAT	K00627
ENSG00000152495	0.035895	-1.82353	1	1	CAMK4	K05869
ENSG00000157833	0.035895	-1.82353	1	1	GAREML	_
ENSG00000161132	0.035895	-1.82353	1	1	XXbac-B444P24.10	K00318
ENSG00000161281	0.035895	-1.82353	1	1	COX7A1	K02270
ENSG00000161929	0.035895	-1.82353	1	1	SCIMP	_
ENSG00000162399	0.035895	-1.82353	1	1	BSND	K19331
ENSG00000166394	0.035895	-1.82353	1	1	CYB5R2	K00326
ENSG00000167077	0.035895	-1.82353	1	1	MEI1	_
ENSG00000167244	0.035895	-1.82353	1	1	IGF2	K13769
ENSG00000170092	0.035895	-1.82353	1	1	AC018720.10	K08694
ENSG00000170476	0.035895	-1.82353	1	1	MZB1	_
ENSG00000172650	0.035895	-1.82353	1	1	AGAP5	K12491
ENSG00000173193	0.035895	-1.82353	1	1	PARP14	K15261
ENSG00000173662	0.035895	-1.82353	1	1	TAS1R1	K04624
ENSG00000173867	0.035895	-1.82353	1	1	RP11-97O12.7	_

ENSG00000174196	0.035895	-1.82353	1	1	FAM21D	K18462
ENSG00000174225	0.035895	-1.82353	1	1	ARL13A	K07961
ENSG00000174837	0.035895	-1.82353	1	1	EMR1	K04591
ENSG00000176428	0.035895	-1.82353	1	1	VPS37D	K12185
ENSG00000177363	0.035895	-1.82353	1	1	LRRN4CL	K17985
ENSG00000177757	0.035895	-1.82353	1	1	FAM87B	_
ENSG00000178440	0.035895	-1.82353	1	1	LINC00843	_
ENSG00000178715	0.035895	-1.82353	1	1	RP11-169K16.8	K02960
ENSG00000179066	0.035895	-1.82353	1	1	AC020907.1	_
ENSG00000179136	0.035895	-1.82353	1	1	LINC00670	_
ENSG00000179909	0.035895	-1.82353	1	1	ZNF154	K09228
ENSG00000180422	0.035895	-1.82353	1	1	LINC00304	_
ENSG00000180638	0.035895	-1.82353	1	1	SLC47A2	K03327
ENSG00000182909	0.035895	-1.82353	1	1	LENG9	_
ENSG00000183307	0.035895	-1.82353	1	1	CECR6	_
ENSG00000183423	0.035895	-1.82353	1	1	LRIT3	K09537
ENSG00000184319	0.035895	-1.82353	1	1	RPL23AP82	K02893
ENSG00000184368	0.035895	-1.82353	1	1	MAP7D2	_
ENSG00000184619	0.035895	-1.82353	1	1	KRBA2	K15696
ENSG00000185988	0.035895	-1.82353	1	1	PLK5	K19596
ENSG00000186645	0.035895	-1.82353	1	1	SPDYE9P	K08694
ENSG00000186676	0.035895	-1.82353	1	1	EEF1GP1	K03233
ENSG00000186952	0.035895	-1.82353	1	1	TMEM232	_
ENSG00000188051	0.035895	-1.82353	1	1	TMEM221	_
ENSG00000188338	0.035895	-1.82353	1	1	SLC38A3	_
ENSG00000188611	0.035895	-1.82353	1	1	ASAH2	K12349
ENSG00000188992	0.035895	-1.82353	1	1	LIPI	K19404
ENSG00000189157	0.035895	-1.82353	1	1	FAM47E	K08857
ENSG00000189233	0.035895	-1.82353	1	1	NUGGC	K11447
ENSG00000196963	0.035895	-1.82353	1	1	PCDHB16	K16494
ENSG00000197808	0.035895	-1.82353	1	1	ZNF461	K09228
ENSG00000198553	0.035895	-1.82353	1	1	KCNRG	_
ENSG00000199787	0.035895	-1.82353	1	1	SNORA42	_
ENSG00000199836	0.035895	-1.82353	1	1	RNU1-47P	_
ENSG00000199906	0.035895	-1.82353	1	1	RNU5B-2P	_
ENSG00000200419	0.035895	-1.82353	1	1	Y_RNA	_
ENSG00000200488	0.035895	-1.82353	1	1	RN7SKP203	_
ENSG00000200693	0.035895	-1.82353	1	1	U3	_
ENSG00000202259	0.035895	-1.82353	1	1	RNU6-1318P	_
ENSG00000202399	0.035895	-1.82353	1	1	Y_RNA	_
ENSG00000203602	0.035895	-1.82353	1	1	AL137059.1	K09191
ENSG00000203799	0.035895	-1.82353	1	1	CCDC162P	_
ENSG00000203804	0.035895	-1.82353	1	1	ADAMTSL4-AS1	_
ENSG00000203811	0.035895	-1.82353	1	1	HIST2H3C	K11253
ENSG00000204876	0.035895	-1.82353	1	1	AC021218.2	_
ENSG00000205281	0.035895	-1.82353	1	1	GOLGA6L10	K10595
ENSG00000205325	0.035895	-1.82353	1	1	AC005863.1	_
ENSG00000205663	0.035895	-1.82353	1	1	RP11-706O15.5	_
ENSG00000205794	0.035895	-1.82353	1	1	RP11-333E13.4	K17360
ENSG00000206601	0.035895	-1.82353	1	1	RNU6-431P	_
ENSG00000207563	0.035895	-1.82353	1	1	MIR23B	_
ENSG00000207575	0.035895	-1.82353	1	1	MIR649	_
ENSG00000211961	0.035895	-1.82353	1	1	IGHV1-45	K06856
ENSG00000212588	0.035895	-1.82353	1	1	SNORA26	_
ENSG00000213061	0.035895	-1.82353	1	1	PFN1P11	K05759
ENSG00000213557	0.035895	-1.82353	1	1	RP11-240E2.2	K02910
ENSG00000213717	0.035895	-1.82353	1	1	AC004692.5	K02882
ENSG00000214239	0.035895	-1.82353	1	1	AL591025.1	_

ENSG00000215221	0.035895	-1.82353	1	1	UBA52P6	K02927
ENSG00000215749	0.035895	-1.82353	1	1	GOLGA6L18	K10595
ENSG00000221571	0.035895	-1.82353	1	1	RNU6ATAC35P	_
ENSG00000222345	0.035895	-1.82353	1	1	SNORD19	_
ENSG00000223473	0.035895	-1.82353	1	1	GS1-124K5.3	_
ENSG00000223652	0.035895	-1.82353	1	1	AC106786.1	_
ENSG00000223695	0.035895	-1.82353	1	1	RP4-633O19__A.1	_
ENSG00000224294	0.035895	-1.82353	1	1	RP3-326I13.1	_
ENSG00000224462	0.035895	-1.82353	1	1	C4BPAP1	K04002
ENSG00000224646	0.035895	-1.82353	1	1	AC007387.2	K02979
ENSG00000224712	0.035895	-1.82353	1	1	NPIPA3	K01896
ENSG00000224722	0.035895	-1.82353	1	1	PRNCR1	K13171
ENSG00000225273	0.035895	-1.82353	1	1	UBE2Q2P2	K10582
ENSG00000225526	0.035895	-1.82353	1	1	C3orf83	K09228
ENSG00000225762	0.035895	-1.82353	1	1	RP11-511I2.2	_
ENSG00000225833	0.035895	-1.82353	1	1	GS1-594A7.3	_
ENSG00000225981	0.035895	-1.82353	1	1	AC102953.4	_
ENSG00000226432	0.035895	-1.82353	1	1	CTD-2015B23.2	_
ENSG00000226445	0.035895	-1.82353	1	1	XXyac-YX65C7_A.:	_
ENSG00000226496	0.035895	-1.82353	1	1	LINC00323	_
ENSG00000226609	0.035895	-1.82353	1	1	RP11-276E15.4	_
ENSG00000226647	0.035895	-1.82353	1	1	RP11-336A10.2	_
ENSG00000226756	0.035895	-1.82353	1	1	AC007365.3	_
ENSG00000226981	0.035895	-1.82353	1	1	ABHD17AP6	_
ENSG00000227359	0.035895	-1.82353	1	1	AC017074.2	_
ENSG00000227740	0.035895	-1.82353	1	1	RP11-318C24.2	_
ENSG00000227954	0.035895	-1.82353	1	1	RP3-323P13.2	_
ENSG00000228007	0.035895	-1.82353	1	1	AC011933.2	K02929
ENSG00000228049	0.035895	-1.82353	1	1	POLR2J2	K03008
ENSG00000228050	0.035895	-1.82353	1	1	TOP3BP1	K03165
ENSG00000228328	0.035895	-1.82353	1	1	RP11-516A11.1	K00927
ENSG00000228352	0.035895	-1.82353	1	1	RP11-537H15.3	_
ENSG00000228496	0.035895	-1.82353	1	1	AC106875.1	_
ENSG00000228653	0.035895	-1.82353	1	1	HNRNPCP7	K12884
ENSG00000228695	0.035895	-1.82353	1	1	CES1P1	K01044
ENSG00000229111	0.035895	-1.82353	1	1	MED4-AS1	_
ENSG00000230102	0.035895	-1.82353	1	1	RP11-407B7.1	_
ENSG00000230146	0.035895	-1.82353	1	1	SEPHS1P4	K01008
ENSG00000230568	0.035895	-1.82353	1	1	SF3A3P1	K12827
ENSG00000230731	0.035895	-1.82353	1	1	RP11-478K15.6	_
ENSG00000230982	0.035895	-1.82353	1	1	DSTNP1	K10363
ENSG00000231296	0.035895	-1.82353	1	1	RP1-39G22.4	K07179
ENSG00000231409	0.035895	-1.82353	1	1	RP11-83J16.1	K02929
ENSG00000231527	0.035895	-1.82353	1	1	RP11-374M1.2	_
ENSG00000231579	0.035895	-1.82353	1	1	RPL7P21	_
ENSG00000231616	0.035895	-1.82353	1	1	RP11-575L7.4	_
ENSG00000231793	0.035895	-1.82353	1	1	DOC2GP	K18665
ENSG00000231878	0.035895	-1.82353	1	1	SNRPFP1	K11098
ENSG00000231927	0.035895	-1.82353	1	1	AC102953.6	_
ENSG00000232028	0.035895	-1.82353	1	1	AC007391.2	_
ENSG00000232065	0.035895	-1.82353	1	1	LINC01063	_
ENSG00000232385	0.035895	-1.82353	1	1	RPS3AP25	K02984
ENSG00000232412	0.035895	-1.82353	1	1	RP1-315G1.3	_
ENSG00000232564	0.035895	-1.82353	1	1	RP4-591N18.2	_
ENSG00000232656	0.035895	-1.82353	1	1	IDI2-AS1	_
ENSG00000232912	0.035895	-1.82353	1	1	RP5-1115A15.1	_
ENSG00000233360	0.035895	-1.82353	1	1	Z83844.1	_
ENSG00000233367	0.035895	-1.82353	1	1	RP11-307L3.4	_

ENSG00000233404	0.035895	-1.82353	1	1	FLJ20373	_
ENSG00000233406	0.035895	-1.82353	1	1	RP11-296A18.5	K02981
ENSG00000233806	0.035895	-1.82353	1	1	AC131097.3	_
ENSG00000233846	0.035895	-1.82353	1	1	RP11-307L3.2	_
ENSG00000234118	0.035895	-1.82353	1	1	RPL13AP6	K02872
ENSG00000234338	0.035895	-1.82353	1	1	RP11-797H7.1	_
ENSG00000234478	0.035895	-1.82353	1	1	RP11-275I14.4	_
ENSG00000234500	0.035895	-1.82353	1	1	GS1-124K5.10	K03121
ENSG00000234509	0.035895	-1.82353	1	1	AP000253.1	_
ENSG00000234546	0.035895	-1.82353	1	1	RP3-510D11.2	_
ENSG00000234871	0.035895	-1.82353	1	1	LINC01032	_
ENSG00000234913	0.035895	-1.82353	1	1	XXbac-B476C20.13	_
ENSG00000235172	0.035895	-1.82353	1	1	CTB-114C7.3	_
ENSG00000235214	0.035895	-1.82353	1	1	FAM83C-AS1	_
ENSG00000235554	0.035895	-1.82353	1	1	AC005822.1	K12893
ENSG00000235586	0.035895	-1.82353	1	1	AC011247.3	_
ENSG00000235698	0.035895	-1.82353	1	1	PA2G4P2	_
ENSG00000236018	0.035895	-1.82353	1	1	RP4-814D15.1	K14012
ENSG00000236213	0.035895	-1.82353	1	1	AC006369.2	_
ENSG00000236425	0.035895	-1.82353	1	1	AC090018.1	K02910
ENSG00000236654	0.035895	-1.82353	1	1	AC079780.3	_
ENSG00000236699	0.035895	-1.82353	1	1	ARHGEF38	_
ENSG00000236761	0.035895	-1.82353	1	1	CTAGE9	_
ENSG00000236867	0.035895	-1.82353	1	1	RP3-522J7.5	K04695
ENSG00000237292	0.035895	-1.82353	1	1	RP11-540K16.1	_
ENSG00000237489	0.035895	-1.82353	1	1	LINC00959	_
ENSG00000237737	0.035895	-1.82353	1	1	DCTN1-AS1	_
ENSG00000237786	0.035895	-1.82353	1	1	GFOD1-AS1	_
ENSG00000237916	0.035895	-1.82353	1	1	RP11-537E18.1	K02922
ENSG00000238061	0.035895	-1.82353	1	1	RP4-635A23.4	K03038
ENSG00000238269	0.035895	-1.82353	1	1	PAGE2B	_
ENSG00000238390	0.035895	-1.82353	1	1	SNORA81	_
ENSG00000238406	0.035895	-1.82353	1	1	RNU7-171P	_
ENSG00000238821	0.035895	-1.82353	1	1	snoU13	_
ENSG00000240509	0.035895	-1.82353	1	1	RPL34P18	K02915
ENSG00000240898	0.035895	-1.82353	1	1	RP11-48B14.1	K02889
ENSG00000241146	0.035895	-1.82353	1	1	RPL7P41	K02937
ENSG00000241544	0.035895	-1.82353	1	1	RP11-6F2.5	_
ENSG00000242837	0.035895	-1.82353	1	1	RPL21P13	K02889
ENSG00000243071	0.035895	-1.82353	1	1	RP11-613M5.2	K02889
ENSG00000243709	0.035895	-1.82353	1	1	LEFTY1	K04668
ENSG00000243738	0.035895	-1.82353	1	1	RN7SL181P	_
ENSG00000244073	0.035895	-1.82353	1	1	RPS4XP6	K02987
ENSG00000245750	0.035895	-1.82353	1	1	RP11-279F6.1	_
ENSG00000246130	0.035895	-1.82353	1	1	RP11-875O11.2	_
ENSG00000246145	0.035895	-1.82353	1	1	RP11-346I3.4	_
ENSG00000246851	0.035895	-1.82353	1	1	RP11-544A12.8	_
ENSG00000247011	0.035895	-1.82353	1	1	RP11-700H6.1	_
ENSG00000247877	0.035895	-1.82353	1	1	CTD-2001C12.1	_
ENSG00000248751	0.035895	-1.82353	1	1	RP1-130H16.18	_
ENSG00000248773	0.035895	-1.82353	1	1	RP11-231L11.3	_
ENSG00000249476	0.035895	-1.82353	1	1	CTD-2587M2.1	_
ENSG00000249679	0.035895	-1.82353	1	1	RP11-279O9.4	_
ENSG00000250240	0.035895	-1.82353	1	1	CTD-2154I11.2	_
ENSG00000250551	0.035895	-1.82353	1	1	RP11-254I22.1	_
ENSG00000250722	0.035895	-1.82353	1	1	SEPP1	_
ENSG00000250909	0.035895	-1.82353	1	1	RP11-843P14.2	_
ENSG00000251175	0.035895	-1.82353	1	1	RP11-45L9.1	_

ENSG00000251303	0.035895	-1.82353	1	1	CTD-2003D5.1	K08272
ENSG00000251666	0.035895	-1.82353	1	1	ZNF346-IT1	_
ENSG00000251733	0.035895	-1.82353	1	1	SCARNA8	_
ENSG00000251957	0.035895	-1.82353	1	1	RNU6-1095P	_
ENSG00000253167	0.035895	-1.82353	1	1	KIAA0196-AS1	_
ENSG00000253438	0.035895	-1.82353	1	1	PCAT1	_
ENSG00000253541	0.035895	-1.82353	1	1	SEPT10P1	K16940
ENSG00000253633	0.035895	-1.82353	1	1	KB-1980E6.3	_
ENSG00000253921	0.035895	-1.82353	1	1	CTB-113P19.3	_
ENSG00000253944	0.035895	-1.82353	1	1	RP11-156K13.1	_
ENSG00000254016	0.035895	-1.82353	1	1	ALG1L10P	K03842
ENSG00000254363	0.035895	-1.82353	1	1	CTB-131B5.5	_
ENSG00000254388	0.035895	-1.82353	1	1	DUTP2	_
ENSG00000254732	0.035895	-1.82353	1	1	RP11-691N7.6	K05690
ENSG00000254759	0.035895	-1.82353	1	1	NAP1L1P1	K11279
ENSG00000254788	0.035895	-1.82353	1	1	CKLF-CMTM1	_
ENSG00000254905	0.035895	-1.82353	1	1	RP11-712L6.7	_
ENSG00000254907	0.035895	-1.82353	1	1	RP11-484D2.2	_
ENSG00000255045	0.035895	-1.82353	1	1	RP11-677M14.2	_
ENSG00000255440	0.035895	-1.82353	1	1	RP11-632K5.2	_
ENSG00000255710	0.035895	-1.82353	1	1	RP11-667M19.9	K18329
ENSG00000255974	0.035895	-1.82353	1	1	CYP2A6	K17683
ENSG00000256097	0.035895	-1.82353	1	1	LINC00696	_
ENSG00000256481	0.035895	-1.82353	1	1	RP11-21A7A.4	_
ENSG00000256604	0.035895	-1.82353	1	1	AC093668.2	_
ENSG00000256616	0.035895	-1.82353	1	1	RP11-815J4.6	K08462
ENSG00000256843	0.035895	-1.82353	1	1	RP11-467L13.4	K14965
ENSG00000256968	0.035895	-1.82353	1	1	SNRPEP2	K11097
ENSG00000257327	0.035895	-1.82353	1	1	RP11-650K20.3	_
ENSG00000257475	0.035895	-1.82353	1	1	RP11-983P16.2	_
ENSG00000257595	0.035895	-1.82353	1	1	RP3-473L9.4	_
ENSG00000257957	0.035895	-1.82353	1	1	QRSL1P3	K02433
ENSG00000258099	0.035895	-1.82353	1	1	RP11-686G8.2	_
ENSG00000258285	0.035895	-1.82353	1	1	RP11-103B5.2	_
ENSG00000258711	0.035895	-1.82353	1	1	RP11-218E20.3	_
ENSG00000258725	0.035895	-1.82353	1	1	PRC1-AS1	_
ENSG00000258813	0.035895	-1.82353	1	1	RP11-109N23.4	_
ENSG00000259091	0.035895	-1.82353	1	1	LINC00517	_
ENSG00000259158	0.035895	-1.82353	1	1	ADAM20P1	_
ENSG00000259251	0.035895	-1.82353	1	1	RP11-643M14.1	_
ENSG00000259318	0.035895	-1.82353	1	1	RP11-454L9.2	K11299
ENSG00000259408	0.035895	-1.82353	1	1	RP11-3D4.3	_
ENSG00000259516	0.035895	-1.82353	1	1	ANP32AP1	K18646
ENSG00000259595	0.035895	-1.82353	1	1	RP11-516C1.1	_
ENSG00000259894	0.035895	-1.82353	1	1	RP11-697E2.4	_
ENSG00000260144	0.035895	-1.82353	1	1	RP11-361M10.3	K10595
ENSG00000260145	0.035895	-1.82353	1	1	RP11-322D14.2	_
ENSG00000260170	0.035895	-1.82353	1	1	RP11-96O20.4	K17218
ENSG00000260284	0.035895	-1.82353	1	1	TPSP2	_
ENSG00000260342	0.035895	-1.82353	1	1	RP11-1035H13.3	K02957
ENSG00000260352	0.035895	-1.82353	1	1	CTD-2288F12.1	_
ENSG00000260372	0.035895	-1.82353	1	1	AQP4-AS1	_
ENSG00000260382	0.035895	-1.82353	1	1	RP11-540B6.2	_
ENSG00000260571	0.035895	-1.82353	1	1	RP11-546B15.2	K15464
ENSG00000260773	0.035895	-1.82353	1	1	RP11-352G18.2	_
ENSG00000260792	0.035895	-1.82353	1	1	RP11-982M15.8	_
ENSG00000260855	0.035895	-1.82353	1	1	RP11-439E19.10	_
ENSG00000260955	0.035895	-1.82353	1	1	RP11-30L15.6	_

ENSG00000261156	0.035895	-1.82353	1	1	RP11-215E13.1	_
ENSG00000261430	0.035895	-1.82353	1	1	LA16c-390E6.3	_
ENSG00000261596	0.035895	-1.82353	1	1	CTB-31N19.3	_
ENSG00000261613	0.035895	-1.82353	1	1	RP11-20I23.13	_
ENSG00000261668	0.035895	-1.82353	1	1	RP11-50D9.3	_
ENSG00000261915	0.035895	-1.82353	1	1	RP11-542C16.2	K15307
ENSG00000261924	0.035895	-1.82353	1	1	CTD-2561B21.5	_
ENSG00000262663	0.035895	-1.82353	1	1	RP11-497H17.1	_
ENSG00000262833	0.035895	-1.82353	1	1	RP11-28G8.1	_
ENSG00000263307	0.035895	-1.82353	1	1	RP11-166B2.8	_
ENSG00000263432	0.035895	-1.82353	1	1	RN7SL689P	_
ENSG00000263463	0.035895	-1.82353	1	1	MIR378I	_
ENSG00000263612	0.035895	-1.82353	1	1	AF186192.6	_
ENSG00000264177	0.035895	-1.82353	1	1	RP1-37N7.1	_
ENSG00000264187	0.035895	-1.82353	1	1	RP11-45M22.4	K09594
ENSG00000264635	0.035895	-1.82353	1	1	RP11-769O8.3	_
ENSG00000264668	0.035895	-1.82353	1	1	ZFP41	K09228
ENSG00000264952	0.035895	-1.82353	1	1	AC004000.1	_
ENSG00000265106	0.035895	-1.82353	1	1	Z93241.1	_
ENSG00000265293	0.035895	-1.82353	1	1	ARGFXP2	_
ENSG00000265490	0.035895	-1.82353	1	1	RP11-806L2.6	_
ENSG00000266124	0.035895	-1.82353	1	1	MIR5587	_
ENSG00000266127	0.035895	-1.82353	1	1	RP11-474N24.2	K09228
ENSG00000266601	0.035895	-1.82353	1	1	RP11-6N17.3	_
ENSG00000266905	0.035895	-1.82353	1	1	RP11-1058N17.1	_
ENSG00000267018	0.035895	-1.82353	1	1	AC010525.7	_
ENSG00000267200	0.035895	-1.82353	1	1	MIR132	_
ENSG00000267312	0.035895	-1.82353	1	1	RP11-1094M14.7	K01939
ENSG00000267454	0.035895	-1.82353	1	1	ZNF582-AS1	_
ENSG00000267757	0.035895	-1.82353	1	1	C19orf83	K18595
ENSG00000267905	0.035895	-1.82353	1	1	CTD-2616J11.16	_
ENSG00000268564	0.035895	-1.82353	1	1	AC003956.1	_
ENSG00000268756	0.035895	-1.82353	1	1	AC104534.2	_
ENSG00000269482	0.035895	-1.82353	1	1	Z69720.2	_
ENSG00000269720	0.035895	-1.82353	1	1	CTD-2521M24.5	_
ENSG00000269794	0.035895	-1.82353	1	1	AC010642.2	K09228
ENSG00000270061	0.035895	-1.82353	1	1	RP11-214K3.19	_
ENSG00000270097	0.035895	-1.82353	1	1	LA16c-380H5.3	_
ENSG00000270264	0.035895	-1.82353	1	1	NDUFB8P2	K03964
ENSG00000270760	0.035895	-1.82353	1	1	AD001527.7	_
ENSG00000271009	0.035895	-1.82353	1	1	RP11-346C20.3	_
ENSG00000271259	0.035895	-1.82353	1	1	RP11-1109F11.5	_
ENSG00000271308	0.035895	-1.82353	1	1	AP003900.6	_
ENSG00000271590	0.035895	-1.82353	1	1	RP11-181E10.3	_
ENSG00000271963	0.035895	-1.82353	1	1	CTD-2140B24.6	_
ENSG00000272043	0.035895	-1.82353	1	1	RP11-44N11.3	_
ENSG00000272182	0.035895	-1.82353	1	1	RP11-802O23.3	_
ENSG00000272426	0.035895	-1.82353	1	1	RP11-108M9.6	_
ENSG00000272442	0.035895	-1.82353	1	1	RP11-444E17.6	K01872
ENSG00000272444	0.035895	-1.82353	1	1	RP11-1017G21.6	_
ENSG00000272459	0.035895	-1.82353	1	1	RP11-1277A3.3	_
ENSG00000272465	0.035895	-1.82353	1	1	RP1-136B1.1	_
ENSG00000272588	0.035895	-1.82353	1	1	RP11-440L14.4	_
ENSG00000272695	0.035895	-1.82353	1	1	GAS6-AS2	_
ENSG00000272862	0.035895	-1.82353	1	1	RP11-814H16.2	_
ENSG00000272914	0.035895	-1.82353	1	1	RP11-330O11.3	_
ENSG00000272916	0.035895	-1.82353	1	1	RP11-574K11.31	K02577
ENSG00000273006	0.035895	-1.82353	1	1	RP11-314C9.2	_

ENSG00000273032	0.035895	-1.82353	1	1	DGCR9	_
ENSG00000273056	0.035895	-1.82353	1	1	RP11-77E14.2	_
ENSG00000273176	0.035895	-1.82353	1	1	RP3-510H16.3	_
ENSG00000273403	0.035895	-1.82353	1	1	RP11-329B9.3	_
ENSG00000273416	0.035895	-1.82353	1	1	RP4-597N16.4	_
ENSG00000273424	0.035895	-1.82353	1	1	CTA-223H9.9	_
ENSG00000273442	0.035895	-1.82353	1	1	AC006946.17	_
ENSG00000213443	-0.035054	2.295208	1	1	RP11-75L1.2	K11276
ENSG00000138696	-0.034319	2.621676	1	1	BMPR1B	K13578
ENSG00000214222	-0.033209	2.330449	1	1	TUBBP2	K07375
ENSG00000260261	-0.031432	1.965101	1	1	RP11-480A16.1	_
ENSG00000227403	-0.03139	1.410107	1	1	AC009299.3	_
ENSG00000184163	-0.031264	0.495923	1	1	FAM132A	_
ENSG00000230373	-0.031264	0.495923	1	1	GOLGA6L5P	K10595
ENSG00000246203	-0.031264	0.495923	1	1	RP11-29H23.5	_
ENSG00000250934	-0.031264	0.495923	1	1	RP11-71E19.1	_
ENSG00000172687	-0.028827	2.731249	1	1	ZNF738	K09228
ENSG00000161048	-0.027581	2.757392	1	1	NAPEPLD	K13985
ENSG00000247092	0.027432	2.972415	1	1	SNHG10	_
ENSG00000113763	-0.027182	3.02319	1	1	UNC5A	K07521
ENSG00000162419	-0.025227	3.065667	1	1	GMEB1	_
ENSG00000139192	-0.025165	1.536137	1	1	TAPBPL	K08510
ENSG00000187800	-0.025165	1.536137	1	1	PEAR1	_
ENSG00000226539	-0.025165	1.536137	1	1	AC012512.1	K09113
ENSG00000070731	-0.025073	0.614885	1	1	ST6GALNAC2	K06616
ENSG00000136859	-0.025073	0.614885	1	1	ANGPTL2	_
ENSG00000171956	-0.025073	0.614885	1	1	FOXB1	K09395
ENSG00000249492	-0.025073	0.614885	1	1	RP11-159F24.3	_
ENSG00000257511	-0.025073	0.614885	1	1	RP11-278C7.1	K11279
ENSG00000258301	-0.025073	0.614885	1	1	RP11-488C13.5	_
ENSG00000267698	-0.025073	0.614885	1	1	AC002116.7	_
ENSG00000273281	-0.025073	0.614885	1	1	RP11-339B21.14	_
ENSG00000105088	0.023564	2.561584	1	1	OLFM2	_
ENSG00000158315	0.023564	2.561584	1	1	RHBDL2	K02857
ENSG00000137841	-0.023023	2.857482	1	1	PLCB2	K05858
ENSG00000078114	-0.022488	2.555675	1	1	NEBL	_
ENSG00000137070	-0.022449	1.595261	1	1	IL11RA	K05056
ENSG00000172986	-0.022449	1.595261	1	1	GXYLT2	K13676
ENSG00000135540	0.021342	2.371375	1	1	NHSL1	_
ENSG00000149380	-0.02097	2.905043	1	1	P4HA3	K00472
ENSG00000204959	0.01997	2.265969	1	1	ARHGEF34P	_
ENSG00000121769	-0.019887	0.724805	1	1	FABP3	K08752
ENSG00000130783	-0.019887	0.724805	1	1	CCDC62	_
ENSG00000163295	-0.019887	0.724805	1	1	ALPI	K01077
ENSG00000164406	-0.019887	0.724805	1	1	LEAP2	K04135
ENSG00000197927	-0.019887	0.724805	1	1	C2orf27A	_
ENSG00000203797	-0.019887	0.724805	1	1	DDO	K00272
ENSG00000225422	-0.019887	0.724805	1	1	RBMS1P1	_
ENSG00000258713	-0.019887	0.724805	1	1	C20orf141	_
ENSG00000080823	-0.018812	2.642408	1	1	MOK	K08830
ENSG00000151917	-0.018812	2.642408	1	1	BEND6	_
ENSG00000261652	0.018387	3.126198	1	1	C15orf65	_
ENSG00000144445	-0.018013	3.453568	1	1	KANSL1L	_
ENSG00000169750	-0.018013	3.453568	1	1	RAC3	K07861
ENSG00000105649	-0.017654	1.706721	1	1	RAB3A	K07882
ENSG00000145687	-0.017654	1.706721	1	1	SSBP2	_
ENSG00000136895	0.017135	2.071157	1	1	GARNL3	_
ENSG00000149489	0.017135	2.071157	1	1	ROM1	K17344

ENSG00000187240	-0.016945	2.285535	1	1	DYNC2H1	K10414
ENSG00000157445	0.015775	3.530923	1	1	CACNA2D3	K04860
ENSG00000089356	0.015747	1.985229	1	1	FXYD3	K13361
ENSG00000183621	-0.015551	2.72423	1	1	ZNF438	_
ENSG00000110876	-0.015527	1.759388	1	1	SELPLG	K06544
ENSG00000163633	-0.015527	1.759388	1	1	C4orf36	K00543
ENSG00000240498	-0.015527	1.759388	1	1	CDKN2B-AS1	_
ENSG00000249740	-0.015527	1.759388	1	1	CTD-2127H9.1	_
ENSG00000226210	-0.015478	0.826961	1	1	ABC7-42389800N1	K18461
ENSG00000197763	0.014604	2.886411	1	1	TXNRD3	K00384
ENSG00000166263	-0.014545	2.750507	1	1	STXBP4	K15302
ENSG00000163788	0.0142	3.846617	1	1	SNRK	K08802
ENSG00000215908	0.013019	3.370613	1	1	CROCCP2	_
ENSG00000005189	-0.012639	2.801669	1	1	AC004381.6	K14570
ENSG00000007944	0.012381	2.762694	1	1	MYLIP	K10637
ENSG00000162927	0.012381	2.762694	1	1	PUS10	K07583
ENSG00000197580	0.012363	1.796332	1	1	BCO2	K10252
ENSG00000229358	0.012363	1.796332	1	1	DPY19L1P1	K09654
ENSG00000154319	0.011887	2.736626	1	1	FAM167A	_
ENSG00000181404	-0.01172	1.859296	1	1	XXyac-YRM2039.2	K18461
ENSG00000168427	-0.011686	0.922376	1	1	KLHL30	K10466
ENSG00000196605	-0.011686	0.922376	1	1	ZNF846	K09228
ENSG00000235423	-0.011686	0.922376	1	1	RP11-282O18.3	_
ENSG00000240376	-0.011686	0.922376	1	1	RP11-36C20.1	K02995
ENSG00000260552	0.011356	1.74498	1	1	RP11-49I11.1	_
ENSG00000270346	0.011356	1.74498	1	1	RP1-90J20.12	_
ENSG00000198205	0.010664	3.246766	1	1	ZXDA	_
ENSG00000050344	-0.010359	3.181049	1	1	NFE2L3	K09040
ENSG00000153933	-0.01003	3.859062	1	1	DGKE	K00901
ENSG00000166321	-0.010009	1.906775	1	1	NUDT13	K03426
ENSG00000216866	-0.010009	1.906775	1	1	RPS2P55	K02981
ENSG00000126091	-0.009697	3.200248	1	1	ST3GAL3	K00781
ENSG00000175898	-0.009473	2.486472	1	1	CTD-2369P2.2	_
ENSG00000179859	-0.009473	2.486472	1	1	AC025335.1	_
ENSG00000242498	0.009117	3.579531	1	1	C15orf38	K09537
ENSG00000142082	0.008914	4.14819	1	1	SIRT3	K11413
ENSG00000029639	-0.008775	4.064345	1	1	TFB1M	K15266
ENSG00000266469	-0.008428	4.074789	1	1	CTB-131K11.1	_
ENSG00000198795	-0.008424	3.237897	1	1	ZNF521	_
ENSG00000170899	-0.008388	1.011885	1	1	GSTA4	K00799
ENSG00000203279	-0.008388	1.011885	1	1	RP11-498P14.5	_
ENSG00000204362	-0.008388	1.011885	1	1	RP11-380J14.1	_
ENSG00000272365	-0.008388	1.011885	1	1	RP11-389C8.3	_
ENSG00000237550	0.008062	4.108069	1	1	UBE2Q2P6	K02940
ENSG00000139567	0.007818	1.578978	1	1	ACVRL1	K13594
ENSG00000272779	0.007818	1.578978	1	1	LL22NC03-80A10.6	K14569
ENSG00000169031	-0.007812	3.25636	1	1	COL4A3	K06237
ENSG00000238105	0.00739	4.07723	1	1	GOLGA2P5	_
ENSG00000132950	-0.007216	3.27459	1	1	ZMYM5	_
ENSG00000163472	0.006921	3.070133	1	1	TMEM79	K11125
ENSG00000121481	-0.006517	4.766426	1	1	RNF2	K10695
ENSG00000160961	0.006427	1.519118	1	1	ZNF333	K09228
ENSG00000184227	0.006427	1.519118	1	1	ACOT1	K01068
ENSG00000185522	0.006427	1.519118	1	1	C11orf35	_
ENSG00000196912	0.006427	1.519118	1	1	ANKRD36B	K17299
ENSG00000136169	-0.006069	3.310374	1	1	SETDB2	K18494
ENSG00000087258	0.005702	4.0026	1	1	GNAO1	K04534
ENSG00000175691	0.0057	3.424841	1	1	ZNF77	K09228

ENSG00000153233	-0.005494	1.096176	1	1	PTPRR	K04458
ENSG00000169122	-0.005494	1.096176	1	1	FAM110B	_
ENSG00000172943	-0.005494	1.096176	1	1	PHF8	K19415
ENSG00000220201	-0.005494	1.096176	1	1	ZGLP1	_
ENSG00000261167	-0.005494	1.096176	1	1	RP11-517B11.7	_
ENSG00000187257	-0.005133	4.810846	1	1	RSBN1L	_
ENSG00000131374	-0.005067	3.601599	1	1	TBC1D5	_
ENSG00000176182	-0.004977	3.345293	1	1	MYPOP	_
ENSG00000187860	0.004915	2.413737	1	1	CCDC157	_
ENSG00000152284	0.004906	1.456672	1	1	TCF7L1	K04490
ENSG00000184209	-0.004787	4.476496	1	1	SNRNP35	K13155
ENSG00000101363	-0.004668	5.475176	1	1	MANBAL	_
ENSG00000068885	0.004654	3.958156	1	1	IFT80	K19678
ENSG00000076555	-0.004622	2.634915	1	1	ACACB	K01946
ENSG00000136925	0.004516	3.375007	1	1	TSTD2	_
ENSG00000102977	-0.004423	5.112842	1	1	ACD	K11114
ENSG00000139354	0.004332	4.812372	1	1	GAS2L3	K14404
ENSG00000123095	-0.004193	3.630202	1	1	BHLHE41	K03730
ENSG00000231259	-0.004191	3.054648	1	1	AC125232.1	K03348
ENSG00000163888	0.003935	3.668524	1	1	CAMK2N2	K01415
ENSG00000101452	0.003894	4.338008	1	1	DHX35	K13117
ENSG00000119650	0.003742	4.140799	1	1	IFT43	K19675
ENSG00000254470	0.003422	4.638403	1	1	AP5B1	K19022
ENSG00000177728	0.003248	5.305901	1	1	KIAA0195	_
ENSG00000049192	0.003242	2.346409	1	1	ADAMTS6	K08621
ENSG00000116668	0.003242	2.346409	1	1	SWT1	_
ENSG00000259838	0.003235	1.391407	1	1	TCEB1P2	K03872
ENSG00000165660	-0.003218	5.235481	1	1	FAM175B	_
ENSG00000083544	-0.003134	4.771238	1	1	TDRD3	K18404
ENSG00000127220	0.002975	4.996747	1	1	ABHD8	K13701
ENSG00000115661	0.002852	4.464578	1	1	STK16	K08856
ENSG00000204174	0.002649	2.892651	1	1	NPY4R	K04206
ENSG00000103064	-0.002626	4.994012	1	1	SLC7A6	K13872
ENSG00000106588	-0.002587	5.408593	1	1	PSMA2	K13137
ENSG00000109016	-0.002497	5.09113	1	1	DHRS7B	K11166
ENSG00000106268	0.002477	5.083379	1	1	NUDT1	K17816
ENSG00000174276	0.002477	4.090211	1	1	ZNHIT2	_
ENSG00000161692	-0.002468	4.999506	1	1	DBF4B	_
ENSG00000108039	-0.002427	5.615068	1	1	XPNPEP1	K01262
ENSG00000162650	0.002344	3.287928	1	1	ATXN7L2	K11318
ENSG00000164088	0.002344	3.287928	1	1	PPM1M	K17507
ENSG00000165630	0.002344	3.287928	1	1	PRPF18	K12817
ENSG00000072958	-0.002324	5.618642	1	1	AP1M1	K12393
ENSG00000165410	0.002288	6.26469	1	1	CFL2	K05765
ENSG00000156735	0.002035	4.432446	1	1	BAG4	K09558
ENSG00000104973	0.001981	5.777389	1	1	MED25	K15168
ENSG00000197172	-0.001972	5.196543	1	1	MAGEA6	_
ENSG00000166900	-0.001913	5.111569	1	1	STX3	K08486
ENSG00000103051	-0.001897	5.864268	1	1	COG4	_
ENSG00000080802	-0.001769	4.442591	1	1	CNOT4	K10643
ENSG00000115568	0.001718	3.840665	1	1	ZNF142	_
ENSG00000112237	0.001598	6.096067	1	1	CCNC	K15161
ENSG00000188522	0.001505	5.406525	1	1	FAM83G	K14390
ENSG00000161395	0.001395	2.275789	1	1	PGAP3	_
ENSG00000215386	0.001392	1.323056	1	1	LINC00478	_
ENSG00000119446	-0.001125	4.947863	1	1	RBM18	K02838
ENSG00000186222	0.001117	4.037786	1	1	BLOC1S4	K08366
ENSG00000086102	0.001096	6.503341	1	1	NFX1	K12236

ENSG00000134697	-0.001013	6.753881	1	1	GNL2	K14537
ENSG00000186566	-0.000928	5.729422	1	1	GPATCH8	_
ENSG00000267321	-0.000898	4.322785	1	1	RP11-1094M14.11	_
ENSG00000187951	0.000833	4.027069	1	1	ARHGAP11B	K17545
ENSG00000108774	-0.000817	5.902896	1	1	RAB5C	K07889
ENSG00000188647	0.000795	5.731902	1	1	PTAR1	K14137
ENSG00000155975	-0.000792	5.151598	1	1	VPS37A	K12185
ENSG00000176018	0.000779	5.209635	1	1	LYSMD3	_
ENSG00000103356	0.000737	4.382866	1	1	EARS2	K01885
ENSG00000205138	-0.000656	4.331523	1	1	SDHAF1	K18167
ENSG00000168781	-0.000656	3.972256	1	1	PPIP5K1	K13024
ENSG00000228175	-0.000654	2.201541	1	1	GEMIN8P4	K13136
ENSG00000170961	-0.000653	1.251313	1	1	HAS2	K00752
ENSG00000233527	-0.000653	1.251313	1	1	AC092295.7	_
ENSG00000256940	-0.000653	1.251313	1	1	RP11-783K16.5	_
ENSG00000126432	-0.000536	7.910218	1	1	PRDX5	K11187
ENSG00000105518	-0.000468	5.684933	1	1	TMEM205	K07884
ENSG00000203668	0.000452	7.025678	1	1	CHML	K11667
ENSG00000085433	0.000398	4.782422	1	1	WDR47	_
ENSG00000156650	0.000398	3.790854	1	1	KAT6B	K11306
ENSG00000250132	0.000397	2.239142	1	1	RP11-359B12.2	_
ENSG00000141456	-0.000314	7.272558	1	1	PELP1	K16913
ENSG00000123179	0.000251	6.315678	1	1	EBPL	_
ENSG00000174989	0.000251	4.005393	1	1	FBXW8	K10264
ENSG00000205246	0.00015	6.825623	1	1	RPSAP58	K02998
ENSG00000144535	0.00014	4.192119	1	1	DIS3L2	K18758
ENSG00000101413	-0.000122	5.176065	1	1	RPRD1B	K15559
ENSG00000235823	5.31E-05	3.778129	1	1	LINC00263	_
ENSG00000000005	5.13E-15	-2.63127	1	1	TNMD	_
ENSG00000001561	5.13E-15	-2.63127	1	1	ENPP4	K18424
ENSG00000002587	5.13E-15	-2.63127	1	1	HS3ST1	K01024
ENSG00000002726	5.13E-15	-2.63127	1	1	AOC1	K11182
ENSG00000002933	5.13E-15	-2.63127	1	1	TMEM176A	_
ENSG00000004142	5.13E-15	-2.63127	1	1	POLDIP2	K17809
ENSG00000004468	5.13E-15	-2.63127	1	1	CD38	K01242
ENSG00000004848	5.13E-15	-2.63127	1	1	ARX	K09452
ENSG00000004948	5.13E-15	-2.63127	1	1	CALCR	K04576
ENSG00000005108	5.13E-15	-2.63127	1	1	THSD7A	_
ENSG00000005249	5.13E-15	-2.63127	1	1	PRKAR2B	K04739
ENSG00000005379	5.13E-15	-2.63127	1	1	BZRAP1	_
ENSG00000005381	5.13E-15	-2.63127	1	1	MPO	K10789
ENSG00000005471	5.13E-15	-2.63127	1	1	ABCB4	K05659
ENSG00000005513	5.13E-15	-2.63127	1	1	SOX8	K09270
ENSG00000005981	5.13E-15	-2.63127	1	1	ASB4	K10326
ENSG00000006042	5.13E-15	-2.63127	1	1	TMEM98	_
ENSG00000006059	5.13E-15	-2.63127	1	1	KRT33A	K07604
ENSG00000006071	5.13E-15	-2.63127	1	1	ABCC8	K05032
ENSG00000006074	5.13E-15	-2.63127	1	1	CCL18	K05514
ENSG00000006128	5.13E-15	-2.63127	1	1	TAC1	K05239
ENSG00000006377	5.13E-15	-2.63127	1	1	DLX6	K09314
ENSG00000006468	5.13E-15	-2.63127	1	1	ETV1	K09431
ENSG00000006606	5.13E-15	-2.63127	1	1	CCL26	K05514
ENSG00000006659	5.13E-15	-2.63127	1	1	LGALS14	K10097
ENSG00000006747	5.13E-15	-2.63127	1	1	SCIN	K05768
ENSG00000006756	5.13E-15	-2.63127	1	1	ARSD	K12374
ENSG00000006788	5.13E-15	-2.63127	1	1	MYH13	K10352
ENSG00000007001	5.13E-15	-2.63127	1	1	UPP2	K00757
ENSG00000007062	5.13E-15	-2.63127	1	1	PROM1	K06532

ENSG00000007129	5.13E-15	-2.63127	1	1	CEACAM21	_
ENSG00000007171	5.13E-15	-2.63127	1	1	NOS2	K13241
ENSG00000007174	5.13E-15	-2.63127	1	1	DNAH9	K10408
ENSG00000007216	5.13E-15	-2.63127	1	1	SLC13A2	K14445
ENSG00000007306	5.13E-15	-2.63127	1	1	CEACAM7	K06499
ENSG00000007350	5.13E-15	-2.63127	1	1	TKTL1	K00615
ENSG00000007908	5.13E-15	-2.63127	1	1	SELE	K06494
ENSG00000007933	5.13E-15	-2.63127	1	1	FMO3	K00485
ENSG00000008118	5.13E-15	-2.63127	1	1	CAMK1G	K08794
ENSG00000008196	5.13E-15	-2.63127	1	1	TFAP2B	K09176
ENSG00000008197	5.13E-15	-2.63127	1	1	TFAP2D	K09178
ENSG00000008311	5.13E-15	-2.63127	1	1	AASS	K14157
ENSG00000010030	5.13E-15	-2.63127	1	1	ETV7	K03211
ENSG00000010282	5.13E-15	-2.63127	1	1	HHATL	_
ENSG00000010438	5.13E-15	-2.63127	1	1	PRSS3	K01312
ENSG00000010610	5.13E-15	-2.63127	1	1	CD4	K06454
ENSG00000010671	5.13E-15	-2.63127	1	1	BTK	K07370
ENSG00000011052	5.13E-15	-2.63127	1	1	NME2	K00940
ENSG00000011083	5.13E-15	-2.63127	1	1	SLC6A7	K05038
ENSG00000011201	5.13E-15	-2.63127	1	1	KAL1	_
ENSG00000011600	5.13E-15	-2.63127	1	1	TYROBP	K07992
ENSG00000012504	5.13E-15	-2.63127	1	1	NR1H4	K08537
ENSG00000012779	5.13E-15	-2.63127	1	1	ALOX5	K00461
ENSG00000012817	5.13E-15	-2.63127	1	1	KDM5D	K11446
ENSG00000013016	5.13E-15	-2.63127	1	1	EHD3	K12476
ENSG00000013293	5.13E-15	-2.63127	1	1	SLC7A14	_
ENSG00000013725	5.13E-15	-2.63127	1	1	CD6	K06456
ENSG00000014257	5.13E-15	-2.63127	1	1	ACPP	K19283
ENSG00000015285	5.13E-15	-2.63127	1	1	WAS	K05747
ENSG00000015592	5.13E-15	-2.63127	1	1	STMN4	_
ENSG00000016082	5.13E-15	-2.63127	1	1	ISL1	K09370
ENSG00000016402	5.13E-15	-2.63127	1	1	IL20RA	K05136
ENSG00000016490	5.13E-15	-2.63127	1	1	CLCA1	K05027
ENSG00000016602	5.13E-15	-2.63127	1	1	CLCA4	K05030
ENSG00000017427	5.13E-15	-2.63127	1	1	IGF1	K05459
ENSG00000018236	5.13E-15	-2.63127	1	1	CNTN1	K06759
ENSG00000018607	5.13E-15	-2.63127	1	1	ZNF806	K09228
ENSG00000020219	5.13E-15	-2.63127	1	1	CCT8L1P	K09500
ENSG00000021461	5.13E-15	-2.63127	1	1	CYP3A43	K17692
ENSG00000021488	5.13E-15	-2.63127	1	1	SLC7A9	K13868
ENSG00000021852	5.13E-15	-2.63127	1	1	C8B	K03998
ENSG00000022355	5.13E-15	-2.63127	1	1	GABRA1	K05175
ENSG00000022556	5.13E-15	-2.63127	1	1	NLRP2	K19409
ENSG00000025039	5.13E-15	-2.63127	1	1	RRAGD	K16186
ENSG00000026751	5.13E-15	-2.63127	1	1	SLAMF7	K06733
ENSG00000027644	5.13E-15	-2.63127	1	1	INSRR	K05086
ENSG00000029559	5.13E-15	-2.63127	1	1	IBSP	K06253
ENSG00000030304	5.13E-15	-2.63127	1	1	MUSK	K05129
ENSG00000030419	5.13E-15	-2.63127	1	1	IKZF2	K09220
ENSG00000034053	5.13E-15	-2.63127	1	1	APBA2	_
ENSG00000034239	5.13E-15	-2.63127	1	1	EFCAB1	_
ENSG00000034971	5.13E-15	-2.63127	1	1	MYOC	_
ENSG00000035720	5.13E-15	-2.63127	1	1	STAP1	_
ENSG00000036448	5.13E-15	-2.63127	1	1	MYOM2	_
ENSG00000036473	5.13E-15	-2.63127	1	1	OTC	K00611
ENSG00000036565	5.13E-15	-2.63127	1	1	SLC18A1	K08155
ENSG00000036828	5.13E-15	-2.63127	1	1	CASR	K04612
ENSG00000038427	5.13E-15	-2.63127	1	1	VCAN	K06793

ENSG00000038945	5.13E-15	-2.63127	1	1	MSR1	K06558
ENSG00000039537	5.13E-15	-2.63127	1	1	C6	K03995
ENSG00000039600	5.13E-15	-2.63127	1	1	SOX30	K09271
ENSG00000042304	5.13E-15	-2.63127	1	1	C2orf83	K11447
ENSG00000042813	5.13E-15	-2.63127	1	1	ZPBP	_
ENSG00000043039	5.13E-15	-2.63127	1	1	BARX2	K09361
ENSG00000043462	5.13E-15	-2.63127	1	1	LCP2	K07361
ENSG00000044012	5.13E-15	-2.63127	1	1	GUCA2B	_
ENSG00000046774	5.13E-15	-2.63127	1	1	MAGEC2	_
ENSG00000046889	5.13E-15	-2.63127	1	1	PREX2	K17588
ENSG00000048462	5.13E-15	-2.63127	1	1	TNFRSF17	K05153
ENSG00000048740	5.13E-15	-2.63127	1	1	CELF2	K13207
ENSG00000049247	5.13E-15	-2.63127	1	1	UTS2	K05248
ENSG00000049319	5.13E-15	-2.63127	1	1	SRD5A2	_
ENSG00000049540	5.13E-15	-2.63127	1	1	ELN	K14211
ENSG00000049768	5.13E-15	-2.63127	1	1	FOXP3	K10163
ENSG00000050030	5.13E-15	-2.63127	1	1	KIAA2022	_
ENSG00000050730	5.13E-15	-2.63127	1	1	TNIP3	_
ENSG00000050767	5.13E-15	-2.63127	1	1	COL23A1	_
ENSG00000053108	5.13E-15	-2.63127	1	1	FSTL4	_
ENSG00000053328	5.13E-15	-2.63127	1	1	METTL24	_
ENSG00000054796	5.13E-15	-2.63127	1	1	SPO11	K10878
ENSG00000054803	5.13E-15	-2.63127	1	1	CBLN4	_
ENSG00000054983	5.13E-15	-2.63127	1	1	GALC	K01202
ENSG00000055118	5.13E-15	-2.63127	1	1	KCNH2	K04905
ENSG00000055163	5.13E-15	-2.63127	1	1	CYFIP2	K05749
ENSG00000055813	5.13E-15	-2.63127	1	1	CCDC85A	_
ENSG00000055957	5.13E-15	-2.63127	1	1	ITIH1	K19014
ENSG00000056487	5.13E-15	-2.63127	1	1	PHF21B	_
ENSG00000056998	5.13E-15	-2.63127	1	1	GYG2	K00750
ENSG00000058866	5.13E-15	-2.63127	1	1	DGKG	K00901
ENSG00000059377	5.13E-15	-2.63127	1	1	TBXAS1	K01832
ENSG00000060303	5.13E-15	-2.63127	1	1	RPS17P5	K02962
ENSG00000060718	5.13E-15	-2.63127	1	1	COL11A1	K19721
ENSG00000060982	5.13E-15	-2.63127	1	1	BCAT1	K02927
ENSG00000061337	5.13E-15	-2.63127	1	1	LZTS1	_
ENSG00000061492	5.13E-15	-2.63127	1	1	WNT8A	K00714
ENSG00000061918	5.13E-15	-2.63127	1	1	GUCY1B3	K12319
ENSG00000062096	5.13E-15	-2.63127	1	1	ARSF	K12374
ENSG00000063515	5.13E-15	-2.63127	1	1	GSC2	K09325
ENSG00000063587	5.13E-15	-2.63127	1	1	ZNF275	_
ENSG00000064218	5.13E-15	-2.63127	1	1	DMRT3	K19490
ENSG00000064300	5.13E-15	-2.63127	1	1	NGFR	K02583
ENSG00000064692	5.13E-15	-2.63127	1	1	SNCAIP	K04558
ENSG00000064787	5.13E-15	-2.63127	1	1	BCAS1	_
ENSG00000064835	5.13E-15	-2.63127	1	1	POU1F1	K09363
ENSG00000065320	5.13E-15	-2.63127	1	1	NTN1	K06843
ENSG00000065371	5.13E-15	-2.63127	1	1	ROPN1	_
ENSG00000065609	5.13E-15	-2.63127	1	1	SNAP91	_
ENSG00000065675	5.13E-15	-2.63127	1	1	PRKCQ	K18052
ENSG00000066185	5.13E-15	-2.63127	1	1	ZMYND12	_
ENSG00000066294	5.13E-15	-2.63127	1	1	CD84	K06511
ENSG00000066336	5.13E-15	-2.63127	1	1	SPI1	K09438
ENSG00000066379	5.13E-15	-2.63127	1	1	ZNRD1	K03000
ENSG00000066382	5.13E-15	-2.63127	1	1	MPPED2	_
ENSG00000066813	5.13E-15	-2.63127	1	1	ACSM2B	K01896
ENSG00000067048	5.13E-15	-2.63127	1	1	DDX3Y	K17642
ENSG00000067445	5.13E-15	-2.63127	1	1	TRO	_

ENSG00000067646	5.13E-15	-2.63127	1	1	ZFY	_
ENSG00000067840	5.13E-15	-2.63127	1	1	PDZD4	K15682
ENSG00000067842	5.13E-15	-2.63127	1	1	ATP2B3	K05850
ENSG00000069018	5.13E-15	-2.63127	1	1	TRPC7	K04970
ENSG00000069122	5.13E-15	-2.63127	1	1	GPR116	K08458
ENSG00000069431	5.13E-15	-2.63127	1	1	ABCC9	K05033
ENSG00000069535	5.13E-15	-2.63127	1	1	MAOB	K00274
ENSG00000069764	5.13E-15	-2.63127	1	1	PLA2G10	K01047
ENSG00000070019	5.13E-15	-2.63127	1	1	GUCY2C	K12320
ENSG00000070193	5.13E-15	-2.63127	1	1	FGF10	K04358
ENSG00000070601	5.13E-15	-2.63127	1	1	FRMPD1	_
ENSG00000070748	5.13E-15	-2.63127	1	1	CHAT	K00623
ENSG00000070886	5.13E-15	-2.63127	1	1	EPHA8	K05109
ENSG00000070985	5.13E-15	-2.63127	1	1	TRPM5	K04980
ENSG00000071203	5.13E-15	-2.63127	1	1	MS4A12	_
ENSG00000071575	5.13E-15	-2.63127	1	1	TRIB2	K08814
ENSG00000071677	5.13E-15	-2.63127	1	1	PRLH	K05269
ENSG00000071909	5.13E-15	-2.63127	1	1	MYO3B	K08834
ENSG00000071991	5.13E-15	-2.63127	1	1	CDH19	K06806
ENSG00000072041	5.13E-15	-2.63127	1	1	SLC6A15	K05048
ENSG00000072080	5.13E-15	-2.63127	1	1	SPP2	_
ENSG00000072133	5.13E-15	-2.63127	1	1	RPS6KA6	K04373
ENSG00000072315	5.13E-15	-2.63127	1	1	TRPC5	K04968
ENSG00000072444	5.13E-15	-2.63127	1	1	ASAH2C	K12349
ENSG00000072506	5.13E-15	-2.63127	1	1	HSD17B10	K08683
ENSG00000072657	5.13E-15	-2.63127	1	1	TRHDE	K01306
ENSG00000072694	5.13E-15	-2.63127	1	1	FCGR2B	K12560
ENSG00000073734	5.13E-15	-2.63127	1	1	ABCB11	K05664
ENSG00000073754	5.13E-15	-2.63127	1	1	CD5L	_
ENSG00000073861	5.13E-15	-2.63127	1	1	TBX21	K10166
ENSG00000074211	5.13E-15	-2.63127	1	1	PPP2R2C	K04354
ENSG00000074317	5.13E-15	-2.63127	1	1	SNCB	_
ENSG00000074410	5.13E-15	-2.63127	1	1	CA12	K01672
ENSG00000074706	5.13E-15	-2.63127	1	1	IPCEF1	_
ENSG00000074771	5.13E-15	-2.63127	1	1	NOX3	K08008
ENSG00000074803	5.13E-15	-2.63127	1	1	SLC12A1	K14425
ENSG00000075035	5.13E-15	-2.63127	1	1	WSCD2	_
ENSG00000075073	5.13E-15	-2.63127	1	1	TACR2	K04223
ENSG00000075213	5.13E-15	-2.63127	1	1	SEMA3A	K06840
ENSG00000075290	5.13E-15	-2.63127	1	1	WNT8B	K00714
ENSG00000075340	5.13E-15	-2.63127	1	1	ADD2	K18622
ENSG00000075388	5.13E-15	-2.63127	1	1	FGF4	K04358
ENSG00000075429	5.13E-15	-2.63127	1	1	CACNG5	K04870
ENSG00000075461	5.13E-15	-2.63127	1	1	CACNG4	K04869
ENSG00000075673	5.13E-15	-2.63127	1	1	ATP12A	K01544
ENSG00000075891	5.13E-15	-2.63127	1	1	PAX2	K15608
ENSG00000076716	5.13E-15	-2.63127	1	1	GPC4	K08110
ENSG00000077009	5.13E-15	-2.63127	1	1	NMRK2	K10524
ENSG00000077264	5.13E-15	-2.63127	1	1	PAK3	K05733
ENSG00000077274	5.13E-15	-2.63127	1	1	CAPN6	K08575
ENSG00000077279	5.13E-15	-2.63127	1	1	DCX	K16579
ENSG00000077498	5.13E-15	-2.63127	1	1	TYR	K00505
ENSG00000077585	5.13E-15	-2.63127	1	1	GPR137B	_
ENSG00000077800	5.13E-15	-2.63127	1	1	FKBP6	K09572
ENSG00000077943	5.13E-15	-2.63127	1	1	ITGA8	K06584
ENSG00000077984	5.13E-15	-2.63127	1	1	CST7	K13903
ENSG00000078053	5.13E-15	-2.63127	1	1	AMPH	K12562
ENSG00000078081	5.13E-15	-2.63127	1	1	LAMP3	K06562

ENSG00000078295	5.13E-15	-2.63127	1	1	ADCY2	K08042
ENSG00000078328	5.13E-15	-2.63127	1	1	RBFOX1	K14946
ENSG00000078549	5.13E-15	-2.63127	1	1	ADCYAP1R1	K04587
ENSG00000078579	5.13E-15	-2.63127	1	1	FGF20	K04358
ENSG00000078725	5.13E-15	-2.63127	1	1	BRINP1	_
ENSG00000078898	5.13E-15	-2.63127	1	1	BPIFB2	_
ENSG00000079102	5.13E-15	-2.63127	1	1	RUNX1T1	K10053
ENSG00000079112	5.13E-15	-2.63127	1	1	CDH17	K06811
ENSG00000079557	5.13E-15	-2.63127	1	1	AFM	_
ENSG00000079689	5.13E-15	-2.63127	1	1	SCGN	_
ENSG00000080224	5.13E-15	-2.63127	1	1	EPHA6	K05107
ENSG00000080293	5.13E-15	-2.63127	1	1	SCTR	K04588
ENSG00000080493	5.13E-15	-2.63127	1	1	SLC4A4	K13575
ENSG00000080511	5.13E-15	-2.63127	1	1	RDH8	K11150
ENSG00000080572	5.13E-15	-2.63127	1	1	PIH1D3	K12581
ENSG00000080709	5.13E-15	-2.63127	1	1	KCNN2	K04943
ENSG00000080910	5.13E-15	-2.63127	1	1	CFHR2	K04004
ENSG00000081051	5.13E-15	-2.63127	1	1	AFP	K16144
ENSG00000081189	5.13E-15	-2.63127	1	1	MEF2C	_
ENSG00000081277	5.13E-15	-2.63127	1	1	PKP1	K10387
ENSG00000081479	5.13E-15	-2.63127	1	1	LRP2	K06233
ENSG00000081800	5.13E-15	-2.63127	1	1	SLC13A1	K14444
ENSG00000081803	5.13E-15	-2.63127	1	1	CADPS2	_
ENSG00000081818	5.13E-15	-2.63127	1	1	PCDHB4	K16494
ENSG00000081842	5.13E-15	-2.63127	1	1	PCDHA6	K16493
ENSG00000082175	5.13E-15	-2.63127	1	1	PGR	K08556
ENSG00000082397	5.13E-15	-2.63127	1	1	EPB41L3	K06107
ENSG00000082482	5.13E-15	-2.63127	1	1	KCNK2	K04913
ENSG00000082556	5.13E-15	-2.63127	1	1	OPRK1	K04214
ENSG00000082684	5.13E-15	-2.63127	1	1	SEMA5B	K06841
ENSG00000082929	5.13E-15	-2.63127	1	1	C4orf6	_
ENSG00000083622	5.13E-15	-2.63127	1	1	AC000111.6	_
ENSG00000083782	5.13E-15	-2.63127	1	1	EPYC	K08127
ENSG00000083842	5.13E-15	-2.63127	1	1	ZNF8	K09228
ENSG00000083896	5.13E-15	-2.63127	1	1	YTHDC1	_
ENSG00000084207	5.13E-15	-2.63127	1	1	GSTP1	K00799
ENSG00000084674	5.13E-15	-2.63127	1	1	APOB	K14462
ENSG00000084734	5.13E-15	-2.63127	1	1	GCKR	_
ENSG00000085265	5.13E-15	-2.63127	1	1	FCN1	K10104
ENSG00000086205	5.13E-15	-2.63127	1	1	FOLH1	K14592
ENSG00000086288	5.13E-15	-2.63127	1	1	NME8	_
ENSG00000086548	5.13E-15	-2.63127	1	1	CEACAM6	K06499
ENSG00000086619	5.13E-15	-2.63127	1	1	ERO1LB	K10976
ENSG00000086696	5.13E-15	-2.63127	1	1	HSD17B2	K13368
ENSG00000086848	5.13E-15	-2.63127	1	1	ALG9	K03846
ENSG00000086967	5.13E-15	-2.63127	1	1	MYBPC2	K12558
ENSG00000087128	5.13E-15	-2.63127	1	1	TMPRSS11E	K09642
ENSG00000087250	5.13E-15	-2.63127	1	1	MT3	K14740
ENSG00000087494	5.13E-15	-2.63127	1	1	PTHLH	_
ENSG00000087495	5.13E-15	-2.63127	1	1	PHACTR3	K17594
ENSG00000088038	5.13E-15	-2.63127	1	1	CNOT3	K12580
ENSG00000088053	5.13E-15	-2.63127	1	1	GP6	K06264
ENSG00000088320	5.13E-15	-2.63127	1	1	REM1	K07847
ENSG00000088386	5.13E-15	-2.63127	1	1	SLC15A1	K14206
ENSG00000088782	5.13E-15	-2.63127	1	1	DEFB127	_
ENSG00000088926	5.13E-15	-2.63127	1	1	F11	K01323
ENSG00000089012	5.13E-15	-2.63127	1	1	SIRPG	K06551
ENSG00000089041	5.13E-15	-2.63127	1	1	P2RX7	K05220

ENSG00000089116	5.13E-15	-2.63127	1	1	LHX5	K18493
ENSG00000089169	5.13E-15	-2.63127	1	1	RPH3A	_
ENSG00000089199	5.13E-15	-2.63127	1	1	CHGB	_
ENSG00000089225	5.13E-15	-2.63127	1	1	TBX5	K10179
ENSG00000089250	5.13E-15	-2.63127	1	1	NOS1	_
ENSG00000089472	5.13E-15	-2.63127	1	1	HEPH	K14735
ENSG00000090104	5.13E-15	-2.63127	1	1	RGS1	K16449
ENSG00000090382	5.13E-15	-2.63127	1	1	LYZ	K13915
ENSG00000090402	5.13E-15	-2.63127	1	1	SI	K01203
ENSG00000090512	5.13E-15	-2.63127	1	1	FETUB	_
ENSG00000090659	5.13E-15	-2.63127	1	1	CD209	K06563
ENSG00000091010	5.13E-15	-2.63127	1	1	POU4F3	K09366
ENSG00000091128	5.13E-15	-2.63127	1	1	LAMB4	K06245
ENSG00000091129	5.13E-15	-2.63127	1	1	NRCAM	K06756
ENSG00000091138	5.13E-15	-2.63127	1	1	SLC26A3	K14078
ENSG00000091181	5.13E-15	-2.63127	1	1	IL5RA	K05067
ENSG00000091583	5.13E-15	-2.63127	1	1	APOH	K17305
ENSG00000091664	5.13E-15	-2.63127	1	1	SLC17A6	K12302
ENSG00000091704	5.13E-15	-2.63127	1	1	CPA1	K08779
ENSG00000092009	5.13E-15	-2.63127	1	1	CMA1	K01329
ENSG00000092051	5.13E-15	-2.63127	1	1	JPH4	K19530
ENSG00000092054	5.13E-15	-2.63127	1	1	MYH7	K17751
ENSG00000092096	5.13E-15	-2.63127	1	1	SLC22A17	K08213
ENSG00000092345	5.13E-15	-2.63127	1	1	DAZL	_
ENSG00000092377	5.13E-15	-2.63127	1	1	TBL1Y	K04508
ENSG00000092607	5.13E-15	-2.63127	1	1	TBX15	K10182
ENSG00000093072	5.13E-15	-2.63127	1	1	CECR1	K19572
ENSG00000094661	5.13E-15	-2.63127	1	1	OR1I1	K04257
ENSG00000094755	5.13E-15	-2.63127	1	1	GABRP	K05189
ENSG00000094796	5.13E-15	-2.63127	1	1	KRT31	K07604
ENSG00000094963	5.13E-15	-2.63127	1	1	FMO2	K00485
ENSG00000095110	5.13E-15	-2.63127	1	1	NXPE1	_
ENSG00000095585	5.13E-15	-2.63127	1	1	BLNK	K07371
ENSG00000095627	5.13E-15	-2.63127	1	1	TDRD1	K18405
ENSG00000095713	5.13E-15	-2.63127	1	1	CRTAC1	_
ENSG00000095777	5.13E-15	-2.63127	1	1	MYO3A	K08834
ENSG00000095917	5.13E-15	-2.63127	1	1	TPSD1	K01340
ENSG00000095932	5.13E-15	-2.63127	1	1	C19orf77	_
ENSG00000095970	5.13E-15	-2.63127	1	1	TREM2	K14378
ENSG00000095981	5.13E-15	-2.63127	1	1	KCNK16	K04924
ENSG00000096006	5.13E-15	-2.63127	1	1	CRISP3	_
ENSG00000096088	5.13E-15	-2.63127	1	1	PGC	K01377
ENSG00000096264	5.13E-15	-2.63127	1	1	NCR2	K06742
ENSG00000096395	5.13E-15	-2.63127	1	1	MLN	K05265
ENSG00000099139	5.13E-15	-2.63127	1	1	PCSK5	K08654
ENSG00000099284	5.13E-15	-2.63127	1	1	H2AFY2	K11251
ENSG00000099715	5.13E-15	-2.63127	1	1	PCDH11Y	K16498
ENSG00000099721	5.13E-15	-2.63127	1	1	AMELY	_
ENSG00000099725	5.13E-15	-2.63127	1	1	PRKY	K19584
ENSG00000099869	5.13E-15	-2.63127	1	1	IGF2-AS	_
ENSG00000099953	5.13E-15	-2.63127	1	1	MMP11	K07993
ENSG00000100012	5.13E-15	-2.63127	1	1	SEC14L3	_
ENSG00000100024	5.13E-15	-2.63127	1	1	UPB1	K01431
ENSG00000100095	5.13E-15	-2.63127	1	1	SEZ6L	_
ENSG00000100121	5.13E-15	-2.63127	1	1	GGTLC2	K18592
ENSG00000100122	5.13E-15	-2.63127	1	1	CRYBB1	_
ENSG00000100170	5.13E-15	-2.63127	1	1	SLC5A1	K14158
ENSG00000100181	5.13E-15	-2.63127	1	1	TPTEP1	_

ENSG00000100191	5.13E-15	-2.63127	1	1	SLC5A4	K14384
ENSG00000100218	5.13E-15	-2.63127	1	1	RTDR1	_
ENSG00000100228	5.13E-15	-2.63127	1	1	RAB36	K07922
ENSG00000100249	5.13E-15	-2.63127	1	1	C22orf31	_
ENSG00000100312	5.13E-15	-2.63127	1	1	ACR	K01317
ENSG00000100321	5.13E-15	-2.63127	1	1	SYNGR1	K04403
ENSG00000100336	5.13E-15	-2.63127	1	1	APOL4	K14480
ENSG00000100341	5.13E-15	-2.63127	1	1	PNPLA5	K16814
ENSG00000100346	5.13E-15	-2.63127	1	1	CACNA1I	K04856
ENSG00000100365	5.13E-15	-2.63127	1	1	NCF4	K08012
ENSG00000100373	5.13E-15	-2.63127	1	1	UPK3A	K19520
ENSG00000100379	5.13E-15	-2.63127	1	1	KCTD17	_
ENSG00000100433	5.13E-15	-2.63127	1	1	KCNK10	K04920
ENSG00000100448	5.13E-15	-2.63127	1	1	CTSG	K01319
ENSG00000100450	5.13E-15	-2.63127	1	1	GZMH	K09616
ENSG00000100453	5.13E-15	-2.63127	1	1	GZMB	K01353
ENSG00000100557	5.13E-15	-2.63127	1	1	C14orf105	_
ENSG00000100565	5.13E-15	-2.63127	1	1	C14orf166B	_
ENSG00000100593	5.13E-15	-2.63127	1	1	ISM2	_
ENSG00000100599	5.13E-15	-2.63127	1	1	RIN3	_
ENSG00000100626	5.13E-15	-2.63127	1	1	GALNT16	K00710
ENSG00000100665	5.13E-15	-2.63127	1	1	SERPINA4	K04525
ENSG00000100721	5.13E-15	-2.63127	1	1	TCL1A	K10167
ENSG00000100884	5.13E-15	-2.63127	1	1	CPNE6	_
ENSG00000100987	5.13E-15	-2.63127	1	1	VSX1	K09335
ENSG00000101074	5.13E-15	-2.63127	1	1	R3HDML	_
ENSG00000101134	5.13E-15	-2.63127	1	1	DOK5	_
ENSG00000101144	5.13E-15	-2.63127	1	1	BMP7	K16621
ENSG00000101180	5.13E-15	-2.63127	1	1	HRH3	K04151
ENSG00000101194	5.13E-15	-2.63127	1	1	SLC17A9	K12303
ENSG00000101200	5.13E-15	-2.63127	1	1	AVP	K05242
ENSG00000101251	5.13E-15	-2.63127	1	1	SEL1L2	K14026
ENSG00000101265	5.13E-15	-2.63127	1	1	RASSF2	K09851
ENSG00000101282	5.13E-15	-2.63127	1	1	RSPO4	_
ENSG00000101292	5.13E-15	-2.63127	1	1	PROKR2	K08380
ENSG00000101307	5.13E-15	-2.63127	1	1	SIRPB1	K06551
ENSG00000101323	5.13E-15	-2.63127	1	1	HAO1	K11517
ENSG00000101327	5.13E-15	-2.63127	1	1	PDYN	K15840
ENSG00000101349	5.13E-15	-2.63127	1	1	PAK7	K05736
ENSG00000101405	5.13E-15	-2.63127	1	1	OXT	K05243
ENSG00000101425	5.13E-15	-2.63127	1	1	BPI	_
ENSG00000101435	5.13E-15	-2.63127	1	1	CST9L	K13905
ENSG00000101438	5.13E-15	-2.63127	1	1	SLC32A1	K15015
ENSG00000101441	5.13E-15	-2.63127	1	1	CST4	K13900
ENSG00000101445	5.13E-15	-2.63127	1	1	PPP1R16B	K17459
ENSG00000101446	5.13E-15	-2.63127	1	1	SPINT3	_
ENSG00000101448	5.13E-15	-2.63127	1	1	EPPIN	_
ENSG00000101463	5.13E-15	-2.63127	1	1	SYNDIG1	_
ENSG00000101489	5.13E-15	-2.63127	1	1	CELF4	K13207
ENSG00000101493	5.13E-15	-2.63127	1	1	ZNF516	_
ENSG00000101746	5.13E-15	-2.63127	1	1	NOL4	_
ENSG00000101812	5.13E-15	-2.63127	1	1	H2BFM	K11252
ENSG00000101825	5.13E-15	-2.63127	1	1	MXRA5	_
ENSG00000101883	5.13E-15	-2.63127	1	1	RHOXF1	_
ENSG00000101892	5.13E-15	-2.63127	1	1	ATP1B4	K01540
ENSG00000101938	5.13E-15	-2.63127	1	1	CHRDL1	_
ENSG00000101940	5.13E-15	-2.63127	1	1	WDR13	_
ENSG00000101951	5.13E-15	-2.63127	1	1	PAGE4	_

ENSG00000101958	5.13E-15	-2.63127	1	1	GLRA2	K05194
ENSG00000101981	5.13E-15	-2.63127	1	1	F9	K01321
ENSG00000102001	5.13E-15	-2.63127	1	1	CACNA1F	K04853
ENSG00000102021	5.13E-15	-2.63127	1	1	LUZP4	_
ENSG00000102043	5.13E-15	-2.63127	1	1	MTMR8	K18083
ENSG00000102055	5.13E-15	-2.63127	1	1	PPP1R2P9	K16833
ENSG00000102069	5.13E-15	-2.63127	1	1	UBE2NL	K10580
ENSG00000102076	5.13E-15	-2.63127	1	1	OPN1LW	K04251
ENSG00000102080	5.13E-15	-2.63127	1	1	TEX28P2	_
ENSG00000102096	5.13E-15	-2.63127	1	1	PIM2	K08806
ENSG00000102109	5.13E-15	-2.63127	1	1	PCSK1N	_
ENSG00000102128	5.13E-15	-2.63127	1	1	RAB40AL	K07928
ENSG00000102145	5.13E-15	-2.63127	1	1	GATA1	K09182
ENSG00000102230	5.13E-15	-2.63127	1	1	PCYT1B	K00968
ENSG00000102239	5.13E-15	-2.63127	1	1	BRS3	K04170
ENSG00000102243	5.13E-15	-2.63127	1	1	VGLL1	_
ENSG00000102245	5.13E-15	-2.63127	1	1	CD40LG	K03161
ENSG00000102290	5.13E-15	-2.63127	1	1	PCDH11X	K16498
ENSG00000102313	5.13E-15	-2.63127	1	1	ITIH6	_
ENSG00000102349	5.13E-15	-2.63127	1	1	KLF8	K09205
ENSG00000102383	5.13E-15	-2.63127	1	1	ZDHHC15	K18932
ENSG00000102387	5.13E-15	-2.63127	1	1	TAF7L	K03132
ENSG00000102390	5.13E-15	-2.63127	1	1	PBDC1	_
ENSG00000102401	5.13E-15	-2.63127	1	1	ARMCX3	_
ENSG00000102409	5.13E-15	-2.63127	1	1	BEX4	_
ENSG00000102445	5.13E-15	-2.63127	1	1	KIAA0226L	_
ENSG00000102468	5.13E-15	-2.63127	1	1	HTR2A	K04157
ENSG00000102524	5.13E-15	-2.63127	1	1	TNFSF13B	K05476
ENSG00000102683	5.13E-15	-2.63127	1	1	SGCG	K12564
ENSG00000102755	5.13E-15	-2.63127	1	1	FLT1	K05096
ENSG00000102760	5.13E-15	-2.63127	1	1	RGCC	_
ENSG00000102794	5.13E-15	-2.63127	1	1	IRG1	K17724
ENSG00000102891	5.13E-15	-2.63127	1	1	MT4	K14741
ENSG00000102924	5.13E-15	-2.63127	1	1	CBLN1	_
ENSG00000102935	5.13E-15	-2.63127	1	1	ZNF423	_
ENSG00000102962	5.13E-15	-2.63127	1	1	CCL22	K05514
ENSG00000103023	5.13E-15	-2.63127	1	1	PRSS54	_
ENSG00000103154	5.13E-15	-2.63127	1	1	NECAB2	_
ENSG00000103200	5.13E-15	-2.63127	1	1	RP11-723C11.2	_
ENSG00000103310	5.13E-15	-2.63127	1	1	ZP2	_
ENSG00000103355	5.13E-15	-2.63127	1	1	PRSS33	K09629
ENSG00000103375	5.13E-15	-2.63127	1	1	AQP8	K09869
ENSG00000103426	5.13E-15	-2.63127	1	1	CORO7-PAM16	K18619
ENSG00000103449	5.13E-15	-2.63127	1	1	SALL1	_
ENSG00000103460	5.13E-15	-2.63127	1	1	TOX3	_
ENSG00000103489	5.13E-15	-2.63127	1	1	XYLT1	K00771
ENSG00000103546	5.13E-15	-2.63127	1	1	SLC6A2	K05035
ENSG00000103569	5.13E-15	-2.63127	1	1	AQP9	K09877
ENSG00000103647	5.13E-15	-2.63127	1	1	CORO2B	K13887
ENSG00000104044	5.13E-15	-2.63127	1	1	OCA2	_
ENSG00000104055	5.13E-15	-2.63127	1	1	TGM5	K05622
ENSG00000104059	5.13E-15	-2.63127	1	1	FAM189A1	_
ENSG00000104112	5.13E-15	-2.63127	1	1	SCG3	_
ENSG00000104267	5.13E-15	-2.63127	1	1	CA2	K18245
ENSG00000104332	5.13E-15	-2.63127	1	1	SFRP1	K02166
ENSG00000104371	5.13E-15	-2.63127	1	1	DKK4	K02165
ENSG00000104415	5.13E-15	-2.63127	1	1	WISP1	_
ENSG00000104435	5.13E-15	-2.63127	1	1	STMN2	K04381

ENSG00000104499	5.13E-15	-2.63127	1	1	GML	_
ENSG00000104722	5.13E-15	-2.63127	1	1	NEFM	K04573
ENSG00000104725	5.13E-15	-2.63127	1	1	NEFL	_
ENSG00000104755	5.13E-15	-2.63127	1	1	ADAM2	K06833
ENSG00000104818	5.13E-15	-2.63127	1	1	CGB2	K10045
ENSG00000104826	5.13E-15	-2.63127	1	1	LHB	K08521
ENSG00000104827	5.13E-15	-2.63127	1	1	CGB	K10045
ENSG00000104833	5.13E-15	-2.63127	1	1	TUBB4A	K07375
ENSG00000104903	5.13E-15	-2.63127	1	1	LYL1	K15604
ENSG00000104918	5.13E-15	-2.63127	1	1	RETN	K13438
ENSG00000104938	5.13E-15	-2.63127	1	1	CLEC4M	K06563
ENSG00000104967	5.13E-15	-2.63127	1	1	NOVA2	K14944
ENSG00000104970	5.13E-15	-2.63127	1	1	KIR3DX1	K06725
ENSG00000104972	5.13E-15	-2.63127	1	1	LILRB1	K06512
ENSG00000104974	5.13E-15	-2.63127	1	1	LILRA1	K06512
ENSG00000105143	5.13E-15	-2.63127	1	1	SLC1A6	K05617
ENSG00000105198	5.13E-15	-2.63127	1	1	LGALS13	K10096
ENSG00000105205	5.13E-15	-2.63127	1	1	CLC	K13334
ENSG00000105352	5.13E-15	-2.63127	1	1	CEACAM4	K06499
ENSG00000105369	5.13E-15	-2.63127	1	1	CD79A	K06506
ENSG00000105371	5.13E-15	-2.63127	1	1	ICAM4	K06581
ENSG00000105388	5.13E-15	-2.63127	1	1	CEACAM5	K06499
ENSG00000105392	5.13E-15	-2.63127	1	1	CRX	_
ENSG00000105398	5.13E-15	-2.63127	1	1	SULT2A1	K11822
ENSG00000105428	5.13E-15	-2.63127	1	1	ZNRF4	K15715
ENSG00000105472	5.13E-15	-2.63127	1	1	CLEC11A	K17521
ENSG00000105492	5.13E-15	-2.63127	1	1	SIGLEC6	K06738
ENSG00000105501	5.13E-15	-2.63127	1	1	SIGLEC5	K06549
ENSG00000105507	5.13E-15	-2.63127	1	1	CABP5	_
ENSG00000105609	5.13E-15	-2.63127	1	1	LILRB5	K06512
ENSG00000105610	5.13E-15	-2.63127	1	1	KLF1	K09204
ENSG00000105617	5.13E-15	-2.63127	1	1	LENG1	K12580
ENSG00000105618	5.13E-15	-2.63127	1	1	PRPF31	K12844
ENSG00000105619	5.13E-15	-2.63127	1	1	TFPT	K11670
ENSG00000105675	5.13E-15	-2.63127	1	1	ATP4A	K01542
ENSG00000105694	5.13E-15	-2.63127	1	1	TCEB1P28	K03872
ENSG00000105750	5.13E-15	-2.63127	1	1	ZNF85	K09228
ENSG00000105825	5.13E-15	-2.63127	1	1	TFPI2	_
ENSG00000105851	5.13E-15	-2.63127	1	1	PIK3CG	K00922
ENSG00000105852	5.13E-15	-2.63127	1	1	PON3	K01045
ENSG00000105880	5.13E-15	-2.63127	1	1	DLX5	K18489
ENSG00000105889	5.13E-15	-2.63127	1	1	STEAP1B	K14737
ENSG00000105894	5.13E-15	-2.63127	1	1	PTN	K16642
ENSG00000105954	5.13E-15	-2.63127	1	1	NPVF	_
ENSG00000105989	5.13E-15	-2.63127	1	1	WNT2	K00182
ENSG00000106013	5.13E-15	-2.63127	1	1	ANKRD7	_
ENSG00000106038	5.13E-15	-2.63127	1	1	EVX1	K09320
ENSG00000106066	5.13E-15	-2.63127	1	1	CPVL	K09645
ENSG00000106113	5.13E-15	-2.63127	1	1	CRHR2	K04579
ENSG00000106123	5.13E-15	-2.63127	1	1	EPHB6	K05114
ENSG00000106128	5.13E-15	-2.63127	1	1	GHRHR	K04584
ENSG00000106178	5.13E-15	-2.63127	1	1	CCL24	K05514
ENSG00000106236	5.13E-15	-2.63127	1	1	NPTX2	_
ENSG00000106278	5.13E-15	-2.63127	1	1	PTPRZ1	K08114
ENSG00000106304	5.13E-15	-2.63127	1	1	SPAM1	K01197
ENSG00000106328	5.13E-15	-2.63127	1	1	FSCN3	K17456
ENSG00000106331	5.13E-15	-2.63127	1	1	PAX4	K08032
ENSG00000106341	5.13E-15	-2.63127	1	1	PPP1R17	K08067

ENSG00000106410	5.13E-15	-2.63127	1	1	NOBOX	_
ENSG00000106511	5.13E-15	-2.63127	1	1	MEOX2	K09322
ENSG00000106536	5.13E-15	-2.63127	1	1	POU6F2	K09368
ENSG00000106540	5.13E-15	-2.63127	1	1	AC004837.3	_
ENSG00000106565	5.13E-15	-2.63127	1	1	TMEM176B	_
ENSG00000106648	5.13E-15	-2.63127	1	1	GALNTL5	K00710
ENSG00000106688	5.13E-15	-2.63127	1	1	SLC1A1	K05612
ENSG00000106714	5.13E-15	-2.63127	1	1	CNTNAP3	_
ENSG00000106772	5.13E-15	-2.63127	1	1	PRUNE2	K18449
ENSG00000106952	5.13E-15	-2.63127	1	1	TNFSF8	K05471
ENSG00000107014	5.13E-15	-2.63127	1	1	RLN2	K05255
ENSG00000107018	5.13E-15	-2.63127	1	1	RLN1	K05255
ENSG00000107130	5.13E-15	-2.63127	1	1	NCS1	_
ENSG00000107187	5.13E-15	-2.63127	1	1	LHX3	K09374
ENSG00000107249	5.13E-15	-2.63127	1	1	GLIS3	K09232
ENSG00000107282	5.13E-15	-2.63127	1	1	APBA1	K04531
ENSG00000107447	5.13E-15	-2.63127	1	1	DNTT	K00977
ENSG00000107518	5.13E-15	-2.63127	1	1	ATRNL1	_
ENSG00000107562	5.13E-15	-2.63127	1	1	CXCL12	K10031
ENSG00000107618	5.13E-15	-2.63127	1	1	RBP3	_
ENSG00000107623	5.13E-15	-2.63127	1	1	GDF10	K05496
ENSG00000107719	5.13E-15	-2.63127	1	1	PALD1	_
ENSG00000107807	5.13E-15	-2.63127	1	1	TLX1	K09340
ENSG00000108001	5.13E-15	-2.63127	1	1	EBF3	K09103
ENSG00000108231	5.13E-15	-2.63127	1	1	LGI1	_
ENSG00000108242	5.13E-15	-2.63127	1	1	CYP2C18	K17720
ENSG00000108244	5.13E-15	-2.63127	1	1	KRT23	K07604
ENSG00000108342	5.13E-15	-2.63127	1	1	CSF3	K05423
ENSG00000108381	5.13E-15	-2.63127	1	1	ASPA	K01437
ENSG00000108417	5.13E-15	-2.63127	1	1	KRT37	K07604
ENSG00000108442	5.13E-15	-2.63127	1	1	TVP23BP2	_
ENSG00000108516	5.13E-15	-2.63127	1	1	AC003958.6	_
ENSG00000108576	5.13E-15	-2.63127	1	1	SLC6A4	K05037
ENSG00000108684	5.13E-15	-2.63127	1	1	ASIC2	K04828
ENSG00000108688	5.13E-15	-2.63127	1	1	CCL7	K05509
ENSG00000108700	5.13E-15	-2.63127	1	1	CCL8	K16596
ENSG00000108702	5.13E-15	-2.63127	1	1	CCL1	K05514
ENSG00000108753	5.13E-15	-2.63127	1	1	HNF1B	K08034
ENSG00000108759	5.13E-15	-2.63127	1	1	KRT32	K07604
ENSG00000108798	5.13E-15	-2.63127	1	1	ABI3	_
ENSG00000108830	5.13E-15	-2.63127	1	1	RND2	K07858
ENSG00000108878	5.13E-15	-2.63127	1	1	CACNG1	K04866
ENSG00000108932	5.13E-15	-2.63127	1	1	SLC16A6	K08183
ENSG00000109079	5.13E-15	-2.63127	1	1	TNFAIP1	K15074
ENSG00000109132	5.13E-15	-2.63127	1	1	PHOX2B	K09330
ENSG00000109158	5.13E-15	-2.63127	1	1	GABRA4	K05175
ENSG00000109181	5.13E-15	-2.63127	1	1	UGT2B10	K00699
ENSG00000109182	5.13E-15	-2.63127	1	1	CWH43	_
ENSG00000109193	5.13E-15	-2.63127	1	1	SULT1E1	K01016
ENSG00000109205	5.13E-15	-2.63127	1	1	ODAM	_
ENSG00000109208	5.13E-15	-2.63127	1	1	SMR3A	_
ENSG00000109255	5.13E-15	-2.63127	1	1	NMU	K05249
ENSG00000109272	5.13E-15	-2.63127	1	1	PF4V1	K05407
ENSG00000109424	5.13E-15	-2.63127	1	1	UCP1	K08769
ENSG00000109471	5.13E-15	-2.63127	1	1	IL2	K05429
ENSG00000109576	5.13E-15	-2.63127	1	1	AADAT	K00825
ENSG00000109667	5.13E-15	-2.63127	1	1	SLC2A9	K08146
ENSG00000109684	5.13E-15	-2.63127	1	1	CLNK	K15696

ENSG00000109705	5.13E-15	-2.63127	1	1	NKX3-2	K09995
ENSG00000109794	5.13E-15	-2.63127	1	1	FAM149A	_
ENSG00000109832	5.13E-15	-2.63127	1	1	DDX25	K18656
ENSG00000109846	5.13E-15	-2.63127	1	1	CRYAB	K09542
ENSG00000109851	5.13E-15	-2.63127	1	1	DBX1	K18488
ENSG00000109906	5.13E-15	-2.63127	1	1	ZBTB16	K10055
ENSG00000109943	5.13E-15	-2.63127	1	1	CRTAM	K16361
ENSG00000109944	5.13E-15	-2.63127	1	1	C11orf63	_
ENSG00000109956	5.13E-15	-2.63127	1	1	B3GAT1	K00735
ENSG00000110077	5.13E-15	-2.63127	1	1	MS4A6A	_
ENSG00000110079	5.13E-15	-2.63127	1	1	MS4A4A	_
ENSG00000110148	5.13E-15	-2.63127	1	1	CCKBR	K04195
ENSG00000110244	5.13E-15	-2.63127	1	1	APOA4	K08760
ENSG00000110245	5.13E-15	-2.63127	1	1	APOC3	K08759
ENSG00000110324	5.13E-15	-2.63127	1	1	IL10RA	K05134
ENSG00000110347	5.13E-15	-2.63127	1	1	MMP12	_
ENSG00000110448	5.13E-15	-2.63127	1	1	CD5	K06455
ENSG00000110484	5.13E-15	-2.63127	1	1	SCGB2A2	_
ENSG00000110665	5.13E-15	-2.63127	1	1	C11orf21	_
ENSG00000110675	5.13E-15	-2.63127	1	1	ELMOD1	_
ENSG00000110680	5.13E-15	-2.63127	1	1	CALCA	K12332
ENSG00000110777	5.13E-15	-2.63127	1	1	POU2AF1	_
ENSG00000110786	5.13E-15	-2.63127	1	1	PTPN5	K18018
ENSG00000110811	5.13E-15	-2.63127	1	1	LEPREL2	_
ENSG00000110848	5.13E-15	-2.63127	1	1	CD69	K06502
ENSG00000110934	5.13E-15	-2.63127	1	1	BIN2	_
ENSG00000110975	5.13E-15	-2.63127	1	1	SYT10	_
ENSG00000111046	5.13E-15	-2.63127	1	1	MYF6	K18485
ENSG00000111049	5.13E-15	-2.63127	1	1	MYF5	K18484
ENSG00000111052	5.13E-15	-2.63127	1	1	LIN7A	_
ENSG00000111058	5.13E-15	-2.63127	1	1	ACSS3	K01908
ENSG00000111199	5.13E-15	-2.63127	1	1	TRPV4	K04973
ENSG00000111224	5.13E-15	-2.63127	1	1	PARP11	K15259
ENSG00000111241	5.13E-15	-2.63127	1	1	FGF6	K04358
ENSG00000111245	5.13E-15	-2.63127	1	1	MYL2	K10351
ENSG00000111249	5.13E-15	-2.63127	1	1	CUX2	K09313
ENSG00000111262	5.13E-15	-2.63127	1	1	KCNA1	K04874
ENSG00000111344	5.13E-15	-2.63127	1	1	RASAL1	K17632
ENSG00000111404	5.13E-15	-2.63127	1	1	RERGL	K17198
ENSG00000111536	5.13E-15	-2.63127	1	1	IL26	K05446
ENSG00000111537	5.13E-15	-2.63127	1	1	IFNG	K04687
ENSG00000111664	5.13E-15	-2.63127	1	1	GNB3	K07825
ENSG00000111667	5.13E-15	-2.63127	1	1	USP5	K11836
ENSG00000111671	5.13E-15	-2.63127	1	1	SPSB2	K10344
ENSG00000111674	5.13E-15	-2.63127	1	1	ENO2	K01689
ENSG00000111678	5.13E-15	-2.63127	1	1	C12orf57	_
ENSG00000111701	5.13E-15	-2.63127	1	1	APOBEC1	K16932
ENSG00000111704	5.13E-15	-2.63127	1	1	NANOG	K10164
ENSG00000111713	5.13E-15	-2.63127	1	1	GYS2	K00693
ENSG00000111783	5.13E-15	-2.63127	1	1	RFX4	K09174
ENSG00000111796	5.13E-15	-2.63127	1	1	KLRB1	K06543
ENSG00000111879	5.13E-15	-2.63127	1	1	FAM184A	_
ENSG00000111886	5.13E-15	-2.63127	1	1	GABRR2	K05190
ENSG00000112038	5.13E-15	-2.63127	1	1	OPRM1	K04215
ENSG00000112077	5.13E-15	-2.63127	1	1	RHAG	K06580
ENSG00000112115	5.13E-15	-2.63127	1	1	IL17A	K05489
ENSG00000112116	5.13E-15	-2.63127	1	1	IL17F	K05494
ENSG00000112164	5.13E-15	-2.63127	1	1	GLP1R	K04581

ENSG00000112195	5.13E-15	-2.63127	1	1	TREML2	K14364
ENSG00000112214	5.13E-15	-2.63127	1	1	FHL5	_
ENSG00000112218	5.13E-15	-2.63127	1	1	GPR63	K04321
ENSG00000112232	5.13E-15	-2.63127	1	1	KHDRBS2	K17843
ENSG00000112238	5.13E-15	-2.63127	1	1	PRDM13	_
ENSG00000112273	5.13E-15	-2.63127	1	1	HDGFL1	K16641
ENSG00000112276	5.13E-15	-2.63127	1	1	BVES	_
ENSG00000112280	5.13E-15	-2.63127	1	1	COL9A1	K08131
ENSG00000112293	5.13E-15	-2.63127	1	1	GPLD1	K01127
ENSG00000112320	5.13E-15	-2.63127	1	1	SOBP	_
ENSG00000112333	5.13E-15	-2.63127	1	1	NR2E1	K08545
ENSG00000112337	5.13E-15	-2.63127	1	1	SLC17A2	K12300
ENSG00000112394	5.13E-15	-2.63127	1	1	SLC16A10	K08187
ENSG00000112462	5.13E-15	-2.63127	1	1	OR12D3	K04257
ENSG00000112473	5.13E-15	-2.63127	1	1	SLC39A7	K14713
ENSG00000112486	5.13E-15	-2.63127	1	1	CCR6	K04181
ENSG00000112494	5.13E-15	-2.63127	1	1	UNC93A	_
ENSG00000112530	5.13E-15	-2.63127	1	1	PACRG	_
ENSG00000112539	5.13E-15	-2.63127	1	1	C6orf118	_
ENSG00000112562	5.13E-15	-2.63127	1	1	SMOC2	_
ENSG00000112761	5.13E-15	-2.63127	1	1	WISP3	_
ENSG00000112769	5.13E-15	-2.63127	1	1	LAMA4	K06241
ENSG00000112782	5.13E-15	-2.63127	1	1	CLIC5	K05025
ENSG00000112796	5.13E-15	-2.63127	1	1	ENPP5	K18398
ENSG00000112799	5.13E-15	-2.63127	1	1	LY86	_
ENSG00000112818	5.13E-15	-2.63127	1	1	MEP1A	K01395
ENSG00000112837	5.13E-15	-2.63127	1	1	TBX18	K10183
ENSG00000112852	5.13E-15	-2.63127	1	1	PCDHB2	K16494
ENSG00000112936	5.13E-15	-2.63127	1	1	C7	K03996
ENSG00000112964	5.13E-15	-2.63127	1	1	GHR	K05080
ENSG00000112981	5.13E-15	-2.63127	1	1	NME5	K00940
ENSG00000113088	5.13E-15	-2.63127	1	1	GZMK	K08663
ENSG00000113196	5.13E-15	-2.63127	1	1	HAND1	K09071
ENSG00000113205	5.13E-15	-2.63127	1	1	PCDHB3	K16494
ENSG00000113209	5.13E-15	-2.63127	1	1	PCDHB5	K16494
ENSG00000113211	5.13E-15	-2.63127	1	1	PCDHB6	K16494
ENSG00000113212	5.13E-15	-2.63127	1	1	PCDHB7	K16494
ENSG00000113248	5.13E-15	-2.63127	1	1	PCDHB15	K16494
ENSG00000113249	5.13E-15	-2.63127	1	1	HAVCR1	_
ENSG00000113262	5.13E-15	-2.63127	1	1	GRM6	K04608
ENSG00000113296	5.13E-15	-2.63127	1	1	THBS4	K04659
ENSG00000113302	5.13E-15	-2.63127	1	1	IL12B	K05425
ENSG00000113303	5.13E-15	-2.63127	1	1	BTNL8	K06712
ENSG00000113327	5.13E-15	-2.63127	1	1	GABRG2	K05186
ENSG00000113361	5.13E-15	-2.63127	1	1	CDH6	K06798
ENSG00000113396	5.13E-15	-2.63127	1	1	SLC27A6	K08749
ENSG00000113430	5.13E-15	-2.63127	1	1	IRX4	_
ENSG00000113492	5.13E-15	-2.63127	1	1	AGXT2	K00827
ENSG00000113494	5.13E-15	-2.63127	1	1	PRLR	K05081
ENSG00000113525	5.13E-15	-2.63127	1	1	IL5	K05428
ENSG00000113532	5.13E-15	-2.63127	1	1	ST8SIA4	K06614
ENSG00000113600	5.13E-15	-2.63127	1	1	C9	K04000
ENSG00000113749	5.13E-15	-2.63127	1	1	HRH2	K04150
ENSG00000113805	5.13E-15	-2.63127	1	1	CNTN3	K06761
ENSG00000113905	5.13E-15	-2.63127	1	1	HRG	K09654
ENSG00000113924	5.13E-15	-2.63127	1	1	HGD	K00451
ENSG00000114113	5.13E-15	-2.63127	1	1	RBP2	K14622
ENSG00000114115	5.13E-15	-2.63127	1	1	RBP1	K06210

ENSG00000114204	5.13E-15	-2.63127	1	1	SERPINI2	_
ENSG00000114248	5.13E-15	-2.63127	1	1	LRRC31	_
ENSG00000114279	5.13E-15	-2.63127	1	1	FGF12	_
ENSG00000114349	5.13E-15	-2.63127	1	1	GNAT1	K04631
ENSG00000114374	5.13E-15	-2.63127	1	1	USP9Y	K11840
ENSG00000114405	5.13E-15	-2.63127	1	1	C3orf14	_
ENSG00000114446	5.13E-15	-2.63127	1	1	IFT57	K04638
ENSG00000114455	5.13E-15	-2.63127	1	1	HHLA2	_
ENSG00000114487	5.13E-15	-2.63127	1	1	MORC1	_
ENSG00000114654	5.13E-15	-2.63127	1	1	EFCC1	_
ENSG00000114656	5.13E-15	-2.63127	1	1	KIAA1257	_
ENSG00000114771	5.13E-15	-2.63127	1	1	AADAC	K13616
ENSG00000114790	5.13E-15	-2.63127	1	1	ARHGEF26	K13744
ENSG00000114805	5.13E-15	-2.63127	1	1	PLCH1	K19006
ENSG00000115138	5.13E-15	-2.63127	1	1	POMC	K05228
ENSG00000115165	5.13E-15	-2.63127	1	1	CYTIP	_
ENSG00000115226	5.13E-15	-2.63127	1	1	FNDC4	_
ENSG00000115232	5.13E-15	-2.63127	1	1	ITGA4	K06483
ENSG00000115263	5.13E-15	-2.63127	1	1	GCG	K05259
ENSG00000115290	5.13E-15	-2.63127	1	1	GRB14	_
ENSG00000115339	5.13E-15	-2.63127	1	1	GALNT3	K00710
ENSG00000115361	5.13E-15	-2.63127	1	1	ACADL	K00255
ENSG00000115457	5.13E-15	-2.63127	1	1	IGFBP2	_
ENSG00000115474	5.13E-15	-2.63127	1	1	KCNJ13	K05006
ENSG00000115488	5.13E-15	-2.63127	1	1	NEU2	K12357
ENSG00000115526	5.13E-15	-2.63127	1	1	CHST10	K09674
ENSG00000115592	5.13E-15	-2.63127	1	1	PRKAG3	K07200
ENSG00000115593	5.13E-15	-2.63127	1	1	SMYD1	K11426
ENSG00000115598	5.13E-15	-2.63127	1	1	IL1RL2	K05172
ENSG00000115604	5.13E-15	-2.63127	1	1	IL18R1	K05173
ENSG00000115607	5.13E-15	-2.63127	1	1	IL18RAP	K05174
ENSG00000115616	5.13E-15	-2.63127	1	1	SLC9A2	K14722
ENSG00000115648	5.13E-15	-2.63127	1	1	MLPH	_
ENSG00000115665	5.13E-15	-2.63127	1	1	SLC5A7	K14387
ENSG00000115850	5.13E-15	-2.63127	1	1	LCT	K01229
ENSG00000115896	5.13E-15	-2.63127	1	1	PLCL1	K15375
ENSG00000115934	5.13E-15	-2.63127	1	1	RP11-153K16.2	_
ENSG00000116014	5.13E-15	-2.63127	1	1	KISS1R	K08374
ENSG00000116031	5.13E-15	-2.63127	1	1	CD207	K06561
ENSG00000116035	5.13E-15	-2.63127	1	1	VAX2	K09318
ENSG00000116039	5.13E-15	-2.63127	1	1	ATP6V1B1	K02147
ENSG00000116106	5.13E-15	-2.63127	1	1	EPHA4	K05105
ENSG00000116132	5.13E-15	-2.63127	1	1	PRRX1	K09329
ENSG00000116147	5.13E-15	-2.63127	1	1	TNR	K06252
ENSG00000116183	5.13E-15	-2.63127	1	1	PAPPA2	_
ENSG00000116218	5.13E-15	-2.63127	1	1	NPHS2	K18268
ENSG00000116329	5.13E-15	-2.63127	1	1	OPRD1	K04213
ENSG00000116703	5.13E-15	-2.63127	1	1	PDC	K08327
ENSG00000116721	5.13E-15	-2.63127	1	1	PRAMEF1	_
ENSG00000116726	5.13E-15	-2.63127	1	1	PRAMEF12	_
ENSG00000116745	5.13E-15	-2.63127	1	1	RPE65	K11158
ENSG00000116748	5.13E-15	-2.63127	1	1	AMPD1	K01490
ENSG00000116824	5.13E-15	-2.63127	1	1	CD2	K06449
ENSG00000116882	5.13E-15	-2.63127	1	1	HAO2	K11517
ENSG00000116962	5.13E-15	-2.63127	1	1	NID1	K06826
ENSG00000116981	5.13E-15	-2.63127	1	1	NT5C1A	K01081
ENSG00000116996	5.13E-15	-2.63127	1	1	ZP4	_
ENSG00000117069	5.13E-15	-2.63127	1	1	ST6GALNAC5	K03375

ENSG00000117090	5.13E-15	-2.63127	1	1	SLAMF1	K06536
ENSG00000117091	5.13E-15	-2.63127	1	1	CD48	K06479
ENSG00000117115	5.13E-15	-2.63127	1	1	PADI2	K01481
ENSG00000117122	5.13E-15	-2.63127	1	1	MFAP2	_
ENSG00000117154	5.13E-15	-2.63127	1	1	IGSF21	_
ENSG00000117281	5.13E-15	-2.63127	1	1	CD160	K06542
ENSG00000117322	5.13E-15	-2.63127	1	1	CR2	K04012
ENSG00000117400	5.13E-15	-2.63127	1	1	MPL	K05082
ENSG00000117501	5.13E-15	-2.63127	1	1	MROH9	_
ENSG00000117507	5.13E-15	-2.63127	1	1	FMO6P	K00485
ENSG00000117519	5.13E-15	-2.63127	1	1	CNN3	_
ENSG00000117560	5.13E-15	-2.63127	1	1	FASLG	K04389
ENSG00000117594	5.13E-15	-2.63127	1	1	HSD11B1	K15680
ENSG00000117595	5.13E-15	-2.63127	1	1	IRF6	K10154
ENSG00000117598	5.13E-15	-2.63127	1	1	LPPR5	K19581
ENSG00000117600	5.13E-15	-2.63127	1	1	LPPR4	K19582
ENSG00000117601	5.13E-15	-2.63127	1	1	SERPINC1	K03911
ENSG00000117707	5.13E-15	-2.63127	1	1	PROX1	_
ENSG00000117834	5.13E-15	-2.63127	1	1	SLC5A9	K14389
ENSG00000118094	5.13E-15	-2.63127	1	1	TREH	K01194
ENSG00000118113	5.13E-15	-2.63127	1	1	MMP8	K01402
ENSG00000118137	5.13E-15	-2.63127	1	1	APOA1	K08757
ENSG00000118231	5.13E-15	-2.63127	1	1	CRYGD	_
ENSG00000118245	5.13E-15	-2.63127	1	1	TNP1	_
ENSG00000118271	5.13E-15	-2.63127	1	1	TTR	_
ENSG00000118298	5.13E-15	-2.63127	1	1	CA14	K01672
ENSG00000118308	5.13E-15	-2.63127	1	1	LRMP	_
ENSG00000118322	5.13E-15	-2.63127	1	1	ATP10B	K01530
ENSG00000118402	5.13E-15	-2.63127	1	1	ELOVL4	K10249
ENSG00000118432	5.13E-15	-2.63127	1	1	CNR1	K04277
ENSG00000118434	5.13E-15	-2.63127	1	1	SPACA1	_
ENSG00000118491	5.13E-15	-2.63127	1	1	ZC2HC1B	K14798
ENSG00000118492	5.13E-15	-2.63127	1	1	ADGB	_
ENSG00000118507	5.13E-15	-2.63127	1	1	AKAP7	K16524
ENSG00000118596	5.13E-15	-2.63127	1	1	SLC16A7	K08184
ENSG00000118702	5.13E-15	-2.63127	1	1	GHRH	K05260
ENSG00000118729	5.13E-15	-2.63127	1	1	CASQ2	_
ENSG00000118733	5.13E-15	-2.63127	1	1	OLFM3	_
ENSG00000118903	5.13E-15	-2.63127	1	1	BTF3P11	K01527
ENSG00000118971	5.13E-15	-2.63127	1	1	CCND2	K10151
ENSG00000118972	5.13E-15	-2.63127	1	1	FGF23	K04358
ENSG00000118976	5.13E-15	-2.63127	1	1	HIN1L	K13718
ENSG00000119121	5.13E-15	-2.63127	1	1	TRPM6	K04981
ENSG00000119147	5.13E-15	-2.63127	1	1	C2orf40	_
ENSG00000119440	5.13E-15	-2.63127	1	1	LCN1P1	_
ENSG00000119457	5.13E-15	-2.63127	1	1	SLC46A2	K14614
ENSG00000119535	5.13E-15	-2.63127	1	1	CSF3R	K05061
ENSG00000119614	5.13E-15	-2.63127	1	1	VSX2	K09336
ENSG00000119660	5.13E-15	-2.63127	1	1	RP11-293M10.4	_
ENSG00000119698	5.13E-15	-2.63127	1	1	PPP4R4	K15426
ENSG00000119714	5.13E-15	-2.63127	1	1	GPR68	_
ENSG00000119715	5.13E-15	-2.63127	1	1	ESRRB	K08553
ENSG00000119782	5.13E-15	-2.63127	1	1	FKBP1B	K09568
ENSG00000119866	5.13E-15	-2.63127	1	1	BCL11A	_
ENSG00000119888	5.13E-15	-2.63127	1	1	EPCAM	K06737
ENSG00000119913	5.13E-15	-2.63127	1	1	TECTB	_
ENSG00000119919	5.13E-15	-2.63127	1	1	NKX2-3	K09343
ENSG00000119946	5.13E-15	-2.63127	1	1	CNNM1	K16302

ENSG00000119973	5.13E-15	-2.63127	1	1	PRLHR	K04314
ENSG00000120054	5.13E-15	-2.63127	1	1	CPN1	K01292
ENSG00000120057	5.13E-15	-2.63127	1	1	SFRP5	K02222
ENSG00000120068	5.13E-15	-2.63127	1	1	HOXB8	K09308
ENSG00000120094	5.13E-15	-2.63127	1	1	HOXB1	K09301
ENSG00000120149	5.13E-15	-2.63127	1	1	MSX2	K09341
ENSG00000120210	5.13E-15	-2.63127	1	1	INSL6	K05255
ENSG00000120235	5.13E-15	-2.63127	1	1	IFNA6	K05414
ENSG00000120251	5.13E-15	-2.63127	1	1	GRIA2	K05198
ENSG00000120262	5.13E-15	-2.63127	1	1	CCDC170	K09228
ENSG00000120279	5.13E-15	-2.63127	1	1	MYCT1	_
ENSG00000120280	5.13E-15	-2.63127	1	1	CXorf21	K09654
ENSG00000120289	5.13E-15	-2.63127	1	1	MAGEB4	_
ENSG00000120322	5.13E-15	-2.63127	1	1	PCDHB8	K16494
ENSG00000120324	5.13E-15	-2.63127	1	1	PCDHB10	K16494
ENSG00000120327	5.13E-15	-2.63127	1	1	PCDHB14	K16494
ENSG00000120329	5.13E-15	-2.63127	1	1	SLC25A2	K15101
ENSG00000120341	5.13E-15	-2.63127	1	1	SEC16B	_
ENSG00000120436	5.13E-15	-2.63127	1	1	GPR31	K08406
ENSG00000120440	5.13E-15	-2.63127	1	1	TTLL2	K16600
ENSG00000120457	5.13E-15	-2.63127	1	1	KCNJ5	K04999
ENSG00000120471	5.13E-15	-2.63127	1	1	TP53AIP1	K13773
ENSG00000120498	5.13E-15	-2.63127	1	1	TEX11	_
ENSG00000120555	5.13E-15	-2.63127	1	1	SEPT7P9	K16944
ENSG00000120563	5.13E-15	-2.63127	1	1	LYZL1	K01185
ENSG00000120586	5.13E-15	-2.63127	1	1	MRC1	K06560
ENSG00000120594	5.13E-15	-2.63127	1	1	PLXDC2	K04613
ENSG00000120645	5.13E-15	-2.63127	1	1	IQSEC3	K12495
ENSG00000120659	5.13E-15	-2.63127	1	1	TNFSF11	K05473
ENSG00000120903	5.13E-15	-2.63127	1	1	CHRNA2	K04804
ENSG00000120907	5.13E-15	-2.63127	1	1	ADRA1A	K04135
ENSG00000120915	5.13E-15	-2.63127	1	1	EPHX2	K08726
ENSG00000120952	5.13E-15	-2.63127	1	1	PRAMEF2	_
ENSG00000121068	5.13E-15	-2.63127	1	1	TBX2	K10176
ENSG00000121075	5.13E-15	-2.63127	1	1	TBX4	K10178
ENSG00000121089	5.13E-15	-2.63127	1	1	NACA3P	K03626
ENSG00000121207	5.13E-15	-2.63127	1	1	LRAT	_
ENSG00000121297	5.13E-15	-2.63127	1	1	TSHZ3	K09236
ENSG00000121314	5.13E-15	-2.63127	1	1	TAS2R8	K08474
ENSG00000121316	5.13E-15	-2.63127	1	1	PLBD1	K12795
ENSG00000121318	5.13E-15	-2.63127	1	1	TAS2R10	K08474
ENSG00000121335	5.13E-15	-2.63127	1	1	PRB2	K13911
ENSG00000121351	5.13E-15	-2.63127	1	1	IAPP	K08039
ENSG00000121361	5.13E-15	-2.63127	1	1	KCNJ8	K05001
ENSG00000121377	5.13E-15	-2.63127	1	1	TAS2R7	K08474
ENSG00000121380	5.13E-15	-2.63127	1	1	BCL2L14	K17985
ENSG00000121381	5.13E-15	-2.63127	1	1	TAS2R9	K08474
ENSG00000121388	5.13E-15	-2.63127	1	1	RP11-408E5.4	K16307
ENSG00000121410	5.13E-15	-2.63127	1	1	A1BG	_
ENSG00000121413	5.13E-15	-2.63127	1	1	ZSCAN18	K09230
ENSG00000121440	5.13E-15	-2.63127	1	1	PDZRN3	K15682
ENSG00000121446	5.13E-15	-2.63127	1	1	RGSL1	_
ENSG00000121552	5.13E-15	-2.63127	1	1	CSTA	K13907
ENSG00000121570	5.13E-15	-2.63127	1	1	DPPA4	K17985
ENSG00000121634	5.13E-15	-2.63127	1	1	GJA8	K07617
ENSG00000121742	5.13E-15	-2.63127	1	1	GJB6	K07625
ENSG00000121743	5.13E-15	-2.63127	1	1	GJA3	K07612
ENSG00000121797	5.13E-15	-2.63127	1	1	CCRL2	K08373

ENSG00000121807	5.13E-15	-2.63127	1	1	CCR2	K04177
ENSG00000121853	5.13E-15	-2.63127	1	1	GHSR	K04284
ENSG00000121871	5.13E-15	-2.63127	1	1	SLITRK3	_
ENSG00000121933	5.13E-15	-2.63127	1	1	ADORA3	K04268
ENSG00000122012	5.13E-15	-2.63127	1	1	SV2C	K06258
ENSG00000122025	5.13E-15	-2.63127	1	1	FLT3	K05092
ENSG00000122043	5.13E-15	-2.63127	1	1	LINC00544	_
ENSG00000122121	5.13E-15	-2.63127	1	1	XPNPEP2	K14208
ENSG00000122122	5.13E-15	-2.63127	1	1	SASH3	_
ENSG00000122133	5.13E-15	-2.63127	1	1	PAEP	_
ENSG00000122136	5.13E-15	-2.63127	1	1	OBP2A	_
ENSG00000122145	5.13E-15	-2.63127	1	1	TBX22	K10186
ENSG00000122176	5.13E-15	-2.63127	1	1	FMOD	K08121
ENSG00000122180	5.13E-15	-2.63127	1	1	MYOG	K18483
ENSG00000122194	5.13E-15	-2.63127	1	1	PLG	K01315
ENSG00000122223	5.13E-15	-2.63127	1	1	CD244	K06582
ENSG00000122224	5.13E-15	-2.63127	1	1	LY9	K06570
ENSG00000122254	5.13E-15	-2.63127	1	1	HS3ST2	K07808
ENSG00000122304	5.13E-15	-2.63127	1	1	PRM2	_
ENSG00000122375	5.13E-15	-2.63127	1	1	OPN4	K04255
ENSG00000122543	5.13E-15	-2.63127	1	1	OCM	_
ENSG00000122548	5.13E-15	-2.63127	1	1	KIAA0087	K02927
ENSG00000122574	5.13E-15	-2.63127	1	1	WIPF3	K02927
ENSG00000122584	5.13E-15	-2.63127	1	1	NXPH1	K16656
ENSG00000122585	5.13E-15	-2.63127	1	1	NPY	K05232
ENSG00000122592	5.13E-15	-2.63127	1	1	HOXA7	K09307
ENSG00000122679	5.13E-15	-2.63127	1	1	RAMP3	K08449
ENSG00000122691	5.13E-15	-2.63127	1	1	TWIST1	K09069
ENSG00000122694	5.13E-15	-2.63127	1	1	GLIPR2	_
ENSG00000122728	5.13E-15	-2.63127	1	1	TAF1L	K03125
ENSG00000122756	5.13E-15	-2.63127	1	1	CNTFR	K05059
ENSG00000122824	5.13E-15	-2.63127	1	1	NUDT10	K07766
ENSG00000122852	5.13E-15	-2.63127	1	1	SFTPA1	K10067
ENSG00000122859	5.13E-15	-2.63127	1	1	NEUROG3	K08028
ENSG00000122872	5.13E-15	-2.63127	1	1	ARL4P	K07945
ENSG00000123096	5.13E-15	-2.63127	1	1	SSPN	K03730
ENSG00000123119	5.13E-15	-2.63127	1	1	NECAB1	_
ENSG00000123165	5.13E-15	-2.63127	1	1	ACTRT1	_
ENSG00000123171	5.13E-15	-2.63127	1	1	CCDC70	_
ENSG00000123243	5.13E-15	-2.63127	1	1	ITIH5	_
ENSG00000123307	5.13E-15	-2.63127	1	1	NEUROD4	K09079
ENSG00000123338	5.13E-15	-2.63127	1	1	NCKAP1L	K05750
ENSG00000123360	5.13E-15	-2.63127	1	1	PDE1B	K13755
ENSG00000123407	5.13E-15	-2.63127	1	1	HOXC12	K09297
ENSG00000123447	5.13E-15	-2.63127	1	1	TYRL	K00505
ENSG00000123454	5.13E-15	-2.63127	1	1	DBH	K00503
ENSG00000123496	5.13E-15	-2.63127	1	1	IL13RA2	K05077
ENSG00000123560	5.13E-15	-2.63127	1	1	PLP1	K17271
ENSG00000123561	5.13E-15	-2.63127	1	1	SERPINA7	K04525
ENSG00000123569	5.13E-15	-2.63127	1	1	H2BFWT	K11252
ENSG00000123570	5.13E-15	-2.63127	1	1	RAB9B	K07900
ENSG00000123572	5.13E-15	-2.63127	1	1	NRK	K16313
ENSG00000123576	5.13E-15	-2.63127	1	1	ESX1	K18491
ENSG00000123584	5.13E-15	-2.63127	1	1	MAGEA9B	_
ENSG00000123594	5.13E-15	-2.63127	1	1	ATXN3L	K11863
ENSG00000123612	5.13E-15	-2.63127	1	1	ACVR1C	K13568
ENSG00000123689	5.13E-15	-2.63127	1	1	G0S2	_
ENSG00000123870	5.13E-15	-2.63127	1	1	ZNF137P	K09228

ENSG00000124003	5.13E-15	-2.63127	1	1	MOGAT1	K14458
ENSG00000124019	5.13E-15	-2.63127	1	1	FAM124B	_
ENSG00000124089	5.13E-15	-2.63127	1	1	MC3R	K04201
ENSG00000124097	5.13E-15	-2.63127	1	1	HMGB1P1	K10802
ENSG00000124143	5.13E-15	-2.63127	1	1	ARHGAP40	_
ENSG00000124157	5.13E-15	-2.63127	1	1	SEMG2	_
ENSG00000124196	5.13E-15	-2.63127	1	1	GTSF1L	_
ENSG00000124208	5.13E-15	-2.63127	1	1	TMEM189-UBE2V1	K10704
ENSG00000124227	5.13E-15	-2.63127	1	1	ANKRD60	_
ENSG00000124233	5.13E-15	-2.63127	1	1	SEMG1	_
ENSG00000124237	5.13E-15	-2.63127	1	1	C20orf85	_
ENSG00000124249	5.13E-15	-2.63127	1	1	KCNK15	K04923
ENSG00000124253	5.13E-15	-2.63127	1	1	PCK1	K01596
ENSG00000124256	5.13E-15	-2.63127	1	1	ZBP1	K12965
ENSG00000124260	5.13E-15	-2.63127	1	1	MAGEA10	_
ENSG00000124302	5.13E-15	-2.63127	1	1	CHST8	K09672
ENSG00000124313	5.13E-15	-2.63127	1	1	IQSEC2	K12495
ENSG00000124334	5.13E-15	-2.63127	1	1	IL9R	K05073
ENSG00000124343	5.13E-15	-2.63127	1	1	XG	_
ENSG00000124391	5.13E-15	-2.63127	1	1	IL17C	K05491
ENSG00000124440	5.13E-15	-2.63127	1	1	HIF3A	K09096
ENSG00000124469	5.13E-15	-2.63127	1	1	CEACAM8	K06499
ENSG00000124479	5.13E-15	-2.63127	1	1	NDP	_
ENSG00000124490	5.13E-15	-2.63127	1	1	CRISP2	_
ENSG00000124507	5.13E-15	-2.63127	1	1	PACSIN1	_
ENSG00000124564	5.13E-15	-2.63127	1	1	SLC17A3	K12300
ENSG00000124568	5.13E-15	-2.63127	1	1	SLC17A1	K12300
ENSG00000124575	5.13E-15	-2.63127	1	1	HIST1H1D	K11275
ENSG00000124578	5.13E-15	-2.63127	1	1	HIST1H4G	K11254
ENSG00000124610	5.13E-15	-2.63127	1	1	HIST1H1A	K11275
ENSG00000124613	5.13E-15	-2.63127	1	1	ZNF391	_
ENSG00000124615	5.13E-15	-2.63127	1	1	MOCS1	K03639
ENSG00000124678	5.13E-15	-2.63127	1	1	TCP11	_
ENSG00000124721	5.13E-15	-2.63127	1	1	DNAH8	K10408
ENSG00000124780	5.13E-15	-2.63127	1	1	KCNK17	K04925
ENSG00000124785	5.13E-15	-2.63127	1	1	NRN1	_
ENSG00000124812	5.13E-15	-2.63127	1	1	CRISP1	_
ENSG00000124818	5.13E-15	-2.63127	1	1	OPN5	K08385
ENSG00000124827	5.13E-15	-2.63127	1	1	GCM2	_
ENSG00000124875	5.13E-15	-2.63127	1	1	CXCL6	K05506
ENSG00000124900	5.13E-15	-2.63127	1	1	TRIM51	K12019
ENSG00000124915	5.13E-15	-2.63127	1	1	RP11-467L20.10	_
ENSG00000124939	5.13E-15	-2.63127	1	1	SCGB2A1	_
ENSG00000125046	5.13E-15	-2.63127	1	1	SSUH2	_
ENSG00000125084	5.13E-15	-2.63127	1	1	WNT1	K03209
ENSG00000125144	5.13E-15	-2.63127	1	1	MT1G	K14739
ENSG00000125255	5.13E-15	-2.63127	1	1	SLC10A2	K14342
ENSG00000125285	5.13E-15	-2.63127	1	1	SOX21	K09267
ENSG00000125355	5.13E-15	-2.63127	1	1	TMEM255A	_
ENSG00000125363	5.13E-15	-2.63127	1	1	AMELX	_
ENSG00000125384	5.13E-15	-2.63127	1	1	PTGER2	K04259
ENSG00000125385	5.13E-15	-2.63127	1	1	RP11-297B17.2	K02912
ENSG00000125398	5.13E-15	-2.63127	1	1	SOX9	K18435
ENSG00000125430	5.13E-15	-2.63127	1	1	HS3ST3B1	K07809
ENSG00000125492	5.13E-15	-2.63127	1	1	BARHL1	K09360
ENSG00000125498	5.13E-15	-2.63127	1	1	KIR2DL1	K07981
ENSG00000125505	5.13E-15	-2.63127	1	1	MBOAT7	K13516
ENSG00000125514	5.13E-15	-2.63127	1	1	LINC00029	_

ENSG00000125522	5.13E-15	-2.63127	1	1	NPBWR2	K08377
ENSG00000125533	5.13E-15	-2.63127	1	1	BHLHE23	K09086
ENSG00000125551	5.13E-15	-2.63127	1	1	PLGLB2	K01315
ENSG00000125695	5.13E-15	-2.63127	1	1	RP11-51F16.8	K08271
ENSG00000125780	5.13E-15	-2.63127	1	1	TGM3	K05620
ENSG00000125788	5.13E-15	-2.63127	1	1	DEFB126	_
ENSG00000125798	5.13E-15	-2.63127	1	1	FOXA2	K08035
ENSG00000125804	5.13E-15	-2.63127	1	1	FAM182A	_
ENSG00000125810	5.13E-15	-2.63127	1	1	CD93	K06702
ENSG00000125813	5.13E-15	-2.63127	1	1	PAX1	K09382
ENSG00000125815	5.13E-15	-2.63127	1	1	CST8	K13904
ENSG00000125816	5.13E-15	-2.63127	1	1	NKX2-4	K09344
ENSG00000125820	5.13E-15	-2.63127	1	1	NKX2-2	K08029
ENSG00000125823	5.13E-15	-2.63127	1	1	CSTL1	_
ENSG00000125831	5.13E-15	-2.63127	1	1	CST11	K13906
ENSG00000125850	5.13E-15	-2.63127	1	1	OVOL2	K09216
ENSG00000125851	5.13E-15	-2.63127	1	1	PCSK2	K01360
ENSG00000125861	5.13E-15	-2.63127	1	1	GFRA4	_
ENSG00000125869	5.13E-15	-2.63127	1	1	LAMP5	_
ENSG00000125878	5.13E-15	-2.63127	1	1	TCF15	K09070
ENSG00000125879	5.13E-15	-2.63127	1	1	OTOR	_
ENSG00000125888	5.13E-15	-2.63127	1	1	BANF2	_
ENSG00000125899	5.13E-15	-2.63127	1	1	C20orf187	_
ENSG00000125900	5.13E-15	-2.63127	1	1	SIRPD	K16827
ENSG00000125903	5.13E-15	-2.63127	1	1	DEFB129	_
ENSG00000125954	5.13E-15	-2.63127	1	1	CHURC1-FNTB	K05954
ENSG00000125962	5.13E-15	-2.63127	1	1	ARMCX5	K14284
ENSG00000125975	5.13E-15	-2.63127	1	1	C20orf173	_
ENSG00000125998	5.13E-15	-2.63127	1	1	FAM83C	_
ENSG00000125999	5.13E-15	-2.63127	1	1	BPIFB1	K02927
ENSG00000126016	5.13E-15	-2.63127	1	1	AMOT	K16819
ENSG00000126233	5.13E-15	-2.63127	1	1	SLURP1	_
ENSG00000126251	5.13E-15	-2.63127	1	1	GPR42	K04326
ENSG00000126259	5.13E-15	-2.63127	1	1	KIRREL2	_
ENSG00000126262	5.13E-15	-2.63127	1	1	FFAR2	K04328
ENSG00000126266	5.13E-15	-2.63127	1	1	FFAR1	K04325
ENSG00000126337	5.13E-15	-2.63127	1	1	KRT36	K07604
ENSG00000126542	5.13E-15	-2.63127	1	1	PTPN20CP	K18039
ENSG00000126545	5.13E-15	-2.63127	1	1	CSN1S1	K17281
ENSG00000126549	5.13E-15	-2.63127	1	1	STATH	K13914
ENSG00000126550	5.13E-15	-2.63127	1	1	HTN1	K13913
ENSG00000126733	5.13E-15	-2.63127	1	1	DACH2	_
ENSG00000126752	5.13E-15	-2.63127	1	1	SSX1	K15624
ENSG00000126785	5.13E-15	-2.63127	1	1	RHOJ	K07864
ENSG00000126856	5.13E-15	-2.63127	1	1	PRDM7	K09228
ENSG00000126947	5.13E-15	-2.63127	1	1	ARMCX1	_
ENSG00000126970	5.13E-15	-2.63127	1	1	ZC4H2	K05255
ENSG00000127074	5.13E-15	-2.63127	1	1	RGS13	K16449
ENSG00000127152	5.13E-15	-2.63127	1	1	BCL11B	K09229
ENSG00000127252	5.13E-15	-2.63127	1	1	HRASLS	_
ENSG00000127318	5.13E-15	-2.63127	1	1	IL22	K05445
ENSG00000127377	5.13E-15	-2.63127	1	1	CRYGN	_
ENSG00000127412	5.13E-15	-2.63127	1	1	TRPV5	K04974
ENSG00000127472	5.13E-15	-2.63127	1	1	PLA2G5	K01047
ENSG00000127515	5.13E-15	-2.63127	1	1	OR7A10	K04257
ENSG00000127529	5.13E-15	-2.63127	1	1	OR7C2	K04257
ENSG00000127530	5.13E-15	-2.63127	1	1	OR7C1	K04257
ENSG00000127743	5.13E-15	-2.63127	1	1	IL17B	K05490

ENSG00000127780	5.13E-15	-2.63127	1	1	OR1E2	K04257
ENSG00000127831	5.13E-15	-2.63127	1	1	VIL1	K05761
ENSG00000127863	5.13E-15	-2.63127	1	1	TNFRSF19	K05155
ENSG00000127903	5.13E-15	-2.63127	1	1	ZNF835	K17985
ENSG00000127928	5.13E-15	-2.63127	1	1	GNGT1	K04548
ENSG00000127951	5.13E-15	-2.63127	1	1	FGL2	K12581
ENSG00000128040	5.13E-15	-2.63127	1	1	SPINK2	_
ENSG00000128045	5.13E-15	-2.63127	1	1	RASL11B	K07853
ENSG00000128052	5.13E-15	-2.63127	1	1	KDR	K05098
ENSG00000128253	5.13E-15	-2.63127	1	1	RFPL2	_
ENSG00000128322	5.13E-15	-2.63127	1	1	IGLL1	K06554
ENSG00000128383	5.13E-15	-2.63127	1	1	APOBEC3A	_
ENSG00000128408	5.13E-15	-2.63127	1	1	RIBC2	_
ENSG00000128438	5.13E-15	-2.63127	1	1	TBC1D27	K12581
ENSG00000128519	5.13E-15	-2.63127	1	1	TAS2R16	K08474
ENSG00000128602	5.13E-15	-2.63127	1	1	SMO	K06226
ENSG00000128610	5.13E-15	-2.63127	1	1	FEZF1	_
ENSG00000128645	5.13E-15	-2.63127	1	1	HOXD1	K09301
ENSG00000128655	5.13E-15	-2.63127	1	1	PDE11A	K13298
ENSG00000128683	5.13E-15	-2.63127	1	1	GAD1	K01580
ENSG00000128714	5.13E-15	-2.63127	1	1	HOXD13	K09298
ENSG00000128739	5.13E-15	-2.63127	1	1	SNRPN	K11100
ENSG00000128802	5.13E-15	-2.63127	1	1	GDF2	K05503
ENSG00000128805	5.13E-15	-2.63127	1	1	ARHGAP22	_
ENSG00000128815	5.13E-15	-2.63127	1	1	WDFY4	_
ENSG00000129152	5.13E-15	-2.63127	1	1	MYOD1	K09064
ENSG00000129159	5.13E-15	-2.63127	1	1	KCNC1	K04887
ENSG00000129170	5.13E-15	-2.63127	1	1	CSRP3	K09377
ENSG00000129221	5.13E-15	-2.63127	1	1	AIPL1	K17767
ENSG00000129277	5.13E-15	-2.63127	1	1	CCL4	K12964
ENSG00000129295	5.13E-15	-2.63127	1	1	LRRC6	K19753
ENSG00000129437	5.13E-15	-2.63127	1	1	KLK14	K09622
ENSG00000129450	5.13E-15	-2.63127	1	1	SIGLEC9	K06740
ENSG00000129451	5.13E-15	-2.63127	1	1	KLK10	K09619
ENSG00000129465	5.13E-15	-2.63127	1	1	RIPK3	K08847
ENSG00000129467	5.13E-15	-2.63127	1	1	ADCY4	K08044
ENSG00000129816	5.13E-15	-2.63127	1	1	TTTY1B	_
ENSG00000129824	5.13E-15	-2.63127	1	1	RPS4Y1	K02987
ENSG00000129845	5.13E-15	-2.63127	1	1	TTTY1	_
ENSG00000129862	5.13E-15	-2.63127	1	1	VCY1B	_
ENSG00000129864	5.13E-15	-2.63127	1	1	VCY	_
ENSG00000129873	5.13E-15	-2.63127	1	1	CDY2B	K00653
ENSG00000129946	5.13E-15	-2.63127	1	1	SHC2	K17447
ENSG00000129965	5.13E-15	-2.63127	1	1	INS-IGF2	K04526
ENSG00000130032	5.13E-15	-2.63127	1	1	PRRG3	K04949
ENSG00000130035	5.13E-15	-2.63127	1	1	GALNT8	_
ENSG00000130037	5.13E-15	-2.63127	1	1	KCNA5	K04878
ENSG00000130038	5.13E-15	-2.63127	1	1	EFCAB4B	K17199
ENSG00000130054	5.13E-15	-2.63127	1	1	FAM155B	_
ENSG00000130167	5.13E-15	-2.63127	1	1	TSPAN16	K17357
ENSG00000130176	5.13E-15	-2.63127	1	1	CNN1	_
ENSG00000130182	5.13E-15	-2.63127	1	1	ZSCAN10	K09230
ENSG00000130208	5.13E-15	-2.63127	1	1	APOC1	_
ENSG00000130222	5.13E-15	-2.63127	1	1	GADD45G	K04402
ENSG00000130224	5.13E-15	-2.63127	1	1	LRCH2	_
ENSG00000130226	5.13E-15	-2.63127	1	1	DPP6	_
ENSG00000130283	5.13E-15	-2.63127	1	1	GDF1	K05495
ENSG00000130294	5.13E-15	-2.63127	1	1	KIF1A	K10392

ENSG00000130349	5.13E-15	-2.63127	1	1	C6orf203	K15696
ENSG00000130368	5.13E-15	-2.63127	1	1	MAS1	K04303
ENSG00000130383	5.13E-15	-2.63127	1	1	FUT5	K07633
ENSG00000130385	5.13E-15	-2.63127	1	1	BMP15	K05498
ENSG00000130538	5.13E-15	-2.63127	1	1	OR11H1	K04257
ENSG00000130540	5.13E-15	-2.63127	1	1	SULT4A1	K11823
ENSG00000130561	5.13E-15	-2.63127	1	1	SAG	K19627
ENSG00000130595	5.13E-15	-2.63127	1	1	TNNT3	K12046
ENSG00000130598	5.13E-15	-2.63127	1	1	TNNI2	K12043
ENSG00000130656	5.13E-15	-2.63127	1	1	HBZ	K13826
ENSG00000130700	5.13E-15	-2.63127	1	1	GATA5	K17896
ENSG00000130775	5.13E-15	-2.63127	1	1	THEMIS2	_
ENSG00000130830	5.13E-15	-2.63127	1	1	MPP1	_
ENSG00000130950	5.13E-15	-2.63127	1	1	NUTM2F	_
ENSG00000130988	5.13E-15	-2.63127	1	1	RGN	K01053
ENSG00000131002	5.13E-15	-2.63127	1	1	TXLNG2P	_
ENSG00000131007	5.13E-15	-2.63127	1	1	TTTY9B	_
ENSG00000131042	5.13E-15	-2.63127	1	1	LILRB2	K06512
ENSG00000131050	5.13E-15	-2.63127	1	1	BPIFA2	_
ENSG00000131055	5.13E-15	-2.63127	1	1	COX4I2	K02263
ENSG00000131059	5.13E-15	-2.63127	1	1	BPIFA3	_
ENSG00000131068	5.13E-15	-2.63127	1	1	DEFB118	_
ENSG00000131089	5.13E-15	-2.63127	1	1	ARHGEF9	K05769
ENSG00000131126	5.13E-15	-2.63127	1	1	TEX101	_
ENSG00000131127	5.13E-15	-2.63127	1	1	ZNF141	K09228
ENSG00000131142	5.13E-15	-2.63127	1	1	CCL25	K13072
ENSG00000131183	5.13E-15	-2.63127	1	1	SLC34A1	K14683
ENSG00000131233	5.13E-15	-2.63127	1	1	GJA9	K07618
ENSG00000131264	5.13E-15	-2.63127	1	1	CDX4	K09312
ENSG00000131398	5.13E-15	-2.63127	1	1	KCNC3	K04889
ENSG00000131400	5.13E-15	-2.63127	1	1	NAPSA	K08565
ENSG00000131401	5.13E-15	-2.63127	1	1	NAPSB	K08565
ENSG00000131477	5.13E-15	-2.63127	1	1	RAMP2	K08448
ENSG00000131482	5.13E-15	-2.63127	1	1	G6PC	K01084
ENSG00000131538	5.13E-15	-2.63127	1	1	TTTY6	_
ENSG00000131548	5.13E-15	-2.63127	1	1	TTTY6B	_
ENSG00000131686	5.13E-15	-2.63127	1	1	CA6	K01672
ENSG00000131721	5.13E-15	-2.63127	1	1	RHOXF2	_
ENSG00000131738	5.13E-15	-2.63127	1	1	KRT33B	K07604
ENSG00000131808	5.13E-15	-2.63127	1	1	FSHB	K05250
ENSG00000131831	5.13E-15	-2.63127	1	1	RAI2	_
ENSG00000131864	5.13E-15	-2.63127	1	1	USP29	K11850
ENSG00000131910	5.13E-15	-2.63127	1	1	NR0B2	K08563
ENSG00000131914	5.13E-15	-2.63127	1	1	LIN28A	K18754
ENSG00000131951	5.13E-15	-2.63127	1	1	LRRC9	_
ENSG00000131982	5.13E-15	-2.63127	1	1	UBE2L2	K04552
ENSG00000132122	5.13E-15	-2.63127	1	1	SPATA6	K17532
ENSG00000132139	5.13E-15	-2.63127	1	1	GAS2L2	_
ENSG00000132185	5.13E-15	-2.63127	1	1	FCRLA	_
ENSG00000132205	5.13E-15	-2.63127	1	1	EMILIN2	_
ENSG00000132259	5.13E-15	-2.63127	1	1	CNGA4	K04951
ENSG00000132297	5.13E-15	-2.63127	1	1	HHLA1	_
ENSG00000132321	5.13E-15	-2.63127	1	1	IQCA1	_
ENSG00000132329	5.13E-15	-2.63127	1	1	RAMP1	K08447
ENSG00000132429	5.13E-15	-2.63127	1	1	POPDC3	_
ENSG00000132446	5.13E-15	-2.63127	1	1	FTHL17	K00522
ENSG00000132464	5.13E-15	-2.63127	1	1	ENAM	K12795
ENSG00000132465	5.13E-15	-2.63127	1	1	IGJ	_

ENSG00000132498	5.13E-15	-2.63127	1	1	ANKRD20A3	K17299
ENSG00000132514	5.13E-15	-2.63127	1	1	CLEC10A	K06721
ENSG00000132517	5.13E-15	-2.63127	1	1	SLC52A1	K11447
ENSG00000132518	5.13E-15	-2.63127	1	1	GUCY2D	K12321
ENSG00000132554	5.13E-15	-2.63127	1	1	RGS22	K16449
ENSG00000132563	5.13E-15	-2.63127	1	1	REEP2	K17338
ENSG00000132631	5.13E-15	-2.63127	1	1	SCP2D1	_
ENSG00000132671	5.13E-15	-2.63127	1	1	SSTR4	K04220
ENSG00000132677	5.13E-15	-2.63127	1	1	RHBG	K06580
ENSG00000132681	5.13E-15	-2.63127	1	1	ATP1A4	K01539
ENSG00000132698	5.13E-15	-2.63127	1	1	RAB25	K07906
ENSG00000132703	5.13E-15	-2.63127	1	1	APCS	_
ENSG00000132704	5.13E-15	-2.63127	1	1	FCRL2	K06727
ENSG00000132718	5.13E-15	-2.63127	1	1	SYT11	K17985
ENSG00000132744	5.13E-15	-2.63127	1	1	ACY3	K18458
ENSG00000132746	5.13E-15	-2.63127	1	1	ALDH3B2	K00129
ENSG00000132821	5.13E-15	-2.63127	1	1	VSTM2L	_
ENSG00000132840	5.13E-15	-2.63127	1	1	BHMT2	_
ENSG00000132854	5.13E-15	-2.63127	1	1	KANK4	_
ENSG00000132872	5.13E-15	-2.63127	1	1	SYT4	_
ENSG00000132932	5.13E-15	-2.63127	1	1	ATP8A2	K14802
ENSG00000132958	5.13E-15	-2.63127	1	1	TPTE2	K18079
ENSG00000132970	5.13E-15	-2.63127	1	1	WASF3	K06083
ENSG00000132972	5.13E-15	-2.63127	1	1	RNF17	K18405
ENSG00000132975	5.13E-15	-2.63127	1	1	GPR12	K04315
ENSG00000133019	5.13E-15	-2.63127	1	1	CHRM3	K04131
ENSG00000133048	5.13E-15	-2.63127	1	1	CHI3L1	K17523
ENSG00000133055	5.13E-15	-2.63127	1	1	MYBPH	K12558
ENSG00000133083	5.13E-15	-2.63127	1	1	DCLK1	K08805
ENSG00000133105	5.13E-15	-2.63127	1	1	RXFP2	K04307
ENSG00000133107	5.13E-15	-2.63127	1	1	TRPC4	K04967
ENSG00000133115	5.13E-15	-2.63127	1	1	STOML3	K17286
ENSG00000133116	5.13E-15	-2.63127	1	1	KL	K14756
ENSG00000133124	5.13E-15	-2.63127	1	1	IRS4	K17446
ENSG00000133134	5.13E-15	-2.63127	1	1	BEX2	_
ENSG00000133135	5.13E-15	-2.63127	1	1	RNF128	K10629
ENSG00000133136	5.13E-15	-2.63127	1	1	GNG5P2	K04542
ENSG00000133149	5.13E-15	-2.63127	1	1	TEX13A	_
ENSG00000133169	5.13E-15	-2.63127	1	1	BEX1	K12465
ENSG00000133317	5.13E-15	-2.63127	1	1	LGALS12	K10095
ENSG00000133424	5.13E-15	-2.63127	1	1	LARGE	K09668
ENSG00000133454	5.13E-15	-2.63127	1	1	MYO18B	K10362
ENSG00000133475	5.13E-15	-2.63127	1	1	GGT2	K18592
ENSG00000133519	5.13E-15	-2.63127	1	1	ZDHHC8P1	K18932
ENSG00000133561	5.13E-15	-2.63127	1	1	GIMAP6	_
ENSG00000133574	5.13E-15	-2.63127	1	1	GIMAP4	_
ENSG00000133661	5.13E-15	-2.63127	1	1	SFTPD	K10068
ENSG00000133742	5.13E-15	-2.63127	1	1	CA1	K01672
ENSG00000133800	5.13E-15	-2.63127	1	1	LYVE1	K19012
ENSG00000133878	5.13E-15	-2.63127	1	1	DUSP26	K14165
ENSG00000133937	5.13E-15	-2.63127	1	1	GSC	K09324
ENSG00000133958	5.13E-15	-2.63127	1	1	UNC79	_
ENSG00000133980	5.13E-15	-2.63127	1	1	VRTN	_
ENSG00000134020	5.13E-15	-2.63127	1	1	PEBP4	_
ENSG00000134028	5.13E-15	-2.63127	1	1	ADAMDEC1	_
ENSG00000134115	5.13E-15	-2.63127	1	1	CNTN6	_
ENSG00000134121	5.13E-15	-2.63127	1	1	CHL1	K06758
ENSG00000134184	5.13E-15	-2.63127	1	1	GSTM1	K00799

ENSG00000134193	5.13E-15	-2.63127	1	1	REG4	_
ENSG00000134200	5.13E-15	-2.63127	1	1	TSHB	K05251
ENSG00000134201	5.13E-15	-2.63127	1	1	GSTM5	K00799
ENSG00000134207	5.13E-15	-2.63127	1	1	SYT6	_
ENSG00000134216	5.13E-15	-2.63127	1	1	CHIA	K01183
ENSG00000134240	5.13E-15	-2.63127	1	1	HMGCS2	K01641
ENSG00000134242	5.13E-15	-2.63127	1	1	PTPN22	K18024
ENSG00000134249	5.13E-15	-2.63127	1	1	ADAM30	K08615
ENSG00000134259	5.13E-15	-2.63127	1	1	NGF	K02582
ENSG00000134323	5.13E-15	-2.63127	1	1	MYCN	K09109
ENSG00000134343	5.13E-15	-2.63127	1	1	ANO3	K19498
ENSG00000134389	5.13E-15	-2.63127	1	1	CFHR5	K04004
ENSG00000134438	5.13E-15	-2.63127	1	1	RAX	K09332
ENSG00000134443	5.13E-15	-2.63127	1	1	GRP	K05224
ENSG00000134460	5.13E-15	-2.63127	1	1	IL2RA	K05068
ENSG00000134489	5.13E-15	-2.63127	1	1	HRH4	K04152
ENSG00000134532	5.13E-15	-2.63127	1	1	SOX5	K09269
ENSG00000134538	5.13E-15	-2.63127	1	1	SLCO1B1	K05043
ENSG00000134539	5.13E-15	-2.63127	1	1	KLRD1	K06516
ENSG00000134545	5.13E-15	-2.63127	1	1	KLRC1	K06541
ENSG00000134551	5.13E-15	-2.63127	1	1	PRH2	K13910
ENSG00000134571	5.13E-15	-2.63127	1	1	MYBPC3	K12568
ENSG00000134588	5.13E-15	-2.63127	1	1	USP26	_
ENSG00000134595	5.13E-15	-2.63127	1	1	SOX3	K09267
ENSG00000134612	5.13E-15	-2.63127	1	1	FOLH1B	K14592
ENSG00000134627	5.13E-15	-2.63127	1	1	PIWIL4	K02156
ENSG00000134640	5.13E-15	-2.63127	1	1	MTNR1B	K04286
ENSG00000134716	5.13E-15	-2.63127	1	1	CYP2J2	K07418
ENSG00000134760	5.13E-15	-2.63127	1	1	DSG1	K07596
ENSG00000134762	5.13E-15	-2.63127	1	1	DSC3	K07602
ENSG00000134765	5.13E-15	-2.63127	1	1	DSC1	K07600
ENSG00000134812	5.13E-15	-2.63127	1	1	GIF	K14615
ENSG00000134853	5.13E-15	-2.63127	1	1	PDGFRA	K04363
ENSG00000134873	5.13E-15	-2.63127	1	1	CLDN10	K06087
ENSG00000134917	5.13E-15	-2.63127	1	1	ADAMTS8	K08623
ENSG00000135074	5.13E-15	-2.63127	1	1	ADAM19	K08608
ENSG00000135097	5.13E-15	-2.63127	1	1	MSI1	K14411
ENSG00000135144	5.13E-15	-2.63127	1	1	DTX1	K06058
ENSG00000135220	5.13E-15	-2.63127	1	1	UGT2A3	K00699
ENSG00000135222	5.13E-15	-2.63127	1	1	CSN2	K17107
ENSG00000135298	5.13E-15	-2.63127	1	1	BAI3	K04598
ENSG00000135312	5.13E-15	-2.63127	1	1	HTR1B	K04153
ENSG00000135314	5.13E-15	-2.63127	1	1	KHDC1	K12581
ENSG00000135324	5.13E-15	-2.63127	1	1	MRAP2	_
ENSG00000135333	5.13E-15	-2.63127	1	1	EPHA7	K05108
ENSG00000135355	5.13E-15	-2.63127	1	1	GJA10	K07631
ENSG00000135374	5.13E-15	-2.63127	1	1	ELF5	K17101
ENSG00000135413	5.13E-15	-2.63127	1	1	LACRT	_
ENSG00000135426	5.13E-15	-2.63127	1	1	TESPA1	_
ENSG00000135447	5.13E-15	-2.63127	1	1	PPP1R1A	K08050
ENSG00000135502	5.13E-15	-2.63127	1	1	SLC26A10	K14707
ENSG00000135569	5.13E-15	-2.63127	1	1	TAAR5	K05051
ENSG00000135577	5.13E-15	-2.63127	1	1	NMBR	K04168
ENSG00000135638	5.13E-15	-2.63127	1	1	EMX1	K09317
ENSG00000135697	5.13E-15	-2.63127	1	1	BCMO1	K00515
ENSG00000135773	5.13E-15	-2.63127	1	1	CAPN9	K08578
ENSG00000135824	5.13E-15	-2.63127	1	1	RGS8	K16449
ENSG00000135898	5.13E-15	-2.63127	1	1	GPR55	K08413

ENSG00000135903	5.13E-15	-2.63127	1	1	PAX3	K09381
ENSG00000135917	5.13E-15	-2.63127	1	1	SLC19A3	K14610
ENSG00000135973	5.13E-15	-2.63127	1	1	GPR45	K08409
ENSG00000136099	5.13E-15	-2.63127	1	1	PCDH8	K16499
ENSG00000136110	5.13E-15	-2.63127	1	1	LECT1	_
ENSG00000136155	5.13E-15	-2.63127	1	1	SCEL	_
ENSG00000136167	5.13E-15	-2.63127	1	1	LCP1	K17276
ENSG00000136237	5.13E-15	-2.63127	1	1	RAPGEF5	K08019
ENSG00000136250	5.13E-15	-2.63127	1	1	AOAH	K01065
ENSG00000136297	5.13E-15	-2.63127	1	1	MMD2	K11064
ENSG00000136305	5.13E-15	-2.63127	1	1	CIDEB	K04297
ENSG00000136315	5.13E-15	-2.63127	1	1	RP11-84C10.2	_
ENSG00000136404	5.13E-15	-2.63127	1	1	TM6SF1	_
ENSG00000136449	5.13E-15	-2.63127	1	1	MYCBPAP	K17985
ENSG00000136487	5.13E-15	-2.63127	1	1	GH2	K05438
ENSG00000136488	5.13E-15	-2.63127	1	1	CSH1	K05438
ENSG00000136531	5.13E-15	-2.63127	1	1	SCN2A	K04834
ENSG00000136535	5.13E-15	-2.63127	1	1	TBR1	K10174
ENSG00000136542	5.13E-15	-2.63127	1	1	GALNT5	_
ENSG00000136546	5.13E-15	-2.63127	1	1	SCN7A	K04839
ENSG00000136573	5.13E-15	-2.63127	1	1	BLK	_
ENSG00000136634	5.13E-15	-2.63127	1	1	IL10	K05443
ENSG00000136688	5.13E-15	-2.63127	1	1	IL36G	K05487
ENSG00000136694	5.13E-15	-2.63127	1	1	IL36A	K05484
ENSG00000136695	5.13E-15	-2.63127	1	1	IL36RN	K05483
ENSG00000136696	5.13E-15	-2.63127	1	1	IL36B	K05486
ENSG00000136697	5.13E-15	-2.63127	1	1	IL1F10	K05488
ENSG00000136698	5.13E-15	-2.63127	1	1	CFC1	_
ENSG00000136732	5.13E-15	-2.63127	1	1	GYPC	K06576
ENSG00000136750	5.13E-15	-2.63127	1	1	GAD2	K01580
ENSG00000136834	5.13E-15	-2.63127	1	1	OR1J1	K04257
ENSG00000136839	5.13E-15	-2.63127	1	1	OR13C9	K04257
ENSG00000136866	5.13E-15	-2.63127	1	1	ZFP37	K09228
ENSG00000136928	5.13E-15	-2.63127	1	1	GABBR2	K04615
ENSG00000136929	5.13E-15	-2.63127	1	1	HEMGN	_
ENSG00000136939	5.13E-15	-2.63127	1	1	OR1L4	K04257
ENSG00000136944	5.13E-15	-2.63127	1	1	LMX1B	K09371
ENSG00000136960	5.13E-15	-2.63127	1	1	ENPP2	K01122
ENSG00000137080	5.13E-15	-2.63127	1	1	IFNA21	K05414
ENSG00000137090	5.13E-15	-2.63127	1	1	DMRT1	K19488
ENSG00000137142	5.13E-15	-2.63127	1	1	IGFBPL1	_
ENSG00000137198	5.13E-15	-2.63127	1	1	GMPR	K00364
ENSG00000137204	5.13E-15	-2.63127	1	1	SLC22A7	K08204
ENSG00000137251	5.13E-15	-2.63127	1	1	TINAG	_
ENSG00000137252	5.13E-15	-2.63127	1	1	HCRTR2	K04239
ENSG00000137265	5.13E-15	-2.63127	1	1	IRF4	K09445
ENSG00000137285	5.13E-15	-2.63127	1	1	TUBB2B	K07375
ENSG00000137312	5.13E-15	-2.63127	1	1	FLOT1	K07192
ENSG00000137337	5.13E-15	-2.63127	1	1	MDC1	_
ENSG00000137343	5.13E-15	-2.63127	1	1	ATAT1	K19573
ENSG00000137392	5.13E-15	-2.63127	1	1	CLPS	K14460
ENSG00000137404	5.13E-15	-2.63127	1	1	NRM	_
ENSG00000137441	5.13E-15	-2.63127	1	1	FGFBP2	_
ENSG00000137462	5.13E-15	-2.63127	1	1	TLR2	K10159
ENSG00000137473	5.13E-15	-2.63127	1	1	TTC29	_
ENSG00000137491	5.13E-15	-2.63127	1	1	SLCO2B1	K14352
ENSG00000137558	5.13E-15	-2.63127	1	1	PI15	_
ENSG00000137571	5.13E-15	-2.63127	1	1	SLCO5A1	K14356

ENSG00000137634	5.13E-15	-2.63127	1	1	NXPE4	_
ENSG00000137648	5.13E-15	-2.63127	1	1	TMPRSS4	K09635
ENSG00000137672	5.13E-15	-2.63127	1	1	TRPC6	K04969
ENSG00000137674	5.13E-15	-2.63127	1	1	MMP20	K07999
ENSG00000137675	5.13E-15	-2.63127	1	1	MMP27	K08005
ENSG00000137700	5.13E-15	-2.63127	1	1	SLC37A4	K08171
ENSG00000137707	5.13E-15	-2.63127	1	1	BTG4	K14443
ENSG00000137720	5.13E-15	-2.63127	1	1	C11orf1	K17985
ENSG00000137726	5.13E-15	-2.63127	1	1	FXYD6	K13363
ENSG00000137727	5.13E-15	-2.63127	1	1	ARHGAP20	_
ENSG00000137731	5.13E-15	-2.63127	1	1	FXYD2	K01538
ENSG00000137747	5.13E-15	-2.63127	1	1	TMPRSS13	K09643
ENSG00000137757	5.13E-15	-2.63127	1	1	CASP5	K04395
ENSG00000137766	5.13E-15	-2.63127	1	1	UNC13C	K15293
ENSG00000137808	5.13E-15	-2.63127	1	1	RP11-809H16.2	_
ENSG00000137809	5.13E-15	-2.63127	1	1	ITGA11	K06587
ENSG00000137860	5.13E-15	-2.63127	1	1	SLC28A2	K11536
ENSG00000137875	5.13E-15	-2.63127	1	1	BCL2L10	K18451
ENSG00000138068	5.13E-15	-2.63127	1	1	SULT6B1	_
ENSG00000138083	5.13E-15	-2.63127	1	1	SIX3	K19473
ENSG00000138109	5.13E-15	-2.63127	1	1	CYP2C9	K17719
ENSG00000138115	5.13E-15	-2.63127	1	1	CYP2C8	K17718
ENSG00000138135	5.13E-15	-2.63127	1	1	CH25H	K10223
ENSG00000138136	5.13E-15	-2.63127	1	1	LBX1	K09353
ENSG00000138207	5.13E-15	-2.63127	1	1	RBP4	K18271
ENSG00000138294	5.13E-15	-2.63127	1	1	MSMB	_
ENSG00000138308	5.13E-15	-2.63127	1	1	PLA2G12B	K01047
ENSG00000138311	5.13E-15	-2.63127	1	1	ZNF365	K16737
ENSG00000138379	5.13E-15	-2.63127	1	1	MSTN	K05497
ENSG00000138395	5.13E-15	-2.63127	1	1	CDK15	K15594
ENSG00000138435	5.13E-15	-2.63127	1	1	CHRNA1	K04803
ENSG00000138449	5.13E-15	-2.63127	1	1	SLC40A1	K14685
ENSG00000138472	5.13E-15	-2.63127	1	1	GUCA1C	K08328
ENSG00000138587	5.13E-15	-2.63127	1	1	MNS1	_
ENSG00000138615	5.13E-15	-2.63127	1	1	CILP	_
ENSG00000138622	5.13E-15	-2.63127	1	1	HCN4	K04957
ENSG00000138653	5.13E-15	-2.63127	1	1	NDST4	K02579
ENSG00000138669	5.13E-15	-2.63127	1	1	PRKG2	K19477
ENSG00000138670	5.13E-15	-2.63127	1	1	RASGEF1B	K19477
ENSG00000138675	5.13E-15	-2.63127	1	1	FGF5	K04358
ENSG00000138684	5.13E-15	-2.63127	1	1	IL21	K05434
ENSG00000138722	5.13E-15	-2.63127	1	1	MMRN1	_
ENSG00000138738	5.13E-15	-2.63127	1	1	PRDM5	_
ENSG00000138741	5.13E-15	-2.63127	1	1	TRPC3	K04966
ENSG00000138755	5.13E-15	-2.63127	1	1	CXCL9	K05416
ENSG00000138792	5.13E-15	-2.63127	1	1	ENPEP	K11141
ENSG00000138795	5.13E-15	-2.63127	1	1	LEF1	K04492
ENSG00000138813	5.13E-15	-2.63127	1	1	C4orf17	_
ENSG00000139053	5.13E-15	-2.63127	1	1	PDE6H	K13760
ENSG00000139055	5.13E-15	-2.63127	1	1	ERP27	_
ENSG00000139144	5.13E-15	-2.63127	1	1	PIK3C2G	K00923
ENSG00000139151	5.13E-15	-2.63127	1	1	PLCZ1	K05861
ENSG00000139155	5.13E-15	-2.63127	1	1	SLCO1C1	K08747
ENSG00000139174	5.13E-15	-2.63127	1	1	PRICKLE1	K04511
ENSG00000139220	5.13E-15	-2.63127	1	1	PPFIA2	_
ENSG00000139223	5.13E-15	-2.63127	1	1	ANP32D	K18646
ENSG00000139287	5.13E-15	-2.63127	1	1	TPH2	K00502
ENSG00000139304	5.13E-15	-2.63127	1	1	PTPRQ	K16910

ENSG00000139330	5.13E-15	-2.63127	1	1	KERA	K08123
ENSG00000139352	5.13E-15	-2.63127	1	1	ASCL1	K09067
ENSG00000139364	5.13E-15	-2.63127	1	1	TMEM132B	K17599
ENSG00000139445	5.13E-15	-2.63127	1	1	FOXN4	K09407
ENSG00000139515	5.13E-15	-2.63127	1	1	PDX1	K07594
ENSG00000139574	5.13E-15	-2.63127	1	1	NPFF	K05247
ENSG00000139610	5.13E-15	-2.63127	1	1	CELA1	K01326
ENSG00000139648	5.13E-15	-2.63127	1	1	KRT71	K07605
ENSG00000139714	5.13E-15	-2.63127	1	1	MORN3	_
ENSG00000139737	5.13E-15	-2.63127	1	1	SLAIN1	K16580
ENSG00000139780	5.13E-15	-2.63127	1	1	METTL21C	_
ENSG00000139797	5.13E-15	-2.63127	1	1	RNF113B	K15696
ENSG00000139910	5.13E-15	-2.63127	1	1	NOVA1	K14944
ENSG00000139970	5.13E-15	-2.63127	1	1	RTN1	_
ENSG00000140015	5.13E-15	-2.63127	1	1	KCNH5	K04908
ENSG00000140030	5.13E-15	-2.63127	1	1	GPR65	K08416
ENSG00000140067	5.13E-15	-2.63127	1	1	FAM181A	_
ENSG00000140090	5.13E-15	-2.63127	1	1	SLC24A4	K13752
ENSG00000140093	5.13E-15	-2.63127	1	1	SERPINA10	K04525
ENSG00000140254	5.13E-15	-2.63127	1	1	DUOXA1	K17233
ENSG00000140274	5.13E-15	-2.63127	1	1	DUOXA2	K17232
ENSG00000140279	5.13E-15	-2.63127	1	1	DUOX2	K13411
ENSG00000140379	5.13E-15	-2.63127	1	1	BCL2A1	K02162
ENSG00000140470	5.13E-15	-2.63127	1	1	ADAMTS17	K08631
ENSG00000140478	5.13E-15	-2.63127	1	1	GOLGA6D	K10595
ENSG00000140488	5.13E-15	-2.63127	1	1	CELF6	K13207
ENSG00000140506	5.13E-15	-2.63127	1	1	LMAN1L	K10081
ENSG00000140522	5.13E-15	-2.63127	1	1	RLBP1	K19625
ENSG00000140538	5.13E-15	-2.63127	1	1	NTRK3	K05101
ENSG00000140600	5.13E-15	-2.63127	1	1	SH3GL3	K11247
ENSG00000140682	5.13E-15	-2.63127	1	1	TGFB1I1	_
ENSG00000140798	5.13E-15	-2.63127	1	1	ABCC12	K05672
ENSG00000140807	5.13E-15	-2.63127	1	1	NKD1	K03213
ENSG00000140835	5.13E-15	-2.63127	1	1	CHST4	K04746
ENSG00000140937	5.13E-15	-2.63127	1	1	CDH11	K06803
ENSG00000140945	5.13E-15	-2.63127	1	1	CDH13	K06808
ENSG00000140955	5.13E-15	-2.63127	1	1	ADAD2	_
ENSG00000140968	5.13E-15	-2.63127	1	1	IRF8	K10155
ENSG00000141040	5.13E-15	-2.63127	1	1	ZNF287	K09229
ENSG00000141096	5.13E-15	-2.63127	1	1	DPEP3	K01273
ENSG00000141161	5.13E-15	-2.63127	1	1	UNC45B	_
ENSG00000141194	5.13E-15	-2.63127	1	1	OR4D1	K04257
ENSG00000141200	5.13E-15	-2.63127	1	1	KIF2B	K10393
ENSG00000141255	5.13E-15	-2.63127	1	1	SPATA22	_
ENSG00000141293	5.13E-15	-2.63127	1	1	SKAP1	K17699
ENSG00000141314	5.13E-15	-2.63127	1	1	RHBDL3	K02857
ENSG00000141316	5.13E-15	-2.63127	1	1	SPACA3	_
ENSG00000141338	5.13E-15	-2.63127	1	1	ABCA8	K05650
ENSG00000141371	5.13E-15	-2.63127	1	1	C17orf64	_
ENSG00000141431	5.13E-15	-2.63127	1	1	ASXL3	K11471
ENSG00000141433	5.13E-15	-2.63127	1	1	ADCYAP1	K05262
ENSG00000141437	5.13E-15	-2.63127	1	1	SLC25A52	_
ENSG00000141485	5.13E-15	-2.63127	1	1	SLC13A5	K14445
ENSG00000141506	5.13E-15	-2.63127	1	1	PIK3R5	K02649
ENSG00000141665	5.13E-15	-2.63127	1	1	FBXO15	K10298
ENSG00000141668	5.13E-15	-2.63127	1	1	CBLN2	_
ENSG00000141744	5.13E-15	-2.63127	1	1	PNMT	K00553
ENSG00000141753	5.13E-15	-2.63127	1	1	IGFBP4	_

ENSG00000141946	5.13E-15	-2.63127	1	1	ZIM3	K09228
ENSG00000141977	5.13E-15	-2.63127	1	1	CIB3	_
ENSG00000141979	5.13E-15	-2.63127	1	1	CTD-3222D19.2	K15169
ENSG00000142025	5.13E-15	-2.63127	1	1	DMRTC2	K19493
ENSG00000142149	5.13E-15	-2.63127	1	1	HUNK	K08797
ENSG00000142163	5.13E-15	-2.63127	1	1	OR1E3	K04257
ENSG00000142273	5.13E-15	-2.63127	1	1	CBLC	K04707
ENSG00000142484	5.13E-15	-2.63127	1	1	TM4SF5	_
ENSG00000142511	5.13E-15	-2.63127	1	1	GPR32	K08381
ENSG00000142512	5.13E-15	-2.63127	1	1	SIGLEC10	K06749
ENSG00000142515	5.13E-15	-2.63127	1	1	KLK3	K01351
ENSG00000142539	5.13E-15	-2.63127	1	1	CTD-2545M3.6	K09439
ENSG00000142606	5.13E-15	-2.63127	1	1	MMEL1	K08635
ENSG00000142698	5.13E-15	-2.63127	1	1	C1orf94	_
ENSG00000142748	5.13E-15	-2.63127	1	1	FCN3	K10104
ENSG00000142789	5.13E-15	-2.63127	1	1	CELA3A	K01345
ENSG00000142794	5.13E-15	-2.63127	1	1	NBPF3	_
ENSG00000142973	5.13E-15	-2.63127	1	1	CYP4B1	K07426
ENSG00000143006	5.13E-15	-2.63127	1	1	DMRTB1	K19492
ENSG00000143032	5.13E-15	-2.63127	1	1	BARHL2	K09360
ENSG00000143105	5.13E-15	-2.63127	1	1	KCNA10	K04881
ENSG00000143125	5.13E-15	-2.63127	1	1	PROK1	_
ENSG00000143127	5.13E-15	-2.63127	1	1	ITGA10	K06586
ENSG00000143167	5.13E-15	-2.63127	1	1	GPA33	K06790
ENSG00000143171	5.13E-15	-2.63127	1	1	RXRG	K08526
ENSG00000143194	5.13E-15	-2.63127	1	1	MAEL	K18411
ENSG00000143195	5.13E-15	-2.63127	1	1	ILDR2	K12756
ENSG00000143196	5.13E-15	-2.63127	1	1	DPT	_
ENSG00000143278	5.13E-15	-2.63127	1	1	F13B	K03906
ENSG00000143297	5.13E-15	-2.63127	1	1	FCRL5	K06727
ENSG00000143333	5.13E-15	-2.63127	1	1	RGS16	K16449
ENSG00000143340	5.13E-15	-2.63127	1	1	FAM163A	_
ENSG00000143341	5.13E-15	-2.63127	1	1	HMCN1	K17341
ENSG00000143469	5.13E-15	-2.63127	1	1	SYT14	K19328
ENSG00000143473	5.13E-15	-2.63127	1	1	KCNH1	K04904
ENSG00000143502	5.13E-15	-2.63127	1	1	SUSD4	_
ENSG00000143520	5.13E-15	-2.63127	1	1	FLG2	K10384
ENSG00000143536	5.13E-15	-2.63127	1	1	CRNN	_
ENSG00000143595	5.13E-15	-2.63127	1	1	AQP10	K09878
ENSG00000143627	5.13E-15	-2.63127	1	1	PKLR	K12406
ENSG00000143630	5.13E-15	-2.63127	1	1	HCN3	_
ENSG00000143768	5.13E-15	-2.63127	1	1	LEFTY2	K04668
ENSG00000143851	5.13E-15	-2.63127	1	1	PTPN7	K18019
ENSG00000143867	5.13E-15	-2.63127	1	1	OSR1	_
ENSG00000143869	5.13E-15	-2.63127	1	1	GDF7	K04664
ENSG00000143921	5.13E-15	-2.63127	1	1	ABCG8	K05684
ENSG00000143954	5.13E-15	-2.63127	1	1	REG3G	_
ENSG00000144010	5.13E-15	-2.63127	1	1	TRIM43B	K12019
ENSG00000144015	5.13E-15	-2.63127	1	1	TRIM43	K12019
ENSG00000144031	5.13E-15	-2.63127	1	1	ANKRD53	_
ENSG00000144043	5.13E-15	-2.63127	1	1	TEX261	_
ENSG00000144057	5.13E-15	-2.63127	1	1	ST6GAL2	K00779
ENSG00000144115	5.13E-15	-2.63127	1	1	THNSL2	_
ENSG00000144119	5.13E-15	-2.63127	1	1	C1QL2	_
ENSG00000144140	5.13E-15	-2.63127	1	1	RP11-143M1.7	K15636
ENSG00000144152	5.13E-15	-2.63127	1	1	FBLN7	K17342
ENSG00000144188	5.13E-15	-2.63127	1	1	TRIM43CP	K12019
ENSG00000144191	5.13E-15	-2.63127	1	1	CNGA3	K04950

ENSG00000144218	5.13E-15	-2.63127	1	1	AFF3	K15195
ENSG00000144227	5.13E-15	-2.63127	1	1	NXPH2	K16657
ENSG00000144290	5.13E-15	-2.63127	1	1	SLC4A10	K13861
ENSG00000144331	5.13E-15	-2.63127	1	1	ZNF385B	_
ENSG00000144339	5.13E-15	-2.63127	1	1	TMEFF2	_
ENSG00000144406	5.13E-15	-2.63127	1	1	UNC80	_
ENSG00000144407	5.13E-15	-2.63127	1	1	PTH2R	K04586
ENSG00000144410	5.13E-15	-2.63127	1	1	CPO	_
ENSG00000144451	5.13E-15	-2.63127	1	1	SPAG16	_
ENSG00000144481	5.13E-15	-2.63127	1	1	TRPM8	K04983
ENSG00000144619	5.13E-15	-2.63127	1	1	CNTN4	K06762
ENSG00000144644	5.13E-15	-2.63127	1	1	GADL1	K18966
ENSG00000144648	5.13E-15	-2.63127	1	1	ACKR2	K04187
ENSG00000144649	5.13E-15	-2.63127	1	1	FAM198A	_
ENSG00000144668	5.13E-15	-2.63127	1	1	ITGA9	K06585
ENSG00000144681	5.13E-15	-2.63127	1	1	STAC	_
ENSG00000144820	5.13E-15	-2.63127	1	1	GPR128	K08464
ENSG00000144834	5.13E-15	-2.63127	1	1	TAGLN3	_
ENSG00000144843	5.13E-15	-2.63127	1	1	ADPRH	K01245
ENSG00000144847	5.13E-15	-2.63127	1	1	IGSF11	K06791
ENSG00000144857	5.13E-15	-2.63127	1	1	BOC	_
ENSG00000144868	5.13E-15	-2.63127	1	1	TMEM108	K12581
ENSG00000144891	5.13E-15	-2.63127	1	1	AGTR1	K04166
ENSG00000144908	5.13E-15	-2.63127	1	1	ALDH1L1	K00289
ENSG00000144962	5.13E-15	-2.63127	1	1	SPATA16	_
ENSG00000145103	5.13E-15	-2.63127	1	1	ILDR1	_
ENSG00000145147	5.13E-15	-2.63127	1	1	SLIT2	K06839
ENSG00000145192	5.13E-15	-2.63127	1	1	AHSG	_
ENSG00000145247	5.13E-15	-2.63127	1	1	OCIAD2	_
ENSG00000145283	5.13E-15	-2.63127	1	1	SLC10A6	K14346
ENSG00000145309	5.13E-15	-2.63127	1	1	CABS1	_
ENSG00000145321	5.13E-15	-2.63127	1	1	GC	K12258
ENSG00000145358	5.13E-15	-2.63127	1	1	DDIT4L	_
ENSG00000145362	5.13E-15	-2.63127	1	1	ANK2	K10380
ENSG00000145384	5.13E-15	-2.63127	1	1	FABP2	K08751
ENSG00000145423	5.13E-15	-2.63127	1	1	SFRP2	K02176
ENSG00000145428	5.13E-15	-2.63127	1	1	RNF175	_
ENSG00000145476	5.13E-15	-2.63127	1	1	CYP4V2	K07427
ENSG00000145526	5.13E-15	-2.63127	1	1	CDH18	K06805
ENSG00000145626	5.13E-15	-2.63127	1	1	UGT3A1	_
ENSG00000145642	5.13E-15	-2.63127	1	1	FAM159B	_
ENSG00000145649	5.13E-15	-2.63127	1	1	GZMA	K01352
ENSG00000145692	5.13E-15	-2.63127	1	1	BHMT	K00544
ENSG00000145708	5.13E-15	-2.63127	1	1	CRHBP	_
ENSG00000145721	5.13E-15	-2.63127	1	1	LIX1	_
ENSG00000145723	5.13E-15	-2.63127	1	1	GIN1	_
ENSG00000145734	5.13E-15	-2.63127	1	1	BDP1	K15198
ENSG00000145777	5.13E-15	-2.63127	1	1	TSLP	K05436
ENSG00000145794	5.13E-15	-2.63127	1	1	MEGF10	_
ENSG00000145808	5.13E-15	-2.63127	1	1	ADAMTS19	K08633
ENSG00000145824	5.13E-15	-2.63127	1	1	CXCL14	K10033
ENSG00000145826	5.13E-15	-2.63127	1	1	LECT2	_
ENSG00000145832	5.13E-15	-2.63127	1	1	SLC25A48	K15124
ENSG00000145835	5.13E-15	-2.63127	1	1	CDKN2AIPNLP2	_
ENSG00000145839	5.13E-15	-2.63127	1	1	IL9	K05432
ENSG00000145850	5.13E-15	-2.63127	1	1	TIMD4	_
ENSG00000145863	5.13E-15	-2.63127	1	1	GABRA6	_
ENSG00000145864	5.13E-15	-2.63127	1	1	GABRB2	K05181

ENSG00000145888	5.13E-15	-2.63127	1	1	GLRA1	K05193
ENSG00000145920	5.13E-15	-2.63127	1	1	CPLX2	K15294
ENSG00000146001	5.13E-15	-2.63127	1	1	PCDHB18	K16494
ENSG00000146038	5.13E-15	-2.63127	1	1	DCDC2	K15696
ENSG00000146039	5.13E-15	-2.63127	1	1	SLC17A4	K12300
ENSG00000146047	5.13E-15	-2.63127	1	1	HIST1H2BA	K11252
ENSG00000146049	5.13E-15	-2.63127	1	1	KAAG1	_
ENSG00000146070	5.13E-15	-2.63127	1	1	PLA2G7	K01062
ENSG00000146112	5.13E-15	-2.63127	1	1	PPP1R18	K17559
ENSG00000146122	5.13E-15	-2.63127	1	1	DAAM2	K04512
ENSG00000146147	5.13E-15	-2.63127	1	1	MLIP	_
ENSG00000146151	5.13E-15	-2.63127	1	1	HMGCLL1	K01640
ENSG00000146192	5.13E-15	-2.63127	1	1	FGD2	K05721
ENSG00000146215	5.13E-15	-2.63127	1	1	CRIP3	_
ENSG00000146250	5.13E-15	-2.63127	1	1	PRSS35	_
ENSG00000146285	5.13E-15	-2.63127	1	1	SCML4	K11466
ENSG00000146350	5.13E-15	-2.63127	1	1	TBC1D32	K02927
ENSG00000146352	5.13E-15	-2.63127	1	1	CLVS2	_
ENSG00000146360	5.13E-15	-2.63127	1	1	GPR6	K04313
ENSG00000146374	5.13E-15	-2.63127	1	1	RSPO3	_
ENSG00000146378	5.13E-15	-2.63127	1	1	TAAR2	K05051
ENSG00000146383	5.13E-15	-2.63127	1	1	TAAR6	K05051
ENSG00000146385	5.13E-15	-2.63127	1	1	TAAR8	K05051
ENSG00000146386	5.13E-15	-2.63127	1	1	ABRACL	_
ENSG00000146399	5.13E-15	-2.63127	1	1	TAAR1	K05051
ENSG00000146453	5.13E-15	-2.63127	1	1	PNLDC1	_
ENSG00000146521	5.13E-15	-2.63127	1	1	C6orf123	K12581
ENSG00000146555	5.13E-15	-2.63127	1	1	SDK1	K16353
ENSG00000146618	5.13E-15	-2.63127	1	1	FERD3L	_
ENSG00000146678	5.13E-15	-2.63127	1	1	IGFBP1	_
ENSG00000146722	5.13E-15	-2.63127	1	1	AC006014.8	K09572
ENSG00000146755	5.13E-15	-2.63127	1	1	TRIM50	K12024
ENSG00000146802	5.13E-15	-2.63127	1	1	TMEM168	K17985
ENSG00000146809	5.13E-15	-2.63127	1	1	ASB15	K10337
ENSG00000146857	5.13E-15	-2.63127	1	1	STRA8	_
ENSG00000146910	5.13E-15	-2.63127	1	1	CNPY1	_
ENSG00000146926	5.13E-15	-2.63127	1	1	ASB10	K10332
ENSG00000146950	5.13E-15	-2.63127	1	1	SHROOM2	K18625
ENSG00000147027	5.13E-15	-2.63127	1	1	TMEM47	_
ENSG00000147036	5.13E-15	-2.63127	1	1	LANCL3	K19522
ENSG00000147041	5.13E-15	-2.63127	1	1	SYTL5	K17598
ENSG00000147059	5.13E-15	-2.63127	1	1	SPIN2A	_
ENSG00000147081	5.13E-15	-2.63127	1	1	AKAP4	K16521
ENSG00000147113	5.13E-15	-2.63127	1	1	CXorf36	_
ENSG00000147117	5.13E-15	-2.63127	1	1	ZNF157	K09228
ENSG00000147118	5.13E-15	-2.63127	1	1	ZNF182	K09228
ENSG00000147138	5.13E-15	-2.63127	1	1	GPR174	K08391
ENSG00000147155	5.13E-15	-2.63127	1	1	EBP	K01824
ENSG00000147160	5.13E-15	-2.63127	1	1	AWAT2	K11156
ENSG00000147180	5.13E-15	-2.63127	1	1	ZNF711	_
ENSG00000147223	5.13E-15	-2.63127	1	1	RIPPLY1	_
ENSG00000147231	5.13E-15	-2.63127	1	1	CXorf57	_
ENSG00000147246	5.13E-15	-2.63127	1	1	HTR2C	K04157
ENSG00000147256	5.13E-15	-2.63127	1	1	ARHGAP36	_
ENSG00000147257	5.13E-15	-2.63127	1	1	GPC3	K08109
ENSG00000147262	5.13E-15	-2.63127	1	1	GPR119	K08424
ENSG00000147364	5.13E-15	-2.63127	1	1	FBXO25	K10305
ENSG00000147378	5.13E-15	-2.63127	1	1	FATE1	_

ENSG00000147380	5.13E-15	-2.63127	1	1	OPN1MW	K04251
ENSG00000147381	5.13E-15	-2.63127	1	1	MAGEA4	_
ENSG00000147432	5.13E-15	-2.63127	1	1	CHRNB3	K04814
ENSG00000147434	5.13E-15	-2.63127	1	1	CHRNA6	K04808
ENSG00000147443	5.13E-15	-2.63127	1	1	DOK2	_
ENSG00000147481	5.13E-15	-2.63127	1	1	SNTG1	_
ENSG00000147570	5.13E-15	-2.63127	1	1	DNAJC5B	K09525
ENSG00000147571	5.13E-15	-2.63127	1	1	CRH	K05256
ENSG00000147573	5.13E-15	-2.63127	1	1	TRIM55	K10654
ENSG00000147596	5.13E-15	-2.63127	1	1	PRDM14	_
ENSG00000147606	5.13E-15	-2.63127	1	1	SLC26A7	K13962
ENSG00000147613	5.13E-15	-2.63127	1	1	PSKH2	K08808
ENSG00000147647	5.13E-15	-2.63127	1	1	DPYS	K01464
ENSG00000147655	5.13E-15	-2.63127	1	1	RSPO2	_
ENSG00000147753	5.13E-15	-2.63127	1	1	TTTY7	_
ENSG00000147761	5.13E-15	-2.63127	1	1	TTTY7B	_
ENSG00000147869	5.13E-15	-2.63127	1	1	CER1	K01645
ENSG00000147873	5.13E-15	-2.63127	1	1	IFNA5	K05414
ENSG00000147885	5.13E-15	-2.63127	1	1	IFNA16	K05414
ENSG00000147926	5.13E-15	-2.63127	1	1	SPATA31A3	_
ENSG00000148053	5.13E-15	-2.63127	1	1	NTRK2	K04360
ENSG00000148082	5.13E-15	-2.63127	1	1	SHC3	K17448
ENSG00000148123	5.13E-15	-2.63127	1	1	LPPR1	K19581
ENSG00000148136	5.13E-15	-2.63127	1	1	OR13C4	K04257
ENSG00000148156	5.13E-15	-2.63127	1	1	ACTL7B	_
ENSG00000148215	5.13E-15	-2.63127	1	1	OR5C1	K04257
ENSG00000148288	5.13E-15	-2.63127	1	1	GBGT1	K00722
ENSG00000148386	5.13E-15	-2.63127	1	1	LCN9	_
ENSG00000148408	5.13E-15	-2.63127	1	1	CACNA1B	K04849
ENSG00000148468	5.13E-15	-2.63127	1	1	FAM171A1	_
ENSG00000148482	5.13E-15	-2.63127	1	1	SLC39A12	K14718
ENSG00000148483	5.13E-15	-2.63127	1	1	TMEM236	K14404
ENSG00000148488	5.13E-15	-2.63127	1	1	ST8SIA6	K06615
ENSG00000148513	5.13E-15	-2.63127	1	1	ANKRD30A	K17299
ENSG00000148541	5.13E-15	-2.63127	1	1	FAM13C	_
ENSG00000148584	5.13E-15	-2.63127	1	1	A1CF	_
ENSG00000148600	5.13E-15	-2.63127	1	1	CDHR1	K16501
ENSG00000148602	5.13E-15	-2.63127	1	1	LRIT1	_
ENSG00000148604	5.13E-15	-2.63127	1	1	RGR	K04254
ENSG00000148655	5.13E-15	-2.63127	1	1	C10orf11	K12022
ENSG00000148671	5.13E-15	-2.63127	1	1	ADIRF	_
ENSG00000148702	5.13E-15	-2.63127	1	1	HABP2	K08648
ENSG00000148704	5.13E-15	-2.63127	1	1	VAX1	K09318
ENSG00000148734	5.13E-15	-2.63127	1	1	NPFFR1	K04240
ENSG00000148798	5.13E-15	-2.63127	1	1	INA	K07608
ENSG00000148826	5.13E-15	-2.63127	1	1	NKX6-2	K09350
ENSG00000148935	5.13E-15	-2.63127	1	1	GAS2	_
ENSG00000148942	5.13E-15	-2.63127	1	1	SLC5A12	K14388
ENSG00000148948	5.13E-15	-2.63127	1	1	LRRC4C	K07523
ENSG00000148965	5.13E-15	-2.63127	1	1	SAA4	K17310
ENSG00000149021	5.13E-15	-2.63127	1	1	SCGB1A1	_
ENSG00000149043	5.13E-15	-2.63127	1	1	SYT8	_
ENSG00000149090	5.13E-15	-2.63127	1	1	PAMR1	_
ENSG00000149124	5.13E-15	-2.63127	1	1	GLYAT	K00628
ENSG00000149133	5.13E-15	-2.63127	1	1	OR5F1	K04257
ENSG00000149256	5.13E-15	-2.63127	1	1	TENM4	_
ENSG00000149260	5.13E-15	-2.63127	1	1	CAPN5	K08574
ENSG00000149294	5.13E-15	-2.63127	1	1	NCAM1	K06491

ENSG00000149295	5.13E-15	-2.63127	1	1	DRD2	K04145
ENSG00000149300	5.13E-15	-2.63127	1	1	C11orf52	_
ENSG00000149305	5.13E-15	-2.63127	1	1	HTR3B	K04819
ENSG00000149403	5.13E-15	-2.63127	1	1	GRIK4	K05204
ENSG00000149435	5.13E-15	-2.63127	1	1	GGTLC1	K18592
ENSG00000149443	5.13E-15	-2.63127	1	1	C20orf78	_
ENSG00000149451	5.13E-15	-2.63127	1	1	ADAM33	K08616
ENSG00000149452	5.13E-15	-2.63127	1	1	SLC22A8	K08205
ENSG00000149507	5.13E-15	-2.63127	1	1	OOSP2	_
ENSG00000149516	5.13E-15	-2.63127	1	1	MS4A3	_
ENSG00000149534	5.13E-15	-2.63127	1	1	MS4A2	K08090
ENSG00000149548	5.13E-15	-2.63127	1	1	CCDC15	K16752
ENSG00000149557	5.13E-15	-2.63127	1	1	FEZ1	_
ENSG00000149582	5.13E-15	-2.63127	1	1	TMEM25	_
ENSG00000149635	5.13E-15	-2.63127	1	1	OCSTAMP	_
ENSG00000149651	5.13E-15	-2.63127	1	1	SPINT4	_
ENSG00000149654	5.13E-15	-2.63127	1	1	CDH22	K06812
ENSG00000149735	5.13E-15	-2.63127	1	1	GPHA2	_
ENSG00000149742	5.13E-15	-2.63127	1	1	SLC22A9	K08206
ENSG00000149968	5.13E-15	-2.63127	1	1	MMP3	K01394
ENSG00000149970	5.13E-15	-2.63127	1	1	CNKSR2	K17536
ENSG00000149972	5.13E-15	-2.63127	1	1	CNTN5	K06763
ENSG00000150045	5.13E-15	-2.63127	1	1	KLRF1	K10075
ENSG00000150048	5.13E-15	-2.63127	1	1	CLEC1A	K10069
ENSG00000150175	5.13E-15	-2.63127	1	1	FRMPD2P2	_
ENSG00000150201	5.13E-15	-2.63127	1	1	FXYD4	K13359
ENSG00000150244	5.13E-15	-2.63127	1	1	TRIM48	K12019
ENSG00000150261	5.13E-15	-2.63127	1	1	OR8K1	K04257
ENSG00000150269	5.13E-15	-2.63127	1	1	OR5M9	K04257
ENSG00000150275	5.13E-15	-2.63127	1	1	PCDH15	K16500
ENSG00000150276	5.13E-15	-2.63127	1	1	PPIAP26	K03767
ENSG00000150337	5.13E-15	-2.63127	1	1	FCGR1A	K06498
ENSG00000150361	5.13E-15	-2.63127	1	1	KLHL1	K10442
ENSG00000150394	5.13E-15	-2.63127	1	1	CDH8	K06800
ENSG00000150556	5.13E-15	-2.63127	1	1	LYPD6B	_
ENSG00000150594	5.13E-15	-2.63127	1	1	ADRA2A	K04138
ENSG00000150625	5.13E-15	-2.63127	1	1	GPM6A	_
ENSG00000150627	5.13E-15	-2.63127	1	1	WDR17	_
ENSG00000150628	5.13E-15	-2.63127	1	1	SPATA4	_
ENSG00000150630	5.13E-15	-2.63127	1	1	VEGFC	K05449
ENSG00000150637	5.13E-15	-2.63127	1	1	CD226	K06567
ENSG00000150656	5.13E-15	-2.63127	1	1	CNDP1	K12581
ENSG00000150722	5.13E-15	-2.63127	1	1	PPP1R1C	K17549
ENSG00000150732	5.13E-15	-2.63127	1	1	RP11-134C1.1	K09228
ENSG00000150750	5.13E-15	-2.63127	1	1	C11orf53	_
ENSG00000150764	5.13E-15	-2.63127	1	1	DIXDC1	K00627
ENSG00000150773	5.13E-15	-2.63127	1	1	PIH1D2	_
ENSG00000150873	5.13E-15	-2.63127	1	1	C2orf50	_
ENSG00000150893	5.13E-15	-2.63127	1	1	FREM2	_
ENSG00000151005	5.13E-15	-2.63127	1	1	TKTL2	K00615
ENSG00000151033	5.13E-15	-2.63127	1	1	LYZL2	K01185
ENSG00000151065	5.13E-15	-2.63127	1	1	DCP1B	K12611
ENSG00000151067	5.13E-15	-2.63127	1	1	CACNA1C	K04850
ENSG00000151079	5.13E-15	-2.63127	1	1	KCNA6	K04879
ENSG00000151136	5.13E-15	-2.63127	1	1	BTBD11	K10483
ENSG00000151224	5.13E-15	-2.63127	1	1	MAT1A	K00789
ENSG00000151322	5.13E-15	-2.63127	1	1	NPAS3	K09098
ENSG00000151365	5.13E-15	-2.63127	1	1	THRSP	_

ENSG00000151379	5.13E-15	-2.63127	1	1	MSGN1	K09077
ENSG00000151388	5.13E-15	-2.63127	1	1	ADAMTS12	K08626
ENSG00000151418	5.13E-15	-2.63127	1	1	ATP6V1G3	K02152
ENSG00000151475	5.13E-15	-2.63127	1	1	SLC25A31	K05863
ENSG00000151490	5.13E-15	-2.63127	1	1	PTPRO	K18035
ENSG00000151577	5.13E-15	-2.63127	1	1	DRD3	K04146
ENSG00000151612	5.13E-15	-2.63127	1	1	ZNF827	_
ENSG00000151615	5.13E-15	-2.63127	1	1	POU4F2	K09366
ENSG00000151631	5.13E-15	-2.63127	1	1	AKR1C6P	K04119
ENSG00000151632	5.13E-15	-2.63127	1	1	AKR1C2	K00089
ENSG00000151650	5.13E-15	-2.63127	1	1	VENTX	K17985
ENSG00000151692	5.13E-15	-2.63127	1	1	RNF144A	K11975
ENSG00000151702	5.13E-15	-2.63127	1	1	FLI1	K09436
ENSG00000151704	5.13E-15	-2.63127	1	1	KCNJ1	K04995
ENSG00000151778	5.13E-15	-2.63127	1	1	SERP2	_
ENSG00000151789	5.13E-15	-2.63127	1	1	ZNF385D	_
ENSG00000151790	5.13E-15	-2.63127	1	1	TDO2	K00453
ENSG00000151812	5.13E-15	-2.63127	1	1	SLC35F4	K15288
ENSG00000151834	5.13E-15	-2.63127	1	1	GABRA2	K05175
ENSG00000151838	5.13E-15	-2.63127	1	1	CCDC175	_
ENSG00000151883	5.13E-15	-2.63127	1	1	PARP8	K15258
ENSG00000151948	5.13E-15	-2.63127	1	1	GLT1D1	_
ENSG00000151952	5.13E-15	-2.63127	1	1	TMEM132D	K17599
ENSG00000151962	5.13E-15	-2.63127	1	1	RBM46	_
ENSG00000152034	5.13E-15	-2.63127	1	1	MCHR2	K05054
ENSG00000152049	5.13E-15	-2.63127	1	1	KCNE4	K04898
ENSG00000152092	5.13E-15	-2.63127	1	1	ASTN1	_
ENSG00000152093	5.13E-15	-2.63127	1	1	CFC1B	_
ENSG00000152192	5.13E-15	-2.63127	1	1	POU4F1	K09366
ENSG00000152207	5.13E-15	-2.63127	1	1	CYSLTR2	K04323
ENSG00000152208	5.13E-15	-2.63127	1	1	GRID2	K05207
ENSG00000152213	5.13E-15	-2.63127	1	1	ARL11	K07959
ENSG00000152214	5.13E-15	-2.63127	1	1	RIT2	K07833
ENSG00000152254	5.13E-15	-2.63127	1	1	G6PC2	K01084
ENSG00000152266	5.13E-15	-2.63127	1	1	PTH	K05261
ENSG00000152268	5.13E-15	-2.63127	1	1	SPON1	_
ENSG00000152315	5.13E-15	-2.63127	1	1	KCNK13	K04922
ENSG00000152454	5.13E-15	-2.63127	1	1	ZNF256	K09228
ENSG00000152463	5.13E-15	-2.63127	1	1	OLAH	K01071
ENSG00000152467	5.13E-15	-2.63127	1	1	ZSCAN1	K09230
ENSG00000152583	5.13E-15	-2.63127	1	1	SPARCL1	_
ENSG00000152591	5.13E-15	-2.63127	1	1	DSPP	_
ENSG00000152592	5.13E-15	-2.63127	1	1	DMP1	_
ENSG00000152595	5.13E-15	-2.63127	1	1	MEPE	_
ENSG00000152670	5.13E-15	-2.63127	1	1	DDX4	K13982
ENSG00000152672	5.13E-15	-2.63127	1	1	CLEC4F	K10060
ENSG00000152779	5.13E-15	-2.63127	1	1	SLC16A12	K11810
ENSG00000152784	5.13E-15	-2.63127	1	1	PRDM8	_
ENSG00000152785	5.13E-15	-2.63127	1	1	BMP3	K05496
ENSG00000152910	5.13E-15	-2.63127	1	1	CNTNAP4	_
ENSG00000152931	5.13E-15	-2.63127	1	1	PART1	_
ENSG00000152953	5.13E-15	-2.63127	1	1	STK32B	K08793
ENSG00000152954	5.13E-15	-2.63127	1	1	NRSN1	_
ENSG00000152969	5.13E-15	-2.63127	1	1	JAKMIP1	_
ENSG00000153002	5.13E-15	-2.63127	1	1	CPB1	K01291
ENSG00000153012	5.13E-15	-2.63127	1	1	LGI2	_
ENSG00000153130	5.13E-15	-2.63127	1	1	SCOC	_
ENSG00000153132	5.13E-15	-2.63127	1	1	CLGN	K09551

ENSG00000153157	5.13E-15	-2.63127	1	1	SYCP2L	_
ENSG00000153230	5.13E-15	-2.63127	1	1	OR14K1	K04257
ENSG00000153246	5.13E-15	-2.63127	1	1	PLA2R1	K06560
ENSG00000153266	5.13E-15	-2.63127	1	1	FEZF2	_
ENSG00000153283	5.13E-15	-2.63127	1	1	CD96	K06517
ENSG00000153292	5.13E-15	-2.63127	1	1	GPR110	K08453
ENSG00000153303	5.13E-15	-2.63127	1	1	FRMD1	K16821
ENSG00000153347	5.13E-15	-2.63127	1	1	FAM81B	_
ENSG00000153404	5.13E-15	-2.63127	1	1	PLEKHG4B	_
ENSG00000153498	5.13E-15	-2.63127	1	1	SPACA7	_
ENSG00000153551	5.13E-15	-2.63127	1	1	CMTM7	_
ENSG00000153563	5.13E-15	-2.63127	1	1	CD8A	K06458
ENSG00000153666	5.13E-15	-2.63127	1	1	GOLGA8I	K10595
ENSG00000153684	5.13E-15	-2.63127	1	1	GOLGA8F	_
ENSG00000153707	5.13E-15	-2.63127	1	1	PTPRD	K06777
ENSG00000153779	5.13E-15	-2.63127	1	1	TGIF2LX	K19554
ENSG00000153789	5.13E-15	-2.63127	1	1	FAM92B	_
ENSG00000153820	5.13E-15	-2.63127	1	1	SPHKAP	_
ENSG00000153822	5.13E-15	-2.63127	1	1	KCNJ16	K12581
ENSG00000153898	5.13E-15	-2.63127	1	1	MCOLN2	K04993
ENSG00000153923	5.13E-15	-2.63127	1	1	CLCA3P	K05030
ENSG00000153956	5.13E-15	-2.63127	1	1	CACNA2D1	K04858
ENSG00000154007	5.13E-15	-2.63127	1	1	ASB17	K10339
ENSG00000154027	5.13E-15	-2.63127	1	1	AK5	K00939
ENSG00000154080	5.13E-15	-2.63127	1	1	CHST9	K09673
ENSG00000154143	5.13E-15	-2.63127	1	1	PANX3	K03443
ENSG00000154162	5.13E-15	-2.63127	1	1	CDH12	K06804
ENSG00000154165	5.13E-15	-2.63127	1	1	GPR15	K08403
ENSG00000154198	5.13E-15	-2.63127	1	1	CYP4Z2P	K07429
ENSG00000154227	5.13E-15	-2.63127	1	1	CERS3	K04710
ENSG00000154258	5.13E-15	-2.63127	1	1	ABCA9	K05651
ENSG00000154262	5.13E-15	-2.63127	1	1	ABCA6	K05649
ENSG00000154269	5.13E-15	-2.63127	1	1	ENPP3	K01513
ENSG00000154274	5.13E-15	-2.63127	1	1	C4orf19	_
ENSG00000154316	5.13E-15	-2.63127	1	1	TDH	K15789
ENSG00000154415	5.13E-15	-2.63127	1	1	PPP1R3A	K07189
ENSG00000154478	5.13E-15	-2.63127	1	1	GPR26	K08411
ENSG00000154529	5.13E-15	-2.63127	1	1	CNTNAP3B	_
ENSG00000154545	5.13E-15	-2.63127	1	1	MAGED4	_
ENSG00000154548	5.13E-15	-2.63127	1	1	SRSF12	K12900
ENSG00000154553	5.13E-15	-2.63127	1	1	PDLIM3	_
ENSG00000154611	5.13E-15	-2.63127	1	1	PSMA8	K02731
ENSG00000154620	5.13E-15	-2.63127	1	1	TMSB4Y	K05764
ENSG00000154655	5.13E-15	-2.63127	1	1	L3MBTL4	_
ENSG00000154760	5.13E-15	-2.63127	1	1	SLFN13	K12581
ENSG00000154764	5.13E-15	-2.63127	1	1	WNT7A	K00572
ENSG00000154768	5.13E-15	-2.63127	1	1	C17orf50	_
ENSG00000154783	5.13E-15	-2.63127	1	1	FGD5	K05724
ENSG00000154856	5.13E-15	-2.63127	1	1	APCDD1	K17985
ENSG00000154928	5.13E-15	-2.63127	1	1	EPHB1	_
ENSG00000154930	5.13E-15	-2.63127	1	1	ACSS1	K01895
ENSG00000154975	5.13E-15	-2.63127	1	1	CA10	_
ENSG00000154997	5.13E-15	-2.63127	1	1	14-Sep	K16941
ENSG00000155011	5.13E-15	-2.63127	1	1	DKK2	K02165
ENSG00000155052	5.13E-15	-2.63127	1	1	CNTNAP5	_
ENSG00000155070	5.13E-15	-2.63127	1	1	UNC93B2	K04257
ENSG00000155087	5.13E-15	-2.63127	1	1	ODF1	_
ENSG00000155093	5.13E-15	-2.63127	1	1	PTPRN2	K07817

ENSG00000155249	5.13E-15	-2.63127	1	1	OR4K1	K04257
ENSG00000155282	5.13E-15	-2.63127	1	1	RP11-195B21.3	K17299
ENSG00000155307	5.13E-15	-2.63127	1	1	SAMSN1	_
ENSG00000155495	5.13E-15	-2.63127	1	1	MAGEC1	K03006
ENSG00000155511	5.13E-15	-2.63127	1	1	GRIA1	K05197
ENSG00000155622	5.13E-15	-2.63127	1	1	XAGE2B	_
ENSG00000155629	5.13E-15	-2.63127	1	1	PIK3AP1	K12230
ENSG00000155659	5.13E-15	-2.63127	1	1	VSIG4	_
ENSG00000155714	5.13E-15	-2.63127	1	1	PDZD9	K05069
ENSG00000155719	5.13E-15	-2.63127	1	1	OTOA	_
ENSG00000155816	5.13E-15	-2.63127	1	1	FMN2	K02184
ENSG00000155833	5.13E-15	-2.63127	1	1	CYLC2	K08834
ENSG00000155849	5.13E-15	-2.63127	1	1	ELMO1	K12366
ENSG00000155875	5.13E-15	-2.63127	1	1	FAM154A	_
ENSG00000155886	5.13E-15	-2.63127	1	1	SLC24A2	K13750
ENSG00000155890	5.13E-15	-2.63127	1	1	TRIM42	K12018
ENSG00000155897	5.13E-15	-2.63127	1	1	ADCY8	K08048
ENSG00000155961	5.13E-15	-2.63127	1	1	RAB39B	K07925
ENSG00000155962	5.13E-15	-2.63127	1	1	CLIC2	K05022
ENSG00000155966	5.13E-15	-2.63127	1	1	AFF2	K15194
ENSG00000155970	5.13E-15	-2.63127	1	1	MICU3	_
ENSG00000156006	5.13E-15	-2.63127	1	1	NAT2	K00622
ENSG00000156009	5.13E-15	-2.63127	1	1	MAGEA8	_
ENSG00000156076	5.13E-15	-2.63127	1	1	WIF1	K01691
ENSG00000156096	5.13E-15	-2.63127	1	1	UGT2B4	K00699
ENSG00000156103	5.13E-15	-2.63127	1	1	MMP16	K07996
ENSG00000156113	5.13E-15	-2.63127	1	1	KCNMA1	K04936
ENSG00000156127	5.13E-15	-2.63127	1	1	BATF	K09034
ENSG00000156150	5.13E-15	-2.63127	1	1	ALX3	K09323
ENSG00000156194	5.13E-15	-2.63127	1	1	PPEF2	K13807
ENSG00000156206	5.13E-15	-2.63127	1	1	C15orf26	_
ENSG00000156218	5.13E-15	-2.63127	1	1	ADAMTSL3	_
ENSG00000156222	5.13E-15	-2.63127	1	1	SLC28A1	K11536
ENSG00000156234	5.13E-15	-2.63127	1	1	CXCL13	K10032
ENSG00000156269	5.13E-15	-2.63127	1	1	NAA11	K00670
ENSG00000156282	5.13E-15	-2.63127	1	1	CLDN17	K06087
ENSG00000156284	5.13E-15	-2.63127	1	1	CLDN8	K06087
ENSG00000156298	5.13E-15	-2.63127	1	1	TSPAN7	K06571
ENSG00000156395	5.13E-15	-2.63127	1	1	SORCS3	_
ENSG00000156413	5.13E-15	-2.63127	1	1	FUT6	K07634
ENSG00000156466	5.13E-15	-2.63127	1	1	GDF6	K04664
ENSG00000156486	5.13E-15	-2.63127	1	1	KCNS2	K04932
ENSG00000156564	5.13E-15	-2.63127	1	1	LRFN2	K16355
ENSG00000156587	5.13E-15	-2.63127	1	1	UBE2L6	K04553
ENSG00000156687	5.13E-15	-2.63127	1	1	UNC5D	K07521
ENSG00000156738	5.13E-15	-2.63127	1	1	MS4A1	K06466
ENSG00000156750	5.13E-15	-2.63127	1	1	AQP7P3	K08771
ENSG00000156755	5.13E-15	-2.63127	1	1	IGKV1OR-2	_
ENSG00000156885	5.13E-15	-2.63127	1	1	COX6A2	K02266
ENSG00000156925	5.13E-15	-2.63127	1	1	ZIC3	K18487
ENSG00000156959	5.13E-15	-2.63127	1	1	LHFPL4	_
ENSG00000157021	5.13E-15	-2.63127	1	1	FAM92A1P1	_
ENSG00000157060	5.13E-15	-2.63127	1	1	SHCBP1L	_
ENSG00000157087	5.13E-15	-2.63127	1	1	ATP2B2	K05850
ENSG00000157093	5.13E-15	-2.63127	1	1	LYZL4	_
ENSG00000157103	5.13E-15	-2.63127	1	1	SLC6A1	K05034
ENSG00000157111	5.13E-15	-2.63127	1	1	TMEM171	_
ENSG00000157119	5.13E-15	-2.63127	1	1	KLHL40	K10473

ENSG00000157131	5.13E-15	-2.63127	1	1	C8A	K03997
ENSG00000157214	5.13E-15	-2.63127	1	1	STEAP2	K14738
ENSG00000157219	5.13E-15	-2.63127	1	1	HTR5A	K04161
ENSG00000157322	5.13E-15	-2.63127	1	1	CLEC18A	_
ENSG00000157330	5.13E-15	-2.63127	1	1	C1orf158	_
ENSG00000157335	5.13E-15	-2.63127	1	1	CLEC18C	_
ENSG00000157358	5.13E-15	-2.63127	1	1	PRAMEF15	_
ENSG00000157399	5.13E-15	-2.63127	1	1	ARSE	K18222
ENSG00000157404	5.13E-15	-2.63127	1	1	KIT	K05091
ENSG00000157502	5.13E-15	-2.63127	1	1	MUM1L1	_
ENSG00000157542	5.13E-15	-2.63127	1	1	KCNJ6	K05000
ENSG00000157570	5.13E-15	-2.63127	1	1	TSPAN18	K17353
ENSG00000157601	5.13E-15	-2.63127	1	1	MX1	K14754
ENSG00000157654	5.13E-15	-2.63127	1	1	PALM2-AKAP2	K16519
ENSG00000157765	5.13E-15	-2.63127	1	1	SLC34A2	K14683
ENSG00000157766	5.13E-15	-2.63127	1	1	ACAN	K06792
ENSG00000157828	5.13E-15	-2.63127	1	1	RPS4Y2	K02987
ENSG00000157884	5.13E-15	-2.63127	1	1	CIB4	_
ENSG00000157965	5.13E-15	-2.63127	1	1	SSX8	K15624
ENSG00000158014	5.13E-15	-2.63127	1	1	SLC30A2	K14689
ENSG00000158022	5.13E-15	-2.63127	1	1	TRIM63	K10655
ENSG00000158089	5.13E-15	-2.63127	1	1	GALNT14	K00710
ENSG00000158164	5.13E-15	-2.63127	1	1	TMSB15A	K11252
ENSG00000158270	5.13E-15	-2.63127	1	1	COLEC12	K10062
ENSG00000158296	5.13E-15	-2.63127	1	1	SLC13A3	K14445
ENSG00000158301	5.13E-15	-2.63127	1	1	GPRASP2	_
ENSG00000158352	5.13E-15	-2.63127	1	1	SHROOM4	K18625
ENSG00000158427	5.13E-15	-2.63127	1	1	TMSB15B	K11252
ENSG00000158473	5.13E-15	-2.63127	1	1	CD1D	K06448
ENSG00000158477	5.13E-15	-2.63127	1	1	CD1A	K06448
ENSG00000158481	5.13E-15	-2.63127	1	1	CD1C	K06448
ENSG00000158482	5.13E-15	-2.63127	1	1	SNX29P1	K17935
ENSG00000158485	5.13E-15	-2.63127	1	1	CD1B	K06448
ENSG00000158488	5.13E-15	-2.63127	1	1	CD1E	K06448
ENSG00000158497	5.13E-15	-2.63127	1	1	HMHB1	_
ENSG00000158517	5.13E-15	-2.63127	1	1	NCF1	K08011
ENSG00000158553	5.13E-15	-2.63127	1	1	POM121L2	K14316
ENSG00000158571	5.13E-15	-2.63127	1	1	PFKFB1	K19028
ENSG00000158578	5.13E-15	-2.63127	1	1	ALAS2	K00643
ENSG00000158639	5.13E-15	-2.63127	1	1	PAGE5	K11299
ENSG00000158731	5.13E-15	-2.63127	1	1	OR10J6P	K04257
ENSG00000158748	5.13E-15	-2.63127	1	1	HTR6	K04162
ENSG00000158764	5.13E-15	-2.63127	1	1	ITLN2	K17527
ENSG00000158786	5.13E-15	-2.63127	1	1	PLA2G2F	K01047
ENSG00000158816	5.13E-15	-2.63127	1	1	VWA5B1	_
ENSG00000158874	5.13E-15	-2.63127	1	1	APOA2	K08758
ENSG00000158887	5.13E-15	-2.63127	1	1	MPZ	K06770
ENSG00000158901	5.13E-15	-2.63127	1	1	WFDC8	_
ENSG00000158955	5.13E-15	-2.63127	1	1	WNT9B	K01064
ENSG00000158966	5.13E-15	-2.63127	1	1	CACHD1	_
ENSG00000159123	5.13E-15	-2.63127	1	1	DMRTC1	K19494
ENSG00000159164	5.13E-15	-2.63127	1	1	SV2A	K06258
ENSG00000159173	5.13E-15	-2.63127	1	1	TNNI1	K10371
ENSG00000159182	5.13E-15	-2.63127	1	1	PRAC1	_
ENSG00000159186	5.13E-15	-2.63127	1	1	AC007383.6	K02137
ENSG00000159189	5.13E-15	-2.63127	1	1	C1QC	K03988
ENSG00000159247	5.13E-15	-2.63127	1	1	TUBBP5	K07375
ENSG00000159248	5.13E-15	-2.63127	1	1	GJD2	K07373

ENSG00000159289	5.13E-15	-2.63127	1	1	GOLGA6A	_
ENSG00000159387	5.13E-15	-2.63127	1	1	IRX6	_
ENSG00000159398	5.13E-15	-2.63127	1	1	CES5A	K15743
ENSG00000159409	5.13E-15	-2.63127	1	1	CELF3	K13207
ENSG00000159450	5.13E-15	-2.63127	1	1	TCHH	K18626
ENSG00000159455	5.13E-15	-2.63127	1	1	LCE2B	_
ENSG00000159495	5.13E-15	-2.63127	1	1	TGM7	K05623
ENSG00000159516	5.13E-15	-2.63127	1	1	SPRR2G	_
ENSG00000159527	5.13E-15	-2.63127	1	1	PGLYRP3	K01446
ENSG00000159640	5.13E-15	-2.63127	1	1	ACE	K01283
ENSG00000159648	5.13E-15	-2.63127	1	1	TEPP	K02927
ENSG00000159650	5.13E-15	-2.63127	1	1	UROC1	K01712
ENSG00000159723	5.13E-15	-2.63127	1	1	AGRP	K05231
ENSG00000159763	5.13E-15	-2.63127	1	1	PIP	_
ENSG00000159961	5.13E-15	-2.63127	1	1	OR3A3	K04257
ENSG00000160013	5.13E-15	-2.63127	1	1	PTGIR	K04263
ENSG00000160180	5.13E-15	-2.63127	1	1	TFF3	_
ENSG00000160202	5.13E-15	-2.63127	1	1	CRYAA	K09541
ENSG00000160219	5.13E-15	-2.63127	1	1	GAB3	_
ENSG00000160229	5.13E-15	-2.63127	1	1	ZNF66	K09228
ENSG00000160307	5.13E-15	-2.63127	1	1	S100B	_
ENSG00000160325	5.13E-15	-2.63127	1	1	CACFD1	_
ENSG00000160326	5.13E-15	-2.63127	1	1	SLC2A6	K08144
ENSG00000160349	5.13E-15	-2.63127	1	1	LCN1	_
ENSG00000160439	5.13E-15	-2.63127	1	1	RDH13	K11161
ENSG00000160505	5.13E-15	-2.63127	1	1	NLRP4	_
ENSG00000160593	5.13E-15	-2.63127	1	1	AMICA1	_
ENSG00000160654	5.13E-15	-2.63127	1	1	CD3G	K06452
ENSG00000160683	5.13E-15	-2.63127	1	1	CXCR5	K04190
ENSG00000160695	5.13E-15	-2.63127	1	1	VPS11	_
ENSG00000160791	5.13E-15	-2.63127	1	1	CCR5	K04180
ENSG00000160808	5.13E-15	-2.63127	1	1	MYL3	K12749
ENSG00000160844	5.13E-15	-2.63127	1	1	GATS	K13055
ENSG00000160856	5.13E-15	-2.63127	1	1	FCRL3	K06727
ENSG00000160882	5.13E-15	-2.63127	1	1	CYP11B1	K00497
ENSG00000160883	5.13E-15	-2.63127	1	1	HK3	K00844
ENSG00000160994	5.13E-15	-2.63127	1	1	CCDC105	_
ENSG00000161031	5.13E-15	-2.63127	1	1	PGLYRP2	K01446
ENSG00000161055	5.13E-15	-2.63127	1	1	SCGB3A1	_
ENSG00000161405	5.13E-15	-2.63127	1	1	IKZF3	K09220
ENSG00000161551	5.13E-15	-2.63127	1	1	ZNF577	K09228
ENSG00000161572	5.13E-15	-2.63127	1	1	LYZL6	K01185
ENSG00000161573	5.13E-15	-2.63127	1	1	CCL16	K05514
ENSG00000161574	5.13E-15	-2.63127	1	1	CCL15-CCL14	K05511
ENSG00000161583	5.13E-15	-2.63127	1	1	TBC1D3G	K11837
ENSG00000161594	5.13E-15	-2.63127	1	1	KLHL10	K10448
ENSG00000161609	5.13E-15	-2.63127	1	1	CCDC155	_
ENSG00000161634	5.13E-15	-2.63127	1	1	DCD	_
ENSG00000161640	5.13E-15	-2.63127	1	1	SIGLEC11	K06750
ENSG00000161649	5.13E-15	-2.63127	1	1	CD300LG	K16663
ENSG00000161652	5.13E-15	-2.63127	1	1	IZUMO2	_
ENSG00000161681	5.13E-15	-2.63127	1	1	SHANK1	K15009
ENSG00000161807	5.13E-15	-2.63127	1	1	OR7G1	K04257
ENSG00000161849	5.13E-15	-2.63127	1	1	KRT84	K07605
ENSG00000161850	5.13E-15	-2.63127	1	1	KRT82	K07605
ENSG00000161905	5.13E-15	-2.63127	1	1	ALOX15	K00460
ENSG00000161911	5.13E-15	-2.63127	1	1	TREML1	K14363
ENSG00000161944	5.13E-15	-2.63127	1	1	ASGR2	K10064

ENSG00000161973	5.13E-15	-2.63127	1	1	CCDC42	_
ENSG00000161992	5.13E-15	-2.63127	1	1	C16orf11	_
ENSG00000162040	5.13E-15	-2.63127	1	1	HS3ST6	K09679
ENSG00000162105	5.13E-15	-2.63127	1	1	SHANK2	K15009
ENSG00000162344	5.13E-15	-2.63127	1	1	FGF19	K04358
ENSG00000162365	5.13E-15	-2.63127	1	1	CYP4A22	K17688
ENSG00000162367	5.13E-15	-2.63127	1	1	TAL1	K09068
ENSG00000162373	5.13E-15	-2.63127	1	1	BEND5	_
ENSG00000162374	5.13E-15	-2.63127	1	1	ELAVL4	K13208
ENSG00000162383	5.13E-15	-2.63127	1	1	SLC1A7	K05618
ENSG00000162444	5.13E-15	-2.63127	1	1	RBP7	K10597
ENSG00000162456	5.13E-15	-2.63127	1	1	KNCN	_
ENSG00000162493	5.13E-15	-2.63127	1	1	PDPN	K16778
ENSG00000162494	5.13E-15	-2.63127	1	1	LRRC38	_
ENSG00000162543	5.13E-15	-2.63127	1	1	UBXN10	_
ENSG00000162571	5.13E-15	-2.63127	1	1	TTLL10	_
ENSG00000162594	5.13E-15	-2.63127	1	1	IL23R	K05065
ENSG00000162598	5.13E-15	-2.63127	1	1	C1orf87	_
ENSG00000162621	5.13E-15	-2.63127	1	1	LRRC53	_
ENSG00000162624	5.13E-15	-2.63127	1	1	LHX8	K09375
ENSG00000162631	5.13E-15	-2.63127	1	1	NTNG1	K07522
ENSG00000162643	5.13E-15	-2.63127	1	1	WDR63	_
ENSG00000162669	5.13E-15	-2.63127	1	1	HFM1	K15271
ENSG00000162685	5.13E-15	-2.63127	1	1	LSP1P3	K17608
ENSG00000162692	5.13E-15	-2.63127	1	1	VCAM1	K06527
ENSG00000162699	5.13E-15	-2.63127	1	1	DNAJA1P5	K09502
ENSG00000162706	5.13E-15	-2.63127	1	1	CADM3	K06780
ENSG00000162711	5.13E-15	-2.63127	1	1	NLRP3	K12800
ENSG00000162722	5.13E-15	-2.63127	1	1	TRIM58	K12027
ENSG00000162723	5.13E-15	-2.63127	1	1	SLAMF9	_
ENSG00000162727	5.13E-15	-2.63127	1	1	OR2M5	K04257
ENSG00000162738	5.13E-15	-2.63127	1	1	VANGL2	K04510
ENSG00000162739	5.13E-15	-2.63127	1	1	SLAMF6	K16852
ENSG00000162747	5.13E-15	-2.63127	1	1	FCGR3B	K06463
ENSG00000162755	5.13E-15	-2.63127	1	1	KLHDC9	_
ENSG00000162761	5.13E-15	-2.63127	1	1	LMX1A	K09371
ENSG00000162763	5.13E-15	-2.63127	1	1	LRRC52	_
ENSG00000162779	5.13E-15	-2.63127	1	1	AXDND1	_
ENSG00000162782	5.13E-15	-2.63127	1	1	TDRD5	K18407
ENSG00000162817	5.13E-15	-2.63127	1	1	C1orf115	_
ENSG00000162840	5.13E-15	-2.63127	1	1	MT2P1	K14739
ENSG00000162849	5.13E-15	-2.63127	1	1	KIF26B	K10404
ENSG00000162877	5.13E-15	-2.63127	1	1	PM20D1	K13049
ENSG00000162881	5.13E-15	-2.63127	1	1	OXER1	K10043
ENSG00000162896	5.13E-15	-2.63127	1	1	PIGR	K13073
ENSG00000162947	5.13E-15	-2.63127	1	1	AC007364.1	_
ENSG00000162949	5.13E-15	-2.63127	1	1	CAPN13	K08581
ENSG00000162951	5.13E-15	-2.63127	1	1	LRRTM1	K16665
ENSG00000162975	5.13E-15	-2.63127	1	1	KCNF1	K04899
ENSG00000162981	5.13E-15	-2.63127	1	1	FAM84A	_
ENSG00000162989	5.13E-15	-2.63127	1	1	KCNJ3	K04997
ENSG00000162992	5.13E-15	-2.63127	1	1	NEUROD1	K08033
ENSG00000163012	5.13E-15	-2.63127	1	1	ZSWIM2	K15716
ENSG00000163016	5.13E-15	-2.63127	1	1	ALMS1P	K16741
ENSG00000163032	5.13E-15	-2.63127	1	1	VSNL1	_
ENSG00000163040	5.13E-15	-2.63127	1	1	CCDC74A	_
ENSG00000163046	5.13E-15	-2.63127	1	1	ANKRD30BL	K17299
ENSG00000163060	5.13E-15	-2.63127	1	1	TEKT4	K18631

ENSG00000163064	5.13E-15	-2.63127	1	1	EN1	_
ENSG00000163069	5.13E-15	-2.63127	1	1	SGCB	K12566
ENSG00000163071	5.13E-15	-2.63127	1	1	SPATA18	_
ENSG00000163075	5.13E-15	-2.63127	1	1	PCDP1	_
ENSG00000163081	5.13E-15	-2.63127	1	1	CCDC140	_
ENSG00000163092	5.13E-15	-2.63127	1	1	XIRP2	_
ENSG00000163093	5.13E-15	-2.63127	1	1	BBS5	K16748
ENSG00000163098	5.13E-15	-2.63127	1	1	BIRC8	K04725
ENSG00000163114	5.13E-15	-2.63127	1	1	PDHA2	K00161
ENSG00000163116	5.13E-15	-2.63127	1	1	STPG2	_
ENSG00000163145	5.13E-15	-2.63127	1	1	C1QTNF7	_
ENSG00000163157	5.13E-15	-2.63127	1	1	TMOD4	K10370
ENSG00000163202	5.13E-15	-2.63127	1	1	LCE3D	_
ENSG00000163206	5.13E-15	-2.63127	1	1	SMCP	_
ENSG00000163207	5.13E-15	-2.63127	1	1	IVL	_
ENSG00000163209	5.13E-15	-2.63127	1	1	SPRR3	_
ENSG00000163217	5.13E-15	-2.63127	1	1	BMP10	K05503
ENSG00000163218	5.13E-15	-2.63127	1	1	PGLYRP4	K01446
ENSG00000163219	5.13E-15	-2.63127	1	1	ARHGAP25	_
ENSG00000163235	5.13E-15	-2.63127	1	1	TGFA	K08774
ENSG00000163254	5.13E-15	-2.63127	1	1	CRYGC	_
ENSG00000163273	5.13E-15	-2.63127	1	1	NPPC	K12336
ENSG00000163285	5.13E-15	-2.63127	1	1	GABRG1	K05186
ENSG00000163288	5.13E-15	-2.63127	1	1	GABRB1	K05181
ENSG00000163331	5.13E-15	-2.63127	1	1	DAPL1	_
ENSG00000163364	5.13E-15	-2.63127	1	1	LINC01116	_
ENSG00000163377	5.13E-15	-2.63127	1	1	FAM19A4	_
ENSG00000163394	5.13E-15	-2.63127	1	1	CCKAR	K04194
ENSG00000163421	5.13E-15	-2.63127	1	1	PROK2	_
ENSG00000163424	5.13E-15	-2.63127	1	1	C3orf30	_
ENSG00000163440	5.13E-15	-2.63127	1	1	PDCL2	_
ENSG00000163464	5.13E-15	-2.63127	1	1	CXCR1	K04175
ENSG00000163492	5.13E-15	-2.63127	1	1	CCDC141	_
ENSG00000163499	5.13E-15	-2.63127	1	1	CRYBA2	_
ENSG00000163501	5.13E-15	-2.63127	1	1	IHH	K11989
ENSG00000163515	5.13E-15	-2.63127	1	1	RETNLB	_
ENSG00000163518	5.13E-15	-2.63127	1	1	FCRL4	_
ENSG00000163519	5.13E-15	-2.63127	1	1	TRAT1	_
ENSG00000163534	5.13E-15	-2.63127	1	1	FCRL1	K06727
ENSG00000163554	5.13E-15	-2.63127	1	1	SPTA1	K06114
ENSG00000163563	5.13E-15	-2.63127	1	1	MNDA	_
ENSG00000163564	5.13E-15	-2.63127	1	1	PYHIN1	_
ENSG00000163581	5.13E-15	-2.63127	1	1	SLC2A2	K07593
ENSG00000163586	5.13E-15	-2.63127	1	1	FABP1	K08750
ENSG00000163599	5.13E-15	-2.63127	1	1	CTLA4	K06538
ENSG00000163600	5.13E-15	-2.63127	1	1	ICOS	K06713
ENSG00000163606	5.13E-15	-2.63127	1	1	CD200R1	_
ENSG00000163612	5.13E-15	-2.63127	1	1	FAM86KP	K12354
ENSG00000163623	5.13E-15	-2.63127	1	1	NKX6-1	K08030
ENSG00000163624	5.13E-15	-2.63127	1	1	CDS1	K00981
ENSG00000163629	5.13E-15	-2.63127	1	1	PTPN13	K02374
ENSG00000163630	5.13E-15	-2.63127	1	1	SYNPR	_
ENSG00000163631	5.13E-15	-2.63127	1	1	ALB	K16141
ENSG00000163638	5.13E-15	-2.63127	1	1	ADAMTS9	K08624
ENSG00000163645	5.13E-15	-2.63127	1	1	FAM194A	_
ENSG00000163646	5.13E-15	-2.63127	1	1	CLRN1	_
ENSG00000163661	5.13E-15	-2.63127	1	1	PTX3	_
ENSG00000163673	5.13E-15	-2.63127	1	1	DCLK3	K17530

ENSG00000163705	5.13E-15	-2.63127	1	1	FANCD2OS	K10891
ENSG00000163735	5.13E-15	-2.63127	1	1	CXCL5	K05506
ENSG00000163736	5.13E-15	-2.63127	1	1	PPBP	K10029
ENSG00000163737	5.13E-15	-2.63127	1	1	PF4	K05407
ENSG00000163739	5.13E-15	-2.63127	1	1	CXCL1	K05505
ENSG00000163746	5.13E-15	-2.63127	1	1	PLSCR2	_
ENSG00000163751	5.13E-15	-2.63127	1	1	CPA3	K08780
ENSG00000163792	5.13E-15	-2.63127	1	1	TCF23	_
ENSG00000163793	5.13E-15	-2.63127	1	1	DNAJC5G	K09525
ENSG00000163825	5.13E-15	-2.63127	1	1	RTP3	_
ENSG00000163827	5.13E-15	-2.63127	1	1	LRRC2	K17985
ENSG00000163833	5.13E-15	-2.63127	1	1	FBXO40	K10315
ENSG00000163864	5.13E-15	-2.63127	1	1	NMNAT3	K06210
ENSG00000163873	5.13E-15	-2.63127	1	1	GRIK3	K05203
ENSG00000163885	5.13E-15	-2.63127	1	1	CCDC37	_
ENSG00000163909	5.13E-15	-2.63127	1	1	HEYL	K09091
ENSG00000163914	5.13E-15	-2.63127	1	1	RHO	K04250
ENSG00000163958	5.13E-15	-2.63127	1	1	ZDHHC19	K18932
ENSG00000163982	5.13E-15	-2.63127	1	1	OTOP1	_
ENSG00000164002	5.13E-15	-2.63127	1	1	EXO5	K17815
ENSG00000164035	5.13E-15	-2.63127	1	1	EMCN	K16664
ENSG00000164047	5.13E-15	-2.63127	1	1	CAMP	K13916
ENSG00000164082	5.13E-15	-2.63127	1	1	GRM2	K04605
ENSG00000164093	5.13E-15	-2.63127	1	1	PITX2	K04686
ENSG00000164100	5.13E-15	-2.63127	1	1	NDST3	K02578
ENSG00000164106	5.13E-15	-2.63127	1	1	SCRG1	_
ENSG00000164107	5.13E-15	-2.63127	1	1	HAND2	K18486
ENSG00000164112	5.13E-15	-2.63127	1	1	TMEM155	_
ENSG00000164113	5.13E-15	-2.63127	1	1	ADAD1	_
ENSG00000164114	5.13E-15	-2.63127	1	1	MAP9	K10434
ENSG00000164116	5.13E-15	-2.63127	1	1	GUCY1A3	K12318
ENSG00000164120	5.13E-15	-2.63127	1	1	HPGD	K00069
ENSG00000164123	5.13E-15	-2.63127	1	1	C4orf45	_
ENSG00000164124	5.13E-15	-2.63127	1	1	TMEM144	K09488
ENSG00000164125	5.13E-15	-2.63127	1	1	FAM198B	_
ENSG00000164128	5.13E-15	-2.63127	1	1	NPY1R	K04204
ENSG00000164129	5.13E-15	-2.63127	1	1	NPY5R	K04207
ENSG00000164161	5.13E-15	-2.63127	1	1	HHIP	K06231
ENSG00000164197	5.13E-15	-2.63127	1	1	RNF180	K15708
ENSG00000164220	5.13E-15	-2.63127	1	1	F2RL2	K04235
ENSG00000164241	5.13E-15	-2.63127	1	1	C5orf63	_
ENSG00000164256	5.13E-15	-2.63127	1	1	PRDM9	K09228
ENSG00000164270	5.13E-15	-2.63127	1	1	HTR4	K04160
ENSG00000164283	5.13E-15	-2.63127	1	1	ESM1	_
ENSG00000164287	5.13E-15	-2.63127	1	1	CDC20B	_
ENSG00000164299	5.13E-15	-2.63127	1	1	SPZ1	K17482
ENSG00000164325	5.13E-15	-2.63127	1	1	TMEM174	_
ENSG00000164326	5.13E-15	-2.63127	1	1	CARTPT	_
ENSG00000164334	5.13E-15	-2.63127	1	1	FAM170A	_
ENSG00000164344	5.13E-15	-2.63127	1	1	KLKB1	K01324
ENSG00000164363	5.13E-15	-2.63127	1	1	SLC6A18	K05048
ENSG00000164379	5.13E-15	-2.63127	1	1	FOXQ1	K09410
ENSG00000164393	5.13E-15	-2.63127	1	1	GPR111	K08454
ENSG00000164398	5.13E-15	-2.63127	1	1	ACSL6	K01897
ENSG00000164399	5.13E-15	-2.63127	1	1	IL3	K04736
ENSG00000164400	5.13E-15	-2.63127	1	1	CSF2	K05427
ENSG00000164411	5.13E-15	-2.63127	1	1	GJB7	K07626
ENSG00000164434	5.13E-15	-2.63127	1	1	FABP7	K08756

ENSG00000164440	5.13E-15	-2.63127	1	1	TXLNB	_
ENSG00000164483	5.13E-15	-2.63127	1	1	SAMD3	_
ENSG00000164484	5.13E-15	-2.63127	1	1	TMEM200A	_
ENSG00000164488	5.13E-15	-2.63127	1	1	DACT2	_
ENSG00000164500	5.13E-15	-2.63127	1	1	C7orf72	_
ENSG00000164508	5.13E-15	-2.63127	1	1	HIST1H2AA	K11251
ENSG00000164556	5.13E-15	-2.63127	1	1	FAM183B	_
ENSG00000164588	5.13E-15	-2.63127	1	1	HCN1	K04954
ENSG00000164600	5.13E-15	-2.63127	1	1	NEUROD6	K09080
ENSG00000164604	5.13E-15	-2.63127	1	1	GPR85	K04302
ENSG00000164616	5.13E-15	-2.63127	1	1	FBXL21	K10287
ENSG00000164627	5.13E-15	-2.63127	1	1	KIF6	K10397
ENSG00000164645	5.13E-15	-2.63127	1	1	C7orf62	_
ENSG00000164687	5.13E-15	-2.63127	1	1	FABP5	K08754
ENSG00000164690	5.13E-15	-2.63127	1	1	SHH	K11988
ENSG00000164692	5.13E-15	-2.63127	1	1	COL1A2	K06236
ENSG00000164694	5.13E-15	-2.63127	1	1	FNDC1	_
ENSG00000164708	5.13E-15	-2.63127	1	1	PGAM2	K01834
ENSG00000164729	5.13E-15	-2.63127	1	1	SLC35G3	_
ENSG00000164736	5.13E-15	-2.63127	1	1	SOX17	K04495
ENSG00000164740	5.13E-15	-2.63127	1	1	SEPT7P5	K16944
ENSG00000164743	5.13E-15	-2.63127	1	1	C8orf48	_
ENSG00000164756	5.13E-15	-2.63127	1	1	SLC30A8	K14695
ENSG00000164778	5.13E-15	-2.63127	1	1	EN2	K09319
ENSG00000164794	5.13E-15	-2.63127	1	1	KCNV1	K04934
ENSG00000164796	5.13E-15	-2.63127	1	1	CSMD3	K17495
ENSG00000164816	5.13E-15	-2.63127	1	1	DEFA5	K05230
ENSG00000164821	5.13E-15	-2.63127	1	1	DEFA4	K05230
ENSG00000164822	5.13E-15	-2.63127	1	1	DEFA6	K05230
ENSG00000164825	5.13E-15	-2.63127	1	1	DEFB1	_
ENSG00000164853	5.13E-15	-2.63127	1	1	UNCX	K09328
ENSG00000164871	5.13E-15	-2.63127	1	1	SPAG11B	_
ENSG00000164879	5.13E-15	-2.63127	1	1	CA3	K01672
ENSG00000164893	5.13E-15	-2.63127	1	1	SLC7A13	K13870
ENSG00000164900	5.13E-15	-2.63127	1	1	GBX1	K09321
ENSG00000164929	5.13E-15	-2.63127	1	1	BAALC	_
ENSG00000164932	5.13E-15	-2.63127	1	1	CTHRC1	_
ENSG00000164935	5.13E-15	-2.63127	1	1	DCSTAMP	_
ENSG00000164972	5.13E-15	-2.63127	1	1	C9orf24	K18634
ENSG00000165023	5.13E-15	-2.63127	1	1	DIRAS2	K07841
ENSG00000165025	5.13E-15	-2.63127	1	1	SYK	K05855
ENSG00000165059	5.13E-15	-2.63127	1	1	PRKACG	K04345
ENSG00000165061	5.13E-15	-2.63127	1	1	ZMAT4	K03941
ENSG00000165066	5.13E-15	-2.63127	1	1	NKX6-3	K09351
ENSG00000165071	5.13E-15	-2.63127	1	1	TMEM71	_
ENSG00000165078	5.13E-15	-2.63127	1	1	CPA6	K08782
ENSG00000165091	5.13E-15	-2.63127	1	1	TMC1	_
ENSG00000165105	5.13E-15	-2.63127	1	1	RASEF	K17199
ENSG00000165131	5.13E-15	-2.63127	1	1	C7orf34	_
ENSG00000165152	5.13E-15	-2.63127	1	1	TMEM246	_
ENSG00000165164	5.13E-15	-2.63127	1	1	CXorf22	_
ENSG00000165178	5.13E-15	-2.63127	1	1	NCF1C	K08011
ENSG00000165182	5.13E-15	-2.63127	1	1	CXorf58	_
ENSG00000165186	5.13E-15	-2.63127	1	1	PTCHD1	_
ENSG00000165192	5.13E-15	-2.63127	1	1	ASB11	K10333
ENSG00000165202	5.13E-15	-2.63127	1	1	OR1Q1	K04257
ENSG00000165204	5.13E-15	-2.63127	1	1	OR1K1	K04257
ENSG00000165215	5.13E-15	-2.63127	1	1	CLDN3	K06087

ENSG00000165259	5.13E-15	-2.63127	1	1	HDX	_
ENSG00000165309	5.13E-15	-2.63127	1	1	ARMC3	_
ENSG00000165323	5.13E-15	-2.63127	1	1	FAT3	K16506
ENSG00000165349	5.13E-15	-2.63127	1	1	SLC7A3	K13865
ENSG00000165359	5.13E-15	-2.63127	1	1	DDX26B	K13180
ENSG00000165370	5.13E-15	-2.63127	1	1	GPR101	K08423
ENSG00000165376	5.13E-15	-2.63127	1	1	CLDN2	K06087
ENSG00000165379	5.13E-15	-2.63127	1	1	LRFN5	K16358
ENSG00000165383	5.13E-15	-2.63127	1	1	LRRC18	_
ENSG00000165388	5.13E-15	-2.63127	1	1	ZNF488	_
ENSG00000165443	5.13E-15	-2.63127	1	1	PHYHIPL	_
ENSG00000165471	5.13E-15	-2.63127	1	1	MBL2	K03991
ENSG00000165474	5.13E-15	-2.63127	1	1	GJB2	K07621
ENSG00000165478	5.13E-15	-2.63127	1	1	HEPACAM	_
ENSG00000165495	5.13E-15	-2.63127	1	1	PKNOX2	_
ENSG00000165496	5.13E-15	-2.63127	1	1	RPL10L	K02866
ENSG00000165509	5.13E-15	-2.63127	1	1	MAGEC3	_
ENSG00000165511	5.13E-15	-2.63127	1	1	C10orf25	_
ENSG00000165512	5.13E-15	-2.63127	1	1	ZNF22	K09228
ENSG00000165553	5.13E-15	-2.63127	1	1	NGB	_
ENSG00000165556	5.13E-15	-2.63127	1	1	CDX2	K09312
ENSG00000165566	5.13E-15	-2.63127	1	1	AMER2	K12581
ENSG00000165568	5.13E-15	-2.63127	1	1	AKR1E2	K13981
ENSG00000165583	5.13E-15	-2.63127	1	1	SSX5	K15624
ENSG00000165584	5.13E-15	-2.63127	1	1	SSX3	K15625
ENSG00000165588	5.13E-15	-2.63127	1	1	OTX2	K18490
ENSG00000165606	5.13E-15	-2.63127	1	1	DRGX	K09452
ENSG00000165621	5.13E-15	-2.63127	1	1	OXGR1	K08419
ENSG00000165623	5.13E-15	-2.63127	1	1	UCMA	_
ENSG00000165626	5.13E-15	-2.63127	1	1	BEND7	_
ENSG00000165633	5.13E-15	-2.63127	1	1	VSTM4	K17985
ENSG00000165643	5.13E-15	-2.63127	1	1	SOHLH1	_
ENSG00000165659	5.13E-15	-2.63127	1	1	DACH1	_
ENSG00000165682	5.13E-15	-2.63127	1	1	CLEC1B	K10070
ENSG00000165694	5.13E-15	-2.63127	1	1	FRMD7	_
ENSG00000165731	5.13E-15	-2.63127	1	1	RET	K05126
ENSG00000165762	5.13E-15	-2.63127	1	1	OR4K2	K04257
ENSG00000165794	5.13E-15	-2.63127	1	1	SLC39A2	K14709
ENSG00000165799	5.13E-15	-2.63127	1	1	RNASE7	K01172
ENSG00000165810	5.13E-15	-2.63127	1	1	BTNL9	K06712
ENSG00000165816	5.13E-15	-2.63127	1	1	VWA2	K09228
ENSG00000165821	5.13E-15	-2.63127	1	1	SALL2	_
ENSG00000165841	5.13E-15	-2.63127	1	1	CYP2C19	K17721
ENSG00000165862	5.13E-15	-2.63127	1	1	PNLIPRP2	K14075
ENSG00000165863	5.13E-15	-2.63127	1	1	C10orf82	_
ENSG00000165868	5.13E-15	-2.63127	1	1	HSPA12A	_
ENSG00000165874	5.13E-15	-2.63127	1	1	FAM35BP	_
ENSG00000165953	5.13E-15	-2.63127	1	1	SERPINA12	K04525
ENSG00000165970	5.13E-15	-2.63127	1	1	SLC6A5	K05038
ENSG00000165973	5.13E-15	-2.63127	1	1	NELL1	_
ENSG00000166006	5.13E-15	-2.63127	1	1	KCNC2	K04888
ENSG00000166007	5.13E-15	-2.63127	1	1	TRIM51HP	K12019
ENSG00000166008	5.13E-15	-2.63127	1	1	MAGEA9	_
ENSG00000166013	5.13E-15	-2.63127	1	1	TRIM53BP	K12019
ENSG00000166035	5.13E-15	-2.63127	1	1	LIPC	K01046
ENSG00000166049	5.13E-15	-2.63127	1	1	PASD1	_
ENSG00000166069	5.13E-15	-2.63127	1	1	TMCO5A	_
ENSG00000166090	5.13E-15	-2.63127	1	1	IL25	K05493

ENSG00000166091	5.13E-15	-2.63127	1	1	CMTM5	_
ENSG00000166104	5.13E-15	-2.63127	1	1	hsa-mir-7162	K17985
ENSG00000166105	5.13E-15	-2.63127	1	1	GLB1L3	_
ENSG00000166111	5.13E-15	-2.63127	1	1	SVOP	_
ENSG00000166118	5.13E-15	-2.63127	1	1	SPATA19	_
ENSG00000166148	5.13E-15	-2.63127	1	1	AVPR1A	K04226
ENSG00000166152	5.13E-15	-2.63127	1	1	C16orf78	_
ENSG00000166157	5.13E-15	-2.63127	1	1	TPTE	K18079
ENSG00000166160	5.13E-15	-2.63127	1	1	OPN1MW2	K04251
ENSG00000166206	5.13E-15	-2.63127	1	1	GABRB3	K05181
ENSG00000166211	5.13E-15	-2.63127	1	1	SPIC	K09440
ENSG00000166265	5.13E-15	-2.63127	1	1	CYYR1	_
ENSG00000166268	5.13E-15	-2.63127	1	1	MYRFL	_
ENSG00000166278	5.13E-15	-2.63127	1	1	C2	K01332
ENSG00000166292	5.13E-15	-2.63127	1	1	TMEM100	_
ENSG00000166329	5.13E-15	-2.63127	1	1	CCDC182	_
ENSG00000166342	5.13E-15	-2.63127	1	1	NETO1	_
ENSG00000166347	5.13E-15	-2.63127	1	1	CYB5A	_
ENSG00000166351	5.13E-15	-2.63127	1	1	POTED	K17299
ENSG00000166363	5.13E-15	-2.63127	1	1	OR10A5	K04257
ENSG00000166368	5.13E-15	-2.63127	1	1	OR2D2	K04257
ENSG00000166391	5.13E-15	-2.63127	1	1	MOGAT2	K14457
ENSG00000166405	5.13E-15	-2.63127	1	1	RIC3	_
ENSG00000166408	5.13E-15	-2.63127	1	1	OR5P1P	K04257
ENSG00000166426	5.13E-15	-2.63127	1	1	CRABP1	K17337
ENSG00000166448	5.13E-15	-2.63127	1	1	TMEM130	_
ENSG00000166503	5.13E-15	-2.63127	1	1	HDGFRP3	K16641
ENSG00000166509	5.13E-15	-2.63127	1	1	CLEC3A	K17519
ENSG00000166523	5.13E-15	-2.63127	1	1	CLEC4E	K10059
ENSG00000166527	5.13E-15	-2.63127	1	1	CLEC4D	K10058
ENSG00000166530	5.13E-15	-2.63127	1	1	HSBP1P2	K19765
ENSG00000166535	5.13E-15	-2.63127	1	1	A2ML1	_
ENSG00000166558	5.13E-15	-2.63127	1	1	SLC38A8	K14994
ENSG00000166569	5.13E-15	-2.63127	1	1	CPLX4	K15295
ENSG00000166573	5.13E-15	-2.63127	1	1	GALR1	K04230
ENSG00000166603	5.13E-15	-2.63127	1	1	MC4R	K04202
ENSG00000166634	5.13E-15	-2.63127	1	1	SERPINB12	K13966
ENSG00000166664	5.13E-15	-2.63127	1	1	CHRFAM7A	K04809
ENSG00000166693	5.13E-15	-2.63127	1	1	OR5M13P	K04257
ENSG00000166707	5.13E-15	-2.63127	1	1	ZCCHC18	_
ENSG00000166743	5.13E-15	-2.63127	1	1	ACSM1	K01896
ENSG00000166748	5.13E-15	-2.63127	1	1	AGBL1	_
ENSG00000166770	5.13E-15	-2.63127	1	1	ZNF667-AS1	_
ENSG00000166787	5.13E-15	-2.63127	1	1	SAA3P	K17310
ENSG00000166796	5.13E-15	-2.63127	1	1	LDHC	K00016
ENSG00000166800	5.13E-15	-2.63127	1	1	LDHAL6A	K00016
ENSG00000166828	5.13E-15	-2.63127	1	1	SCNN1G	K04827
ENSG00000166840	5.13E-15	-2.63127	1	1	GLYATL1	_
ENSG00000166862	5.13E-15	-2.63127	1	1	CACNG2	_
ENSG00000166869	5.13E-15	-2.63127	1	1	CHP2	K17611
ENSG00000166884	5.13E-15	-2.63127	1	1	OR4D6	K04257
ENSG00000166896	5.13E-15	-2.63127	1	1	XRCC6BP1	K18156
ENSG00000166917	5.13E-15	-2.63127	1	1	MIR202HG	_
ENSG00000166926	5.13E-15	-2.63127	1	1	MS4A6E	_
ENSG00000166928	5.13E-15	-2.63127	1	1	MS4A14	_
ENSG00000166930	5.13E-15	-2.63127	1	1	MS4A5	_
ENSG00000166948	5.13E-15	-2.63127	1	1	TGM6	K05624
ENSG00000166959	5.13E-15	-2.63127	1	1	MS4A8	_

ENSG00000166960	5.13E-15	-2.63127	1	1	CCDC178	_
ENSG00000166961	5.13E-15	-2.63127	1	1	MS4A15	_
ENSG00000166984	5.13E-15	-2.63127	1	1	TCP10L2	_
ENSG00000167014	5.13E-15	-2.63127	1	1	C15orf43	_
ENSG00000167080	5.13E-15	-2.63127	1	1	B4GALNT2	K09655
ENSG00000167098	5.13E-15	-2.63127	1	1	SUN5	_
ENSG00000167117	5.13E-15	-2.63127	1	1	LINC00483	_
ENSG00000167139	5.13E-15	-2.63127	1	1	TBC1D21	_
ENSG00000167194	5.13E-15	-2.63127	1	1	C16orf92	_
ENSG00000167195	5.13E-15	-2.63127	1	1	GOLGA6C	K10595
ENSG00000167207	5.13E-15	-2.63127	1	1	NOD2	K10165
ENSG00000167208	5.13E-15	-2.63127	1	1	SNX20	K17931
ENSG00000167210	5.13E-15	-2.63127	1	1	LOXHD1	_
ENSG00000167236	5.13E-15	-2.63127	1	1	CCL23	K05511
ENSG00000167286	5.13E-15	-2.63127	1	1	CD3D	K06450
ENSG00000167346	5.13E-15	-2.63127	1	1	MMP26	K08004
ENSG00000167359	5.13E-15	-2.63127	1	1	OR51I1	K04257
ENSG00000167360	5.13E-15	-2.63127	1	1	OR51Q1	K04257
ENSG00000167390	5.13E-15	-2.63127	1	1	POM121L3P	_
ENSG00000167419	5.13E-15	-2.63127	1	1	LPO	K12550
ENSG00000167434	5.13E-15	-2.63127	1	1	CA4	K18246
ENSG00000167459	5.13E-15	-2.63127	1	1	LINC00905	_
ENSG00000167494	5.13E-15	-2.63127	1	1	NOS2P2	K13241
ENSG00000167554	5.13E-15	-2.63127	1	1	ZNF610	K09228
ENSG00000167562	5.13E-15	-2.63127	1	1	ZNF701	K09228
ENSG00000167580	5.13E-15	-2.63127	1	1	AQP2	K09865
ENSG00000167608	5.13E-15	-2.63127	1	1	TMC4	_
ENSG00000167612	5.13E-15	-2.63127	1	1	ANKRD33	_
ENSG00000167613	5.13E-15	-2.63127	1	1	LAIR1	K06725
ENSG00000167614	5.13E-15	-2.63127	1	1	TTYH1	_
ENSG00000167618	5.13E-15	-2.63127	1	1	LAIR2	K06725
ENSG00000167633	5.13E-15	-2.63127	1	1	KIR3DL1	K07980
ENSG00000167634	5.13E-15	-2.63127	1	1	NLRP7	K19409
ENSG00000167653	5.13E-15	-2.63127	1	1	PSCA	_
ENSG00000167664	5.13E-15	-2.63127	1	1	TMIGD2	K16668
ENSG00000167741	5.13E-15	-2.63127	1	1	GGT6	K00681
ENSG00000167749	5.13E-15	-2.63127	1	1	KLK4	K08666
ENSG00000167755	5.13E-15	-2.63127	1	1	KLK6	K08667
ENSG00000167757	5.13E-15	-2.63127	1	1	KLK11	K09620
ENSG00000167759	5.13E-15	-2.63127	1	1	KLK13	K09463
ENSG00000167766	5.13E-15	-2.63127	1	1	ZNF83	K09228
ENSG00000167768	5.13E-15	-2.63127	1	1	KRT1	K07605
ENSG00000167769	5.13E-15	-2.63127	1	1	ACER1	K01441
ENSG00000167791	5.13E-15	-2.63127	1	1	CABP2	_
ENSG00000167798	5.13E-15	-2.63127	1	1	C3P1	K03990
ENSG00000167800	5.13E-15	-2.63127	1	1	TBX10	K10181
ENSG00000167807	5.13E-15	-2.63127	1	1	CTD-2369P2.10	_
ENSG00000167822	5.13E-15	-2.63127	1	1	OR8J3	K04257
ENSG00000167825	5.13E-15	-2.63127	1	1	OR5I1	K04257
ENSG00000167850	5.13E-15	-2.63127	1	1	CD300C	K06719
ENSG00000167851	5.13E-15	-2.63127	1	1	CD300A	K06719
ENSG00000167858	5.13E-15	-2.63127	1	1	TEKT1	K18628
ENSG00000167910	5.13E-15	-2.63127	1	1	CYP7A1	K00489
ENSG00000167912	5.13E-15	-2.63127	1	1	RP11-25K19.1	_
ENSG00000167914	5.13E-15	-2.63127	1	1	GSDMA	K12581
ENSG00000167916	5.13E-15	-2.63127	1	1	KRT24	K07604
ENSG00000167970	5.13E-15	-2.63127	1	1	AC009065.1	K14325
ENSG00000168004	5.13E-15	-2.63127	1	1	HRASLS5	_

ENSG00000168065	5.13E-15	-2.63127	1	1	SLC22A11	K08207
ENSG00000168070	5.13E-15	-2.63127	1	1	C11orf85	_
ENSG00000168081	5.13E-15	-2.63127	1	1	PNOC	_
ENSG00000168122	5.13E-15	-2.63127	1	1	ZNF355P	K09228
ENSG00000168129	5.13E-15	-2.63127	1	1	AC098817.5	K00287
ENSG00000168131	5.13E-15	-2.63127	1	1	OR2B2	K04257
ENSG00000168158	5.13E-15	-2.63127	1	1	OR2C1	K04257
ENSG00000168229	5.13E-15	-2.63127	1	1	PTGDR	K04332
ENSG00000168260	5.13E-15	-2.63127	1	1	C14orf183	_
ENSG00000168267	5.13E-15	-2.63127	1	1	PTF1A	K09073
ENSG00000168280	5.13E-15	-2.63127	1	1	KIF5C	K10396
ENSG00000168309	5.13E-15	-2.63127	1	1	FAM107A	_
ENSG00000168314	5.13E-15	-2.63127	1	1	MOBP	_
ENSG00000168329	5.13E-15	-2.63127	1	1	CX3CR1	K04192
ENSG00000168334	5.13E-15	-2.63127	1	1	XIRP1	_
ENSG00000168348	5.13E-15	-2.63127	1	1	INSM2	_
ENSG00000168394	5.13E-15	-2.63127	1	1	TAP1	K05653
ENSG00000168412	5.13E-15	-2.63127	1	1	MTNR1A	K04285
ENSG00000168418	5.13E-15	-2.63127	1	1	KCNG4	K04903
ENSG00000168421	5.13E-15	-2.63127	1	1	RHOH	_
ENSG00000168447	5.13E-15	-2.63127	1	1	SCNN1B	K04825
ENSG00000168477	5.13E-15	-2.63127	1	1	TNXB	K06252
ENSG00000168509	5.13E-15	-2.63127	1	1	HFE2	K06847
ENSG00000168515	5.13E-15	-2.63127	1	1	SCGB1D1	_
ENSG00000168530	5.13E-15	-2.63127	1	1	MYL1	K05738
ENSG00000168546	5.13E-15	-2.63127	1	1	GFRA2	_
ENSG00000168582	5.13E-15	-2.63127	1	1	CRYGA	_
ENSG00000168594	5.13E-15	-2.63127	1	1	ADAM29	K16069
ENSG00000168619	5.13E-15	-2.63127	1	1	ADAM18	K16909
ENSG00000168621	5.13E-15	-2.63127	1	1	GDNF	K05452
ENSG00000168630	5.13E-15	-2.63127	1	1	SPINT5P	_
ENSG00000168631	5.13E-15	-2.63127	1	1	DPCR1	_
ENSG00000168634	5.13E-15	-2.63127	1	1	WFDC13	_
ENSG00000168658	5.13E-15	-2.63127	1	1	VWA3B	_
ENSG00000168671	5.13E-15	-2.63127	1	1	UGT3A2	_
ENSG00000168703	5.13E-15	-2.63127	1	1	WFDC12	K10397
ENSG00000168746	5.13E-15	-2.63127	1	1	C20orf62	_
ENSG00000168757	5.13E-15	-2.63127	1	1	TSPY2	K11284
ENSG00000168785	5.13E-15	-2.63127	1	1	TSPAN5	K17345
ENSG00000168787	5.13E-15	-2.63127	1	1	OR12D2	K04257
ENSG00000168811	5.13E-15	-2.63127	1	1	IL12A	K05406
ENSG00000168828	5.13E-15	-2.63127	1	1	OR13J1	K04257
ENSG00000168830	5.13E-15	-2.63127	1	1	HTR1E	K04153
ENSG00000168852	5.13E-15	-2.63127	1	1	TPTE2P5	K18079
ENSG00000168875	5.13E-15	-2.63127	1	1	SOX14	K09267
ENSG00000168903	5.13E-15	-2.63127	1	1	BTNL3	K06712
ENSG00000168907	5.13E-15	-2.63127	1	1	PLA2G4F	K16342
ENSG00000168916	5.13E-15	-2.63127	1	1	ZNF608	_
ENSG00000168925	5.13E-15	-2.63127	1	1	CTRB1	K01310
ENSG00000168928	5.13E-15	-2.63127	1	1	CTRB2	K01310
ENSG00000168930	5.13E-15	-2.63127	1	1	TRIM49	K12019
ENSG00000168952	5.13E-15	-2.63127	1	1	STXBP6	K08519
ENSG00000168959	5.13E-15	-2.63127	1	1	GRM5	K04604
ENSG00000168967	5.13E-15	-2.63127	1	1	PMCHL1	K05229
ENSG00000168992	5.13E-15	-2.63127	1	1	OR7E102P	K04257
ENSG00000168995	5.13E-15	-2.63127	1	1	SIGLEC7	K06739
ENSG00000169006	5.13E-15	-2.63127	1	1	NTSR2	K04212
ENSG00000169035	5.13E-15	-2.63127	1	1	KLK7	K08668

ENSG00000169040	5.13E-15	-2.63127	1	1	PMCHL2	K05229
ENSG00000169059	5.13E-15	-2.63127	1	1	VCX3A	_
ENSG00000169064	5.13E-15	-2.63127	1	1	ZBBX	_
ENSG00000169075	5.13E-15	-2.63127	1	1	BRD7P3	K11723
ENSG00000169083	5.13E-15	-2.63127	1	1	AR	K08557
ENSG00000169116	5.13E-15	-2.63127	1	1	PARM1	_
ENSG00000169154	5.13E-15	-2.63127	1	1	GOT1L1	_
ENSG00000169164	5.13E-15	-2.63127	1	1	RP11-167P23.2	_
ENSG00000169181	5.13E-15	-2.63127	1	1	GSG1L	_
ENSG00000169194	5.13E-15	-2.63127	1	1	IL13	K05435
ENSG00000169202	5.13E-15	-2.63127	1	1	OR10G1P	K04257
ENSG00000169208	5.13E-15	-2.63127	1	1	OR10G3	K04257
ENSG00000169213	5.13E-15	-2.63127	1	1	RAB3B	K06108
ENSG00000169214	5.13E-15	-2.63127	1	1	OR6F1	K04257
ENSG00000169218	5.13E-15	-2.63127	1	1	RSPO1	K19471
ENSG00000169224	5.13E-15	-2.63127	1	1	GCSAML	_
ENSG00000169253	5.13E-15	-2.63127	1	1	RP11-220D10.1	K02920
ENSG00000169255	5.13E-15	-2.63127	1	1	B3GALNT1	_
ENSG00000169291	5.13E-15	-2.63127	1	1	SHE	K02927
ENSG00000169297	5.13E-15	-2.63127	1	1	NR0B1	K08562
ENSG00000169302	5.13E-15	-2.63127	1	1	STK32A	K08793
ENSG00000169306	5.13E-15	-2.63127	1	1	IL1RAPL1	K05170
ENSG00000169313	5.13E-15	-2.63127	1	1	P2RY12	K04298
ENSG00000169325	5.13E-15	-2.63127	1	1	GUSBP12	K01195
ENSG00000169327	5.13E-15	-2.63127	1	1	OR5AU1	K04257
ENSG00000169340	5.13E-15	-2.63127	1	1	PDILT	K09654
ENSG00000169344	5.13E-15	-2.63127	1	1	UMOD	K18274
ENSG00000169347	5.13E-15	-2.63127	1	1	GP2	K04613
ENSG00000169385	5.13E-15	-2.63127	1	1	RNASE2	K01168
ENSG00000169393	5.13E-15	-2.63127	1	1	ELSPBP1	_
ENSG00000169397	5.13E-15	-2.63127	1	1	RNASE3	K10787
ENSG00000169402	5.13E-15	-2.63127	1	1	RSPH10B2	_
ENSG00000169413	5.13E-15	-2.63127	1	1	RNASE6	K01172
ENSG00000169427	5.13E-15	-2.63127	1	1	KCNK9	K04919
ENSG00000169432	5.13E-15	-2.63127	1	1	SCN9A	K04841
ENSG00000169435	5.13E-15	-2.63127	1	1	RASSF6	K09854
ENSG00000169436	5.13E-15	-2.63127	1	1	COL22A1	K16630
ENSG00000169442	5.13E-15	-2.63127	1	1	CD52	K06488
ENSG00000169469	5.13E-15	-2.63127	1	1	SPRR1B	_
ENSG00000169474	5.13E-15	-2.63127	1	1	SPRR1A	_
ENSG00000169484	5.13E-15	-2.63127	1	1	OR4K14	K04257
ENSG00000169488	5.13E-15	-2.63127	1	1	OR4K15	K04257
ENSG00000169495	5.13E-15	-2.63127	1	1	HTRA4	K08786
ENSG00000169507	5.13E-15	-2.63127	1	1	SLC38A11	K14997
ENSG00000169509	5.13E-15	-2.63127	1	1	CRCT1	_
ENSG00000169515	5.13E-15	-2.63127	1	1	CCDC8	K17561
ENSG00000169548	5.13E-15	-2.63127	1	1	ZNF280A	_
ENSG00000169550	5.13E-15	-2.63127	1	1	MUC15	K19498
ENSG00000169551	5.13E-15	-2.63127	1	1	CT55	_
ENSG00000169562	5.13E-15	-2.63127	1	1	GJB1	K07620
ENSG00000169575	5.13E-15	-2.63127	1	1	VPREB1	K06553
ENSG00000169594	5.13E-15	-2.63127	1	1	BNC1	_
ENSG00000169618	5.13E-15	-2.63127	1	1	PROKR1	K08379
ENSG00000169662	5.13E-15	-2.63127	1	1	BCRP6	K08878
ENSG00000169676	5.13E-15	-2.63127	1	1	DRD5	K05840
ENSG00000169688	5.13E-15	-2.63127	1	1	MT1B	K14739
ENSG00000169704	5.13E-15	-2.63127	1	1	GP9	K06263
ENSG00000169715	5.13E-15	-2.63127	1	1	MT1E	K14739

ENSG00000169717	5.13E-15	-2.63127	1	1	ACTRT2	_
ENSG00000169744	5.13E-15	-2.63127	1	1	LDB2	_
ENSG00000169760	5.13E-15	-2.63127	1	1	NLGN1	K07378
ENSG00000169763	5.13E-15	-2.63127	1	1	PRYP3	_
ENSG00000169783	5.13E-15	-2.63127	1	1	LINGO1	_
ENSG00000169789	5.13E-15	-2.63127	1	1	PRY	_
ENSG00000169800	5.13E-15	-2.63127	1	1	RBMY1F	K12885
ENSG00000169807	5.13E-15	-2.63127	1	1	PRY2	_
ENSG00000169811	5.13E-15	-2.63127	1	1	RBMY1HP	K12885
ENSG00000169836	5.13E-15	-2.63127	1	1	TACR3	K04224
ENSG00000169840	5.13E-15	-2.63127	1	1	GSX1	K09310
ENSG00000169849	5.13E-15	-2.63127	1	1	TSPY14P	K11284
ENSG00000169856	5.13E-15	-2.63127	1	1	ONECUT1	K08026
ENSG00000169860	5.13E-15	-2.63127	1	1	P2RY1	K04270
ENSG00000169861	5.13E-15	-2.63127	1	1	IGHV1OR21-1	K06856
ENSG00000169862	5.13E-15	-2.63127	1	1	CTNND2	_
ENSG00000169876	5.13E-15	-2.63127	1	1	MUC17	_
ENSG00000169877	5.13E-15	-2.63127	1	1	AHSP	_
ENSG00000169900	5.13E-15	-2.63127	1	1	PYDC1	K12802
ENSG00000169906	5.13E-15	-2.63127	1	1	S100G	K14734
ENSG00000169933	5.13E-15	-2.63127	1	1	FRMPD4	K00948
ENSG00000169953	5.13E-15	-2.63127	1	1	HSFY2	_
ENSG00000170006	5.13E-15	-2.63127	1	1	TMEM154	_
ENSG00000170011	5.13E-15	-2.63127	1	1	MYRIP	_
ENSG00000170017	5.13E-15	-2.63127	1	1	ALCAM	K06547
ENSG00000170054	5.13E-15	-2.63127	1	1	SERPINA9	K04525
ENSG00000170074	5.13E-15	-2.63127	1	1	FAM153A	K12581
ENSG00000170085	5.13E-15	-2.63127	1	1	SIMC1	_
ENSG00000170091	5.13E-15	-2.63127	1	1	NSG2	_
ENSG00000170099	5.13E-15	-2.63127	1	1	SERPINA6	K04525
ENSG00000170128	5.13E-15	-2.63127	1	1	GPR25	K08405
ENSG00000170152	5.13E-15	-2.63127	1	1	RP11-374M1.7	_
ENSG00000170160	5.13E-15	-2.63127	1	1	CCDC144A	_
ENSG00000170165	5.13E-15	-2.63127	1	1	CR848007.2	K17299
ENSG00000170178	5.13E-15	-2.63127	1	1	HOXD12	K09297
ENSG00000170180	5.13E-15	-2.63127	1	1	GYPA	K06575
ENSG00000170209	5.13E-15	-2.63127	1	1	ANKK1	K16289
ENSG00000170214	5.13E-15	-2.63127	1	1	ADRA1B	K04136
ENSG00000170215	5.13E-15	-2.63127	1	1	FAM27B	_
ENSG00000170217	5.13E-15	-2.63127	1	1	RP11-12A20.2	_
ENSG00000170236	5.13E-15	-2.63127	1	1	USP50	K09228
ENSG00000170255	5.13E-15	-2.63127	1	1	MRGPRX1	K08396
ENSG00000170276	5.13E-15	-2.63127	1	1	HSPB2	K09543
ENSG00000170279	5.13E-15	-2.63127	1	1	C7orf33	_
ENSG00000170293	5.13E-15	-2.63127	1	1	CMTM8	_
ENSG00000170323	5.13E-15	-2.63127	1	1	FABP4	K08753
ENSG00000170324	5.13E-15	-2.63127	1	1	FRMPD2	_
ENSG00000170356	5.13E-15	-2.63127	1	1	OR2A20P	K04257
ENSG00000170367	5.13E-15	-2.63127	1	1	CST5	K13901
ENSG00000170369	5.13E-15	-2.63127	1	1	CST2	K13898
ENSG00000170370	5.13E-15	-2.63127	1	1	EMX2	K09317
ENSG00000170373	5.13E-15	-2.63127	1	1	CST1	K13897
ENSG00000170381	5.13E-15	-2.63127	1	1	SEMA3E	K06840
ENSG00000170396	5.13E-15	-2.63127	1	1	ZNF804A	_
ENSG00000170409	5.13E-15	-2.63127	1	1	CTA-313A17.2	K13055
ENSG00000170423	5.13E-15	-2.63127	1	1	KRT78	K07605
ENSG00000170426	5.13E-15	-2.63127	1	1	SDR9C7	K11154
ENSG00000170430	5.13E-15	-2.63127	1	1	MGMT	K00567

ENSG00000170439	5.13E-15	-2.63127	1	1	METTL7B	_
ENSG00000170465	5.13E-15	-2.63127	1	1	KRT6C	K07605
ENSG00000170484	5.13E-15	-2.63127	1	1	KRT74	K07605
ENSG00000170486	5.13E-15	-2.63127	1	1	KRT72	K07605
ENSG00000170500	5.13E-15	-2.63127	1	1	LONRF2	K17985
ENSG00000170516	5.13E-15	-2.63127	1	1	COX7B2	K02271
ENSG00000170542	5.13E-15	-2.63127	1	1	SERPINB9	K13963
ENSG00000170549	5.13E-15	-2.63127	1	1	IRX1	K04947
ENSG00000170558	5.13E-15	-2.63127	1	1	CDH2	K06736
ENSG00000170561	5.13E-15	-2.63127	1	1	IRX2	_
ENSG00000170571	5.13E-15	-2.63127	1	1	EMB	_
ENSG00000170590	5.13E-15	-2.63127	1	1	LINC00266-3	_
ENSG00000170605	5.13E-15	-2.63127	1	1	OR9K2	K04257
ENSG00000170608	5.13E-15	-2.63127	1	1	FOXA3	K08038
ENSG00000170613	5.13E-15	-2.63127	1	1	FAM71B	_
ENSG00000170616	5.13E-15	-2.63127	1	1	SCRT1	K09219
ENSG00000170624	5.13E-15	-2.63127	1	1	SGCD	K12563
ENSG00000170683	5.13E-15	-2.63127	1	1	OR10A3	K04257
ENSG00000170688	5.13E-15	-2.63127	1	1	OR5E1P	K04257
ENSG00000170743	5.13E-15	-2.63127	1	1	SYT9	_
ENSG00000170745	5.13E-15	-2.63127	1	1	KCNS3	K04933
ENSG00000170777	5.13E-15	-2.63127	1	1	TPD52L3	_
ENSG00000170782	5.13E-15	-2.63127	1	1	OR10A4	K04257
ENSG00000170788	5.13E-15	-2.63127	1	1	DYDC1	K09654
ENSG00000170790	5.13E-15	-2.63127	1	1	OR10A2	K04257
ENSG00000170803	5.13E-15	-2.63127	1	1	OR2AG1	K04257
ENSG00000170807	5.13E-15	-2.63127	1	1	LMOD2	_
ENSG00000170819	5.13E-15	-2.63127	1	1	BFSP2	K10379
ENSG00000170820	5.13E-15	-2.63127	1	1	FSHR	K04247
ENSG00000170827	5.13E-15	-2.63127	1	1	CELP	K12298
ENSG00000170837	5.13E-15	-2.63127	1	1	GPR27	K04301
ENSG00000170858	5.13E-15	-2.63127	1	1	LILRP2	K06512
ENSG00000170866	5.13E-15	-2.63127	1	1	LILRA3	K06512
ENSG00000170889	5.13E-15	-2.63127	1	1	RPS9	K02997
ENSG00000170890	5.13E-15	-2.63127	1	1	PLA2G1B	K01047
ENSG00000170891	5.13E-15	-2.63127	1	1	CYTL1	_
ENSG00000170892	5.13E-15	-2.63127	1	1	TSEN34	K15323
ENSG00000170893	5.13E-15	-2.63127	1	1	TRH	K05253
ENSG00000170906	5.13E-15	-2.63127	1	1	NDUFA3	K03947
ENSG00000170909	5.13E-15	-2.63127	1	1	OSCAR	K14377
ENSG00000170920	5.13E-15	-2.63127	1	1	OR7G3	K04257
ENSG00000170923	5.13E-15	-2.63127	1	1	OR7G2	K04257
ENSG00000170925	5.13E-15	-2.63127	1	1	TEX13B	_
ENSG00000170929	5.13E-15	-2.63127	1	1	OR1M1	K04257
ENSG00000170948	5.13E-15	-2.63127	1	1	MBD3L1	_
ENSG00000170950	5.13E-15	-2.63127	1	1	PGK2	K00927
ENSG00000170953	5.13E-15	-2.63127	1	1	OR8B12	K04257
ENSG00000170954	5.13E-15	-2.63127	1	1	ZNF415	K09228
ENSG00000170956	5.13E-15	-2.63127	1	1	CEACAM3	K06499
ENSG00000170959	5.13E-15	-2.63127	1	1	DCDC1	_
ENSG00000170967	5.13E-15	-2.63127	1	1	DDI1	K11885
ENSG00000170983	5.13E-15	-2.63127	1	1	LINC00208	_
ENSG00000170989	5.13E-15	-2.63127	1	1	S1PR1	K04288
ENSG00000171004	5.13E-15	-2.63127	1	1	HS6ST2	K08102
ENSG00000171014	5.13E-15	-2.63127	1	1	OR4D5	K04257
ENSG00000171033	5.13E-15	-2.63127	1	1	PKIA	K15985
ENSG00000171044	5.13E-15	-2.63127	1	1	XKR6	_
ENSG00000171049	5.13E-15	-2.63127	1	1	FPR2	K04173

ENSG00000171053	5.13E-15	-2.63127	1	1	PATE1	_
ENSG00000171054	5.13E-15	-2.63127	1	1	OR13H1	K04257
ENSG00000171056	5.13E-15	-2.63127	1	1	SOX7	K09270
ENSG00000171094	5.13E-15	-2.63127	1	1	ALK	K05119
ENSG00000171101	5.13E-15	-2.63127	1	1	SIGLEC17P	K06473
ENSG00000171102	5.13E-15	-2.63127	1	1	OBP2B	_
ENSG00000171116	5.13E-15	-2.63127	1	1	HSFX1	_
ENSG00000171124	5.13E-15	-2.63127	1	1	FUT3	K00716
ENSG00000171129	5.13E-15	-2.63127	1	1	HSFX2	_
ENSG00000171130	5.13E-15	-2.63127	1	1	ATP6V0E2	K02153
ENSG00000171133	5.13E-15	-2.63127	1	1	OR2K2	K04257
ENSG00000171136	5.13E-15	-2.63127	1	1	RLN3	K05255
ENSG00000171180	5.13E-15	-2.63127	1	1	OR2M4	K04257
ENSG00000171195	5.13E-15	-2.63127	1	1	MUC7	K13909
ENSG00000171199	5.13E-15	-2.63127	1	1	PROL1	_
ENSG00000171201	5.13E-15	-2.63127	1	1	SMR3B	_
ENSG00000171209	5.13E-15	-2.63127	1	1	CSN3	K17282
ENSG00000171234	5.13E-15	-2.63127	1	1	UGT2B7	K00699
ENSG00000171243	5.13E-15	-2.63127	1	1	SOSTDC1	_
ENSG00000171360	5.13E-15	-2.63127	1	1	KRT38	K07604
ENSG00000171396	5.13E-15	-2.63127	1	1	KRTAP4-4	_
ENSG00000171402	5.13E-15	-2.63127	1	1	XAGE3	_
ENSG00000171405	5.13E-15	-2.63127	1	1	XAGE5	_
ENSG00000171431	5.13E-15	-2.63127	1	1	KRT20	K07604
ENSG00000171433	5.13E-15	-2.63127	1	1	GLOD5	_
ENSG00000171435	5.13E-15	-2.63127	1	1	KSR2	K18529
ENSG00000171446	5.13E-15	-2.63127	1	1	KRT27	K07604
ENSG00000171459	5.13E-15	-2.63127	1	1	OR1L6	K04257
ENSG00000171476	5.13E-15	-2.63127	1	1	HOPX	_
ENSG00000171478	5.13E-15	-2.63127	1	1	SPACA5B	K01185
ENSG00000171481	5.13E-15	-2.63127	1	1	OR1L3	K04257
ENSG00000171483	5.13E-15	-2.63127	1	1	SSX6	K15624
ENSG00000171484	5.13E-15	-2.63127	1	1	OR1B1	K04257
ENSG00000171487	5.13E-15	-2.63127	1	1	NLRP5	_
ENSG00000171488	5.13E-15	-2.63127	1	1	LRRC8C	_
ENSG00000171489	5.13E-15	-2.63127	1	1	SPACA5	K01185
ENSG00000171495	5.13E-15	-2.63127	1	1	MROH2B	_
ENSG00000171496	5.13E-15	-2.63127	1	1	OR1L8	K04257
ENSG00000171501	5.13E-15	-2.63127	1	1	OR1N2	K04257
ENSG00000171505	5.13E-15	-2.63127	1	1	OR1N1	K04257
ENSG00000171517	5.13E-15	-2.63127	1	1	LPAR3	K04294
ENSG00000171540	5.13E-15	-2.63127	1	1	OTP	_
ENSG00000171561	5.13E-15	-2.63127	1	1	OR2AT4	K04257
ENSG00000171564	5.13E-15	-2.63127	1	1	FGB	K03904
ENSG00000171587	5.13E-15	-2.63127	1	1	DSCAM	K06767
ENSG00000171595	5.13E-15	-2.63127	1	1	DNAI2	K11143
ENSG00000171596	5.13E-15	-2.63127	1	1	NMUR1	K05052
ENSG00000171611	5.13E-15	-2.63127	1	1	PTCRA	K06056
ENSG00000171643	5.13E-15	-2.63127	1	1	S100Z	_
ENSG00000171671	5.13E-15	-2.63127	1	1	SHANK2-AS3	_
ENSG00000171695	5.13E-15	-2.63127	1	1	C20orf201	_
ENSG00000171711	5.13E-15	-2.63127	1	1	DEFB4A	_
ENSG00000171714	5.13E-15	-2.63127	1	1	ANO5	K19480
ENSG00000171722	5.13E-15	-2.63127	1	1	C1orf111	_
ENSG00000171724	5.13E-15	-2.63127	1	1	VAT1L	_
ENSG00000171759	5.13E-15	-2.63127	1	1	PAH	K09067
ENSG00000171772	5.13E-15	-2.63127	1	1	SYCE1	K19534
ENSG00000171773	5.13E-15	-2.63127	1	1	NXNL1	_

ENSG00000171777	5.13E-15	-2.63127	1	1	RASGRP4	K12363
ENSG00000171794	5.13E-15	-2.63127	1	1	UTF1	_
ENSG00000171804	5.13E-15	-2.63127	1	1	WDR87	_
ENSG00000171811	5.13E-15	-2.63127	1	1	TTC40	_
ENSG00000171815	5.13E-15	-2.63127	1	1	PCDHB1	K16494
ENSG00000171819	5.13E-15	-2.63127	1	1	ANGPTL7	_
ENSG00000171864	5.13E-15	-2.63127	1	1	PRND	K17985
ENSG00000171885	5.13E-15	-2.63127	1	1	AQP4	K09866
ENSG00000171903	5.13E-15	-2.63127	1	1	CYP4F11	K17729
ENSG00000171936	5.13E-15	-2.63127	1	1	OR10H3	K04257
ENSG00000171942	5.13E-15	-2.63127	1	1	OR10H2	K04257
ENSG00000171944	5.13E-15	-2.63127	1	1	OR52A5	K04257
ENSG00000171954	5.13E-15	-2.63127	1	1	CYP4F22	K17731
ENSG00000171987	5.13E-15	-2.63127	1	1	C11orf40	_
ENSG00000171989	5.13E-15	-2.63127	1	1	LDHAL6B	K00016
ENSG00000171999	5.13E-15	-2.63127	1	1	OR52P2P	K04257
ENSG00000172014	5.13E-15	-2.63127	1	1	ANKRD20A4	K17299
ENSG00000172016	5.13E-15	-2.63127	1	1	REG3A	_
ENSG00000172020	5.13E-15	-2.63127	1	1	GAP43	_
ENSG00000172023	5.13E-15	-2.63127	1	1	REG1B	_
ENSG00000172031	5.13E-15	-2.63127	1	1	EPHX4	K12581
ENSG00000172116	5.13E-15	-2.63127	1	1	CD8B	K06459
ENSG00000172137	5.13E-15	-2.63127	1	1	CALB2	_
ENSG00000172146	5.13E-15	-2.63127	1	1	OR1A1	K04257
ENSG00000172148	5.13E-15	-2.63127	1	1	AC005255.6	K04257
ENSG00000172150	5.13E-15	-2.63127	1	1	OR1A2	K04257
ENSG00000172154	5.13E-15	-2.63127	1	1	OR8I2	K04257
ENSG00000172155	5.13E-15	-2.63127	1	1	LCE1D	_
ENSG00000172156	5.13E-15	-2.63127	1	1	CCL11	K16597
ENSG00000172179	5.13E-15	-2.63127	1	1	PRL	K05439
ENSG00000172186	5.13E-15	-2.63127	1	1	HMGN1P35	K11299
ENSG00000172188	5.13E-15	-2.63127	1	1	OR4C11	K04257
ENSG00000172199	5.13E-15	-2.63127	1	1	OR8U1	K04257
ENSG00000172201	5.13E-15	-2.63127	1	1	ID4	K17695
ENSG00000172208	5.13E-15	-2.63127	1	1	OR4X2	K04257
ENSG00000172209	5.13E-15	-2.63127	1	1	GPR22	K04319
ENSG00000172236	5.13E-15	-2.63127	1	1	TPSAB1	K01340
ENSG00000172238	5.13E-15	-2.63127	1	1	ATOH1	K09083
ENSG00000172283	5.13E-15	-2.63127	1	1	PRYP4	_
ENSG00000172288	5.13E-15	-2.63127	1	1	CDY1	K00653
ENSG00000172289	5.13E-15	-2.63127	1	1	OR10V1	K04257
ENSG00000172292	5.13E-15	-2.63127	1	1	CERS6	K04710
ENSG00000172294	5.13E-15	-2.63127	1	1	CSPG4P4Y	K08115
ENSG00000172297	5.13E-15	-2.63127	1	1	GOLGA2P3Y	_
ENSG00000172318	5.13E-15	-2.63127	1	1	B3GALT1	K07819
ENSG00000172320	5.13E-15	-2.63127	1	1	OR5A1	K04257
ENSG00000172322	5.13E-15	-2.63127	1	1	CLEC12A	K17516
ENSG00000172324	5.13E-15	-2.63127	1	1	OR5A2	K04257
ENSG00000172332	5.13E-15	-2.63127	1	1	AC012005.2	_
ENSG00000172342	5.13E-15	-2.63127	1	1	CSPG4P3Y	K08115
ENSG00000172348	5.13E-15	-2.63127	1	1	RCAN2	K17903
ENSG00000172352	5.13E-15	-2.63127	1	1	CDY1B	K00653
ENSG00000172362	5.13E-15	-2.63127	1	1	OR5B12	K04257
ENSG00000172365	5.13E-15	-2.63127	1	1	OR5B2	K04257
ENSG00000172377	5.13E-15	-2.63127	1	1	OR9I1	K04257
ENSG00000172381	5.13E-15	-2.63127	1	1	OR6Q1	K04257
ENSG00000172399	5.13E-15	-2.63127	1	1	MYOZ2	_
ENSG00000172410	5.13E-15	-2.63127	1	1	INSL5	K05255

ENSG00000172421	5.13E-15	-2.63127	1	1	EFCAB3	_
ENSG00000172425	5.13E-15	-2.63127	1	1	TTC36	_
ENSG00000172457	5.13E-15	-2.63127	1	1	OR9G4	K04257
ENSG00000172459	5.13E-15	-2.63127	1	1	OR5AR1	K04257
ENSG00000172461	5.13E-15	-2.63127	1	1	FUT9	K03663
ENSG00000172464	5.13E-15	-2.63127	1	1	OR5AP2	K04257
ENSG00000172468	5.13E-15	-2.63127	1	1	HSFY1	_
ENSG00000172487	5.13E-15	-2.63127	1	1	OR8J1	K04257
ENSG00000172489	5.13E-15	-2.63127	1	1	OR5T3	K04257
ENSG00000172497	5.13E-15	-2.63127	1	1	ACOT12	K01067
ENSG00000172519	5.13E-15	-2.63127	1	1	OR10H5	K04257
ENSG00000172538	5.13E-15	-2.63127	1	1	FAM170B	_
ENSG00000172548	5.13E-15	-2.63127	1	1	NIPAL4	_
ENSG00000172551	5.13E-15	-2.63127	1	1	MUCL1	_
ENSG00000172554	5.13E-15	-2.63127	1	1	SNTG2	K14209
ENSG00000172568	5.13E-15	-2.63127	1	1	FNDC9	_
ENSG00000172578	5.13E-15	-2.63127	1	1	KLHL6	K10444
ENSG00000172640	5.13E-15	-2.63127	1	1	OR10AD1	K04257
ENSG00000172653	5.13E-15	-2.63127	1	1	C17orf66	_
ENSG00000172673	5.13E-15	-2.63127	1	1	THEMIS	_
ENSG00000172680	5.13E-15	-2.63127	1	1	MOS	K04367
ENSG00000172689	5.13E-15	-2.63127	1	1	MS4A10	K12581
ENSG00000172716	5.13E-15	-2.63127	1	1	SLFN11	_
ENSG00000172724	5.13E-15	-2.63127	1	1	CCL19	K05512
ENSG00000172733	5.13E-15	-2.63127	1	1	PURG	_
ENSG00000172742	5.13E-15	-2.63127	1	1	OR4D9	K04257
ENSG00000172746	5.13E-15	-2.63127	1	1	RP11-344H11.4	K02870
ENSG00000172769	5.13E-15	-2.63127	1	1	OR5B3	K04257
ENSG00000172772	5.13E-15	-2.63127	1	1	OR10W1	K04257
ENSG00000172774	5.13E-15	-2.63127	1	1	OR1S1	K04257
ENSG00000172782	5.13E-15	-2.63127	1	1	FADS6	K12419
ENSG00000172794	5.13E-15	-2.63127	1	1	RAB37	K07914
ENSG00000172799	5.13E-15	-2.63127	1	1	ZBTB8OSP2	_
ENSG00000172817	5.13E-15	-2.63127	1	1	CYP7B1	K07430
ENSG00000172824	5.13E-15	-2.63127	1	1	CES4A	_
ENSG00000172867	5.13E-15	-2.63127	1	1	KRT2	K07605
ENSG00000172900	5.13E-15	-2.63127	1	1	AP002387.1	_
ENSG00000172912	5.13E-15	-2.63127	1	1	COX6B1P3	K02267
ENSG00000172915	5.13E-15	-2.63127	1	1	NBEA	_
ENSG00000172935	5.13E-15	-2.63127	1	1	MRGPRF	K08394
ENSG00000172938	5.13E-15	-2.63127	1	1	MRGPRD	K08392
ENSG00000172940	5.13E-15	-2.63127	1	1	SLC22A13	K08209
ENSG00000172969	5.13E-15	-2.63127	1	1	FRG2C	K19328
ENSG00000172971	5.13E-15	-2.63127	1	1	UNC93B3	K04257
ENSG00000172987	5.13E-15	-2.63127	1	1	HPSE2	K07965
ENSG00000172995	5.13E-15	-2.63127	1	1	ARPP21	_
ENSG00000173040	5.13E-15	-2.63127	1	1	EVC2	K19608
ENSG00000173077	5.13E-15	-2.63127	1	1	1-Dec	K11447
ENSG00000173093	5.13E-15	-2.63127	1	1	CCDC63	_
ENSG00000173157	5.13E-15	-2.63127	1	1	ADAMTS20	K09609
ENSG00000173200	5.13E-15	-2.63127	1	1	PARP15	K15261
ENSG00000173208	5.13E-15	-2.63127	1	1	ABCD2	K05676
ENSG00000173210	5.13E-15	-2.63127	1	1	ABLIM3	K07520
ENSG00000173213	5.13E-15	-2.63127	1	1	RP11-683L23.1	K07375
ENSG00000173231	5.13E-15	-2.63127	1	1	TERF1P1	K11110
ENSG00000173239	5.13E-15	-2.63127	1	1	LIPM	K19406
ENSG00000173250	5.13E-15	-2.63127	1	1	GPR151	K08435
ENSG00000173253	5.13E-15	-2.63127	1	1	DMRT2	K19489

ENSG00000173285	5.13E-15	-2.63127	1	1	OR10K1	K04257
ENSG00000173302	5.13E-15	-2.63127	1	1	GPR148	K08432
ENSG00000173335	5.13E-15	-2.63127	1	1	CST9	K13905
ENSG00000173349	5.13E-15	-2.63127	1	1	SFT2D3	_
ENSG00000173357	5.13E-15	-2.63127	1	1	AC007967.3	K12885
ENSG00000173366	5.13E-15	-2.63127	1	1	TLR9	K10161
ENSG00000173369	5.13E-15	-2.63127	1	1	C1QB	K03987
ENSG00000173372	5.13E-15	-2.63127	1	1	C1QA	K03986
ENSG00000173376	5.13E-15	-2.63127	1	1	NDNF	_
ENSG00000173389	5.13E-15	-2.63127	1	1	IQCF1	_
ENSG00000173404	5.13E-15	-2.63127	1	1	INSM1	_
ENSG00000173406	5.13E-15	-2.63127	1	1	DAB1	_
ENSG00000173431	5.13E-15	-2.63127	1	1	RNASE8	K01172
ENSG00000173452	5.13E-15	-2.63127	1	1	TMEM196	_
ENSG00000173464	5.13E-15	-2.63127	1	1	RNASE11	K16633
ENSG00000173467	5.13E-15	-2.63127	1	1	AGR3	_
ENSG00000173530	5.13E-15	-2.63127	1	1	TNFRSF10D	K04722
ENSG00000173557	5.13E-15	-2.63127	1	1	C2orf70	_
ENSG00000173572	5.13E-15	-2.63127	1	1	NLRP13	_
ENSG00000173578	5.13E-15	-2.63127	1	1	XCR1	K04193
ENSG00000173585	5.13E-15	-2.63127	1	1	CCR9	K04184
ENSG00000173597	5.13E-15	-2.63127	1	1	SULT1B1	K01025
ENSG00000173610	5.13E-15	-2.63127	1	1	UGT2A1	K00699
ENSG00000173612	5.13E-15	-2.63127	1	1	GPRC6A	K04622
ENSG00000173627	5.13E-15	-2.63127	1	1	APOBEC4	K18773
ENSG00000173679	5.13E-15	-2.63127	1	1	OR1L1	K04257
ENSG00000173699	5.13E-15	-2.63127	1	1	SPATA3	_
ENSG00000173714	5.13E-15	-2.63127	1	1	WFIKKN2	_
ENSG00000173769	5.13E-15	-2.63127	1	1	TOPAZ1	_
ENSG00000173805	5.13E-15	-2.63127	1	1	HAP1	K04647
ENSG00000173810	5.13E-15	-2.63127	1	1	PPIAP7	K03767
ENSG00000173862	5.13E-15	-2.63127	1	1	RP11-89N17.1	_
ENSG00000173876	5.13E-15	-2.63127	1	1	TUBB8	K07375
ENSG00000173908	5.13E-15	-2.63127	1	1	KRT28	K07604
ENSG00000173930	5.13E-15	-2.63127	1	1	SLCO4C1	K14355
ENSG00000173947	5.13E-15	-2.63127	1	1	PIFO	_
ENSG00000173954	5.13E-15	-2.63127	1	1	SNURFL	_
ENSG00000173966	5.13E-15	-2.63127	1	1	RP11-18M17.1	_
ENSG00000173976	5.13E-15	-2.63127	1	1	RAX2	K09333
ENSG00000173988	5.13E-15	-2.63127	1	1	LRRC63	_
ENSG00000174015	5.13E-15	-2.63127	1	1	SPERT	_
ENSG00000174016	5.13E-15	-2.63127	1	1	FAM46D	_
ENSG00000174059	5.13E-15	-2.63127	1	1	CD34	K06474
ENSG00000174083	5.13E-15	-2.63127	1	1	PIK3R6	_
ENSG00000174100	5.13E-15	-2.63127	1	1	MRPL45	K17426
ENSG00000174123	5.13E-15	-2.63127	1	1	TLR10	K10171
ENSG00000174145	5.13E-15	-2.63127	1	1	KIAA1239	_
ENSG00000174156	5.13E-15	-2.63127	1	1	GSTA3	K00799
ENSG00000174175	5.13E-15	-2.63127	1	1	SELP	K06496
ENSG00000174226	5.13E-15	-2.63127	1	1	SNX31	K17937
ENSG00000174255	5.13E-15	-2.63127	1	1	ZNF80	K09228
ENSG00000174279	5.13E-15	-2.63127	1	1	EVX2	K09320
ENSG00000174325	5.13E-15	-2.63127	1	1	DIRC1	_
ENSG00000174339	5.13E-15	-2.63127	1	1	OR2Y1	K04257
ENSG00000174348	5.13E-15	-2.63127	1	1	PODN	K11447
ENSG00000174368	5.13E-15	-2.63127	1	1	PMS2P2	K10858
ENSG00000174370	5.13E-15	-2.63127	1	1	C11orf45	K04995
ENSG00000174384	5.13E-15	-2.63127	1	1	RP11-313P13.4	K10858

ENSG00000174403	5.13E-15	-2.63127	1	1	C20orf166-AS1	_
ENSG00000174407	5.13E-15	-2.63127	1	1	C20orf166	_
ENSG00000174408	5.13E-15	-2.63127	1	1	PPIAP24	K03767
ENSG00000174417	5.13E-15	-2.63127	1	1	TRHR	K04282
ENSG00000174418	5.13E-15	-2.63127	1	1	RPL35P9	K02918
ENSG00000174429	5.13E-15	-2.63127	1	1	ABRA	_
ENSG00000174450	5.13E-15	-2.63127	1	1	GOLGA6L2	_
ENSG00000174453	5.13E-15	-2.63127	1	1	VWC2L	_
ENSG00000174460	5.13E-15	-2.63127	1	1	ZCCHC12	_
ENSG00000174469	5.13E-15	-2.63127	1	1	CNTNAP2	K07380
ENSG00000174495	5.13E-15	-2.63127	1	1	AC005017.2	K03767
ENSG00000174567	5.13E-15	-2.63127	1	1	GOLT1A	_
ENSG00000174572	5.13E-15	-2.63127	1	1	RP11-209A2.1	K02866
ENSG00000174599	5.13E-15	-2.63127	1	1	TRAM1L1	K14010
ENSG00000174600	5.13E-15	-2.63127	1	1	CMKLR1	K04245
ENSG00000174611	5.13E-15	-2.63127	1	1	KY	_
ENSG00000174667	5.13E-15	-2.63127	1	1	OR7D4	K04257
ENSG00000174677	5.13E-15	-2.63127	1	1	VN1R6P	K04614
ENSG00000174678	5.13E-15	-2.63127	1	1	FAM47DP	_
ENSG00000174680	5.13E-15	-2.63127	1	1	GRIK1-AS1	_
ENSG00000174697	5.13E-15	-2.63127	1	1	LEP	K05424
ENSG00000174740	5.13E-15	-2.63127	1	1	PABPC5	K13126
ENSG00000174776	5.13E-15	-2.63127	1	1	WDR49	_
ENSG00000174792	5.13E-15	-2.63127	1	1	C4orf26	K09228
ENSG00000174808	5.13E-15	-2.63127	1	1	BTC	K09783
ENSG00000174876	5.13E-15	-2.63127	1	1	AMY1B	K01176
ENSG00000174899	5.13E-15	-2.63127	1	1	C3orf55	_
ENSG00000174914	5.13E-15	-2.63127	1	1	OR9G1	K04257
ENSG00000174930	5.13E-15	-2.63127	1	1	VN2R1P	K04613
ENSG00000174937	5.13E-15	-2.63127	1	1	OR5M3	K04257
ENSG00000174942	5.13E-15	-2.63127	1	1	OR5R1	K04257
ENSG00000174944	5.13E-15	-2.63127	1	1	P2RY14	K04299
ENSG00000174946	5.13E-15	-2.63127	1	1	GPR171	K08441
ENSG00000174948	5.13E-15	-2.63127	1	1	GPR149	K08433
ENSG00000174950	5.13E-15	-2.63127	1	1	CD164L2	K10104
ENSG00000174957	5.13E-15	-2.63127	1	1	OR5J2	K04257
ENSG00000174963	5.13E-15	-2.63127	1	1	ZIC4	K09225
ENSG00000174970	5.13E-15	-2.63127	1	1	OR10AG1	K04257
ENSG00000174982	5.13E-15	-2.63127	1	1	OR4S2	K04257
ENSG00000175040	5.13E-15	-2.63127	1	1	CHST2	K04745
ENSG00000175065	5.13E-15	-2.63127	1	1	DSG4	K07599
ENSG00000175093	5.13E-15	-2.63127	1	1	SPSB4	K10343
ENSG00000175097	5.13E-15	-2.63127	1	1	RAG2	K10988
ENSG00000175143	5.13E-15	-2.63127	1	1	OR2T1	K04257
ENSG00000175161	5.13E-15	-2.63127	1	1	CADM2	K06782
ENSG00000175164	5.13E-15	-2.63127	1	1	ABO	_
ENSG00000175175	5.13E-15	-2.63127	1	1	PPM1E	K17501
ENSG00000175202	5.13E-15	-2.63127	1	1	HIGD2B	K18038
ENSG00000175264	5.13E-15	-2.63127	1	1	CHST1	K01022
ENSG00000175267	5.13E-15	-2.63127	1	1	VWA3A	_
ENSG00000175302	5.13E-15	-2.63127	1	1	ANKRD30BP1	_
ENSG00000175311	5.13E-15	-2.63127	1	1	ANKS4B	K06115
ENSG00000175319	5.13E-15	-2.63127	1	1	NF1P5	K08052
ENSG00000175325	5.13E-15	-2.63127	1	1	PROP1	K09327
ENSG00000175329	5.13E-15	-2.63127	1	1	ISX	_
ENSG00000175336	5.13E-15	-2.63127	1	1	APOF	K17985
ENSG00000175344	5.13E-15	-2.63127	1	1	CHRNA7	K04809
ENSG00000175352	5.13E-15	-2.63127	1	1	NRIP3	K13215

ENSG00000175393	5.13E-15	-2.63127	1	1	OR10A6	K04257
ENSG00000175398	5.13E-15	-2.63127	1	1	OR10P1	K04257
ENSG00000175485	5.13E-15	-2.63127	1	1	OR52W1	K04257
ENSG00000175489	5.13E-15	-2.63127	1	1	LRRC25	K17985
ENSG00000175497	5.13E-15	-2.63127	1	1	DPP10	_
ENSG00000175509	5.13E-15	-2.63127	1	1	RP11-395L14.13	K01317
ENSG00000175513	5.13E-15	-2.63127	1	1	TSGA10IP	_
ENSG00000175514	5.13E-15	-2.63127	1	1	GPR152	K08436
ENSG00000175520	5.13E-15	-2.63127	1	1	UBQLN3	K04523
ENSG00000175535	5.13E-15	-2.63127	1	1	PNLIP	K14073
ENSG00000175544	5.13E-15	-2.63127	1	1	CABP4	K08436
ENSG00000175604	5.13E-15	-2.63127	1	1	RP11-276H1.3	K06115
ENSG00000175619	5.13E-15	-2.63127	1	1	OR4B1	K04257
ENSG00000175646	5.13E-15	-2.63127	1	1	PRM1	_
ENSG00000175658	5.13E-15	-2.63127	1	1	DRD5P2	K05840
ENSG00000175664	5.13E-15	-2.63127	1	1	TEX26	_
ENSG00000175676	5.13E-15	-2.63127	1	1	GOLGA8EP	K10595
ENSG00000175699	5.13E-15	-2.63127	1	1	LINC00521	_
ENSG00000175707	5.13E-15	-2.63127	1	1	C1orf172	_
ENSG00000175718	5.13E-15	-2.63127	1	1	RBMXL3	K12885
ENSG00000175728	5.13E-15	-2.63127	1	1	C11orf44	K09228
ENSG00000175746	5.13E-15	-2.63127	1	1	C15orf54	_
ENSG00000175766	5.13E-15	-2.63127	1	1	EIF4E1B	K03259
ENSG00000175779	5.13E-15	-2.63127	1	1	C15orf53	_
ENSG00000175785	5.13E-15	-2.63127	1	1	PRIMA1	_
ENSG00000175800	5.13E-15	-2.63127	1	1	OR52B3P	K04257
ENSG00000175809	5.13E-15	-2.63127	1	1	ZNF645	K15714
ENSG00000175827	5.13E-15	-2.63127	1	1	AP001266.1	K19022
ENSG00000175857	5.13E-15	-2.63127	1	1	GAPT	_
ENSG00000175877	5.13E-15	-2.63127	1	1	WBSCR28	_
ENSG00000175893	5.13E-15	-2.63127	1	1	ZDHHC21	K18932
ENSG00000175894	5.13E-15	-2.63127	1	1	TSPEAR	_
ENSG00000175928	5.13E-15	-2.63127	1	1	LRRN1	_
ENSG00000175967	5.13E-15	-2.63127	1	1	RP11-544L8__B.4	_
ENSG00000175985	5.13E-15	-2.63127	1	1	PLEKHD1	_
ENSG00000176007	5.13E-15	-2.63127	1	1	FAM220BP	_
ENSG00000176020	5.13E-15	-2.63127	1	1	AMIGO3	K00966
ENSG00000176034	5.13E-15	-2.63127	1	1	CHDC2	_
ENSG00000176040	5.13E-15	-2.63127	1	1	TMPRSS7	K09638
ENSG00000176043	5.13E-15	-2.63127	1	1	RP1-146I3.1	K02920
ENSG00000176049	5.13E-15	-2.63127	1	1	JAKMIP2	K12581
ENSG00000176075	5.13E-15	-2.63127	1	1	LINC00302	_
ENSG00000176082	5.13E-15	-2.63127	1	1	AL357153.1	_
ENSG00000176083	5.13E-15	-2.63127	1	1	ZNF683	_
ENSG00000176115	5.13E-15	-2.63127	1	1	AQP7P4	K08771
ENSG00000176134	5.13E-15	-2.63127	1	1	AL445665.1	K13402
ENSG00000176136	5.13E-15	-2.63127	1	1	MC5R	K04203
ENSG00000176160	5.13E-15	-2.63127	1	1	HSF5	_
ENSG00000176165	5.13E-15	-2.63127	1	1	FOXG1	K09385
ENSG00000176177	5.13E-15	-2.63127	1	1	ENTHD1	_
ENSG00000176183	5.13E-15	-2.63127	1	1	CH17-12M21.1	K05692
ENSG00000176194	5.13E-15	-2.63127	1	1	CIDEA	_
ENSG00000176198	5.13E-15	-2.63127	1	1	OR11H4	K04257
ENSG00000176200	5.13E-15	-2.63127	1	1	OR4D11	K04257
ENSG00000176204	5.13E-15	-2.63127	1	1	LRRTM4	_
ENSG00000176219	5.13E-15	-2.63127	1	1	OR11H6	K04257
ENSG00000176227	5.13E-15	-2.63127	1	1	CTAGE15	_
ENSG00000176230	5.13E-15	-2.63127	1	1	OR4K17	K04257

ENSG00000176231	5.13E-15	-2.63127	1	1	OR10H4	K04257
ENSG00000176232	5.13E-15	-2.63127	1	1	RP5-1193P9.4	K04614
ENSG00000176239	5.13E-15	-2.63127	1	1	OR51B6	K04257
ENSG00000176243	5.13E-15	-2.63127	1	1	CDV3P1	_
ENSG00000176246	5.13E-15	-2.63127	1	1	OR4L1	K04257
ENSG00000176253	5.13E-15	-2.63127	1	1	OR4K13	K04257
ENSG00000176256	5.13E-15	-2.63127	1	1	HMGB4	K11297
ENSG00000176268	5.13E-15	-2.63127	1	1	CYCSP34	K08738
ENSG00000176269	5.13E-15	-2.63127	1	1	OR4F21	K04257
ENSG00000176274	5.13E-15	-2.63127	1	1	SLC25A53	_
ENSG00000176281	5.13E-15	-2.63127	1	1	OR4K5	K04257
ENSG00000176289	5.13E-15	-2.63127	1	1	IDSP1	K01136
ENSG00000176290	5.13E-15	-2.63127	1	1	OR4K3	K04257
ENSG00000176293	5.13E-15	-2.63127	1	1	ZNF135	K09228
ENSG00000176294	5.13E-15	-2.63127	1	1	OR4N2	K04257
ENSG00000176299	5.13E-15	-2.63127	1	1	OR4M1	K04257
ENSG00000176302	5.13E-15	-2.63127	1	1	FOXR1	_
ENSG00000176305	5.13E-15	-2.63127	1	1	AC087269.1	K06087
ENSG00000176312	5.13E-15	-2.63127	1	1	OR4H12P	K04257
ENSG00000176318	5.13E-15	-2.63127	1	1	FOXN3P1	K09407
ENSG00000176320	5.13E-15	-2.63127	1	1	RP11-404O13.5	_
ENSG00000176343	5.13E-15	-2.63127	1	1	RPL37AP8	K02921
ENSG00000176378	5.13E-15	-2.63127	1	1	PFN1P10	K05759
ENSG00000176381	5.13E-15	-2.63127	1	1	PRR18	K06236
ENSG00000176395	5.13E-15	-2.63127	1	1	CEACAM20	_
ENSG00000176402	5.13E-15	-2.63127	1	1	GJC3	K07628
ENSG00000176435	5.13E-15	-2.63127	1	1	CLEC14A	K17528
ENSG00000176495	5.13E-15	-2.63127	1	1	OR5AN1	K04257
ENSG00000176532	5.13E-15	-2.63127	1	1	PRR15	_
ENSG00000176540	5.13E-15	-2.63127	1	1	OR4C5	K04257
ENSG00000176547	5.13E-15	-2.63127	1	1	OR4C3	K04257
ENSG00000176555	5.13E-15	-2.63127	1	1	OR4S1	K04257
ENSG00000176566	5.13E-15	-2.63127	1	1	DCAF4L2	K11799
ENSG00000176567	5.13E-15	-2.63127	1	1	OR4X1	K04257
ENSG00000176584	5.13E-15	-2.63127	1	1	DMBT1P1	K13912
ENSG00000176593	5.13E-15	-2.63127	1	1	CTD-2368P22.1	K12581
ENSG00000176595	5.13E-15	-2.63127	1	1	KBTBD11	K10476
ENSG00000176605	5.13E-15	-2.63127	1	1	C14orf177	_
ENSG00000176635	5.13E-15	-2.63127	1	1	HORMAD2	_
ENSG00000176641	5.13E-15	-2.63127	1	1	RNF152	_
ENSG00000176654	5.13E-15	-2.63127	1	1	NANOGP1	K10164
ENSG00000176659	5.13E-15	-2.63127	1	1	C20orf197	_
ENSG00000176679	5.13E-15	-2.63127	1	1	TGIF2LY	K19554
ENSG00000176695	5.13E-15	-2.63127	1	1	OR4F17	K04257
ENSG00000176716	5.13E-15	-2.63127	1	1	OR10AB1P	K04257
ENSG00000176728	5.13E-15	-2.63127	1	1	TTTY14	_
ENSG00000176734	5.13E-15	-2.63127	1	1	TRIL	_
ENSG00000176742	5.13E-15	-2.63127	1	1	OR51V1	K04257
ENSG00000176748	5.13E-15	-2.63127	1	1	OR52Z1	K04257
ENSG00000176752	5.13E-15	-2.63127	1	1	OR51A1P	K04257
ENSG00000176753	5.13E-15	-2.63127	1	1	C15orf56	_
ENSG00000176754	5.13E-15	-2.63127	1	1	LINC00303	_
ENSG00000176769	5.13E-15	-2.63127	1	1	TCERG1L	K16467
ENSG00000176782	5.13E-15	-2.63127	1	1	DEFB104A	_
ENSG00000176787	5.13E-15	-2.63127	1	1	OR52E2	K04257
ENSG00000176797	5.13E-15	-2.63127	1	1	DEFB103A	_
ENSG00000176798	5.13E-15	-2.63127	1	1	OR51L1	K04257
ENSG00000176812	5.13E-15	-2.63127	1	1	RP11-629O4.1	K03439

ENSG00000176824	5.13E-15	-2.63127	1	1	AC009951.1	K10408
ENSG00000176855	5.13E-15	-2.63127	1	1	KRT18P28	K07604
ENSG00000176857	5.13E-15	-2.63127	1	1	GJA1P1	K07372
ENSG00000176868	5.13E-15	-2.63127	1	1	AL358781.1	_
ENSG00000176879	5.13E-15	-2.63127	1	1	OR51G1	K04257
ENSG00000176882	5.13E-15	-2.63127	1	1	RP11-432M24.4	_
ENSG00000176887	5.13E-15	-2.63127	1	1	SOX11	K09268
ENSG00000176893	5.13E-15	-2.63127	1	1	OR51G2	K04257
ENSG00000176895	5.13E-15	-2.63127	1	1	OR51A7	K04257
ENSG00000176900	5.13E-15	-2.63127	1	1	OR51T1	K04257
ENSG00000176904	5.13E-15	-2.63127	1	1	OR51H1P	K04257
ENSG00000176922	5.13E-15	-2.63127	1	1	OR51S1	K04257
ENSG00000176923	5.13E-15	-2.63127	1	1	OR7A15P	K04257
ENSG00000176925	5.13E-15	-2.63127	1	1	OR51F2	K04257
ENSG00000176928	5.13E-15	-2.63127	1	1	GCNT4	K09663
ENSG00000176937	5.13E-15	-2.63127	1	1	OR52R1	K04257
ENSG00000176951	5.13E-15	-2.63127	1	1	OR51N1P	K04257
ENSG00000176956	5.13E-15	-2.63127	1	1	LY6H	K06846
ENSG00000176970	5.13E-15	-2.63127	1	1	RPL7L1P11	_
ENSG00000176979	5.13E-15	-2.63127	1	1	TRIM60	K12029
ENSG00000176984	5.13E-15	-2.63127	1	1	AP000679.2	_
ENSG00000176988	5.13E-15	-2.63127	1	1	FMR1NB	_
ENSG00000176998	5.13E-15	-2.63127	1	1	HCG4	K06751
ENSG00000177023	5.13E-15	-2.63127	1	1	DEFB104B	_
ENSG00000177025	5.13E-15	-2.63127	1	1	C19orf18	_
ENSG00000177047	5.13E-15	-2.63127	1	1	IFNW1	K05440
ENSG00000177103	5.13E-15	-2.63127	1	1	DSCAML1	K06768
ENSG00000177108	5.13E-15	-2.63127	1	1	ZDHHC22	K18932
ENSG00000177112	5.13E-15	-2.63127	1	1	MRVI1-AS1	_
ENSG00000177133	5.13E-15	-2.63127	1	1	LINC00982	_
ENSG00000177143	5.13E-15	-2.63127	1	1	CETN1	K16465
ENSG00000177151	5.13E-15	-2.63127	1	1	OR2T35	K04257
ENSG00000177174	5.13E-15	-2.63127	1	1	OR14C36	K04257
ENSG00000177181	5.13E-15	-2.63127	1	1	RIMKLA	K18311
ENSG00000177186	5.13E-15	-2.63127	1	1	OR2M7	K04257
ENSG00000177201	5.13E-15	-2.63127	1	1	OR2T12	K04257
ENSG00000177212	5.13E-15	-2.63127	1	1	OR2T33	K04257
ENSG00000177233	5.13E-15	-2.63127	1	1	OR2M1P	K04257
ENSG00000177234	5.13E-15	-2.63127	1	1	C10orf85	_
ENSG00000177243	5.13E-15	-2.63127	1	1	DEFB103B	_
ENSG00000177257	5.13E-15	-2.63127	1	1	DEFB4B	_
ENSG00000177261	5.13E-15	-2.63127	1	1	ENSAP3	_
ENSG00000177272	5.13E-15	-2.63127	1	1	KCNA3	K04876
ENSG00000177275	5.13E-15	-2.63127	1	1	OR2AJ1	K04257
ENSG00000177294	5.13E-15	-2.63127	1	1	FBXO39	K10314
ENSG00000177300	5.13E-15	-2.63127	1	1	CLDN22	K06087
ENSG00000177301	5.13E-15	-2.63127	1	1	KCNA2	K04875
ENSG00000177306	5.13E-15	-2.63127	1	1	OR7E125P	K04257
ENSG00000177324	5.13E-15	-2.63127	1	1	BEND2	K17985
ENSG00000177338	5.13E-15	-2.63127	1	1	LINC00469	_
ENSG00000177350	5.13E-15	-2.63127	1	1	RPL13AP3	K02872
ENSG00000177354	5.13E-15	-2.63127	1	1	C10orf71	_
ENSG00000177369	5.13E-15	-2.63127	1	1	RP1-62O9.3	_
ENSG00000177398	5.13E-15	-2.63127	1	1	UMODL1	_
ENSG00000177400	5.13E-15	-2.63127	1	1	OR7E8P	K04257
ENSG00000177414	5.13E-15	-2.63127	1	1	UBE2U	K10584
ENSG00000177418	5.13E-15	-2.63127	1	1	AC006001.1	_
ENSG00000177447	5.13E-15	-2.63127	1	1	CBX3P1	K11586

ENSG00000177462	5.13E-15	-2.63127	1	1	OR2T8	K04257
ENSG00000177465	5.13E-15	-2.63127	1	1	ACOT4	K01068
ENSG00000177468	5.13E-15	-2.63127	1	1	OLIG3	K09085
ENSG00000177476	5.13E-15	-2.63127	1	1	OR2G3	K04257
ENSG00000177489	5.13E-15	-2.63127	1	1	OR2G2	K04257
ENSG00000177504	5.13E-15	-2.63127	1	1	VCX2	_
ENSG00000177511	5.13E-15	-2.63127	1	1	ST8SIA3	_
ENSG00000177535	5.13E-15	-2.63127	1	1	OR2B11	K04257
ENSG00000177551	5.13E-15	-2.63127	1	1	NHLH2	K09075
ENSG00000177553	5.13E-15	-2.63127	1	1	RP11-56N19.5	_
ENSG00000177558	5.13E-15	-2.63127	1	1	FAM187B	_
ENSG00000177575	5.13E-15	-2.63127	1	1	CD163	K06545
ENSG00000177586	5.13E-15	-2.63127	1	1	OR7E149P	K04257
ENSG00000177590	5.13E-15	-2.63127	1	1	RP5-1051J4.6	_
ENSG00000177596	5.13E-15	-2.63127	1	1	AL355390.1	_
ENSG00000177599	5.13E-15	-2.63127	1	1	ZNF491	K09228
ENSG00000177673	5.13E-15	-2.63127	1	1	C2orf57	_
ENSG00000177679	5.13E-15	-2.63127	1	1	SRRM3	_
ENSG00000177684	5.13E-15	-2.63127	1	1	DEFB114	_
ENSG00000177689	5.13E-15	-2.63127	1	1	MAGEB10	K17985
ENSG00000177692	5.13E-15	-2.63127	1	1	DNAJC28	K19373
ENSG00000177693	5.13E-15	-2.63127	1	1	OR4F4	K04257
ENSG00000177699	5.13E-15	-2.63127	1	1	RP11-16K12.1	_
ENSG00000177725	5.13E-15	-2.63127	1	1	AC105206.1	K09228
ENSG00000177736	5.13E-15	-2.63127	1	1	RP11-474P12.3	K19054
ENSG00000177752	5.13E-15	-2.63127	1	1	YIPF7	_
ENSG00000177776	5.13E-15	-2.63127	1	1	RPS2P2	K02981
ENSG00000177800	5.13E-15	-2.63127	1	1	TMEM78	K09488
ENSG00000177803	5.13E-15	-2.63127	1	1	RP11-606P2.1	K16197
ENSG00000177820	5.13E-15	-2.63127	1	1	AC004917.1	_
ENSG00000177839	5.13E-15	-2.63127	1	1	PCDHB9	K16494
ENSG00000177853	5.13E-15	-2.63127	1	1	ZNF518A	_
ENSG00000177875	5.13E-15	-2.63127	1	1	C12orf68	_
ENSG00000177910	5.13E-15	-2.63127	1	1	SPATA31C2	_
ENSG00000177932	5.13E-15	-2.63127	1	1	ZNF354C	K09228
ENSG00000177938	5.13E-15	-2.63127	1	1	CAPZA3	K10364
ENSG00000177947	5.13E-15	-2.63127	1	1	ODF3	_
ENSG00000177992	5.13E-15	-2.63127	1	1	SPATA31E1	_
ENSG00000177994	5.13E-15	-2.63127	1	1	C2orf73	_
ENSG00000178015	5.13E-15	-2.63127	1	1	GPR150	K08434
ENSG00000178021	5.13E-15	-2.63127	1	1	TSPYL6	K11289
ENSG00000178031	5.13E-15	-2.63127	1	1	ADAMTSL1	_
ENSG00000178053	5.13E-15	-2.63127	1	1	MLF1	K15622
ENSG00000178055	5.13E-15	-2.63127	1	1	PRSS42	_
ENSG00000178081	5.13E-15	-2.63127	1	1	ULK4P3	K17545
ENSG00000178115	5.13E-15	-2.63127	1	1	GOLGA8Q	K10595
ENSG00000178130	5.13E-15	-2.63127	1	1	FAM27L	_
ENSG00000178163	5.13E-15	-2.63127	1	1	ZNF518B	_
ENSG00000178175	5.13E-15	-2.63127	1	1	ZNF366	_
ENSG00000178187	5.13E-15	-2.63127	1	1	ZNF454	K09228
ENSG00000178206	5.13E-15	-2.63127	1	1	CYP4F31P	K17726
ENSG00000178217	5.13E-15	-2.63127	1	1	SH2D4B	_
ENSG00000178235	5.13E-15	-2.63127	1	1	SLITRK1	_
ENSG00000178243	5.13E-15	-2.63127	1	1	C9orf62	_
ENSG00000178248	5.13E-15	-2.63127	1	1	AP000345.1	_
ENSG00000178257	5.13E-15	-2.63127	1	1	PRM3	_
ENSG00000178279	5.13E-15	-2.63127	1	1	TNP2	_
ENSG00000178287	5.13E-15	-2.63127	1	1	SPAG11A	_

ENSG00000178343	5.13E-15	-2.63127	1	1	SHISA3	_
ENSG00000178358	5.13E-15	-2.63127	1	1	OR2D3	K04257
ENSG00000178363	5.13E-15	-2.63127	1	1	CALML3	K02183
ENSG00000178372	5.13E-15	-2.63127	1	1	CALML5	K02183
ENSG00000178394	5.13E-15	-2.63127	1	1	HTR1A	K04153
ENSG00000178395	5.13E-15	-2.63127	1	1	C1orf65	_
ENSG00000178403	5.13E-15	-2.63127	1	1	NEUROG2	K09082
ENSG00000178409	5.13E-15	-2.63127	1	1	BEND3	_
ENSG00000178457	5.13E-15	-2.63127	1	1	LINC00314	_
ENSG00000178462	5.13E-15	-2.63127	1	1	TUBAL3	K07374
ENSG00000178473	5.13E-15	-2.63127	1	1	UCN3	K05257
ENSG00000178498	5.13E-15	-2.63127	1	1	DTX3	K06058
ENSG00000178502	5.13E-15	-2.63127	1	1	KLHL11	K10449
ENSG00000178503	5.13E-15	-2.63127	1	1	NECAP1P1	_
ENSG00000178522	5.13E-15	-2.63127	1	1	AMBN	_
ENSG00000178538	5.13E-15	-2.63127	1	1	CA8	K01672
ENSG00000178550	5.13E-15	-2.63127	1	1	AC010170.1	K01869
ENSG00000178556	5.13E-15	-2.63127	1	1	CKS1BP6	K02219
ENSG00000178562	5.13E-15	-2.63127	1	1	CD28	K06470
ENSG00000178568	5.13E-15	-2.63127	1	1	ERBB4	K05085
ENSG00000178573	5.13E-15	-2.63127	1	1	MAF	K09035
ENSG00000178586	5.13E-15	-2.63127	1	1	OR6B3	K04257
ENSG00000178591	5.13E-15	-2.63127	1	1	DEFB125	_
ENSG00000178596	5.13E-15	-2.63127	1	1	GAPDHP29	K00134
ENSG00000178602	5.13E-15	-2.63127	1	1	OTOS	_
ENSG00000178636	5.13E-15	-2.63127	1	1	RP11-455G16.1	K12581
ENSG00000178642	5.13E-15	-2.63127	1	1	AL513477.1	K04613
ENSG00000178645	5.13E-15	-2.63127	1	1	C10orf53	_
ENSG00000178654	5.13E-15	-2.63127	1	1	RP11-366I21.1	K03767
ENSG00000178662	5.13E-15	-2.63127	1	1	CSRNP3	K04834
ENSG00000178690	5.13E-15	-2.63127	1	1	DYNAP	_
ENSG00000178722	5.13E-15	-2.63127	1	1	C5orf64	_
ENSG00000178732	5.13E-15	-2.63127	1	1	GP5	K06260
ENSG00000178750	5.13E-15	-2.63127	1	1	STX19	K08487
ENSG00000178762	5.13E-15	-2.63127	1	1	HIST1H2BPS1	K11252
ENSG00000178789	5.13E-15	-2.63127	1	1	CD300LB	_
ENSG00000178795	5.13E-15	-2.63127	1	1	GDPD4	_
ENSG00000178796	5.13E-15	-2.63127	1	1	RIIAD1	_
ENSG00000178803	5.13E-15	-2.63127	1	1	ADORA2A-AS1	_
ENSG00000178804	5.13E-15	-2.63127	1	1	H1FOO	_
ENSG00000178828	5.13E-15	-2.63127	1	1	RNF186	_
ENSG00000178836	5.13E-15	-2.63127	1	1	AC114812.5	K09509
ENSG00000178863	5.13E-15	-2.63127	1	1	CEBPA-AS1	_
ENSG00000178894	5.13E-15	-2.63127	1	1	AC073416.1	_
ENSG00000178928	5.13E-15	-2.63127	1	1	TPRX1	_
ENSG00000178934	5.13E-15	-2.63127	1	1	LGALS7B	K10092
ENSG00000178947	5.13E-15	-2.63127	1	1	LINC00086	_
ENSG00000178965	5.13E-15	-2.63127	1	1	C1orf173	_
ENSG00000179002	5.13E-15	-2.63127	1	1	TAS1R2	K04625
ENSG00000179008	5.13E-15	-2.63127	1	1	C14orf39	_
ENSG00000179028	5.13E-15	-2.63127	1	1	RP11-363G10.2	_
ENSG00000179031	5.13E-15	-2.63127	1	1	LL0XNC01-131B10	K14842
ENSG00000179038	5.13E-15	-2.63127	1	1	AP001885.1	K15712
ENSG00000179055	5.13E-15	-2.63127	1	1	OR13D1	K04257
ENSG00000179071	5.13E-15	-2.63127	1	1	CCDC89	_
ENSG00000179073	5.13E-15	-2.63127	1	1	TAAR3	K05051
ENSG00000179088	5.13E-15	-2.63127	1	1	C12orf42	_
ENSG00000179097	5.13E-15	-2.63127	1	1	HTR1F	K04153

ENSG00000179104	5.13E-15	-2.63127	1	1	TMTC2	_
ENSG00000179131	5.13E-15	-2.63127	1	1	RP11-458F8.3	K02910
ENSG00000179141	5.13E-15	-2.63127	1	1	MTUS2-AS1	_
ENSG00000179142	5.13E-15	-2.63127	1	1	CYP11B2	K00497
ENSG00000179144	5.13E-15	-2.63127	1	1	GIMAP7	_
ENSG00000179157	5.13E-15	-2.63127	1	1	RPS2P28	K02981
ENSG00000179170	5.13E-15	-2.63127	1	1	OR7E97P	K04257
ENSG00000179172	5.13E-15	-2.63127	1	1	HNRNPCL1	K12884
ENSG00000179178	5.13E-15	-2.63127	1	1	TMEM125	_
ENSG00000179213	5.13E-15	-2.63127	1	1	SIGLECL1	_
ENSG00000179222	5.13E-15	-2.63127	1	1	MAGED1	K12464
ENSG00000179253	5.13E-15	-2.63127	1	1	RP11-429E11.3	K08834
ENSG00000179270	5.13E-15	-2.63127	1	1	C2orf71	_
ENSG00000179277	5.13E-15	-2.63127	1	1	MEIS3P1	K16671
ENSG00000179299	5.13E-15	-2.63127	1	1	NSUN7	_
ENSG00000179300	5.13E-15	-2.63127	1	1	ZCCHC5	_
ENSG00000179314	5.13E-15	-2.63127	1	1	WSCD1	_
ENSG00000179331	5.13E-15	-2.63127	1	1	RAB39A	K07924
ENSG00000179342	5.13E-15	-2.63127	1	1	GS1-124K5.9	K04635
ENSG00000179344	5.13E-15	-2.63127	1	1	HLA-DQB1	_
ENSG00000179363	5.13E-15	-2.63127	1	1	TMEM31	K05271
ENSG00000179397	5.13E-15	-2.63127	1	1	C1orf101	K16895
ENSG00000179412	5.13E-15	-2.63127	1	1	HNRNPCP5	K12884
ENSG00000179420	5.13E-15	-2.63127	1	1	OR6W1P	K04257
ENSG00000179443	5.13E-15	-2.63127	1	1	OR13C6P	K04257
ENSG00000179447	5.13E-15	-2.63127	1	1	RP5-1027G4.3	_
ENSG00000179449	5.13E-15	-2.63127	1	1	AC124309.1	_
ENSG00000179460	5.13E-15	-2.63127	1	1	EEF1A1P27	K03231
ENSG00000179467	5.13E-15	-2.63127	1	1	AL109806.1	K10653
ENSG00000179468	5.13E-15	-2.63127	1	1	OR9A2	K04257
ENSG00000179520	5.13E-15	-2.63127	1	1	SLC17A8	K12302
ENSG00000179542	5.13E-15	-2.63127	1	1	SLITRK4	_
ENSG00000179564	5.13E-15	-2.63127	1	1	LSMEM2	_
ENSG00000179571	5.13E-15	-2.63127	1	1	NBPF23	_
ENSG00000179577	5.13E-15	-2.63127	1	1	AP003471.1	_
ENSG00000179600	5.13E-15	-2.63127	1	1	GPHB5	_
ENSG00000179603	5.13E-15	-2.63127	1	1	GRM8	K04608
ENSG00000179615	5.13E-15	-2.63127	1	1	OR2AP1	K04257
ENSG00000179626	5.13E-15	-2.63127	1	1	OR6C4	K04257
ENSG00000179636	5.13E-15	-2.63127	1	1	TPPP2	_
ENSG00000179639	5.13E-15	-2.63127	1	1	FCER1A	K08089
ENSG00000179676	5.13E-15	-2.63127	1	1	LINC00305	_
ENSG00000179695	5.13E-15	-2.63127	1	1	OR6C2	K04257
ENSG00000179709	5.13E-15	-2.63127	1	1	NLRP8	K04264
ENSG00000179751	5.13E-15	-2.63127	1	1	SYCN	_
ENSG00000179755	5.13E-15	-2.63127	1	1	CTD-2144E22.5	_
ENSG00000179796	5.13E-15	-2.63127	1	1	LRRC3B	_
ENSG00000179799	5.13E-15	-2.63127	1	1	OR7E22P	K04257
ENSG00000179813	5.13E-15	-2.63127	1	1	FAM216B	_
ENSG00000179817	5.13E-15	-2.63127	1	1	MRGPRX4	K08396
ENSG00000179826	5.13E-15	-2.63127	1	1	MRGPRX3	K08396
ENSG00000179837	5.13E-15	-2.63127	1	1	RBM15B	K13190
ENSG00000179840	5.13E-15	-2.63127	1	1	C1orf200	_
ENSG00000179869	5.13E-15	-2.63127	1	1	ABCA13	K05647
ENSG00000179873	5.13E-15	-2.63127	1	1	NLRP11	_
ENSG00000179914	5.13E-15	-2.63127	1	1	ITLN1	K17527
ENSG00000179919	5.13E-15	-2.63127	1	1	OR10A7	K04257
ENSG00000179930	5.13E-15	-2.63127	1	1	ZNF648	K09230

ENSG00000179934	5.13E-15	-2.63127	1	1	CCR8	K04183
ENSG00000179935	5.13E-15	-2.63127	1	1	LINC00652	_
ENSG00000179938	5.13E-15	-2.63127	1	1	GOLGA8J	K10595
ENSG00000179978	5.13E-15	-2.63127	1	1	RP11-1319K7.1	K12807
ENSG00000179994	5.13E-15	-2.63127	1	1	SPDYE7P	K08694
ENSG00000179997	5.13E-15	-2.63127	1	1	AC010894.4	K10740
ENSG00000180016	5.13E-15	-2.63127	1	1	OR1E1	K04257
ENSG00000180042	5.13E-15	-2.63127	1	1	OR1R1P	K04257
ENSG00000180044	5.13E-15	-2.63127	1	1	C3orf80	_
ENSG00000180053	5.13E-15	-2.63127	1	1	NKX2-6	K09346
ENSG00000180061	5.13E-15	-2.63127	1	1	TMEM150B	_
ENSG00000180068	5.13E-15	-2.63127	1	1	OR3A4P	K04257
ENSG00000180081	5.13E-15	-2.63127	1	1	CTC-451A6.4	_
ENSG00000180083	5.13E-15	-2.63127	1	1	WFDC11	_
ENSG00000180090	5.13E-15	-2.63127	1	1	OR3A1	K04257
ENSG00000180116	5.13E-15	-2.63127	1	1	C12orf40	_
ENSG00000180138	5.13E-15	-2.63127	1	1	CSNK1A1L	K08957
ENSG00000180150	5.13E-15	-2.63127	1	1	HMGN2P9	K11300
ENSG00000180152	5.13E-15	-2.63127	1	1	AC079753.4	K04725
ENSG00000180172	5.13E-15	-2.63127	1	1	RPS12P23	K02951
ENSG00000180176	5.13E-15	-2.63127	1	1	TH	K00501
ENSG00000180190	5.13E-15	-2.63127	1	1	TDRP	_
ENSG00000180205	5.13E-15	-2.63127	1	1	WFDC9	_
ENSG00000180219	5.13E-15	-2.63127	1	1	FAM71C	_
ENSG00000180221	5.13E-15	-2.63127	1	1	TPT1P10	_
ENSG00000180230	5.13E-15	-2.63127	1	1	NACAP2	K03626
ENSG00000180251	5.13E-15	-2.63127	1	1	SLC9A4	K13961
ENSG00000180259	5.13E-15	-2.63127	1	1	PRNT	_
ENSG00000180269	5.13E-15	-2.63127	1	1	GPR139	K08428
ENSG00000180284	5.13E-15	-2.63127	1	1	RP5-1055C14.6	_
ENSG00000180287	5.13E-15	-2.63127	1	1	PLD5	K16861
ENSG00000180305	5.13E-15	-2.63127	1	1	WFDC10A	_
ENSG00000180318	5.13E-15	-2.63127	1	1	ALX1	K09334
ENSG00000180332	5.13E-15	-2.63127	1	1	KCTD4	_
ENSG00000180344	5.13E-15	-2.63127	1	1	AL035460.1	_
ENSG00000180347	5.13E-15	-2.63127	1	1	CCDC129	_
ENSG00000180383	5.13E-15	-2.63127	1	1	DEFB124	K07847
ENSG00000180386	5.13E-15	-2.63127	1	1	KRTAP9-7	_
ENSG00000180389	5.13E-15	-2.63127	1	1	ATP5EP2	K02135
ENSG00000180409	5.13E-15	-2.63127	1	1	OR10AA1P	K04257
ENSG00000180424	5.13E-15	-2.63127	1	1	DEFB123	_
ENSG00000180432	5.13E-15	-2.63127	1	1	CYP8B1	K07431
ENSG00000180433	5.13E-15	-2.63127	1	1	OR6K6	K04257
ENSG00000180437	5.13E-15	-2.63127	1	1	OR6K4P	K04257
ENSG00000180438	5.13E-15	-2.63127	1	1	TPRXL	K16618
ENSG00000180440	5.13E-15	-2.63127	1	1	SERTM1	_
ENSG00000180458	5.13E-15	-2.63127	1	1	CTD-3064H18.4	_
ENSG00000180475	5.13E-15	-2.63127	1	1	OR10Q1	K04257
ENSG00000180483	5.13E-15	-2.63127	1	1	DEFB119	_
ENSG00000180509	5.13E-15	-2.63127	1	1	KCNE1	K17985
ENSG00000180525	5.13E-15	-2.63127	1	1	PRR26	_
ENSG00000180532	5.13E-15	-2.63127	1	1	ZSCAN4	K09230
ENSG00000180535	5.13E-15	-2.63127	1	1	BHLHA15	K08040
ENSG00000180543	5.13E-15	-2.63127	1	1	TSPYL5	K11288
ENSG00000180613	5.13E-15	-2.63127	1	1	GSX2	K09310
ENSG00000180636	5.13E-15	-2.63127	1	1	OR4E1	K04257
ENSG00000180644	5.13E-15	-2.63127	1	1	PRF1	K07818
ENSG00000180658	5.13E-15	-2.63127	1	1	OR2A4	K04257

ENSG00000180660	5.13E-15	-2.63127	1	1	MAB21L1	_
ENSG00000180662	5.13E-15	-2.63127	1	1	RPL21P8	K02889
ENSG00000180663	5.13E-15	-2.63127	1	1	VN1R3	K04614
ENSG00000180708	5.13E-15	-2.63127	1	1	OR10K2	K04257
ENSG00000180712	5.13E-15	-2.63127	1	1	RP11-290F5.2	_
ENSG00000180714	5.13E-15	-2.63127	1	1	OR5AZ1P	K04257
ENSG00000180723	5.13E-15	-2.63127	1	1	OR51A9P	K04257
ENSG00000180725	5.13E-15	-2.63127	1	1	AC015871.1	_
ENSG00000180730	5.13E-15	-2.63127	1	1	SHISA2	K12581
ENSG00000180745	5.13E-15	-2.63127	1	1	CLRN3	_
ENSG00000180770	5.13E-15	-2.63127	1	1	OR7E129P	K04257
ENSG00000180771	5.13E-15	-2.63127	1	1	SRSF8	K12891
ENSG00000180772	5.13E-15	-2.63127	1	1	AGTR2	K04167
ENSG00000180777	5.13E-15	-2.63127	1	1	ANKRD30B	_
ENSG00000180785	5.13E-15	-2.63127	1	1	OR51E1	K04257
ENSG00000180803	5.13E-15	-2.63127	1	1	RP11-467D10.2	_
ENSG00000180828	5.13E-15	-2.63127	1	1	BHLHE22	K09086
ENSG00000180861	5.13E-15	-2.63127	1	1	C12orf36	_
ENSG00000180869	5.13E-15	-2.63127	1	1	C1orf180	_
ENSG00000180871	5.13E-15	-2.63127	1	1	CXCR2	K05050
ENSG00000180872	5.13E-15	-2.63127	1	1	DEFB112	_
ENSG00000180875	5.13E-15	-2.63127	1	1	GREM2	_
ENSG00000180909	5.13E-15	-2.63127	1	1	OR52B1P	K04257
ENSG00000180910	5.13E-15	-2.63127	1	1	TTTY11	_
ENSG00000180913	5.13E-15	-2.63127	1	1	OR56B3P	K04257
ENSG00000180919	5.13E-15	-2.63127	1	1	OR56B4	K04257
ENSG00000180926	5.13E-15	-2.63127	1	1	OR7E18P	K04257
ENSG00000180929	5.13E-15	-2.63127	1	1	GPR62	K08415
ENSG00000180934	5.13E-15	-2.63127	1	1	OR56A1	K04257
ENSG00000180974	5.13E-15	-2.63127	1	1	OR52E4	K04257
ENSG00000180987	5.13E-15	-2.63127	1	1	AC123768.1	_
ENSG00000180988	5.13E-15	-2.63127	1	1	OR52N2	K04257
ENSG00000181001	5.13E-15	-2.63127	1	1	OR52N1	K04257
ENSG00000181007	5.13E-15	-2.63127	1	1	ZFP82	K09228
ENSG00000181009	5.13E-15	-2.63127	1	1	OR52N5	K04257
ENSG00000181013	5.13E-15	-2.63127	1	1	C17orf47	K16943
ENSG00000181017	5.13E-15	-2.63127	1	1	OR56B2P	K04257
ENSG00000181023	5.13E-15	-2.63127	1	1	OR56B1	K04257
ENSG00000181031	5.13E-15	-2.63127	1	1	RPH3AL	_
ENSG00000181036	5.13E-15	-2.63127	1	1	FCRL6	K17273
ENSG00000181072	5.13E-15	-2.63127	1	1	CHRM2	_
ENSG00000181074	5.13E-15	-2.63127	1	1	OR52N4	K04257
ENSG00000181092	5.13E-15	-2.63127	1	1	ADIPOQ	K07296
ENSG00000181101	5.13E-15	-2.63127	1	1	SDCCAG3P2	_
ENSG00000181109	5.13E-15	-2.63127	1	1	OR52P1P	K04257
ENSG00000181126	5.13E-15	-2.63127	1	1	HLA-V	K06751
ENSG00000181171	5.13E-15	-2.63127	1	1	FER1L6-AS1	_
ENSG00000181195	5.13E-15	-2.63127	1	1	PENK	K18832
ENSG00000181201	5.13E-15	-2.63127	1	1	HIST3H2BA	K11252
ENSG00000181211	5.13E-15	-2.63127	1	1	HECW1-IT1	_
ENSG00000181214	5.13E-15	-2.63127	1	1	OR8G2P	K04257
ENSG00000181215	5.13E-15	-2.63127	1	1	C4orf50	_
ENSG00000181234	5.13E-15	-2.63127	1	1	TMEM132C	K17599
ENSG00000181273	5.13E-15	-2.63127	1	1	OR5AK2	K04257
ENSG00000181282	5.13E-15	-2.63127	1	1	OR5AK3P	K04257
ENSG00000181291	5.13E-15	-2.63127	1	1	TMEM132E	K17599
ENSG00000181296	5.13E-15	-2.63127	1	1	OR5G1P	K04257
ENSG00000181325	5.13E-15	-2.63127	1	1	OR9G3P	K04257

ENSG00000181359	5.13E-15	-2.63127	1	1	HSP90AA6P	K04079
ENSG00000181371	5.13E-15	-2.63127	1	1	OR5M8	K04257
ENSG00000181374	5.13E-15	-2.63127	1	1	CCL13	K16595
ENSG00000181378	5.13E-15	-2.63127	1	1	CCDC108	_
ENSG00000181395	5.13E-15	-2.63127	1	1	OR5AL1	K04257
ENSG00000181408	5.13E-15	-2.63127	1	1	UTS2R	K04241
ENSG00000181433	5.13E-15	-2.63127	1	1	SAGE1	K13180
ENSG00000181449	5.13E-15	-2.63127	1	1	SOX2	K16796
ENSG00000181464	5.13E-15	-2.63127	1	1	CDRT1	K10266
ENSG00000181495	5.13E-15	-2.63127	1	1	AC026703.1	_
ENSG00000181499	5.13E-15	-2.63127	1	1	OR6T1	K04257
ENSG00000181511	5.13E-15	-2.63127	1	1	RP4-655L22.4	K07390
ENSG00000181514	5.13E-15	-2.63127	1	1	RP3-474G15.1	_
ENSG00000181518	5.13E-15	-2.63127	1	1	OR8D4	K04257
ENSG00000181552	5.13E-15	-2.63127	1	1	EDDM3B	_
ENSG00000181562	5.13E-15	-2.63127	1	1	EDDM3A	_
ENSG00000181609	5.13E-15	-2.63127	1	1	OR52D1	K04257
ENSG00000181616	5.13E-15	-2.63127	1	1	OR52H1	K04257
ENSG00000181617	5.13E-15	-2.63127	1	1	FDCSP	_
ENSG00000181626	5.13E-15	-2.63127	1	1	ANKRD62	K04613
ENSG00000181631	5.13E-15	-2.63127	1	1	P2RY13	K08388
ENSG00000181656	5.13E-15	-2.63127	1	1	GPR88	K08422
ENSG00000181689	5.13E-15	-2.63127	1	1	OR8K3	K04257
ENSG00000181693	5.13E-15	-2.63127	1	1	OR8H1	K04257
ENSG00000181698	5.13E-15	-2.63127	1	1	OR5T1	K04257
ENSG00000181705	5.13E-15	-2.63127	1	1	RP11-11L12.2	_
ENSG00000181718	5.13E-15	-2.63127	1	1	OR5T2	K04257
ENSG00000181733	5.13E-15	-2.63127	1	1	OR2Z1	K04257
ENSG00000181752	5.13E-15	-2.63127	1	1	OR8K5	K04257
ENSG00000181761	5.13E-15	-2.63127	1	1	OR8H3	K04257
ENSG00000181767	5.13E-15	-2.63127	1	1	OR8H2	K04257
ENSG00000181778	5.13E-15	-2.63127	1	1	TMEM252	_
ENSG00000181780	5.13E-15	-2.63127	1	1	OR5J1P	K04257
ENSG00000181785	5.13E-15	-2.63127	1	1	OR5AS1	K04257
ENSG00000181786	5.13E-15	-2.63127	1	1	ACTL9	K12314
ENSG00000181791	5.13E-15	-2.63127	1	1	AC009041.1	_
ENSG00000181800	5.13E-15	-2.63127	1	1	CELF2-AS1	_
ENSG00000181803	5.13E-15	-2.63127	1	1	OR6S1	K04257
ENSG00000181804	5.13E-15	-2.63127	1	1	SLC9A9	K14725
ENSG00000181837	5.13E-15	-2.63127	1	1	OR5D17P	K04257
ENSG00000181847	5.13E-15	-2.63127	1	1	TIGIT	K16350
ENSG00000181867	5.13E-15	-2.63127	1	1	FTMT	K18495
ENSG00000181903	5.13E-15	-2.63127	1	1	OR4C6	K04257
ENSG00000181927	5.13E-15	-2.63127	1	1	OR4P4	K04257
ENSG00000181935	5.13E-15	-2.63127	1	1	OR4C16	K04257
ENSG00000181939	5.13E-15	-2.63127	1	1	OR4C15	K04257
ENSG00000181943	5.13E-15	-2.63127	1	1	OR4A21P	K04257
ENSG00000181950	5.13E-15	-2.63127	1	1	OR4A13P	K04257
ENSG00000181958	5.13E-15	-2.63127	1	1	OR4A15	K04257
ENSG00000181961	5.13E-15	-2.63127	1	1	OR4A16	K04257
ENSG00000181963	5.13E-15	-2.63127	1	1	OR52K2	K04257
ENSG00000181965	5.13E-15	-2.63127	1	1	NEUROG1	K09081
ENSG00000181977	5.13E-15	-2.63127	1	1	PYY3	K05233
ENSG00000181982	5.13E-15	-2.63127	1	1	CCDC149	K16627
ENSG00000181984	5.13E-15	-2.63127	1	1	GOLGA8CP	K10595
ENSG00000181995	5.13E-15	-2.63127	1	1	LINC00301	_
ENSG00000181997	5.13E-15	-2.63127	1	1	AQP7P2	K08771
ENSG00000182000	5.13E-15	-2.63127	1	1	RP11-651P23.2	K03767

ENSG00000182013	5.13E-15	-2.63127	1	1	PNMAL1	K12581
ENSG00000182021	5.13E-15	-2.63127	1	1	RP11-381O7.3	_
ENSG00000182035	5.13E-15	-2.63127	1	1	ADIG	_
ENSG00000182048	5.13E-15	-2.63127	1	1	TRPC2	K04965
ENSG00000182050	5.13E-15	-2.63127	1	1	MGAT4C	_
ENSG00000182053	5.13E-15	-2.63127	1	1	TRIM49B	K12019
ENSG00000182070	5.13E-15	-2.63127	1	1	OR52A1	K04257
ENSG00000182077	5.13E-15	-2.63127	1	1	PTCHD3	_
ENSG00000182083	5.13E-15	-2.63127	1	1	OR6B2	K04257
ENSG00000182103	5.13E-15	-2.63127	1	1	FAM181B	_
ENSG00000182107	5.13E-15	-2.63127	1	1	TMEM30B	_
ENSG00000182111	5.13E-15	-2.63127	1	1	ZNF716	K09228
ENSG00000182118	5.13E-15	-2.63127	1	1	FAM89A	_
ENSG00000182132	5.13E-15	-2.63127	1	1	KCNIP1	_
ENSG00000182156	5.13E-15	-2.63127	1	1	ENPP7	K12354
ENSG00000182165	5.13E-15	-2.63127	1	1	TP53TG1	_
ENSG00000182170	5.13E-15	-2.63127	1	1	MRGPRG	K08395
ENSG00000182175	5.13E-15	-2.63127	1	1	RGMA	K06847
ENSG00000182177	5.13E-15	-2.63127	1	1	ASB18	K10340
ENSG00000182183	5.13E-15	-2.63127	1	1	FAM159A	_
ENSG00000182187	5.13E-15	-2.63127	1	1	CRYGB	_
ENSG00000182223	5.13E-15	-2.63127	1	1	ZAR1	K18762
ENSG00000182242	5.13E-15	-2.63127	1	1	TEX28P1	_
ENSG00000182255	5.13E-15	-2.63127	1	1	KCNA4	K04877
ENSG00000182256	5.13E-15	-2.63127	1	1	GABRG3	K05186
ENSG00000182271	5.13E-15	-2.63127	1	1	TMIGD1	_
ENSG00000182315	5.13E-15	-2.63127	1	1	MBD3L3	_
ENSG00000182329	5.13E-15	-2.63127	1	1	AC079354.1	K17985
ENSG00000182330	5.13E-15	-2.63127	1	1	PRAMEF8	_
ENSG00000182334	5.13E-15	-2.63127	1	1	OR5P3	K04257
ENSG00000182346	5.13E-15	-2.63127	1	1	DAOA	_
ENSG00000182347	5.13E-15	-2.63127	1	1	RP11-39K24.9	K12504
ENSG00000182348	5.13E-15	-2.63127	1	1	ZNF804B	K00012
ENSG00000182351	5.13E-15	-2.63127	1	1	CRIP1P4	_
ENSG00000182352	5.13E-15	-2.63127	1	1	C17orf77	K09228
ENSG00000182365	5.13E-15	-2.63127	1	1	OR5F2P	K04257
ENSG00000182366	5.13E-15	-2.63127	1	1	FAM87A	_
ENSG00000182383	5.13E-15	-2.63127	1	1	RPL27AP5	K02900
ENSG00000182393	5.13E-15	-2.63127	1	1	IFNL1	K05447
ENSG00000182397	5.13E-15	-2.63127	1	1	DNM1P46	K01528
ENSG00000182415	5.13E-15	-2.63127	1	1	CDY2A	K00653
ENSG00000182447	5.13E-15	-2.63127	1	1	OTOL1	_
ENSG00000182477	5.13E-15	-2.63127	1	1	OR2B8P	K04257
ENSG00000182487	5.13E-15	-2.63127	1	1	NCF1B	K08011
ENSG00000182489	5.13E-15	-2.63127	1	1	XKRX	_
ENSG00000182492	5.13E-15	-2.63127	1	1	BGN	K08118
ENSG00000182502	5.13E-15	-2.63127	1	1	ABHD17AP5	_
ENSG00000182508	5.13E-15	-2.63127	1	1	LHFPL1	_
ENSG00000182521	5.13E-15	-2.63127	1	1	TBPL2	K03120
ENSG00000182531	5.13E-15	-2.63127	1	1	OR7E115P	K04257
ENSG00000182565	5.13E-15	-2.63127	1	1	OR4C2P	K04257
ENSG00000182566	5.13E-15	-2.63127	1	1	CLEC4G	K10061
ENSG00000182568	5.13E-15	-2.63127	1	1	SATB1	_
ENSG00000182572	5.13E-15	-2.63127	1	1	HIST1H3I	K11253
ENSG00000182574	5.13E-15	-2.63127	1	1	RP3-341D10.1	K00746
ENSG00000182575	5.13E-15	-2.63127	1	1	NXPH3	K16658
ENSG00000182583	5.13E-15	-2.63127	1	1	VCX	_
ENSG00000182586	5.13E-15	-2.63127	1	1	LINC00334	_

ENSG00000182591	5.13E-15	-2.63127	1	1	KRTAP11-1	_
ENSG00000182601	5.13E-15	-2.63127	1	1	HS3ST4	K09678
ENSG00000182613	5.13E-15	-2.63127	1	1	OR2V2	K04257
ENSG00000182631	5.13E-15	-2.63127	1	1	RXFP3	K08397
ENSG00000182632	5.13E-15	-2.63127	1	1	CCNYL2	K04237
ENSG00000182634	5.13E-15	-2.63127	1	1	OR10G7	K04257
ENSG00000182636	5.13E-15	-2.63127	1	1	NDN	K19482
ENSG00000182645	5.13E-15	-2.63127	1	1	CCDC172	_
ENSG00000182648	5.13E-15	-2.63127	1	1	LINC01006	_
ENSG00000182652	5.13E-15	-2.63127	1	1	OR4Q3	K04257
ENSG00000182667	5.13E-15	-2.63127	1	1	NTM	K06774
ENSG00000182674	5.13E-15	-2.63127	1	1	KCNB2	K04886
ENSG00000182698	5.13E-15	-2.63127	1	1	RESP18	_
ENSG00000182707	5.13E-15	-2.63127	1	1	FXYD6P3	K13363
ENSG00000182712	5.13E-15	-2.63127	1	1	CMC4	_
ENSG00000182722	5.13E-15	-2.63127	1	1	SEPHS1P1	K01008
ENSG00000182732	5.13E-15	-2.63127	1	1	RGS6	K16449
ENSG00000182747	5.13E-15	-2.63127	1	1	SLC35D3	K15281
ENSG00000182771	5.13E-15	-2.63127	1	1	GRID1	K05206
ENSG00000182776	5.13E-15	-2.63127	1	1	RP11-114H20.1	_
ENSG00000182783	5.13E-15	-2.63127	1	1	OR2T29	K04257
ENSG00000182793	5.13E-15	-2.63127	1	1	GSTA5	K00799
ENSG00000182798	5.13E-15	-2.63127	1	1	MAGEB17	_
ENSG00000182816	5.13E-15	-2.63127	1	1	KRTAP13-2	_
ENSG00000182836	5.13E-15	-2.63127	1	1	PLCXD3	_
ENSG00000182854	5.13E-15	-2.63127	1	1	OR4F15	K04257
ENSG00000182888	5.13E-15	-2.63127	1	1	RP11-558O12.1	K02977
ENSG00000182896	5.13E-15	-2.63127	1	1	TMEM95	_
ENSG00000182898	5.13E-15	-2.63127	1	1	TCHHL1	_
ENSG00000182903	5.13E-15	-2.63127	1	1	ZNF721	K09228
ENSG00000182912	5.13E-15	-2.63127	1	1	C21orf90	K12581
ENSG00000182916	5.13E-15	-2.63127	1	1	TCEAL7	_
ENSG00000182921	5.13E-15	-2.63127	1	1	CCDC75P1	_
ENSG00000182931	5.13E-15	-2.63127	1	1	WFDC10B	_
ENSG00000182938	5.13E-15	-2.63127	1	1	OTOP3	_
ENSG00000182965	5.13E-15	-2.63127	1	1	NPM1P14	K11276
ENSG00000182968	5.13E-15	-2.63127	1	1	SOX1	K09267
ENSG00000182974	5.13E-15	-2.63127	1	1	OR4M2	K04257
ENSG00000183019	5.13E-15	-2.63127	1	1	C19orf59	_
ENSG00000183022	5.13E-15	-2.63127	1	1	TPM3P8	K09290
ENSG00000183023	5.13E-15	-2.63127	1	1	SLC8A1	K05849
ENSG00000183024	5.13E-15	-2.63127	1	1	OR1G1	K04257
ENSG00000183034	5.13E-15	-2.63127	1	1	OTOP2	_
ENSG00000183035	5.13E-15	-2.63127	1	1	CYLC1	_
ENSG00000183036	5.13E-15	-2.63127	1	1	PCP4	_
ENSG00000183038	5.13E-15	-2.63127	1	1	GGTLC3	K18592
ENSG00000183067	5.13E-15	-2.63127	1	1	IGSF5	K06786
ENSG00000183086	5.13E-15	-2.63127	1	1	GATSL1	_
ENSG00000183090	5.13E-15	-2.63127	1	1	FREM3	_
ENSG00000183098	5.13E-15	-2.63127	1	1	GPC6	K08112
ENSG00000183103	5.13E-15	-2.63127	1	1	LYPD4	_
ENSG00000183114	5.13E-15	-2.63127	1	1	FAM43B	K15688
ENSG00000183122	5.13E-15	-2.63127	1	1	OR2A3P	K04257
ENSG00000183130	5.13E-15	-2.63127	1	1	OR2T11	K04257
ENSG00000183145	5.13E-15	-2.63127	1	1	RIPPLY3	K17985
ENSG00000183146	5.13E-15	-2.63127	1	1	CYorf17	_
ENSG00000183148	5.13E-15	-2.63127	1	1	ANKRD20A2	K17299
ENSG00000183154	5.13E-15	-2.63127	1	1	RP11-863K10.7	_

ENSG00000183160	5.13E-15	-2.63127	1	1	TMEM119	_
ENSG00000183166	5.13E-15	-2.63127	1	1	CALN1	_
ENSG00000183171	5.13E-15	-2.63127	1	1	RP11-49C9.2	_
ENSG00000183206	5.13E-15	-2.63127	1	1	POTEC	K17299
ENSG00000183239	5.13E-15	-2.63127	1	1	RP5-1120P11.4	K02905
ENSG00000183242	5.13E-15	-2.63127	1	1	WT1-AS	_
ENSG00000183246	5.13E-15	-2.63127	1	1	RIMBP3C	K04552
ENSG00000183249	5.13E-15	-2.63127	1	1	NF1P3	K08052
ENSG00000183251	5.13E-15	-2.63127	1	1	OR51B4	K04257
ENSG00000183269	5.13E-15	-2.63127	1	1	OR52E8	K04257
ENSG00000183273	5.13E-15	-2.63127	1	1	CCDC60	_
ENSG00000183287	5.13E-15	-2.63127	1	1	CCBE1	K19638
ENSG00000183292	5.13E-15	-2.63127	1	1	AC140481.2	K09628
ENSG00000183303	5.13E-15	-2.63127	1	1	OR5P2	K04257
ENSG00000183304	5.13E-15	-2.63127	1	1	FAM9A	K19528
ENSG00000183308	5.13E-15	-2.63127	1	1	AC005037.3	_
ENSG00000183310	5.13E-15	-2.63127	1	1	OR2T34	K04257
ENSG00000183313	5.13E-15	-2.63127	1	1	OR52L1	K04257
ENSG00000183317	5.13E-15	-2.63127	1	1	EPHA10	K08897
ENSG00000183318	5.13E-15	-2.63127	1	1	SPDYE4	K08694
ENSG00000183336	5.13E-15	-2.63127	1	1	BOLA2	K15078
ENSG00000183347	5.13E-15	-2.63127	1	1	GBP6	K15696
ENSG00000183378	5.13E-15	-2.63127	1	1	OVCH2	_
ENSG00000183385	5.13E-15	-2.63127	1	1	TTTY8	_
ENSG00000183389	5.13E-15	-2.63127	1	1	OR56A4	K04257
ENSG00000183432	5.13E-15	-2.63127	1	1	ZBTB8OSP1	_
ENSG00000183434	5.13E-15	-2.63127	1	1	TFDP3	K09393
ENSG00000183439	5.13E-15	-2.63127	1	1	TRIM61	K12029
ENSG00000183454	5.13E-15	-2.63127	1	1	GRIN2A	K05209
ENSG00000183461	5.13E-15	-2.63127	1	1	XAGE1C	_
ENSG00000183463	5.13E-15	-2.63127	1	1	URAD	K13485
ENSG00000183470	5.13E-15	-2.63127	1	1	AC009365.3	_
ENSG00000183473	5.13E-15	-2.63127	1	1	SSTR3	K04219
ENSG00000183476	5.13E-15	-2.63127	1	1	SH2D7	_
ENSG00000183479	5.13E-15	-2.63127	1	1	TREX2	K16590
ENSG00000183486	5.13E-15	-2.63127	1	1	MX2	K17985
ENSG00000183504	5.13E-15	-2.63127	1	1	AL132868.1	_
ENSG00000183514	5.13E-15	-2.63127	1	1	TDGF1P2	_
ENSG00000183531	5.13E-15	-2.63127	1	1	Z98256.1	_
ENSG00000183549	5.13E-15	-2.63127	1	1	ACSM5	K01896
ENSG00000183558	5.13E-15	-2.63127	1	1	HIST2H2AA3	K11251
ENSG00000183559	5.13E-15	-2.63127	1	1	C10orf120	_
ENSG00000183560	5.13E-15	-2.63127	1	1	FOLR4	K13649
ENSG00000183566	5.13E-15	-2.63127	1	1	BPIFA4P	_
ENSG00000183578	5.13E-15	-2.63127	1	1	TNFAIP8L3	K17985
ENSG00000183586	5.13E-15	-2.63127	1	1	HMGN3P1	K11301
ENSG00000183598	5.13E-15	-2.63127	1	1	HIST2H3D	K11253
ENSG00000183629	5.13E-15	-2.63127	1	1	GOLGA8G	K10595
ENSG00000183631	5.13E-15	-2.63127	1	1	CXorf64	_
ENSG00000183632	5.13E-15	-2.63127	1	1	TP53TG3	_
ENSG00000183640	5.13E-15	-2.63127	1	1	KRTAP8-1	_
ENSG00000183643	5.13E-15	-2.63127	1	1	C15orf32	_
ENSG00000183644	5.13E-15	-2.63127	1	1	C11orf88	K17985
ENSG00000183653	5.13E-15	-2.63127	1	1	GRIK1-AS2	K05201
ENSG00000183654	5.13E-15	-2.63127	1	1	11-Mar	K10659
ENSG00000183662	5.13E-15	-2.63127	1	1	FAM19A1	_
ENSG00000183663	5.13E-15	-2.63127	1	1	RP11-44N17.1	K00134
ENSG00000183675	5.13E-15	-2.63127	1	1	PTPN20B	K18039

ENSG00000183688	5.13E-15	-2.63127	1	1	FAM101B	K02927
ENSG00000183695	5.13E-15	-2.63127	1	1	MRGPRX2	K08396
ENSG00000183704	5.13E-15	-2.63127	1	1	SLC9B1P1	_
ENSG00000183706	5.13E-15	-2.63127	1	1	OR4N4	K04257
ENSG00000183709	5.13E-15	-2.63127	1	1	IFNL2	K05447
ENSG00000183715	5.13E-15	-2.63127	1	1	OPCML	K06773
ENSG00000183729	5.13E-15	-2.63127	1	1	NPBWR1	K05268
ENSG00000183733	5.13E-15	-2.63127	1	1	FIGLA	K09066
ENSG00000183734	5.13E-15	-2.63127	1	1	ASCL2	K09067
ENSG00000183742	5.13E-15	-2.63127	1	1	MACC1	_
ENSG00000183747	5.13E-15	-2.63127	1	1	ACSM2A	K01896
ENSG00000183748	5.13E-15	-2.63127	1	1	MRC1L1	K06560
ENSG00000183753	5.13E-15	-2.63127	1	1	BPY2	_
ENSG00000183760	5.13E-15	-2.63127	1	1	PAPL	K12606
ENSG00000183778	5.13E-15	-2.63127	1	1	B3GALT5	_
ENSG00000183783	5.13E-15	-2.63127	1	1	KCTD8	_
ENSG00000183784	5.13E-15	-2.63127	1	1	C9orf66	K06115
ENSG00000183791	5.13E-15	-2.63127	1	1	TCEB3C	K15076
ENSG00000183795	5.13E-15	-2.63127	1	1	BPY2B	_
ENSG00000183807	5.13E-15	-2.63127	1	1	FAM162B	_
ENSG00000183822	5.13E-15	-2.63127	1	1	CTA-833B7.2	_
ENSG00000183833	5.13E-15	-2.63127	1	1	MAATS1	_
ENSG00000183837	5.13E-15	-2.63127	1	1	PNMA3	_
ENSG00000183850	5.13E-15	-2.63127	1	1	ZNF730	K09228
ENSG00000183862	5.13E-15	-2.63127	1	1	CNGA2	K04949
ENSG00000183873	5.13E-15	-2.63127	1	1	SCN5A	K04838
ENSG00000183878	5.13E-15	-2.63127	1	1	UTY	K11447
ENSG00000183888	5.13E-15	-2.63127	1	1	C1orf64	K17985
ENSG00000183900	5.13E-15	-2.63127	1	1	AC099522.1	K09397
ENSG00000183908	5.13E-15	-2.63127	1	1	LRRC55	K04174
ENSG00000183909	5.13E-15	-2.63127	1	1	OR4G2P	K04257
ENSG00000183911	5.13E-15	-2.63127	1	1	RPL21P132	K02889
ENSG00000183914	5.13E-15	-2.63127	1	1	DNAH2	K10408
ENSG00000183918	5.13E-15	-2.63127	1	1	SH2D1A	K07990
ENSG00000183921	5.13E-15	-2.63127	1	1	SDR42E2	_
ENSG00000183938	5.13E-15	-2.63127	1	1	TRBV21OR9-2	_
ENSG00000183941	5.13E-15	-2.63127	1	1	HIST2H4A	K11254
ENSG00000183960	5.13E-15	-2.63127	1	1	KCNH8	K04911
ENSG00000183981	5.13E-15	-2.63127	1	1	MAGEA13P	_
ENSG00000184005	5.13E-15	-2.63127	1	1	ST6GALNAC3	K03373
ENSG00000184012	5.13E-15	-2.63127	1	1	TMPRSS2	K09633
ENSG00000184022	5.13E-15	-2.63127	1	1	OR2T10	K04257
ENSG00000184029	5.13E-15	-2.63127	1	1	DSCR4	_
ENSG00000184032	5.13E-15	-2.63127	1	1	KRTAP20-2	_
ENSG00000184055	5.13E-15	-2.63127	1	1	OR7E87P	K04257
ENSG00000184106	5.13E-15	-2.63127	1	1	TREML3P	_
ENSG00000184108	5.13E-15	-2.63127	1	1	TRIML1	K12038
ENSG00000184115	5.13E-15	-2.63127	1	1	RP13-1039J1.2	_
ENSG00000184139	5.13E-15	-2.63127	1	1	RPL7AP28	K02936
ENSG00000184140	5.13E-15	-2.63127	1	1	OR4F6	K04257
ENSG00000184144	5.13E-15	-2.63127	1	1	CNTN2	K06760
ENSG00000184148	5.13E-15	-2.63127	1	1	SPRR4	_
ENSG00000184155	5.13E-15	-2.63127	1	1	OR10J5	K04257
ENSG00000184156	5.13E-15	-2.63127	1	1	KCNQ3	K04928
ENSG00000184160	5.13E-15	-2.63127	1	1	ADRA2C	K04140
ENSG00000184166	5.13E-15	-2.63127	1	1	OR1D2	K04257
ENSG00000184188	5.13E-15	-2.63127	1	1	CTC-512J14.7	K05692
ENSG00000184194	5.13E-15	-2.63127	1	1	GPR173	K04300

ENSG00000184205	5.13E-15	-2.63127	1	1	TSPYL2	K11285
ENSG00000184210	5.13E-15	-2.63127	1	1	DGAT2L6	K11172
ENSG00000184221	5.13E-15	-2.63127	1	1	OLIG1	K09085
ENSG00000184247	5.13E-15	-2.63127	1	1	AC100791.1	_
ENSG00000184258	5.13E-15	-2.63127	1	1	CDR1	_
ENSG00000184260	5.13E-15	-2.63127	1	1	HIST2H2AC	K11251
ENSG00000184270	5.13E-15	-2.63127	1	1	HIST2H2AB	K11251
ENSG00000184274	5.13E-15	-2.63127	1	1	LINC00315	_
ENSG00000184276	5.13E-15	-2.63127	1	1	DEFB108B	_
ENSG00000184292	5.13E-15	-2.63127	1	1	TACSTD2	K17288
ENSG00000184293	5.13E-15	-2.63127	1	1	CLECL1	K17518
ENSG00000184302	5.13E-15	-2.63127	1	1	SIX6	K19473
ENSG00000184303	5.13E-15	-2.63127	1	1	DRD5P1	K05840
ENSG00000184304	5.13E-15	-2.63127	1	1	PRKD1	K06070
ENSG00000184305	5.13E-15	-2.63127	1	1	CCSER1	_
ENSG00000184313	5.13E-15	-2.63127	1	1	MROH7	_
ENSG00000184321	5.13E-15	-2.63127	1	1	OR51J1	K04257
ENSG00000184330	5.13E-15	-2.63127	1	1	S100A7A	_
ENSG00000184344	5.13E-15	-2.63127	1	1	GDF3	K05495
ENSG00000184345	5.13E-15	-2.63127	1	1	IQCF2	_
ENSG00000184350	5.13E-15	-2.63127	1	1	MRGPRE	K08393
ENSG00000184351	5.13E-15	-2.63127	1	1	KRTAP19-1	_
ENSG00000184374	5.13E-15	-2.63127	1	1	COLEC10	K10065
ENSG00000184385	5.13E-15	-2.63127	1	1	C21orf128	_
ENSG00000184388	5.13E-15	-2.63127	1	1	PABPC1L2B	K13126
ENSG00000184389	5.13E-15	-2.63127	1	1	A3GALT2	_
ENSG00000184394	5.13E-15	-2.63127	1	1	OR4N5	K04257
ENSG00000184408	5.13E-15	-2.63127	1	1	KCND2	K04892
ENSG00000184423	5.13E-15	-2.63127	1	1	AC090043.1	K02893
ENSG00000184454	5.13E-15	-2.63127	1	1	NCMAP	K11447
ENSG00000184459	5.13E-15	-2.63127	1	1	BPIFC	_
ENSG00000184478	5.13E-15	-2.63127	1	1	OR56A3	K04257
ENSG00000184515	5.13E-15	-2.63127	1	1	BEX5	_
ENSG00000184523	5.13E-15	-2.63127	1	1	PTGER4P2	K04261
ENSG00000184560	5.13E-15	-2.63127	1	1	C17orf74	_
ENSG00000184566	5.13E-15	-2.63127	1	1	AC132216.1	K15612
ENSG00000184586	5.13E-15	-2.63127	1	1	KRTAP7-1	_
ENSG00000184596	5.13E-15	-2.63127	1	1	AF207550.1	_
ENSG00000184601	5.13E-15	-2.63127	1	1	C14orf180	_
ENSG00000184608	5.13E-15	-2.63127	1	1	C8orf12	_
ENSG00000184611	5.13E-15	-2.63127	1	1	KCNH7	K04910
ENSG00000184612	5.13E-15	-2.63127	1	1	RPL7P26	K02937
ENSG00000184613	5.13E-15	-2.63127	1	1	NELL2	_
ENSG00000184616	5.13E-15	-2.63127	1	1	AC004166.6	K08694
ENSG00000184617	5.13E-15	-2.63127	1	1	ZNF840	K09228
ENSG00000184624	5.13E-15	-2.63127	1	1	ZNF72P	K09228
ENSG00000184647	5.13E-15	-2.63127	1	1	PRSS55	K11447
ENSG00000184650	5.13E-15	-2.63127	1	1	ODF4	_
ENSG00000184659	5.13E-15	-2.63127	1	1	FOXD4L4	K09397
ENSG00000184669	5.13E-15	-2.63127	1	1	OR7E14P	K04257
ENSG00000184672	5.13E-15	-2.63127	1	1	RALYL	K12895
ENSG00000184682	5.13E-15	-2.63127	1	1	C11orf89	_
ENSG00000184698	5.13E-15	-2.63127	1	1	OR51M1	K04257
ENSG00000184716	5.13E-15	-2.63127	1	1	SERINC4	_
ENSG00000184724	5.13E-15	-2.63127	1	1	KRTAP6-1	_
ENSG00000184741	5.13E-15	-2.63127	1	1	OR5AQ1P	K04257
ENSG00000184761	5.13E-15	-2.63127	1	1	AC013269.5	K09628
ENSG00000184774	5.13E-15	-2.63127	1	1	RP11-480I12.4	K01623

ENSG00000184789	5.13E-15	-2.63127	1	1	OR4C10P	K04257
ENSG00000184795	5.13E-15	-2.63127	1	1	UNC93B5	K04257
ENSG00000184809	5.13E-15	-2.63127	1	1	C21orf88	_
ENSG00000184811	5.13E-15	-2.63127	1	1	TUSC5	_
ENSG00000184814	5.13E-15	-2.63127	1	1	PRR23B	_
ENSG00000184844	5.13E-15	-2.63127	1	1	CYCSP45	K08738
ENSG00000184845	5.13E-15	-2.63127	1	1	DRD1	K04144
ENSG00000184856	5.13E-15	-2.63127	1	1	LINC00308	_
ENSG00000184860	5.13E-15	-2.63127	1	1	SDR42E1	_
ENSG00000184867	5.13E-15	-2.63127	1	1	ARMCX2	_
ENSG00000184879	5.13E-15	-2.63127	1	1	RP11-7G23.8	_
ENSG00000184881	5.13E-15	-2.63127	1	1	OR51B2	K04257
ENSG00000184895	5.13E-15	-2.63127	1	1	SRY	K09266
ENSG00000184905	5.13E-15	-2.63127	1	1	TCEAL2	_
ENSG00000184906	5.13E-15	-2.63127	1	1	AC129778.2	K13402
ENSG00000184909	5.13E-15	-2.63127	1	1	C1ORF220	K09228
ENSG00000184911	5.13E-15	-2.63127	1	1	DMRTC1B	K19494
ENSG00000184933	5.13E-15	-2.63127	1	1	OR6A2	K04257
ENSG00000184937	5.13E-15	-2.63127	1	1	WT1	K09234
ENSG00000184945	5.13E-15	-2.63127	1	1	AQP12A	K09871
ENSG00000184954	5.13E-15	-2.63127	1	1	OR6C70	K04257
ENSG00000184961	5.13E-15	-2.63127	1	1	AL772307.1	K02936
ENSG00000184986	5.13E-15	-2.63127	1	1	TMEM121	_
ENSG00000184991	5.13E-15	-2.63127	1	1	TTTY13	_
ENSG00000184999	5.13E-15	-2.63127	1	1	SLC22A10	K08206
ENSG00000185002	5.13E-15	-2.63127	1	1	RFX6	K19521
ENSG00000185008	5.13E-15	-2.63127	1	1	ROBO2	K06754
ENSG00000185010	5.13E-15	-2.63127	1	1	F8	K03899
ENSG00000185020	5.13E-15	-2.63127	1	1	RP11-111G23.1	_
ENSG00000185028	5.13E-15	-2.63127	1	1	LRRC14B	_
ENSG00000185031	5.13E-15	-2.63127	1	1	SLC2A3P2	K08142
ENSG00000185037	5.13E-15	-2.63127	1	1	ZNF733P	K09228
ENSG00000185040	5.13E-15	-2.63127	1	1	AC004980.11	K08694
ENSG00000185041	5.13E-15	-2.63127	1	1	RP11-321E2.6	K03135
ENSG00000185052	5.13E-15	-2.63127	1	1	SLC24A3	K13751
ENSG00000185053	5.13E-15	-2.63127	1	1	SGCZ	_
ENSG00000185056	5.13E-15	-2.63127	1	1	C5orf47	_
ENSG00000185069	5.13E-15	-2.63127	1	1	KRT76	K07605
ENSG00000185074	5.13E-15	-2.63127	1	1	OR7E31P	K04257
ENSG00000185078	5.13E-15	-2.63127	1	1	AC093698.1	K02940
ENSG00000185087	5.13E-15	-2.63127	1	1	FAM169B	_
ENSG00000185097	5.13E-15	-2.63127	1	1	OR4F16	K04257
ENSG00000185149	5.13E-15	-2.63127	1	1	NPY2R	K04205
ENSG00000185155	5.13E-15	-2.63127	1	1	MIXL1	_
ENSG00000185156	5.13E-15	-2.63127	1	1	MFSD6L	_
ENSG00000185176	5.13E-15	-2.63127	1	1	AQP12B	K09871
ENSG00000185177	5.13E-15	-2.63127	1	1	ZNF479	K09228
ENSG00000185182	5.13E-15	-2.63127	1	1	GOLGA8DP	K10595
ENSG00000185203	5.13E-15	-2.63127	1	1	WASIR1	_
ENSG00000185221	5.13E-15	-2.63127	1	1	GAPDHP24	K00134
ENSG00000185231	5.13E-15	-2.63127	1	1	MC2R	K04200
ENSG00000185254	5.13E-15	-2.63127	1	1	TEX28	_
ENSG00000185264	5.13E-15	-2.63127	1	1	TEX33	_
ENSG00000185271	5.13E-15	-2.63127	1	1	KLHL33	K13957
ENSG00000185272	5.13E-15	-2.63127	1	1	RBM11	_
ENSG00000185274	5.13E-15	-2.63127	1	1	WBSCR17	K00710
ENSG00000185290	5.13E-15	-2.63127	1	1	NUPR1L	_
ENSG00000185294	5.13E-15	-2.63127	1	1	SPPL2C	K14212

ENSG00000185303	5.13E-15	-2.63127	1	1	SFTPA2	K10067
ENSG00000185313	5.13E-15	-2.63127	1	1	SCN10A	K04842
ENSG00000185319	5.13E-15	-2.63127	1	1	SSXP4	K15625
ENSG00000185345	5.13E-15	-2.63127	1	1	PARK2	K04556
ENSG00000185352	5.13E-15	-2.63127	1	1	HS6ST3	K08103
ENSG00000185372	5.13E-15	-2.63127	1	1	OR2V1	K04257
ENSG00000185385	5.13E-15	-2.63127	1	1	OR7A17	K04257
ENSG00000185390	5.13E-15	-2.63127	1	1	FGF7P2	K04358
ENSG00000185436	5.13E-15	-2.63127	1	1	IFNLR1	K05140
ENSG00000185448	5.13E-15	-2.63127	1	1	FAM47A	_
ENSG00000185477	5.13E-15	-2.63127	1	1	GPRIN3	_
ENSG00000185479	5.13E-15	-2.63127	1	1	KRT6B	K07605
ENSG00000185515	5.13E-15	-2.63127	1	1	BRCC3	K11864
ENSG00000185518	5.13E-15	-2.63127	1	1	SV2B	K06258
ENSG00000185554	5.13E-15	-2.63127	1	1	NXF2	K14284
ENSG00000185594	5.13E-15	-2.63127	1	1	SPATA8	_
ENSG00000185607	5.13E-15	-2.63127	1	1	ACTBP7	K05692
ENSG00000185610	5.13E-15	-2.63127	1	1	DBX2	K09304
ENSG00000185631	5.13E-15	-2.63127	1	1	AC017079.3	K02875
ENSG00000185638	5.13E-15	-2.63127	1	1	FTH1P14	K00522
ENSG00000185640	5.13E-15	-2.63127	1	1	KRT79	K07605
ENSG00000185652	5.13E-15	-2.63127	1	1	NTF3	_
ENSG00000185662	5.13E-15	-2.63127	1	1	SMIM23	_
ENSG00000185668	5.13E-15	-2.63127	1	1	POU3F1	K09365
ENSG00000185674	5.13E-15	-2.63127	1	1	LYG2	_
ENSG00000185681	5.13E-15	-2.63127	1	1	MORN5	_
ENSG00000185700	5.13E-15	-2.63127	1	1	TTTY8B	_
ENSG00000185701	5.13E-15	-2.63127	1	1	OR5M5P	K04257
ENSG00000185737	5.13E-15	-2.63127	1	1	NRG3	K05457
ENSG00000185742	5.13E-15	-2.63127	1	1	C11orf87	_
ENSG00000185751	5.13E-15	-2.63127	1	1	XAGE2	_
ENSG00000185758	5.13E-15	-2.63127	1	1	CLDN24	K06087
ENSG00000185760	5.13E-15	-2.63127	1	1	KCNQ5	K04930
ENSG00000185811	5.13E-15	-2.63127	1	1	IKZF1	K09220
ENSG00000185821	5.13E-15	-2.63127	1	1	OR6C76	K04257
ENSG00000185823	5.13E-15	-2.63127	1	1	NPAP1	K01368
ENSG00000185847	5.13E-15	-2.63127	1	1	RP1-46F2.2	_
ENSG00000185863	5.13E-15	-2.63127	1	1	TMEM210	_
ENSG00000185869	5.13E-15	-2.63127	1	1	ZNF829	K09228
ENSG00000185873	5.13E-15	-2.63127	1	1	TMPRSS11B	K09751
ENSG00000185888	5.13E-15	-2.63127	1	1	PRSS38	_
ENSG00000185894	5.13E-15	-2.63127	1	1	BPY2C	_
ENSG00000185897	5.13E-15	-2.63127	1	1	FFAR3	K04326
ENSG00000185903	5.13E-15	-2.63127	1	1	OR11N1P	K04257
ENSG00000185904	5.13E-15	-2.63127	1	1	LINC00839	_
ENSG00000185905	5.13E-15	-2.63127	1	1	C16orf54	_
ENSG00000185915	5.13E-15	-2.63127	1	1	KLHL34	K10469
ENSG00000185926	5.13E-15	-2.63127	1	1	OR4C46	K04257
ENSG00000185940	5.13E-15	-2.63127	1	1	KRTAP5-5	_
ENSG00000185942	5.13E-15	-2.63127	1	1	NKAIN3	_
ENSG00000185945	5.13E-15	-2.63127	1	1	NXF2B	K14284
ENSG00000185960	5.13E-15	-2.63127	1	1	SHOX	K09331
ENSG00000185962	5.13E-15	-2.63127	1	1	LCE3A	_
ENSG00000185966	5.13E-15	-2.63127	1	1	LCE3E	_
ENSG00000185972	5.13E-15	-2.63127	1	1	CCIN	_
ENSG00000185974	5.13E-15	-2.63127	1	1	GRK1	K00909
ENSG00000185978	5.13E-15	-2.63127	1	1	H2AFB3	K11251
ENSG00000185982	5.13E-15	-2.63127	1	1	DEFB128	_

ENSG00000185985	5.13E-15	-2.63127	1	1	SLITRK2	_
ENSG00000186008	5.13E-15	-2.63127	1	1	RPS4XP21	K02987
ENSG00000186009	5.13E-15	-2.63127	1	1	ATP4B	K01543
ENSG00000186047	5.13E-15	-2.63127	1	1	DLEU7	_
ENSG00000186049	5.13E-15	-2.63127	1	1	KRT73	K07605
ENSG00000186051	5.13E-15	-2.63127	1	1	TAL2	K09068
ENSG00000186074	5.13E-15	-2.63127	1	1	CD300LF	_
ENSG00000186075	5.13E-15	-2.63127	1	1	ZPBP2	_
ENSG00000186081	5.13E-15	-2.63127	1	1	KRT5	K07605
ENSG00000186082	5.13E-15	-2.63127	1	1	KRT18P14	K07604
ENSG00000186088	5.13E-15	-2.63127	1	1	GSAP	_
ENSG00000186090	5.13E-15	-2.63127	1	1	HTR3D	K04819
ENSG00000186092	5.13E-15	-2.63127	1	1	OR4F5	K04257
ENSG00000186094	5.13E-15	-2.63127	1	1	AGBL4	_
ENSG00000186103	5.13E-15	-2.63127	1	1	ARGFX	_
ENSG00000186104	5.13E-15	-2.63127	1	1	CYP2R1	K07419
ENSG00000186113	5.13E-15	-2.63127	1	1	OR5D14	K04257
ENSG00000186115	5.13E-15	-2.63127	1	1	CYP4F2	K17726
ENSG00000186117	5.13E-15	-2.63127	1	1	OR5L1	K04257
ENSG00000186118	5.13E-15	-2.63127	1	1	TEX38	_
ENSG00000186119	5.13E-15	-2.63127	1	1	OR5D18	K04257
ENSG00000186124	5.13E-15	-2.63127	1	1	OR9M1P	K04257
ENSG00000186136	5.13E-15	-2.63127	1	1	TAS2R42	K08474
ENSG00000186143	5.13E-15	-2.63127	1	1	C2orf53	_
ENSG00000186146	5.13E-15	-2.63127	1	1	DEFB131	K10803
ENSG00000186148	5.13E-15	-2.63127	1	1	AC013271.3	_
ENSG00000186152	5.13E-15	-2.63127	1	1	LILRP1	K06512
ENSG00000186163	5.13E-15	-2.63127	1	1	U66059.29	_
ENSG00000186188	5.13E-15	-2.63127	1	1	FFAR4	K08425
ENSG00000186190	5.13E-15	-2.63127	1	1	BPIFB3	_
ENSG00000186191	5.13E-15	-2.63127	1	1	BPIFB4	_
ENSG00000186212	5.13E-15	-2.63127	1	1	SOWAHB	_
ENSG00000186223	5.13E-15	-2.63127	1	1	SSU72P4	K15544
ENSG00000186226	5.13E-15	-2.63127	1	1	LCE1E	_
ENSG00000186231	5.13E-15	-2.63127	1	1	KLHL32	K10468
ENSG00000186232	5.13E-15	-2.63127	1	1	SSU72P3	K15544
ENSG00000186234	5.13E-15	-2.63127	1	1	FAM86MP	K12354
ENSG00000186235	5.13E-15	-2.63127	1	1	AC016757.3	_
ENSG00000186244	5.13E-15	-2.63127	1	1	RP11-1079K10.1	K02889
ENSG00000186265	5.13E-15	-2.63127	1	1	BTLA	K06707
ENSG00000186268	5.13E-15	-2.63127	1	1	OR10D4P	K04257
ENSG00000186280	5.13E-15	-2.63127	1	1	KDM4D	K06709
ENSG00000186288	5.13E-15	-2.63127	1	1	PABPC1L2A	K13126
ENSG00000186297	5.13E-15	-2.63127	1	1	GABRA5	K05175
ENSG00000186306	5.13E-15	-2.63127	1	1	OR10T2	K04257
ENSG00000186310	5.13E-15	-2.63127	1	1	NAP1L3	K11281
ENSG00000186322	5.13E-15	-2.63127	1	1	GOLGA6L17P	K10595
ENSG00000186328	5.13E-15	-2.63127	1	1	RP11-140K17.2	_
ENSG00000186329	5.13E-15	-2.63127	1	1	TMEM212	_
ENSG00000186334	5.13E-15	-2.63127	1	1	SLC36A3	K14209
ENSG00000186335	5.13E-15	-2.63127	1	1	SLC36A2	K14209
ENSG00000186367	5.13E-15	-2.63127	1	1	KIAA1024L	K15696
ENSG00000186369	5.13E-15	-2.63127	1	1	LINC00643	_
ENSG00000186399	5.13E-15	-2.63127	1	1	GOLGA8R	K10595
ENSG00000186400	5.13E-15	-2.63127	1	1	OR10X1	K04257
ENSG00000186407	5.13E-15	-2.63127	1	1	CD300E	K06719
ENSG00000186431	5.13E-15	-2.63127	1	1	FCAR	K06513
ENSG00000186439	5.13E-15	-2.63127	1	1	TRDN	_

ENSG00000186440	5.13E-15	-2.63127	1	1	OR6P1	K04257
ENSG00000186446	5.13E-15	-2.63127	1	1	ZNF501	K09228
ENSG00000186452	5.13E-15	-2.63127	1	1	TMPRSS12	_
ENSG00000186453	5.13E-15	-2.63127	1	1	FAM228A	_
ENSG00000186458	5.13E-15	-2.63127	1	1	DEFB132	_
ENSG00000186462	5.13E-15	-2.63127	1	1	NAP1L2	K11280
ENSG00000186469	5.13E-15	-2.63127	1	1	GNG2	_
ENSG00000186472	5.13E-15	-2.63127	1	1	PCLO	K16882
ENSG00000186474	5.13E-15	-2.63127	1	1	KLK12	K09621
ENSG00000186479	5.13E-15	-2.63127	1	1	RGS7BP	_
ENSG00000186481	5.13E-15	-2.63127	1	1	ANKRD20A5P	K17299
ENSG00000186493	5.13E-15	-2.63127	1	1	C5orf38	_
ENSG00000186496	5.13E-15	-2.63127	1	1	ZNF396	K09230
ENSG00000186508	5.13E-15	-2.63127	1	1	OR9I2P	K04257
ENSG00000186509	5.13E-15	-2.63127	1	1	OR9Q1	K04257
ENSG00000186513	5.13E-15	-2.63127	1	1	OR9Q2	K04257
ENSG00000186526	5.13E-15	-2.63127	1	1	CYP4F8	_
ENSG00000186530	5.13E-15	-2.63127	1	1	XKR5	_
ENSG00000186543	5.13E-15	-2.63127	1	1	CROCCP5	K16469
ENSG00000186562	5.13E-15	-2.63127	1	1	DEFB105A	_
ENSG00000186572	5.13E-15	-2.63127	1	1	DEFB107A	_
ENSG00000186579	5.13E-15	-2.63127	1	1	DEFB106A	_
ENSG00000186583	5.13E-15	-2.63127	1	1	SPATC1	_
ENSG00000186599	5.13E-15	-2.63127	1	1	DEFB105B	_
ENSG00000186675	5.13E-15	-2.63127	1	1	MAGEE2	_
ENSG00000186678	5.13E-15	-2.63127	1	1	RP11-266I3.7	_
ENSG00000186723	5.13E-15	-2.63127	1	1	OR10H1	K04257
ENSG00000186732	5.13E-15	-2.63127	1	1	MPPED1	_
ENSG00000186743	5.13E-15	-2.63127	1	1	TPI1P3	K01803
ENSG00000186766	5.13E-15	-2.63127	1	1	FOXI2	K09401
ENSG00000186777	5.13E-15	-2.63127	1	1	ZNF732	K09228
ENSG00000186788	5.13E-15	-2.63127	1	1	SPATA31D3	_
ENSG00000186795	5.13E-15	-2.63127	1	1	KCNK18	_
ENSG00000186803	5.13E-15	-2.63127	1	1	IFNA10	K05414
ENSG00000186810	5.13E-15	-2.63127	1	1	CXCR3	K04188
ENSG00000186818	5.13E-15	-2.63127	1	1	LILRB4	K06512
ENSG00000186825	5.13E-15	-2.63127	1	1	C2orf27B	_
ENSG00000186827	5.13E-15	-2.63127	1	1	TNFRSF4	K05142
ENSG00000186838	5.13E-15	-2.63127	1	1	SELV	_
ENSG00000186844	5.13E-15	-2.63127	1	1	LCE1A	_
ENSG00000186860	5.13E-15	-2.63127	1	1	KRTAP17-1	_
ENSG00000186867	5.13E-15	-2.63127	1	1	QRFPR	K08378
ENSG00000186881	5.13E-15	-2.63127	1	1	OR13F1	K04257
ENSG00000186886	5.13E-15	-2.63127	1	1	OR5D3P	K04257
ENSG00000186889	5.13E-15	-2.63127	1	1	TMEM17	K19384
ENSG00000186895	5.13E-15	-2.63127	1	1	FGF3	K04358
ENSG00000186910	5.13E-15	-2.63127	1	1	SERPINA11	K04525
ENSG00000186912	5.13E-15	-2.63127	1	1	P2RY4	K04271
ENSG00000186924	5.13E-15	-2.63127	1	1	KRTAP22-1	_
ENSG00000186925	5.13E-15	-2.63127	1	1	KRTAP19-6	_
ENSG00000186930	5.13E-15	-2.63127	1	1	KRTAP6-2	_
ENSG00000186943	5.13E-15	-2.63127	1	1	OR13C8	K04257
ENSG00000186960	5.13E-15	-2.63127	1	1	C14orf23	_
ENSG00000186965	5.13E-15	-2.63127	1	1	KRTAP19-2	_
ENSG00000186967	5.13E-15	-2.63127	1	1	KRTAP19-4	_
ENSG00000186970	5.13E-15	-2.63127	1	1	KRTAP15-1	_
ENSG00000186971	5.13E-15	-2.63127	1	1	KRTAP13-4	_
ENSG00000186973	5.13E-15	-2.63127	1	1	FAM183A	_

ENSG00000186980	5.13E-15	-2.63127	1	1	KRTAP23-1	_
ENSG00000187003	5.13E-15	-2.63127	1	1	ACTL7A	_
ENSG00000187005	5.13E-15	-2.63127	1	1	KRTAP21-1	_
ENSG00000187012	5.13E-15	-2.63127	1	1	LINC00207	_
ENSG00000187013	5.13E-15	-2.63127	1	1	C17orf82	_
ENSG00000187021	5.13E-15	-2.63127	1	1	PNLIPRP1	K14074
ENSG00000187026	5.13E-15	-2.63127	1	1	KRTAP21-2	_
ENSG00000187033	5.13E-15	-2.63127	1	1	SAMD7	_
ENSG00000187037	5.13E-15	-2.63127	1	1	GPR141	K08429
ENSG00000187048	5.13E-15	-2.63127	1	1	CYP4A11	K17687
ENSG00000187054	5.13E-15	-2.63127	1	1	TMPRSS11A	K09750
ENSG00000187060	5.13E-15	-2.63127	1	1	RP11-187C18.3	K02936
ENSG00000187068	5.13E-15	-2.63127	1	1	C3orf70	K17985
ENSG00000187080	5.13E-15	-2.63127	1	1	OR2AK2	K04257
ENSG00000187082	5.13E-15	-2.63127	1	1	DEFB106B	_
ENSG00000187094	5.13E-15	-2.63127	1	1	CCK	K05226
ENSG00000187103	5.13E-15	-2.63127	1	1	FUNDC2P3	_
ENSG00000187116	5.13E-15	-2.63127	1	1	LILRA5	K06512
ENSG00000187135	5.13E-15	-2.63127	1	1	VSTM2B	_
ENSG00000187170	5.13E-15	-2.63127	1	1	LCE4A	_
ENSG00000187172	5.13E-15	-2.63127	1	1	BAGE2	K09188
ENSG00000187173	5.13E-15	-2.63127	1	1	LCE2A	_
ENSG00000187175	5.13E-15	-2.63127	1	1	KRTAP12-1	_
ENSG00000187180	5.13E-15	-2.63127	1	1	LCE2C	_
ENSG00000187185	5.13E-15	-2.63127	1	1	CTD-2600O9.1	_
ENSG00000187191	5.13E-15	-2.63127	1	1	DAZ3	_
ENSG00000187210	5.13E-15	-2.63127	1	1	GCNT1	K00727
ENSG00000187223	5.13E-15	-2.63127	1	1	LCE2D	_
ENSG00000187238	5.13E-15	-2.63127	1	1	LCE3B	_
ENSG00000187242	5.13E-15	-2.63127	1	1	KRT12	K07604
ENSG00000187243	5.13E-15	-2.63127	1	1	MAGED4B	K12464
ENSG00000187258	5.13E-15	-2.63127	1	1	NPSR1	K08376
ENSG00000187272	5.13E-15	-2.63127	1	1	KRTAP9-8	_
ENSG00000187323	5.13E-15	-2.63127	1	1	DCC	_
ENSG00000187325	5.13E-15	-2.63127	1	1	TAF9B	K03133
ENSG00000187372	5.13E-15	-2.63127	1	1	PCDHB13	K16494
ENSG00000187391	5.13E-15	-2.63127	1	1	MAGI2	K05629
ENSG00000187398	5.13E-15	-2.63127	1	1	LUZP2	_
ENSG00000187461	5.13E-15	-2.63127	1	1	AL583828.1	_
ENSG00000187472	5.13E-15	-2.63127	1	1	RP11-67P15.1	K02934
ENSG00000187475	5.13E-15	-2.63127	1	1	HIST1H1T	K11275
ENSG00000187481	5.13E-15	-2.63127	1	1	HSD3BP1	K00070
ENSG00000187483	5.13E-15	-2.63127	1	1	SERPINA13P	K04525
ENSG00000187504	5.13E-15	-2.63127	1	1	RPL7P48	K02937
ENSG00000187510	5.13E-15	-2.63127	1	1	PLEKHG7	_
ENSG00000187516	5.13E-15	-2.63127	1	1	CXorf27	_
ENSG00000187527	5.13E-15	-2.63127	1	1	ATP13A5	K14951
ENSG00000187533	5.13E-15	-2.63127	1	1	C4orf40	K05725
ENSG00000187536	5.13E-15	-2.63127	1	1	TPM3P7	K09290
ENSG00000187537	5.13E-15	-2.63127	1	1	POTEM	K17299
ENSG00000187545	5.13E-15	-2.63127	1	1	PRAMEF10	_
ENSG00000187546	5.13E-15	-2.63127	1	1	AGMO	K15537
ENSG00000187553	5.13E-15	-2.63127	1	1	CYP26C1	K12665
ENSG00000187559	5.13E-15	-2.63127	1	1	FOXD4L3	K09397
ENSG00000187566	5.13E-15	-2.63127	1	1	NHLRC1	K10602
ENSG00000187569	5.13E-15	-2.63127	1	1	DPPA3	_
ENSG00000187581	5.13E-15	-2.63127	1	1	COX8C	K02273
ENSG00000187589	5.13E-15	-2.63127	1	1	TERF1P2	K11110

ENSG00000187595	5.13E-15	-2.63127	1	1	ZNF385C	_
ENSG00000187600	5.13E-15	-2.63127	1	1	TMEM247	_
ENSG00000187612	5.13E-15	-2.63127	1	1	OR5W2	K04257
ENSG00000187624	5.13E-15	-2.63127	1	1	C17orf97	_
ENSG00000187657	5.13E-15	-2.63127	1	1	TSPY13P	K11284
ENSG00000187658	5.13E-15	-2.63127	1	1	C5orf52	_
ENSG00000187664	5.13E-15	-2.63127	1	1	HAPLN4	K06853
ENSG00000187672	5.13E-15	-2.63127	1	1	ERC2	K16072
ENSG00000187682	5.13E-15	-2.63127	1	1	ERAS	K17196
ENSG00000187690	5.13E-15	-2.63127	1	1	CXorf67	_
ENSG00000187701	5.13E-15	-2.63127	1	1	OR2T27	K04257
ENSG00000187714	5.13E-15	-2.63127	1	1	SLC18A3	K14636
ENSG00000187715	5.13E-15	-2.63127	1	1	KBTBD12	_
ENSG00000187721	5.13E-15	-2.63127	1	1	GTF2IP3	K03121
ENSG00000187730	5.13E-15	-2.63127	1	1	GABRD	K05184
ENSG00000187733	5.13E-15	-2.63127	1	1	AMY1C	K01176
ENSG00000187747	5.13E-15	-2.63127	1	1	OR52B6	K04257
ENSG00000187754	5.13E-15	-2.63127	1	1	SSX7	K15625
ENSG00000187758	5.13E-15	-2.63127	1	1	ADH1A	K13951
ENSG00000187766	5.13E-15	-2.63127	1	1	KRTAP10-8	_
ENSG00000187772	5.13E-15	-2.63127	1	1	LIN28B	K18754
ENSG00000187773	5.13E-15	-2.63127	1	1	FAM69C	_
ENSG00000187783	5.13E-15	-2.63127	1	1	TMEM72	_
ENSG00000187786	5.13E-15	-2.63127	1	1	SCXB	K09070
ENSG00000187806	5.13E-15	-2.63127	1	1	TMEM202	_
ENSG00000187811	5.13E-15	-2.63127	1	1	AP000867.1	K04257
ENSG00000187812	5.13E-15	-2.63127	1	1	RP11-24M17.5	_
ENSG00000187815	5.13E-15	-2.63127	1	1	ZFP69	K09228
ENSG00000187821	5.13E-15	-2.63127	1	1	HELT	_
ENSG00000187823	5.13E-15	-2.63127	1	1	ZCCHC16	_
ENSG00000187833	5.13E-15	-2.63127	1	1	C2orf78	_
ENSG00000187847	5.13E-15	-2.63127	1	1	OR7E25P	K04257
ENSG00000187855	5.13E-15	-2.63127	1	1	ASCL4	K09067
ENSG00000187857	5.13E-15	-2.63127	1	1	OR6C75	K04257
ENSG00000187870	5.13E-15	-2.63127	1	1	RNFT1P3	_
ENSG00000187871	5.13E-15	-2.63127	1	1	GFRAL	K17985
ENSG00000187889	5.13E-15	-2.63127	1	1	C1orf168	_
ENSG00000187893	5.13E-15	-2.63127	1	1	CXXC1P1	_
ENSG00000187900	5.13E-15	-2.63127	1	1	OR5H7P	K04257
ENSG00000187904	5.13E-15	-2.63127	1	1	AC097382.5	_
ENSG00000187918	5.13E-15	-2.63127	1	1	OR51I2	K04257
ENSG00000187922	5.13E-15	-2.63127	1	1	LCN10	_
ENSG00000187950	5.13E-15	-2.63127	1	1	OVCH1	_
ENSG00000187959	5.13E-15	-2.63127	1	1	CPSF4L	K14404
ENSG00000187969	5.13E-15	-2.63127	1	1	ZCCHC13	K09250
ENSG00000187979	5.13E-15	-2.63127	1	1	AC008079.9	_
ENSG00000187980	5.13E-15	-2.63127	1	1	PLA2G2C	K01047
ENSG00000187988	5.13E-15	-2.63127	1	1	RP11-405L18.2	_
ENSG00000187997	5.13E-15	-2.63127	1	1	C17orf99	_
ENSG00000187999	5.13E-15	-2.63127	1	1	HNRNPA1P61	K12741
ENSG00000188000	5.13E-15	-2.63127	1	1	OR7D2	K04257
ENSG00000188011	5.13E-15	-2.63127	1	1	CXXC11	_
ENSG00000188013	5.13E-15	-2.63127	1	1	MEIS3P2	K16671
ENSG00000188029	5.13E-15	-2.63127	1	1	CTSL3P	K01365
ENSG00000188038	5.13E-15	-2.63127	1	1	NRN1L	_
ENSG00000188039	5.13E-15	-2.63127	1	1	NWD1	_
ENSG00000188050	5.13E-15	-2.63127	1	1	RNF133	K15702
ENSG00000188056	5.13E-15	-2.63127	1	1	TREML4	_

ENSG00000188069	5.13E-15	-2.63127	1	1	OR51F1	K04257
ENSG00000188076	5.13E-15	-2.63127	1	1	SCGB1C1	_
ENSG00000188078	5.13E-15	-2.63127	1	1	RP5-1119A7.11	_
ENSG00000188086	5.13E-15	-2.63127	1	1	PRSS45	_
ENSG00000188101	5.13E-15	-2.63127	1	1	ALOX15P2	K00460
ENSG00000188120	5.13E-15	-2.63127	1	1	DAZ1	_
ENSG00000188155	5.13E-15	-2.63127	1	1	KRTAP10-6	_
ENSG00000188171	5.13E-15	-2.63127	1	1	ZNF626	K09228
ENSG00000188219	5.13E-15	-2.63127	1	1	POTEE	K17299
ENSG00000188223	5.13E-15	-2.63127	1	1	AC002398.9	K06170
ENSG00000188227	5.13E-15	-2.63127	1	1	ZNF793	K09228
ENSG00000188269	5.13E-15	-2.63127	1	1	OR7A5	_
ENSG00000188279	5.13E-15	-2.63127	1	1	FAM25C	_
ENSG00000188280	5.13E-15	-2.63127	1	1	FAM230A	_
ENSG00000188282	5.13E-15	-2.63127	1	1	RUFY4	K05050
ENSG00000188295	5.13E-15	-2.63127	1	1	ZNF669	K09228
ENSG00000188306	5.13E-15	-2.63127	1	1	LRRIQ4	_
ENSG00000188314	5.13E-15	-2.63127	1	1	OR7D1P	K04257
ENSG00000188324	5.13E-15	-2.63127	1	1	OR6C6	K04257
ENSG00000188334	5.13E-15	-2.63127	1	1	BSPH1	_
ENSG00000188340	5.13E-15	-2.63127	1	1	OR6N2	K04257
ENSG00000188373	5.13E-15	-2.63127	1	1	C10orf99	_
ENSG00000188379	5.13E-15	-2.63127	1	1	IFNA2	K05414
ENSG00000188386	5.13E-15	-2.63127	1	1	PPP3R2	K06268
ENSG00000188388	5.13E-15	-2.63127	1	1	GOLGA6L3	K10595
ENSG00000188389	5.13E-15	-2.63127	1	1	PDCD1	K06744
ENSG00000188393	5.13E-15	-2.63127	1	1	CLEC2A	_
ENSG00000188394	5.13E-15	-2.63127	1	1	GPR21	K04318
ENSG00000188399	5.13E-15	-2.63127	1	1	ANKRD36P1	K17299
ENSG00000188403	5.13E-15	-2.63127	1	1	IGHV1OR15-9	_
ENSG00000188404	5.13E-15	-2.63127	1	1	SELL	K06495
ENSG00000188408	5.13E-15	-2.63127	1	1	MAGEB5	_
ENSG00000188425	5.13E-15	-2.63127	1	1	NANOS2	K18760
ENSG00000188438	5.13E-15	-2.63127	1	1	RP11-1396O13.14	_
ENSG00000188439	5.13E-15	-2.63127	1	1	OR4P1P	K04257
ENSG00000188452	5.13E-15	-2.63127	1	1	CERKL	K19602
ENSG00000188459	5.13E-15	-2.63127	1	1	WASF4P	K05748
ENSG00000188460	5.13E-15	-2.63127	1	1	ACTBP11	K05692
ENSG00000188467	5.13E-15	-2.63127	1	1	SLC24A5	K13753
ENSG00000188474	5.13E-15	-2.63127	1	1	ZNF788	K09228
ENSG00000188477	5.13E-15	-2.63127	1	1	AC003003.5	_
ENSG00000188488	5.13E-15	-2.63127	1	1	SERPINA5	K03913
ENSG00000188508	5.13E-15	-2.63127	1	1	KRTDAP	_
ENSG00000188512	5.13E-15	-2.63127	1	1	RP1-273G13.2	K08738
ENSG00000188523	5.13E-15	-2.63127	1	1	C9orf171	_
ENSG00000188525	5.13E-15	-2.63127	1	1	AC010969.1	_
ENSG00000188558	5.13E-15	-2.63127	1	1	OR2G6	K04257
ENSG00000188580	5.13E-15	-2.63127	1	1	NKAIN2	_
ENSG00000188581	5.13E-15	-2.63127	1	1	KRTAP1-1	_
ENSG00000188582	5.13E-15	-2.63127	1	1	PAQR9	_
ENSG00000188585	5.13E-15	-2.63127	1	1	LINC00083	_
ENSG00000188620	5.13E-15	-2.63127	1	1	HMX3	K09349
ENSG00000188629	5.13E-15	-2.63127	1	1	ZNF177	K09228
ENSG00000188646	5.13E-15	-2.63127	1	1	RP11-361A23.2	K10435
ENSG00000188649	5.13E-15	-2.63127	1	1	CC2D2B	K19352
ENSG00000188655	5.13E-15	-2.63127	1	1	RNASE9	_
ENSG00000188656	5.13E-15	-2.63127	1	1	TSPY7P	K11284
ENSG00000188668	5.13E-15	-2.63127	1	1	OR7E90P	K04257

ENSG00000188674	5.13E-15	-2.63127	1	1	C2orf80	_
ENSG00000188691	5.13E-15	-2.63127	1	1	RP11-451K18.7	K04257
ENSG00000188694	5.13E-15	-2.63127	1	1	KRTAP24-1	_
ENSG00000188712	5.13E-15	-2.63127	1	1	OR13D3P	K04257
ENSG00000188716	5.13E-15	-2.63127	1	1	DUPD1	K14165
ENSG00000188722	5.13E-15	-2.63127	1	1	AL391005.1	_
ENSG00000188729	5.13E-15	-2.63127	1	1	OSTN	_
ENSG00000188730	5.13E-15	-2.63127	1	1	VWC2	K11447
ENSG00000188738	5.13E-15	-2.63127	1	1	FSIP2	_
ENSG00000188755	5.13E-15	-2.63127	1	1	TBC1D3P2	K11837
ENSG00000188765	5.13E-15	-2.63127	1	1	TMSB4XP2	K05764
ENSG00000188782	5.13E-15	-2.63127	1	1	CATSPER4	K16892
ENSG00000188784	5.13E-15	-2.63127	1	1	PLA2G2E	K01047
ENSG00000188800	5.13E-15	-2.63127	1	1	TMCO2	_
ENSG00000188803	5.13E-15	-2.63127	1	1	SHISA6	_
ENSG00000188816	5.13E-15	-2.63127	1	1	HMX2	K09349
ENSG00000188817	5.13E-15	-2.63127	1	1	SNTN	K11447
ENSG00000188820	5.13E-15	-2.63127	1	1	FAM26F	_
ENSG00000188822	5.13E-15	-2.63127	1	1	CNR2	K04278
ENSG00000188828	5.13E-15	-2.63127	1	1	GLRA4	K05271
ENSG00000188831	5.13E-15	-2.63127	1	1	DPPA3P2	_
ENSG00000188848	5.13E-15	-2.63127	1	1	BEND4	_
ENSG00000188868	5.13E-15	-2.63127	1	1	ZNF563	K09228
ENSG00000188873	5.13E-15	-2.63127	1	1	RPL10AP2	K02865
ENSG00000188877	5.13E-15	-2.63127	1	1	POTEA	K17299
ENSG00000188883	5.13E-15	-2.63127	1	1	KLRG2	_
ENSG00000188888	5.13E-15	-2.63127	1	1	GPR179	_
ENSG00000188906	5.13E-15	-2.63127	1	1	LRRK2	K08844
ENSG00000188909	5.13E-15	-2.63127	1	1	BSX	K09317
ENSG00000188933	5.13E-15	-2.63127	1	1	USP32P1	K11837
ENSG00000188984	5.13E-15	-2.63127	1	1	AADACL3	K14351
ENSG00000188991	5.13E-15	-2.63127	1	1	SLC15A5	K14639
ENSG00000188993	5.13E-15	-2.63127	1	1	LRRC66	_
ENSG00000189002	5.13E-15	-2.63127	1	1	PROSP	K03908
ENSG00000189011	5.13E-15	-2.63127	1	1	AC138783.12	K10858
ENSG00000189013	5.13E-15	-2.63127	1	1	KIR2DL4	K07981
ENSG00000189014	5.13E-15	-2.63127	1	1	FAM35DP	K07208
ENSG00000189023	5.13E-15	-2.63127	1	1	MAGEB16	_
ENSG00000189030	5.13E-15	-2.63127	1	1	VHLL	K03871
ENSG00000189037	5.13E-15	-2.63127	1	1	DUSP21	K14165
ENSG00000189045	5.13E-15	-2.63127	1	1	ANKDD1B	_
ENSG00000189052	5.13E-15	-2.63127	1	1	CGB5	K10045
ENSG00000189056	5.13E-15	-2.63127	1	1	RELN	K06249
ENSG00000189064	5.13E-15	-2.63127	1	1	GAGE2A	_
ENSG00000189068	5.13E-15	-2.63127	1	1	VSTM1	_
ENSG00000189089	5.13E-15	-2.63127	1	1	RIMKLBP1	K18310
ENSG00000189090	5.13E-15	-2.63127	1	1	FAM25B	_
ENSG00000189108	5.13E-15	-2.63127	1	1	IL1RAPL2	K05170
ENSG00000189127	5.13E-15	-2.63127	1	1	ANKRD34B	_
ENSG00000189132	5.13E-15	-2.63127	1	1	FAM47B	_
ENSG00000189134	5.13E-15	-2.63127	1	1	NKAPL	_
ENSG00000189139	5.13E-15	-2.63127	1	1	FSCB	_
ENSG00000189145	5.13E-15	-2.63127	1	1	RP11-77G22.2	K02912
ENSG00000189152	5.13E-15	-2.63127	1	1	GRAPL	K04364
ENSG00000189166	5.13E-15	-2.63127	1	1	TNRC18P3	_
ENSG00000189169	5.13E-15	-2.63127	1	1	KRTAP10-12	_
ENSG00000189181	5.13E-15	-2.63127	1	1	OR14I1	K04257
ENSG00000189182	5.13E-15	-2.63127	1	1	KRT77	K07605

ENSG00000189184	5.13E-15	-2.63127	1	1	PCDH18	K16499
ENSG00000189186	5.13E-15	-2.63127	1	1	DCAF8L2	K11804
ENSG00000189195	5.13E-15	-2.63127	1	1	BTBD8	K10480
ENSG00000189196	5.13E-15	-2.63127	1	1	LINC00994	_
ENSG00000189238	5.13E-15	-2.63127	1	1	LINC00943	_
ENSG00000189252	5.13E-15	-2.63127	1	1	SPANXN3	_
ENSG00000189253	5.13E-15	-2.63127	1	1	TRIM64B	K12019
ENSG00000189283	5.13E-15	-2.63127	1	1	FHIT	K01522
ENSG00000189295	5.13E-15	-2.63127	1	1	ANKRD62P1-PARP	_
ENSG00000189299	5.13E-15	-2.63127	1	1	FOXR2	_
ENSG00000189316	5.13E-15	-2.63127	1	1	RP11-797H7.5	_
ENSG00000189325	5.13E-15	-2.63127	1	1	C6orf222	_
ENSG00000189326	5.13E-15	-2.63127	1	1	SPANXN4	_
ENSG00000189332	5.13E-15	-2.63127	1	1	RP11-113D6.10	_
ENSG00000189348	5.13E-15	-2.63127	1	1	FAM90A27P	_
ENSG00000189357	5.13E-15	-2.63127	1	1	SPATA31D4	_
ENSG00000189367	5.13E-15	-2.63127	1	1	KIAA0408	_
ENSG00000189369	5.13E-15	-2.63127	1	1	GSPT2	K03267
ENSG00000189372	5.13E-15	-2.63127	1	1	RP6-149D17.1	K14075
ENSG00000189393	5.13E-15	-2.63127	1	1	FAM90A14P	_
ENSG00000189398	5.13E-15	-2.63127	1	1	OR7E12P	K04257
ENSG00000189401	5.13E-15	-2.63127	1	1	OTUD6A	K18342
ENSG00000189419	5.13E-15	-2.63127	1	1	SPATA41	_
ENSG00000189420	5.13E-15	-2.63127	1	1	ZFP92	K09228
ENSG00000189430	5.13E-15	-2.63127	1	1	NCR1	K06741
ENSG00000194270	5.13E-15	-2.63127	1	1	Y_RNA	_
ENSG00000194297	5.13E-15	-2.63127	1	1	RNU1-75P	_
ENSG00000194717	5.13E-15	-2.63127	1	1	MIR494	_
ENSG00000195024	5.13E-15	-2.63127	1	1	RNU1-15P	_
ENSG00000195401	5.13E-15	-2.63127	1	1	Y_RNA	_
ENSG00000196071	5.13E-15	-2.63127	1	1	OR2L13	K04257
ENSG00000196081	5.13E-15	-2.63127	1	1	ZNF724P	K09228
ENSG00000196082	5.13E-15	-2.63127	1	1	AC140061.1	K04257
ENSG00000196085	5.13E-15	-2.63127	1	1	AC013442.1	_
ENSG00000196090	5.13E-15	-2.63127	1	1	PTPRT	K13297
ENSG00000196091	5.13E-15	-2.63127	1	1	MYBPC1	K12557
ENSG00000196092	5.13E-15	-2.63127	1	1	PAX5	K09383
ENSG00000196095	5.13E-15	-2.63127	1	1	AC126182.1	_
ENSG00000196096	5.13E-15	-2.63127	1	1	AC079610.2	_
ENSG00000196098	5.13E-15	-2.63127	1	1	OR5K4	K04257
ENSG00000196099	5.13E-15	-2.63127	1	1	OR6M1	K04257
ENSG00000196104	5.13E-15	-2.63127	1	1	SPOCK3	K08136
ENSG00000196109	5.13E-15	-2.63127	1	1	ZNF676	K09228
ENSG00000196114	5.13E-15	-2.63127	1	1	RP3-391O22.3	K06566
ENSG00000196115	5.13E-15	-2.63127	1	1	ADAM5	_
ENSG00000196119	5.13E-15	-2.63127	1	1	OR8A1	K04257
ENSG00000196126	5.13E-15	-2.63127	1	1	HLA-DRB1	K06752
ENSG00000196131	5.13E-15	-2.63127	1	1	VN1R2	K04614
ENSG00000196136	5.13E-15	-2.63127	1	1	SERPINA3	K04525
ENSG00000196138	5.13E-15	-2.63127	1	1	AC005831.1	_
ENSG00000196139	5.13E-15	-2.63127	1	1	AKR1C3	K04119
ENSG00000196143	5.13E-15	-2.63127	1	1	OR11H13P	K04257
ENSG00000196156	5.13E-15	-2.63127	1	1	KRTAP4-3	_
ENSG00000196166	5.13E-15	-2.63127	1	1	C8orf86	K17985
ENSG00000196167	5.13E-15	-2.63127	1	1	COLCA1	_
ENSG00000196169	5.13E-15	-2.63127	1	1	KIF19	K10401
ENSG00000196171	5.13E-15	-2.63127	1	1	OR6K2	K04257
ENSG00000196172	5.13E-15	-2.63127	1	1	ZNF681	K09228

ENSG00000196184	5.13E-15	-2.63127	1	1	OR10J1	K04257
ENSG00000196188	5.13E-15	-2.63127	1	1	CTSE	K01382
ENSG00000196209	5.13E-15	-2.63127	1	1	SIRPB2	K16826
ENSG00000196224	5.13E-15	-2.63127	1	1	KRTAP5-3	_
ENSG00000196226	5.13E-15	-2.63127	1	1	HIST1H2BB	K11252
ENSG00000196228	5.13E-15	-2.63127	1	1	SULT1C3	K01025
ENSG00000196230	5.13E-15	-2.63127	1	1	TUBB	K07375
ENSG00000196240	5.13E-15	-2.63127	1	1	OR2T2	K04257
ENSG00000196242	5.13E-15	-2.63127	1	1	OR2C3	K04257
ENSG00000196243	5.13E-15	-2.63127	1	1	LINC00615	_
ENSG00000196248	5.13E-15	-2.63127	1	1	OR10S1	K04257
ENSG00000196260	5.13E-15	-2.63127	1	1	SFTA2	_
ENSG00000196263	5.13E-15	-2.63127	1	1	ZNF471	K09228
ENSG00000196273	5.13E-15	-2.63127	1	1	LINC00523	_
ENSG00000196274	5.13E-15	-2.63127	1	1	AC090519.1	K04613
ENSG00000196277	5.13E-15	-2.63127	1	1	GRM7	K04608
ENSG00000196301	5.13E-15	-2.63127	1	1	HLA-DRB9	K06752
ENSG00000196326	5.13E-15	-2.63127	1	1	AKR1CL1	K04119
ENSG00000196329	5.13E-15	-2.63127	1	1	GIMAP5	K08504
ENSG00000196341	5.13E-15	-2.63127	1	1	OR8D1	K04257
ENSG00000196344	5.13E-15	-2.63127	1	1	ADH7	K13951
ENSG00000196345	5.13E-15	-2.63127	1	1	ZKSCAN7	K09229
ENSG00000196350	5.13E-15	-2.63127	1	1	ZNF729	K09228
ENSG00000196353	5.13E-15	-2.63127	1	1	CPNE4	_
ENSG00000196355	5.13E-15	-2.63127	1	1	AC021860.1	K14404
ENSG00000196364	5.13E-15	-2.63127	1	1	PRSS29P	_
ENSG00000196368	5.13E-15	-2.63127	1	1	NUDT11	K07766
ENSG00000196376	5.13E-15	-2.63127	1	1	SLC35F1	K15287
ENSG00000196381	5.13E-15	-2.63127	1	1	ZNF781	K09228
ENSG00000196383	5.13E-15	-2.63127	1	1	OR4Q2	K04257
ENSG00000196390	5.13E-15	-2.63127	1	1	CTD-2503O16.3	K09228
ENSG00000196395	5.13E-15	-2.63127	1	1	RP13-77O11.11	_
ENSG00000196400	5.13E-15	-2.63127	1	1	BX255923.1	K13194
ENSG00000196403	5.13E-15	-2.63127	1	1	OR10D1P	K04257
ENSG00000196406	5.13E-15	-2.63127	1	1	SPANXD	_
ENSG00000196431	5.13E-15	-2.63127	1	1	CRYBA4	_
ENSG00000196433	5.13E-15	-2.63127	1	1	ASMT	K00543
ENSG00000196440	5.13E-15	-2.63127	1	1	ARMCX4	K03767
ENSG00000196460	5.13E-15	-2.63127	1	1	RFX8	_
ENSG00000196468	5.13E-15	-2.63127	1	1	FGF16	K04358
ENSG00000196472	5.13E-15	-2.63127	1	1	RP13-644M16.4	K00699
ENSG00000196475	5.13E-15	-2.63127	1	1	GK2	K00864
ENSG00000196476	5.13E-15	-2.63127	1	1	C20orf96	_
ENSG00000196482	5.13E-15	-2.63127	1	1	ESRRG	K08554
ENSG00000196533	5.13E-15	-2.63127	1	1	C1orf186	K02872
ENSG00000196534	5.13E-15	-2.63127	1	1	OR9K1P	K04257
ENSG00000196539	5.13E-15	-2.63127	1	1	OR2T3	K04257
ENSG00000196542	5.13E-15	-2.63127	1	1	SPTSSB	_
ENSG00000196553	5.13E-15	-2.63127	1	1	LINC00238	_
ENSG00000196564	5.13E-15	-2.63127	1	1	RP3-499B10.3	K12581
ENSG00000196565	5.13E-15	-2.63127	1	1	HBG2	K13824
ENSG00000196578	5.13E-15	-2.63127	1	1	OR5AC2	K04257
ENSG00000196581	5.13E-15	-2.63127	1	1	AJAP1	_
ENSG00000196589	5.13E-15	-2.63127	1	1	CTB-25J19.1	_
ENSG00000196600	5.13E-15	-2.63127	1	1	SLC22A25	K08206
ENSG00000196616	5.13E-15	-2.63127	1	1	ADH1B	K13951
ENSG00000196620	5.13E-15	-2.63127	1	1	UGT2B15	K00699
ENSG00000196628	5.13E-15	-2.63127	1	1	TCF4	K15603

ENSG00000196632	5.13E-15	-2.63127	1	1	WNK3	K08867
ENSG00000196653	5.13E-15	-2.63127	1	1	ZNF502	K09228
ENSG00000196655	5.13E-15	-2.63127	1	1	TRAPPC4	K08171
ENSG00000196660	5.13E-15	-2.63127	1	1	SLC30A10	K14697
ENSG00000196661	5.13E-15	-2.63127	1	1	OR8B3	K04257
ENSG00000196664	5.13E-15	-2.63127	1	1	TLR7	_
ENSG00000196711	5.13E-15	-2.63127	1	1	FAM150A	_
ENSG00000196724	5.13E-15	-2.63127	1	1	ZNF418	K09228
ENSG00000196734	5.13E-15	-2.63127	1	1	LCE1B	_
ENSG00000196735	5.13E-15	-2.63127	1	1	HLA-DQA1	K06752
ENSG00000196737	5.13E-15	-2.63127	1	1	AC087392.1	K12581
ENSG00000196748	5.13E-15	-2.63127	1	1	CLPSL2	_
ENSG00000196758	5.13E-15	-2.63127	1	1	AC079612.1	_
ENSG00000196767	5.13E-15	-2.63127	1	1	POU3F4	K09365
ENSG00000196772	5.13E-15	-2.63127	1	1	OR14A16	K04257
ENSG00000196774	5.13E-15	-2.63127	1	1	ANKRD20A1	K17299
ENSG00000196777	5.13E-15	-2.63127	1	1	AL162574.1	_
ENSG00000196778	5.13E-15	-2.63127	1	1	OR52K1	K04257
ENSG00000196796	5.13E-15	-2.63127	1	1	CTB-134H23.2	_
ENSG00000196800	5.13E-15	-2.63127	1	1	SPINK14	_
ENSG00000196805	5.13E-15	-2.63127	1	1	SPRR2B	_
ENSG00000196826	5.13E-15	-2.63127	1	1	ZNF709	K09228
ENSG00000196832	5.13E-15	-2.63127	1	1	OR11G2	K04257
ENSG00000196834	5.13E-15	-2.63127	1	1	POTEI	K17299
ENSG00000196844	5.13E-15	-2.63127	1	1	PATE2	_
ENSG00000196859	5.13E-15	-2.63127	1	1	KRT39	K07604
ENSG00000196861	5.13E-15	-2.63127	1	1	AC002994.1	K03626
ENSG00000196893	5.13E-15	-2.63127	1	1	AC090286.4	_
ENSG00000196932	5.13E-15	-2.63127	1	1	TMEM26	_
ENSG00000196933	5.13E-15	-2.63127	1	1	RPS26P11	K02976
ENSG00000196936	5.13E-15	-2.63127	1	1	OR2L8	K04257
ENSG00000196944	5.13E-15	-2.63127	1	1	OR2T4	K04257
ENSG00000196946	5.13E-15	-2.63127	1	1	ZNF705A	K09228
ENSG00000196951	5.13E-15	-2.63127	1	1	RP11-425I13.3	_
ENSG00000196967	5.13E-15	-2.63127	1	1	ZNF585A	K09228
ENSG00000196970	5.13E-15	-2.63127	1	1	NXF4	K14284
ENSG00000196972	5.13E-15	-2.63127	1	1	LINC00087	_
ENSG00000196979	5.13E-15	-2.63127	1	1	AL360004.1	K04613
ENSG00000196987	5.13E-15	-2.63127	1	1	LCA10	K03539
ENSG00000196990	5.13E-15	-2.63127	1	1	FAM163B	_
ENSG00000197013	5.13E-15	-2.63127	1	1	ZNF429	K09228
ENSG00000197015	5.13E-15	-2.63127	1	1	AL353898.1	K12581
ENSG00000197016	5.13E-15	-2.63127	1	1	ZNF470	K09228
ENSG00000197020	5.13E-15	-2.63127	1	1	ZNF100	K09228
ENSG00000197023	5.13E-15	-2.63127	1	1	OR51A6P	K04257
ENSG00000197038	5.13E-15	-2.63127	1	1	RBMY1A3P	K12885
ENSG00000197046	5.13E-15	-2.63127	1	1	SIGLEC15	K16352
ENSG00000197057	5.13E-15	-2.63127	1	1	DTHD1	_
ENSG00000197067	5.13E-15	-2.63127	1	1	OR2T32P	K04257
ENSG00000197071	5.13E-15	-2.63127	1	1	AC010243.1	_
ENSG00000197079	5.13E-15	-2.63127	1	1	KRT35	K07604
ENSG00000197083	5.13E-15	-2.63127	1	1	ZNF300P1	K09228
ENSG00000197084	5.13E-15	-2.63127	1	1	LCE1C	_
ENSG00000197085	5.13E-15	-2.63127	1	1	NPSR1-AS1	_
ENSG00000197092	5.13E-15	-2.63127	1	1	GOLGA6L16P	K10595
ENSG00000197099	5.13E-15	-2.63127	1	1	RP11-573D15.8	_
ENSG00000197106	5.13E-15	-2.63127	1	1	SLC6A17	K05048
ENSG00000197110	5.13E-15	-2.63127	1	1	IFNL3	K05447

ENSG00000197123	5.13E-15	-2.63127	1	1	ZNF679	K09228
ENSG00000197124	5.13E-15	-2.63127	1	1	ZNF682	K09228
ENSG00000197125	5.13E-15	-2.63127	1	1	OR8B8	K04257
ENSG00000197134	5.13E-15	-2.63127	1	1	ZNF257	K10087
ENSG00000197161	5.13E-15	-2.63127	1	1	OR4C4P	K04257
ENSG00000197171	5.13E-15	-2.63127	1	1	OR2B4P	K04257
ENSG00000197176	5.13E-15	-2.63127	1	1	RP11-76E12.1	_
ENSG00000197177	5.13E-15	-2.63127	1	1	GPR123	K08460
ENSG00000197185	5.13E-15	-2.63127	1	1	SSXP1	K15624
ENSG00000197213	5.13E-15	-2.63127	1	1	ZSCAN5B	K09230
ENSG00000197214	5.13E-15	-2.63127	1	1	TCEA1P1	K03145
ENSG00000197233	5.13E-15	-2.63127	1	1	OR1J2	K04257
ENSG00000197241	5.13E-15	-2.63127	1	1	SLC2A7	K12201
ENSG00000197251	5.13E-15	-2.63127	1	1	LINC00336	_
ENSG00000197253	5.13E-15	-2.63127	1	1	TPSB2	K01340
ENSG00000197254	5.13E-15	-2.63127	1	1	AP000445.1	K00628
ENSG00000197261	5.13E-15	-2.63127	1	1	C6orf141	K11447
ENSG00000197262	5.13E-15	-2.63127	1	1	CCL4L2	K12964
ENSG00000197263	5.13E-15	-2.63127	1	1	OR8D2	K04257
ENSG00000197272	5.13E-15	-2.63127	1	1	IL27	_
ENSG00000197273	5.13E-15	-2.63127	1	1	GUCA2A	_
ENSG00000197291	5.13E-15	-2.63127	1	1	RAMP2-AS1	_
ENSG00000197301	5.13E-15	-2.63127	1	1	RP11-366L20.2	K01557
ENSG00000197308	5.13E-15	-2.63127	1	1	GATA3-AS1	_
ENSG00000197309	5.13E-15	-2.63127	1	1	OR10D3	K04257
ENSG00000197320	5.13E-15	-2.63127	1	1	AC060834.2	_
ENSG00000197322	5.13E-15	-2.63127	1	1	C17orf102	_
ENSG00000197334	5.13E-15	-2.63127	1	1	AC032027.1	K08857
ENSG00000197353	5.13E-15	-2.63127	1	1	LYPD2	_
ENSG00000197364	5.13E-15	-2.63127	1	1	S100A7L2	_
ENSG00000197403	5.13E-15	-2.63127	1	1	OR6N1	K04257
ENSG00000197408	5.13E-15	-2.63127	1	1	CYP2B6	K17709
ENSG00000197410	5.13E-15	-2.63127	1	1	DCHS2	K16507
ENSG00000197414	5.13E-15	-2.63127	1	1	GOLGA6L1	K09488
ENSG00000197421	5.13E-15	-2.63127	1	1	GGT3P	K18592
ENSG00000197428	5.13E-15	-2.63127	1	1	OR51D1	K04257
ENSG00000197430	5.13E-15	-2.63127	1	1	OPALIN	_
ENSG00000197437	5.13E-15	-2.63127	1	1	OR13G1	K04257
ENSG00000197454	5.13E-15	-2.63127	1	1	OR2L5	K04257
ENSG00000197462	5.13E-15	-2.63127	1	1	AC005276.1	_
ENSG00000197465	5.13E-15	-2.63127	1	1	GYPE	K06575
ENSG00000197468	5.13E-15	-2.63127	1	1	RP11-747H12.1	K04257
ENSG00000197471	5.13E-15	-2.63127	1	1	SPN	K06477
ENSG00000197472	5.13E-15	-2.63127	1	1	ZNF695	K09228
ENSG00000197475	5.13E-15	-2.63127	1	1	ADAM3A	_
ENSG00000197476	5.13E-15	-2.63127	1	1	RP11-170L3.7	K06856
ENSG00000197479	5.13E-15	-2.63127	1	1	PCDHB11	K16494
ENSG00000197487	5.13E-15	-2.63127	1	1	GALP	_
ENSG00000197496	5.13E-15	-2.63127	1	1	SLC2A10	K08147
ENSG00000197502	5.13E-15	-2.63127	1	1	AL627171.1	_
ENSG00000197503	5.13E-15	-2.63127	1	1	LINC00477	_
ENSG00000197520	5.13E-15	-2.63127	1	1	FAM177B	_
ENSG00000197532	5.13E-15	-2.63127	1	1	OR6Y1	K04257
ENSG00000197549	5.13E-15	-2.63127	1	1	PRAMENP	_
ENSG00000197550	5.13E-15	-2.63127	1	1	RP11-460N11.2	K09228
ENSG00000197575	5.13E-15	-2.63127	1	1	RPS17P2	K02962
ENSG00000197585	5.13E-15	-2.63127	1	1	AC107218.3	_
ENSG00000197588	5.13E-15	-2.63127	1	1	KLKP1	_

ENSG00000197591	5.13E-15	-2.63127	1	1	OR11L1	K04257
ENSG00000197594	5.13E-15	-2.63127	1	1	ENPP1	K01513
ENSG00000197595	5.13E-15	-2.63127	1	1	C13orf35	_
ENSG00000197616	5.13E-15	-2.63127	1	1	MYH6	K17751
ENSG00000197617	5.13E-15	-2.63127	1	1	VN1R5	K04614
ENSG00000197619	5.13E-15	-2.63127	1	1	ZNF615	K09228
ENSG00000197627	5.13E-15	-2.63127	1	1	UBE2Q2P12	K10582
ENSG00000197641	5.13E-15	-2.63127	1	1	SERPINB13	K13963
ENSG00000197644	5.13E-15	-2.63127	1	1	AC106870.2	_
ENSG00000197647	5.13E-15	-2.63127	1	1	ZNF433	K09228
ENSG00000197651	5.13E-15	-2.63127	1	1	CCER1	_
ENSG00000197658	5.13E-15	-2.63127	1	1	SLC22A24	K08206
ENSG00000197665	5.13E-15	-2.63127	1	1	AC007952.6	_
ENSG00000197674	5.13E-15	-2.63127	1	1	OR51C1P	K04257
ENSG00000197683	5.13E-15	-2.63127	1	1	KRTAP26-1	_
ENSG00000197692	5.13E-15	-2.63127	1	1	CBX3P7	K11586
ENSG00000197704	5.13E-15	-2.63127	1	1	FRMPD2P1	_
ENSG00000197705	5.13E-15	-2.63127	1	1	KLHL14	K10451
ENSG00000197706	5.13E-15	-2.63127	1	1	OR6C74	K04257
ENSG00000197721	5.13E-15	-2.63127	1	1	CR1L	K04011
ENSG00000197723	5.13E-15	-2.63127	1	1	HSPB9	K09547
ENSG00000197734	5.13E-15	-2.63127	1	1	C14orf178	K09228
ENSG00000197745	5.13E-15	-2.63127	1	1	SCGB1D4	_
ENSG00000197775	5.13E-15	-2.63127	1	1	DHRS4-AS1	_
ENSG00000197776	5.13E-15	-2.63127	1	1	KLHDC1	_
ENSG00000197779	5.13E-15	-2.63127	1	1	ZNF81	K09228
ENSG00000197786	5.13E-15	-2.63127	1	1	OR5B17	K04257
ENSG00000197790	5.13E-15	-2.63127	1	1	OR52M1	K04257
ENSG00000197794	5.13E-15	-2.63127	1	1	IGKV7-3	_
ENSG00000197805	5.13E-15	-2.63127	1	1	BX649597.1	K06053
ENSG00000197822	5.13E-15	-2.63127	1	1	OCLN	K06088
ENSG00000197825	5.13E-15	-2.63127	1	1	AC061975.1	K01368
ENSG00000197826	5.13E-15	-2.63127	1	1	C4orf22	_
ENSG00000197838	5.13E-15	-2.63127	1	1	CYP2A13	K17685
ENSG00000197844	5.13E-15	-2.63127	1	1	AL358813.1	_
ENSG00000197849	5.13E-15	-2.63127	1	1	OR8G1	K04257
ENSG00000197851	5.13E-15	-2.63127	1	1	C7orf76	_
ENSG00000197863	5.13E-15	-2.63127	1	1	ZNF790	K09228
ENSG00000197866	5.13E-15	-2.63127	1	1	OR5M12P	K04257
ENSG00000197870	5.13E-15	-2.63127	1	1	PRB3	K13911
ENSG00000197872	5.13E-15	-2.63127	1	1	FAM49A	_
ENSG00000197887	5.13E-15	-2.63127	1	1	OR1S2	K04257
ENSG00000197889	5.13E-15	-2.63127	1	1	MEIG1	_
ENSG00000197891	5.13E-15	-2.63127	1	1	SLC22A12	K08208
ENSG00000197893	5.13E-15	-2.63127	1	1	NRAP	_
ENSG00000197901	5.13E-15	-2.63127	1	1	SLC22A6	K08203
ENSG00000197910	5.13E-15	-2.63127	1	1	FAM25G	_
ENSG00000197919	5.13E-15	-2.63127	1	1	IFNA1	K05414
ENSG00000197921	5.13E-15	-2.63127	1	1	HES5	K06055
ENSG00000197928	5.13E-15	-2.63127	1	1	ZNF677	K09228
ENSG00000197932	5.13E-15	-2.63127	1	1	F8A1	_
ENSG00000197934	5.13E-15	-2.63127	1	1	AP001597.1	_
ENSG00000197935	5.13E-15	-2.63127	1	1	ZNF311	K09228
ENSG00000197937	5.13E-15	-2.63127	1	1	ZNF347	K12581
ENSG00000197938	5.13E-15	-2.63127	1	1	OR5H2	K04257
ENSG00000197953	5.13E-15	-2.63127	1	1	AADACL2	K14350
ENSG00000197984	5.13E-15	-2.63127	1	1	OR51A8P	K04257
ENSG00000197990	5.13E-15	-2.63127	1	1	ZNF734P	K09228

ENSG00000197991	5.13E-15	-2.63127	1	1	PCDH20	K16498
ENSG00000197992	5.13E-15	-2.63127	1	1	CLEC9A	K17515
ENSG00000197993	5.13E-15	-2.63127	1	1	KEL	K06577
ENSG00000198010	5.13E-15	-2.63127	1	1	DLGAP2	_
ENSG00000198019	5.13E-15	-2.63127	1	1	FCGR1B	K06498
ENSG00000198021	5.13E-15	-2.63127	1	1	SPANXA1	_
ENSG00000198022	5.13E-15	-2.63127	1	1	RP11-432N13.4	_
ENSG00000198028	5.13E-15	-2.63127	1	1	ZNF560	K09228
ENSG00000198033	5.13E-15	-2.63127	1	1	TUBA3C	K07374
ENSG00000198046	5.13E-15	-2.63127	1	1	ZNF667	K09228
ENSG00000198049	5.13E-15	-2.63127	1	1	AVPR1B	K04227
ENSG00000198054	5.13E-15	-2.63127	1	1	DSCR8	_
ENSG00000198062	5.13E-15	-2.63127	1	1	POTEH	K17299
ENSG00000198064	5.13E-15	-2.63127	1	1	RP11-347C12.1	K01896
ENSG00000198075	5.13E-15	-2.63127	1	1	SULT1C4	K01025
ENSG00000198077	5.13E-15	-2.63127	1	1	CYP2A7	K17684
ENSG00000198082	5.13E-15	-2.63127	1	1	H2AFB1	K11251
ENSG00000198083	5.13E-15	-2.63127	1	1	KRTAP9-9	_
ENSG00000198090	5.13E-15	-2.63127	1	1	KRTAP4-6	_
ENSG00000198092	5.13E-15	-2.63127	1	1	TMPRSS11F	K09752
ENSG00000198093	5.13E-15	-2.63127	1	1	ZNF649	K09228
ENSG00000198099	5.13E-15	-2.63127	1	1	ADH4	K13980
ENSG00000198100	5.13E-15	-2.63127	1	1	AL138751.1	K12581
ENSG00000198104	5.13E-15	-2.63127	1	1	OR2T6	K04257
ENSG00000198108	5.13E-15	-2.63127	1	1	CHSY3	K13499
ENSG00000198128	5.13E-15	-2.63127	1	1	OR2L3	K04257
ENSG00000198129	5.13E-15	-2.63127	1	1	DEFB107B	_
ENSG00000198133	5.13E-15	-2.63127	1	1	TMEM229B	_
ENSG00000198153	5.13E-15	-2.63127	1	1	ZNF849P	K09228
ENSG00000198155	5.13E-15	-2.63127	1	1	ZNF876P	K09228
ENSG00000198161	5.13E-15	-2.63127	1	1	PPIAL4C	K12738
ENSG00000198173	5.13E-15	-2.63127	1	1	FAM47C	_
ENSG00000198178	5.13E-15	-2.63127	1	1	CLEC4C	K06723
ENSG00000198183	5.13E-15	-2.63127	1	1	BPIFA1	_
ENSG00000198185	5.13E-15	-2.63127	1	1	ZNF334	K09228
ENSG00000198203	5.13E-15	-2.63127	1	1	SULT1C2	K01025
ENSG00000198216	5.13E-15	-2.63127	1	1	CACNA1E	K04852
ENSG00000198217	5.13E-15	-2.63127	1	1	OR51H2P	K04257
ENSG00000198250	5.13E-15	-2.63127	1	1	ANTXRL	_
ENSG00000198251	5.13E-15	-2.63127	1	1	CTC-490E21.13	K17685
ENSG00000198261	5.13E-15	-2.63127	1	1	OR5BB1P	K04257
ENSG00000198271	5.13E-15	-2.63127	1	1	KRTAP4-5	_
ENSG00000198277	5.13E-15	-2.63127	1	1	RP11-468N14.09	K00699
ENSG00000198283	5.13E-15	-2.63127	1	1	OR5B21	K04257
ENSG00000198284	5.13E-15	-2.63127	1	1	NUP210P1	K14314
ENSG00000198300	5.13E-15	-2.63127	1	1	PEG3	K09230
ENSG00000198305	5.13E-15	-2.63127	1	1	SPDYE8P	K08694
ENSG00000198307	5.13E-15	-2.63127	1	1	H2AFB2	K11251
ENSG00000198327	5.13E-15	-2.63127	1	1	HIST1H4F	K11254
ENSG00000198342	5.13E-15	-2.63127	1	1	ZNF442	K09228
ENSG00000198346	5.13E-15	-2.63127	1	1	ZNF813	K09228
ENSG00000198354	5.13E-15	-2.63127	1	1	DCAF12L2	K11803
ENSG00000198358	5.13E-15	-2.63127	1	1	RP11-544M22.1	_
ENSG00000198367	5.13E-15	-2.63127	1	1	OR7A11P	K04257
ENSG00000198388	5.13E-15	-2.63127	1	1	AC092291.1	_
ENSG00000198390	5.13E-15	-2.63127	1	1	KRTAP13-1	_
ENSG00000198398	5.13E-15	-2.63127	1	1	TMEM207	_
ENSG00000198414	5.13E-15	-2.63127	1	1	TATDN2P1	K03424

ENSG00000198417	5.13E-15	-2.63127	1	1	MT1F	K14739
ENSG00000198429	5.13E-15	-2.63127	1	1	ZNF69	K09228
ENSG00000198434	5.13E-15	-2.63127	1	1	AF241725.1	_
ENSG00000198443	5.13E-15	-2.63127	1	1	KRTAP4-1	_
ENSG00000198444	5.13E-15	-2.63127	1	1	F8A2	_
ENSG00000198445	5.13E-15	-2.63127	1	1	CCT8L2	K09500
ENSG00000198452	5.13E-15	-2.63127	1	1	OR14L1P	K04257
ENSG00000198453	5.13E-15	-2.63127	1	1	ZNF568	K09228
ENSG00000198454	5.13E-15	-2.63127	1	1	C9orf141	_
ENSG00000198477	5.13E-15	-2.63127	1	1	ZNF280B	_
ENSG00000198482	5.13E-15	-2.63127	1	1	ZNF808	K09228
ENSG00000198483	5.13E-15	-2.63127	1	1	ANKRD35	_
ENSG00000198488	5.13E-15	-2.63127	1	1	B3GNT6	K00739
ENSG00000198491	5.13E-15	-2.63127	1	1	RP11-208N14.4	_
ENSG00000198502	5.13E-15	-2.63127	1	1	HLA-DRB5	K06752
ENSG00000198521	5.13E-15	-2.63127	1	1	ZNF43	_
ENSG00000198526	5.13E-15	-2.63127	1	1	PABPC1P2	K13126
ENSG00000198555	5.13E-15	-2.63127	1	1	RP11-598D12.4	K05039
ENSG00000198558	5.13E-15	-2.63127	1	1	HIST1H4L	K11254
ENSG00000198563	5.13E-15	-2.63127	1	1	DDX39B	K12812
ENSG00000198573	5.13E-15	-2.63127	1	1	SPANXC	_
ENSG00000198597	5.13E-15	-2.63127	1	1	ZNF536	K09228
ENSG00000198601	5.13E-15	-2.63127	1	1	OR2M2	K04257
ENSG00000198610	5.13E-15	-2.63127	1	1	AKR1C4	K00037
ENSG00000198624	5.13E-15	-2.63127	1	1	CCDC69	K17985
ENSG00000198633	5.13E-15	-2.63127	1	1	ZNF534	K09228
ENSG00000198643	5.13E-15	-2.63127	1	1	FAM3D	_
ENSG00000198648	5.13E-15	-2.63127	1	1	STK39	K08835
ENSG00000198650	5.13E-15	-2.63127	1	1	TAT	K00815
ENSG00000198657	5.13E-15	-2.63127	1	1	OR8B4	K04257
ENSG00000198670	5.13E-15	-2.63127	1	1	LPA	K09644
ENSG00000198671	5.13E-15	-2.63127	1	1	RP11-168J19.2	_
ENSG00000198674	5.13E-15	-2.63127	1	1	OR10G6	K04257
ENSG00000198678	5.13E-15	-2.63127	1	1	OR5BS1P	K04257
ENSG00000198681	5.13E-15	-2.63127	1	1	MAGEA1	_
ENSG00000198683	5.13E-15	-2.63127	1	1	AC012615.1	K17985
ENSG00000198685	5.13E-15	-2.63127	1	1	C3orf27	K18047
ENSG00000198692	5.13E-15	-2.63127	1	1	EIF1AY	K03236
ENSG00000198703	5.13E-15	-2.63127	1	1	OR10R3P	K04257
ENSG00000198704	5.13E-15	-2.63127	1	1	GPX6	K00432
ENSG00000198719	5.13E-15	-2.63127	1	1	DLL1	K06051
ENSG00000198729	5.13E-15	-2.63127	1	1	PPP1R14C	K17556
ENSG00000198734	5.13E-15	-2.63127	1	1	F5	K03902
ENSG00000198738	5.13E-15	-2.63127	1	1	SMIM11P1	_
ENSG00000198743	5.13E-15	-2.63127	1	1	SLC5A3	K14383
ENSG00000198756	5.13E-15	-2.63127	1	1	COLGALT2	K11703
ENSG00000198759	5.13E-15	-2.63127	1	1	EGFL6	_
ENSG00000198765	5.13E-15	-2.63127	1	1	SYCP1	K19533
ENSG00000198787	5.13E-15	-2.63127	1	1	OR4D12P	K04257
ENSG00000198788	5.13E-15	-2.63127	1	1	MUC2	K10955
ENSG00000198797	5.13E-15	-2.63127	1	1	BRINP2	_
ENSG00000198798	5.13E-15	-2.63127	1	1	MAGEB3	_
ENSG00000198812	5.13E-15	-2.63127	1	1	LRRC10	K17692
ENSG00000198821	5.13E-15	-2.63127	1	1	CD247	K06453
ENSG00000198842	5.13E-15	-2.63127	1	1	DUSP27	K14165
ENSG00000198844	5.13E-15	-2.63127	1	1	ARHGEF15	_
ENSG00000198848	5.13E-15	-2.63127	1	1	CES1	K01044
ENSG00000198851	5.13E-15	-2.63127	1	1	CD3E	K06451

ENSG00000198854	5.13E-15	-2.63127	1	1	C1orf68	_
ENSG00000198865	5.13E-15	-2.63127	1	1	CCDC152	_
ENSG00000198868	5.13E-15	-2.63127	1	1	MIR4461	K03882
ENSG00000198877	5.13E-15	-2.63127	1	1	OR5D13	K04257
ENSG00000198881	5.13E-15	-2.63127	1	1	ASB12	K10334
ENSG00000198883	5.13E-15	-2.63127	1	1	PNMA5	_
ENSG00000198885	5.13E-15	-2.63127	1	1	ITPRIPL1	_
ENSG00000198889	5.13E-15	-2.63127	1	1	DCAF12L1	K11803
ENSG00000198908	5.13E-15	-2.63127	1	1	BHLHB9	_
ENSG00000198914	5.13E-15	-2.63127	1	1	POU3F3	K09365
ENSG00000198923	5.13E-15	-2.63127	1	1	RP11-290L1.7	K11130
ENSG00000198932	5.13E-15	-2.63127	1	1	GPRASP1	_
[truncated: 725,550 more chars]
